# Novel Driver Strength Index highlights important cancer genes in TCGA PanCanAtlas patients

**DOI:** 10.7717/peerj.13860

**Published:** 2022-08-11

**Authors:** Aleksey V. Belikov, Alexey D. Vyatkin, Sergey V. Leonov

**Affiliations:** Laboratory of Innovative Medicine, School of Biological and Medical Physics, Moscow Institute of Physics and Technology, Dolgoprudny, Moscow Region, Russia

**Keywords:** Cancer, Driver, Genes, Mutations, Pathways

## Abstract

**Background:**

Cancer driver genes are usually ranked by mutation frequency, which does not necessarily reflect their driver strength. We hypothesize that driver strength is higher for genes preferentially mutated in patients with few driver mutations overall, because these few mutations should be strong enough to initiate cancer.

**Methods:**

We propose formulas for the Driver Strength Index (DSI) and the Normalized Driver Strength Index (NDSI), the latter independent of gene mutation frequency. We validate them using TCGA PanCanAtlas datasets, established driver prediction algorithms and custom computational pipelines integrating SNA, CNA and aneuploidy driver contributions at the patient-level resolution.

**Results:**

DSI and especially NDSI provide substantially different gene rankings compared to the frequency approach. *E.g*., NDSI prioritized members of specific protein families, including G proteins *GNAQ*, *GNA11* and *GNAS*, isocitrate dehydrogenases *IDH1* and *IDH2*, and fibroblast growth factor receptors *FGFR2* and *FGFR3*. KEGG analysis shows that top NDSI-ranked genes comprise *EGFR/FGFR2/GNAQ/GNA11–NRAS/HRAS/KRAS–BRAF* pathway, *AKT1–MTOR* pathway, and *TCEB1–VHL–HIF1A* pathway.

**Conclusion:**

Our indices are able to select for driver gene attributes not selected by frequency sorting, potentially for driver strength. Genes and pathways prioritized are likely the strongest contributors to cancer initiation and progression and should become future therapeutic targets.

## Introduction

According to the mainstream view, cancer is initiated and promoted by mutations in so-called “driver” genes, whereas mutations in “passenger” genes bear no effect and simply “travel” along, during the course of somatic evolution (Vogelstein et al., 2013). There are multiple approaches to identifying cancer driver genes and to their ranking (Marx, 2014; Raphael et al., 2014). One of the earliest and most intuitive approaches is to use mutation frequency, typically corrected by background mutation rate in a gene, *e.g*. by the rate of synonymous mutations. Some notable examples of such algorithms are MuSiC (Dees et al., 2012), MutSigCV (Lawrence et al., 2013) and dNdScv (Martincorena et al., 2017). Interestingly, even after background correction, high recurrence of a mutated gene in a cancer cohort does not necessarily translate to the statement that this gene is a strong driver. We can imagine a gene that is mutated in the majority of cancer patients (*e.g*., because it has multiple suitable sites for a driver mutation, such as catalytic sites, sites of post-translational modifications or protein-protein interfaces) but has a weak contribution to cancer progression in each of these patients (*e.g*., because this gene is redundant). We can also imagine a gene that is mutated rarely (*e.g*., because it has only one suitable site for a driver mutation) but if the mutation does occur it immediately leads to cancer (*e.g*., because this gene is in a key position to control cell growth). The former would be an example of a frequent but weak driver, whereas the latter would be a rare but strong driver. Overall, algorithms based on mutation recurrence cannot reliably determine driver gene strength.

There is a large group of algorithms that aim to predict and rank driver genes according to the impact of mutations on protein structure and activity. Some notable examples include HotMAPS (Tokheim et al., 2016a), 2020plus (Tokheim et al., 2016b), OncodriveFML (Mularoni et al., 2016), OncodriveCLUSTL (Arnedo-Pac et al., 2019) and CHASMplus (Tokheim & Karchin, 2019). Typically, these algorithms are based on mutation clustering, sequence evolutionary conservation, protein domains and three-dimensional protein structure, or combinations thereof, sometimes with the use of machine learning. These methods can reliably identify if a given protein’s structure and function are disturbed by a given mutation and to what degree. However, they are much less suitable for identification of the role of this protein in the cellular and microenvironmental context, which is critical to determine whether it will drive cancer and how strongly. Thus, the strength of driver genes is unlikely to be determined by structural approaches.

Some algorithms aim to predict and rank drivers based on data from protein interaction networks, *e.g*., NetBox (Cerami et al., 2010), DriverNet (Bashashati et al., 2012), NetSig (Horn et al., 2018) and Hierarchical HotNet (Reyna, Leiserson & Raphael, 2018). The idea is that a gene having multiple connections with other genes, *i.e*. playing the role of a network hub, will have more dramatic influence on the cell in case of mutation (Bowler, Wang & Ewing, 2015). However, whilst mutations in network hubs are indeed likely to cause severe disturbance in the cell, this would rather lead to cell death than to oncogenic transformation. Thus, network-based approaches are also not likely to uncover the actual strength of driver genes.

We have recently quantified the number of driver events in individual patients in various cancer types and discovered very high variability in this number even within the same cancer type (Vyatkin et al., 2022). We therefore asked a question: what is the reason that for some patients one driver event is sufficient to drive cancer, whereas the others do not develop cancer until dozens of driver events accumulate? We hypothesized that the major reason is driver strength–one strong driver is equivalent in its cancer-promoting potency to several weak drivers. Thus, we define the strength of a cancer driver as its share of the contribution to the initiation and progression of a given cancer relative to other drivers. It immediately follows that a driver that is able to singlehandedly initiate cancer is a strong driver, whereas a driver that is present in cancers only amongst the multitude of other drivers is very likely a weak driver.

We reason that a few strong drivers are sufficient to initiate cancer, and there would be no need to accumulate additional drivers. On the other hand, weak drivers would need to accumulate in much higher quantity, until their combined strength would become sufficient to initiate cancer. Therefore, it should be statistically more likely to find strong drivers in patients that have only few driver mutations in their tumours, and less likely to find them in patients with multiple drivers per tumour. Likewise, it should be statistically less likely to find weak drivers in patients that have only few driver mutations in their tumours, and more likely to find them in patients with multiple drivers per tumour. Hence, we propose the Driver Strength Index (DSI) that takes into account the frequencies of mutation of a given driver gene in groups of patients with different total number of driver mutations, and gives priority weights to groups with fewer mutations. We also propose a modification of this index that is completely independent of the overall frequency of mutation of a given driver gene–the Normalized Driver Strength Index (NDSI).

Calculating these indices requires data on the number of driver mutations in each individual patient. The majority of existing driver prediction algorithms work at the cohort level, *i.e*. they predict driver genes for large groups of patients, usually having a particular cancer type. This does not allow to look at the composition of driver mutations in individual patients. We wrote specific scripts to convert cohort-level predictions into patient-level events (Vyatkin et al., 2022), which also allowed seamless integration of the results from various third-party algorithms, including 2020plus, CHASMplus, CompositeDriver, dNdScv, DriverNet, HotMAPS, OncodriveCLUSTL and OncodriveFML. This is useful, as each individual driver prediction algorithm is based on the unique combination of theoretical concepts and computational methodology and thus has its own strengths and shortcomings, and combining results from multiple algorithms allows to obtain more complete and balanced picture, ensuring that less driver mutations have been missed. We also used a consensus driver gene list from 26 algorithms (Bailey et al., 2018), applied separately to each cancer cohort of TCGA PanCanAtlas, and a list of COSMIC Cancer Gene Census Tier 1 genes affected by somatic SNAs and CNAs (CGC), as it represents the current gold standard of verified cancer drivers (Sondka et al., 2018). To minimize false positives, we used only driver gene-cohort pairs that were independently predicted by at least two of our sources (Vyatkin et al., 2022).

## Methods

Our detailed workflow is described in Methods S1. Most of the methodology, except for the PALDRIC modification, enabling the calculation of driver strength indices, and pathway and network analysis of top-ranked driver genes, has been previously published in (Vyatkin et al., 2022). Here, we describe it briefly.

### Source files and initial filtering

TCGA PanCanAtlas (https://www.cell.com/pb-assets/consortium/PanCancerAtlas/PanCani3/index.html) data were used. Files containing data on sample quality, purity, ploidy, SNA, CNA, mRNA and miRNA expression were downloaded from https://gdc.cancer.gov/about-data/publications/PanCan-CellOfOrigin. The file with clinical information was downloaded from https://gdc.cancer.gov/node/905/. Only samples with primary malignant neoplasm histology were used. All samples marked as low quality, with cancer DNA fraction <50% or with subclonal genome fraction >50% were removed. Only patients having data simultaneously for SNA, CNA and aneuploidy were used.

### RNA filtering of CNAs

The median expression level for each gene across patients was determined. If the expression for a given gene in a given patient was below 0.05x median value, it was encoded as “−2”, if between 0.05x and 0.75x median value, it was encoded as “−1”, if between 1.25x and 1.75x median value, it was encoded as “1”, if above 1.75x median value, it was encoded as “2”, otherwise it was encoded as “0”. If the gene CNA status in a given patient was not zero and had the same sign as the gene expression status in the same patient, then the CNA status value was replaced with the gene expression status value, otherwise it was replaced by zero. If the corresponding expression status for a given gene was not found then its CNA status was not changed. We named this algorithm GECNAV (Gene Expression-based CNA Validator) and created a GitHub repository: https://github.com/belikov-av/GECNAV. The package used to generate data in this article is available as Data S2.

### Aneuploidy driver prediction

By drawing arm statuses randomly with replacement (bootstrapping) from the table with chromosomal arm statuses of individual patients, for each cancer type the number of statuses corresponding to the number of patients in that cancer type were generated and their average was calculated. The procedure was repeated 10,000 times and the median of averages for each cancer type was calculated. *P*-value for each arm alteration status was calculated for each cancer type. To do this, first the average alteration status for a given cancer type and a given arm was calculated and compared to the median of averaged bootstrapped arm alteration statuses for this cancer type. If the average status was higher than zero and the bootstrapped median, the number of bootstrapped statuses for this cancer type that are higher than the average status was counted and divided by 5,000. If the average status was lower than zero and the bootstrapped median, the number of bootstrapped statuses for this cancer type that are lower than the average status was counted and divided by 5,000. Other values were ignored (cells left empty). For each cancer type, the Benjamini–Hochberg procedure with FDR = 5% was applied to *P*-values and passing *P*-values were encoded as “DAG” (Driver arm gain) or “DAL” (Driver arm loss). The other cells were made empty. Alterations were classified according to the following rules: if the arm status in a given patient was “−1” and the average alteration status of a given arm in the same cancer type was “DAL”, then the alteration in the patient was classified as “DAL”. If the arm status in a given patient was “1” and the average alteration status of a given arm in the same cancer type was “DAG”, then the alteration in the patient was classified as “DAG”. In all other cases an empty cell was written. The same procedures as described above for chromosomal arms were repeated for the whole chromosomes. Chromosome drivers were considered to override arm drivers, so if a chromosome had “DCL” (Driver chromosome loss) or “DCG” (Driver chromosome gain), no alterations were counted on the arm level, to prevent triple counting of the same event. We named this algorithm ANDRIF (ANeuploidy DRIver Finder) and created a GitHub repository: https://github.com/belikov-av/ANDRIF. The package used to generate data in this article is available as Data S3.

### SNA classification

Frameshift deletions and insertions, nonsense, nonstop and translation start site mutations were considered potentially inactivating; in frame deletions, insertions and *de novo* start, as well as missense mutations, were considered potentially hyperactivating; *de novo* start out of frame and silent mutations were considered passengers; the rest were considered unclear. The sum of all alterations of each type in all patients was calculated for each gene. Genes containing only SNAs with unclear role (likely, noncoding genes) were removed. Next, the Hyperactivating to Inactivating SNA Ratio (HISR) was calculated for each gene as

(1) $${HISR} = \displaystyle{{{\rm  Number}\; {\rm  of}\; {\rm hyperactivating}\; {\rm SNAs} + \rm 1} \over {{\rm Number}\; {\rm of}\; {\rm inactivating}\; {\rm SNAs} + \rm 1}}$$

Genes for which the sum of hyperactivating, inactivating and passenger SNAs was less than 10 were removed to ensure sufficient precision of HISR calculation. We named this algorithm SNADRIF (SNA DRIver Finder) and created a GitHub repository: https://github.com/belikov-av/SNADRIF. The package used to generate data in this article is available as Data S4.

### Driver prediction algorithms sources

Lists of driver genes and mutations predicted by various algorithms applied to PanCanAtlas data were downloaded from https://gdc.cancer.gov/about-data/publications/pancan-driver (2020plus, CompositeDriver, DriverNet, HotMAPS, OncodriveFML), https://karchinlab.github.io/CHASMplus/ (CHASMplus), as well as received by personal communication from Francisco Martínez-Jiménez, Institute for Research in Biomedicine, Barcelona, francisco.martinez@irbbarcelona.org (dNdScv, OncodriveCLUSTL, OncodriveFML). All genes and mutations with q-value > 0.05 were removed. Additionally, a consensus driver gene list from 26 algorithms applied to PanCanAtlas data was downloaded from (Bailey et al., 2018) and COSMIC Cancer Gene Census (CGC) Tier 1 gene list was downloaded from https://cancer.sanger.ac.uk/cosmic/census?tier=1. Only genes affected by somatic SNAs and CNAs present in the TCGA cancer types were used for further analyses from the CGC list. Cancer type names in the CGC list were manually converted to the closest possible TCGA cancer type abbreviation. Entrez Gene IDs were identified for each gene using HUGO Symbol and the NCBI Gene database: http://ftp.ncbi.nih.gov/gene/DATA/GENE_INFO/Mammalia/Homo_sapiens.gene_info.gz.

### Conversion of population-level data to patient-level data

Cohort-level lists of driver genes predicted by third-party algorithms were matched to the patient-level SNA data *via* the simultaneous matching of the Entrez Gene ID and cancer type. Cohort-level lists of driver mutations were matched to the patient-level SNA data *via* the Ensembl Transcript ID, nucleotide/amino acid substitution and cancer type. All cohort-level lists were matched to the patient-level CNA data *via* the Entrez Gene ID and cancer type. Resulting patient-level SNA and CNA lists were combined and duplicates removed.

### Driver event classification and analysis

Third-party algorithm outputs converted to patient-level as described above were combined and all TCGA Barcode-Entrez Gene ID (patient-gene) pairs not present in at least two output files were removed. Numbers of hyperactivating and inactivating SNAs for each TCGA Barcode-Entrez Gene ID pair were taken from the SNADRIF output, in case of no match zeros were written. A HISR value for each Entrez Gene ID was also taken from the SNADRIF output, in case of no match an empty cell was left. A CNA status for each TCGA Barcode-Entrez Gene ID pair was taken from the GECNAV output, in case of no match zero was written. Each TCGA Barcode-Entrez Gene ID pair was classified according to the Table 1.

**Table 1:** Driver event classification rules.

Driver type	Number of nonsynonymous SNAs	Number of inactivating SNAs	HISR	CNA status	Count as … driver event(s)
**SNA-based oncogene**	≥1	0	>5	0	1
**CNA-based oncogene**	0	0	>5	1 or 2	1
**Mixed oncogene**	≥1	0	>5	1 or 2	1
**SNA-based tumour suppressor**	≥1	≥0	≤5	0	1
**CNA-based tumour suppressor**	0	0	≤5	−1 or −2	1
**Mixed tumour suppressor**	≥1	≥0	≤5	−1 or −2	1
**Passenger**	0	0		0	0
**Low-probability driver**	All the rest				0

The names of individual genes, chromosome arms or full chromosomes affected by driver events of each type were catalogued for each patient. Information on the driver chromosome and arm losses and gains for each patient was taken from the ANDRIF output. The number of various types of driver events in individual genes, chromosome arms or full chromosomes was calculated for each cancer type, tumour stage, age group, as well as for patients with each total number of driver events from 1 to 100. Analyses were performed for the total population and for males and females separately, and histograms of top 10 driver events in each class and overall were plotted for each group.

Driver Strength Index (DSI)

(2) $$DS{I_{\; A}} = \mathop \sum \limits_{i = 1}^{100} \displaystyle{{{p_{\; A\; \; i}}} \over {i\; {p_{\; i}}\; }}$$

and Normalized Driver Strength Index (NDSI)

(3) $$NDS{I_{\; A}} = \displaystyle{{\mathop \sum \limits_{i = 1}^{100} \displaystyle{{{p_{\; A\; \; i}}} \over {i\; {p_{\; i}}\; }}} \over {\mathop \sum \limits_{i = 1}^{100} \displaystyle{{{p_{\; A\; \; i}}} \over {\; {p_{\; i}}\; }}}}$$

were calculated, where ${p_{\; A\; \; i}}$ is the number of patients with a driver event in the gene/chromosome *A* amongst patients with *i* driver events in total; $\; {p_{\; i}}$ is the number of patients with *i* driver events in total. We limited *i* to 100 because we have previously shown that there are on average 12 driver events per tumour, patients with one and seven driver events per tumour are the most frequent, and there are very few patients with more than 40 events (Vyatkin et al., 2022). To avoid contamination of NDSI-ranked driver event lists with very rare driver events and to increase precision of the index calculation, all events that were present in less than 10 patients in each driver event class were removed. To compose the top-(N)DSI-ranked driver list, the lists of drivers from various classes were combined, and drivers with lower (N)DSI in case of duplicates and all drivers with NDSI < 0.05 were removed. We named this algorithm PALDRIC GENE and created a GitHub repository: https://github.com/belikov-av/PALDRIC_GENE. The package used to generate data in this article is available as Data S5.

### Pathway and network analysis of top-(N)DSI-ranked driver genes

First, the chromosome arms and full chromosomes were removed from the top-(N)DSI-ranked driver lists, as external pathway and network analysis services can work only with genes.

Next, top 50 DSI-ranked genes and top 50 NDSI-ranked genes were selected, to facilitate proper comparison. The resulting lists were uploaded as Entrez Gene IDs to the “Reactome v77 Analyse gene list” tool (https://reactome.org/PathwayBrowser/#TOOL=AT) (Fabregat et al., 2018). Voronoi visualizations (Reacfoam) were exported as images. The resulting lists were also uploaded as Entrez Gene IDs to the “KEGG Mapper – Color” tool (https://www.genome.jp/kegg/mapper/color.html) (Kanehisa & Sato, 2020), “hsa” Search mode was selected, the search executed and the top result-“Pathways in cancer - *Homo sapiens* (human)” (hsa05200) was selected for mapping. The resulting images were exported. The data were also analysed in Cytoscape 3.8.2 (https://cytoscape.org) (Shannon et al., 2003). The BioGRID: Protein-Protein Interactions (*H. sapiens*) network was imported and then (N)DSI values appended from the top 50 (N)DSI-ranked driver list. First, Degree Sorted Circle Layout was selected and genes not within the circle were removed. Node Fill Color was mapped to (N)DSI values with Continuous Mapping and Node Height and Width were mapped to the degree.layout parameter (number of connections) with Continuous Mapping. Then, yFiles Organic Layout was selected and the legend appended. The resulting images were exported.

## Results

**Figure 1:**
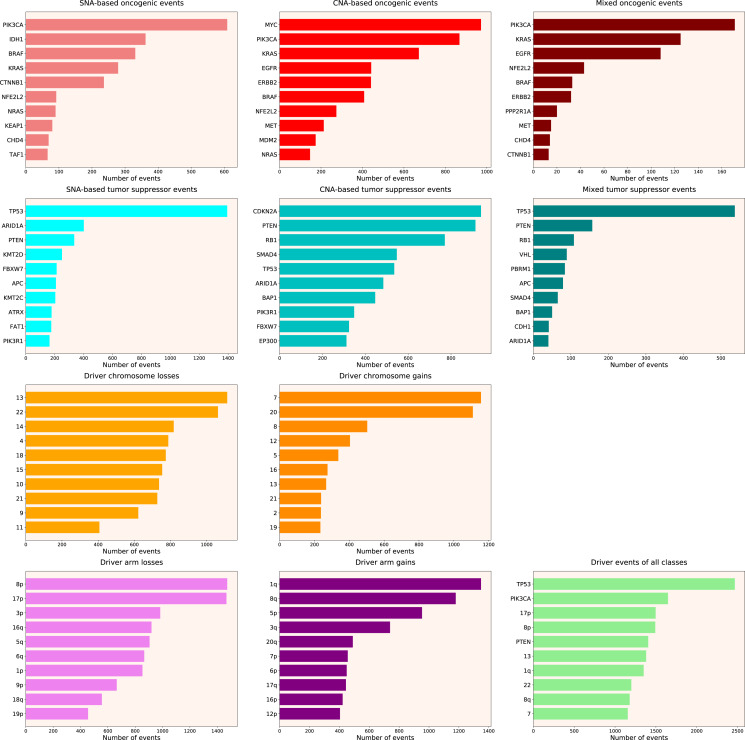
Top 10 driver events from different molecular and functional classes sorted by the number of occurrences in TCGA PanCanAtlas.

We calculated the number of various types of driver events in individual genes, chromosome arms or full chromosomes for each cancer type, tumour stage, age group, as well as for patients with each total number of driver events from 1 to 100. We performed the analyses for the total population and for males and females separately, and, for each group, plotted the histograms of top 10 driver events in each class and overall (for data and graphs see Data S1). In Fig. 1 we present the overall ranking of genes for all TCGA PanCanAtlas cohorts combined. It can be seen that *PIK3CA* is the oncogene with the highest number of SNAs, as well as the highest number of simultaneous occurrences of SNAs and gene amplifications. *MYC* is the oncogene with the highest number of amplifications. *TP53* is the tumour suppressor with the highest number of SNAs, as well as the highest number of instances of simultaneous occurrences of an SNA in one allele and a deletion of the other allele. It is also the top mutated gene when driver events of all classes are counted. *CDKN2A* and *PTEN* are tumour suppressors with the highest number of deletions. Losses of chromosomes 13 and 22 are the most frequent cancer-promoting chromosome losses, whereas gains of chromosomes 7 and 20 are the most frequent cancer-promoting chromosome gains. Losses of 8p and 17p arms are the most frequent cancer-promoting chromosome arm losses, whereas gains of 1q and 8q arms are the most frequent cancer-promoting chromosome arm gains. Overall, these results are expected and indicate that our analytic pipelines work as they should.

**Figure 2:**
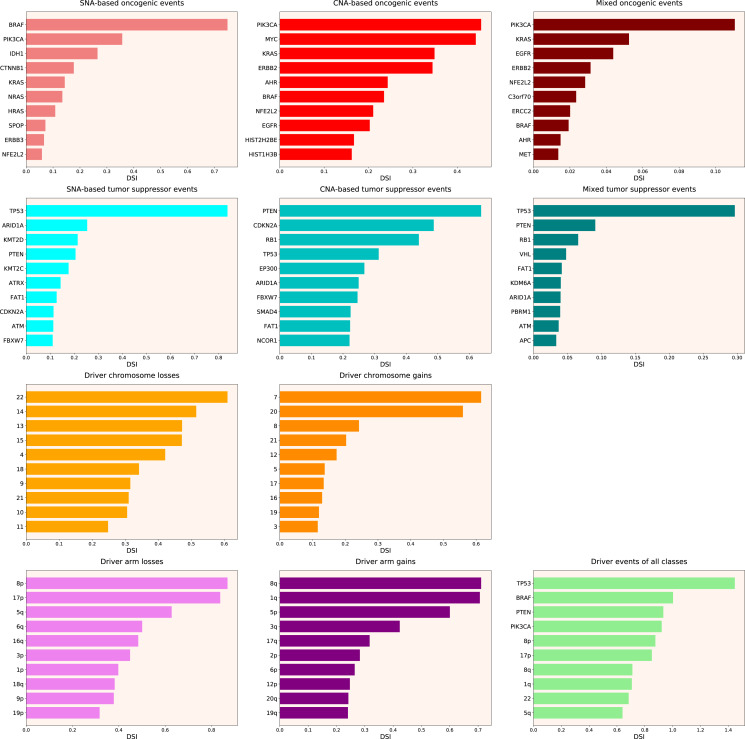
Top 10 driver events from different molecular and functional classes sorted by DSI.

Next, we calculated the Driver Strength Index (DSI) (see Eq. (2) in Methods). Surprisingly, we do not see much change compared to the simple frequency-of-mutation approach (Fig. 2). The only dramatic difference is that *BRAF* became the top SNA-based oncogene according to DSI, whereas *PIK3CA* dropped to the second place, lagging behind by a wide margin. Also, *PIK3CA* overtook *MYC* as the top CNA-based oncogene, and *PTEN* displaced *CDKN2A* from the top CNA-based tumour suppressor spot. Moreover, members of several gene families appeared in the top 10 lists, such as *KRAS*, *NRAS* and *HRAS* in the SNA-based oncogenic events list, histones *HIST2H2BE* and *HIST1H3B* in the CNA-based oncogenic events list, or lysine methyltransferases *KMT2C* and *KMT2D* in the SNA-based tumour suppressor events list. This indicates that our approach is indeed meaningfully selecting for some biological attributes, which are not selected by simple frequency sorting. Finally, multiple small changes of ranking positions happened, nevertheless not affecting the overall picture. We think the reason for the limited effect of changes is that DSI is still very much affected by the overall frequency of gene mutation, due to $\displaystyle{{{p_{\; A\; \; i}}} \over {{p_{\; i}}}}$ component. Hence, to uncover the true driver strength unrelated to the mutation frequency, further normalization is required.

**Figure 3:**
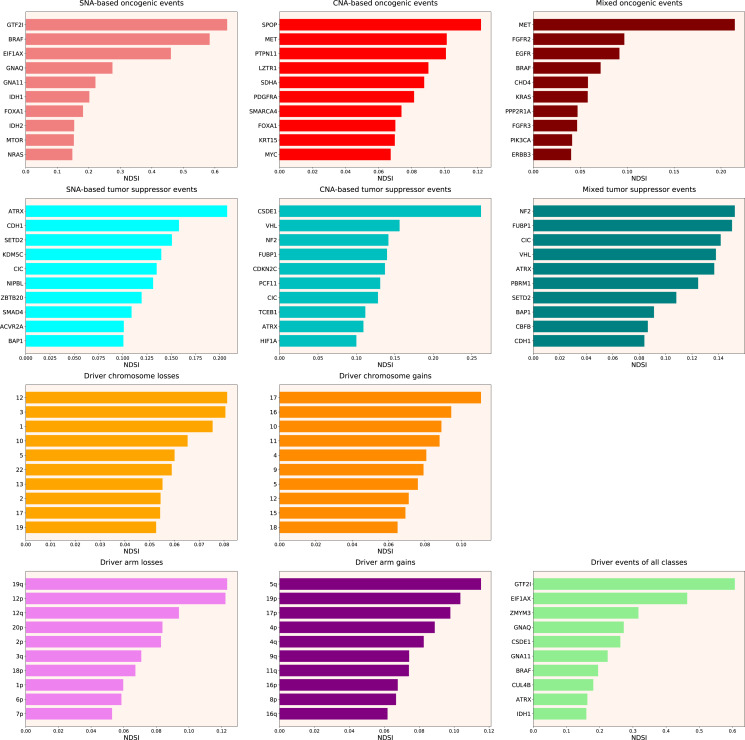
Top 10 driver events from different molecular and functional classes sorted by NDSI.

Therefore, we propose the Normalized Driver Strength Index (NDSI) that corrects for the effects of mutation frequencies (see Eq. (3) in Methods). As can be seen in Fig. 3, this time the rankings are completely different from both DSI and frequency-based approaches. *GTF2I* conquers the top spot amongst SNA-based oncogenes and overall, *SPOP* becomes number one CNA-based oncogene, and *MET* occupies the first line of mixed oncogene rating. *ATRX*, *CSDE1* and *NF2* become the top SNA-based, CNA-based and mixed tumour suppressors, respectively. NDSI reveals the losses of chromosomes 12 and 3 as the strongest cancer-promoting chromosome losses, whereas the gain of chromosome 17 as the strongest cancer-promoting chromosome gain. NDSI shows that the losses of 19q and 12p arms are the strongest cancer-promoting chromosome arm losses, whereas the gain of 5q arm is the strongest cancer-promoting chromosome arm gain.

Like DSI, NDSI is able to select for specific gene families. Two members of the guanine nucleotide-binding protein family, *GNAQ* and *GNA11*, appeared on the top 10 SNA-based oncogenic events and top 10 driver events of all classes lists (Fig. 3). Additionally, one more G protein, *GNAS*, appeared on the top 50 NDSI-ranked driver list (Table 2). Of note, no members of this family are present on the top 50 DSI-ranked driver list (Table 2). Two members of the isocitrate dehydrogenase family, *IDH1* and *IDH2*, appeared on the top 10 SNA-based oncogenic events list, whereas fibroblast growth factor receptors *FGFR2* and *FGFR3* appeared on the top 10 mixed oncogenic events list (Fig. 3). The ability of NDSI to prioritize members of specific protein families suggests that this index has an actual biological meaning.

**Table 2:** Top 50 DSI- and NDSI-ranked genes.

Rank	Entrez ID	Symbol	DSI	Rank	Entrez ID	Symbol	NDSI
1	7157	TP53	1.44664	1	100093631	GTF2I	0.6389
2	673	BRAF	1.00268	2	673	BRAF	0.58335
3	5728	PTEN	0.93317	3	1964	EIF1AX	0.46362
4	5290	PIK3CA	0.9215	4	9203	ZMYM3	0.31661
5	1029	CDKN2A	0.60404	5	2776	GNAQ	0.27502
6	5925	RB1	0.57932	6	7812	CSDE1	0.26198
7	3845	KRAS	0.54447	7	2767	GNA11	0.22382
8	8289	ARID1A	0.54286	8	8731	MET	0.21464
9	4609	MYC	0.46527	9	546	ATRX	0.20772
10	2064	ERBB2	0.41563	10	3417	IDH1	0.20172
11	2033	EP300	0.39411	11	3169	FOXA1	0.18161
12	2195	FAT1	0.39042	12	8450	CUL4B	0.18057
13	55294	FBXW7	0.37252	13	51343	CDH1	0.158
14	472	ATM	0.35055	14	7428	VHL	0.15612
15	3417	IDH1	0.29994	15	3418	IDH2	0.15364
16	4780	NFE2L2	0.2974	16	2475	MTOR	0.15247
17	1499	CTNNB1	0.29	17	4771	NF2	0.15232
18	4893	NRAS	0.27847	18	29072	SETD2	0.1507
19	1387	CREBBP	0.2738	19	8880	FUBP1	0.15022
20	5295	PIK3R1	0.27316	20	4893	NRAS	0.14785
21	4089	SMAD4	0.26931	21	207	AKT1	0.1468
22	196	AHR	0.26508	22	4615	MYD88	0.14625
23	9611	NCOR1	0.25715	23	23152	CIC	0.14165
24	1956	EGFR	0.25138	24	51585	PCF11	0.14083
25	7403	KDM6A	0.24971	25	8242	KDM5C	0.13977
26	9223	BAP1	0.24578	26	1031	CDKN2C	0.13712
27	8085	KMT2D	0.24363	27	6597	SMARCA4	0.13643
28	4297	MLL	0.23565	28	25836	NIPBL	0.13291
29	5624	APC	0.22422	29	7114	TMSB4X	0.12846
30	546	ATRX	0.21608	30	2778	GNAS	0.12708
31	2068	ERCC2	0.21513	31	26137	ZBTB20	0.1269
32	9757	MLL2	0.20883	32	84433	CARD11	0.12676
33	196528	ARID2	0.20073	33	3265	HRAS	0.12612
34	58508	MLL3	0.1921	34	55193	PBRM1	0.12463
35	58508	KMT2C	0.18892	35	8405	SPOP	0.12197
36	3265	HRAS	0.17947	36	6921	TCEB1	0.1115
37	1105	CHD1	0.17591	37	4089	SMAD4	0.10913
38	2065	ERBB3	0.17366	38	5781	PTPN11	0.10708
39	11168	PSIP1	0.17344	39	92	ACVR2A	0.10109
40	8349	HIST2H2BE	0.16951	40	3091	HIF1A	0.10082
41	5934	RBL2	0.16701	41	9223	BAP1	0.10069
42	8358	HIST1H3B	0.16666	42	4221	MEN1	0.10049
43	23019	CNOT1	0.1655	43	3845	KRAS	0.09984
44	8454	CUL1	0.16319	44	2625	GATA3	0.09885
45	55193	PBRM1	0.15965	45	841	CASP8	0.09882
46	677	ZFP36L1	0.15549	46	9361	PIM1	0.09702
47	10735	STAG2	0.15149	47	2263	FGFR2	0.09696
48	285382	C3orf70	0.14784	48	2559	GABRA6	0.09553
49	2261	FGFR3	0.14772	49	1956	EGFR	0.09175
50	4763	NF1	0.14239	50	54894	RNF43	0.09159

Next, we wanted to analyse top DSI- and NDSI-ranked genes using several common gene list analysis tools. To this aim, we combined the lists of drivers from various classes. If the same gene was affected by more than one kind of alteration, we chose the alteration type with the highest DSI or NDSI, depending on the analysis. Also, we removed the data on chromosome arms and full chromosomes, as external pathway and network analysis tools can work only with genes. Then, we selected top 50 DSI- and NDSI-ranked genes. The resulting lists can be seen in Table 2.

**Figure 4:**
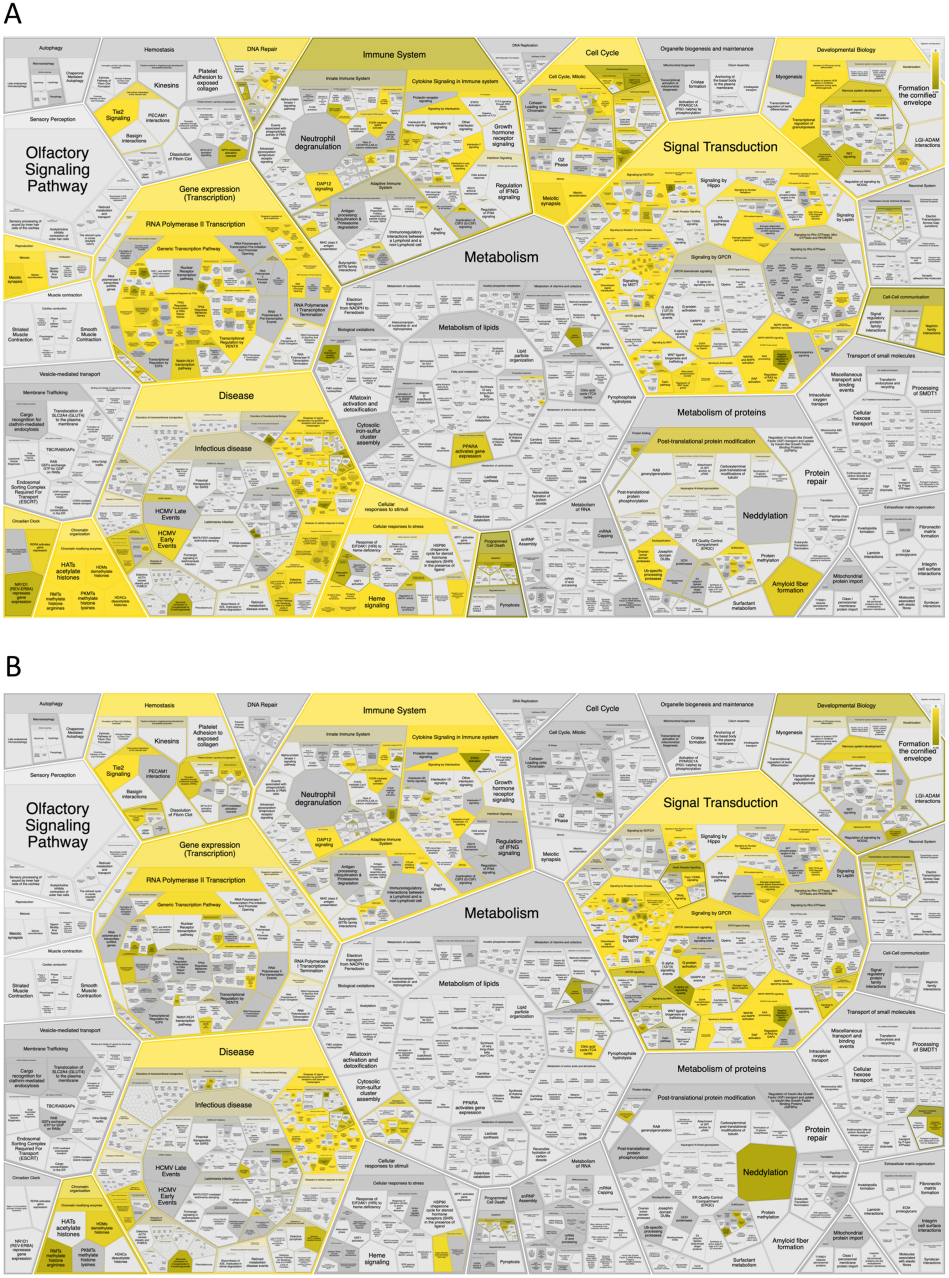
Significant overrepresentation of top 50 DSI-(A) and NDSI-(B) ranked genes in Reactome v77 pathways. The yellow colour indicates significance (see the scale at the upper right corner).

First, we analysed the top 50 DSI- and NDSI-ranked genes for overrepresentation in various Reactome pathways. It can be seen in Fig. 4A that top 50 DSI-ranked genes are significantly overrepresented in such categories as signalling by NOTCH, signalling by PTK6, ESR-mediated signalling, PIP3 activates AKT signalling, signalling by receptor tyrosine kinases, signalling by WNT, signalling by erythropoietin, RAF/MAP kinase cascade, signalling by TGF-beta receptor complex, mitotic cell cycle, meiosis, cell cycle checkpoints, DNA double-stand break repair, generic transcription pathway, epigenetic regulation of gene expression, RNA polymerase I transcription, circadian clock, chromatin modifying enzymes, diseases of signal transduction by growth factor receptors and second messengers, diseases of cellular senescence, diseases of programmed cell death, cellular responses to stress, activation of HOX genes during differentiation, and transcriptional regulation of granulopoiesis. Top 50 NDSI-ranked genes are significantly overrepresented in even fewer categories (Fig. 4B)–signalling by PTK6, extra-nuclear oestrogen signalling, negative regulation of PI3K/AKT signalling, signalling by receptor tyrosine kinases, GPCR downstream signalling, erythropoietin activates RAS, RAF/MAP kinase cascade, cytokine signalling in immune system, adaptive immune system, haemostasis, generic transcription pathway, chromatin modifying enzymes, and diseases of signal transduction by growth factor receptors and second messengers. Surprisingly, several large categories often deemed important for cancer – cell cycle, DNA replication, DNA repair, autophagy, cellular responses to stress, programmed cell death, cell-cell communication and metabolism – are not affected.

**Figure 5:**
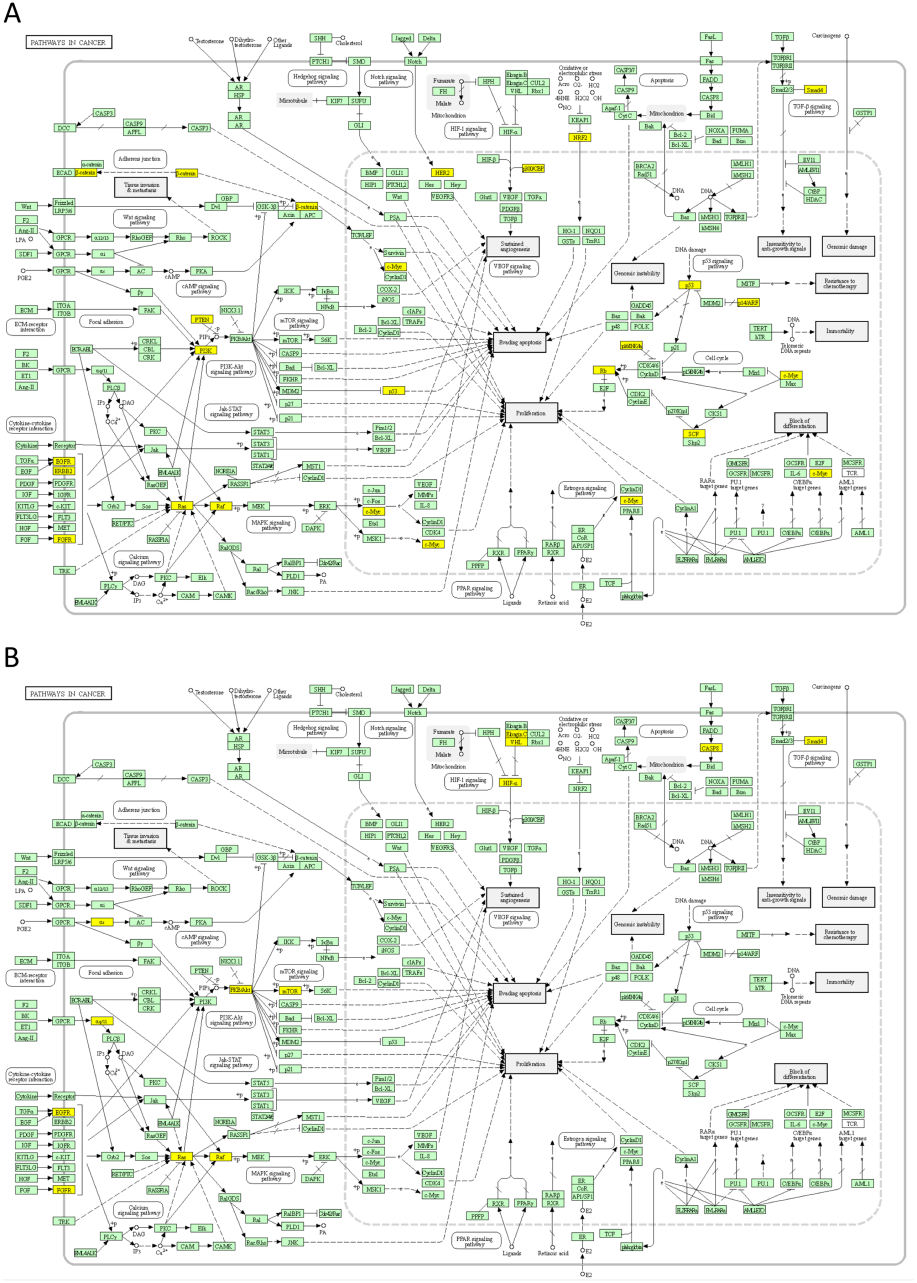
Mapping of top 50 DSI-(A) and NDSI-(B) ranked genes to the KEGG “Pathways in cancer” (hsa05200) map. The yellow colour indicates a top 50 DSI-ranked gene.

Next, we mapped the top 50 DSI- and NDSI-ranked genes to KEGG cancer pathways. Figure 5A and Table 2 together suggest that top DSI-ranked genes comprise the EGFR/ERBB2/FGFR3-KRAS/NRAS/HRAS-BRAF-MYC pathway, PIK3CA-PTEN pathway, CTNNB1-MYC pathway, TP53-CDKN2A-RB1 pathway and MYC-CUL1-RB1 pathway. Figure 5B and Table 2 together suggest that top NDSI-ranked genes comprise the EGFR/FGFR2/GNAQ/GNA11-NRAS/HRAS/KRAS-BRAF pathway, AKT1-MTOR pathway, and TCEB1-VHL-HIF1A pathway.

**Figure 6:**
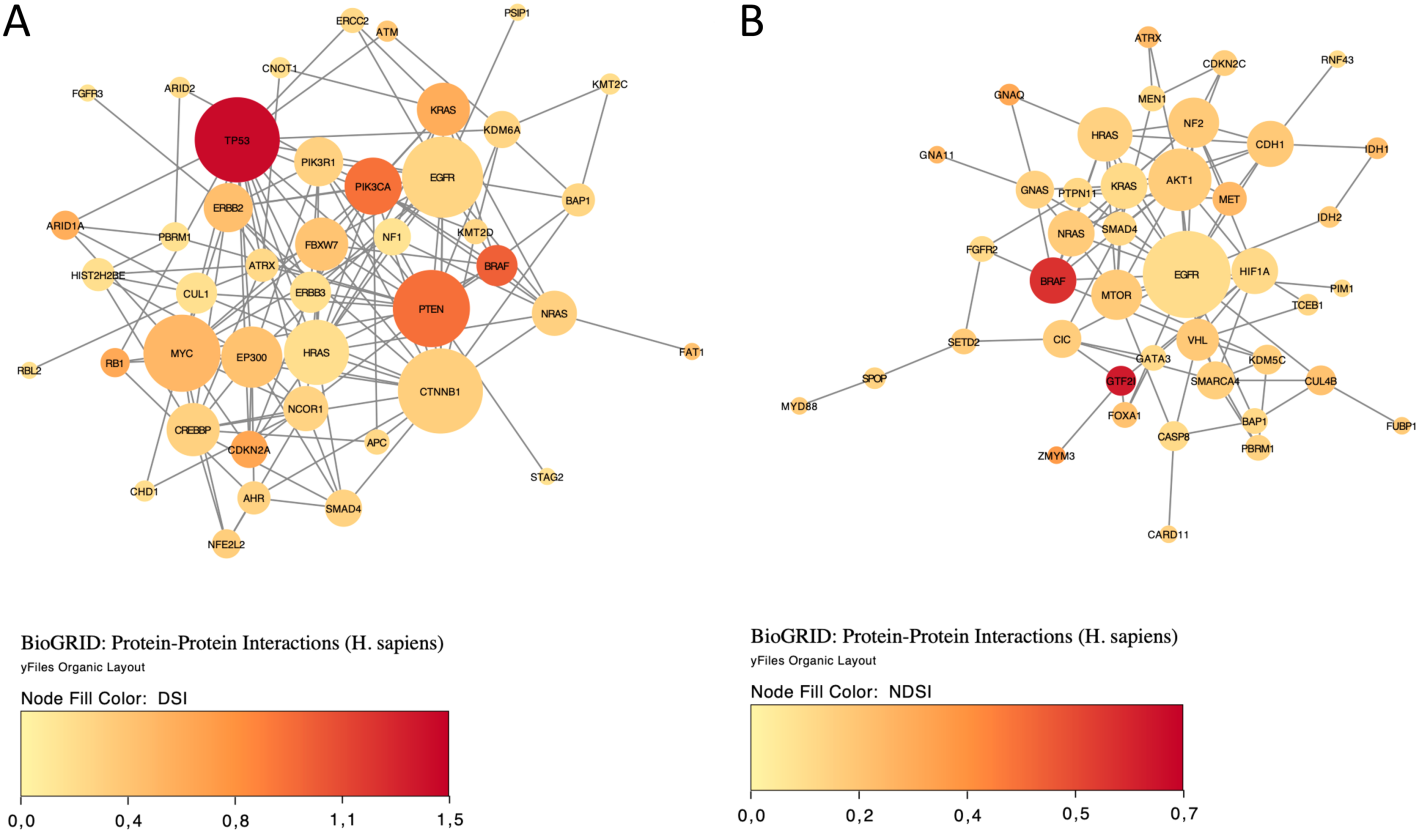
The BioGRID protein-protein interactions network of top 50 DSI-(A) and NDSI-(B) ranked genes. The node colour is mapped to DSI values and the node size is mapped to the degree of connectedness. Genes without connections were removed.

Finally, we explored protein-protein interactions in the top 50 DSI- and NDSI-ranked genes using the BioGRID network. Figure 6A shows that although *CTNNB1* and *EGFR* are the biggest hubs of the top-DSI-ranked gene network, their DSI values are much lower than those of *BRAF* and *PTEN*, which have fewer connections. Notably, *TP53* exhibited the highest DSI value and second-highest connectedness. Similarly, Fig. 6B shows that although *EGFR, AKT1* and *HRAS* are the biggest, centrally located hubs of the top-NDSI-ranked gene network, their NDSI values are much lower than those of *GTF2I*, *BRAF* and *ZMYM3*, located on the periphery of the network. Moreover, the top-NDSI-ranked gene network has much fewer edges than the top-DSI-ranked gene network, despite containing presumably stronger drivers. All of this supports our initially proposed notion that network centrality does not equal driver strength.

## Discussion

NDSI places *GTF2I* on the top spot both amongst the strongest SNA-based oncogenes and amongst the strongest drivers averaged across all classes. The *GTF2I*-encoded protein binds to the initiator element (Inr) and E-box element in promoters and functions as a regulator of transcription. The *GTF2I* c.74146970 T > A mutation was detected in 82% of type A and 74% of type AB thymomas (Petrini et al., 2014). *GTF2I* β and δ isoforms are expressed in thymomas, and both mutant isoforms are able to stimulate cell proliferation *in vitro* (Petrini et al., 2014). Recently, it has been shown that expression of mutant *GTF2I* alters the transcriptome of normal thymic epithelial cells and upregulates several oncogenic genes (Kim et al., 2020). *GTF2I* L424H knockin cells exhibit cell transformation, aneuploidy, and increased tumour growth and survival under glucose deprivation or DNA damage (Kim et al., 2020). Our analysis also shows frequent mutations of *GTF2I* in the TCGA THYM (thymoma) cohort. *GTF2I* has been recently named gene of the month and its role in cancer reviewed (Nathany, Tripathi & Mehta, 2021).

*SPOP* is categorized by NDSI as the strongest CNA-based oncogene. *SPOP* encodes a protein that is a component of a cullin-RING-based E3 ubiquitin-protein ligase complex that mediates the ubiquitination of target proteins, leading most often to their proteasomal degradation. *SPOP* is the most commonly mutated gene in primary prostate cancer (Barbieri et al., 2012). *SPOP* mutations in prostate cancer result in impaired homology-directed repair of double strand breaks and are associated with genomic instability (Boysen et al., 2015). As most cancer-associated mutations in *SPOP* are missense and almost none are frameshift or nonsense, PALDRIC classifies it as an oncogene. However, *SPOP* is usually viewed as a tumour suppressor (Clark & Burleson, 2020). Recently it has been discussed that SPOP actually has a dual role, and while being a tumour suppressor in prostate cancer it performs as an oncogene in kidney cancer (Wang et al., 2020). Indeed, cytoplasmic accumulation of SPOP leads to the ubiquitination and degradation of multiple regulators of cellular proliferation and apoptosis, including the tumour suppressor PTEN, ERK phosphatases, the proapoptotic molecule DAXX, and the Hedgehog pathway transcription factor GLI2, and is sufficient to induce tumorigenesis in clear cell renal cell carcinoma (Li et al., 2014). Our analysis shows frequent mutations and amplifications of *SPOP* in TCGA PRAD (prostate adenocarcinoma) and UCEC (uterine *corpus* endometrial carcinoma) cohorts.

*MET* is the strongest mixed (SNA+CNA) oncogene and the second-strongest CNA-based oncogene, according to the NDSI rating. *MET* encodes a receptor tyrosine kinase that transduces signals from the extracellular space into the cytoplasm by binding to the hepatocyte growth factor ligand. MET regulates many physiological processes including proliferation, morphogenesis and survival. Ligand binding at the cell surface induces dimerization and autophosphorylation of MET on its intracellular domain that provides docking sites for downstream signalling molecules. Following activation by its ligand, MET interacts with the PI3-kinase subunit PIK3R1, PLCG1, SRC, GRB2, STAT3 or the adapter GAB1. Recruitment of these downstream effectors by MET leads to the activation of several signalling cascades including the RAS-ERK, PI3K-AKT, or PLCG-PKC. Mutations in *MET* are associated with papillary renal cell carcinoma, hepatocellular carcinoma, and various head and neck cancers. Amplification and overexpression of this gene are also associated with multiple human cancers (Comoglio, Trusolino & Boccaccio, 2018; Recondo et al., 2020). Our analysis shows frequent mutations and amplifications of *MET* in TCGA KIRP (kidney renal papillary cell carcinoma) and LUAD (lung adenocarcinoma) cohorts.

*ATRX* is ranked by NDSI as the strongest SNA-based tumour suppressor, 9^th^ strongest CNA-based tumour suppressor, 5^th^ strongest mixed (SNA+CNA) tumour suppressor and 9^th^ strongest driver averaged across all classes. *ATRX* (Alpha-Thalassemia/Mental Retardation Syndrome, X-Linked) encodes a protein that contains an ATPase/helicase domain, and thus it belongs to the SWI/SNF family of chromatin remodelling proteins. *ATRX* together with *DAXX* encode a complex that deposits the histone variant H3.3 into repetitive heterochromatin, including retrotransposons, pericentric heterochromatin, and telomeres, the latter of which show deregulation in *ATRX/DAXX*-mutant tumours (Heaphy et al., 2011; Dyer et al., 2017). *ATRX* loss induces extensive changes in chromatin accessibility in both repetitive DNA regions and non-repetitive regulatory regions (Liang et al., 2020). These changes are highly correlated with changes in transcription, which lead to alterations in cancer-related signalling pathways, such as upregulation of the TGF-β pathway and downregulation of the cadherin family of proteins (Liang et al., 2020). Our analysis shows frequent mutations and deletions of *ATRX* in TCGA ACC, GBM, LGG and SARC cohorts.

*CSDE1* is ranked by NDSI as the strongest CNA-based tumour suppressor and 5^th^ strongest driver averaged across all classes. *CSDE1* encodes for an RNA-binding protein involved in translationally coupled mRNA turnover. CSDE1 not only promotes and represses the translation of RNAs but also increases and decreases the abundance of RNAs (Guo et al., 2020). *CSDE1* loss-of-function mutations and deletions define a Wnt-altered subtype of pheochromocytomas and paragangliomas (Fishbein et al., 2017). Our analysis also shows frequent deletions of *CSDE1* in the TCGA PCPG (pheochromocytoma and paraganglioma) cohort.

*NF2* is ranked by NDSI as the strongest mixed tumour suppressor and 3^rd^ strongest CNA-based tumour suppressor. *NF2* encodes Merlin (Moesin-ezrin-radixin-like protein), also known as Neurofibromin 2 and Schwannomin. Merlin is a tumour suppressor classically known for its ability to induce contact-dependent growth inhibition (Mota & Shevde, 2020). Loss-of-function mutations or deletions in *NF2* cause neurofibromatosis type 2, a multiple tumour forming disease of the nervous system, characterized by the development of bilateral schwannomas, as well as meningiomas and ependymomas (Petrilli & Fernández-Valle, 2016). *NF2* is also mutated and deleted in mesotheliomas (Sekido et al., 1995), clear cell renal cell carcinomas (Dalgliesh et al., 2010), collecting duct carcinomas of the kidney (Pal et al., 2016), and renal cell carcinomas with sarcomatoid dedifferentiation (Malouf et al., 2016). Our analysis shows frequent mutations and deletions of *NF2* in TCGA KIRC (kidney renal clear cell carcinoma), KIRP (kidney renal papillary cell carcinoma) and MESO (mesothelioma) cohorts.

Interestingly, NDSI prioritized three members of the guanine nucleotide-binding protein (G protein) family: *GNAQ*, *GNA11*, and *GNAS*. Guanine nucleotide-binding proteins function as transducers downstream of G protein-coupled receptors (GPCRs) in numerous signalling cascades. The alpha chain contains the guanine nucleotide binding site and alternates between an active, GTP-bound state and an inactive, GDP-bound state. Signalling by an activated GPCR promotes GDP release and GTP binding. The alpha subunit has a low GTPase activity that converts bound GTP to GDP, thereby terminating the signal. The *GNAQ*-encoded protein, an α subunit in the Gq class, couples a seven-transmembrane-domain receptor to activation of PLC β. Some *GNAQ* cancer mutants display normal basal activity and GPCR-mediated activation, but deactivate slowly due to GTPase activating protein (GAP) insensitivity (Garcia-Marcos, Maziarz & Leyme, 2018). *GNAQ* mutations occur in about half of uveal melanomas, representing the most common known oncogenic mutation in this cancer (Onken et al., 2008). The presence of this mutation in tumours at all stages of malignant progression suggests that it is an early event in uveal melanoma (Onken et al., 2008). Mutations affecting Q209 in *GNAQ* were present in 45% of primary uveal melanomas and 22% of uveal melanoma metastases (Van Raamsdonk et al., 2010). Our analysis also shows frequent mutations of *GNAQ* in the TCGA UVM (uveal melanoma) cohort. Recently, of the 11,111 patients screened, 117 patients have been found to harbour *GNAQ/GNA11* mutations, in melanoma, colorectal, liver, glioma, lung, bile duct and gastric cancers (Yang et al., 2020). *GNA11* encodes subunit α-11 in the Gq class and acts as an activator of PLC. Mutations affecting Q209 in *GNA11* were present in 32% of primary uveal melanomas and 57% of uveal melanoma metastases (Van Raamsdonk et al., 2010). Our analysis also shows frequent mutations of *GNA11* in the TCGA UVM (uveal melanoma) cohort. *GNAS* encodes subunit α in the Gs class and participates in the activation of adenylyl cyclases, resulting in increased levels of the signalling molecule cAMP. GNAS functions downstream of several GPCRs, including beta-adrenergic receptors. *GNAS* mutations are found in 67% of intraductal papillary mucinous neoplasms and many associated pancreatic ductal adenocarcinomas (Takano et al., 2014). High GNAS expression in a breast tumour tissue showed a close correlation with the reduced overall survival, frequent distal metastasis, advanced clinical stage, stronger cell proliferation and enhanced cancer cell migration (Jin et al., 2019). Recently, it has been shown that GNAS promotes the development of small cell lung cancer *via* PKA (Coles et al., 2020). Our analysis shows frequent mutations and amplifications of *GNAS* in TCGA COAD (colon adenocarcinoma), LIHC (liver hepatocellular carcinoma) and READ (rectum adenocarcinoma) cohorts. The current knowledge on cancer-associated alterations of GPCRs and G proteins has been recently reviewed (Larribère & Utikal, 2020). Strikingly, approximately 36% of all drugs approved by the US Food and Drug Administration during the past three decades target GPCRs (Rask-Andersen, Almén & Schiöth, 2011).

Two members of isocitrate dehydrogenase family, *IDH1* and *IDH2*, appeared on the top 10 SNA-based oncogenic events list as ranked by NDSI. The protein encoded by *IDH1* is the NADP^+^-dependent isocitrate dehydrogenase found in the cytoplasm and peroxisomes. The cytoplasmic enzyme serves a significant role in cytoplasmic NADPH production. The protein encoded by *IDH2* is the NADP^+^-dependent isocitrate dehydrogenase found in the mitochondria. It plays a role in intermediary metabolism and energy production. The most frequent mutations R132 (*IDH1*) and R172 (*IDH2*) involve the active site and result in simultaneous loss of normal catalytic activity, the production of α-ketoglutarate, and gain of a new function, the production of 2-hydroxyglutarate (Yan et al., 2009; Ward et al., 2010; Yang et al., 2012; Bendahou et al., 2020). 2-hydroxyglutarate is structurally similar to α-ketoglutarate, and acts as an α-ketoglutarate antagonist to competitively inhibit multiple α-ketoglutarate–dependent dioxygenases, including both lysine histone demethylases and the 10-11 translocation family of DNA hydroxylases (Yang et al., 2012). Abnormal histone and DNA methylation are emerging as a common feature of tumours with *IDH1* and *IDH2* mutations and may cause altered stem cell differentiation and eventual tumorigenesis (Yang et al., 2012). In acute myeloid leukaemia, *IDH1* and *IDH2* mutations have been associated with the worse outcome, shorter overall survival, and normal karyotype (Marcucci et al., 2010). All the 1p19q co-deleted gliomas have mutations in *IDH1* or *IDH2* (Labussière et al., 2010). Our analysis shows frequent mutations of *IDH1* and *IDH2* in the TCGA LGG (lower grade glioma) cohort and frequent amplifications of *IDH1* in LIHC (liver hepatocellular carcinoma), as well as less frequent mutations and amplifications of *IDH1* in CHOL, GBM, PRAD and SKCM.

Two fibroblast growth factor receptors *FGFR2* and *FGFR3* appeared on the top 10 mixed oncogenic events list as ranked by NDSI. The extracellular region of these proteins, composed of three immunoglobulin-like domains, interacts with fibroblast growth factors, leading to the activation of a cytoplasmic tyrosine kinase domain that phosphorylates PLCG1, FRS2 and other proteins. This sets in motion a cascade of downstream signals, including RAS-MAPK and PI3K-AKT pathways, ultimately influencing cell proliferation, differentiation, migration and apoptosis. FGFR aberrations were found in 7.1% of cancers, with the majority being gene amplification (66% of the aberrations), followed by mutations (26%) and rearrangements (8%) (Helsten et al., 2016). *FGFR1* was affected in 3.5% of 4,853 patients; *FGFR2* in 1.5%; *FGFR3* in 2.0%; and *FGFR4* in 0.5% (Helsten et al., 2016). The cancers most commonly affected were urothelial (32% FGFR-aberrant), breast (18%), endometrial (∼13%), squamous lung (∼13%) and ovarian (∼9%) (Helsten et al., 2016). Our analysis also shows frequent mutations and amplifications of *FGFR2* in TCGA LUSC (lung squamous cell carcinoma) and UCEC (uterine corpus endometrial carcinoma) cohorts, as well as frequent mutations and amplifications of *FGFR3* in BLCA (bladder urothelial carcinoma), HNSC (head and neck squamous cell carcinoma) and LUSC (lung squamous cell carcinoma) cohorts.

Some of the drivers prioritized by NDSI are highly tissue-specific. For example, *GTF2I* is specific to thymomas, whereas *GNAQ* and *GNA11* are specific to uveal melanomas. As discussed above, in many cases a mutation in one of these genes is able to singlehandedly drive cancer in the corresponding tissue (Amaro et al., 2017). This undoubtedly makes them very strong drivers, despite being able to drive cancer in only one tissue type. A contrasting example would be *TP53* which is the most common cancer driver in many tissues, but its individual strength is estimated as relatively low by NDSI, as it requires many other drivers to cooperate for cell transformation. Thus, it is important to differentiate the “weak – strong” axis from the “tissue-specific – universal” axis, as these appear to be orthogonal.

A puzzling question that remains in cancer genomics is why mutations in a given driver gene are typically confined to one or a few cancer types, resulting in each cancer type having its own unique set of driver genes (Iranzo, Martincorena & Koonin, 2018). As mutations are supposed to happen randomly as a result of stochastic mutagenesis processes (Belikov, 2017; Belikov, Vyatkin & Leonov, 2021), it is logical to suggest that mutations in different tissues can affect the same genes. However, the same mutation can be selected for in some tissues and selected against in others (Levine, Jenkins & Copeland, 2019). This selection most likely depends on the tissue-specific epigenetic profiles and microenvironments of the cancer-initiating stem or progenitor cells (Hass, von der Ohe & Ungefroren, 2020; Shlyakhtina, Moran & Portal, 2021). Thus, investigating the interplay between stem cell mutations, epigenetic profiles and microenvironments in various tissues appears to be a promising and exciting avenue for future research.

While both DSI- and NDSI-ranked top 50 genes are significantly overrepresented in such Reactome categories as signalling by PTK6, ESR-mediated signalling, PIP3 activates AKT signalling, signalling by receptor tyrosine kinases, signalling by erythropoietin, RAF/MAP kinase cascade, generic transcription pathway, chromatin modifying enzymes, and diseases of signal transduction by growth factor receptors and second messengers, there are also multiple differences. Top 50 DSI-ranked genes are additionally overrepresented in signalling by NOTCH, signalling by WNT, signalling by TGF-beta receptor complex, mitotic cell cycle, meiosis, cell cycle checkpoints, DNA double-stand break repair, epigenetic regulation of gene expression, RNA polymerase I transcription, circadian clock, diseases of cellular senescence, diseases of programmed cell death, and cellular responses to stress. This suggests that although these pathways are frequently mutated in cancer, none of them possesses strong tumour-promoting activity on its own. On the other hand, top 50 NDSI-ranked genes are additionally overrepresented in GPCR downstream signalling, which suggests that although this pathway is mutated more rarely in cancer, it nevertheless has a very strong tumour-promoting activity. It is also peculiar why neither DSI- nor NDSI-ranked top 50 genes are significantly overrepresented in DNA replication, autophagy, and metabolism categories. This may indicate that the role of these processes in oncogenesis is overestimated.

The major signalling pathway activated by mutations in both top DSI- and top NDSI-ranked driver genes is the RAS-RAF pathway. Although the pathway can be triggered *via* mutations in *EGFR, FGFR, NRAS, HRAS, KRAS* and *BRAF* genes, all of which are in the top 50 of both DSI and NDSI rankings, it can be additionally engaged through mutations in the top DSI-ranked driver *ERBB2* and the top NDSI-ranked drivers *GNAQ* and *GNA11*. This suggests that *ERBB2* driver mutations occur more frequently but are weaker than *GNAQ* and *GNA11* driver mutations. Also, top DSI-ranked driver mutations affect the upper part of the PI3K-AKT-MTOR pathway *via* constitutive PIK3CA activation or PTEN inactivation, whereas top NDSI-ranked mutations affect the lower part of the pathway by activating AKT1 and MTOR. Similarly, this suggests that *PIK3CA* and *PTEN* driver mutations occur more frequently but are weaker than *AKT1* and *MTOR* driver mutations. Moreover, the CTNNB1-MYC pathway, TP53-CDKN2A-RB1 pathway and MYC-CUL1-RB1 pathway are engaged only by top DSI-ranked drivers, indicating their relative weakness in cancer promotion despite high frequency of mutation, whereas the TCEB1-VHL-HIF1A pathway—only by top NDSI-ranked drivers, suggesting that this pathway has very strong tumour-promoting potential whilst being mutated more rarely.

It is interesting to discuss how driver strength should be estimated for genes responsible for the mutator phenotype (Loeb, 2011). On one hand, it appears that mutations in genes responsible for DNA repair should be considered strong drivers as they initiate a cascade of further mutations, many of which occur in other driver genes, thus heavily promoting cancer. On the other hand, by our definition of driver strength, if a driver needs help from many other drivers it cannot be called strong. Thus, it may be more fitting to call them “trigger drivers”, as they trigger the activation of other necessary drivers, rather than call them strong drivers *per se*. This may explain why top NDSI-ranked genes are not enriched in the Reactome DNA repair pathways. Our algorithm “sees” that there are always too many other driver mutations alongside the DNA repair drivers, thus it classifies the latter as weak. It might be viewed as a pitfall of our method, but we think it just faithfully follows the definition of what a strong driver really is, *i.e*. a driver able to drive cancer on its own or with a couple more drivers, as opposed to a “trigger driver” which recruits many additional helper drivers.

Another interesting aspect to discuss is the relation of driver strength to the evolutionary history of cancer. *i.e*. the sequential order of appearance of driver mutations. While our methodology does not allow deciphering the order of driver mutations, this subject was investigated, for example, in a recent article by Gerstung et al. (2020). There appears to be some indication that strong drivers (according to our methodology) overlap with early drivers (according to their methodology). For example, in Fig. 2B they show that, amongst the 50 most recurrent driver lesions, *SMARCA4* and *NF1* have the highest significant odds ratio (>50) of early *versus* late clonal driver mutations, and *SPOP* has the highest significant odds ratio (>50) of clonal *vs*. subclonal driver mutations. In our results (Table 2), *NF1* is the 50th top DSI-ranked gene, whereas *NF2* (not shown in Gerstung et al. (2020)), *SMARCA4* and *SPOP* are the 17th, 27th and 35th top NDSI-ranked genes. Many other significantly early/clonal genes—*KRAS, VHL, BRAF, PBRM1, SETD2, NRAS, MEN1, IDH1, ATRX*—were also found amongst the top 50 NDSI-ranked genes. Thus, despite the bias for recurrence in the Gerstung et al. (2020) figure, many of these genes were recovered in recurrence-independent ranking by NDSI. This suggests that strong drivers might indeed be activated early, which fits with our definition that strong drivers are able to initiate cancer either on their own or with little help.

Finally, it is important to note that our approach is not meant to replace frequency-based ranking, because it serves a different purpose. While the frequency-based approach identifies the most common drivers, our method identifies the strongest ones.

## Conclusions

We have introduced a novel concept of cancer driver strength, formulated algebraic equations for Driver Strength Indices, wrote software to calculate these indices and applied it to TCGA PanCanAtlas datasets. As a result, we presented a comprehensive overview on the landscape of cancer driver genes and chromosomes in TCGA PanCanAtlas patients and highlighted particular genes, gene families and pathways deemed strong drivers according to our Driver Strength Indices. These findings should help to direct future research efforts and selection of promising targets for novel therapeutics.

## Supplemental Information

10.7717/peerj.13860/supp-1 Supplemental Information 1 Supplemental methods.

10.7717/peerj.13860/supp-2 Supplemental Information 2 Output from PALDRIC GENE algorithm.

10.7717/peerj.13860/supp-3 Supplemental Information 3 GECNAV package.

10.7717/peerj.13860/supp-4 Supplemental Information 4 ANDRIF package.

10.7717/peerj.13860/supp-5 Supplemental Information 5 SNADRIF package.

10.7717/peerj.13860/supp-6 Supplemental Information 6 PALDRIC GENE package.

## Additional Information and Declarations

### Data Availability

The following information was supplied regarding data availability: All code and descriptions for our pipelines are available at the following GitHub repositories: https://github.com/belikov-av/SNADRIF. https://github.com/belikov-av/ANDRIF. https://github.com/belikov-av/GECNAV. https://github.com/belikov-av/PALDRIC_GENE. These packages contain scripts to automatically download all required source data from TCGA.  The versions of these packages used to generate data and figures for this article, as well as raw outputs from PALDRIC GENE algorithm, are available as Supplemental files.
