# Supplementary material for: Novel Driver Strength Index highlights important cancer genes in TCGA PanCanAtlas patients
Source: PeerJ. 2022 Aug 11;10:e13860. doi: 10.7717/peerj.13860 (PMC9375969; doi:10.7717/peerj.13860)
Supplement: Supplemental Information 1 [file peerj-10-13860-s001.pdf]

SUPPLEMENTAL METHODS

for the *PeerJ* article

Novel Driver Strength Index highlights important cancer genes in TCGA PanCanAtlas patients

Aleksey V. Belikov*, Alexey D. Vyatkin and Sergey V. Leonov

Laboratory of Innovative Medicine, School of Biological and Medical Physics, Moscow Institute of Physics and Technology, Dolgoprudny, Moscow Region, Russia

*Corresponding author: belikov.research@gmail.com

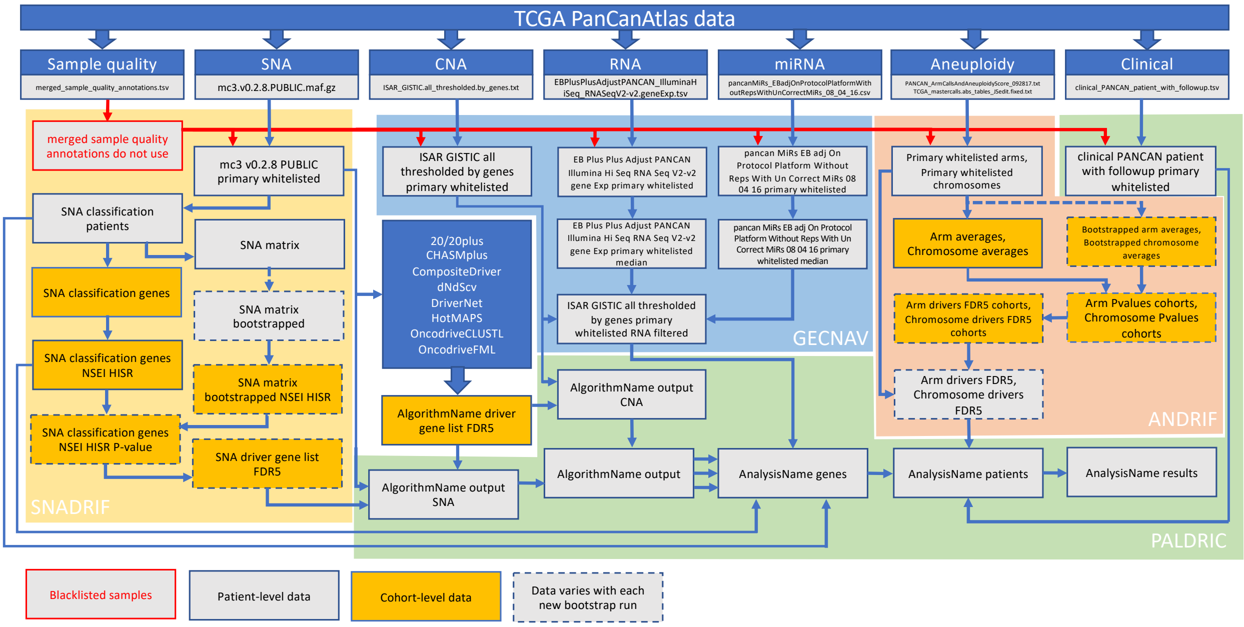

Most of the methodology except for the PALDRIC modification enabling the calculation of driver strength indices and pathway and network analysis of top-ranked driver genes has been previously published in [1].

<u>Source files and initial filtering</u>

TCGA PanCanAtlas [2] data were used. Files “Analyte level annotations - merged_sample_quality_annotations.tsv”, “ABSOLUTE purity/ploidy file - TCGA_mastercalls.abs_tables_JSedit.fixed.txt“, “Aneuploidy scores and arm calls file - PANCAN_ArmCallsAndAneuploidyScore_092817.txt”, “Public mutation annotation file - mc3.v0.2.8.PUBLIC.maf.gz”, “gzipped ISAR-corrected GISTIC2.0 all_thresholded.by_genes file - ISAR_GISTIC.all_thresholded.by_genes.txt”, “RNA batch corrected matrix - EBPlusPlusAdjustPANCAN_IlluminaHiSeq_RNASeqV2.geneExp.tsv”, “miRNA batch corrected

matrix - pancanMiRs_EBadjOnProtocolPlatformWithoutRepsWithUnCorrectMiRs_08_04_16.csv", were downloaded from [3,4].

Using TCGA barcodes [5,6], all samples except primary tumors (barcoded 01, 03, 09) were removed from all files. Based on the information in the column "Do_not_use" in the file "Analyte level annotations - merged_sample_quality_annotations.tsv", all samples with "True" value were removed from all files. All samples with "Cancer DNA fraction" <0.5 or unknown or with "Subclonal genome fraction" >0.5 or unknown in the file "TCGA_mastercalls.abs_tables_JSedit.fixed.txt" were removed from the file "PANCAN_ArmCallsAndAneuploidyScore_092817.txt". Moreover, all samples without "PASS" value in the column "FILTER" were removed from the file "mc3.v0.2.8.PUBLIC.maf.gz" and zeros in the column "Entrez_Gene_Id" were replaced with actual Entrez gene IDs, determined from the corresponding ENSEMBL gene IDs in the column "Gene" and NCBI Gene database [7]. Filtered files were saved as "Primary_whitelisted_arms.tsv",
"mc3.v0.2.8.PUBLIC_primary_whitelisted_Entrez.tsv",
"ISAR_GISTIC.all_thresholded.by_genes_primary_whitelisted.tsv",
"EBPlusPlusAdjustPANCAN_IlluminaHiSeq_RNASeqV2-v2.geneExp_primary_whitelisted.tsv",
"pancanMiRs_EBadjOnProtocolPlatformWithoutRepsWithUnCorrectMiRs_08_04_16_primary_whitelisted.tsv".

<u>RNA filtering of CNAs</u>

Using the file "EBPlusPlusAdjustPANCAN_IlluminaHiSeq_RNASeqV2-v2.geneExp_primary_whitelisted.tsv", the median expression level for each gene across patients was determined. If the expression for a given gene in a given patient was below 0.05x median value, it was encoded as "-2", if between 0.05x and 0.75x median value, it was encoded as "-1", if between 1.25x and 1.75x median value, it was encoded as "1", if above 1.75x median value, it was encoded as "2", otherwise it was encoded as "0". The file was saved as "EBPlusPlusAdjustPANCAN_IlluminaHiSeq_RNASeqV2-v2.geneExp_primary_whitelisted_median.tsv." The same operations were performed with the file "pancanMiRs_EBadjOnProtocolPlatformWithoutRepsWithUnCorrectMiRs_08_04_16_primary_whitelisted.tsv", which was saved as "pancanMiRs_EBadjOnProtocolPlatformWithoutRepsWithUnCorrectMiRs_08_04_16_primary_whitelisted_median.tsv"

Next, the file "ISAR_GISTIC.all_thresholded.by_genes_primary_whitelisted.tsv" was processed according to the following rules: if the gene CNA status in a given patient was not zero and had the same sign as the gene expression status in the same patient (file "EBPlusPlusAdjustPANCAN_IlluminaHiSeq_RNASeqV2-v2.geneExp_primary_whitelisted_median.tsv" or "pancanMiRs_EBadjOnProtocolPlatformWithoutRepsWithUnCorrectMiRs_08_04_16_primary_whitelisted_median.tsv" for miRNA genes), then the CNA status value was replaced with the gene expression status value, otherwise it was replaced by zero. If the corresponding expression status for

a given gene was not found then its CNA status was not changed. The resulting file was saved as "ISAR_GISTIC.all_thresholded.by_genes_primary_whitelisted_RNAfiltered.tsv"

We named this algorithm GECNAV (Gene Expression-based CNA Validator) and created a Github repository [8]. The package used to generate data in this article is available as **Data S2**.

Aneuploidy driver prediction

Using the file "Primary_whitelisted_arms.tsv", the average alteration status of each chromosomal arm was calculated for each cancer type and saved as a matrix file "Arm_averages.tsv". By drawing statuses randomly with replacement (bootstrapping) from any cell of "Primary_whitelisted_arms.tsv", for each cancer type the number of statuses corresponding to the number of patients in that cancer type were generated and their average was calculated. The procedure was repeated 10000 times, the median for each cancer type was calculated and the results were saved as a matrix file "Bootstrapped_arm_averages.tsv".

P-value for each arm alteration status was calculated for each cancer type. To do this, first the alteration status for a given cancer type and a given arm in "Arm_averages.tsv" was compared to the median bootstrapped arm alteration status for this cancer type in "Bootstrapped_arm_averages.tsv". If the status in "Arm_averages.tsv" was higher than zero and the median in "Bootstrapped_arm_averages.tsv", the number of statuses for this cancer type in "Bootstrapped_arm_averages.tsv" that are higher than the status in "Arm_averages.tsv" was counted and divided by 5000. If the status in "Arm_averages.tsv" was lower than zero and the median in "Bootstrapped_arm_averages.tsv", the number of statuses for this cancer type in "Bootstrapped_arm_averages.tsv" that are lower than the status in "Arm_averages.tsv" was counted and divided by 5000, and marked with minus to indicate arm loss. Other values were ignored (cells left empty). The results were saved as a matrix file "Arm_Pvalues_cohorts.tsv".

For each cancer type, Benjamini–Hochberg procedure with FDR=5% was applied to P-values in "Arm_Pvalues_cohorts.tsv" and passing P-values were encoded as "DAG" (Driver arm gain) or "DAL" (Driver arm loss) if marked with minus. The other cells were made empty and the results were saved as a matrix file "Arm_drivers_FDR5_cohorts.tsv".

Alterations were classified according to the following rules: if the arm status in a given patient (file "Primary_whitelisted_arms.tsv") was "-1" and the average alteration status of a given arm in the same cancer type (file "Arm_drivers_FDR5_cohorts.tsv") was "DAL", then the alteration in the patient was classified as "DAL". If the arm status in a given patient was "1" and the average alteration status of a given arm in the same cancer type was "DAG", then the alteration in the patient was classified as "DAG". In all other cases an empty cell was written. The total number of DALs and DAGs was calculated, patients with zero drivers were removed, and the results were saved as a matrix file "Arm_drivers_FDR5.tsv".

Using the file "Primary_whitelisted_arms.tsv", the values for the whole chromosomes were calculated using the following rules: if both p- and q-arm statuses were "1" then the chromosome status was written as "1"; if both p- and q-arm statuses were "-1" then the chromosome status was written as "-1"; if at least one arm status was not known (empty cell) then the chromosome status was written as empty cell; in all other cases the chromosome status was written as "0". For one-arm chromosomes (13, 14, 15, 21, 22), their status equals the status of the arm. The resulting file was saved as "Primary_whitelisted_chromosomes.tsv".

The same procedures as described above for chromosomal arms were repeated for the whole chromosomes, with the resulting file "Chromosome_drivers_FDR5.tsv". Chromosome drivers were considered to override arm drivers, so if a chromosome had "DCL" (Driver chromosome loss) or "DCG" (Driver chromosome gain), no alterations were counted on the arm level, to prevent triple counting of the same event.

We named this algorithm ANDRIF (ANeuploidy DRIver Finder) and created a Github repository [9]. The package used to generate data in this article is available as **Data S3**.

<u>SNA driver prediction</u>

Using the file "mc3.v0.2.8.PUBLIC_primary_whitelisted_Entrez.tsv" all SNAs were classified according to the column "Variant_Classification". "Frame_Shift_Del", "Frame_Shift_Ins", "Nonsense_Mutation", "Nonstop_Mutation" and "Translation_Start_Site" were considered potentially inactivating; "De_novo_Start_InFrame", "In_Frame_Del", "In_Frame_Ins" and "Missense_Mutation" were considered potentially hyperactivating; "De_novo_Start_OutOfFrame" and "Silent" were considered passengers; the rest were considered unclear. The classification results were saved as the file "SNA_classification_patients.tsv", with columns "Tumor_Sample_Barcode", "Hugo_Symbol", "Entrez_Gene_Id", "Gene", "Number of hyperactivating SNAs", "Number of inactivating SNAs", "Number of SNAs with unclear role", "Number of passenger SNAs".

Using this file, the sum of all alterations in all patients was calculated for each gene. Genes containing only SNAs with unclear role (likely, noncoding genes) were removed, also from "SNA_classification_patients.tsv". Next, the Nonsynonymous SNA Enrichment Index (NSEI) was calculated for each gene as

$$NSEI = \frac{\textbf{Number of hyperactivating SNAs} + \textbf{Number of inactivating SNAs} + \mathbf{1}}{\textbf{Number of passenger SNAs} + \mathbf{1}}$$

and the Hyperactivating to Inactivating SNA Ratio (HISR) was calculated for each gene as

$$HISR = \frac{\textbf{Number of hyperactivating SNAs} + \mathbf{1}}{\textbf{Number of inactivating SNAs} + \mathbf{1}}$$

Genes for which the sum of hyperactivating, inactivating and passenger SNAs was less than 10 were removed to ensure sufficient precision of NSEI and HISR calculation, and the results were saved as "SNA_classification_genes_NSEI_HISR.tsv".

Using the file "SNA_classification_patients.tsv", the gene-patient matrix "SNA_matrix.tsv" was constructed, encoding the "Number of hyperactivating SNAs", "Number of inactivating SNAs", "Number of SNAs with unclear role" and "Number of passenger SNAs" as one number separated by dots (e.g. "2.0.1.1"). If data for a given gene were absent in a given patient, it was encoded as "0.0.0.0". By drawing statuses randomly with replacement (bootstrapping) from any cell of "SNA_matrix.tsv" 10000 times for each patient, the matrix file "SNA_matrix_bootstrapped.tsv" was created. The sums of statuses in "SNA_matrix_bootstrapped.tsv" were calculated for each iteration separately, and then the corresponding NSEI and HISR indices were calculated and the results were saved as "SNA_bootstrapped_NSEI_HISR.tsv". Null hypothesis P-values were calculated for each iteration as the number of NSEI values higher than a given iteration's NSEI value and divided by 10000. The histogram of bootstrapped p-values was plotted to check for the uniformity of null hypothesis p-value distribution.

P-value for each gene was calculated as the number of NSEI values in "SNA_bootstrapped_NSEI_HISR.tsv" higher than its NSEI value in "SNA_classification_genes_NSEI_HISR.tsv" and divided by 10000. The results were saved as "SNA_classification_genes_NSEI_HISR_Pvalues.tsv". Benjamini–Hochberg procedure with FDR(Q)=5% was applied to P-values in "SNA_classification_genes_NSEI_HISR_Pvalues.tsv", and genes that pass were saved as "SNA_driver_gene_list_FDR5.tsv".

We named this algorithm SNADRIF (SNA DRIver Finder) and created a Github repository [10]. The package used to generate data in this article is available as **Data S4**.

Driver prediction algorithms sources

Lists of driver genes and mutations predicted by various algorithms (**Table S1**) applied to PanCanAtlas data were downloaded from [11,12] (2020plus, CompositeDriver, DriverNet, HotMAPS, OncodriveFML), [13,14] (CHASMplus), as well as received by personal communication from Francisco Martínez-Jiménez, Institute for Research in Biomedicine, Barcelona, francisco.martinez@irbbarcelona.org (dNdScv, OncodriveCLUSTL, OncodriveFML). All genes and mutations with q-value > 0.05 were removed. Additionally, a consensus driver gene list from 26 algorithms applied to PanCanAtlas data was downloaded from [12] and COSMIC Cancer Gene Census (CGC) Tier 1 gene list was downloaded from [15,16]. Only genes affected by somatic SNAs and CNAs present in the TCGA cancer types were used for further analyses from the CGC list. Cancer type names in the CGC list were manually converted to the closest possible TCGA cancer type abbreviation. Entrez Gene IDs were identified for each gene using HUGO Symbol and NCBI Gene database [7].

Table S1. Driver prediction algorithms.

Name	Ref.	Repository	Level	Principles
20/20plus	[17]	https://github.com/KarchinLab/2020plus	gene	Machine learning, trained on Cancer Genome Landscapes (20/20 rule); Nonsynonymous/Synonymous, clustering, conservation (uses UCSC's 46-way vertebrate alignment and SNVBox), impact (uses VEST), network (uses BioGrid), expression, chromatin, replication (uses MutSigCV)
ANDRIF	[18]	https://github.com/belikov-av/ANDRIF	Chromosomal arm, chromosome	Recurrence
CHASMplus	[14]	https://github.com/KarchinLab/CHASMplus	mutation	Machine learning, trained on TCGA; clustering (uses HotMAPS 1D), conservation (uses UCSC Multiz-100-way and SNV box), network (uses Interactome Insider)
CompositeDriver	[12]	https://github.com/mil2041/CompositeDriver	gene	Recurrence, impact (uses FunSeq2)
dNdScv	[19]	https://github.com/im3sanger/dndscv	gene	Nonsynonymous/Synonymous
DriverNet	[20]	https://github.com/shahcompbio/drivernet<br><br>https://bioconductor.org/packages/release/bioc/html/DriverNet.html	gene	Network (uses MGSA and a human functional protein interaction network), impact (uses gene expression outliers)
HotMAPS	[21]	https://github.com/karchinlab/HotMAPS	mutation	3D clustering (uses Protein Data Bank and ModPipe)
OncodriveCLUSTL	[22]	http://bbglab.irbbarcelona.org/oncodriveclustl/analysis<br><br>https://bitbucket.org/bbglab/oncodriveclustl/src/master/	gene	Clustering
OncodriveFML	[23]	http://bbglab.irbbarcelona.org/oncodrivefml/analysis<br><br>https://bitbucket.org/bbglab/oncodrivefml/src/master/	gene	Recurrence, Impact (uses CADD and RNAsnp)
SNADRIF	[18]	https://github.com/belikov-av/SNADRIF	gene	Nonsynonymous/Synonymous
Bailey et al, 2018	[12]	https://www.cell.com/cell/fulltext/S0092-8674(18)30237-X	gene	Consensus driver gene list from 26 algorithms applied to PanCanAtlas data
COSMIC Cancer Gene Census (CGC)	[15]	https://cancer.sanger.ac.uk/cosmic/census?tier=1	gene	Manually curated list of cancer driver genes, current “gold standard”

Conversion of population-level data to patient-level data

For lists of driver *genes*, all entries from the file "mc3.v0.2.8.PUBLIC_primary_whitelisted_Entrez.tsv" were removed except those that satisfied the following conditions simultaneously: "Entrez Gene ID" matches the one in the driver list; cancer type (identified by matching "Tumor_Sample_Barcode" with "bcr_patient_barcode" and "acronym" in "clinical_PANCAN_patient_with_followup.tsv") matches "cohort" in the driver list or the driver list is for pancancer analysis; "Variant_Classification" column contains one of the following values: "De_novo_Start_InFrame", "Frame_Shift_Del", "Frame_Shift_Ins", "In_Frame_Del", "In_Frame_Ins", "Missense_Mutation", "Nonsense_Mutation", "Nonstop_Mutation", "Translation_Start_Site".

For lists of driver *mutations*, the procedures were the same, except that Ensembl Transcript ID and nucleotide/amino acid substitution were used for matching instead of Entrez Gene ID. These data (only columns "TCGA Barcode", "HUGO Symbol", "Entrez Gene ID") were saved as "AlgorithmName_output_SNA.tsv".

Additionally, all entries from the file "ISAR_GISTIC.all_thresholded.by_genes_primary_whitelisted.tsv" were removed except those that satisfied the following conditions simultaneously: "Locus ID" matches "Entrez Gene ID" in the driver list; cancer type (identified by matching Tumor Sample Barcode with "bcr_patient_barcode" and "acronym" in "clinical_PANCAN_patient_with_followup.tsv") matches "cohort" in the driver list or the driver list is for pancancer analysis; CNA values are "2", "1", "-1" or "-2". These data were converted from the matrix to a list format (with columns "TCGA Barcode", "HUGO Symbol", "Entrez Gene ID") and saved as "AlgorithmName_output_CNA.tsv".

Finally, the files "AlgorithmName_output_SNA.tsv" and "AlgorithmName_output_CNA.tsv" were combined, duplicate TCGA Barcode-Entrez Gene ID pairs were removed, and the results saved as "AlgorithmName_output.tsv".

Driver event classification and analysis

The file "Clinical with Follow-up - clinical_PANCAN_patient_with_followup.tsv" was downloaded from [24]. All patients with "icd_o_3_histology" different from XXXX/3 (primary malignant neoplasm) were removed, as well as all patients not simultaneously present in the following three files: "mc3.v0.2.8.PUBLIC_primary_whitelisted_Entrez.tsv", "ISAR_GISTIC.all_thresholded.by_genes_primary_whitelisted.tsv" and "Primary_whitelisted_arms.tsv". The resulting file was saved as "clinical_PANCAN_patient_with_followup_primary_whitelisted.tsv".

Several chosen "AlgorithmName_output.tsv" files were combined and all TCGA Barcode-Entrez Gene ID pairs not present in at least two output files were removed. Entries with TCGA Barcodes not present in "clinical_PANCAN_patient_with_followup_primary_whitelisted.tsv" were removed as well. Matching "Number of hyperactivating SNAs" and "Number of inactivating SNAs" for each TCGA Barcode-Entrez Gene ID pair were taken from the "SNA_classification_patients.tsv" file, in case of no match zeros were written. Matching HISR value was taken from "SNA_classification_genes_NSEI_HISR.tsv" for each Entrez Gene ID, in case of no match empty cell was left. Matching CNA status was taken from "ISAR_GISTIC.all_thresholded.by_genes_primary_whitelisted_RNAfiltered.tsv" for each TCGA Barcode-Entrez Gene ID pair, in case of no match zero was written.

Each TCGA Barcode-Entrez Gene ID pair was classified according to the **Table S2**:

Table S2. Driver event classification rules.

**Driver type**	**Number of nonsynonymous SNAs**	**Number of inactivating SNAs**	**HISR**	**CNA status**	**Count as … driver event(s)**
**SNA-based oncogene**	≥1	0	>5	0	1
**CNA-based oncogene**	0	0	>5	1 or 2	1
**Mixed oncogene**	≥1	0	>5	1 or 2	1
**SNA-based tumour suppressor**	≥1	≥0	≤5	0	1
**CNA-based tumour suppressor**	0	0	≤5	-1 or -2	1
**Mixed tumour suppressor**	≥1	≥0	≤5	-1 or -2	1
**Passenger**	0	0		0	0
**Low-probability driver**	All the rest				0

Results of this classification were saved as "AnalysisName_genes_level2.tsv".

Using this file, the number of driver events of each type was counted for each patient. Information on the number of driver chromosome and arm losses and gains for each patient was taken from the files "Chromosome_drivers_FDR5.tsv" and "Arm_drivers_FDR5.tsv". All patients not present in the files "AnalysisName_genes_level2.tsv", "Chromosome_drivers_FDR5.tsv" and "Arm_drivers_FDR5.tsv", but present in the file "clinical_PANCAN_patient_with_followup_primary_whitelisted.tsv", were added with zero values for the numbers of driver events. Information on the cancer type ("acronym"), gender ("gender"), age ("age_at_initial_pathologic_diagnosis") and tumor stage ("pathologic_stage", if no data then "clinical_stage", if no data then "pathologic_T", if no data then "clinical_T") was taken from the file

“clinical_PANCAN_patient_with_followup_primary_whitelisted.tsv”. The results were saved as “AnalysisName_patients.tsv”.

Using the file “AnalysisName_patients.tsv”, the number of patients with each integer total number of driver events from 0 to 100 was counted for each cancer type, also for males and females separately, and cumulative histograms were plotted. Using the same file “AnalysisName_patients.tsv”, the average number of various types of driver events was calculated for each cancer type, tumour stage, age group, as well as for patients with each total number of driver events from 1 to 100. Analyses were performed for total population and for males and females separately, and cumulative histograms were plotted for each file.

We named this algorithm PALDRIC (PAtient-Level DRIver Classifier) and created a Github repository [25].

We later developed a modification of PALDRIC that allows analysis and ranking of individual genes, chromosome arms and full chromosomes – PALDRIC GENE - and created a Github repository [26]. The package used to generate data in this article is available as **Data S5**.

Using the files “AnalysisName_genes_level2.tsv”, “Chromosome_drivers_FDR5.tsv” and “Arm_drivers_FDR5.tsv”, the names of individual genes, chromosome arms or full chromosomes affected by driver events of each type were catalogued for each patient. Information on the cancer type, gender, age and tumour stage was taken from the file “clinical_PANCAN_patient_with_followup_primary_whitelisted.tsv”. The results were saved as “AnalysisName_patients_genes.tsv”.

Using the file “AnalysisName_patients_genes.tsv”, the number of various types of driver events in individual genes, chromosome arms or full chromosomes was calculated for each cancer type, tumor stage, age group, as well as for patients with each total number of driver events from 1 to 100. Analyses were performed for total population and for males and females separately, and histograms of top 10 driver events in each class and overall were plotted for each group.

Driver Strength Index (DSI)

$$DSI_A = \sum_{i=1}^{100} \frac{p_{A\ i}}{i\, p_i}$$

and Normalized Driver Strength Index (NDSI)

$$NDSI_A = \frac{\sum_{i=1}^{100} \frac{p_{A\ i}}{i\, p_i}}{\sum_{i=1}^{100} \frac{p_{A\ i}}{p_i}}$$

were calculated, where $p_{A\ i}$ is a number of patients with a driver event in the gene/chromosome A amongst patients with i driver events in total; p_i is a number of patients with i driver events in total. To avoid contamination of NDSI-ranked driver event lists with very rare driver events and to

increase precision of the index calculation, all events that were present in less than 10 patients in each driver event class were removed. To compose the top-(N)DSI-ranked driver list, the lists of drivers from various classes were combined, and drivers with lower (N)DSI in case of duplicates and all drivers with NDSI<0.05 were removed.

Pathway and network analysis of top-(N)DSI-ranked driver genes

First, the chromosome arms and full chromosomes were removed from the top-(N)DSI-ranked driver lists, as external pathway and network analysis services can work only with genes.

Next, top 50 DSI-ranked genes and top 50 NDSI-ranked genes were selected, to facilitate proper comparison.

The resulting lists were uploaded as Entrez Gene IDs to “Reactome v77 Analyse gene list” tool [27,28]. Voronoi visualizations (Reacfoam) were exported as jpg files.

The resulting lists were also uploaded as Entrez Gene IDs to “KEGG Mapper – Color” tool [29,30], “hsa” Search mode was selected, default bgcolor assigned to “yellow”, search executed and the top result - “Pathways in cancer - Homo sapiens (human)” (hsa05200) was selected for mapping. The resulting images were exported as png files.

The data were also analyzed in Cytoscape 3.8.2 [31,32]. BioGRID: Protein-Protein Interactions (H. sapiens) network was imported and then (N)DSI values appended from the top 50 (N)DSI-ranked driver list. First, Degree Sorted Circle Layout was selected and genes not within the circle were removed. Node Fill Color was mapped to (N)DSI values with Continuous Mapping and Node Height and Width were mapped to degree.layout parameter (number of connections) with Continuous Mapping. Then, yFiles Organic Layout was selected and legend appended. The resulting images were exported as pdf files.

References

1. Vyatkin AD, Otnyukov DV, Leonov SV, Belikov AV. Comprehensive patient-level classification and quantification of driver events in TCGA PanCanAtlas cohorts. PLOS Genetics. 2022;18: e1009996. doi:10.1371/journal.pgen.1009996

2. The Cancer Genome Atlas (TCGA) consortium. Welcome to the Pan-Cancer Atlas. Cell Press. 2018. Available: https://www.cell.com/pb-assets/consortium/PanCancerAtlas/PanCani3/index.html

3. Supplemental Data for Cell-of-Origin Patterns Dominate the Molecular Classification of 10,000 Tumors from 33 Types of Cancer. NIH National Cancer Institute Genomic Data Commons; 2018. Available: https://gdc.cancer.gov/about-data/publications/PanCan-CellOfOrigin

4. Hoadley KA, Yau C, Hinoue T, Wolf DM, Lazar AJ, Drill E, et al. Cell-of-Origin Patterns Dominate the Molecular Classification of 10,000 Tumors from 33 Types of Cancer. Cell. 2018;173: 291-304.e6. doi:10.1016/j.cell.2018.03.022

5. TCGA barcode. Encyclopedia. NIH National Cancer Institute; 2021. Available: https://docs.gdc.cancer.gov/Encyclopedia/pages/TCGA_Barcode/

6. Sample Type Codes. NIH National Cancer Institute Genomic Data Commons; 2021. Available: https://gdc.cancer.gov/resources-tcga-users/tcga-code-tables/sample-type-codes

7. Gene Database. NCBI; 2021. Available: ftp://ftp.ncbi.nih.gov/gene/DATA/GENE_INFO/Mammalia/Homo_sapiens.gene_info.gz

8. Belikov AV, Vyatkin AD. GECNAV (Gene Expression-based CNA Validator). GitHub; 2021. Available: https://github.com/belikov-av/GECNAV

9. Belikov AV, Vyatkin AD. ANDRIF (ANeuploidy DRIver Finder). GitHub; 2021. Available: https://github.com/belikov-av/ANDRIF

10. Belikov AV, Otnyukov DV. SNADRIF (SNA DRIver Finder). GitHub; 2021. Available: https://github.com/belikov-av/SNADRIF

11. Supplemental data for Comprehensive Characterization of Cancer Driver Genes and Mutations. NIH National Cancer Institute Genomic Data Commons; 2018. Available: https://gdc.cancer.gov/about-data/publications/pancan-driver

12. Bailey MH, Tokheim C, Porta-Pardo E, Sengupta S, Bertrand D, Weerasinghe A, et al. Comprehensive Characterization of Cancer Driver Genes and Mutations. Cell. 2018;173: 371-385.e18. doi:10.1016/j.cell.2018.02.060

13. Collin Tokheim, Rachel Karchin. CHASMplus. GitHub; 2021. Available: https://karchinlab.github.io/CHASMplus/

14. Tokheim C, Karchin R. CHASMplus Reveals the Scope of Somatic Missense Mutations Driving Human Cancers. Cell Systems. 2019;9: 9-23.e8. doi:10.1016/j.cels.2019.05.005

15. Sondka Z, Bamford S, Cole CG, Ward SA, Dunham I, Forbes SA. The COSMIC Cancer Gene Census: describing genetic dysfunction across all human cancers. Nature Reviews Cancer. 2018;18: 696–705. doi:10.1038/s41568-018-0060-1

16. Cancer Gene Census Tier 1. COSMIC; 2021. Available: https://cancer.sanger.ac.uk/cosmic/census?tier=1

17. Tokheim CJ, Papadopoulos N, Kinzler KW, Vogelstein B, Karchin R. Evaluating the evaluation of cancer driver genes. Proc Natl Acad Sci USA. 2016;113: 14330 LP – 14335. doi:10.1073/pnas.1616440113

18. Vyatkin AD, Otnyukov DV, Leonov SV, Belikov AV. Comprehensive patient-level classification and quantification of driver events in TCGA PanCanAtlas cohorts. PLOS Genetics. 2022. doi:10.1371/journal.pgen.1009996

19. Martincorena I, Raine KM, Gerstung M, Dawson KJ, Haase K, Van Loo P, et al. Universal Patterns of Selection in Cancer and Somatic Tissues. Cell. 2017;171: 1029-1041.e21. doi:10.1016/j.cell.2017.09.042

20. Bashashati A, Haffari G, Ding J, Ha G, Lui K, Rosner J, et al. DriverNet: uncovering the impact of somatic driver mutations on transcriptional networks in cancer. Genome Biology. 2012;13: R124. doi:10.1186/gb-2012-13-12-r124

21. Tokheim C, Bhattacharya R, Niknafs N, Gygax DM, Kim R, Ryan M, et al. Exome-Scale Discovery of Hotspot Mutation Regions in Human Cancer Using 3D Protein Structure. Cancer Research. 2016;76: 3719 LP – 3731. doi:10.1158/0008-5472.CAN-15-3190

22. Arnedo-Pac C, Mularoni L, Muiños F, Gonzalez-Perez A, Lopez-Bigas N. OncodriveCLUSTL: a sequence-based clustering method to identify cancer drivers. Bioinformatics. 2019;35: 4788–4790. doi:10.1093/bioinformatics/btz501

23. Mularoni L, Sabarinathan R, Deu-Pons J, Gonzalez-Perez A, López-Bigas N. OncodriveFML: a general framework to identify coding and non-coding regions with cancer driver mutations. Genome Biology. 2016;17: 128. doi:10.1186/s13059-016-0994-0

24. Supplemental data for PanCanAtlas publications. NIH National Cancer Institute Genomic Data Commons; 2019. Available: https://gdc.cancer.gov/node/905/

25. Belikov AV, Otnyukov DV. PALDRIC (PAtient-Level DRIver Classifier). GitHub; 2021. Available: https://github.com/belikov-av/PALDRIC

26. Belikov AV, Otnyukov DV. PALDRIC GENE (PAtient-Level DRIver Classifier Gene version). GitHub; 2021. Available: https://github.com/belikov-av/PALDRIC_GENE

27. Analyse gene list. Reactome; 2021. Available: https://reactome.org/PathwayBrowser/#TOOL=AT

28. Fabregat A, Sidiropoulos K, Viteri G, Marin-Garcia P, Ping P, Stein L, et al. Reactome diagram viewer: data structures and strategies to boost performance. Bioinformatics (Oxford, England). 2018;34: 1208–1214. doi:10.1093/bioinformatics/btx752

29. Mapper – Color. KEGG; 2021. Available: https://www.genome.jp/kegg/mapper/color.html

30. Kanehisa M, Sato Y. KEGG Mapper for inferring cellular functions from protein sequences. Protein Science. 2020;29: 28–35. doi:10.1002/pro.3711

31. Cytoscape. NIGMS; 2021. Available: https://cytoscape.org

32. Shannon P, Markiel A, Ozier O, Baliga NS, Wang JT, Ramage D, et al. Cytoscape: A Software Environment for Integrated Models of Biomolecular Interaction Networks. Genome Research. 2003;13: 2498–2504. doi:10.1101/gr.1239303
