# Supplementary material for: Novel Driver Strength Index highlights important cancer genes in TCGA PanCanAtlas patients
Source: PeerJ. 2022 Aug 11;10:e13860. doi: 10.7717/peerj.13860 (PMC9375969; doi:10.7717/peerj.13860)
Supplement: Supplemental Information 2 [file peerj-10-13860-s002.zip › COHORTS/genes plots/indices/2021_8_16_14_9_distribution_events_detailed_NDSI_males.pdf]

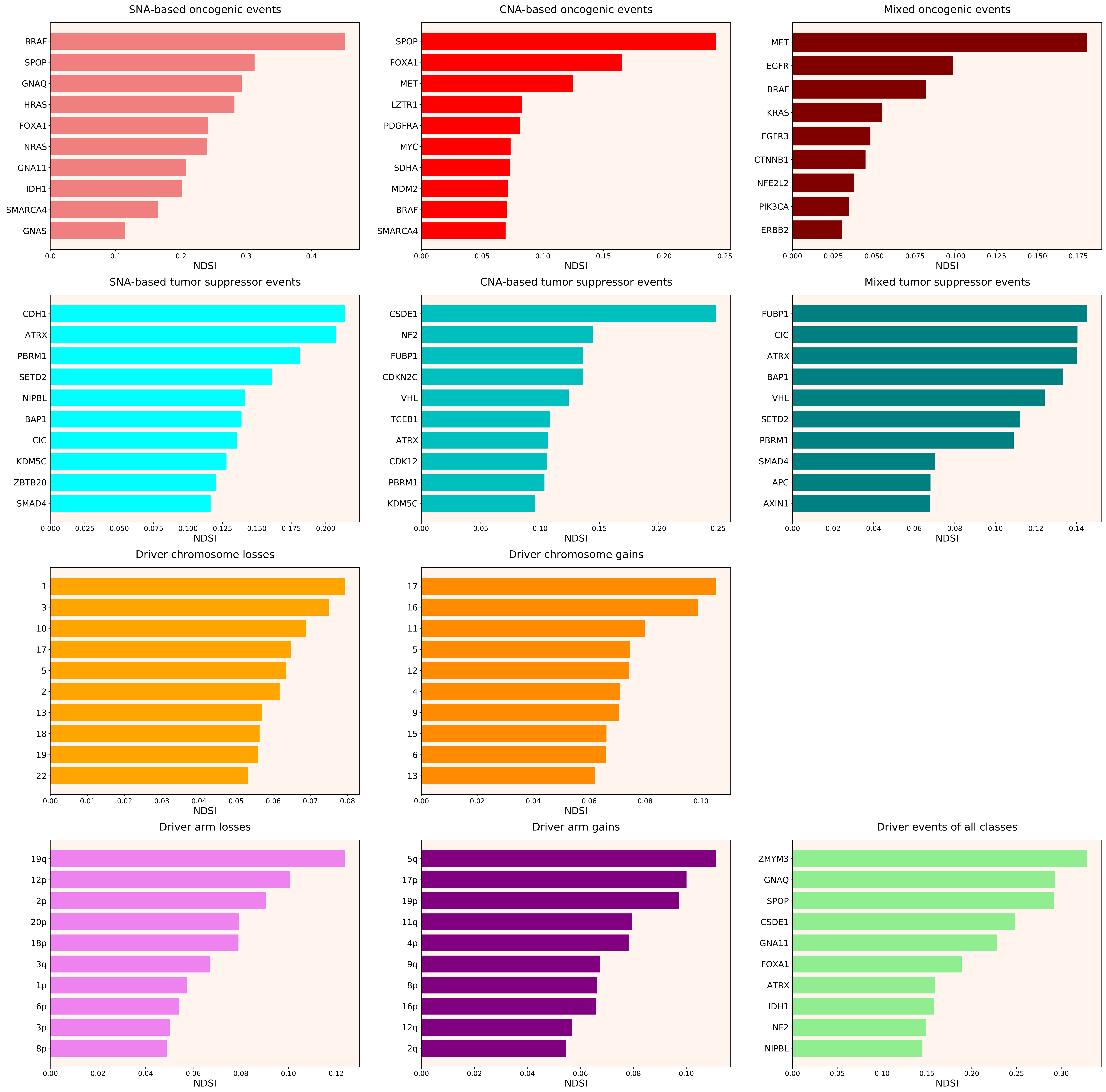

SNA-based oncogenic events
BRAF SPOP GNAQ HRAS FOXA1 NRAS GNA11 IDH1 SMARCA4 GNAS
CNA-based oncogenic events
SPOP FOXA1 MET LZTR1 PDGFRA MYC SDHA MDM2 BRAF SMARCA4
Mixed oncogenic events
MET EGFR BRAF KRAS FGFR3 CTNNB1 NFE2L2 PIK3CA ERBB2
SNA-based tumor suppressor events
CDH1 ATRX PBRM1 SETD2 NIPBL BAP1 CIC KDM5C ZBTB20 SMAD4
CNA-based tumor suppressor events
CSDE1 NF2 FUBP1 CDKN2C VHL TCEB1 ATRX CDK12 PBRM1 KDM5C
Mixed tumor suppressor events
FUBP1 CIC ATRX BAP1 VHL SETD2 PBRM1 SMAD4 APC AXIN1
Driver chromosome losses
1 3 10 17 5 2 13 18 19 22
Driver chromosome gains
17 16 11 5 12 4 9 15 6 13
Driver arm losses
19q 12p 2p 20p 18p 3q 1p 6p 3p 8p
Driver arm gains
5q 17p 19p 11q 4p 9q 8p 16p 12q 2q
Driver events of all classes
ZMYM3 GNAQ SPOP CSDE1 GNA11 FOXA1 ATRX IDH1 NF2 NIPBL
NDSI
