# Supplementary material for: Novel Driver Strength Index highlights important cancer genes in TCGA PanCanAtlas patients
Source: PeerJ. 2022 Aug 11;10:e13860. doi: 10.7717/peerj.13860 (PMC9375969; doi:10.7717/peerj.13860)
Supplement: Supplemental Information 2 [file peerj-10-13860-s002.zip › COHORTS/genes plots/indices/2021_8_16_14_9_distribution_events_detailed_DSI_females.pdf]

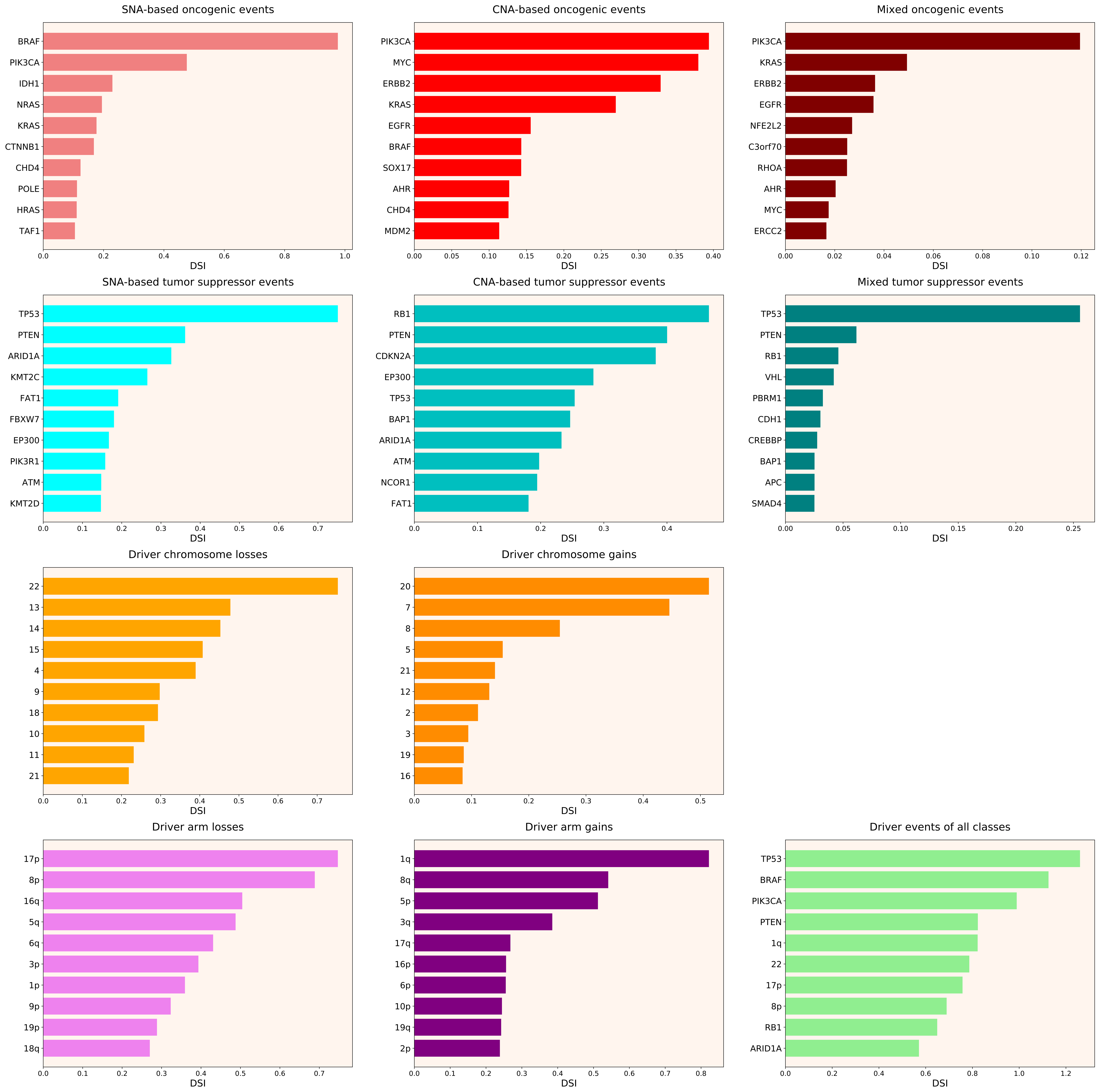
SNA-based oncogenic events
BRAF
PIK3CA
IDH1
NRAS
KRAS
CTNNB1
CHD4
POLE
HRAS
TAF1
DSI
CNA-based oncogenic events
PIK3CA
MYC
ERBB2
KRAS
EGFR
BRAF
SOX17
AHR
CHD4
MDM2
DSI
Mixed oncogenic events
PIK3CA
KRAS
ERBB2
EGFR
NFE2L2
C3orf70
RHOA
AHR
MYC
ERCC2
DSI
SNA-based tumor suppressor events
TP53
PTEN
ARID1A
KMT2C
FAT1
FBXW7
EP300
PIK3R1
ATM
KMT2D
DSI
CNA-based tumor suppressor events
RB1
PTEN
CDKN2A
EP300
TP53
BAP1
ARID1A
ATM
NCOR1
FAT1
DSI
Mixed tumor suppressor events
TP53
PTEN
RB1
VHL
PBRM1
CDH1
CREBBP
BAP1
APC
SMAD4
DSI
Driver chromosome losses
22
13
14
15
4
9
18
10
11
21
DSI
Driver chromosome gains
20
7
8
5
21
12
2
3
19
16
DSI
Driver arm losses
17p
8p
16q
5q
6q
3p
1p
9p
19p
18q
DSI
Driver arm gains
1q
8q
5p
3q
17q
16p
6p
10p
19q
2p
DSI
Driver events of all classes
TP53
BRAF
PIK3CA
PTEN
1q
22
17p
8p
RB1
ARID1A
DSI
