# Supplementary material for: Novel Driver Strength Index highlights important cancer genes in TCGA PanCanAtlas patients
Source: PeerJ. 2022 Aug 11;10:e13860. doi: 10.7717/peerj.13860 (PMC9375969; doi:10.7717/peerj.13860)
Supplement: Supplemental Information 2 [file peerj-10-13860-s002.zip › COHORTS/genes plots/indices/2021_8_16_14_9_distribution_events_detailed_DSI.pdf]

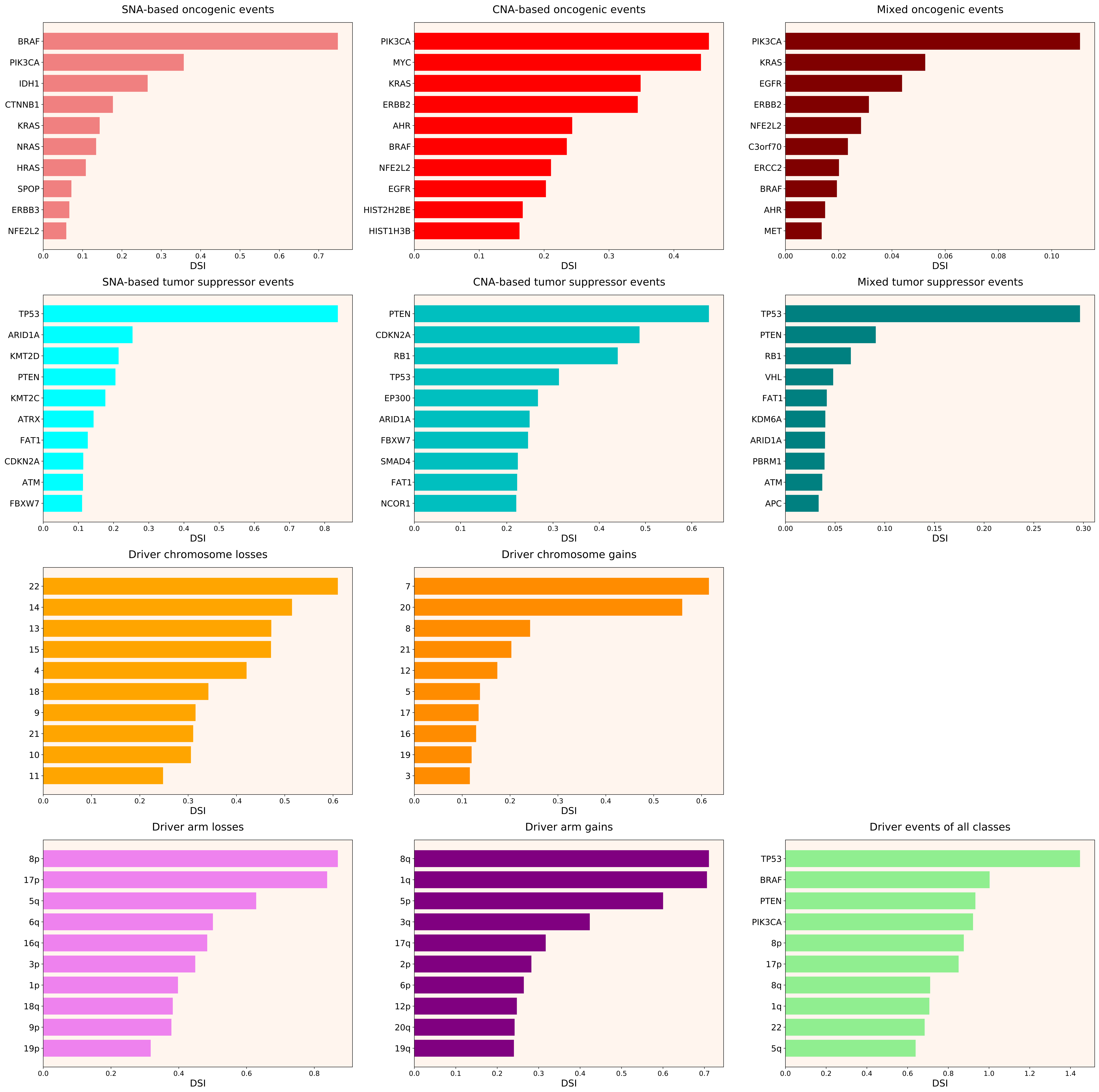

SNA-based oncogenic events
BRAF
PIK3CA
IDH1
CTNNB1
KRAS
NRAS
HRAS
SPOP
ERBB3
NFE2L2
DSI
CNA-based oncogenic events
PIK3CA
MYC
KRAS
ERBB2
AHR
BRAF
NFE2L2
EGFR
HIST2H2BE
HIST1H3B
DSI
Mixed oncogenic events
PIK3CA
KRAS
EGFR
ERBB2
NFE2L2
C3orf70
ERCC2
BRAF
AHR
MET
DSI
SNA-based tumor suppressor events
TP53
ARID1A
KMT2D
PTEN
KMT2C
ATRX
FAT1
CDKN2A
ATM
FBXW7
DSI
CNA-based tumor suppressor events
PTEN
CDKN2A
RB1
TP53
EP300
ARID1A
FBXW7
SMAD4
FAT1
NCOR1
DSI
Mixed tumor suppressor events
TP53
PTEN
RB1
VHL
FAT1
KDM6A
ARID1A
PBRM1
ATM
APC
DSI
Driver chromosome losses
22
14
13
15
4
18
9
21
10
11
DSI
Driver chromosome gains
7
20
8
21
12
5
17
16
19
3
DSI
Driver arm losses
8p
17p
5q
6q
16q
3p
1p
18q
9p
19p
DSI
Driver arm gains
8q
1q
5p
3q
17q
2p
6p
12p
20q
19q
DSI
Driver events of all classes
TP53
BRAF
PTEN
PIK3CA
8p
17p
8q
1q
22
5q
DSI
