# Supplementary material for: Novel Driver Strength Index highlights important cancer genes in TCGA PanCanAtlas patients
Source: PeerJ. 2022 Aug 11;10:e13860. doi: 10.7717/peerj.13860 (PMC9375969; doi:10.7717/peerj.13860)
Supplement: Supplemental Information 2 [file peerj-10-13860-s002.zip › COHORTS/genes plots/indices/2021_8_16_14_9_distribution_events_detailed_DSI_males.pdf]

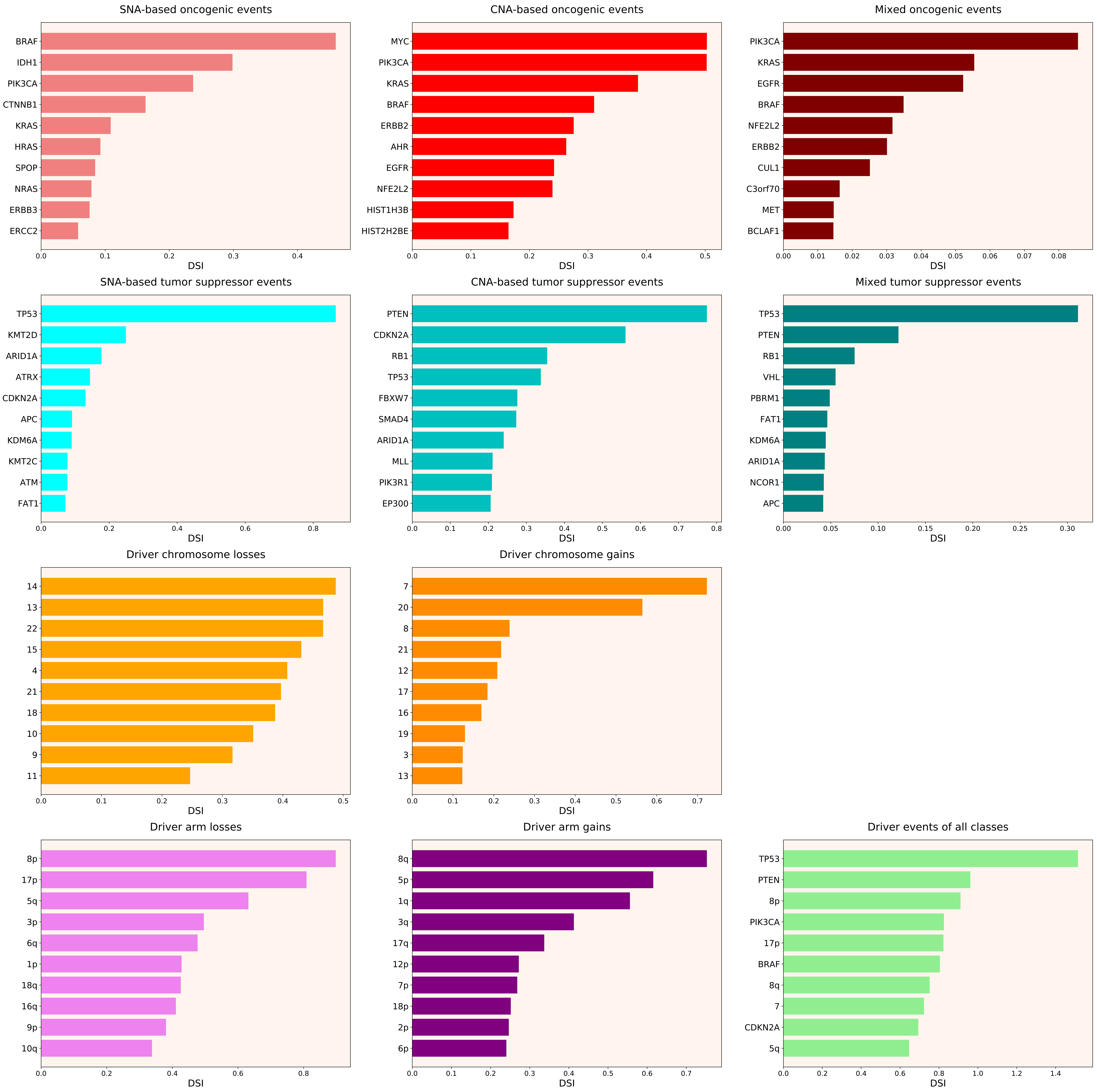
SNA-based oncogenic events
BRAF
IDH1
PIK3CA
CTNNB1
KRAS
HRAS
SPOP
NRAS
ERBB3
ERCC2
0.0 0.1 0.2 0.3 0.4
DSI
CNA-based oncogenic events
MYC
PIK3CA
KRAS
BRAF
ERBB2
AHR
EGFR
NFE2L2
HIST1H3B
HIST2H2BE
0.0 0.1 0.2 0.3 0.4 0.5
DSI
Mixed oncogenic events
PIK3CA
KRAS
EGFR
BRAF
NFE2L2
ERBB2
CUL1
C3orf70
MET
BCLAF1
0.00 0.01 0.02 0.03 0.04 0.05 0.06 0.07 0.08
DSI
SNA-based tumor suppressor events
TP53
KMT2D
ARID1A
ATRX
CDKN2A
APC
KDM6A
KMT2C
ATM
FAT1
0.0 0.2 0.4 0.6 0.8
DSI
CNA-based tumor suppressor events
PTEN
CDKN2A
RB1
TP53
FBXW7
SMAD4
ARID1A
MLL
PIK3R1
EP300
0.0 0.1 0.2 0.3 0.4 0.5 0.6 0.7 0.8
DSI
Mixed tumor suppressor events
TP53
PTEN
RB1
VHL
PBRM1
FAT1
KDM6A
ARID1A
NCOR1
APC
0.00 0.05 0.10 0.15 0.20 0.25 0.30
DSI
Driver chromosome losses
14
13
22
15
4
21
18
10
9
11
0.0 0.1 0.2 0.3 0.4 0.5
DSI
Driver chromosome gains
7
20
8
21
12
17
16
19
3
13
0.0 0.1 0.2 0.3 0.4 0.5 0.6 0.7
DSI
Driver arm losses
8p
17p
5q
3p
6q
1p
18q
16q
9p
10q
0.0 0.2 0.4 0.6 0.8
DSI
Driver arm gains
8q
5p
1q
3q
17q
12p
7p
18p
2p
6p
0.0 0.1 0.2 0.3 0.4 0.5 0.6 0.7
DSI
Driver events of all classes
TP53
PTEN
8p
PIK3CA
17p
BRAF
8q
7
CDKN2A
5q
0.0 0.2 0.4 0.6 0.8 1.0 1.2 1.4
DSI
