# Supplementary material for: Novel Driver Strength Index highlights important cancer genes in TCGA PanCanAtlas patients
Source: PeerJ. 2022 Aug 11;10:e13860. doi: 10.7717/peerj.13860 (PMC9375969; doi:10.7717/peerj.13860)
Supplement: Supplemental Information 2 [file peerj-10-13860-s002.zip › COHORTS/genes plots/detailed/2021_8_16_14_9_distribution_events_detailed_males_19.pdf]

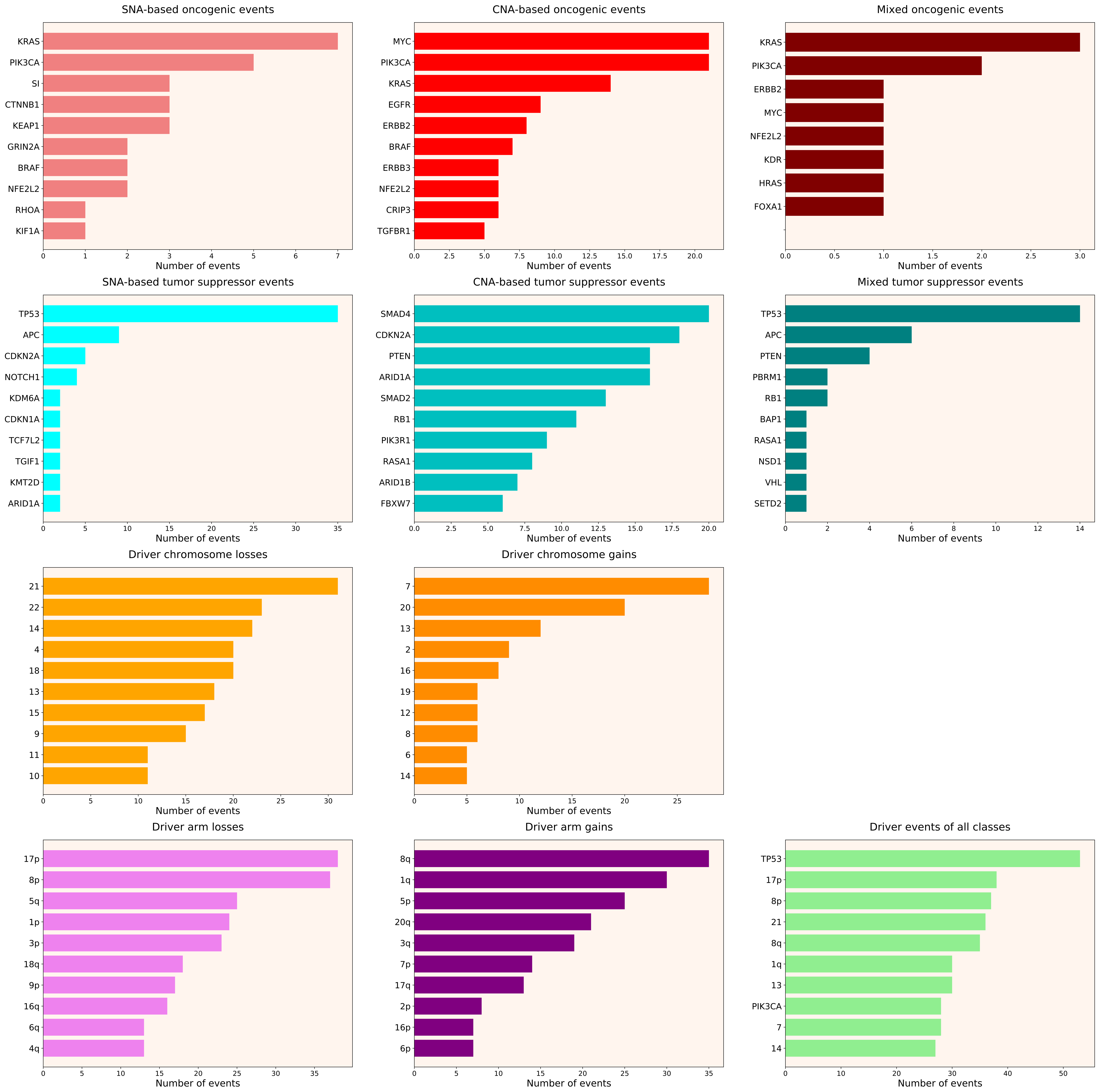

SNA-based oncogenic events
KRAS
PIK3CA
SI
CTNNB1
KEAP1
GRIN2A
BRAF
NFE2L2
RHOA
KIF1A
Number of events
CNA-based oncogenic events
MYC
PIK3CA
KRAS
EGFR
ERBB2
BRAF
ERBB3
NFE2L2
CRIP3
TGFBR1
Number of events
Mixed oncogenic events
KRAS
PIK3CA
ERBB2
MYC
NFE2L2
KDR
HRAS
FOXA1
Number of events
SNA-based tumor suppressor events
TP53
APC
CDKN2A
NOTCH1
KDM6A
CDKN1A
TCF7L2
TGIF1
KMT2D
ARID1A
Number of events
CNA-based tumor suppressor events
SMAD4
CDKN2A
PTEN
ARID1A
SMAD2
RB1
PIK3R1
RASA1
ARID1B
FBXW7
Number of events
Mixed tumor suppressor events
TP53
APC
PTEN
PBRM1
RB1
BAP1
RASA1
NSD1
VHL
SETD2
Number of events
Driver chromosome losses
21
22
14
4
18
13
15
9
11
10
Number of events
Driver chromosome gains
7
20
13
2
16
19
12
8
6
14
Number of events
Driver arm losses
17p
8p
5q
1p
3p
18q
9p
16q
6q
4q
Number of events
Driver arm gains
8q
1q
5p
20q
3q
7p
17q
2p
16p
6p
Number of events
Driver events of all classes
TP53
17p
8p
21
8q
1q
13
PIK3CA
7
14
Number of events
