# Supplementary material for: Novel Driver Strength Index highlights important cancer genes in TCGA PanCanAtlas patients
Source: PeerJ. 2022 Aug 11;10:e13860. doi: 10.7717/peerj.13860 (PMC9375969; doi:10.7717/peerj.13860)
Supplement: Supplemental Information 2 [file peerj-10-13860-s002.zip › COHORTS/genes plots/detailed/2021_8_16_14_9_distribution_events_detailed_males_31.pdf]

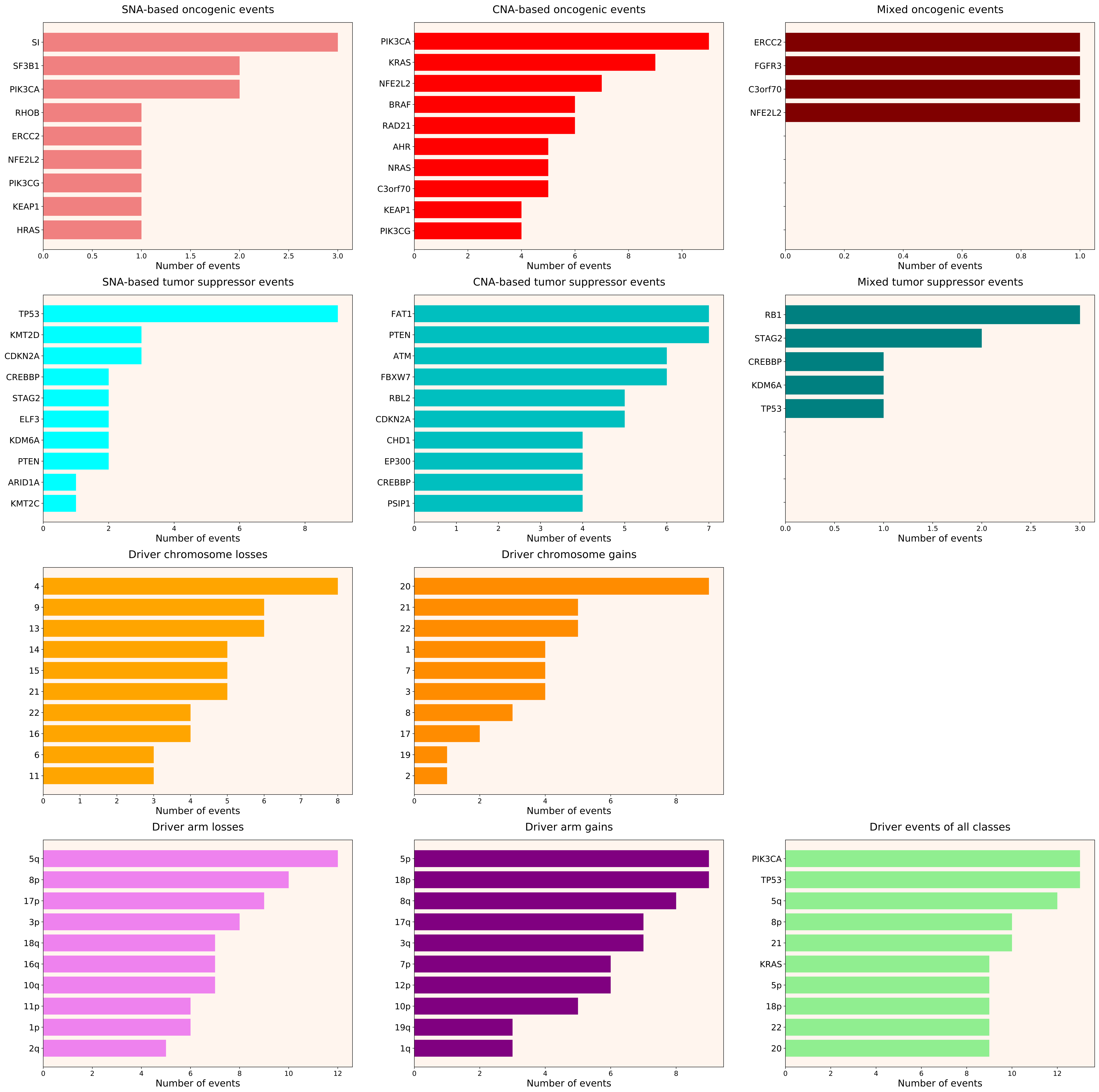
SNA-based oncogenic events
SI
SF3B1
PIK3CA
RHOB
ERCC2
NFE2L2
PIK3CG
KEAP1
HRAS
Number of events
CNA-based oncogenic events
PIK3CA
KRAS
NFE2L2
BRAF
RAD21
AHR
NRAS
C3orf70
KEAP1
PIK3CG
Number of events
Mixed oncogenic events
ERCC2
FGFR3
C3orf70
NFE2L2
Number of events
SNA-based tumor suppressor events
TP53
KMT2D
CDKN2A
CREBBP
STAG2
ELF3
KDM6A
PTEN
ARID1A
KMT2C
Number of events
CNA-based tumor suppressor events
FAT1
PTEN
ATM
FBXW7
RBL2
CDKN2A
CHD1
EP300
CREBBP
PSIP1
Number of events
Mixed tumor suppressor events
RB1
STAG2
CREBBP
KDM6A
TP53
Number of events
Driver chromosome losses
4
9
13
14
15
21
22
16
6
11
Number of events
Driver chromosome gains
20
21
22
1
7
3
8
17
19
2
Number of events
Driver arm losses
5q
8p
17p
3p
18q
16q
10q
11p
1p
2q
Number of events
Driver arm gains
5p
18p
8q
17q
3q
7p
12p
10p
19q
1q
Number of events
Driver events of all classes
PIK3CA
TP53
5q
8p
21
KRAS
5p
18p
22
20
Number of events
