# Supplementary material for: Novel Driver Strength Index highlights important cancer genes in TCGA PanCanAtlas patients
Source: PeerJ. 2022 Aug 11;10:e13860. doi: 10.7717/peerj.13860 (PMC9375969; doi:10.7717/peerj.13860)
Supplement: Supplemental Information 2 [file peerj-10-13860-s002.zip › COHORTS/genes plots/detailed/2021_8_16_14_9_distribution_events_detailed_females_21.pdf]

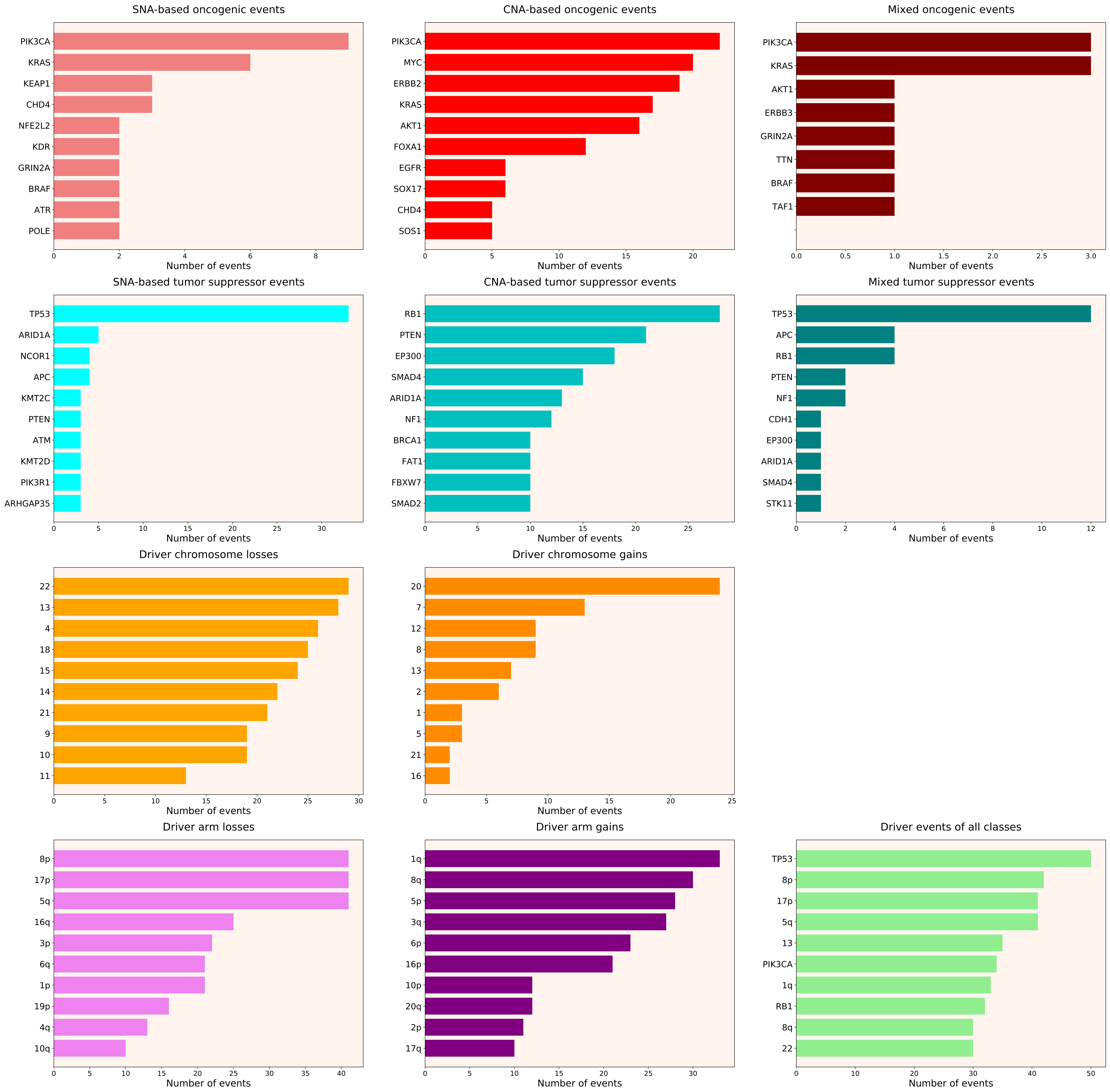
SNA-based oncogenic events
PIK3CA
KRAS
KEAP1
CHD4
NFE2L2
KDR
GRIN2A
BRAF
ATR
POLE
Number of events
CNA-based oncogenic events
PIK3CA
MYC
ERBB2
KRAS
AKT1
FOXA1
EGFR
SOX17
CHD4
SOS1
Number of events
Mixed oncogenic events
PIK3CA
KRAS
AKT1
ERBB3
GRIN2A
TTN
BRAF
TAF1
Number of events
SNA-based tumor suppressor events
TP53
ARID1A
NCOR1
APC
KMT2C
PTEN
ATM
KMT2D
PIK3R1
ARHGAP35
Number of events
CNA-based tumor suppressor events
RB1
PTEN
EP300
SMAD4
ARID1A
NF1
BRCA1
FAT1
FBXW7
SMAD2
Number of events
Mixed tumor suppressor events
TP53
APC
RB1
PTEN
NF1
CDH1
EP300
ARID1A
SMAD4
STK11
Number of events
Driver chromosome losses
22
13
4
18
15
14
21
9
10
11
Number of events
Driver chromosome gains
20
7
12
8
13
2
1
5
21
16
Number of events
Driver arm losses
8p
17p
5q
16q
3p
6q
1p
19p
4q
10q
Number of events
Driver arm gains
1q
8q
5p
3q
6p
16p
10p
20q
2p
17q
Number of events
Driver events of all classes
TP53
8p
17p
5q
13
PIK3CA
1q
RB1
8q
22
Number of events
