# Supplementary material for: Novel Driver Strength Index highlights important cancer genes in TCGA PanCanAtlas patients
Source: PeerJ. 2022 Aug 11;10:e13860. doi: 10.7717/peerj.13860 (PMC9375969; doi:10.7717/peerj.13860)
Supplement: Supplemental Information 2 [file peerj-10-13860-s002.zip › COHORTS/genes plots/detailed/2021_8_16_14_9_distribution_events_detailed_females_20.pdf]

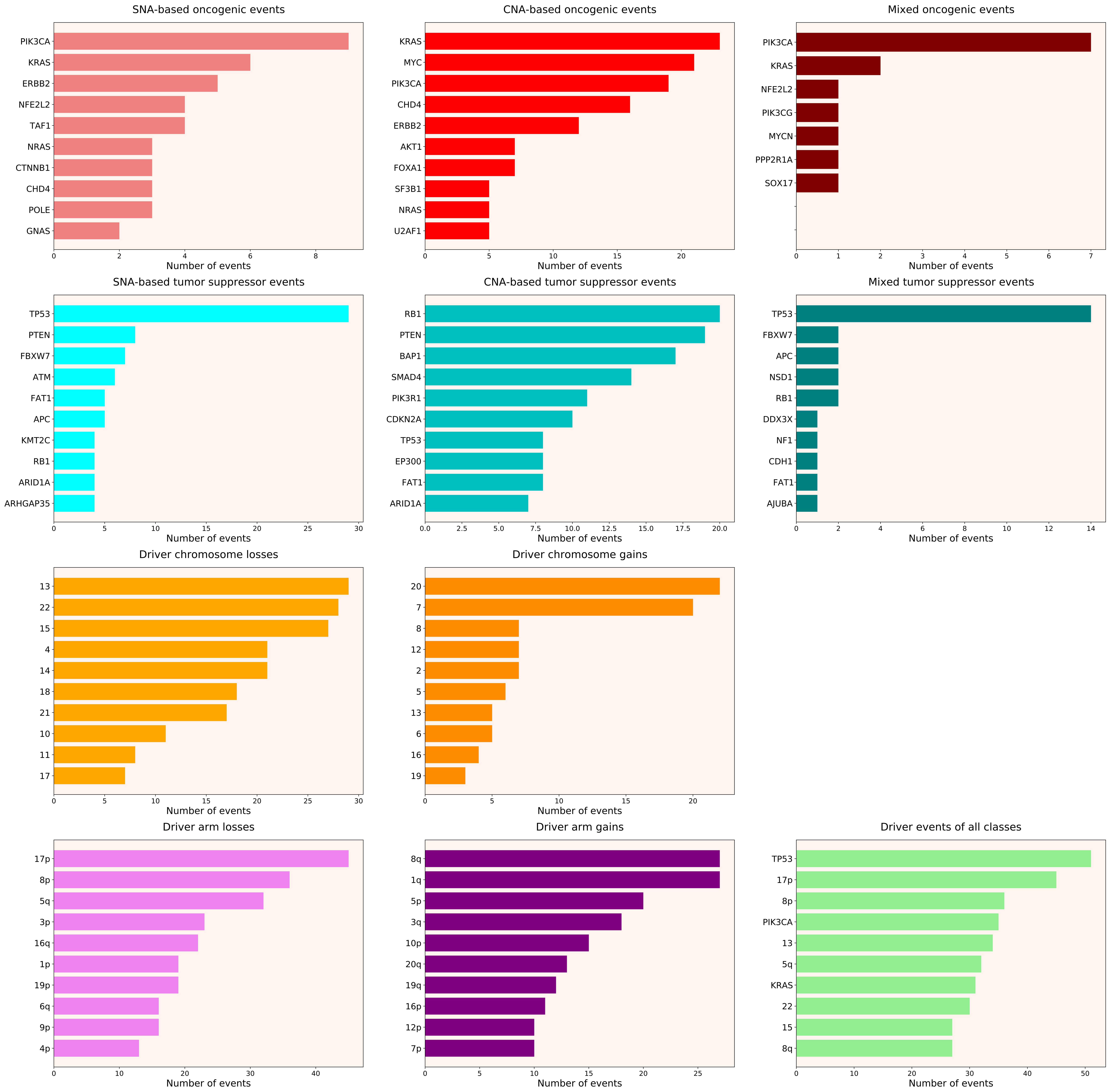
SNA-based oncogenic events
PIK3CA
KRAS
ERBB2
NFE2L2
TAF1
NRAS
CTNNB1
CHD4
POLE
GNAS
0
2
4
6
8
Number of events
CNA-based oncogenic events
KRAS
MYC
PIK3CA
CHD4
ERBB2
AKT1
FOXA1
SF3B1
NRAS
U2AF1
0
5
10
15
20
Number of events
Mixed oncogenic events
PIK3CA
KRAS
NFE2L2
PIK3CG
MYCN
PPP2R1A
SOX17
0
1
2
3
4
5
6
7
Number of events
SNA-based tumor suppressor events
TP53
PTEN
FBXW7
ATM
FAT1
APC
KMT2C
RB1
ARID1A
ARHGAP35
0
5
10
15
20
25
30
Number of events
CNA-based tumor suppressor events
RB1
PTEN
BAP1
SMAD4
PIK3R1
CDKN2A
TP53
EP300
FAT1
ARID1A
0.0
2.5
5.0
7.5
10.0
12.5
15.0
17.5
20.0
Number of events
Mixed tumor suppressor events
TP53
FBXW7
APC
NSD1
RB1
DDX3X
NF1
CDH1
FAT1
AJUBA
0
2
4
6
8
10
12
14
Number of events
Driver chromosome losses
13
22
15
4
14
18
21
10
11
17
0
5
10
15
20
25
30
Number of events
Driver chromosome gains
20
7
8
12
2
5
13
6
16
19
0
5
10
15
20
Number of events
Driver arm losses
17p
8p
5q
3p
16q
1p
19p
6q
9p
4p
0
10
20
30
40
Number of events
Driver arm gains
8q
1q
5p
3q
10p
20q
19q
16p
12p
7p
0
5
10
15
20
25
Number of events
Driver events of all classes
TP53
17p
8p
PIK3CA
13
5q
KRAS
22
15
8q
0
10
20
30
40
50
Number of events
