# Supplementary material for: Novel Driver Strength Index highlights important cancer genes in TCGA PanCanAtlas patients
Source: PeerJ. 2022 Aug 11;10:e13860. doi: 10.7717/peerj.13860 (PMC9375969; doi:10.7717/peerj.13860)
Supplement: Supplemental Information 2 [file peerj-10-13860-s002.zip › COHORTS/genes plots/detailed/2021_8_16_14_9_distribution_events_detailed_males_24.pdf]

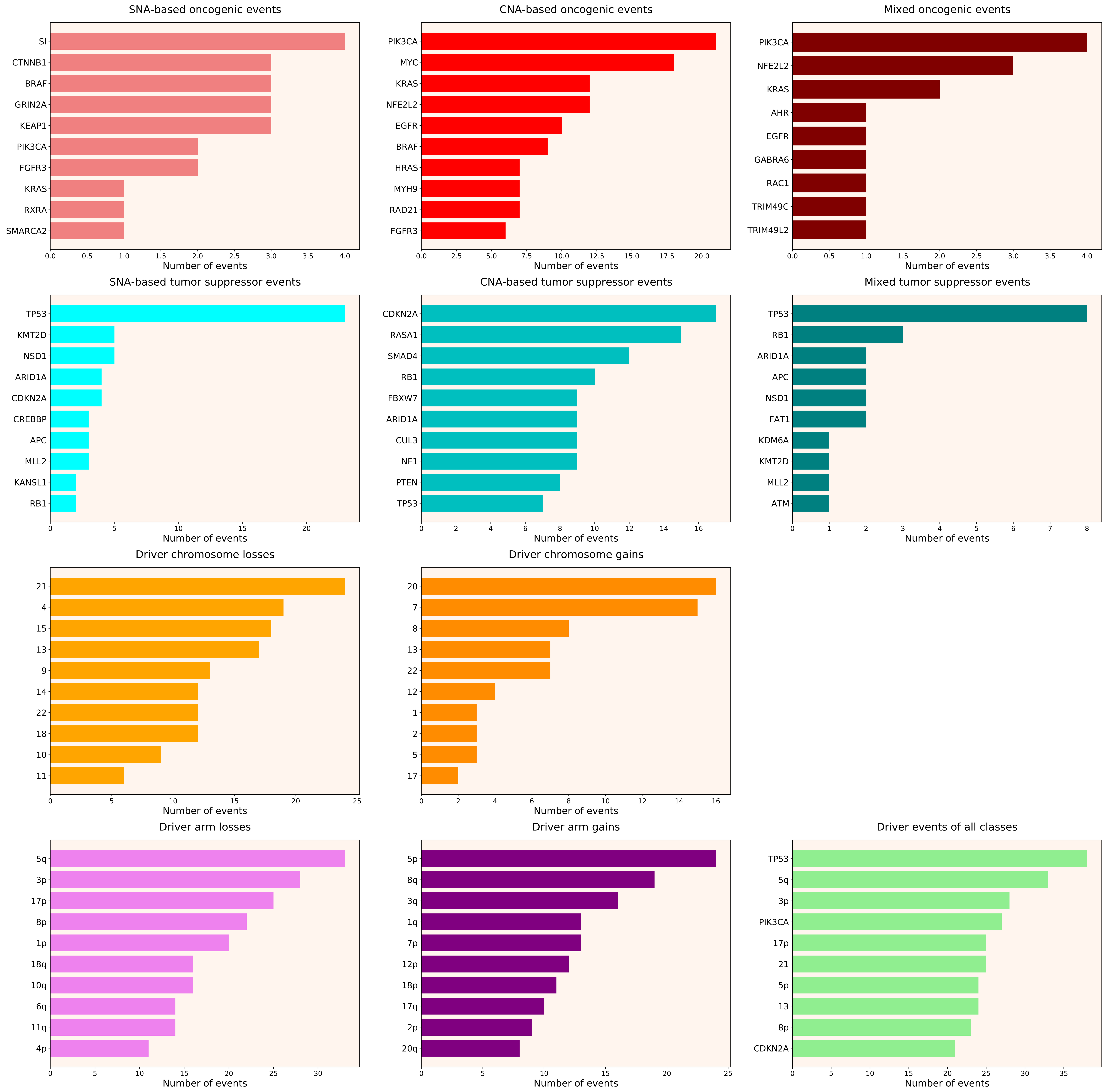
SNA-based oncogenic events
SI
CTNNB1
BRAF
GRIN2A
KEAP1
PIK3CA
FGFR3
KRAS
RXRA
SMARCA2
0.0 0.5 1.0 1.5 2.0 2.5 3.0 3.5 4.0
Number of events
CNA-based oncogenic events
PIK3CA
MYC
KRAS
NFE2L2
EGFR
BRAF
HRAS
MYH9
RAD21
FGFR3
0.0 2.5 5.0 7.5 10.0 12.5 15.0 17.5 20.0
Number of events
Mixed oncogenic events
PIK3CA
NFE2L2
KRAS
AHR
EGFR
GABRA6
RAC1
TRIM49C
TRIM49L2
0.0 0.5 1.0 1.5 2.0 2.5 3.0 3.5 4.0
Number of events
SNA-based tumor suppressor events
TP53
KMT2D
NSD1
ARID1A
CDKN2A
CREBBP
APC
MLL2
KANSL1
RB1
0 5 10 15 20
Number of events
CNA-based tumor suppressor events
CDKN2A
RASA1
SMAD4
RB1
FBXW7
ARID1A
CUL3
NF1
PTEN
TP53
0 2 4 6 8 10 12 14 16
Number of events
Mixed tumor suppressor events
TP53
RB1
ARID1A
APC
NSD1
FAT1
KDM6A
KMT2D
MLL2
ATM
0 1 2 3 4 5 6 7 8
Number of events
Driver chromosome losses
21
4
15
13
9
14
22
18
10
11
0 5 10 15 20 25
Number of events
Driver chromosome gains
20
7
8
13
22
12
1
2
5
17
0 2 4 6 8 10 12 14 16
Number of events
Driver arm losses
5q
3p
17p
8p
1p
18q
10q
6q
11q
4p
0 5 10 15 20 25 30
Number of events
Driver arm gains
5p
8q
3q
1q
7p
12p
18p
17q
2p
20q
0 5 10 15 20 25
Number of events
Driver events of all classes
TP53
5q
3p
PIK3CA
17p
21
5p
13
8p
CDKN2A
0 5 10 15 20 25 30 35
Number of events
