# Supplementary material for: Novel Driver Strength Index highlights important cancer genes in TCGA PanCanAtlas patients
Source: PeerJ. 2022 Aug 11;10:e13860. doi: 10.7717/peerj.13860 (PMC9375969; doi:10.7717/peerj.13860)
Supplement: Supplemental Information 2 [file peerj-10-13860-s002.zip › COHORTS/genes plots/detailed/2021_8_16_14_9_distribution_events_detailed_females_9.pdf]

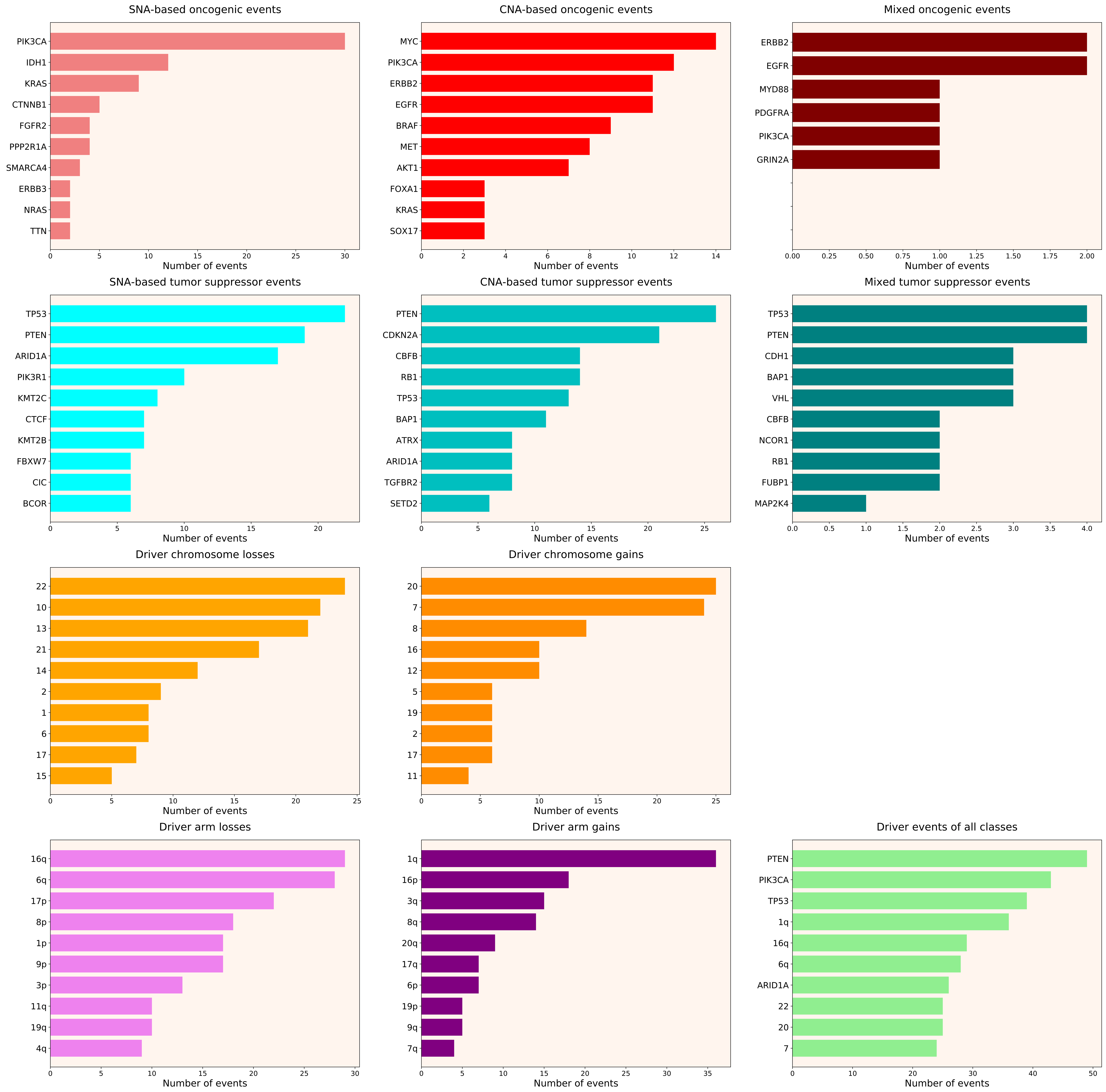

SNA-based oncogenic events
PIK3CA
IDH1
KRAS
CTNNB1
FGFR2
PPP2R1A
SMARCA4
ERBB3
NRAS
TTN
0 5 10 15 20 25 30
Number of events
CNA-based oncogenic events
MYC
PIK3CA
ERBB2
EGFR
BRAF
MET
AKT1
FOXA1
KRAS
SOX17
0 2 4 6 8 10 12 14
Number of events
Mixed oncogenic events
ERBB2
EGFR
MYD88
PDGFRA
PIK3CA
GRIN2A
0.00 0.25 0.50 0.75 1.00 1.25 1.50 1.75 2.00
Number of events
SNA-based tumor suppressor events
TP53
PTEN
ARID1A
PIK3R1
KMT2C
CTCF
KMT2B
FBXW7
CIC
BCOR
0 5 10 15 20
Number of events
CNA-based tumor suppressor events
PTEN
CDKN2A
CBFB
RB1
TP53
BAP1
ATRX
ARID1A
TGFBR2
SETD2
0 5 10 15 20 25
Number of events
Mixed tumor suppressor events
TP53
PTEN
CDH1
BAP1
VHL
CBFB
NCOR1
RB1
FUBP1
MAP2K4
0.0 0.5 1.0 1.5 2.0 2.5 3.0 3.5 4.0
Number of events
Driver chromosome losses
22
10
13
21
14
2
1
6
17
15
0 5 10 15 20 25
Number of events
Driver chromosome gains
20
7
8
16
12
5
19
2
17
11
0 5 10 15 20 25
Number of events
Driver arm losses
16q
6q
17p
8p
1p
9p
3p
11q
19q
4q
0 5 10 15 20 25 30
Number of events
Driver arm gains
1q
16p
3q
8q
20q
17q
6p
19p
9q
7q
0 5 10 15 20 25 30 35
Number of events
Driver events of all classes
PTEN
PIK3CA
TP53
1q
16q
6q
ARID1A
22
20
7
0 10 20 30 40 50
Number of events
