# Supplementary material for: Novel Driver Strength Index highlights important cancer genes in TCGA PanCanAtlas patients
Source: PeerJ. 2022 Aug 11;10:e13860. doi: 10.7717/peerj.13860 (PMC9375969; doi:10.7717/peerj.13860)
Supplement: Supplemental Information 2 [file peerj-10-13860-s002.zip › COHORTS/genes plots/detailed/2021_8_16_14_9_distribution_events_detailed_females_24.pdf]

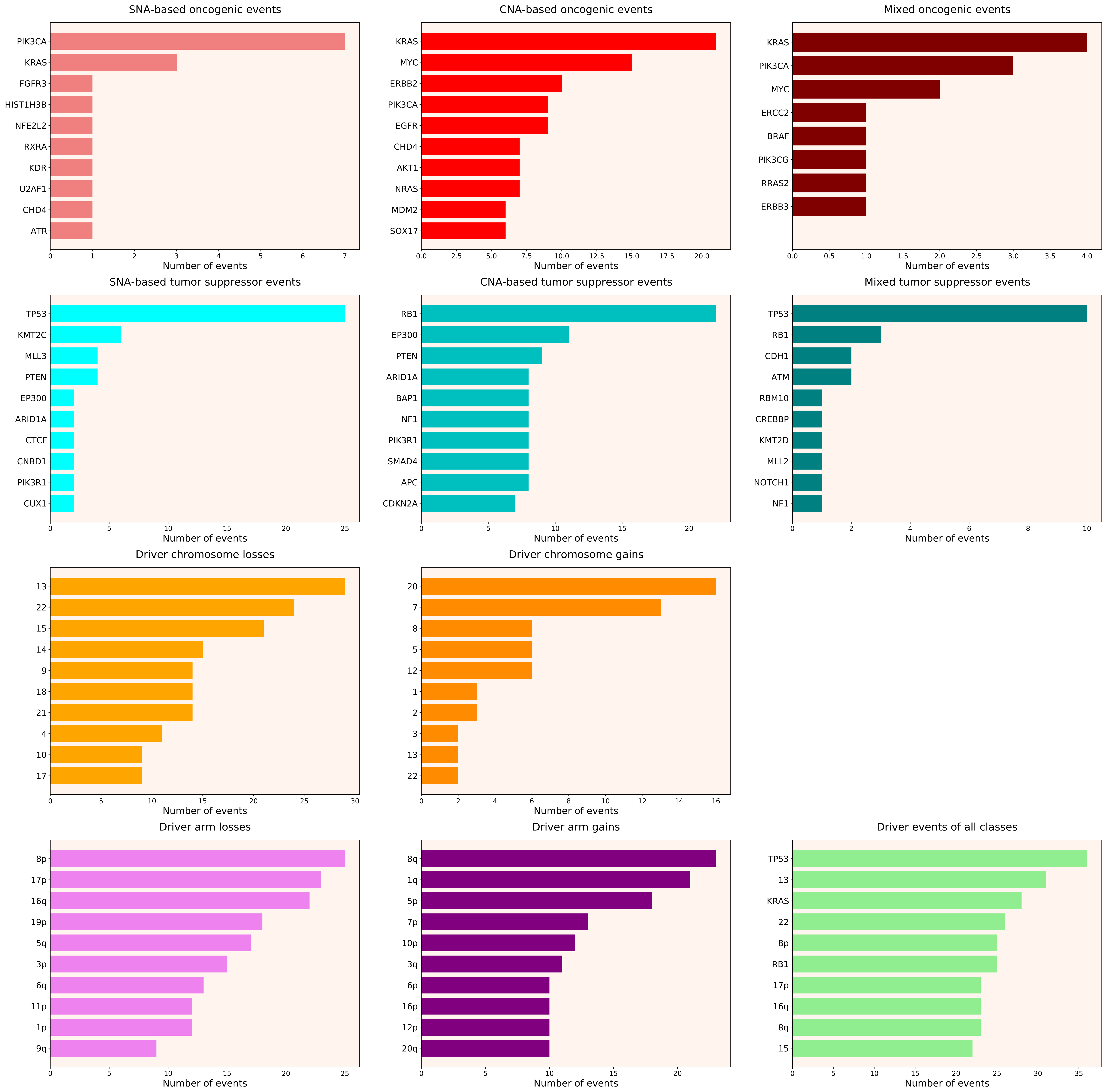
SNA-based oncogenic events
PIK3CA
KRAS
FGFR3
HIST1H3B
NFE2L2
RXRA
KDR
U2AF1
CHD4
ATR
0 1 2 3 4 5 6 7
Number of events
CNA-based oncogenic events
KRAS
MYC
ERBB2
PIK3CA
EGFR
CHD4
AKT1
NRAS
MDM2
SOX17
0.0 2.5 5.0 7.5 10.0 12.5 15.0 17.5 20.0
Number of events
Mixed oncogenic events
KRAS
PIK3CA
MYC
ERCC2
BRAF
PIK3CG
RRAS2
ERBB3
0.0 0.5 1.0 1.5 2.0 2.5 3.0 3.5 4.0
Number of events
SNA-based tumor suppressor events
TP53
KMT2C
MLL3
PTEN
EP300
ARID1A
CTCF
CNBD1
PIK3R1
CUX1
0 5 10 15 20 25
Number of events
CNA-based tumor suppressor events
RB1
EP300
PTEN
ARID1A
BAP1
NF1
PIK3R1
SMAD4
APC
CDKN2A
0 5 10 15 20
Number of events
Mixed tumor suppressor events
TP53
RB1
CDH1
ATM
RBM10
CREBBP
KMT2D
MLL2
NOTCH1
NF1
0 2 4 6 8 10
Number of events
Driver chromosome losses
13
22
15
14
9
18
21
4
10
17
0 5 10 15 20 25 30
Number of events
Driver chromosome gains
20
7
8
5
12
1
2
3
13
22
0 2 4 6 8 10 12 14 16
Number of events
Driver arm losses
8p
17p
16q
19p
5q
3p
6q
11p
1p
9q
0 5 10 15 20 25
Number of events
Driver arm gains
8q
1q
5p
7p
10p
3q
6p
16p
12p
20q
0 5 10 15 20
Number of events
Driver events of all classes
TP53
13
KRAS
22
8p
RB1
17p
16q
8q
15
0 5 10 15 20 25 30 35
Number of events
