# Supplementary material for: Novel Driver Strength Index highlights important cancer genes in TCGA PanCanAtlas patients
Source: PeerJ. 2022 Aug 11;10:e13860. doi: 10.7717/peerj.13860 (PMC9375969; doi:10.7717/peerj.13860)
Supplement: Supplemental Information 2 [file peerj-10-13860-s002.zip › COHORTS/genes plots/detailed/2021_8_16_14_9_distribution_events_detailed_females_25.pdf]

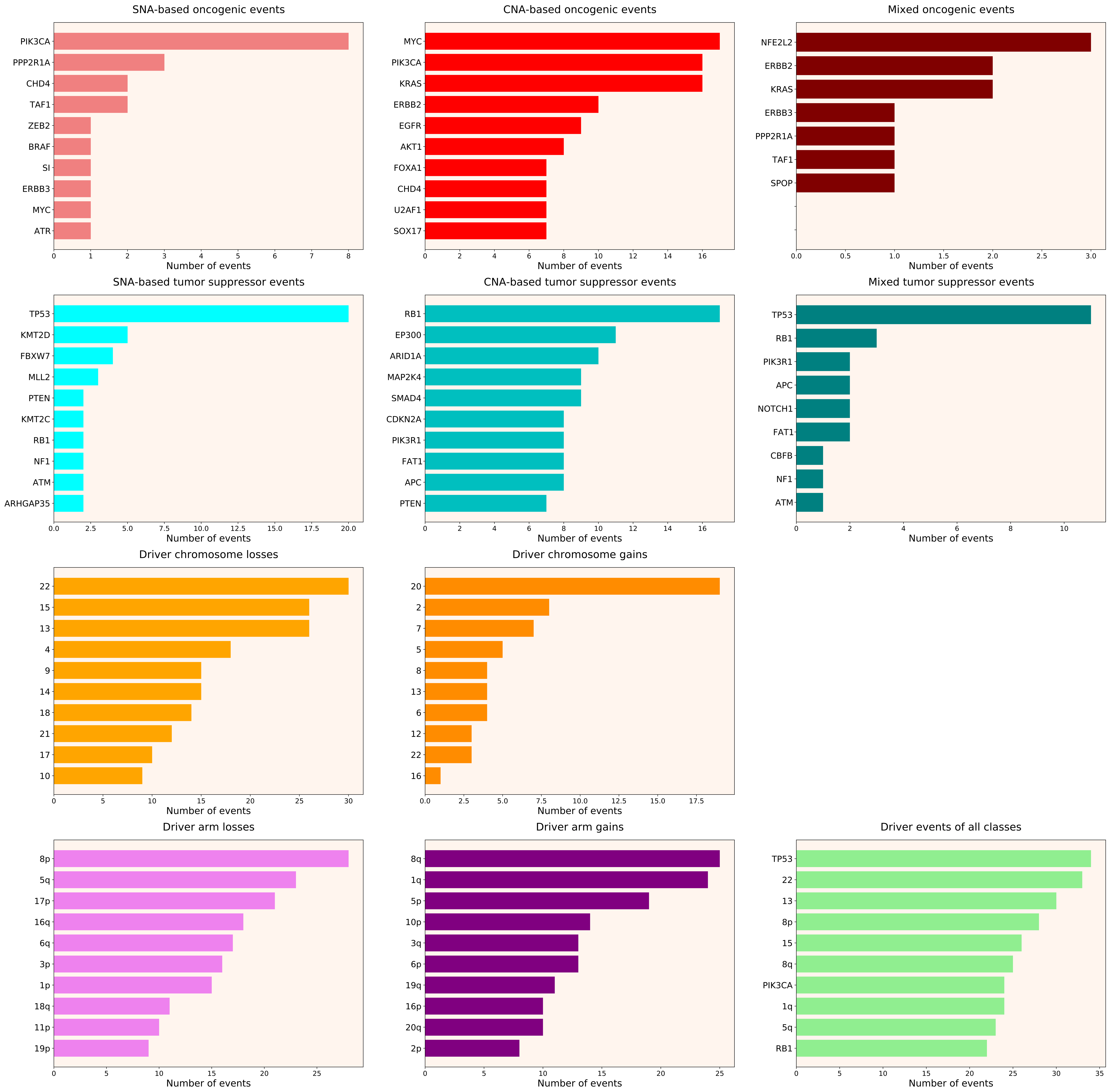
SNA-based oncogenic events
PIK3CA
PPP2R1A
CHD4
TAF1
ZEB2
BRAF
SI
ERBB3
MYC
ATR
Number of events
CNA-based oncogenic events
MYC
PIK3CA
KRAS
ERBB2
EGFR
AKT1
FOXA1
CHD4
U2AF1
SOX17
Number of events
Mixed oncogenic events
NFE2L2
ERBB2
KRAS
ERBB3
PPP2R1A
TAF1
SPOP
Number of events
SNA-based tumor suppressor events
TP53
KMT2D
FBXW7
MLL2
PTEN
KMT2C
RB1
NF1
ATM
ARHGAP35
Number of events
CNA-based tumor suppressor events
RB1
EP300
ARID1A
MAP2K4
SMAD4
CDKN2A
PIK3R1
FAT1
APC
PTEN
Number of events
Mixed tumor suppressor events
TP53
RB1
PIK3R1
APC
NOTCH1
FAT1
CBFB
NF1
ATM
Number of events
Driver chromosome losses
22
15
13
4
9
14
18
21
17
10
Number of events
Driver chromosome gains
20
2
7
5
8
13
6
12
22
16
Number of events
Driver arm losses
8p
5q
17p
16q
6q
3p
1p
18q
11p
19p
Number of events
Driver arm gains
8q
1q
5p
10p
3q
6p
19q
16p
20q
2p
Number of events
Driver events of all classes
TP53
22
13
8p
15
8q
PIK3CA
1q
5q
RB1
Number of events
