# Supplementary material for: Novel Driver Strength Index highlights important cancer genes in TCGA PanCanAtlas patients
Source: PeerJ. 2022 Aug 11;10:e13860. doi: 10.7717/peerj.13860 (PMC9375969; doi:10.7717/peerj.13860)
Supplement: Supplemental Information 2 [file peerj-10-13860-s002.zip › COHORTS/genes plots/detailed/2021_8_16_14_9_distribution_events_detailed_males_35.pdf]

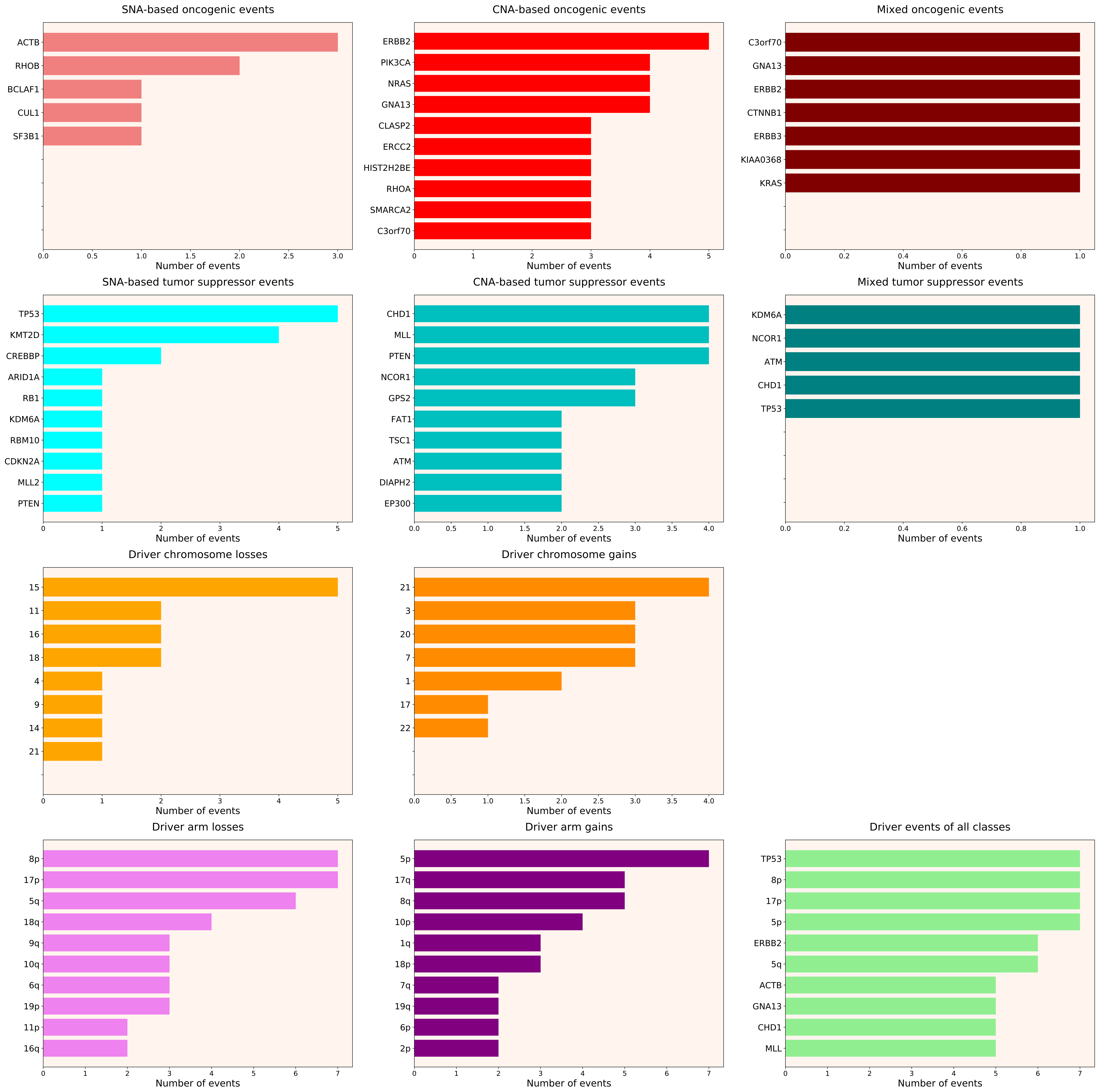

SNA-based oncogenic events
ACTB
RHOB
BCLAF1
CUL1
SF3B1
Number of events
CNA-based oncogenic events
ERBB2
PIK3CA
NRAS
GNA13
CLASP2
ERCC2
HIST2H2BE
RHOA
SMARCA2
C3orf70
Number of events
Mixed oncogenic events
C3orf70
GNA13
ERBB2
CTNNB1
ERBB3
KIAA0368
KRAS
Number of events
SNA-based tumor suppressor events
TP53
KMT2D
CREBBP
ARID1A
RB1
KDM6A
RBM10
CDKN2A
MLL2
PTEN
Number of events
CNA-based tumor suppressor events
CHD1
MLL
PTEN
NCOR1
GPS2
FAT1
TSC1
ATM
DIAPH2
EP300
Number of events
Mixed tumor suppressor events
KDM6A
NCOR1
ATM
CHD1
TP53
Number of events
Driver chromosome losses
15
11
16
18
4
9
14
21
Number of events
Driver chromosome gains
21
3
20
7
1
17
22
Number of events
Driver arm losses
8p
17p
5q
18q
9q
10q
6q
19p
11p
16q
Number of events
Driver arm gains
5p
17q
8q
10p
1q
18p
7q
19q
6p
2p
Number of events
Driver events of all classes
TP53
8p
17p
5p
ERBB2
5q
ACTB
GNA13
CHD1
MLL
Number of events
