# Supplementary material for: Novel Driver Strength Index highlights important cancer genes in TCGA PanCanAtlas patients
Source: PeerJ. 2022 Aug 11;10:e13860. doi: 10.7717/peerj.13860 (PMC9375969; doi:10.7717/peerj.13860)
Supplement: Supplemental Information 2 [file peerj-10-13860-s002.zip › COHORTS/genes plots/detailed/2021_8_16_14_9_distribution_events_detailed_28.pdf]

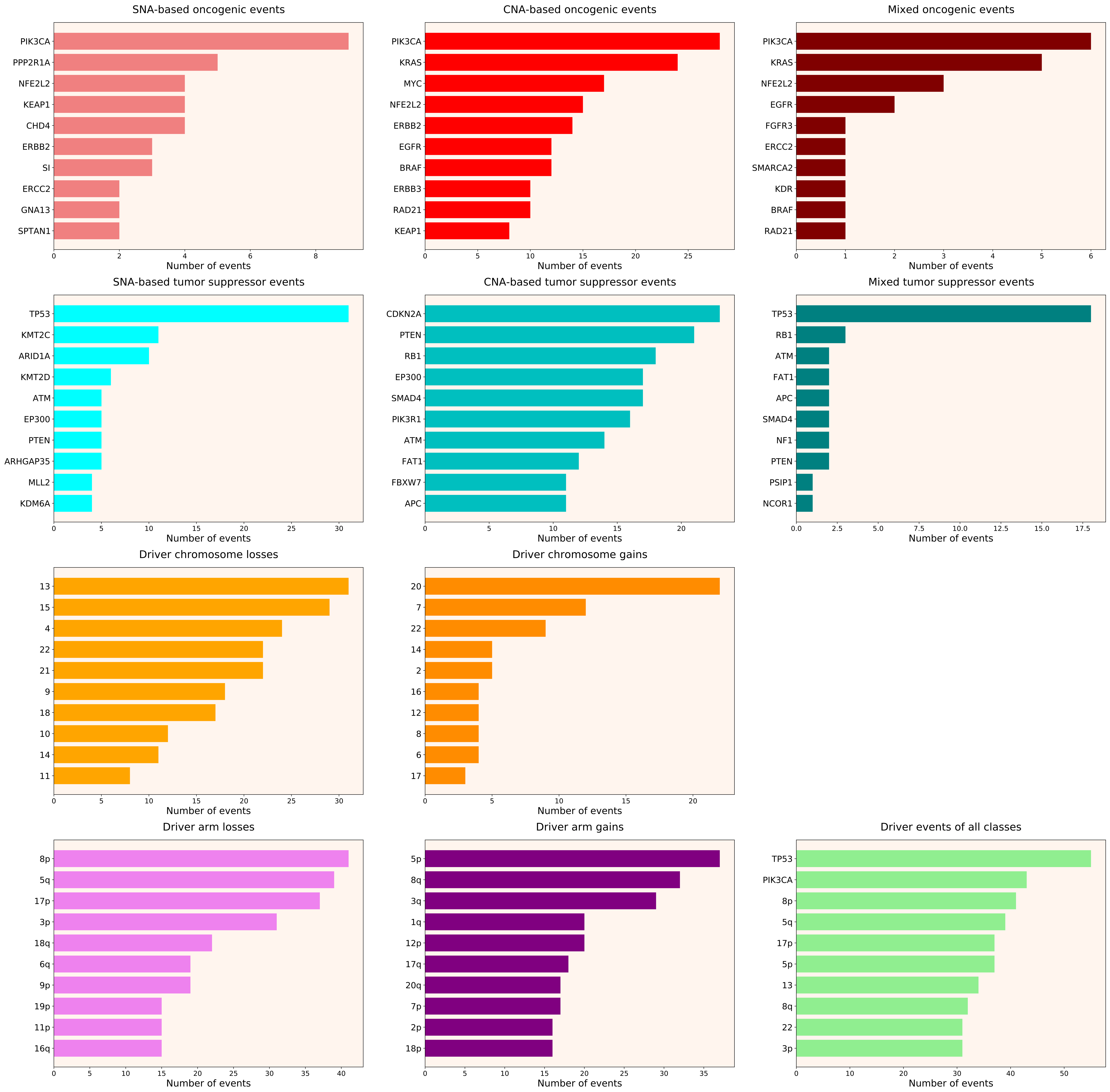

SNA-based oncogenic events
PIK3CA
PPP2R1A
NFE2L2
KEAP1
CHD4
ERBB2
SI
ERCC2
GNA13
SPTAN1
0 2 4 6 8
Number of events
CNA-based oncogenic events
PIK3CA
KRAS
MYC
NFE2L2
ERBB2
EGFR
BRAF
ERBB3
RAD21
KEAP1
0 5 10 15 20 25
Number of events
Mixed oncogenic events
PIK3CA
KRAS
NFE2L2
EGFR
FGFR3
ERCC2
SMARCA2
KDR
BRAF
RAD21
0 1 2 3 4 5 6
Number of events
SNA-based tumor suppressor events
TP53
KMT2C
ARID1A
KMT2D
ATM
EP300
PTEN
ARHGAP35
MLL2
KDM6A
0 5 10 15 20 25 30
Number of events
CNA-based tumor suppressor events
CDKN2A
PTEN
RB1
EP300
SMAD4
PIK3R1
ATM
FAT1
FBXW7
APC
0 5 10 15 20
Number of events
Mixed tumor suppressor events
TP53
RB1
ATM
FAT1
APC
SMAD4
NF1
PTEN
PSIP1
NCOR1
0.0 2.5 5.0 7.5 10.0 12.5 15.0 17.5
Number of events
Driver chromosome losses
13
15
4
22
21
9
18
10
14
11
0 5 10 15 20 25 30
Number of events
Driver chromosome gains
20
7
22
14
2
16
12
8
6
17
0 5 10 15 20
Number of events
Driver arm losses
8p
5q
17p
3p
18q
6q
9p
19p
11p
16q
0 5 10 15 20 25 30 35 40
Number of events
Driver arm gains
5p
8q
3q
1q
12p
17q
20q
7p
2p
18p
0 5 10 15 20 25 30 35
Number of events
Driver events of all classes
TP53
PIK3CA
8p
5q
17p
5p
13
8q
22
3p
0 10 20 30 40 50
Number of events
