# Supplementary figures and images for: Novel Driver Strength Index highlights important cancer genes in TCGA PanCanAtlas patients (part 2 of 2)
Source: PeerJ. 2022 Aug 11;10:e13860. doi: 10.7717/peerj.13860 (PMC9375969; doi:10.7717/peerj.13860)

# CHOL\_MALE

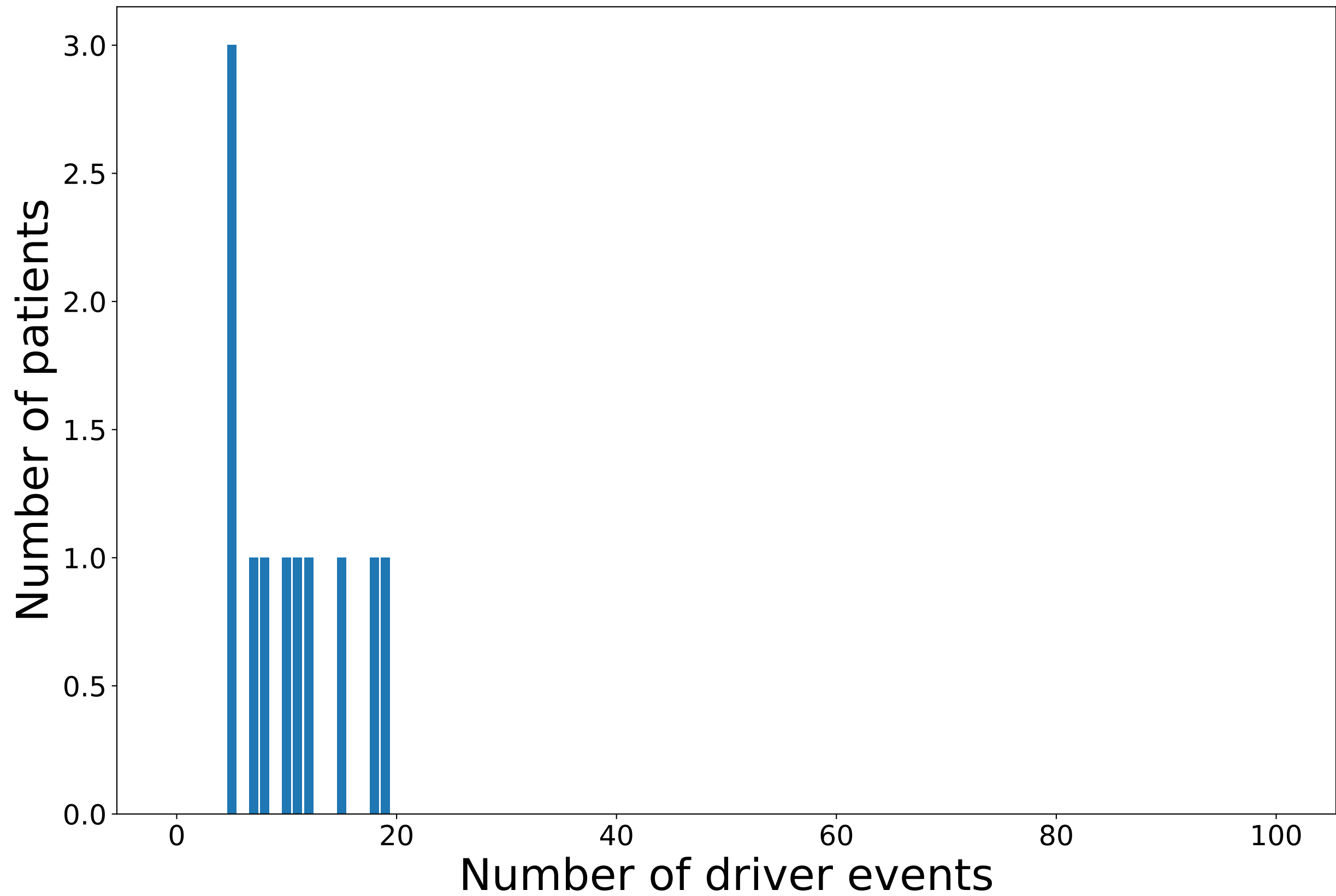

Supplement: Supplemental Information 2 [file peerj-10-13860-s002.zip › COHORTS/patient distributions/2021_8_16_14_9_CHOL_MALE.pdf]

# LUSC

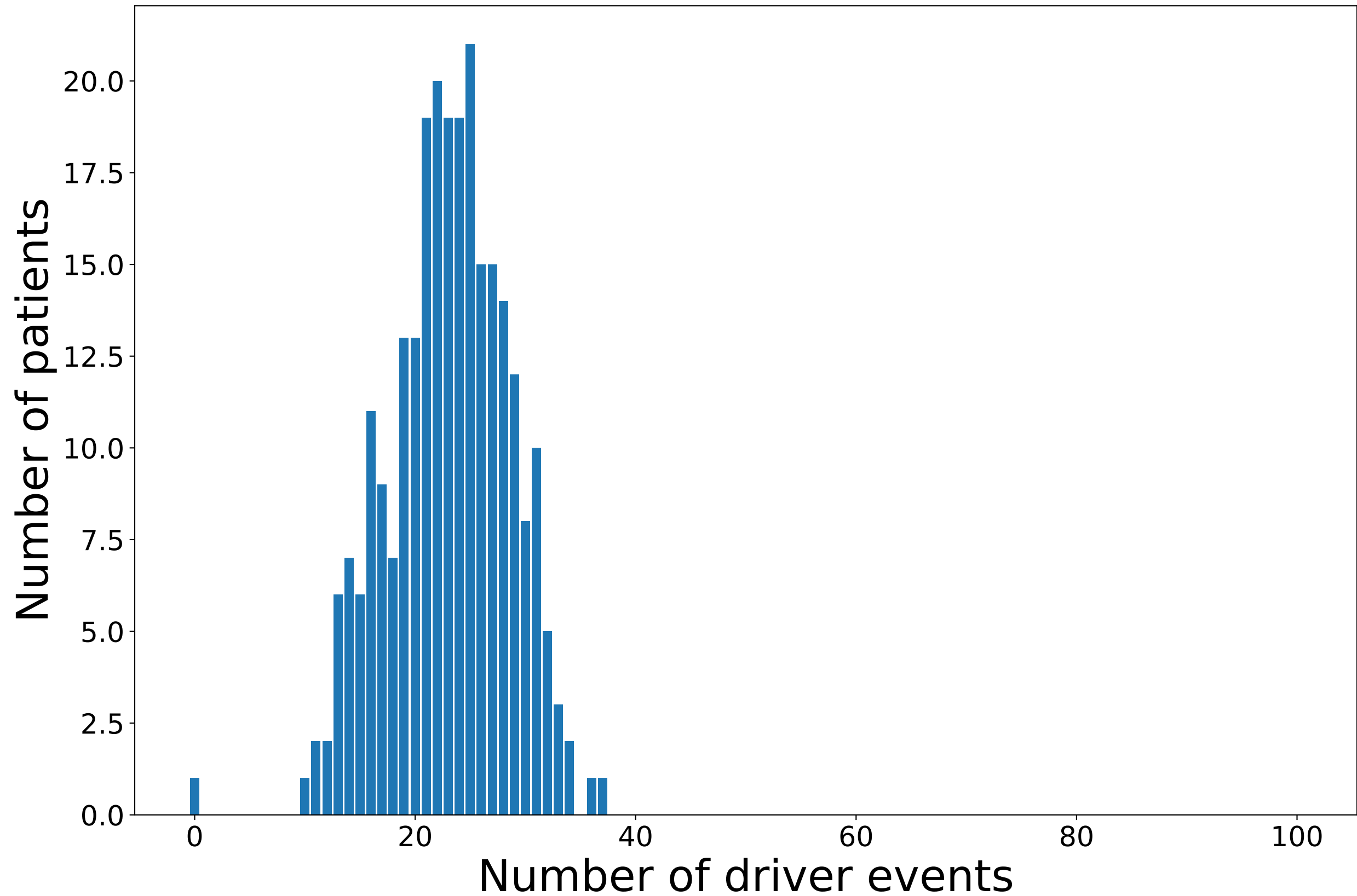

Supplement: Supplemental Information 2 [file peerj-10-13860-s002.zip › COHORTS/patient distributions/2021_8_16_14_9_LUSC.pdf]

# THCA\_FEMALE

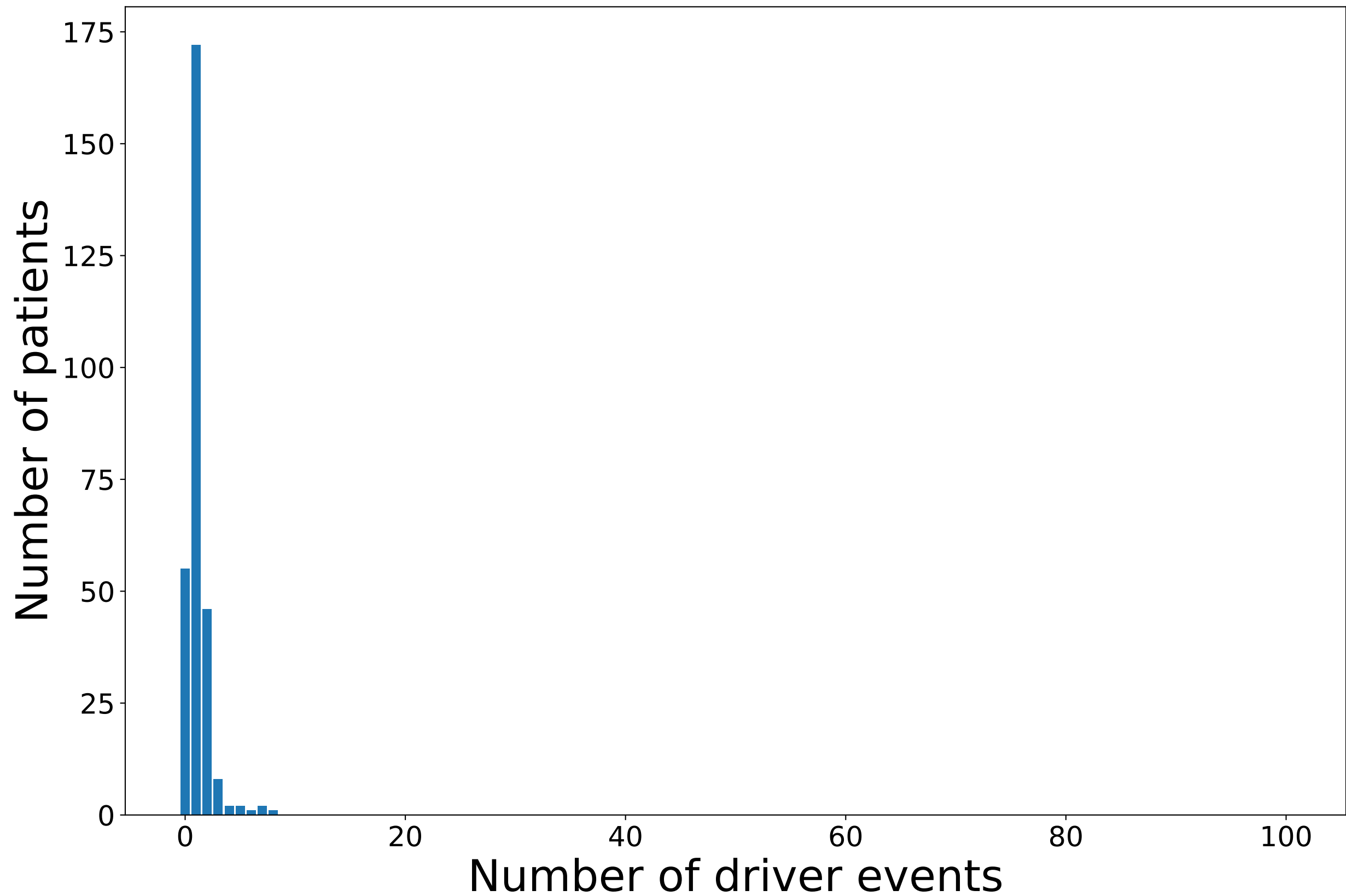

Supplement: Supplemental Information 2 [file peerj-10-13860-s002.zip › COHORTS/patient distributions/2021_8_16_14_9_THCA_FEMALE.pdf]

# COAD\_MALE

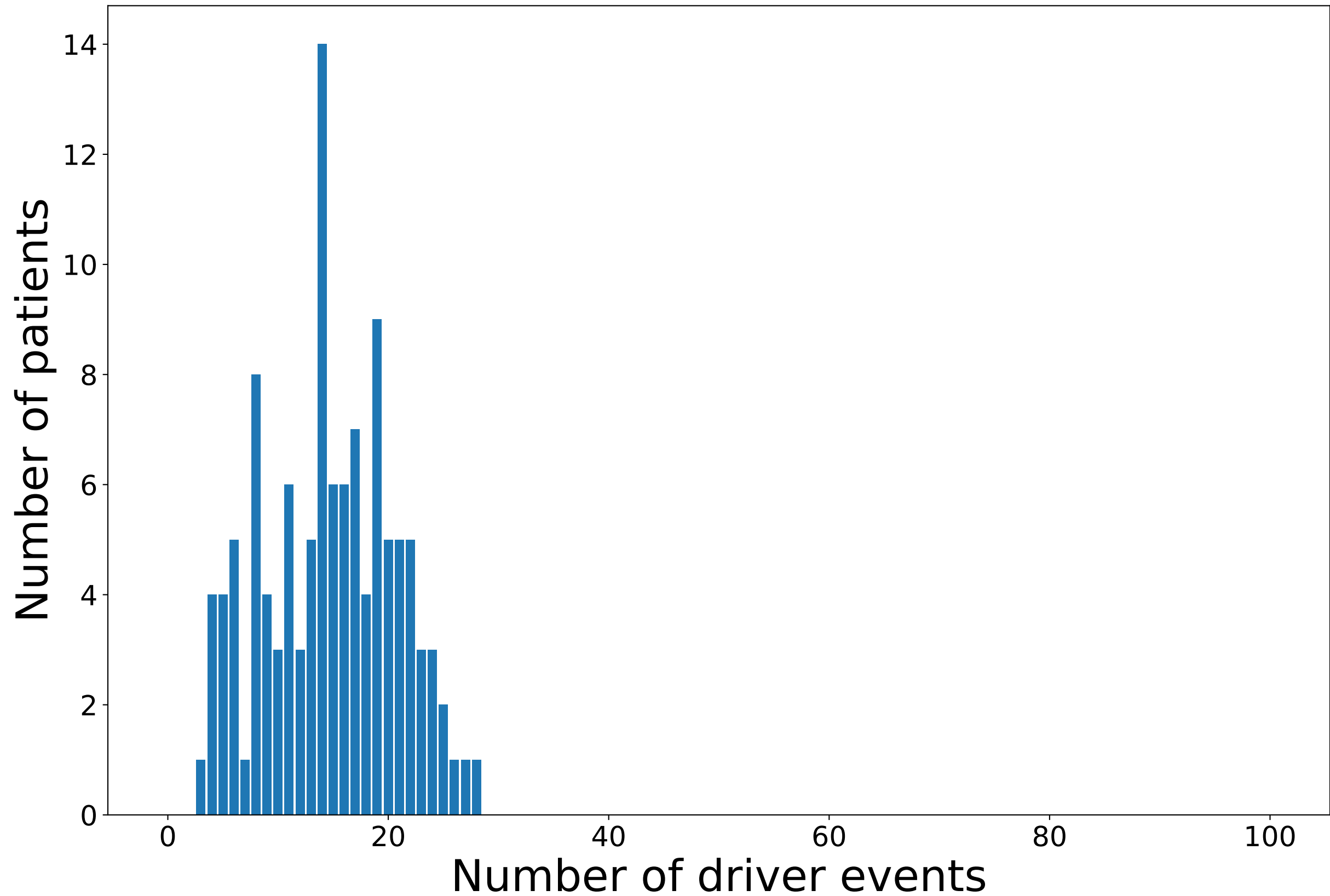

Supplement: Supplemental Information 2 [file peerj-10-13860-s002.zip › COHORTS/patient distributions/2021_8_16_14_9_COAD_MALE.pdf]

# PCPG

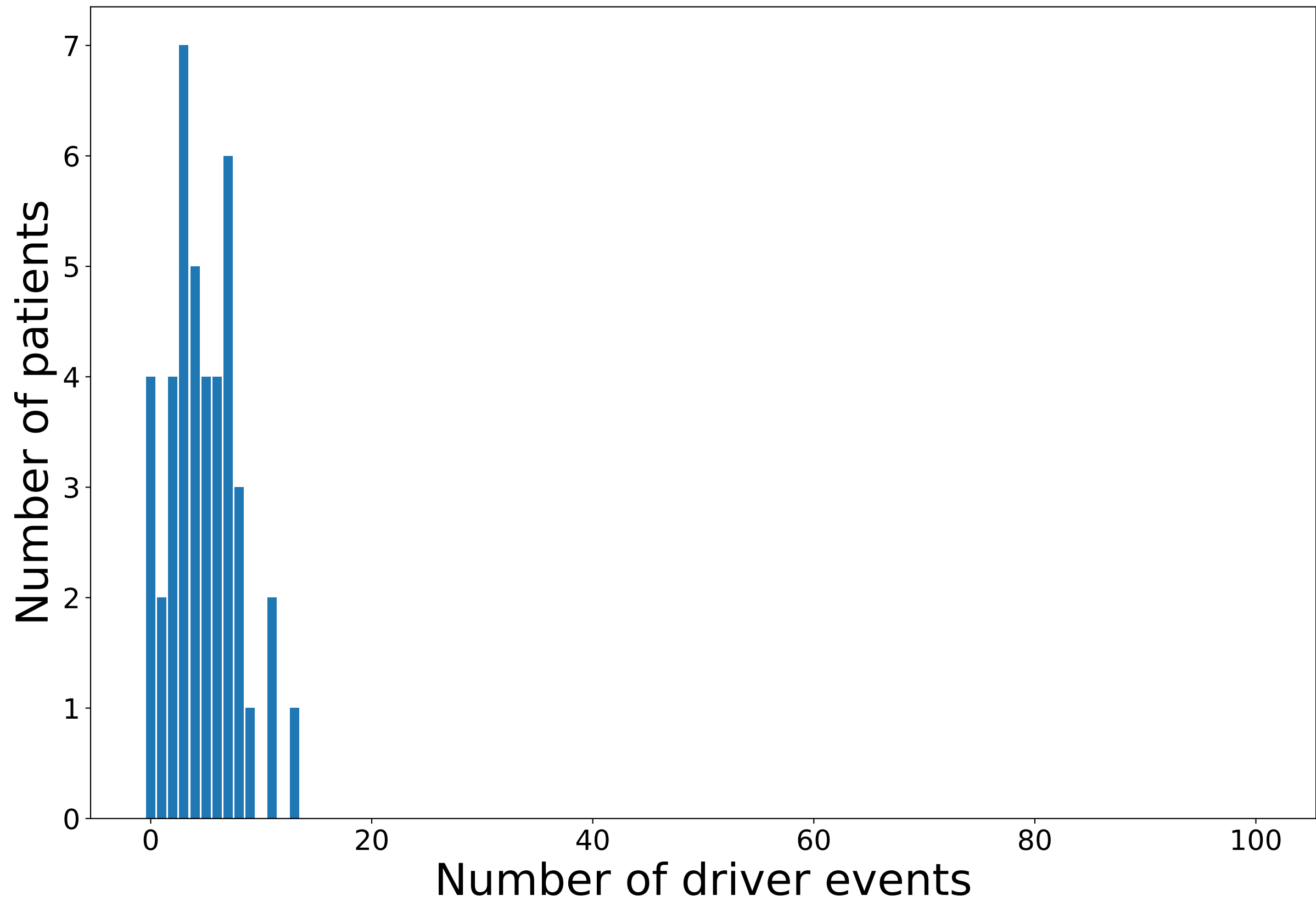

Supplement: Supplemental Information 2 [file peerj-10-13860-s002.zip › COHORTS/patient distributions/2021_8_16_14_9_PCPG.pdf]

# BLCA\_MALE

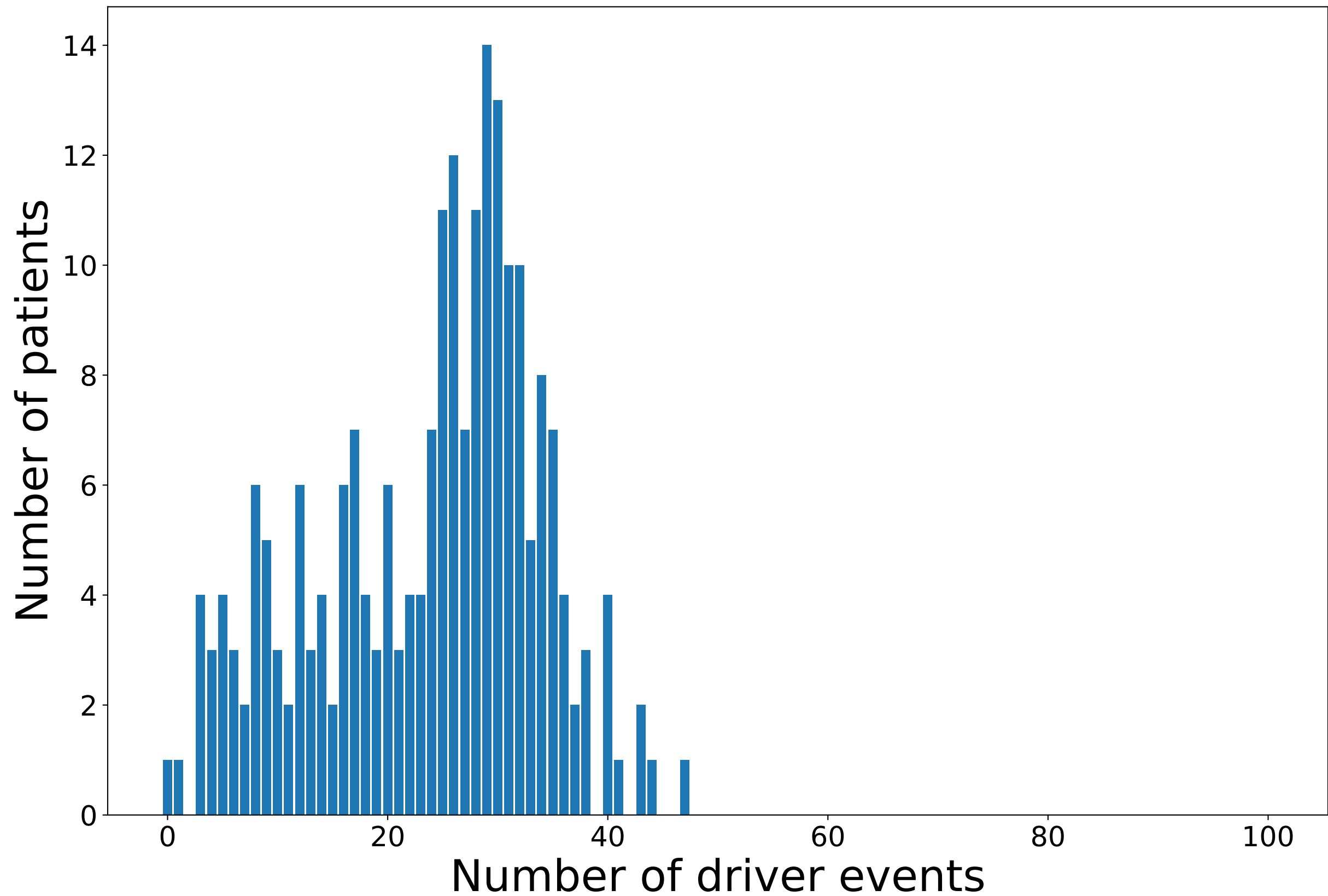

Supplement: Supplemental Information 2 [file peerj-10-13860-s002.zip › COHORTS/patient distributions/2021_8_16_14_9_BLCA_MALE.pdf]

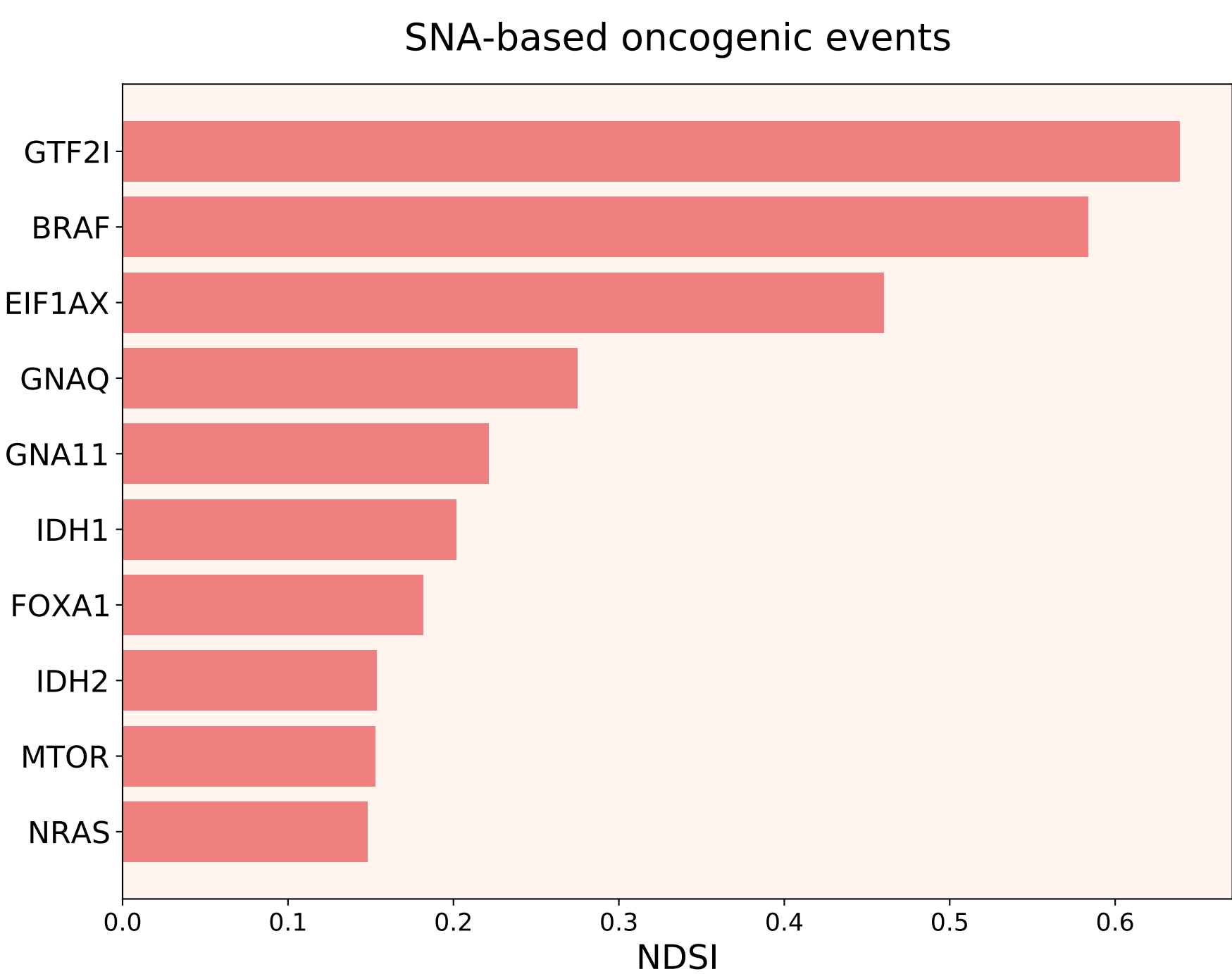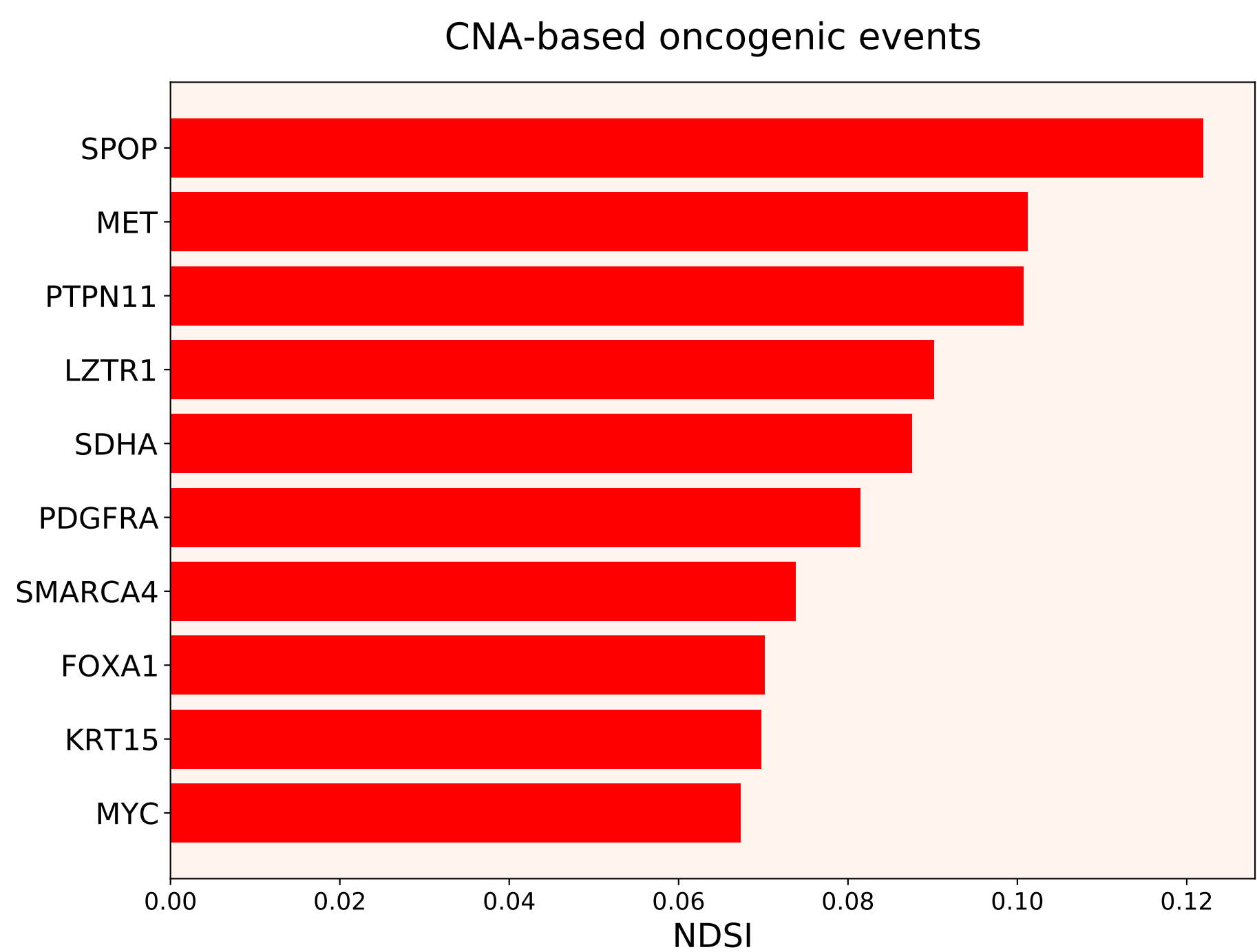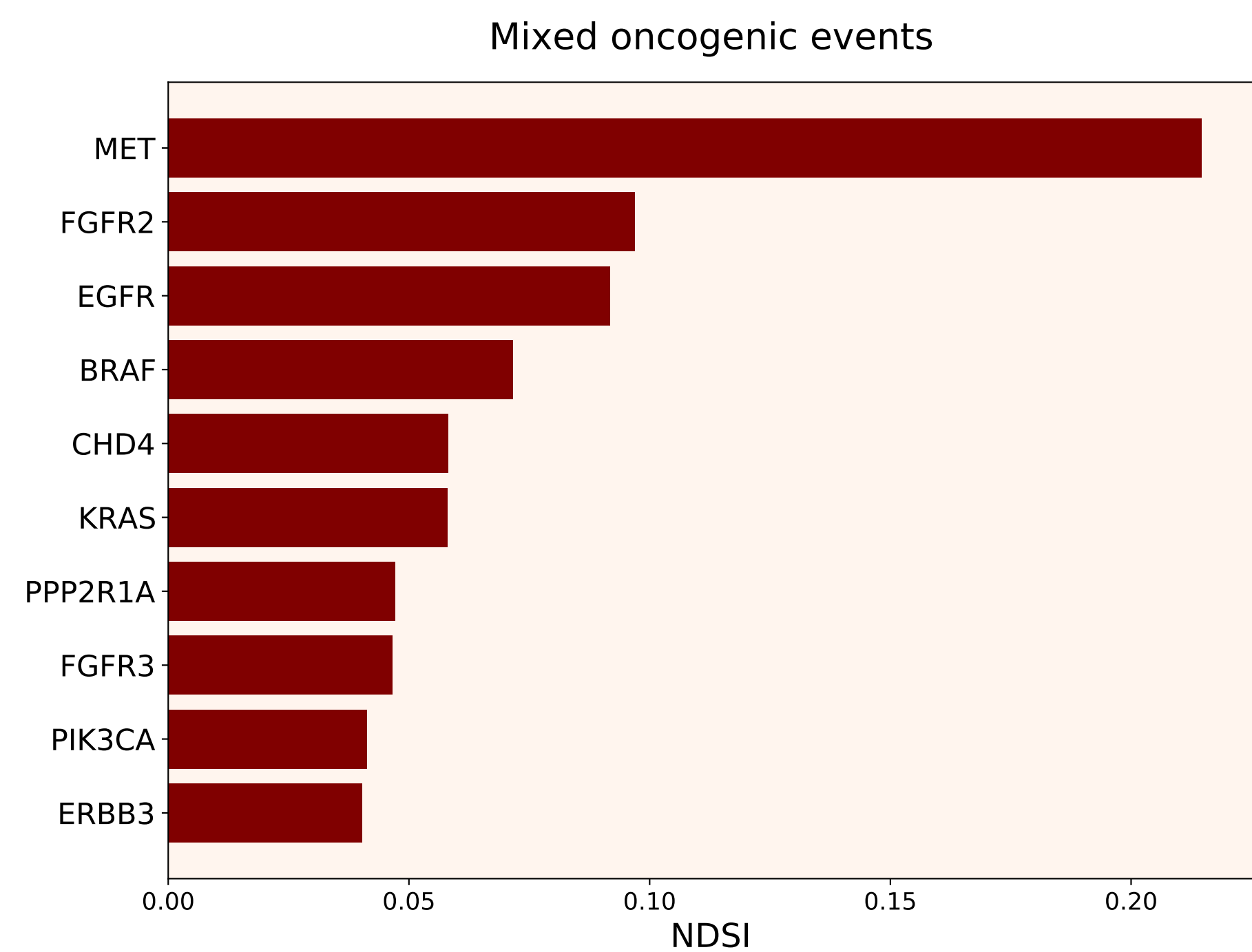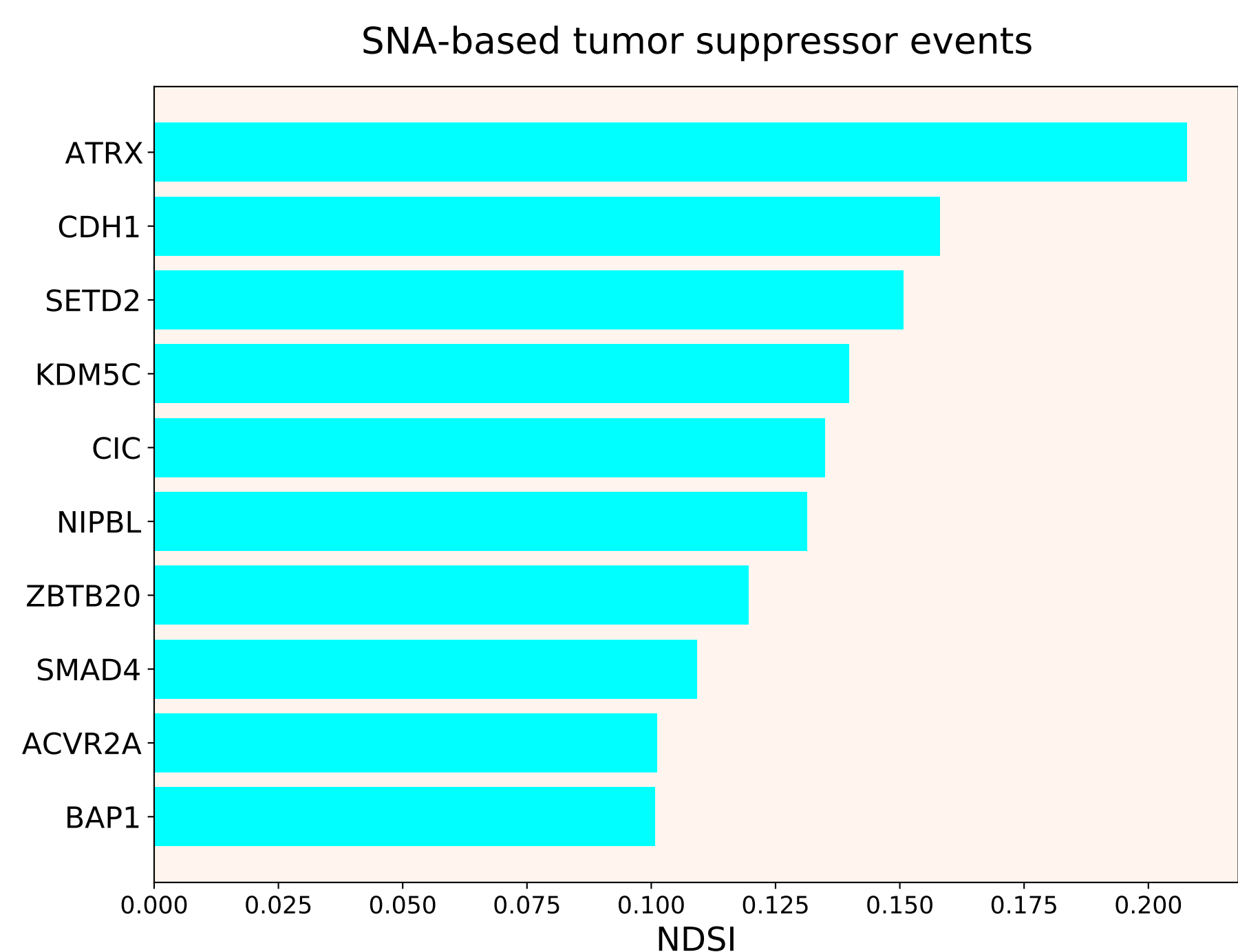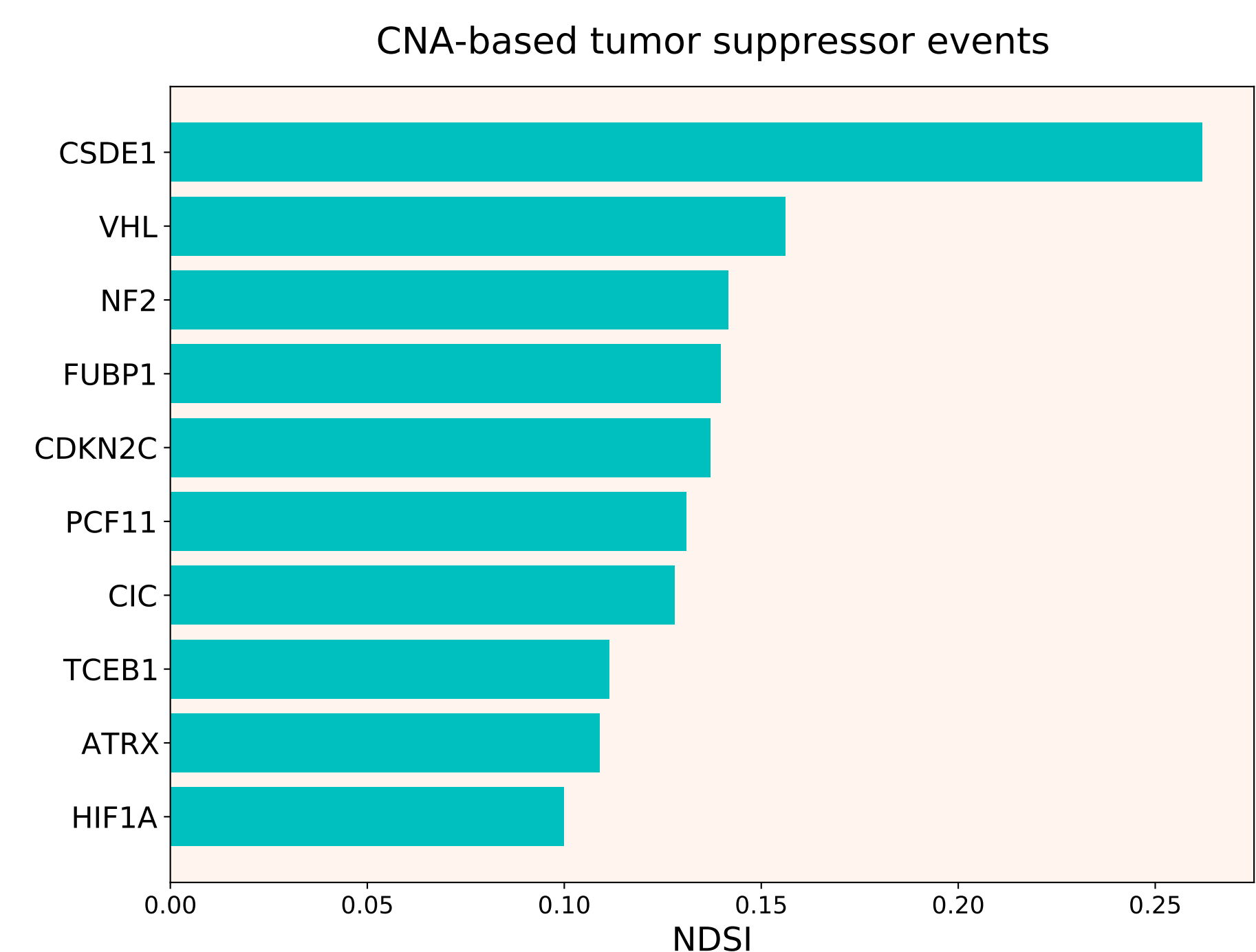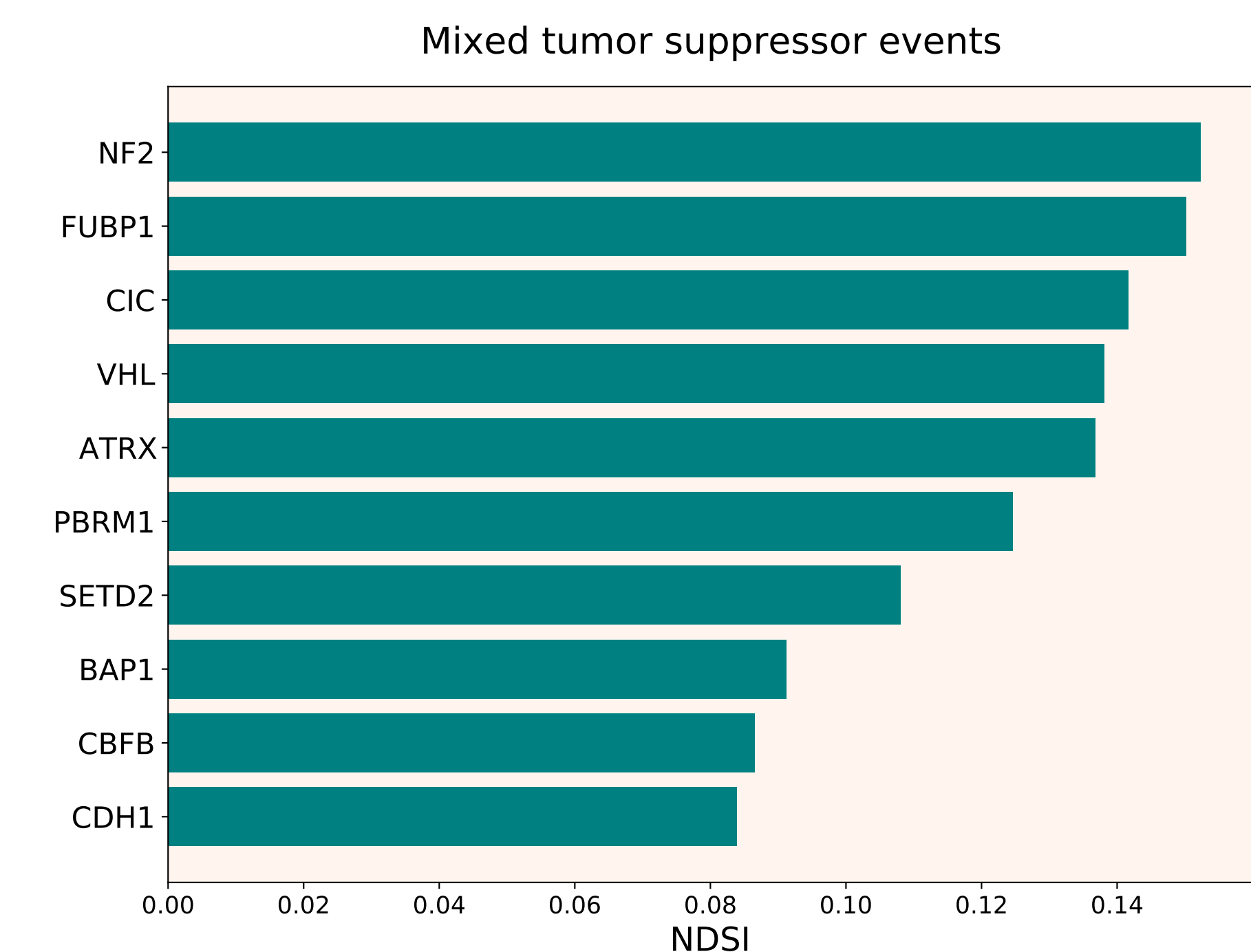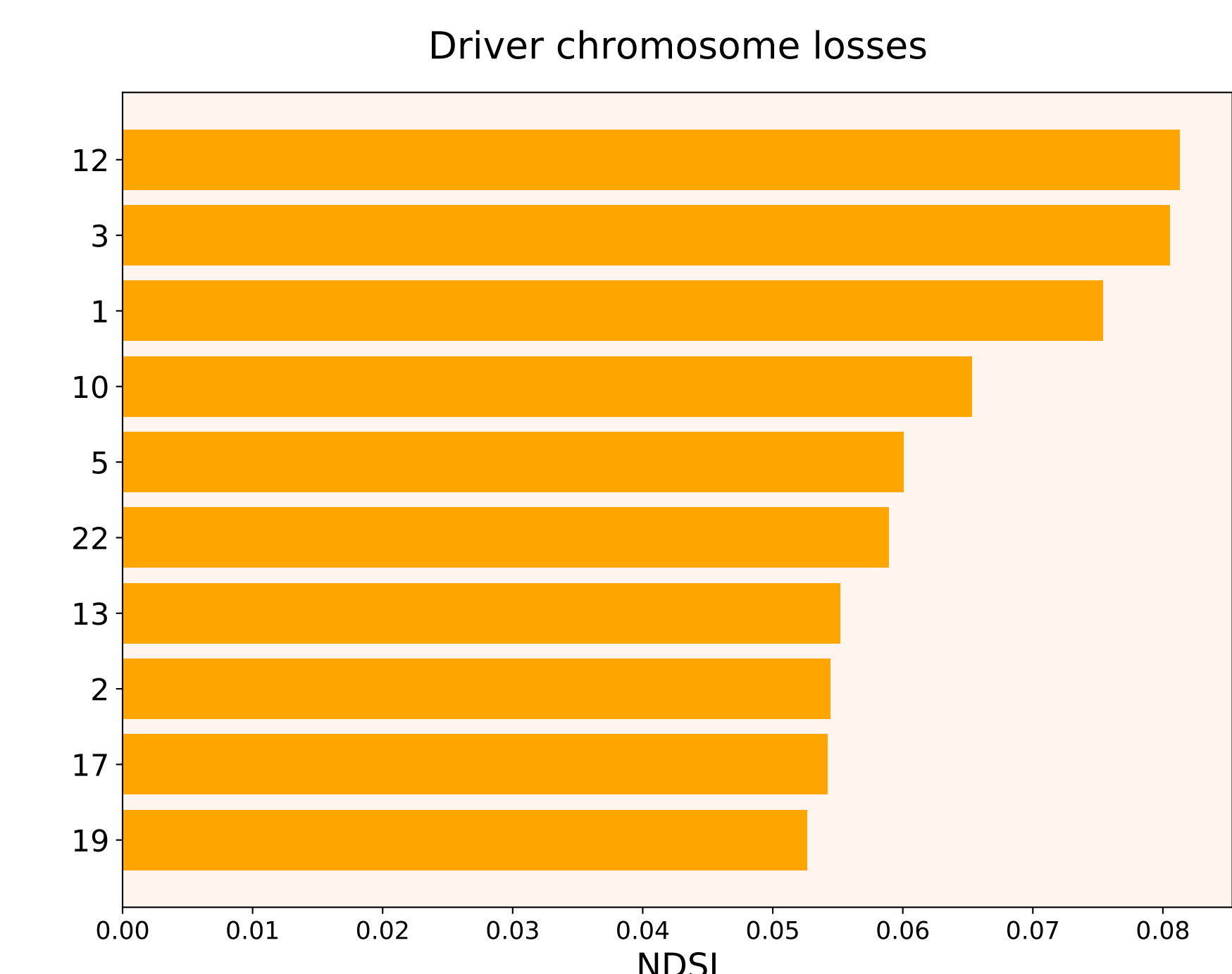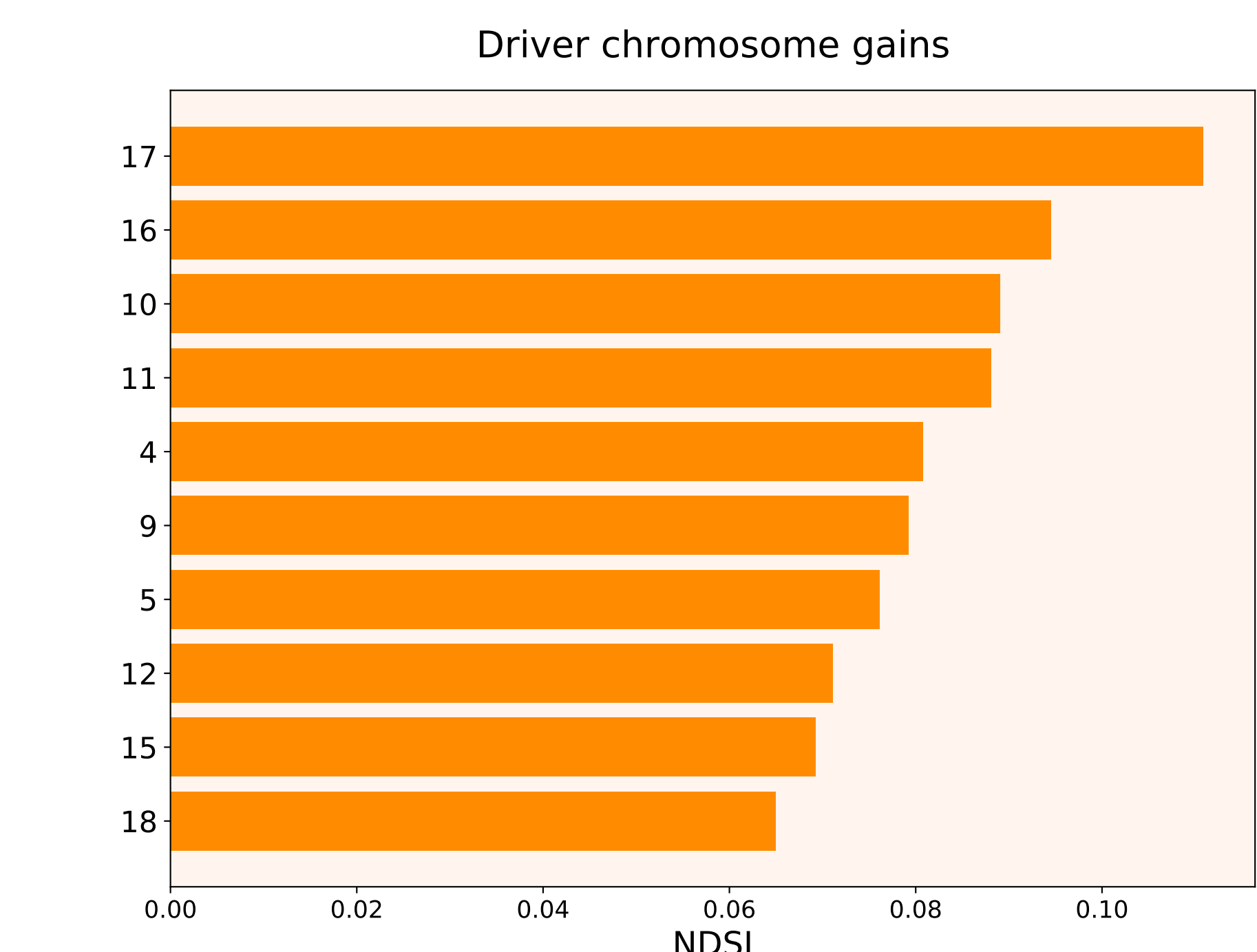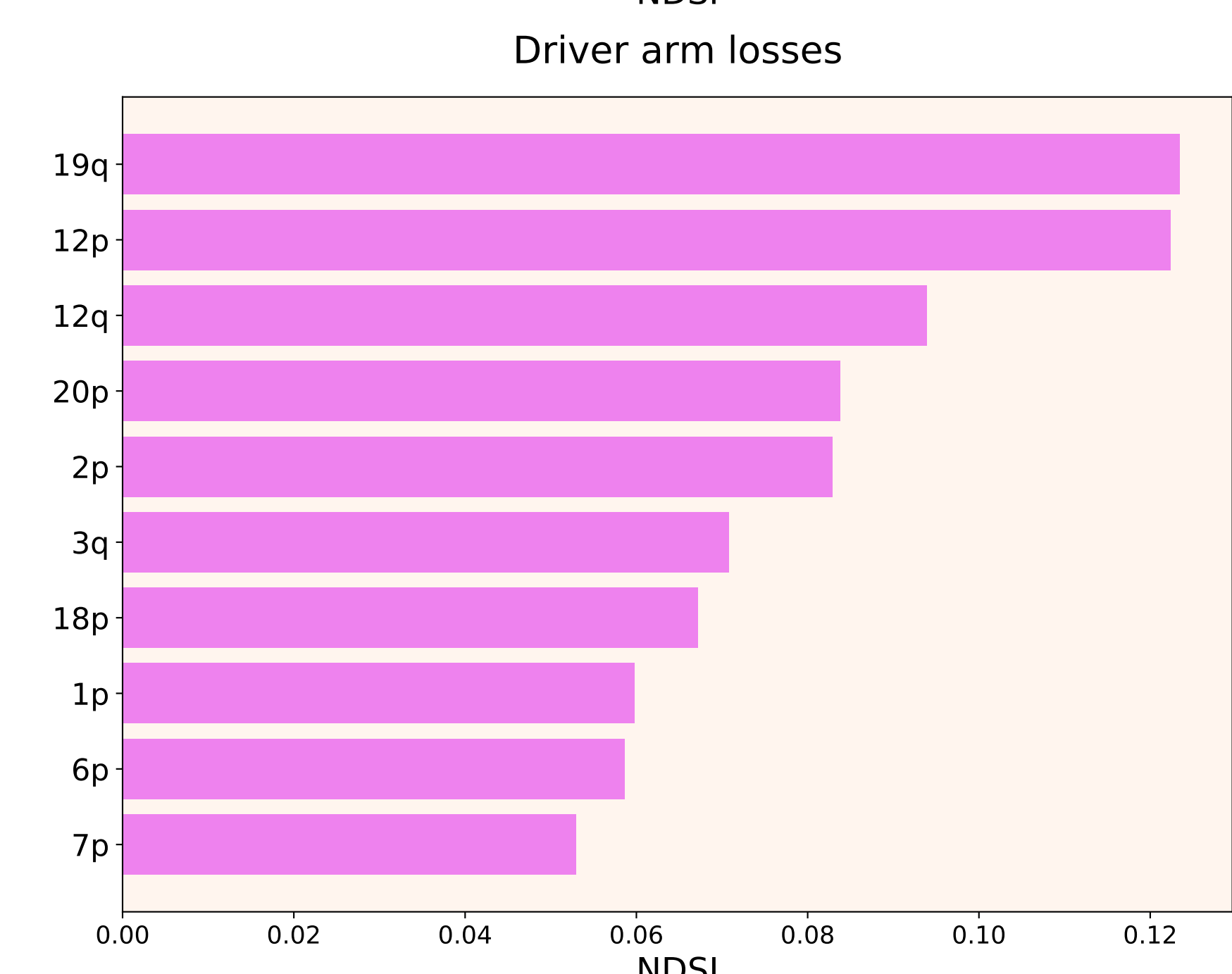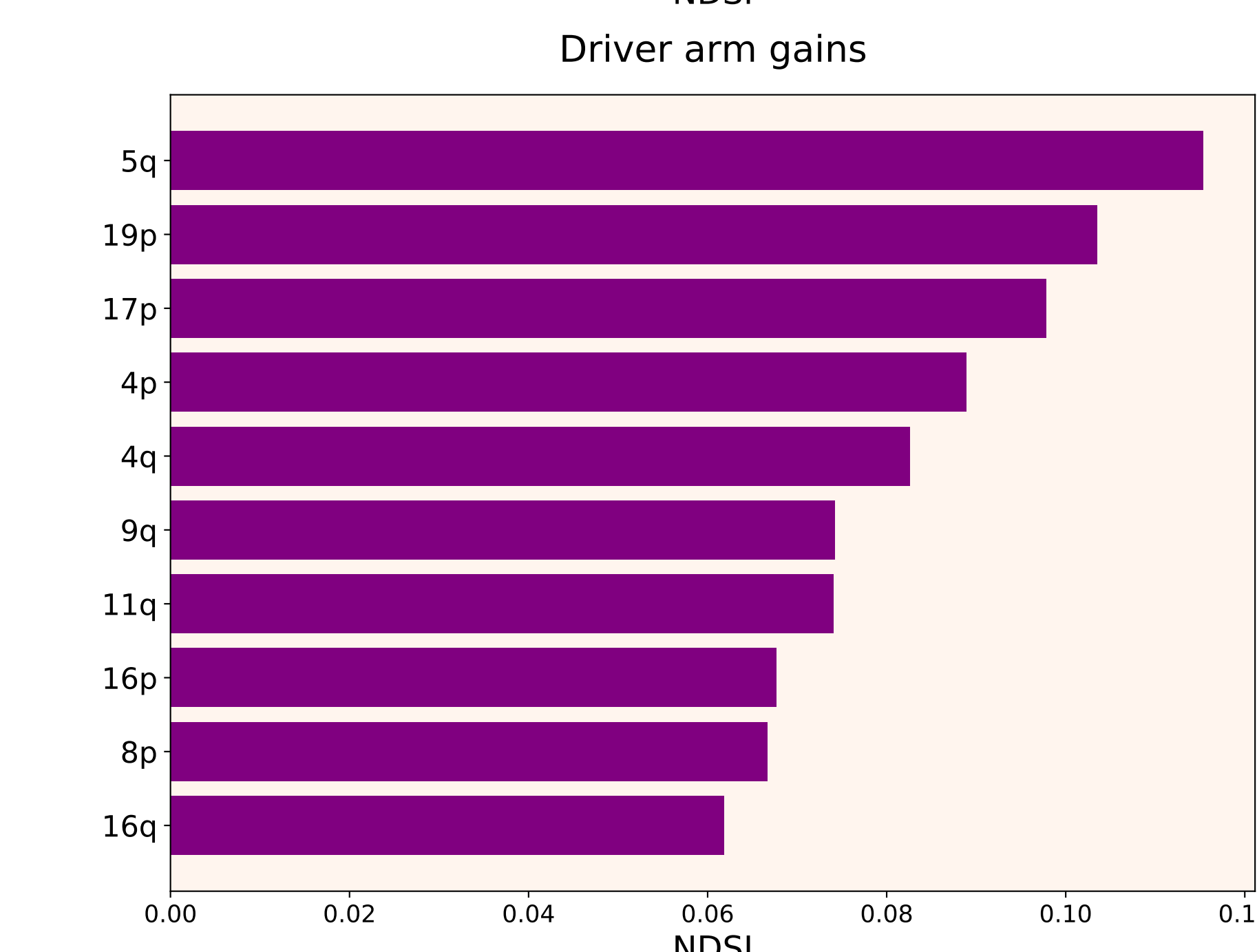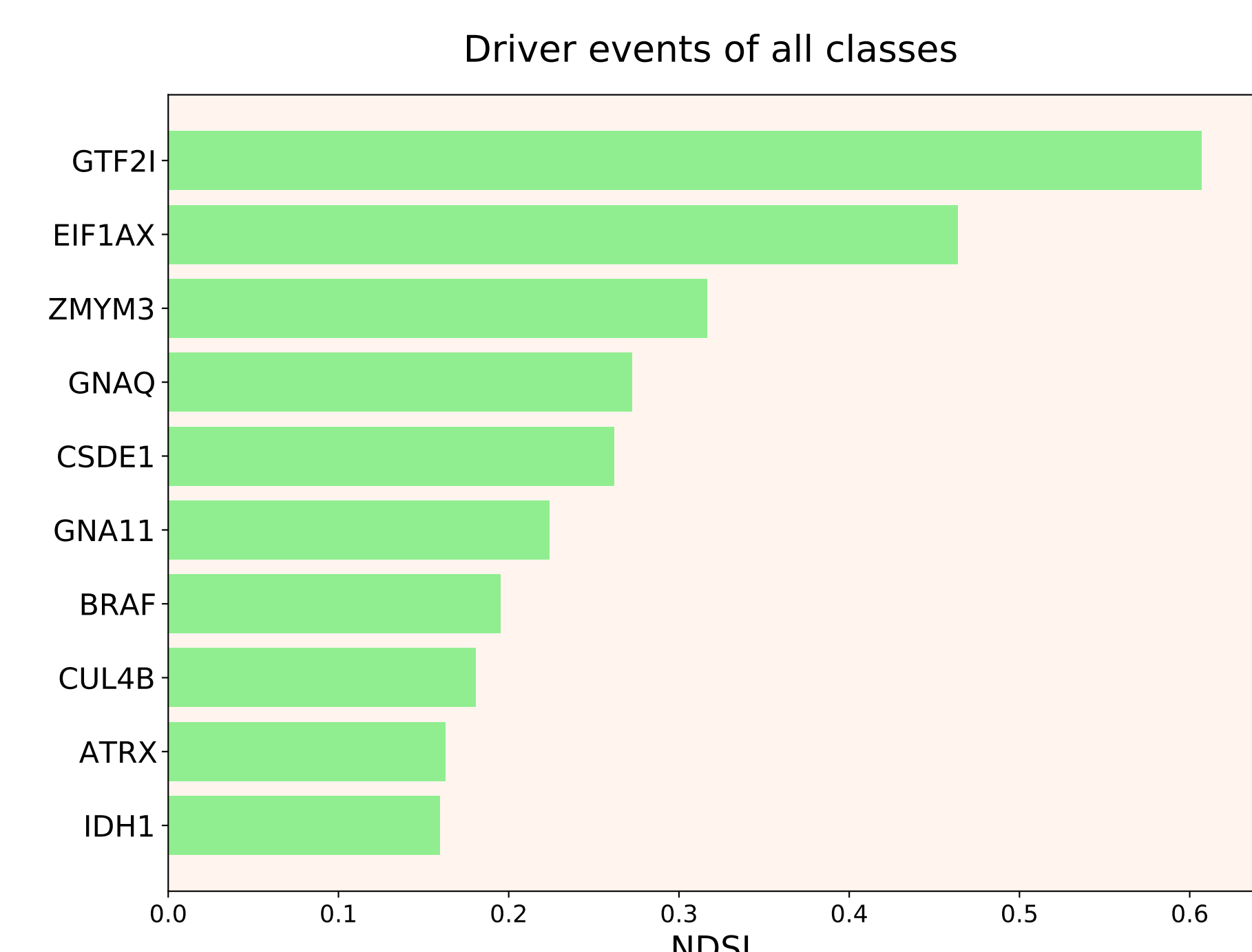

Supplement: Supplemental Information 2 [file peerj-10-13860-s002.zip › COHORTS/genes plots/indices/2021_8_16_14_9_distribution_events_detailed_NDSI.pdf]

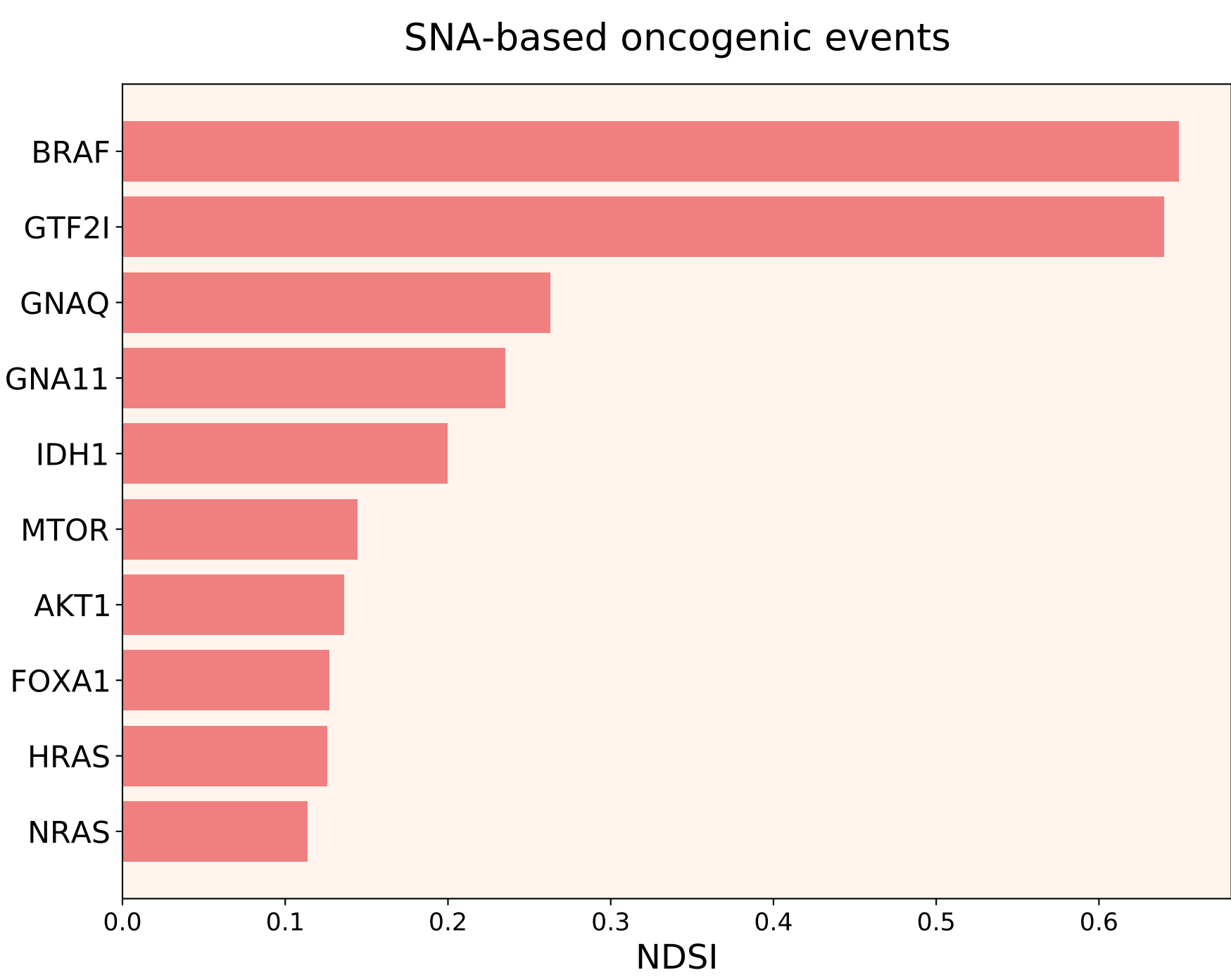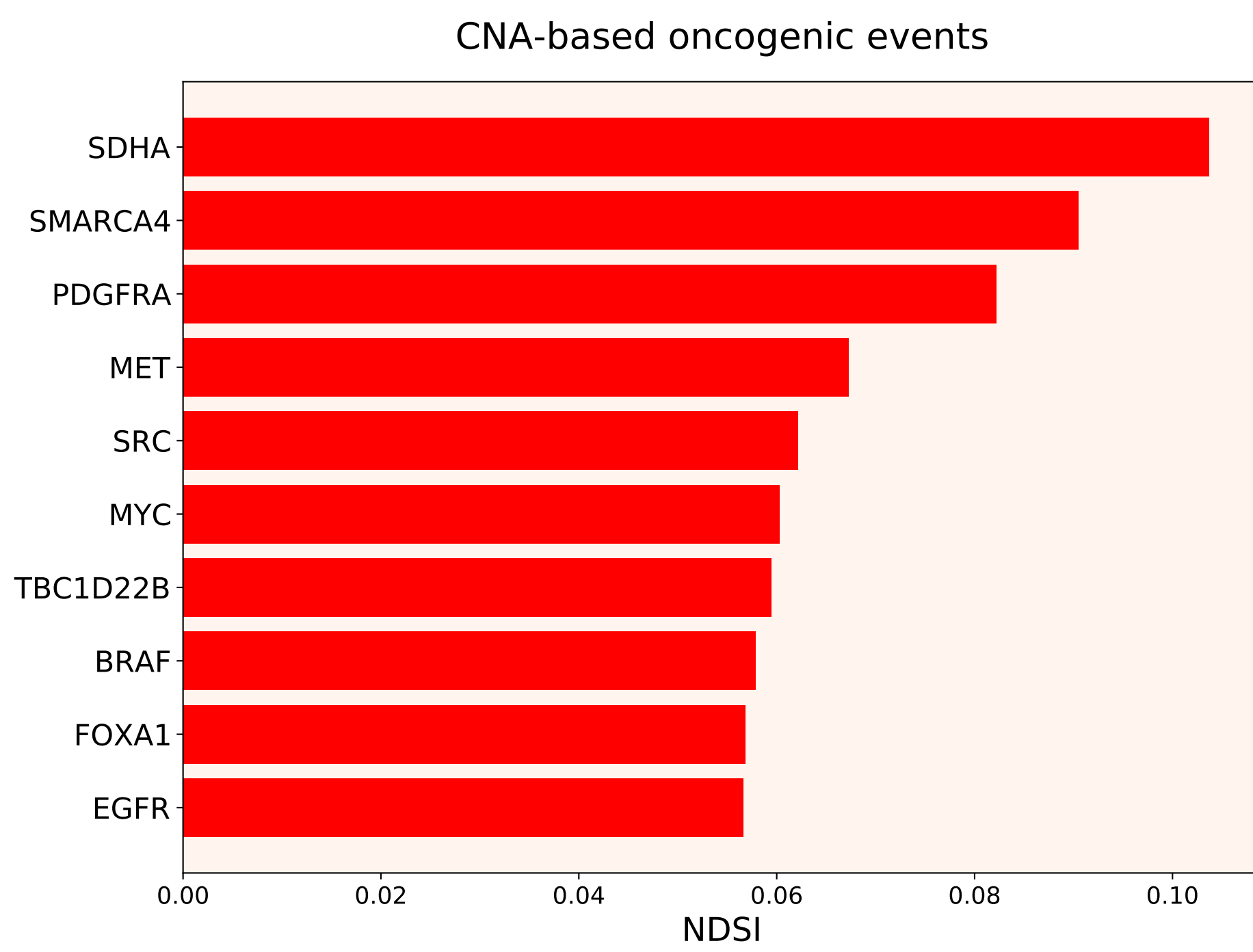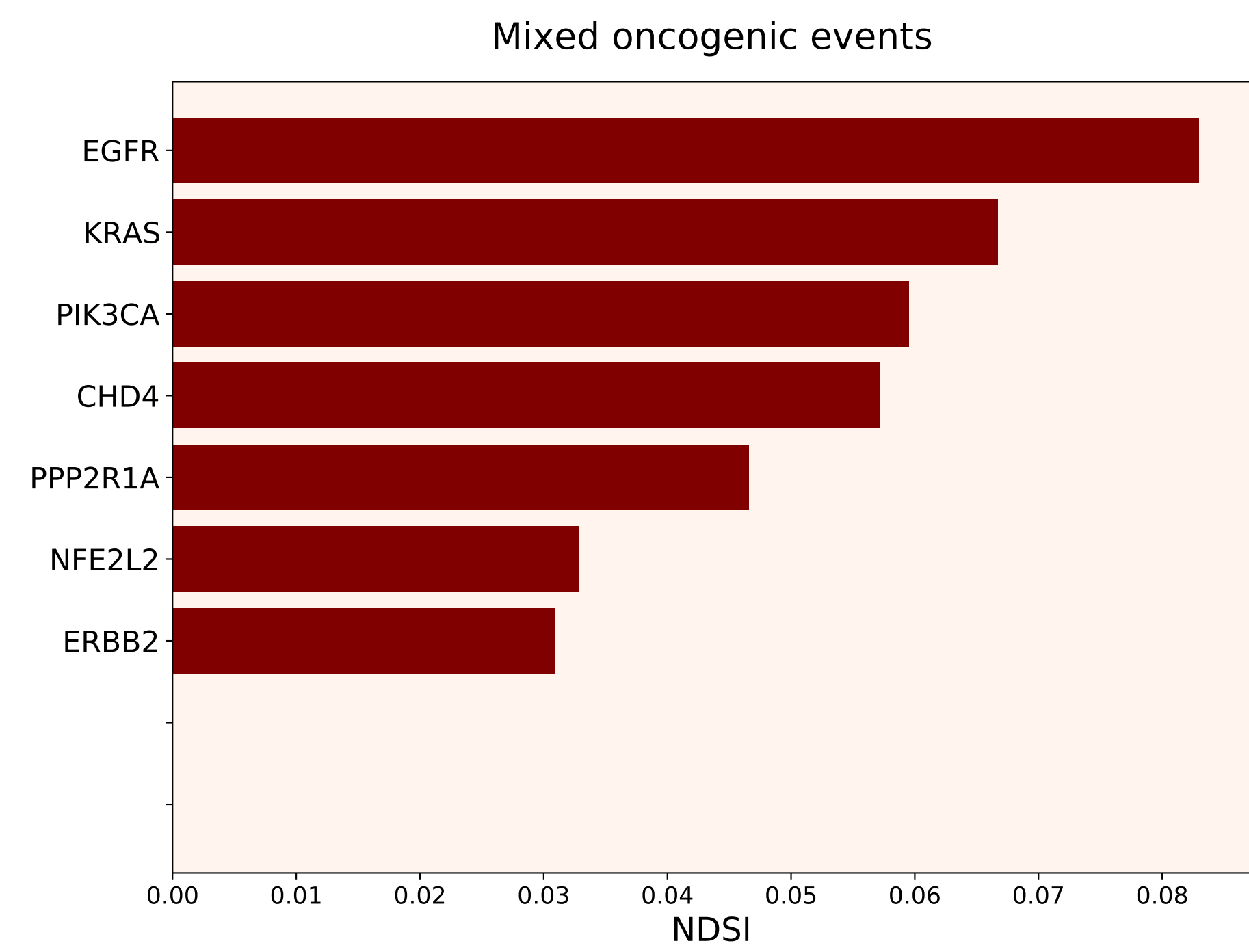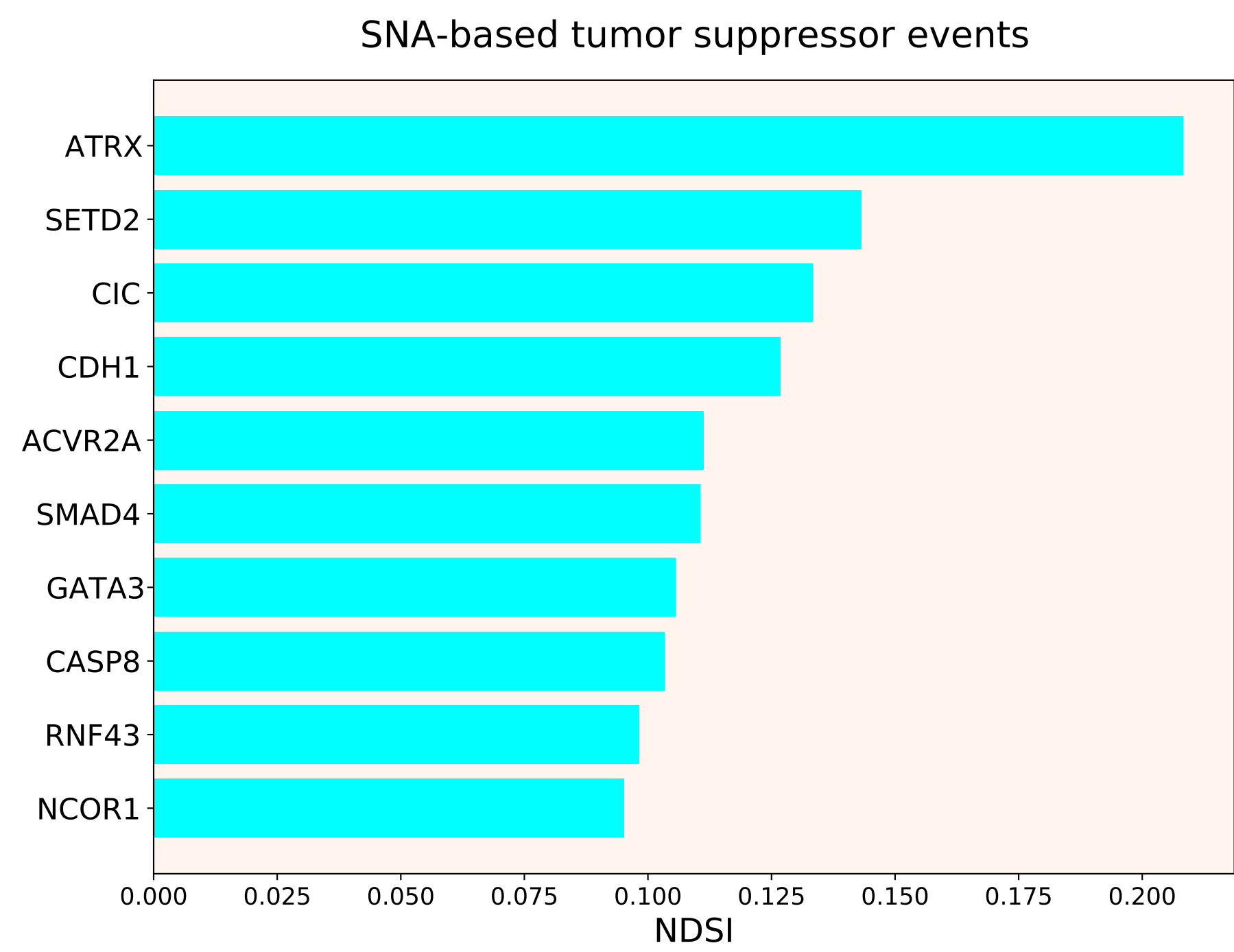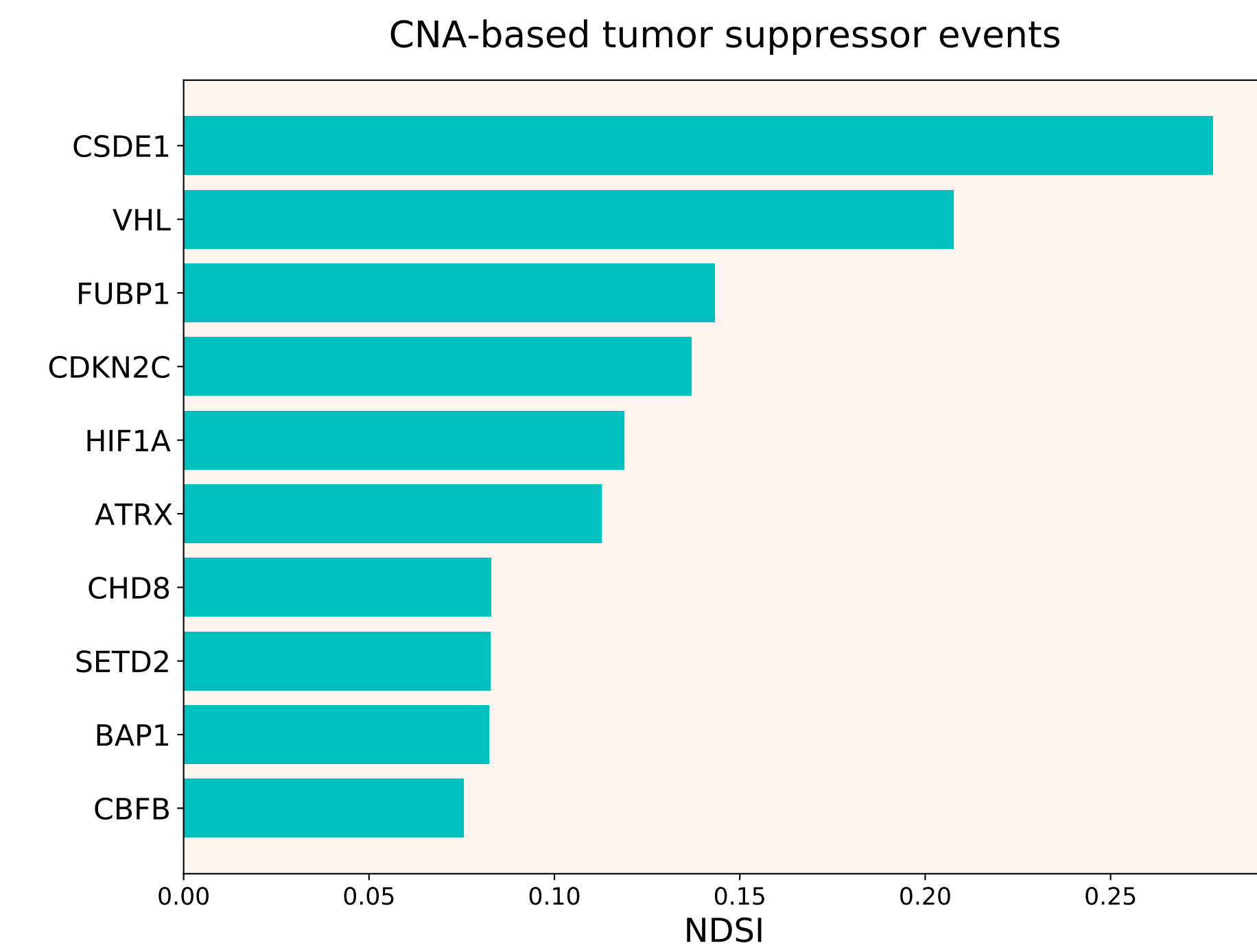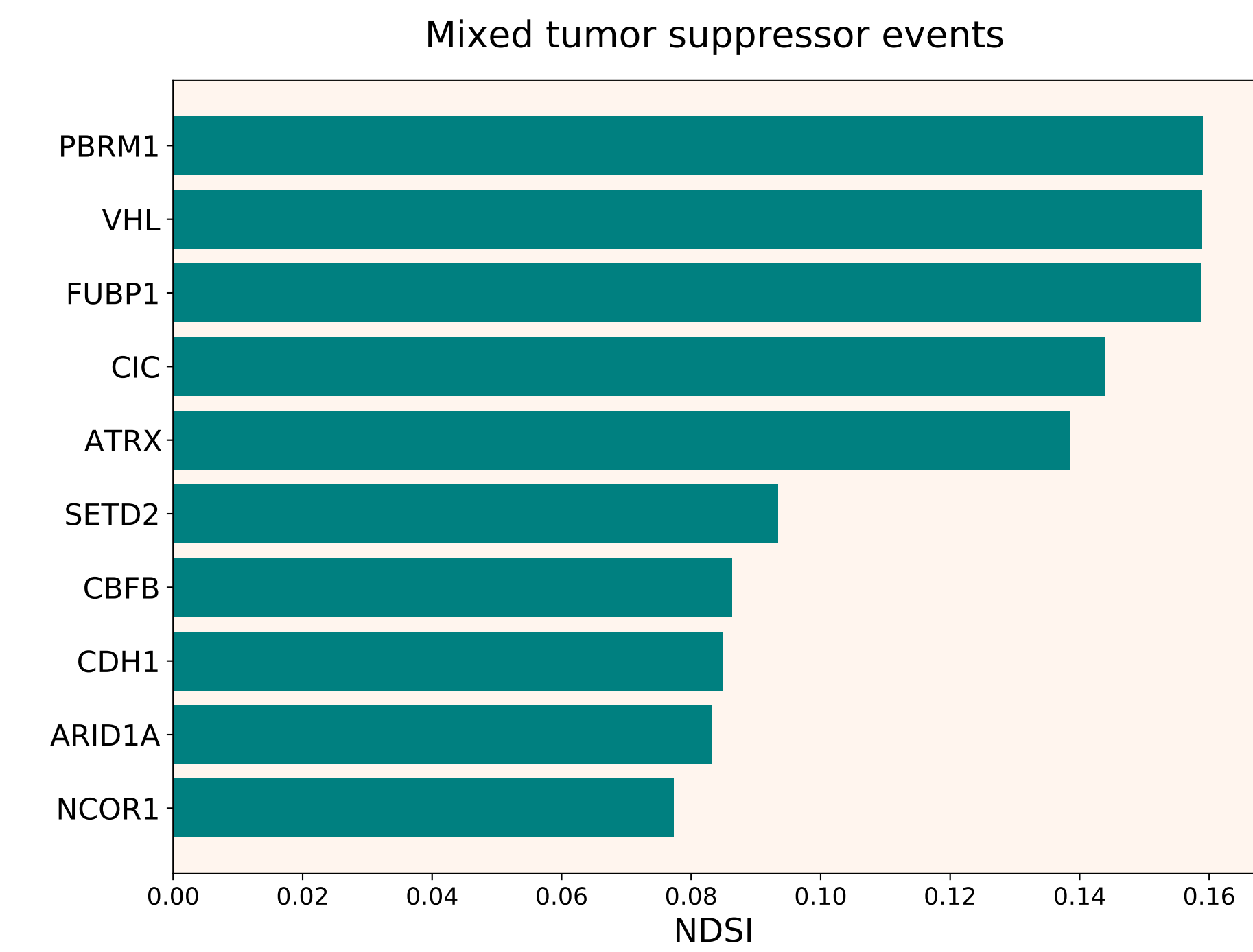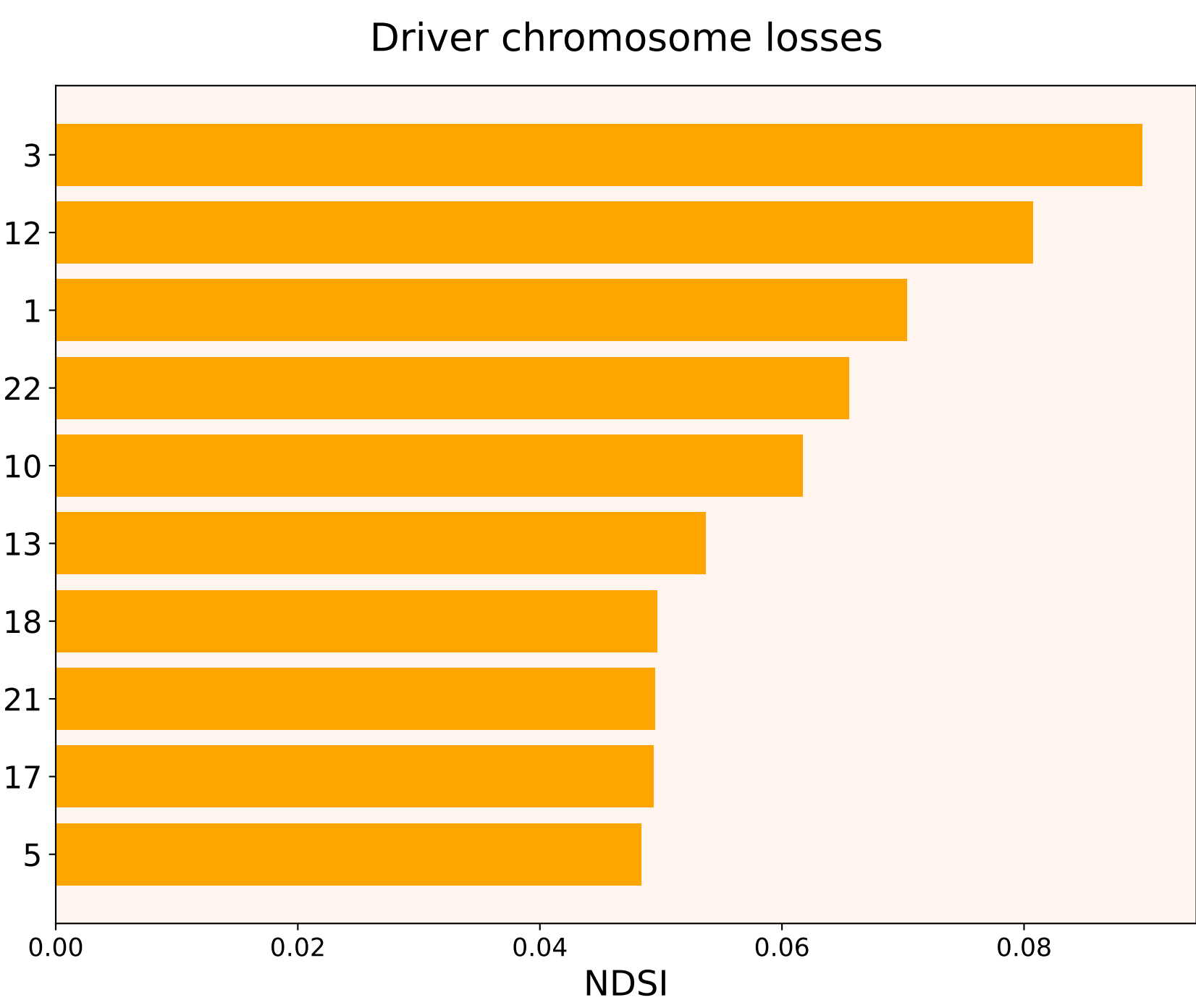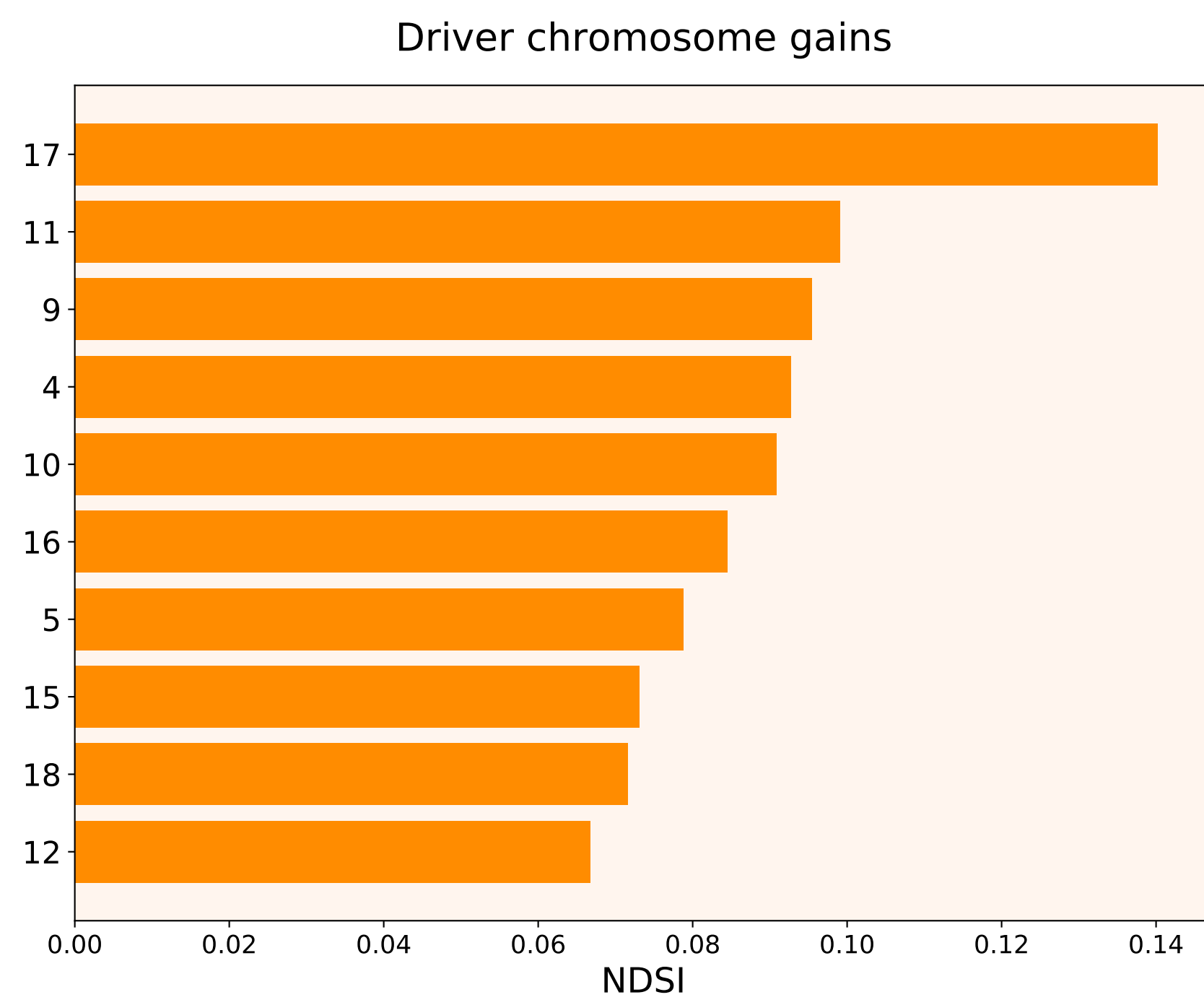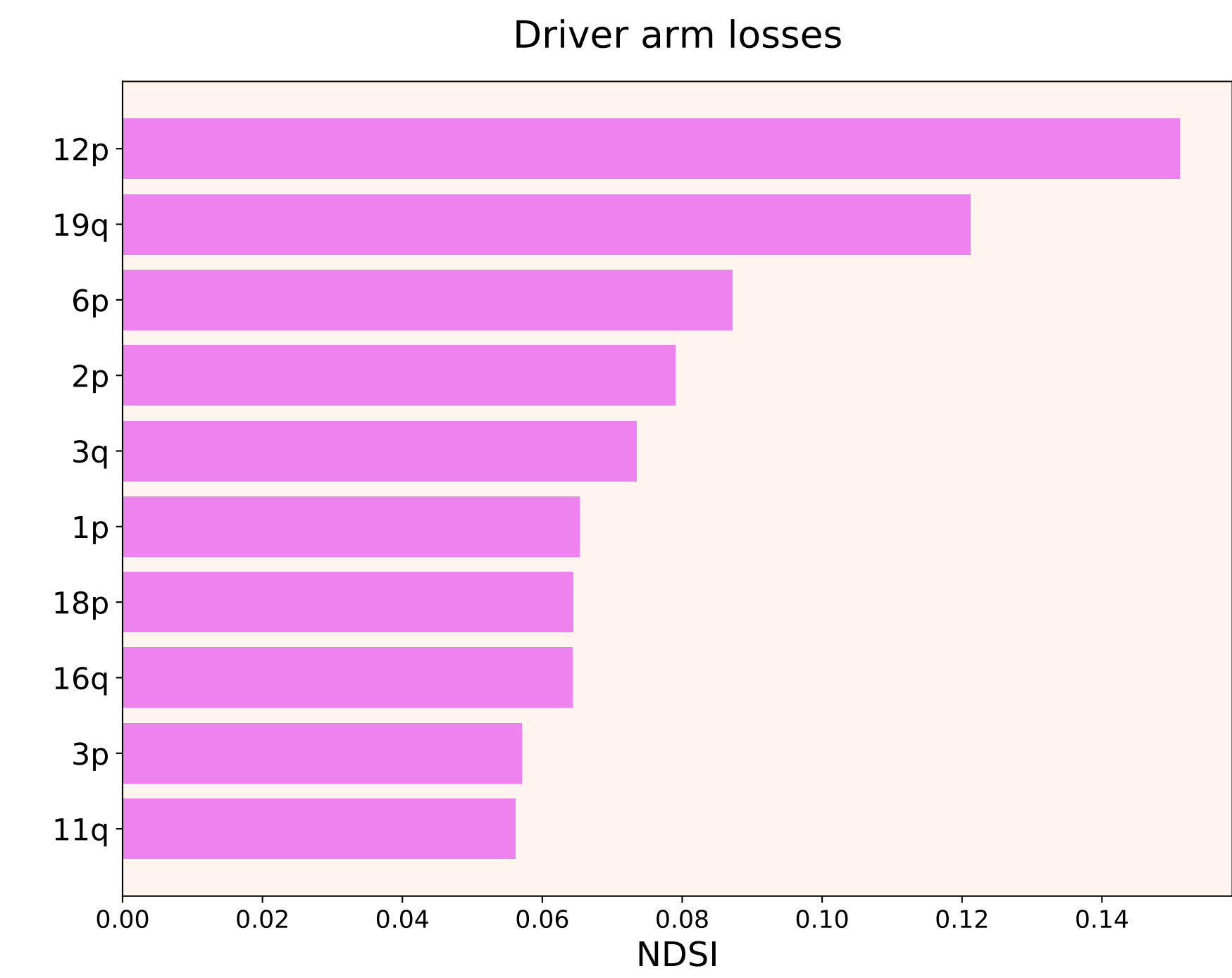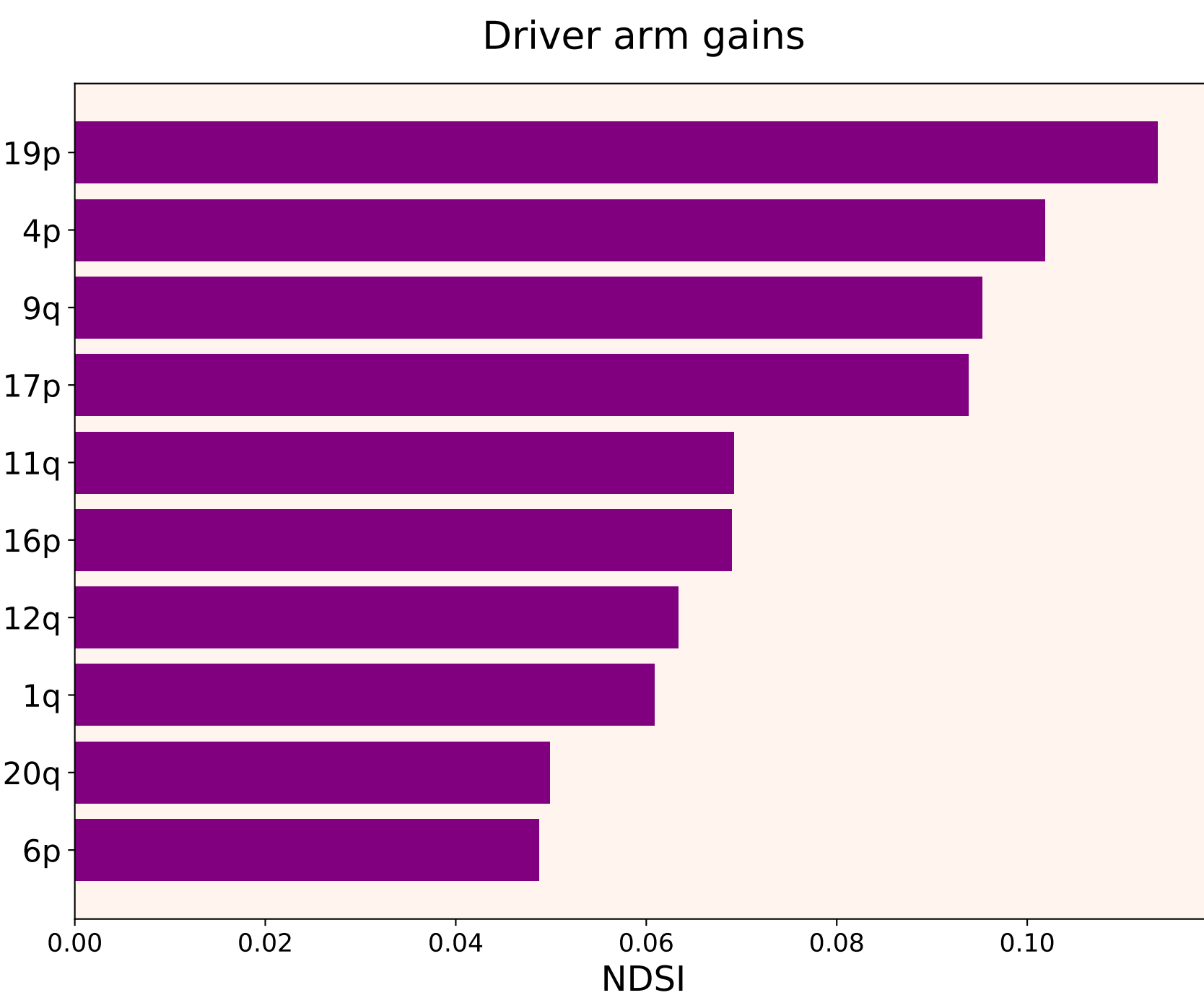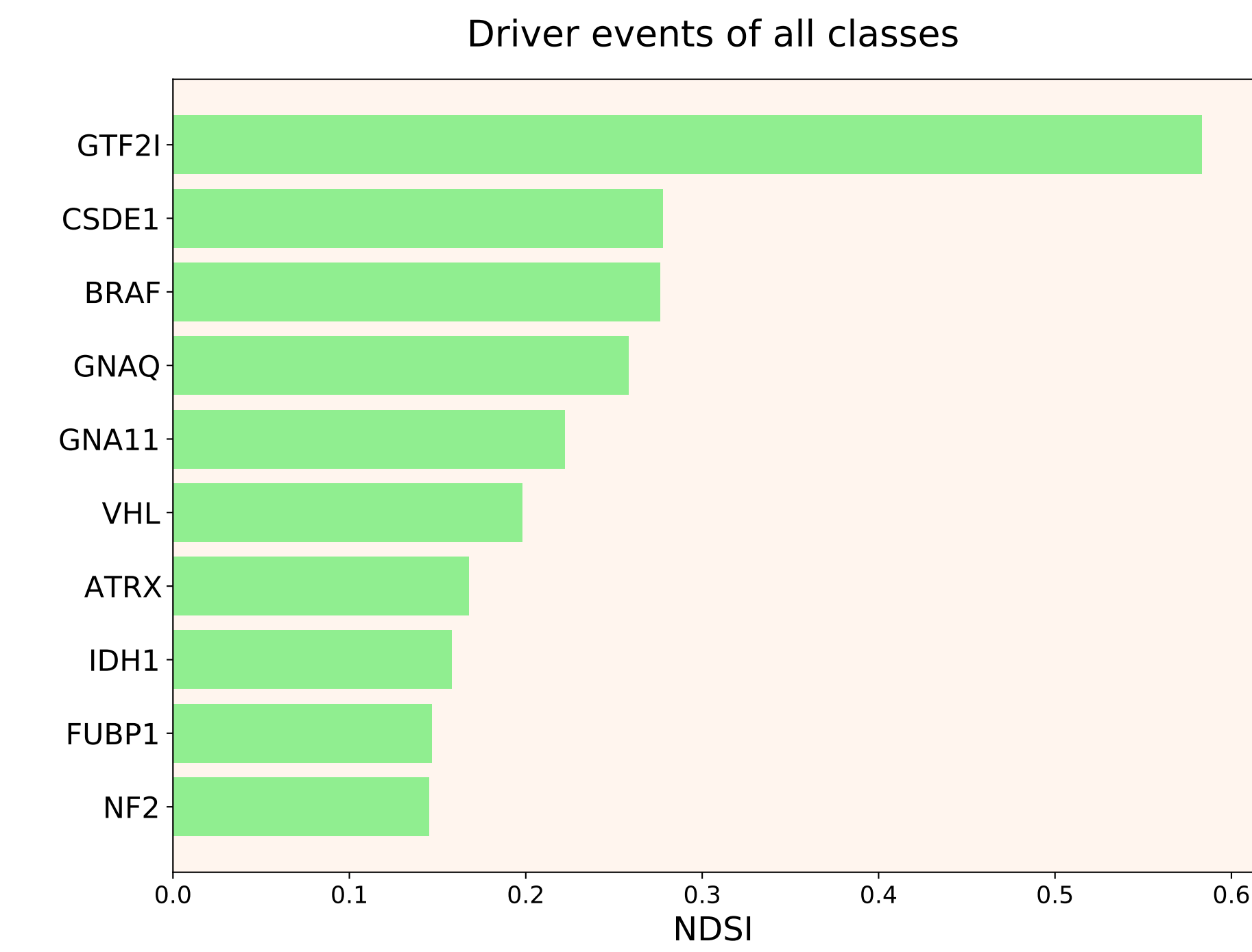

Supplement: Supplemental Information 2 [file peerj-10-13860-s002.zip › COHORTS/genes plots/indices/2021_8_16_14_9_distribution_events_detailed_NDSI_females.pdf]

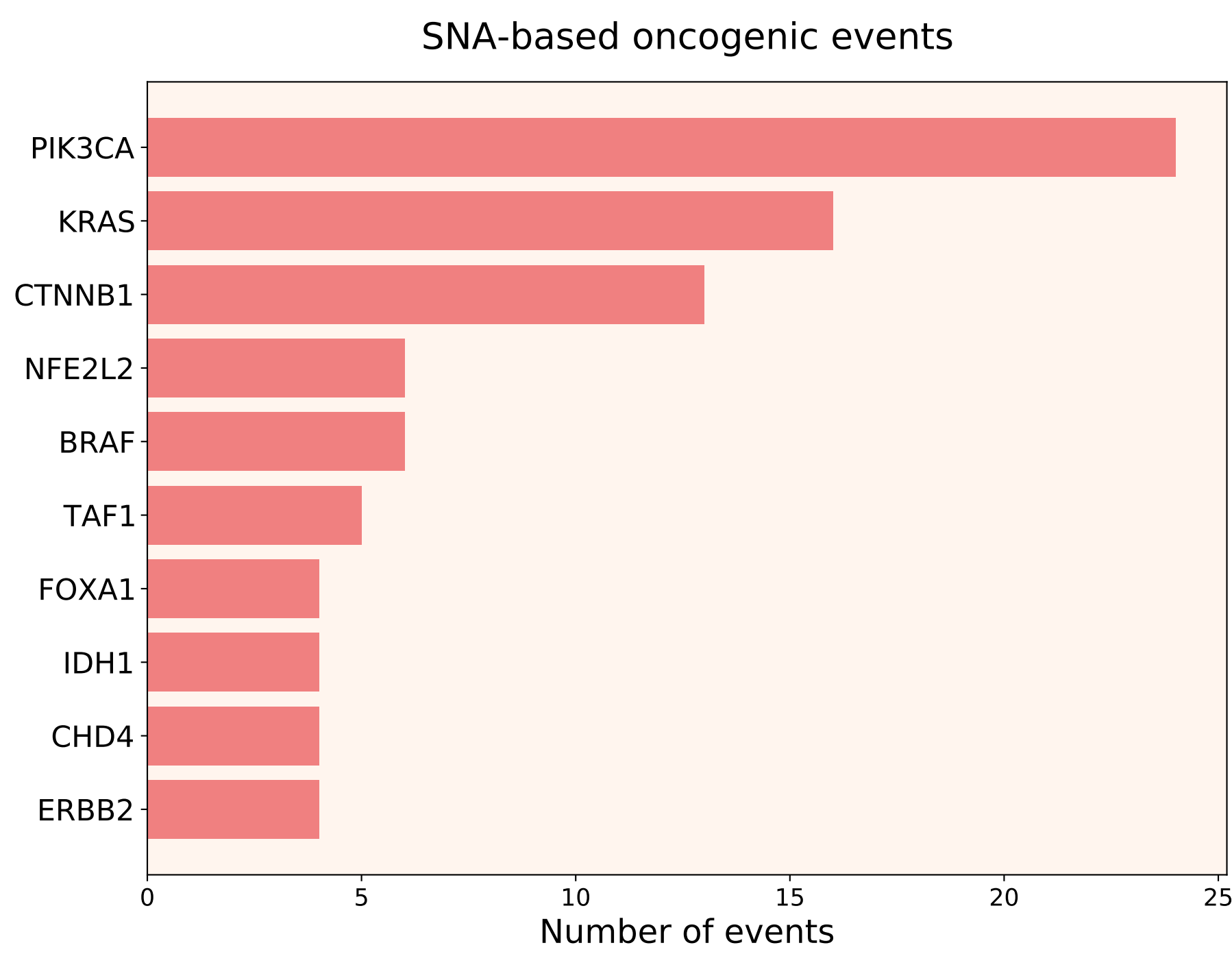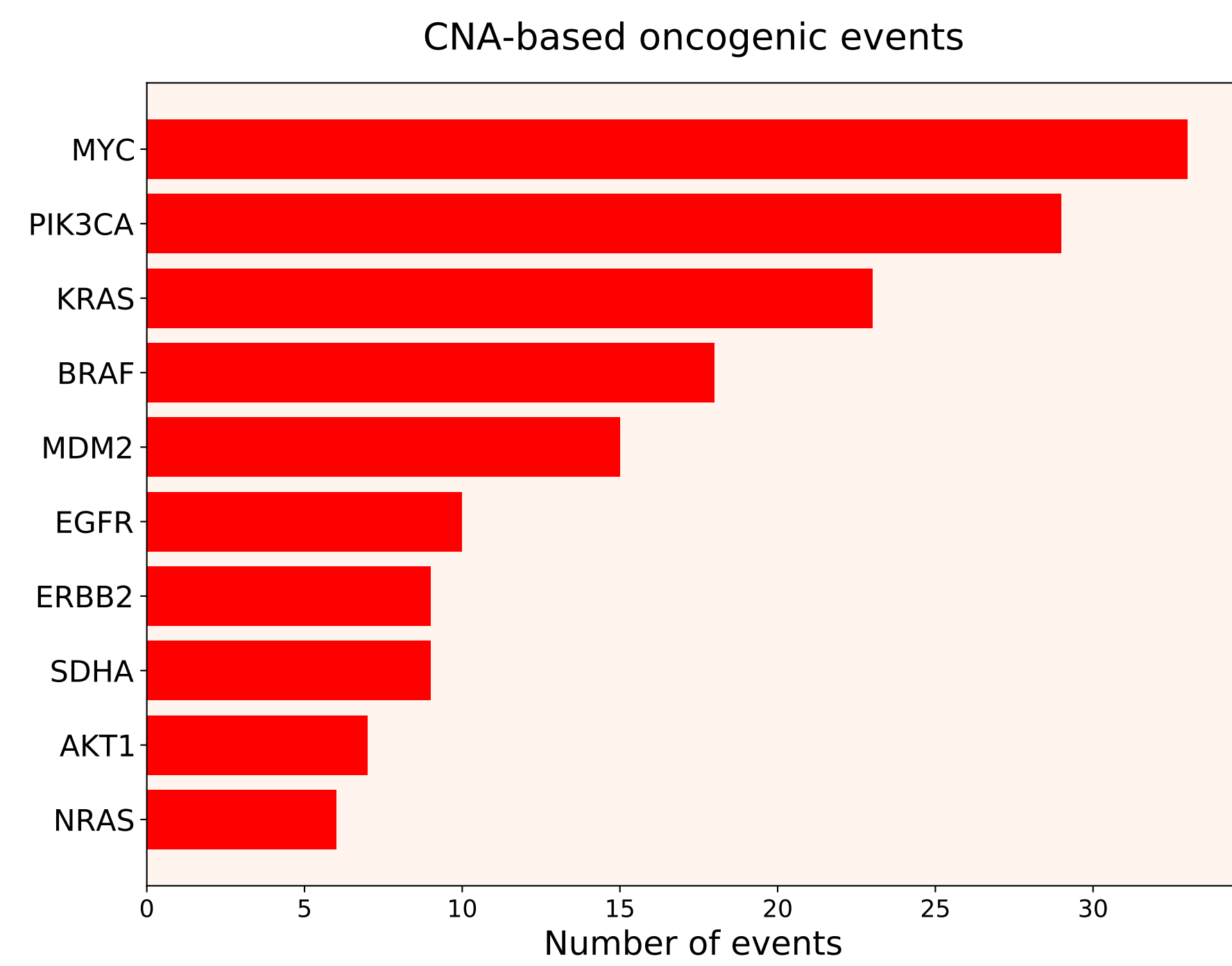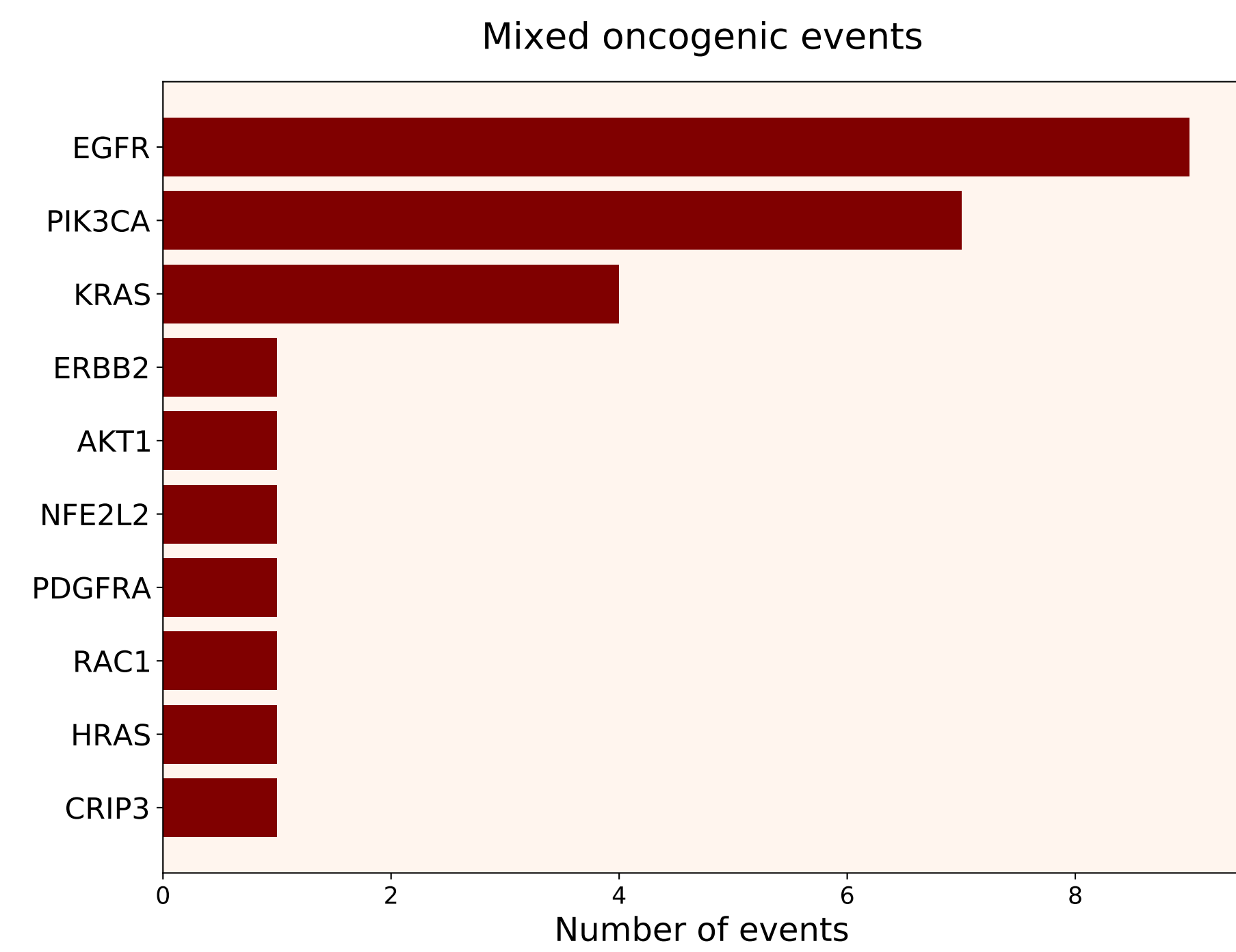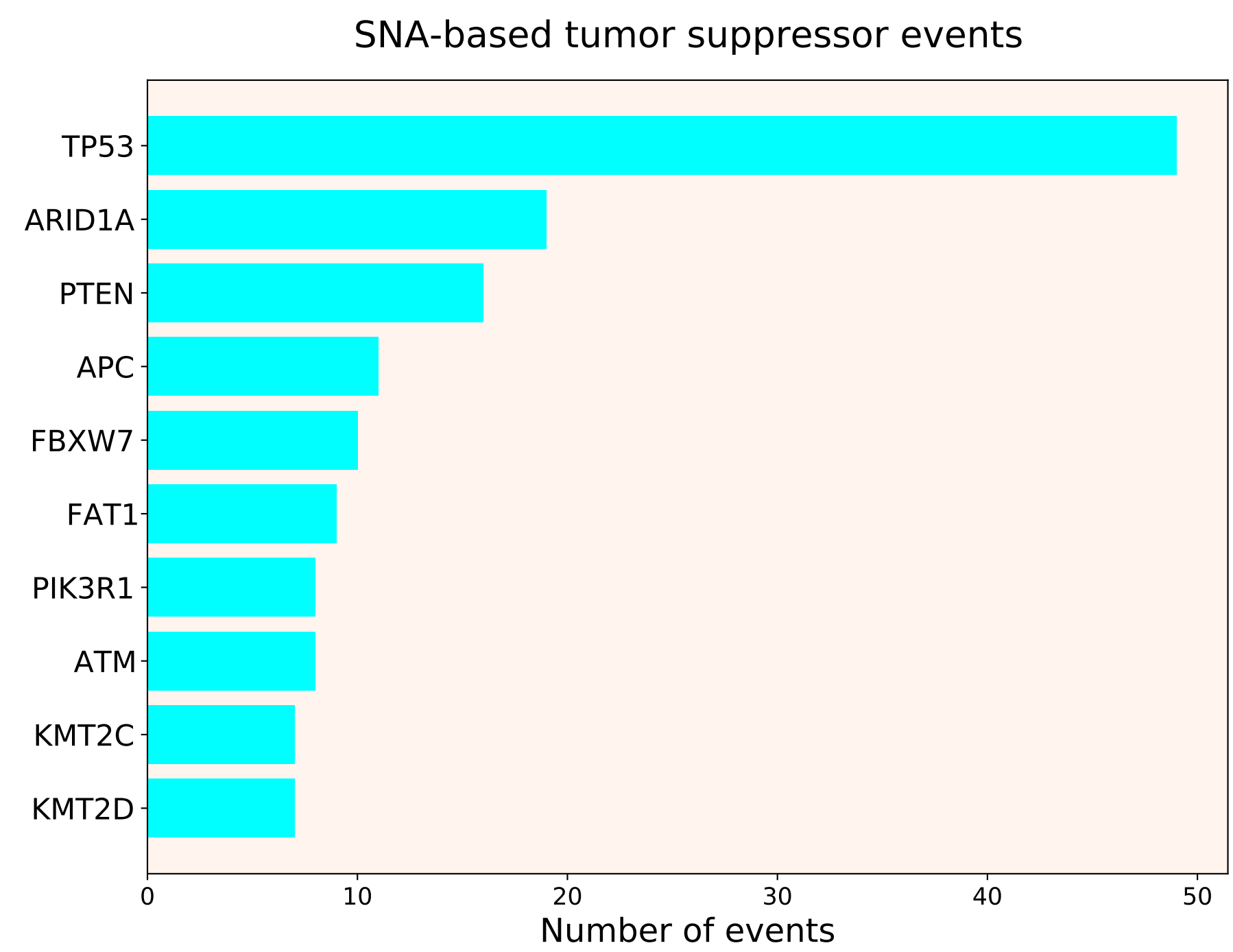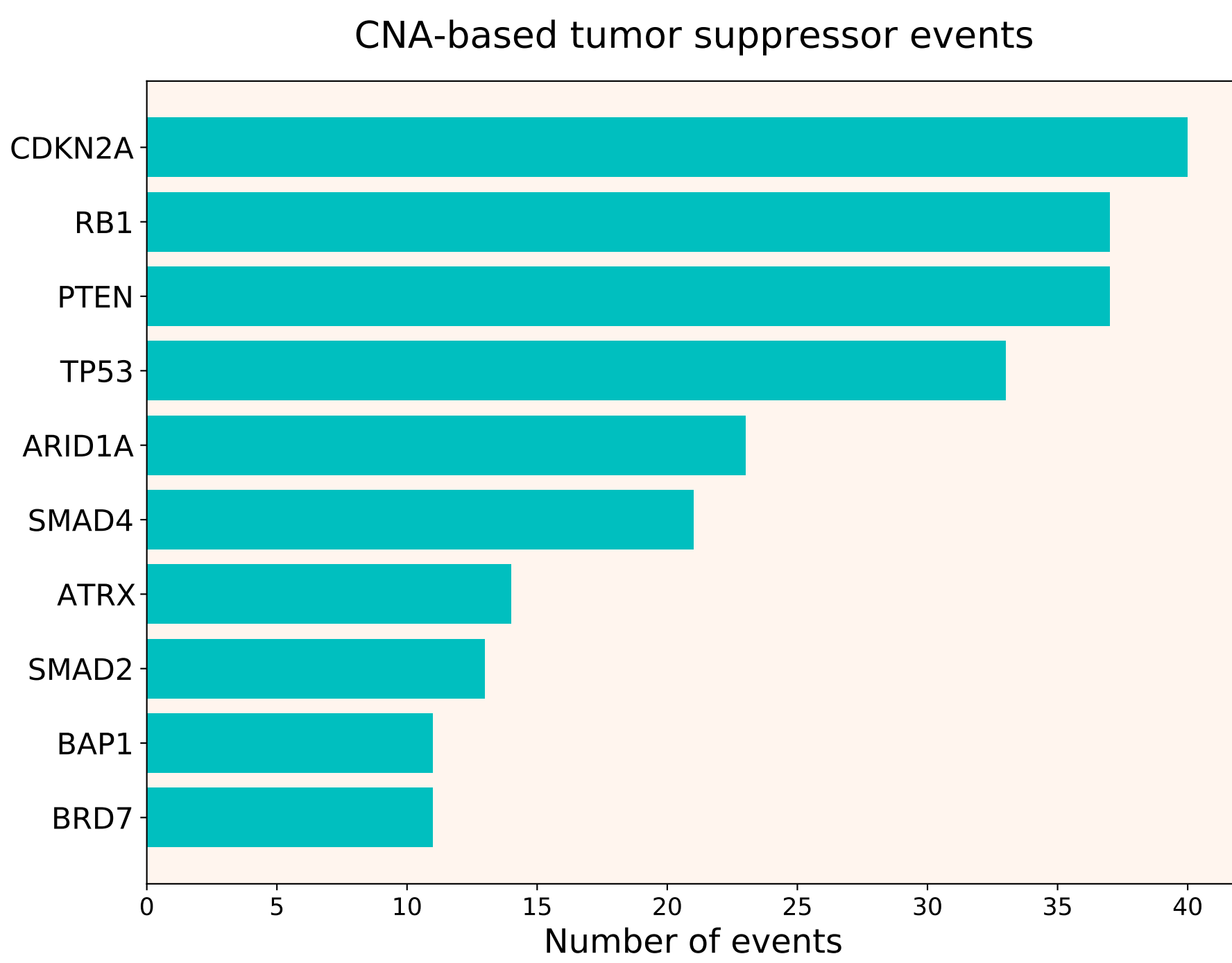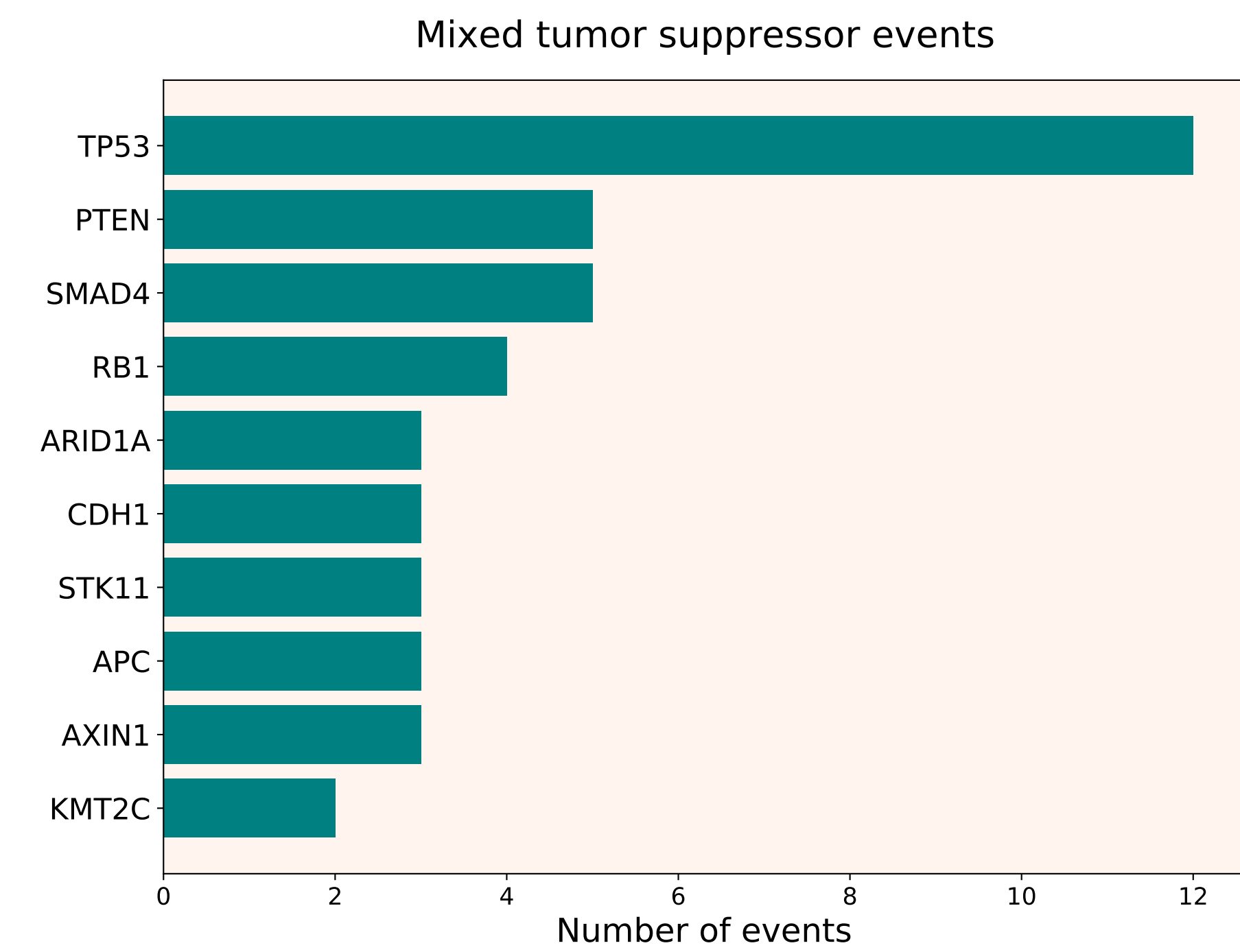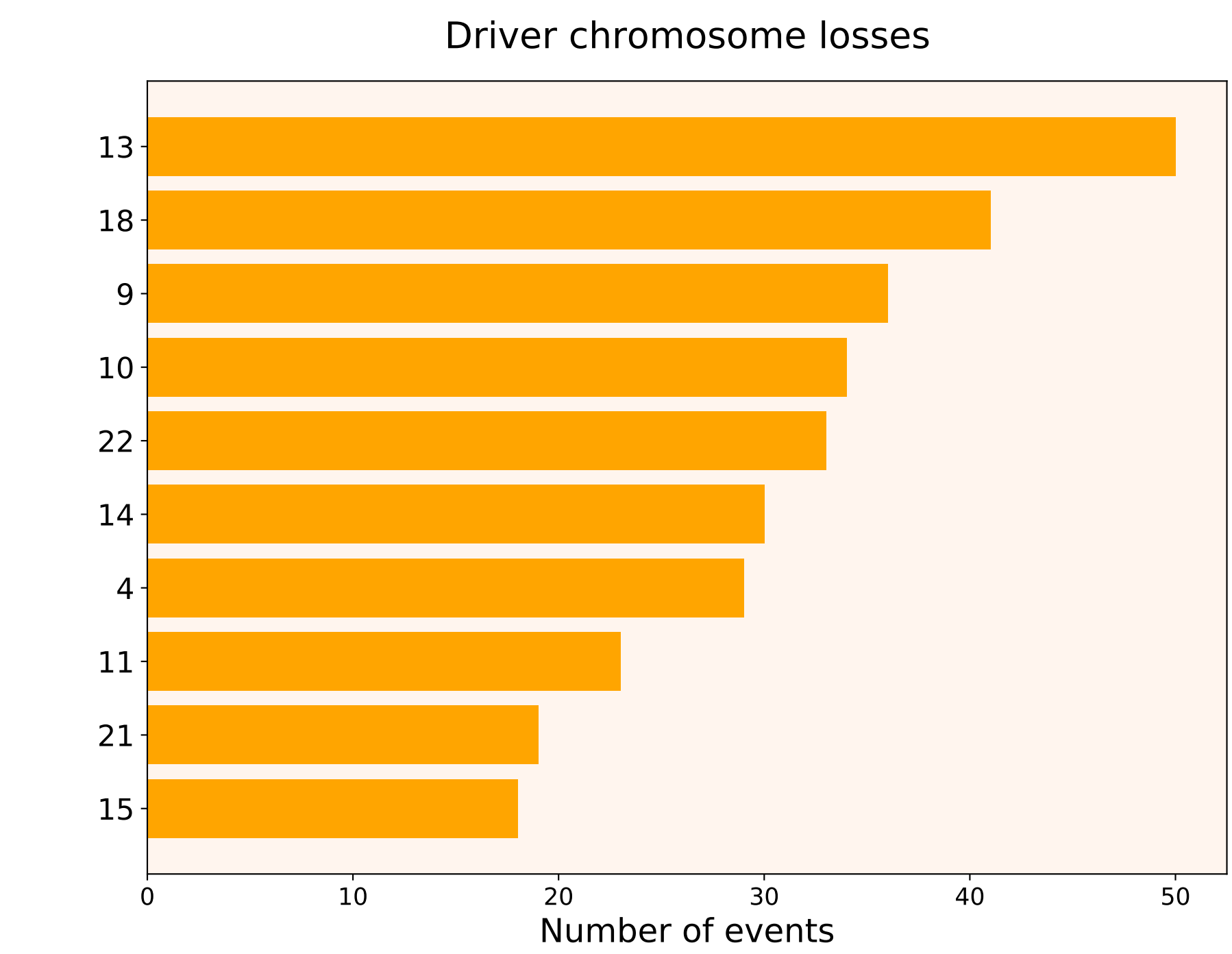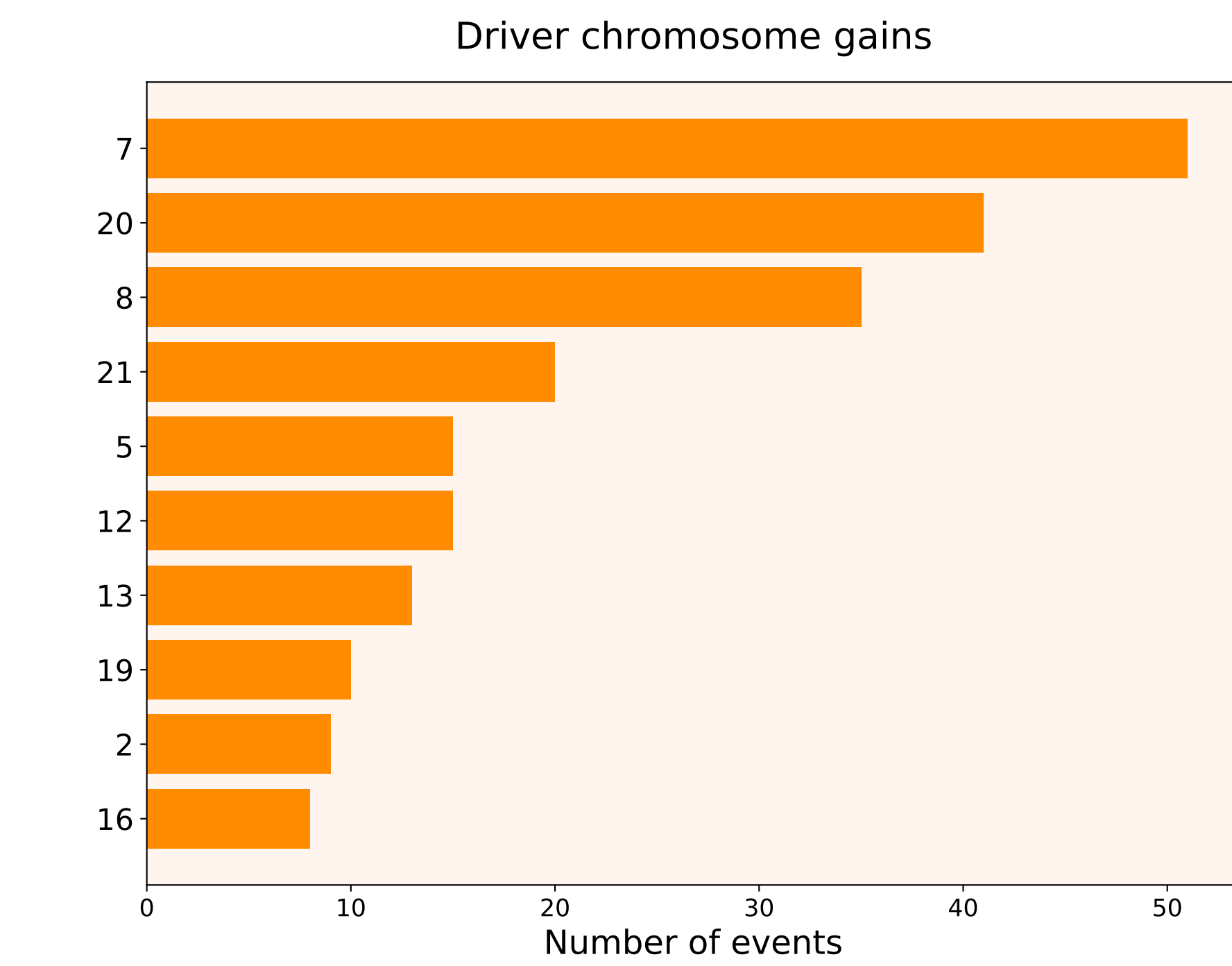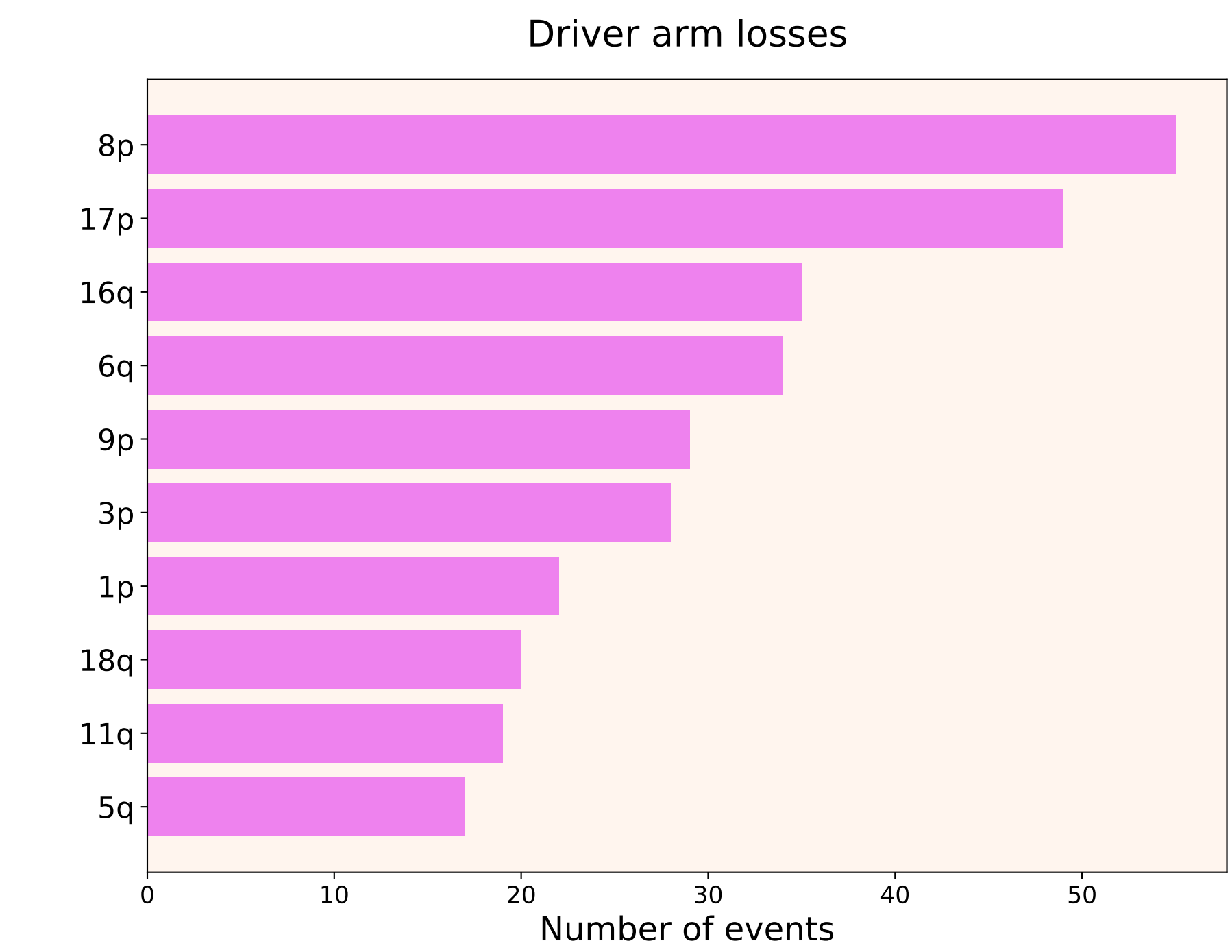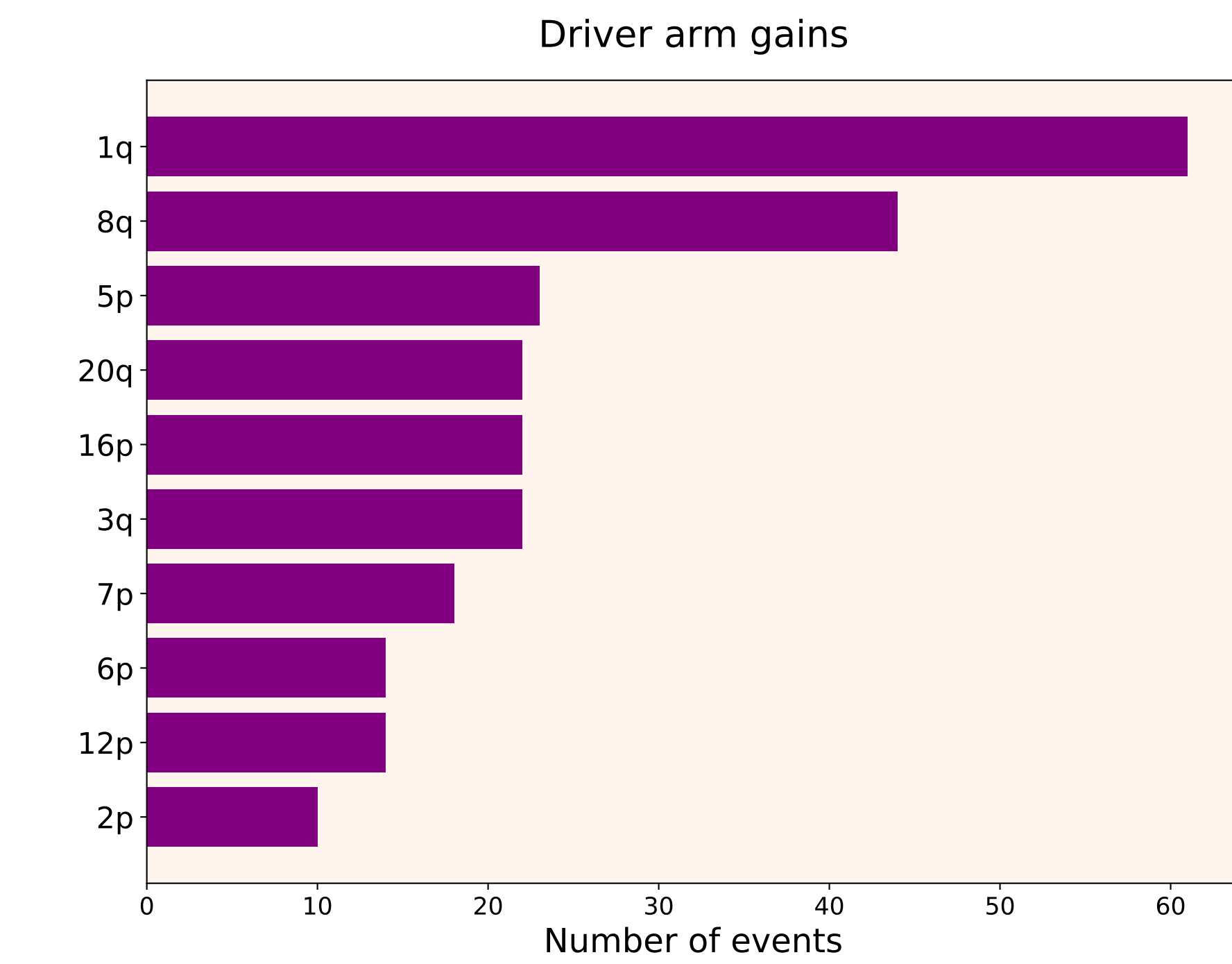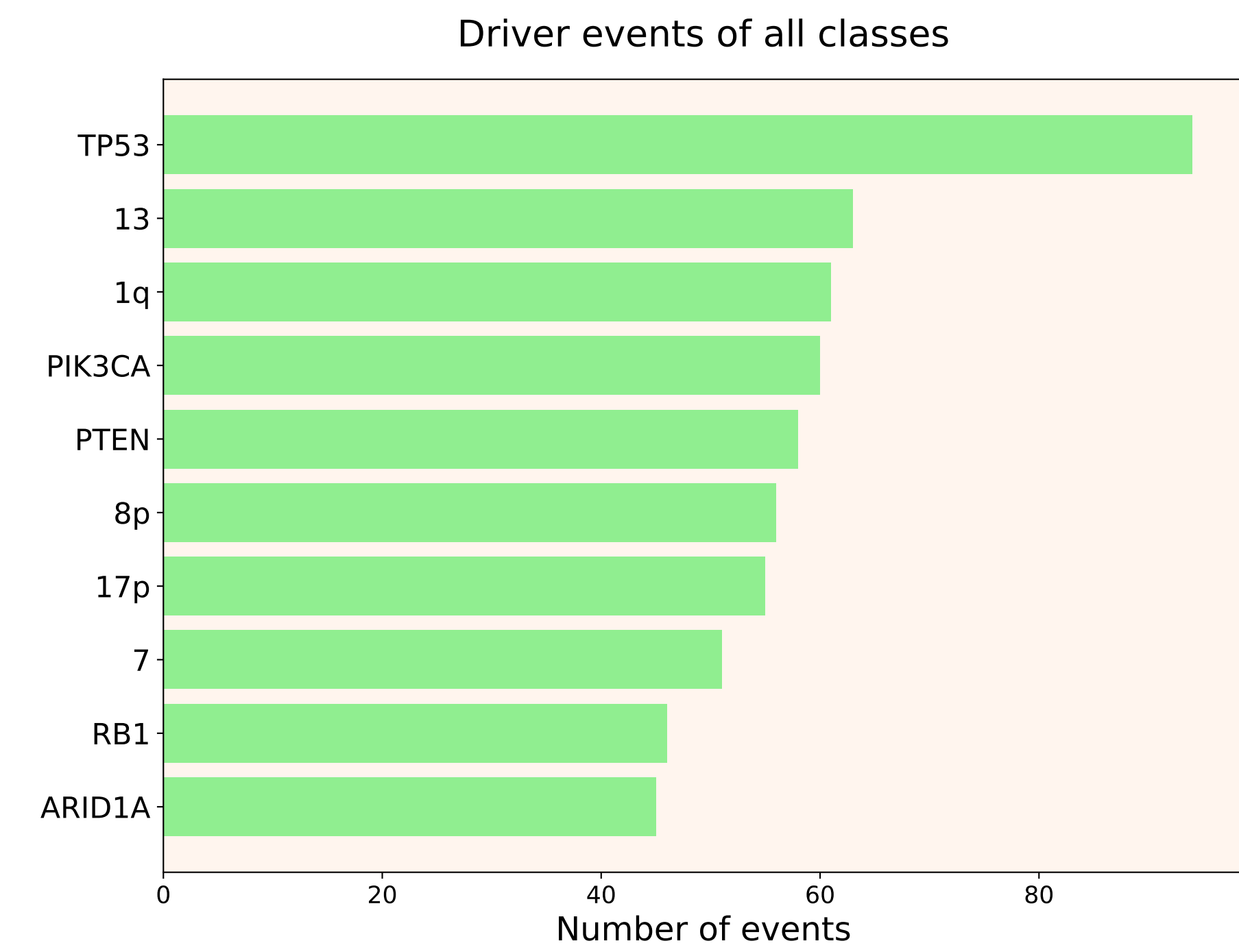

Supplement: Supplemental Information 2 [file peerj-10-13860-s002.zip › COHORTS/genes plots/detailed/2021_8_16_14_9_distribution_events_detailed_13.pdf]

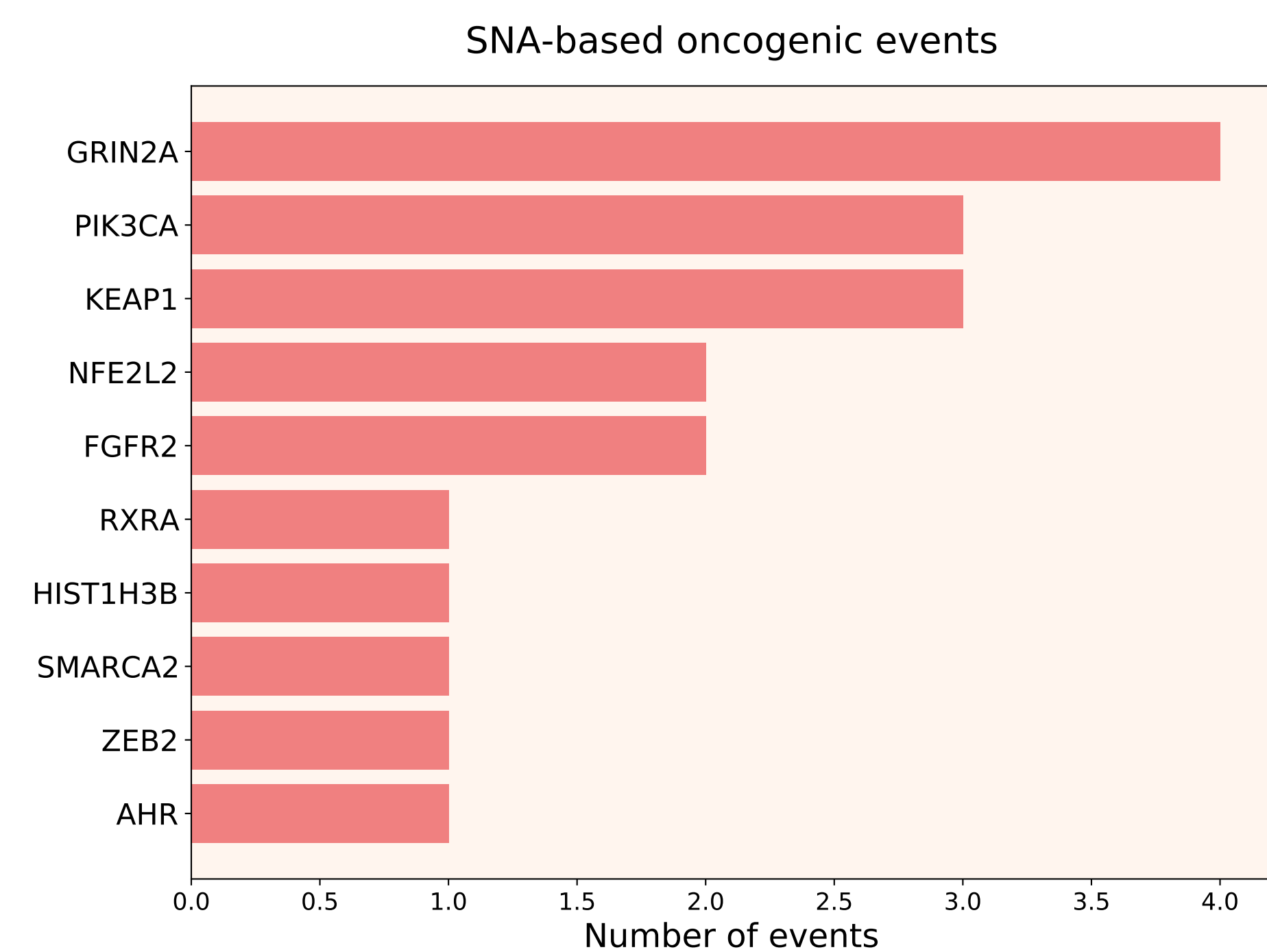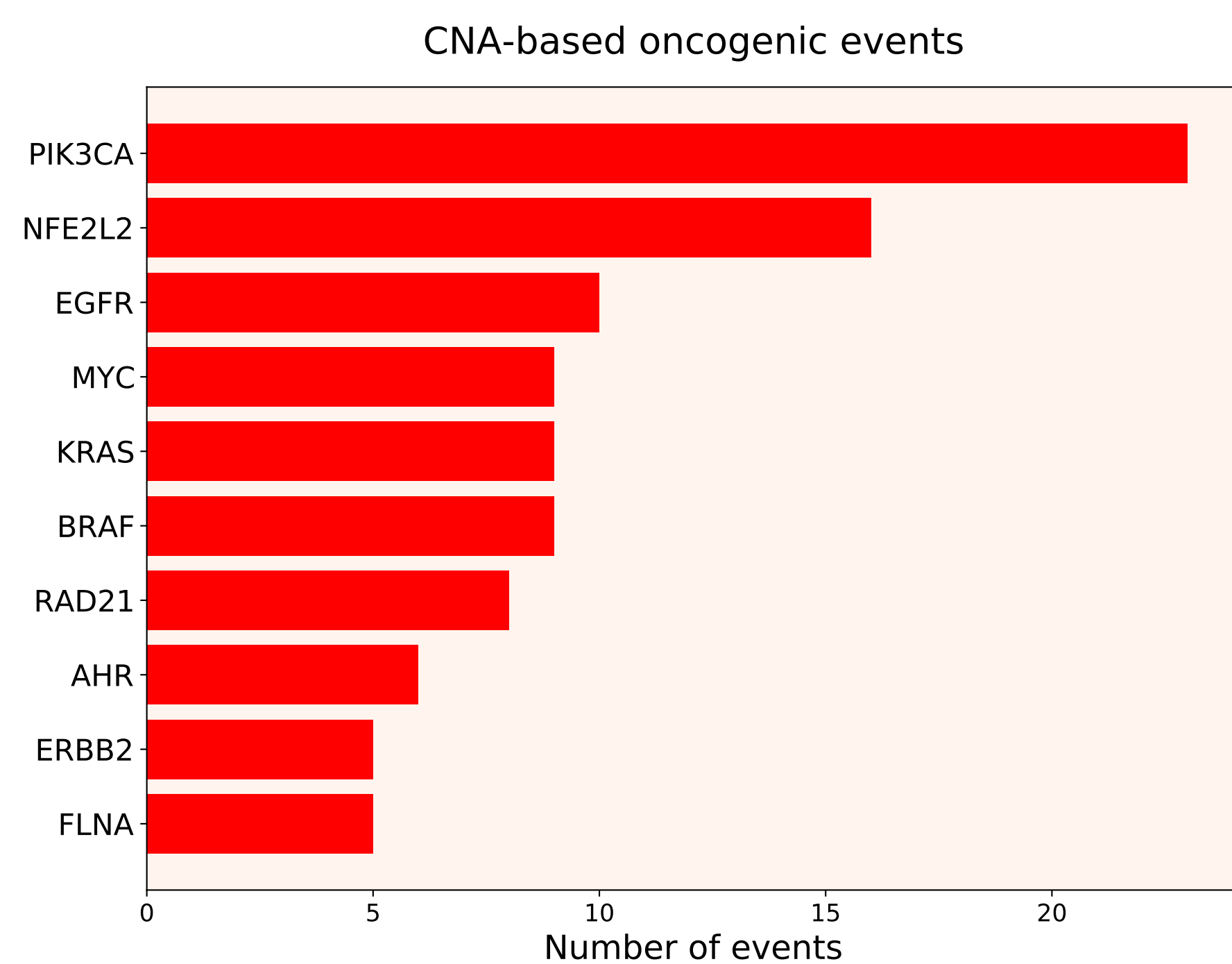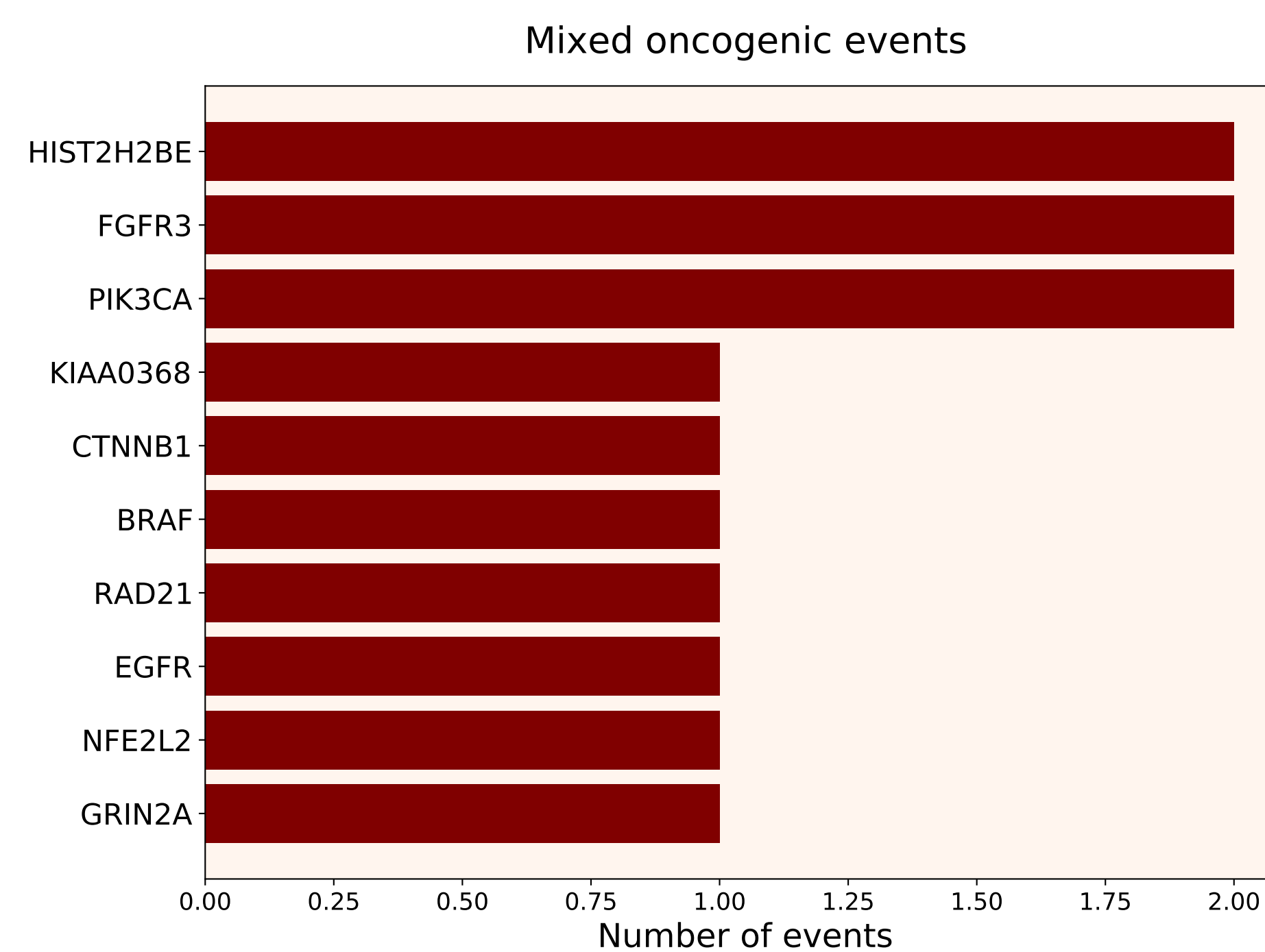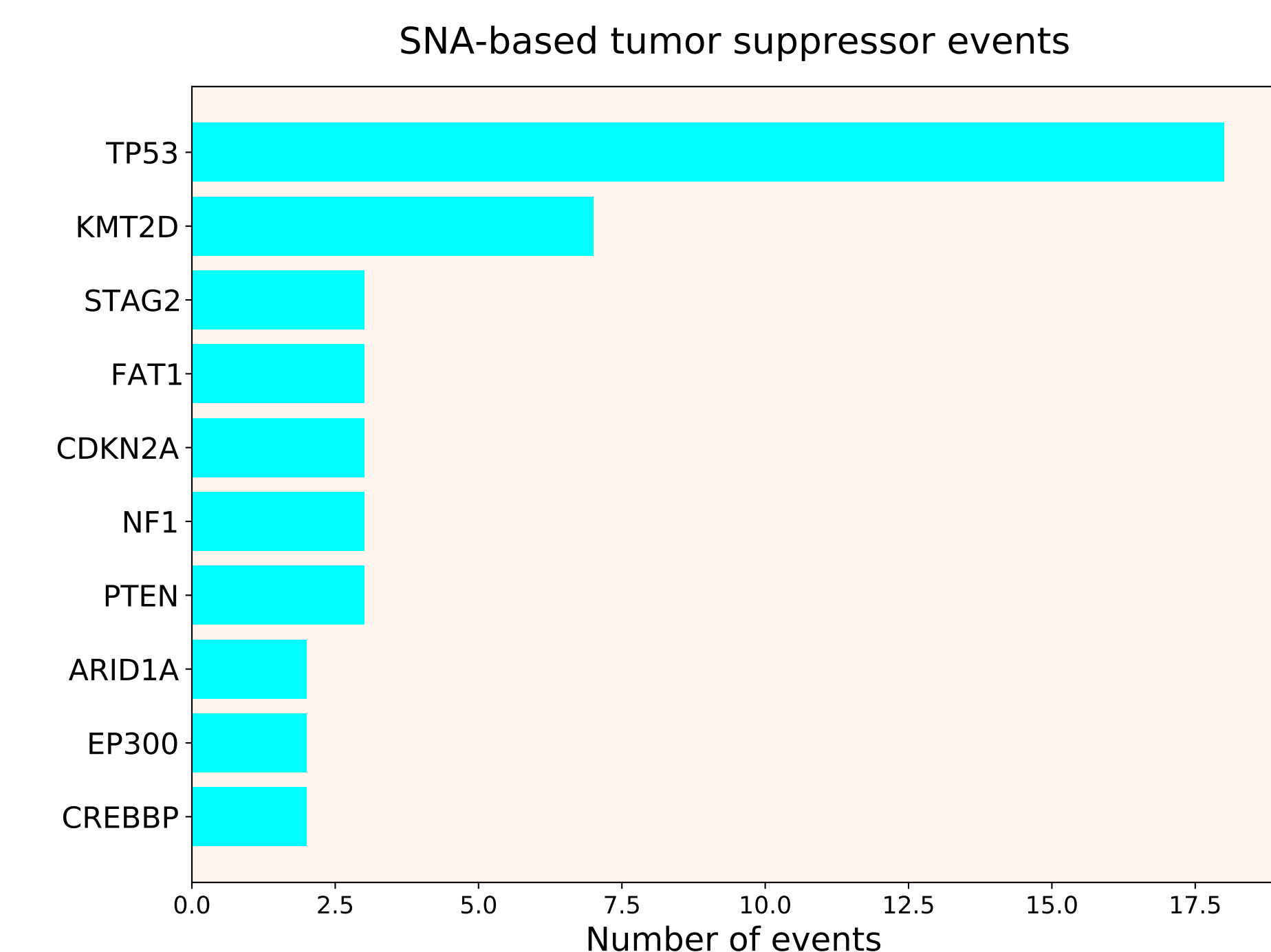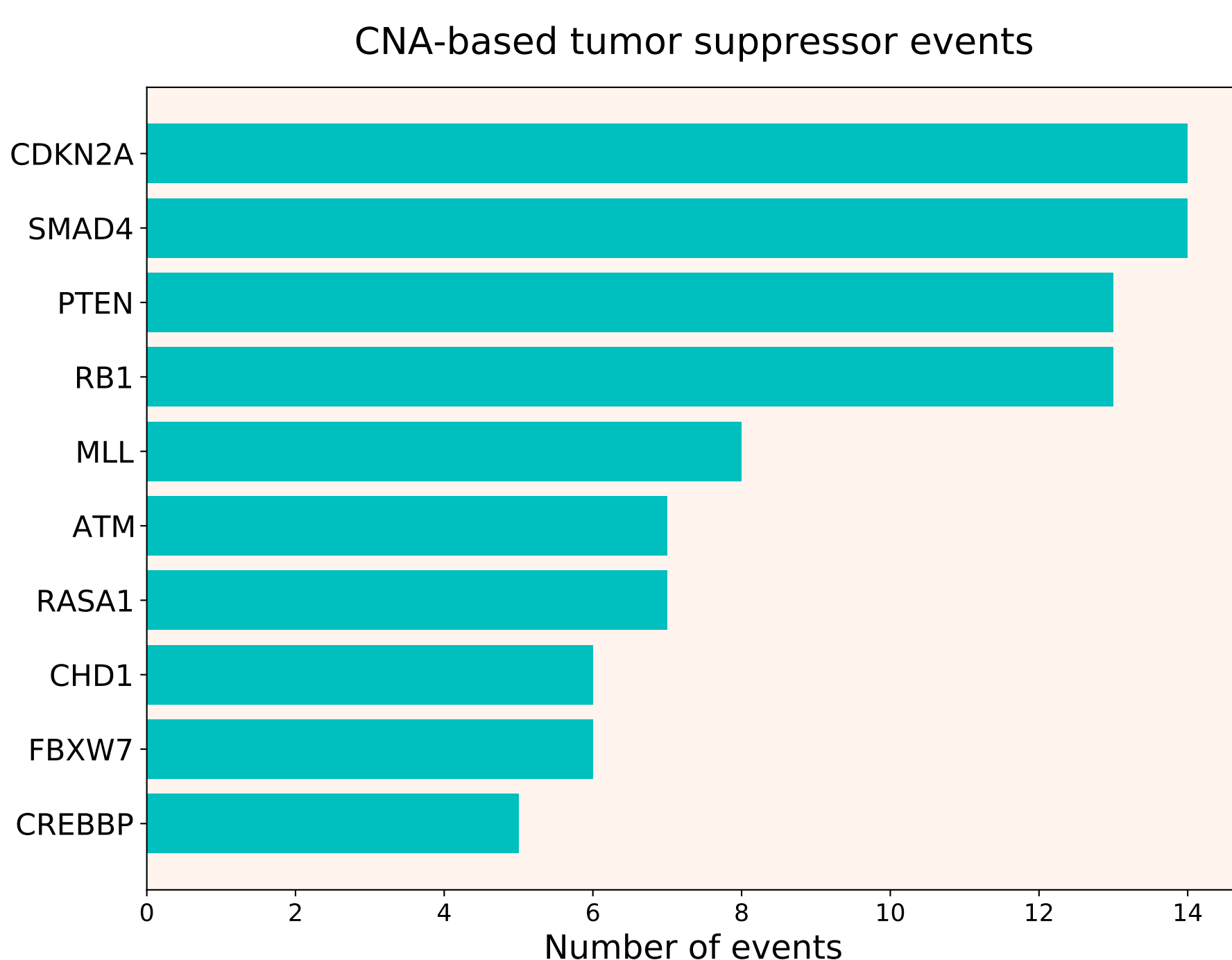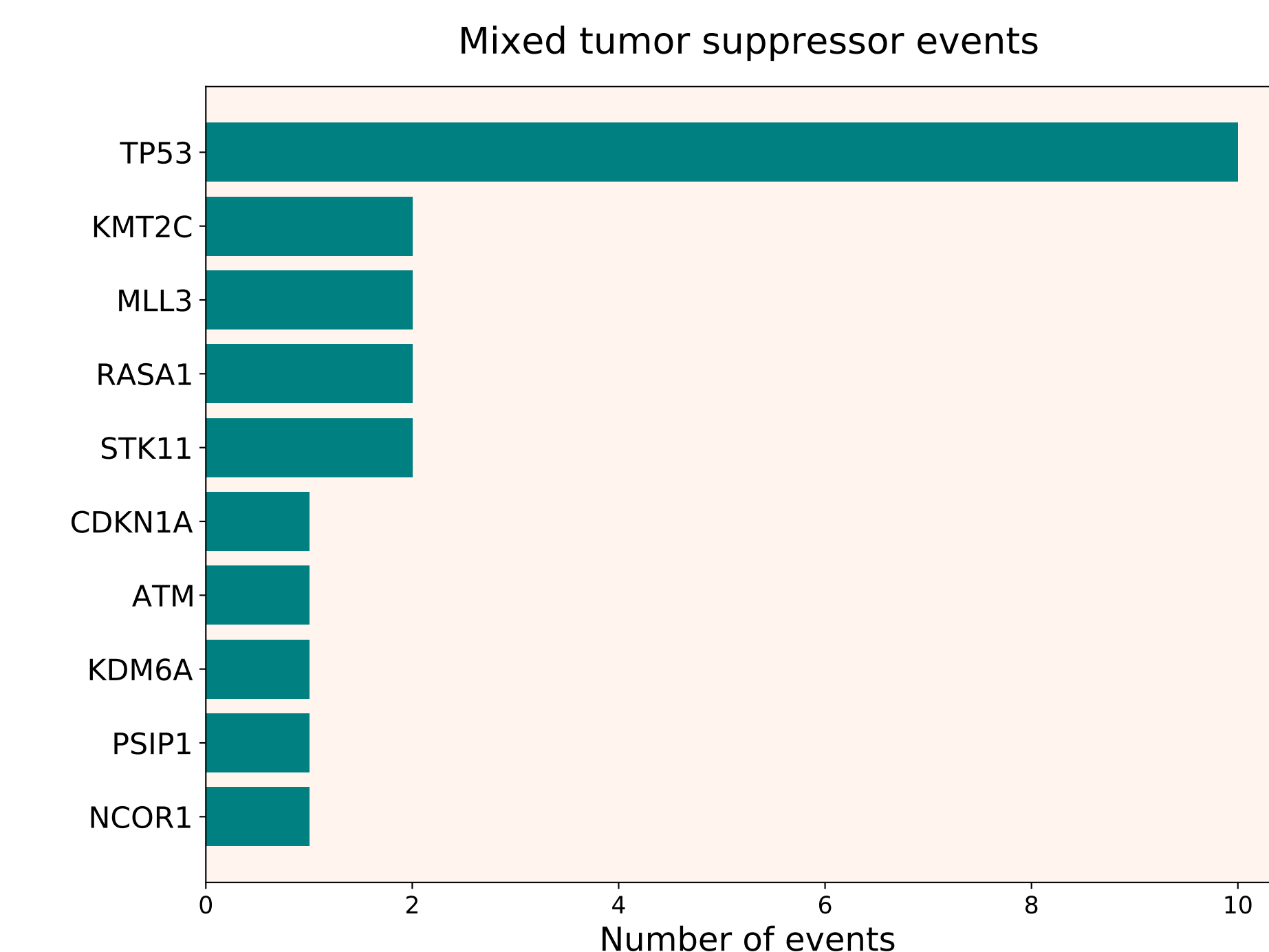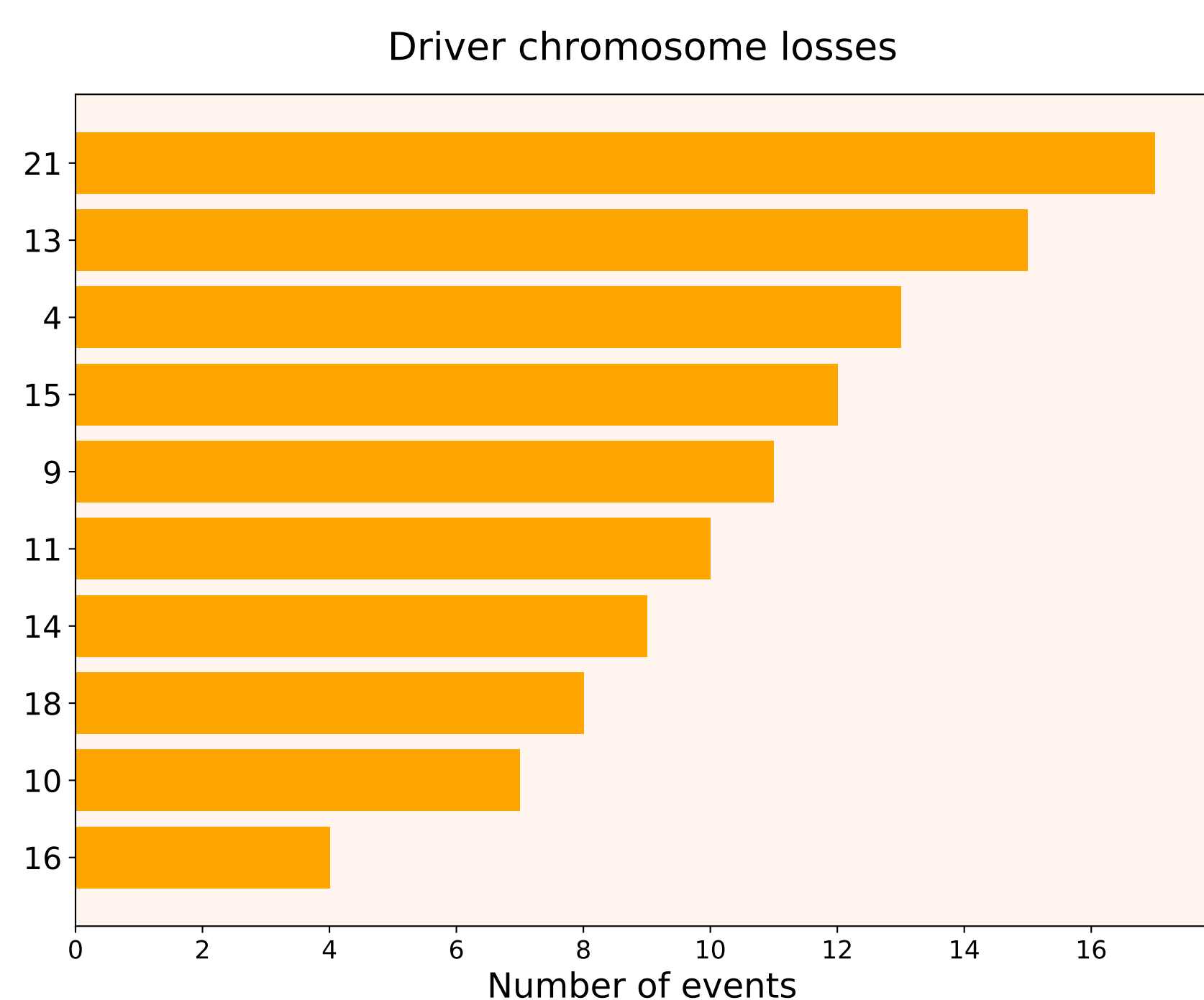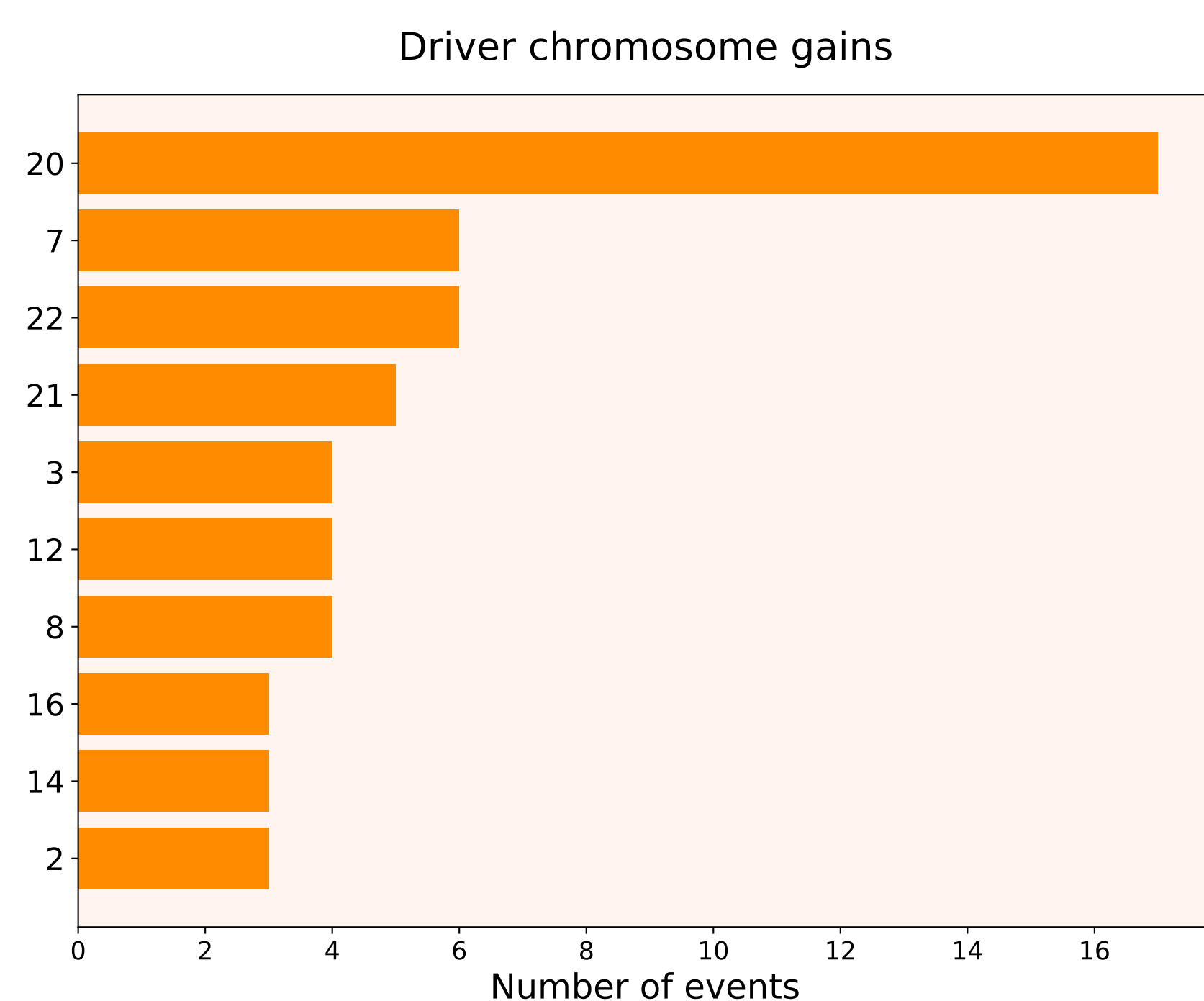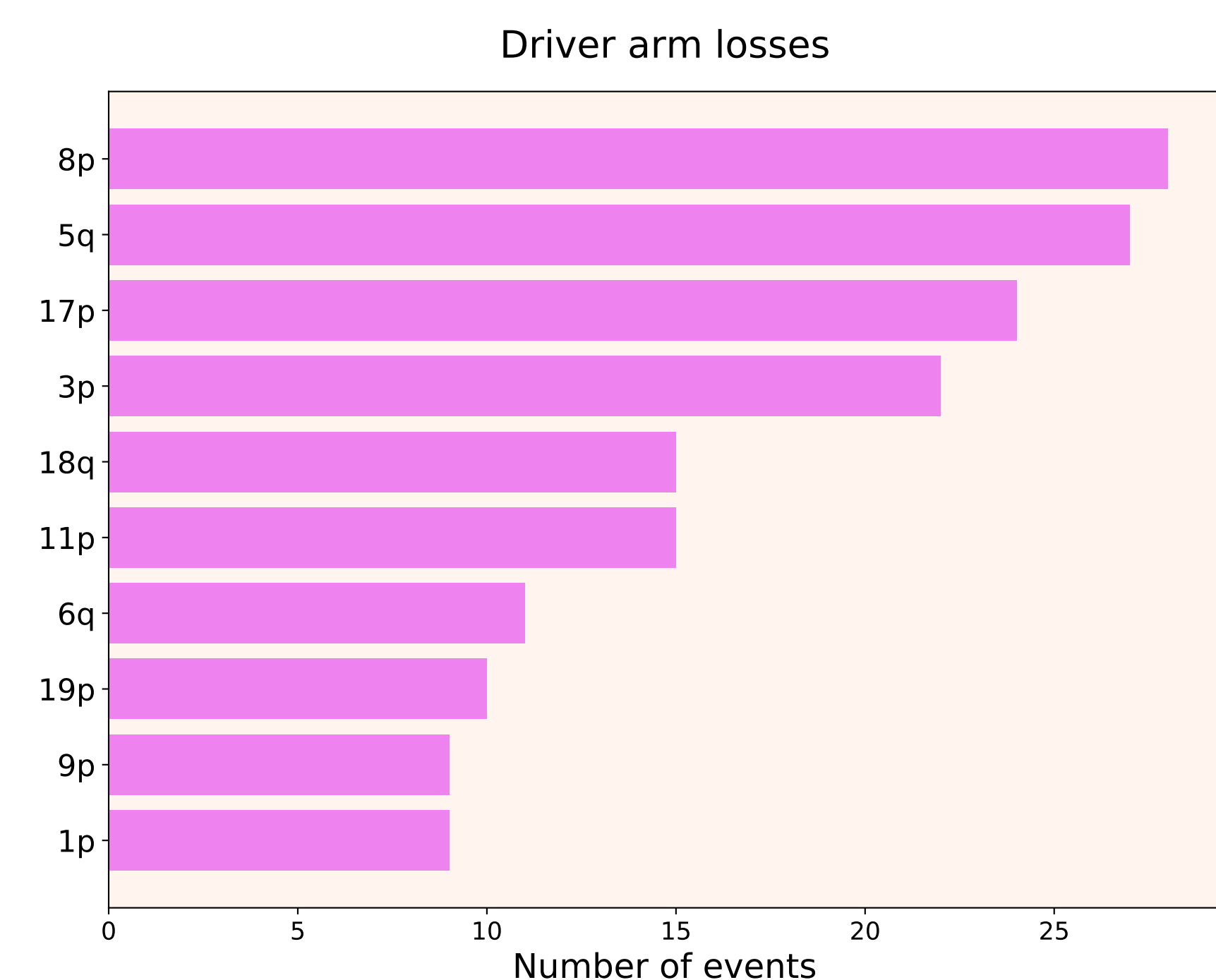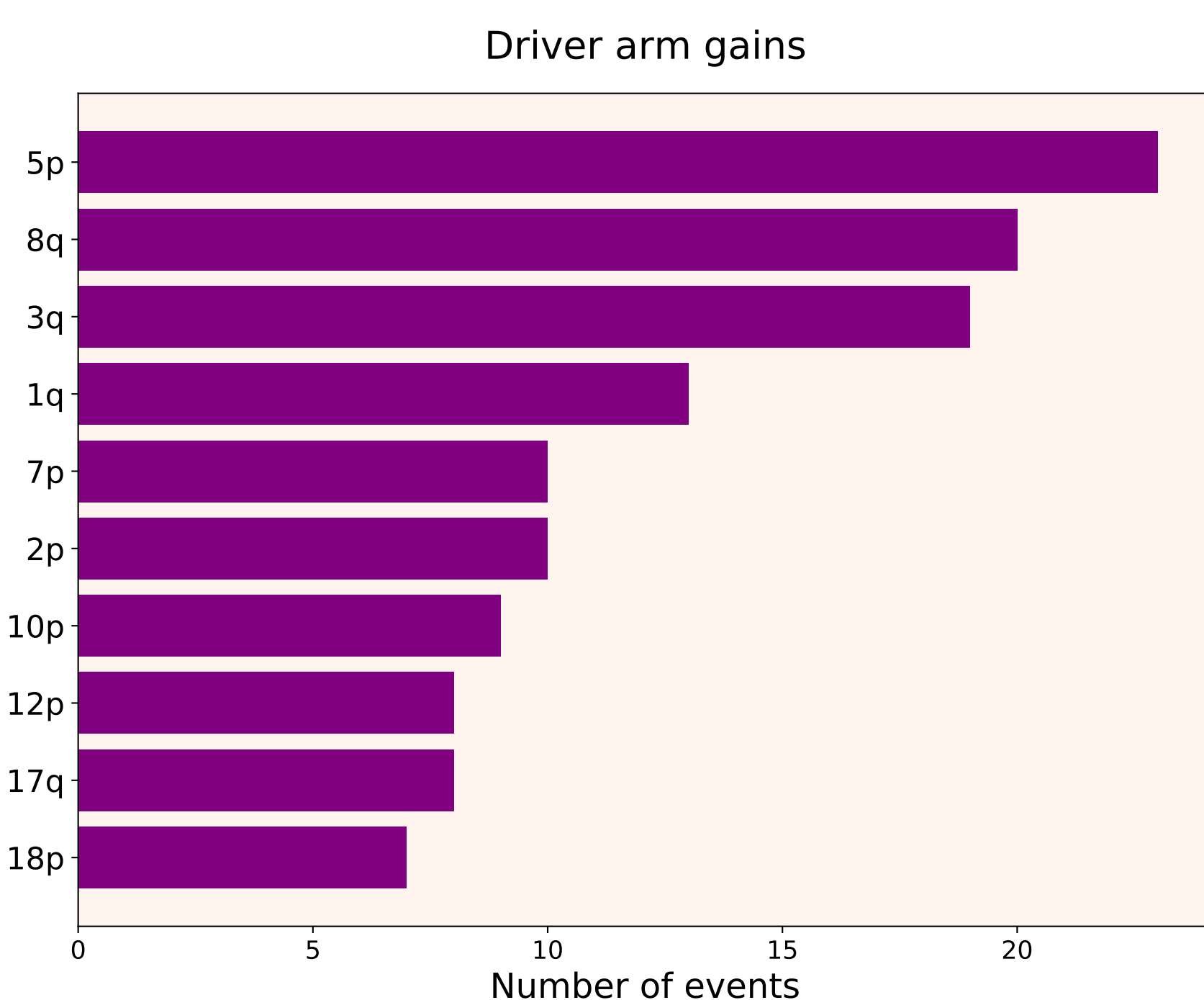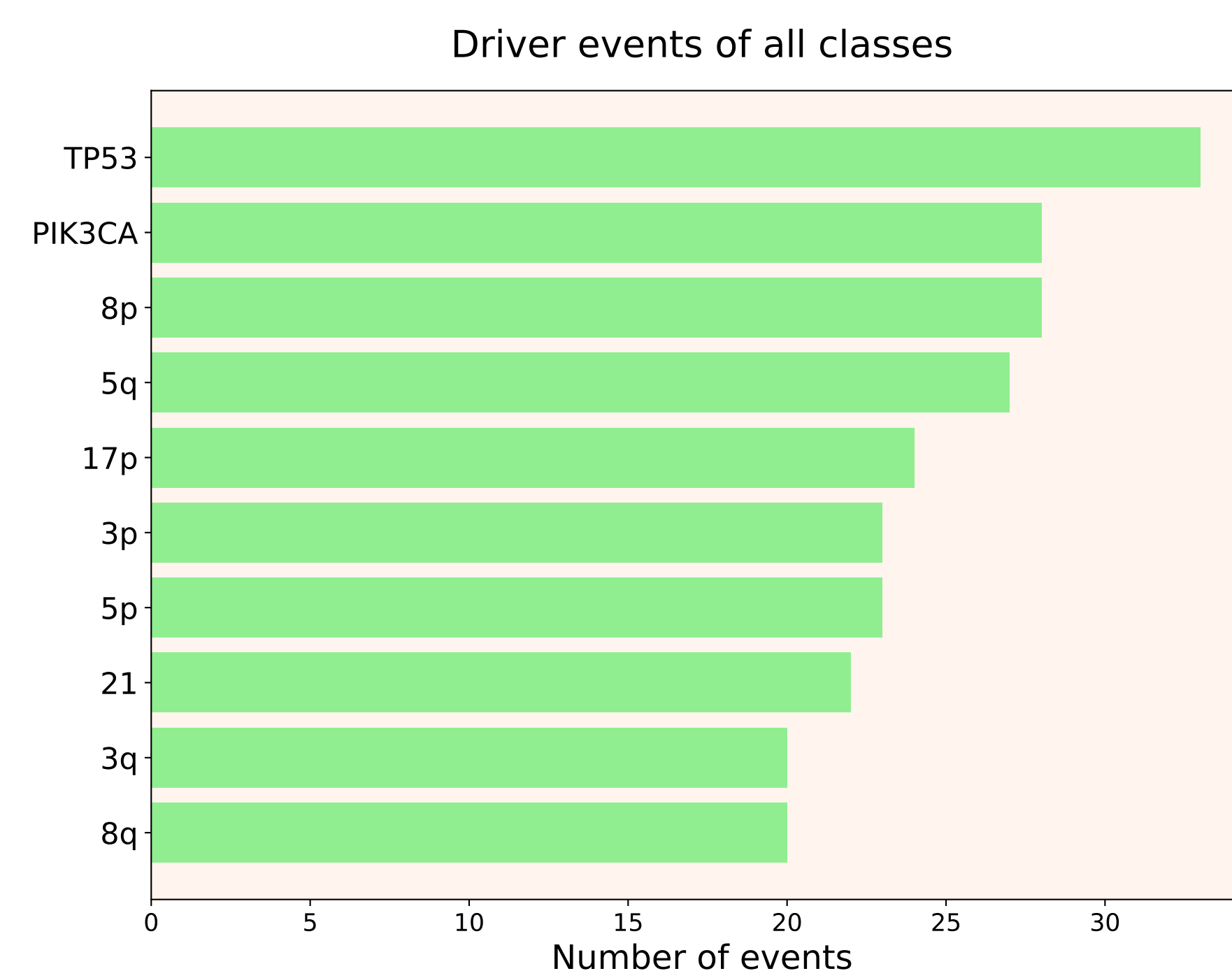

Supplement: Supplemental Information 2 [file peerj-10-13860-s002.zip › COHORTS/genes plots/detailed/2021_8_16_14_9_distribution_events_detailed_males_26.pdf]

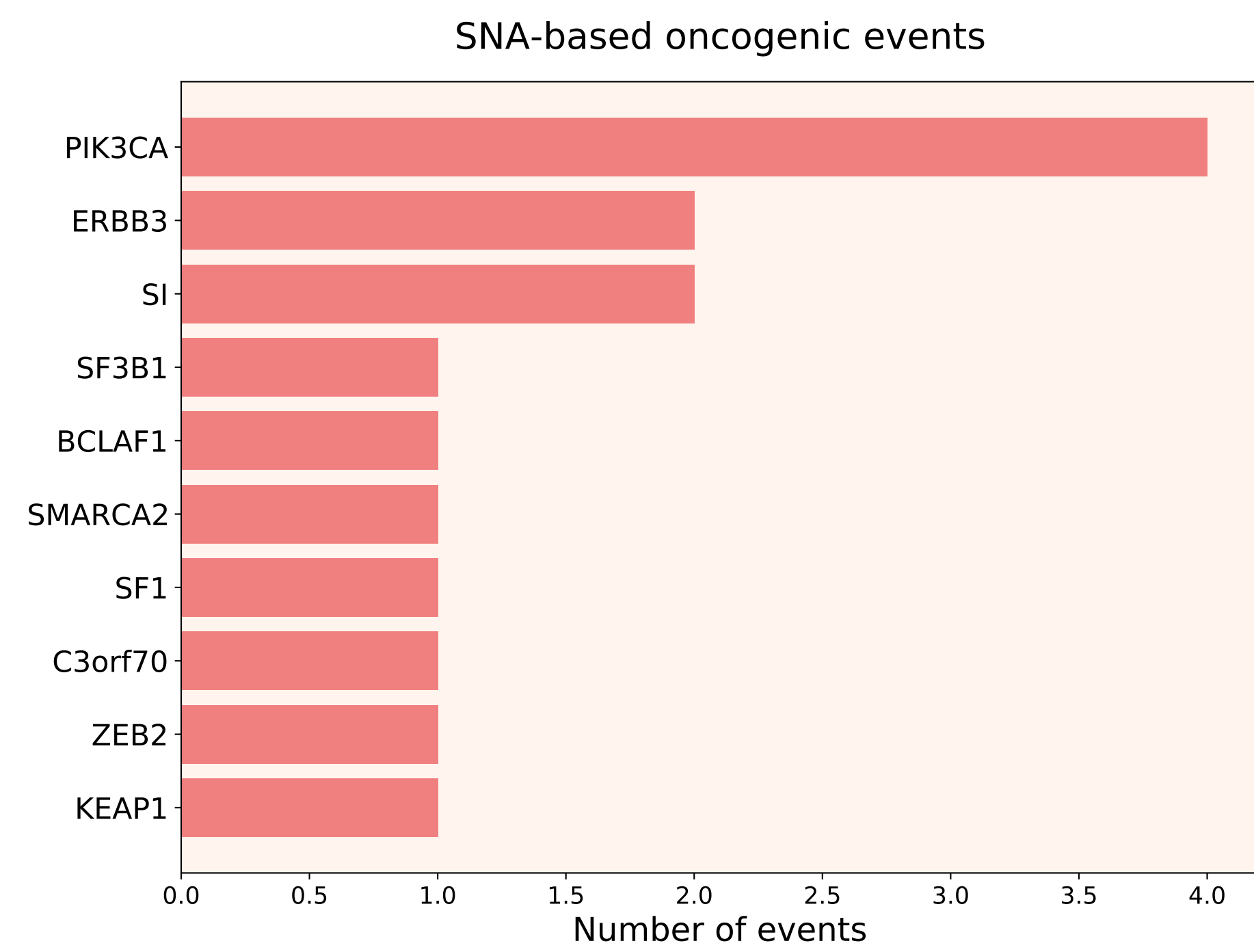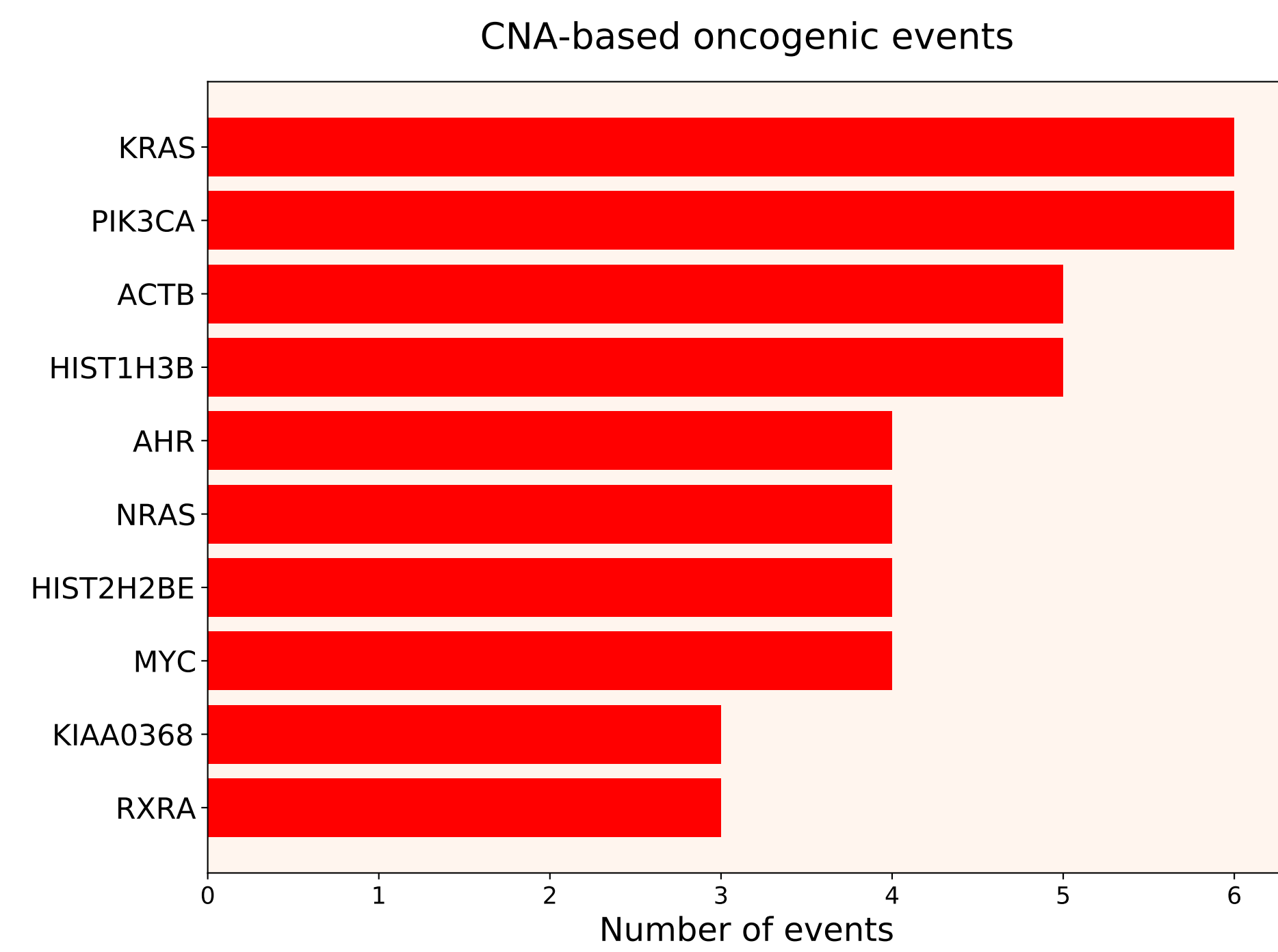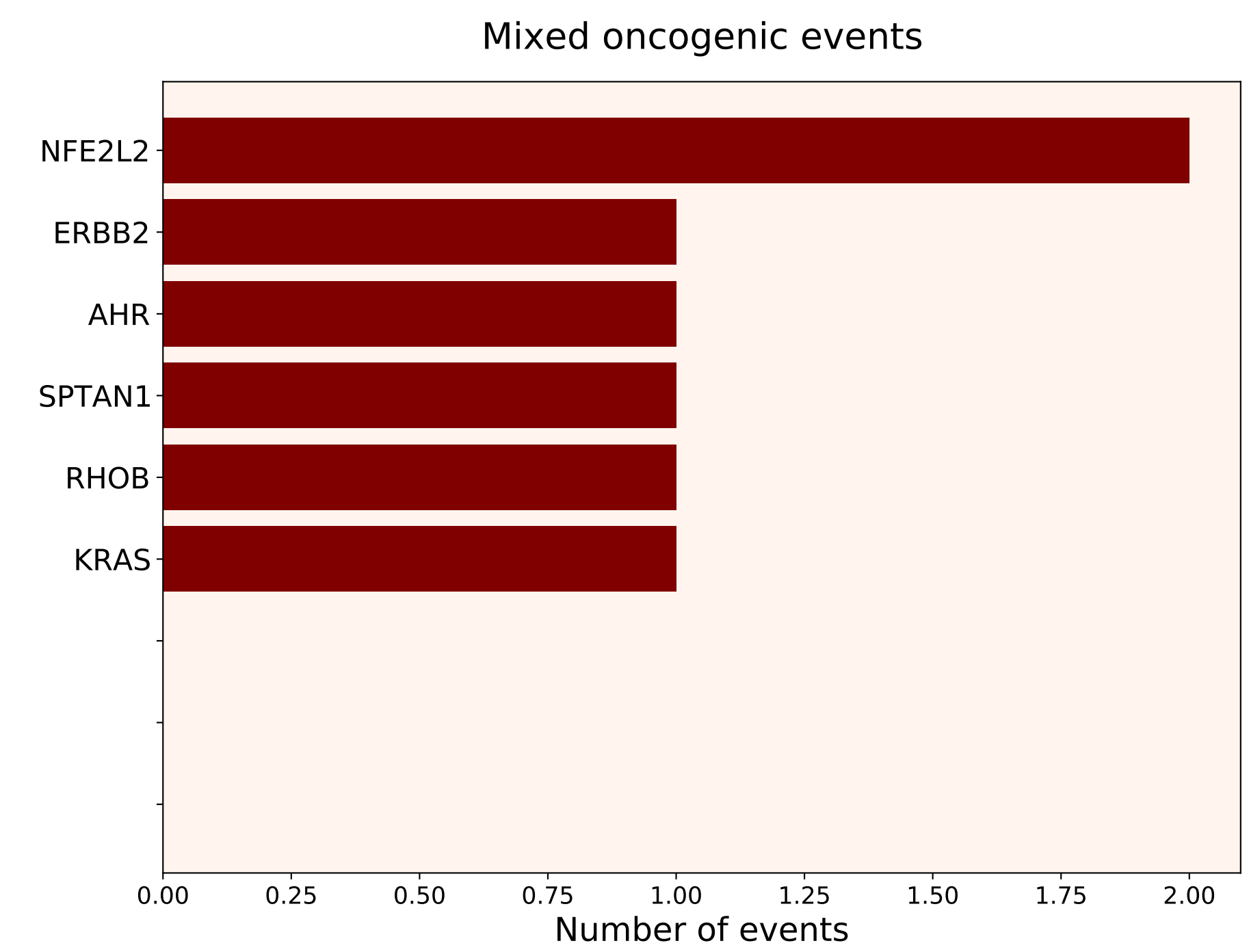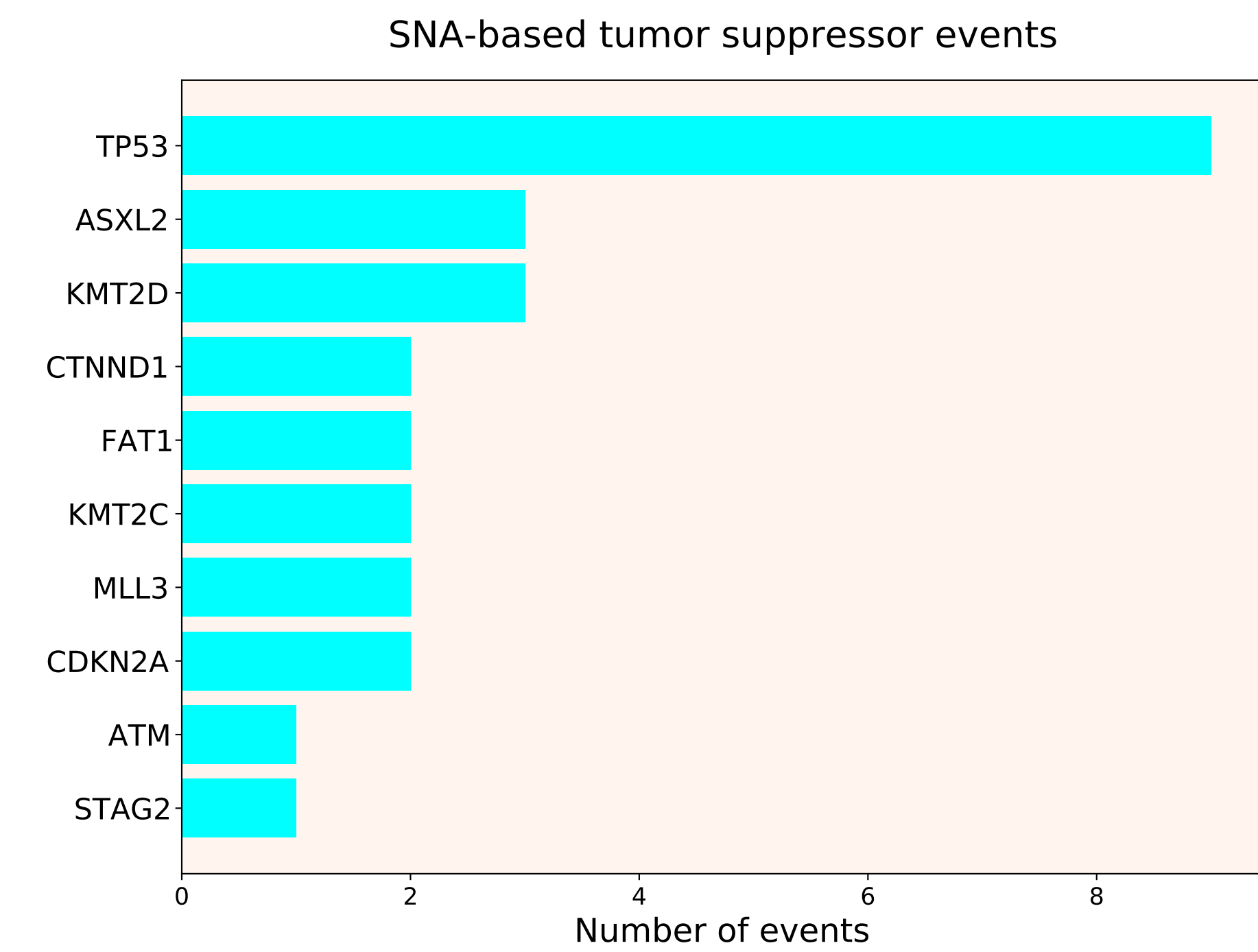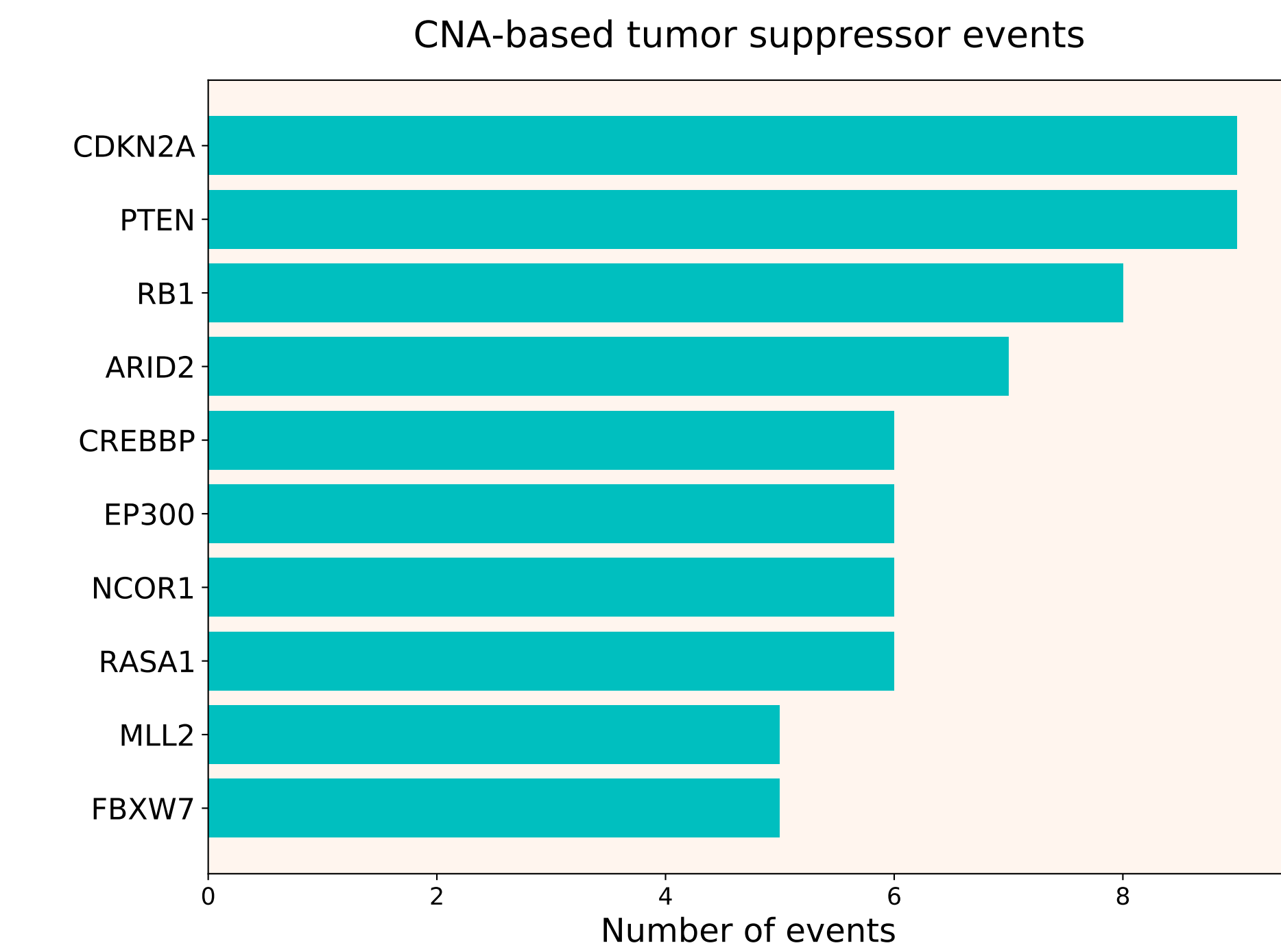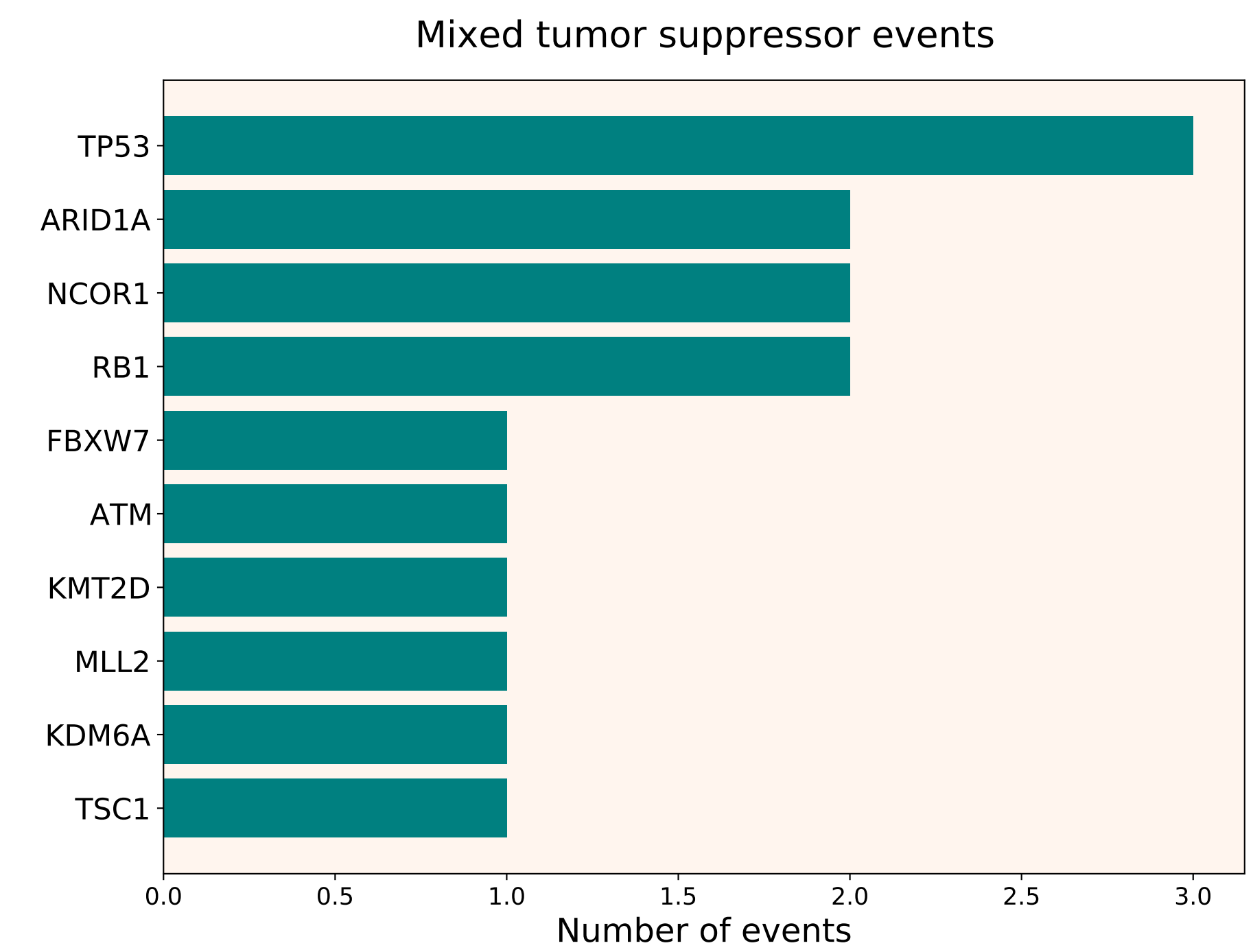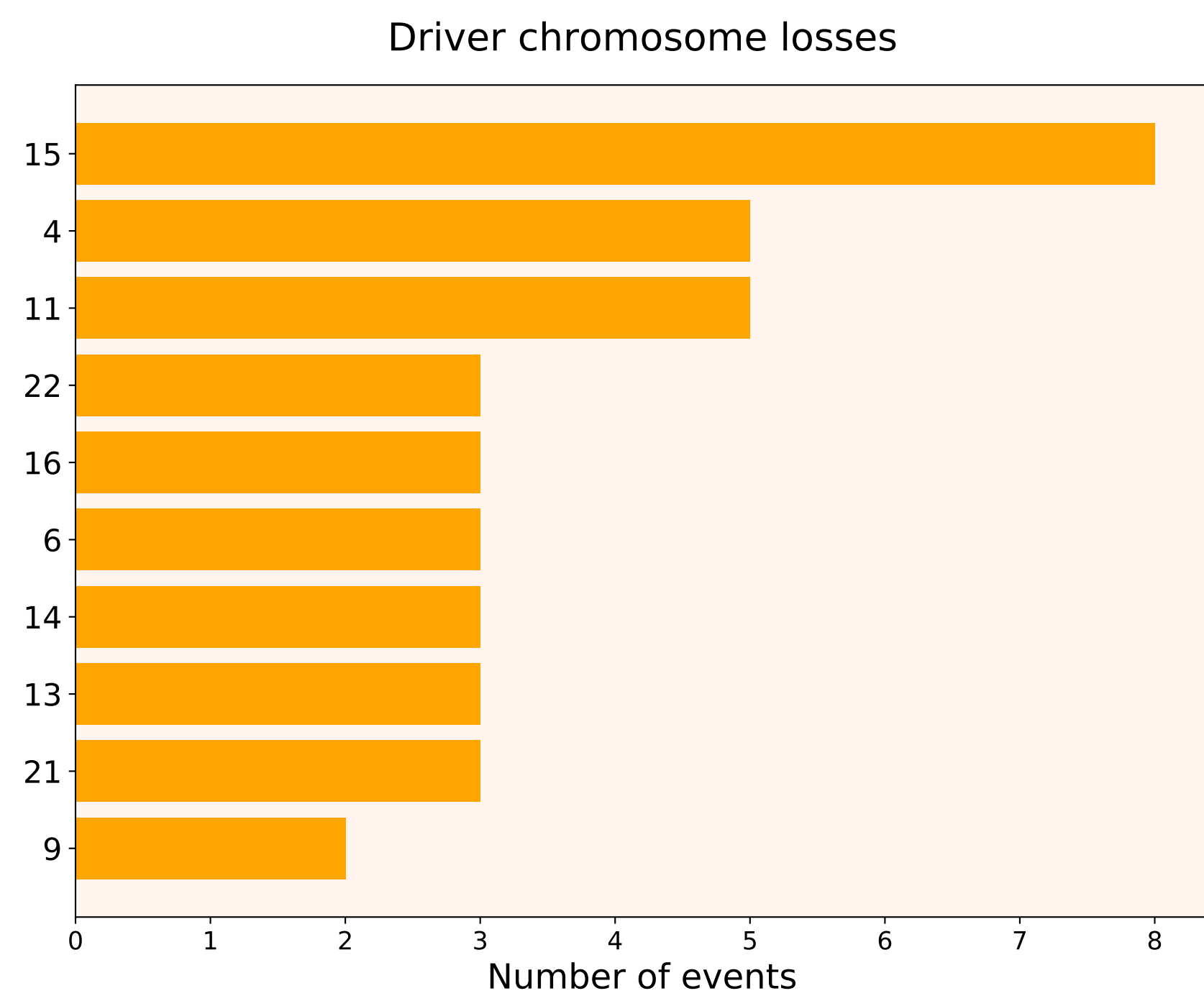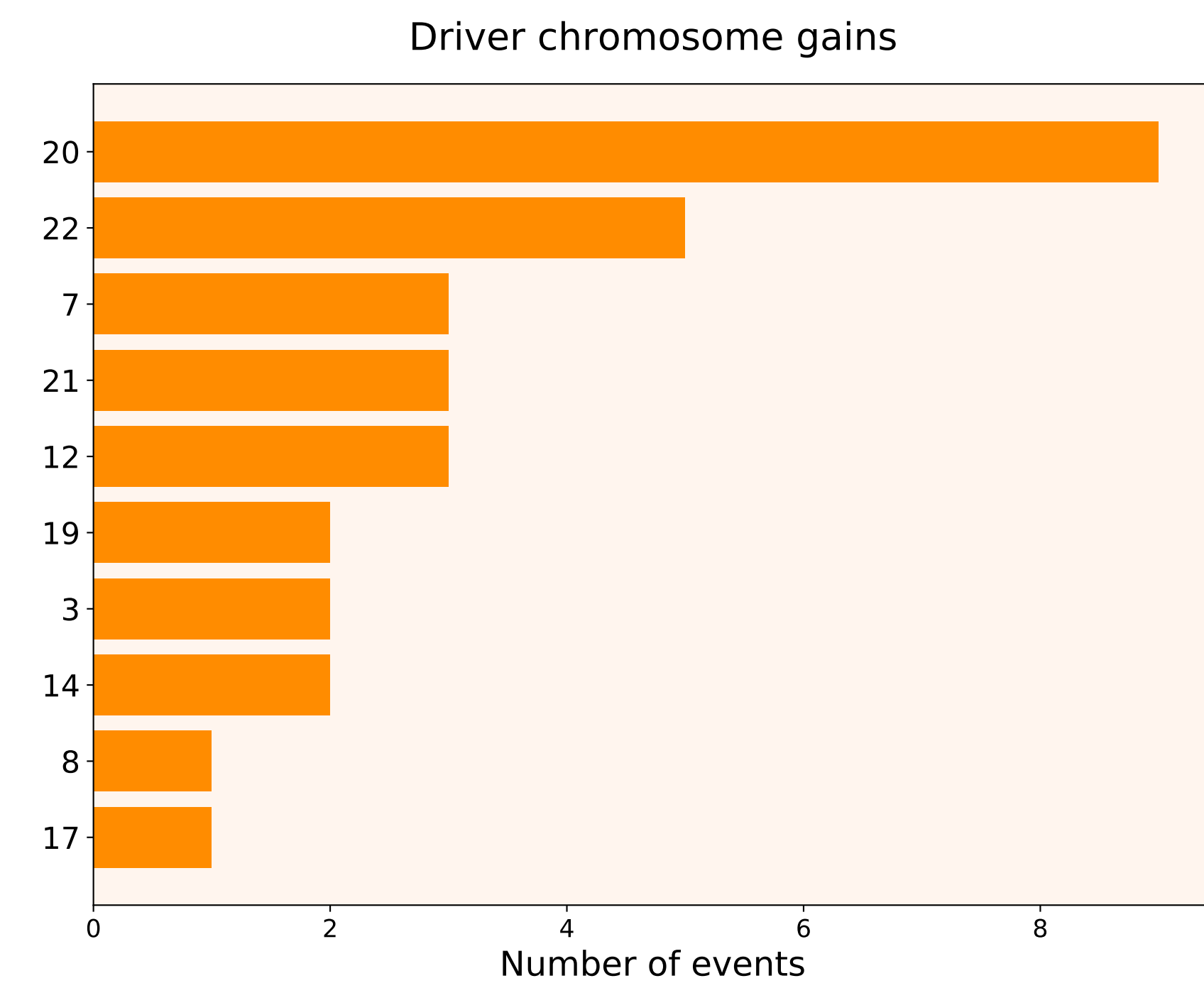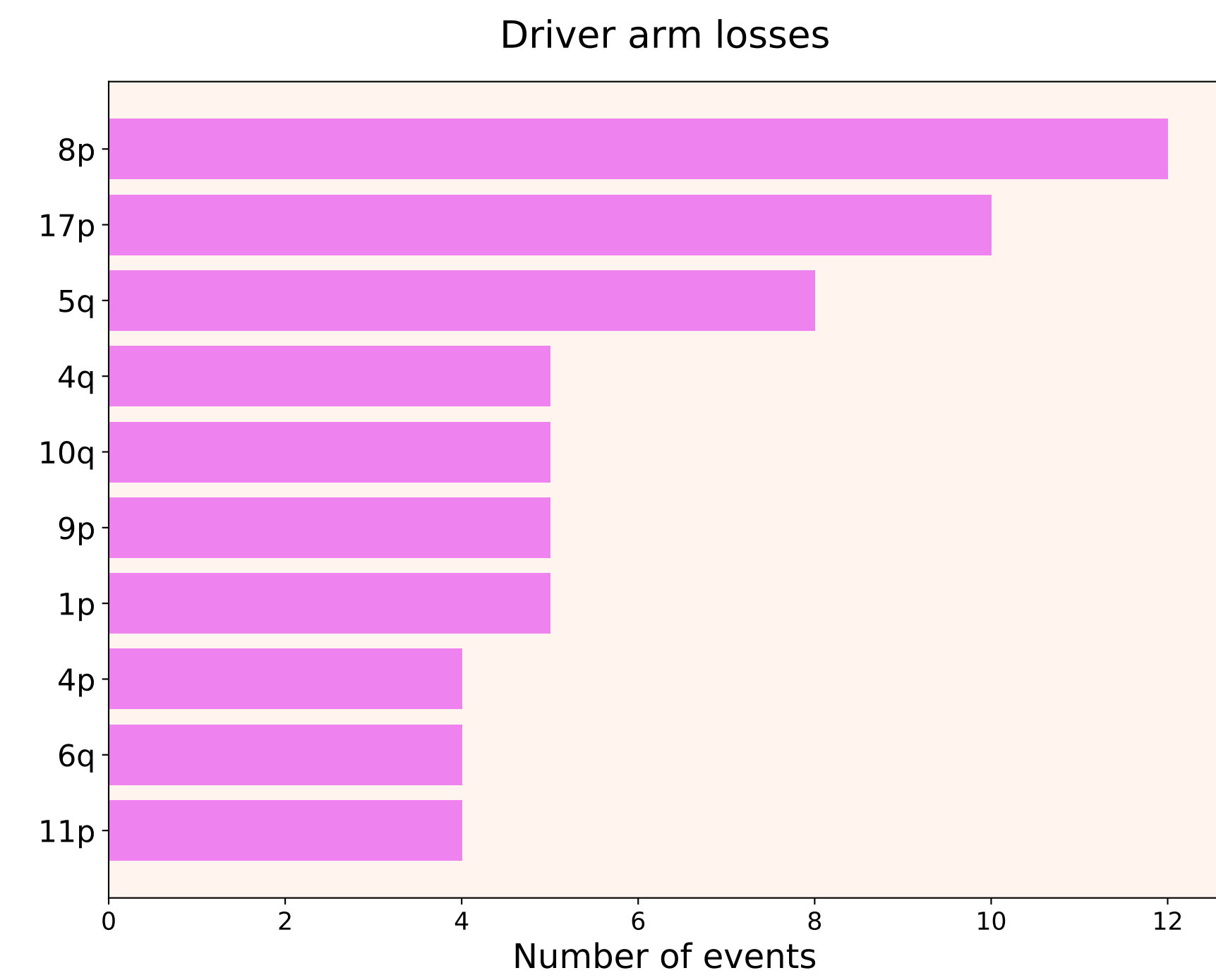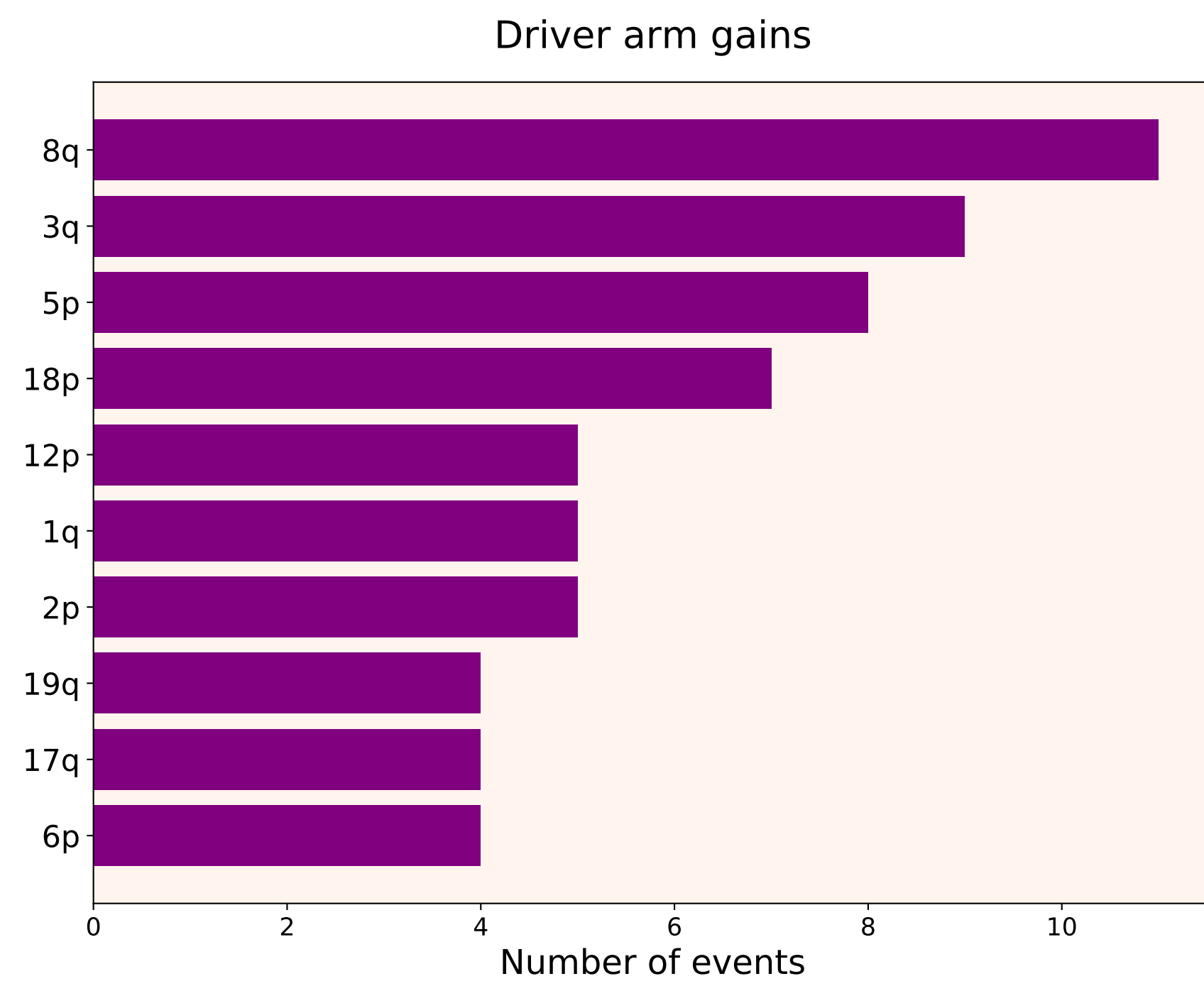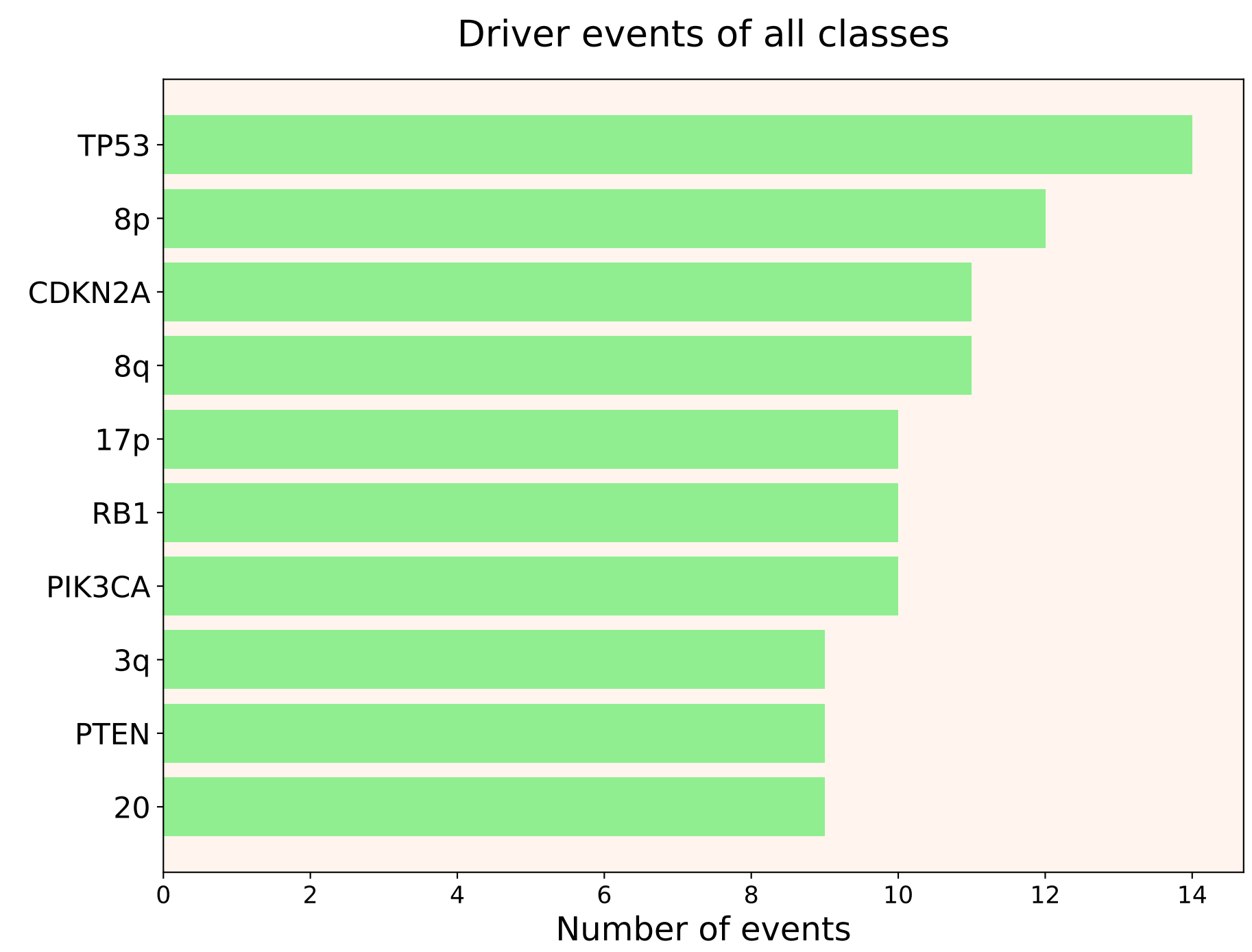

Supplement: Supplemental Information 2 [file peerj-10-13860-s002.zip › COHORTS/genes plots/detailed/2021_8_16_14_9_distribution_events_detailed_males_32.pdf]

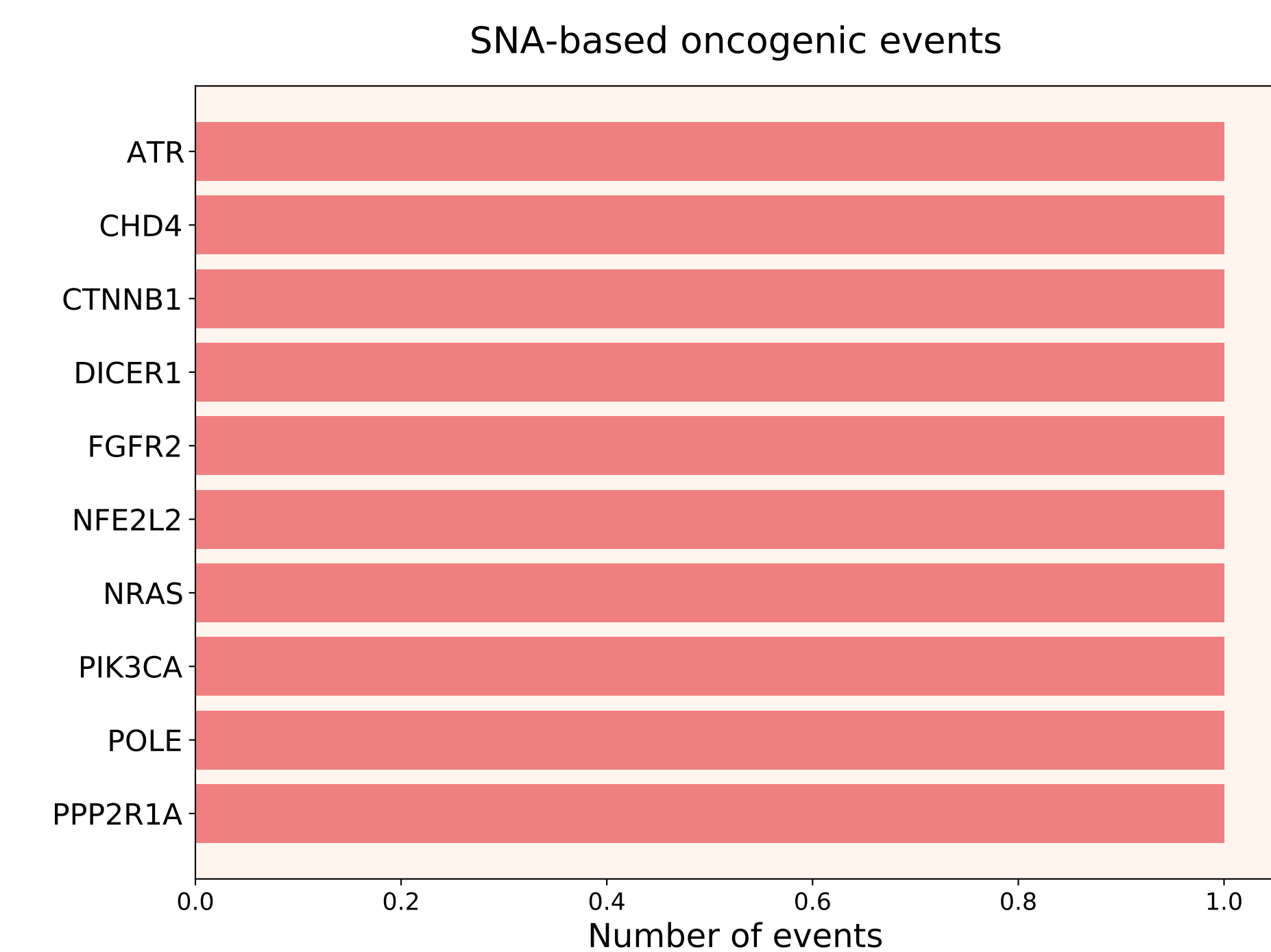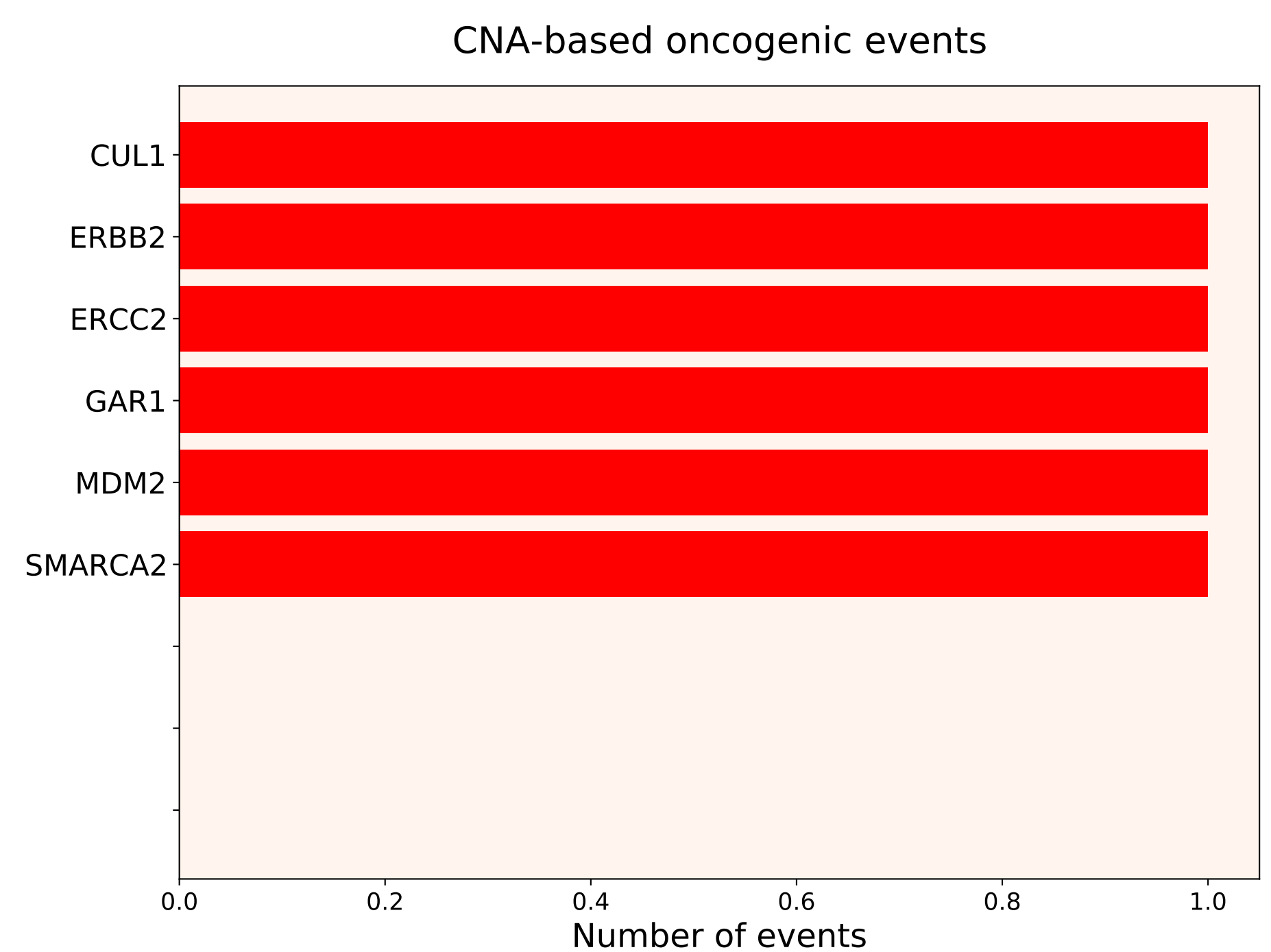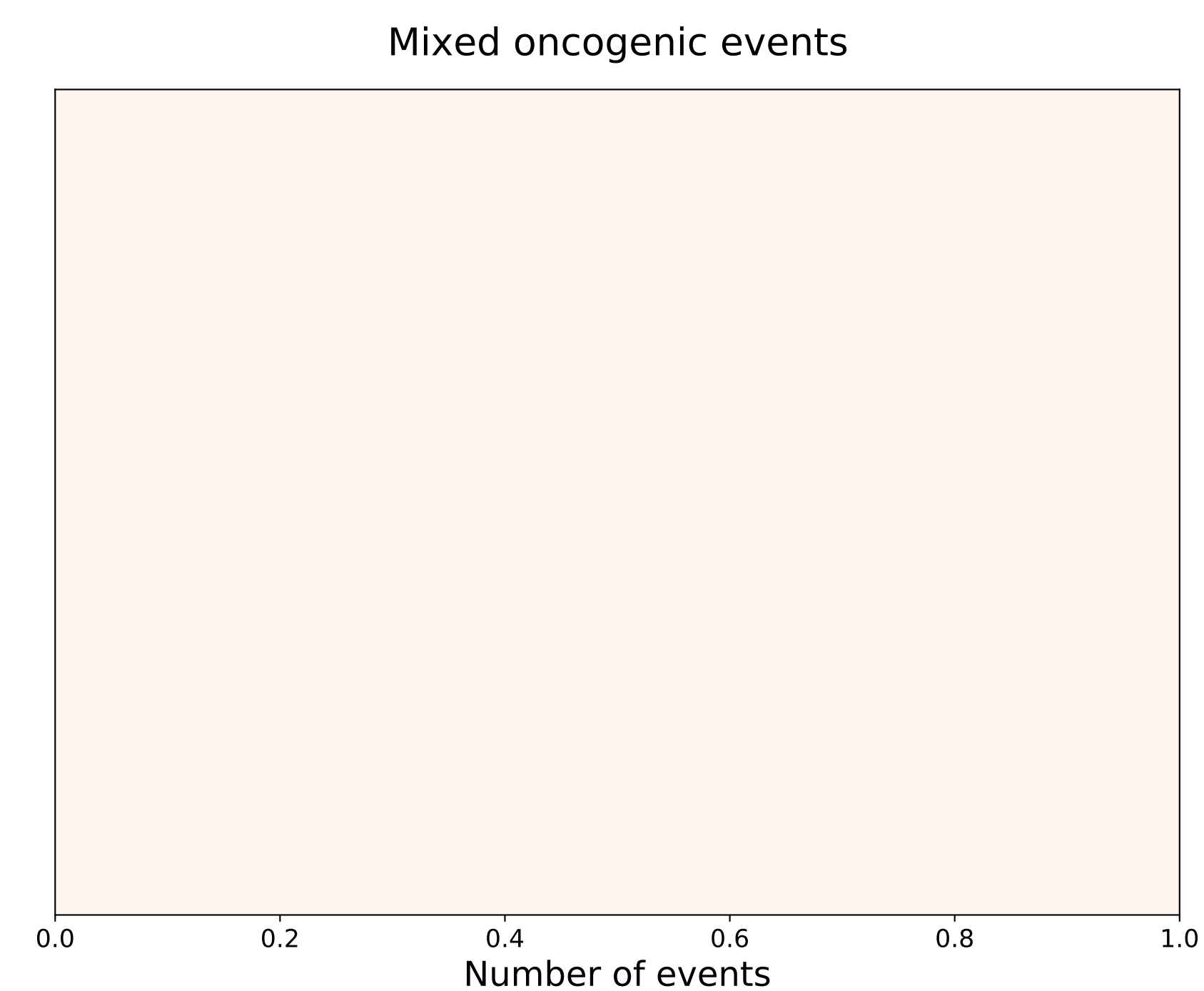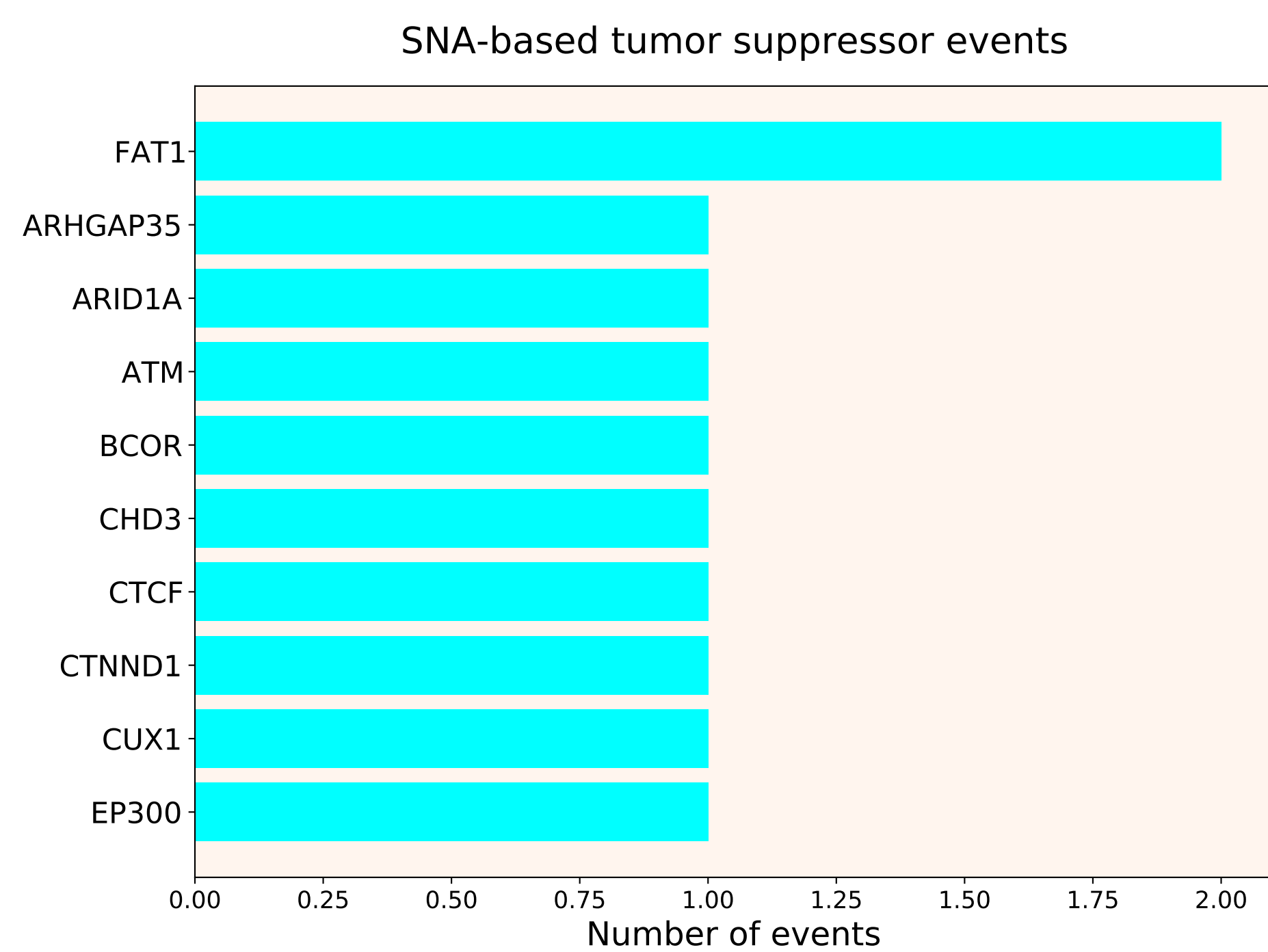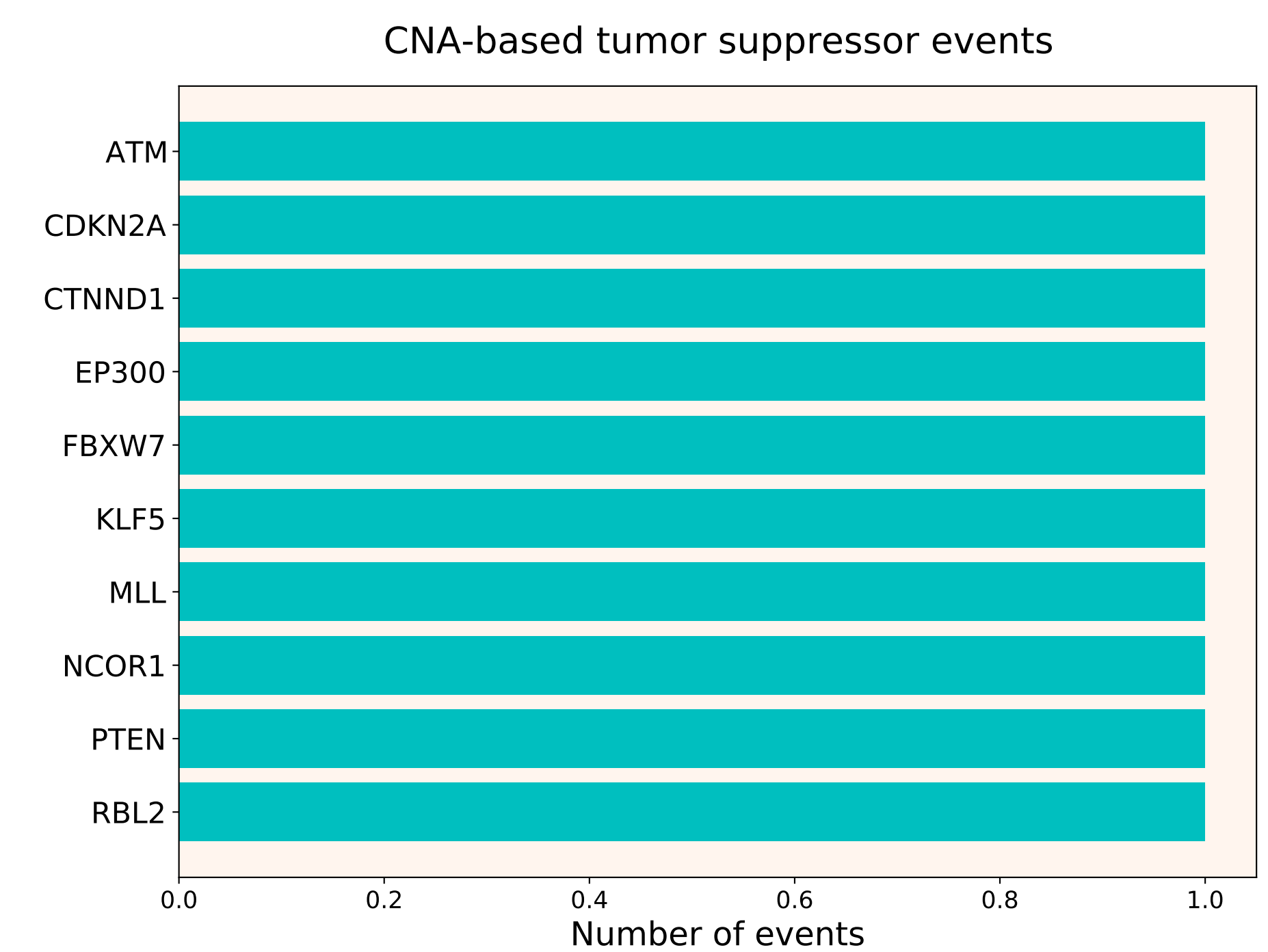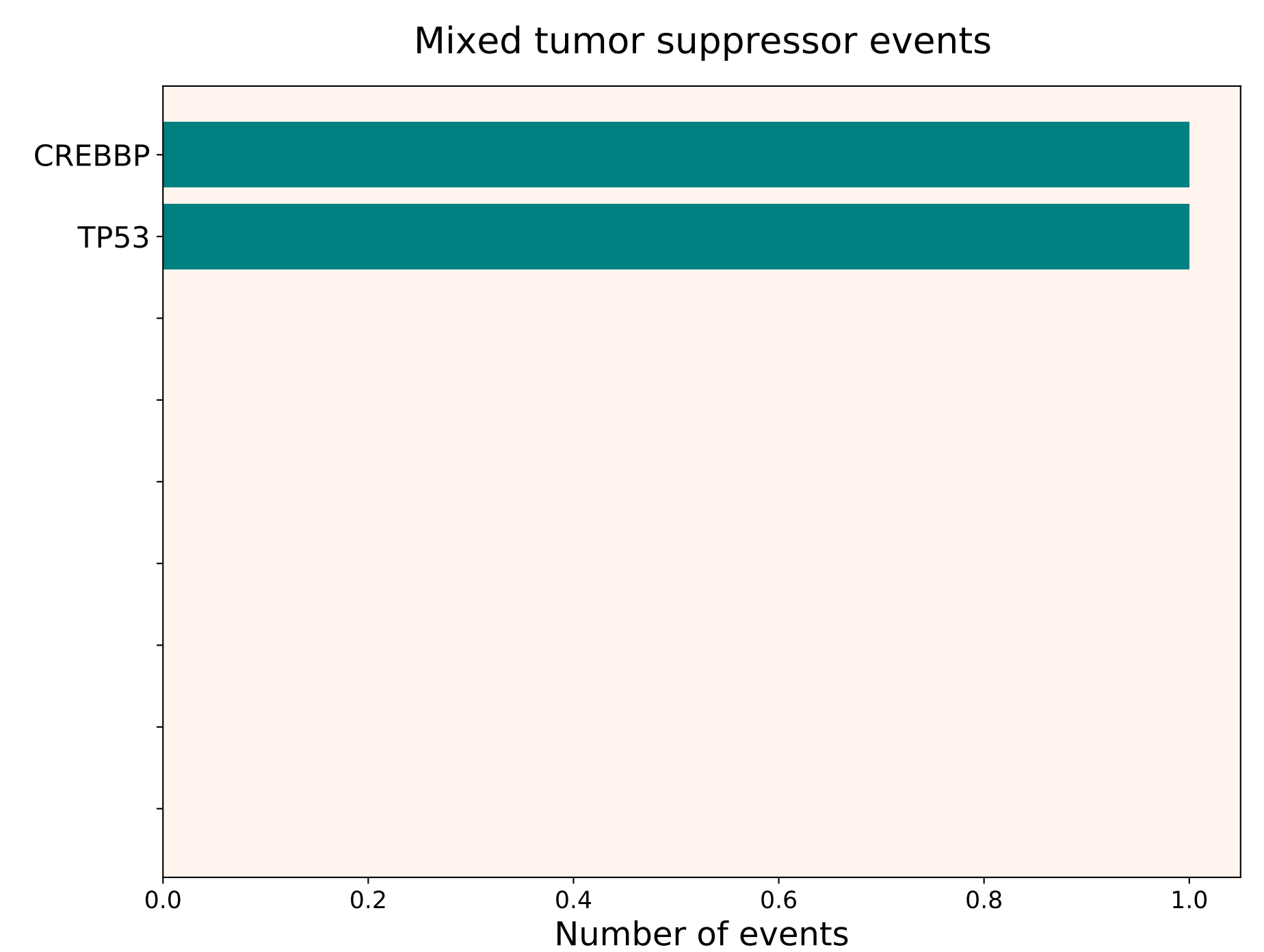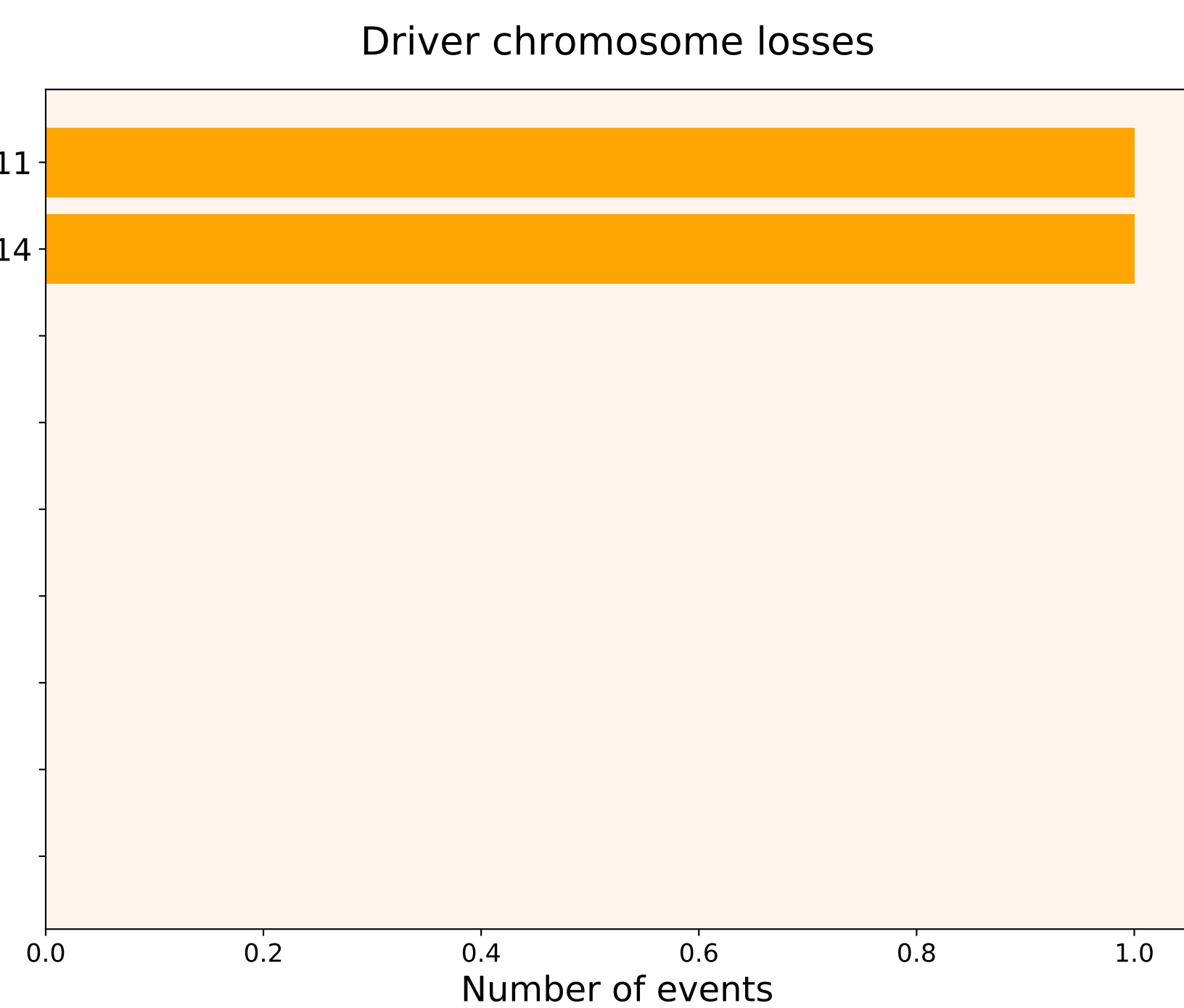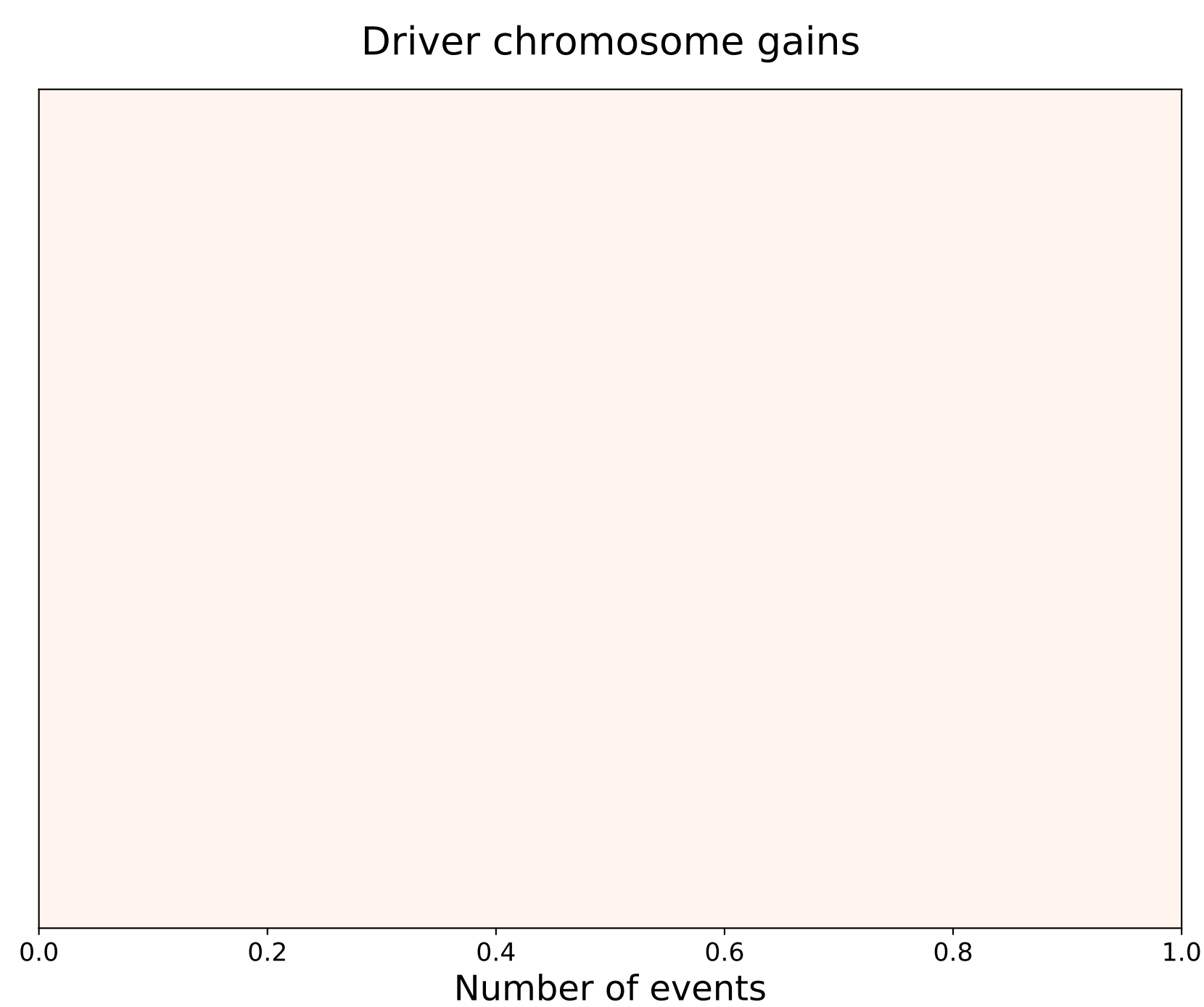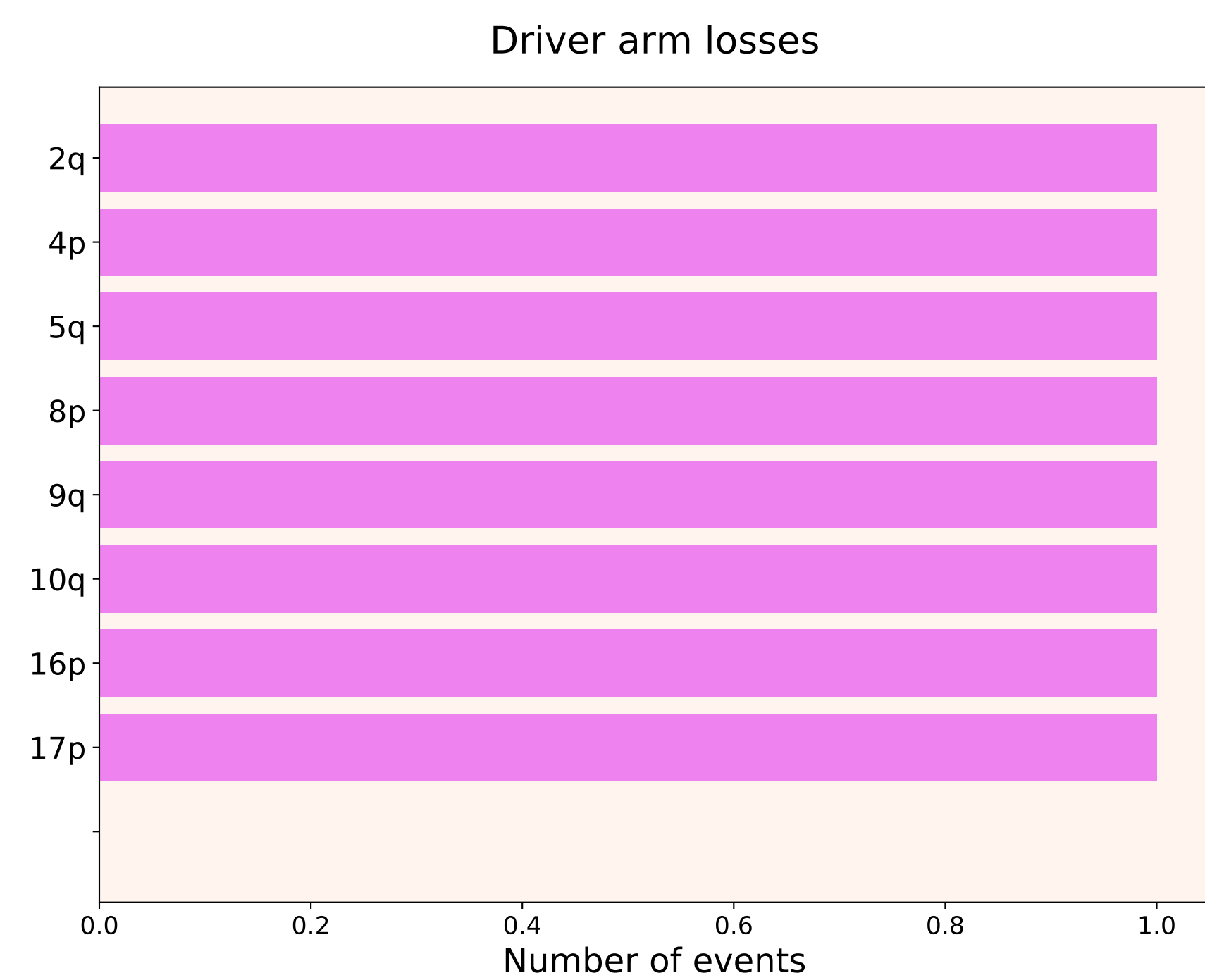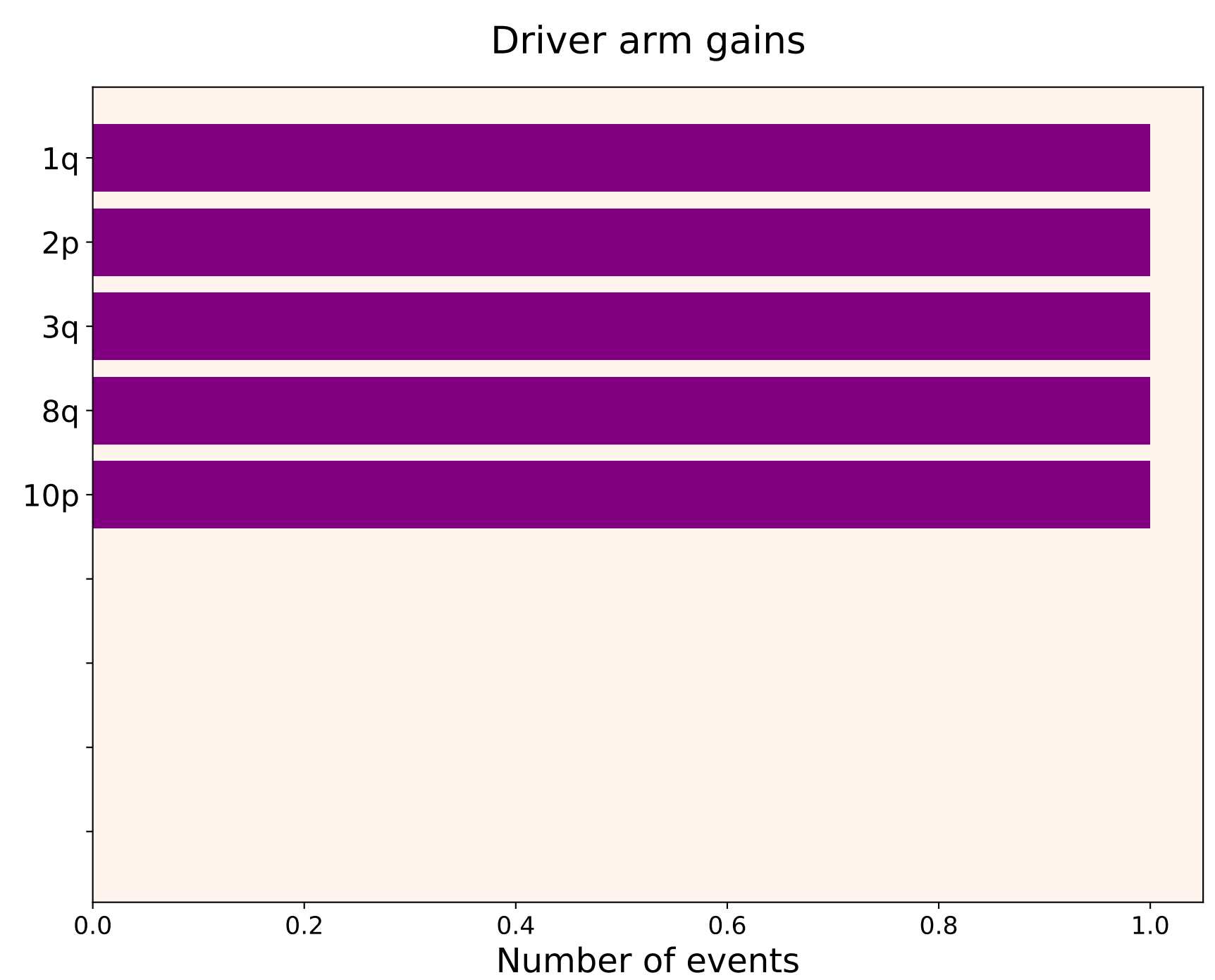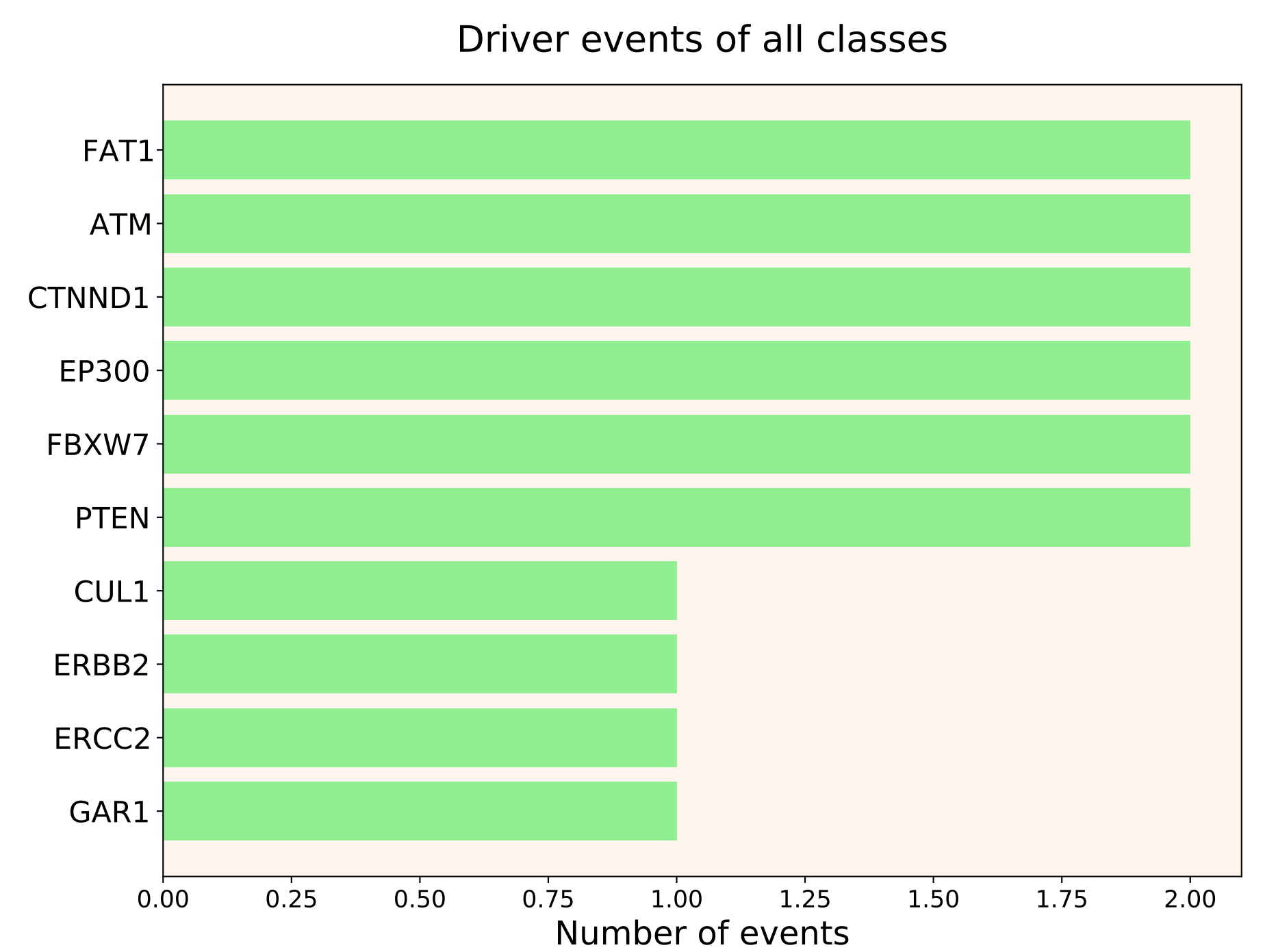

Supplement: Supplemental Information 2 [file peerj-10-13860-s002.zip › COHORTS/genes plots/detailed/2021_8_16_14_9_distribution_events_detailed_females_36.pdf]

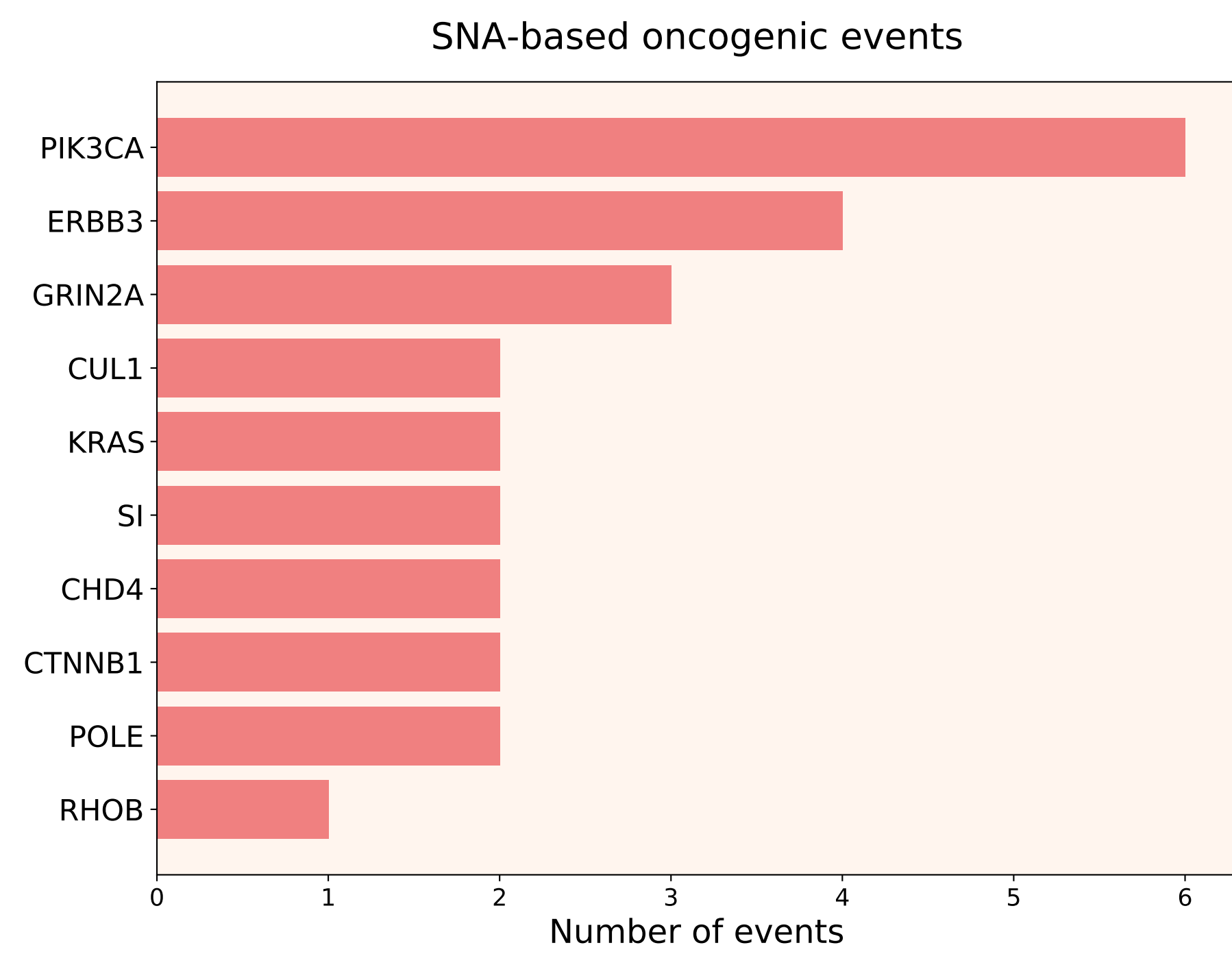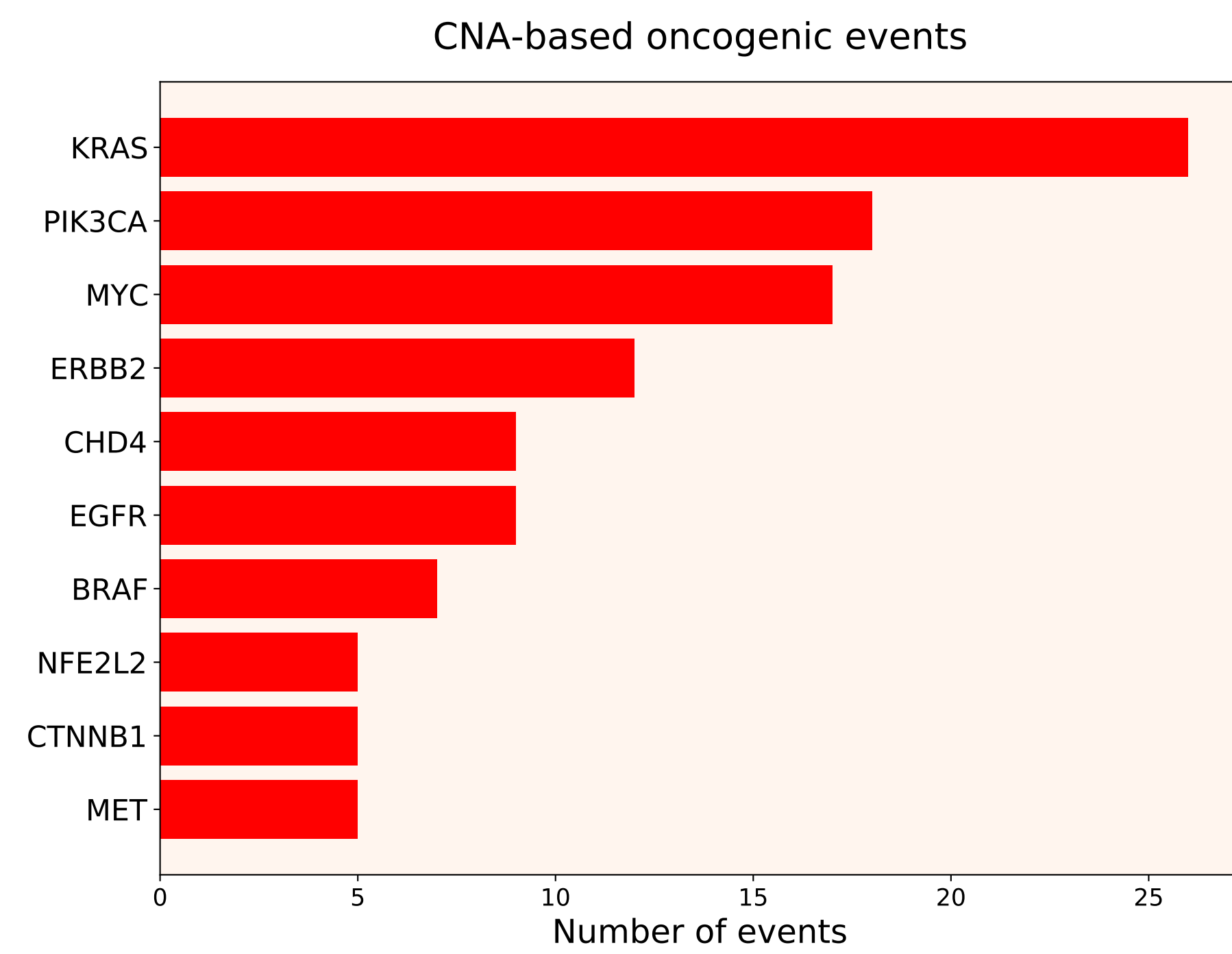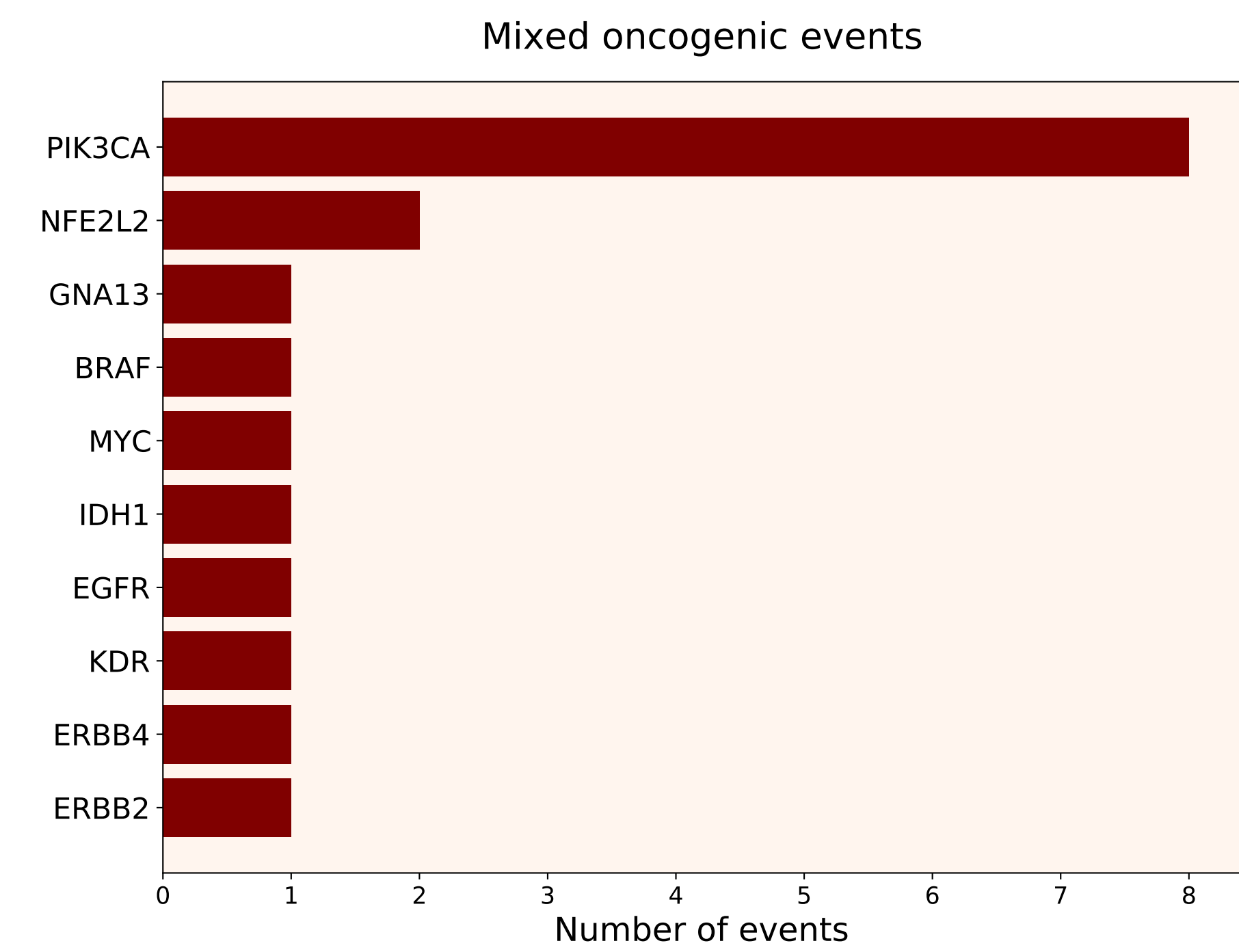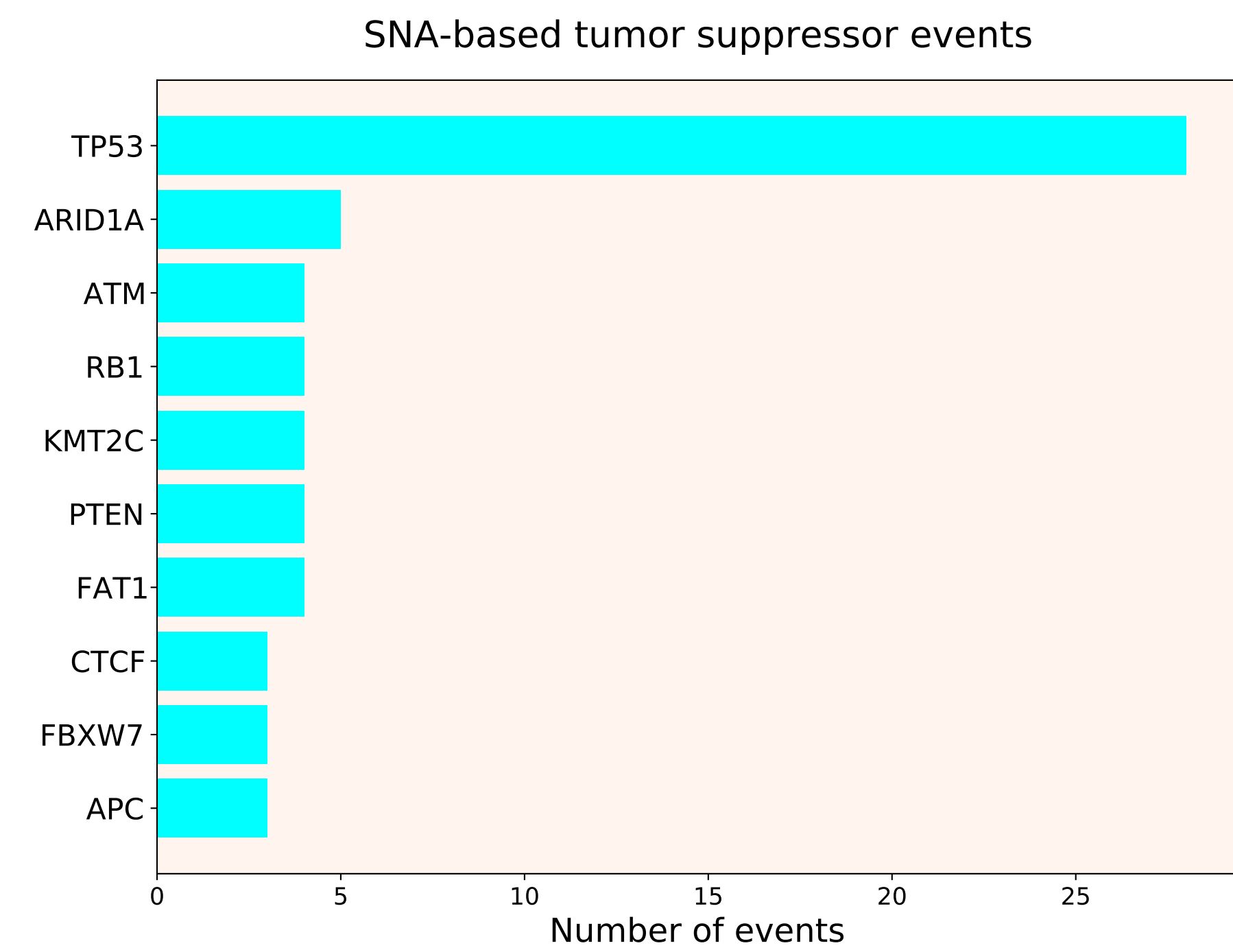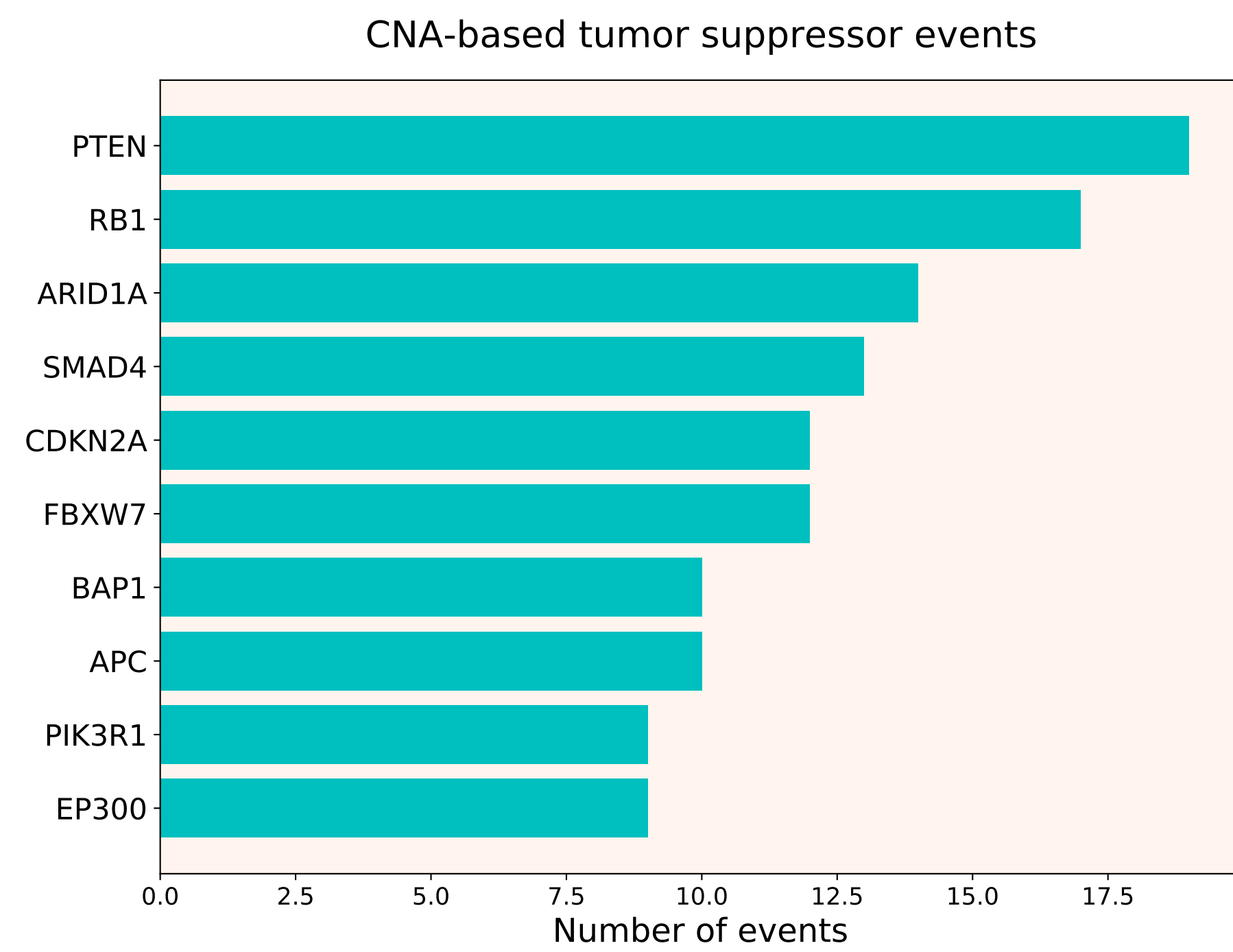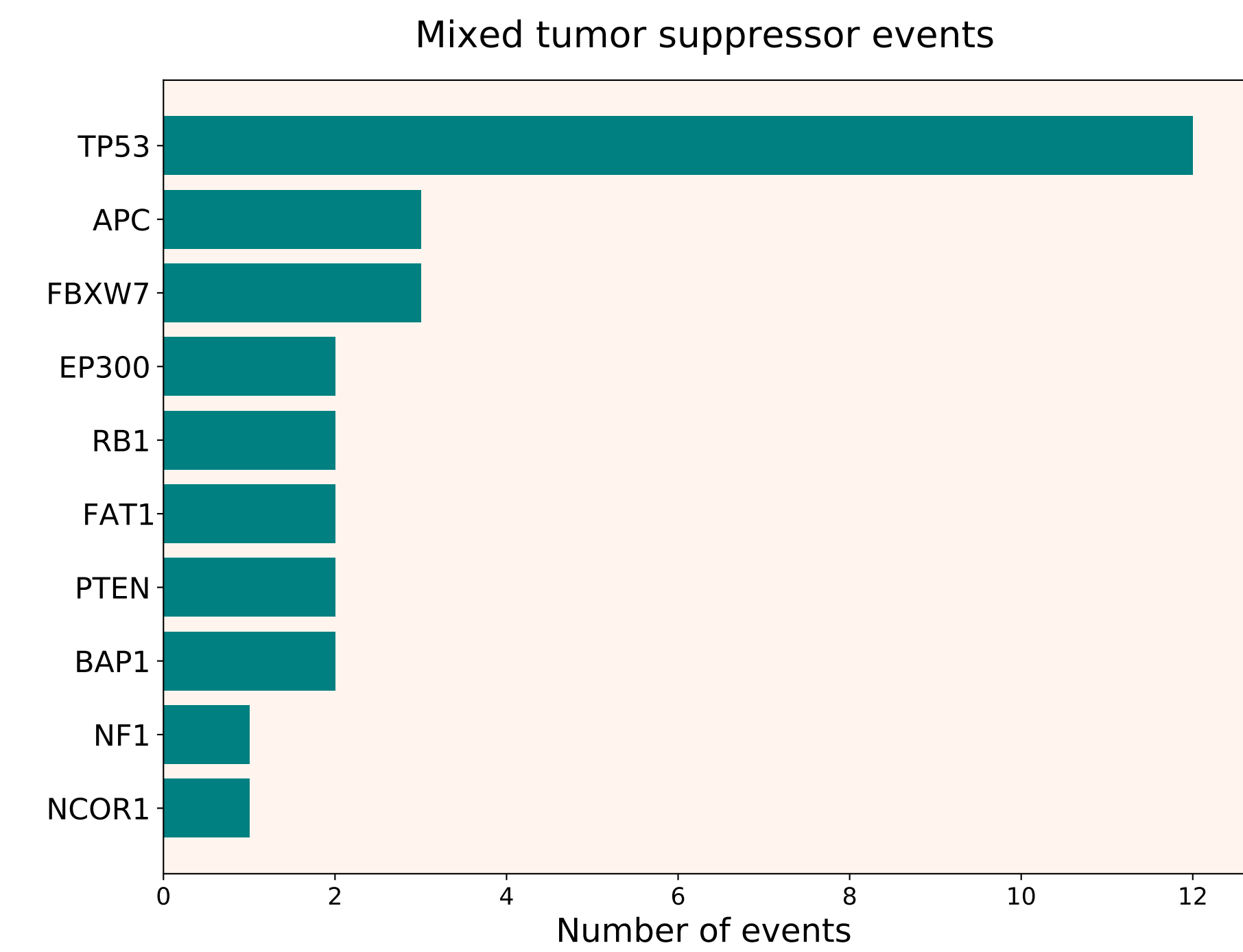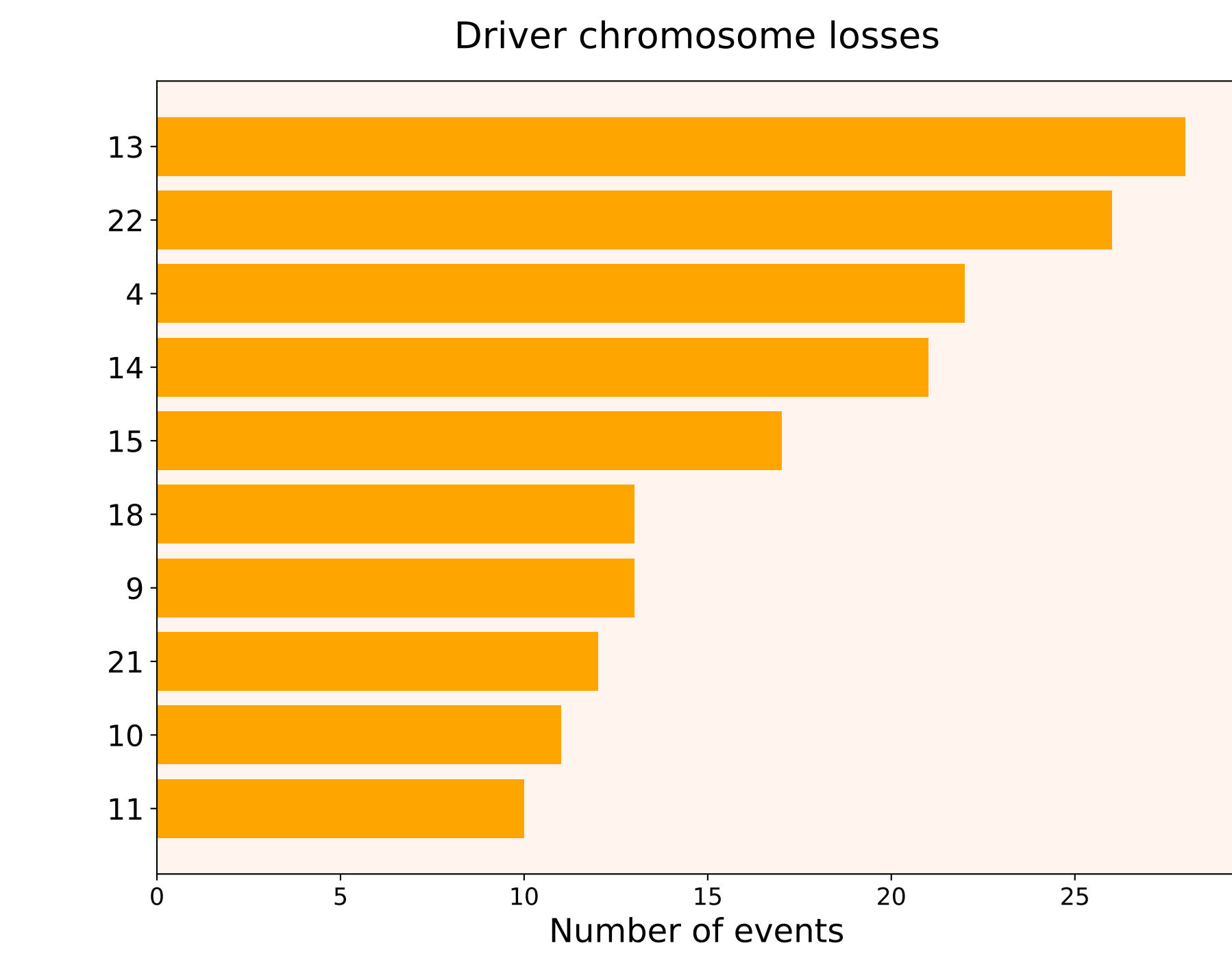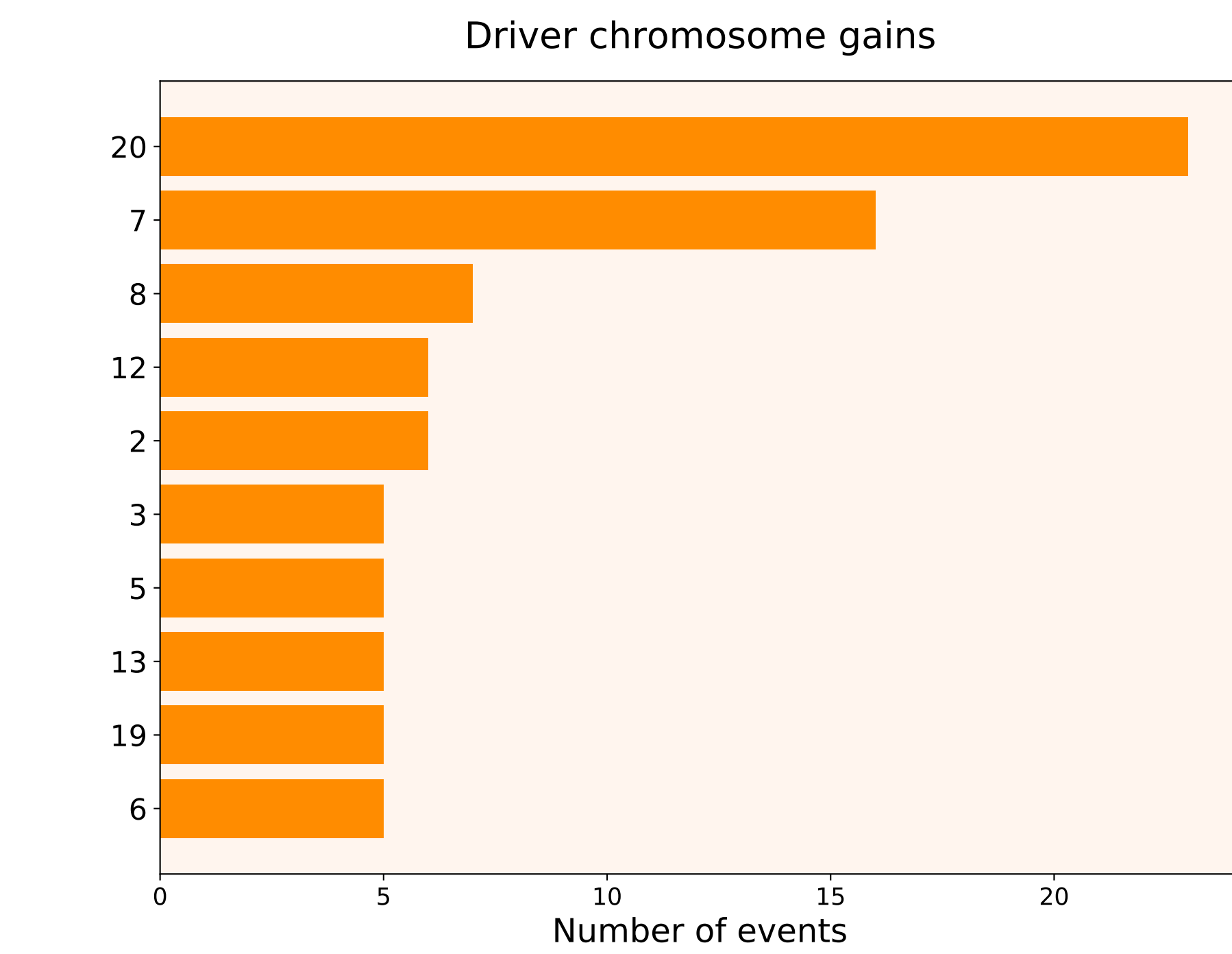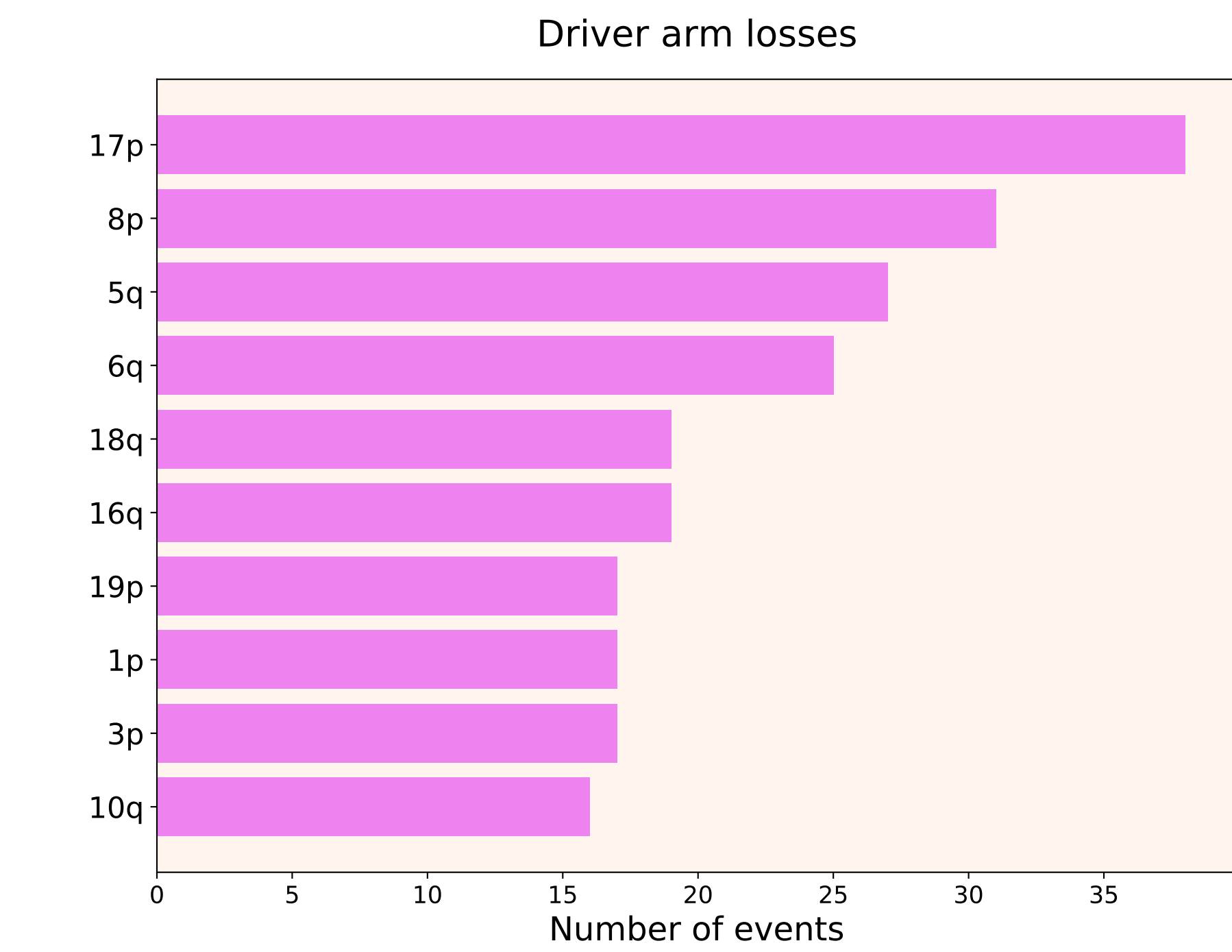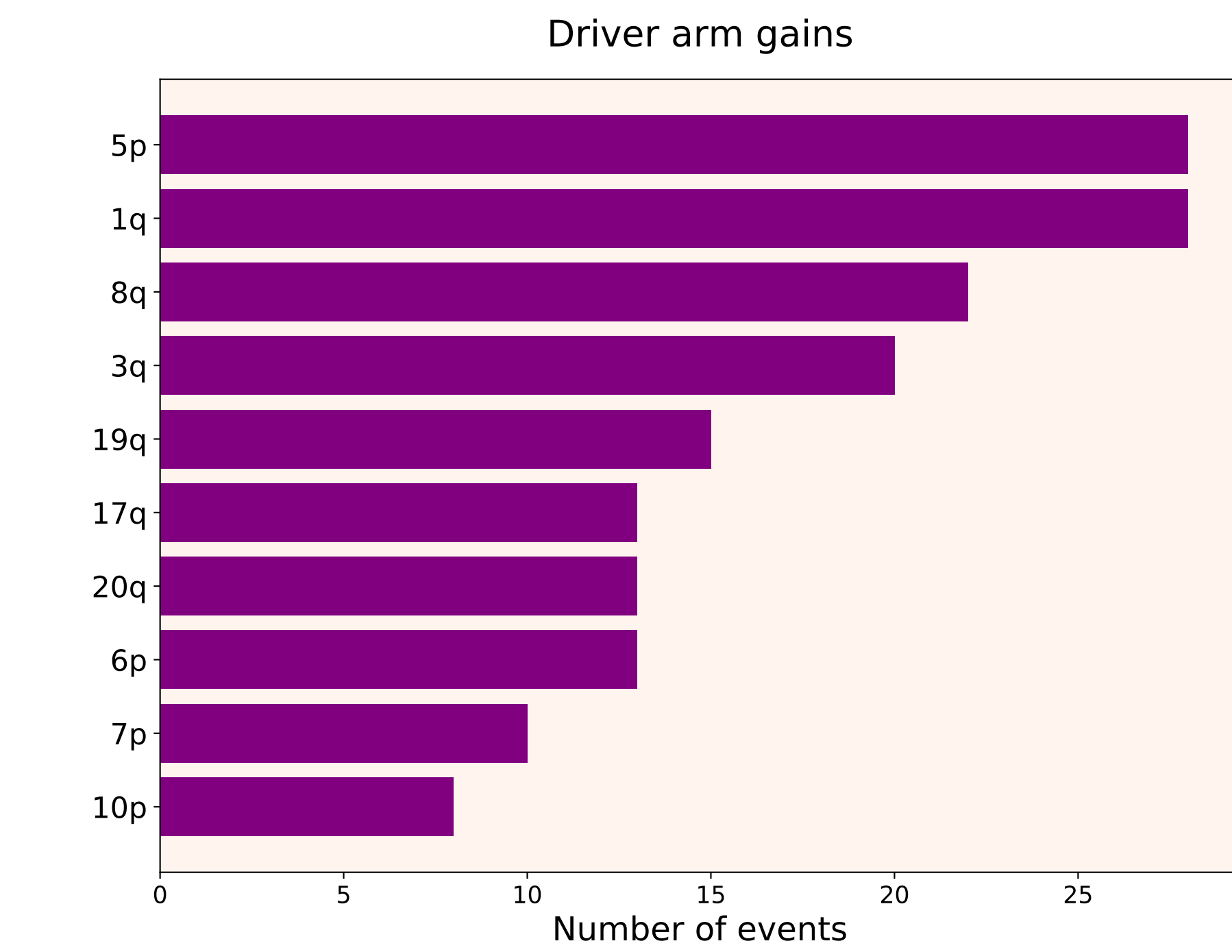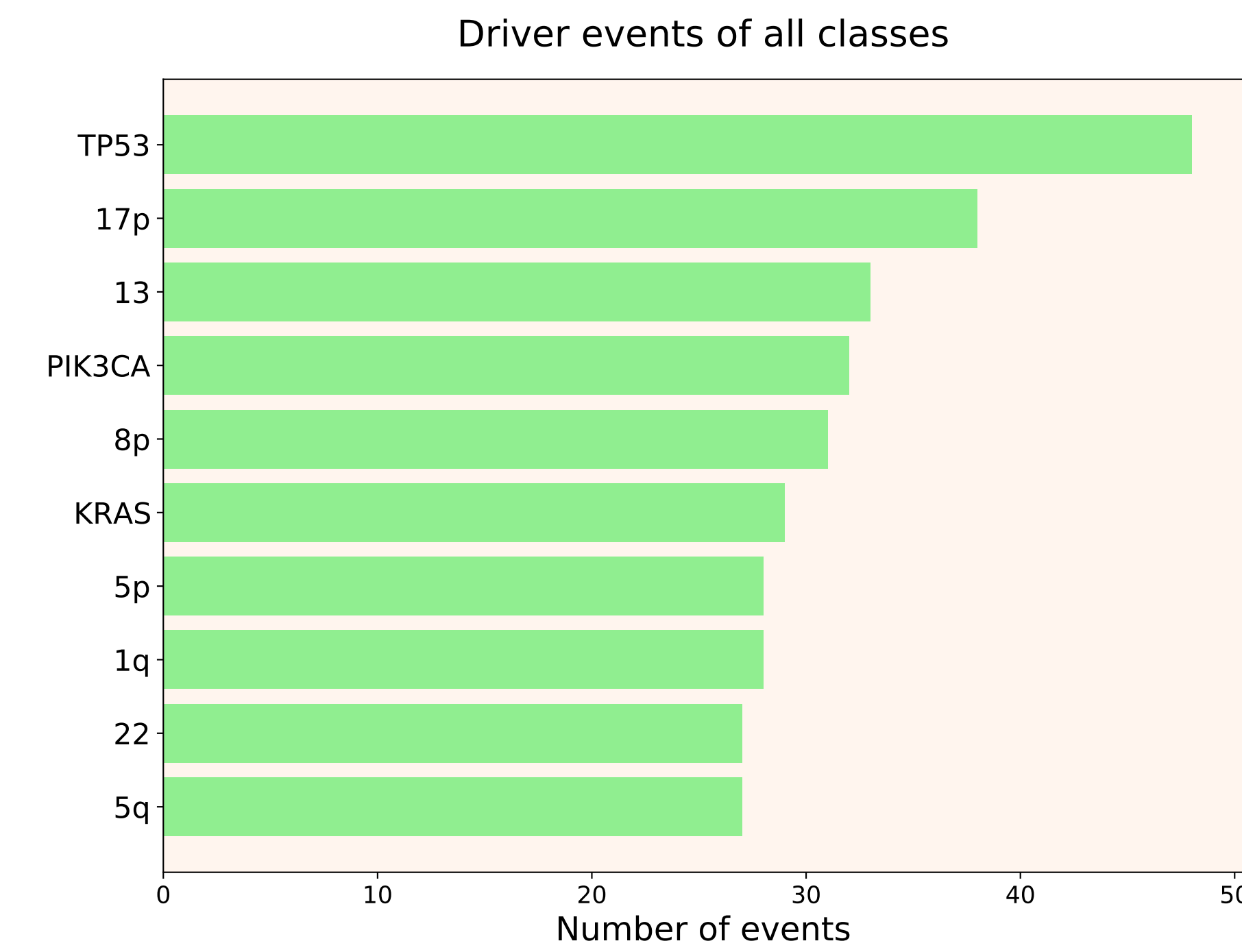

Supplement: Supplemental Information 2 [file peerj-10-13860-s002.zip › COHORTS/genes plots/detailed/2021_8_16_14_9_distribution_events_detailed_females_22.pdf]

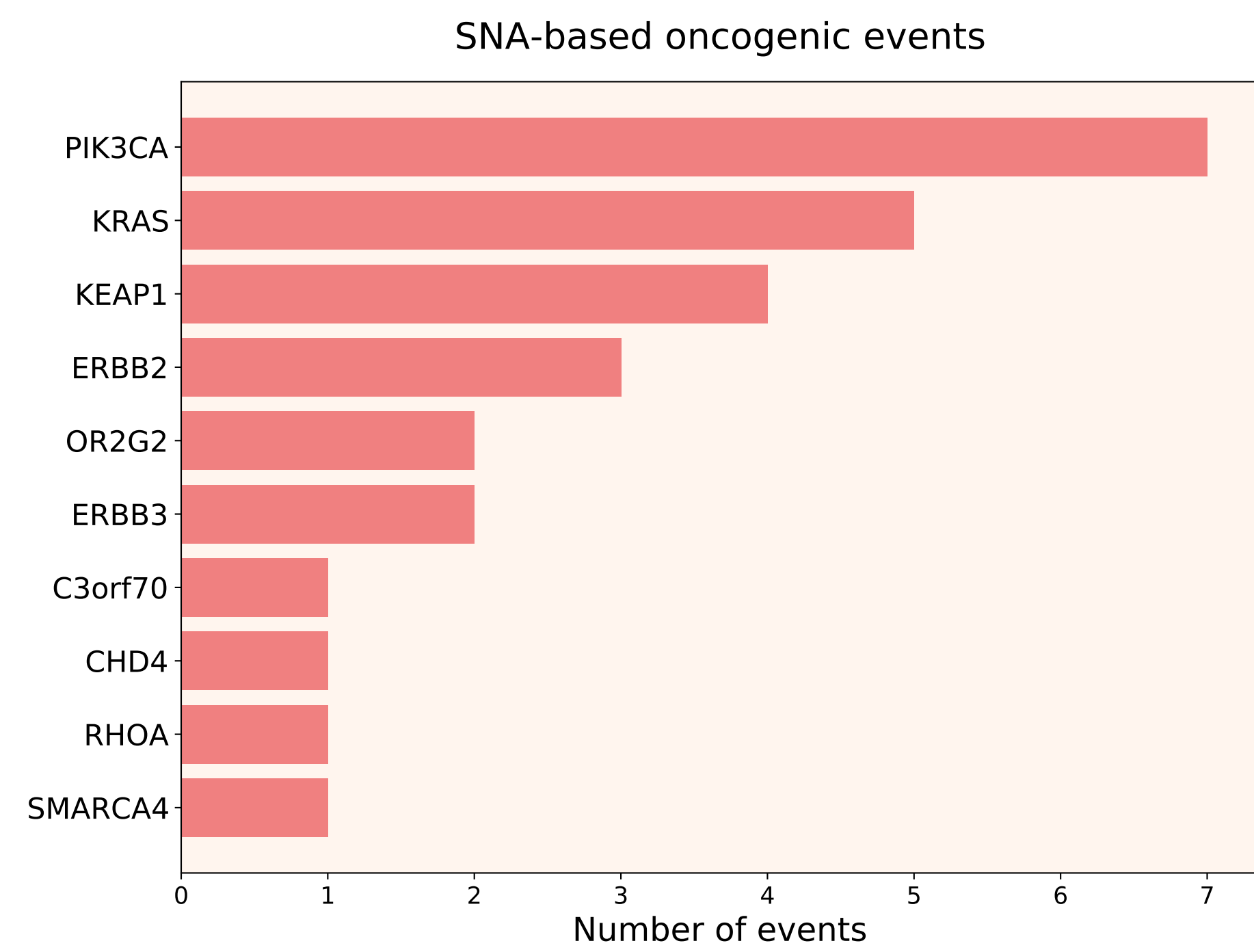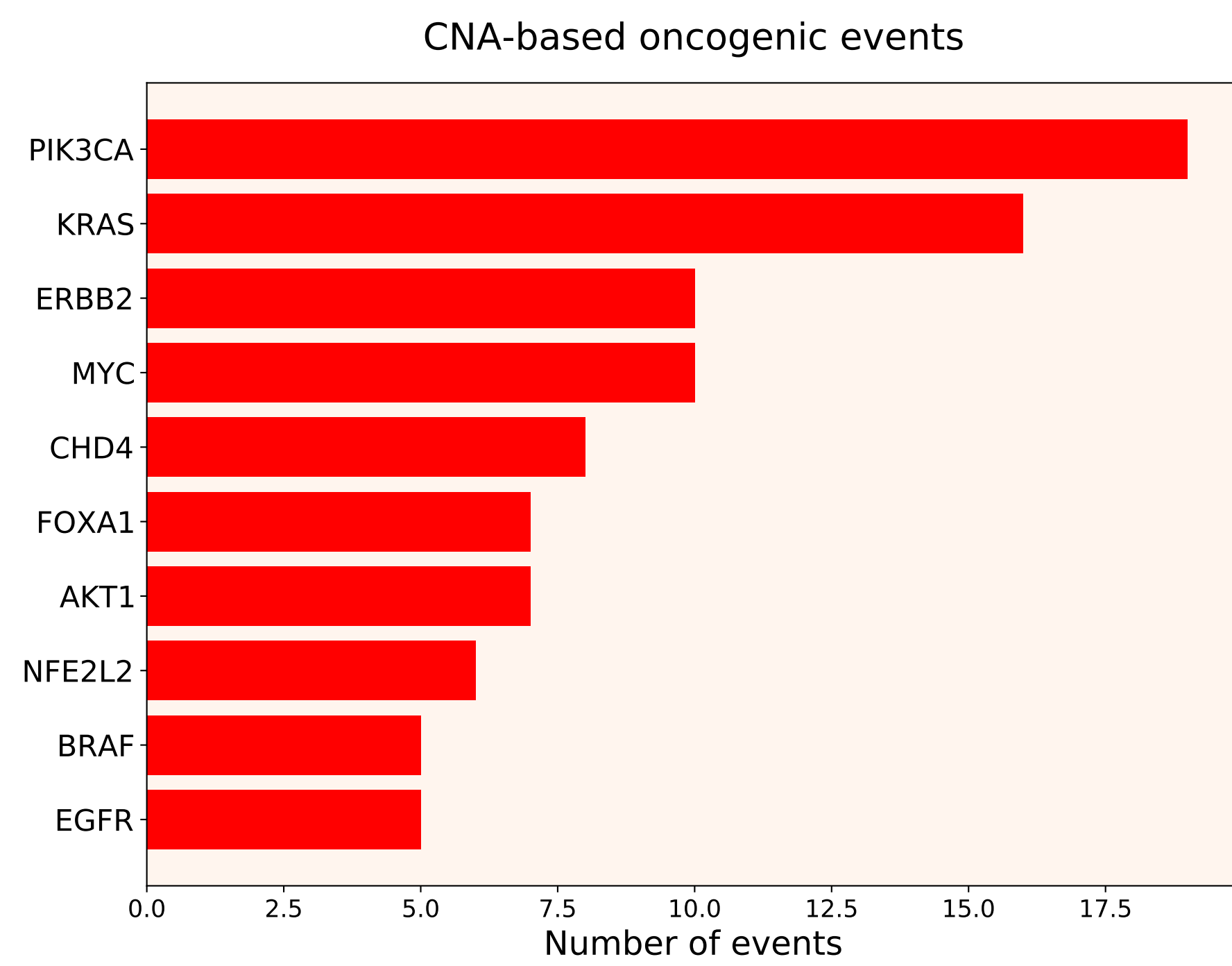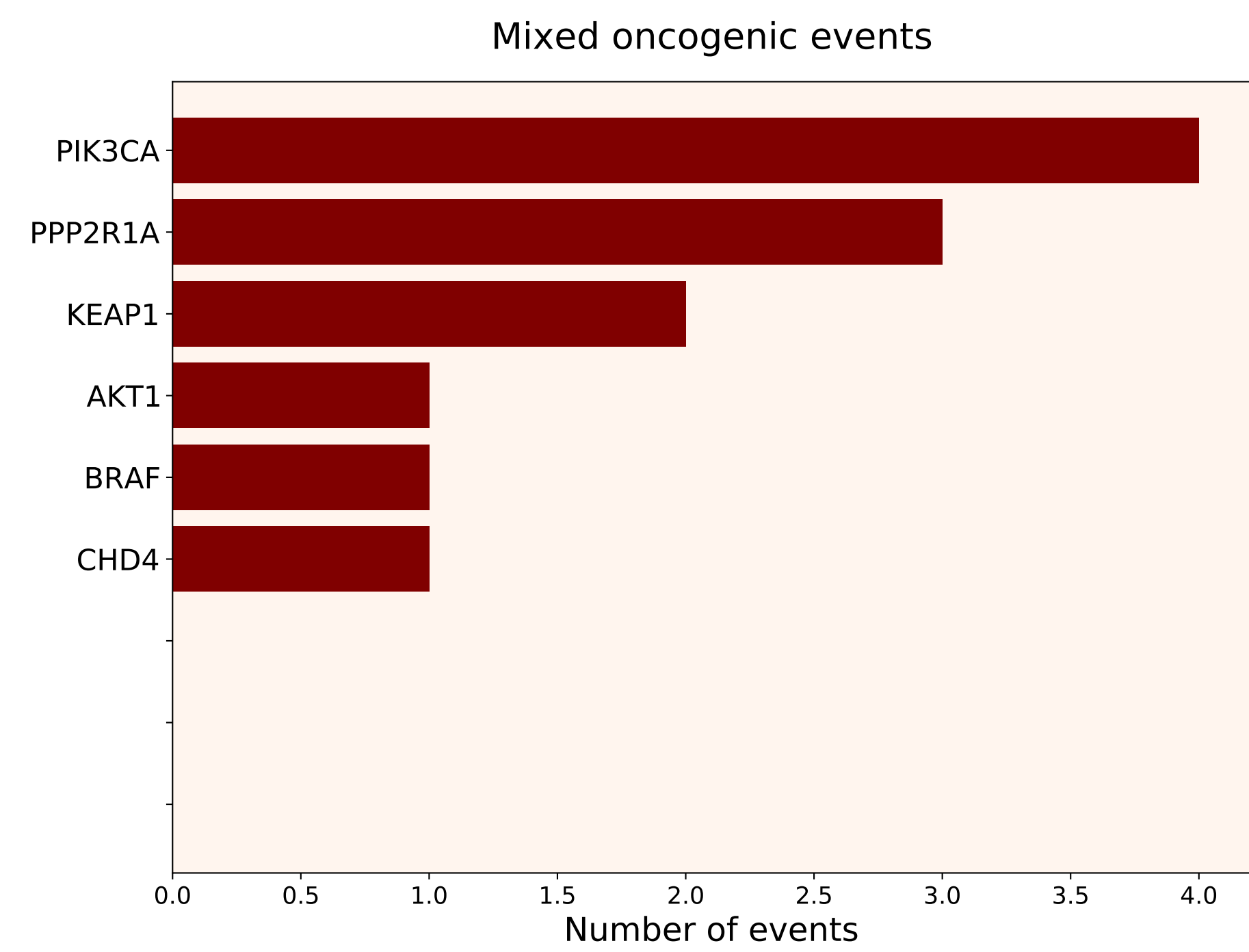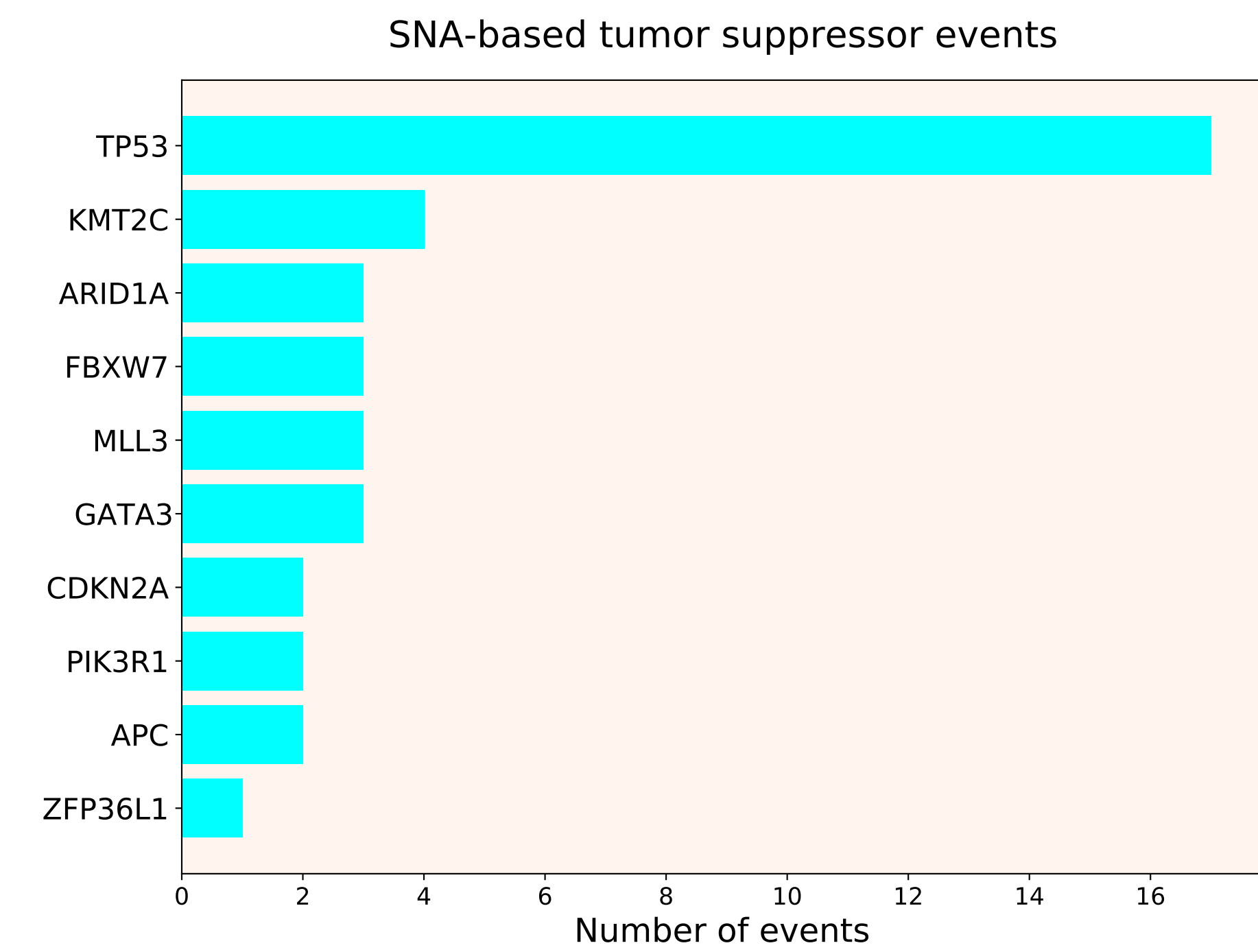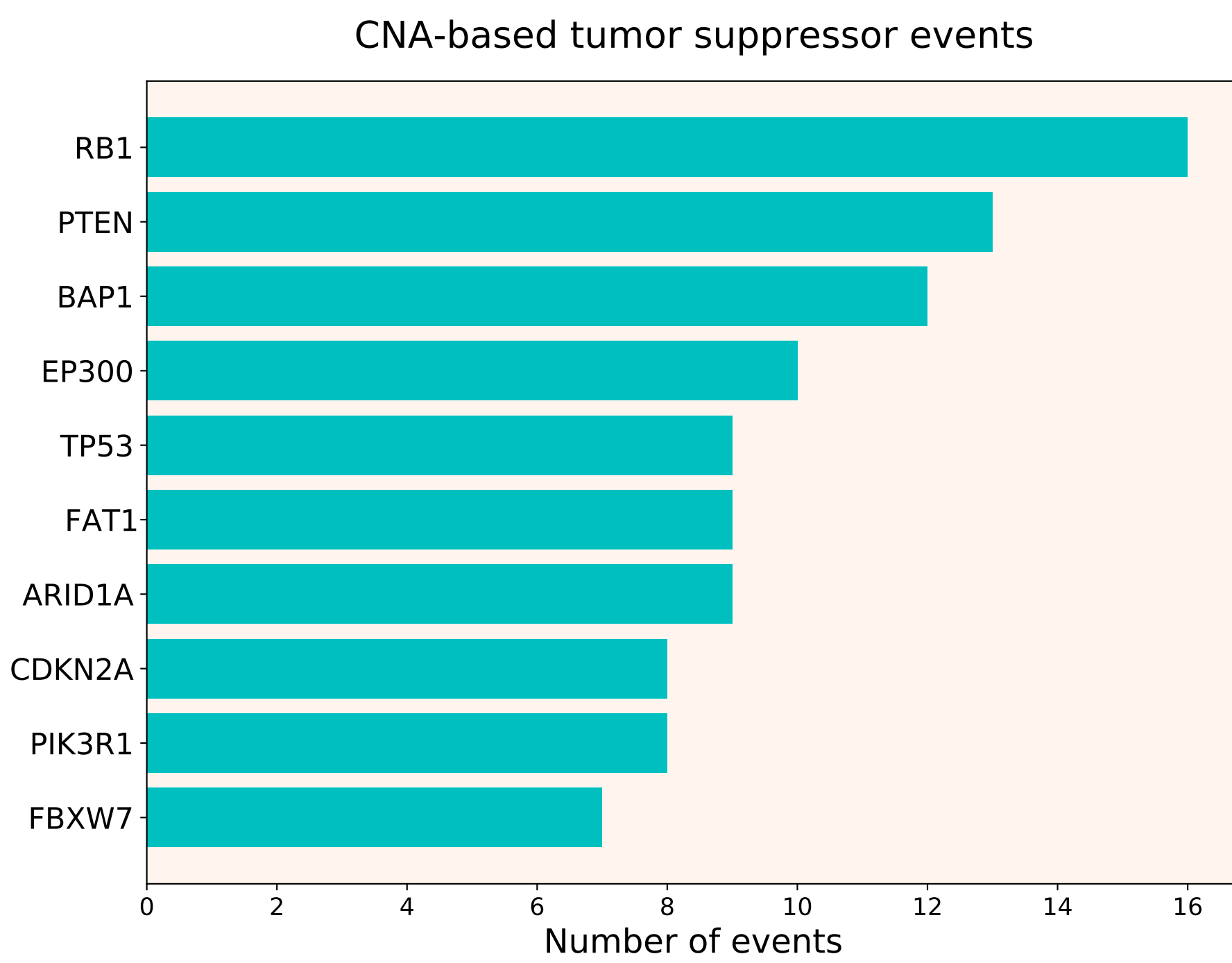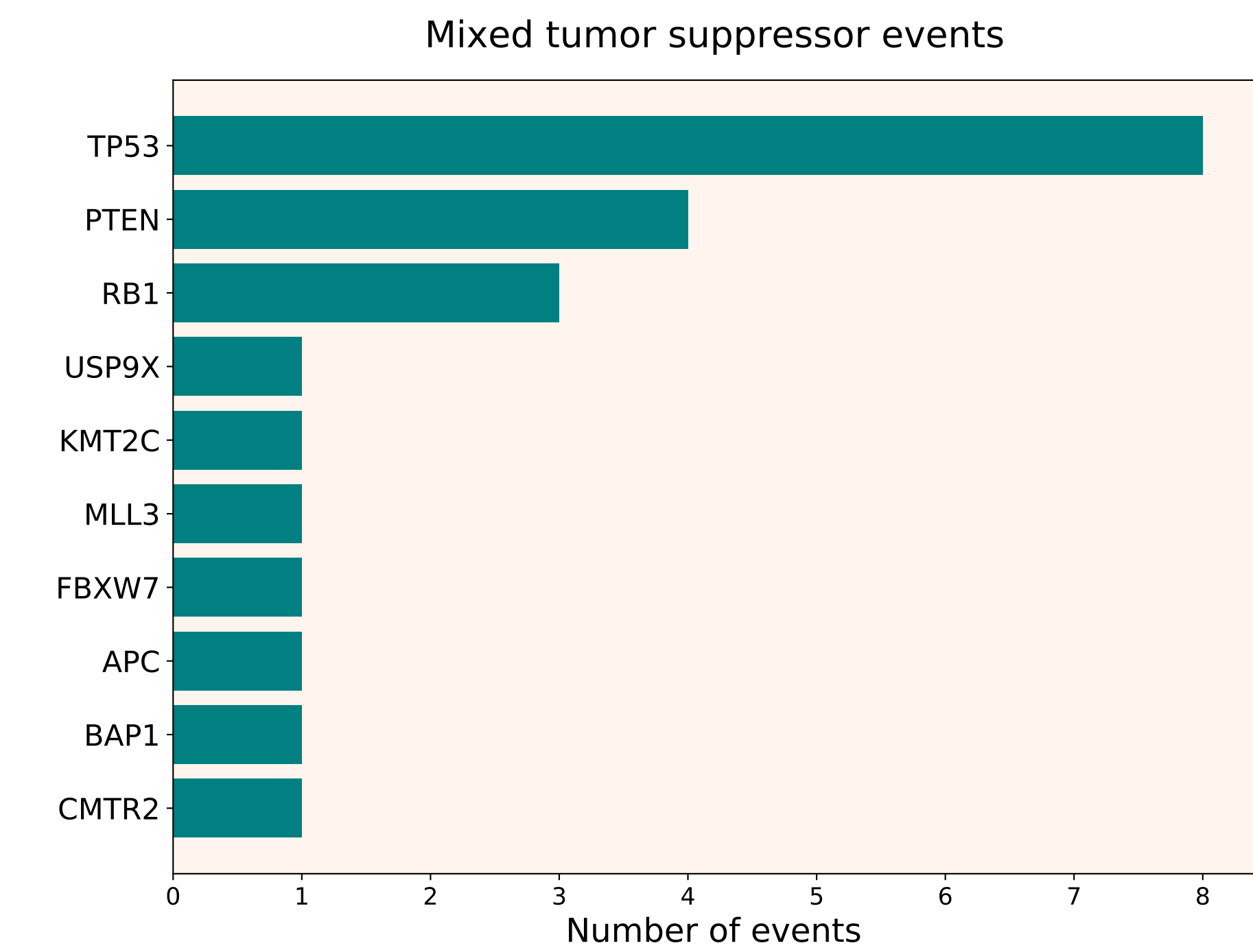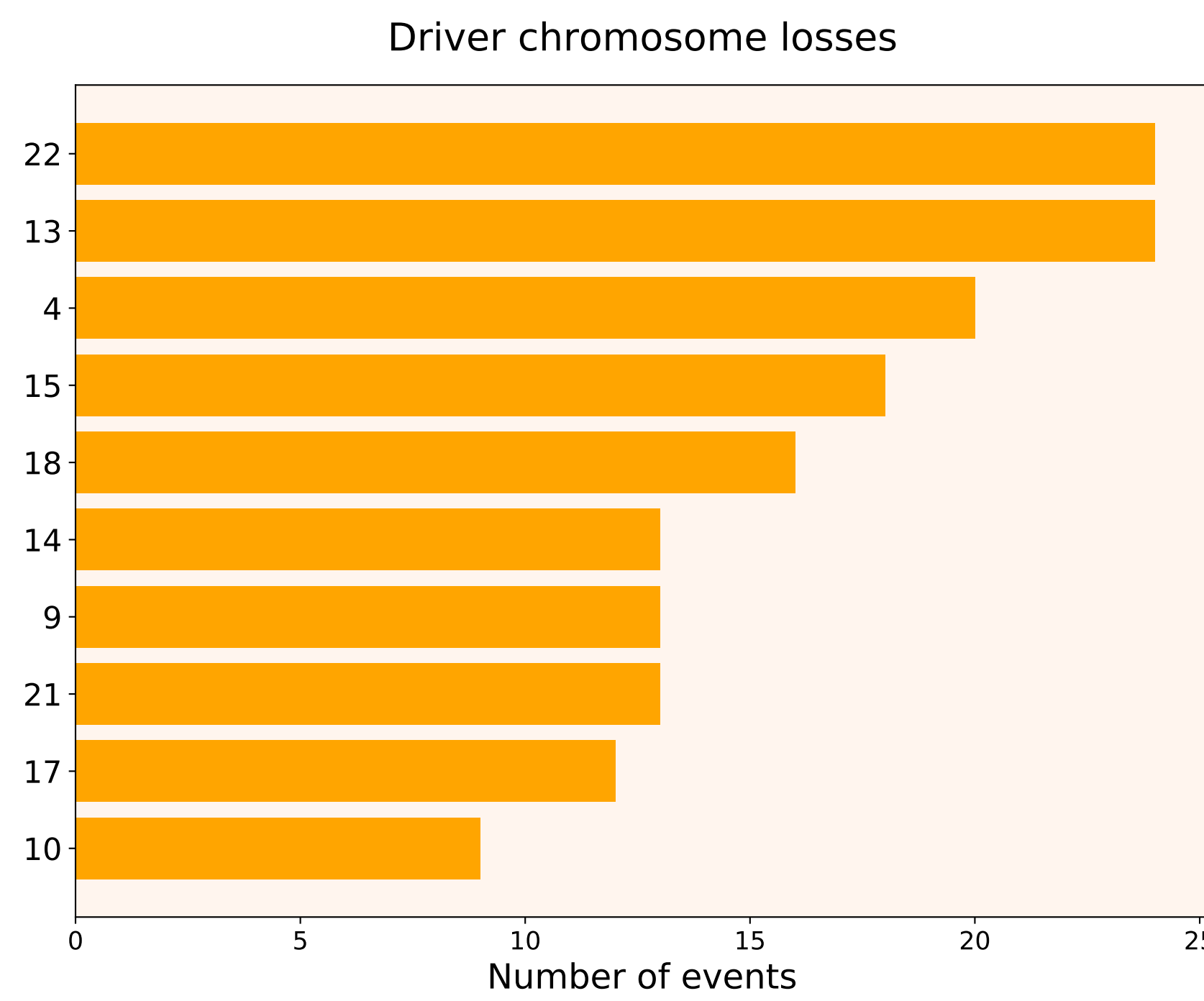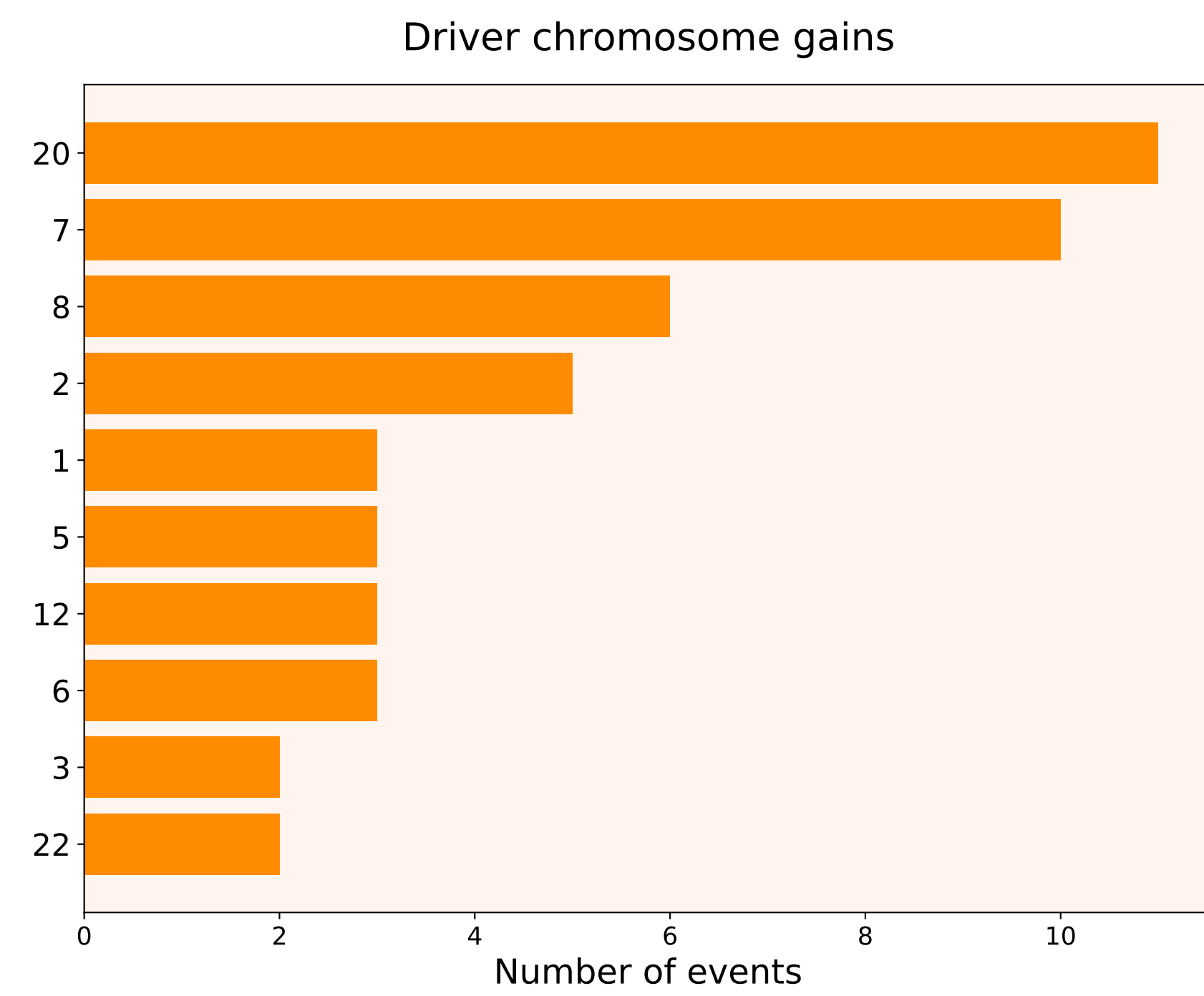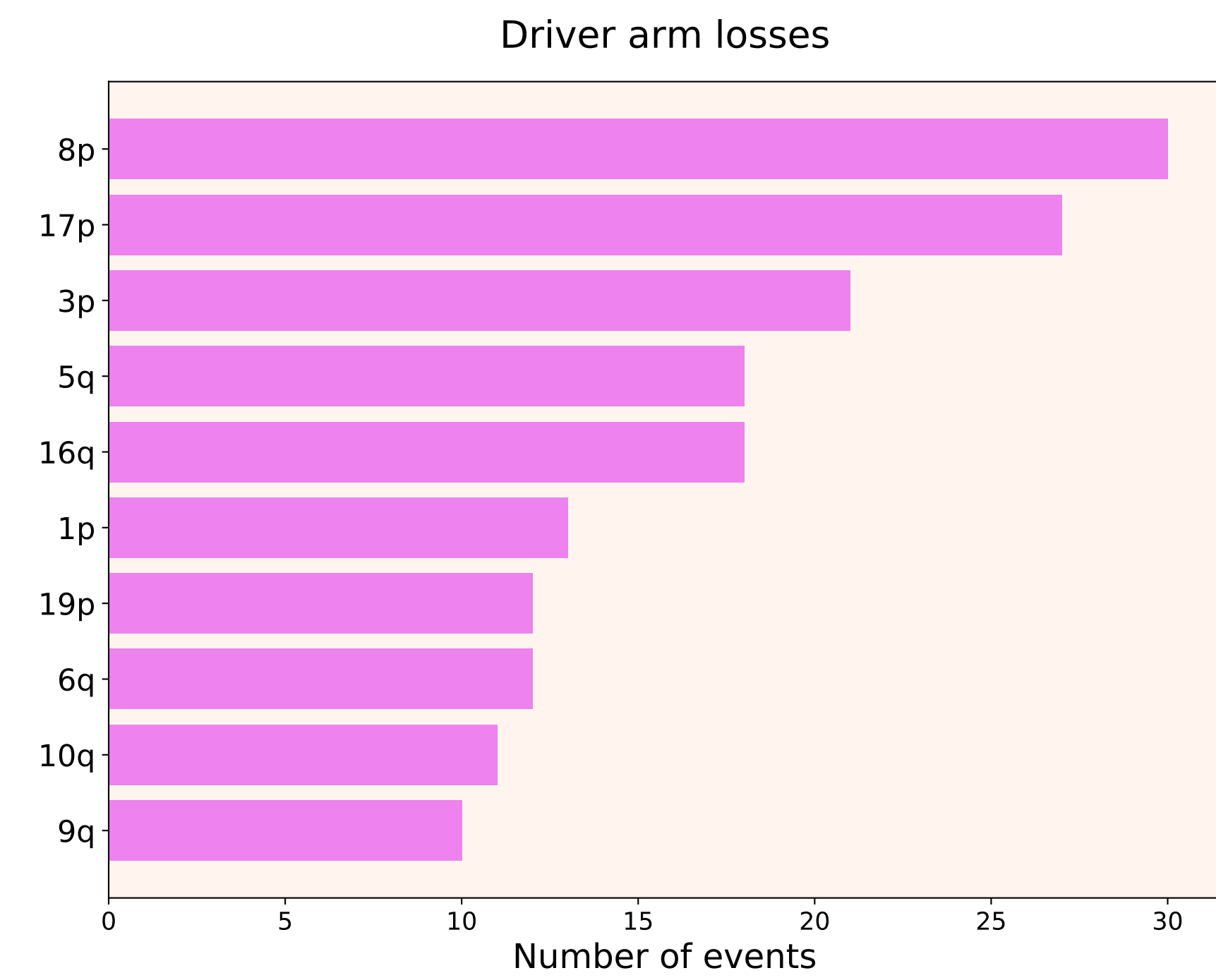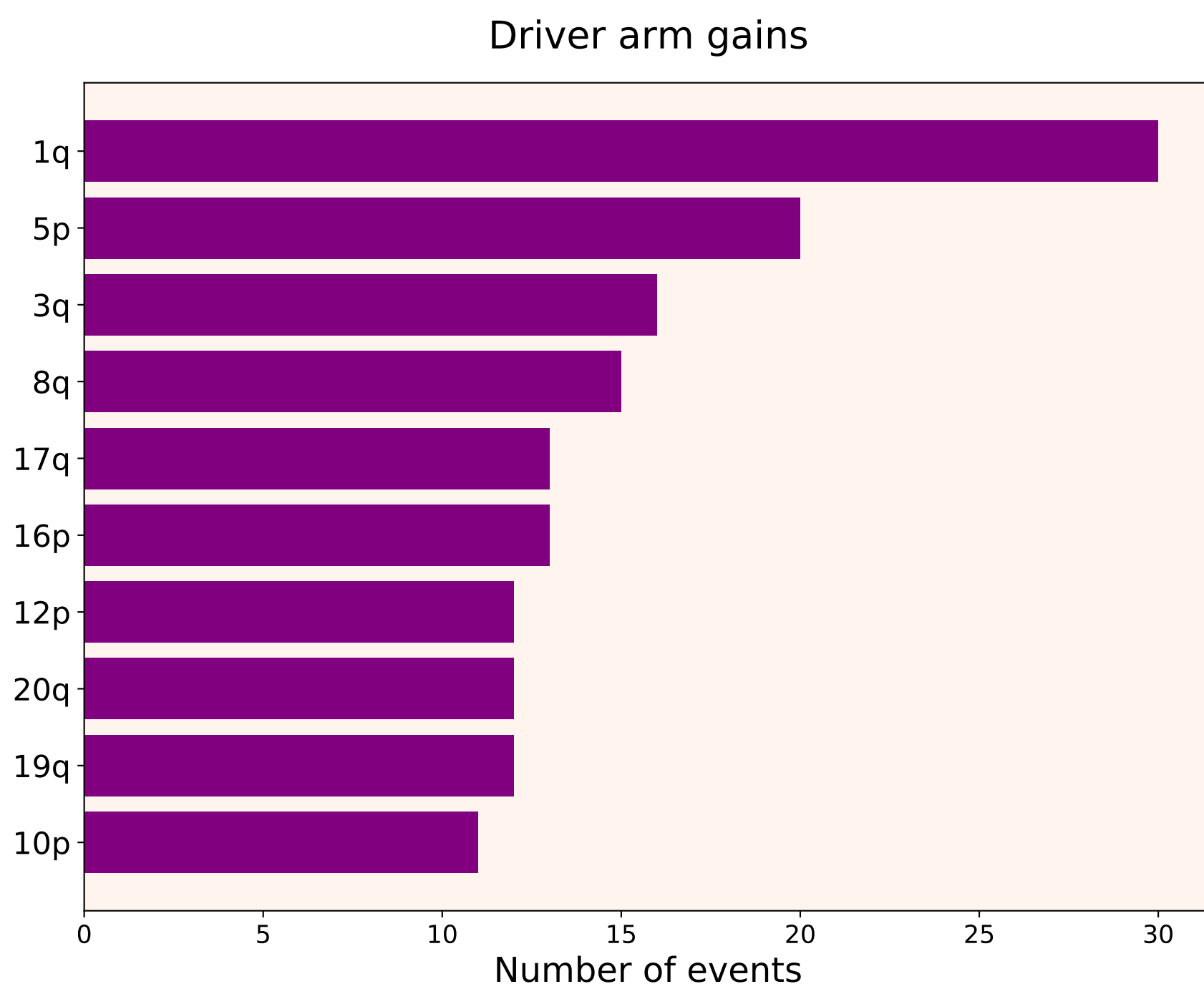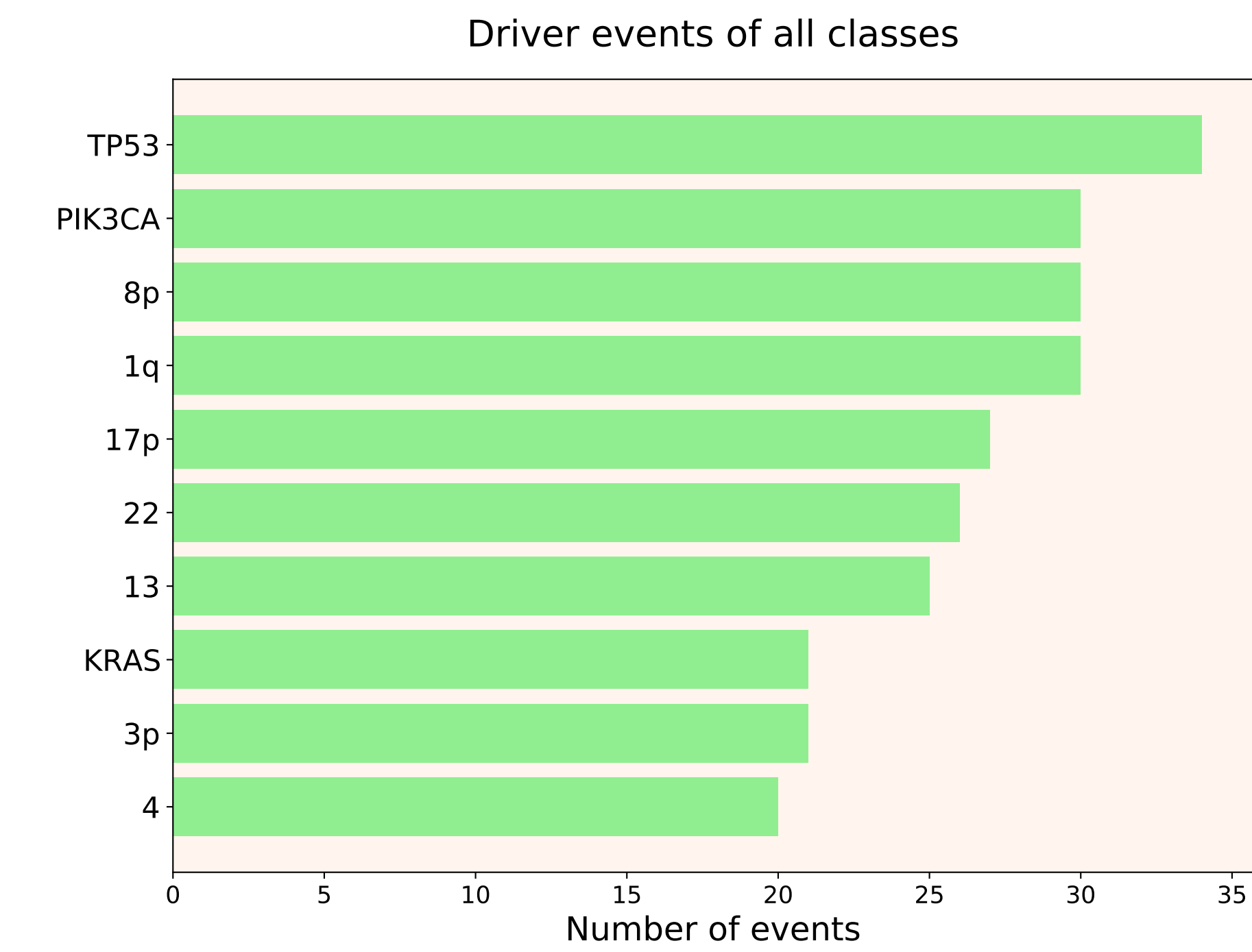

Supplement: Supplemental Information 2 [file peerj-10-13860-s002.zip › COHORTS/genes plots/detailed/2021_8_16_14_9_distribution_events_detailed_females_23.pdf]

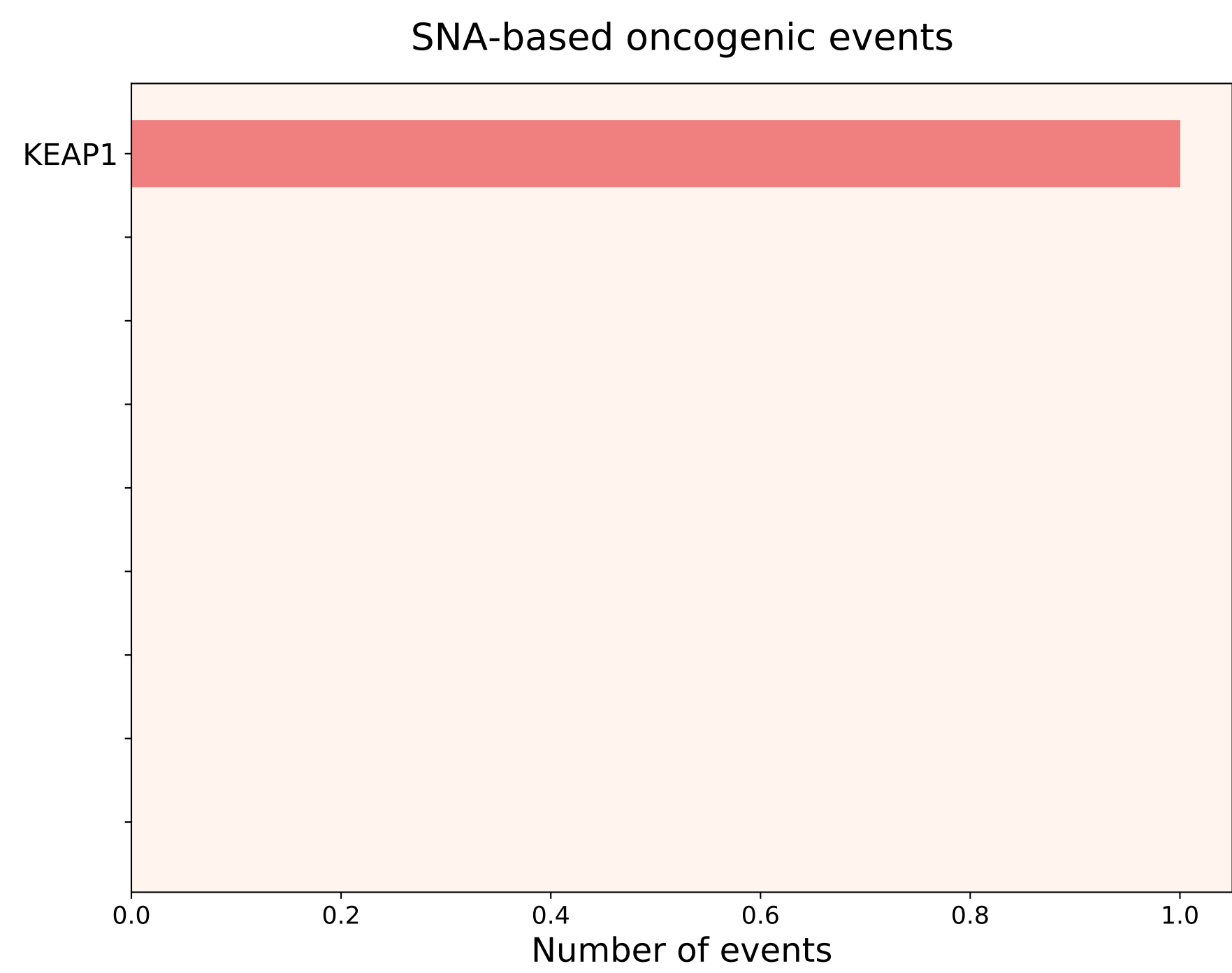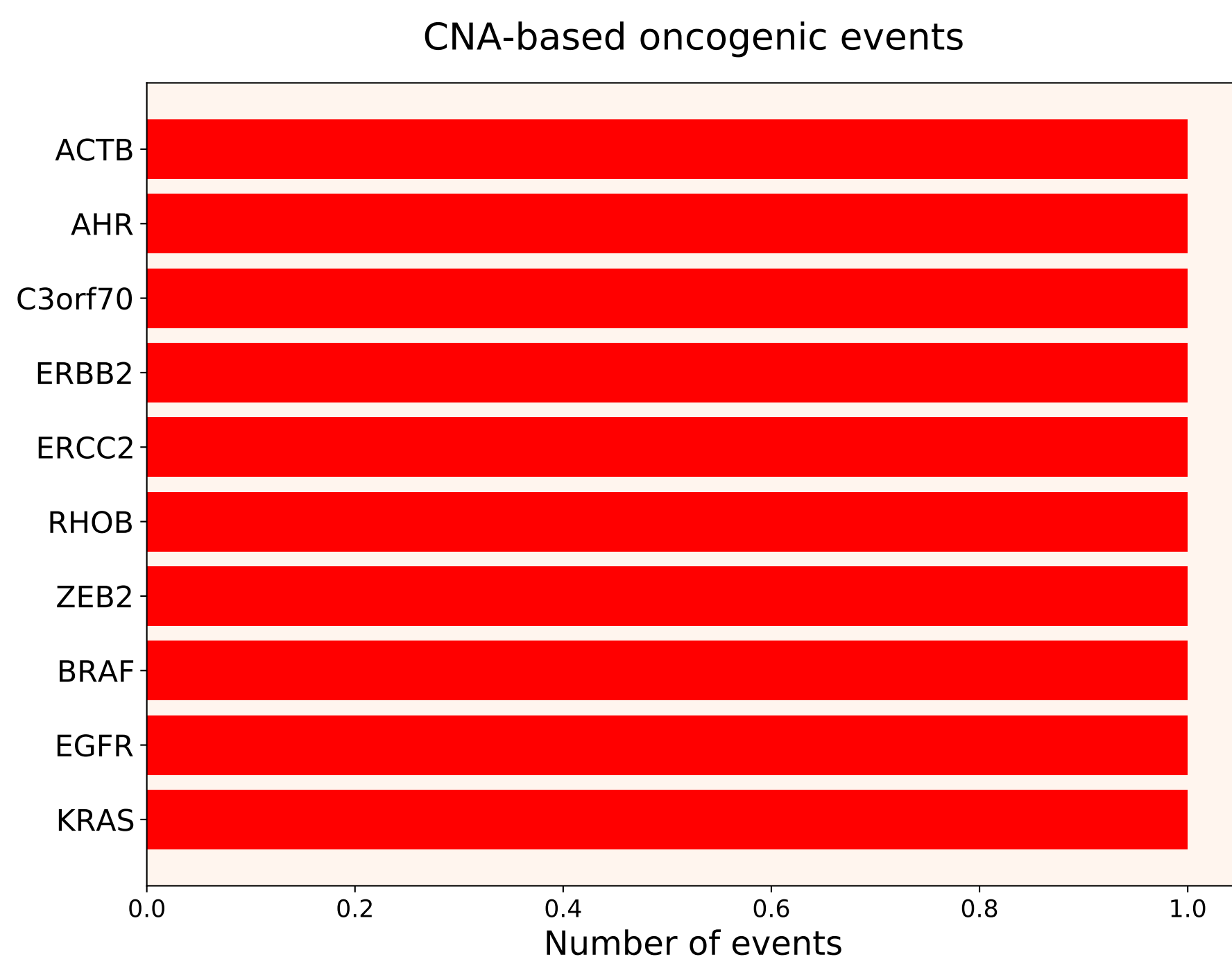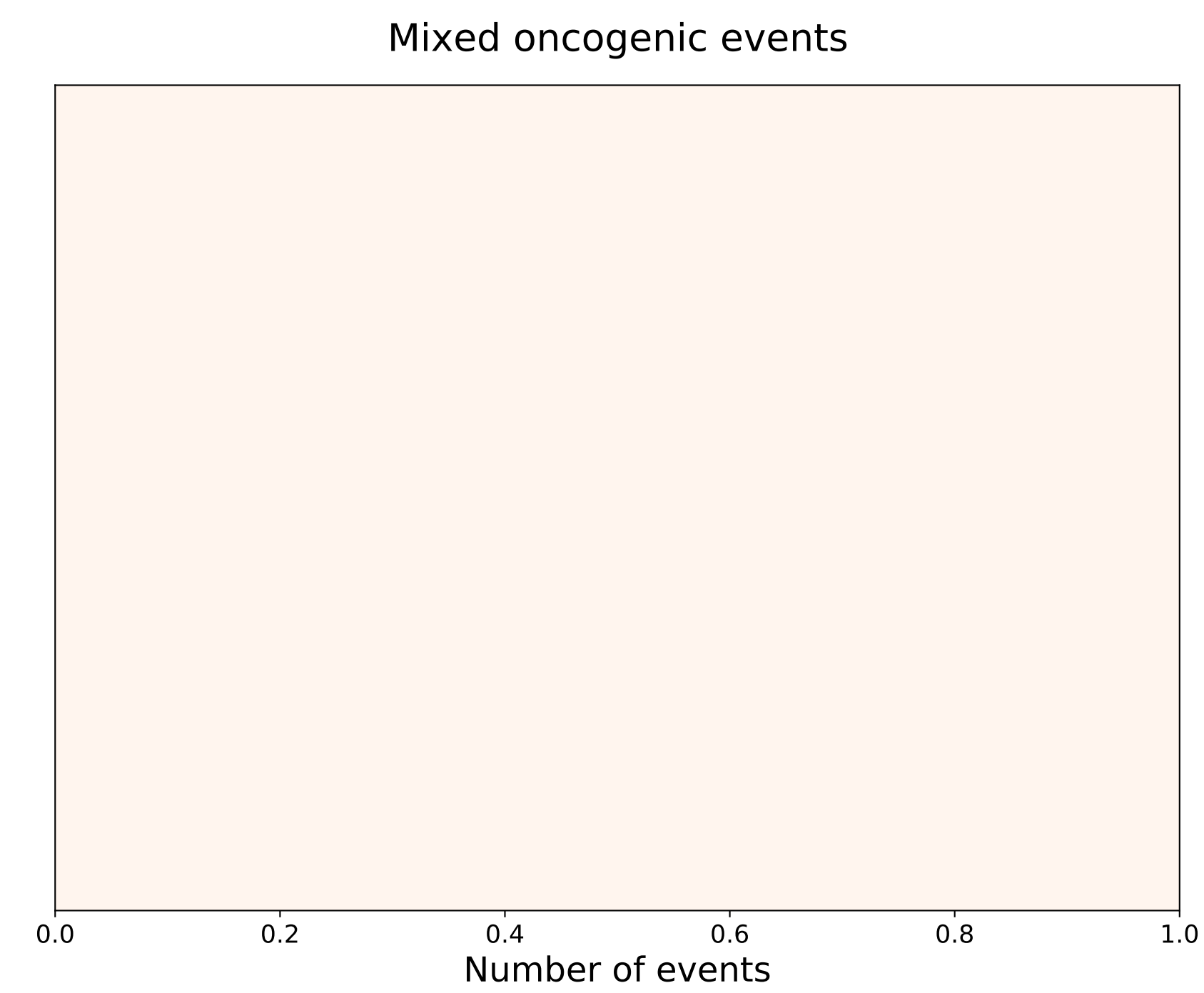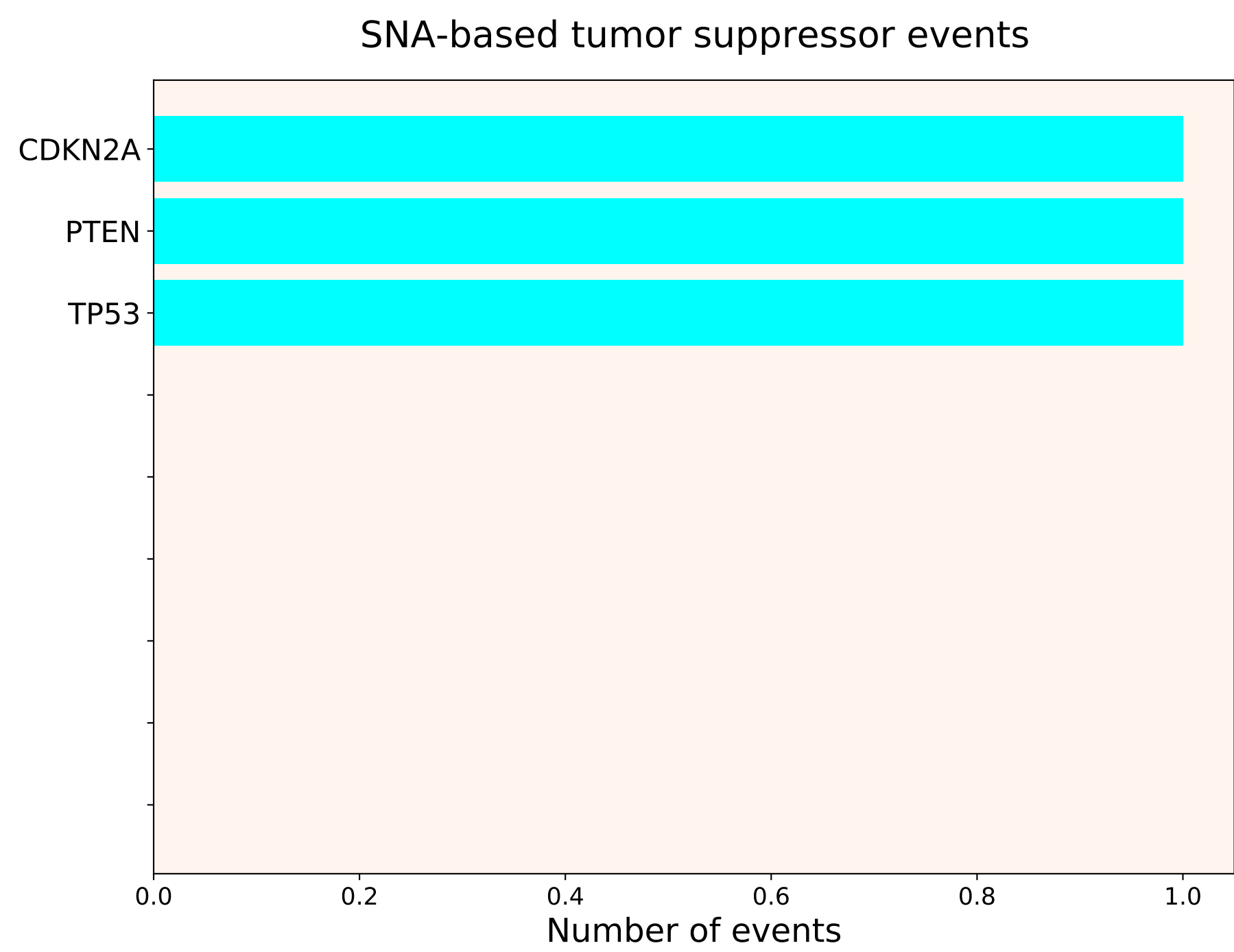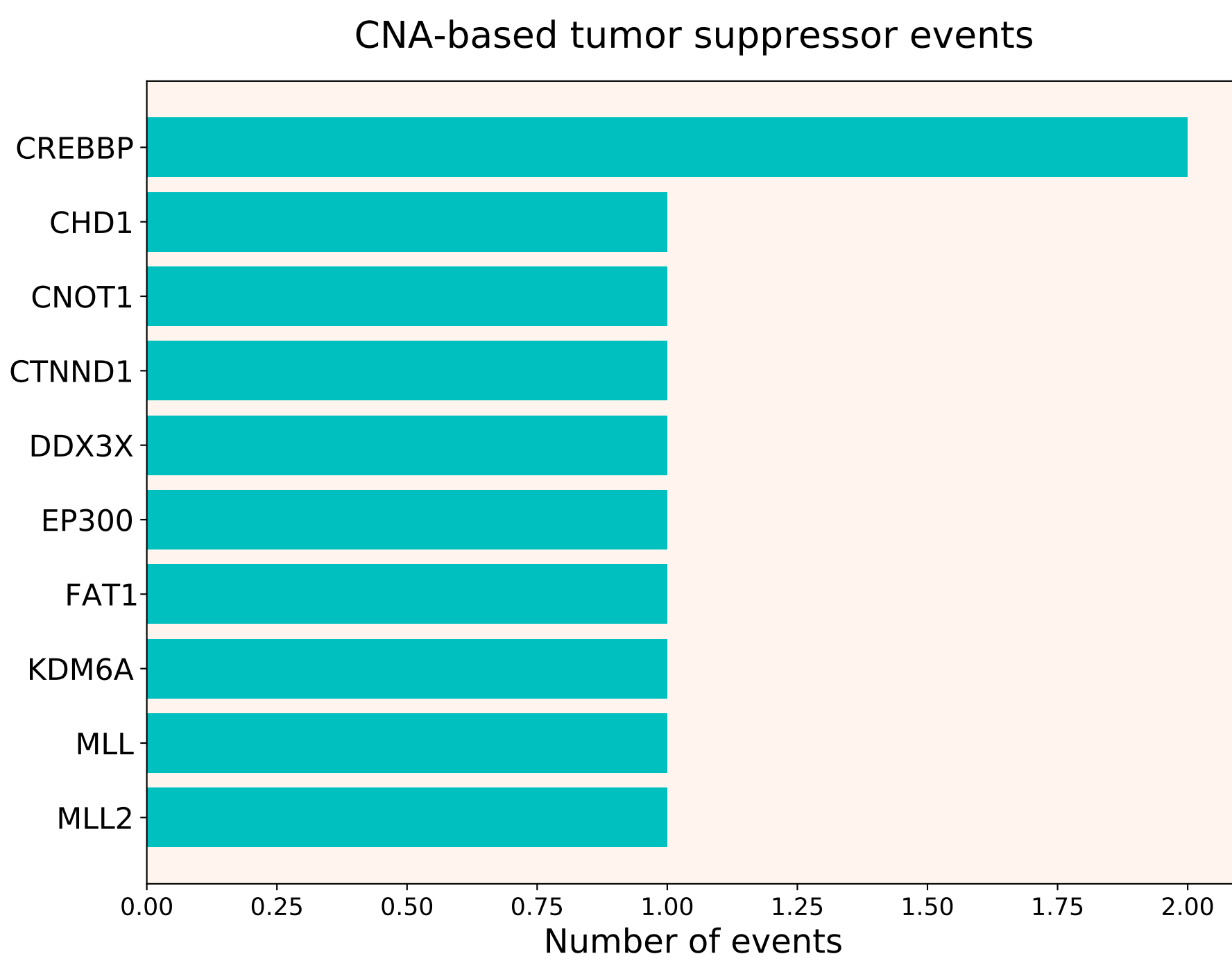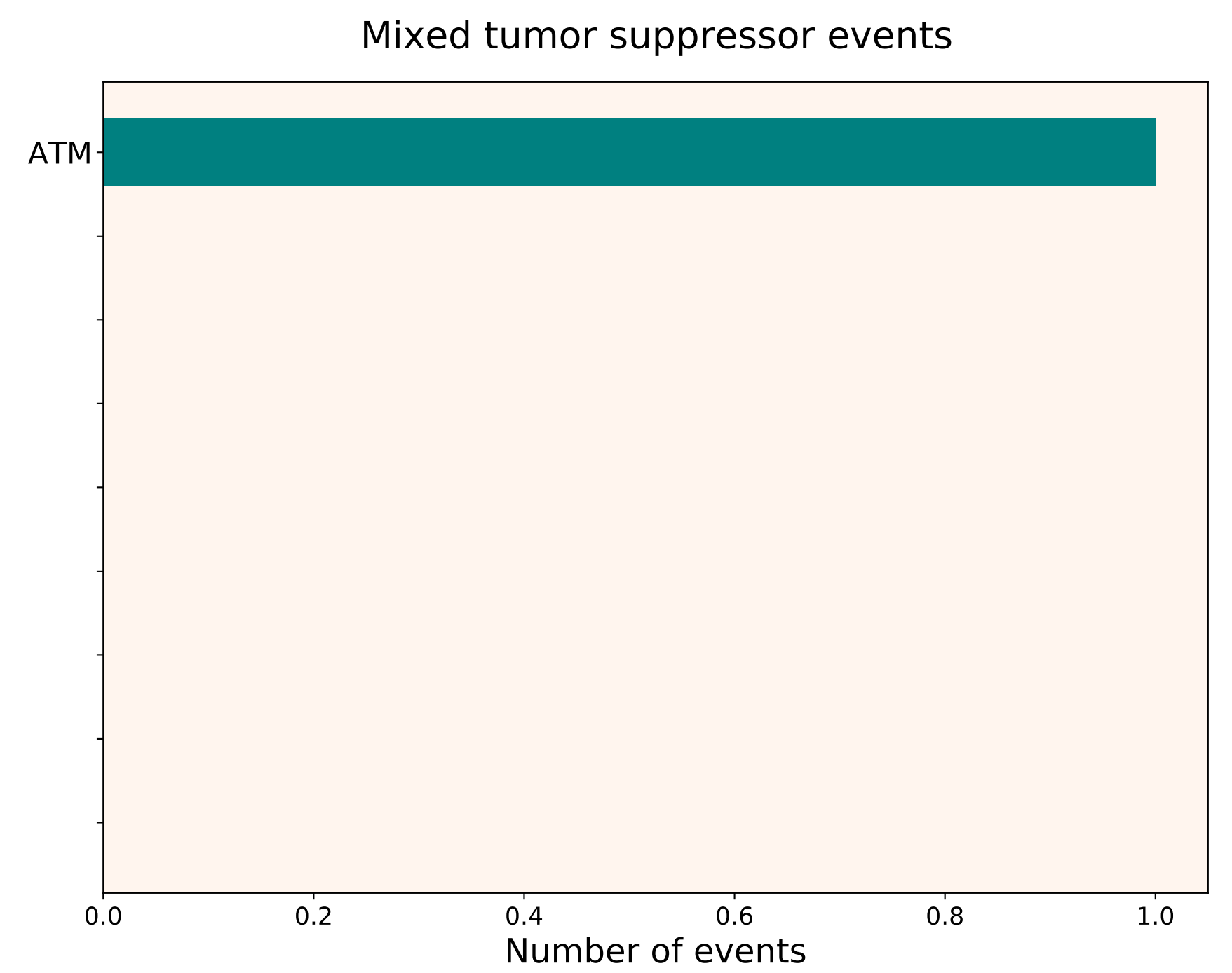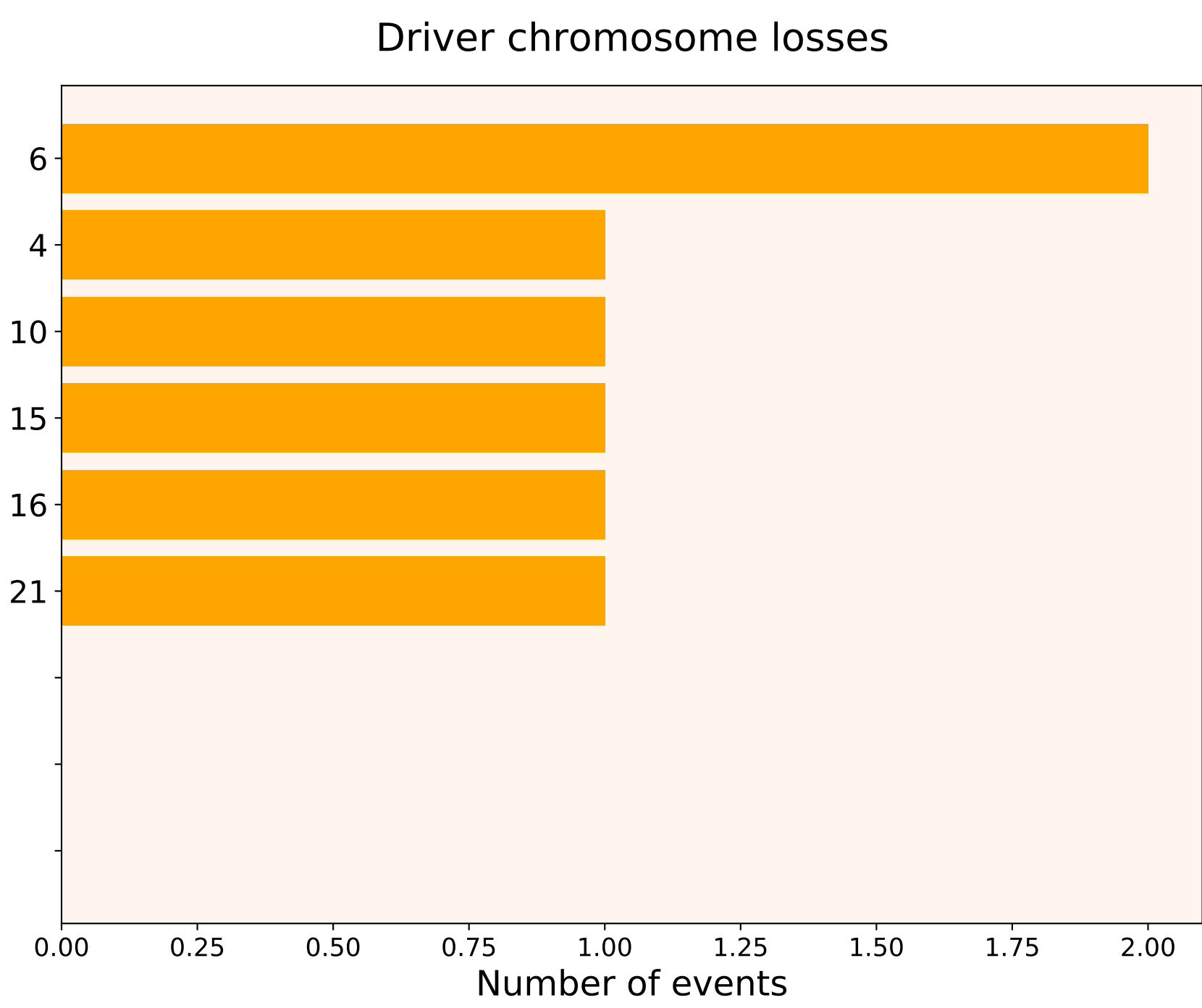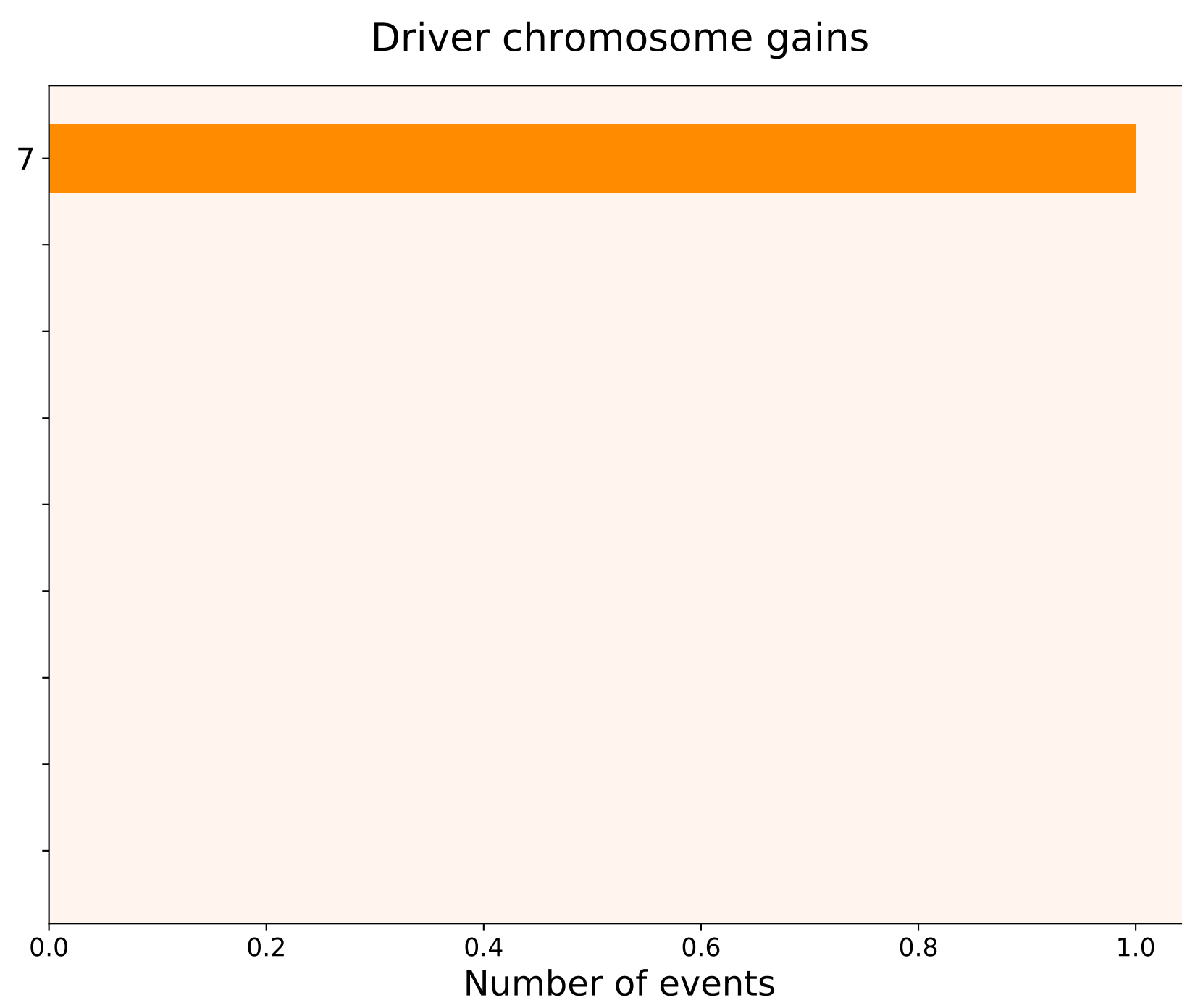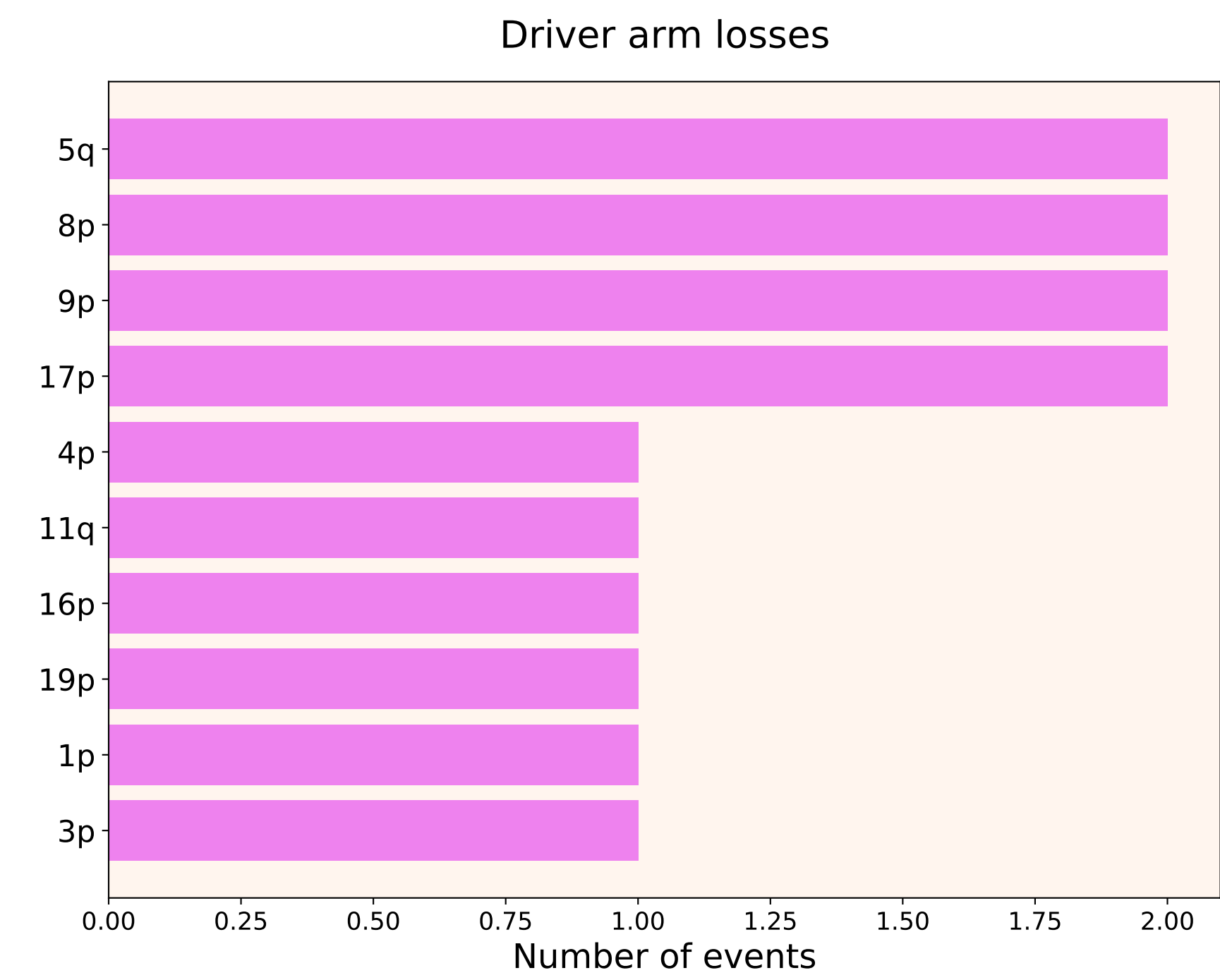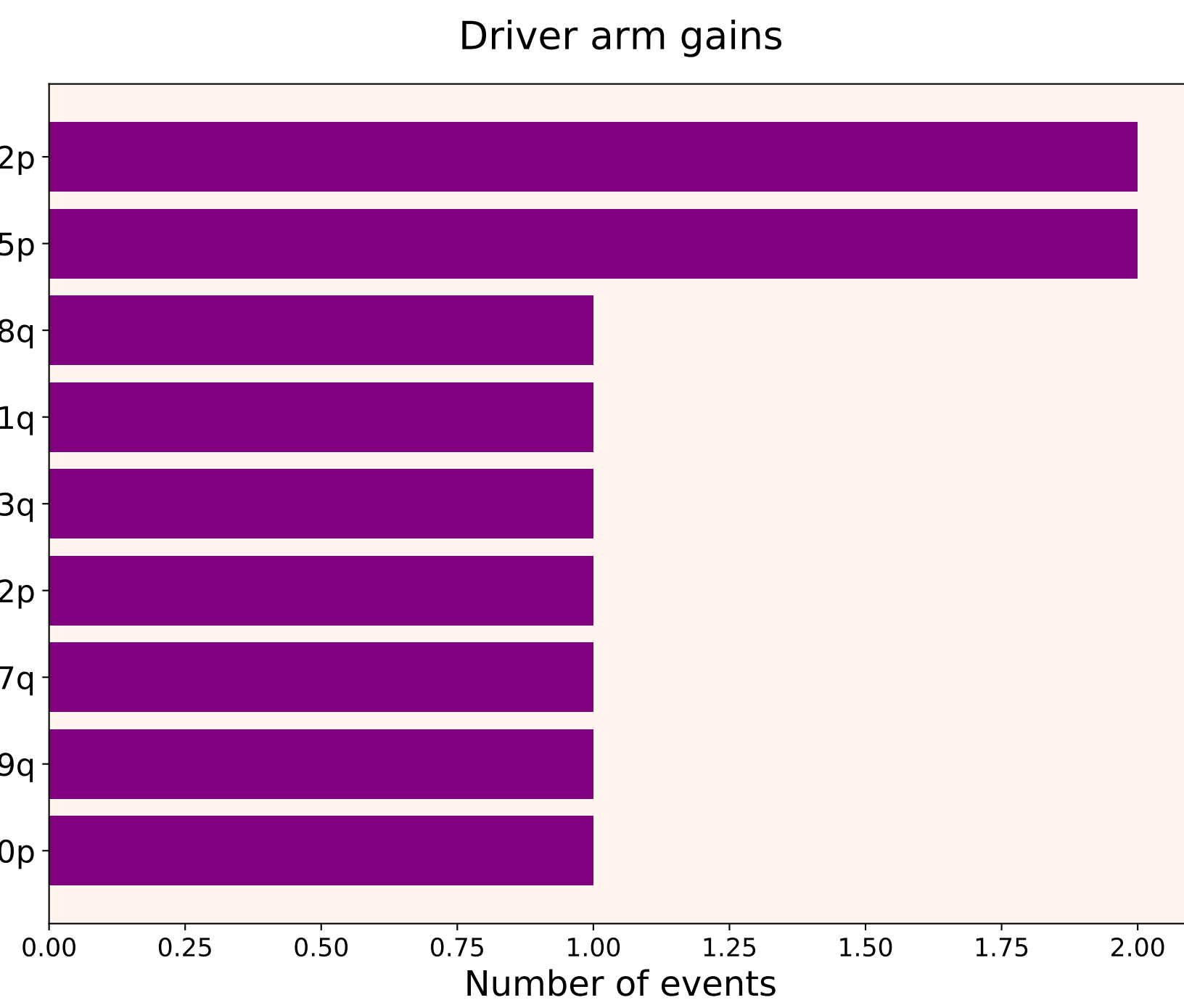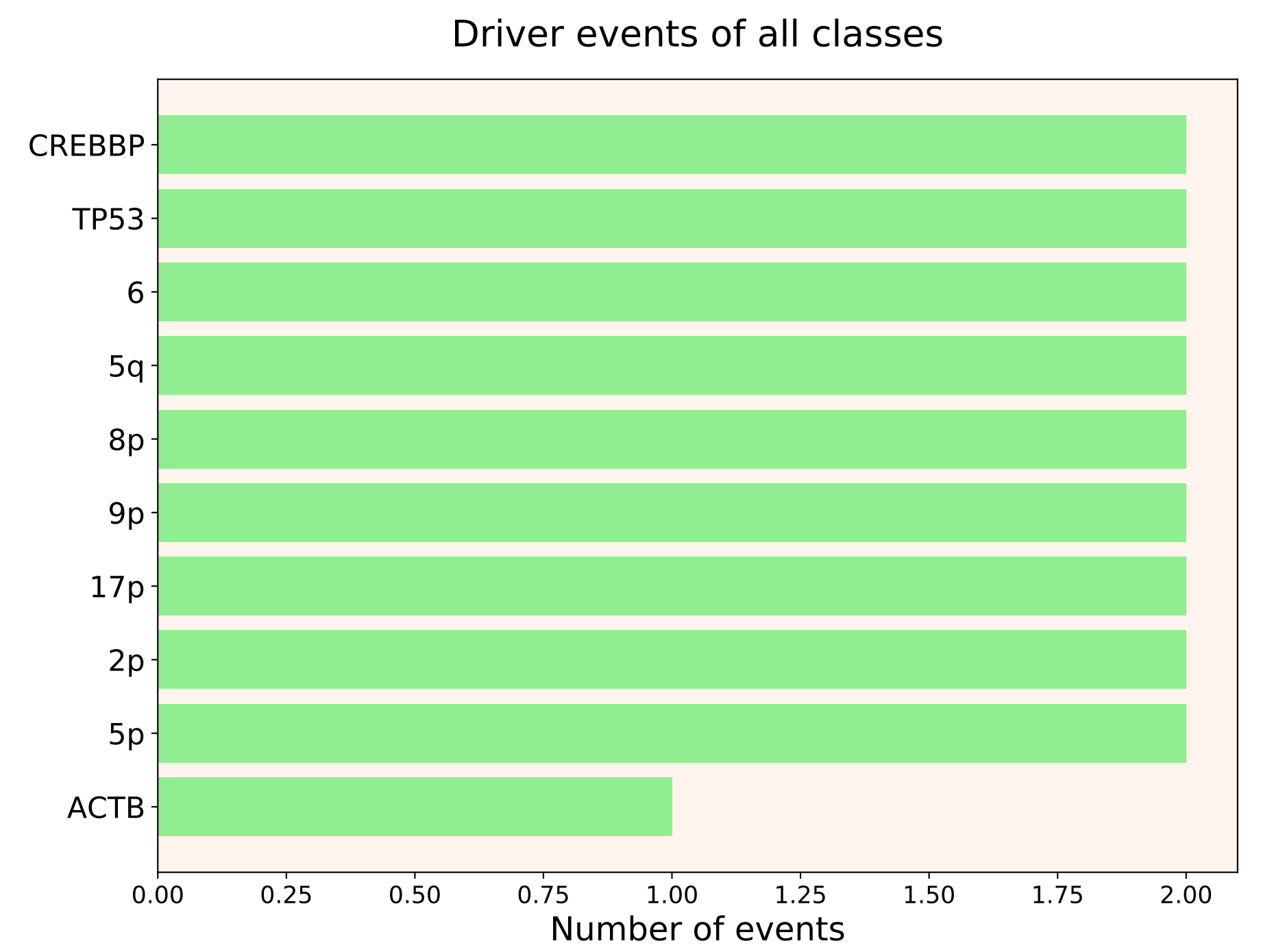

Supplement: Supplemental Information 2 [file peerj-10-13860-s002.zip › COHORTS/genes plots/detailed/2021_8_16_14_9_distribution_events_detailed_females_37.pdf]

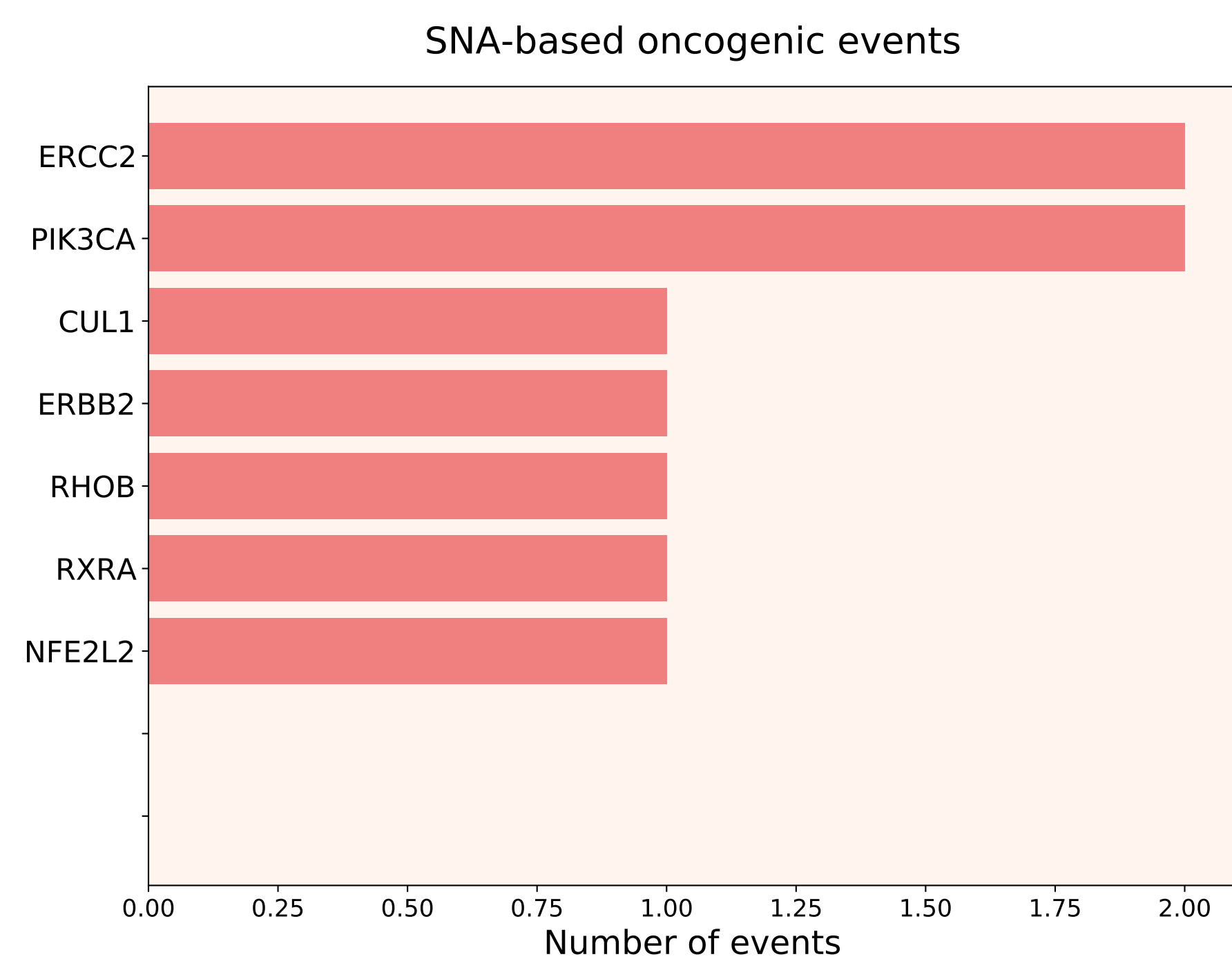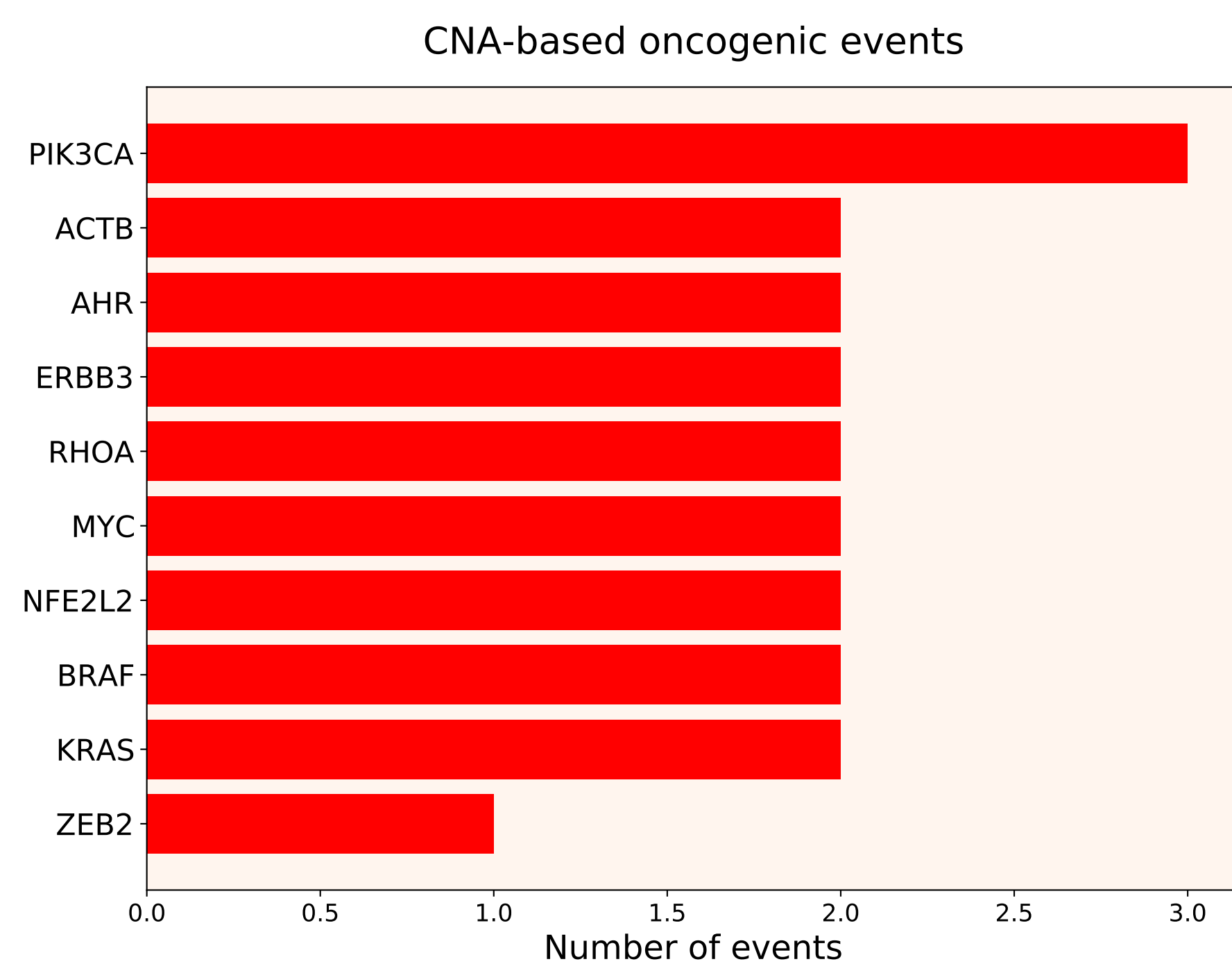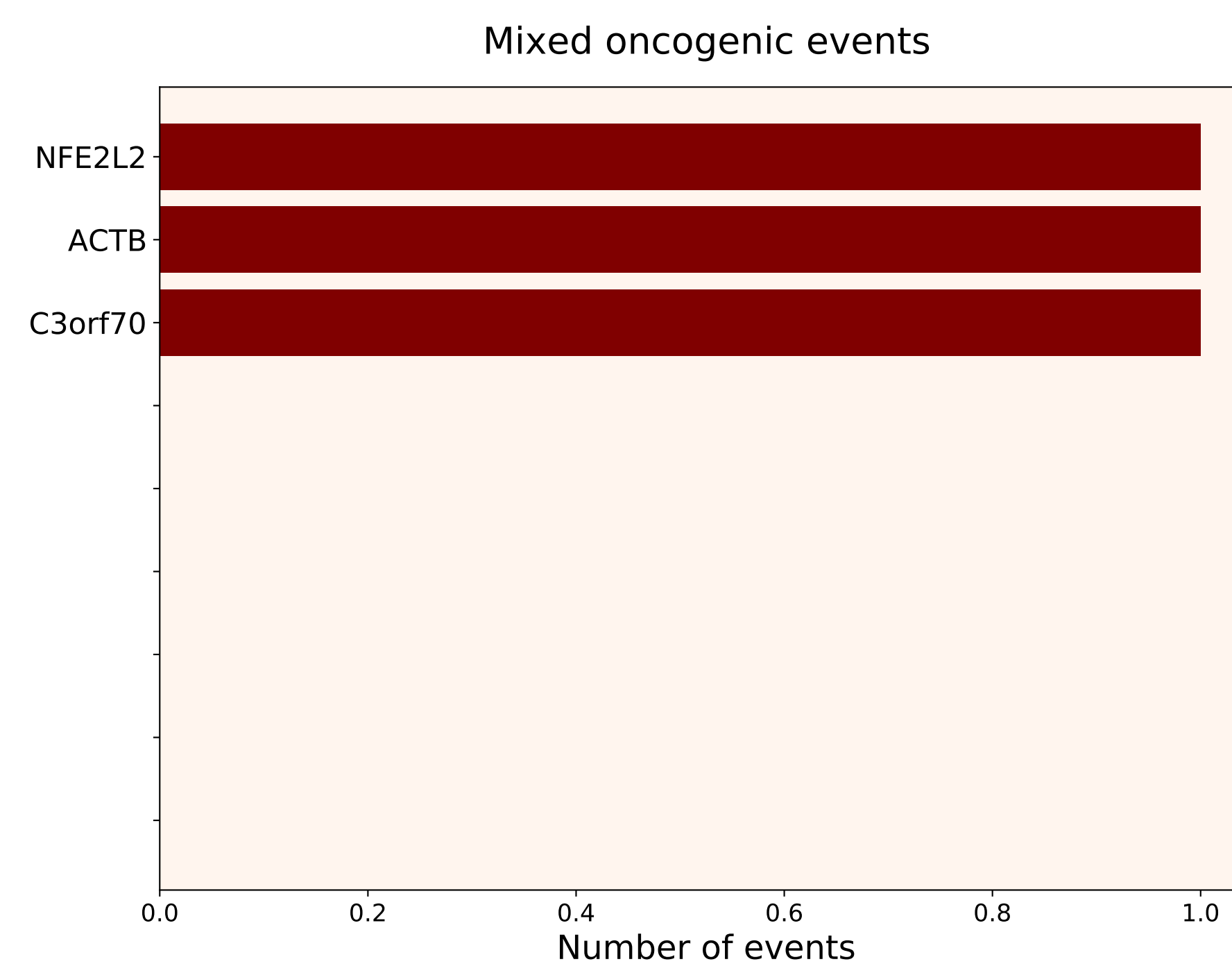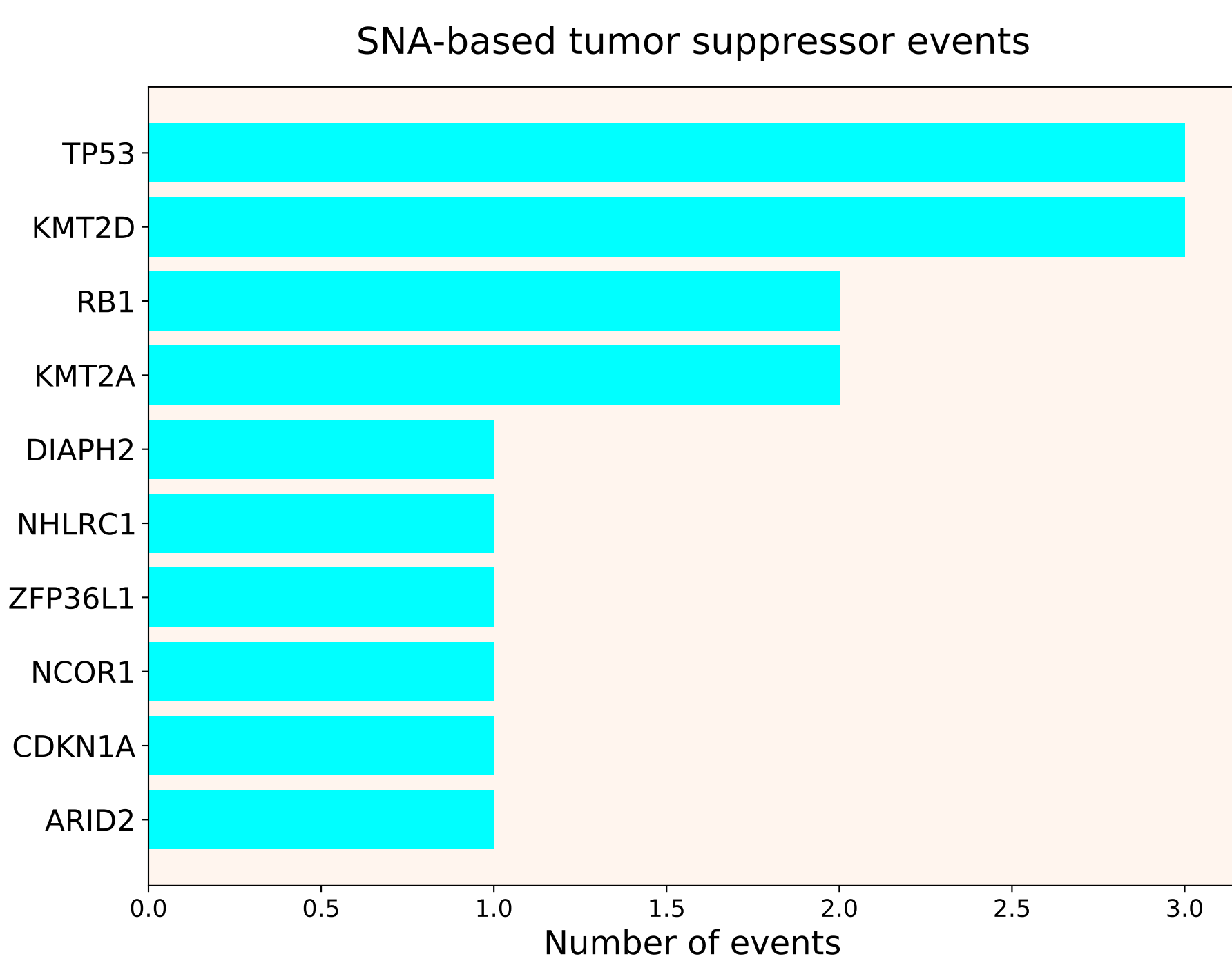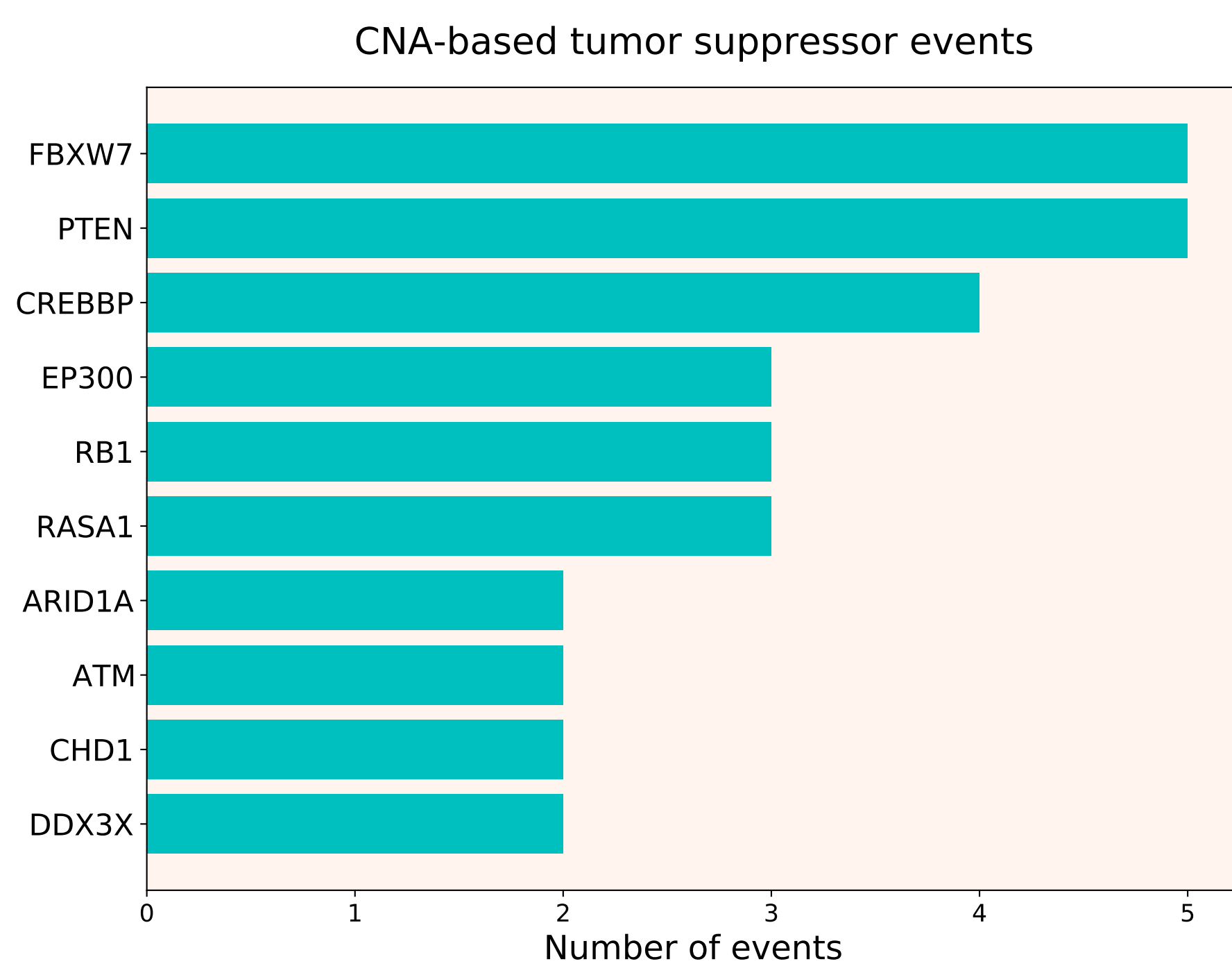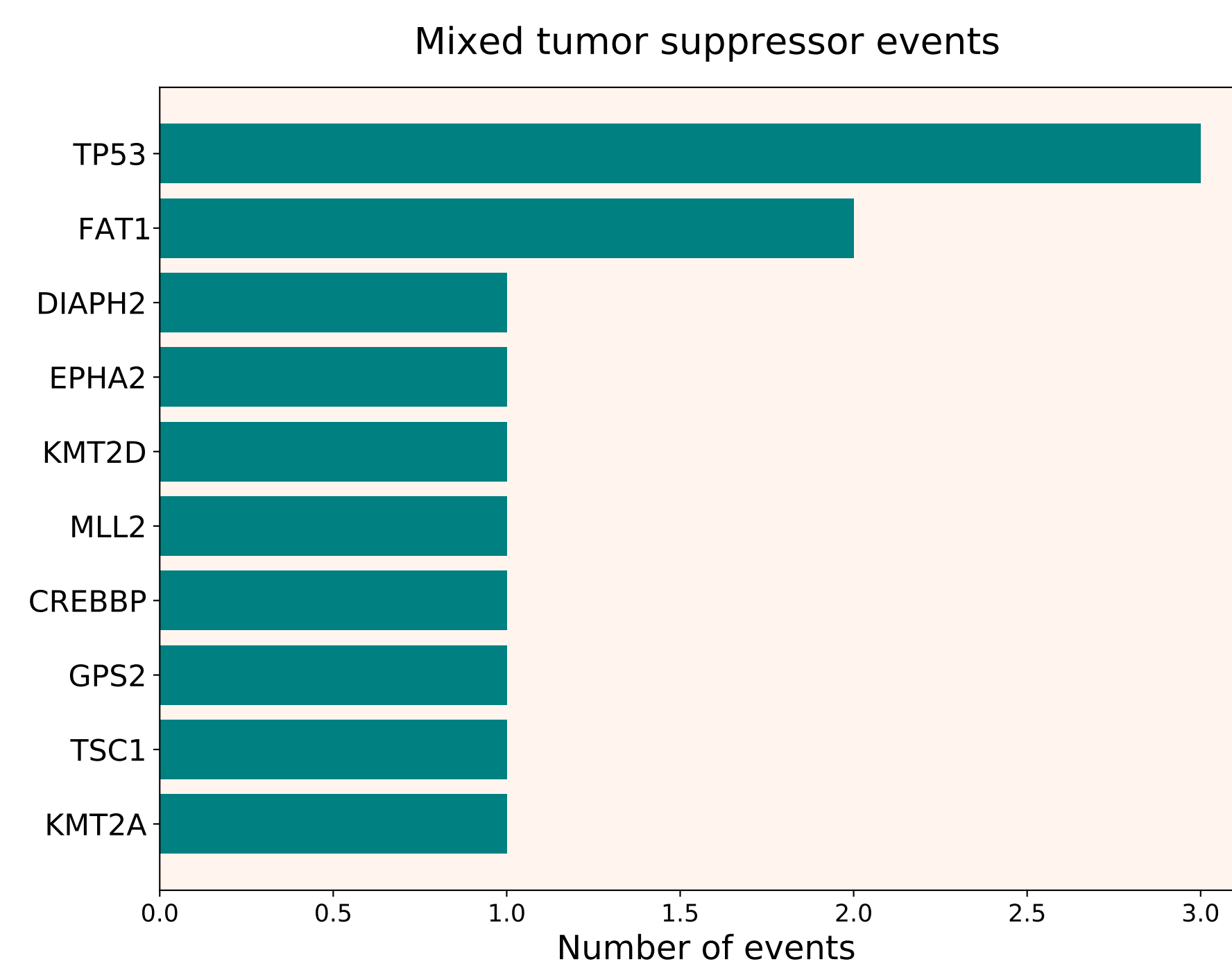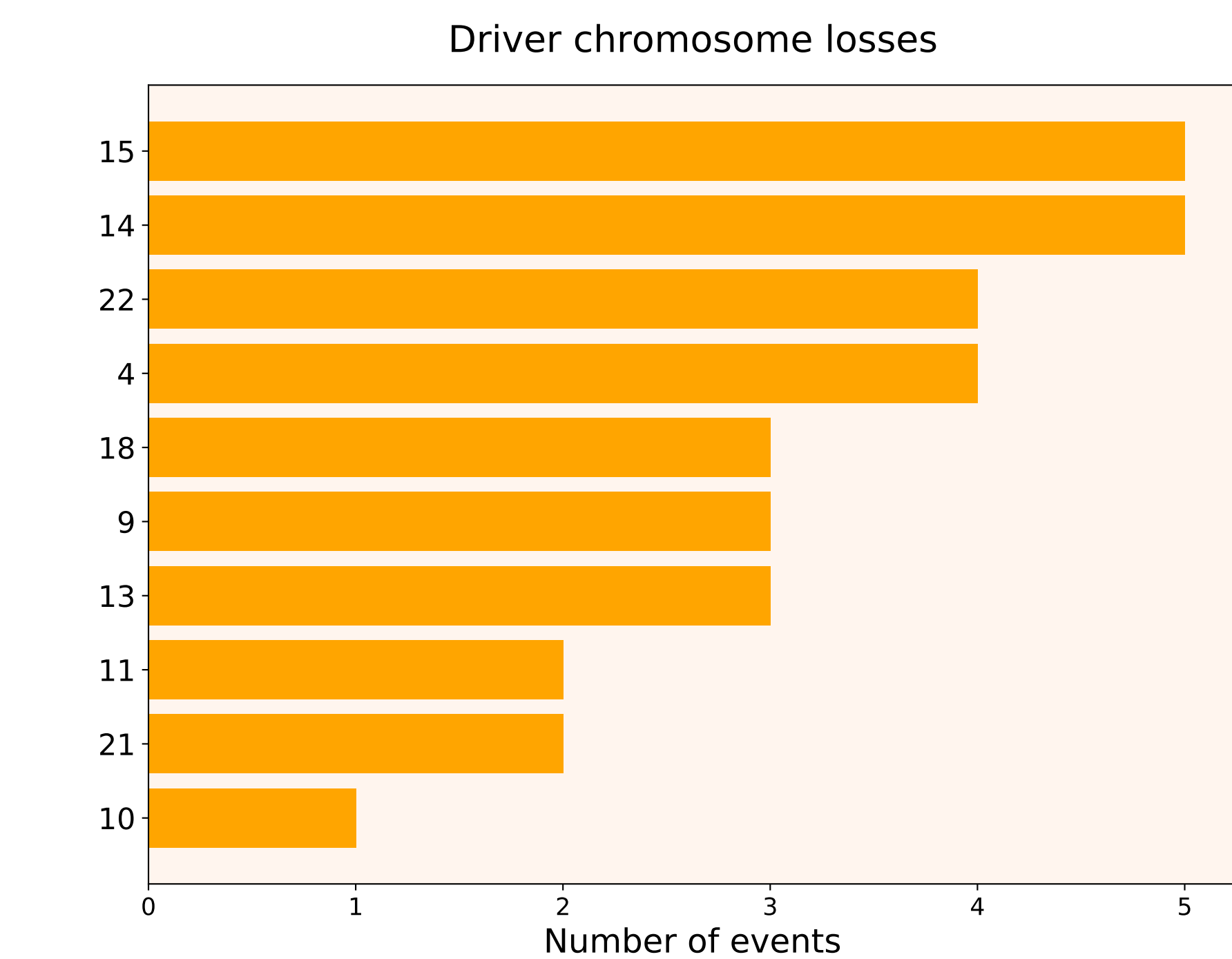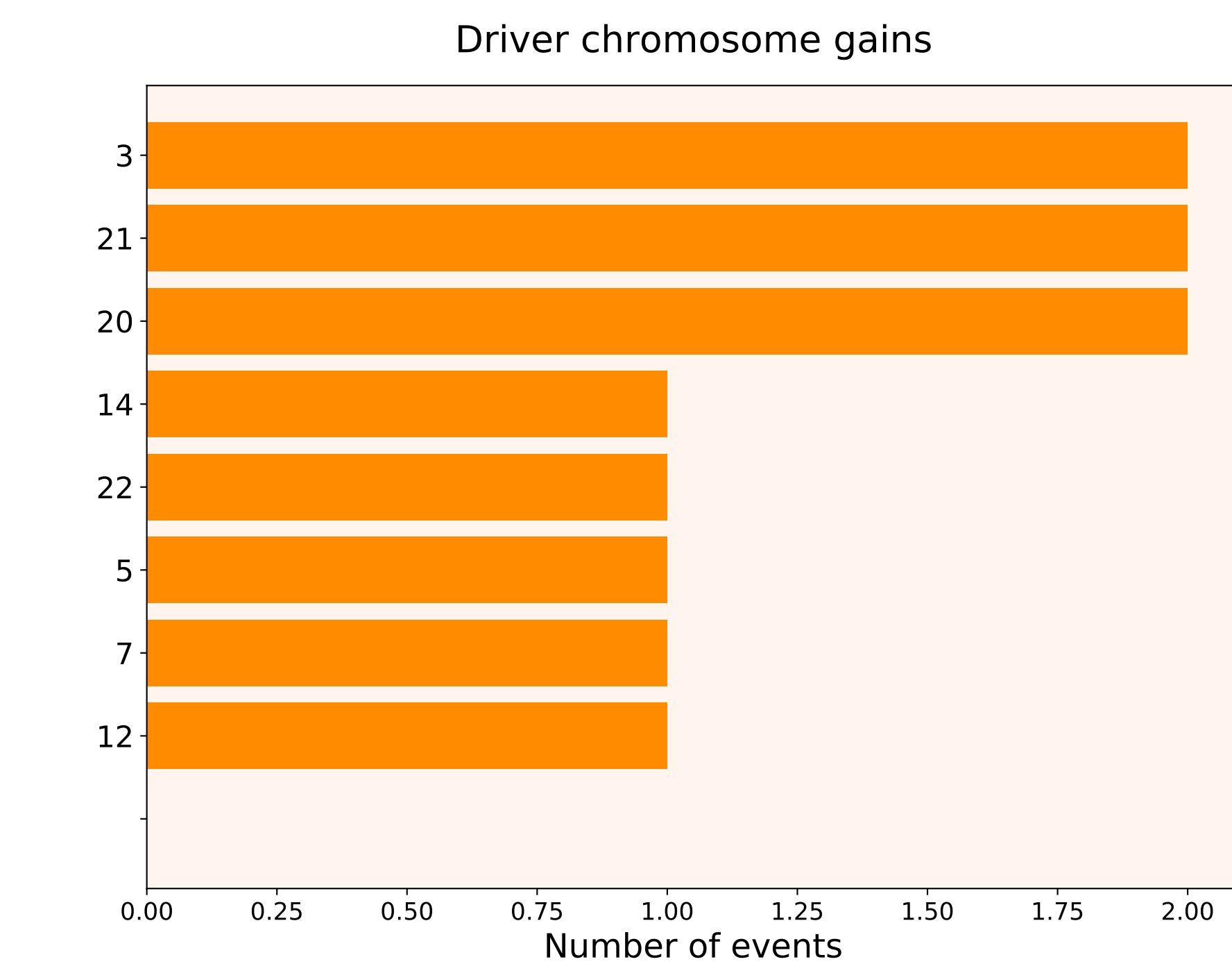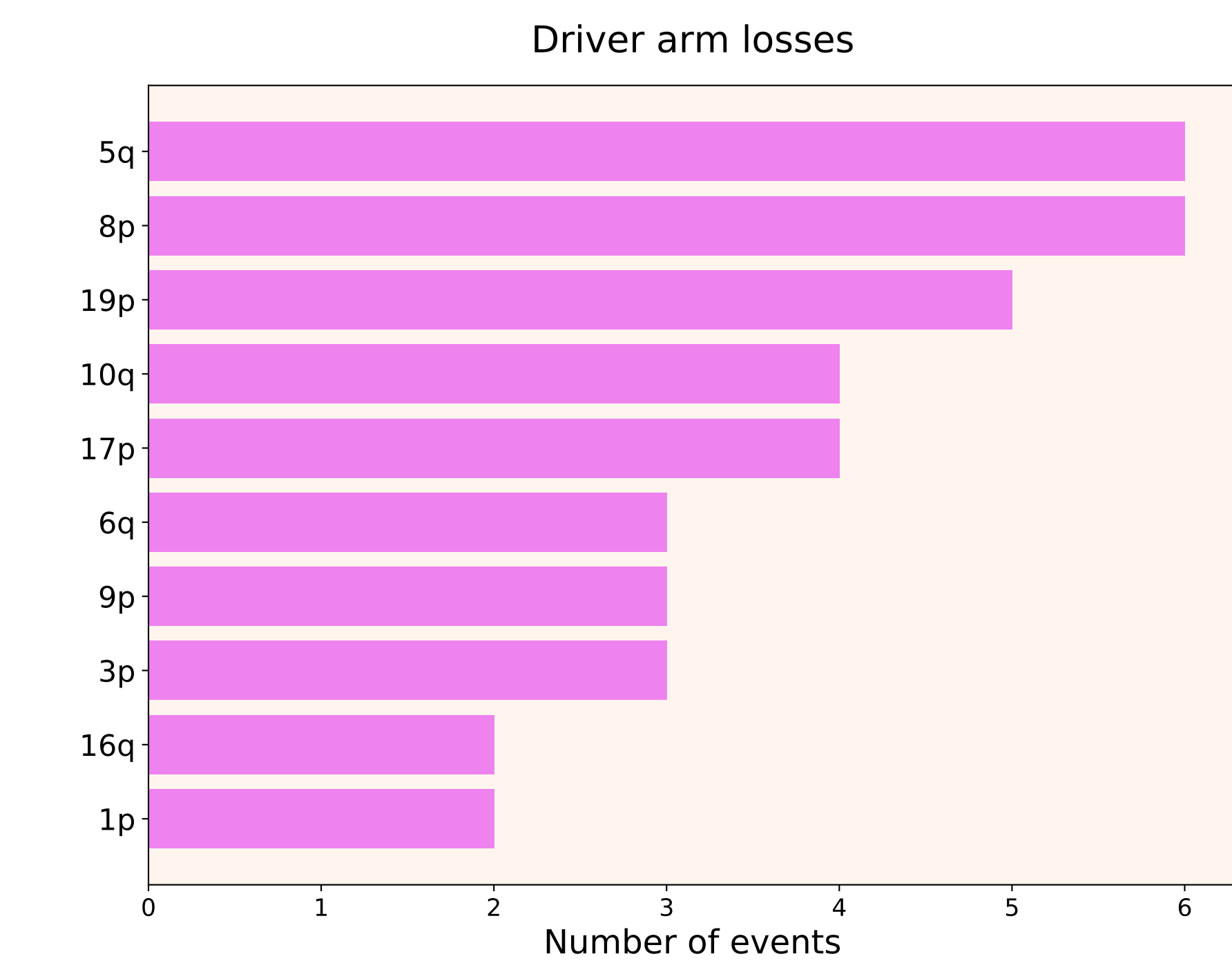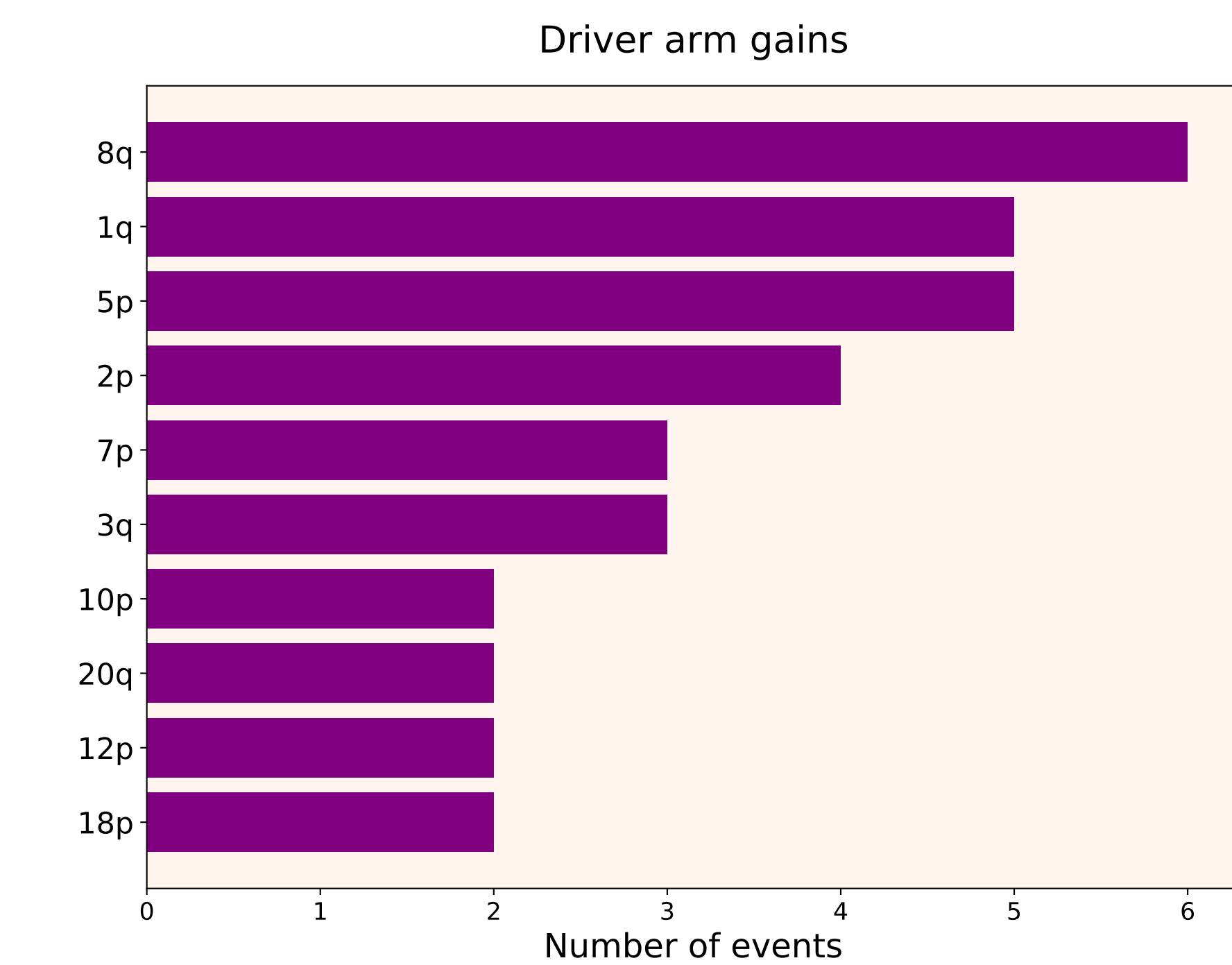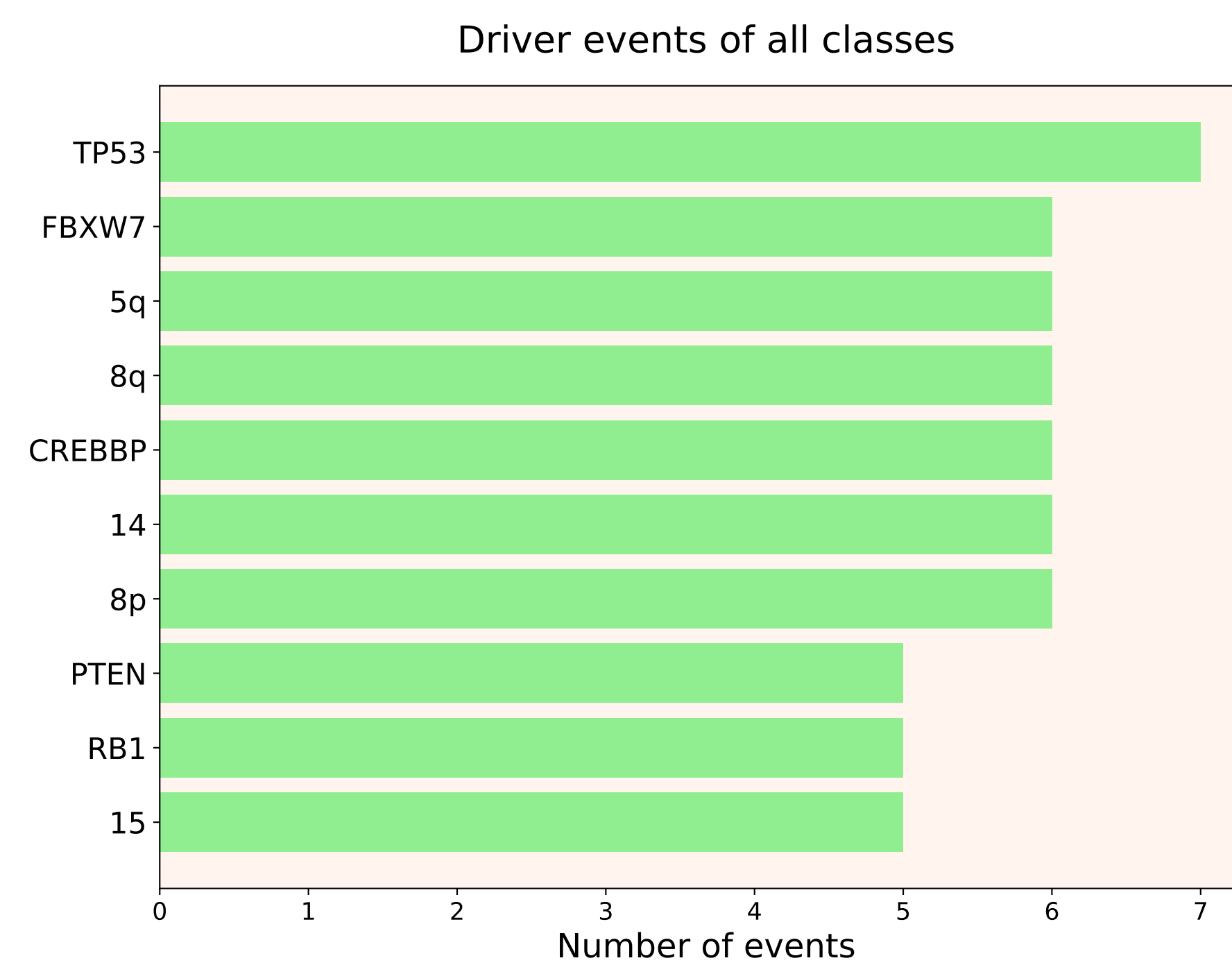

Supplement: Supplemental Information 2 [file peerj-10-13860-s002.zip › COHORTS/genes plots/detailed/2021_8_16_14_9_distribution_events_detailed_males_33.pdf]

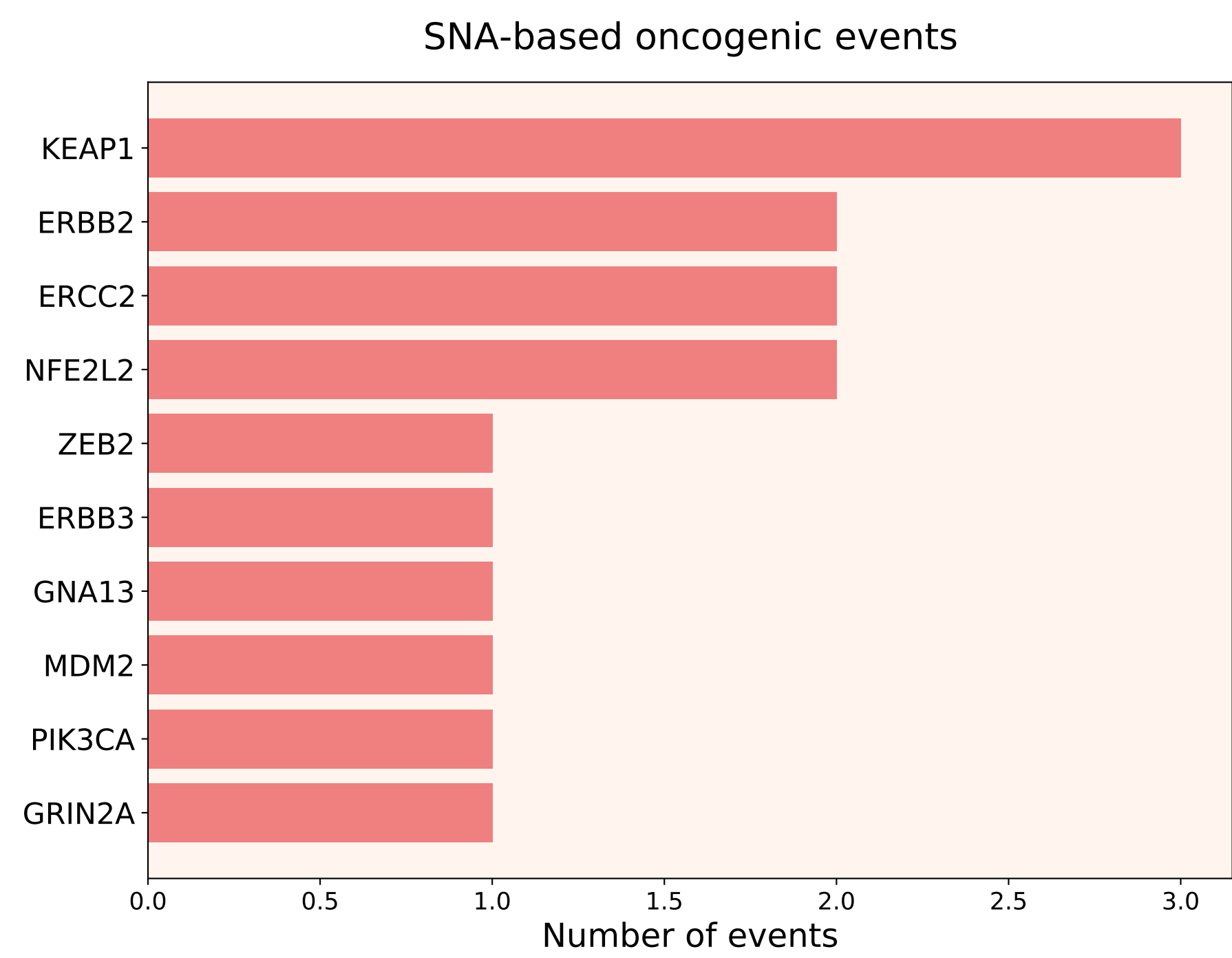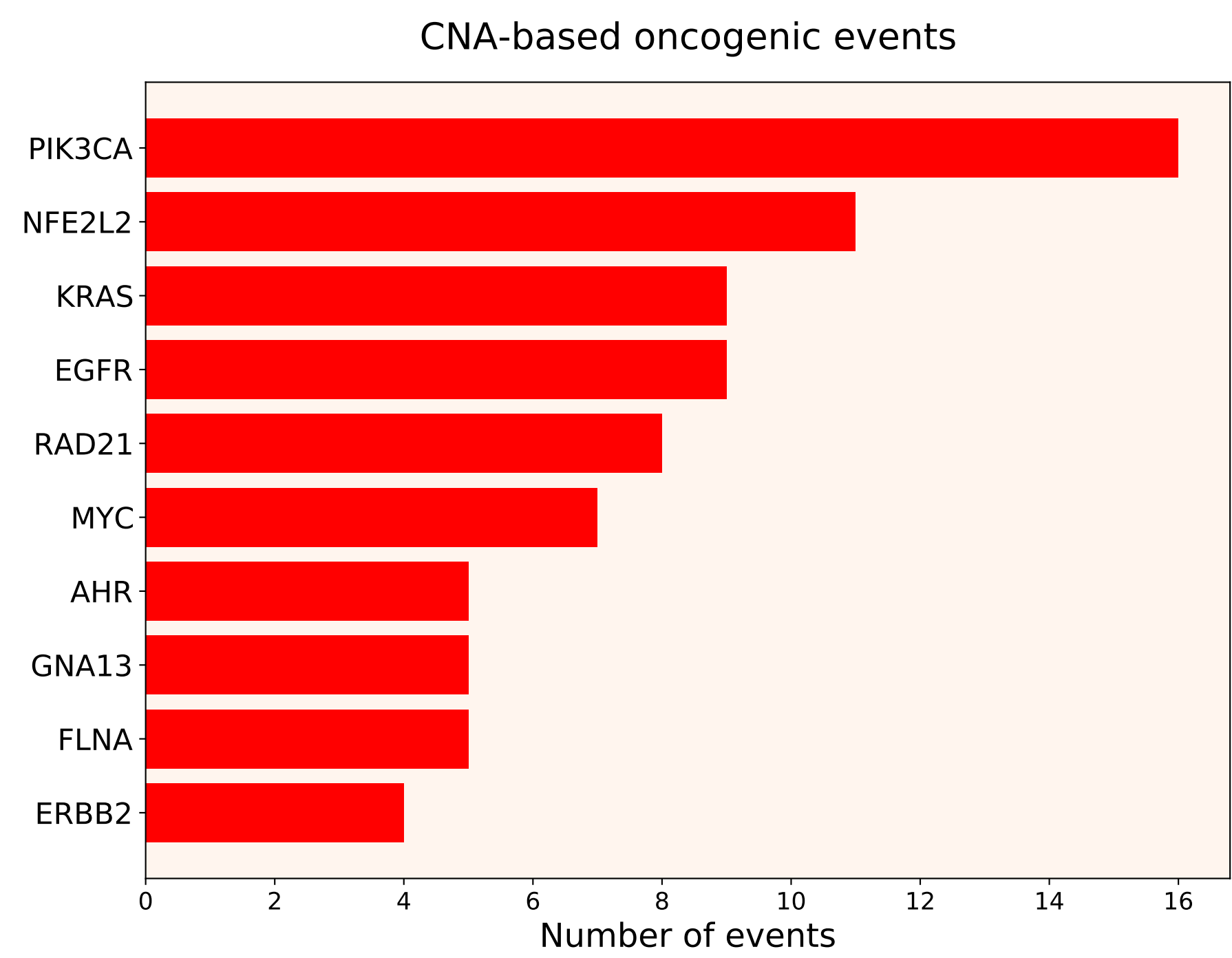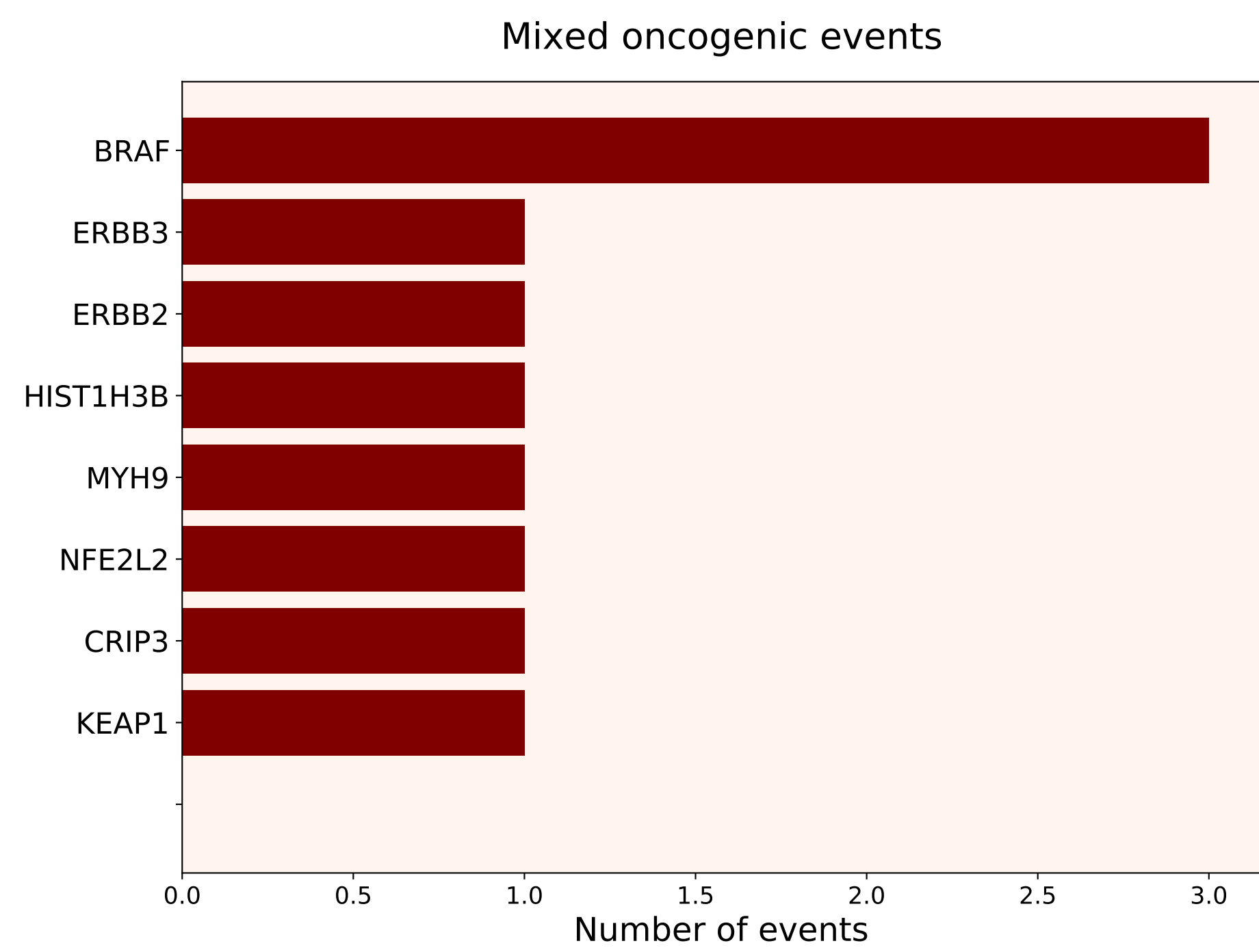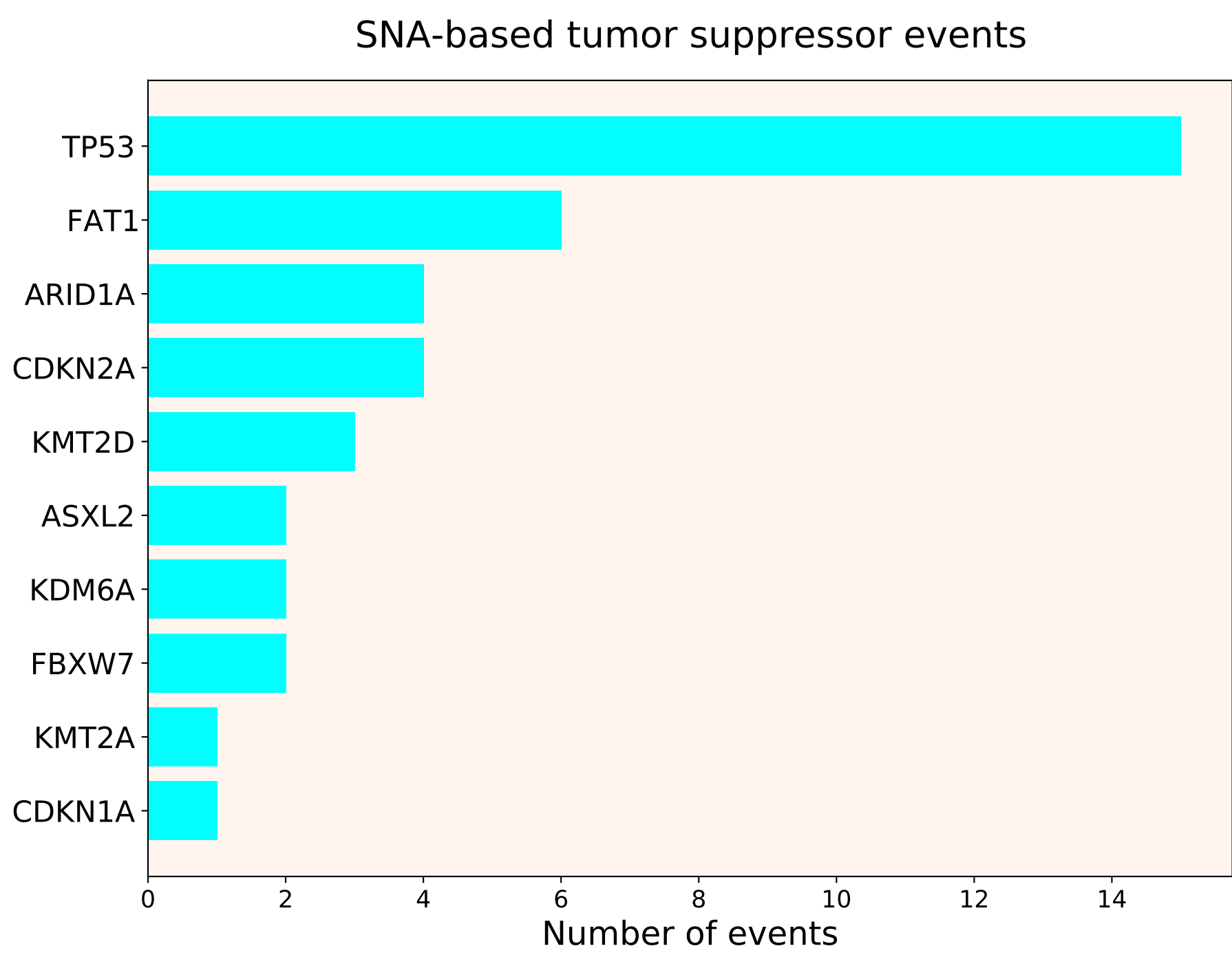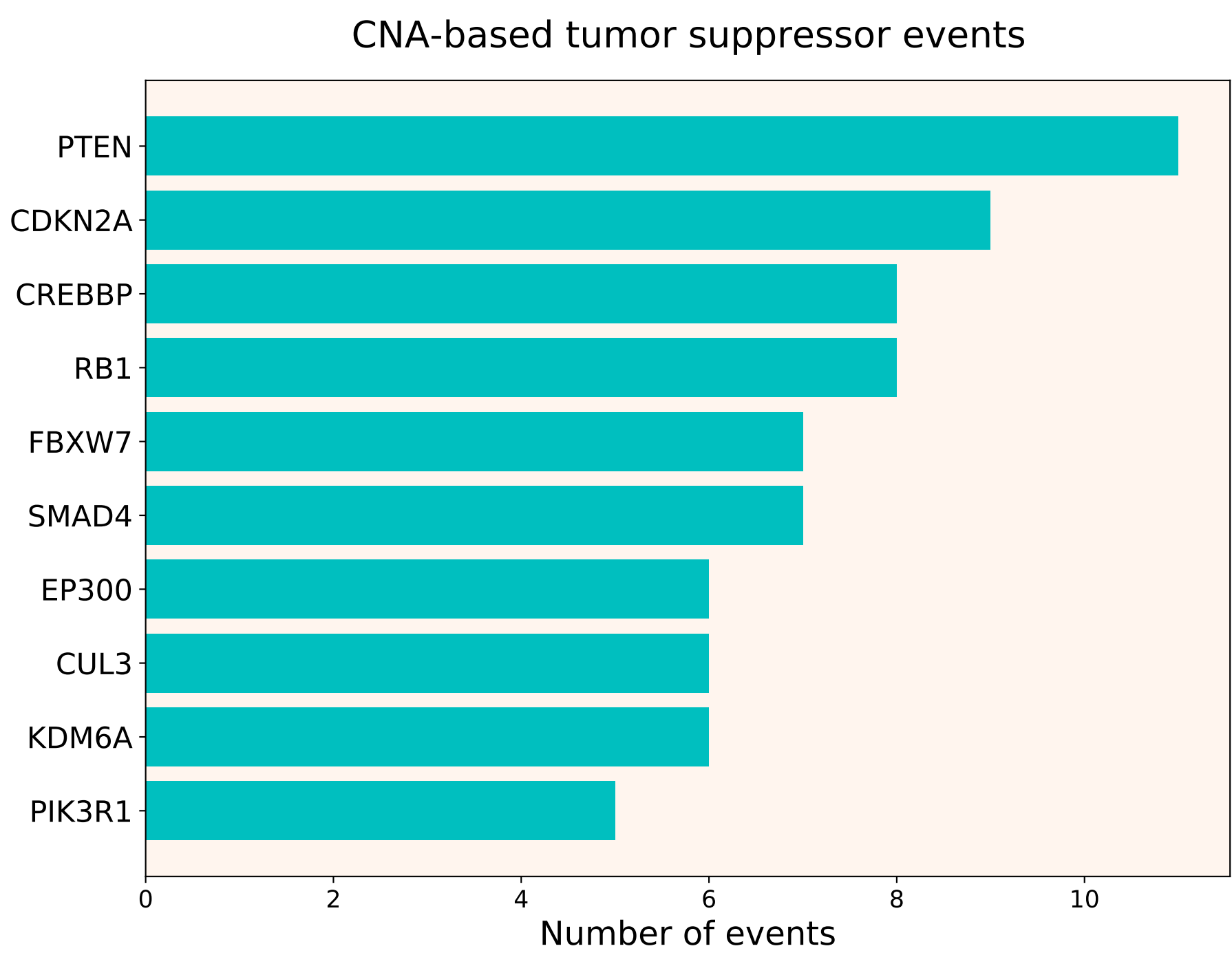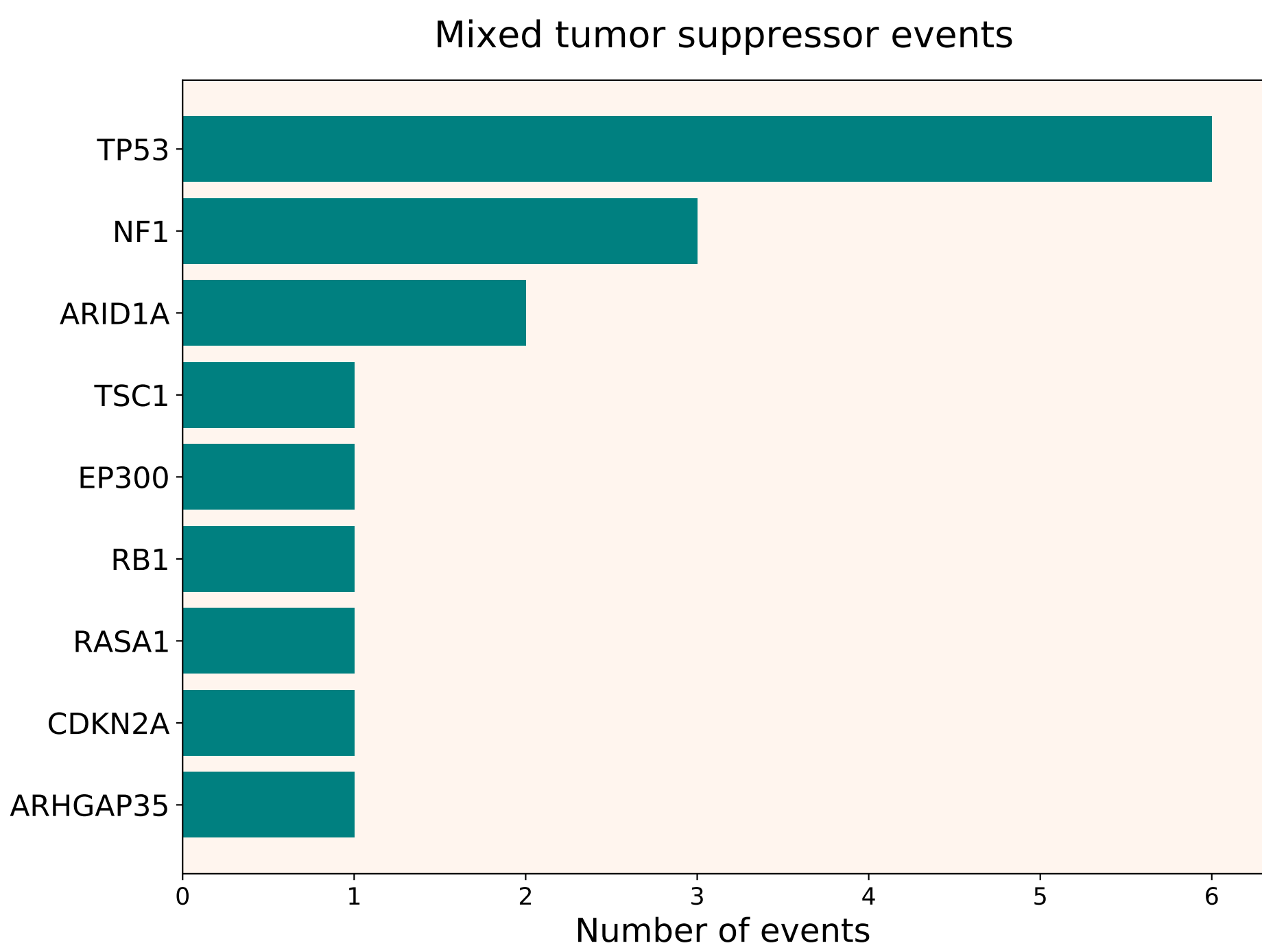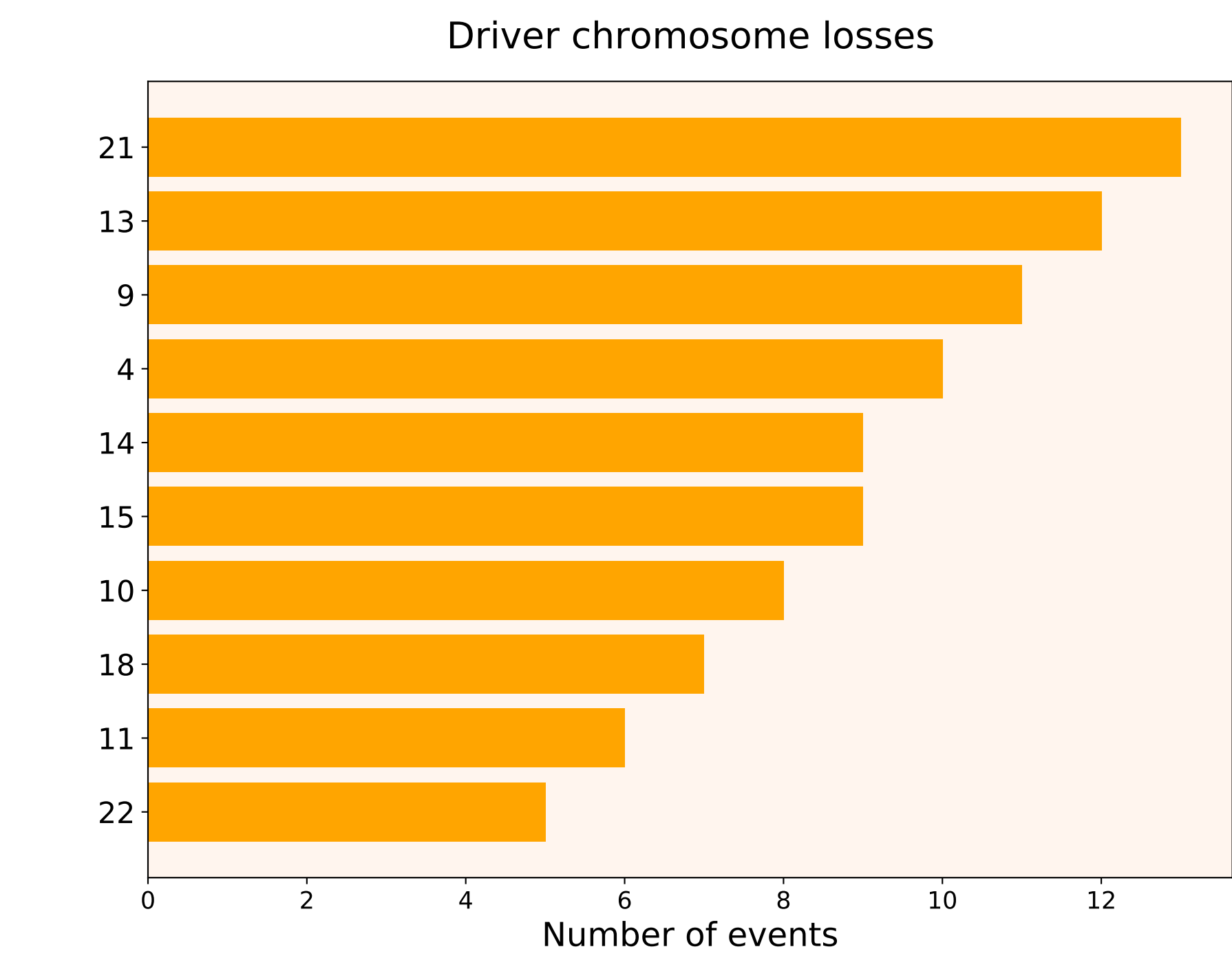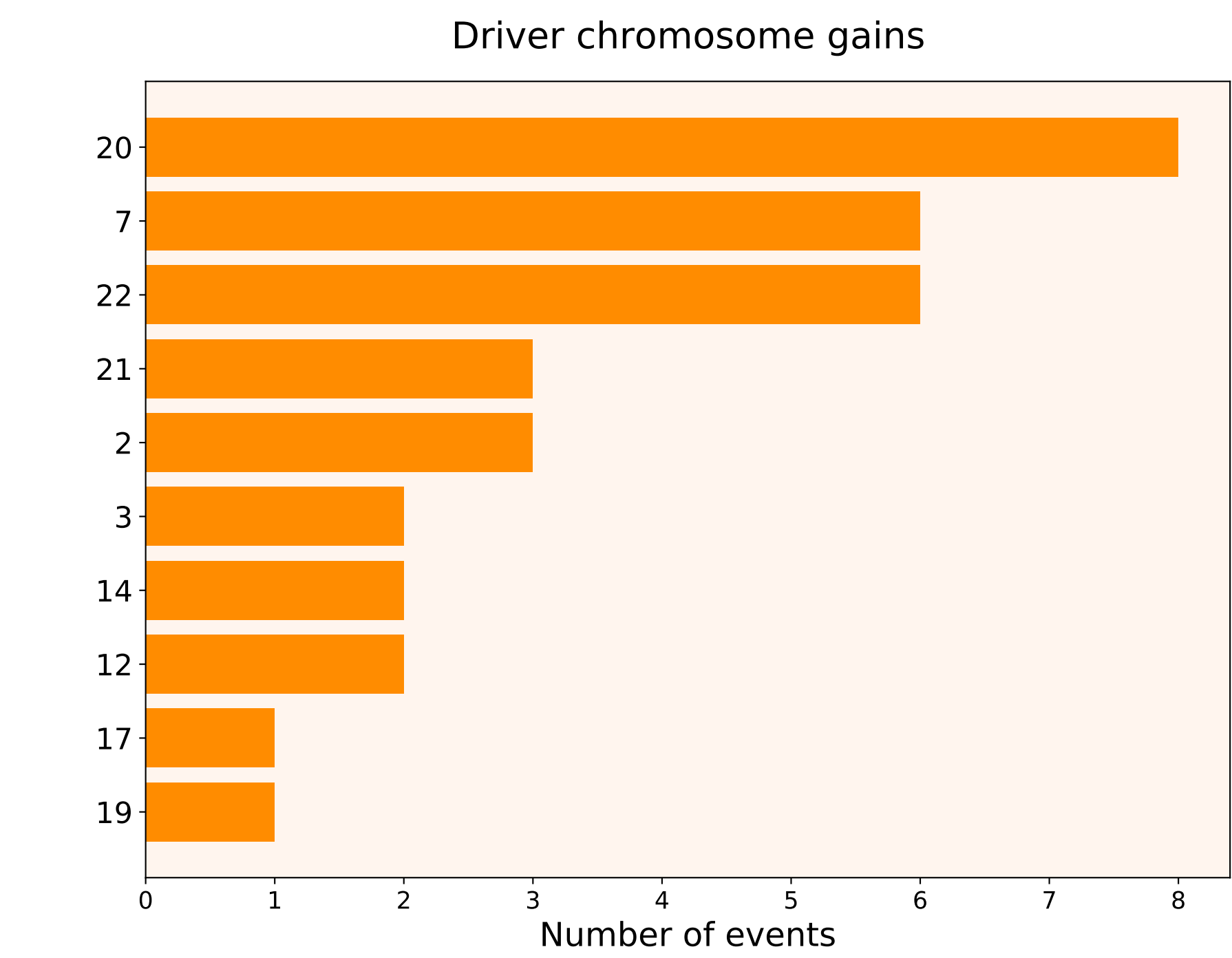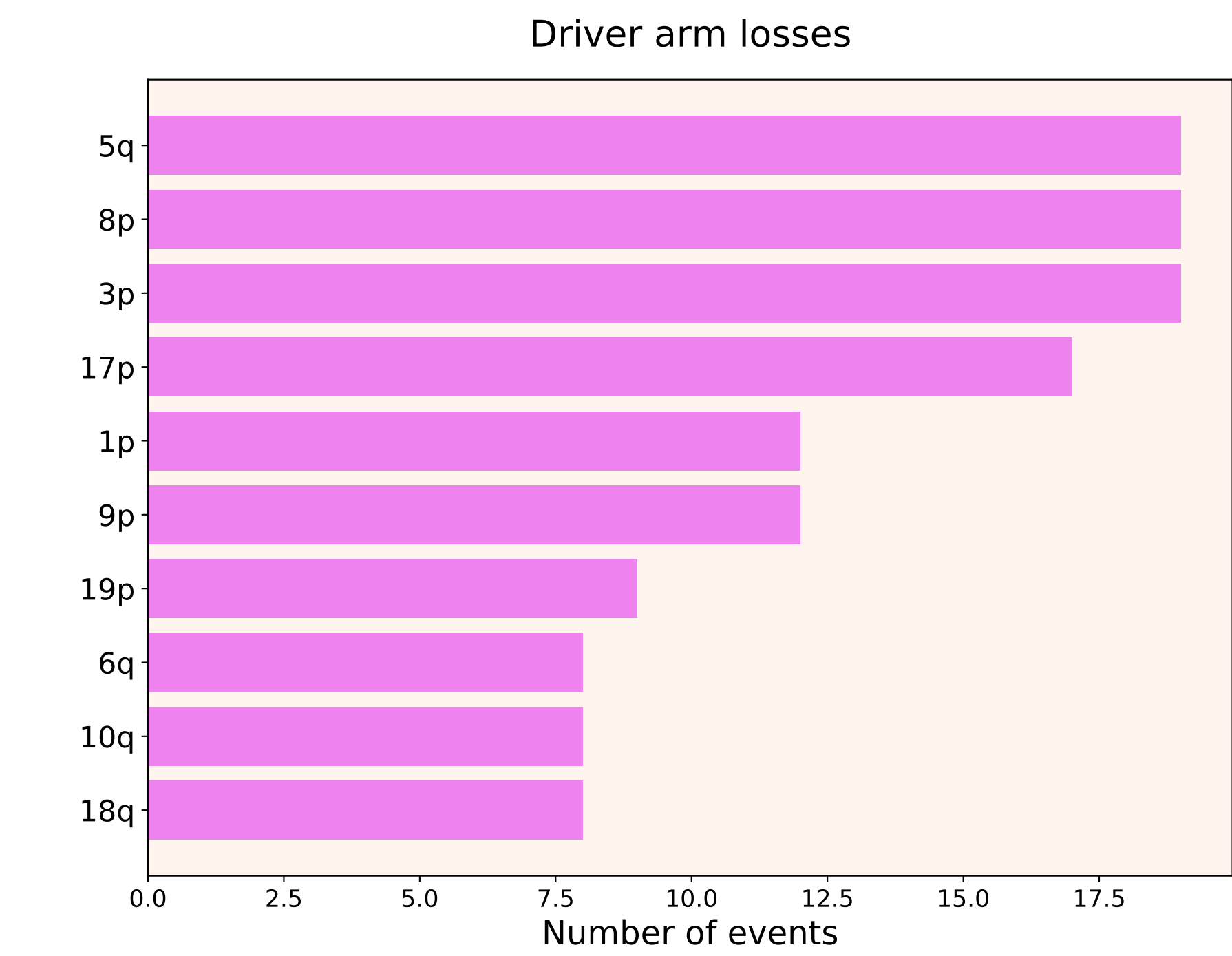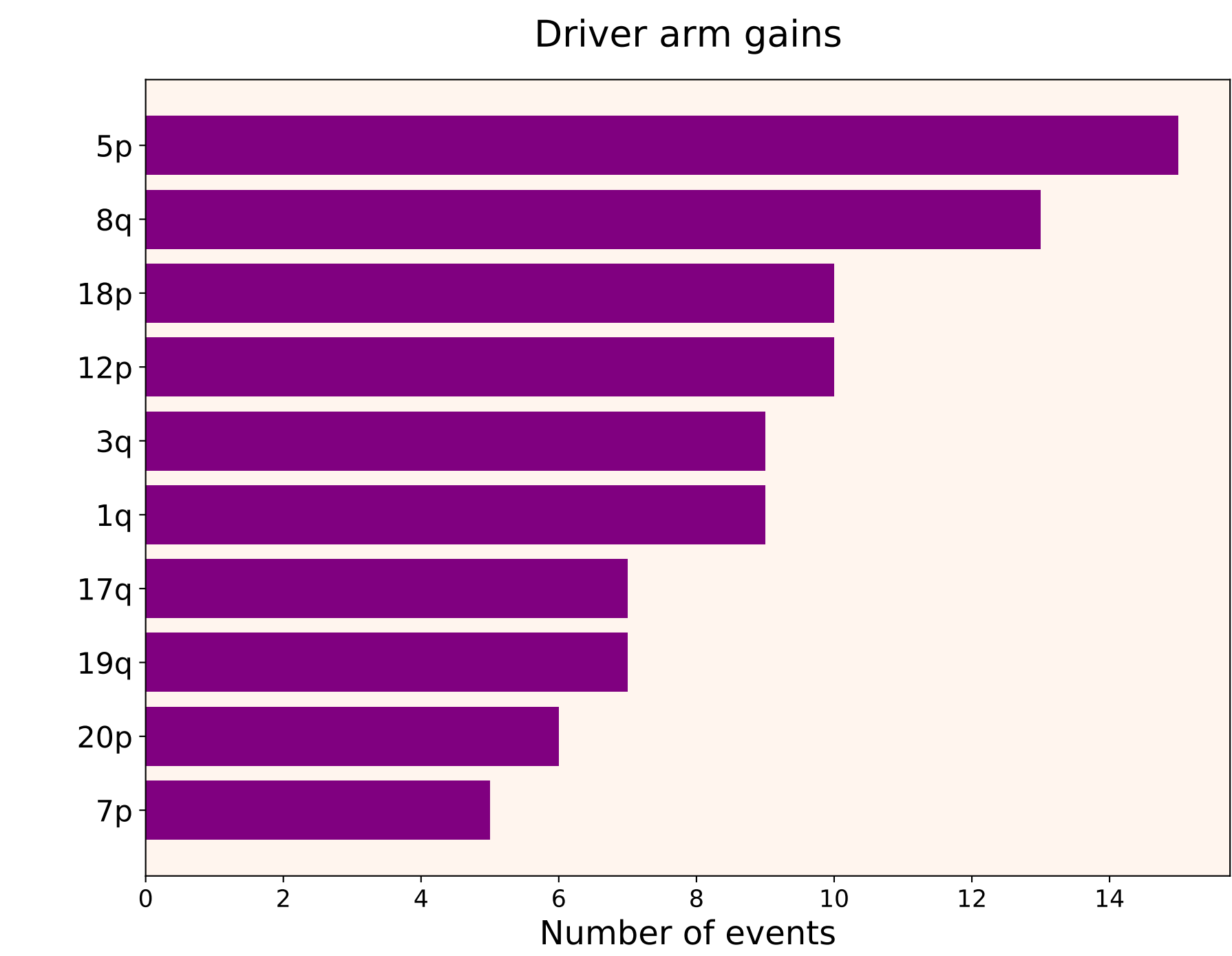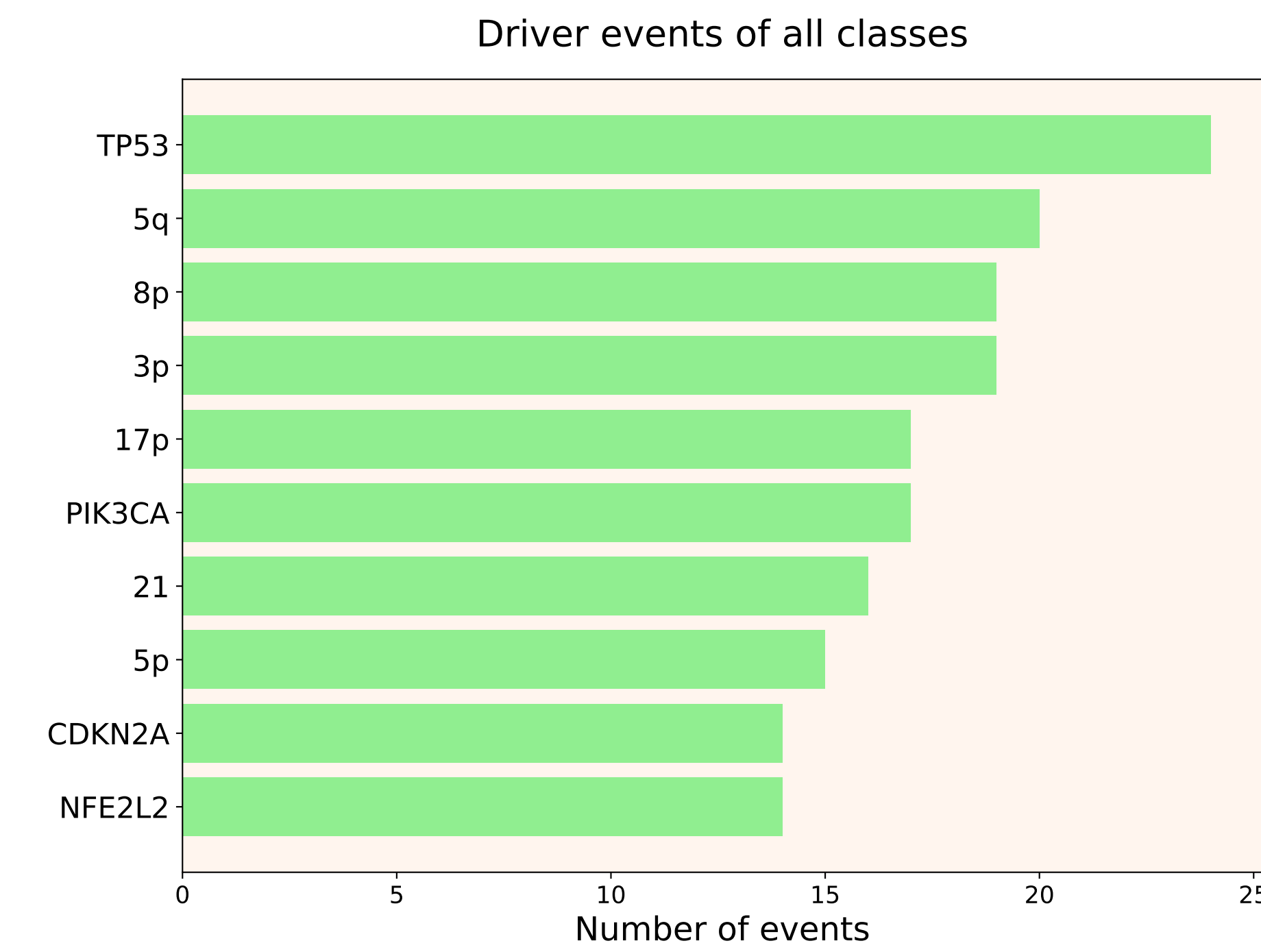

Supplement: Supplemental Information 2 [file peerj-10-13860-s002.zip › COHORTS/genes plots/detailed/2021_8_16_14_9_distribution_events_detailed_males_27.pdf]

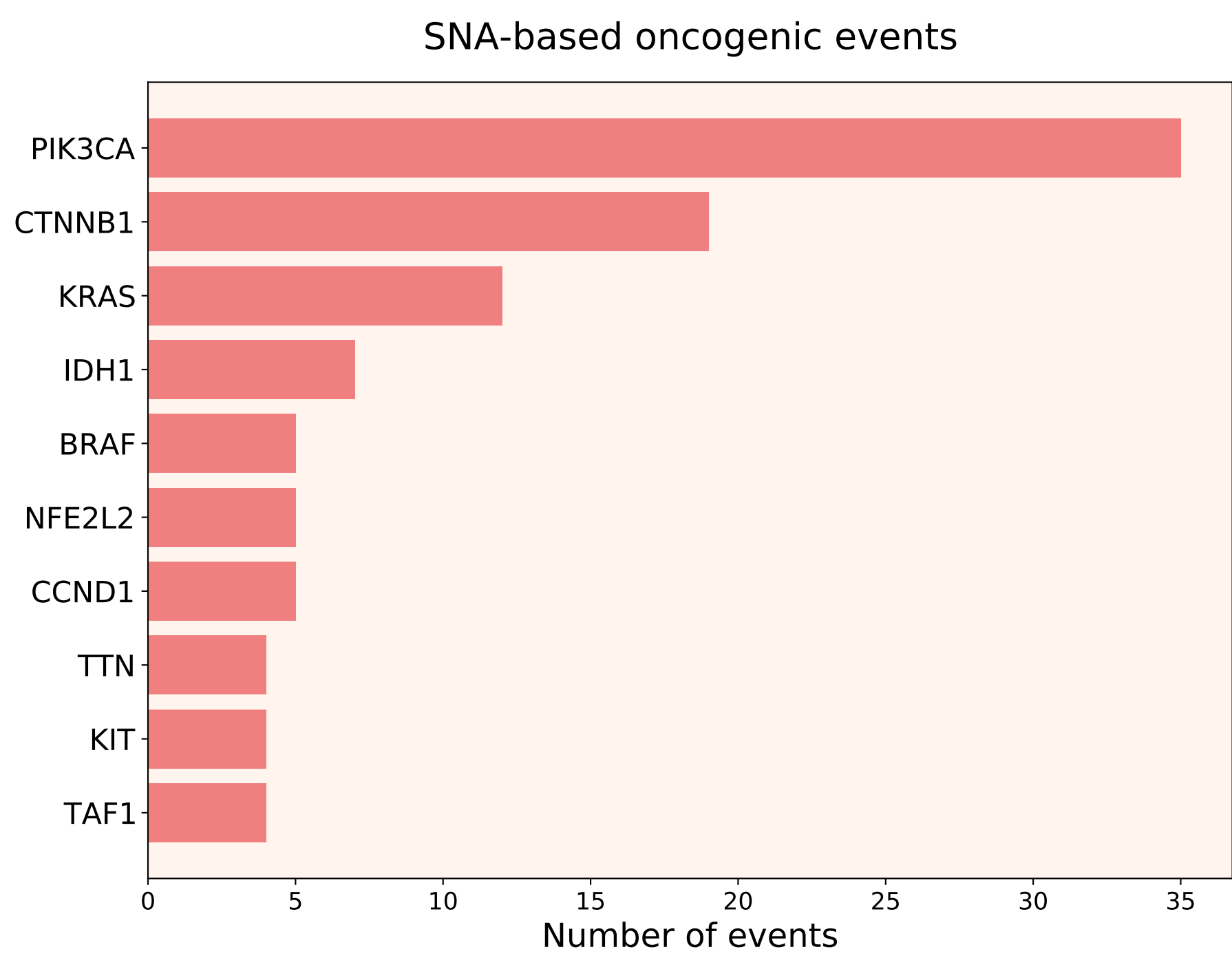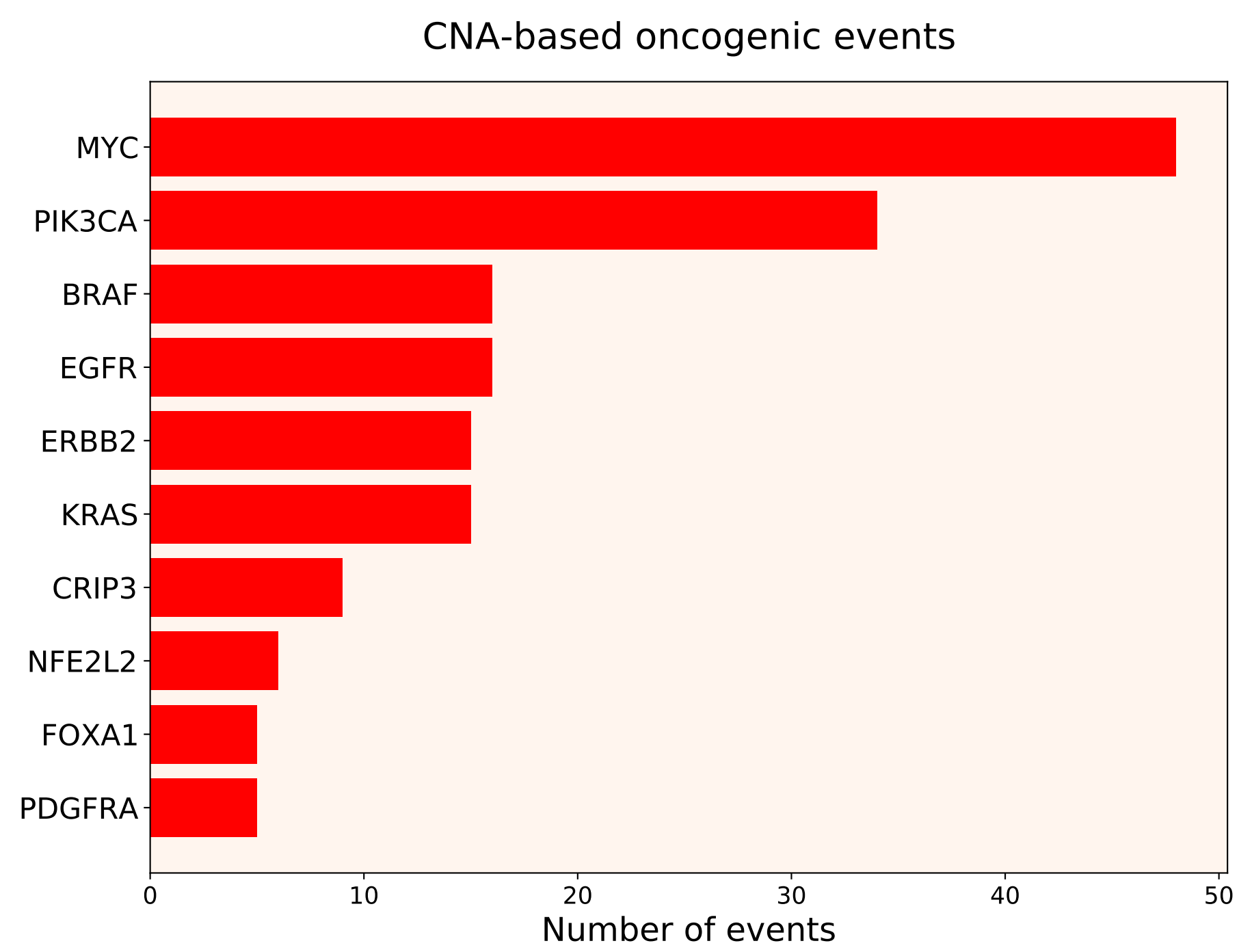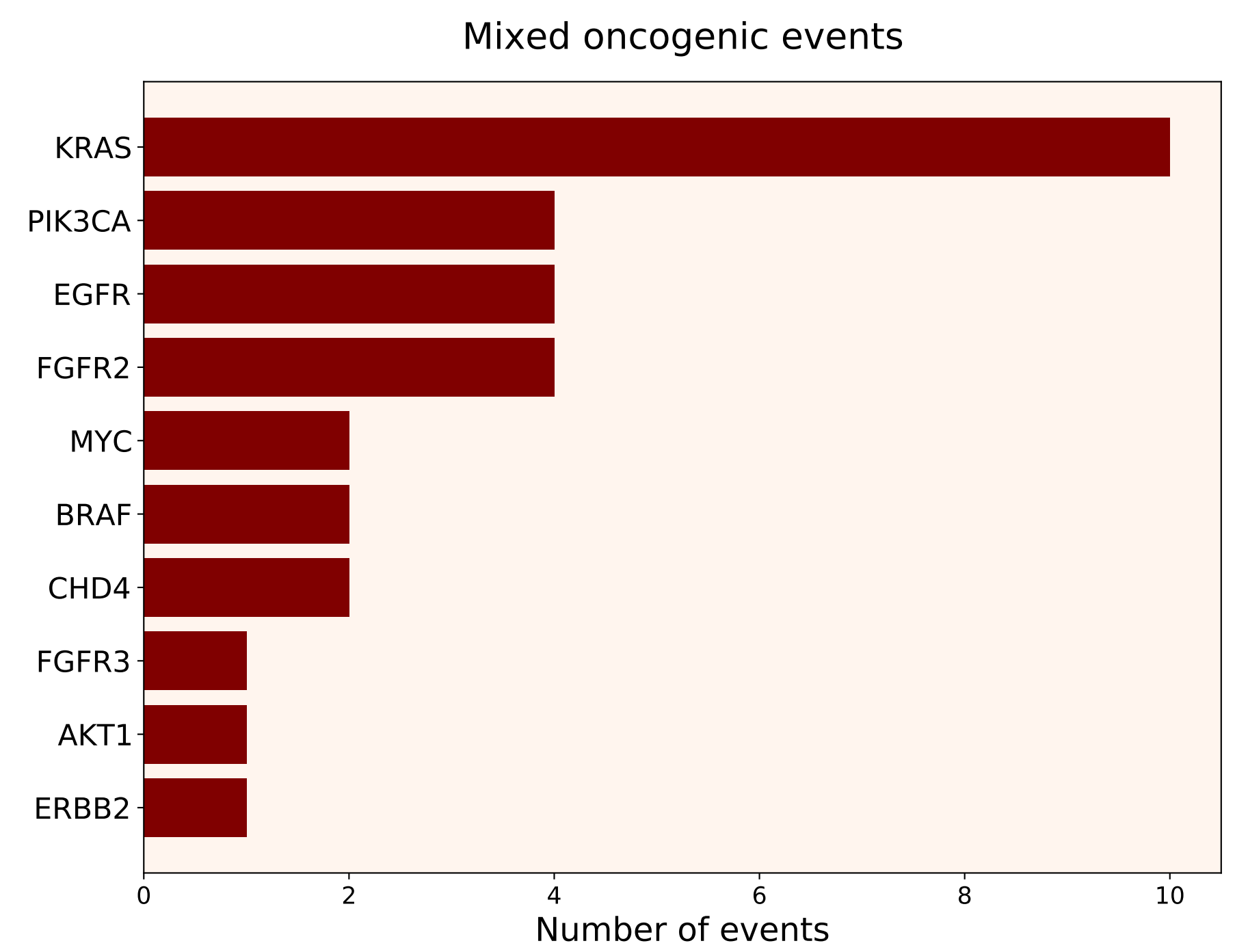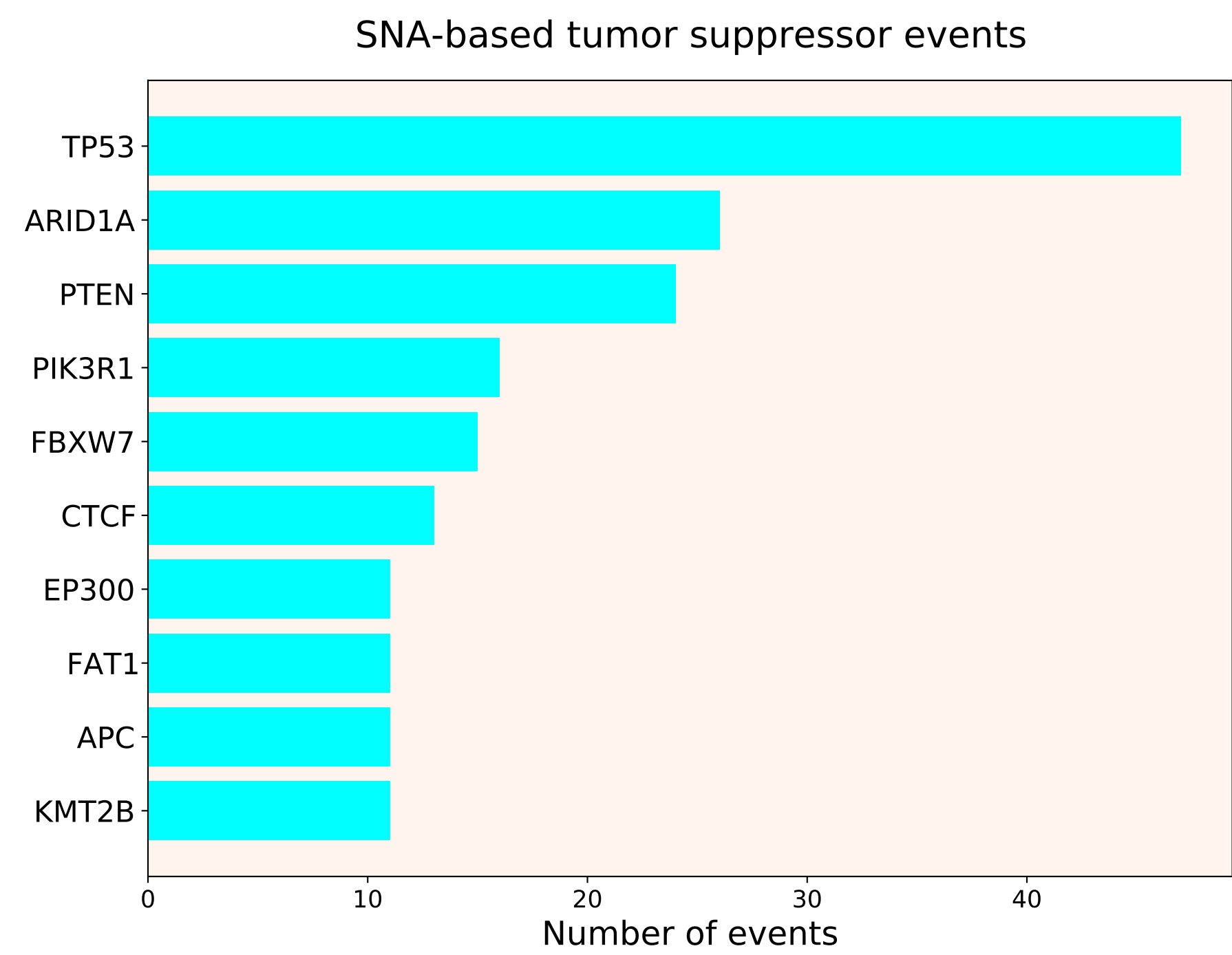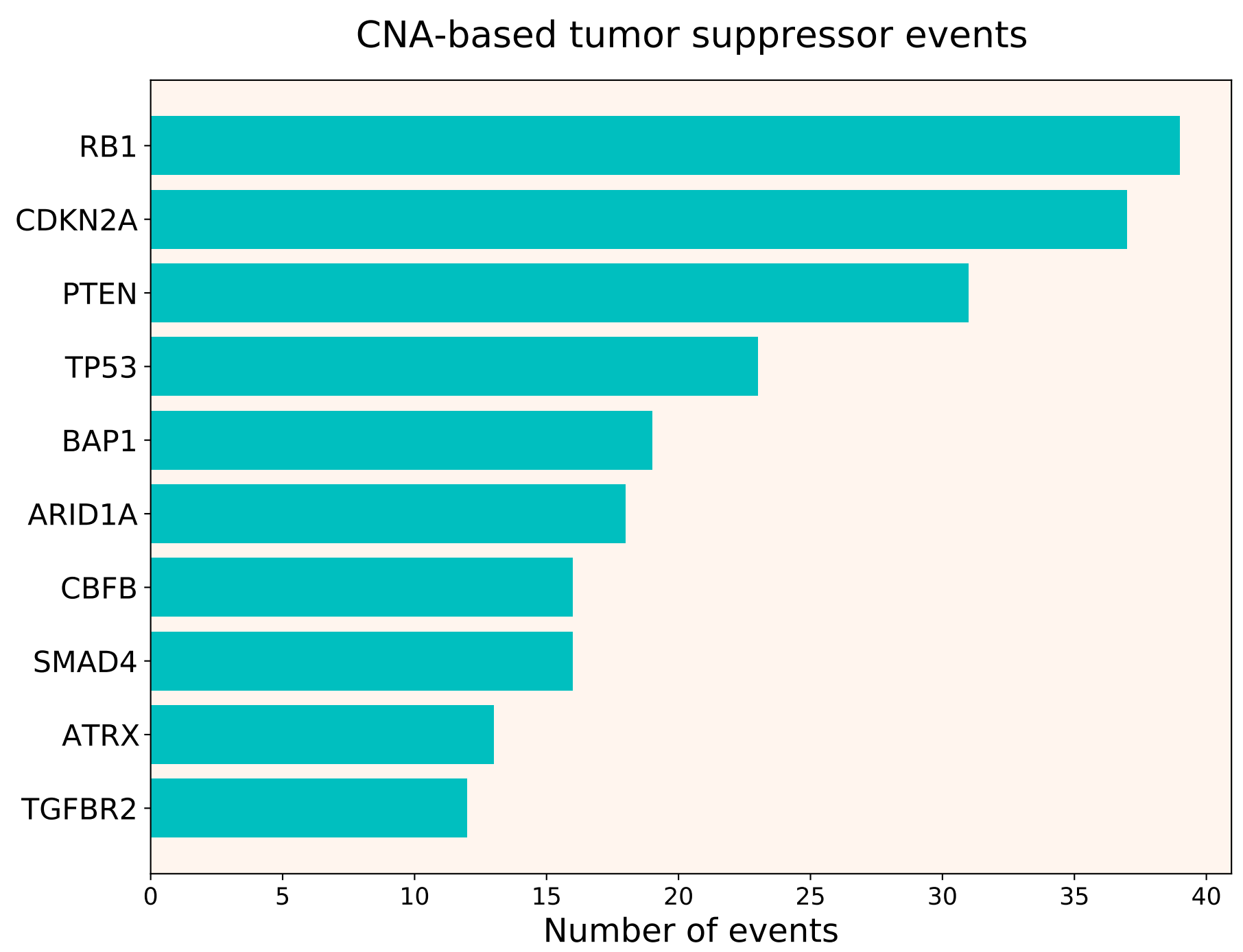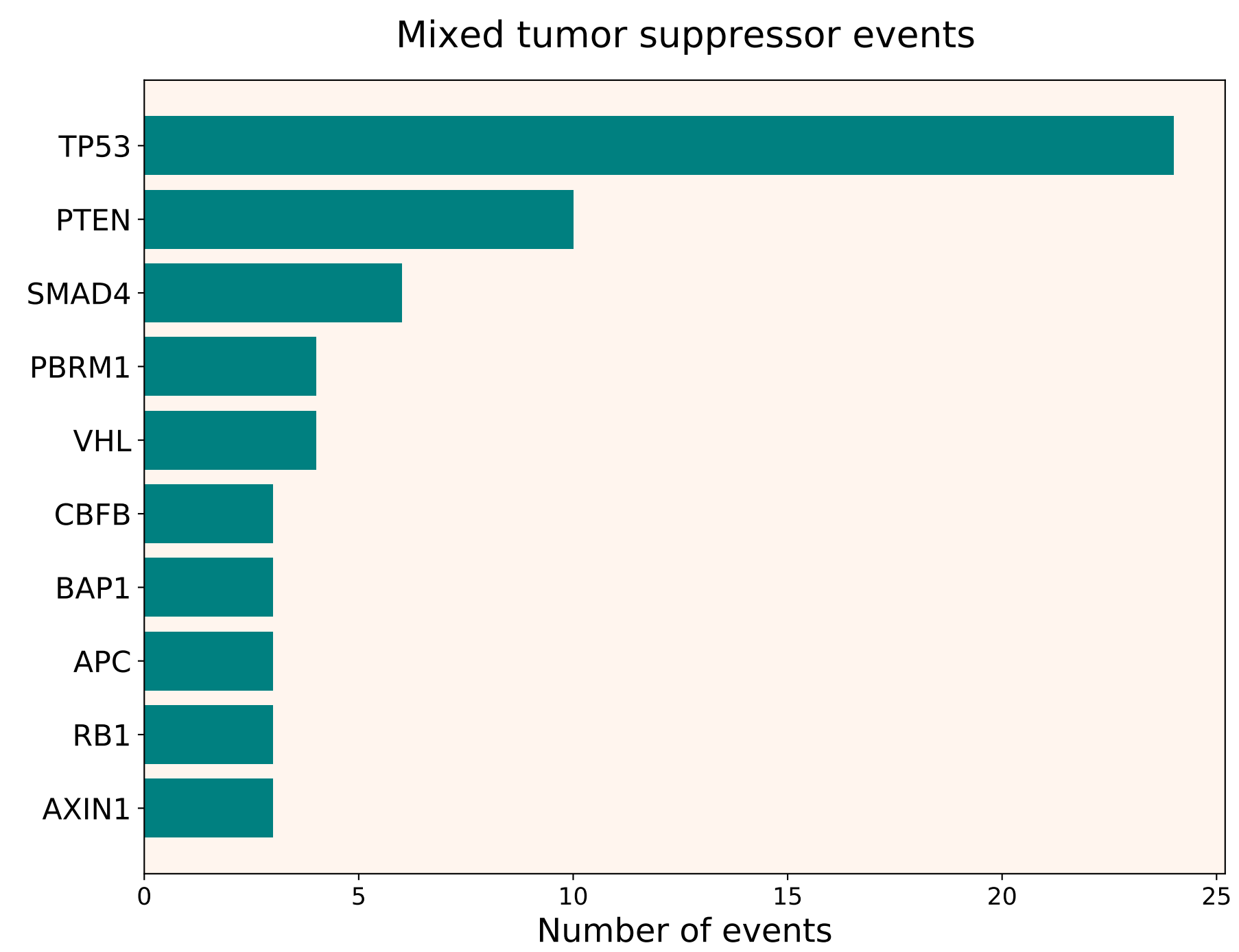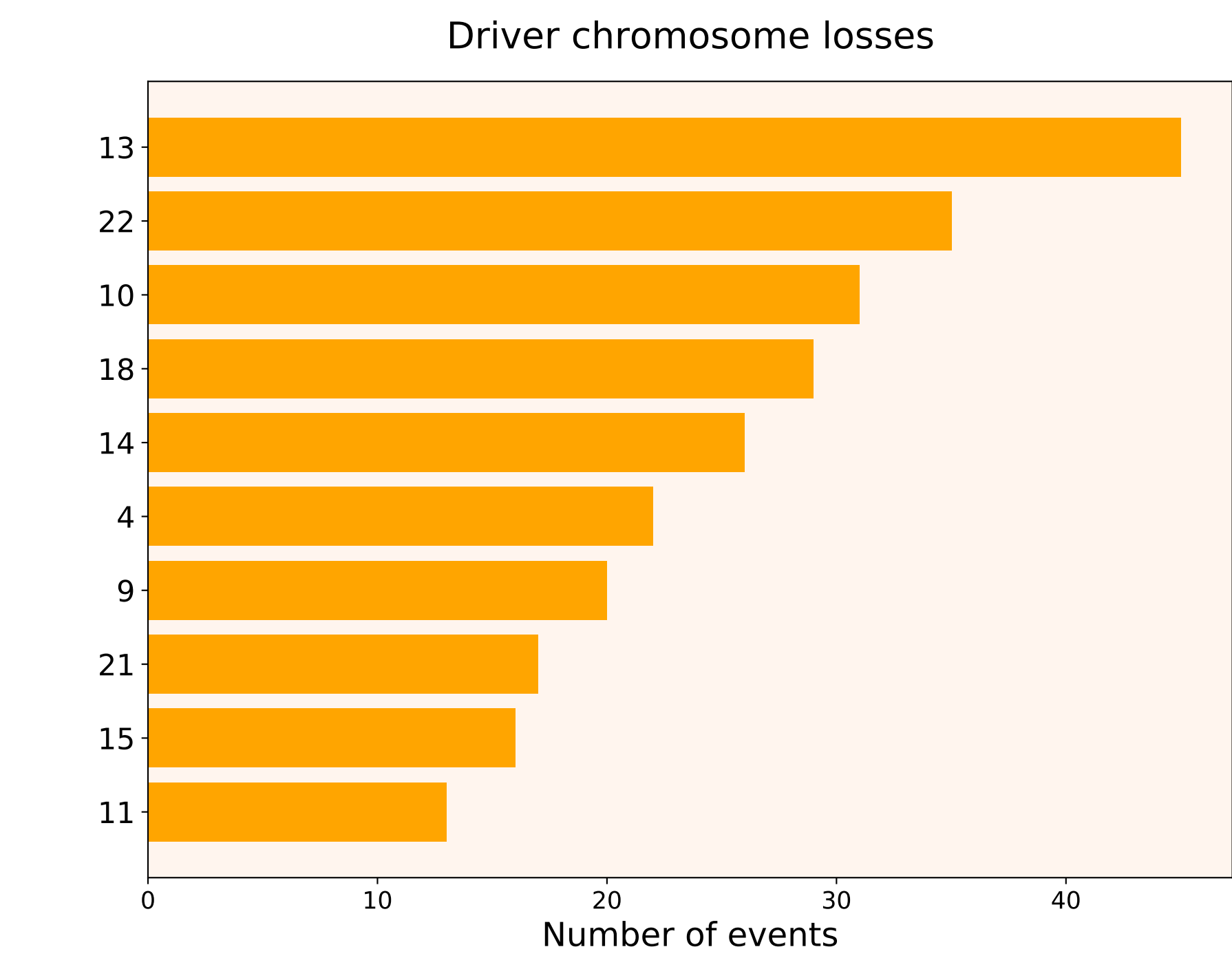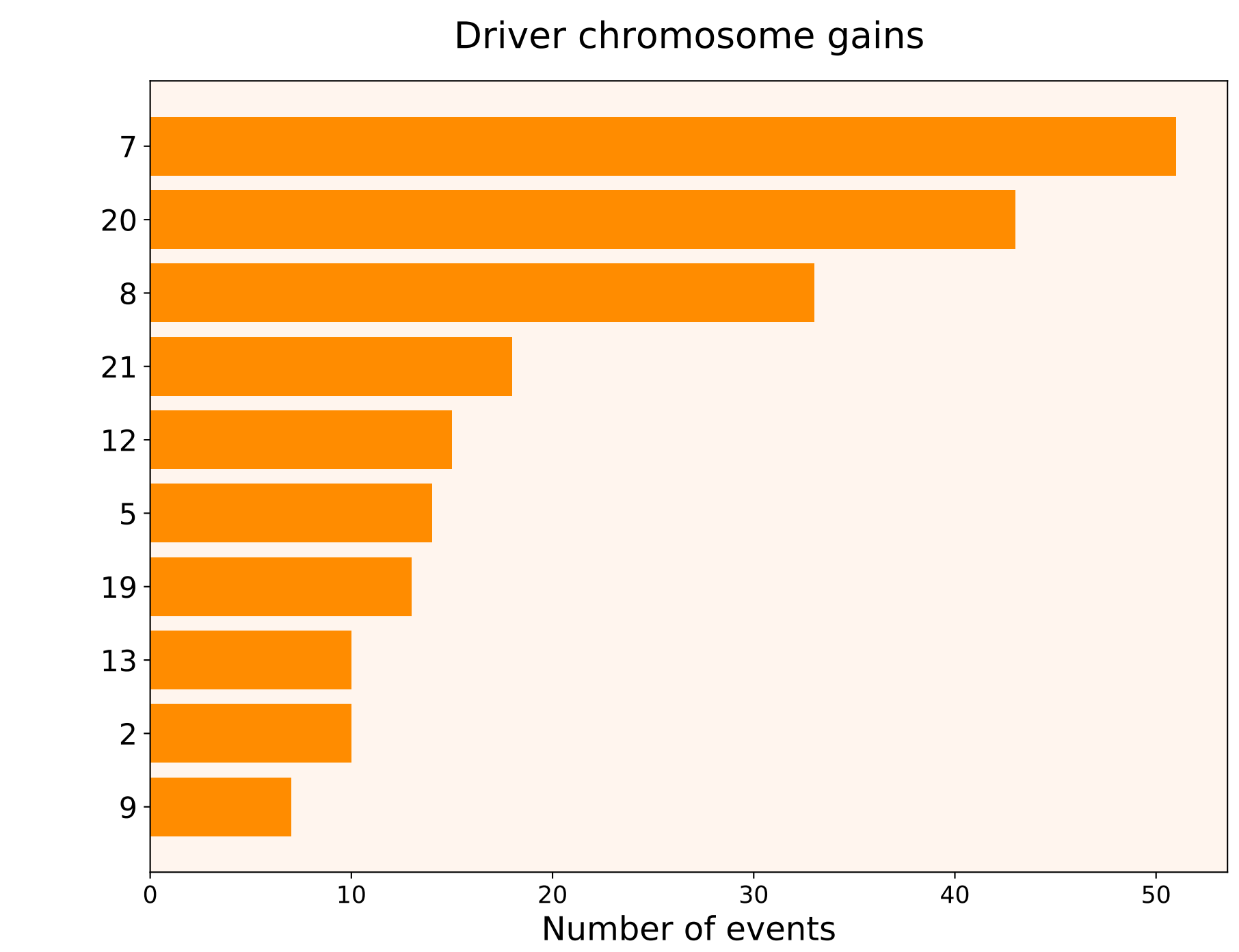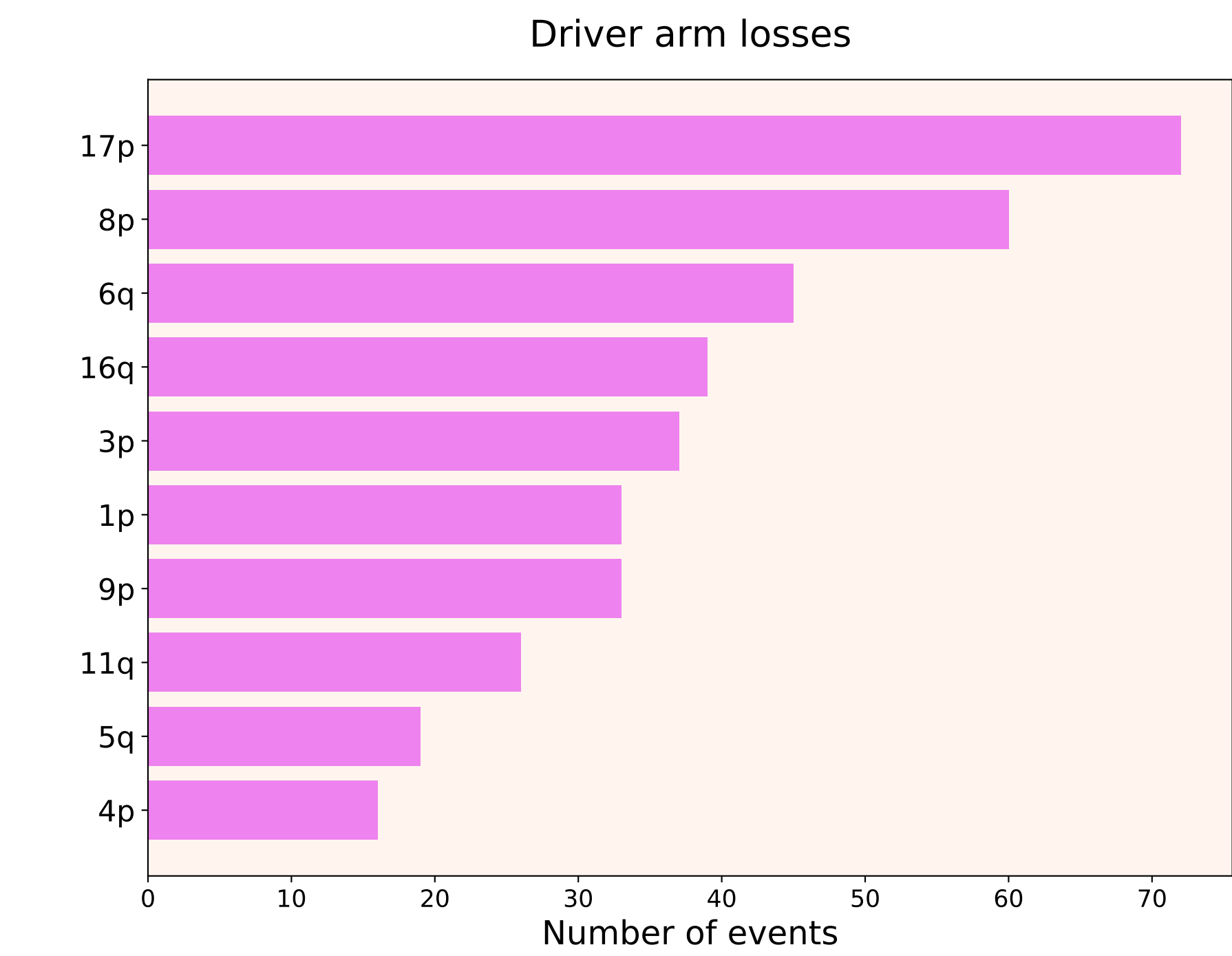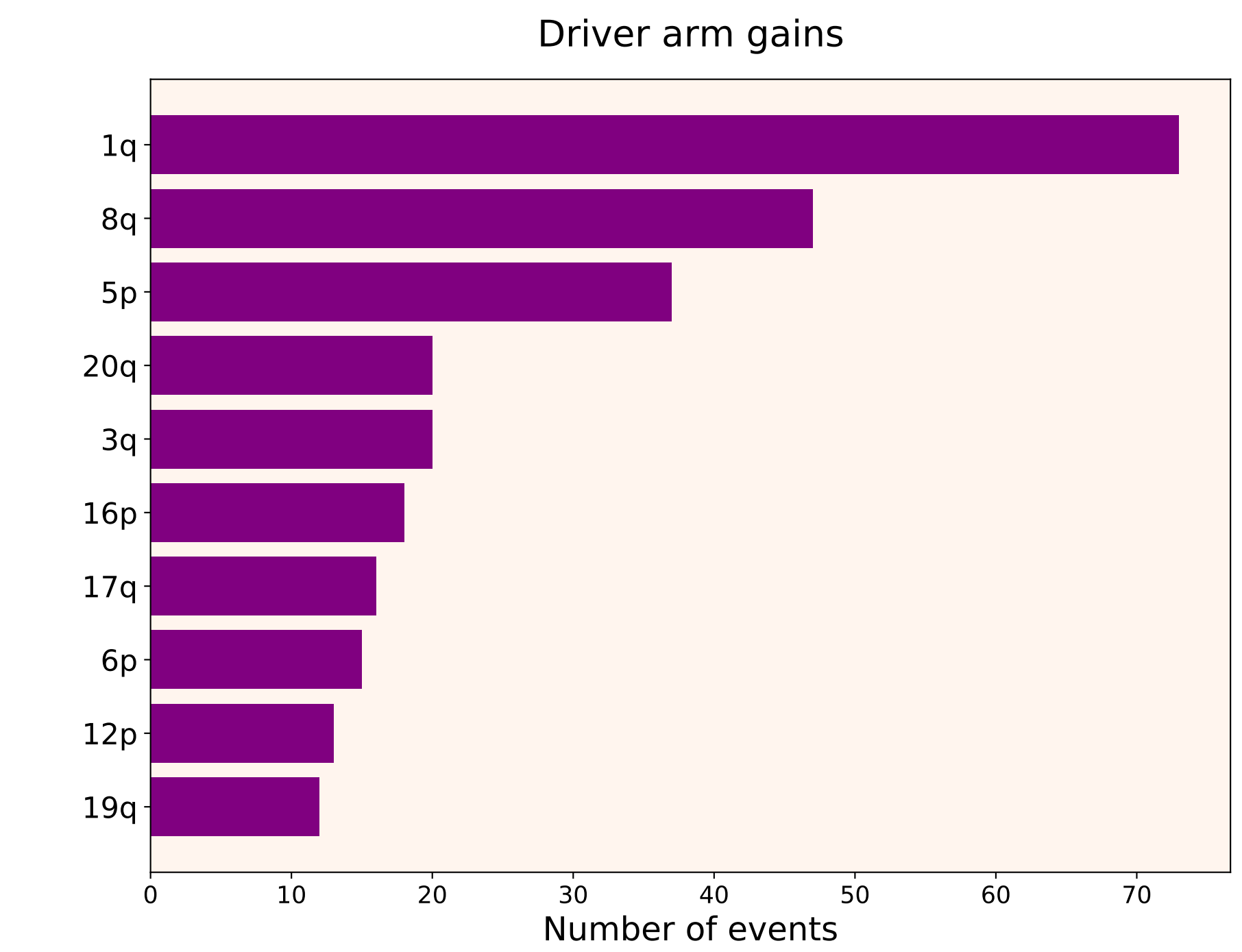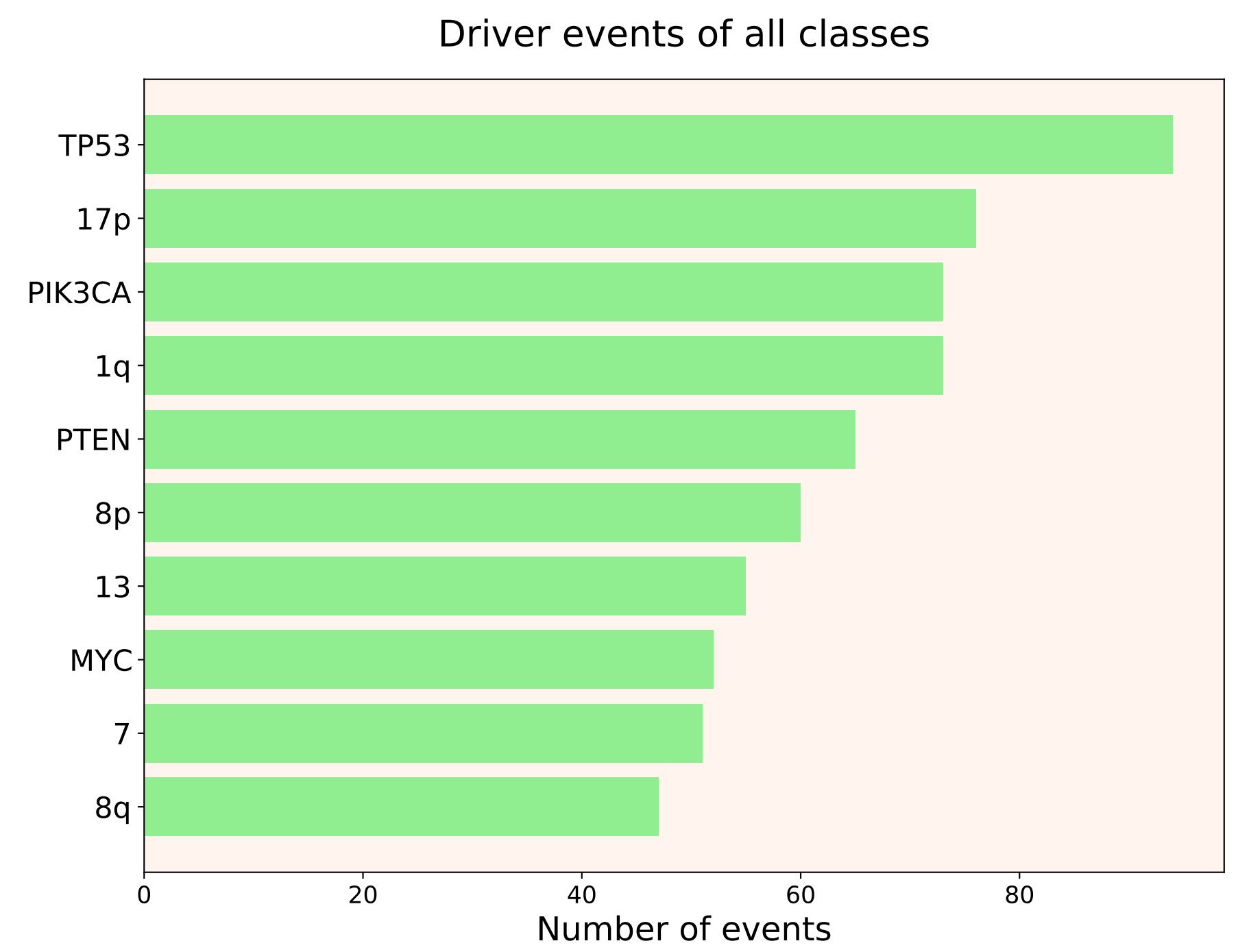

Supplement: Supplemental Information 2 [file peerj-10-13860-s002.zip › COHORTS/genes plots/detailed/2021_8_16_14_9_distribution_events_detailed_12.pdf]

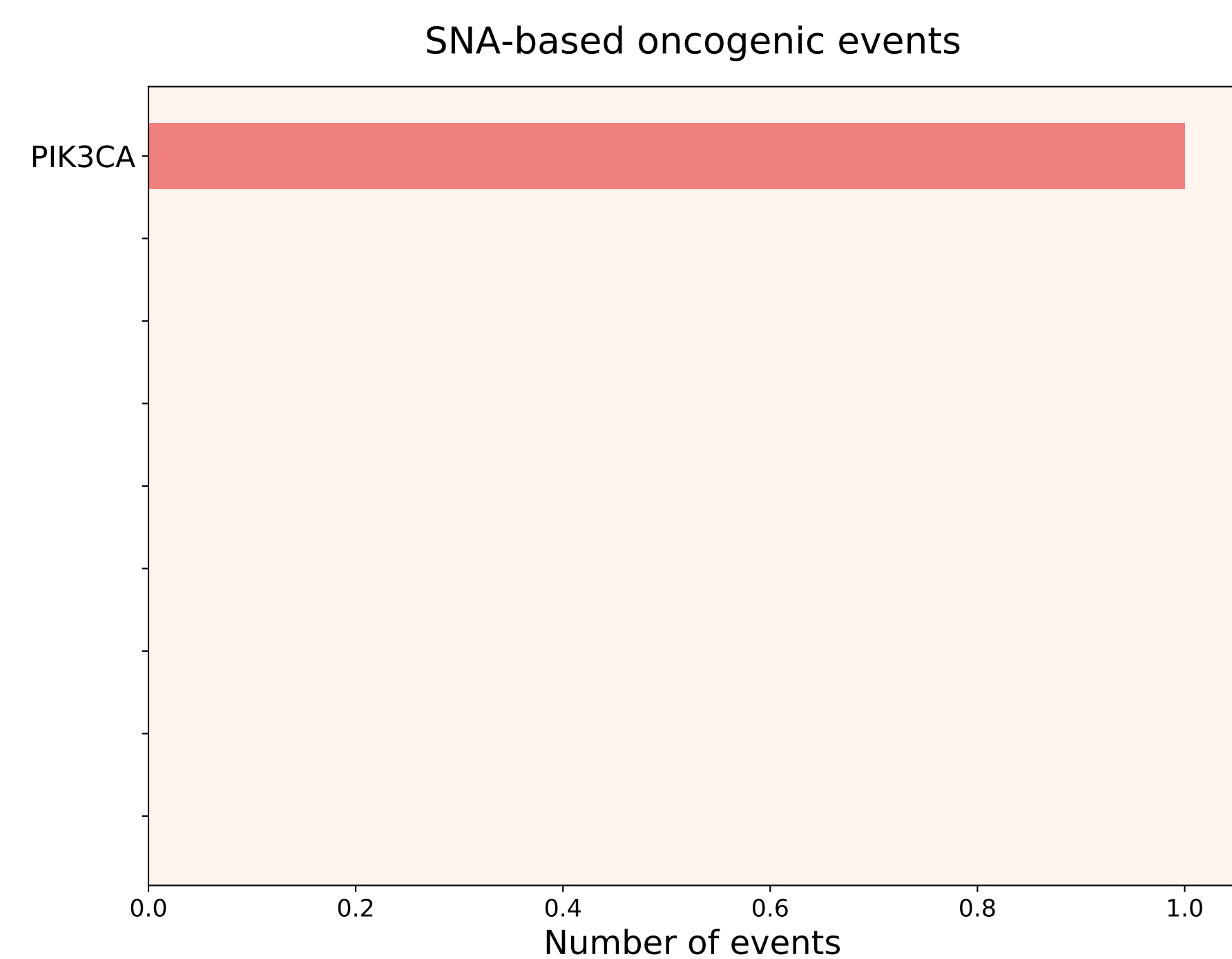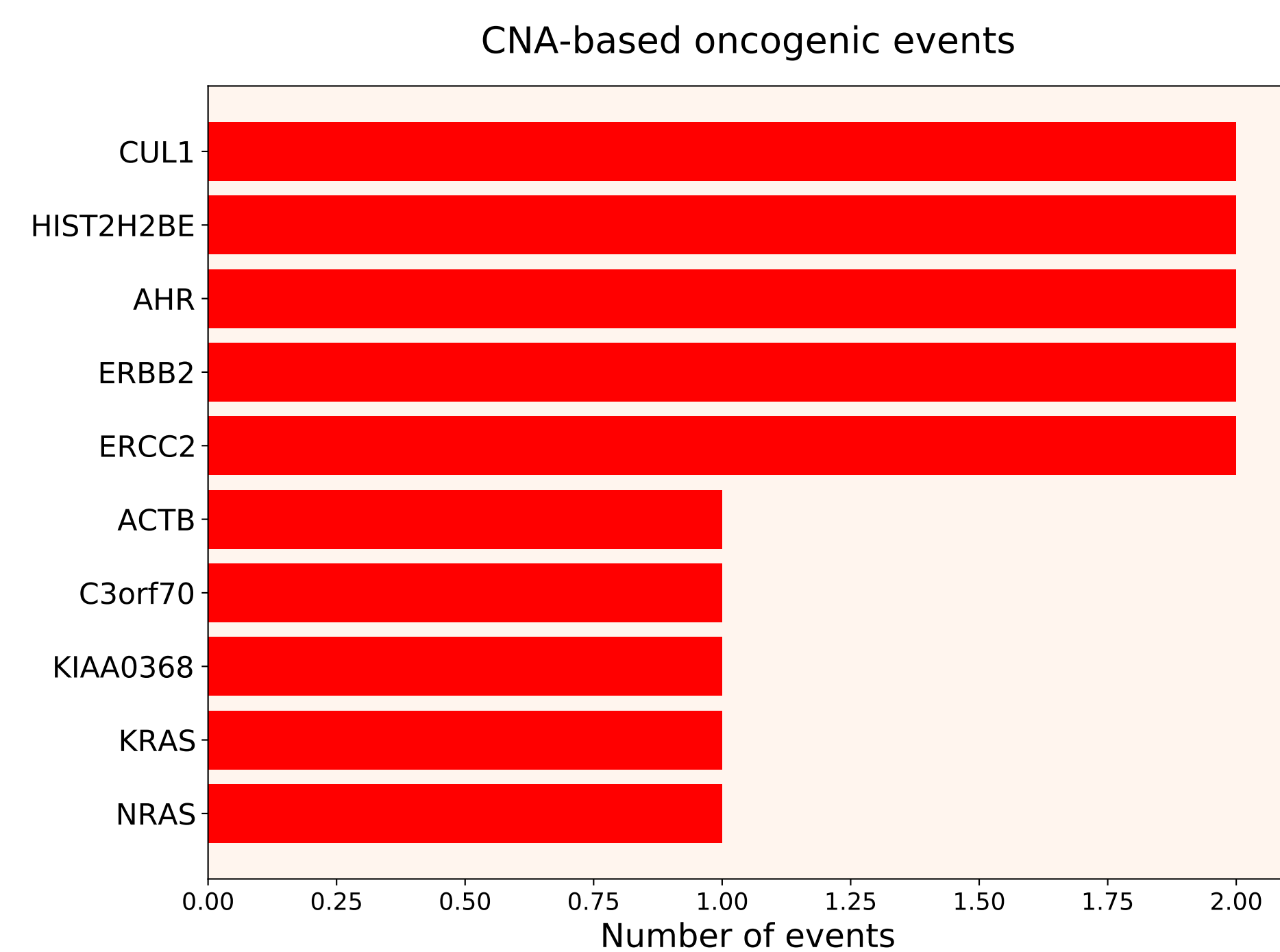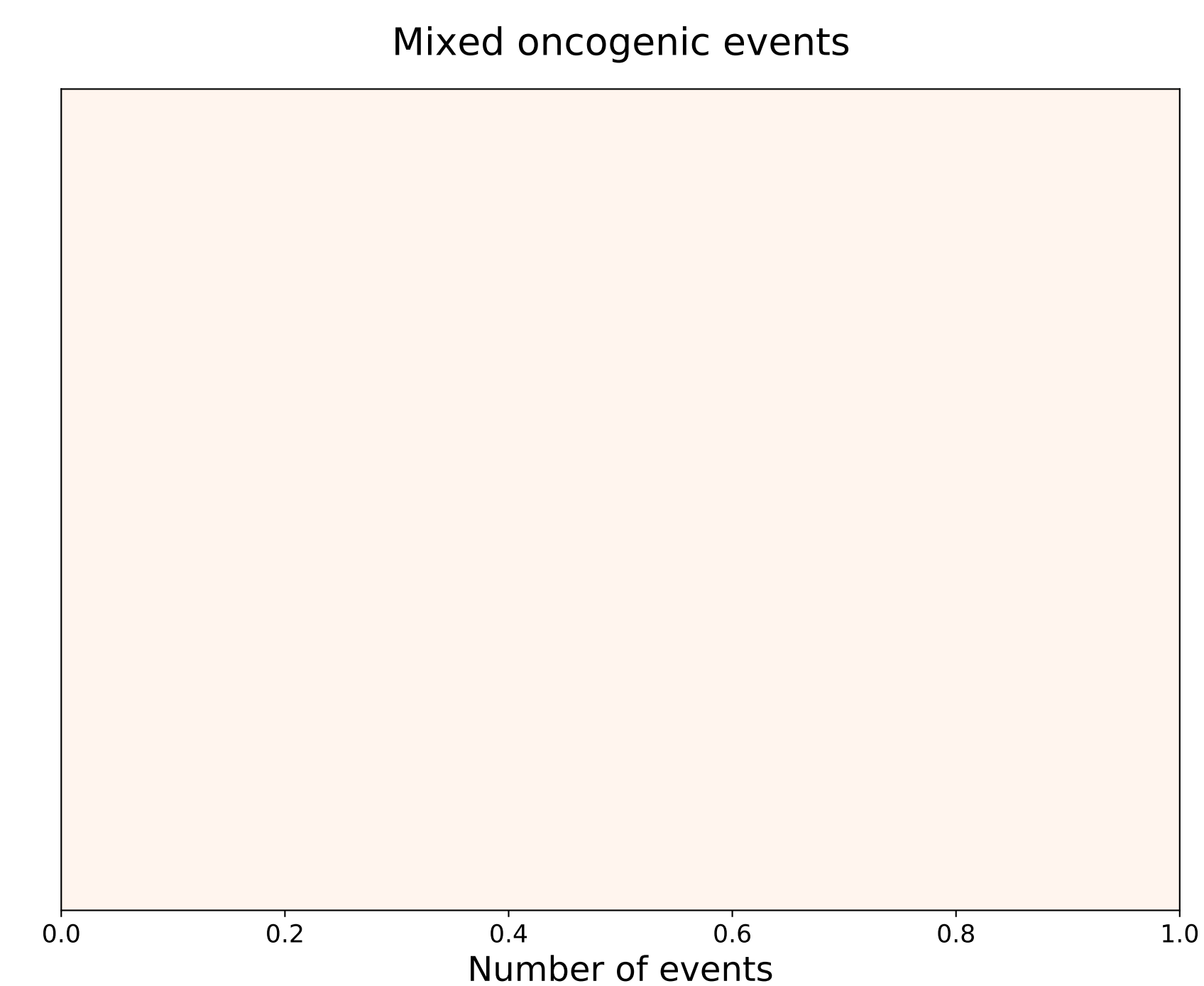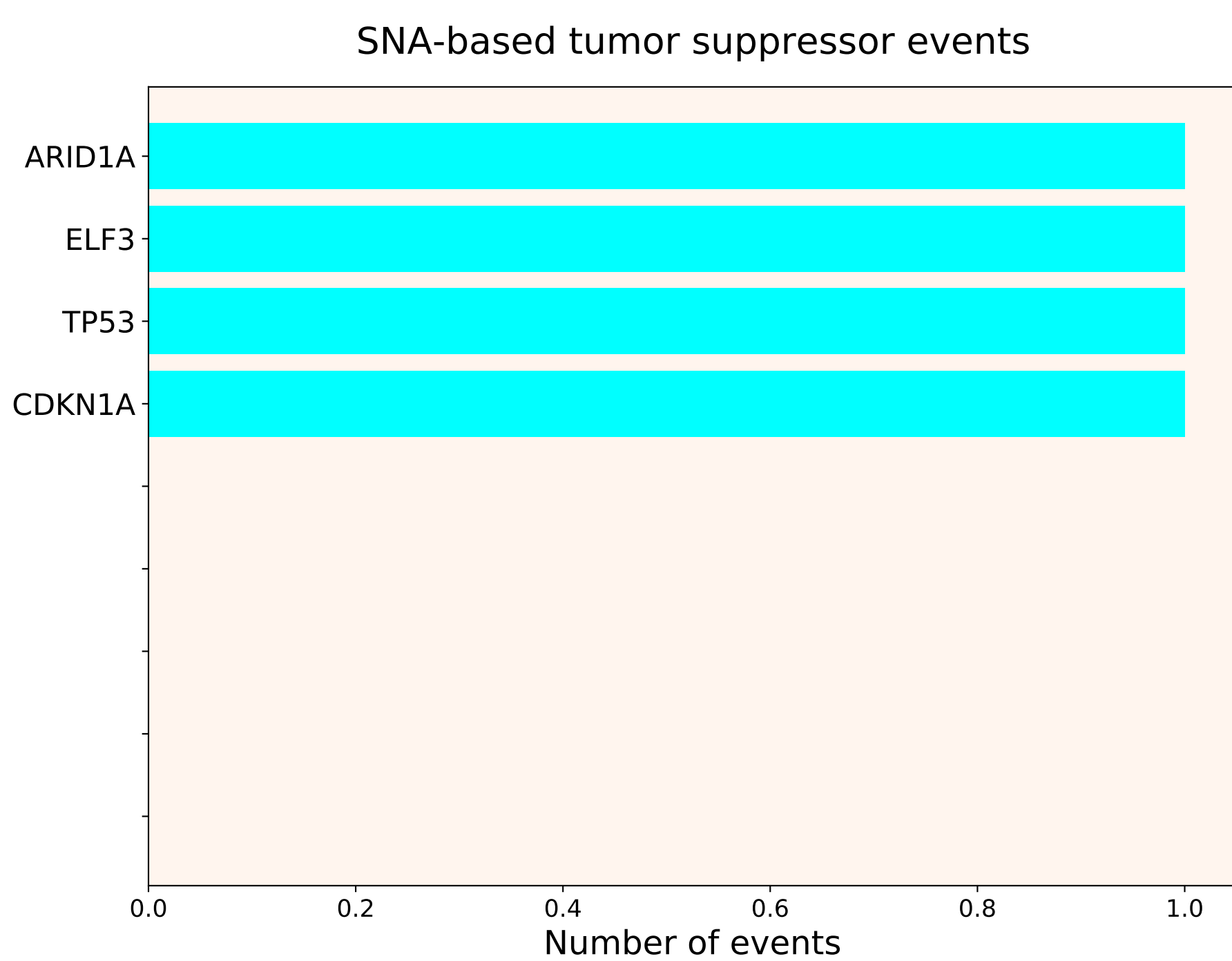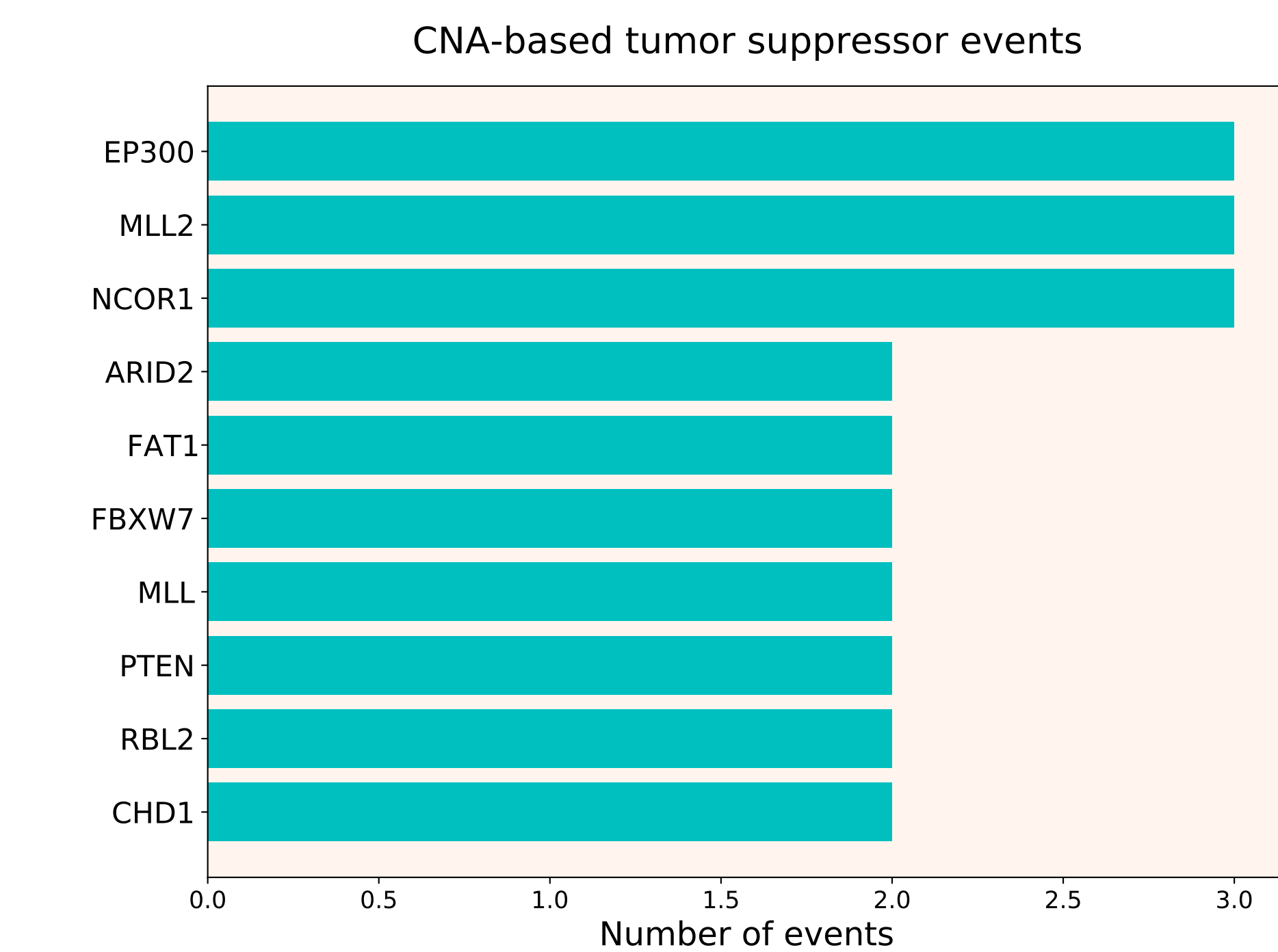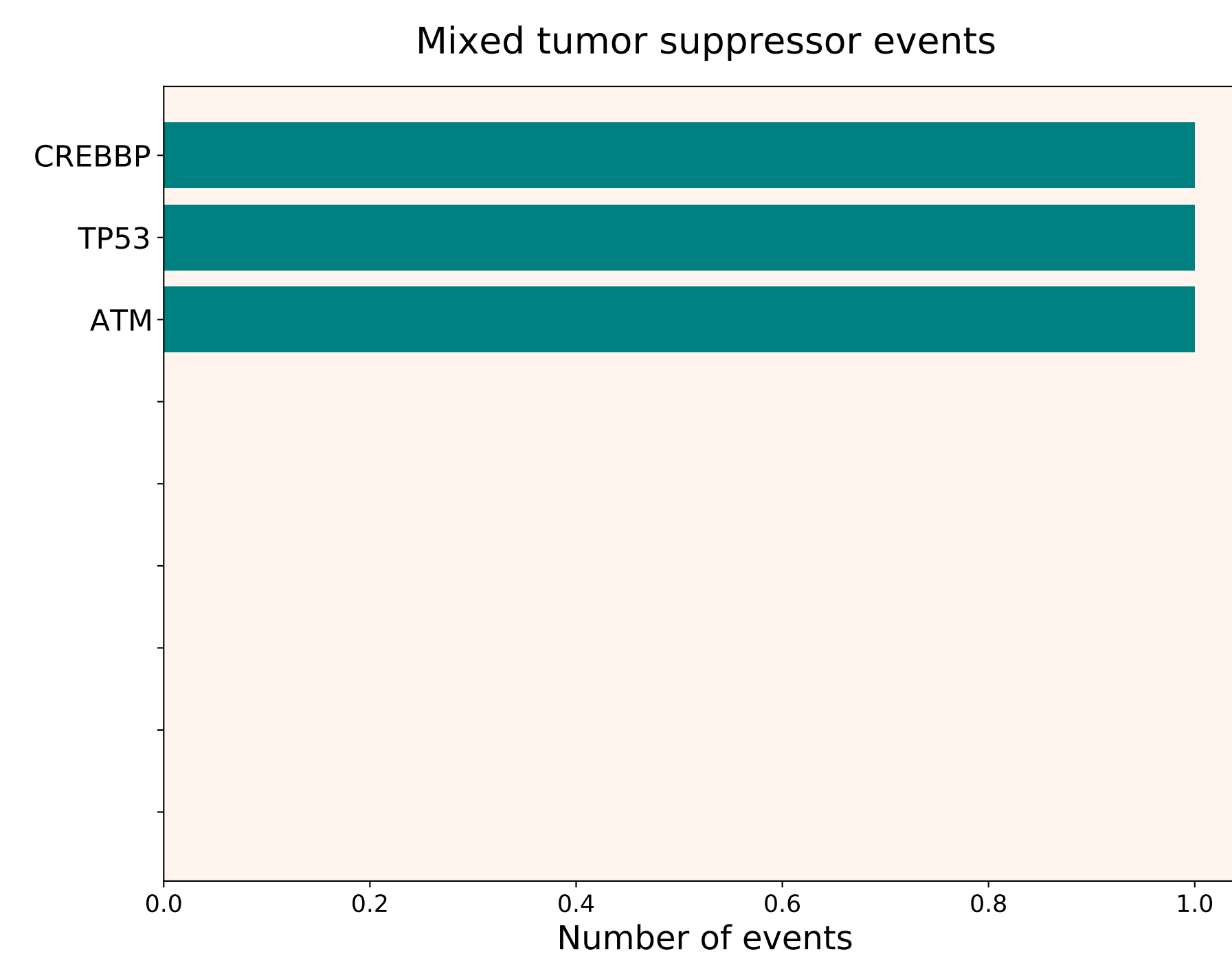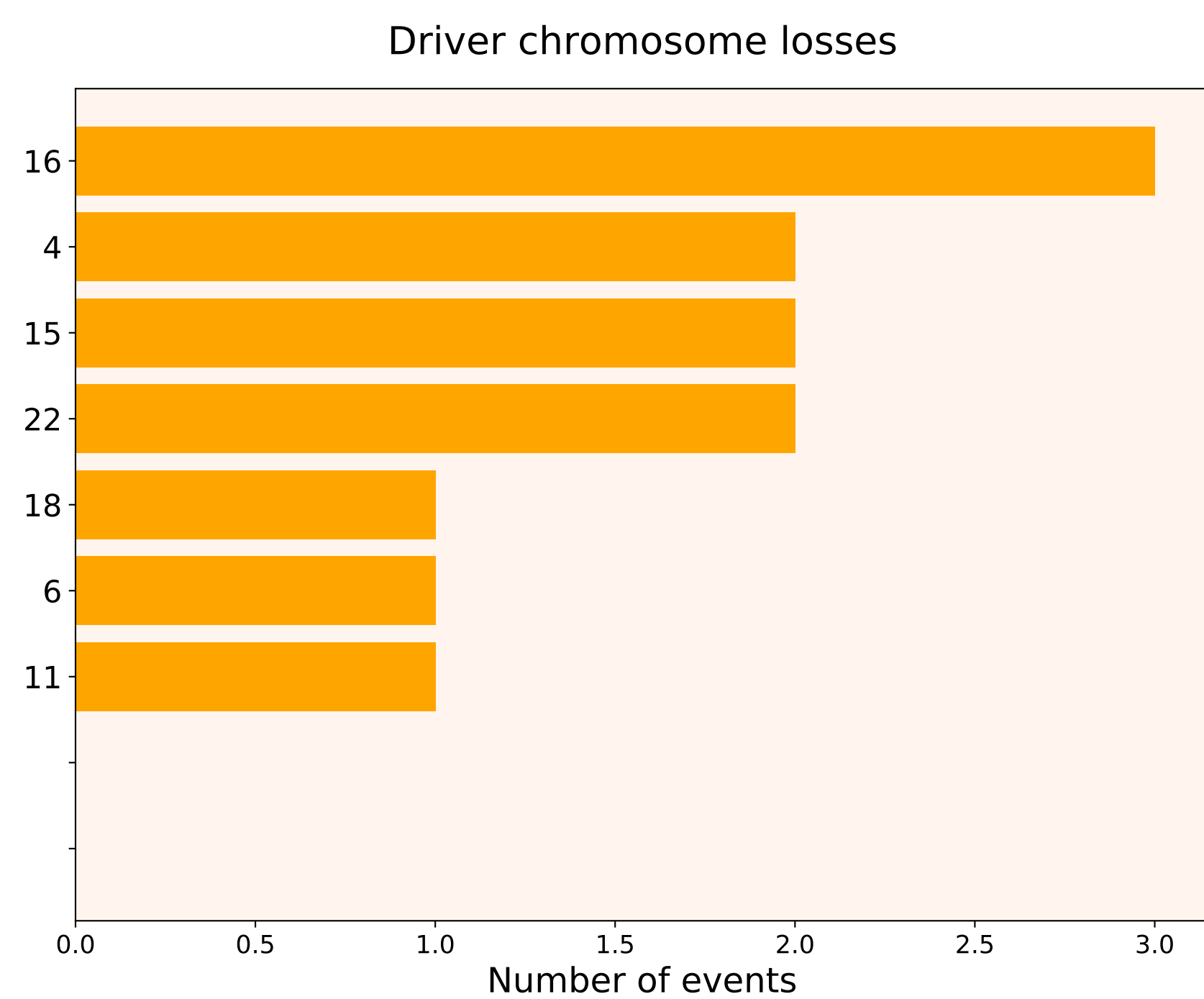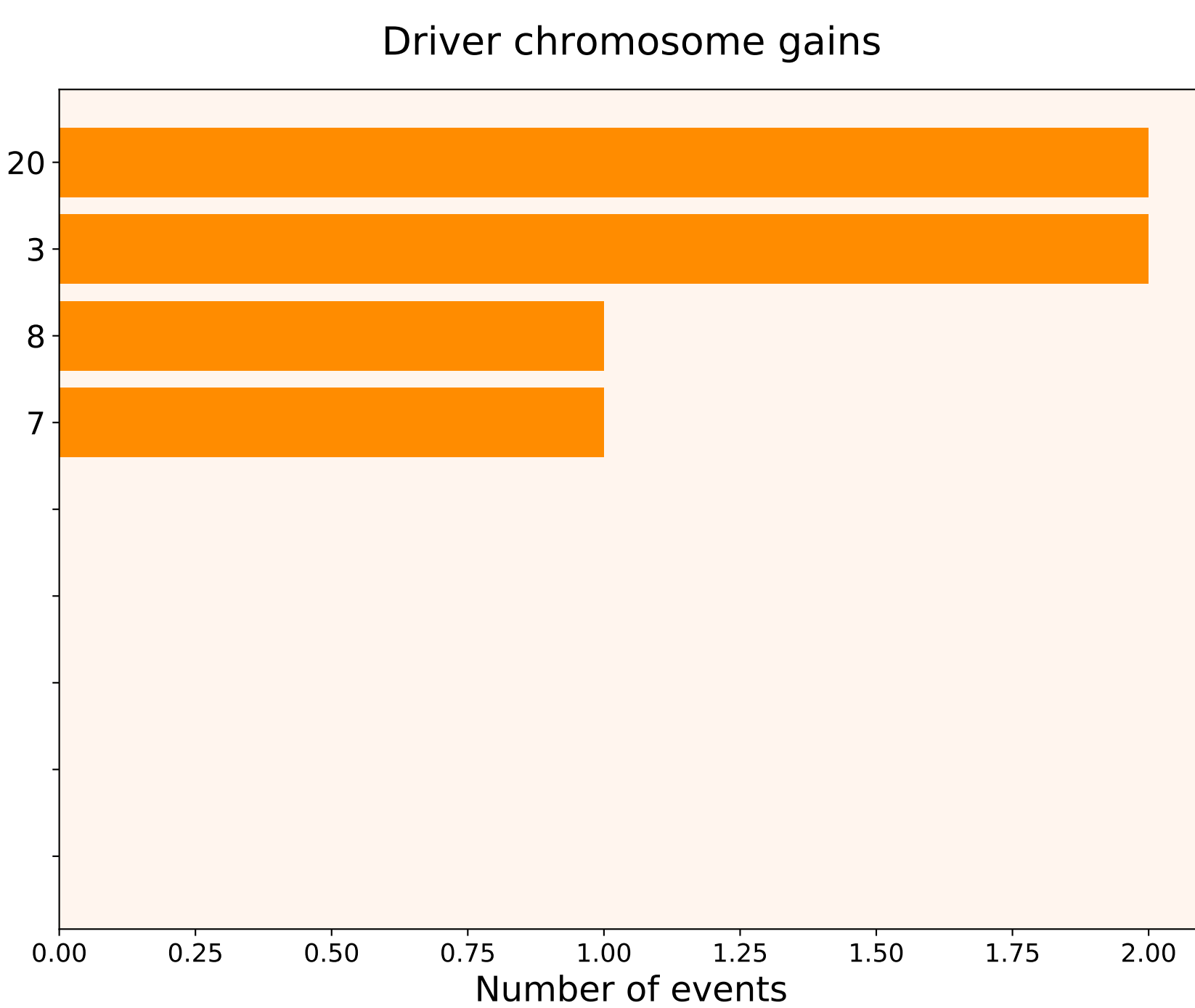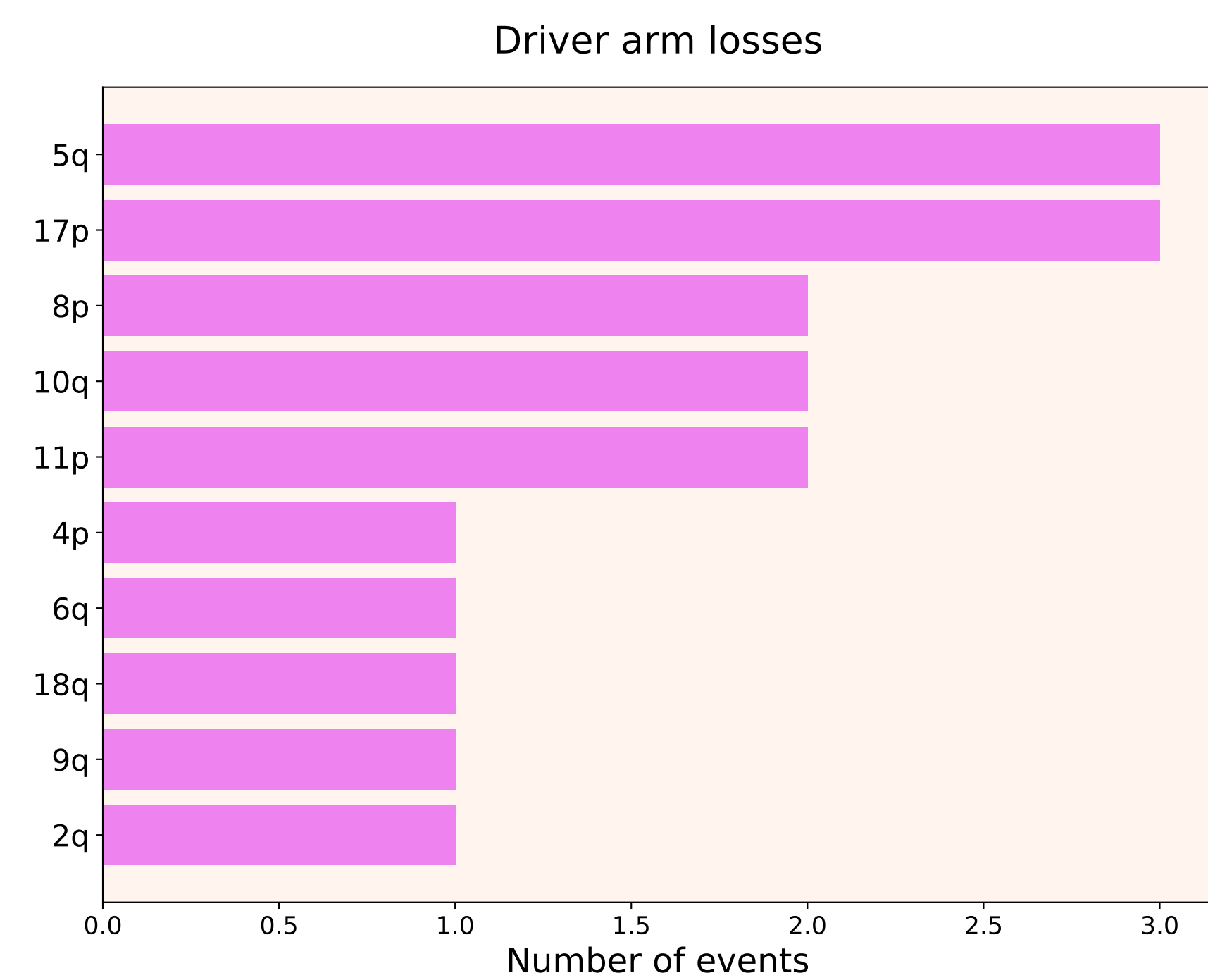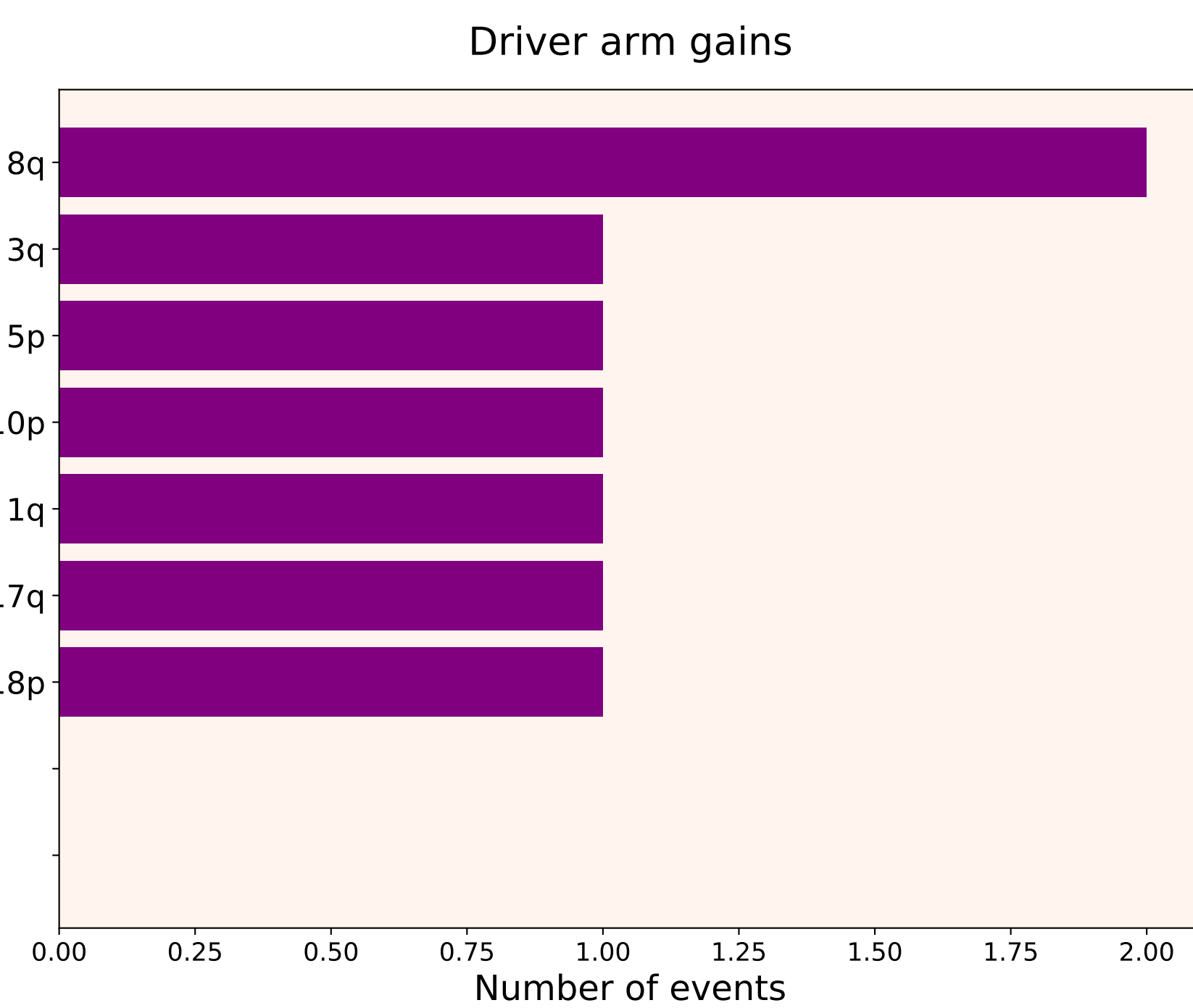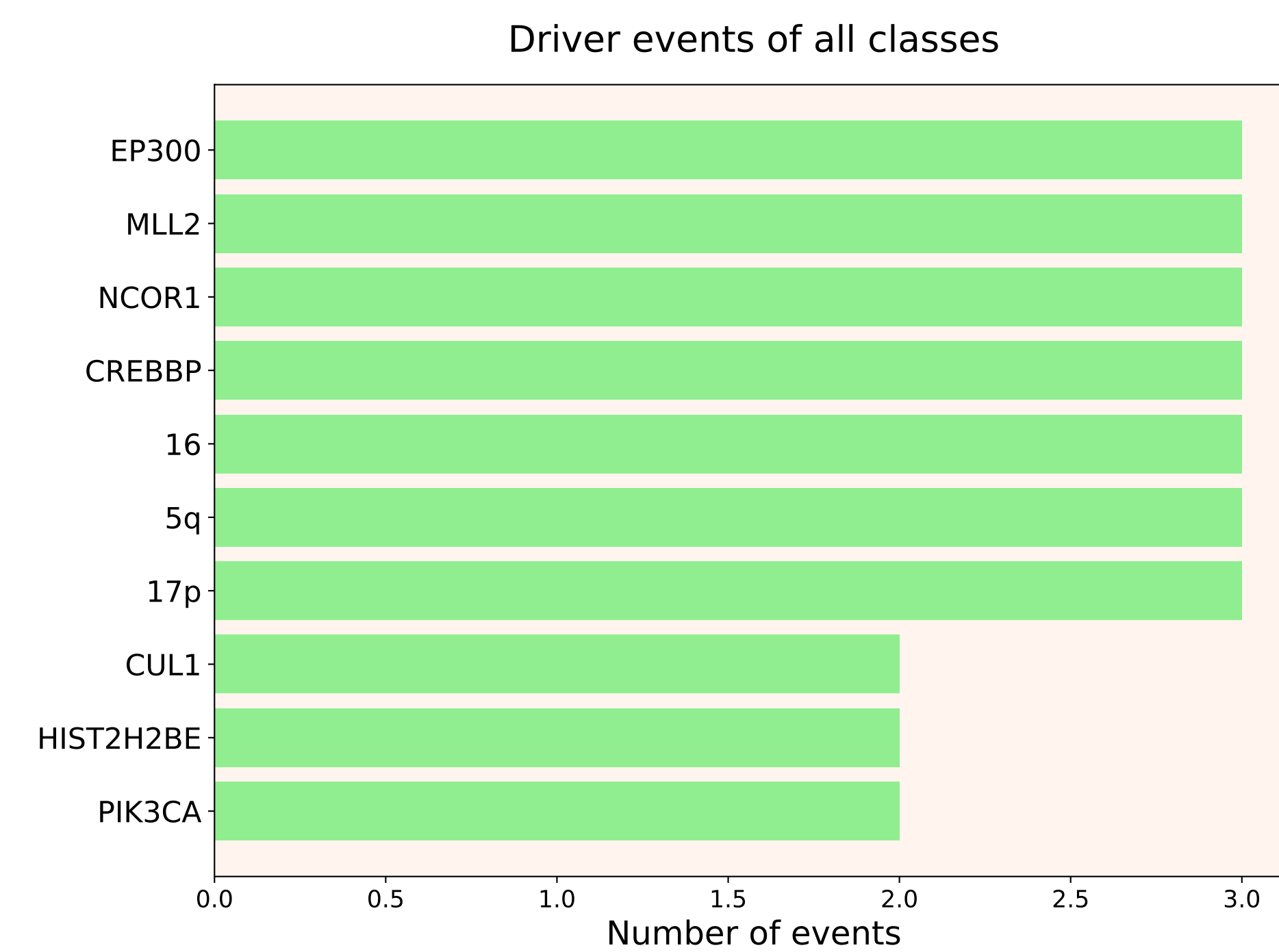

Supplement: Supplemental Information 2 [file peerj-10-13860-s002.zip › COHORTS/genes plots/detailed/2021_8_16_14_9_distribution_events_detailed_38.pdf]

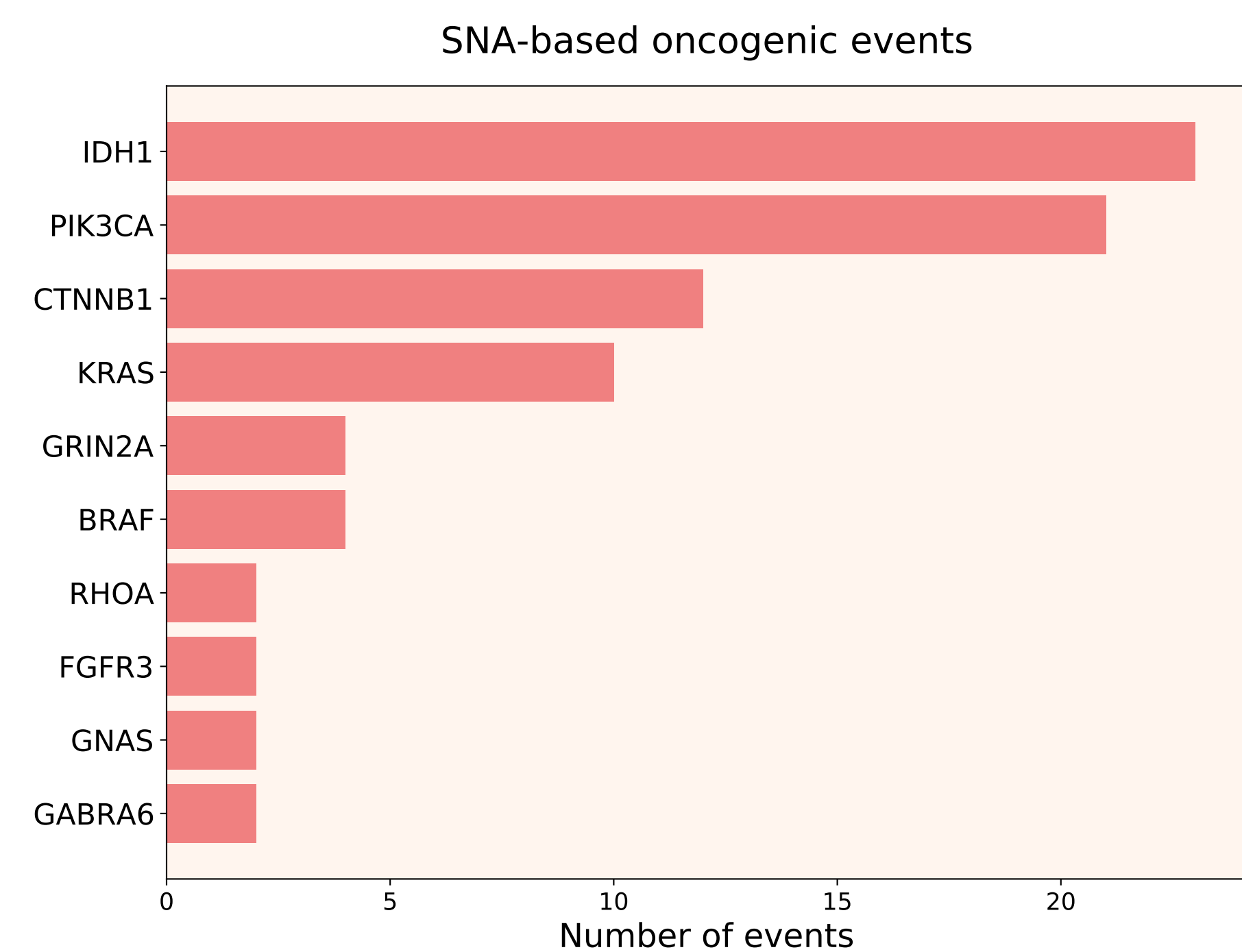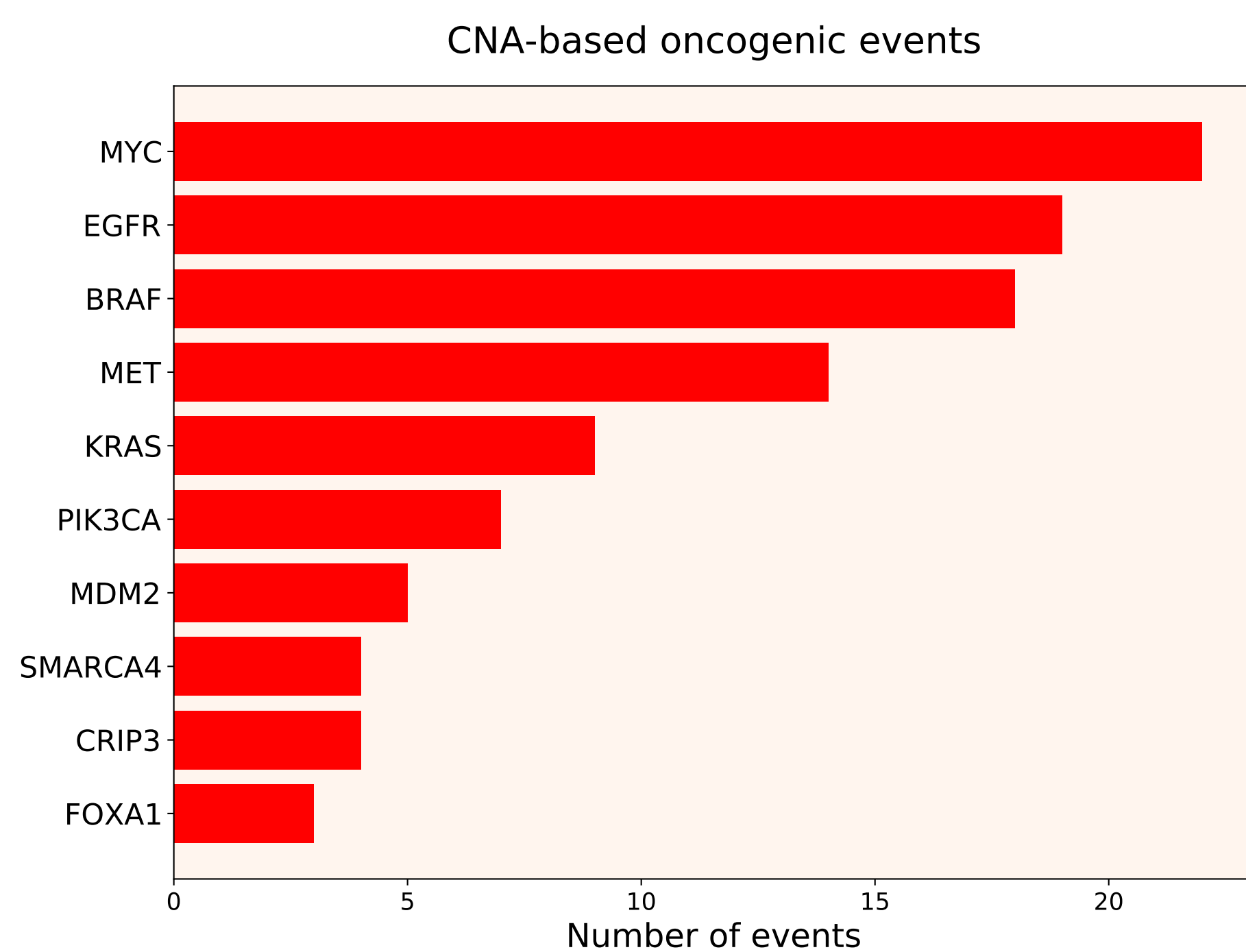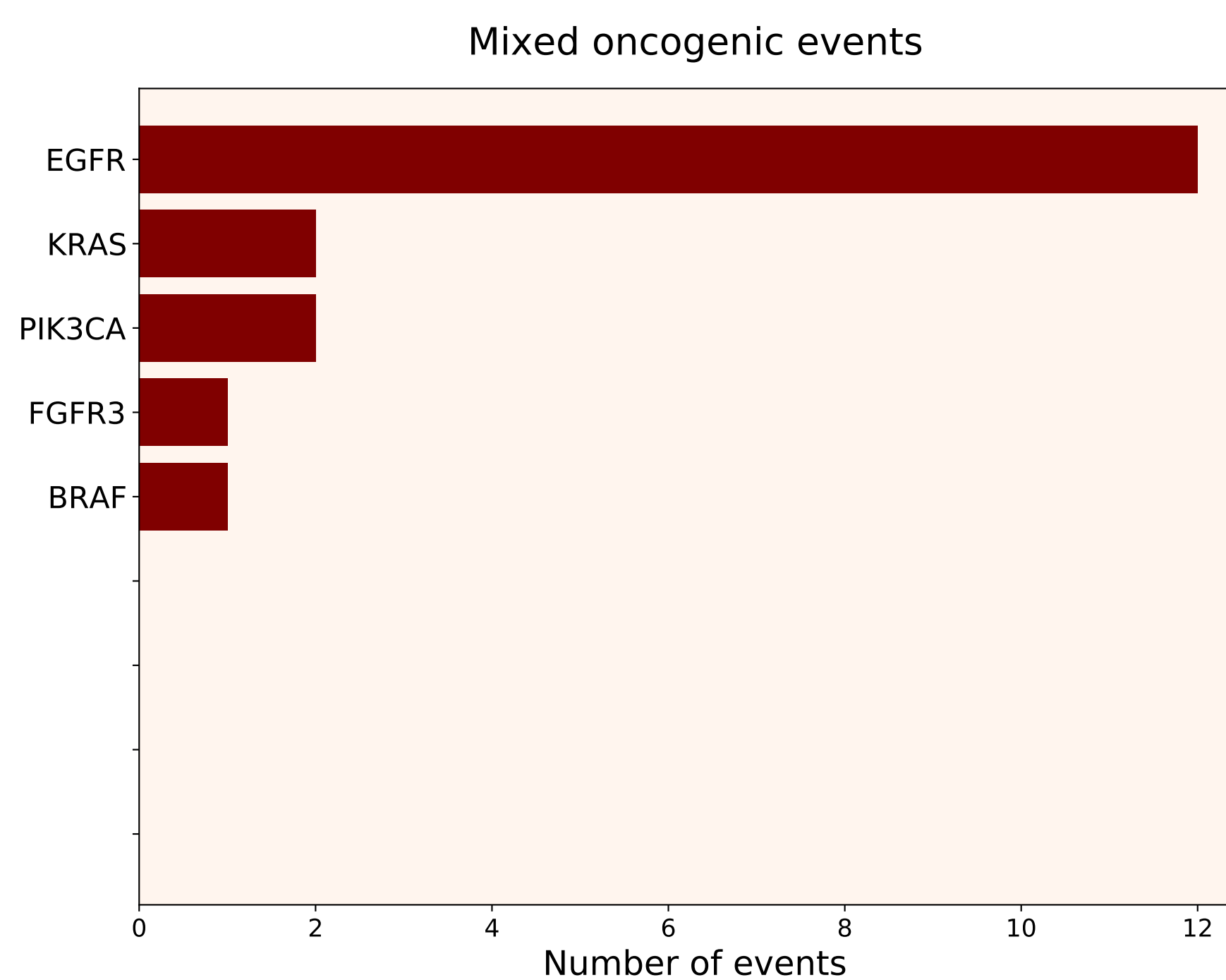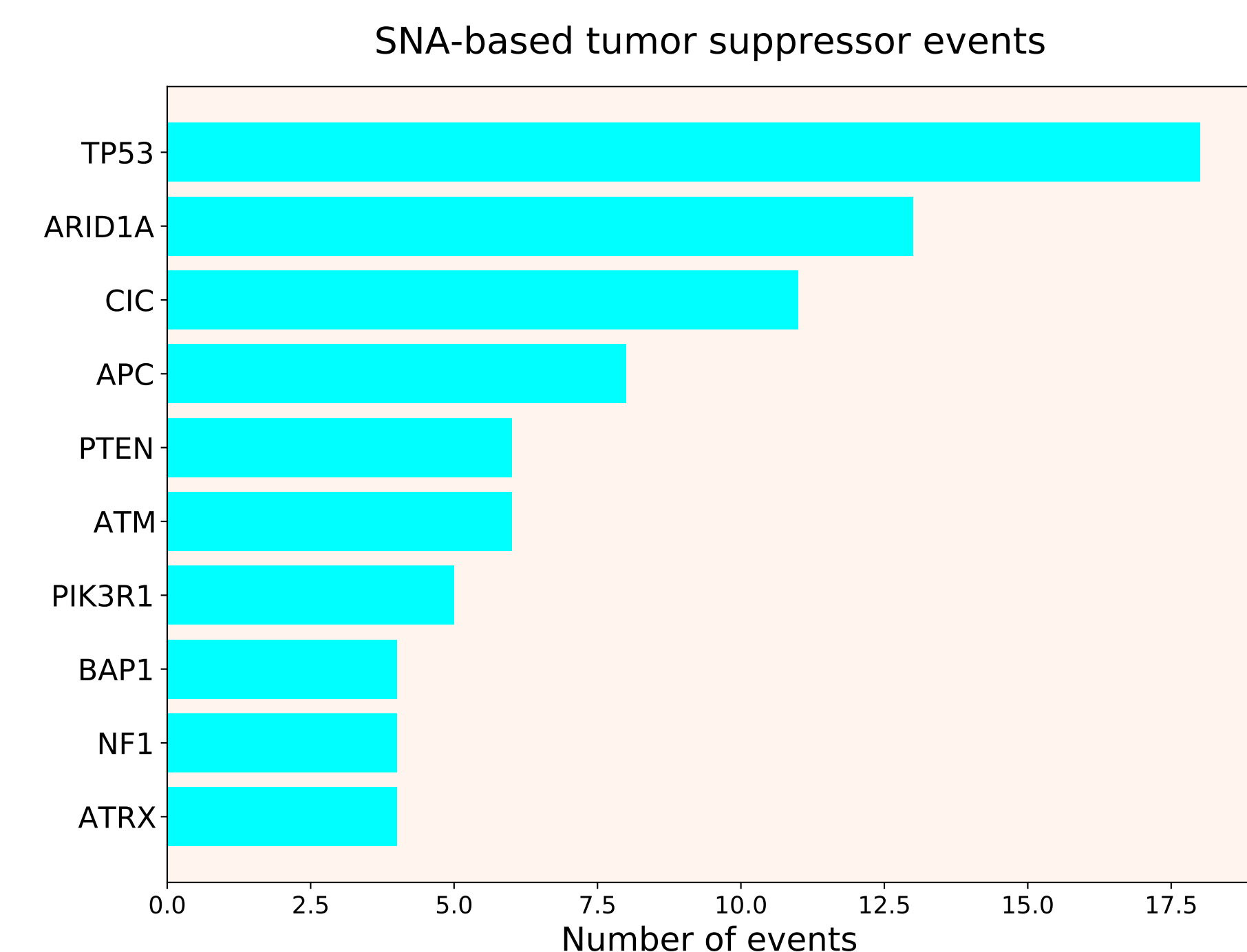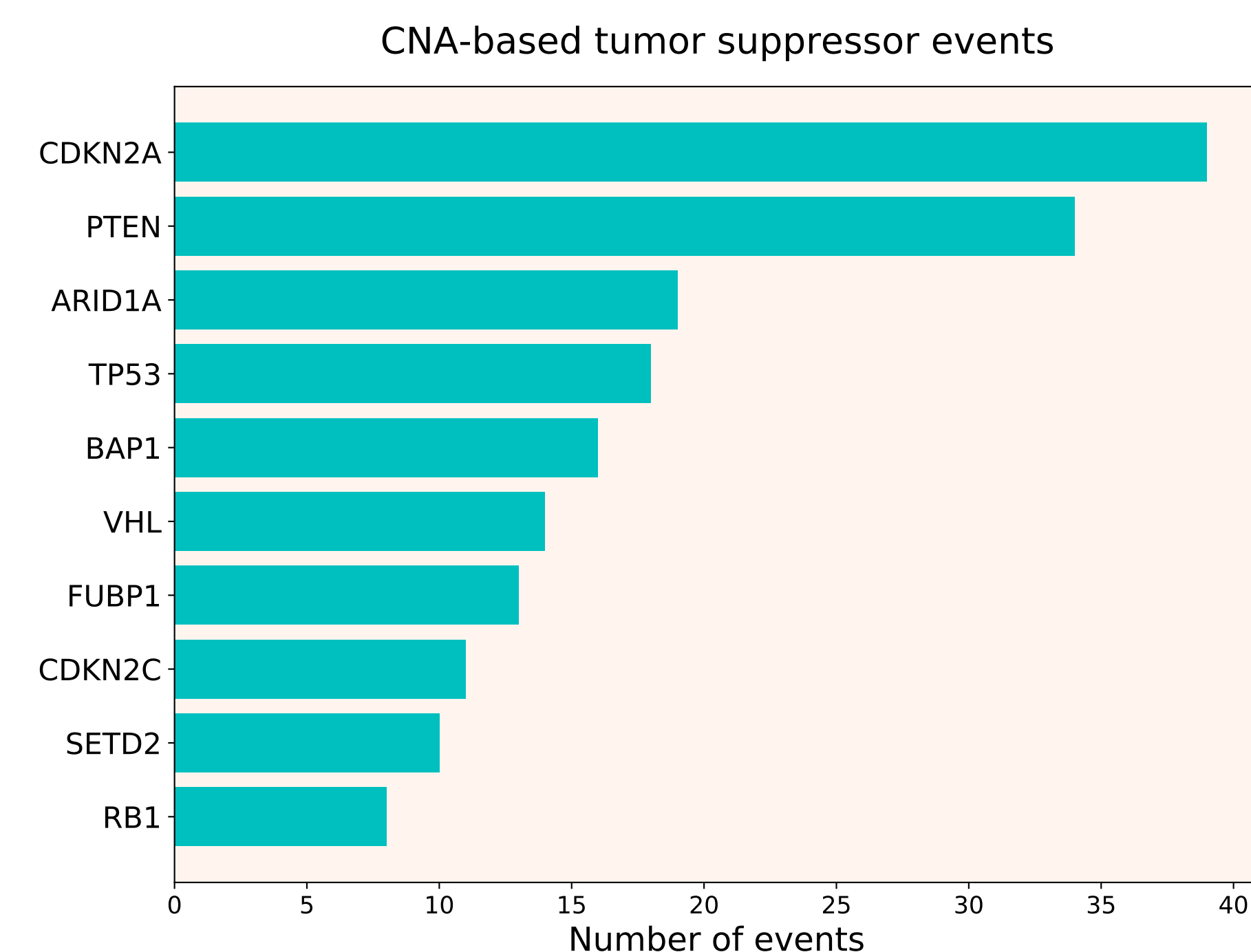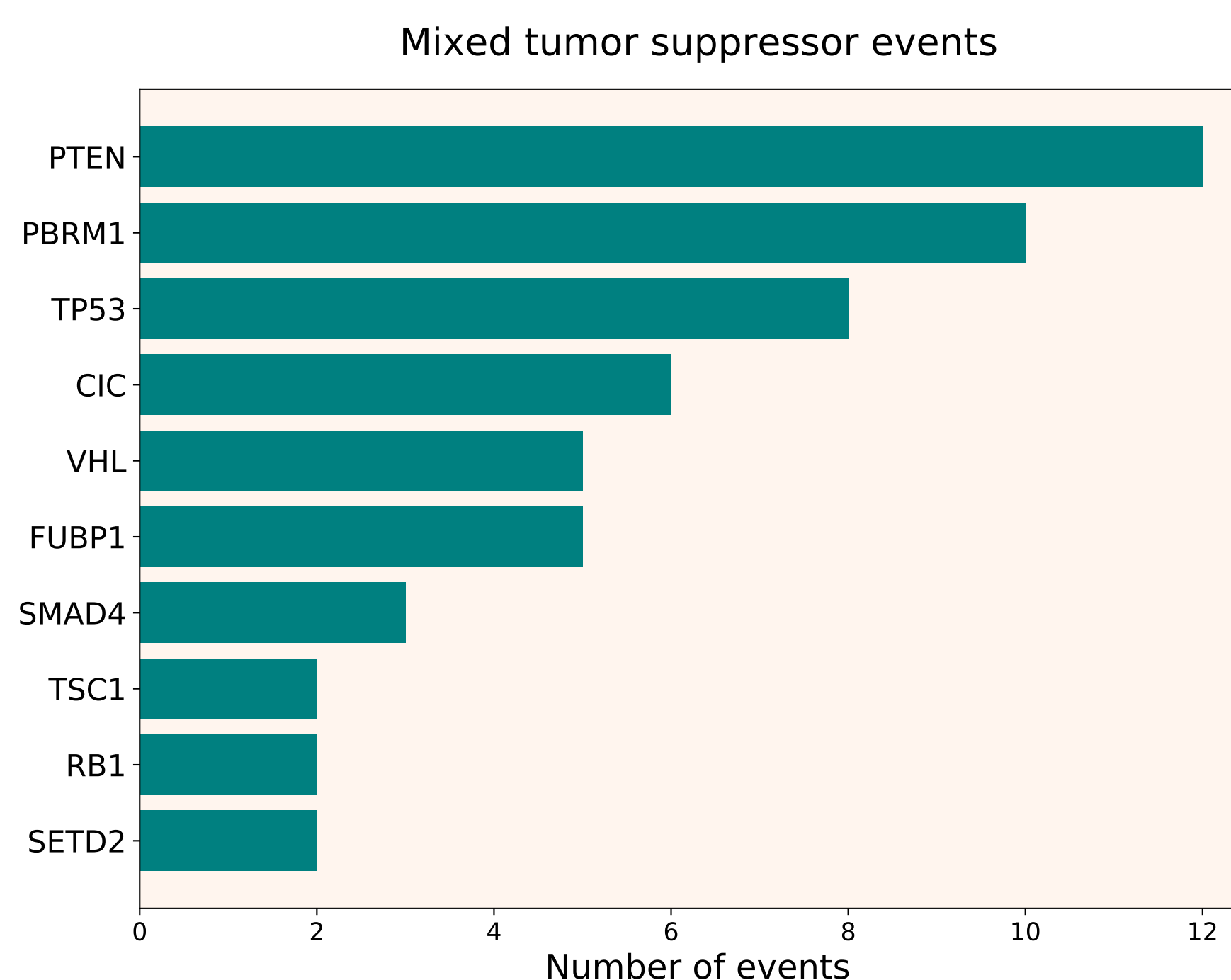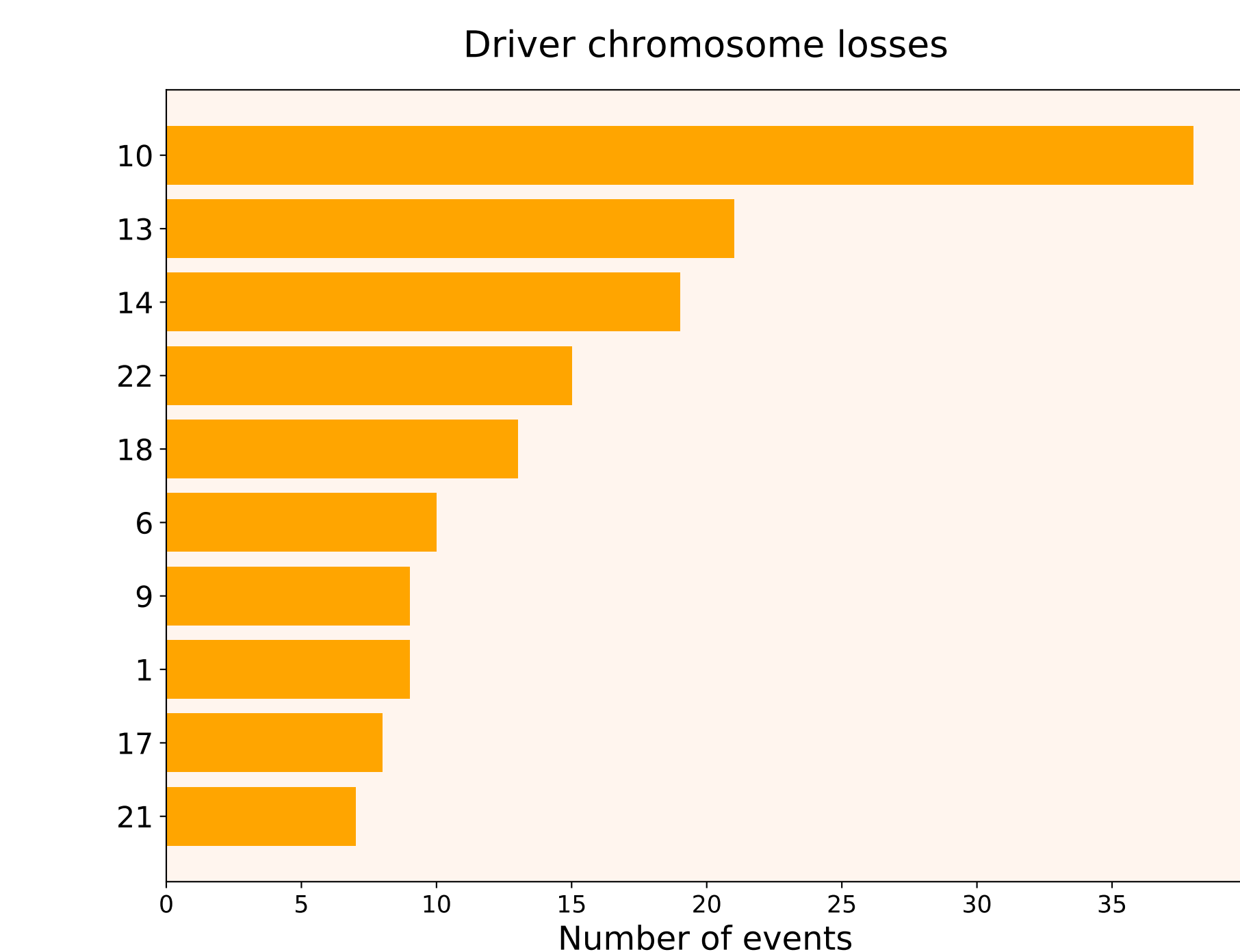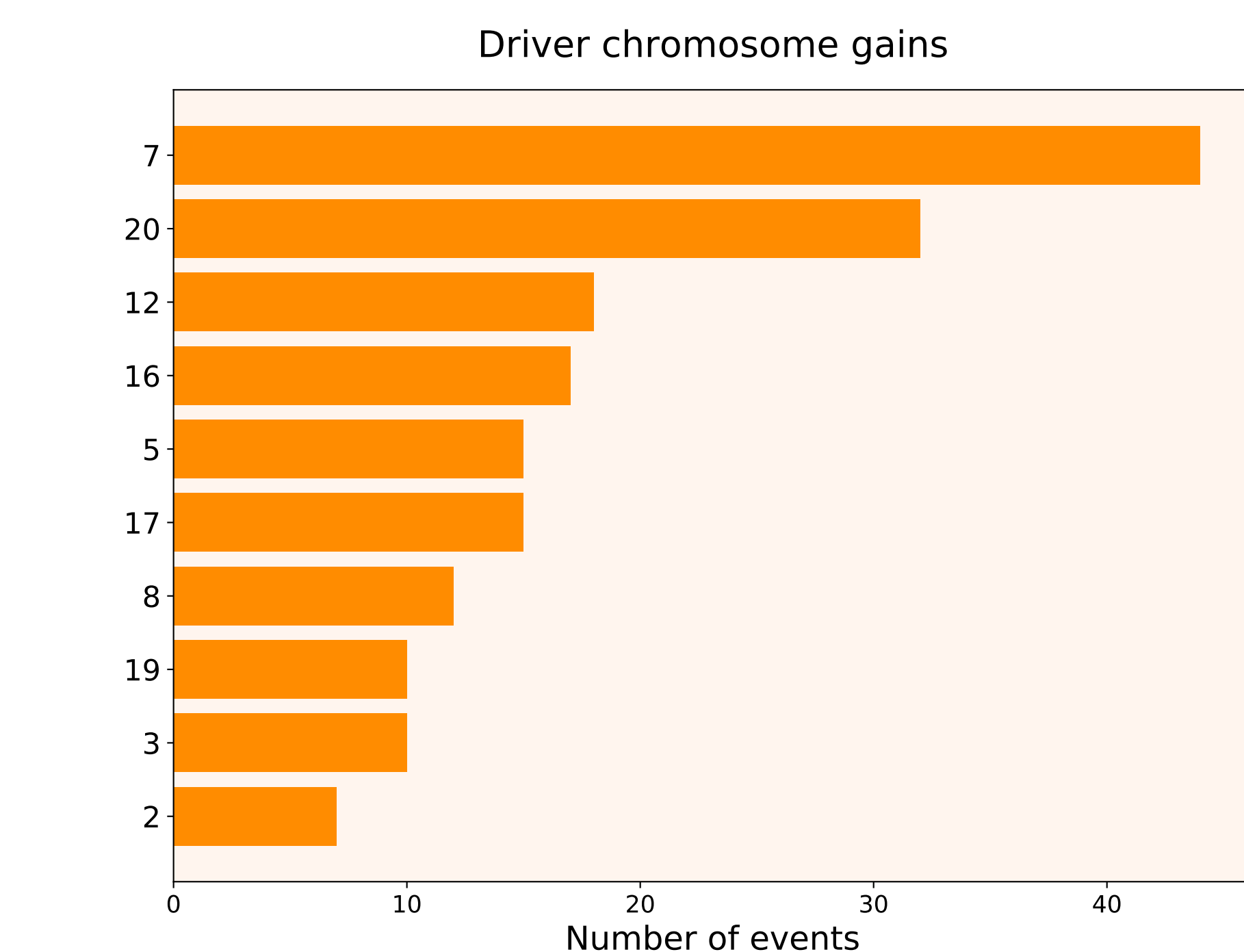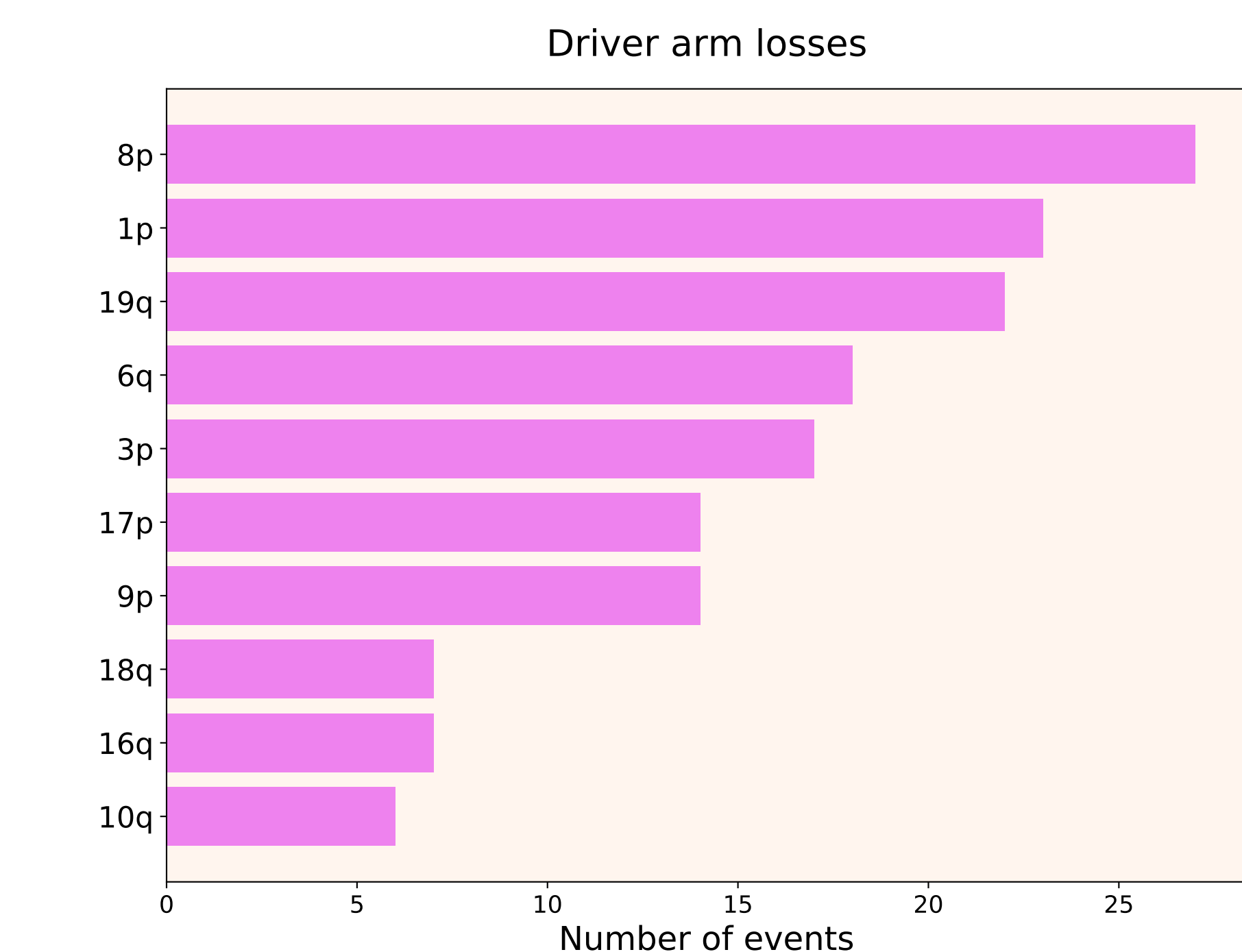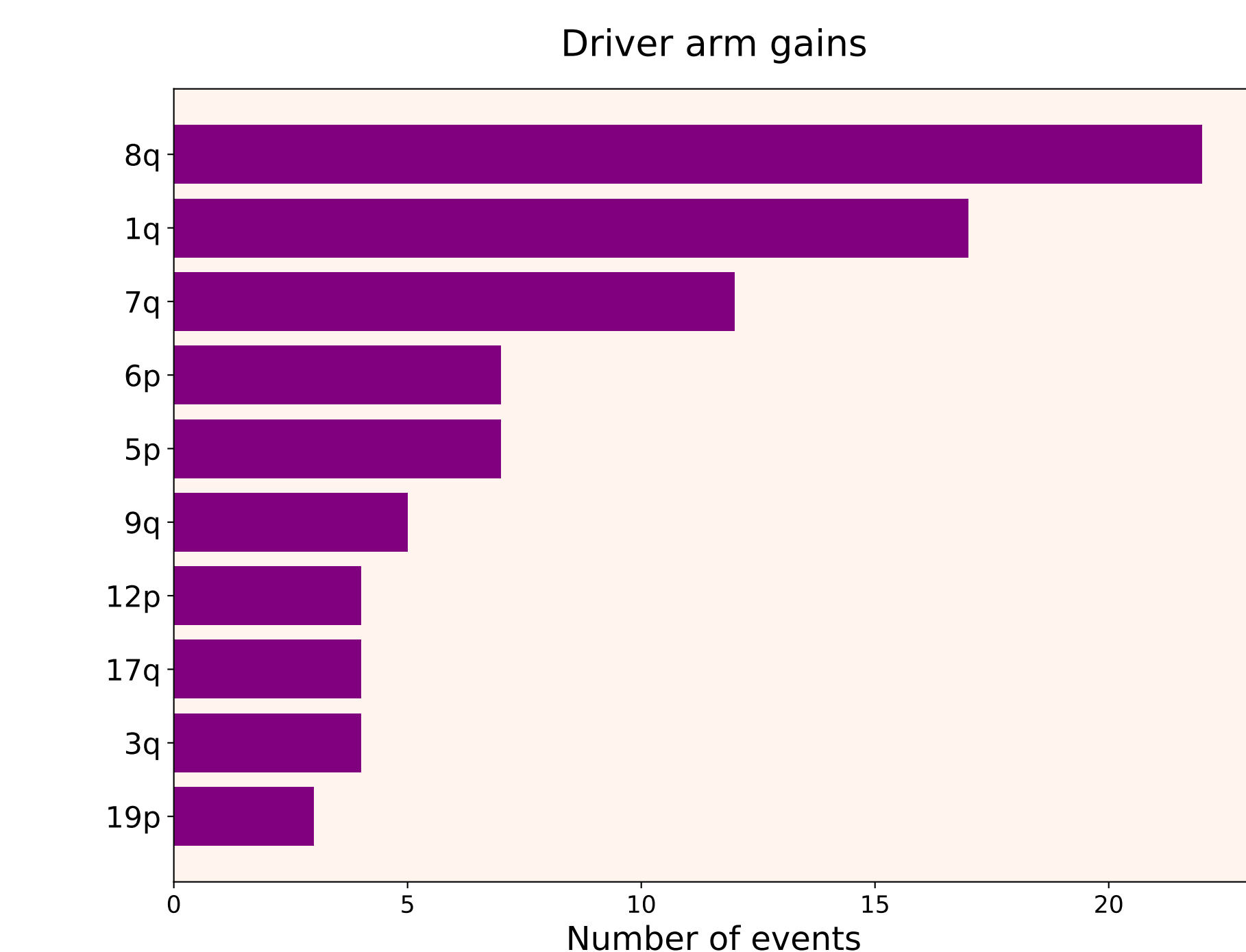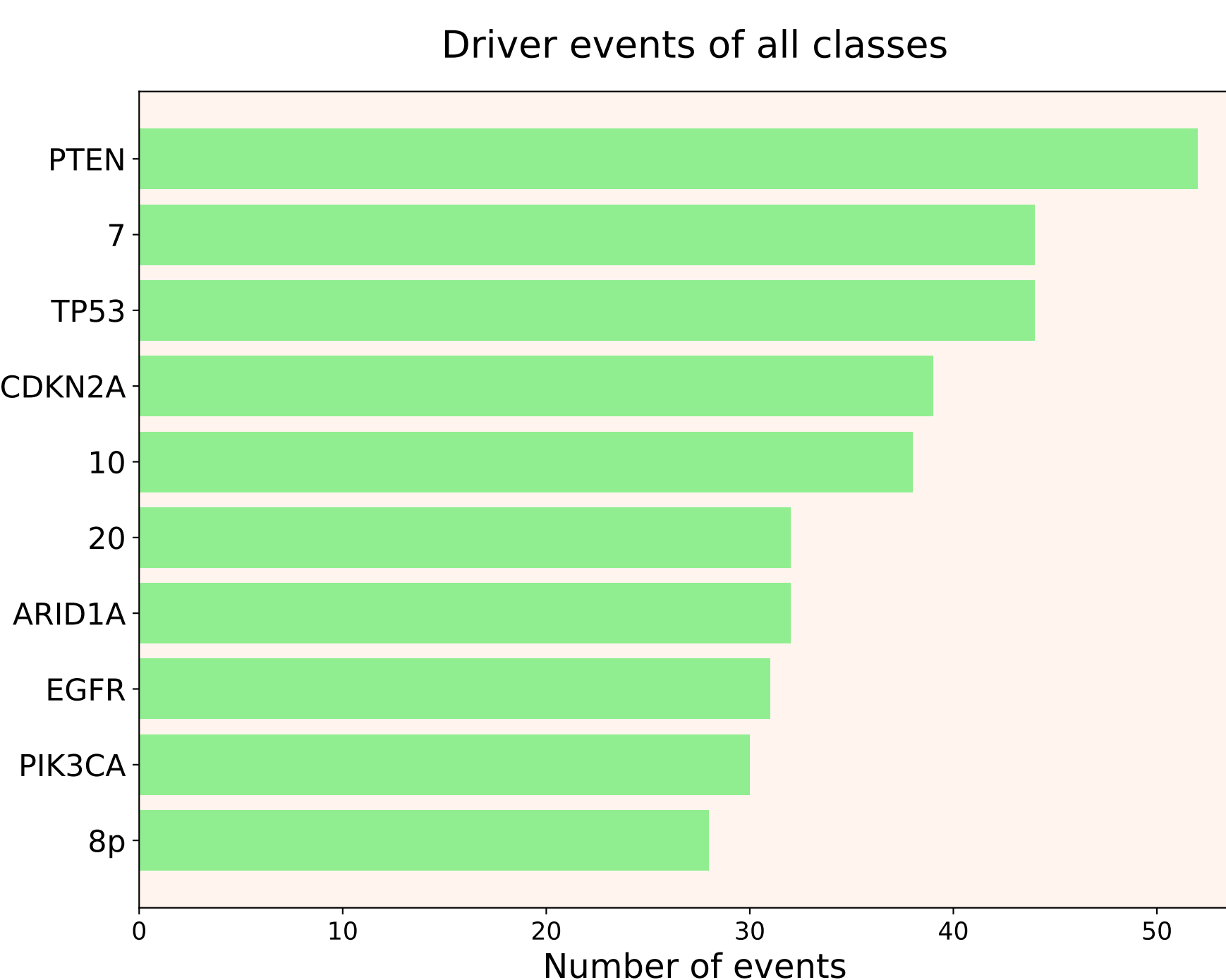

Supplement: Supplemental Information 2 [file peerj-10-13860-s002.zip › COHORTS/genes plots/detailed/2021_8_16_14_9_distribution_events_detailed_males_8.pdf]

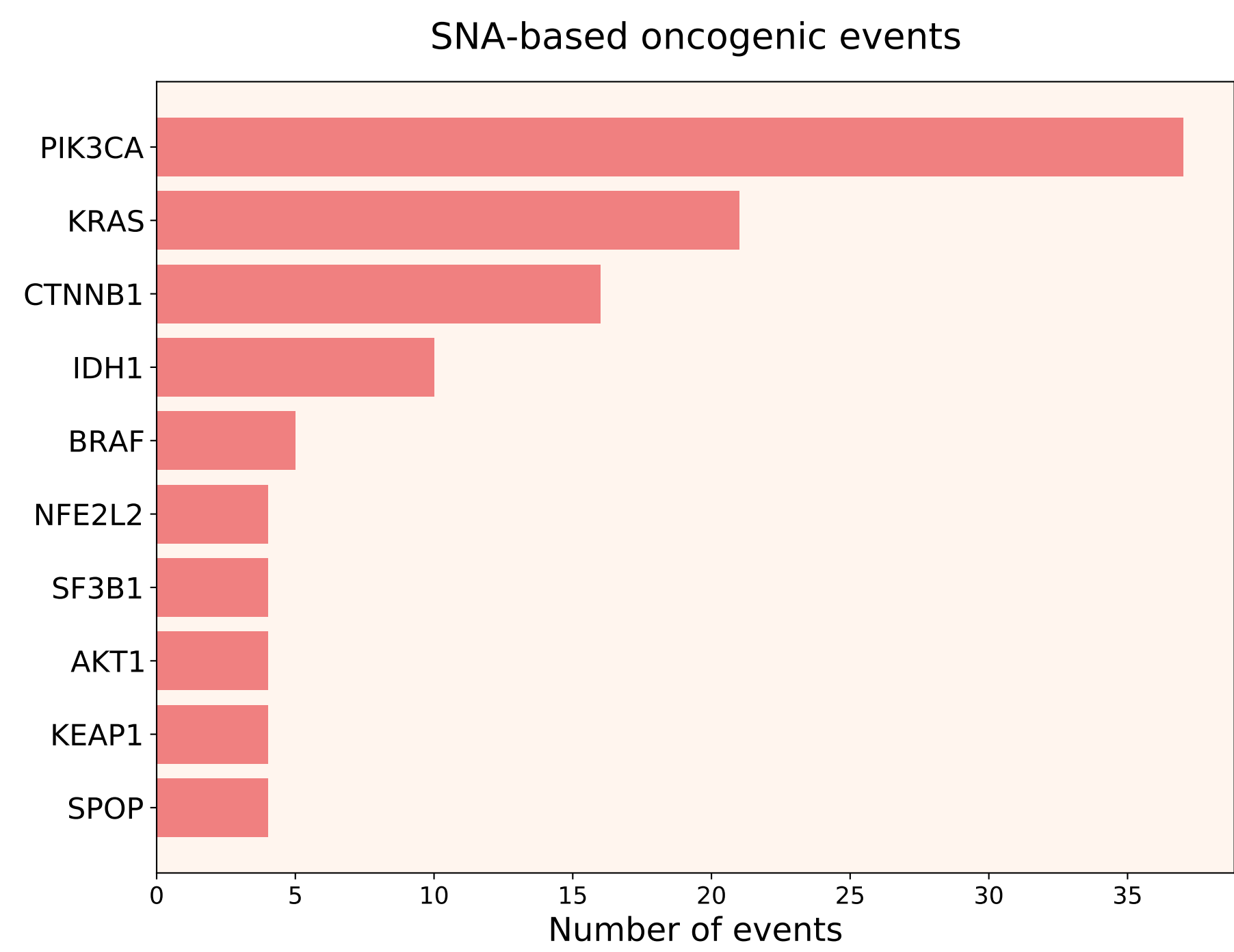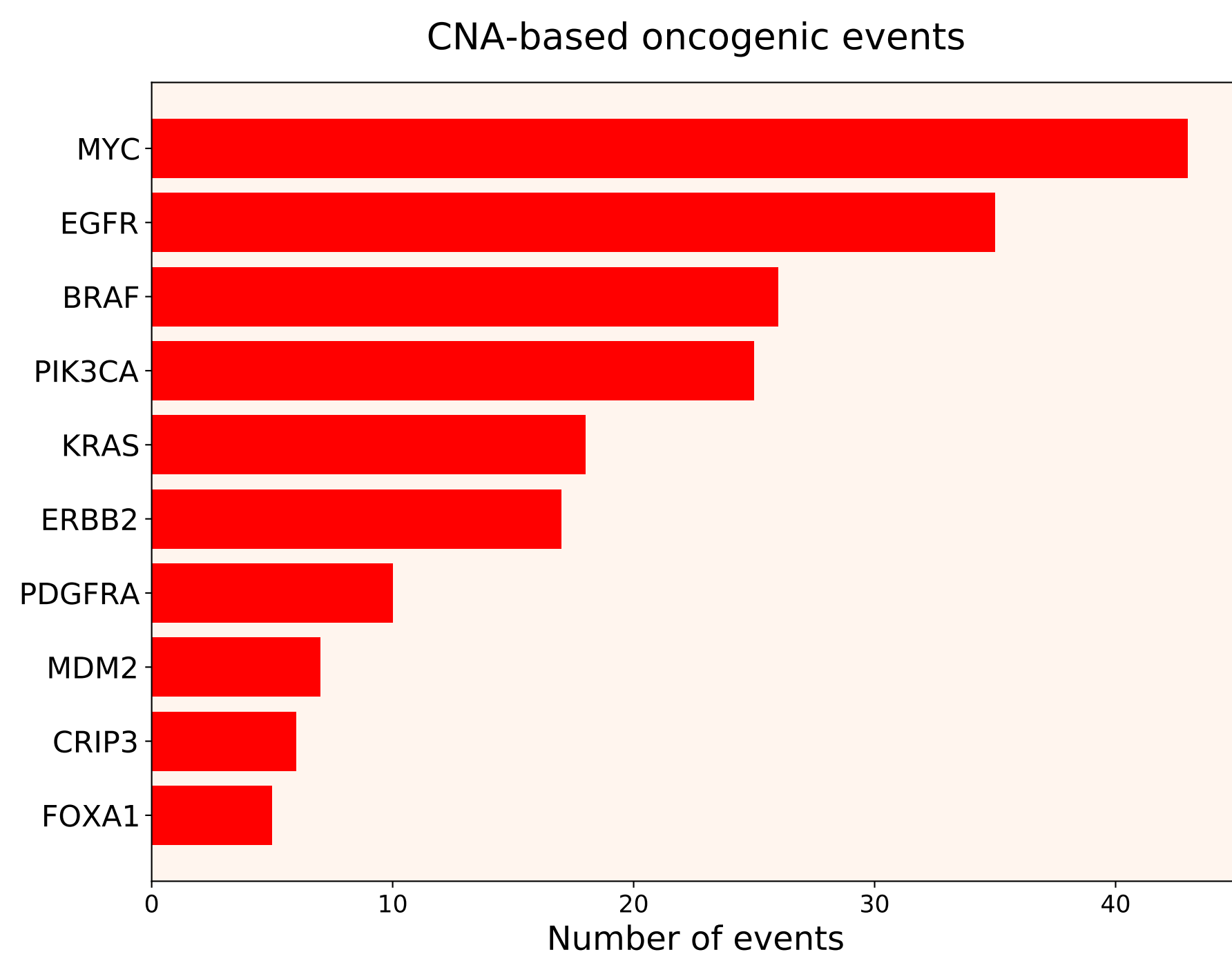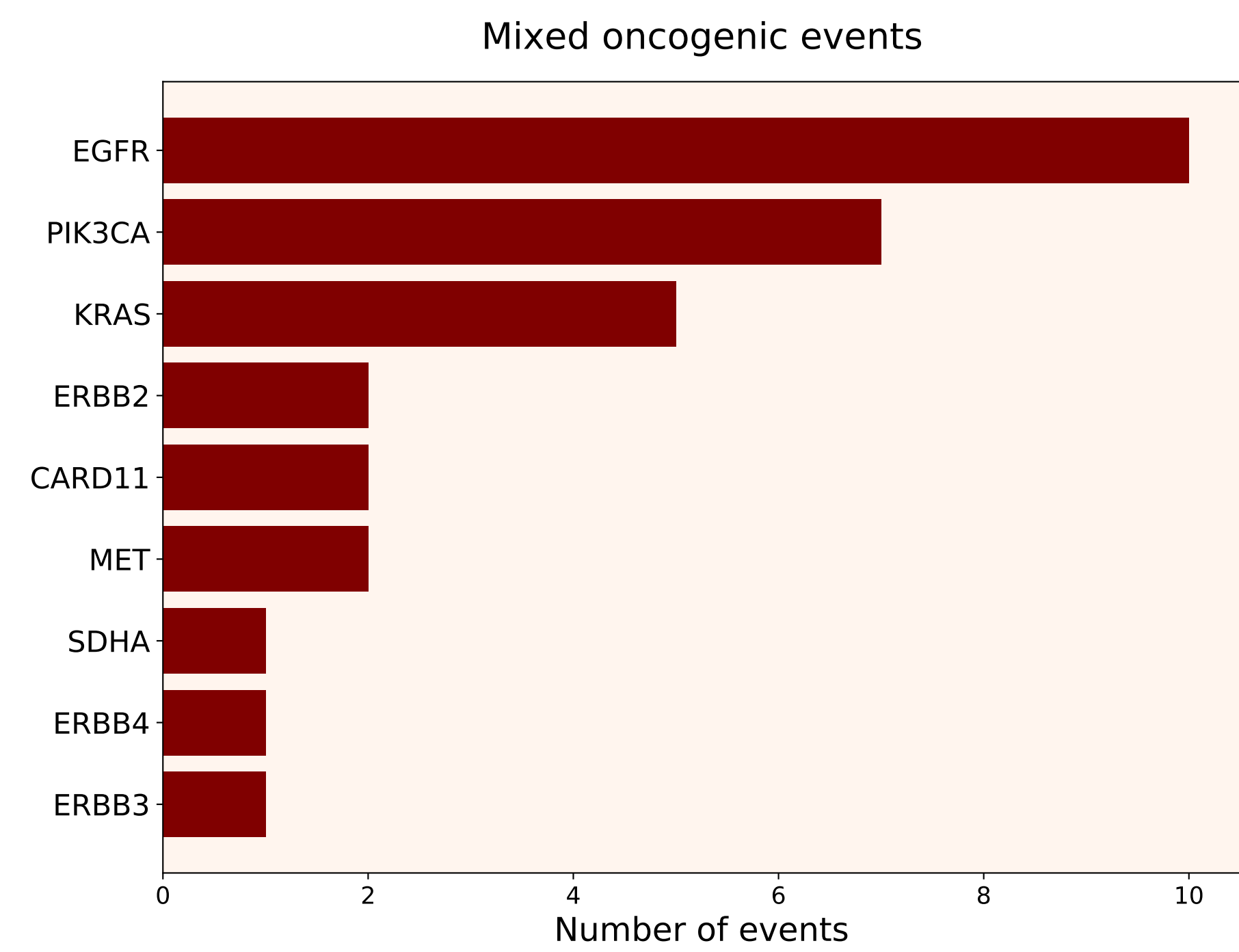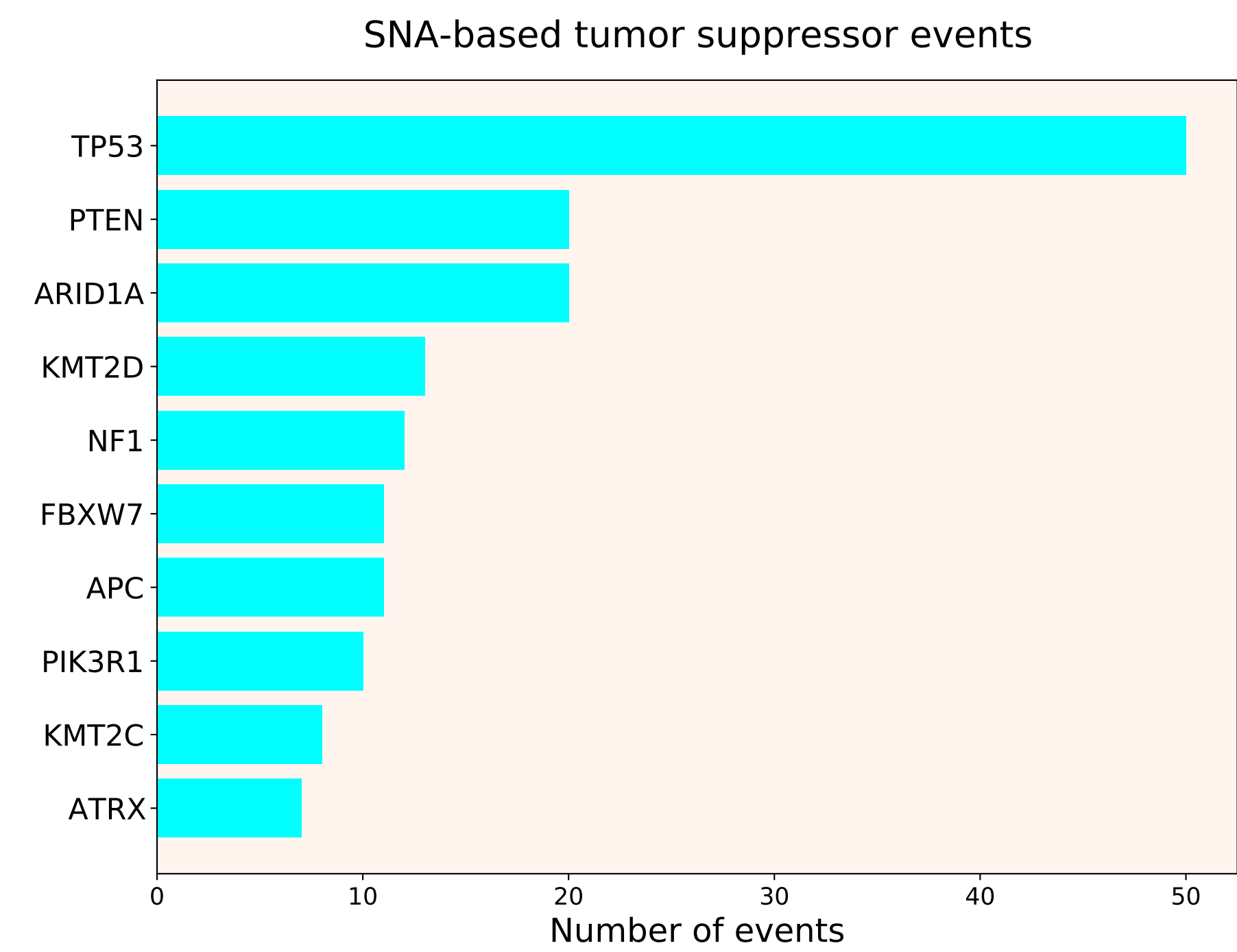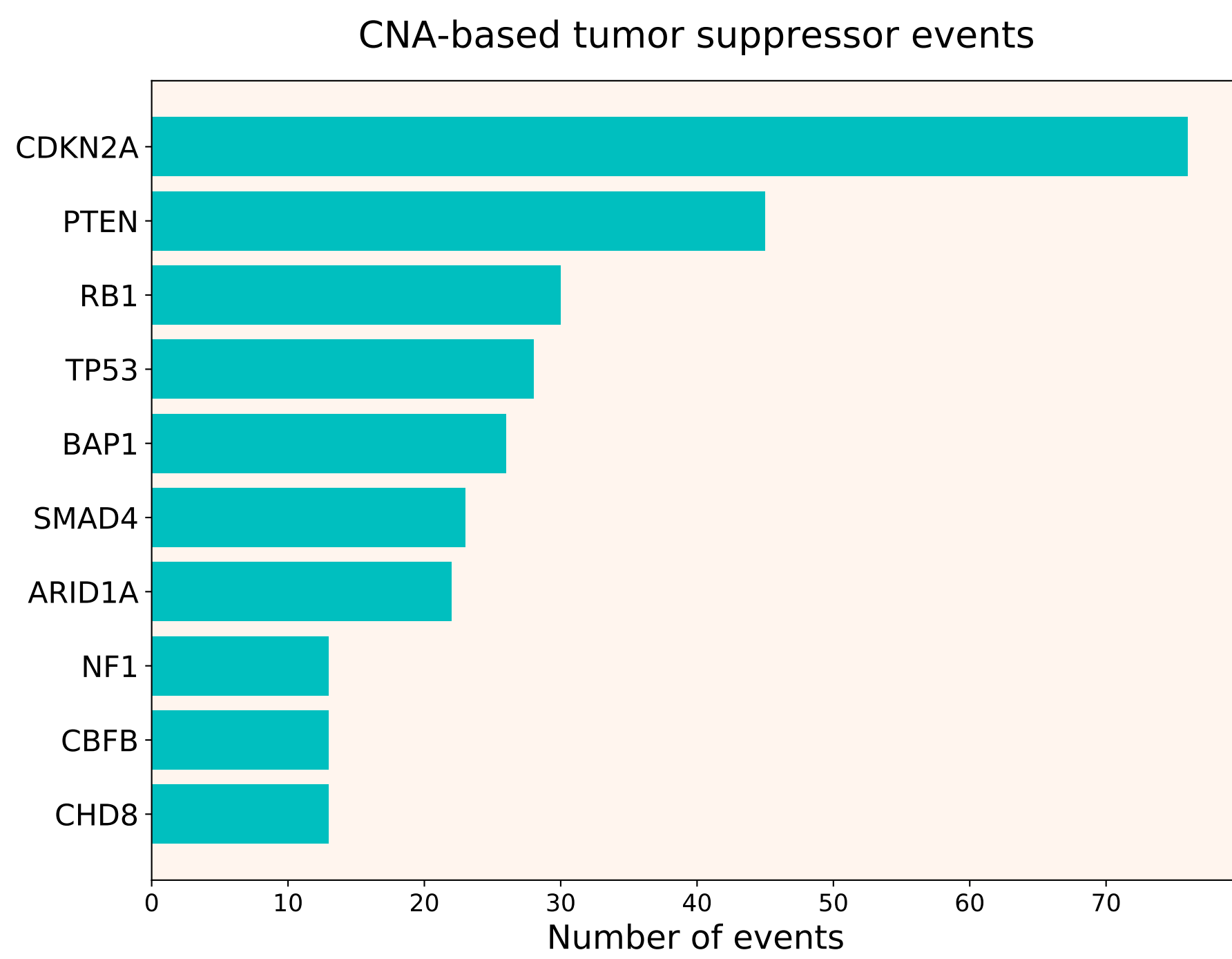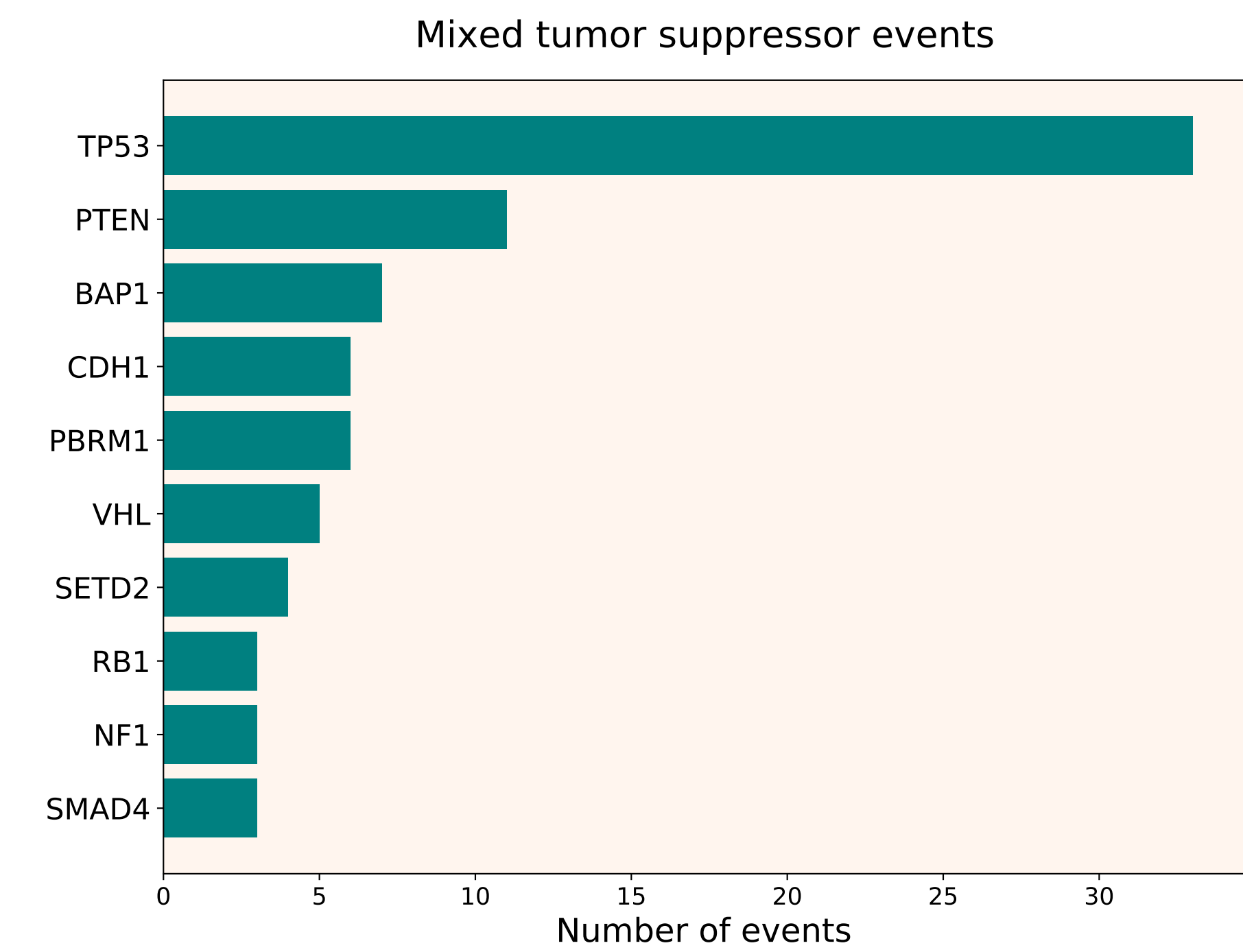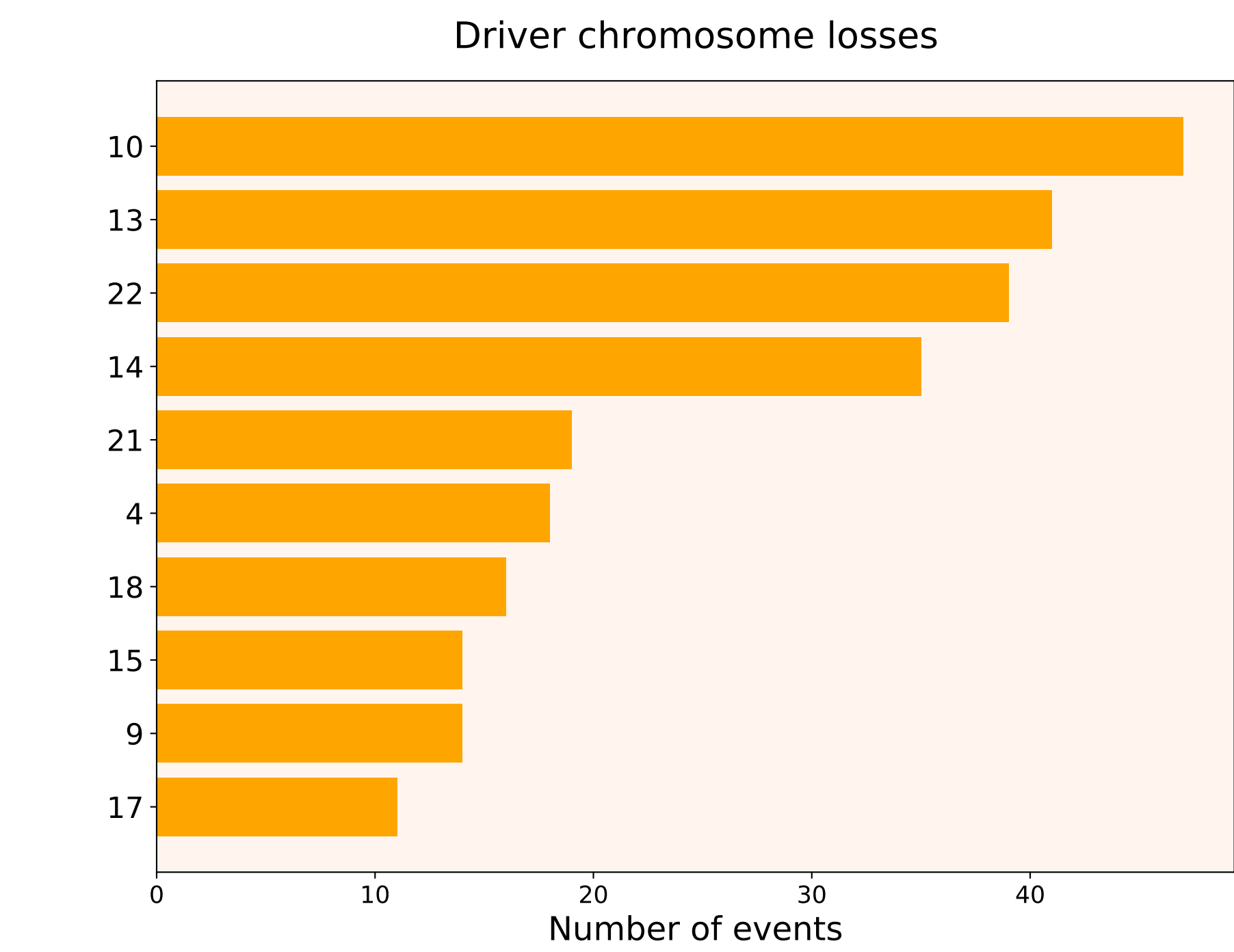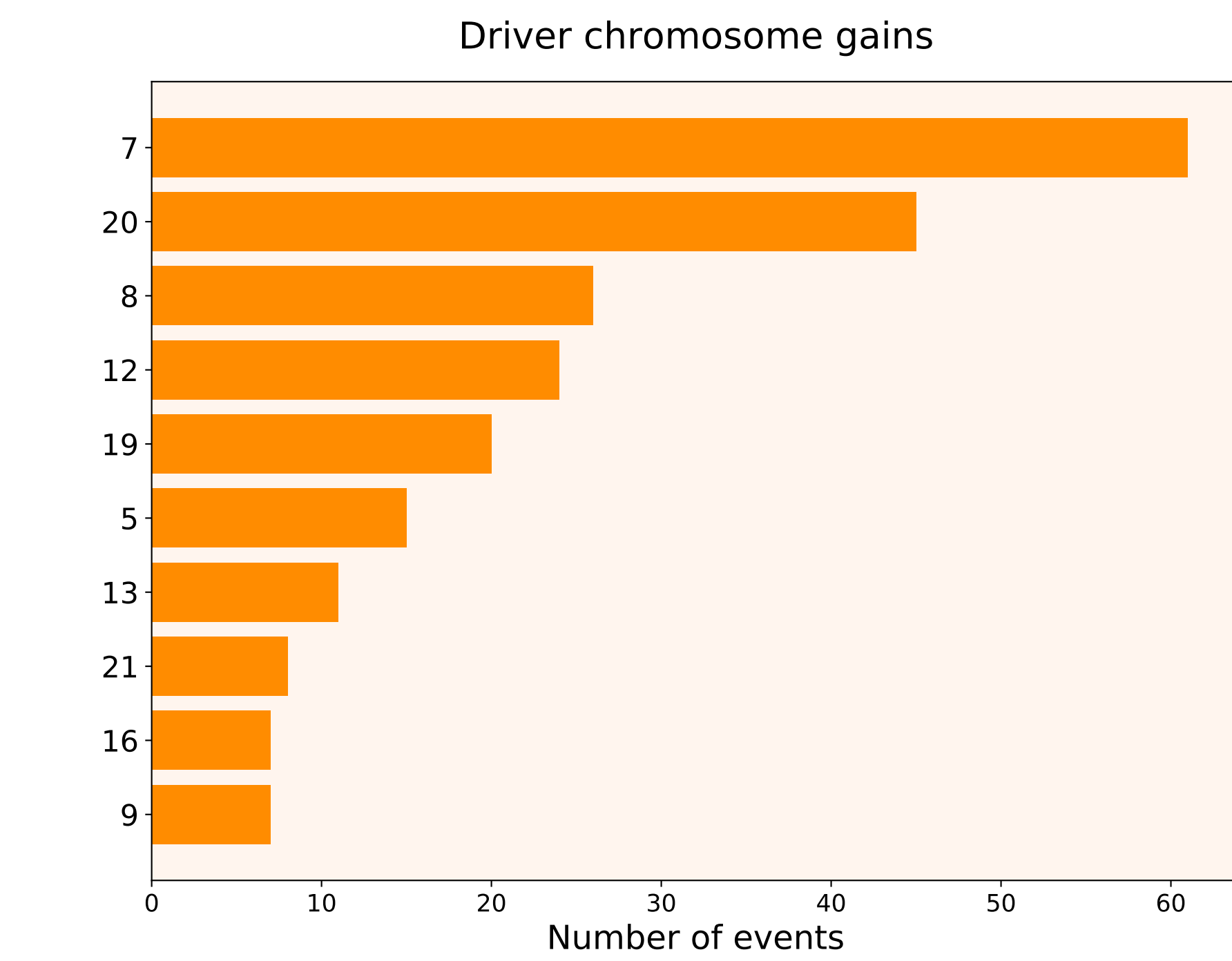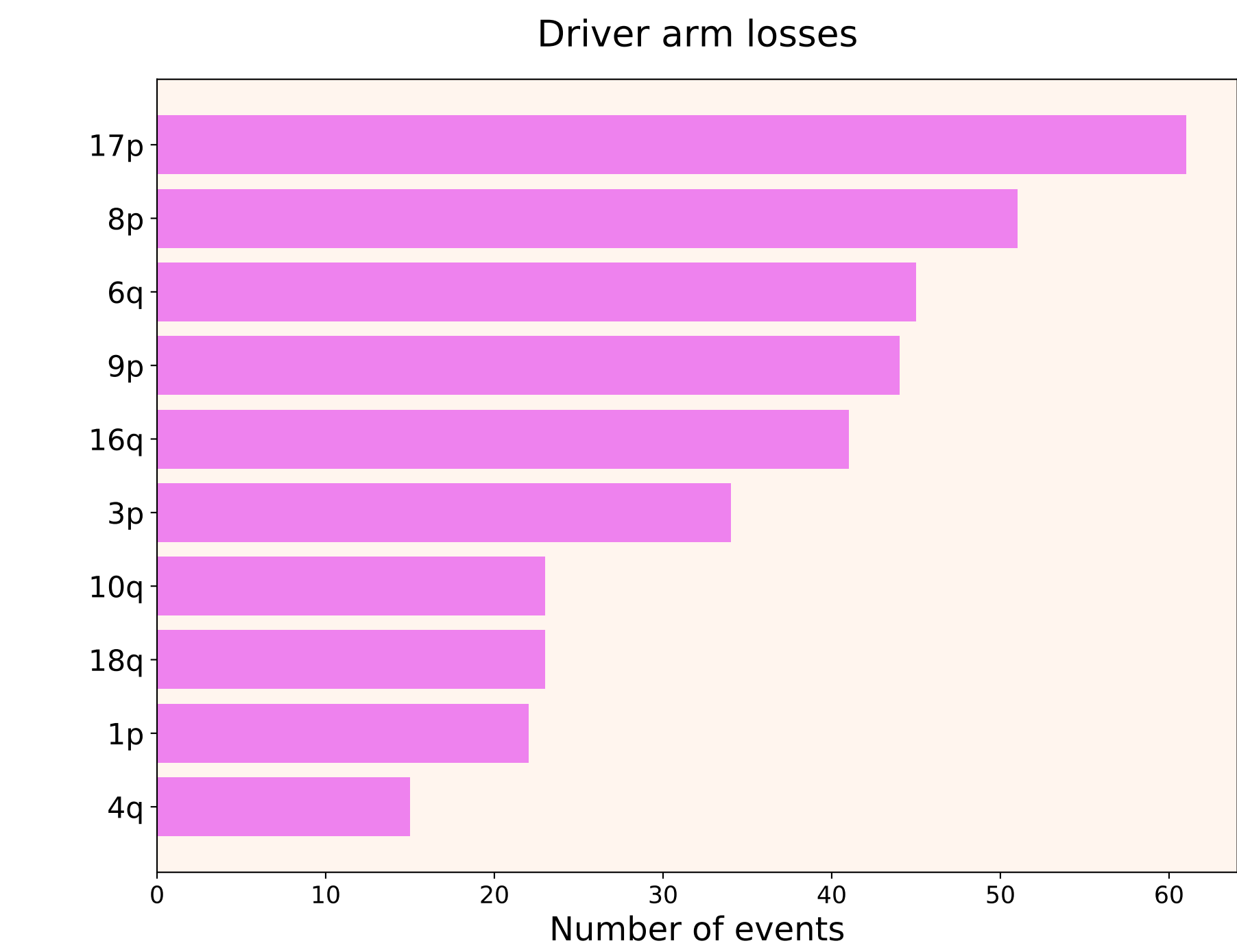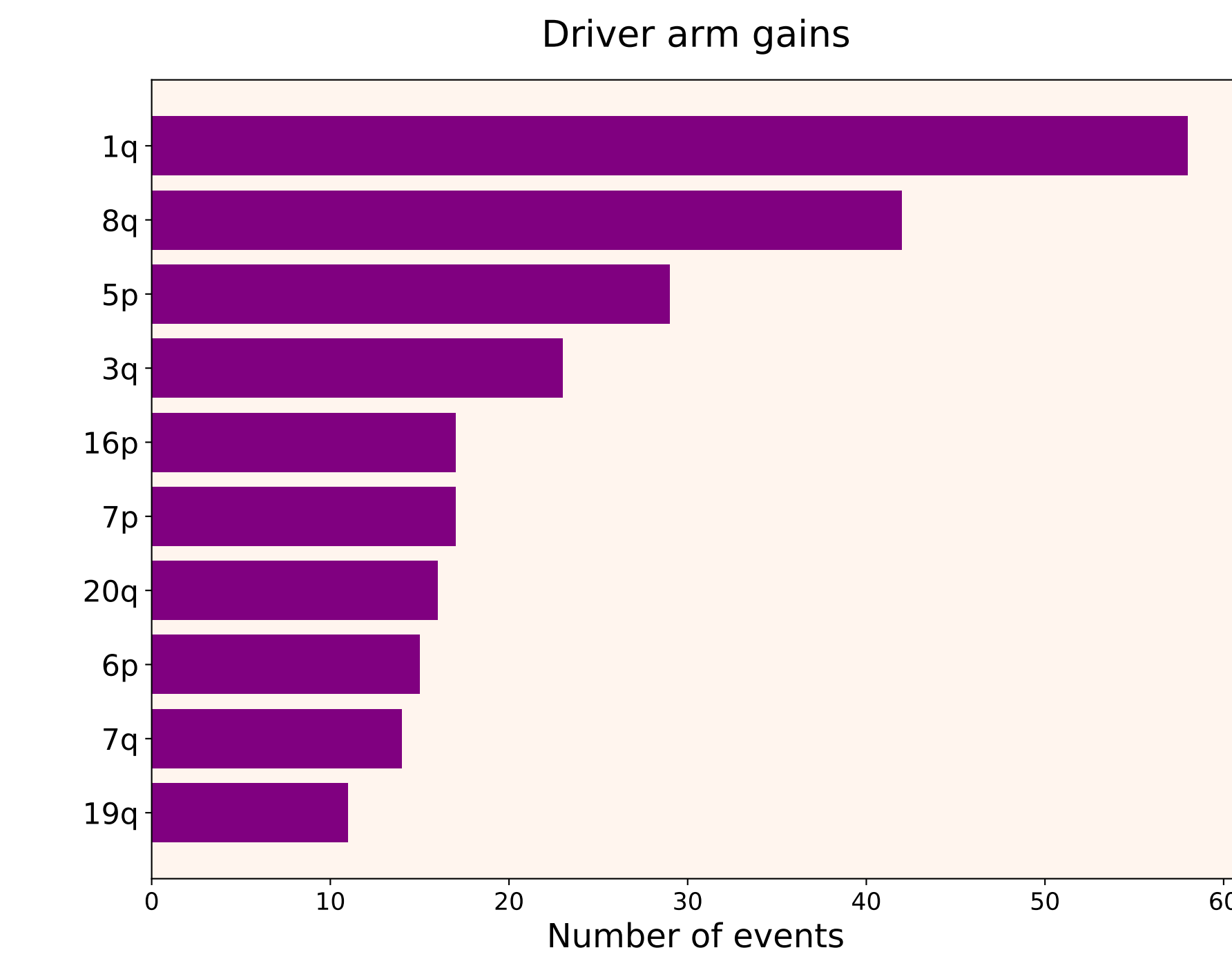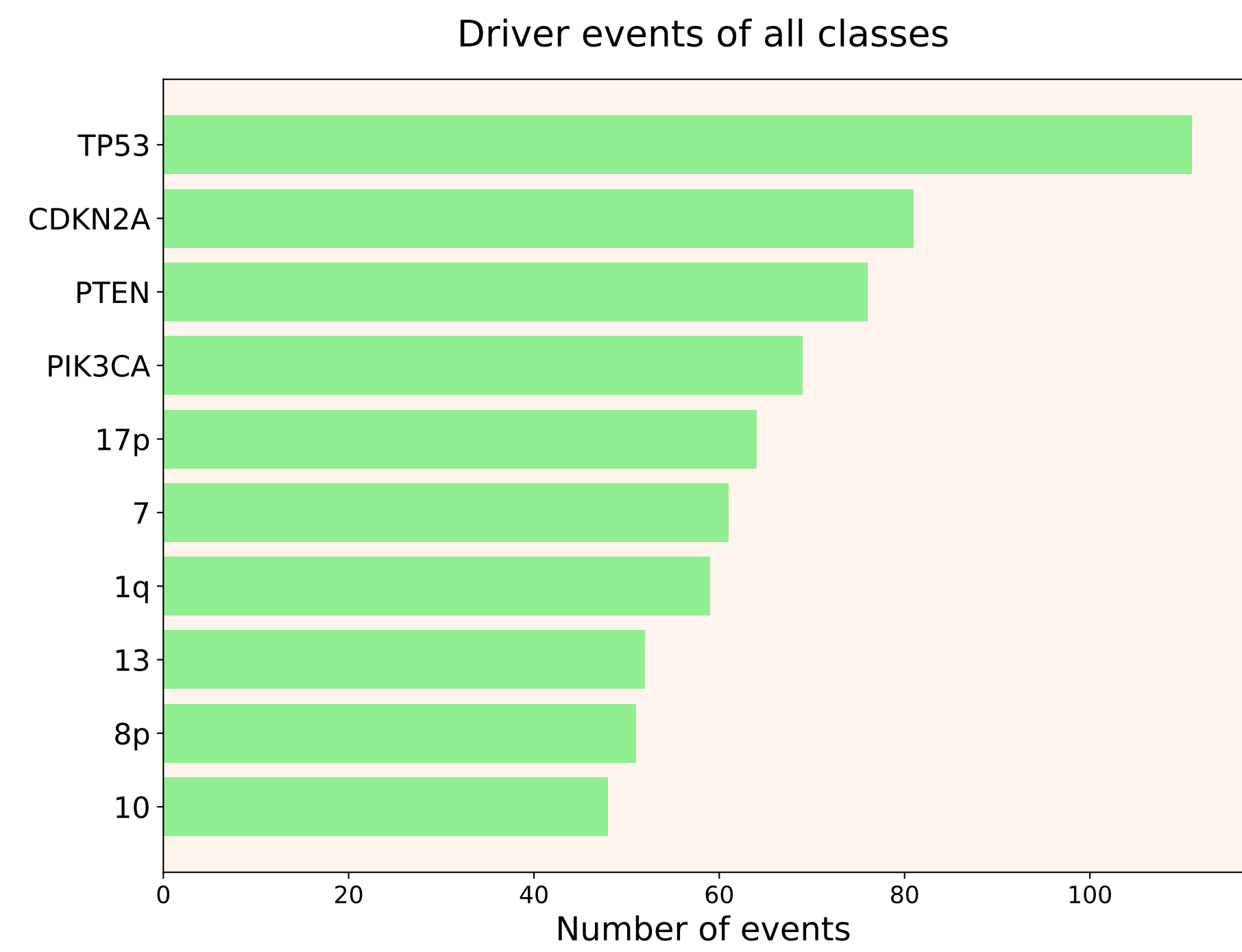

Supplement: Supplemental Information 2 [file peerj-10-13860-s002.zip › COHORTS/genes plots/detailed/2021_8_16_14_9_distribution_events_detailed_10.pdf]

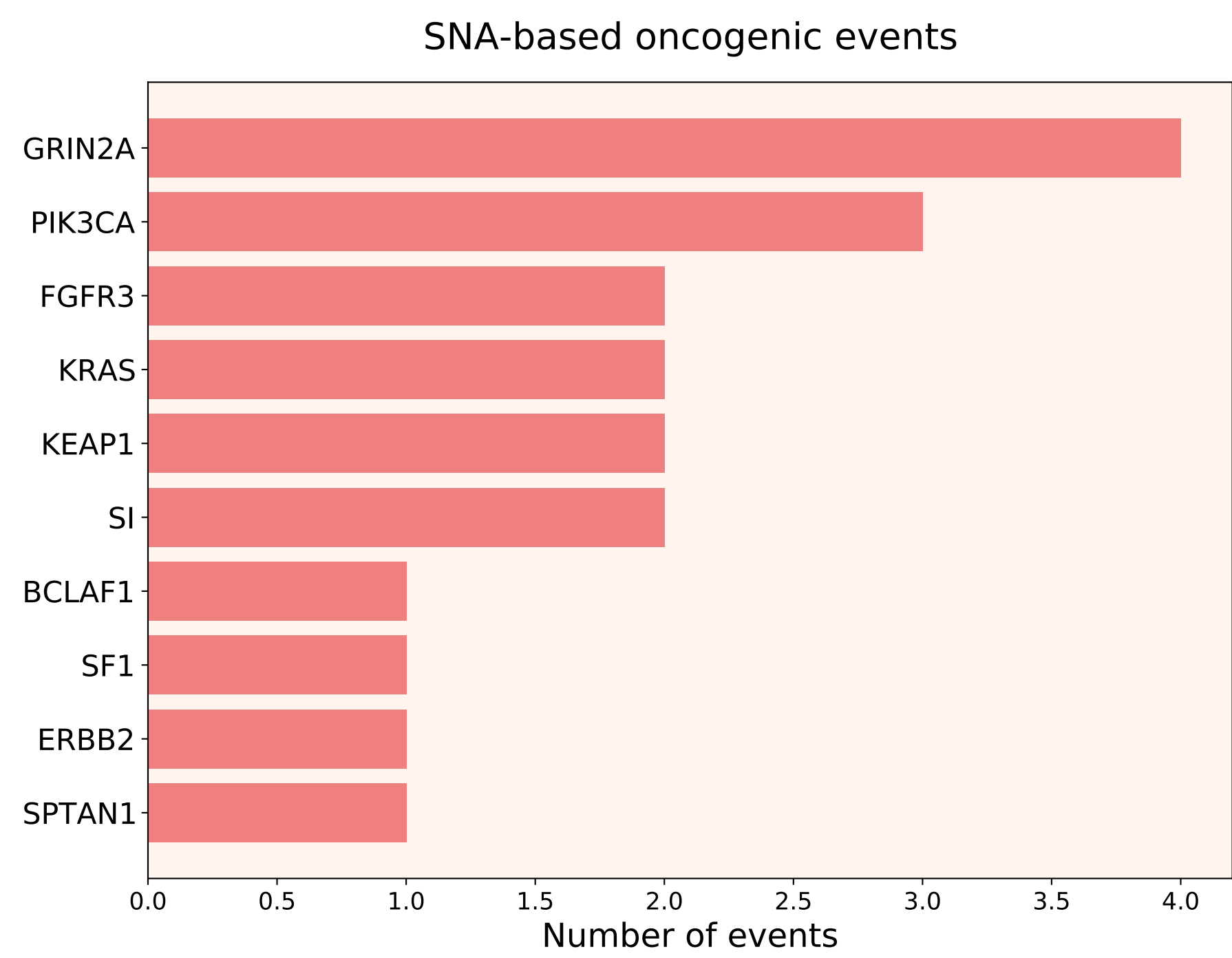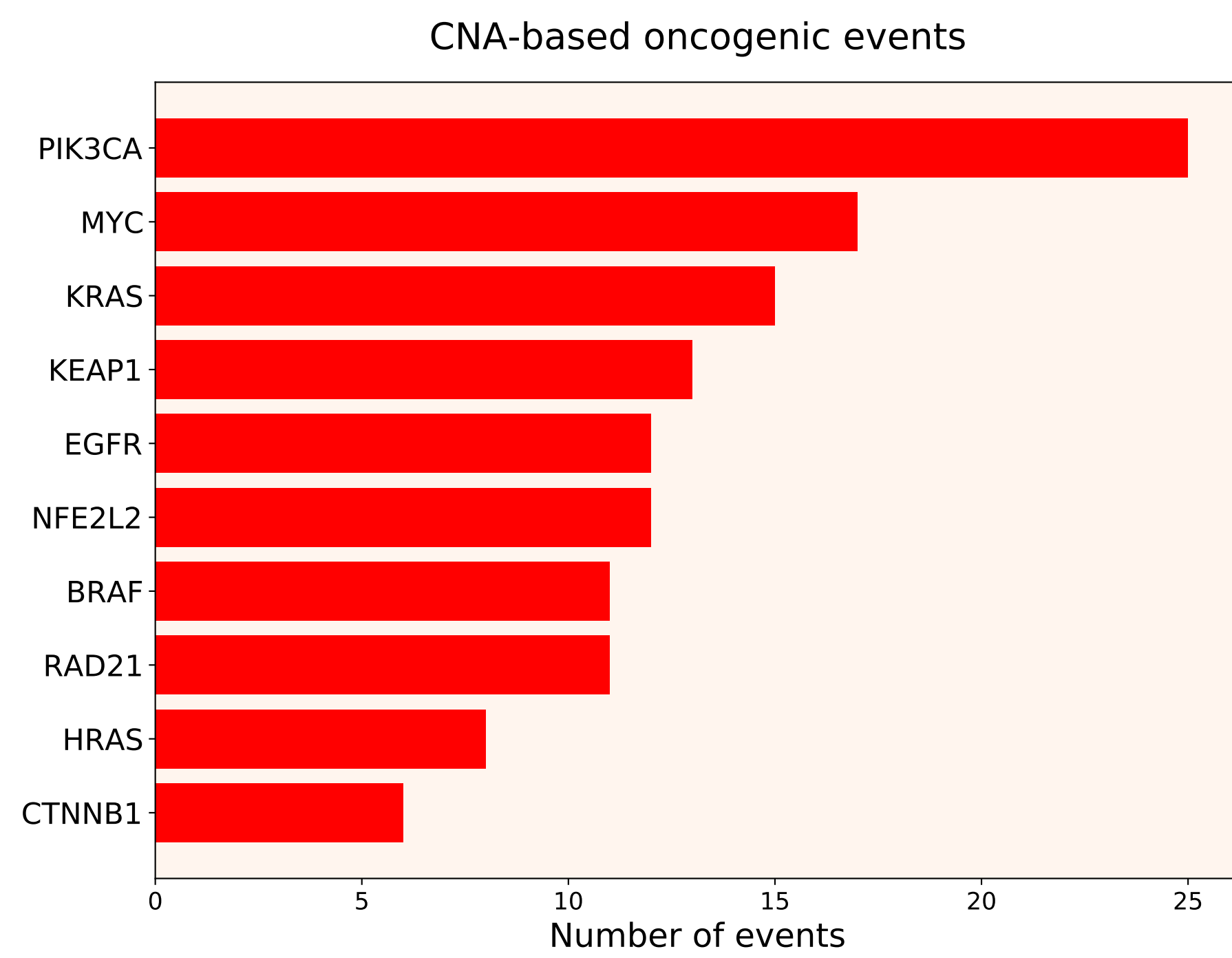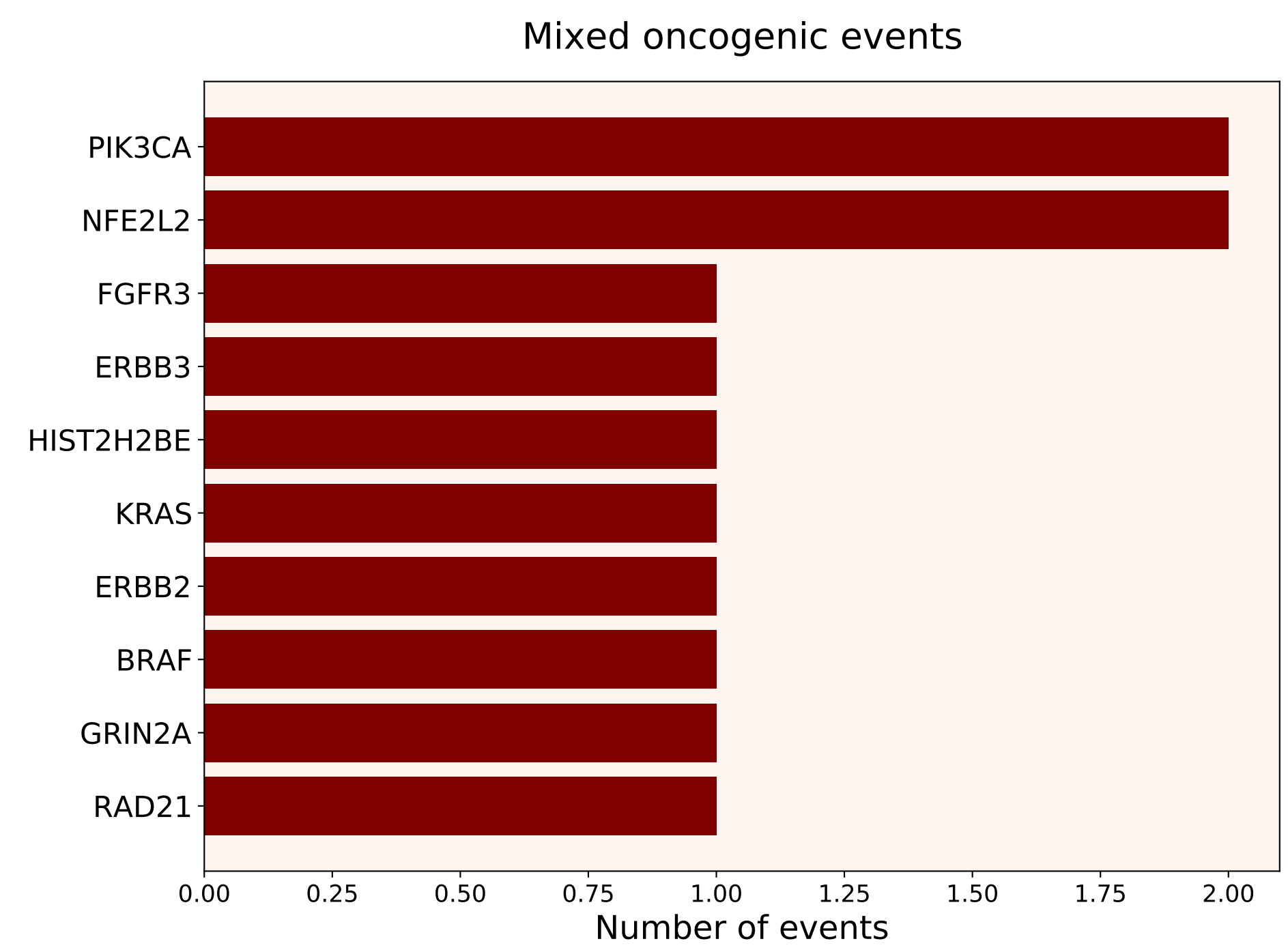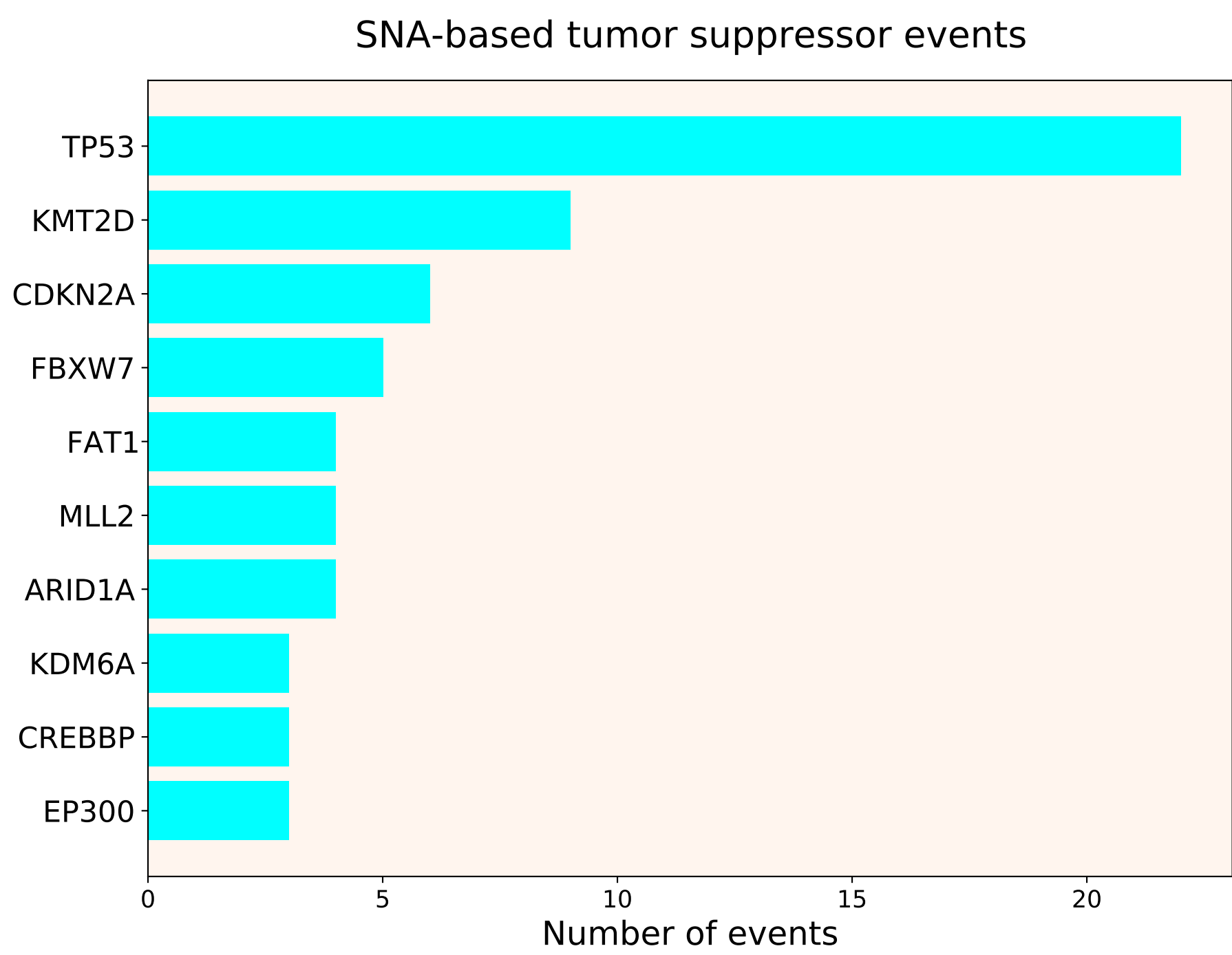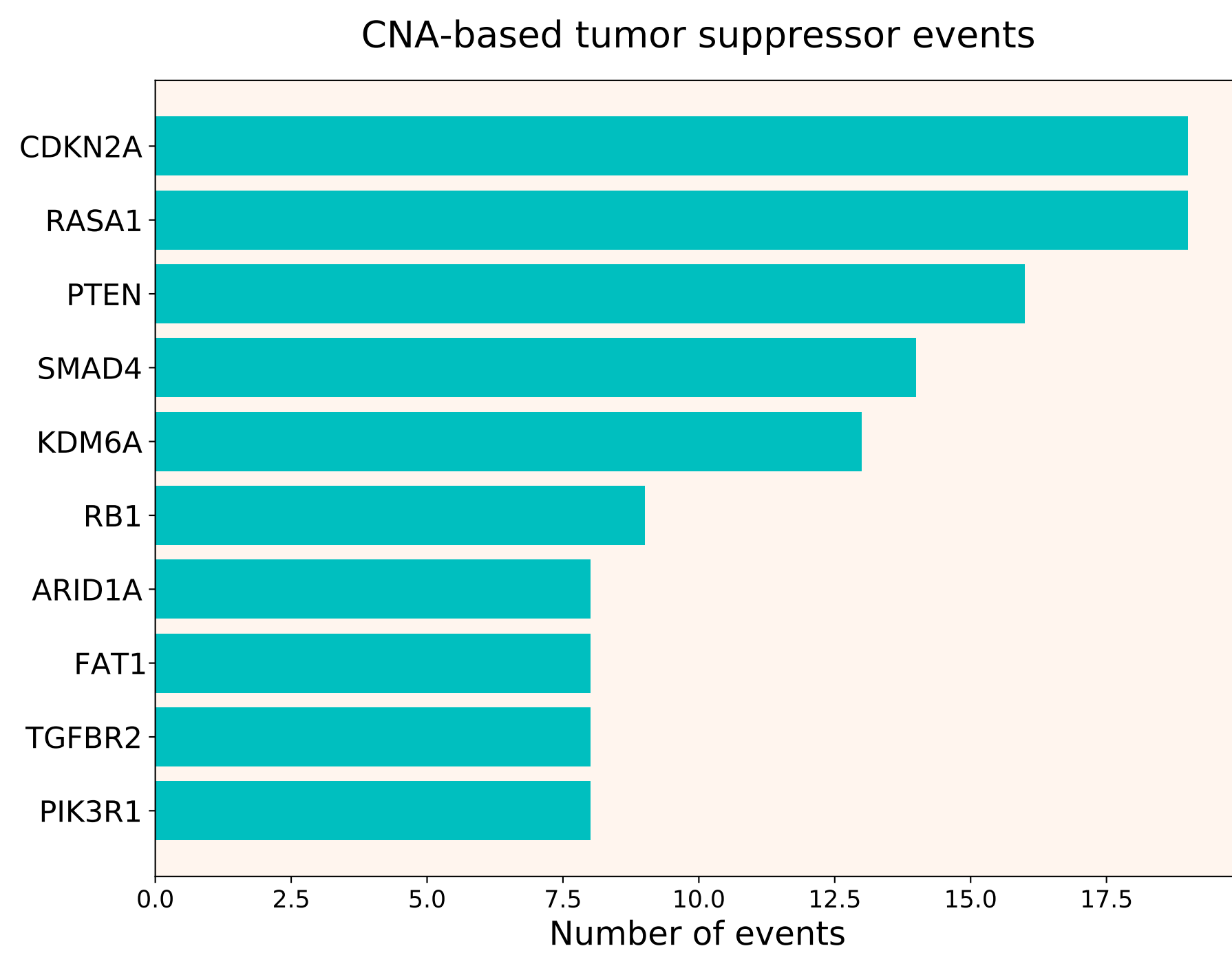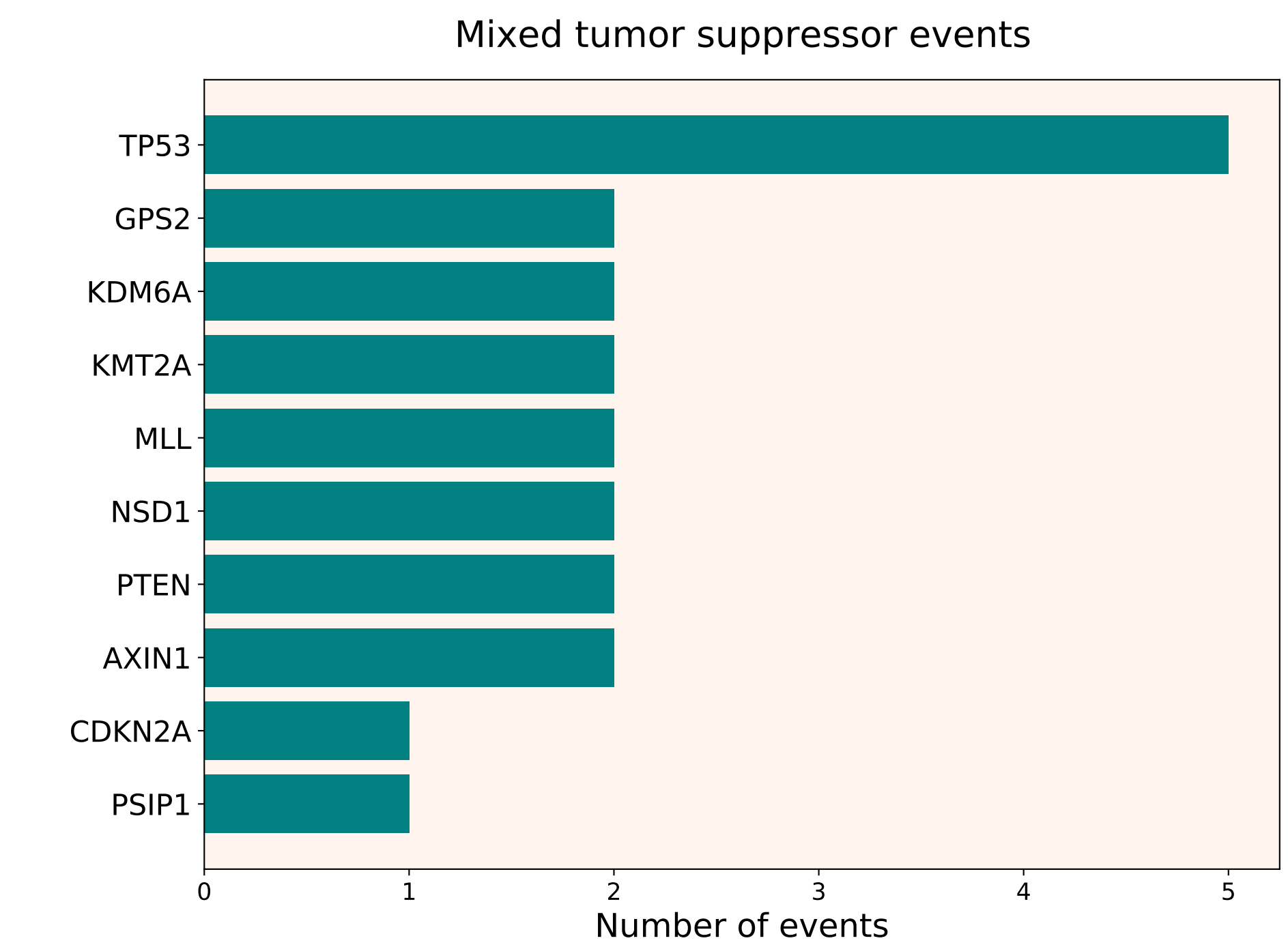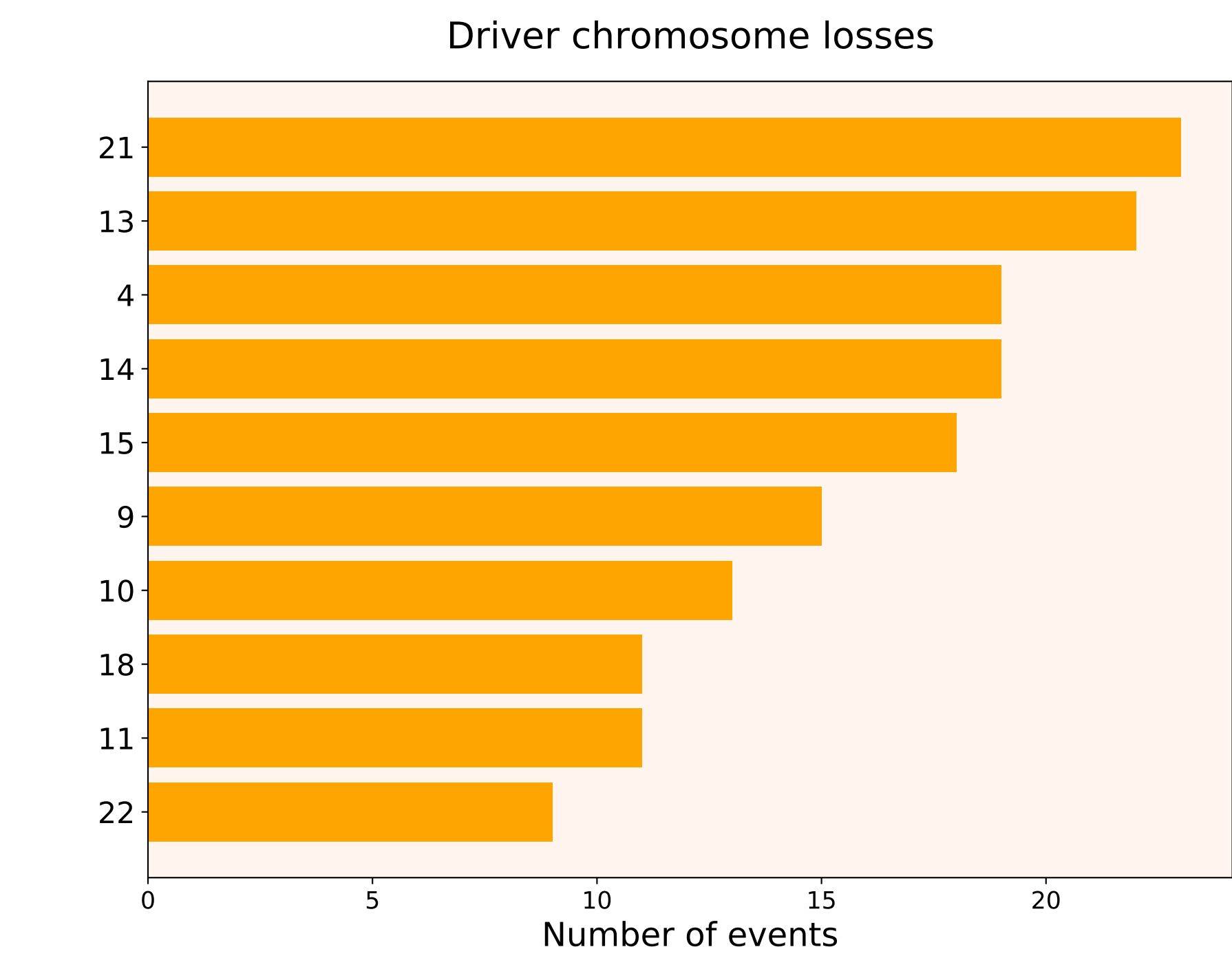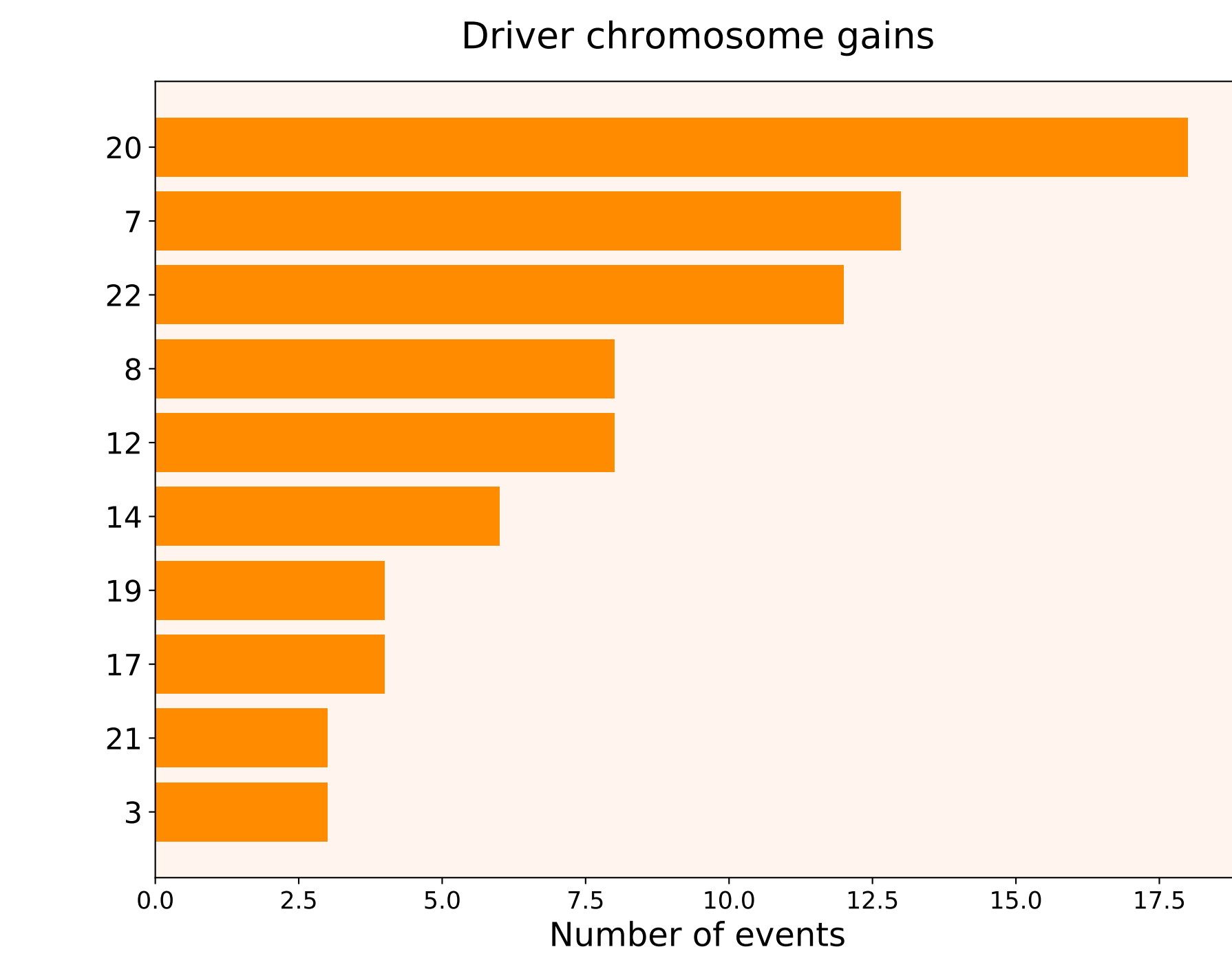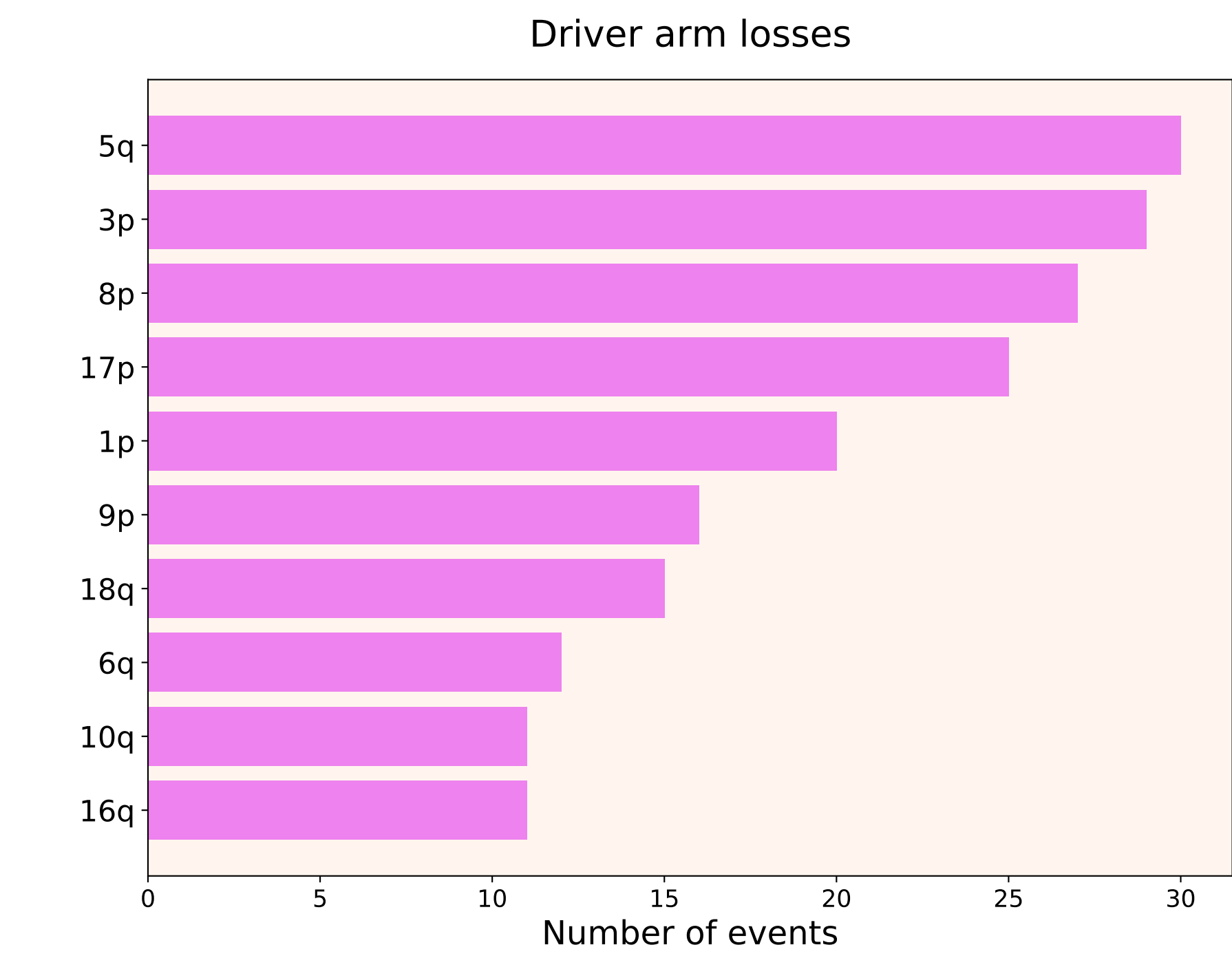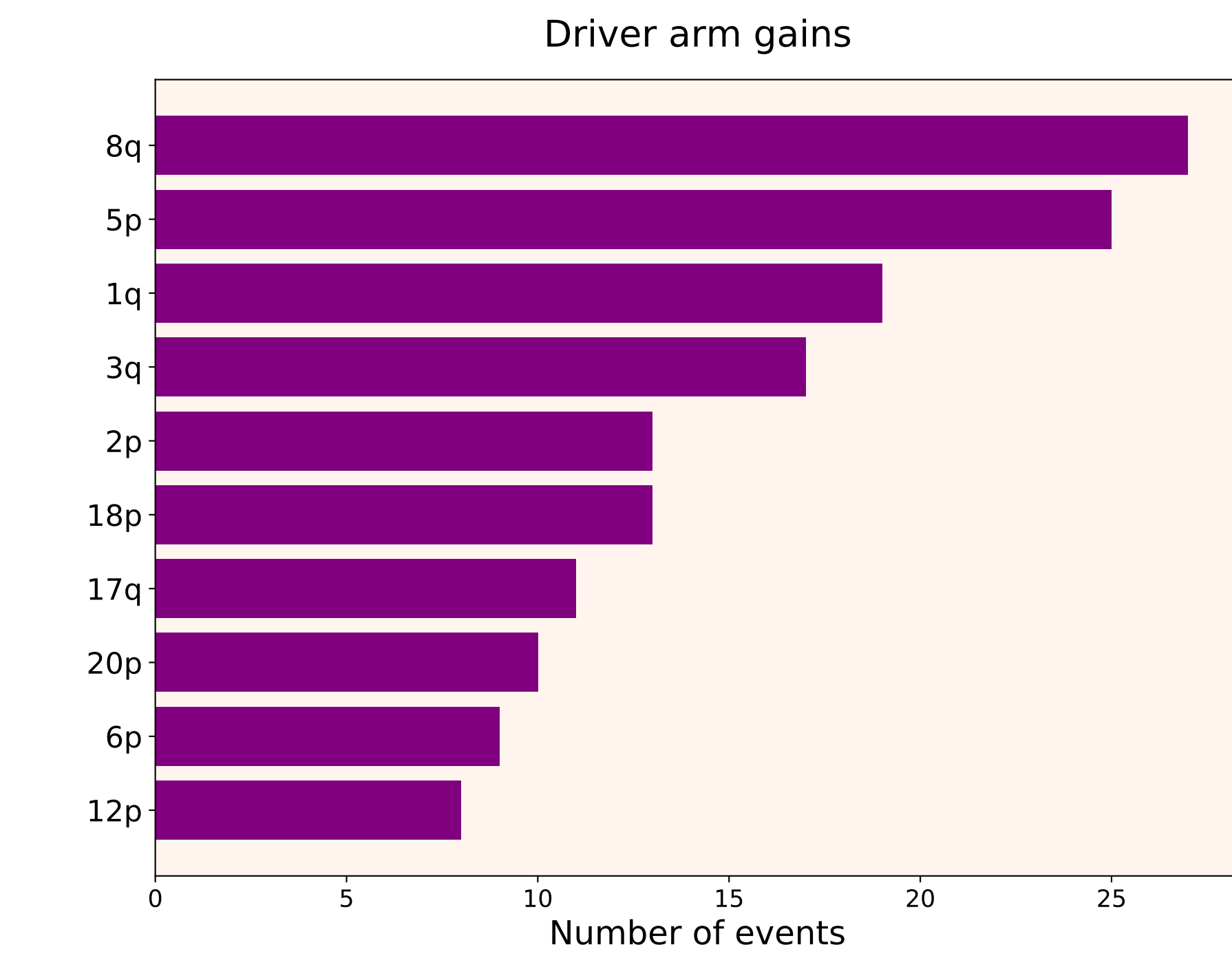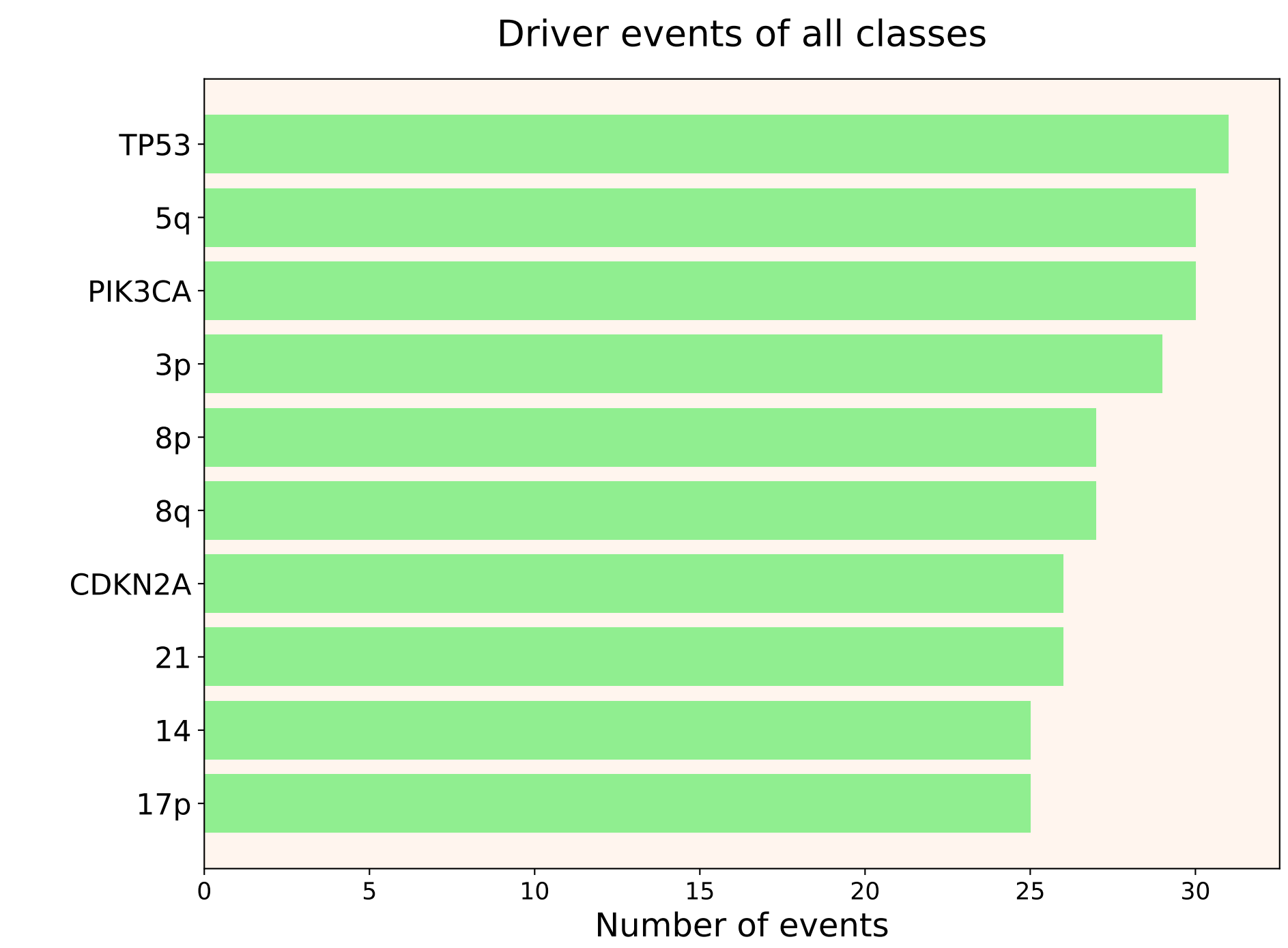

Supplement: Supplemental Information 2 [file peerj-10-13860-s002.zip › COHORTS/genes plots/detailed/2021_8_16_14_9_distribution_events_detailed_males_25.pdf]

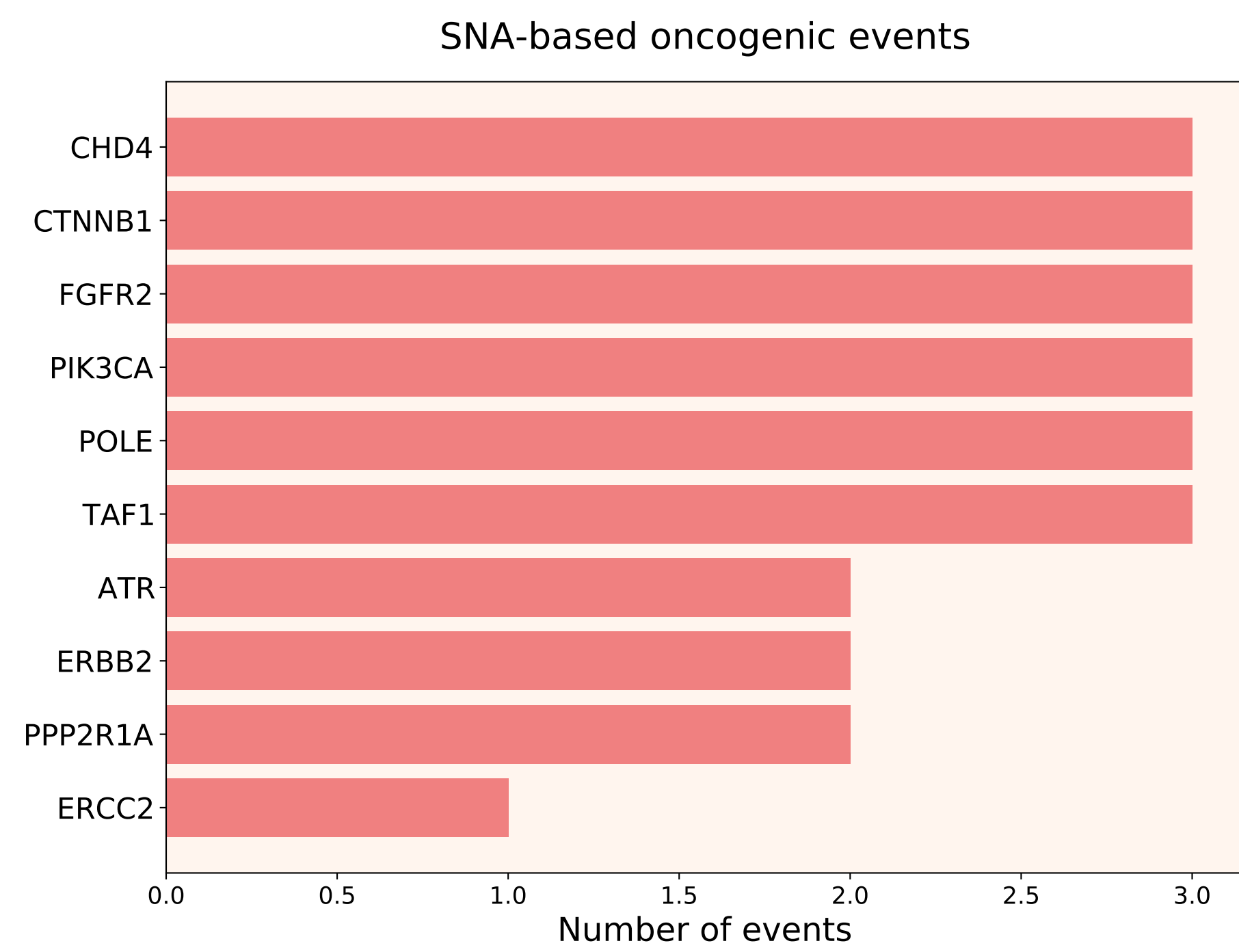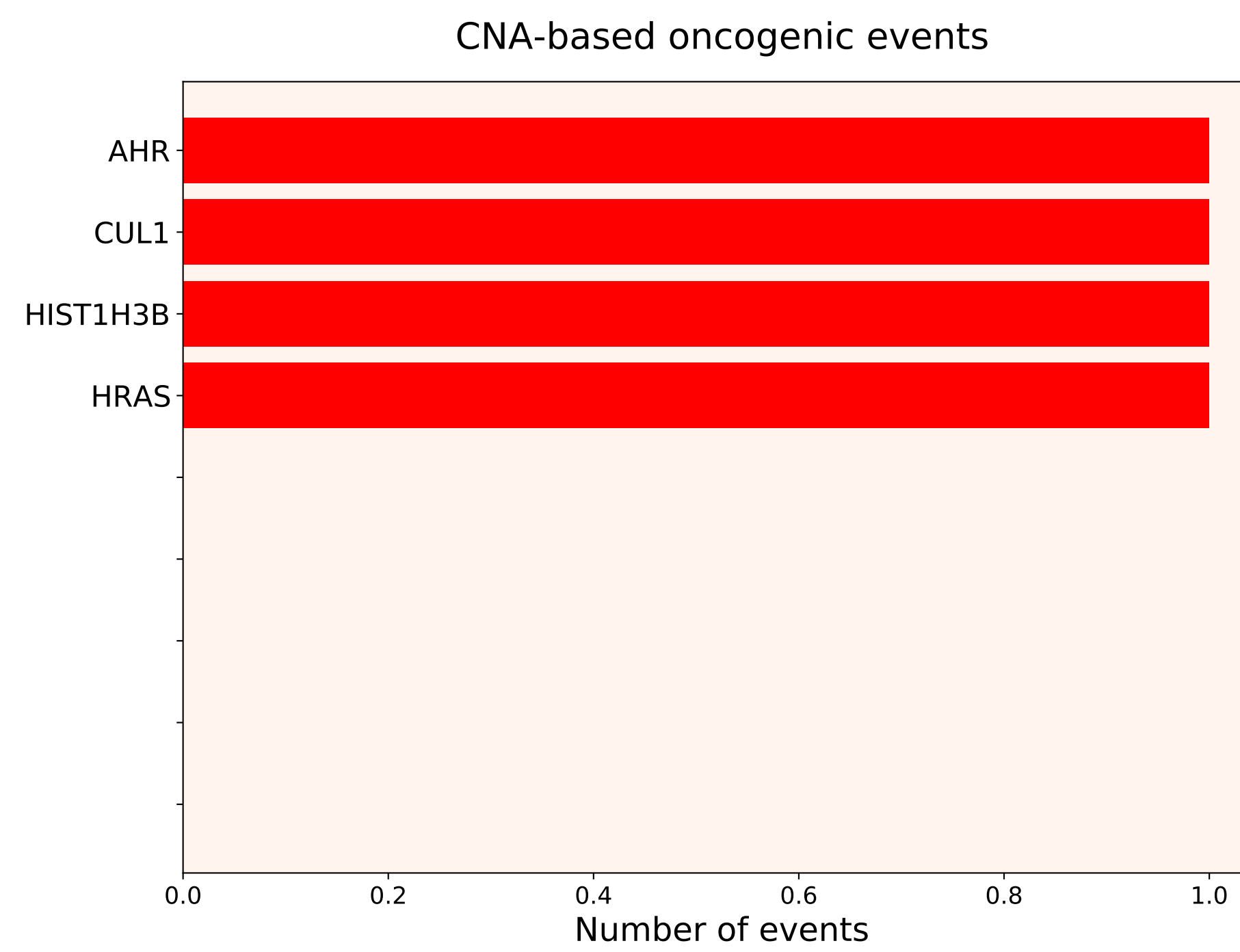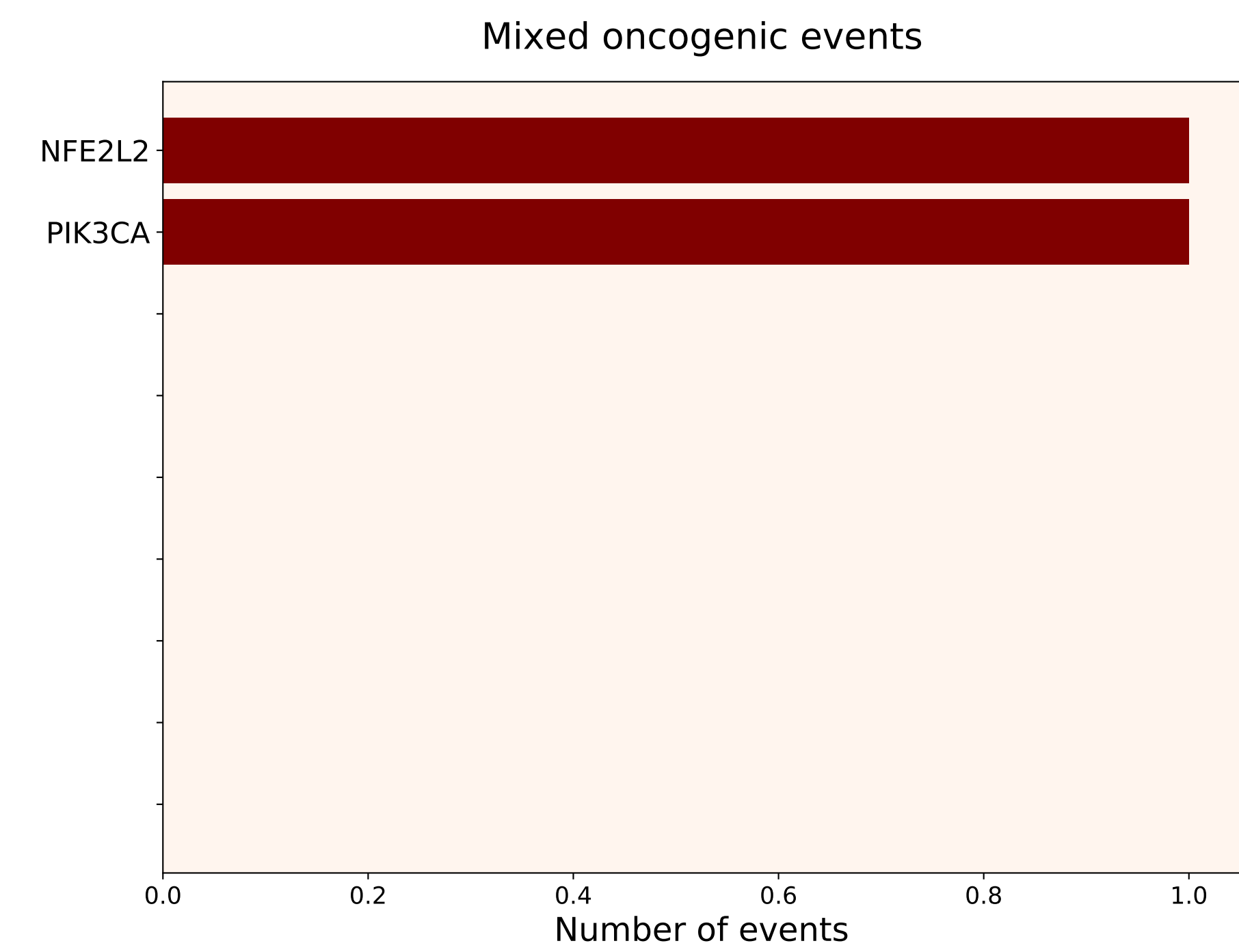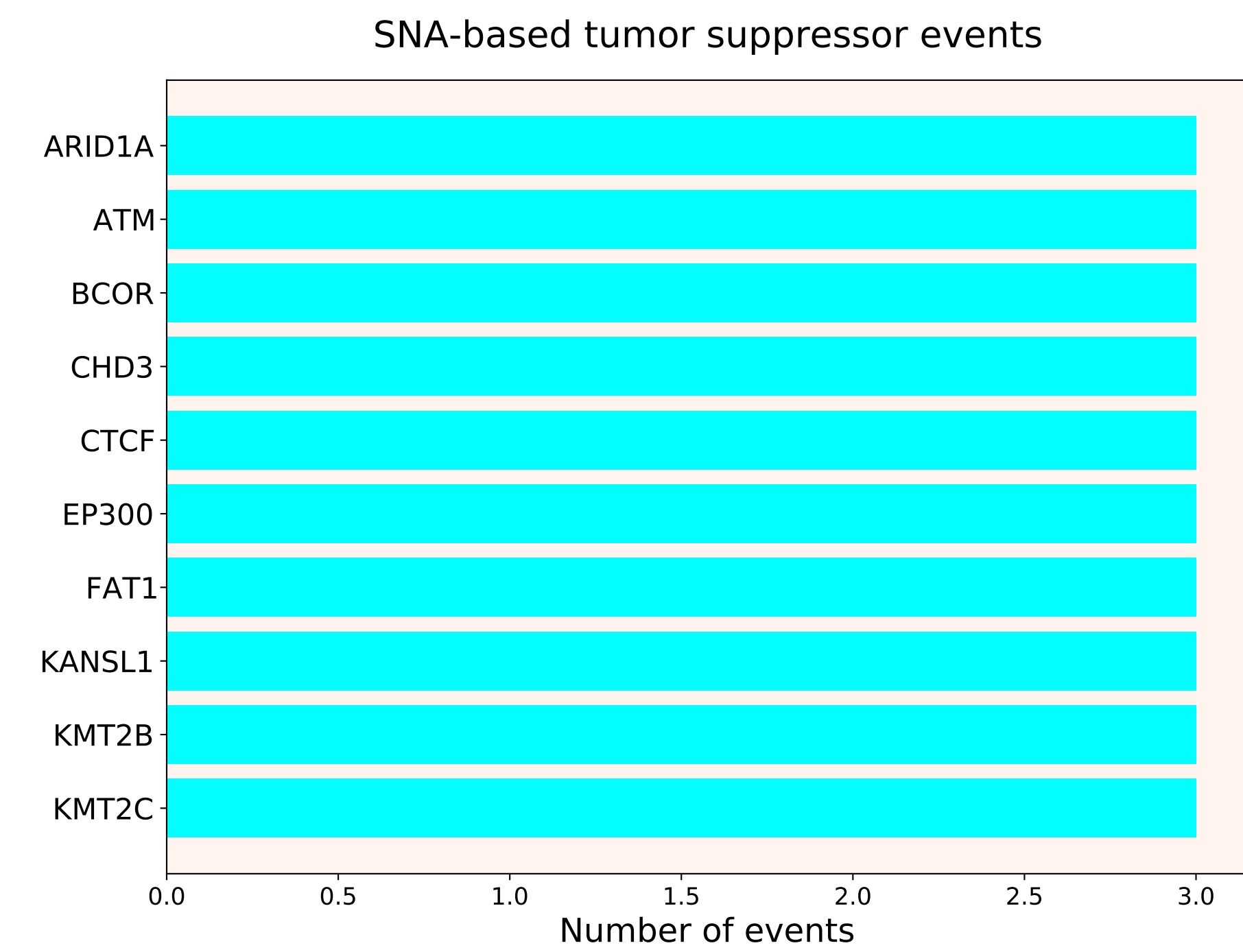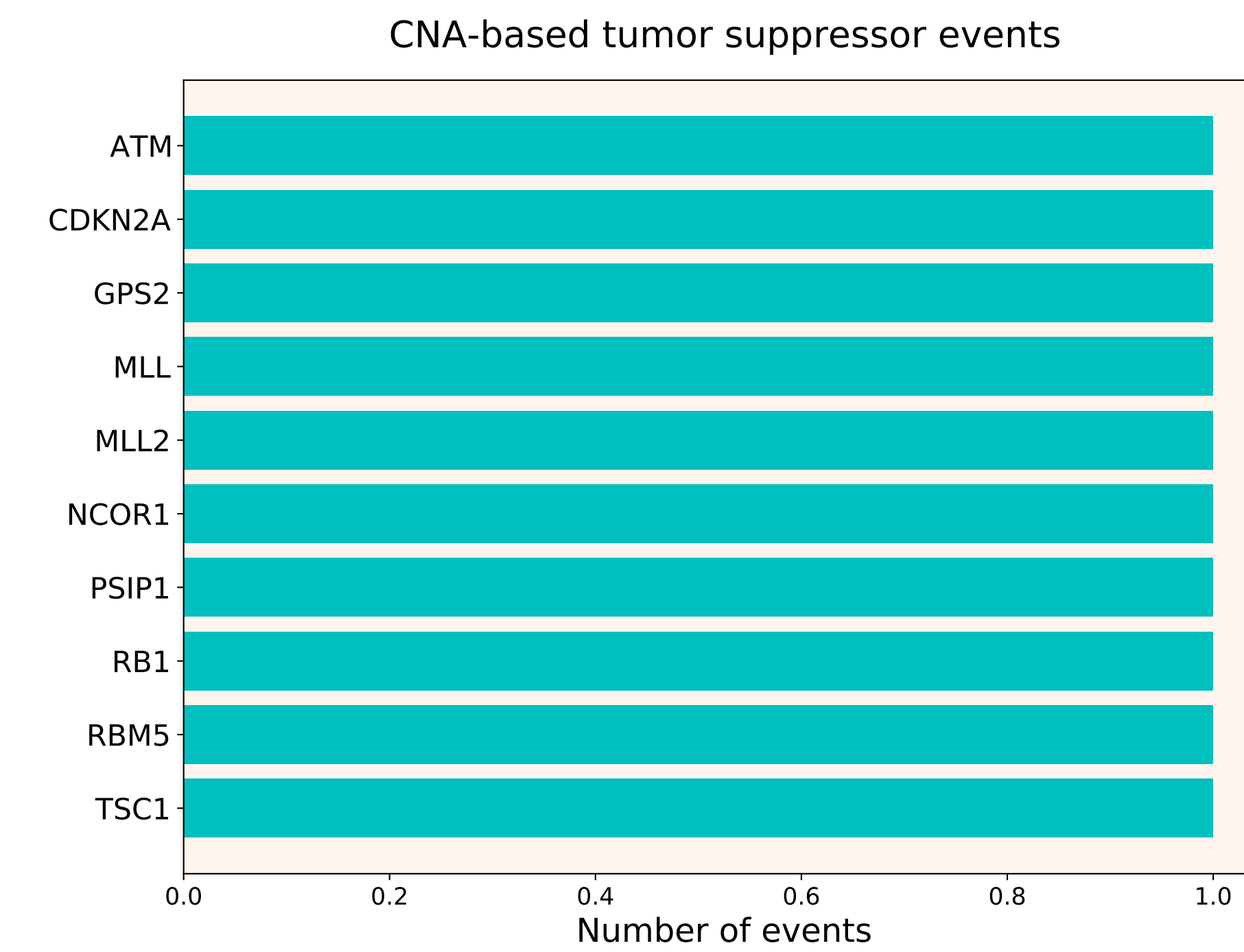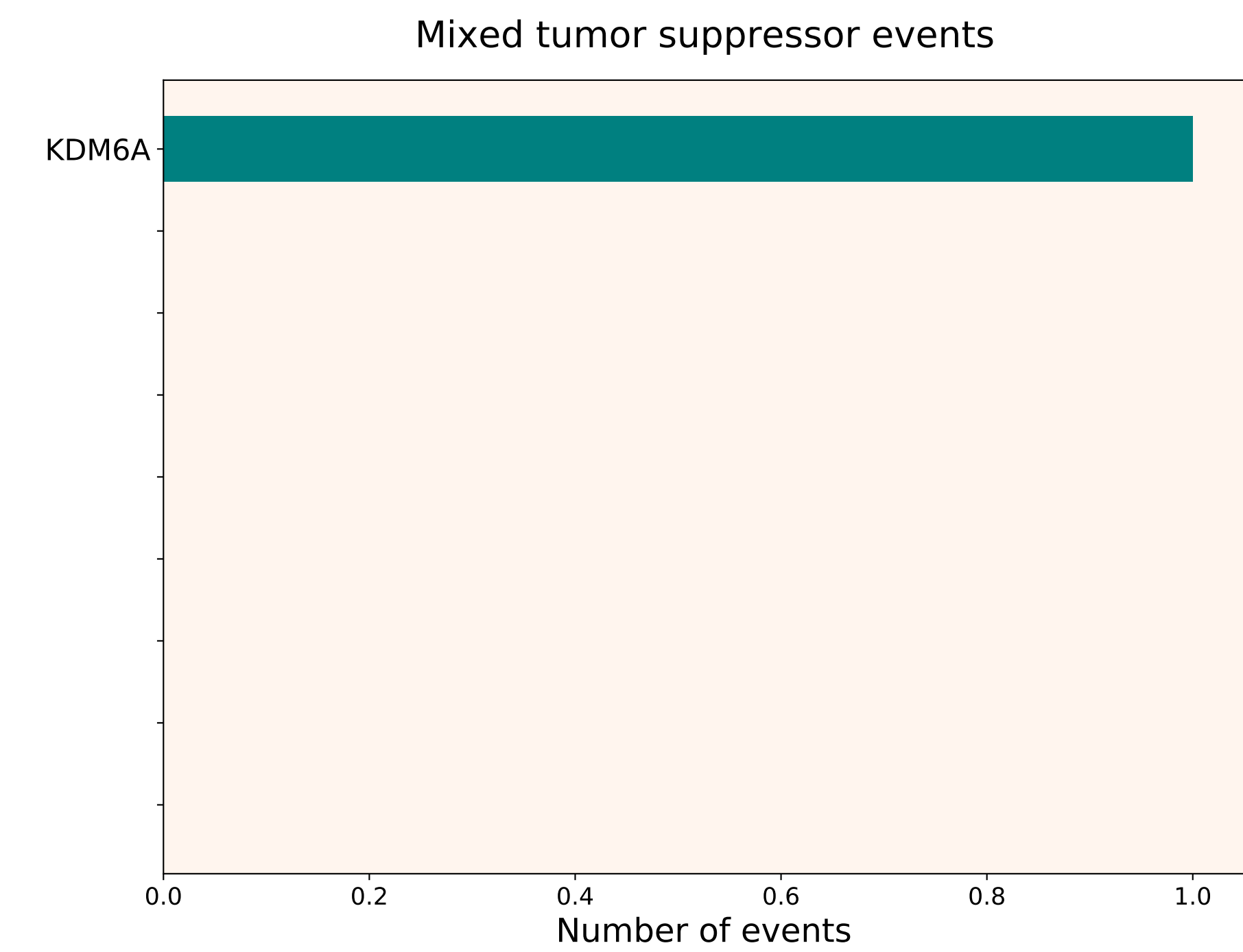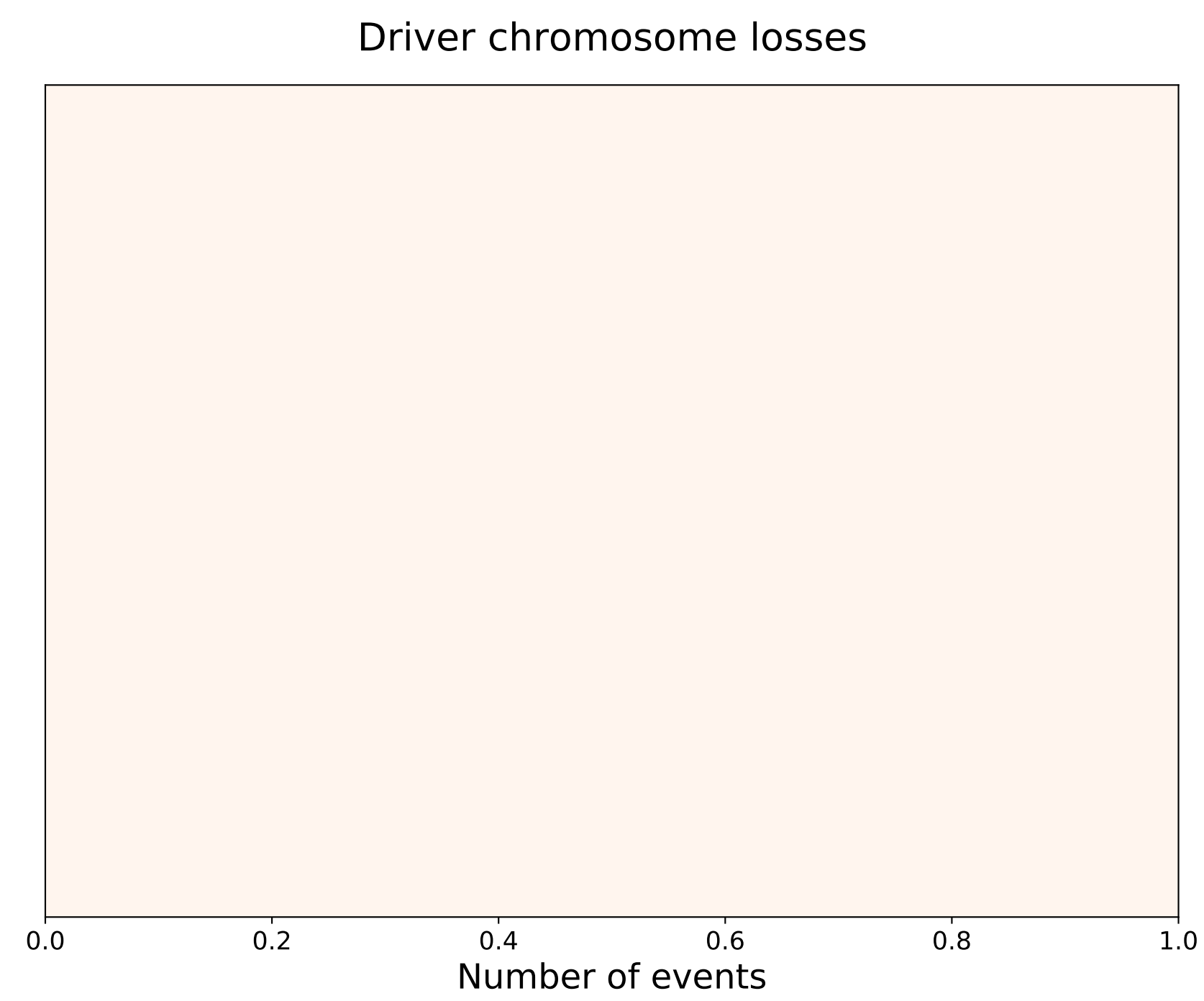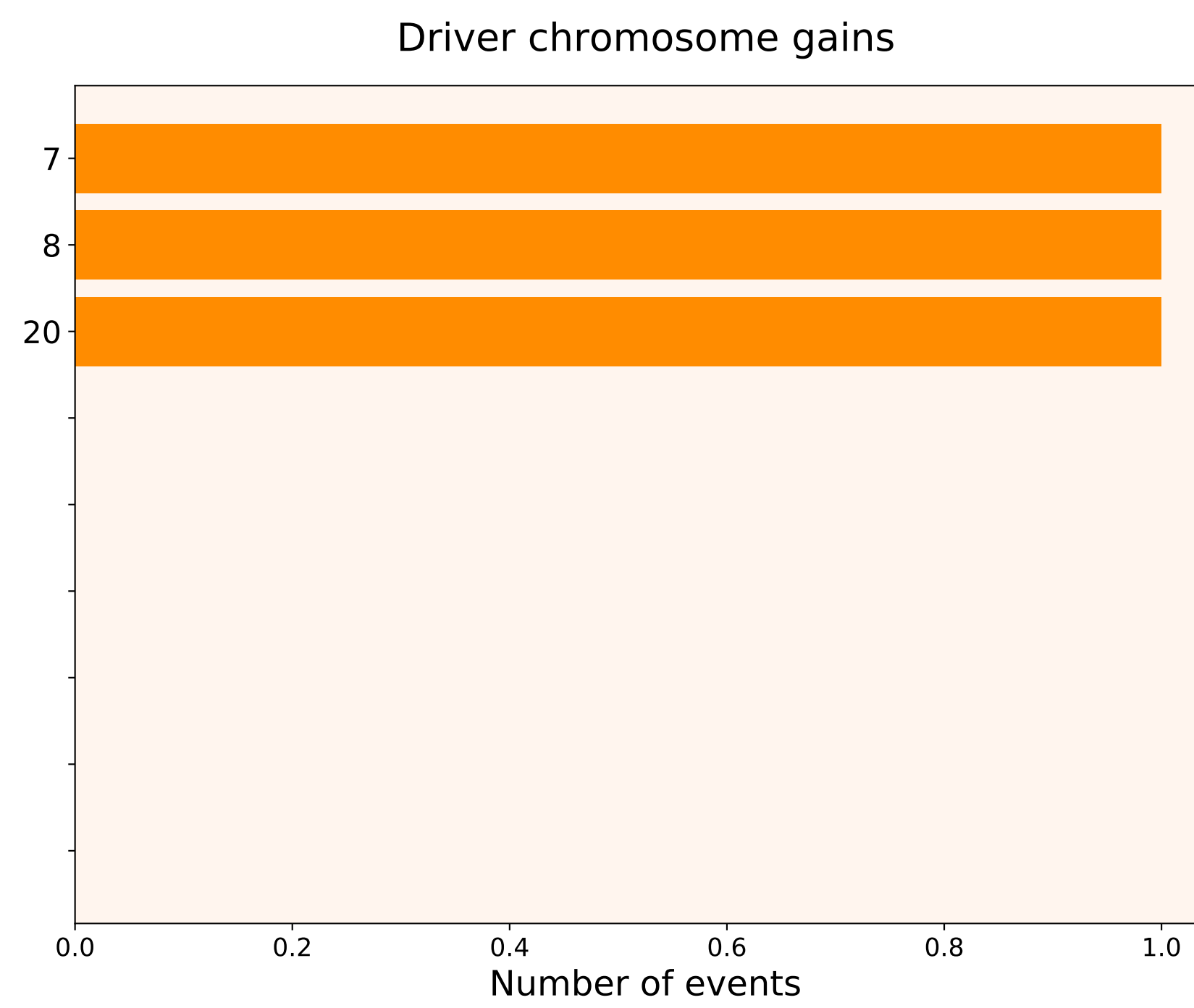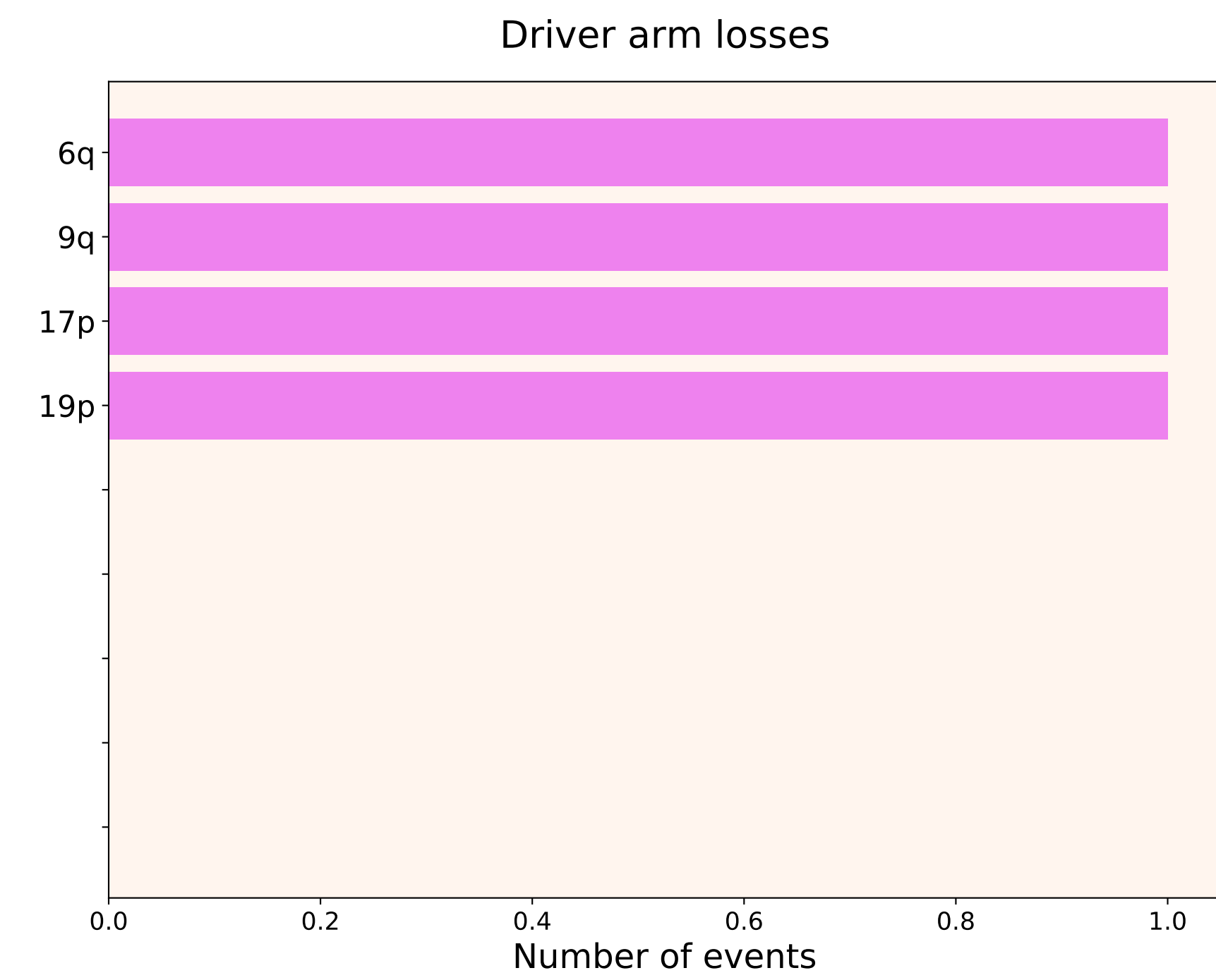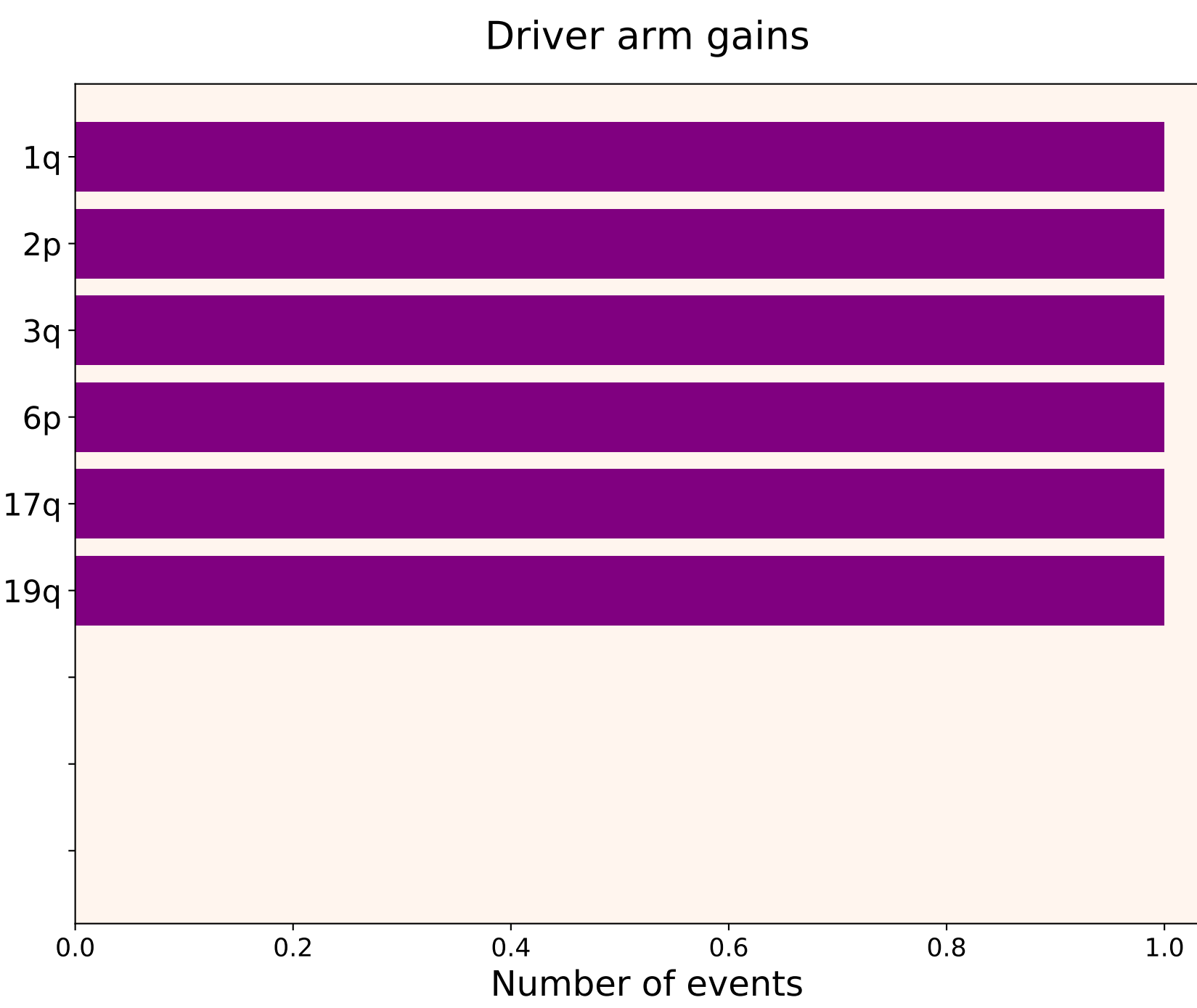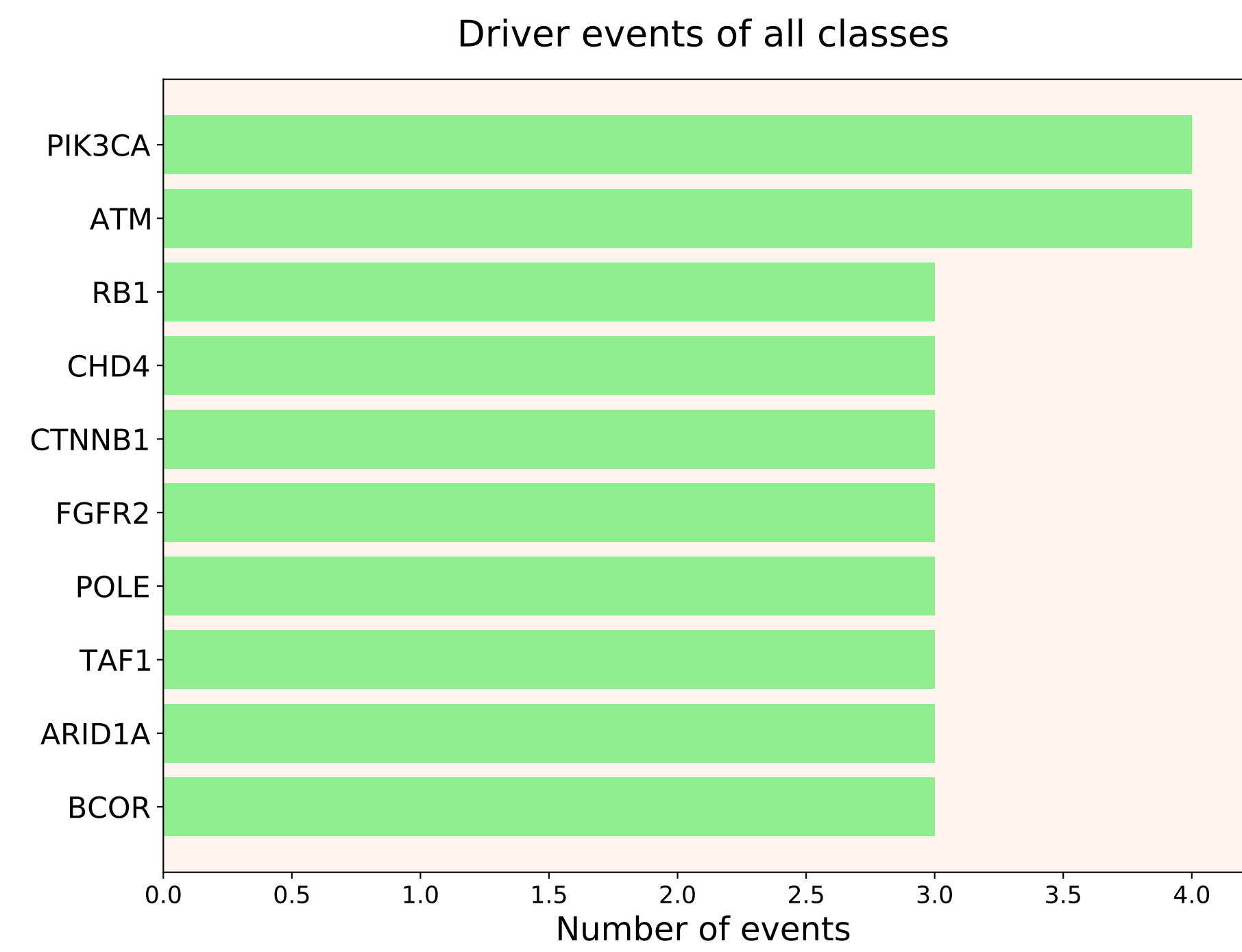

Supplement: Supplemental Information 2 [file peerj-10-13860-s002.zip › COHORTS/genes plots/detailed/2021_8_16_14_9_distribution_events_detailed_females_35.pdf]

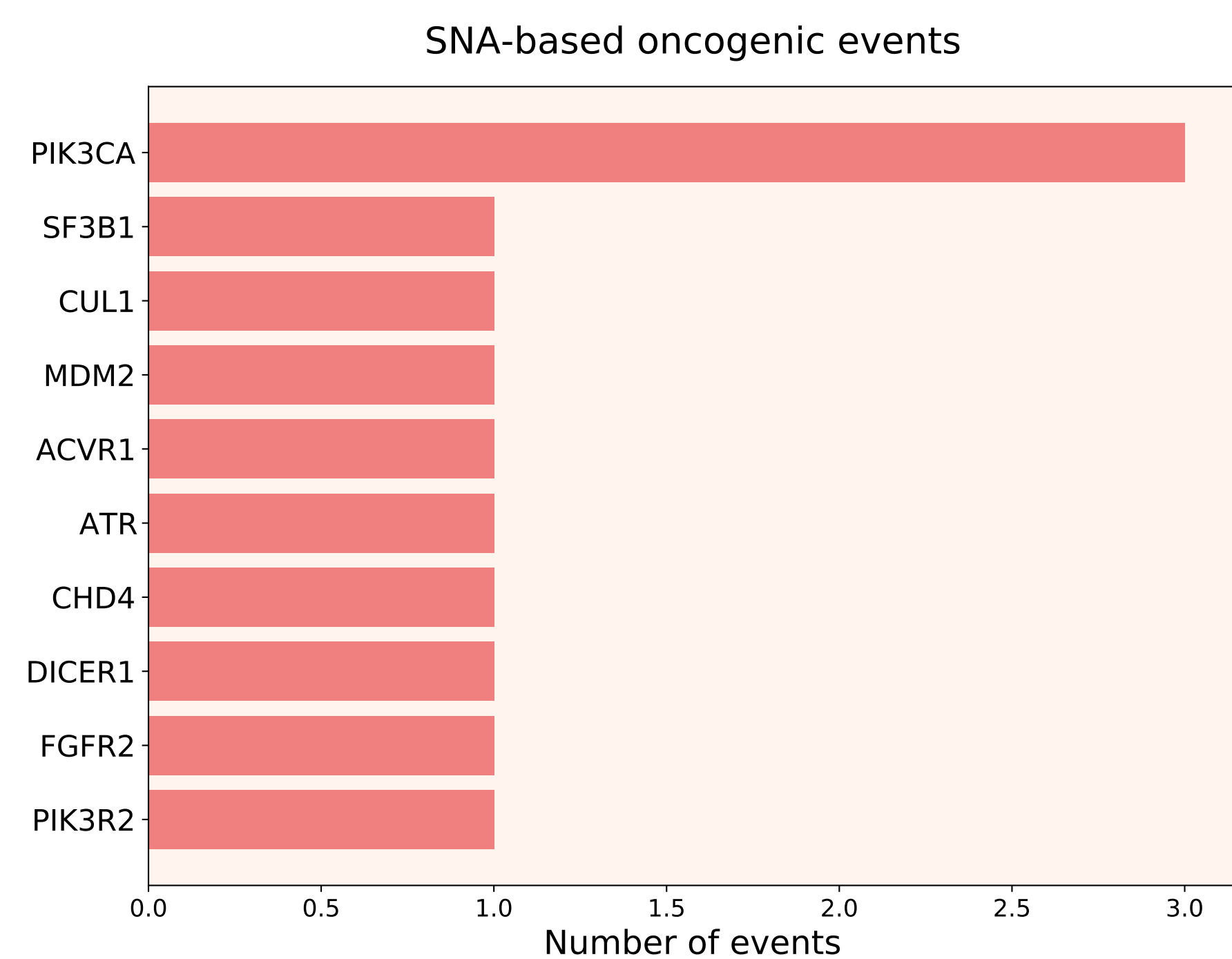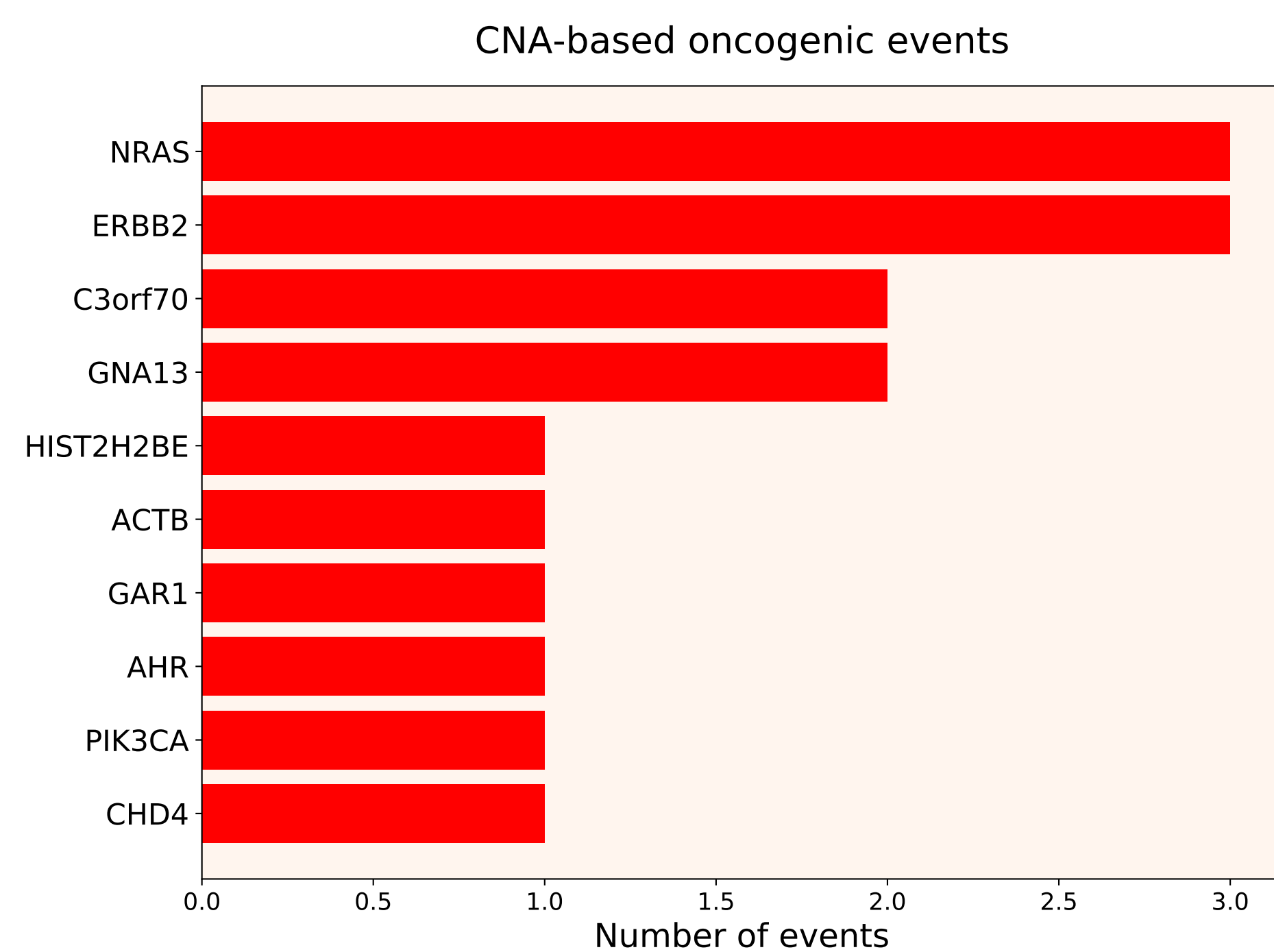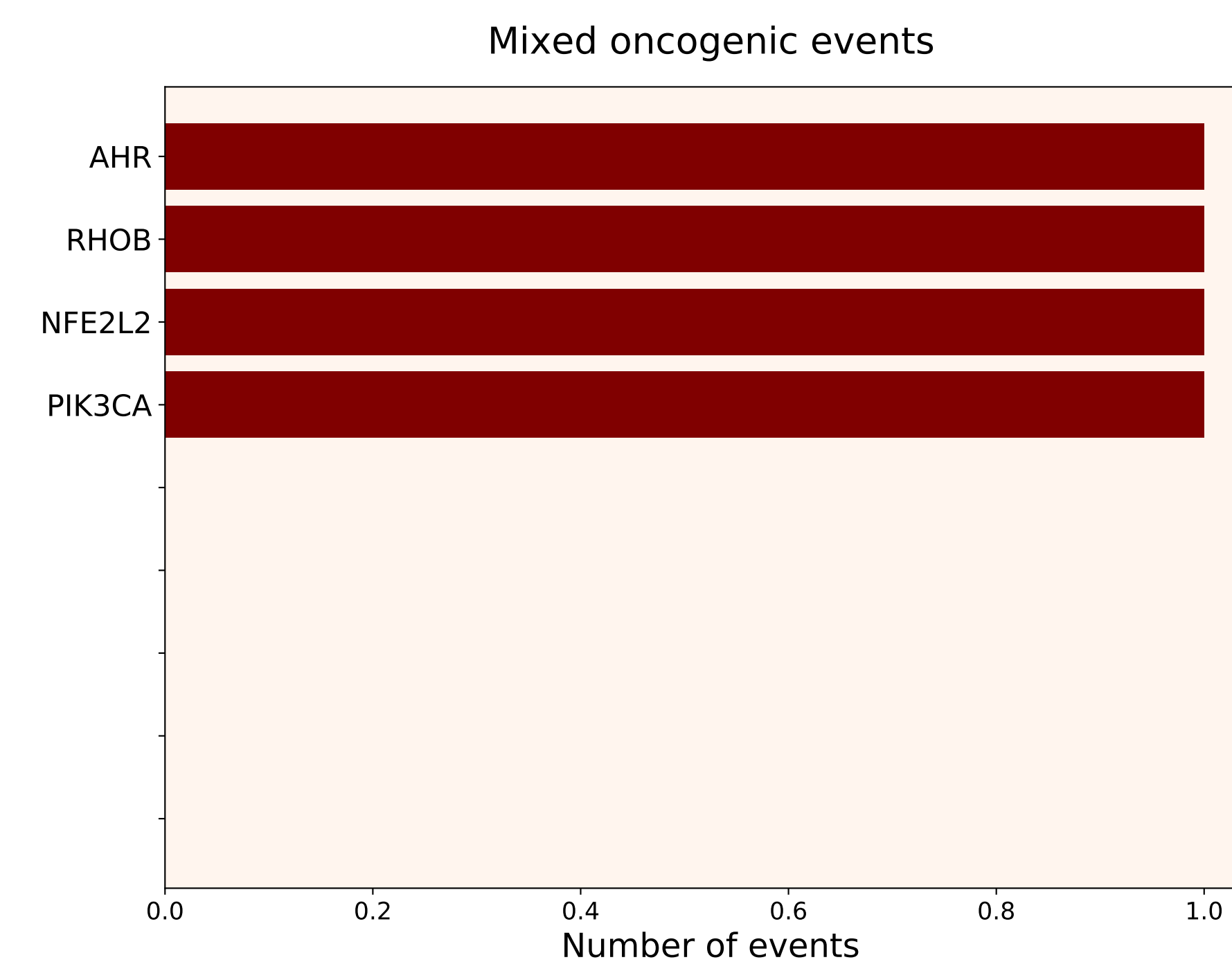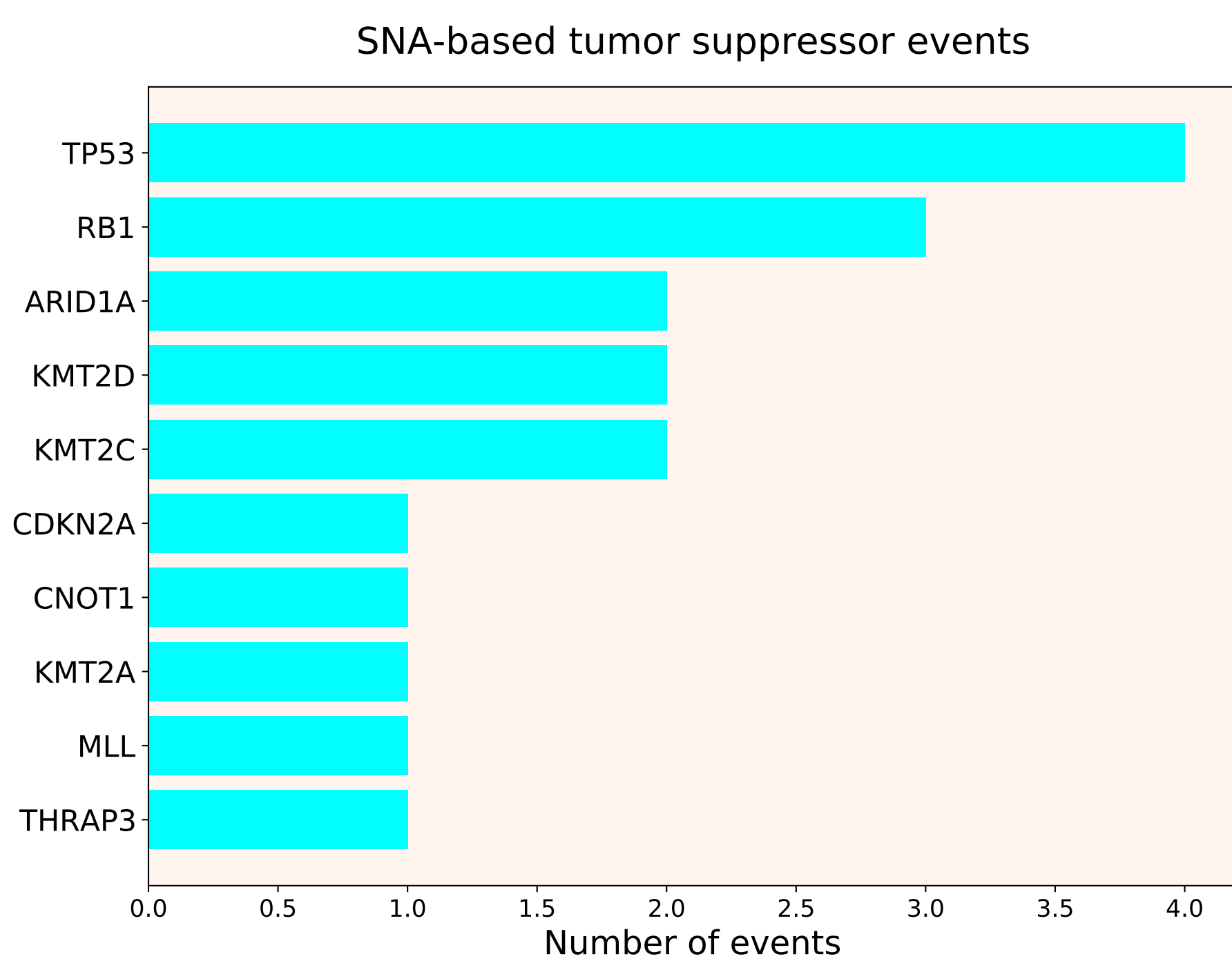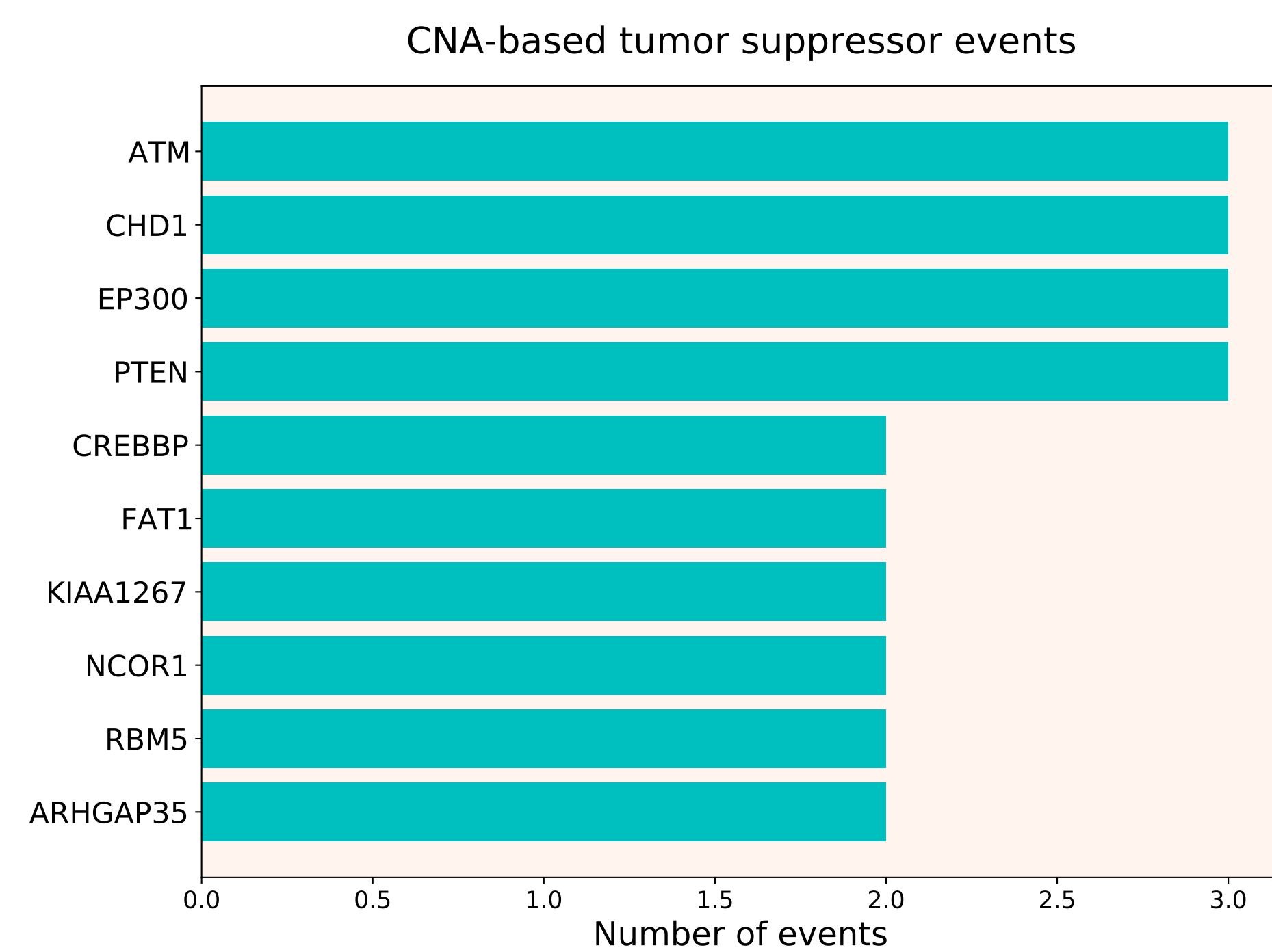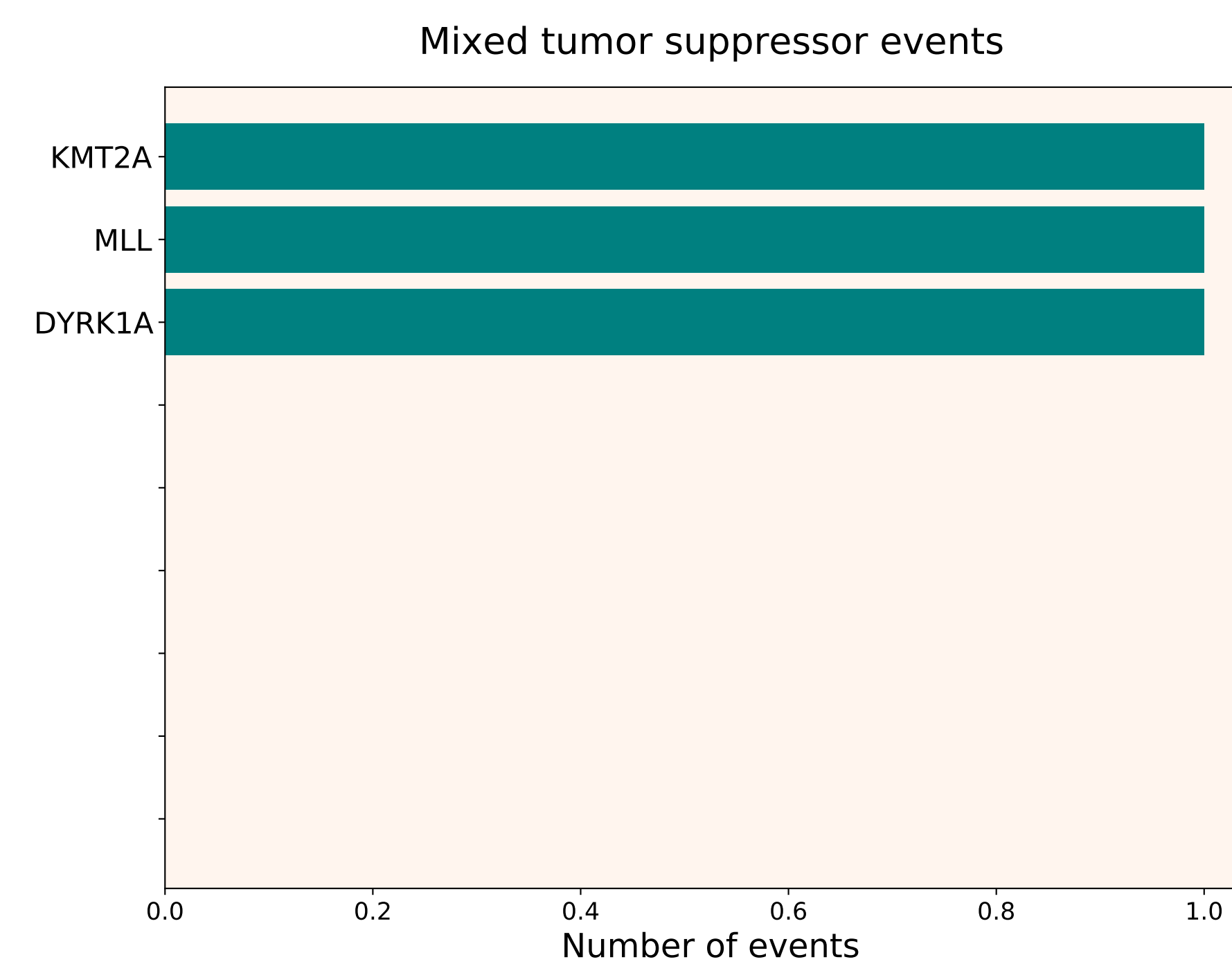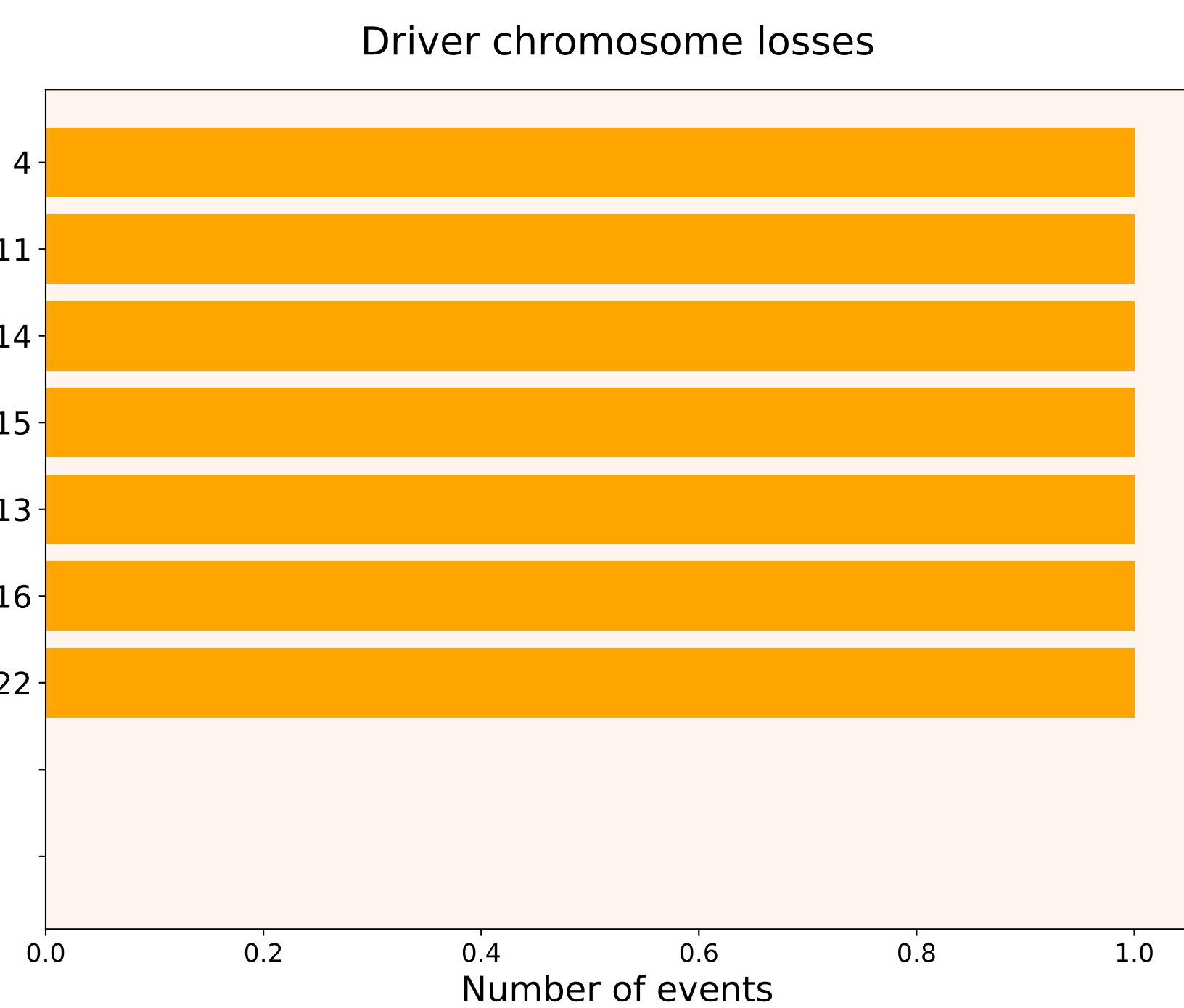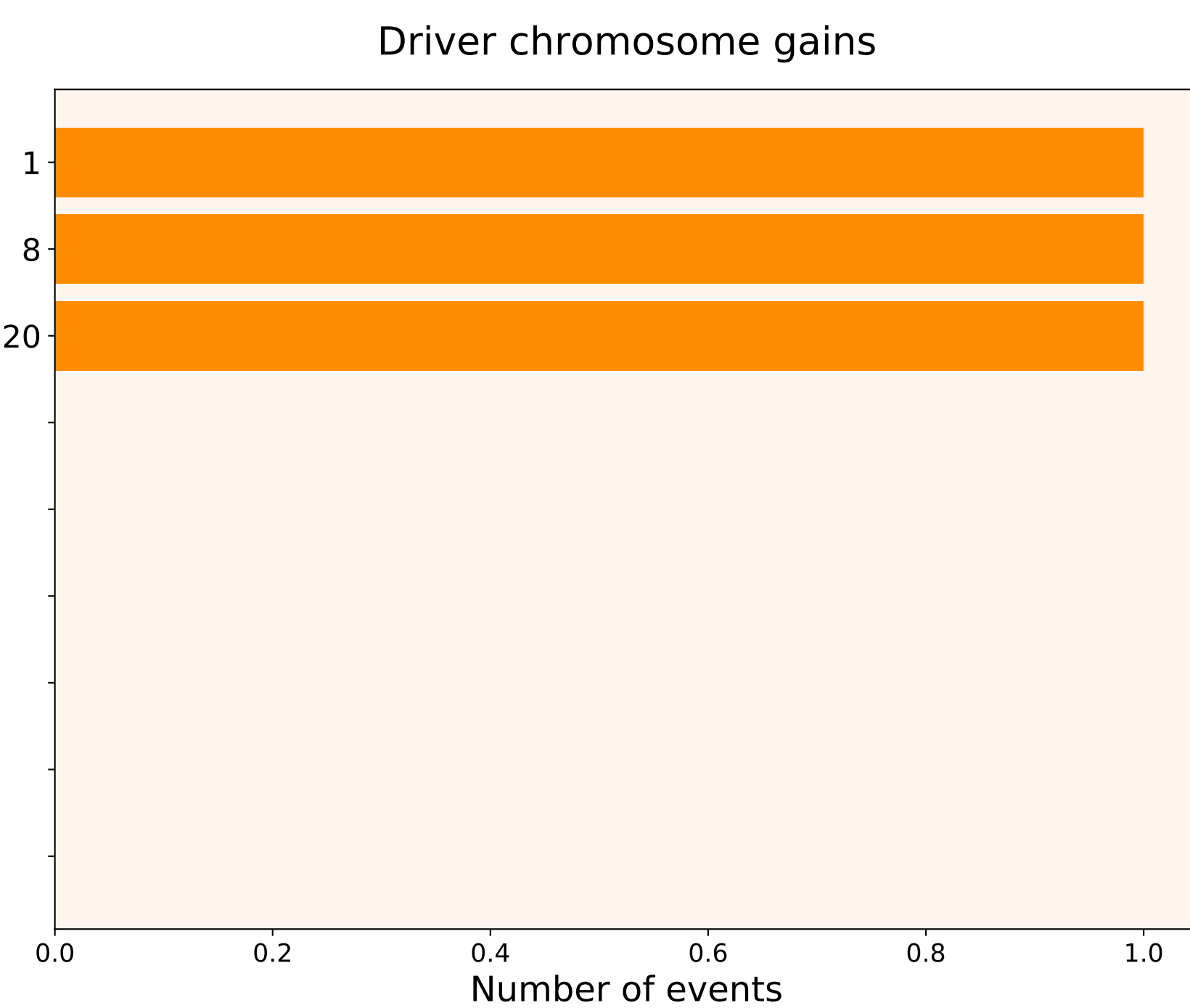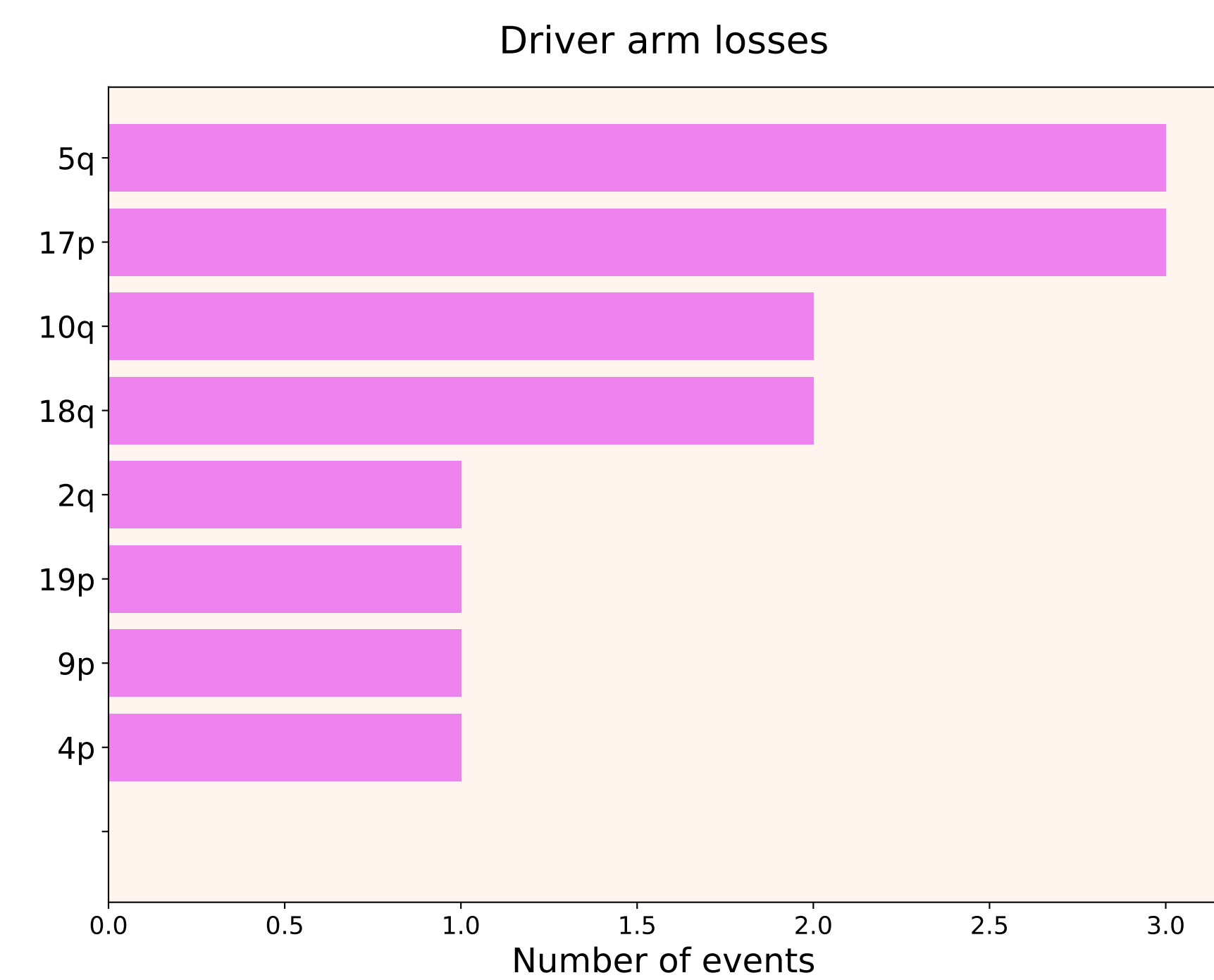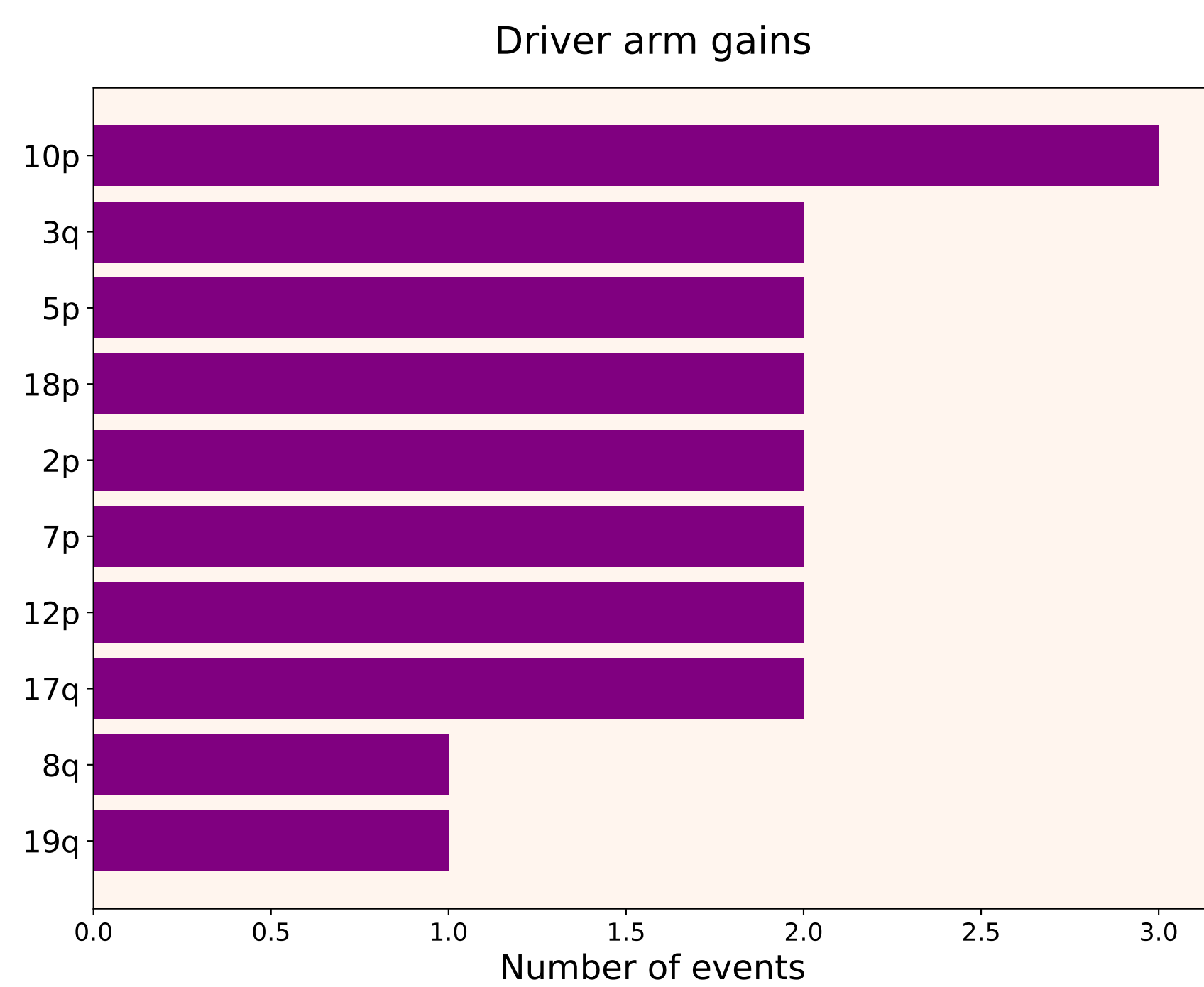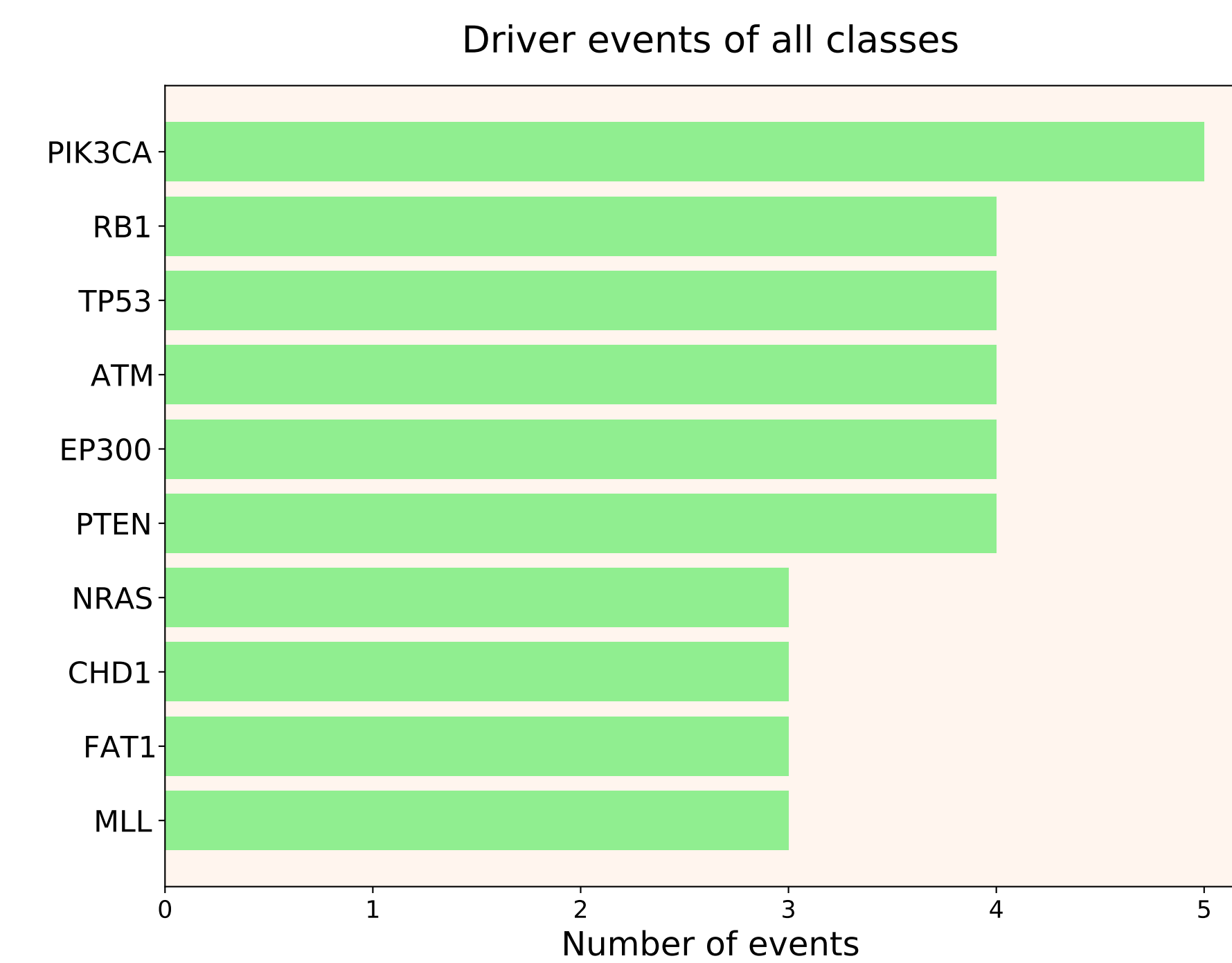

Supplement: Supplemental Information 2 [file peerj-10-13860-s002.zip › COHORTS/genes plots/detailed/2021_8_16_14_9_distribution_events_detailed_females_34.pdf]

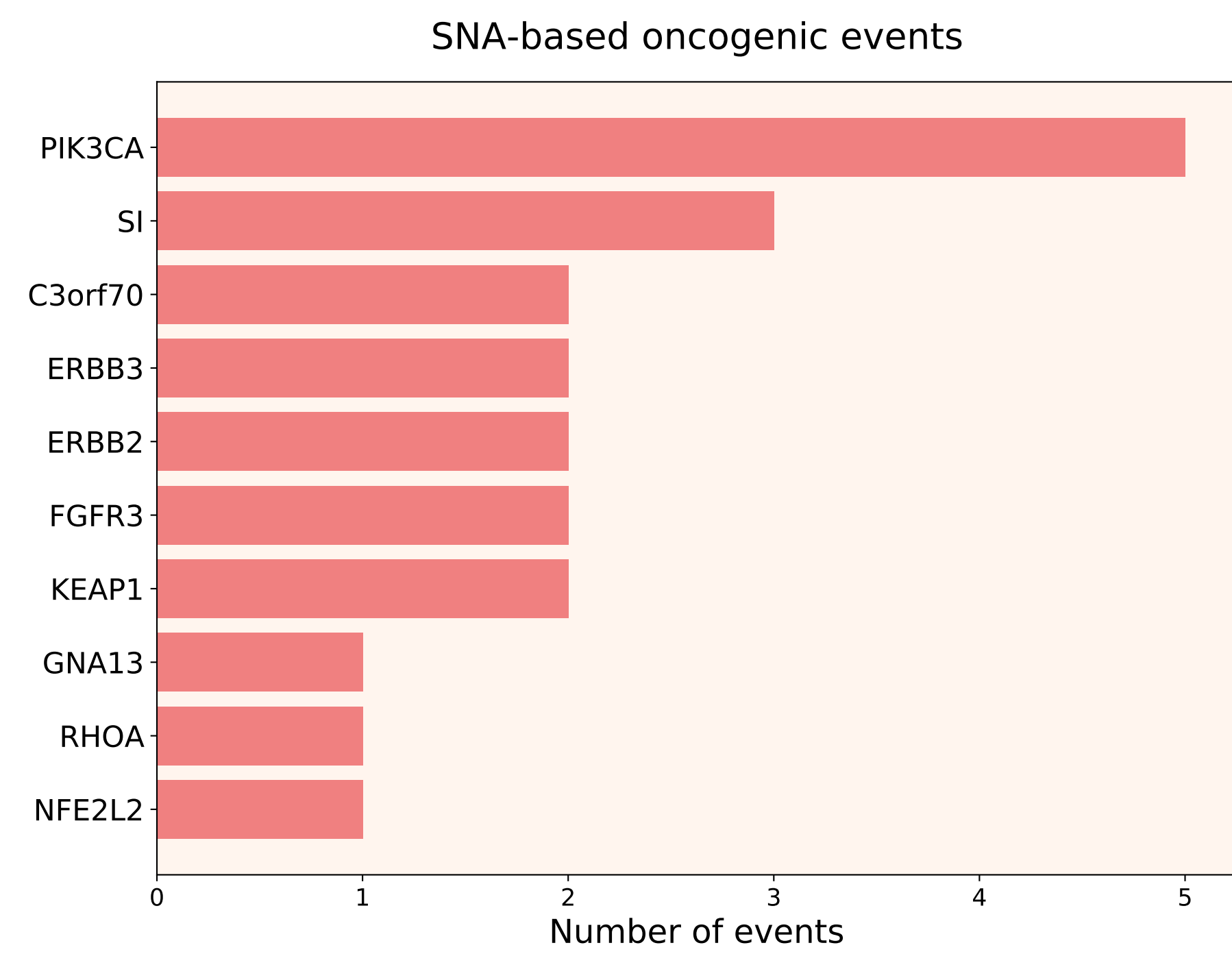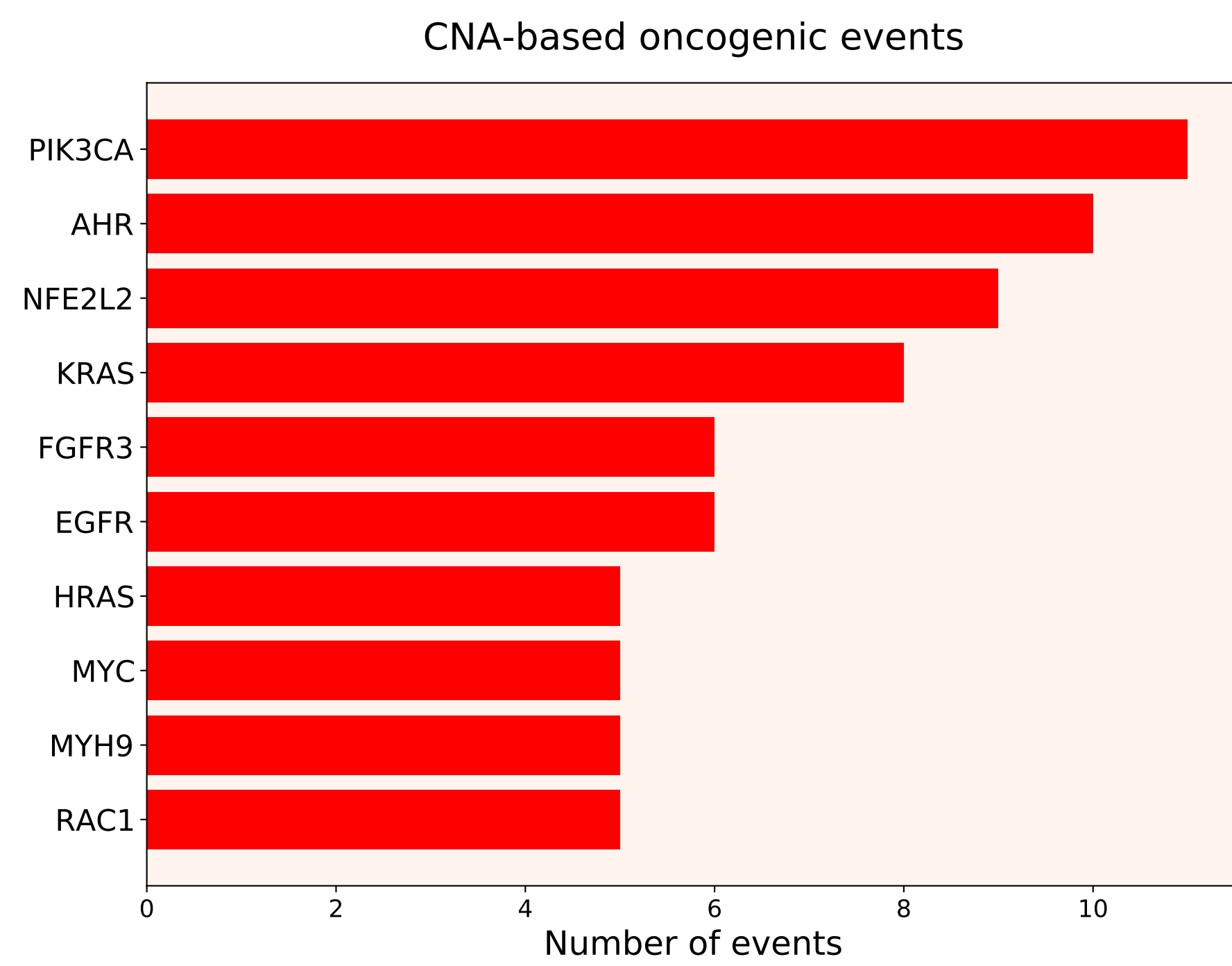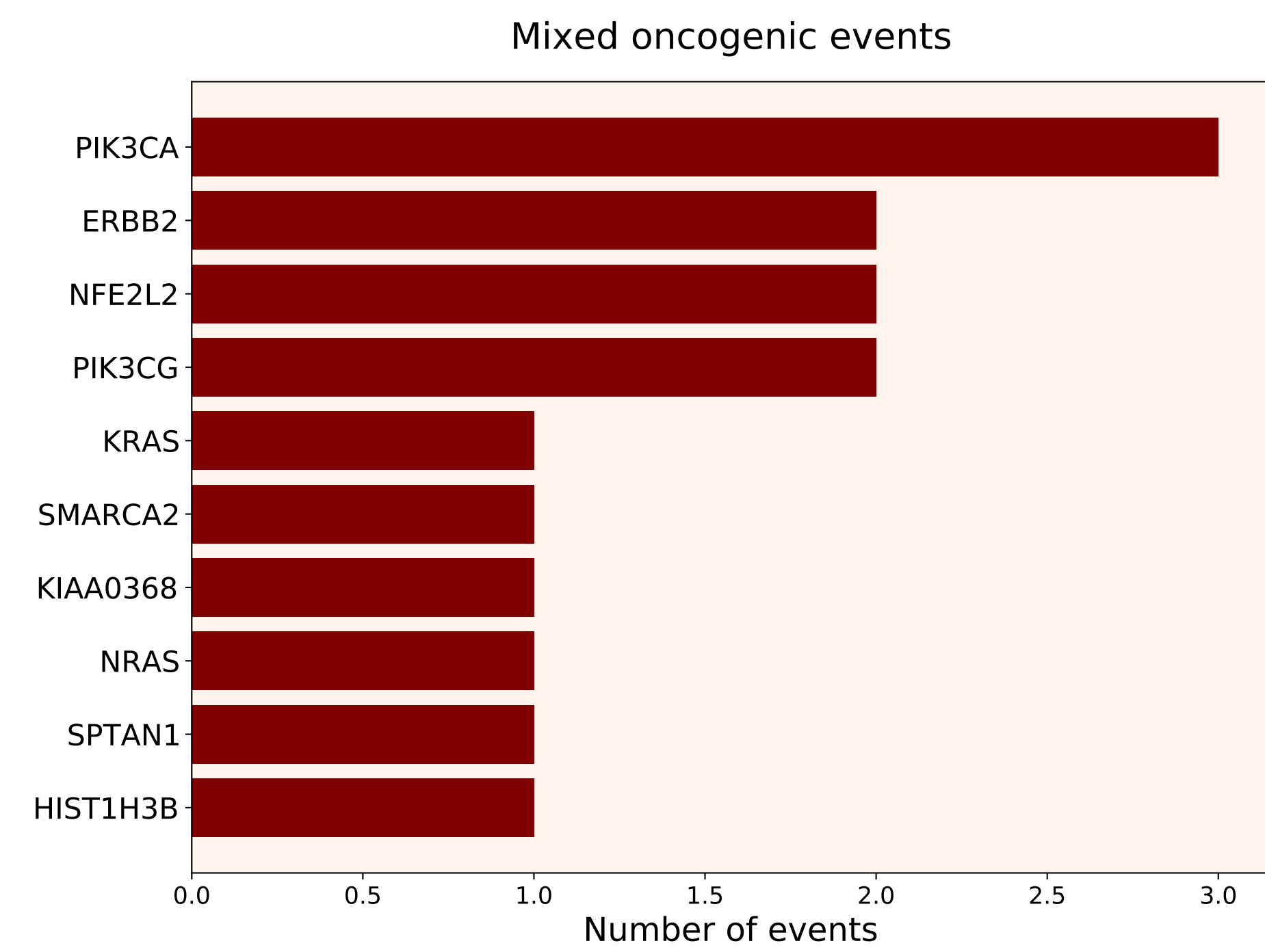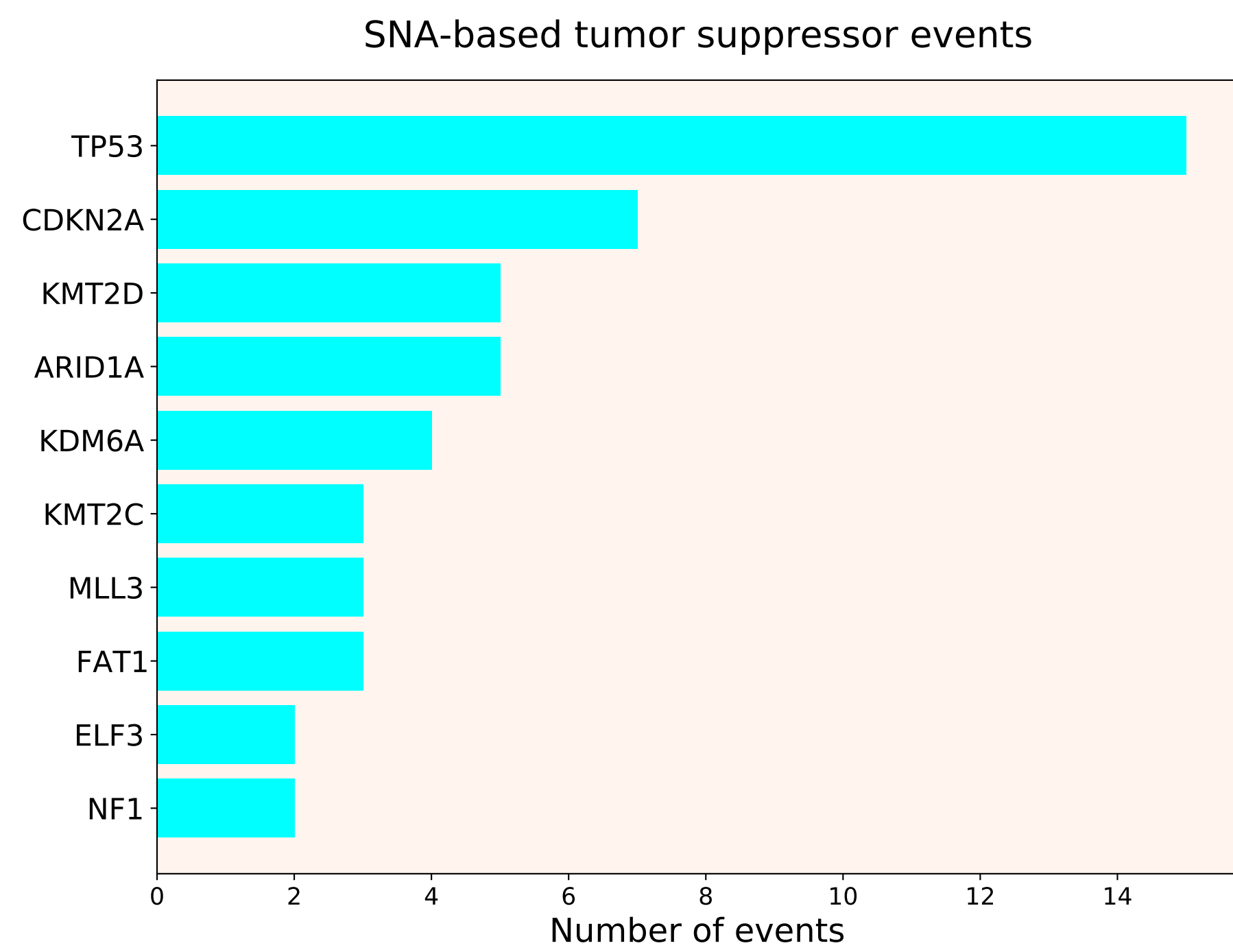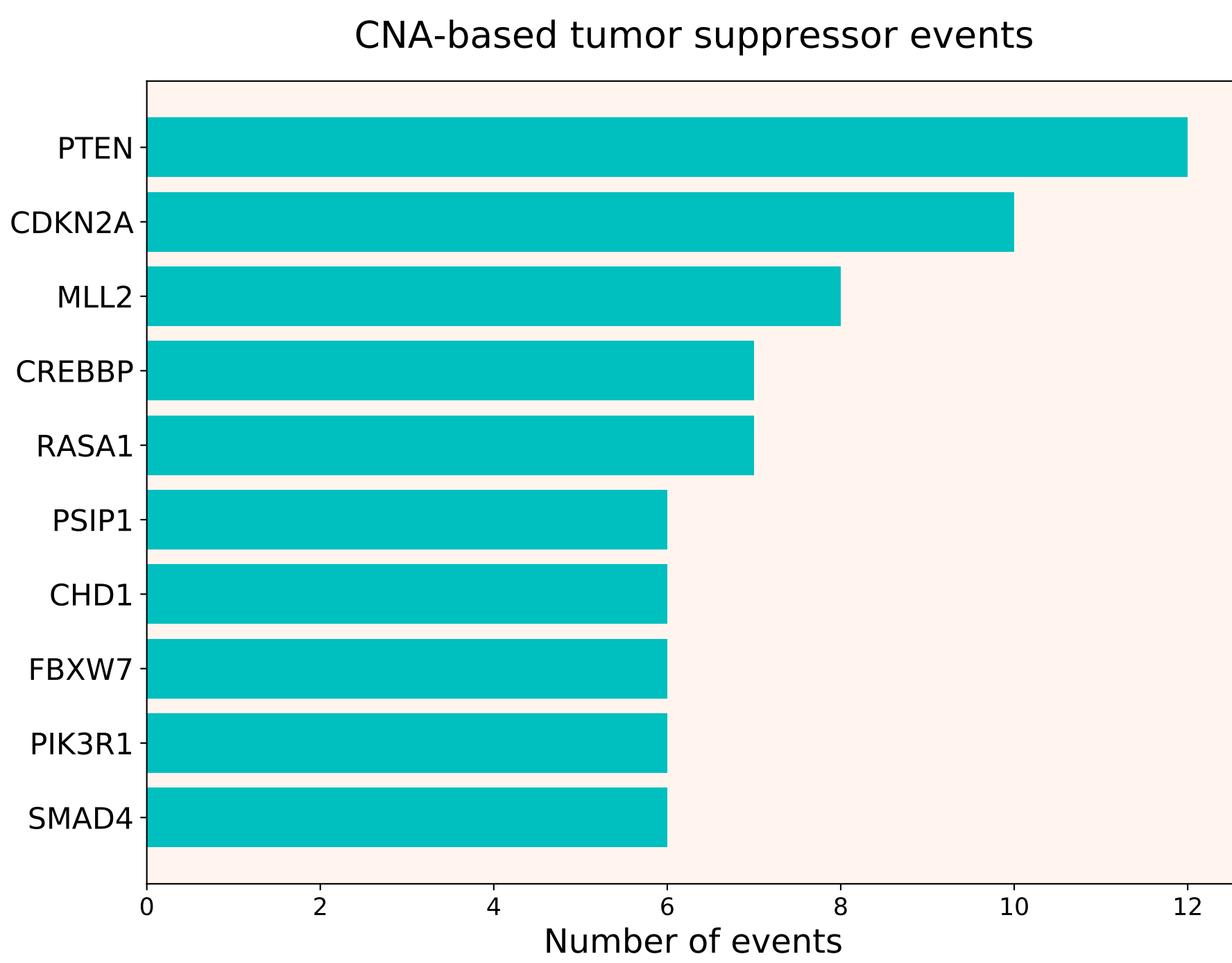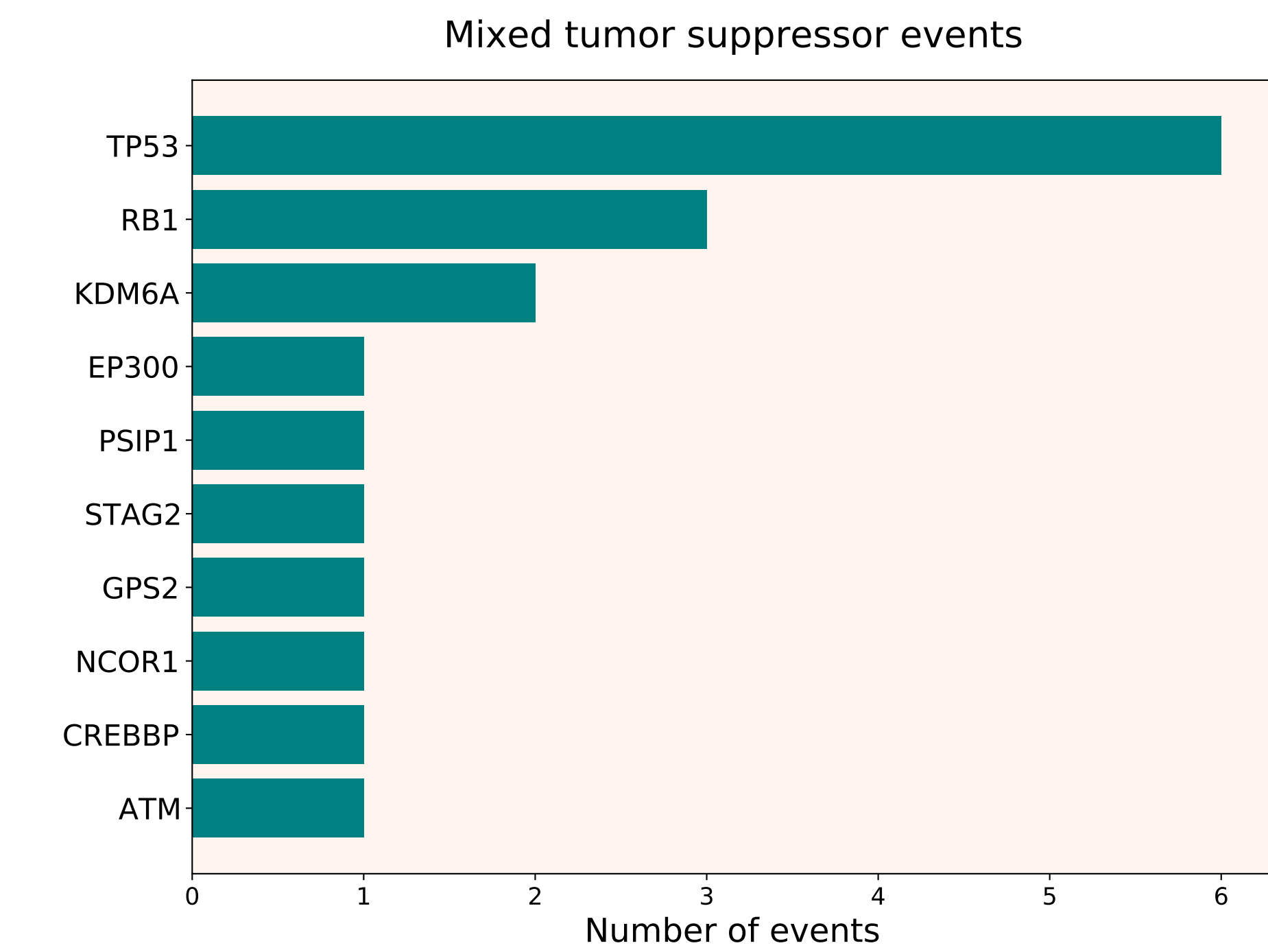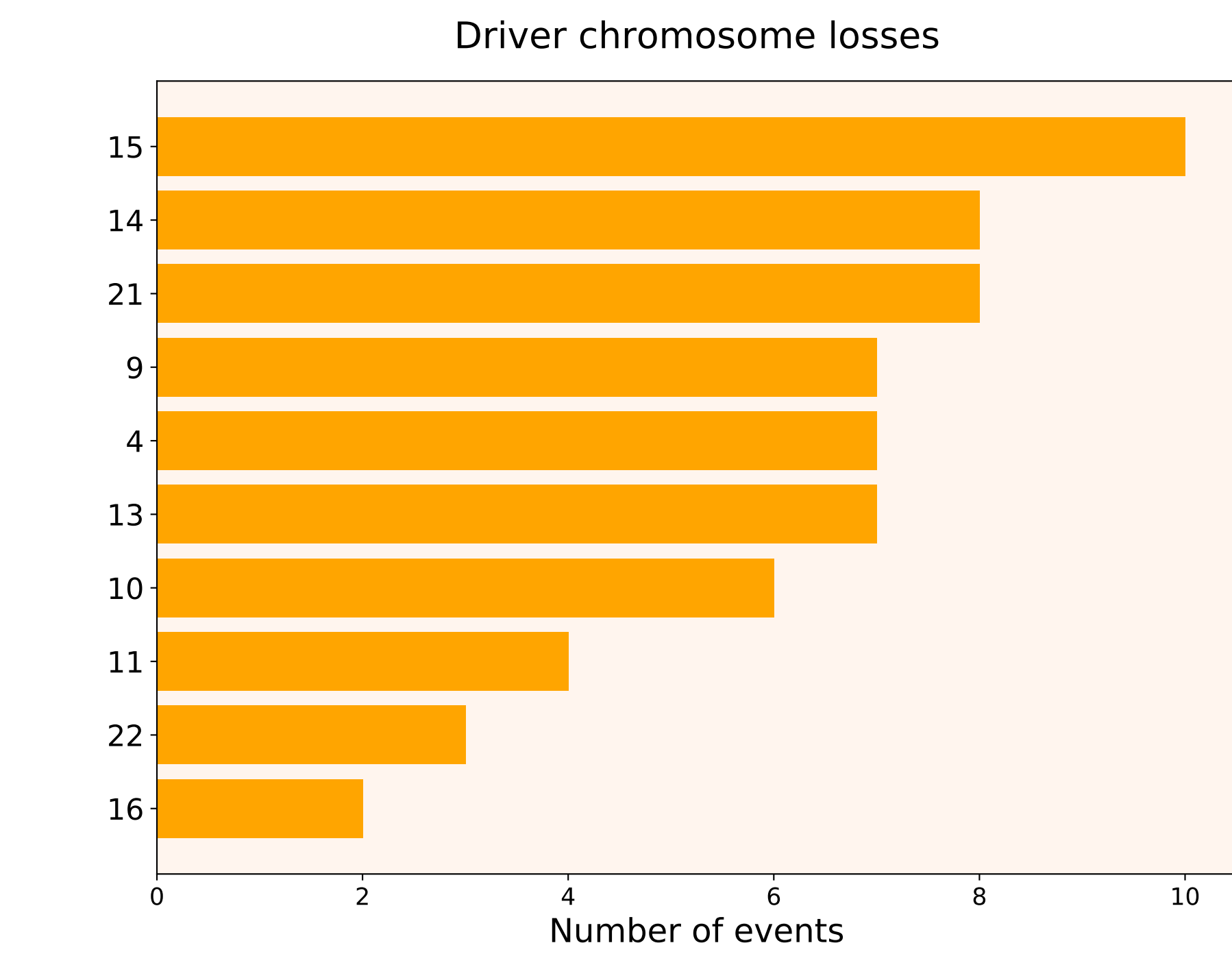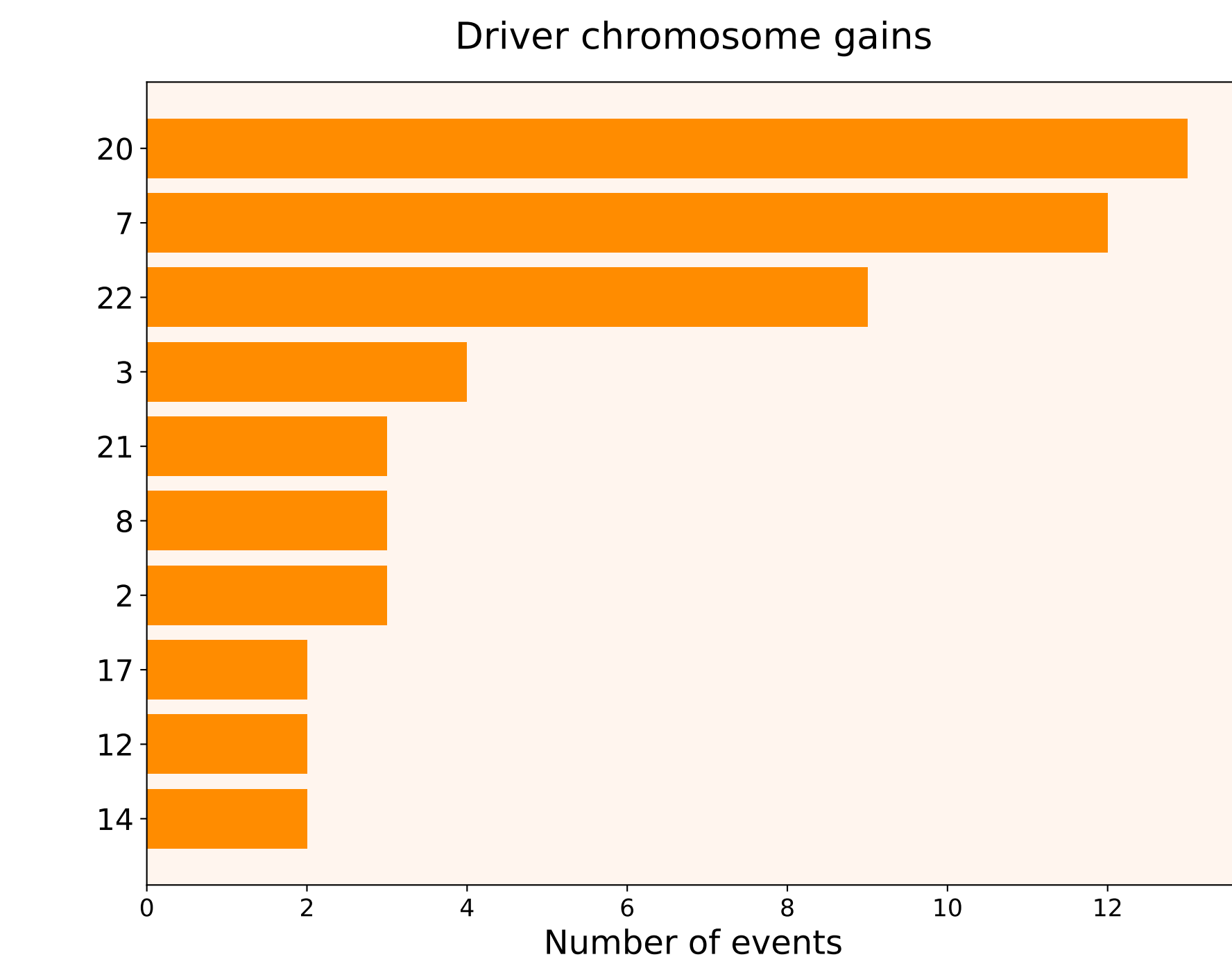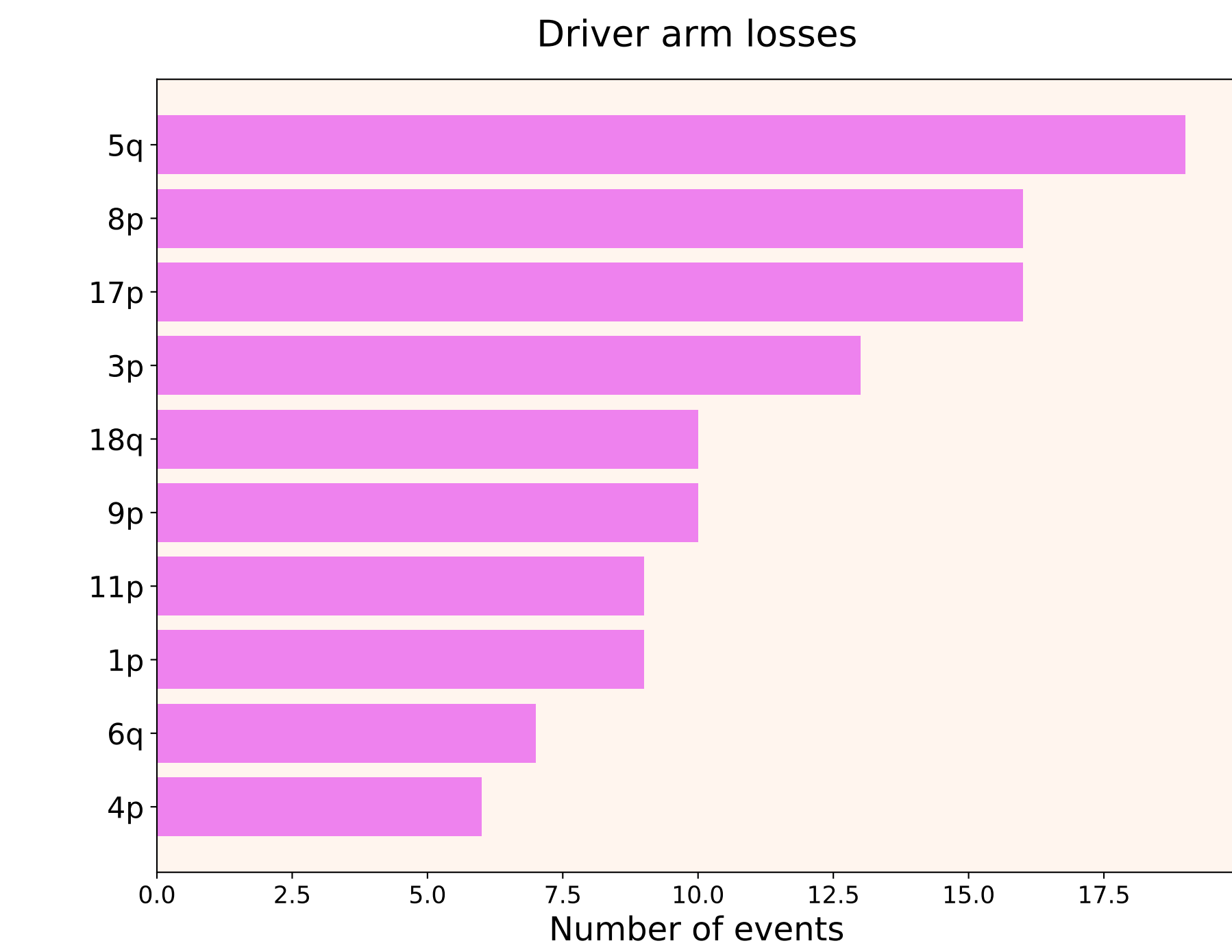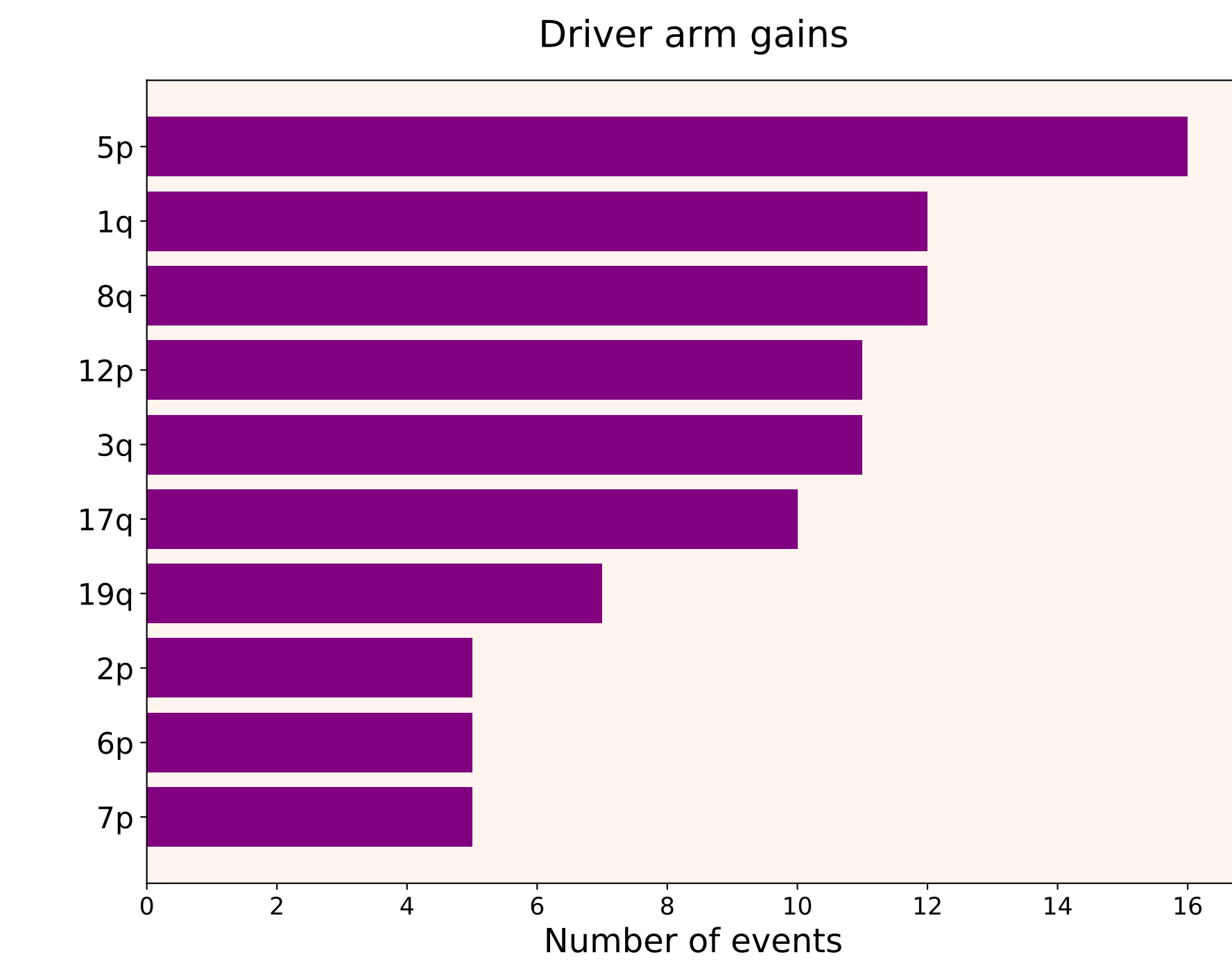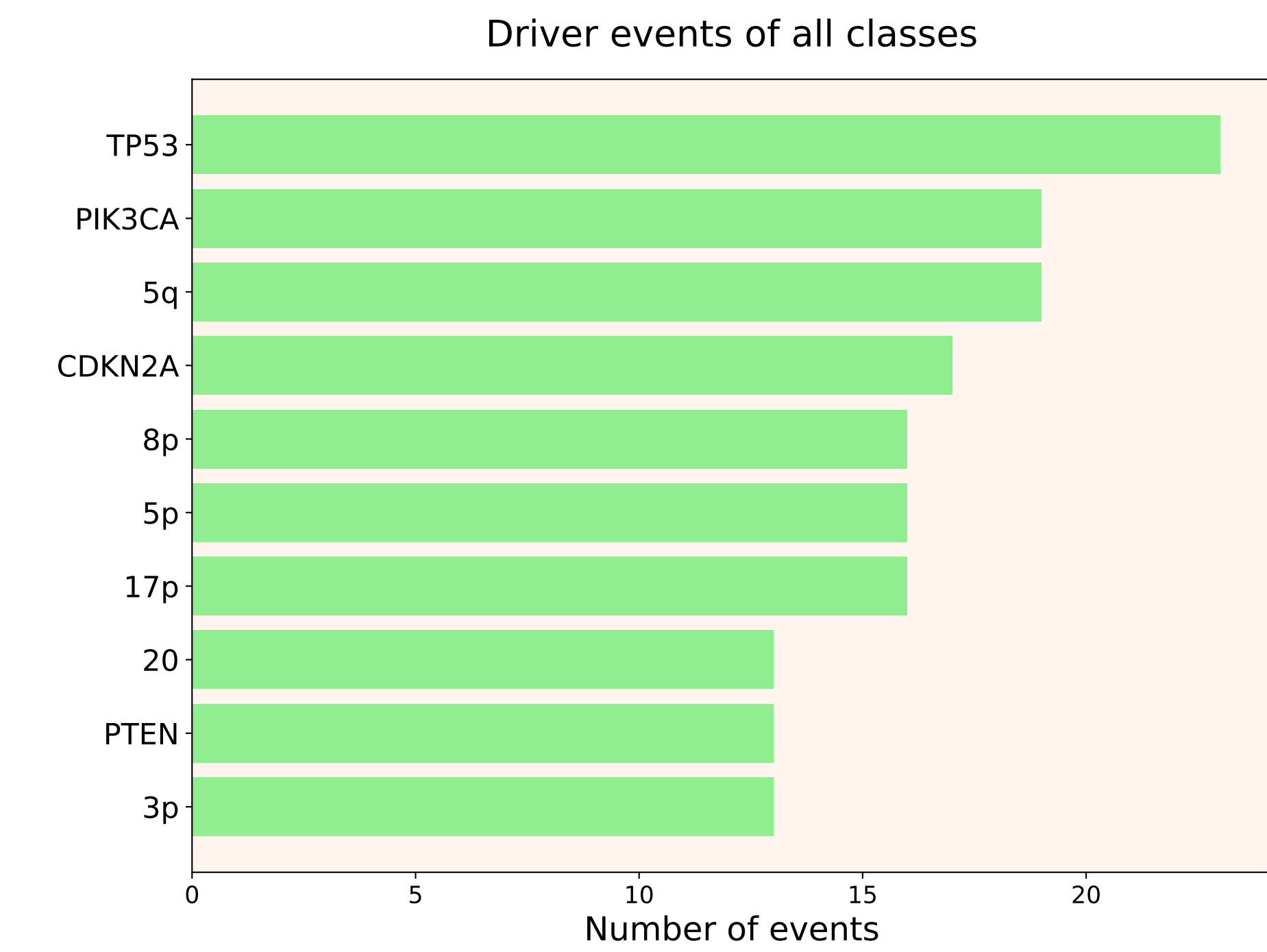

Supplement: Supplemental Information 2 [file peerj-10-13860-s002.zip › COHORTS/genes plots/detailed/2021_8_16_14_9_distribution_events_detailed_males_30.pdf]

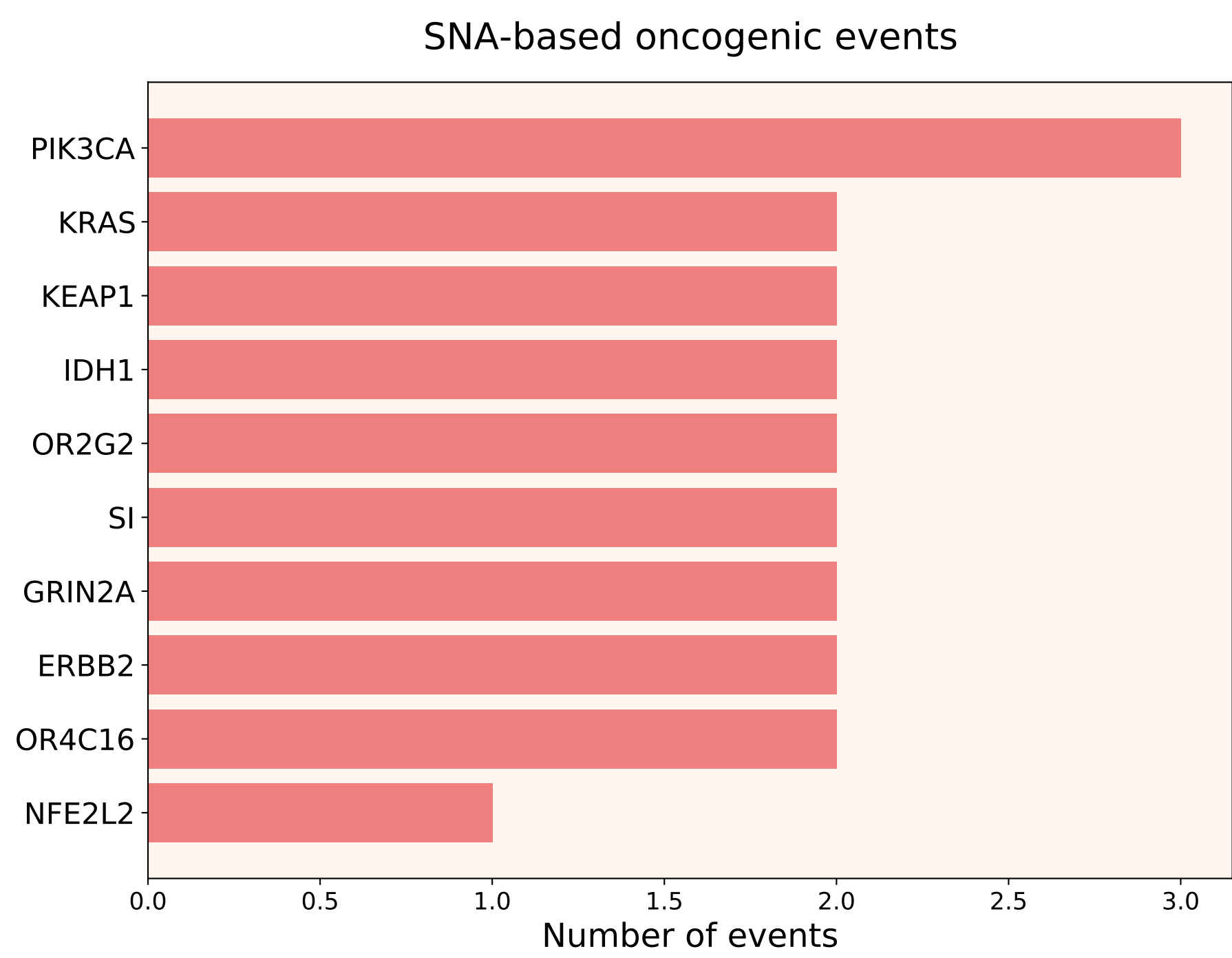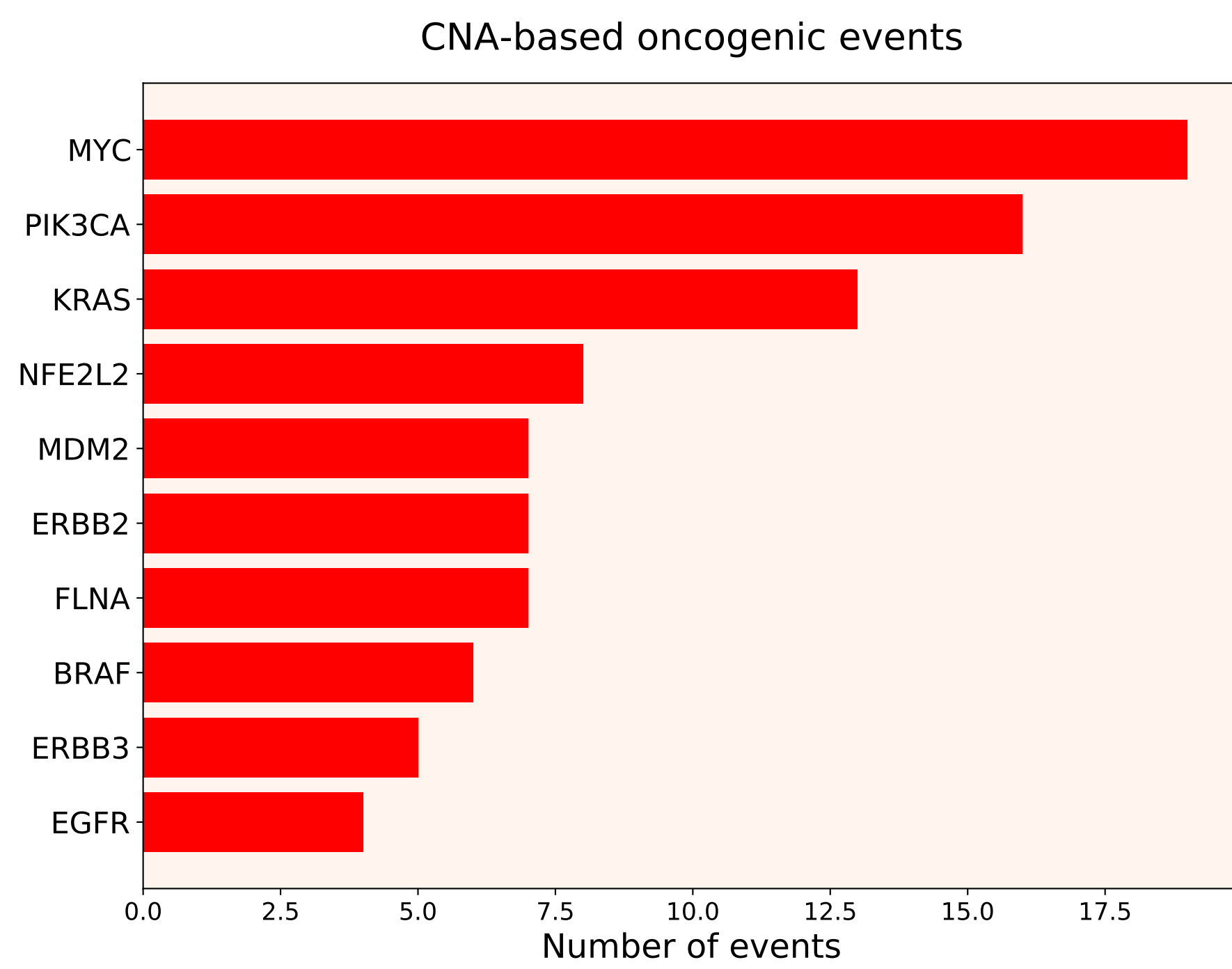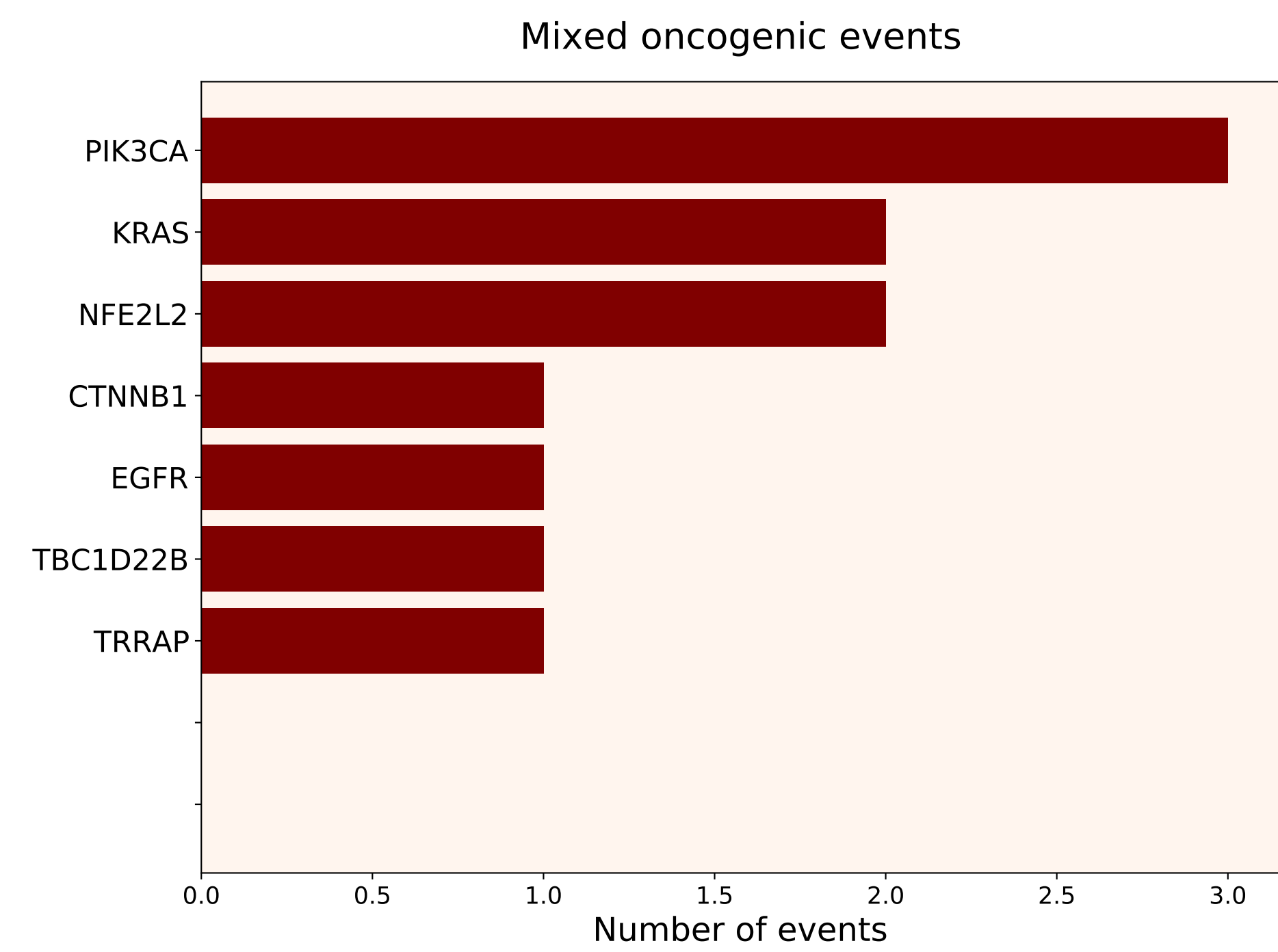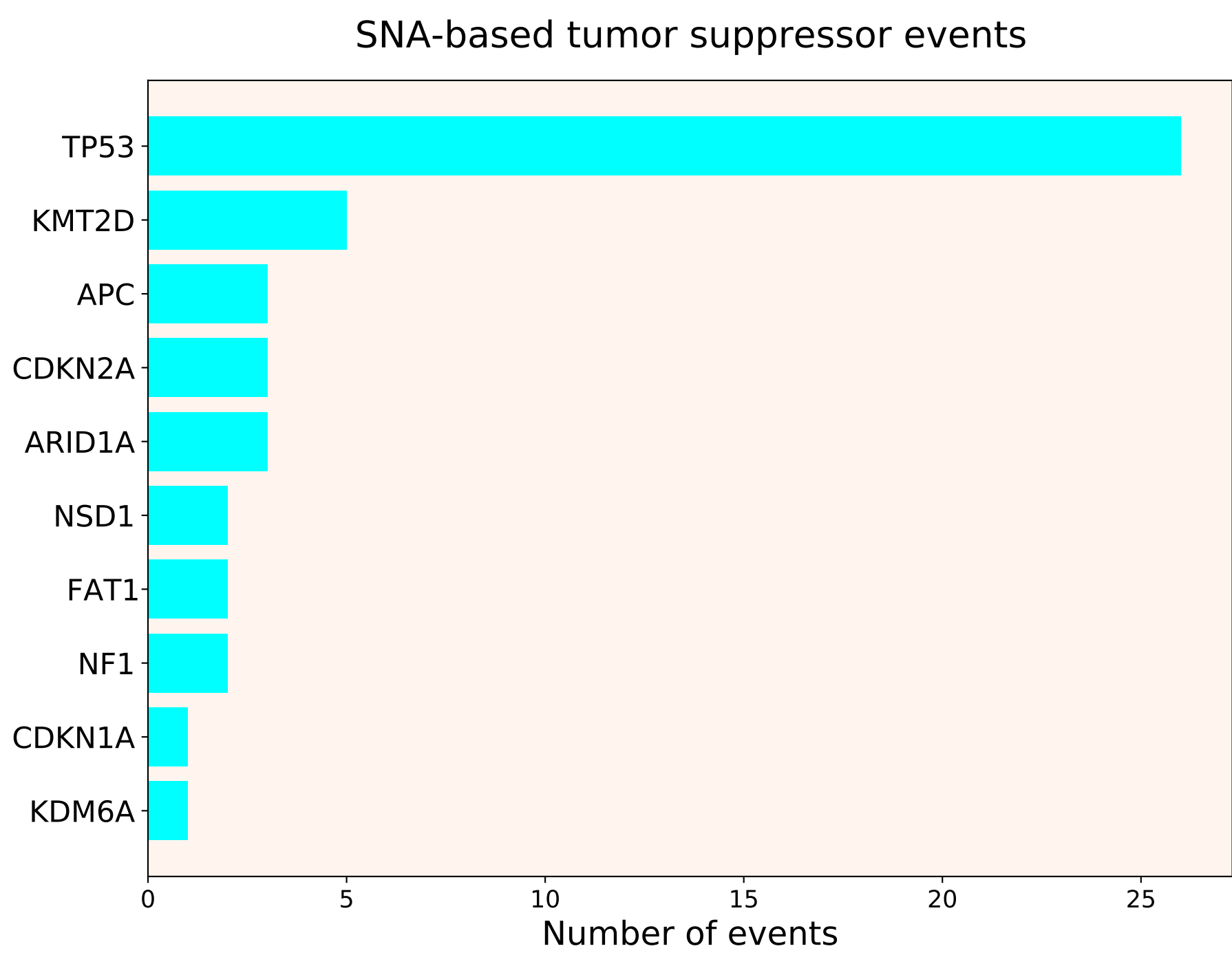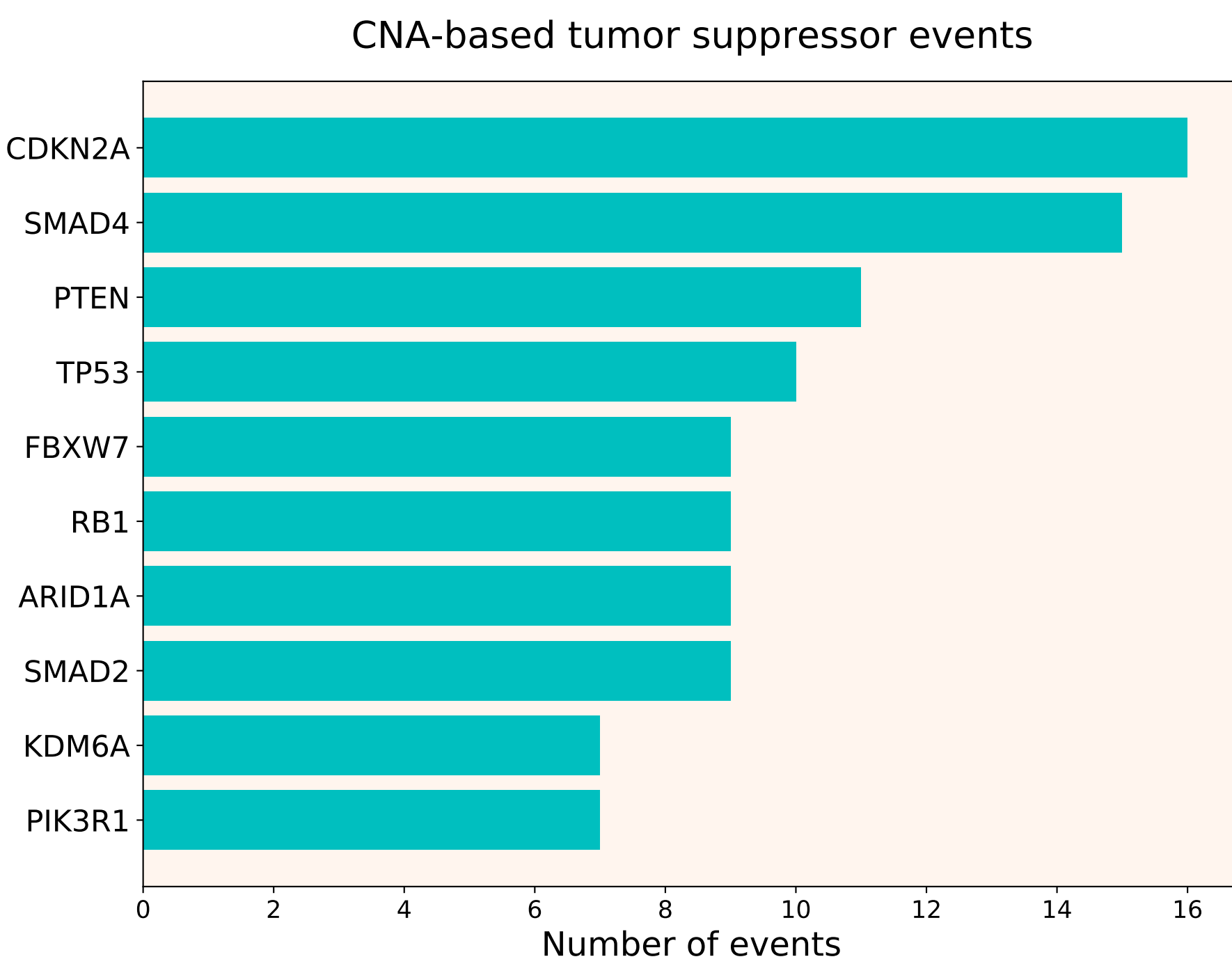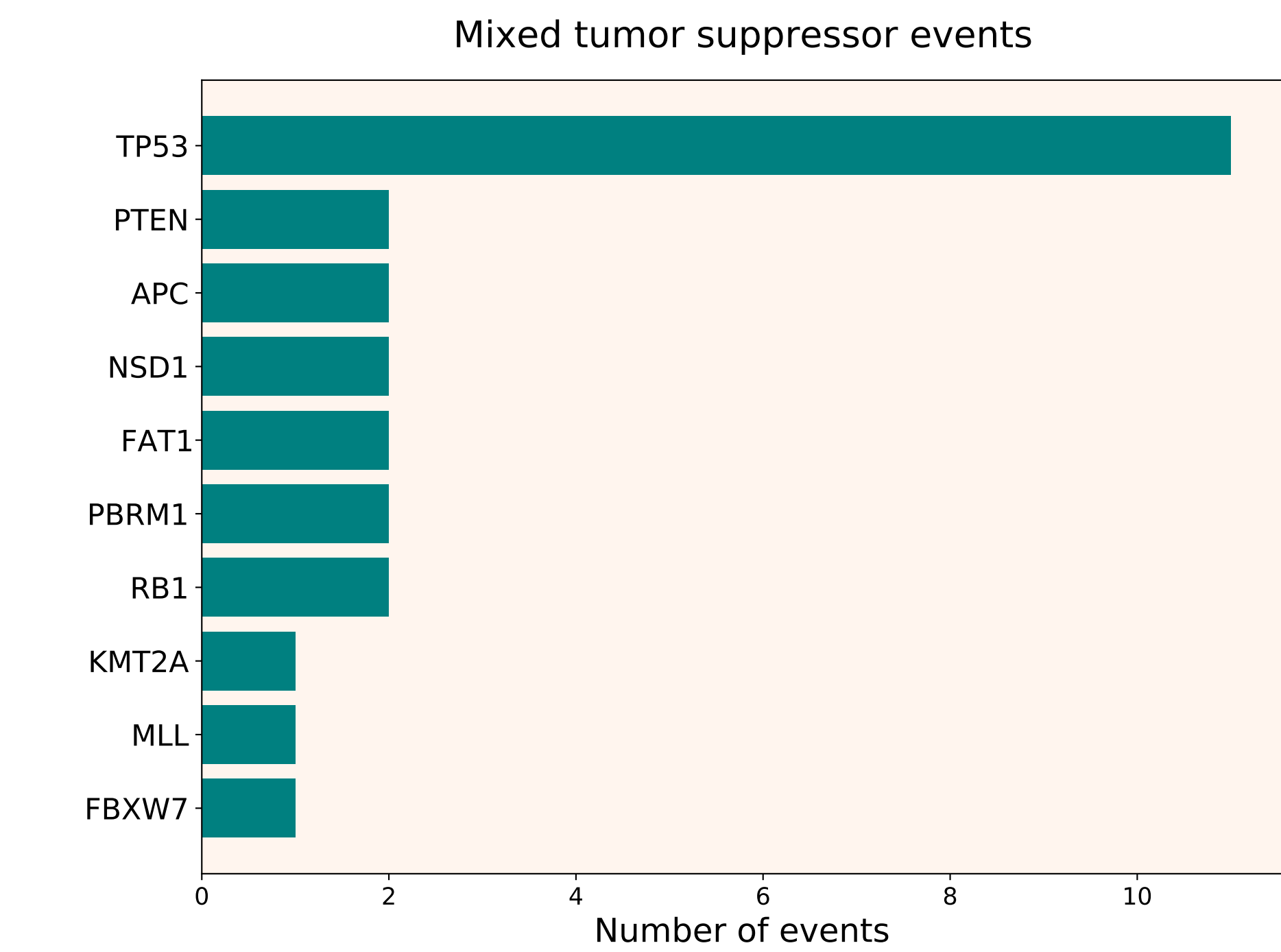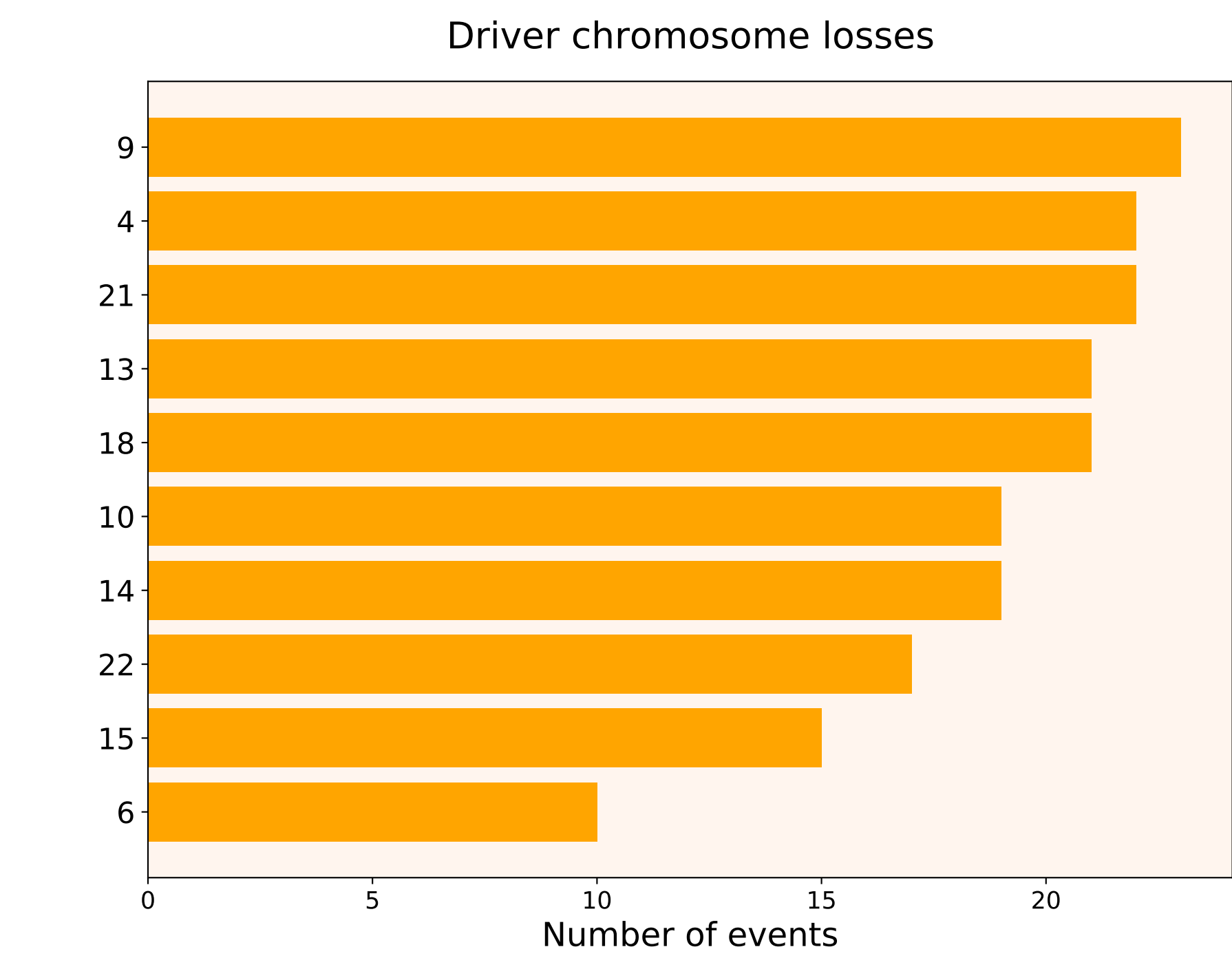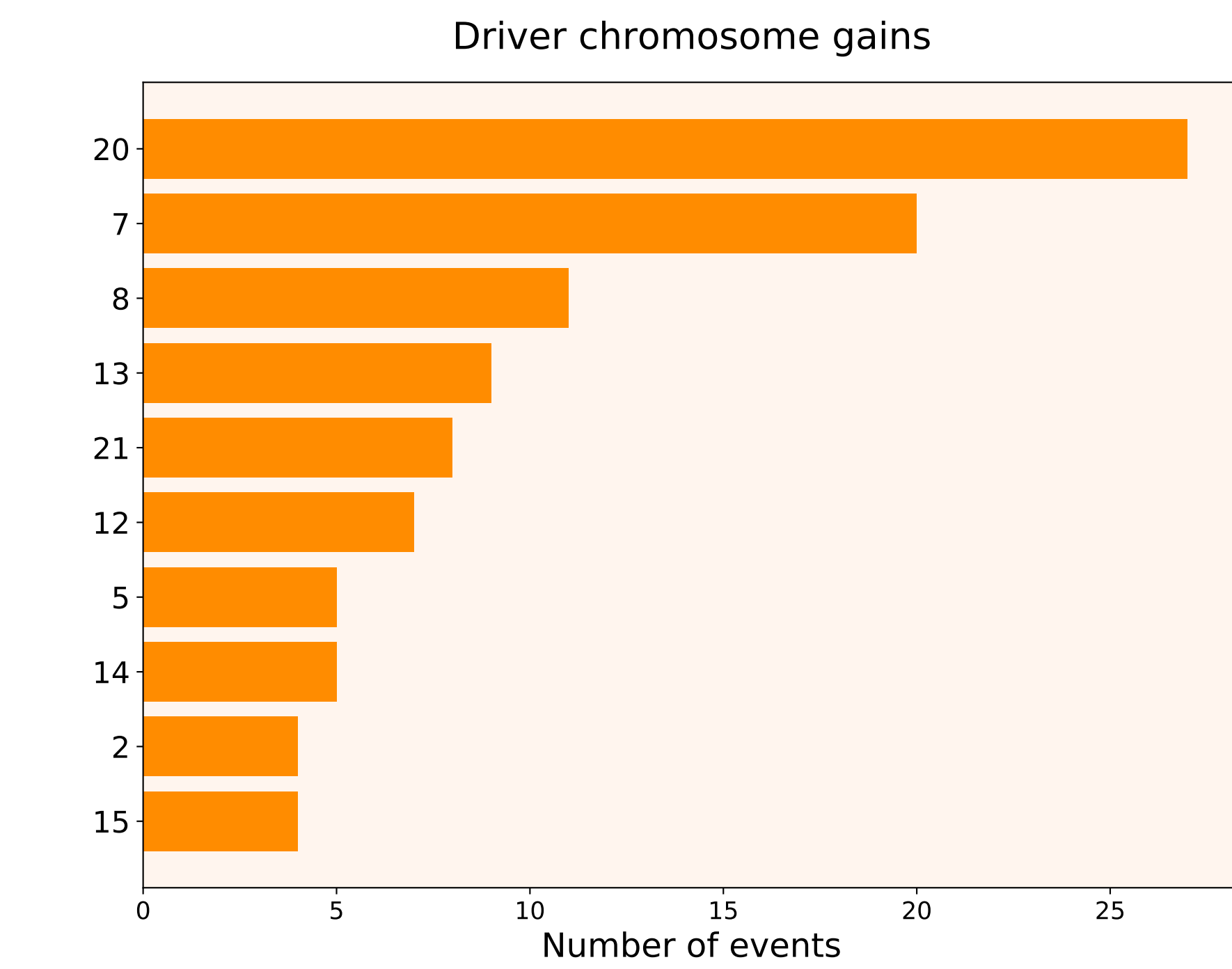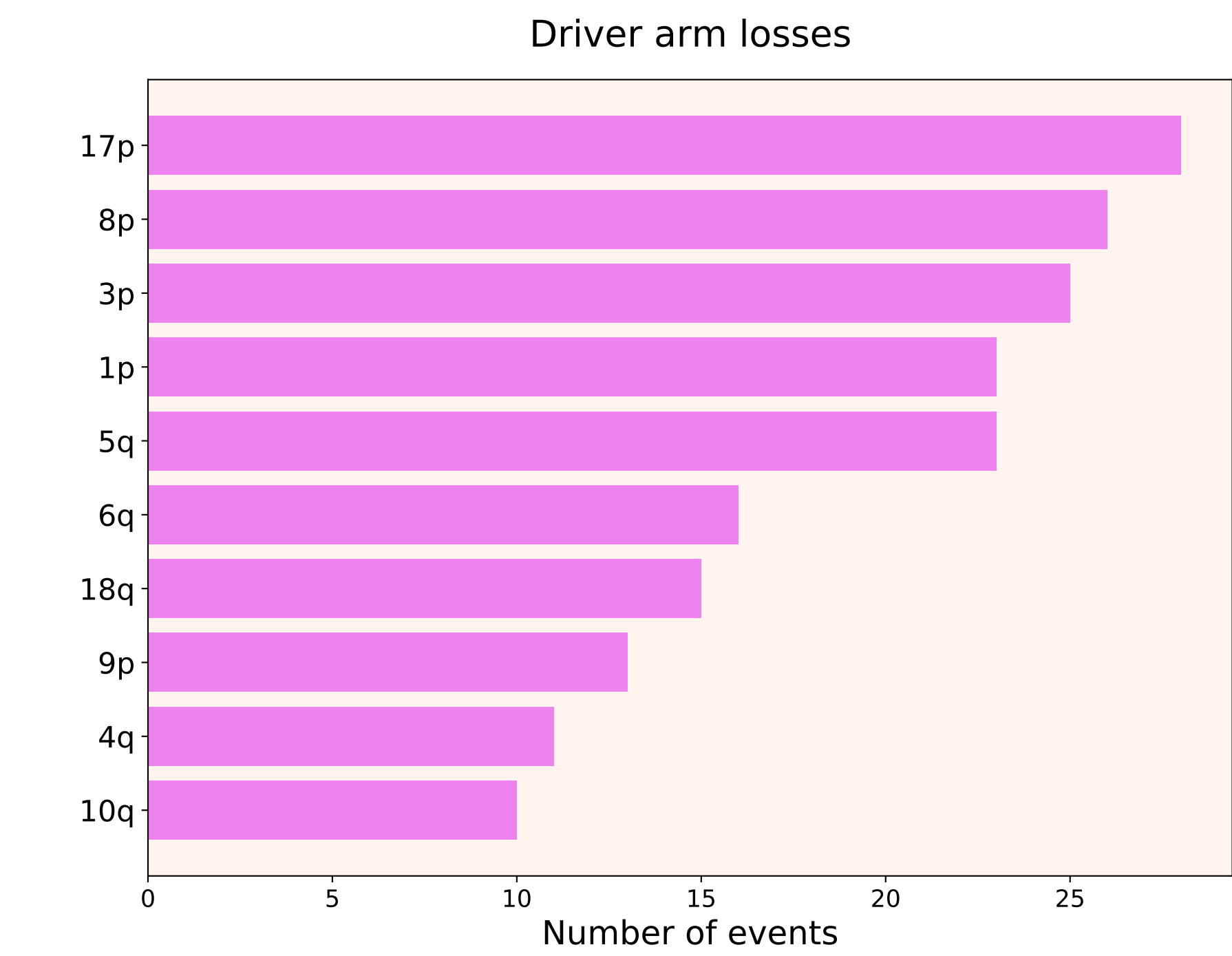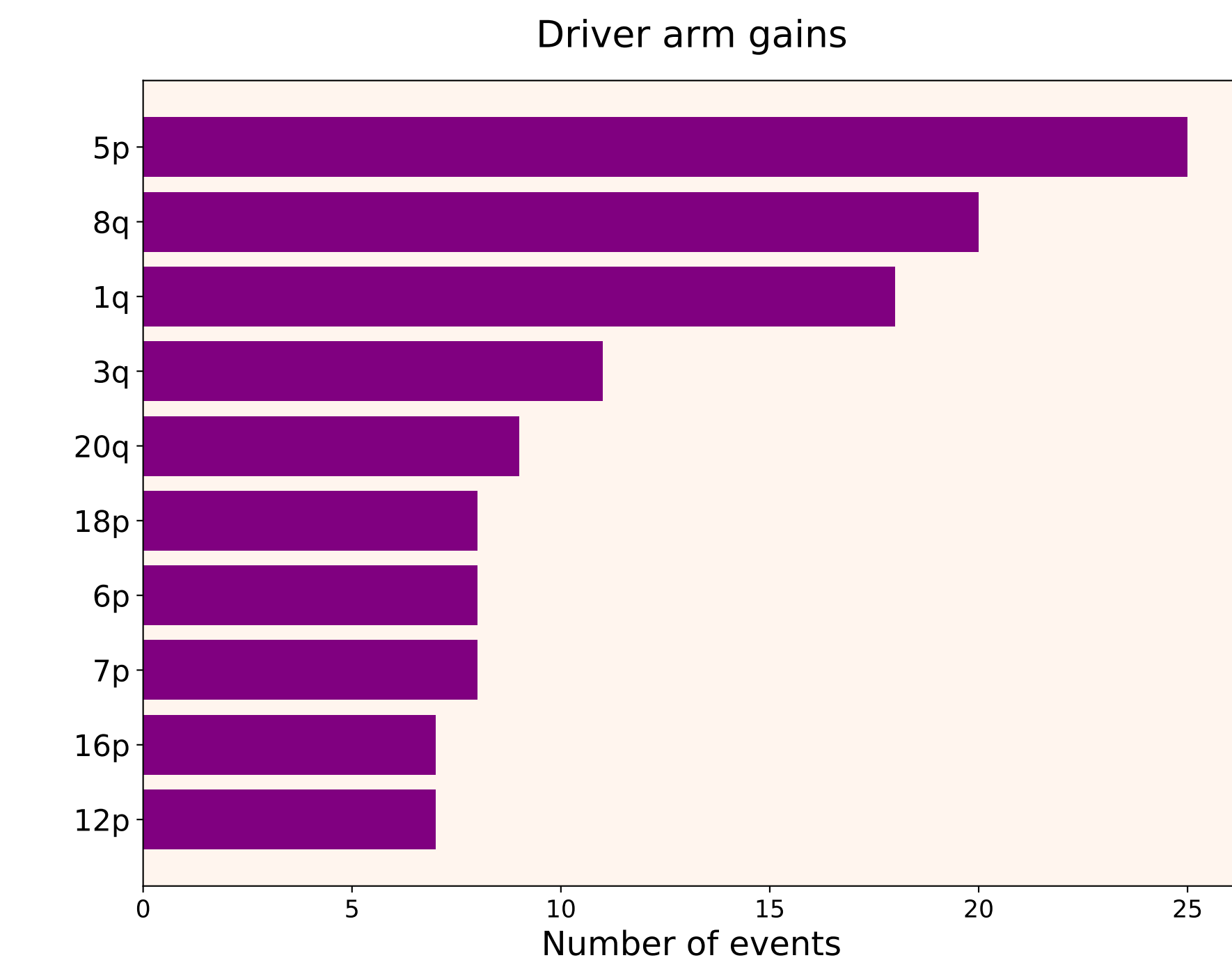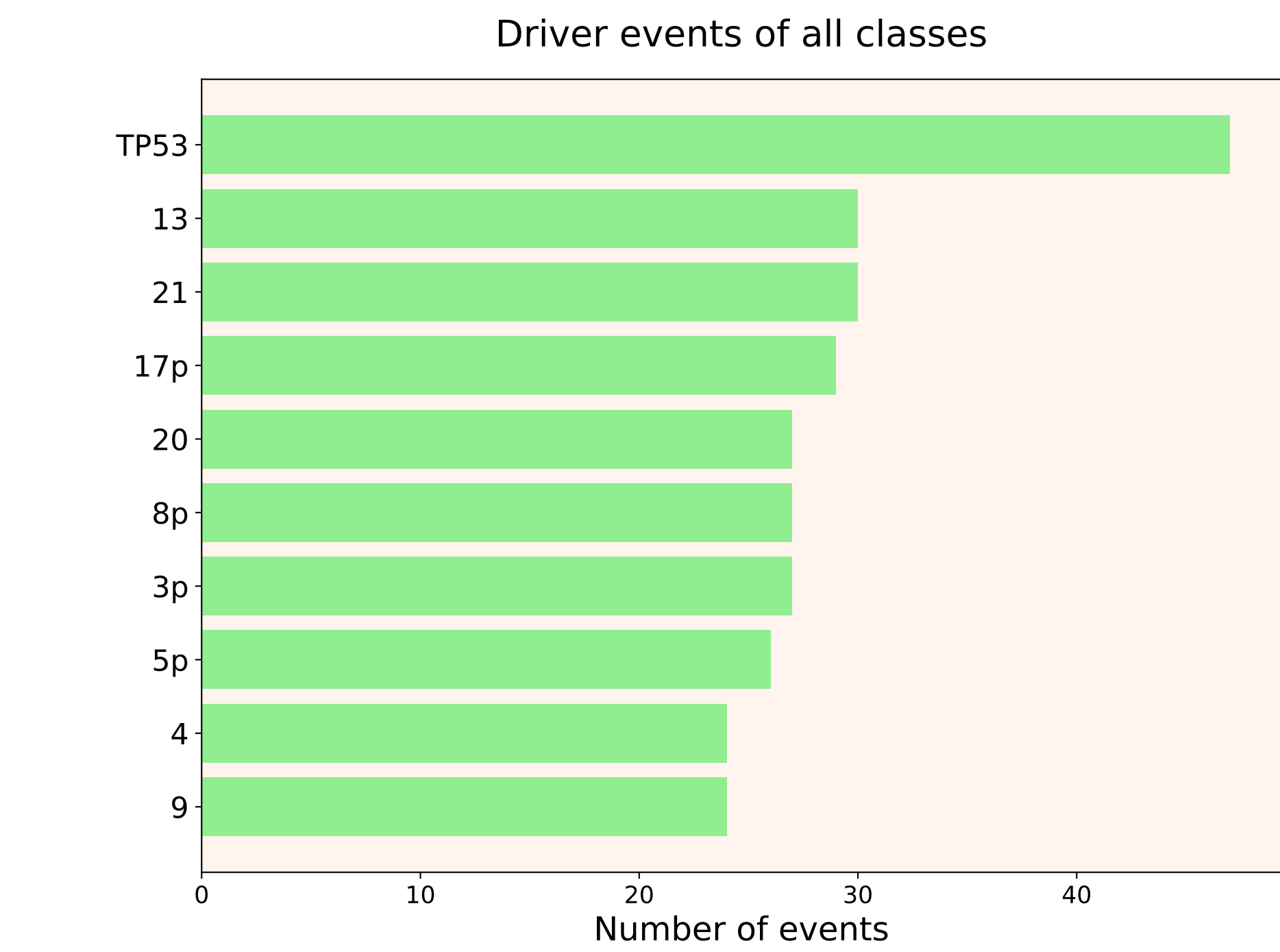

Supplement: Supplemental Information 2 [file peerj-10-13860-s002.zip › COHORTS/genes plots/detailed/2021_8_16_14_9_distribution_events_detailed_males_18.pdf]

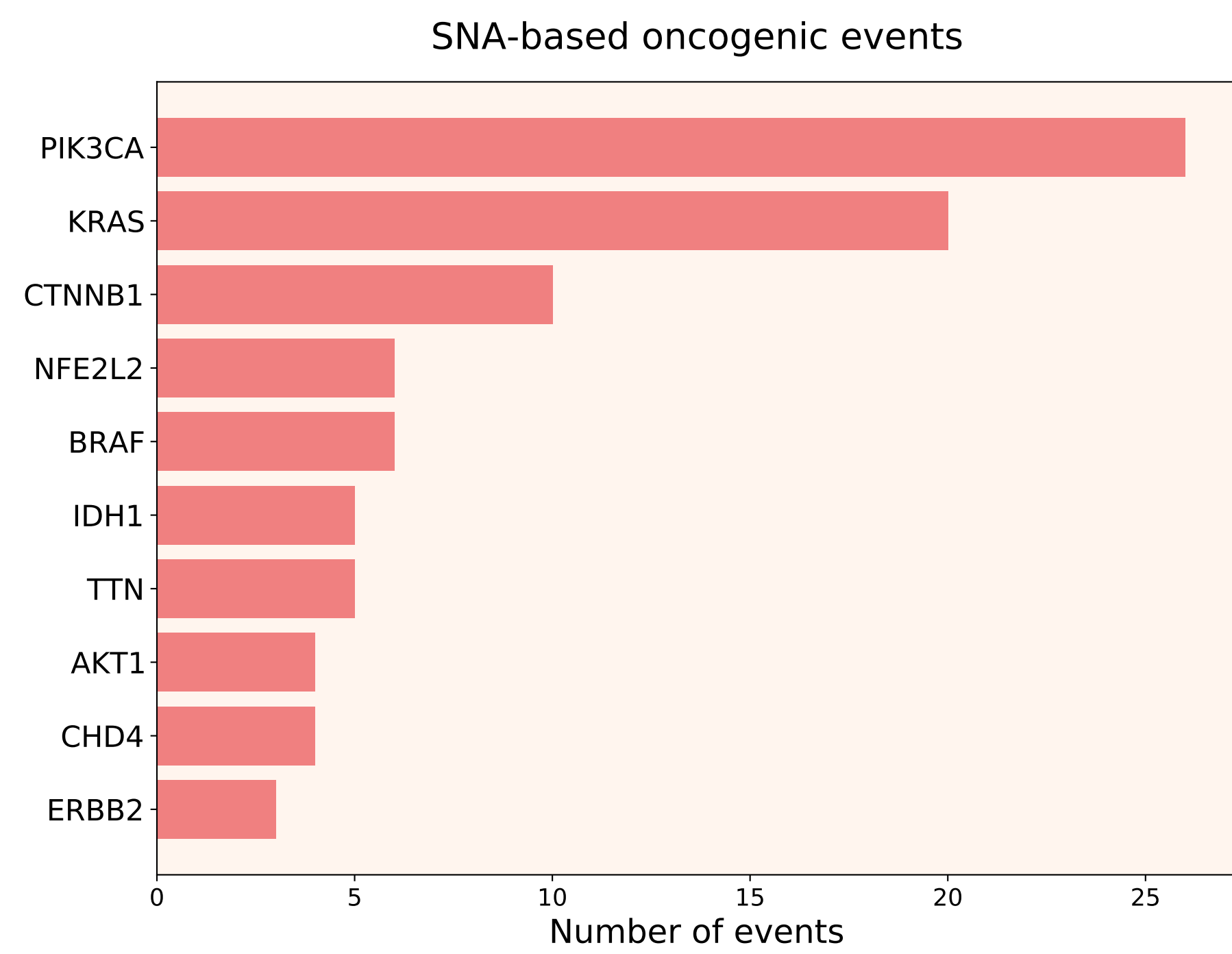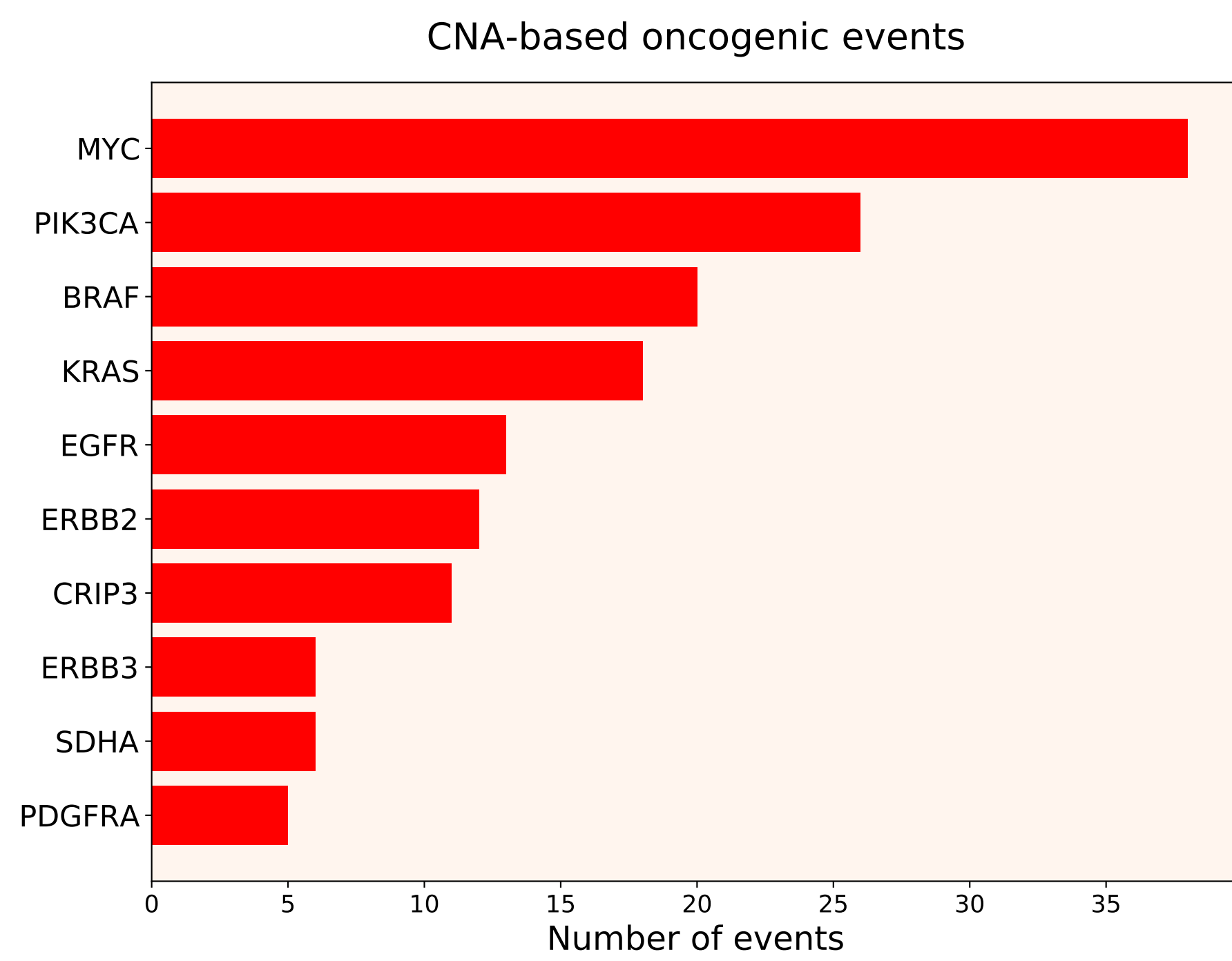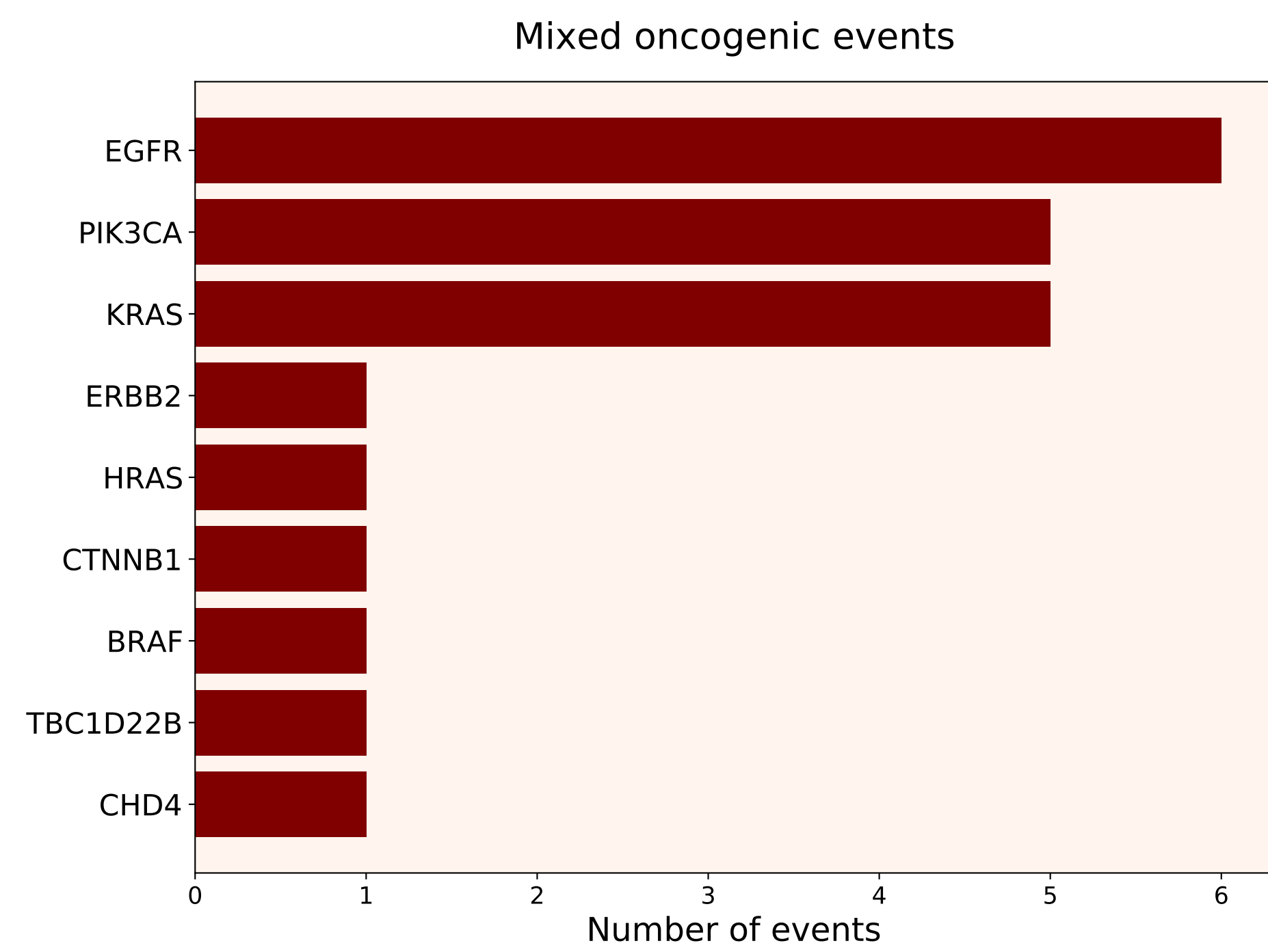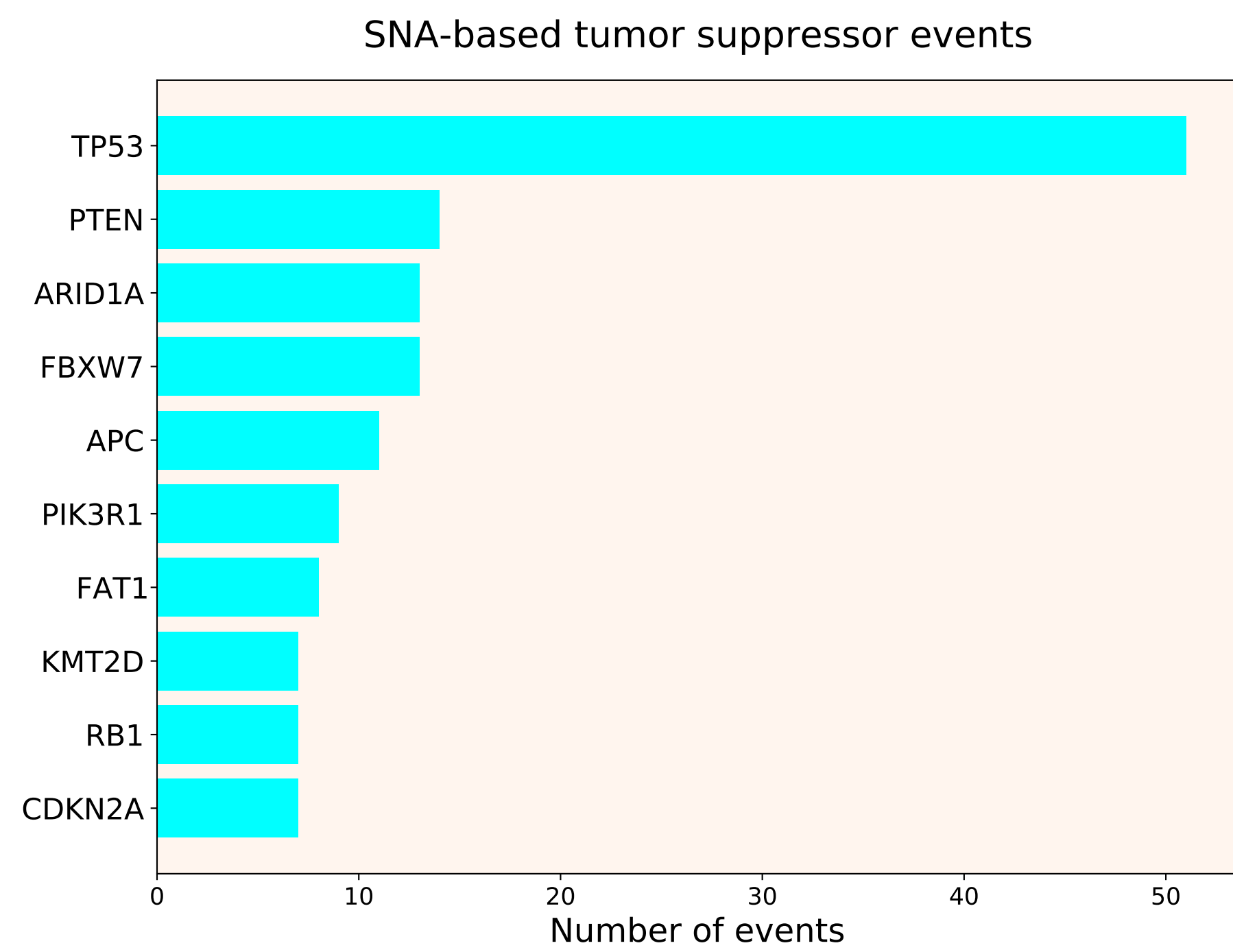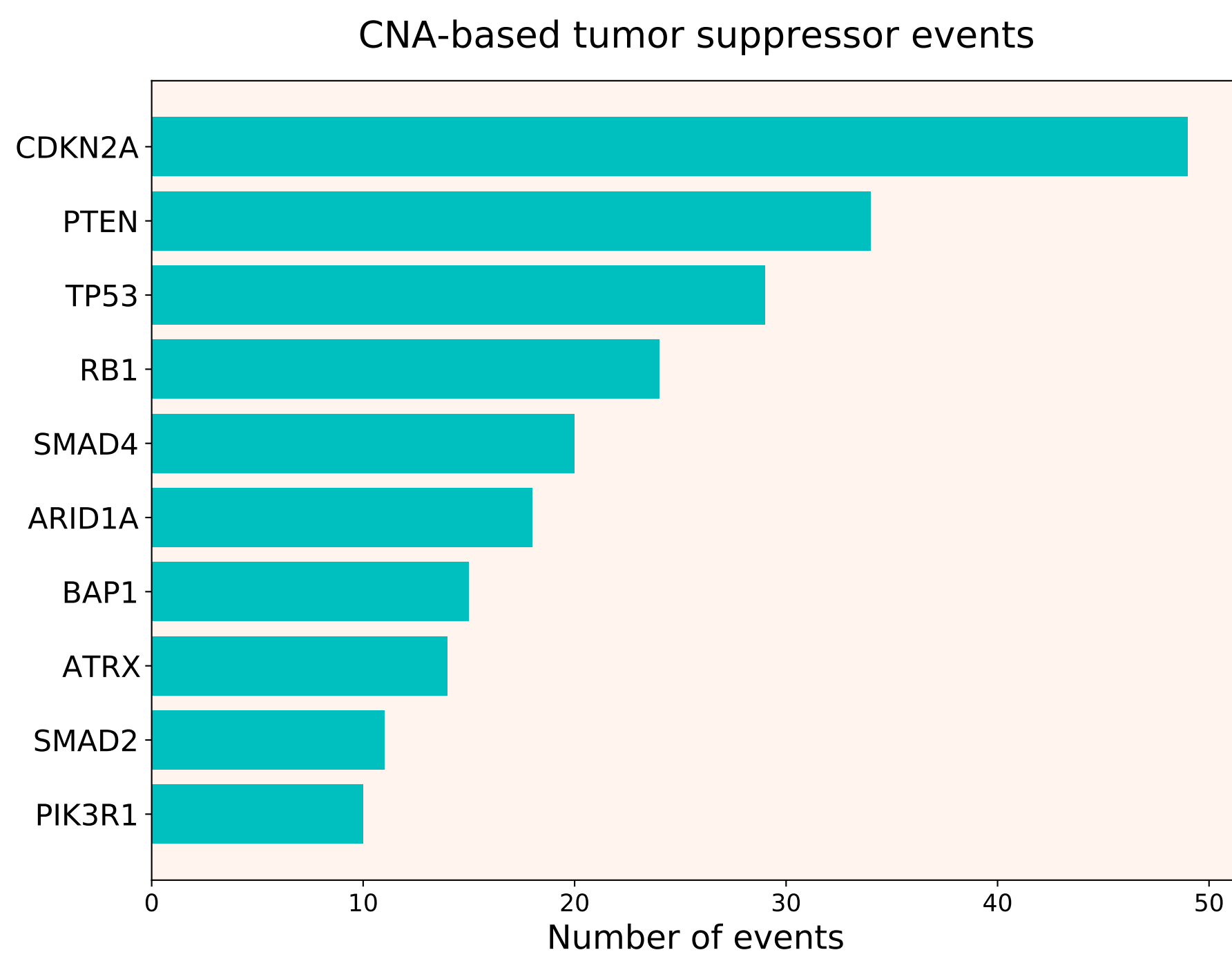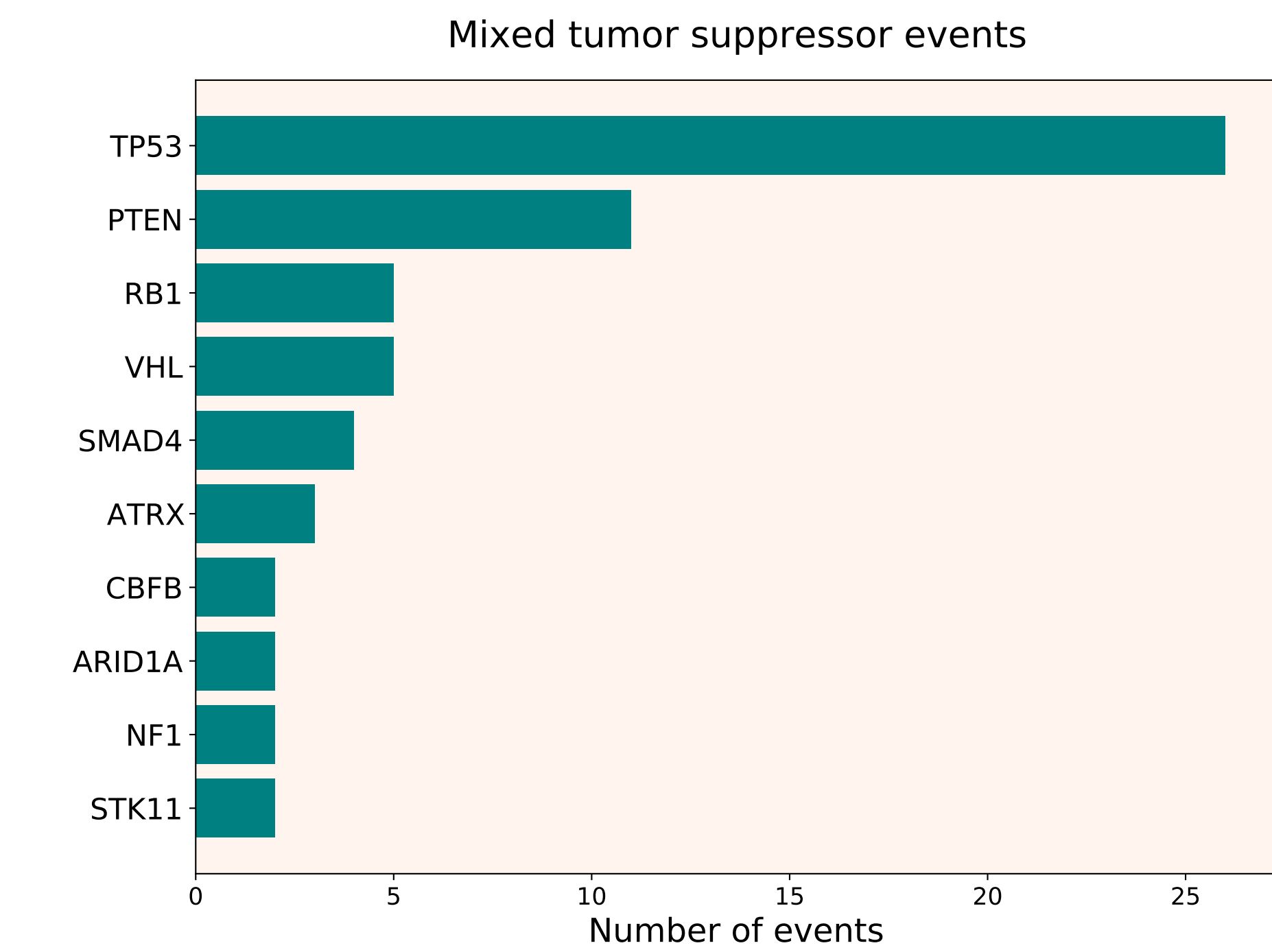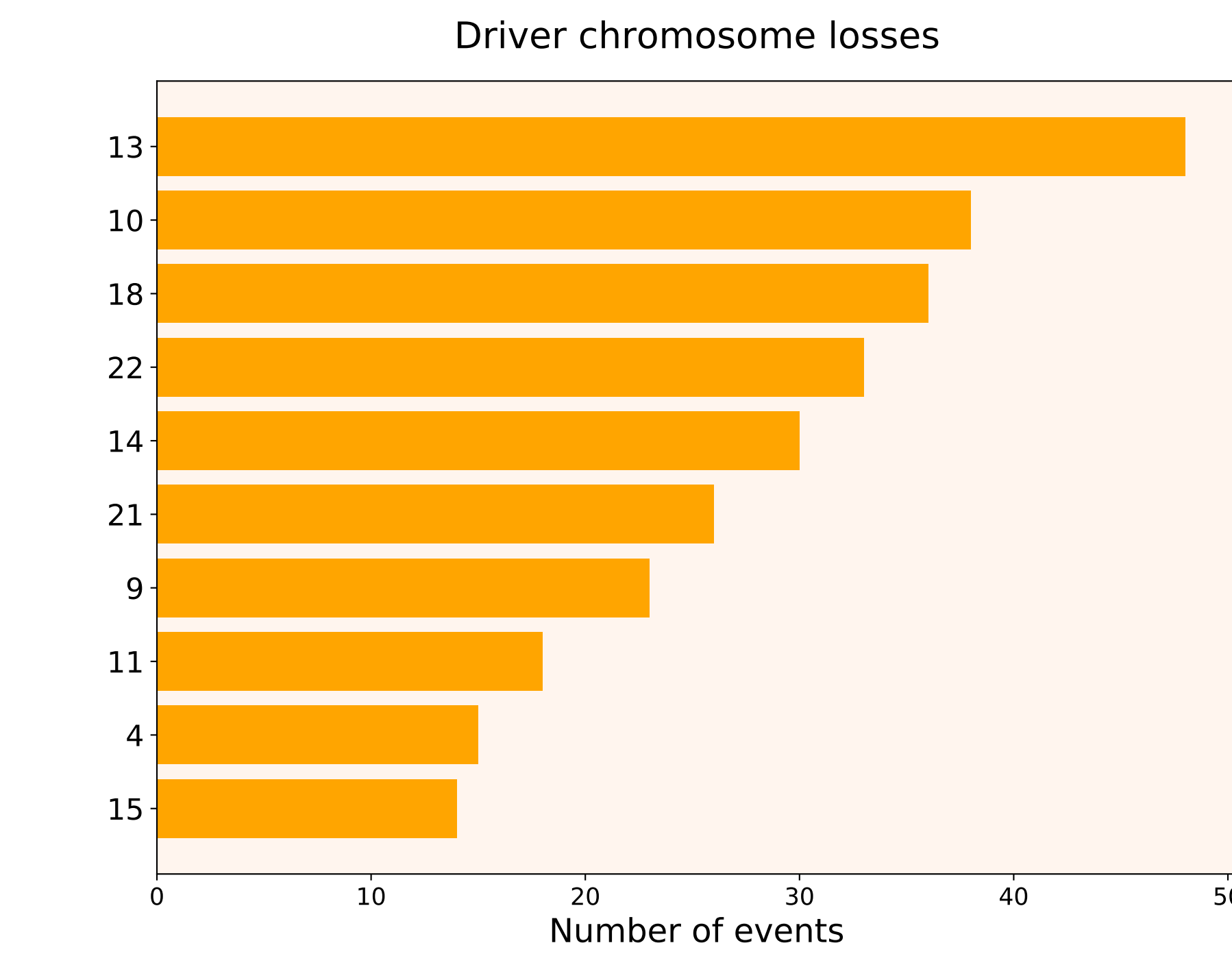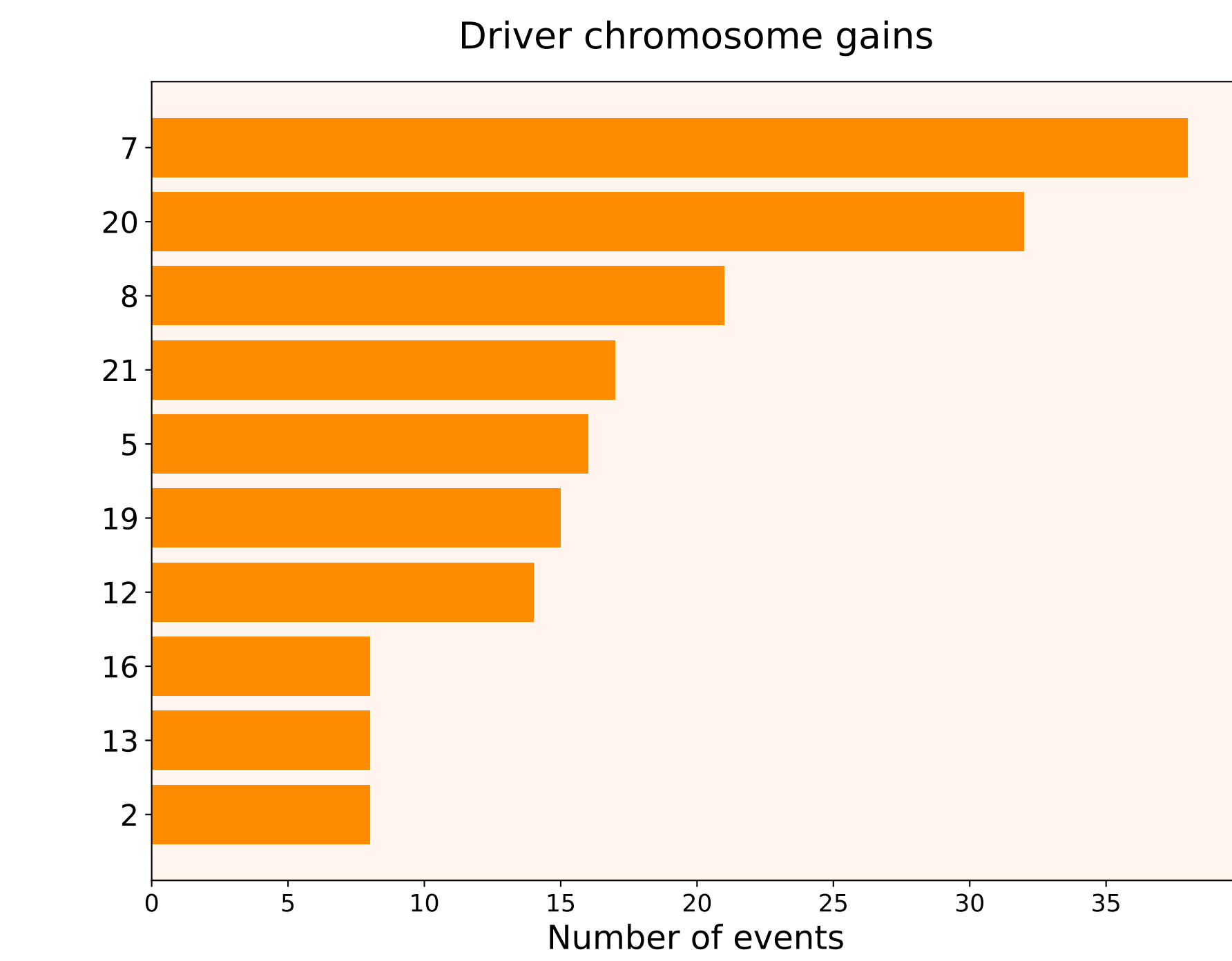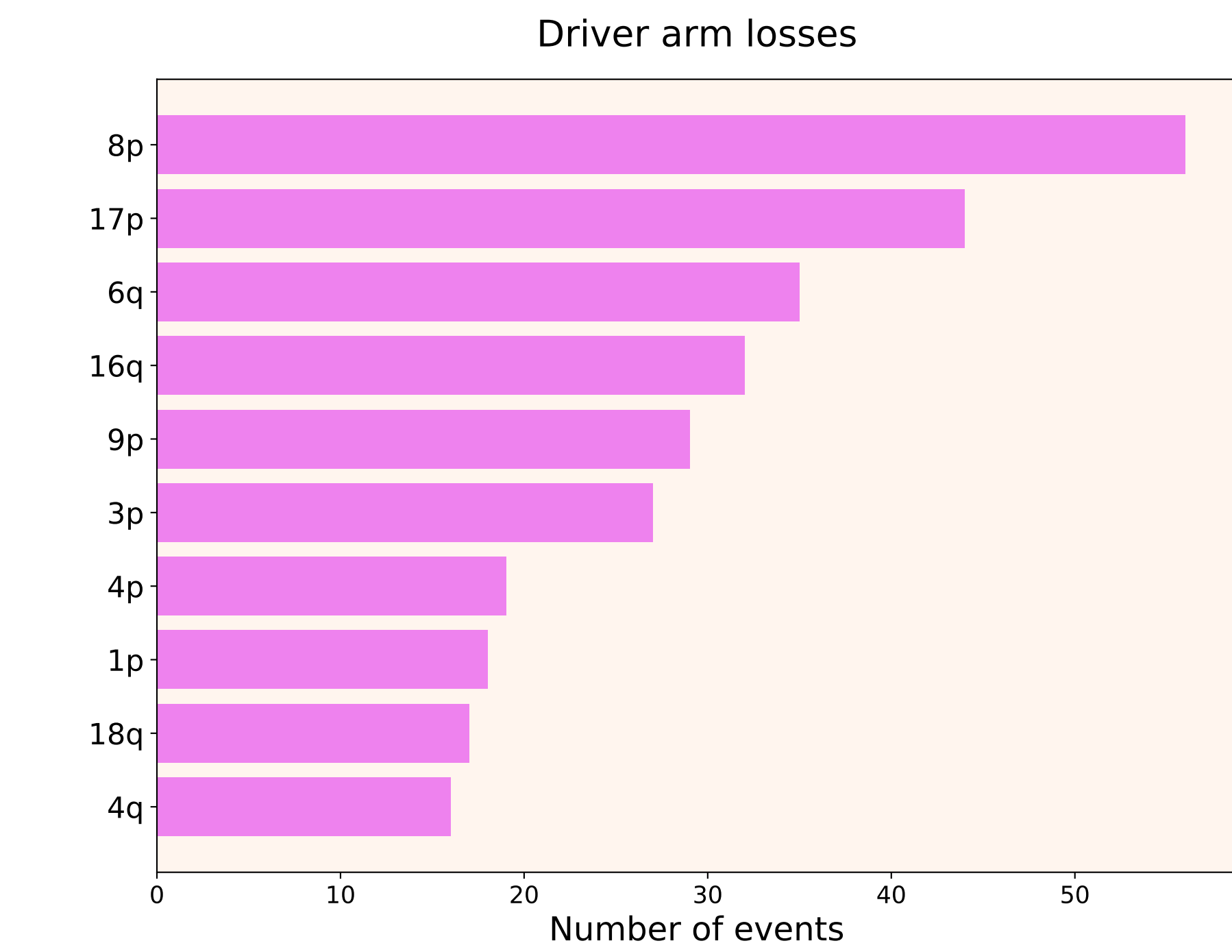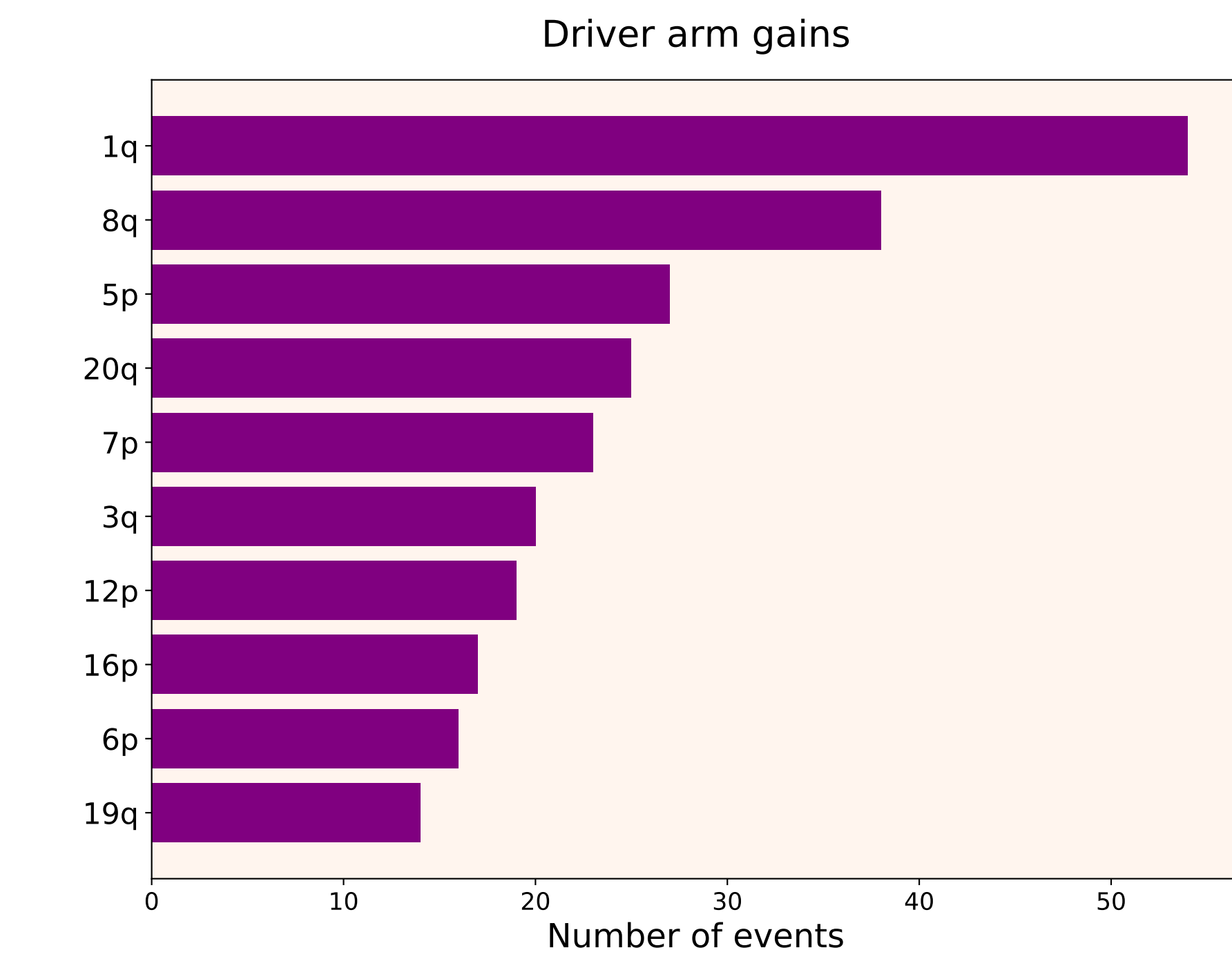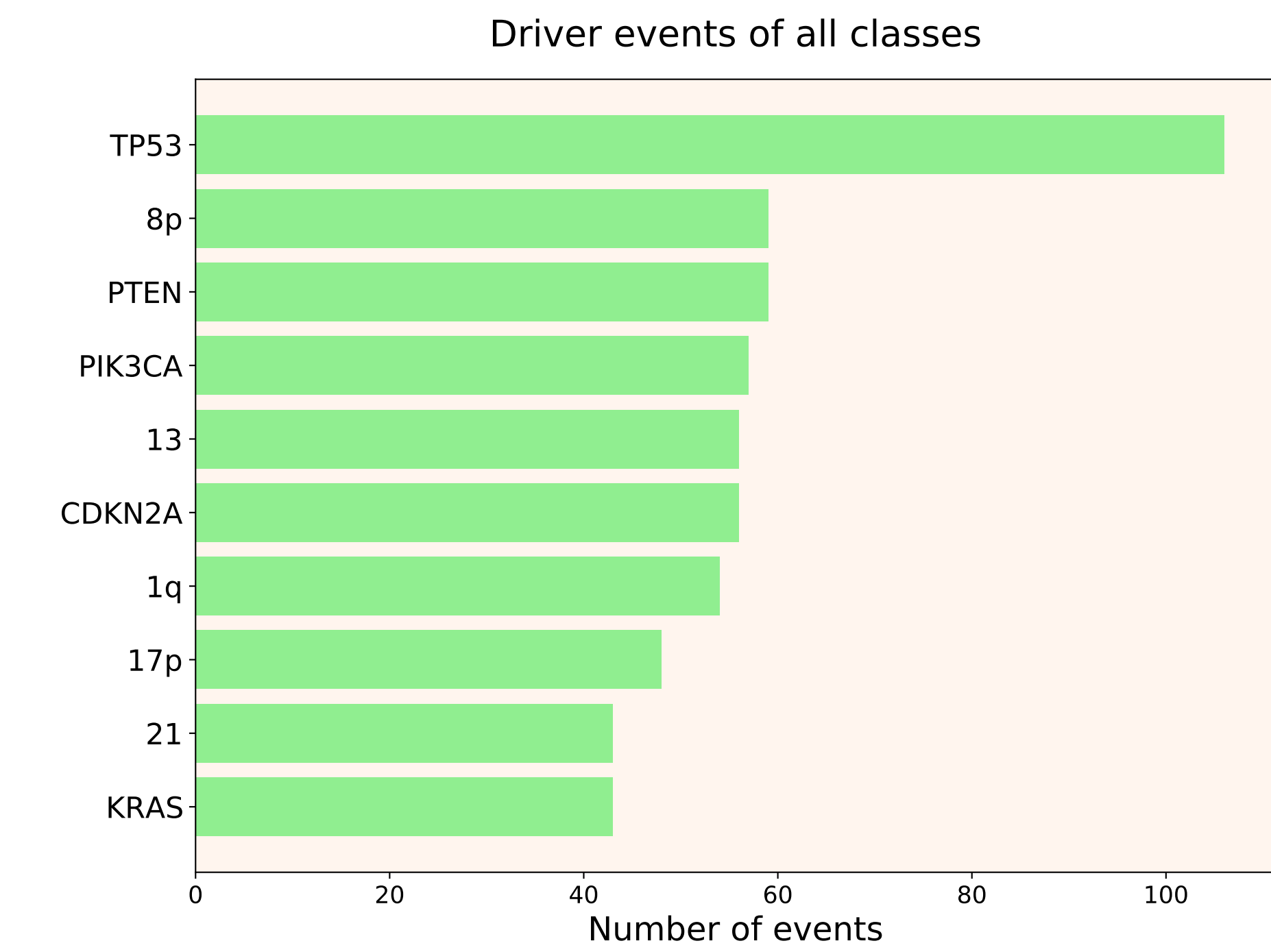

Supplement: Supplemental Information 2 [file peerj-10-13860-s002.zip › COHORTS/genes plots/detailed/2021_8_16_14_9_distribution_events_detailed_11.pdf]

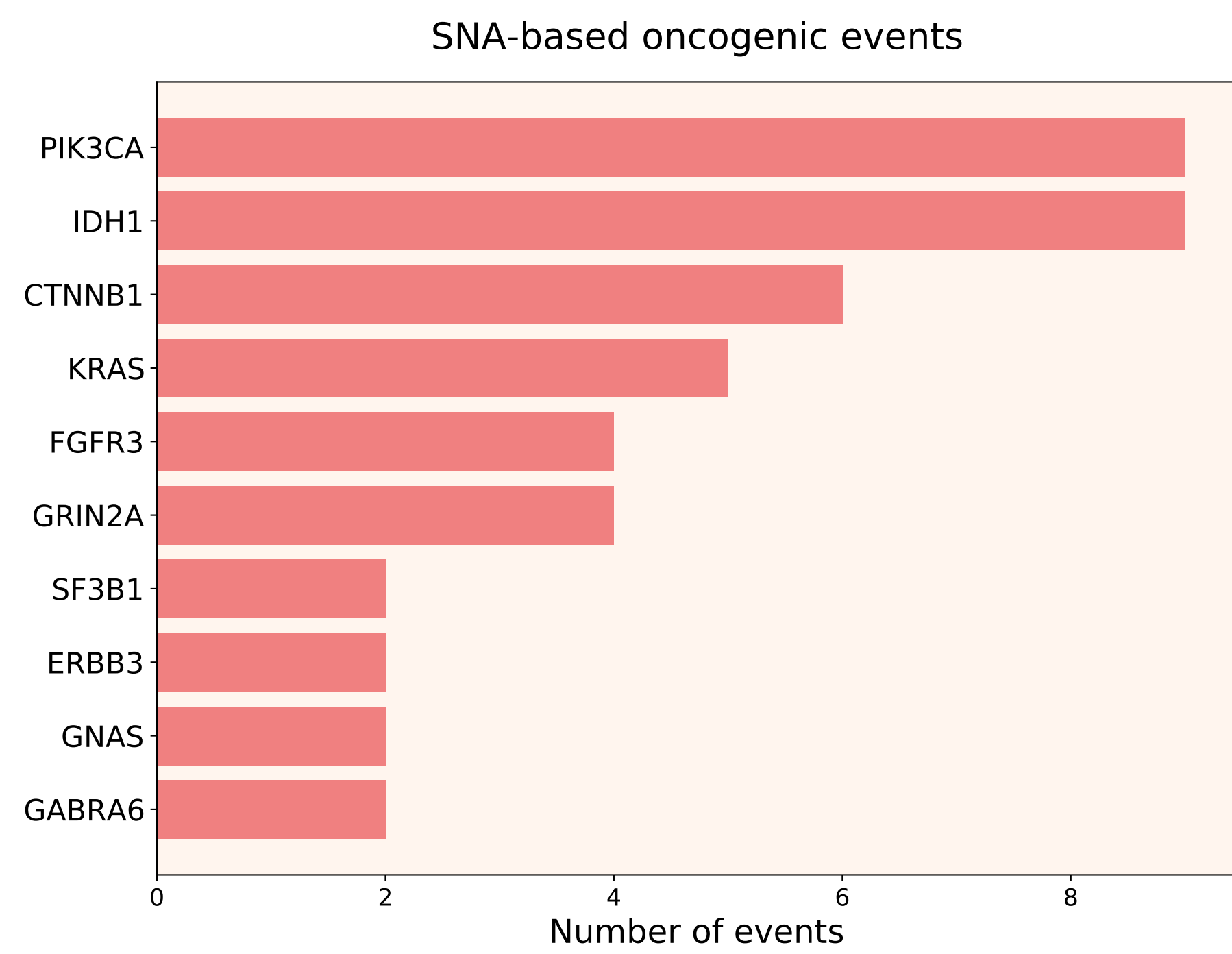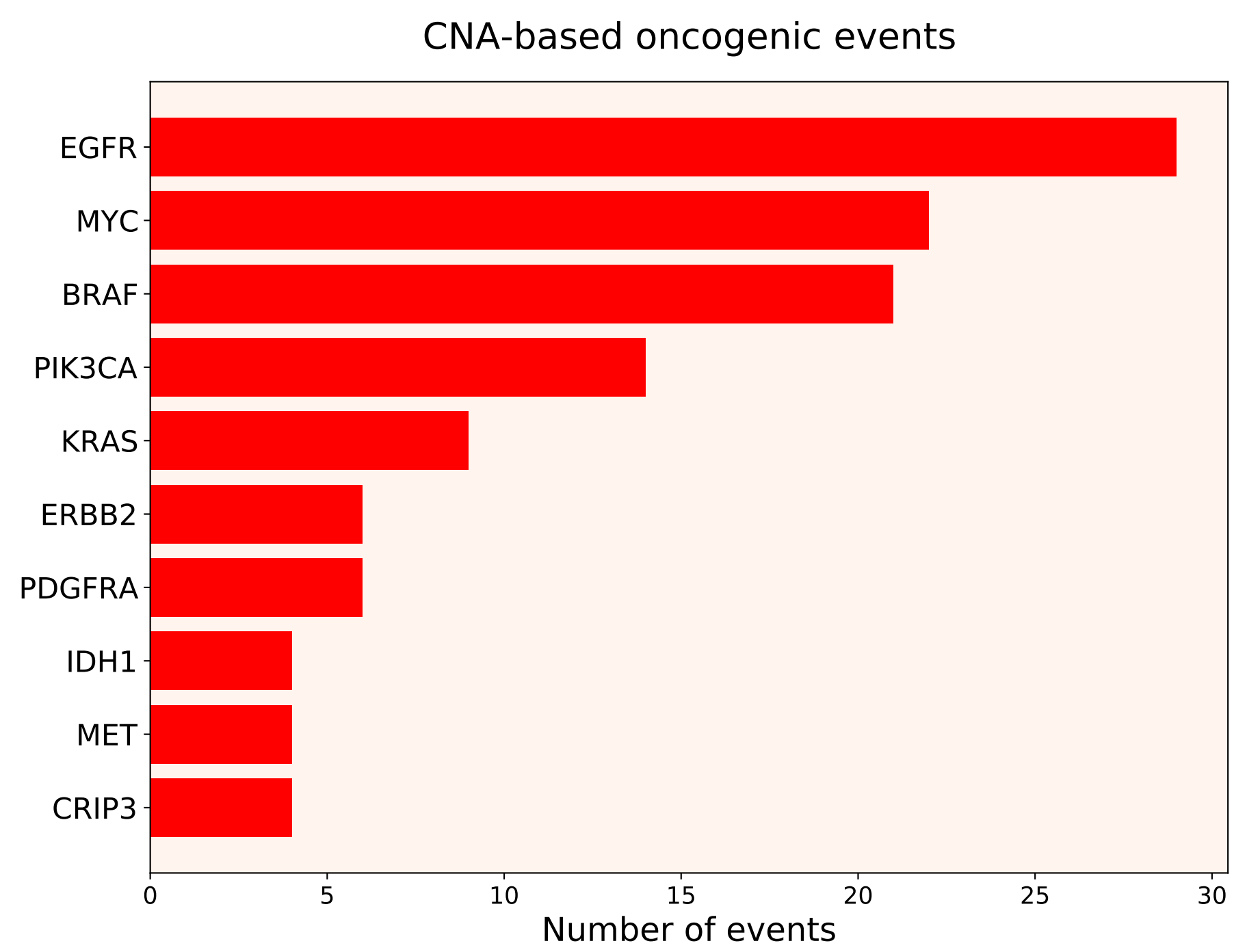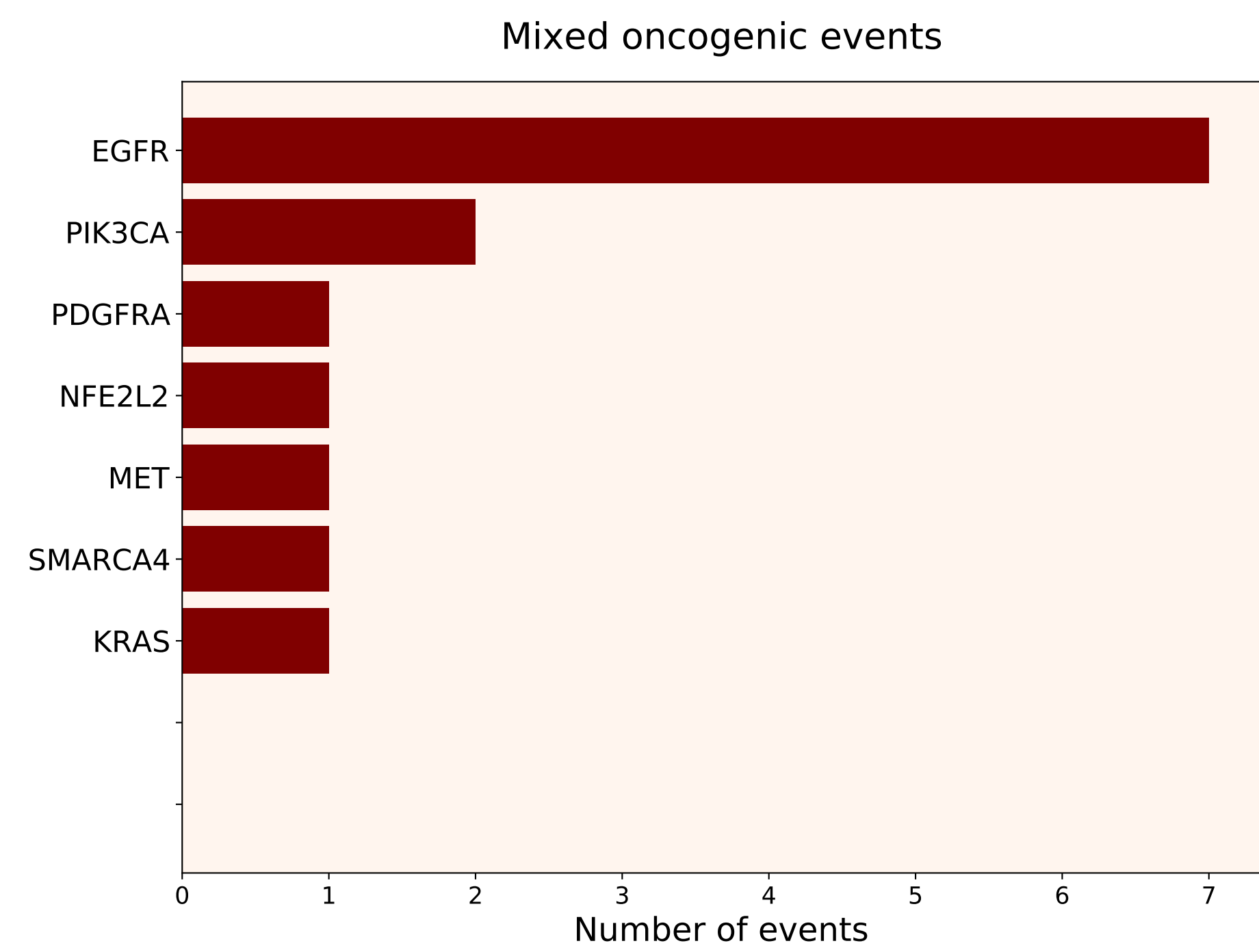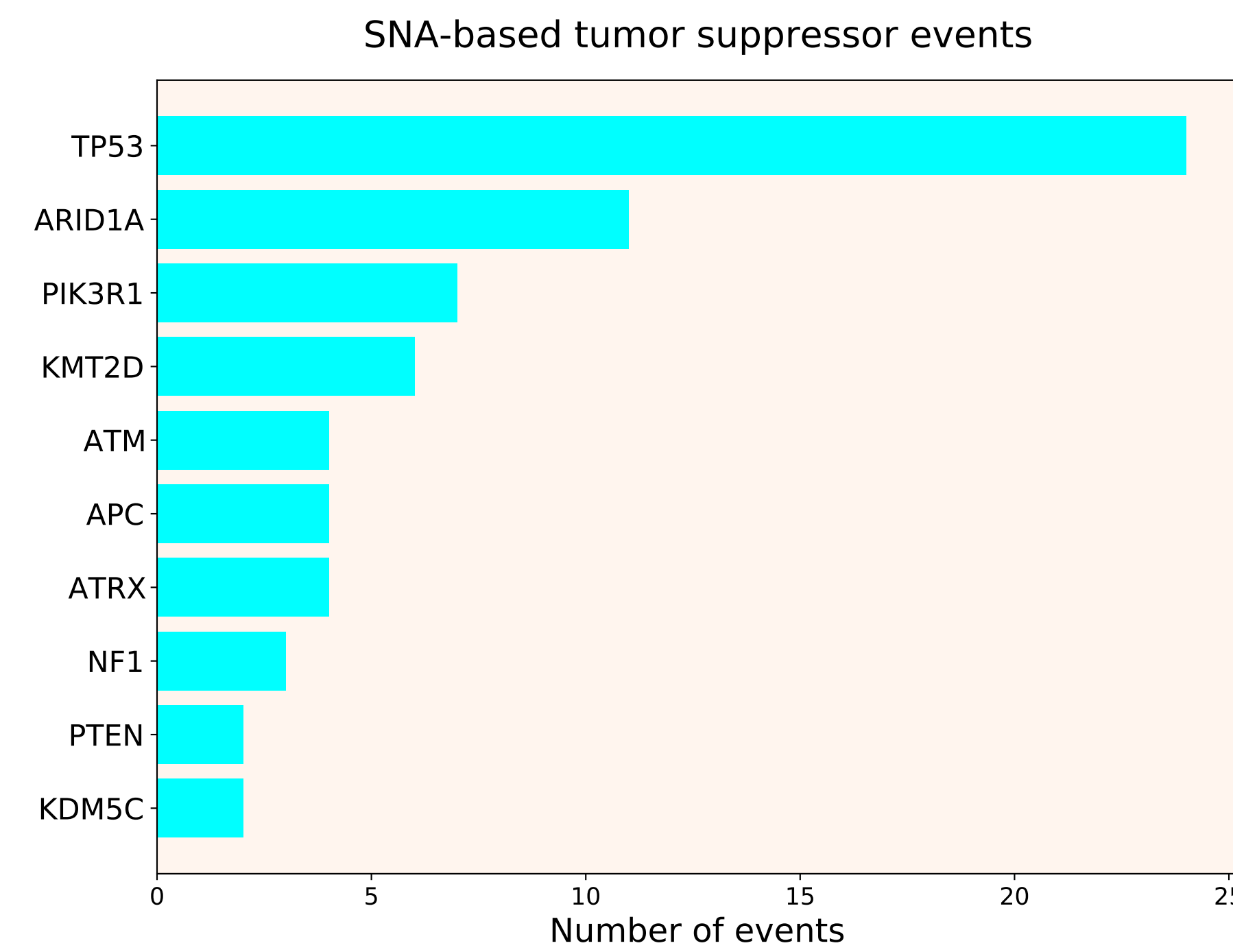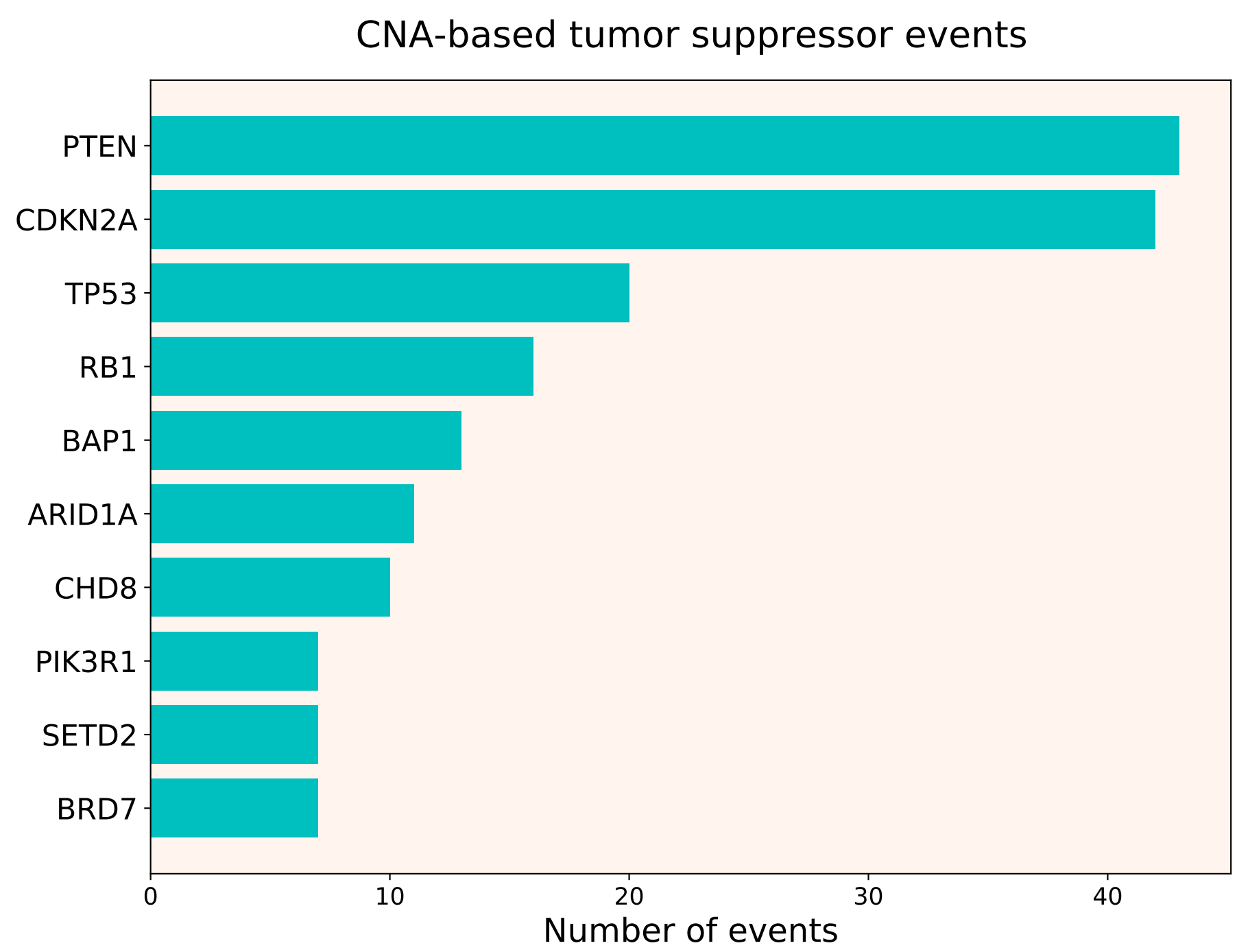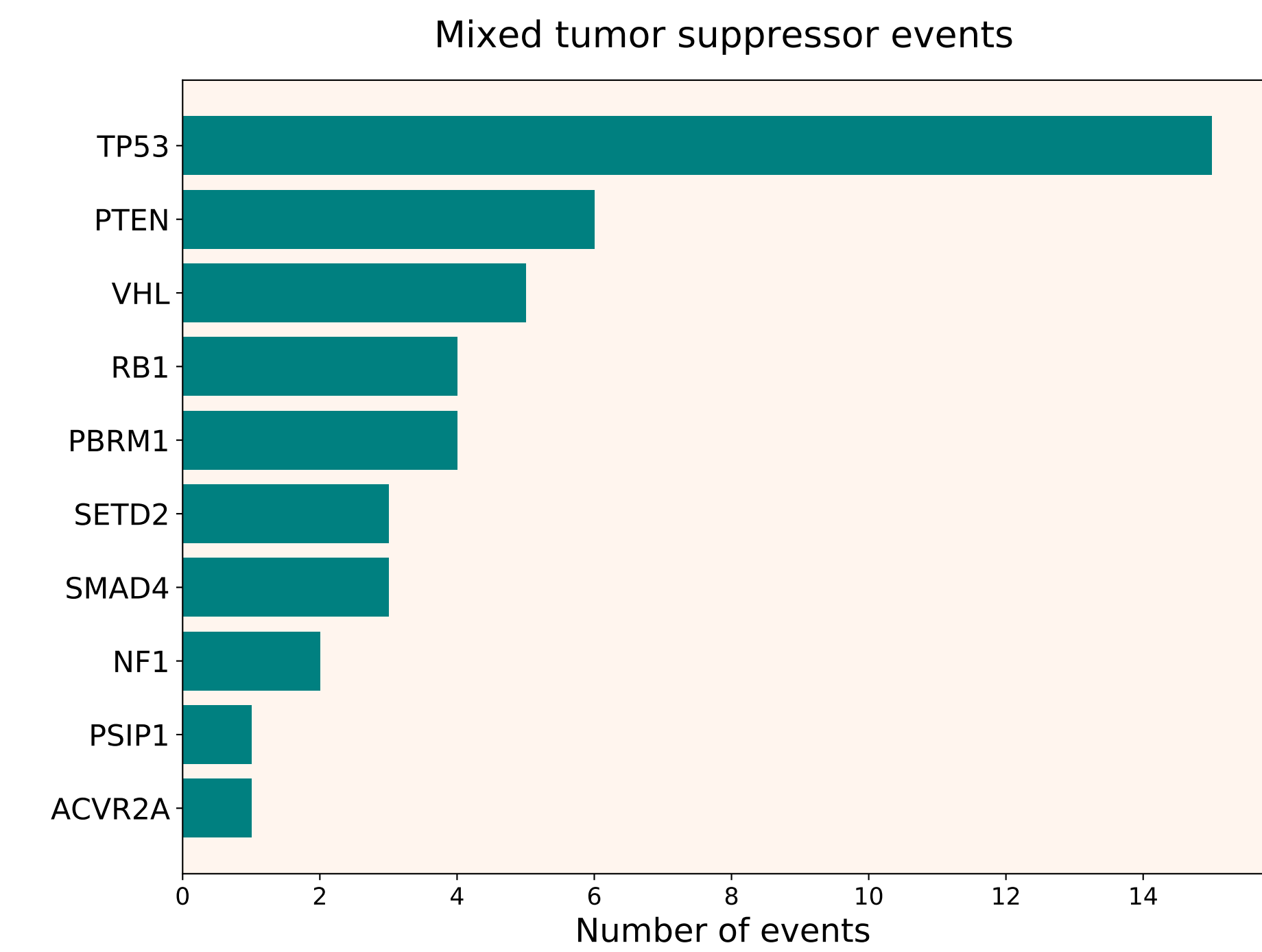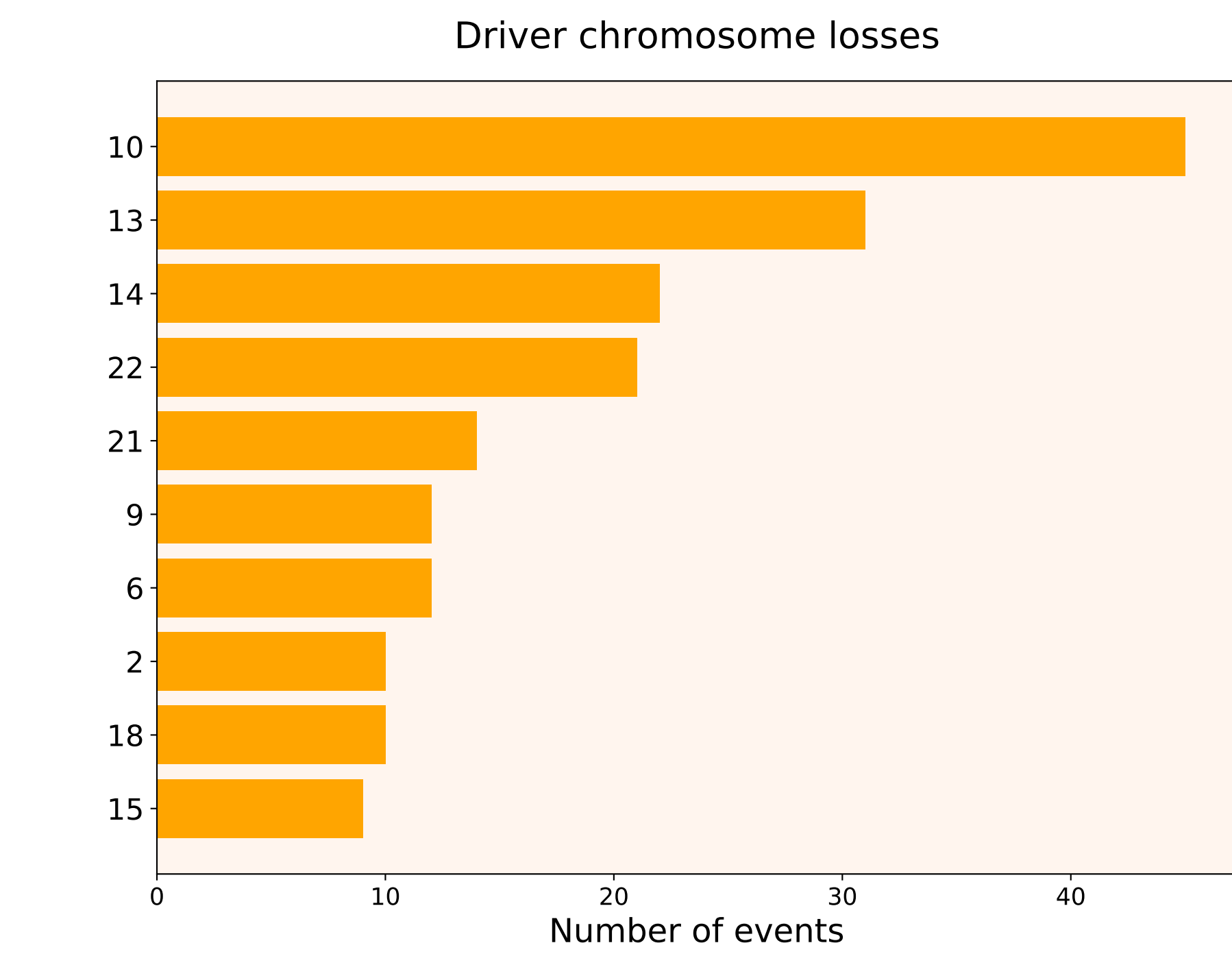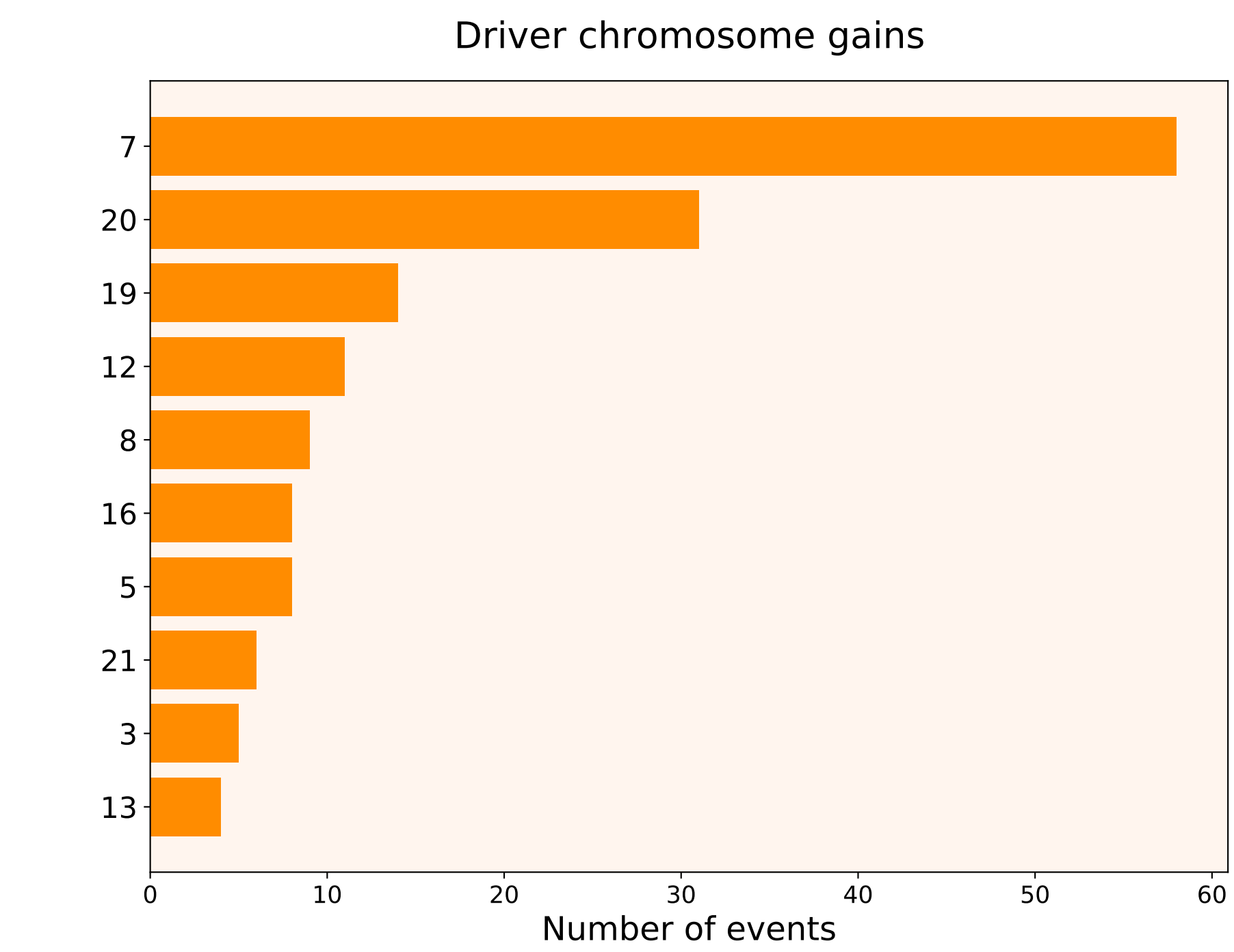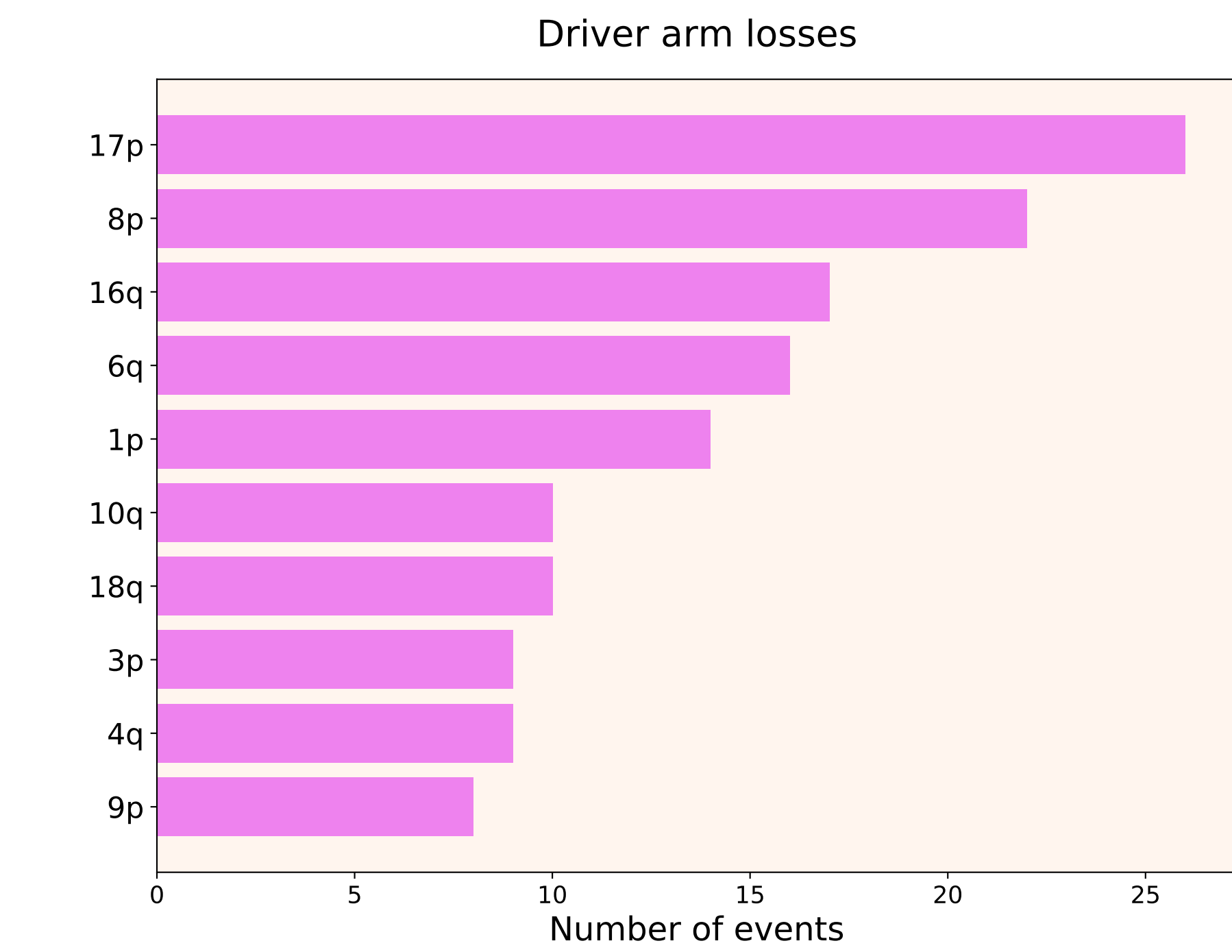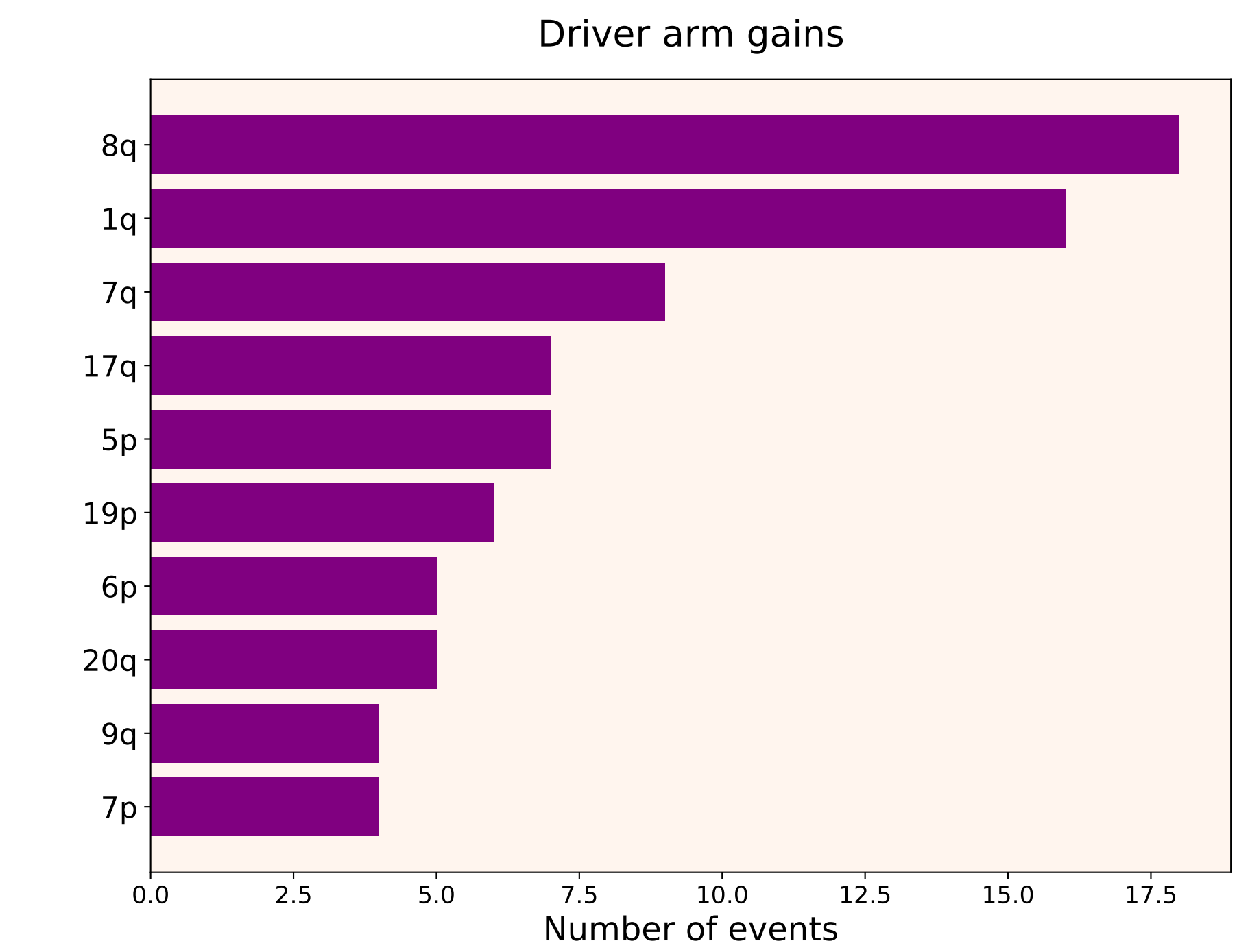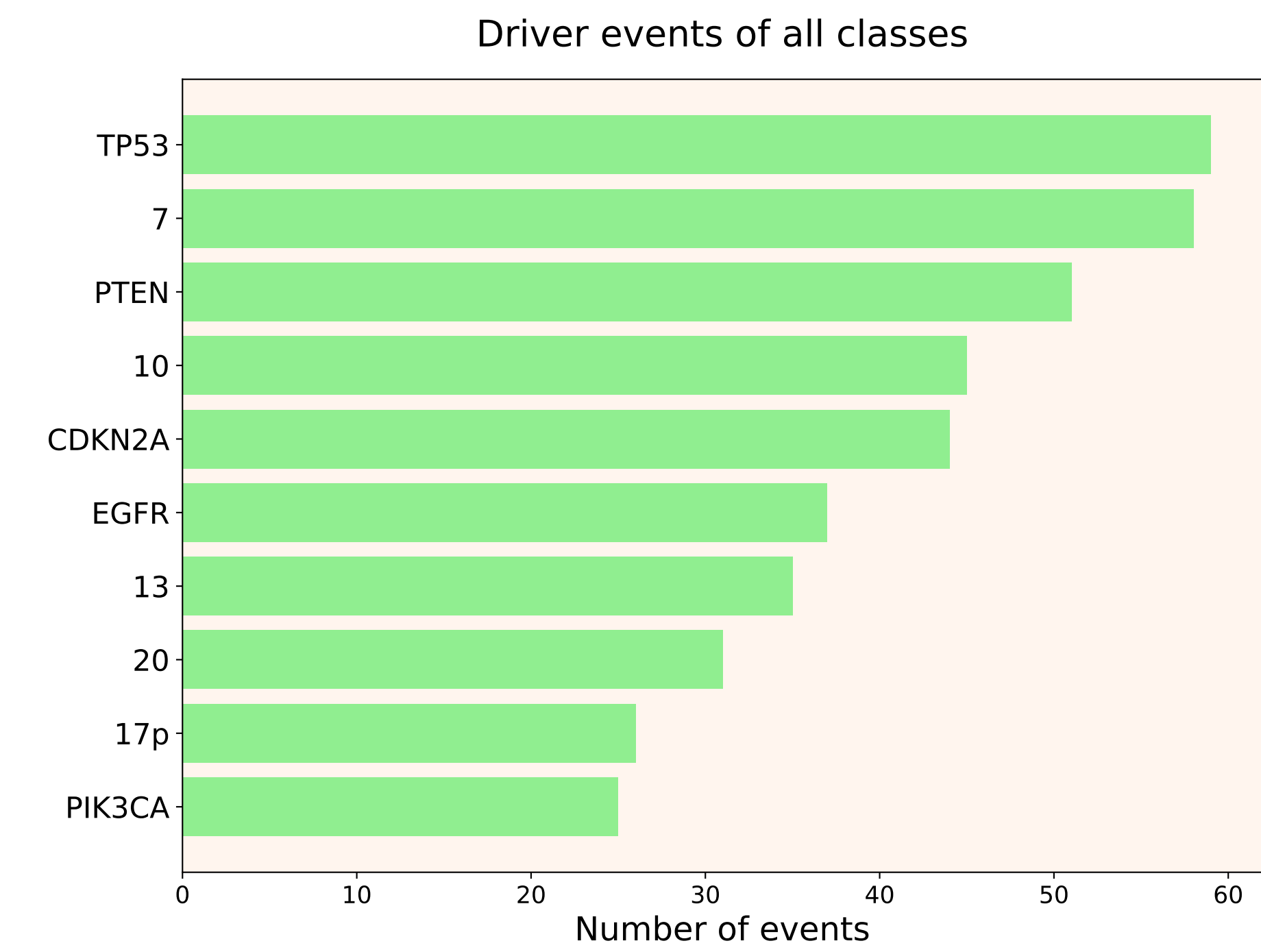

Supplement: Supplemental Information 2 [file peerj-10-13860-s002.zip › COHORTS/genes plots/detailed/2021_8_16_14_9_distribution_events_detailed_males_9.pdf]

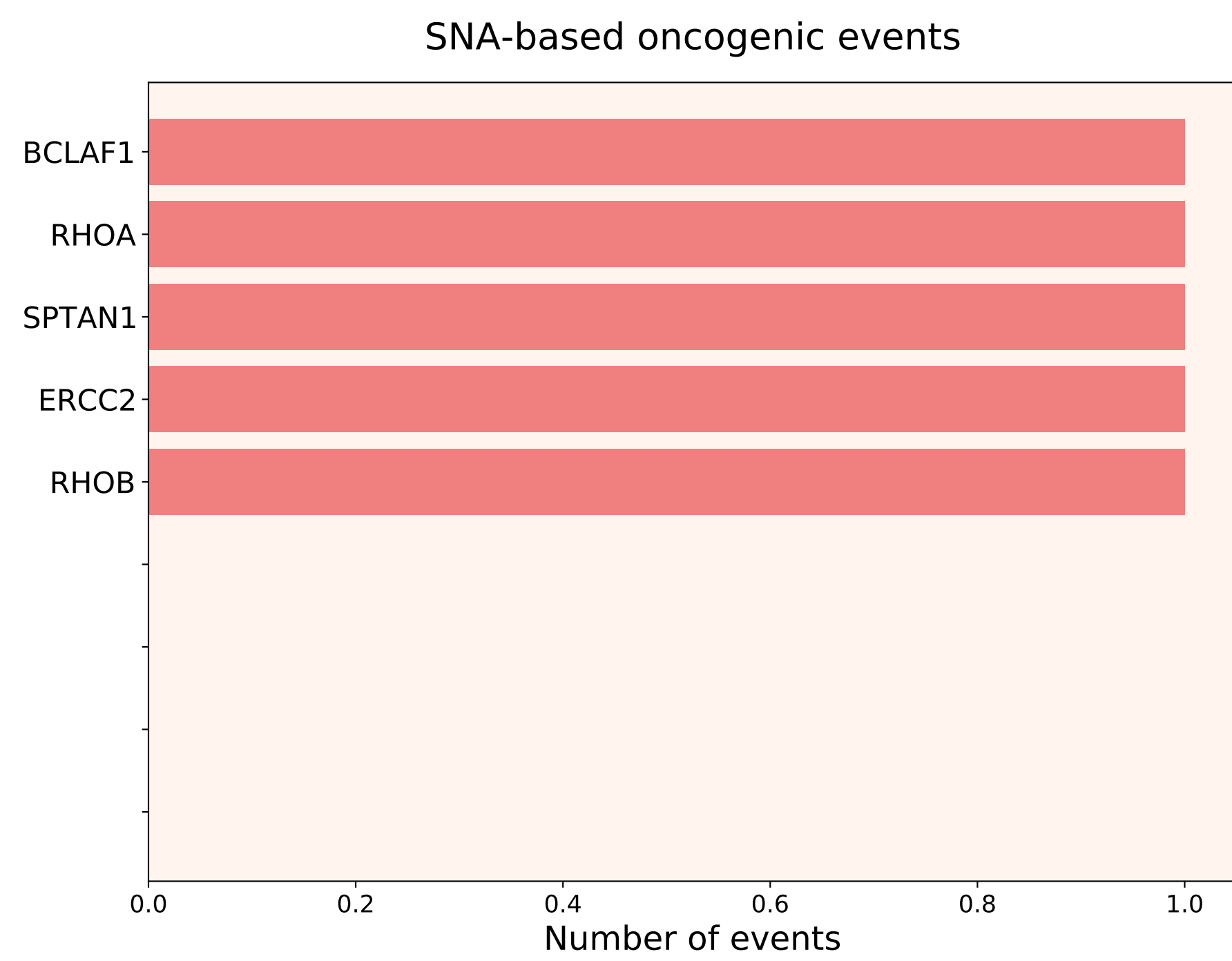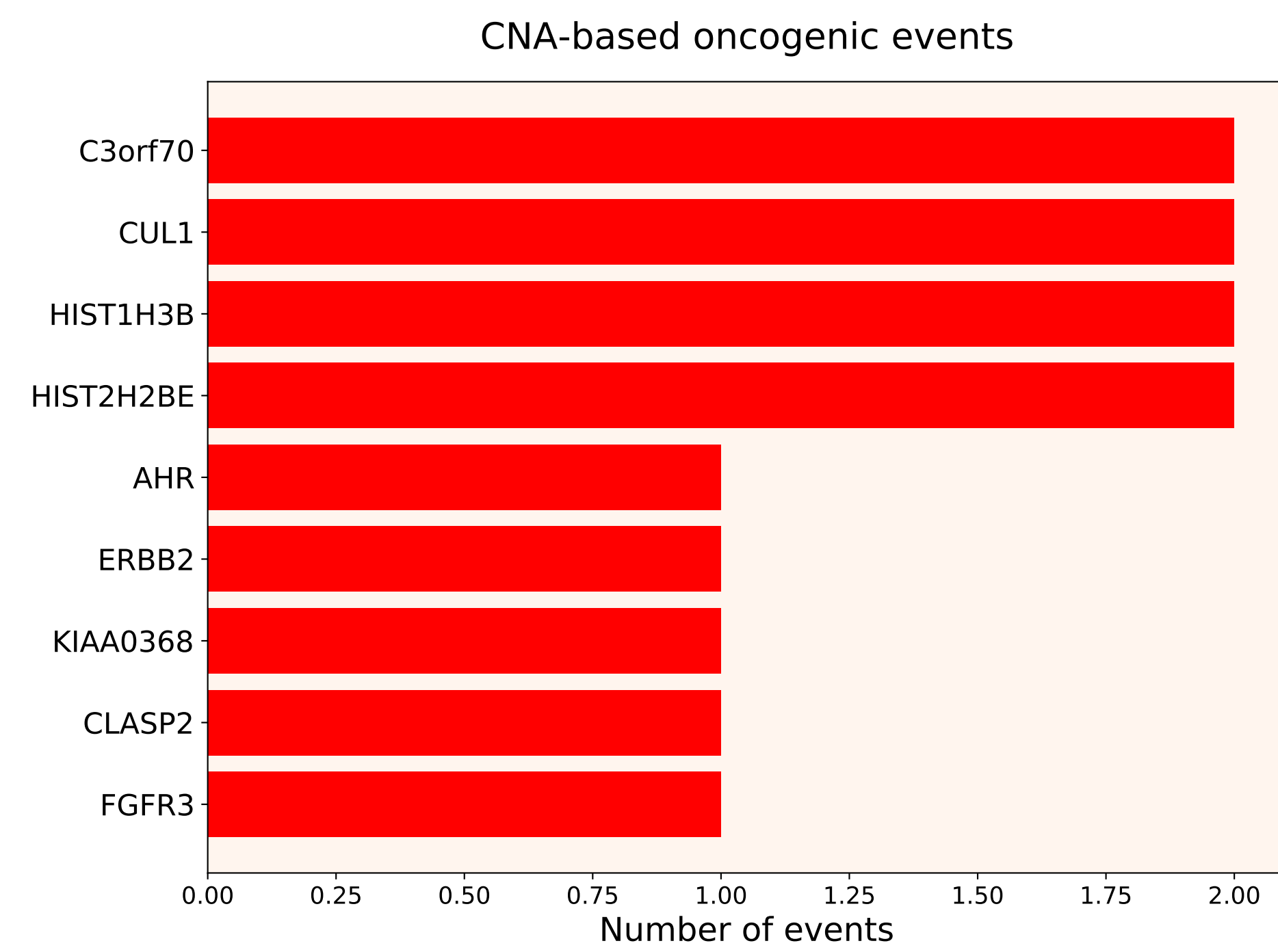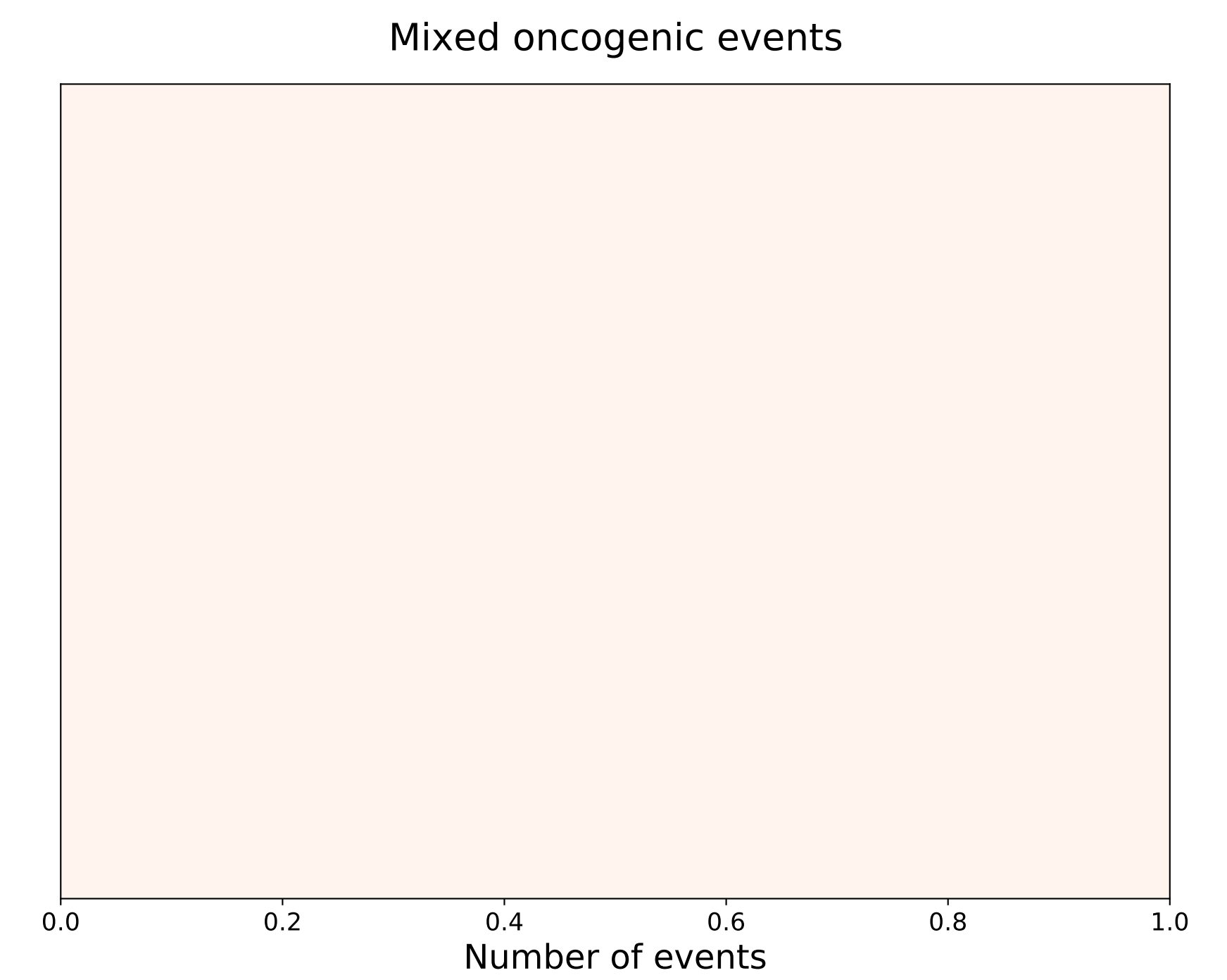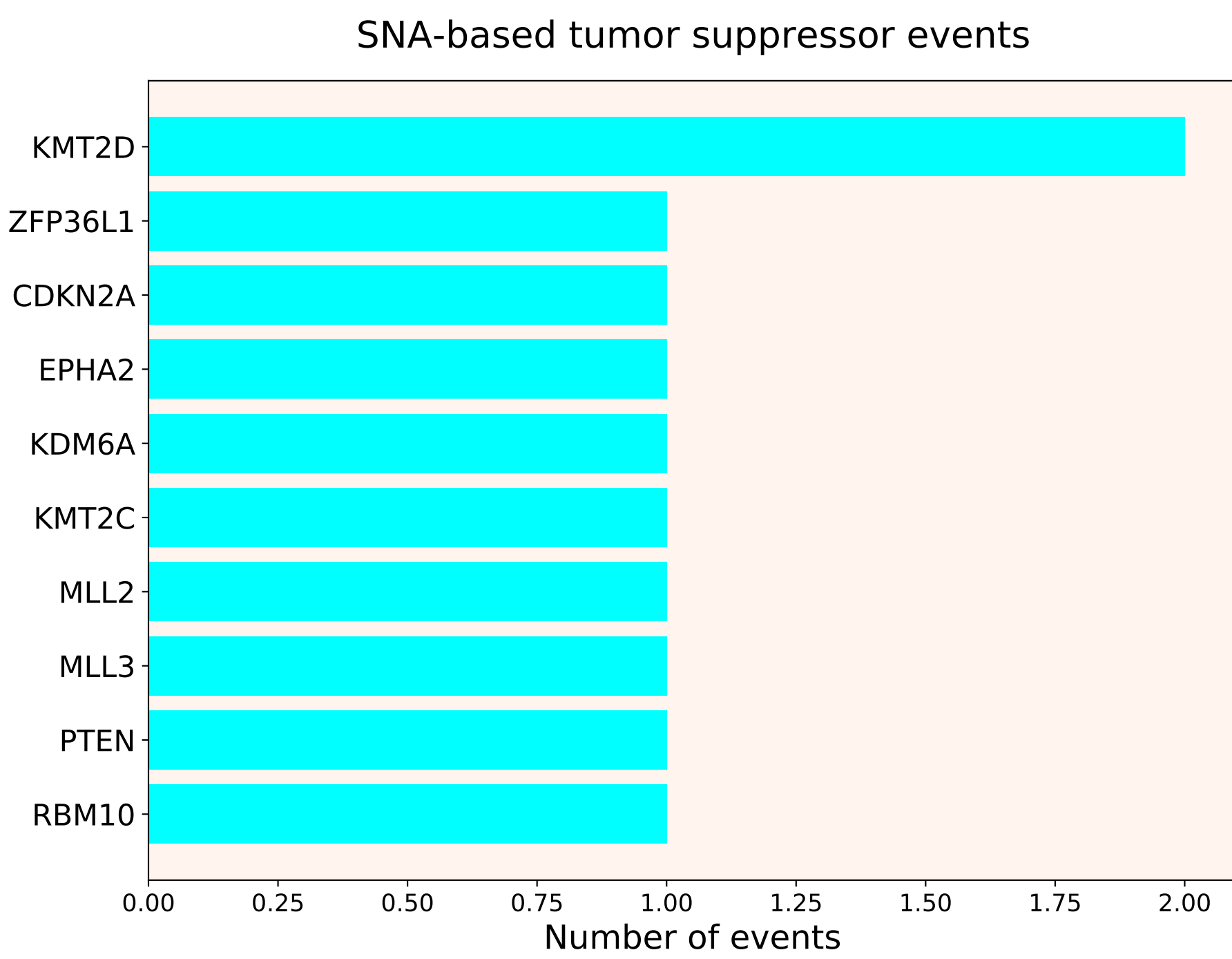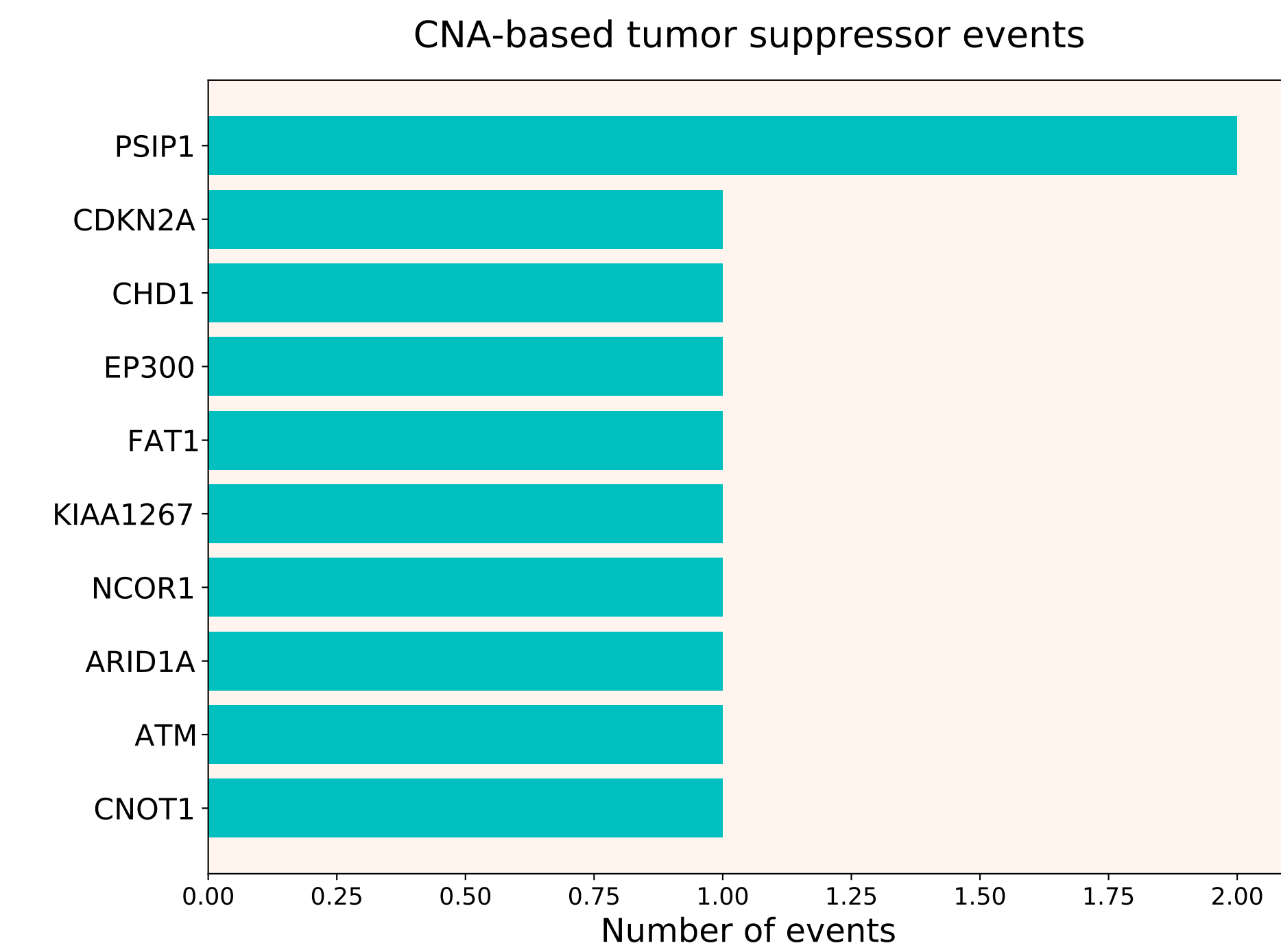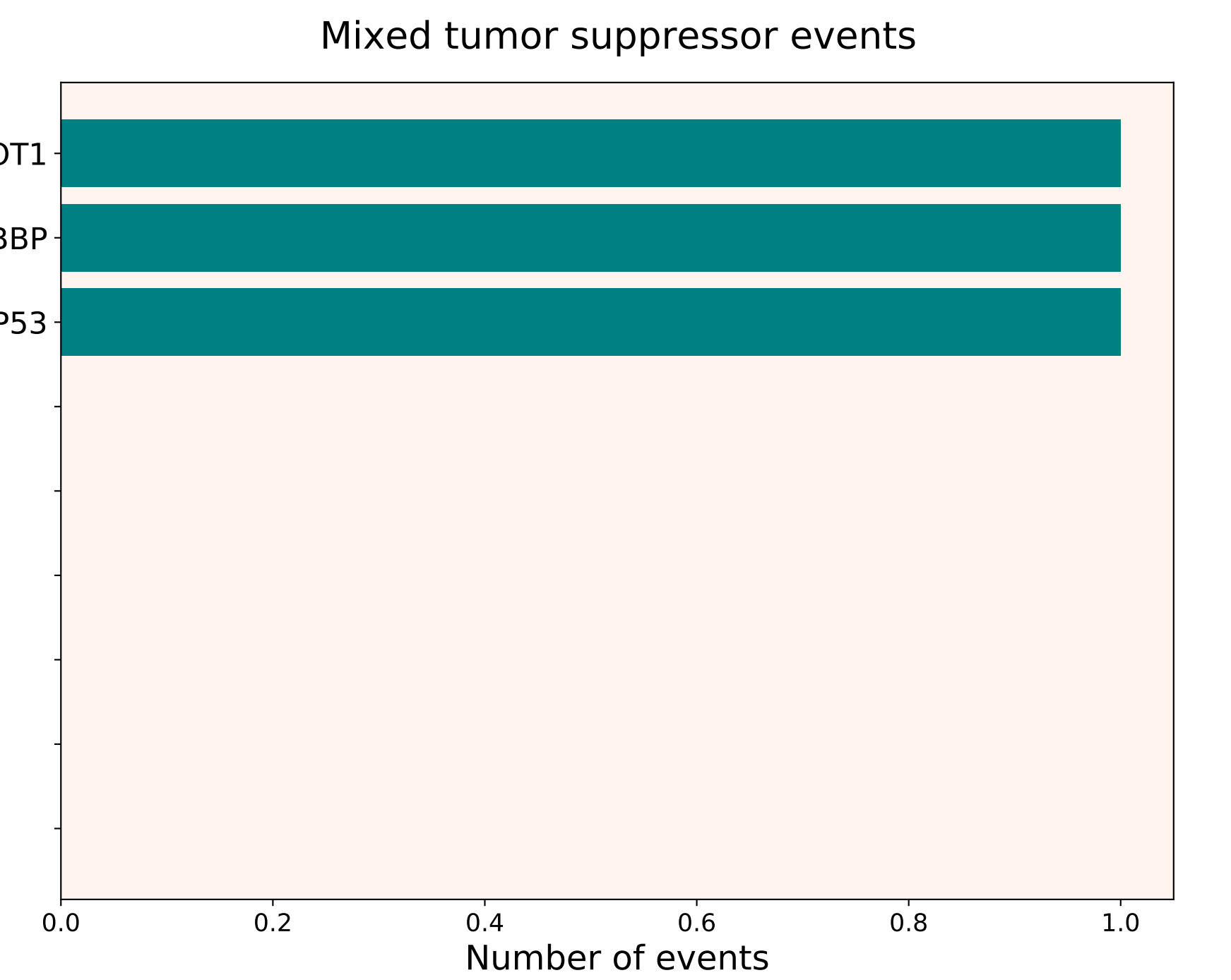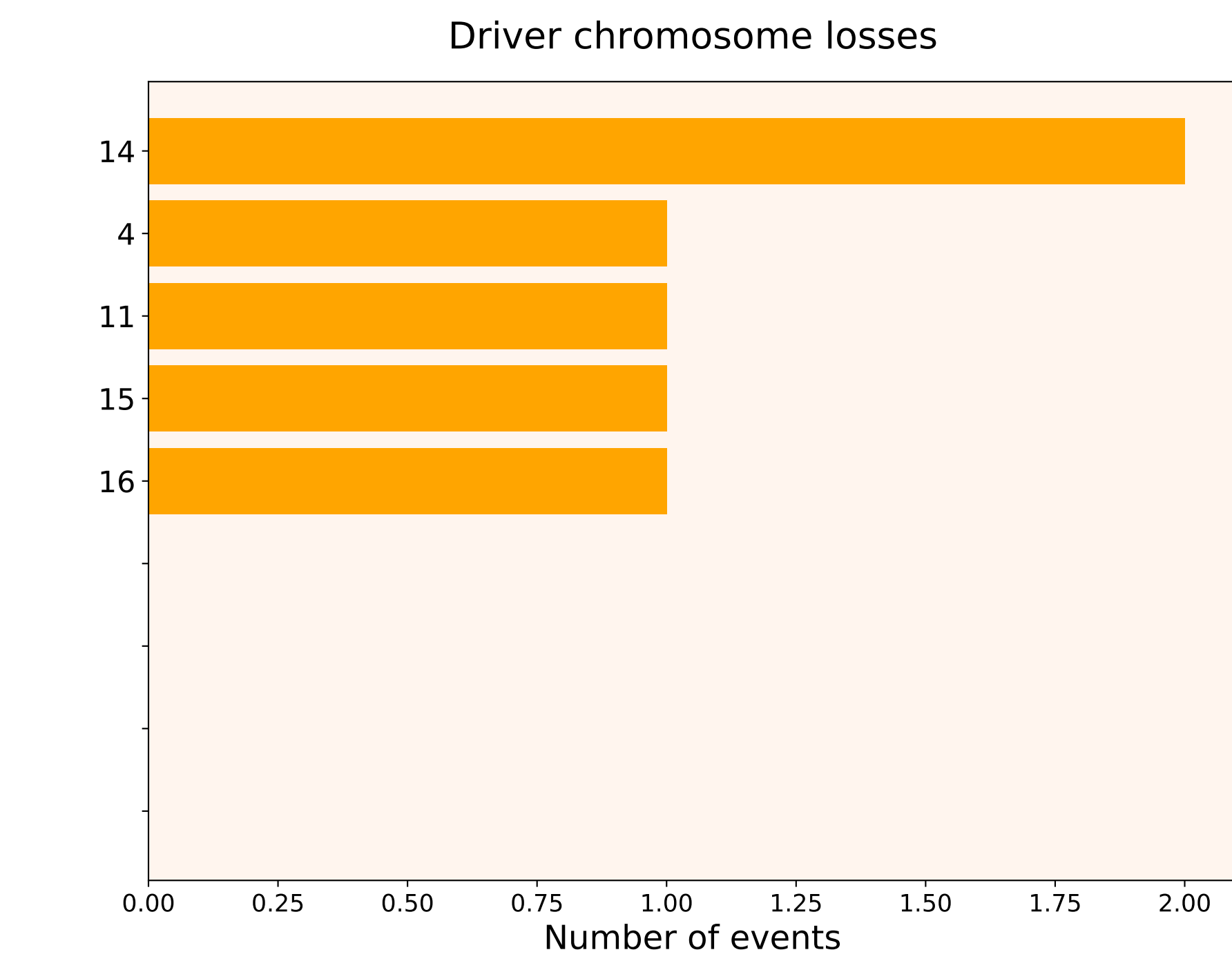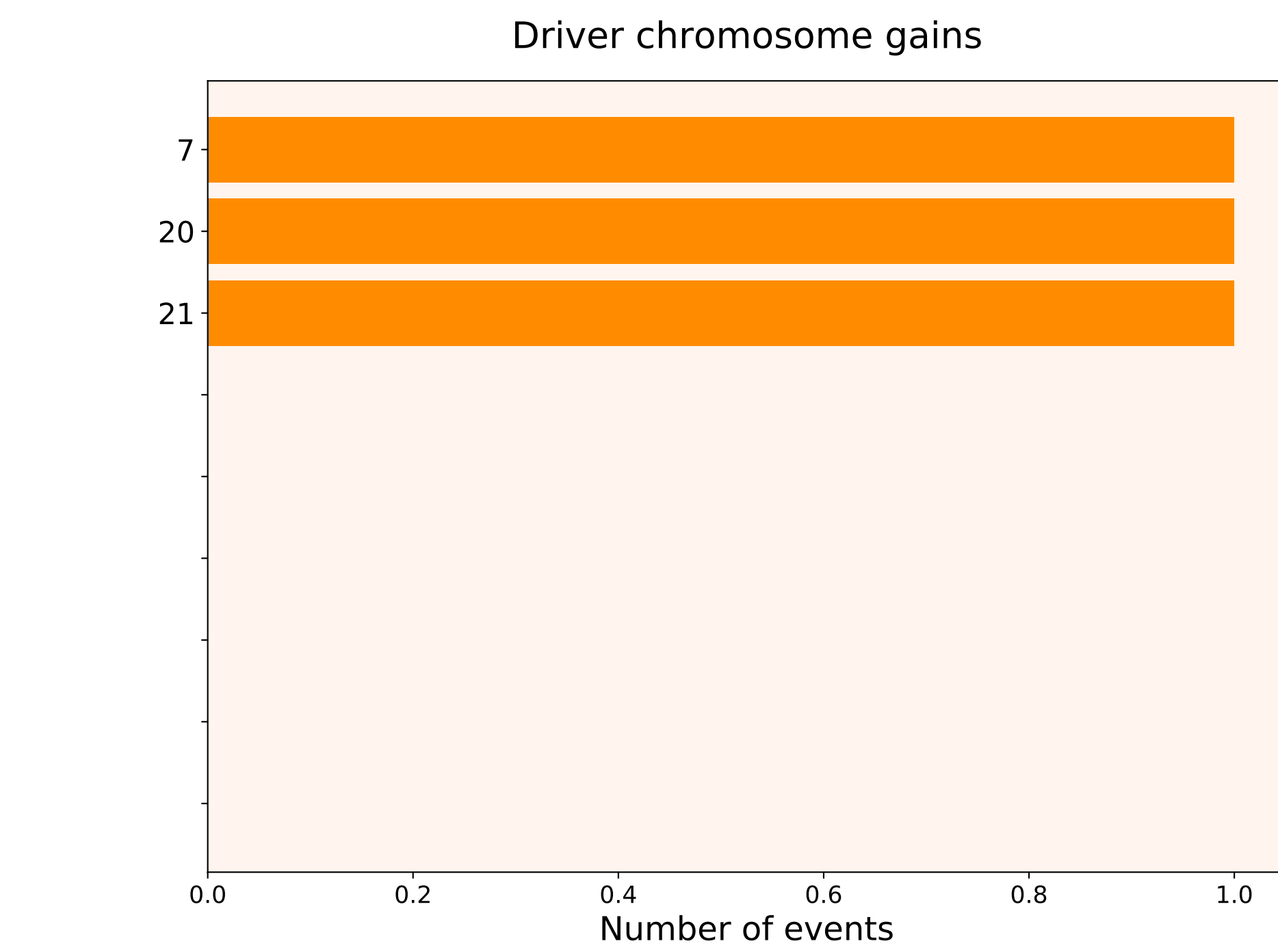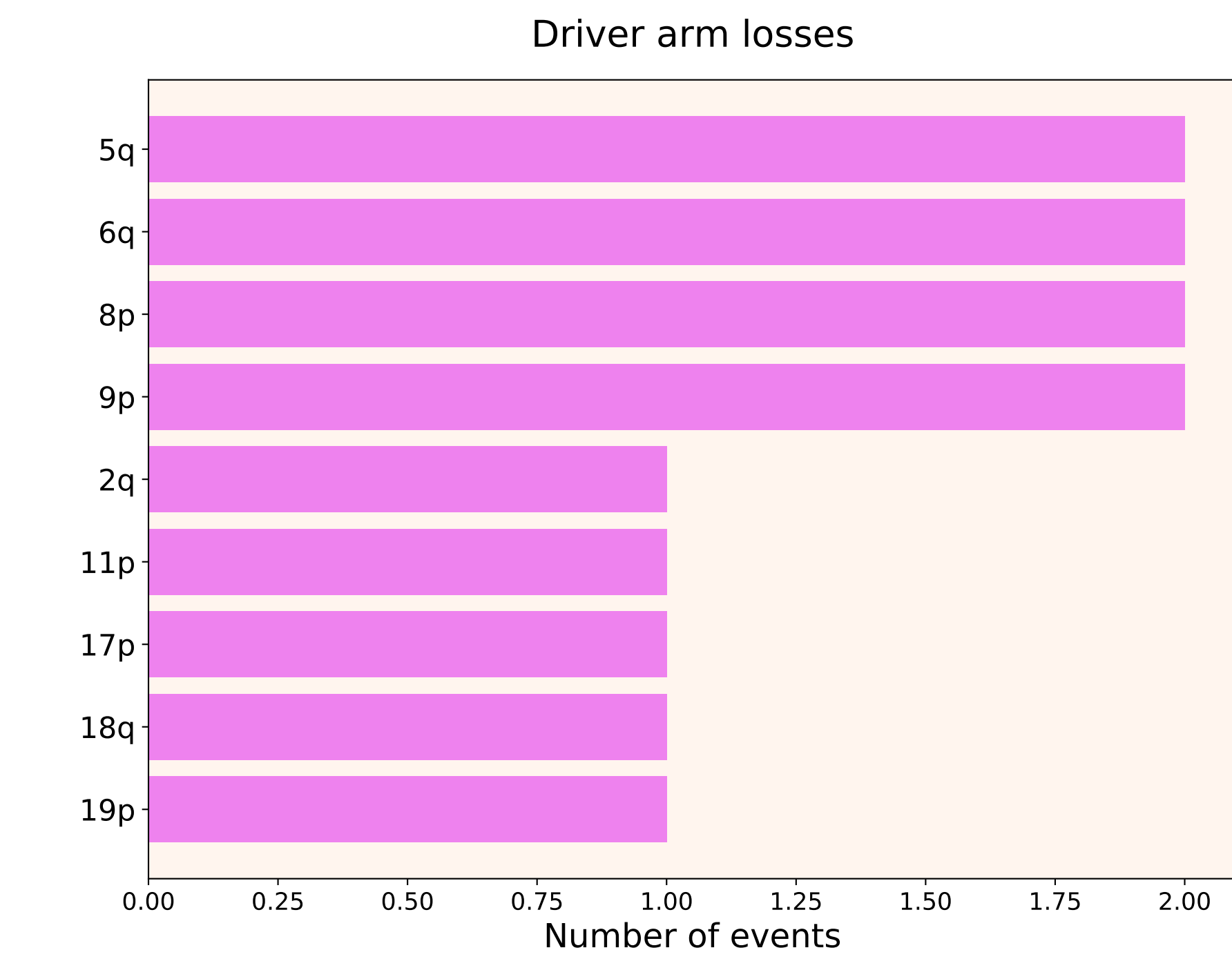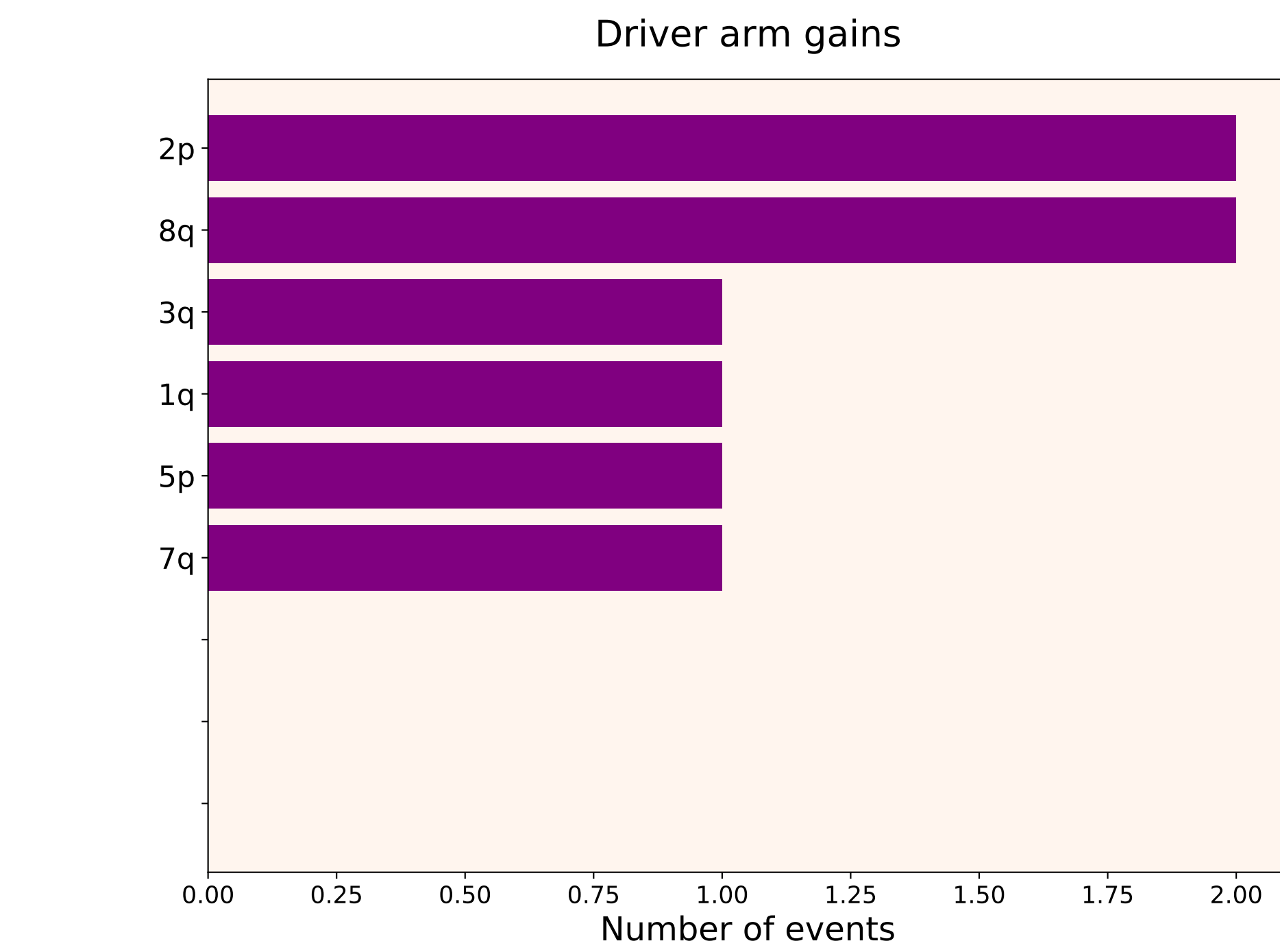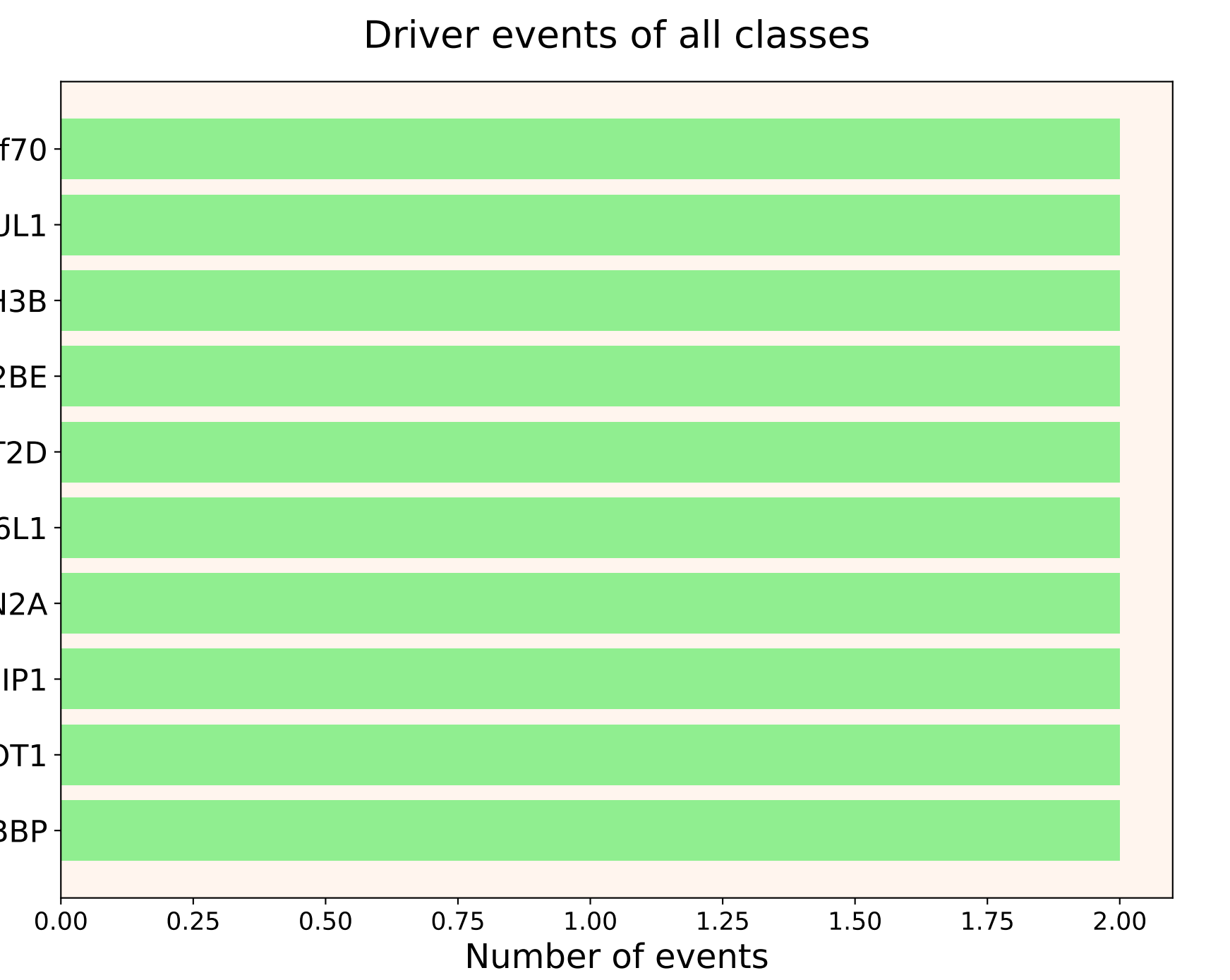

Supplement: Supplemental Information 2 [file peerj-10-13860-s002.zip › COHORTS/genes plots/detailed/2021_8_16_14_9_distribution_events_detailed_39.pdf]

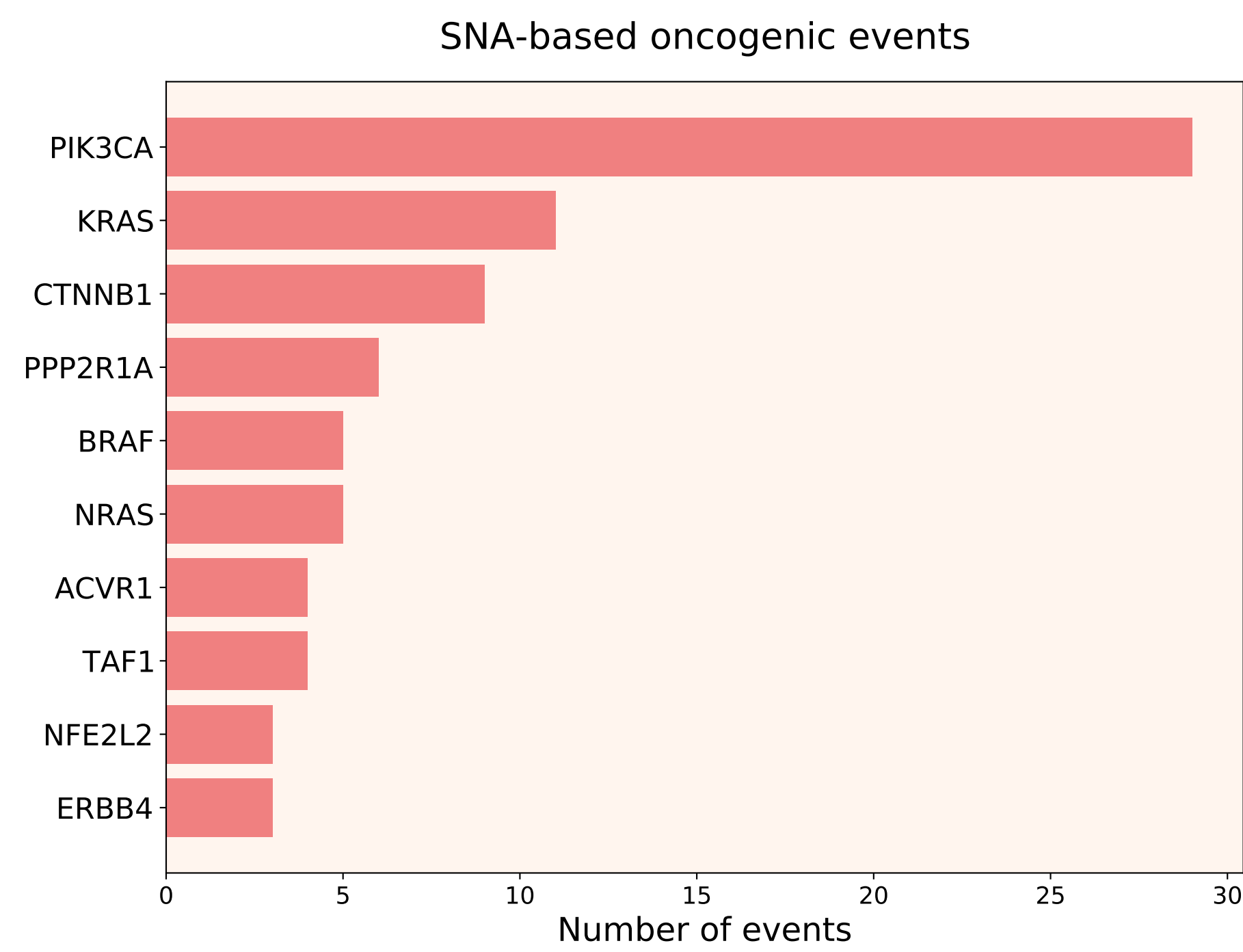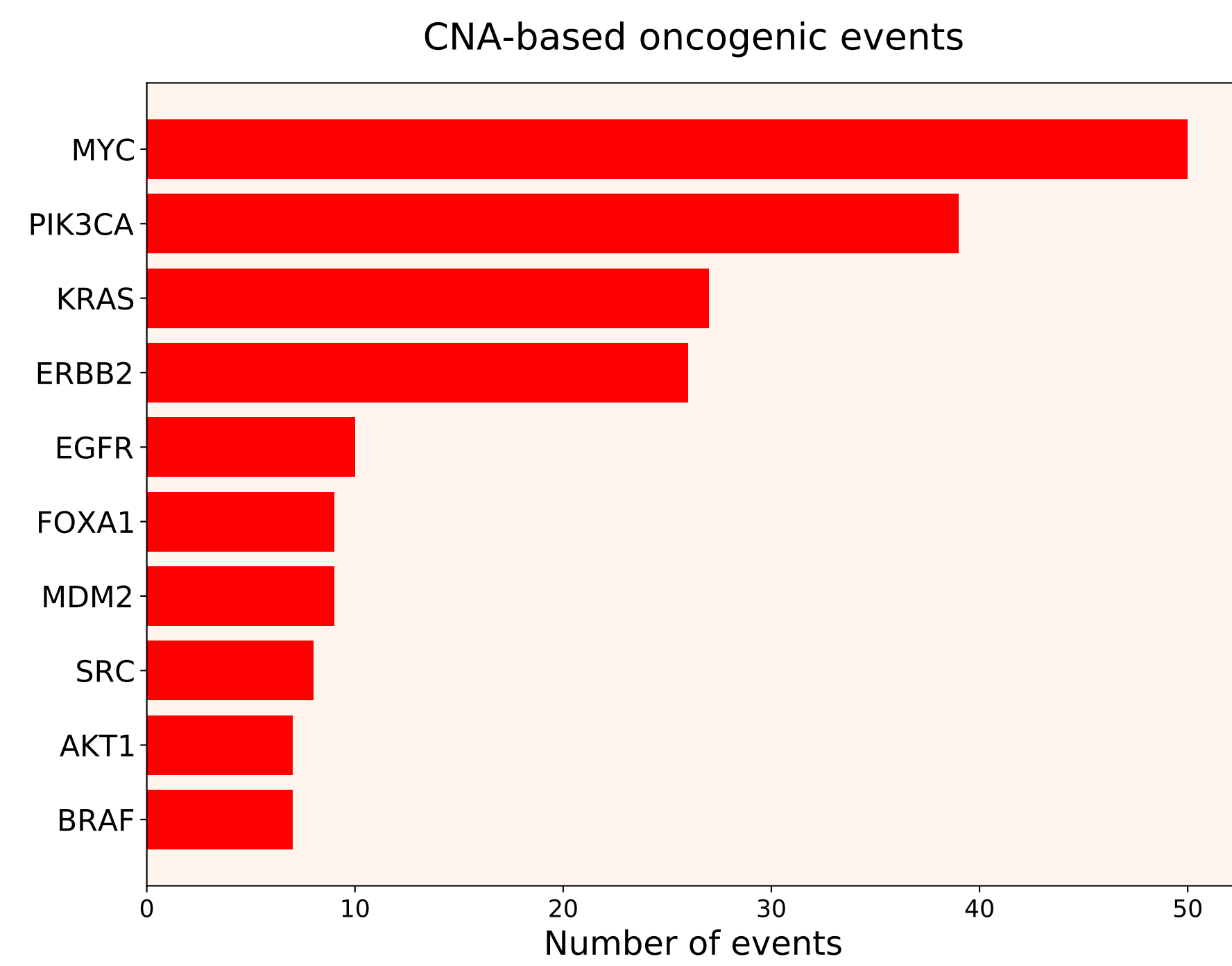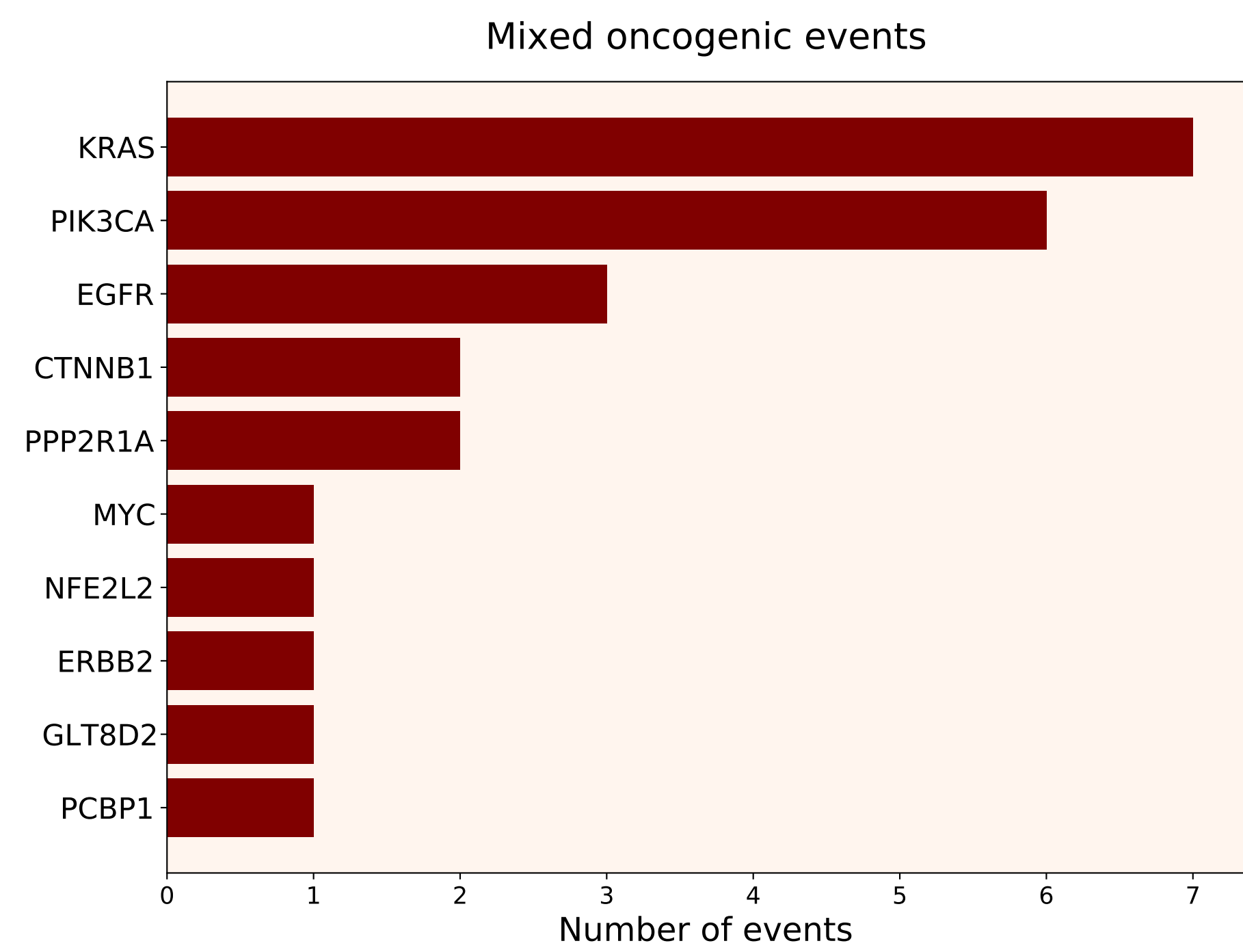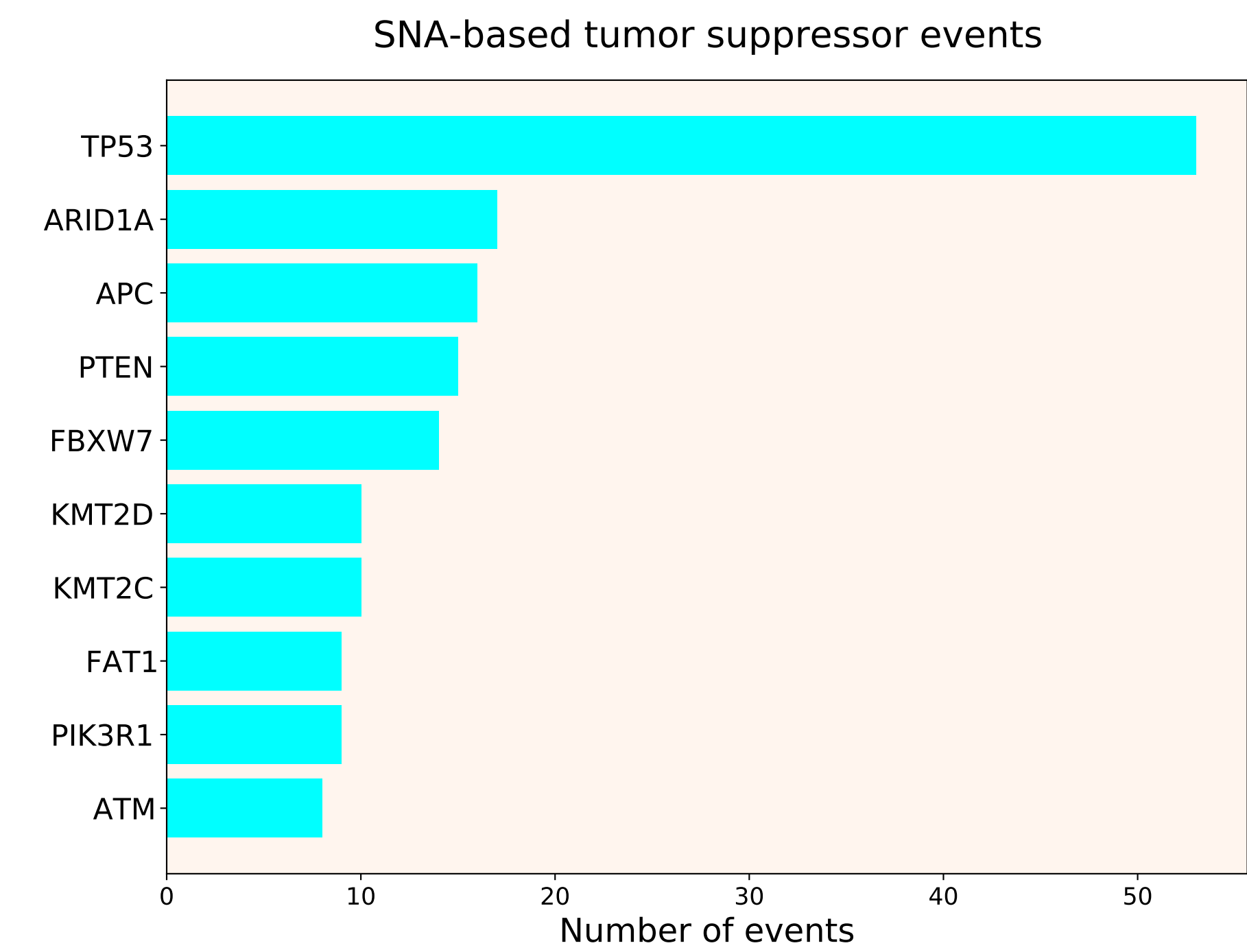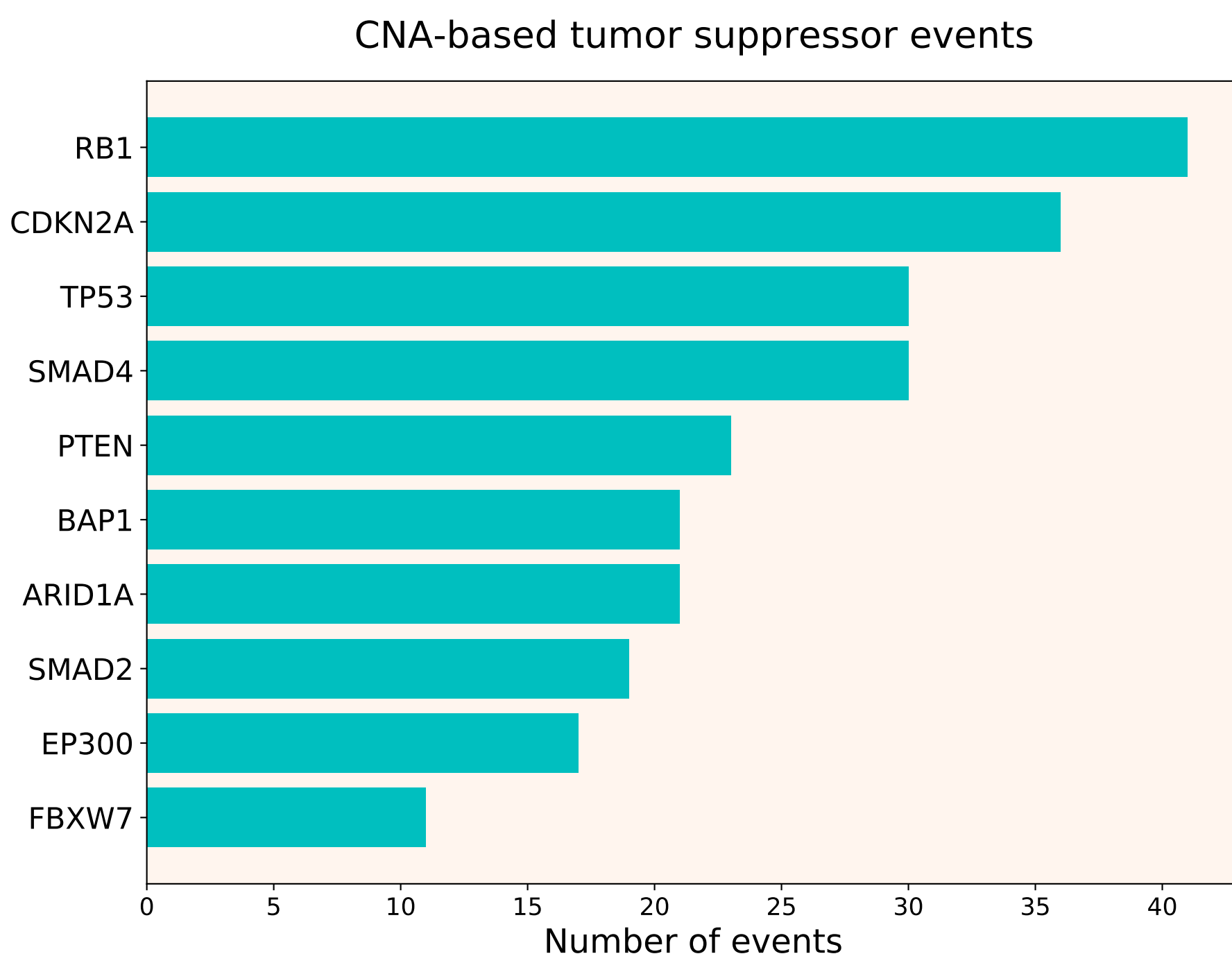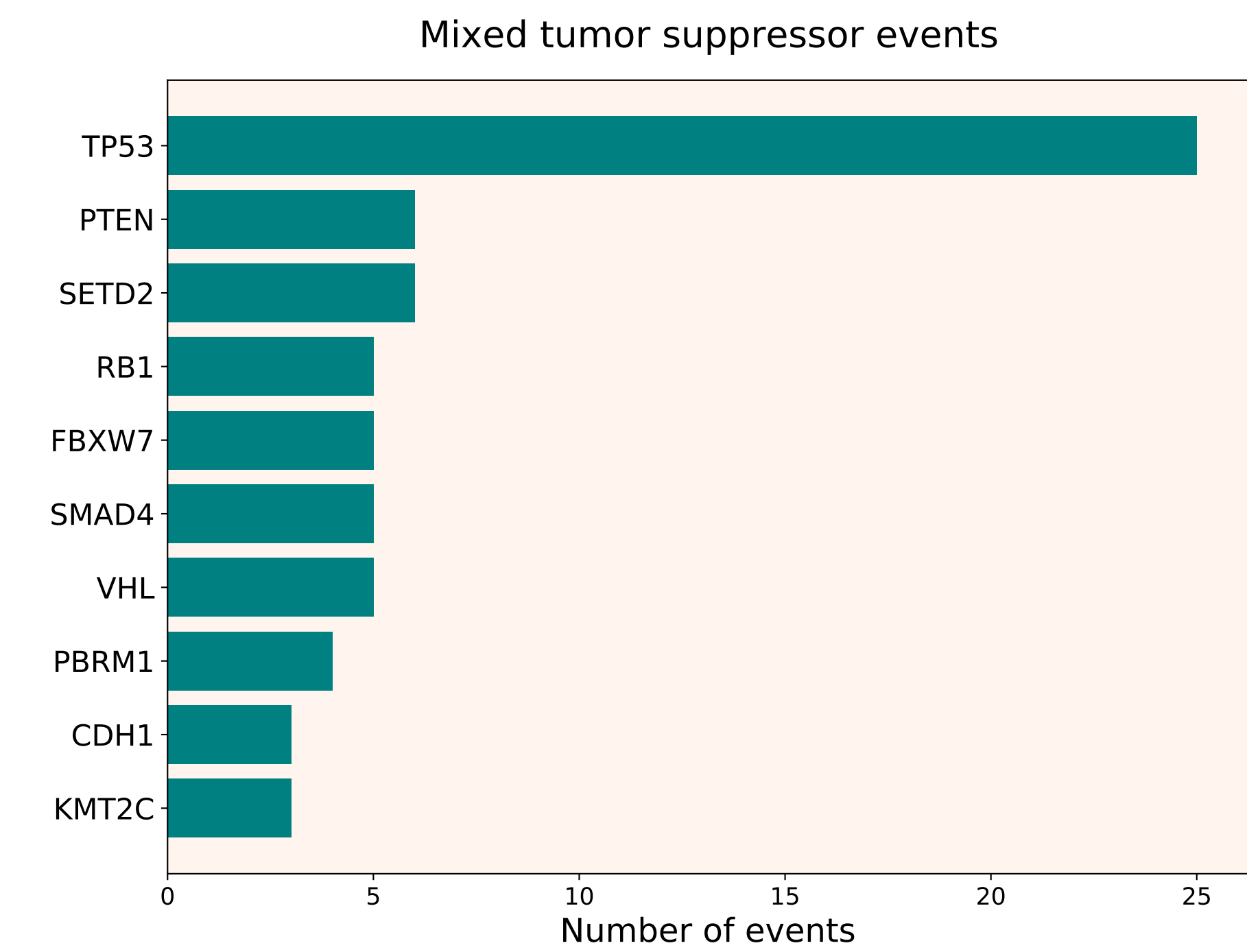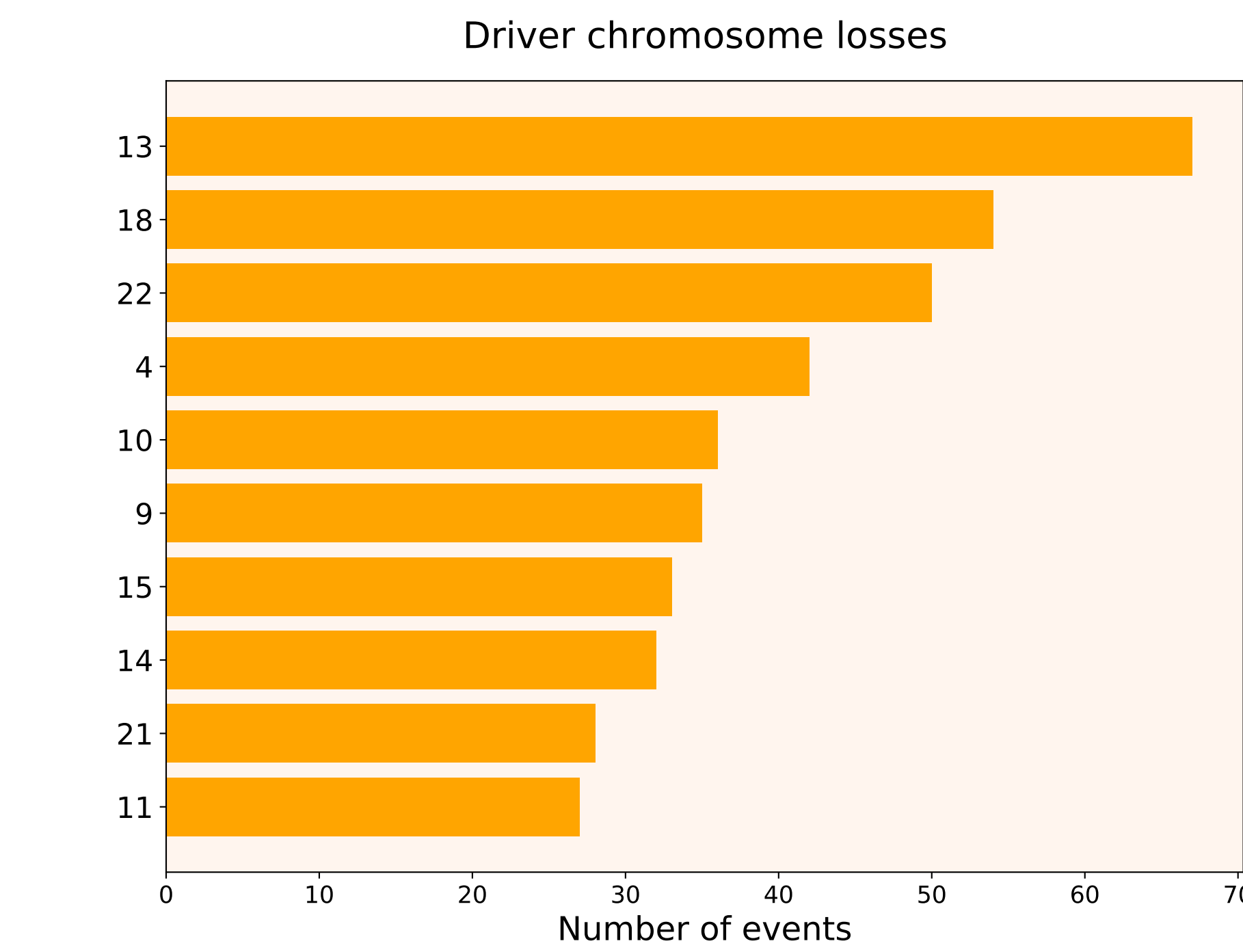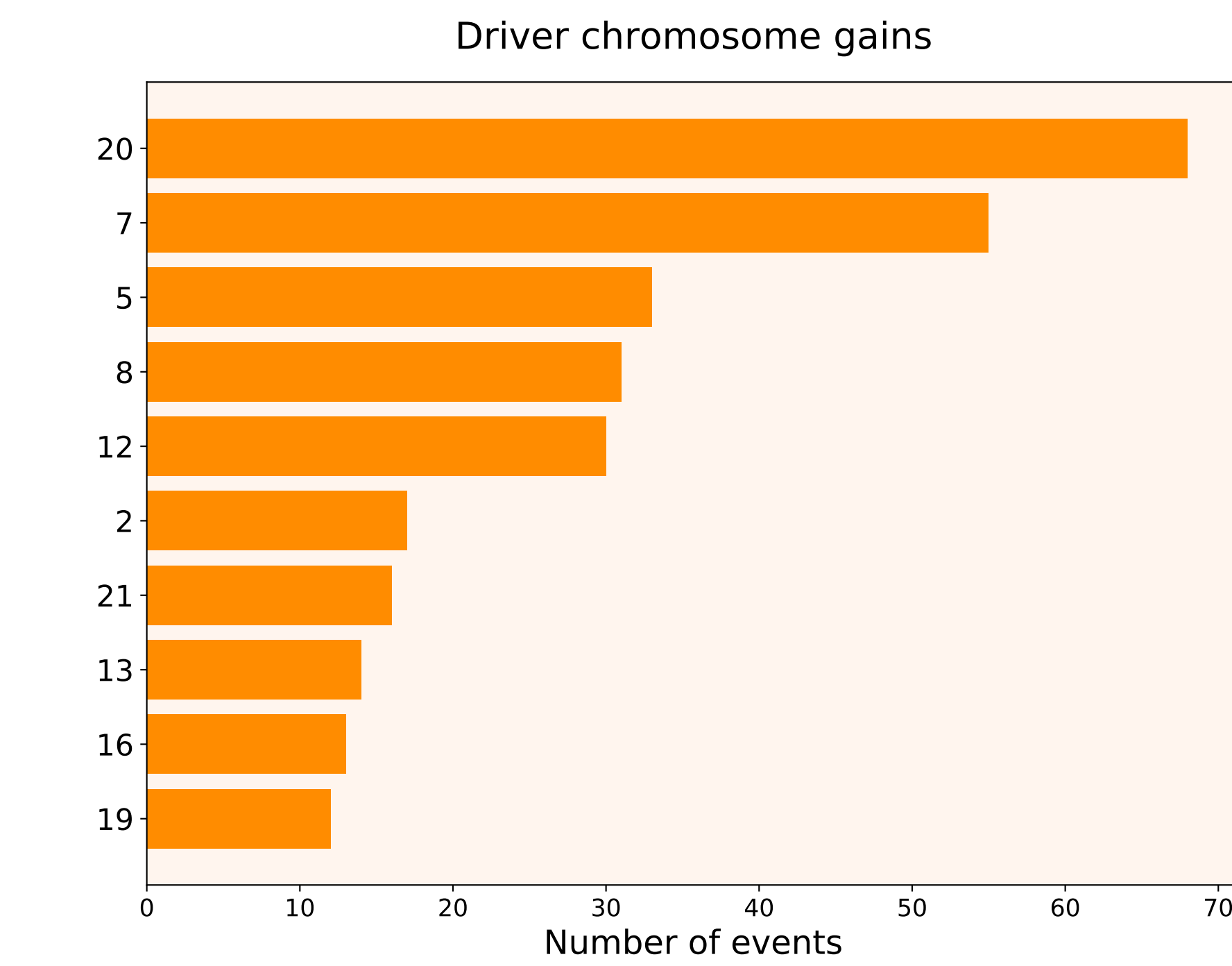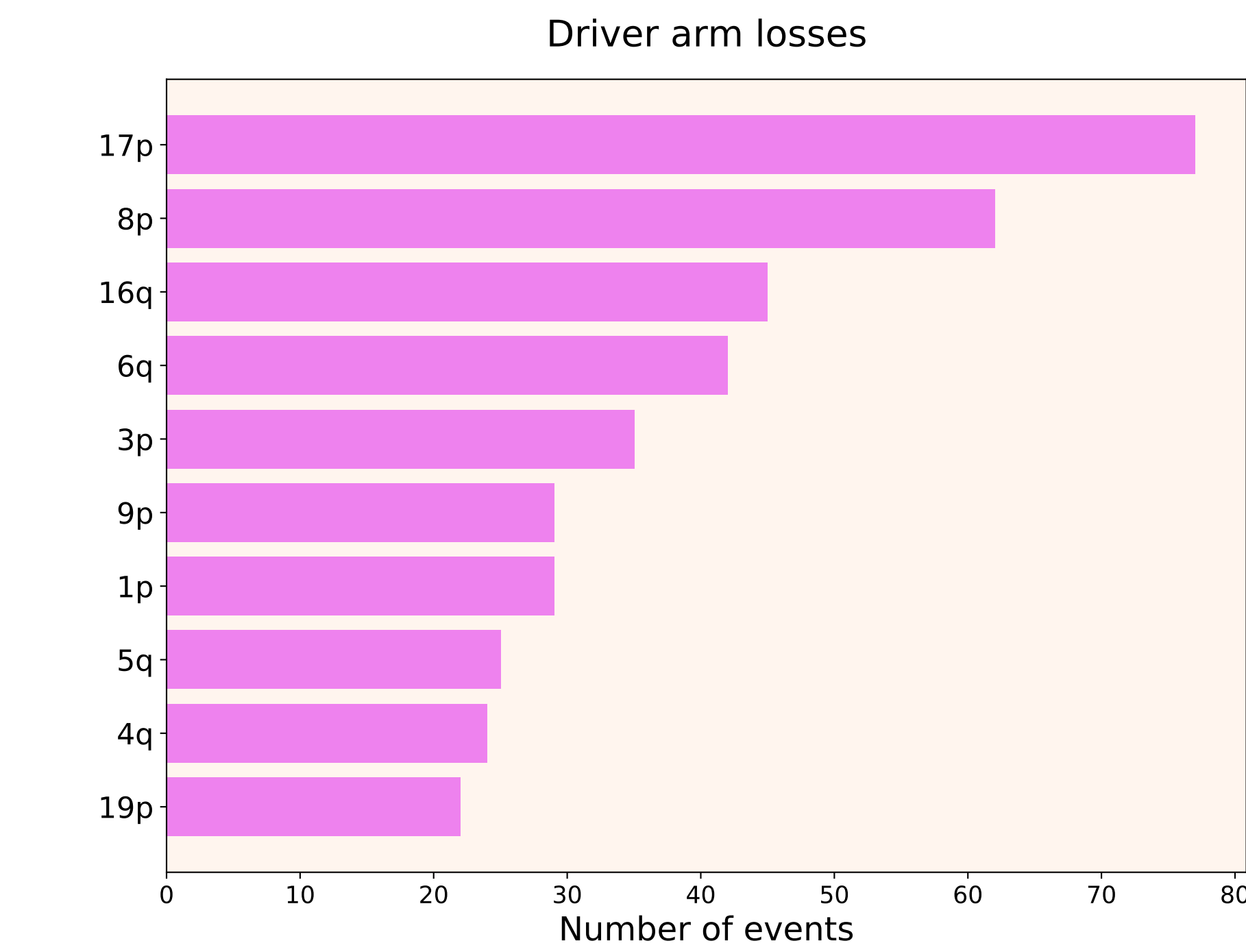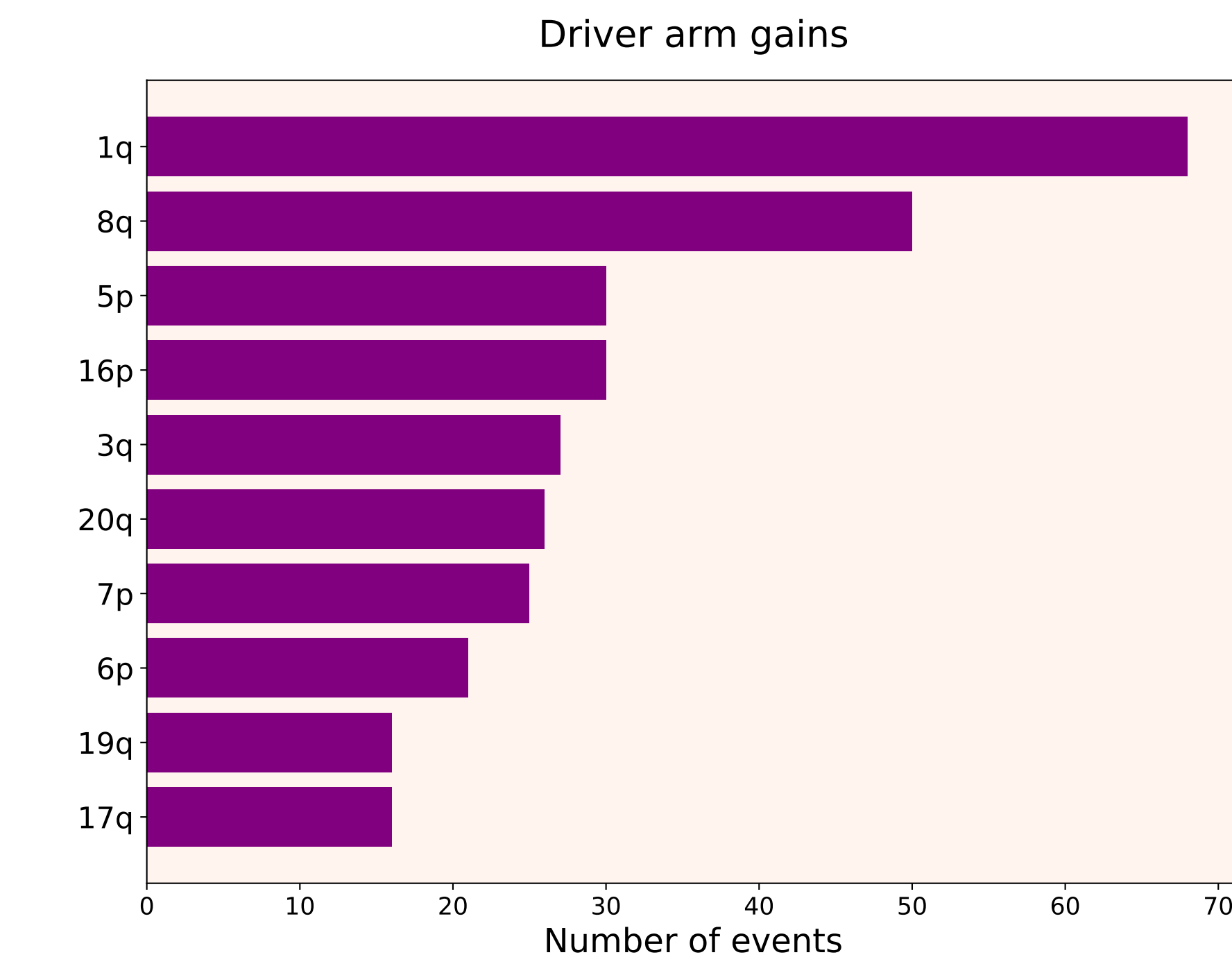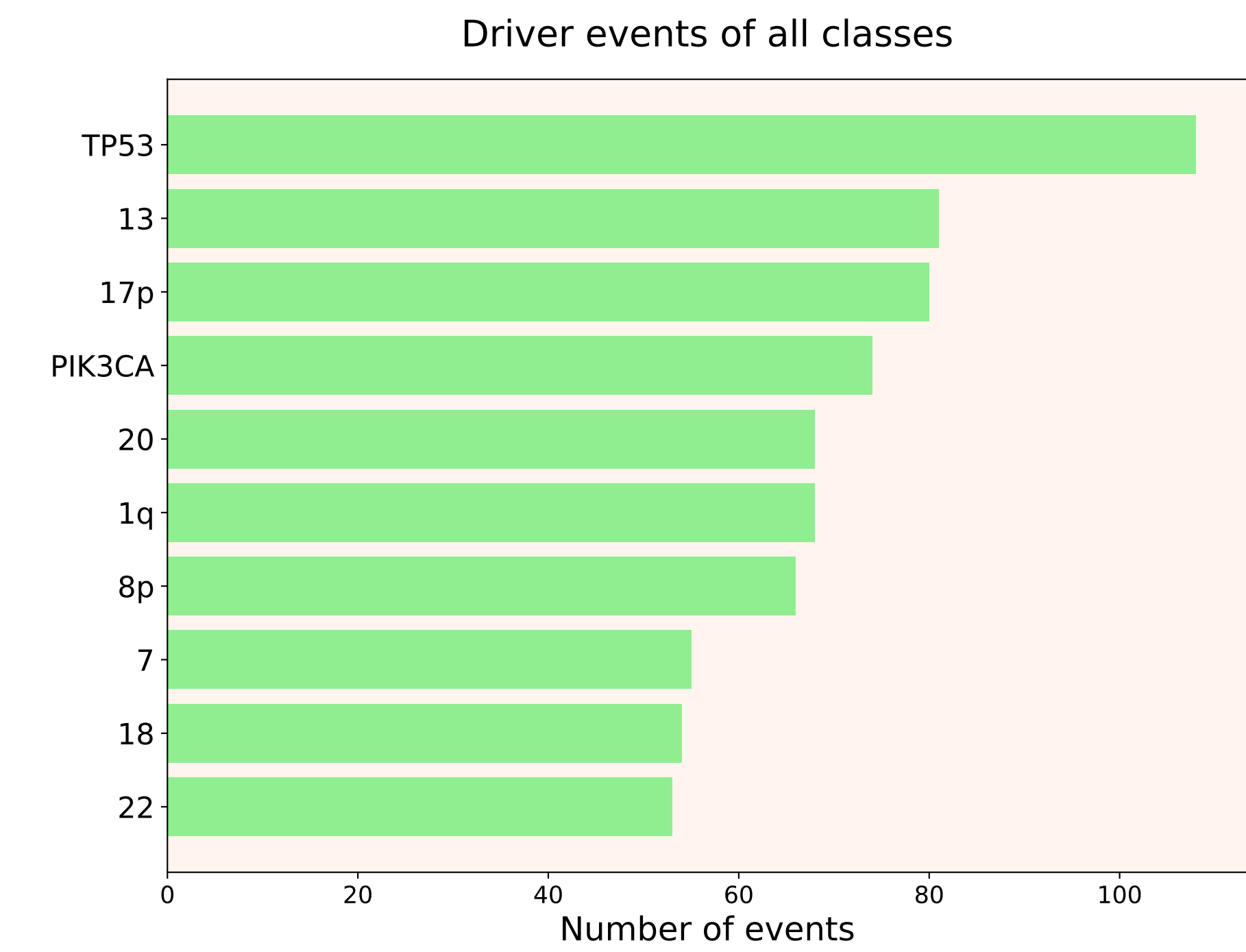

Supplement: Supplemental Information 2 [file peerj-10-13860-s002.zip › COHORTS/genes plots/detailed/2021_8_16_14_9_distribution_events_detailed_15.pdf]

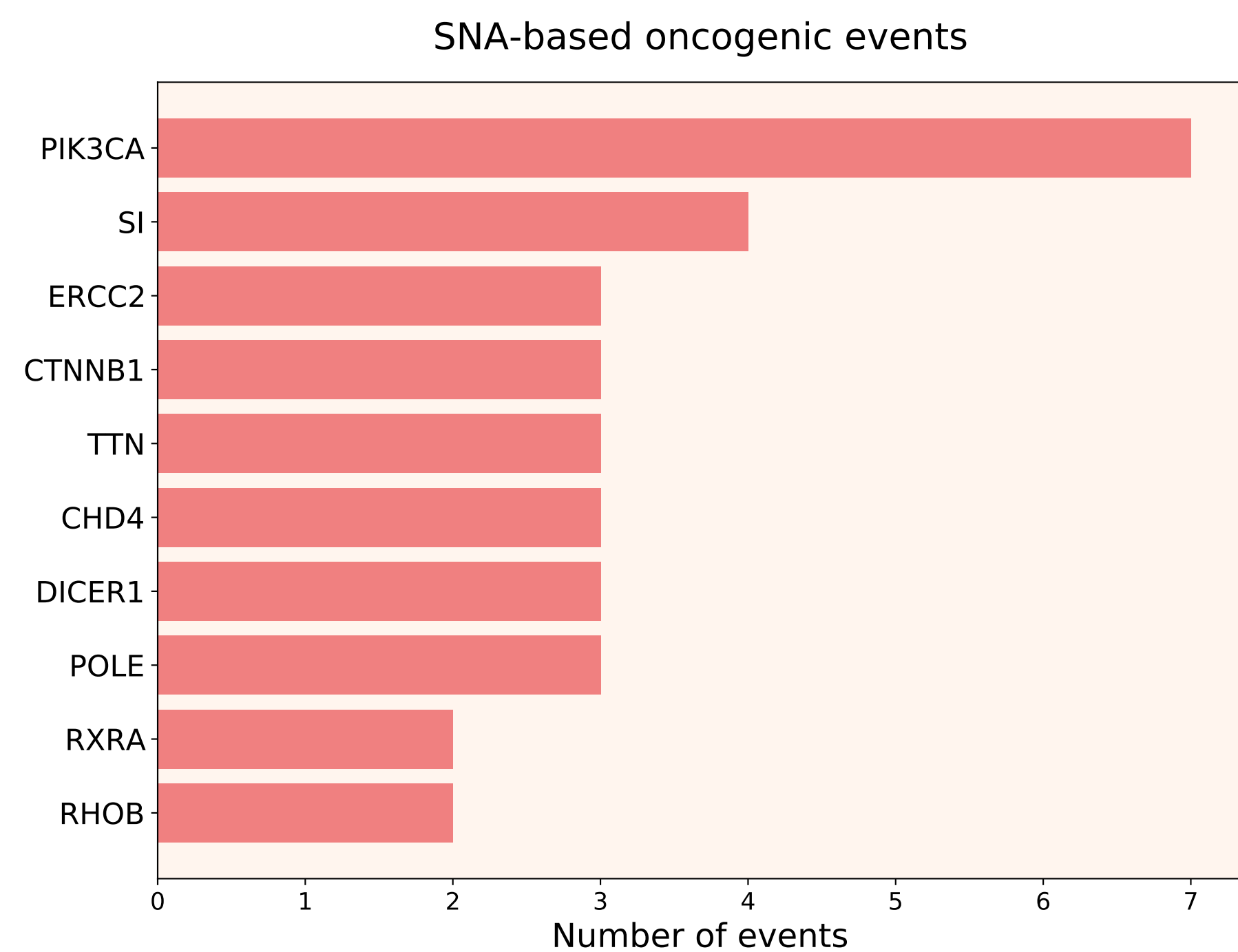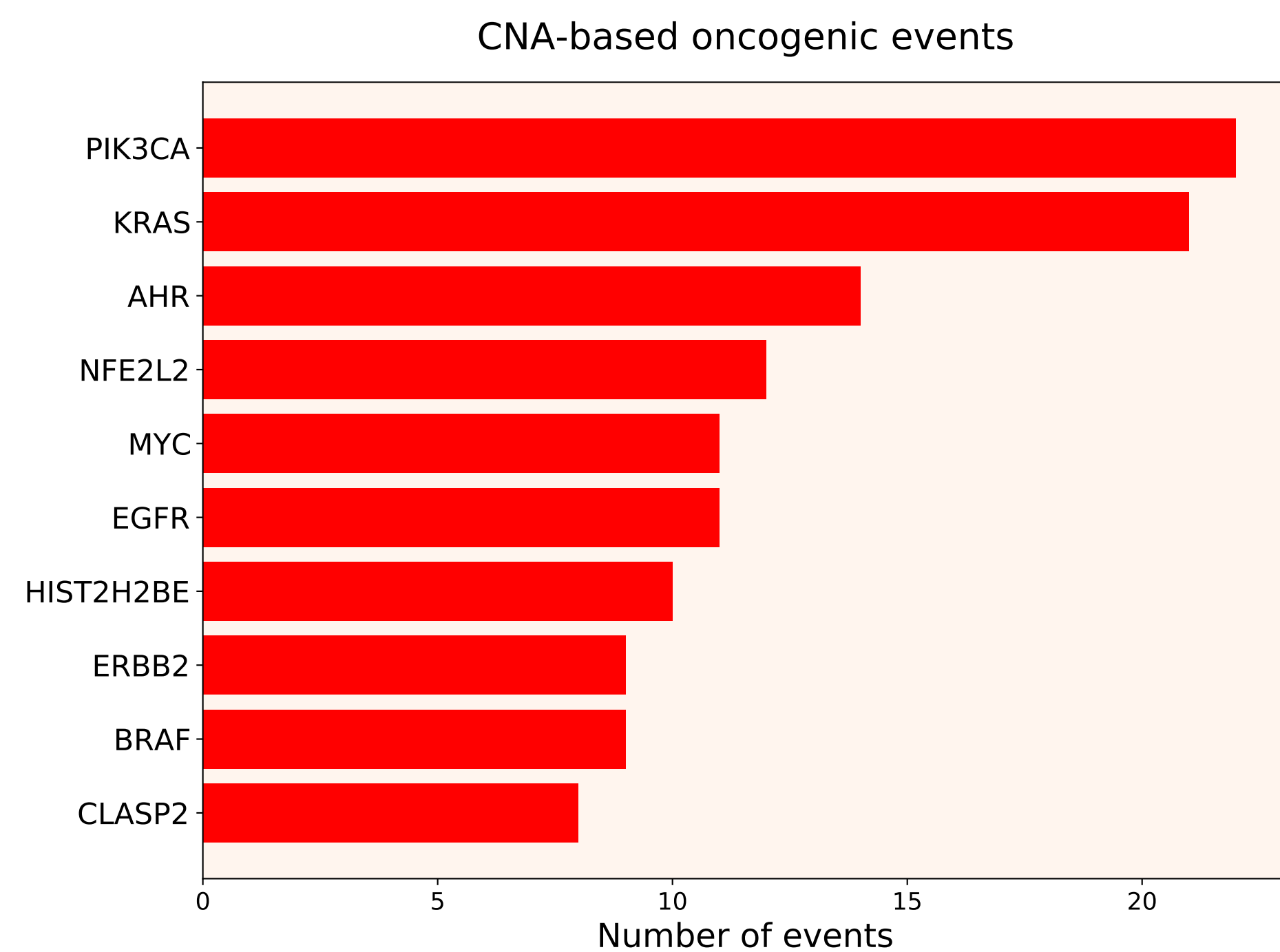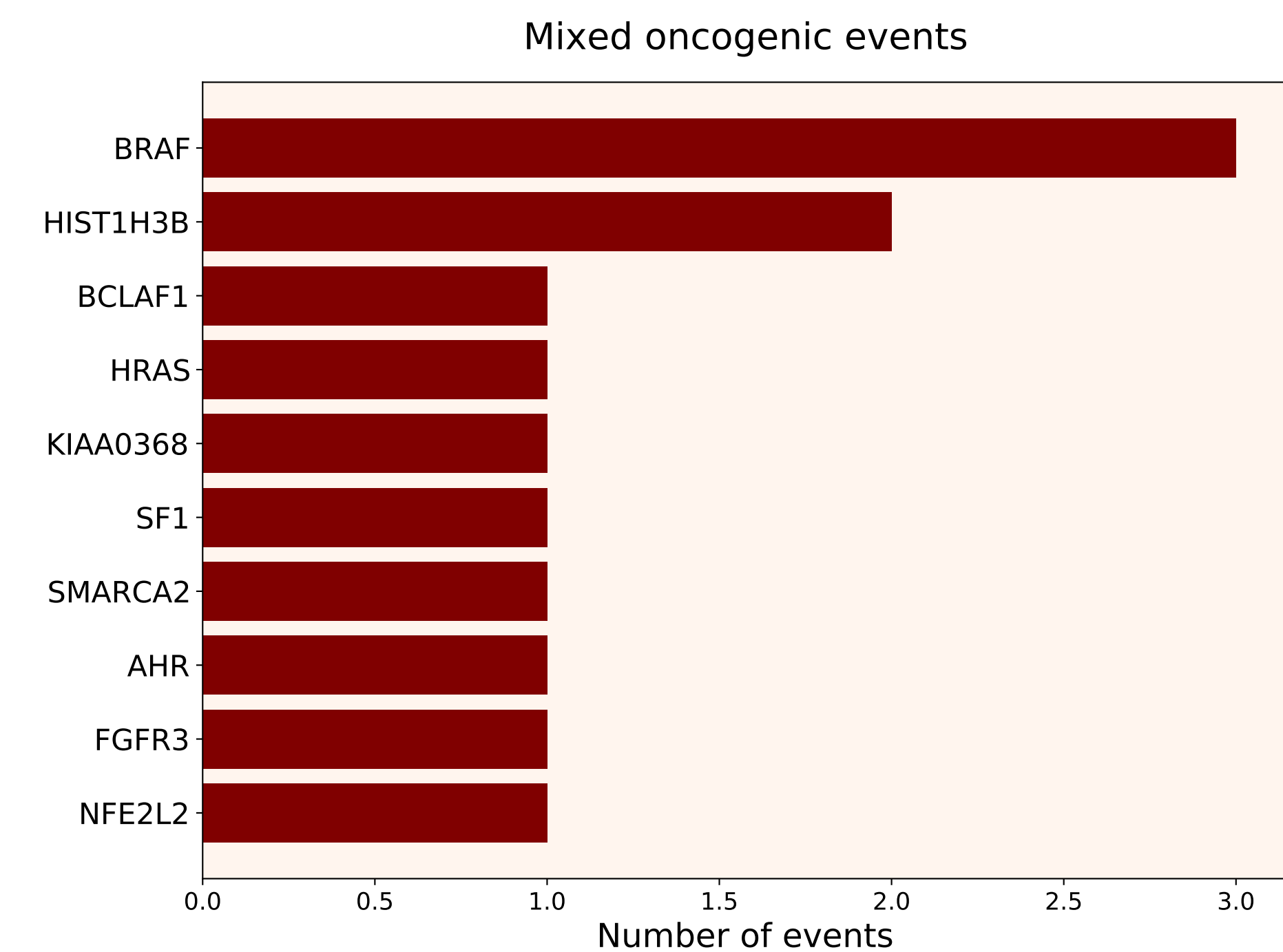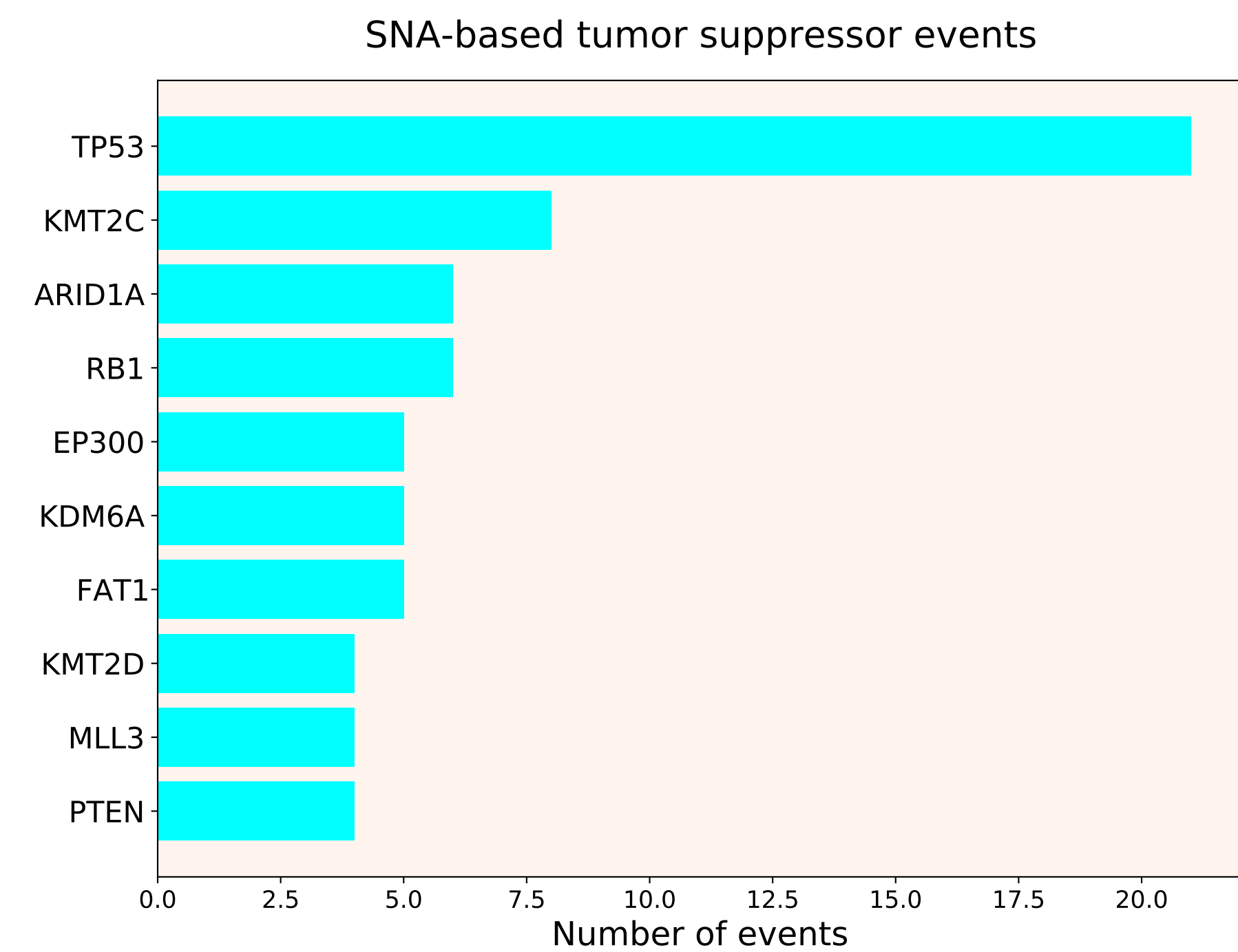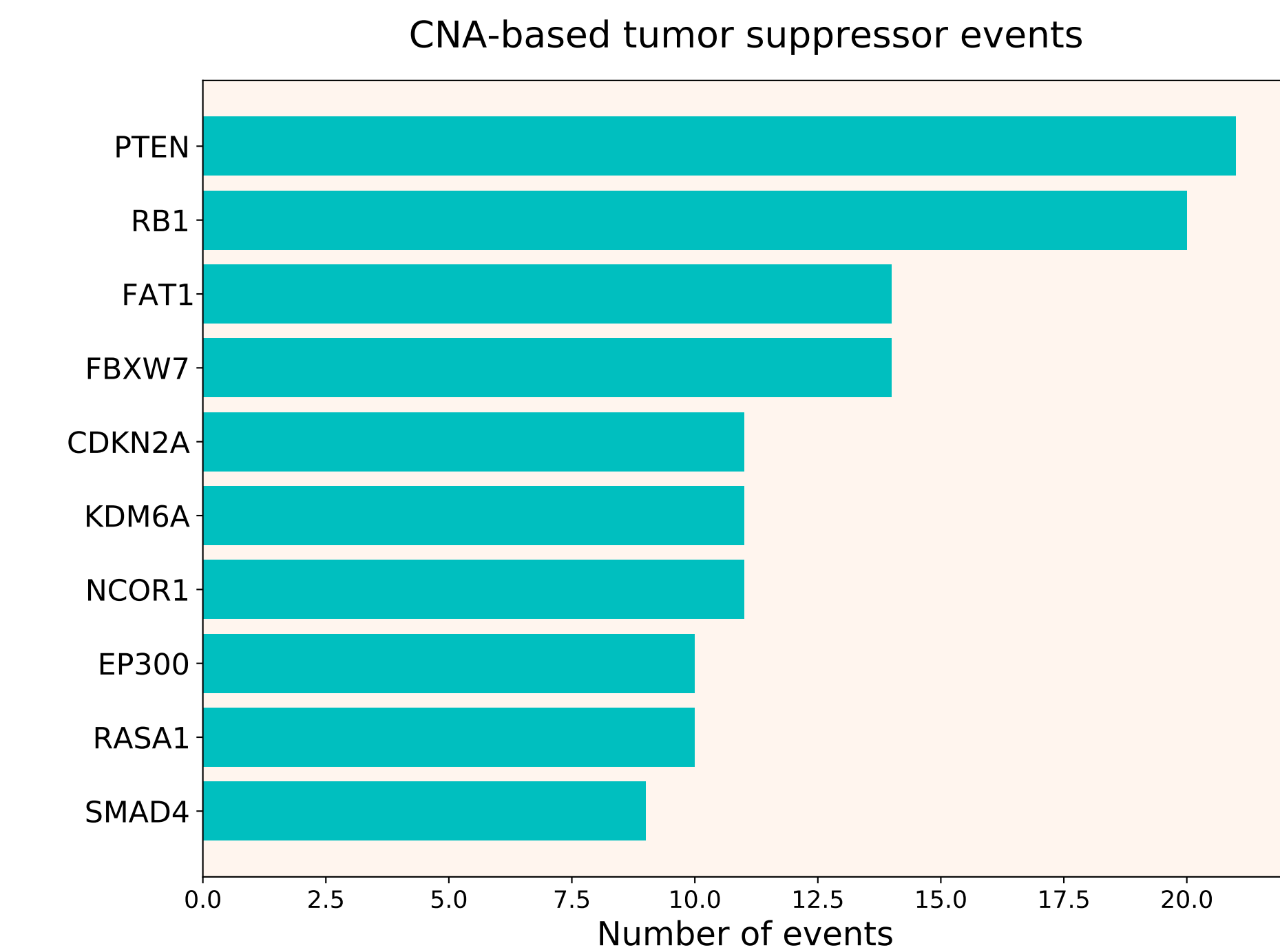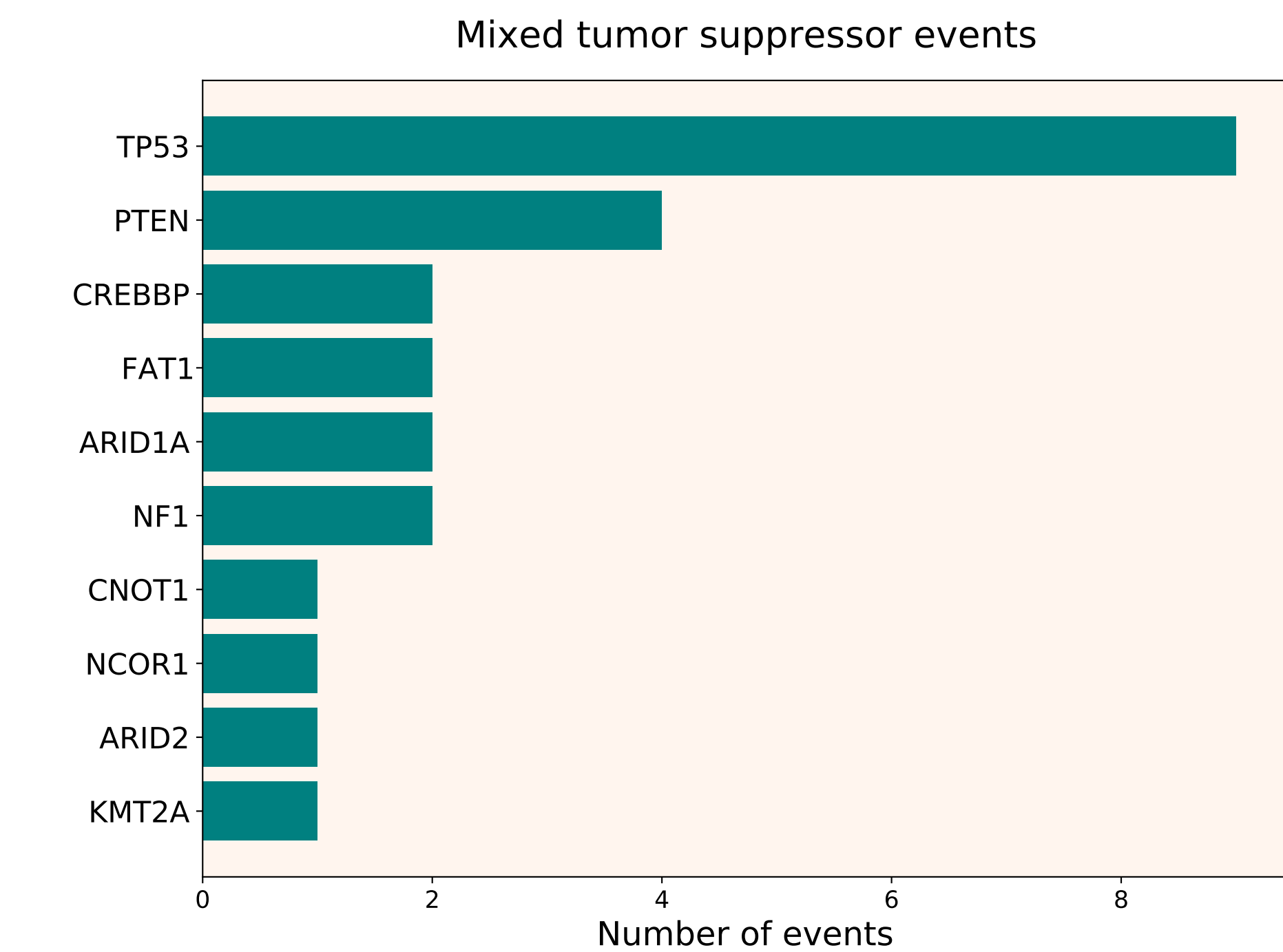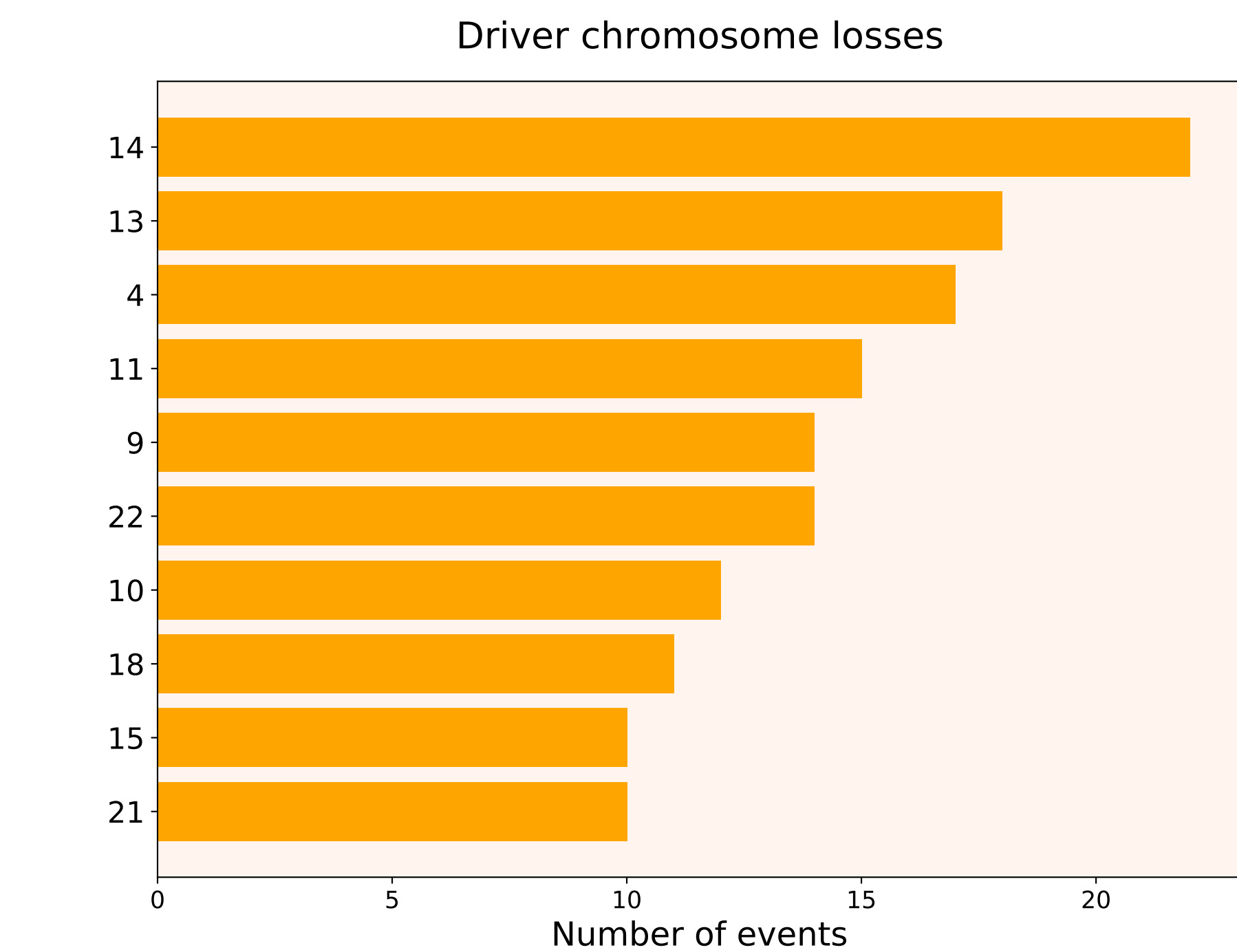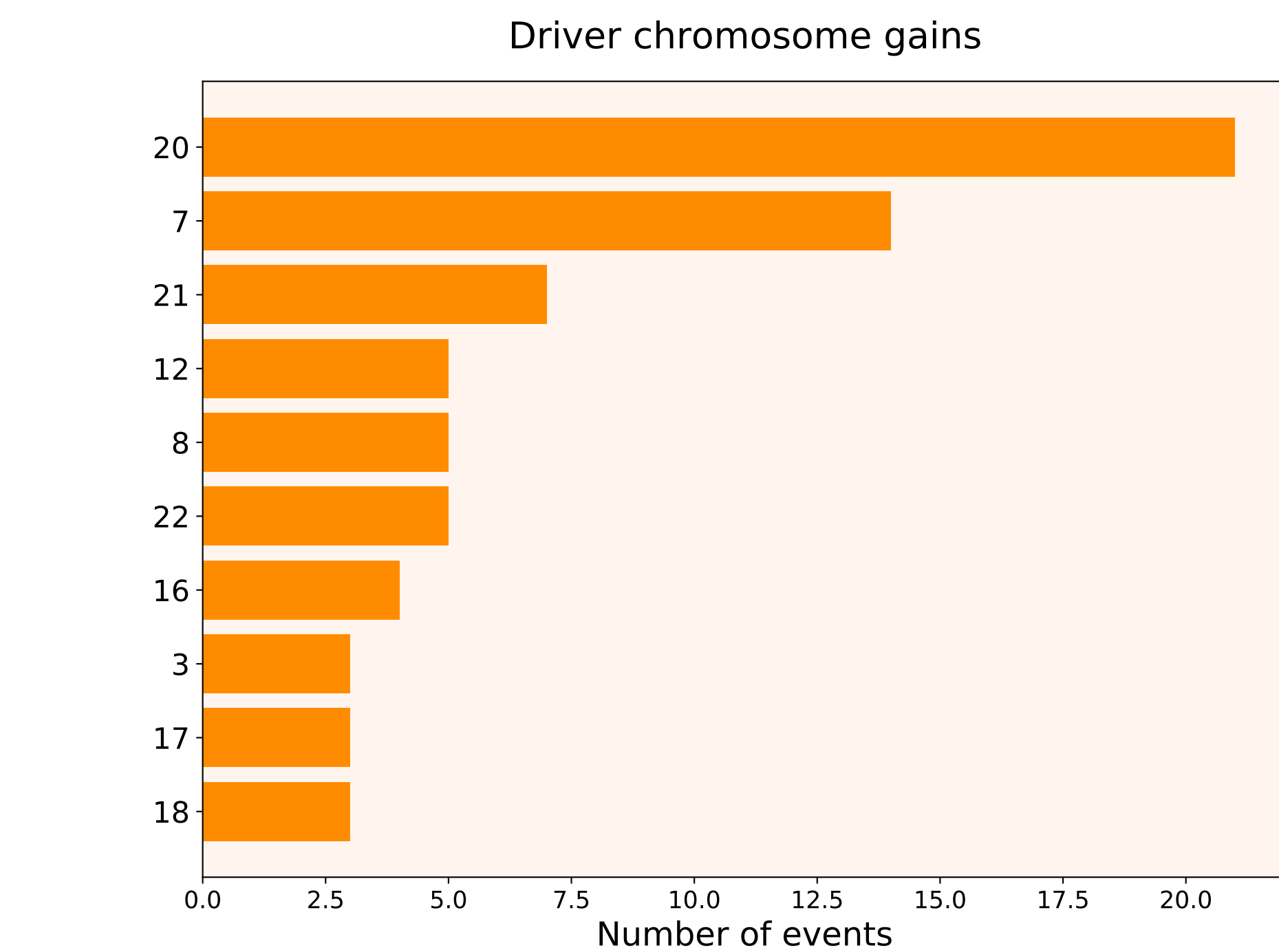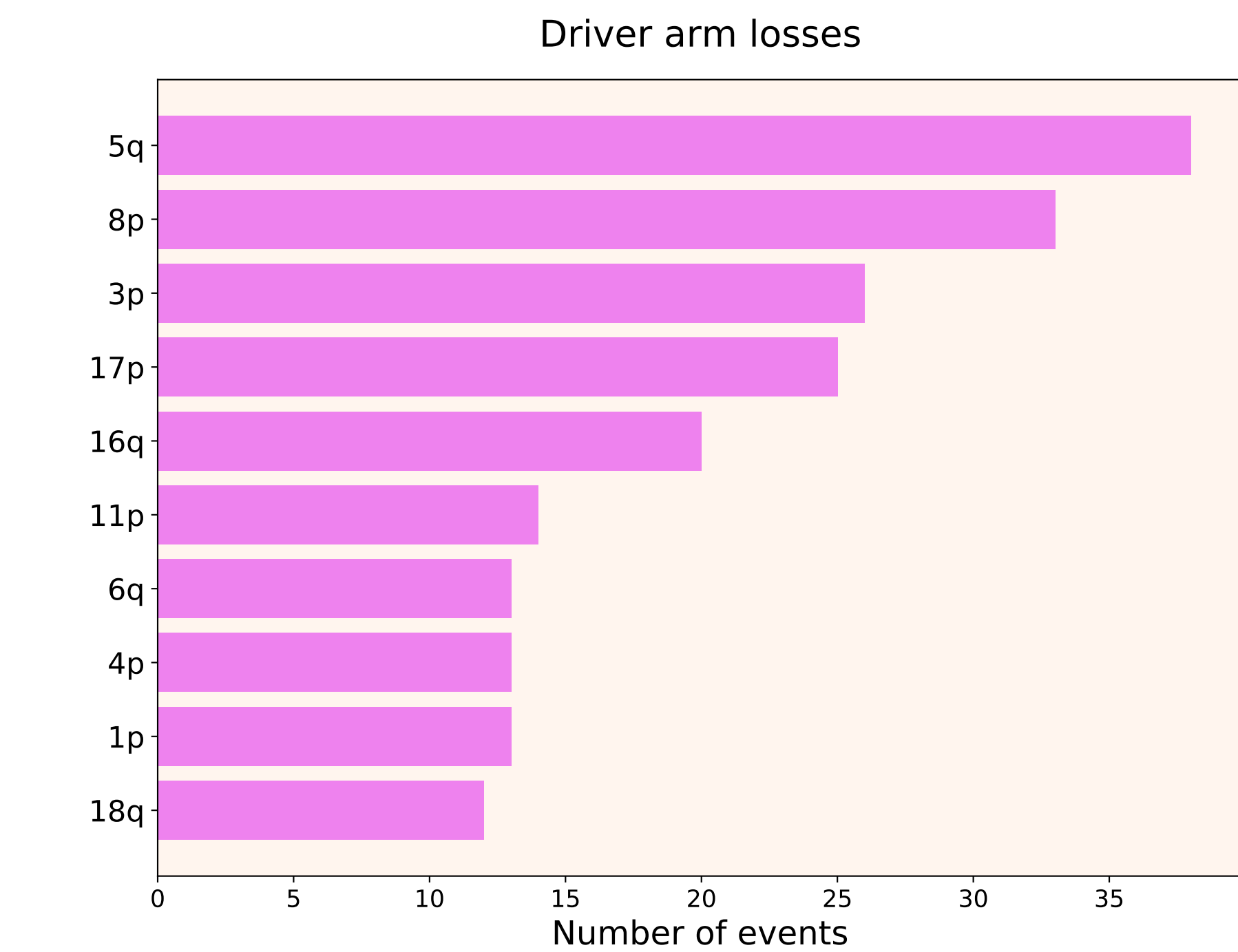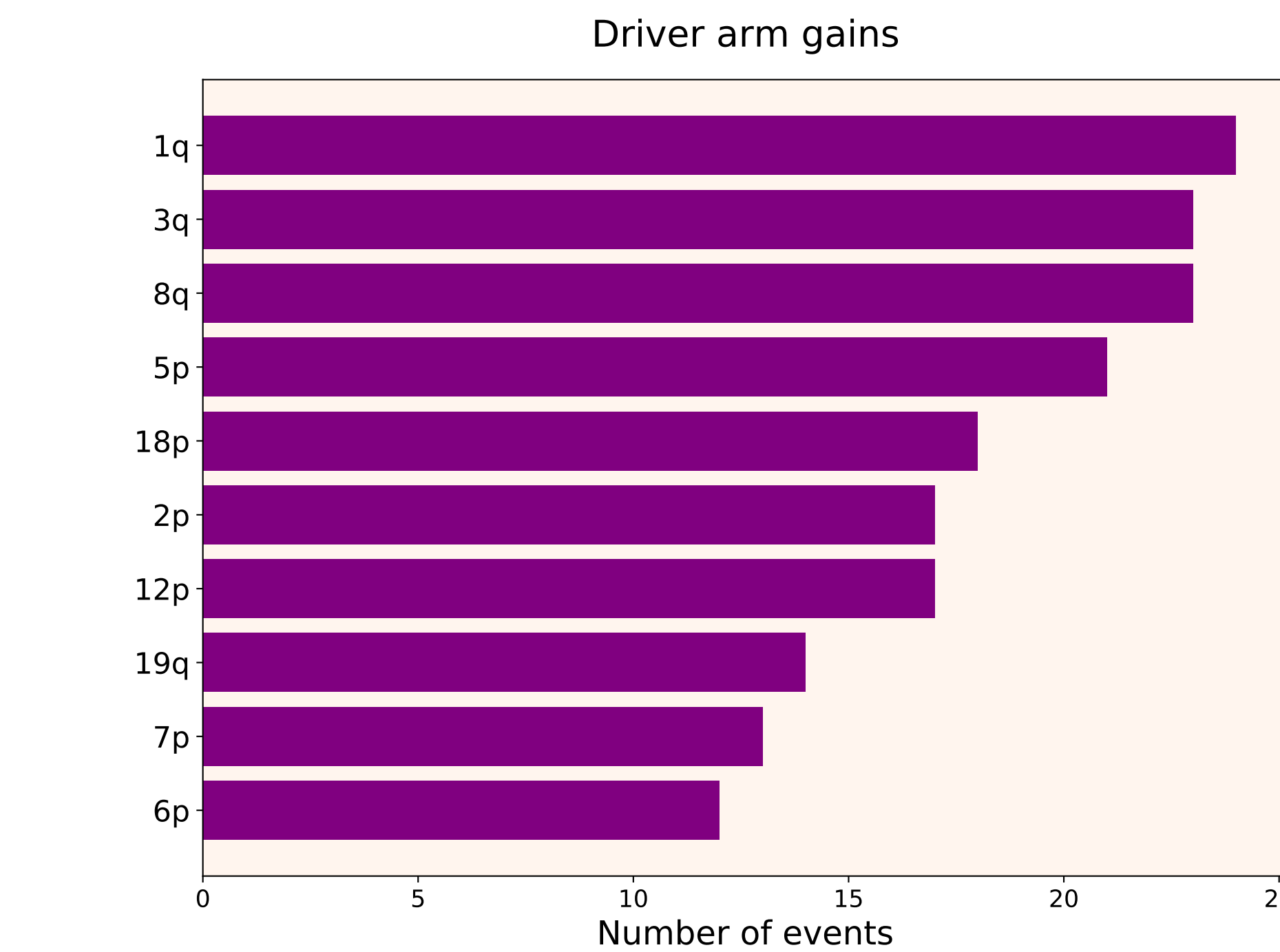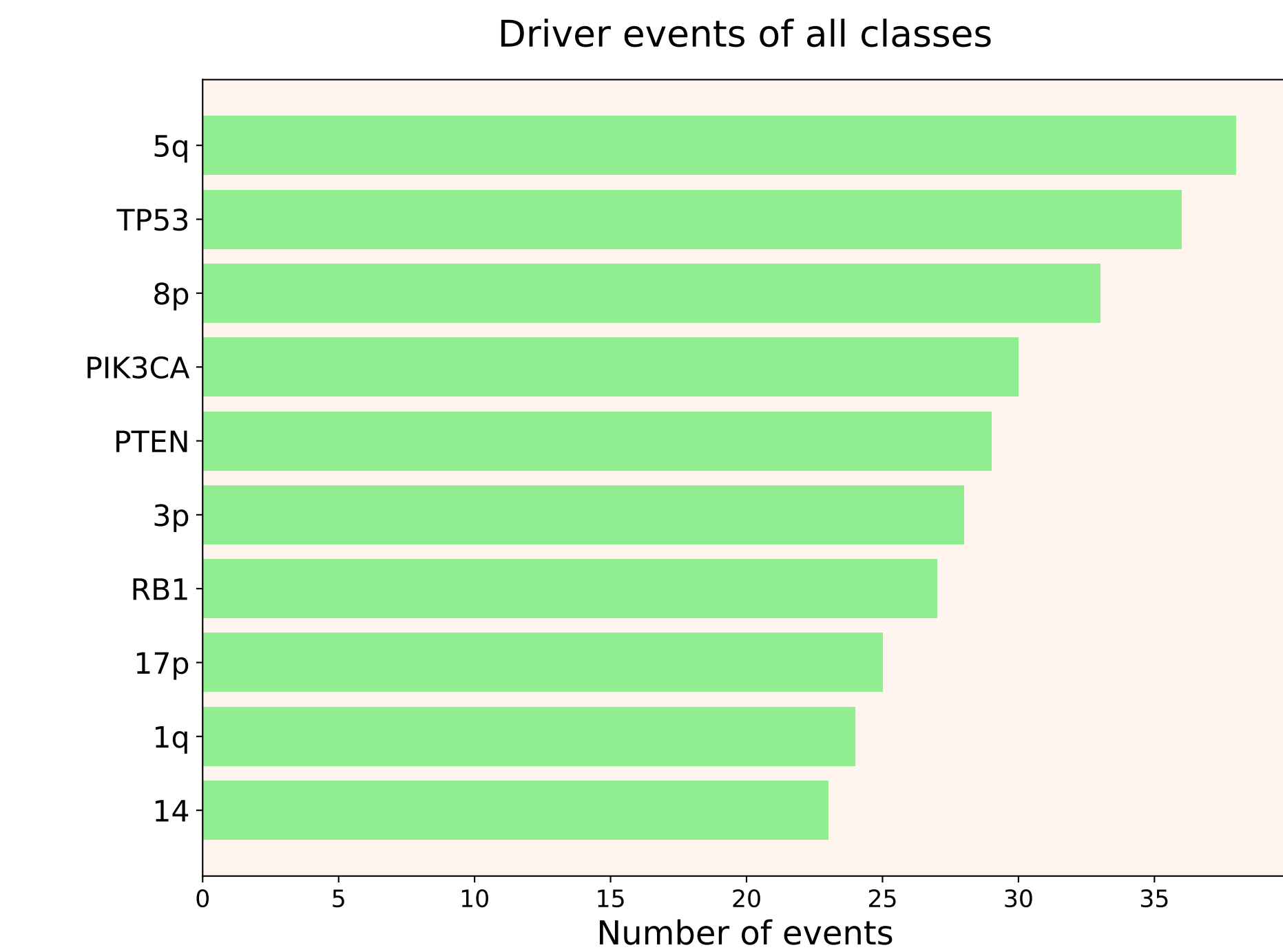

Supplement: Supplemental Information 2 [file peerj-10-13860-s002.zip › COHORTS/genes plots/detailed/2021_8_16_14_9_distribution_events_detailed_29.pdf]

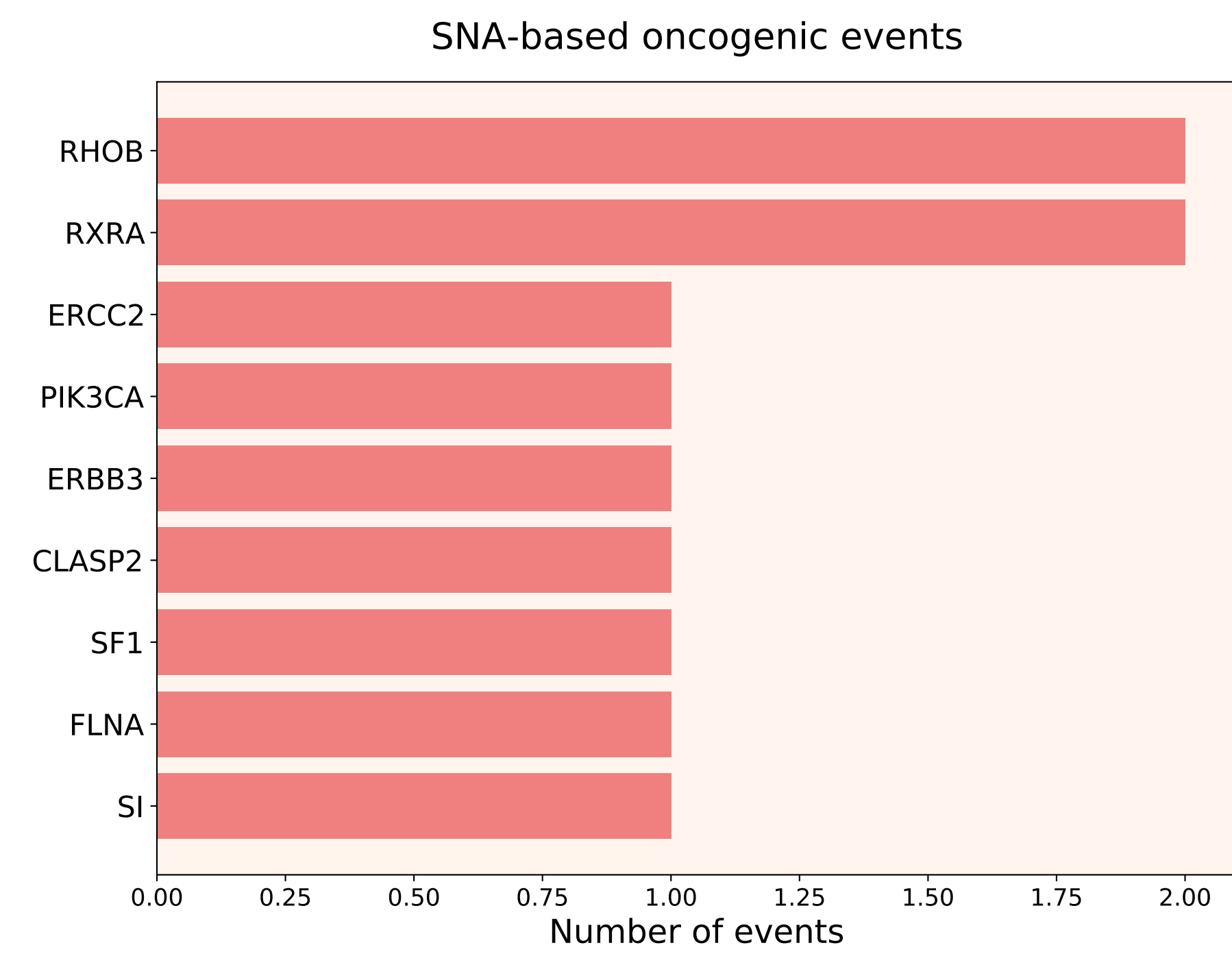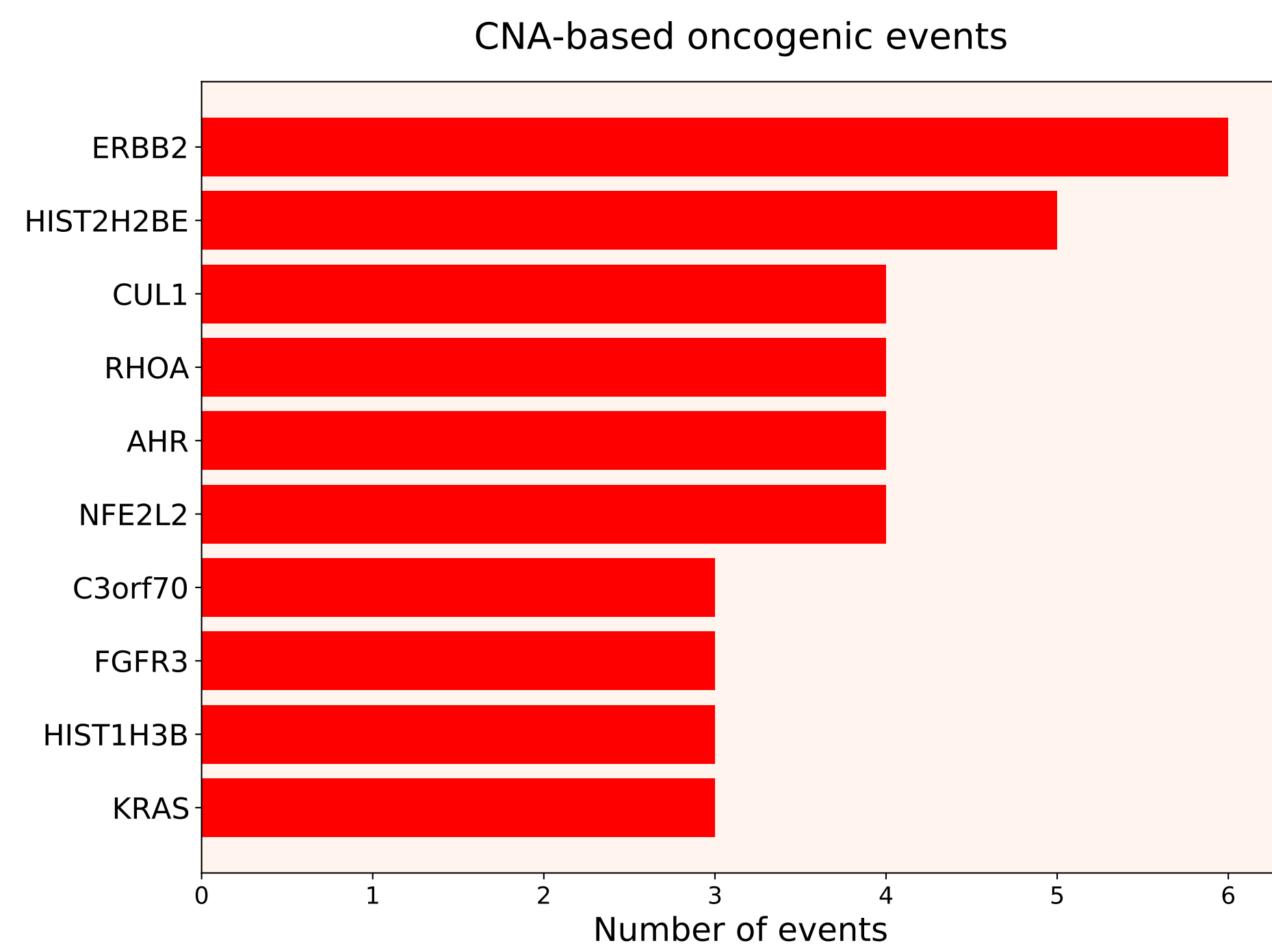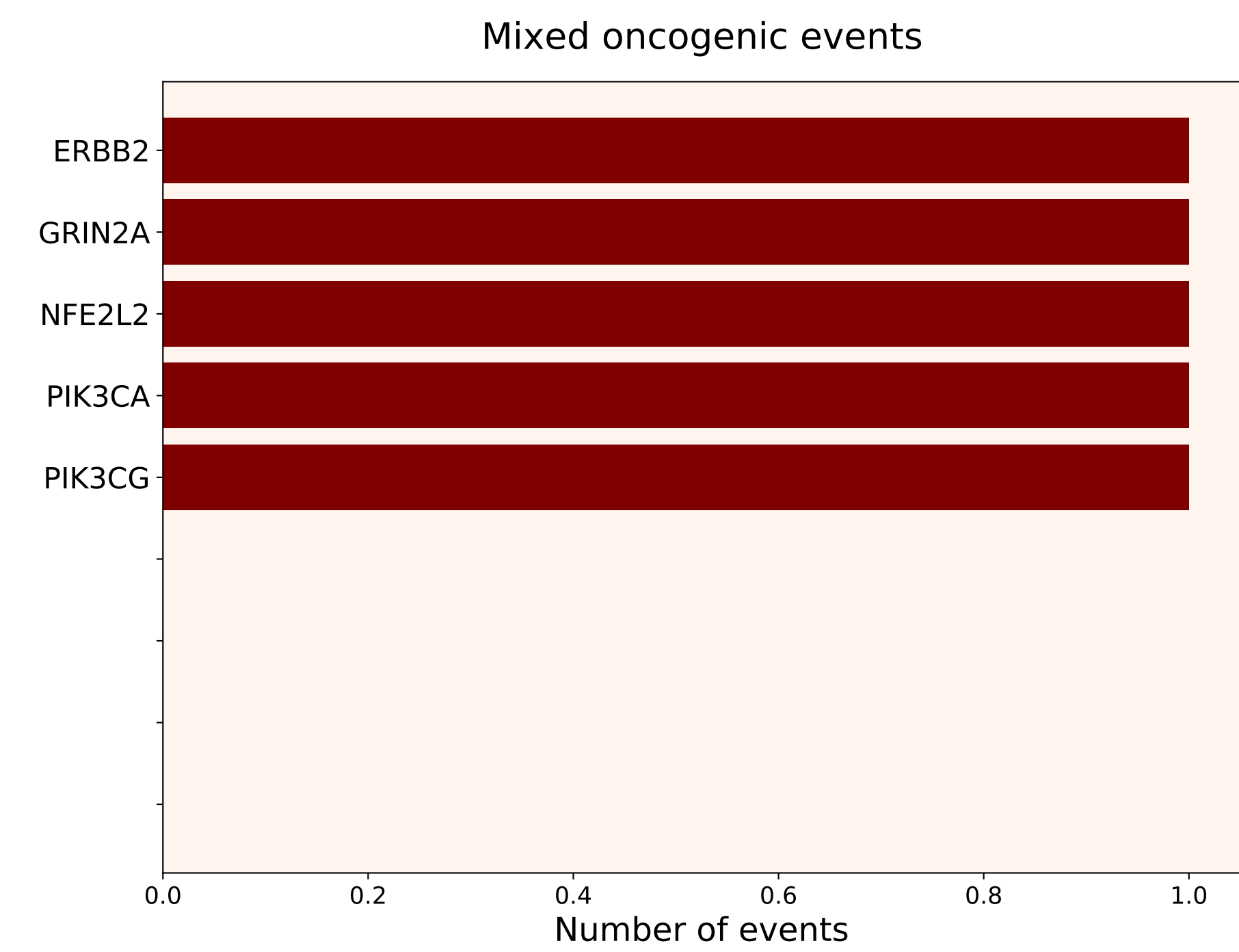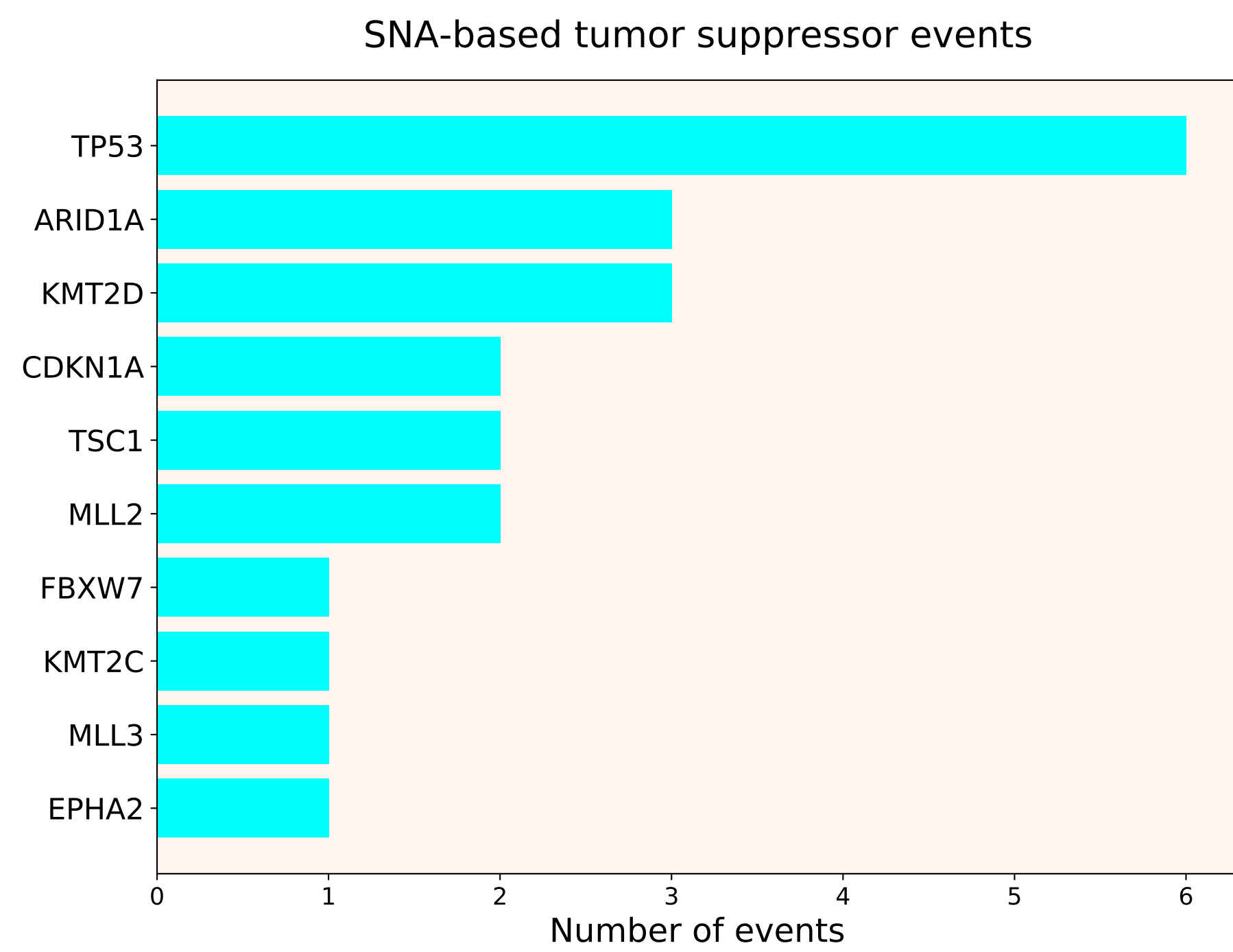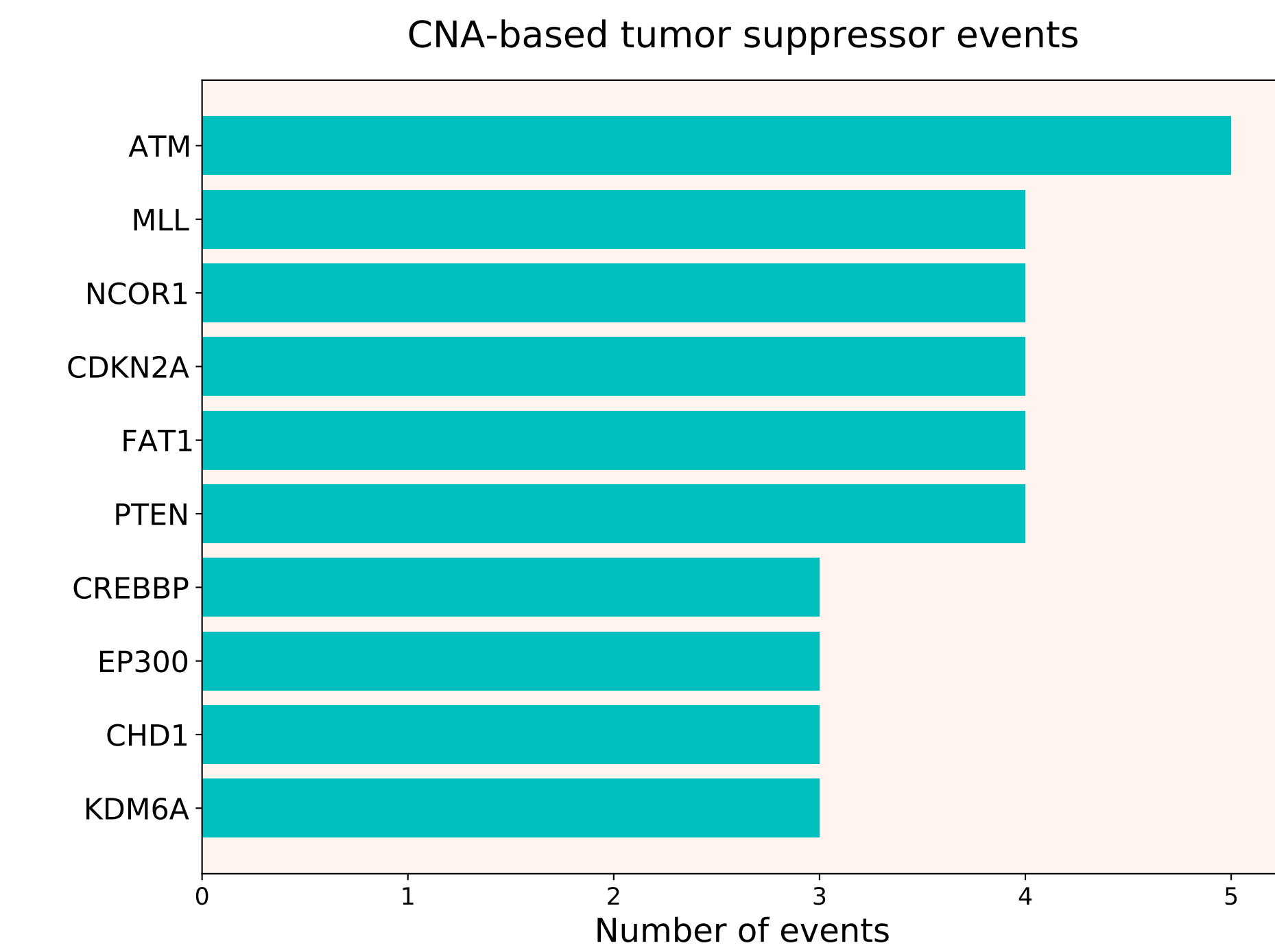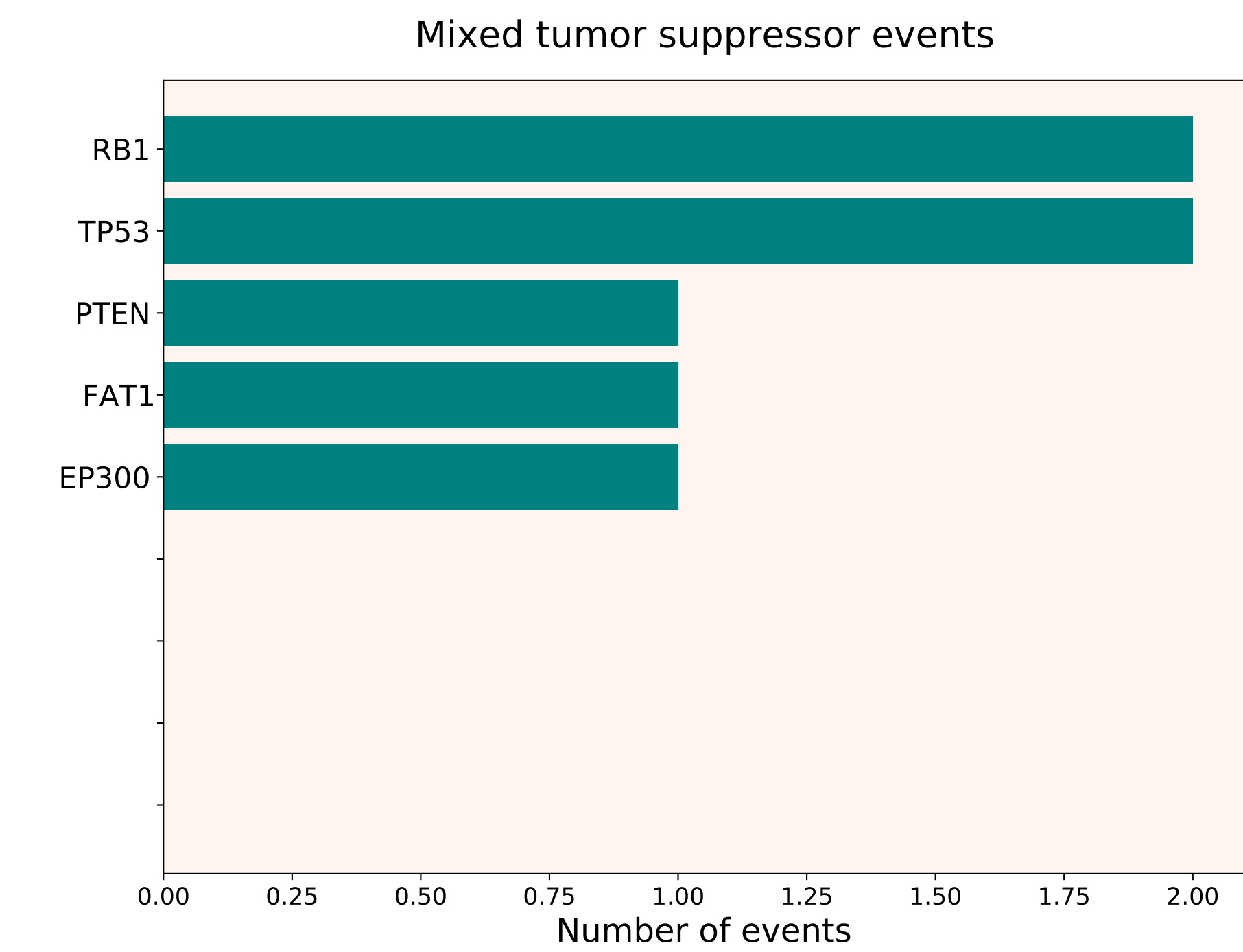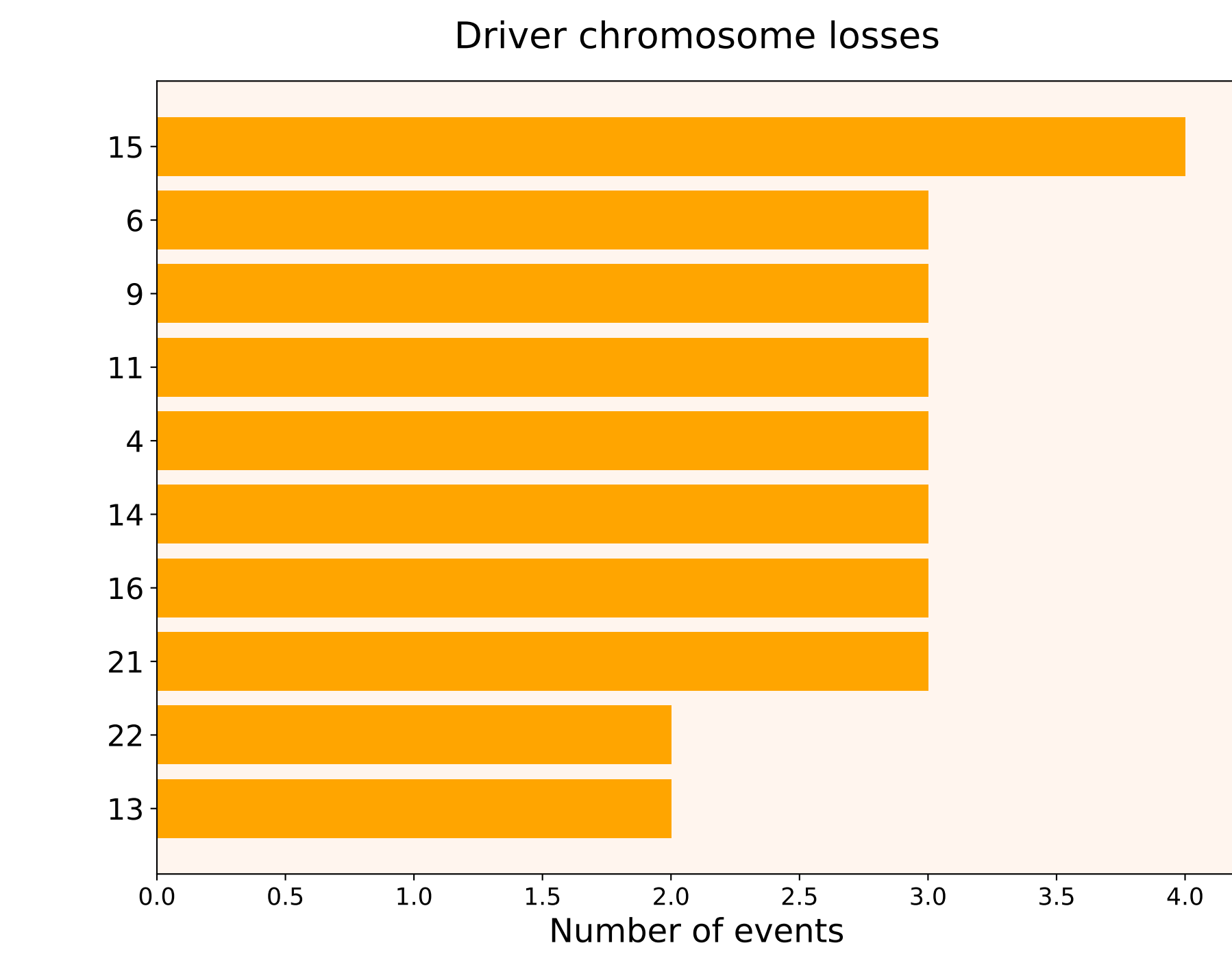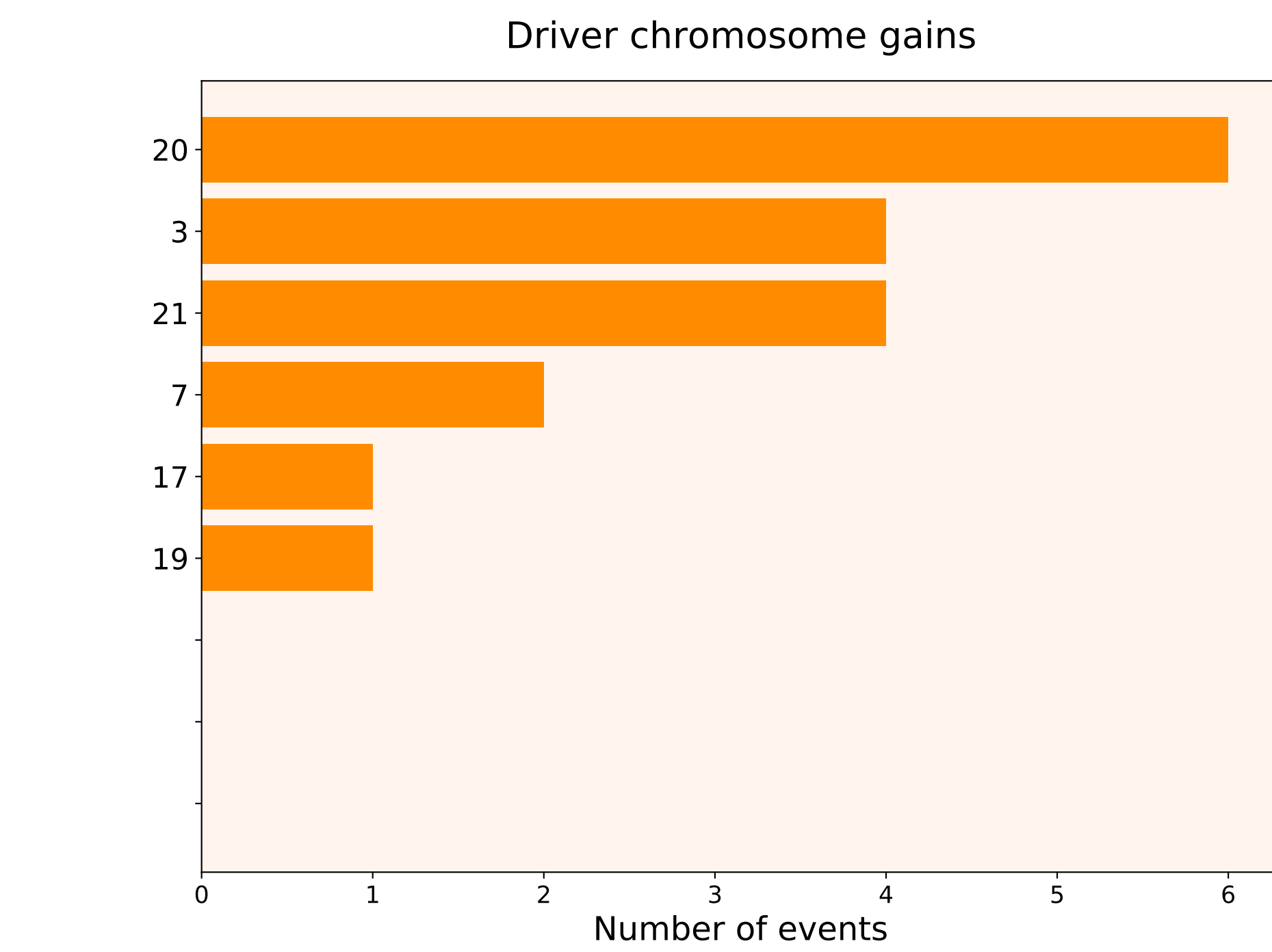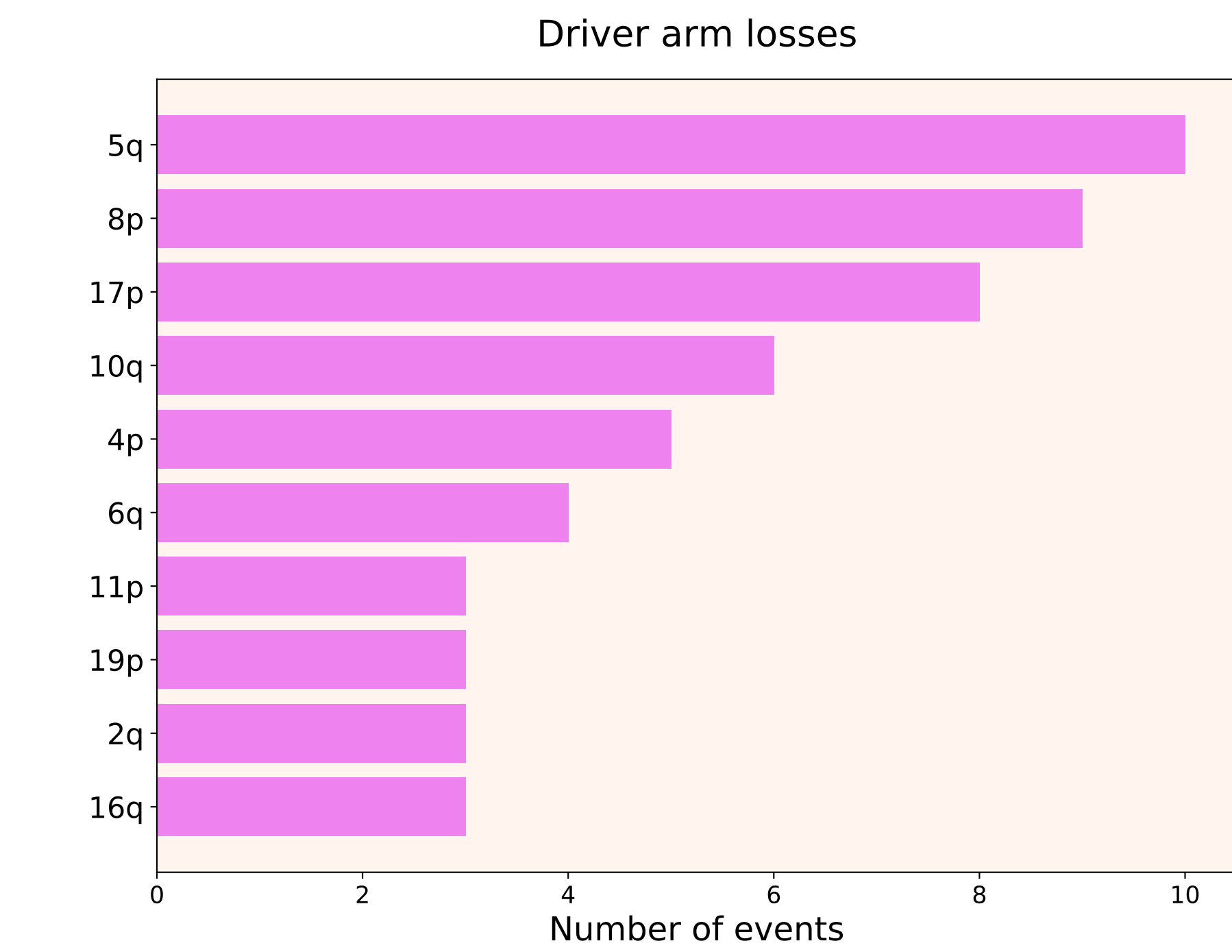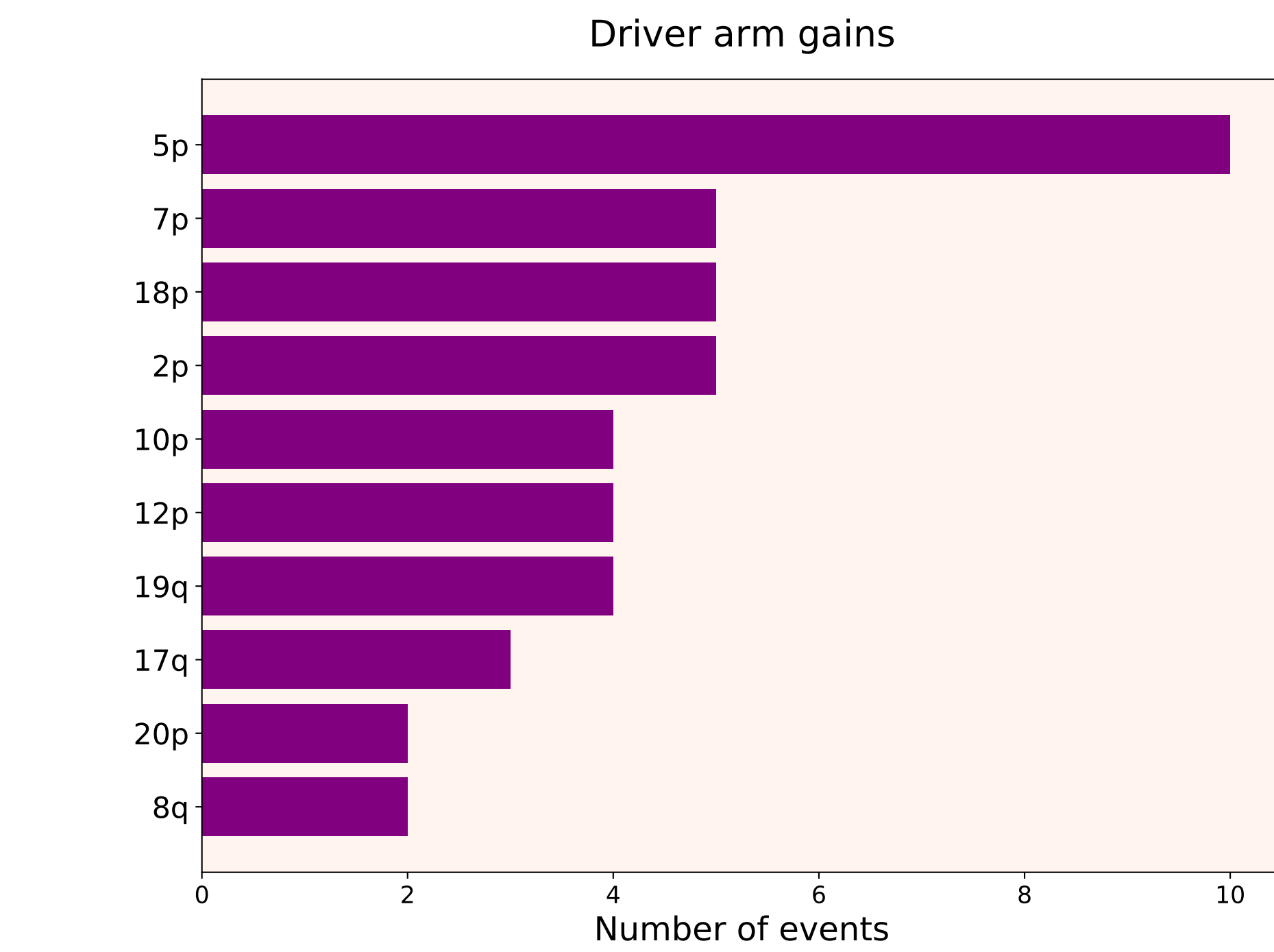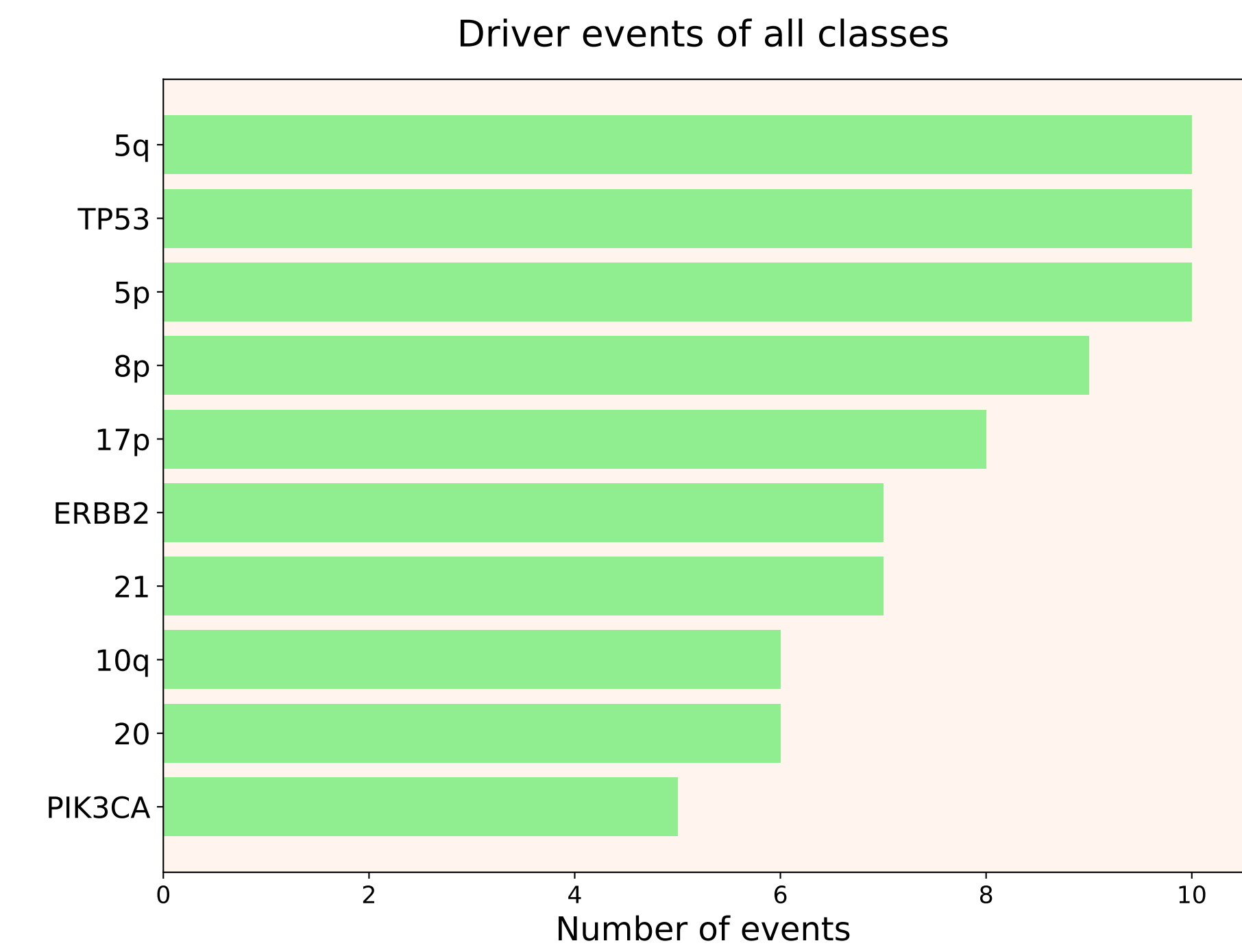

Supplement: Supplemental Information 2 [file peerj-10-13860-s002.zip › COHORTS/genes plots/detailed/2021_8_16_14_9_distribution_events_detailed_males_34.pdf]

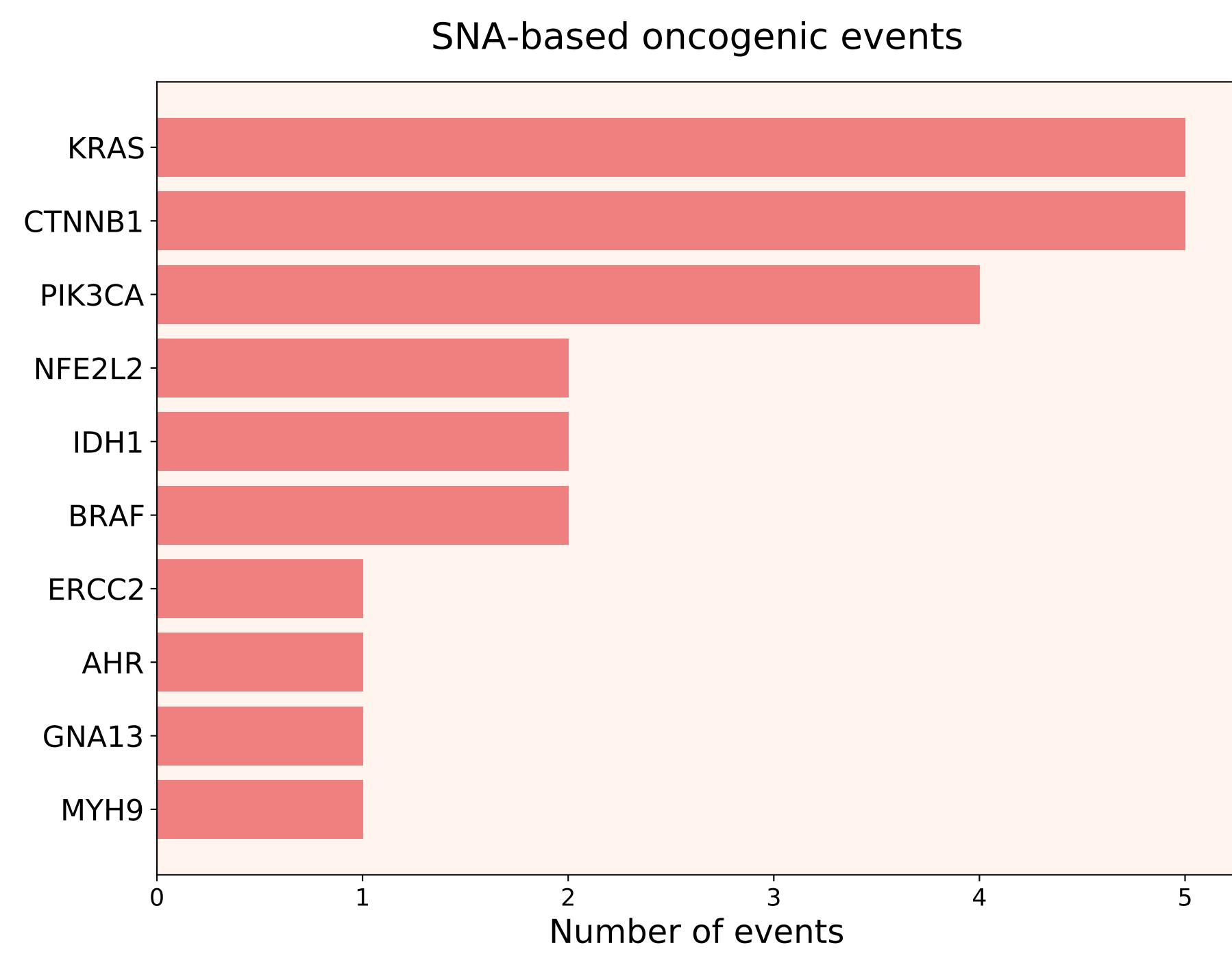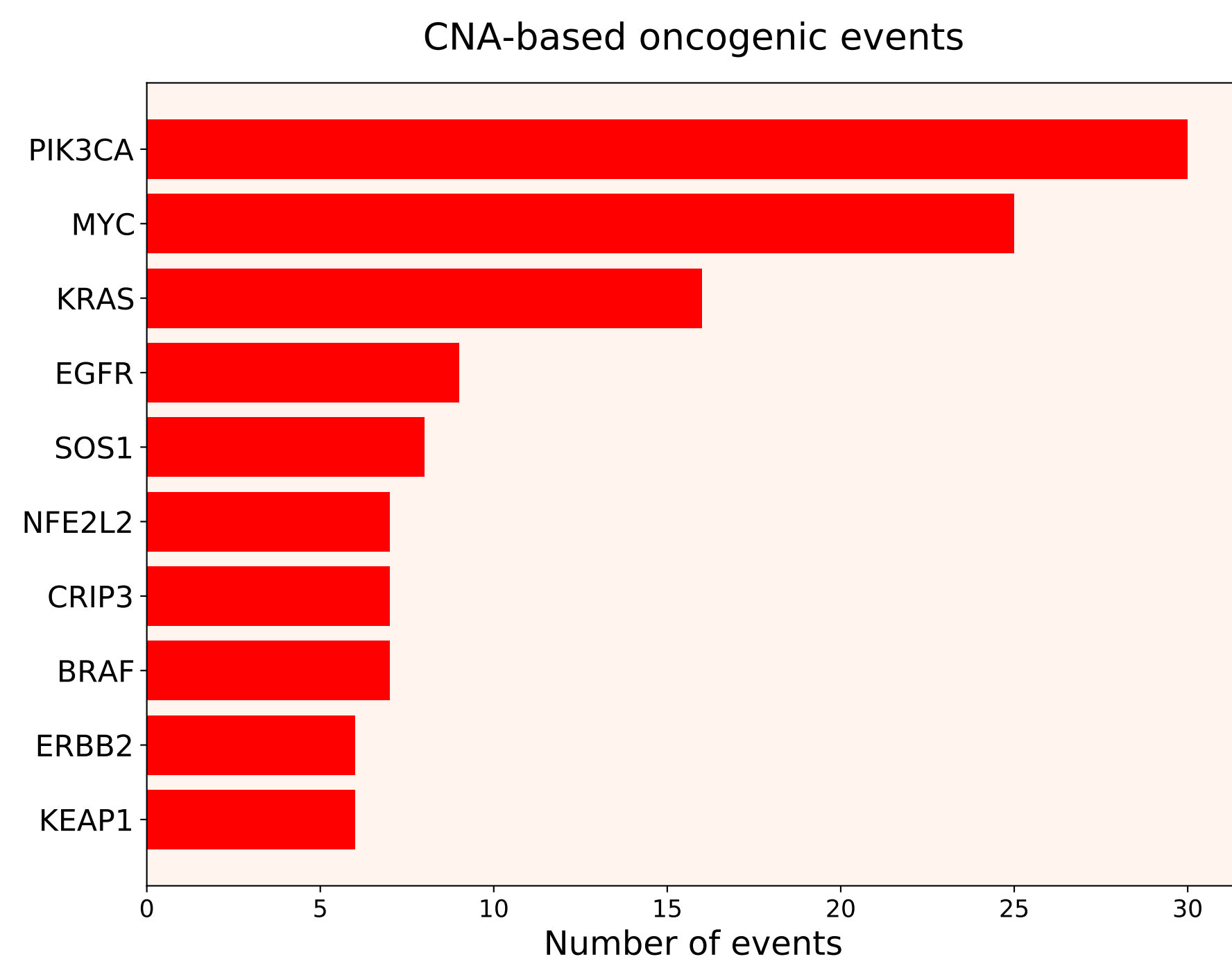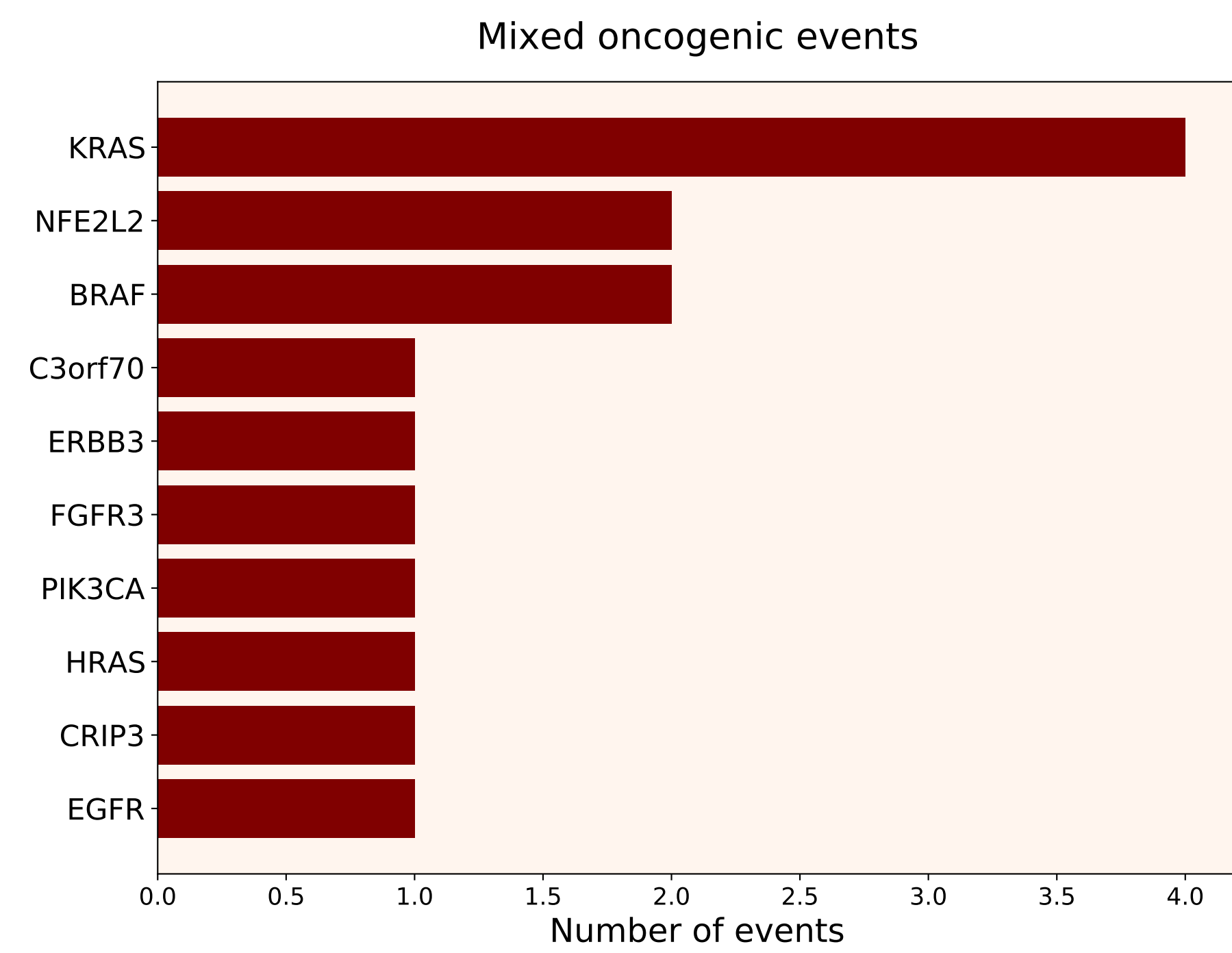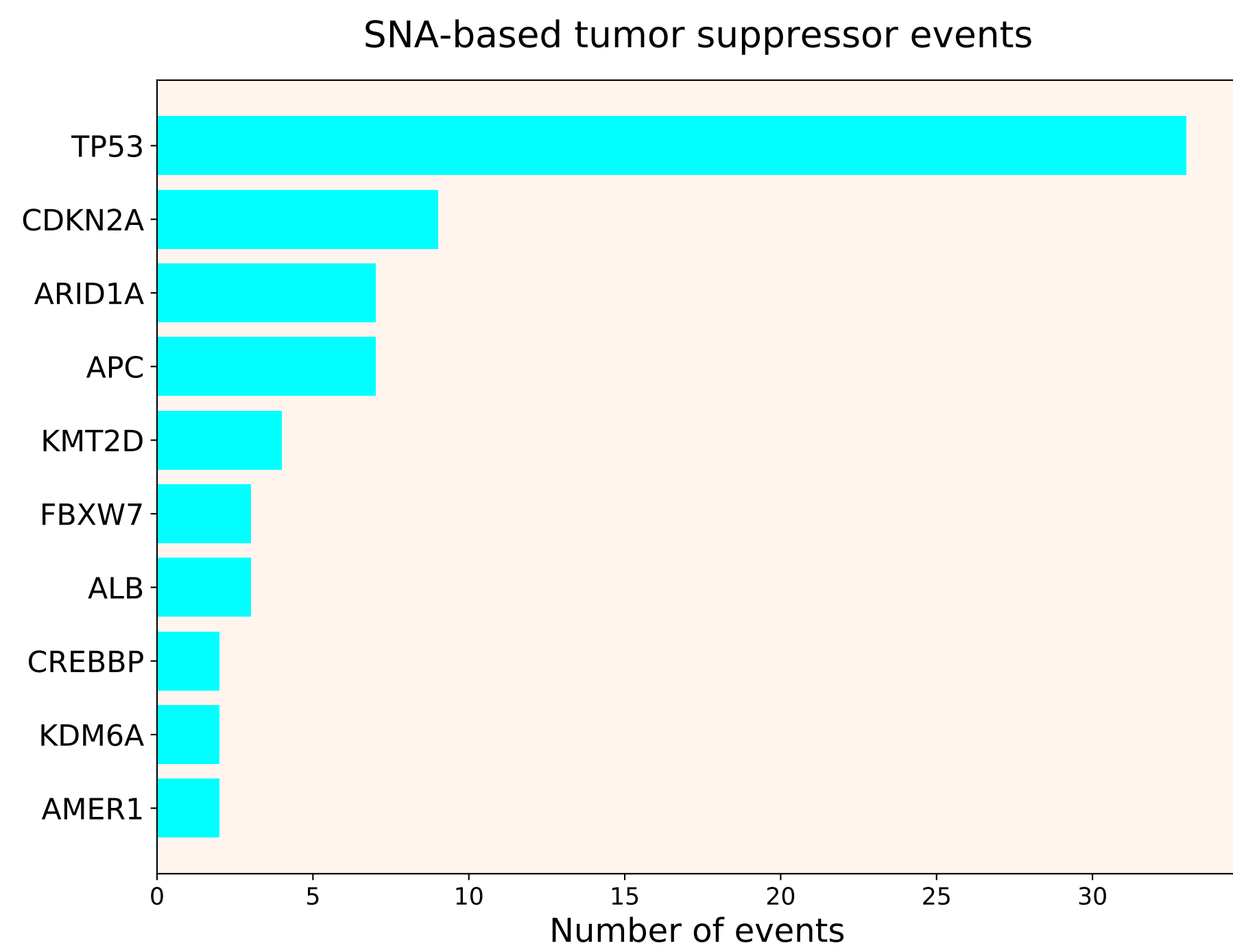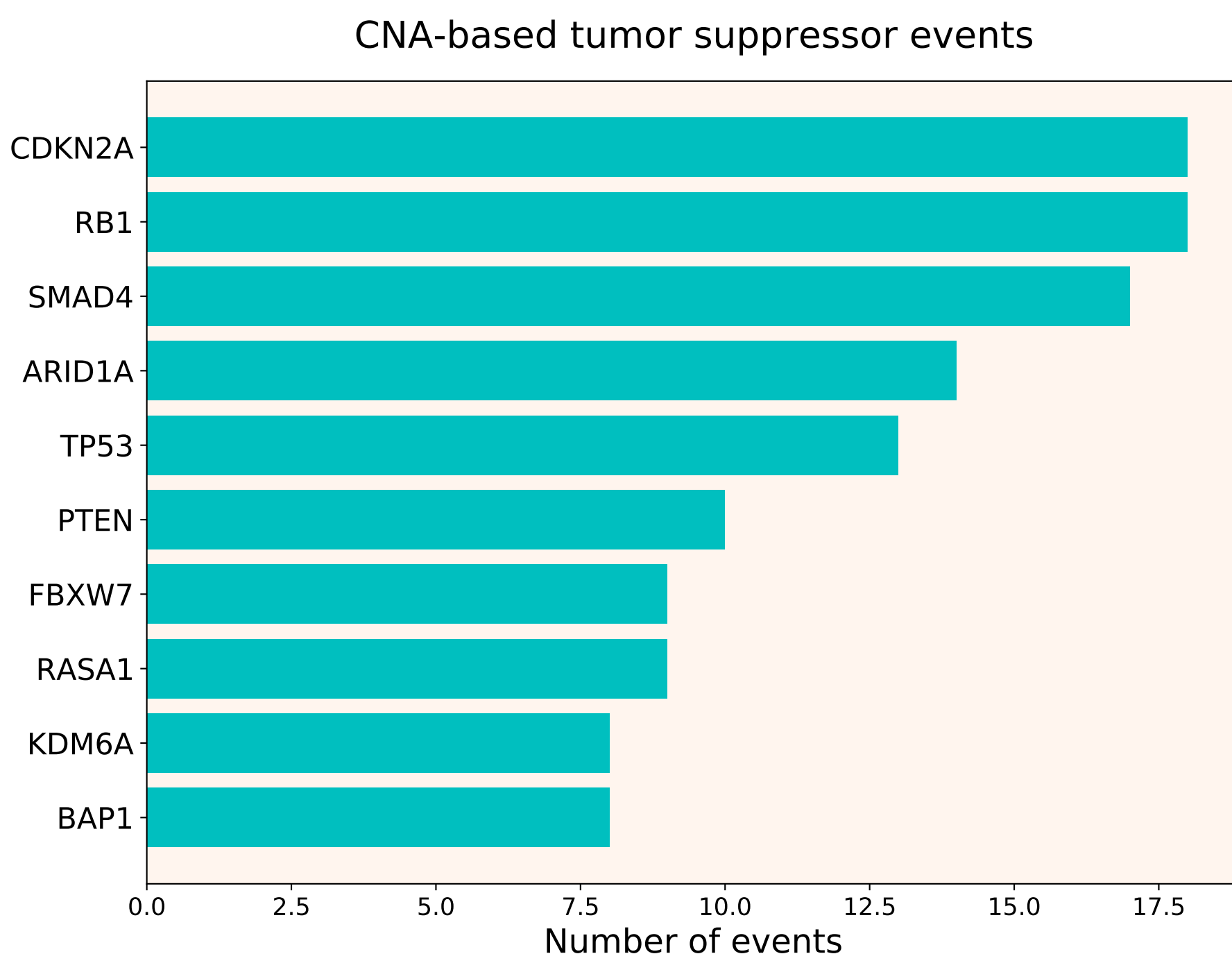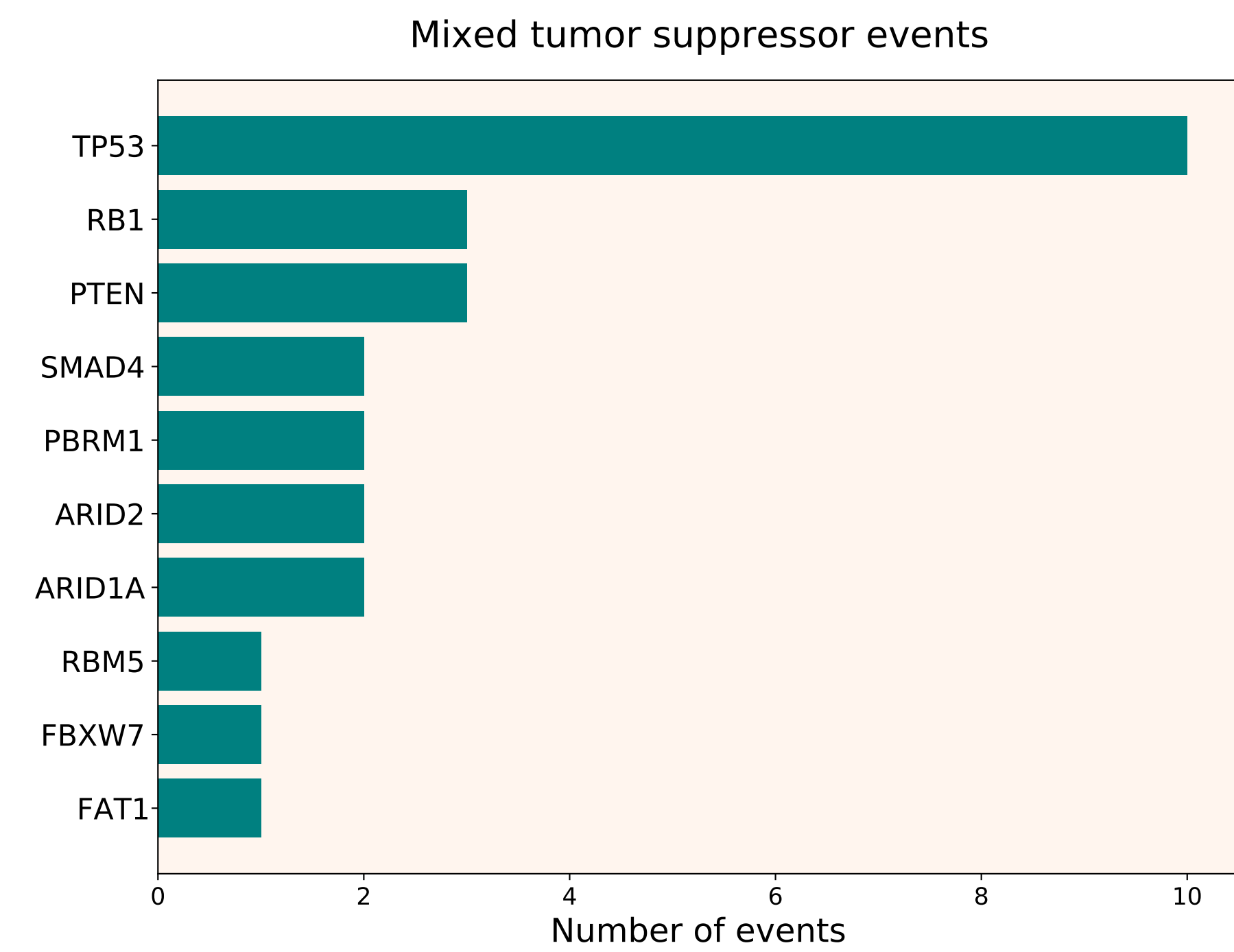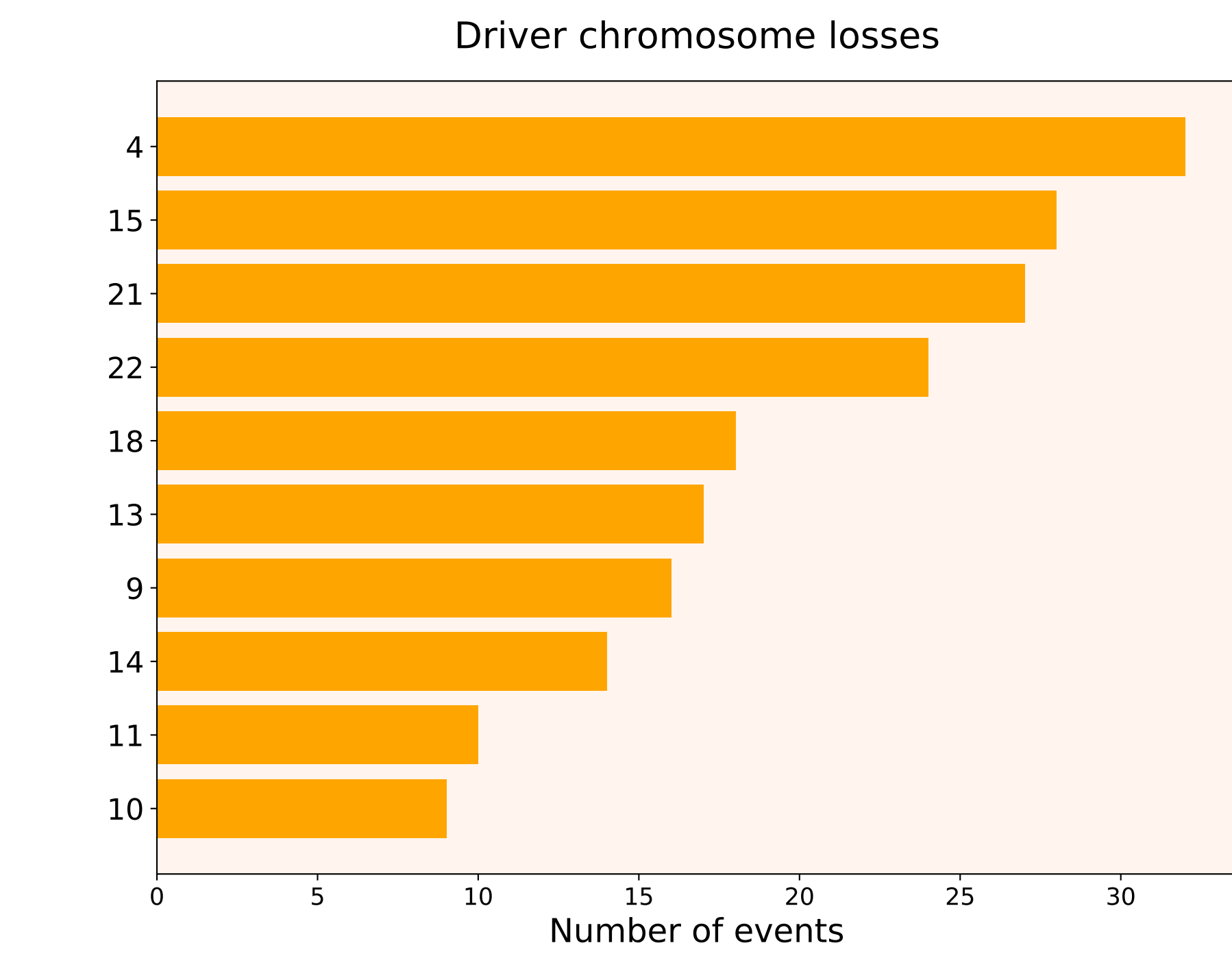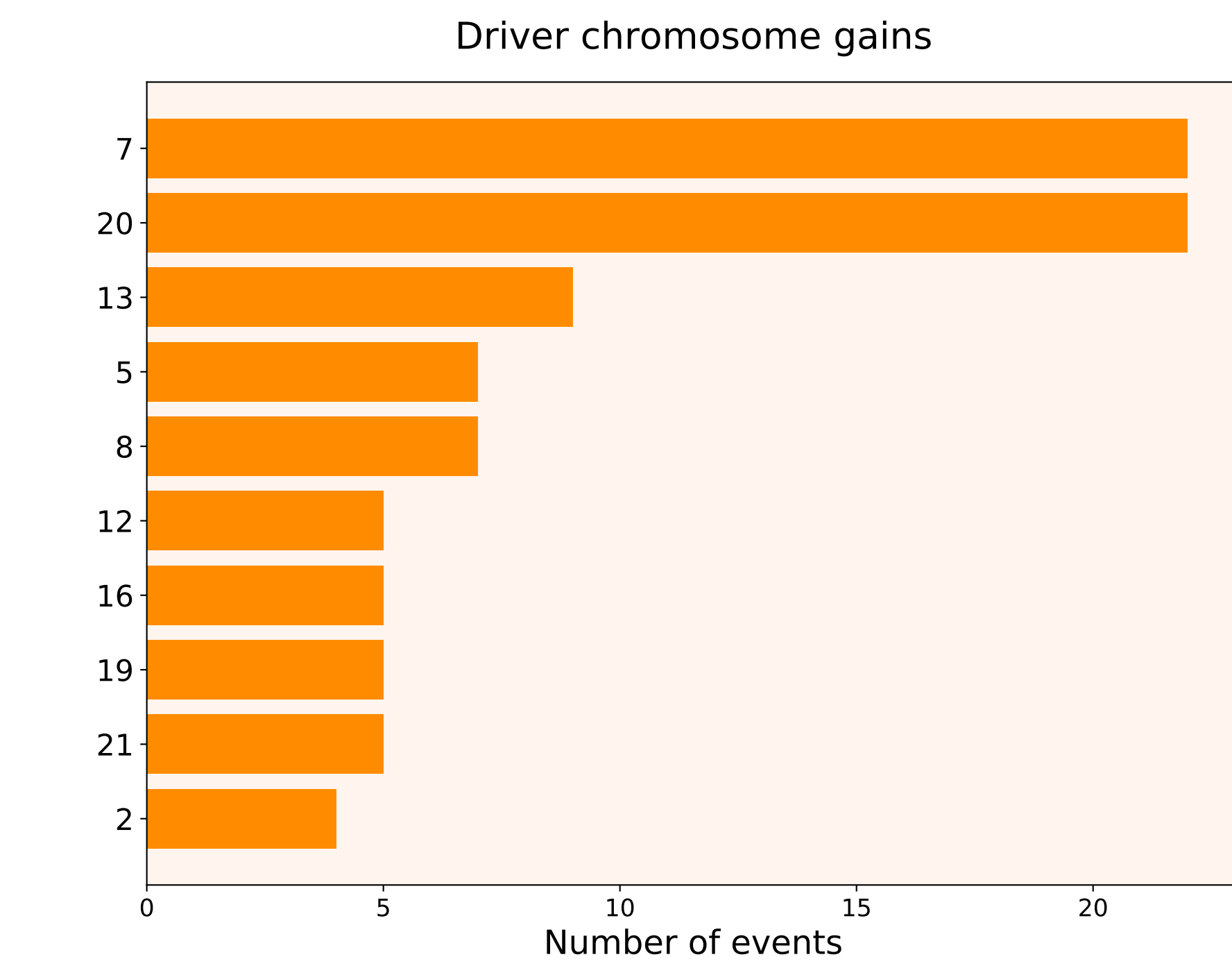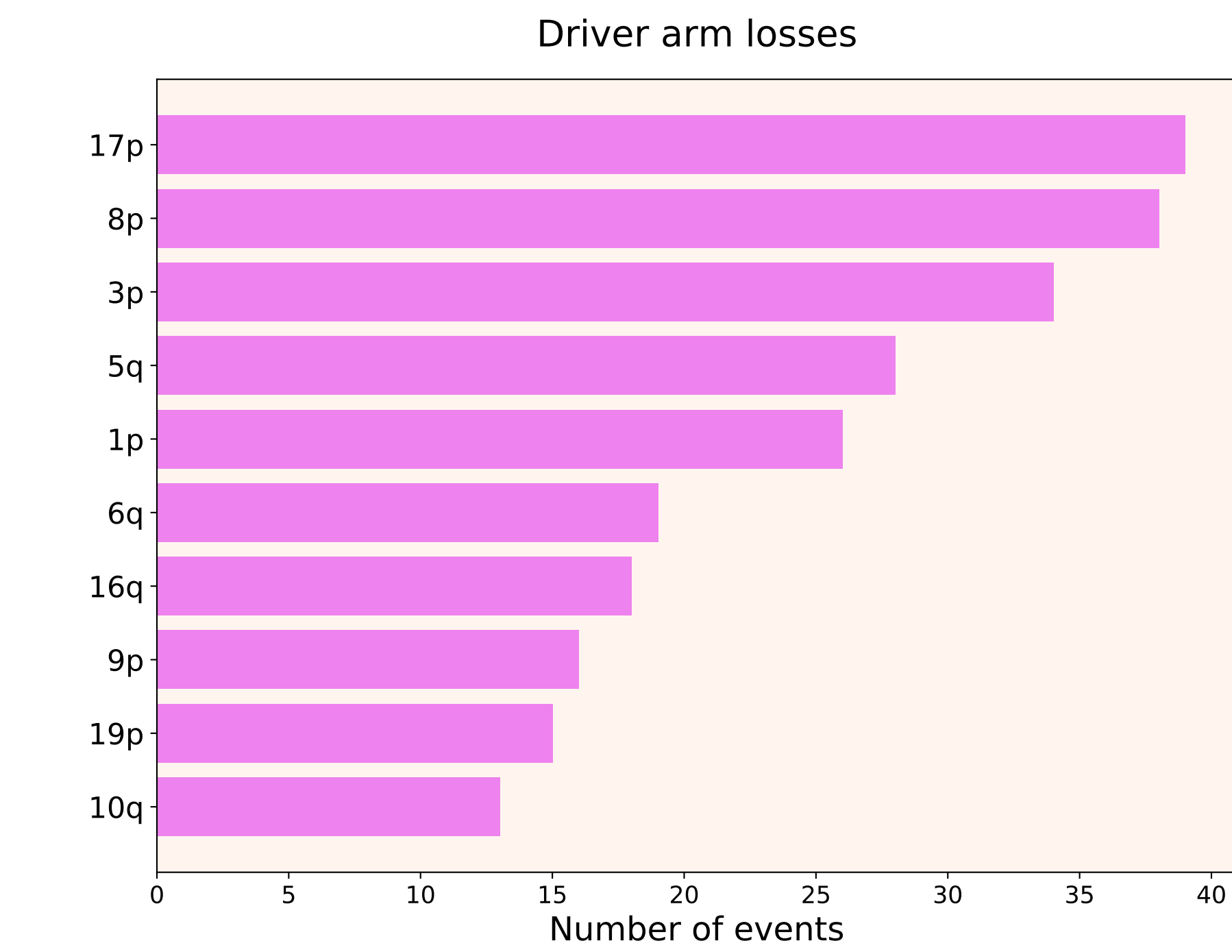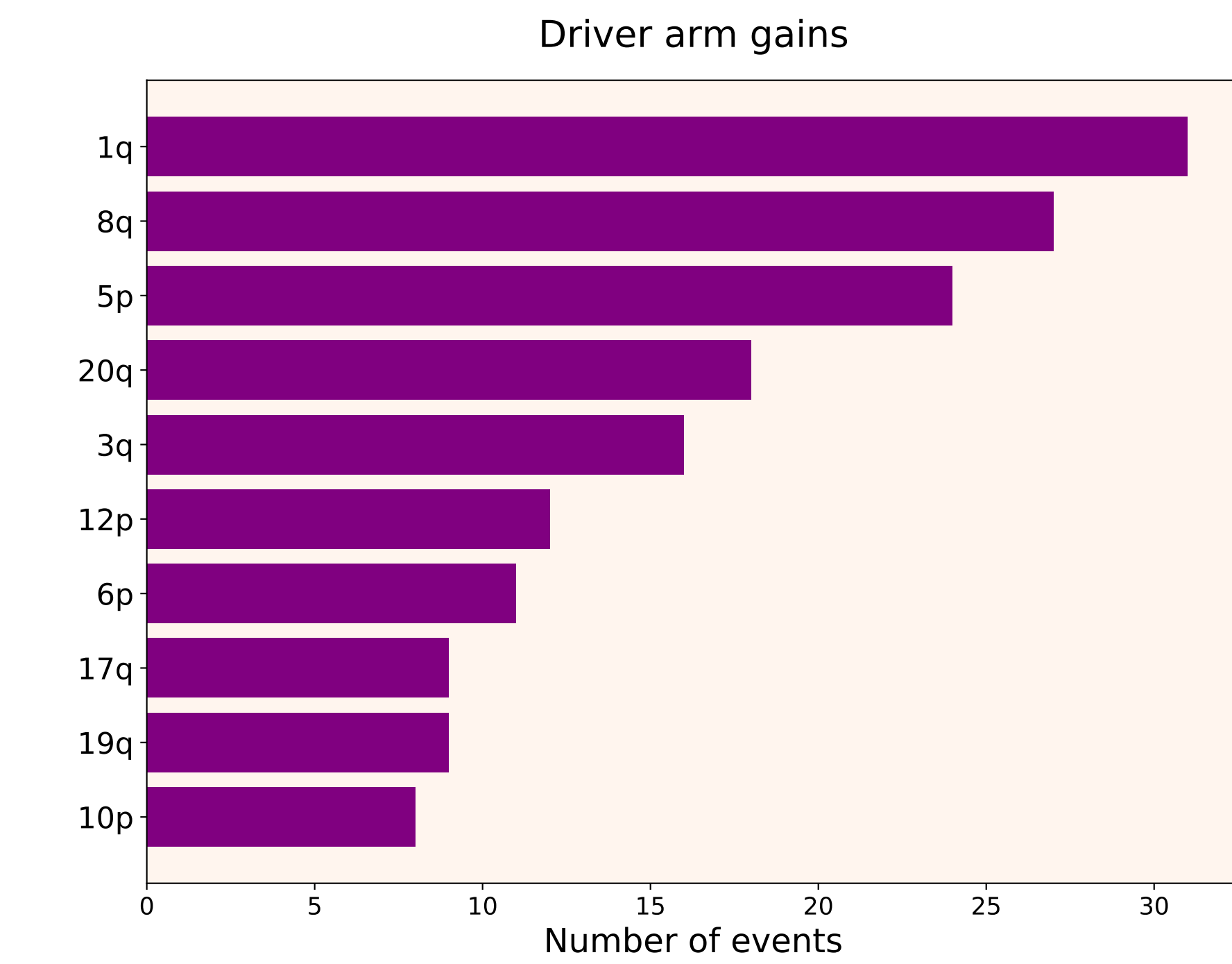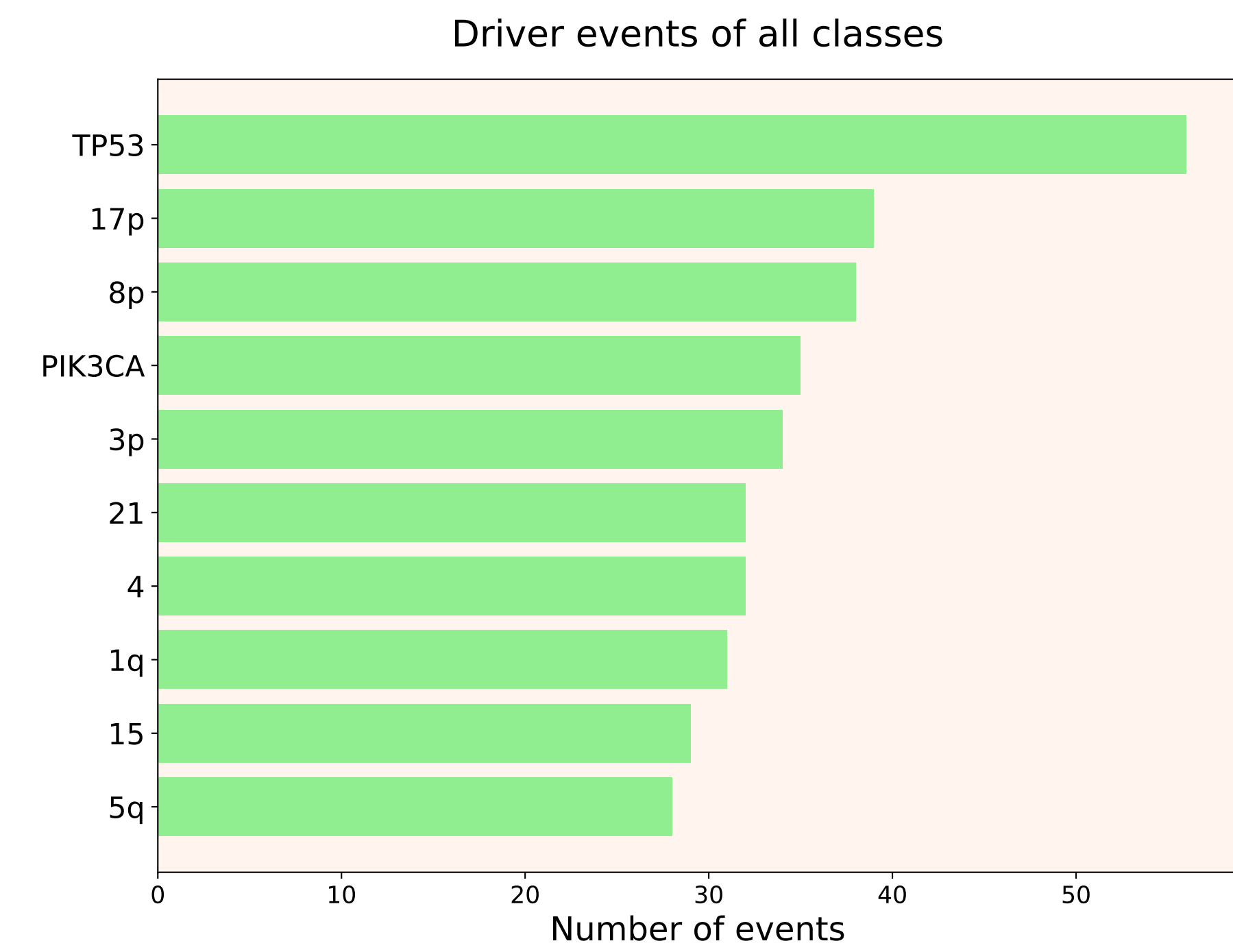

Supplement: Supplemental Information 2 [file peerj-10-13860-s002.zip › COHORTS/genes plots/detailed/2021_8_16_14_9_distribution_events_detailed_males_20.pdf]

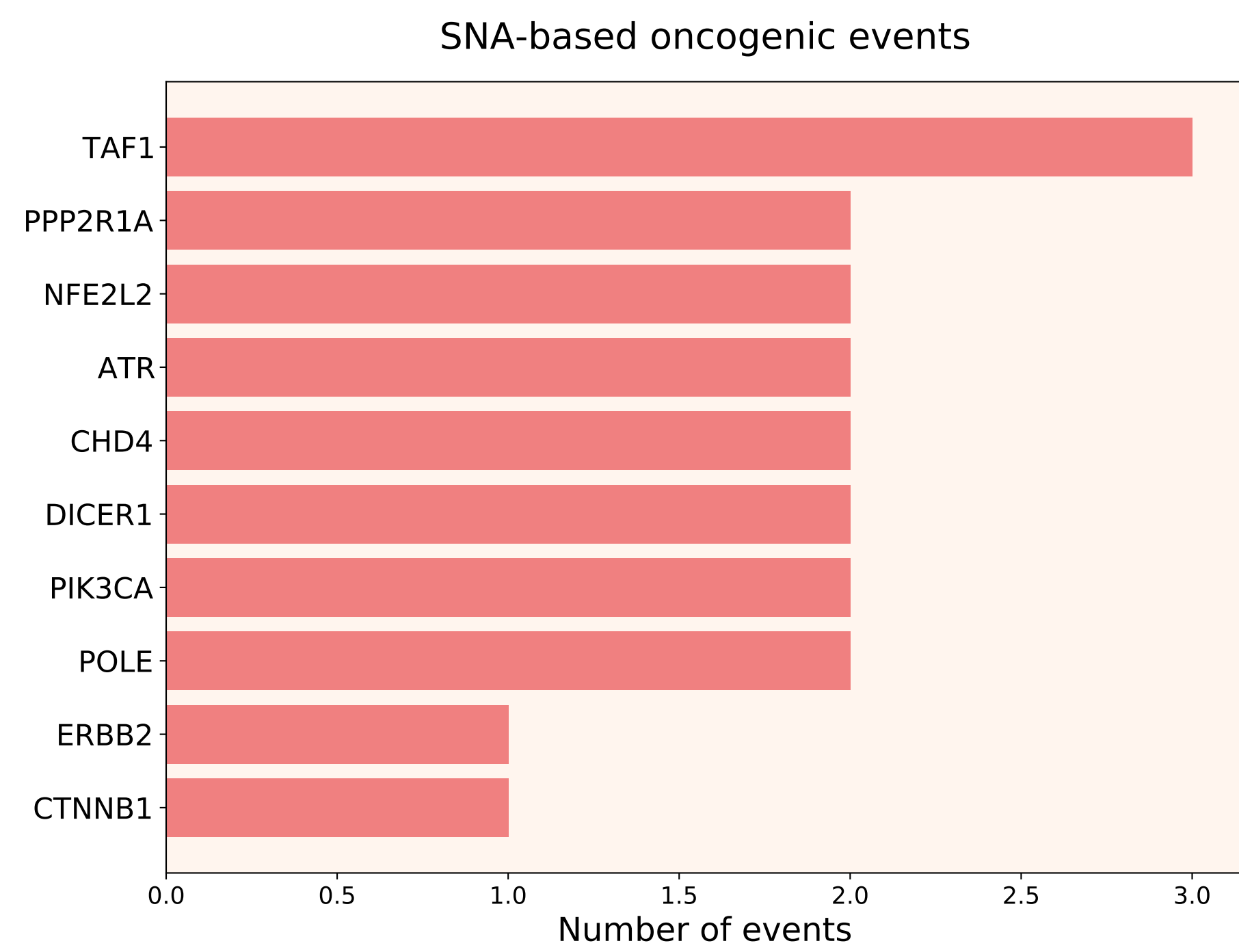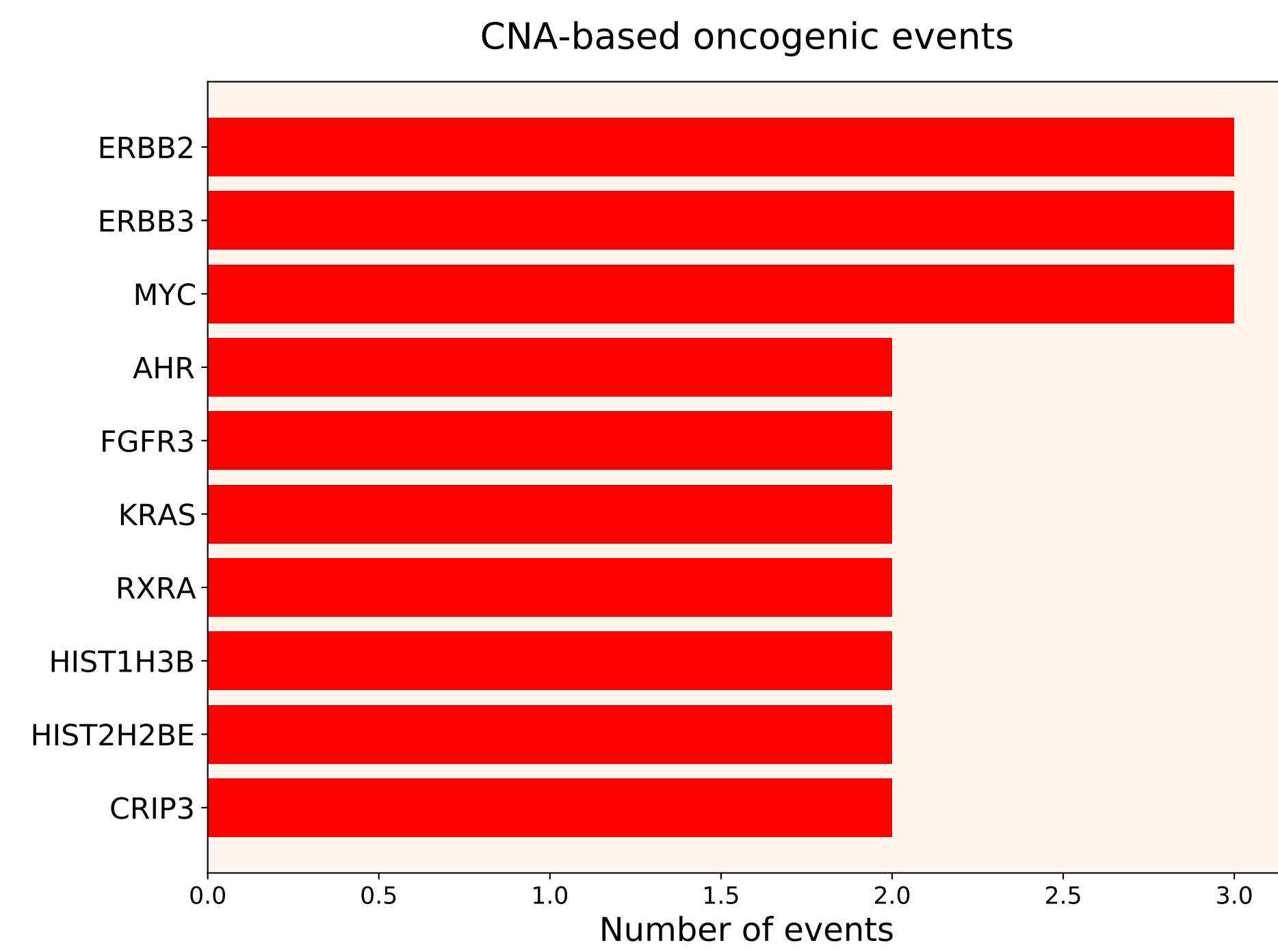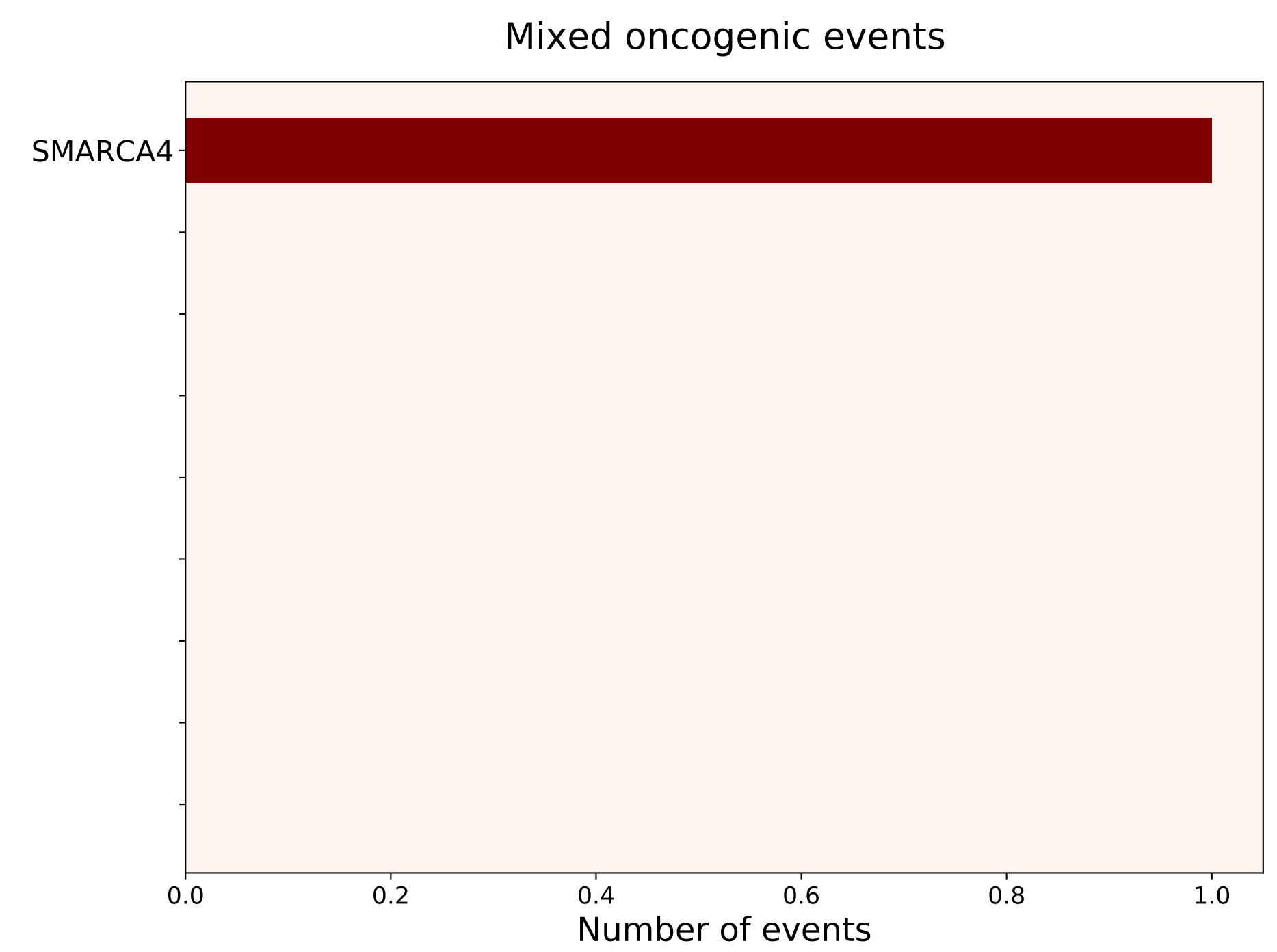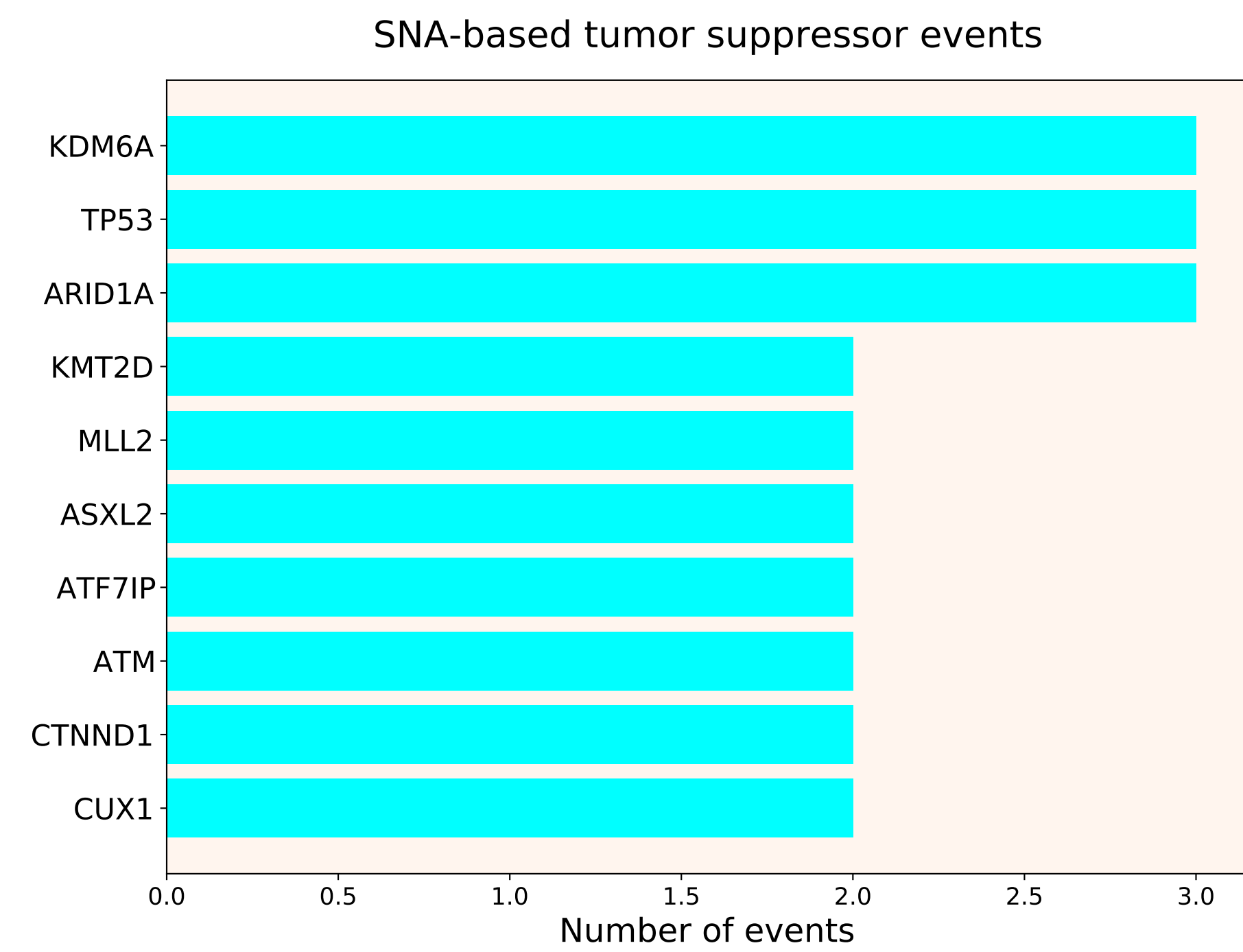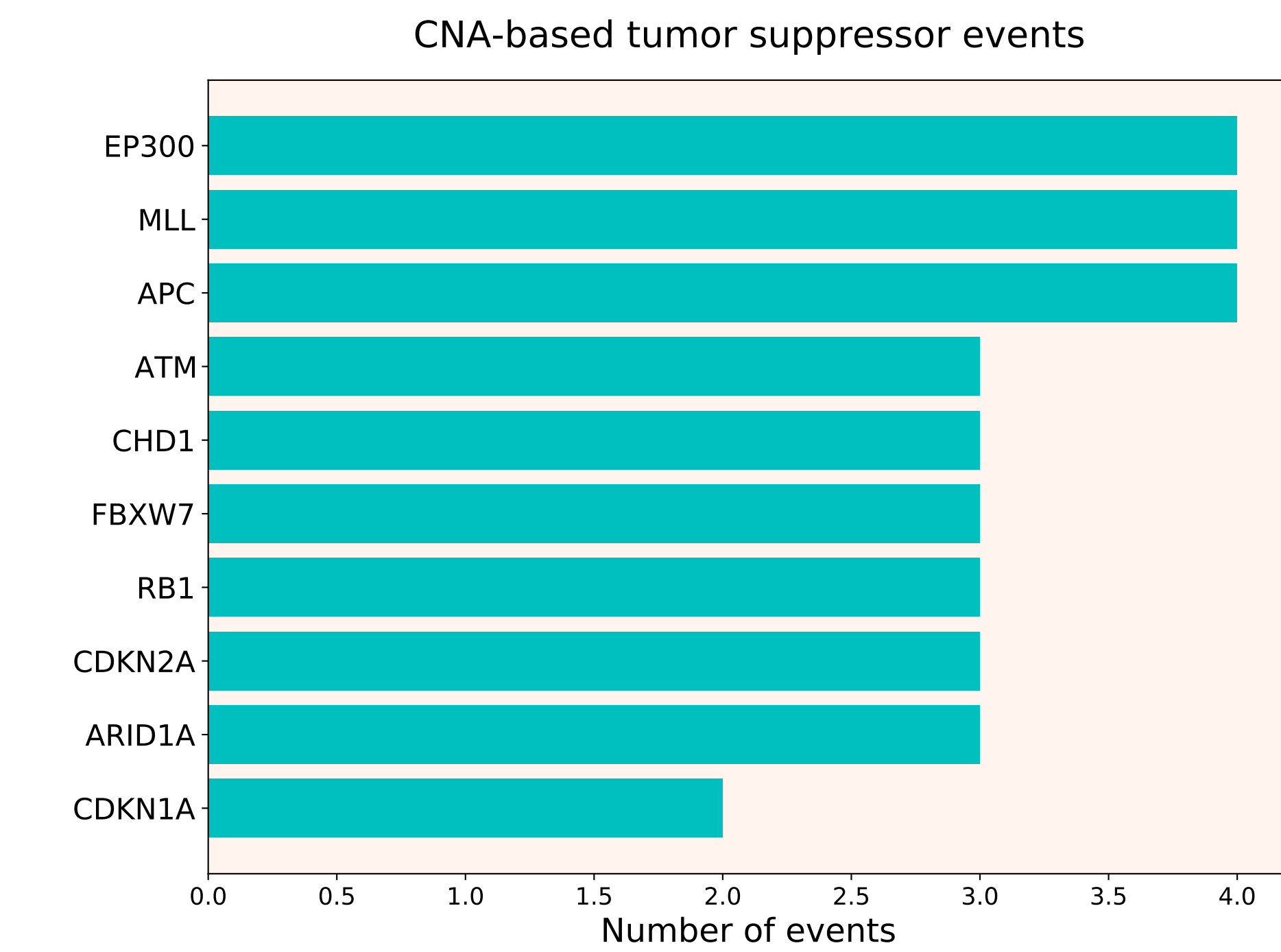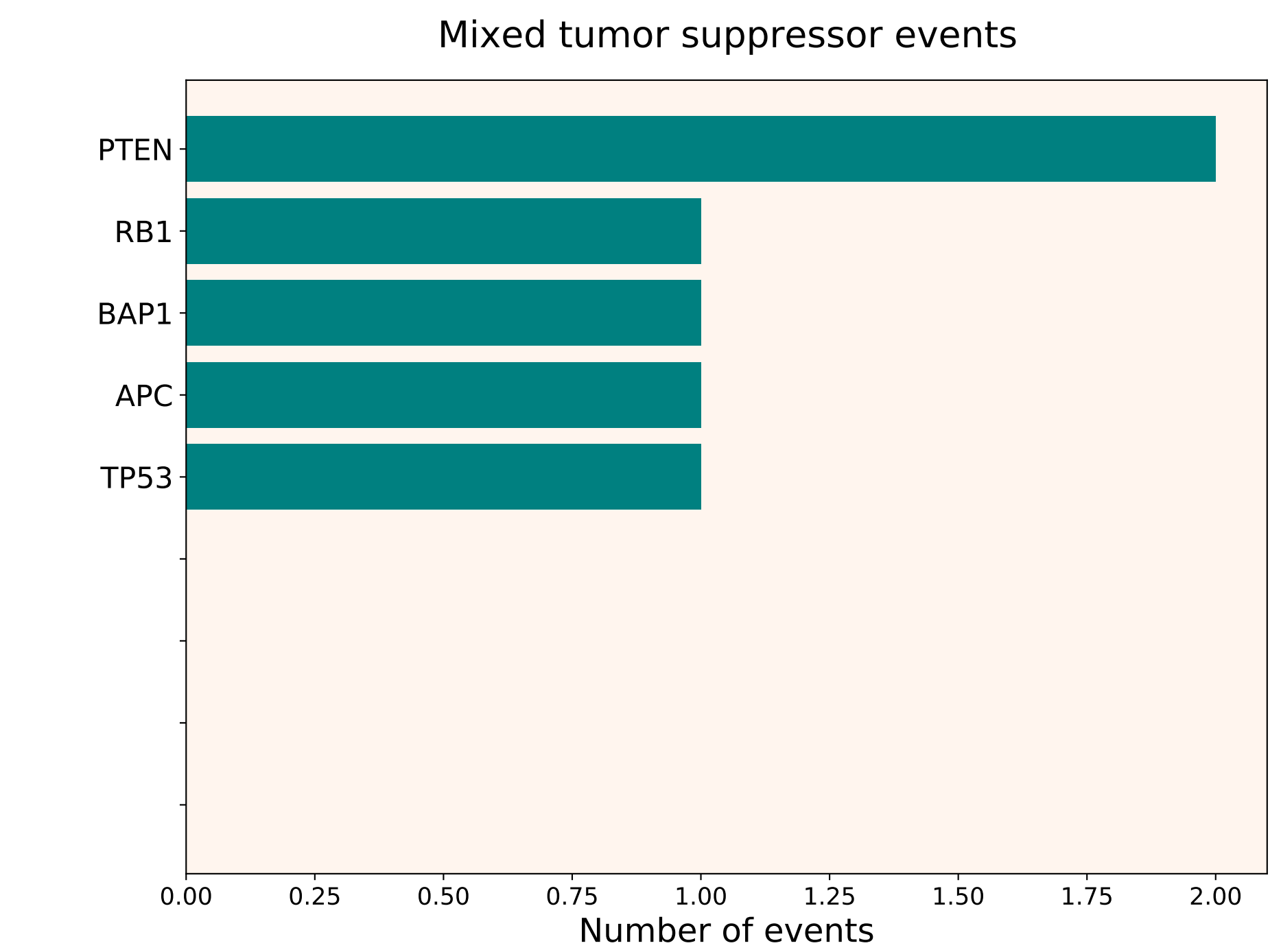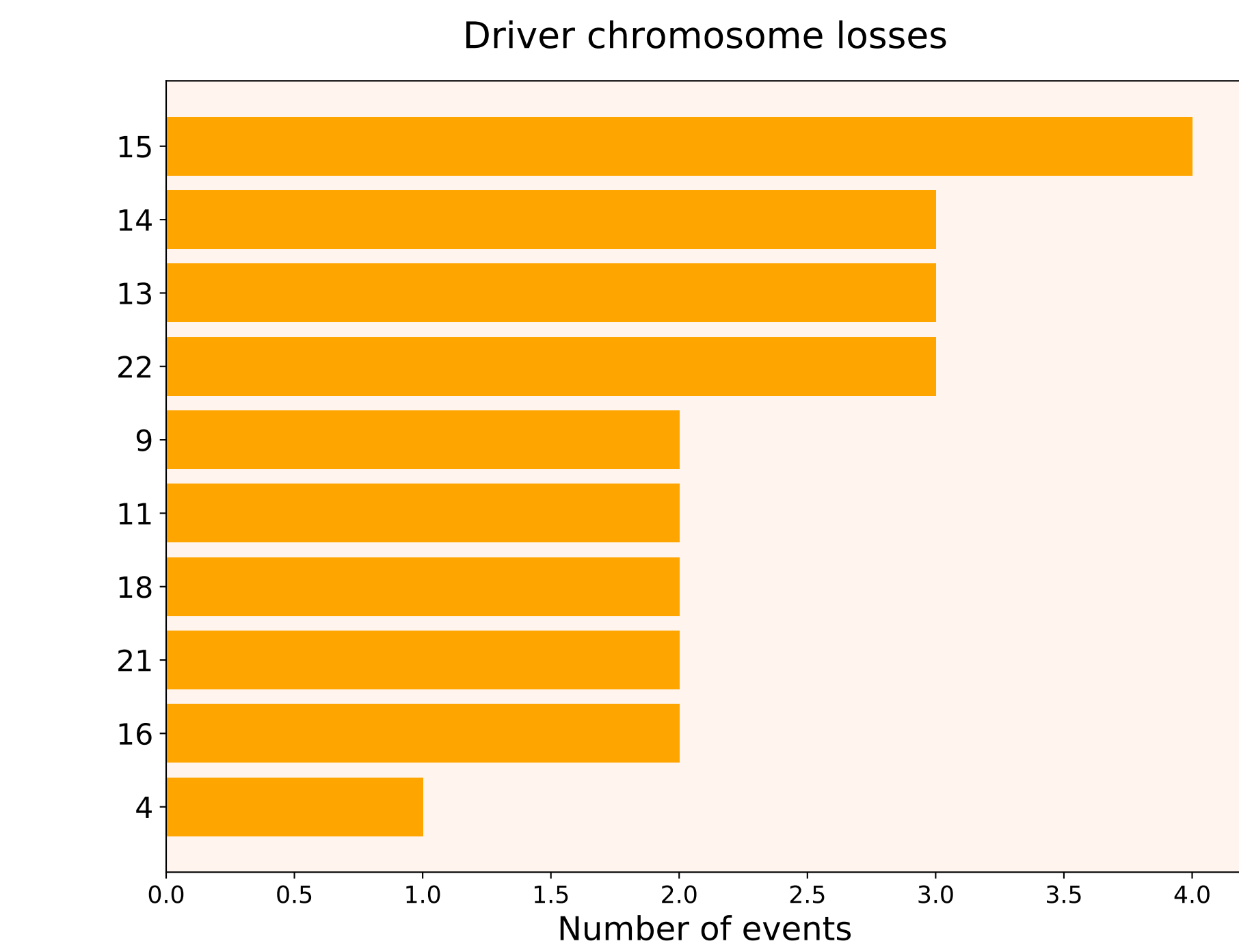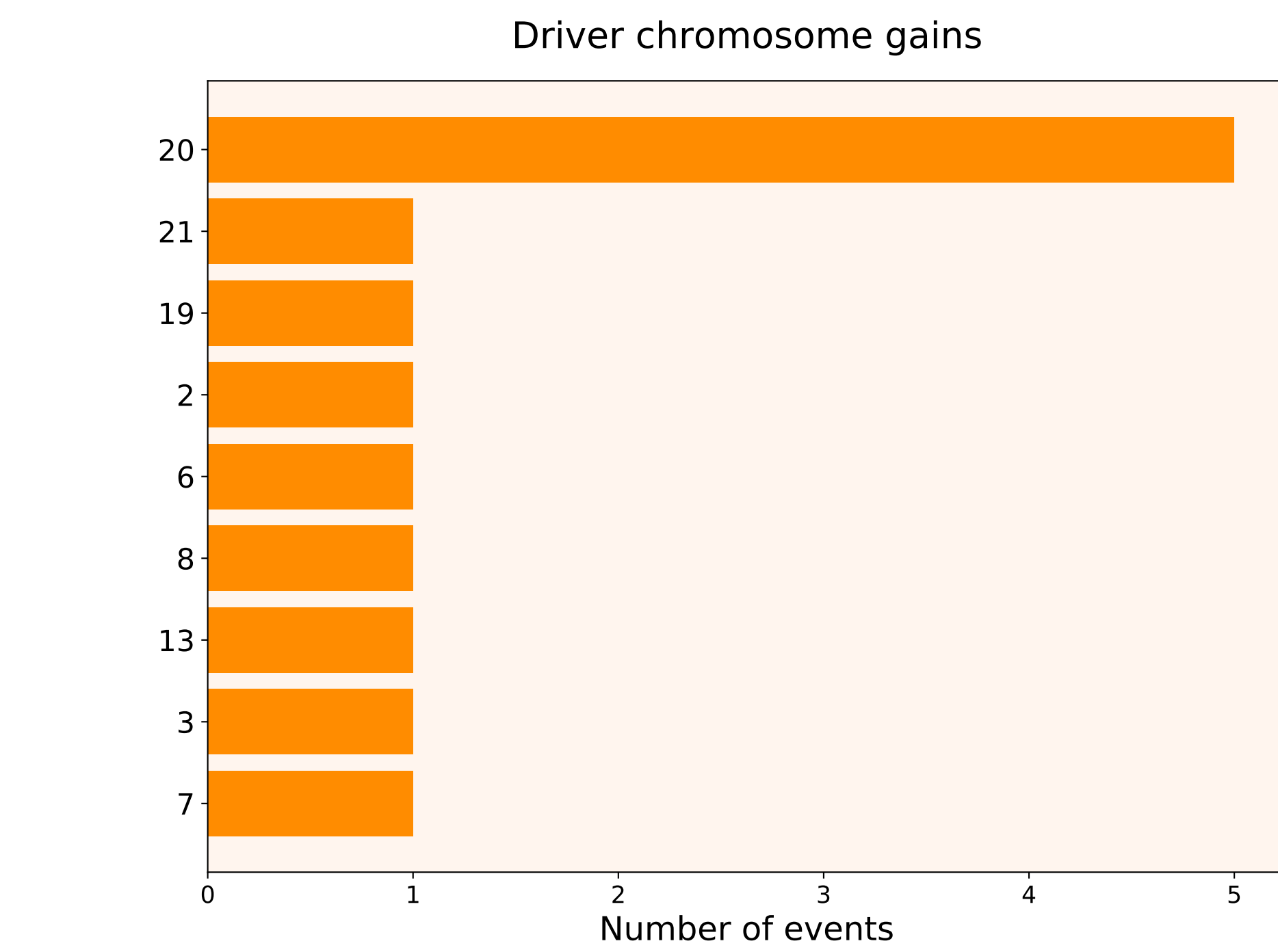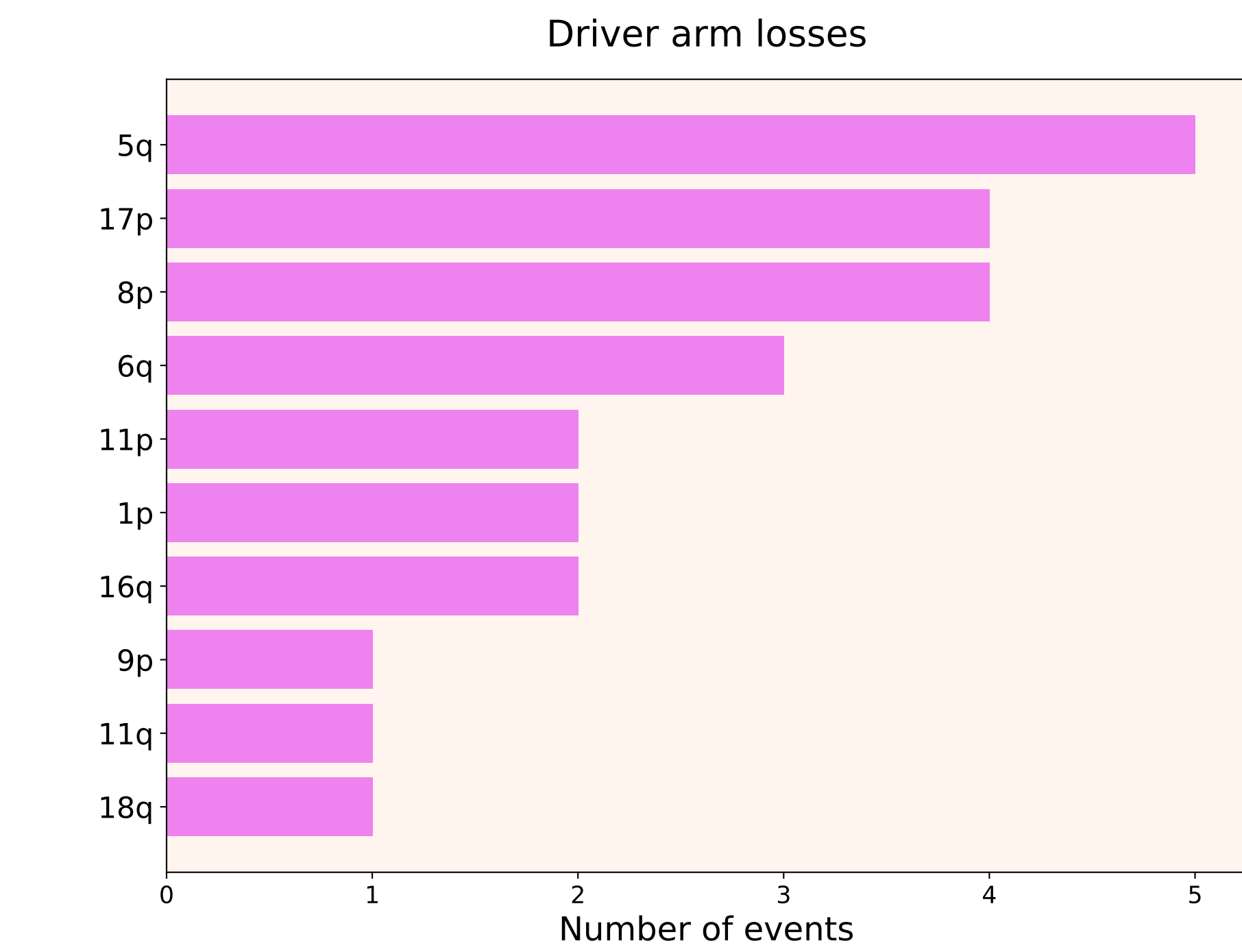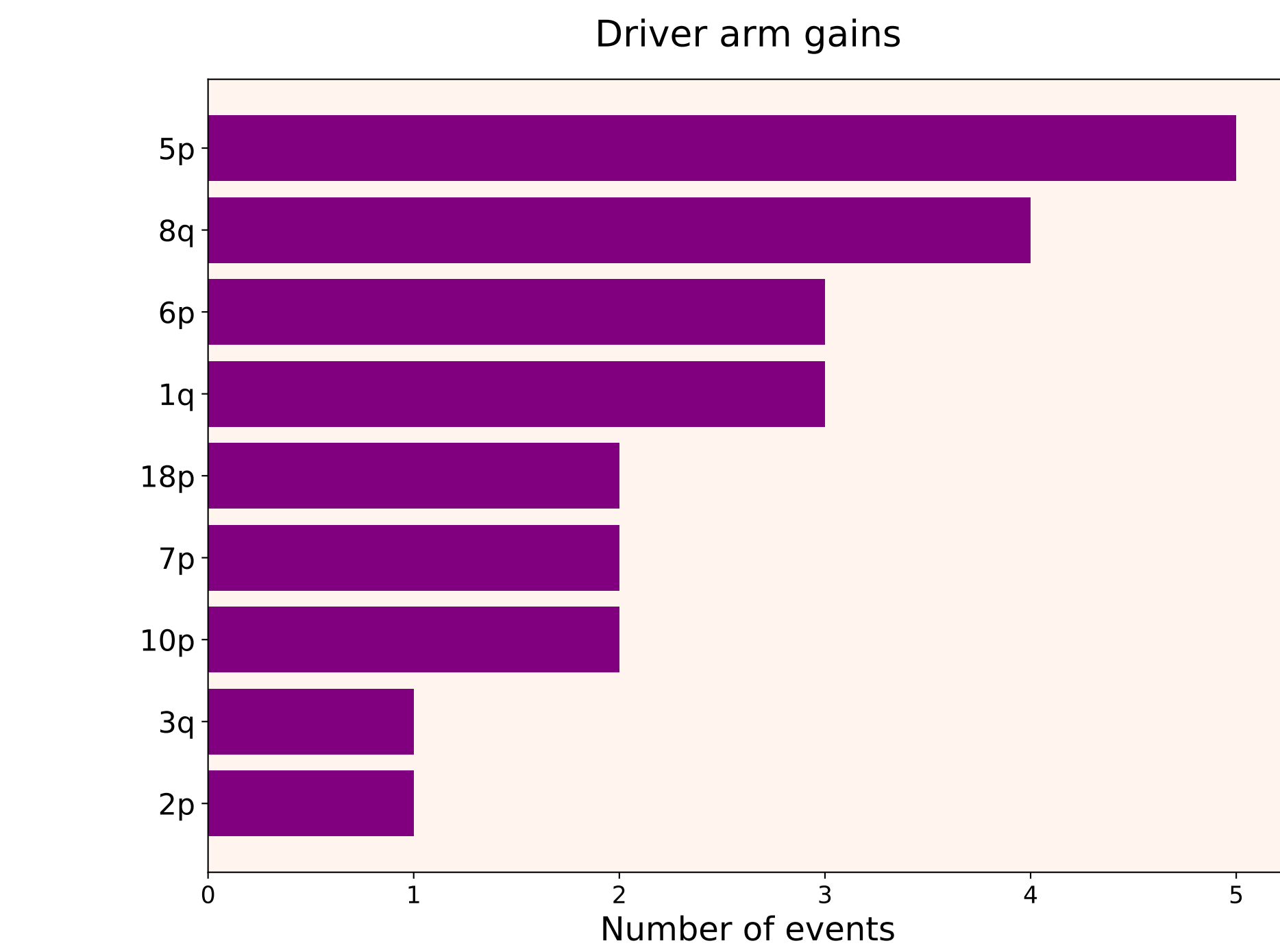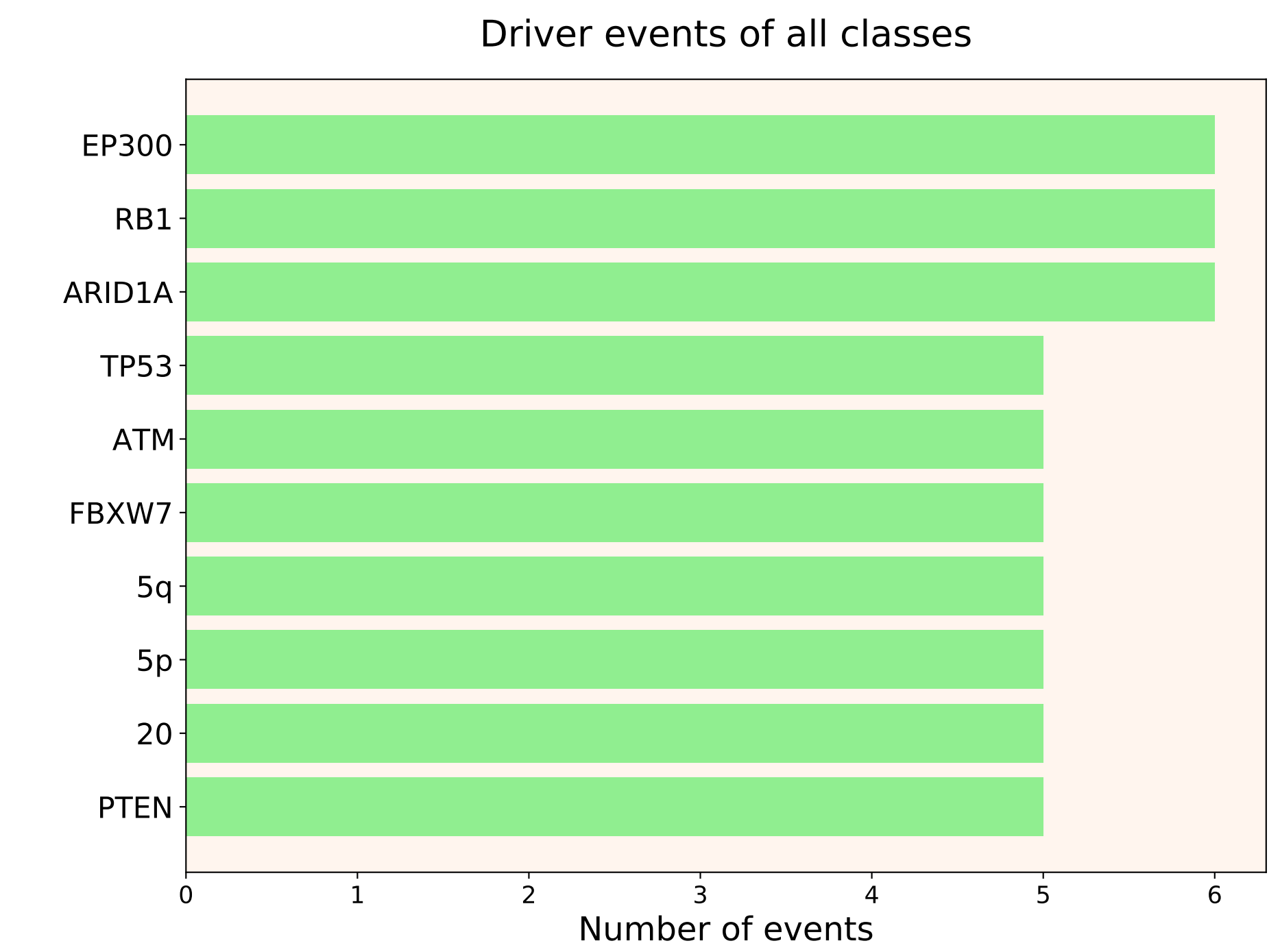

Supplement: Supplemental Information 2 [file peerj-10-13860-s002.zip › COHORTS/genes plots/detailed/2021_8_16_14_9_distribution_events_detailed_females_30.pdf]

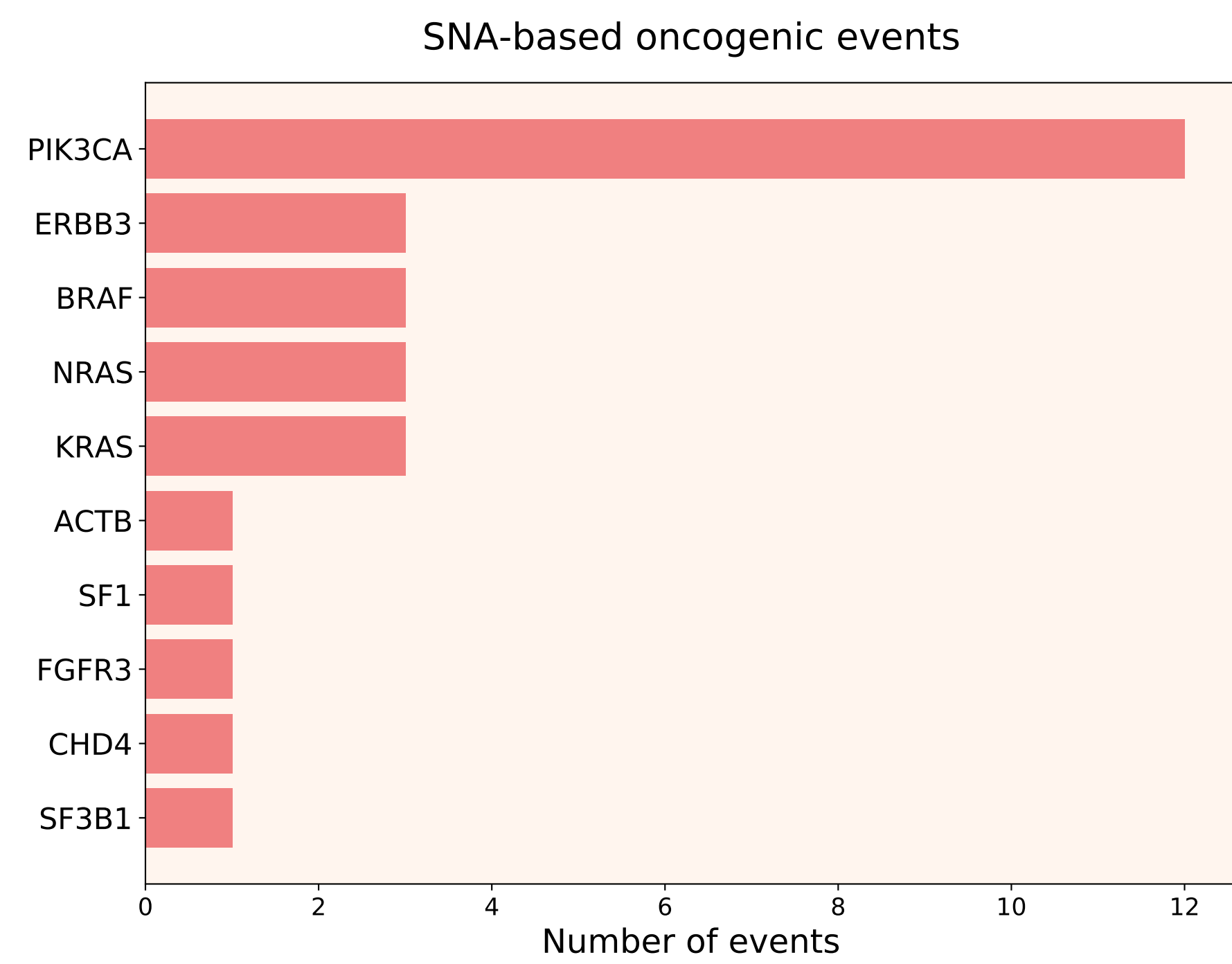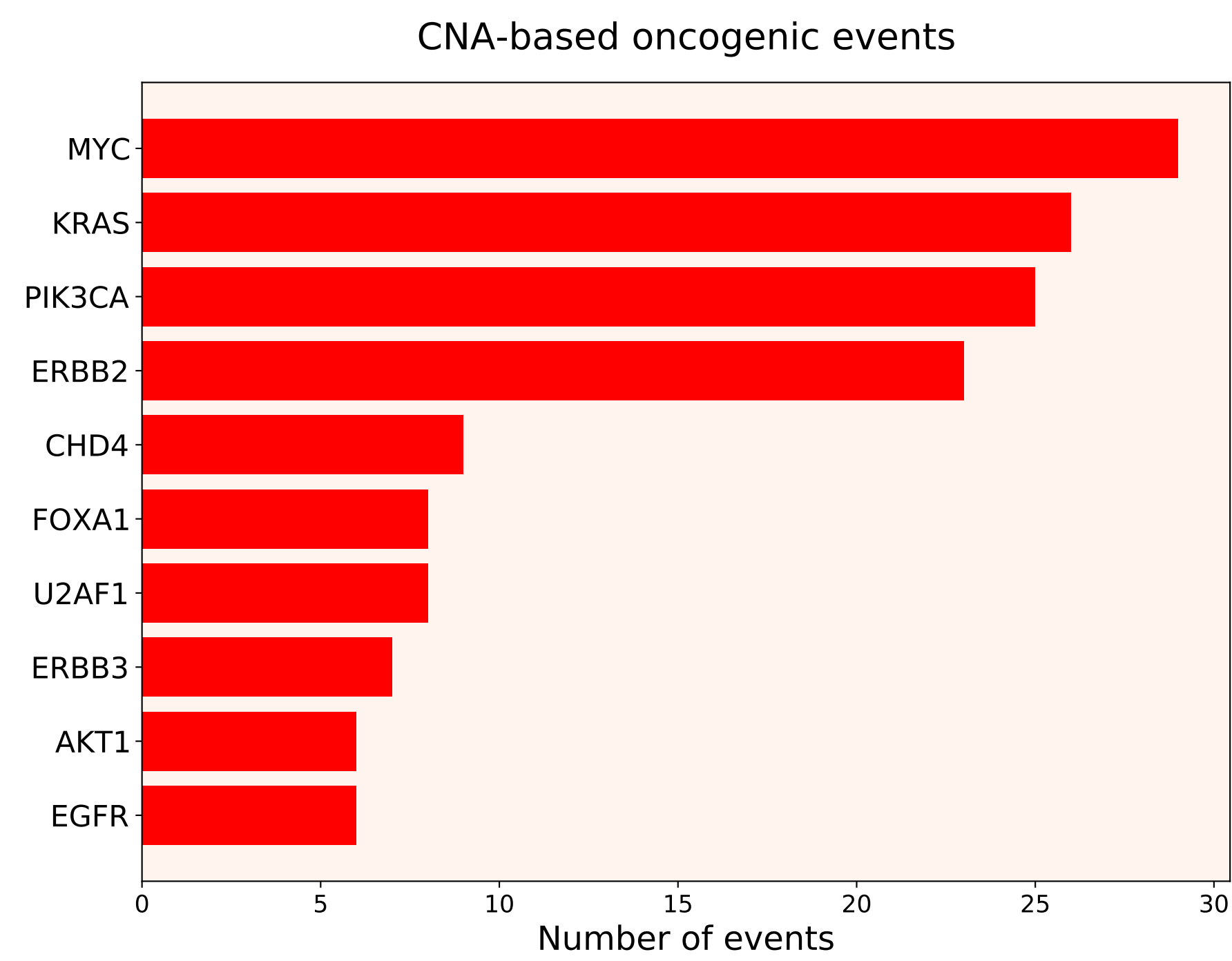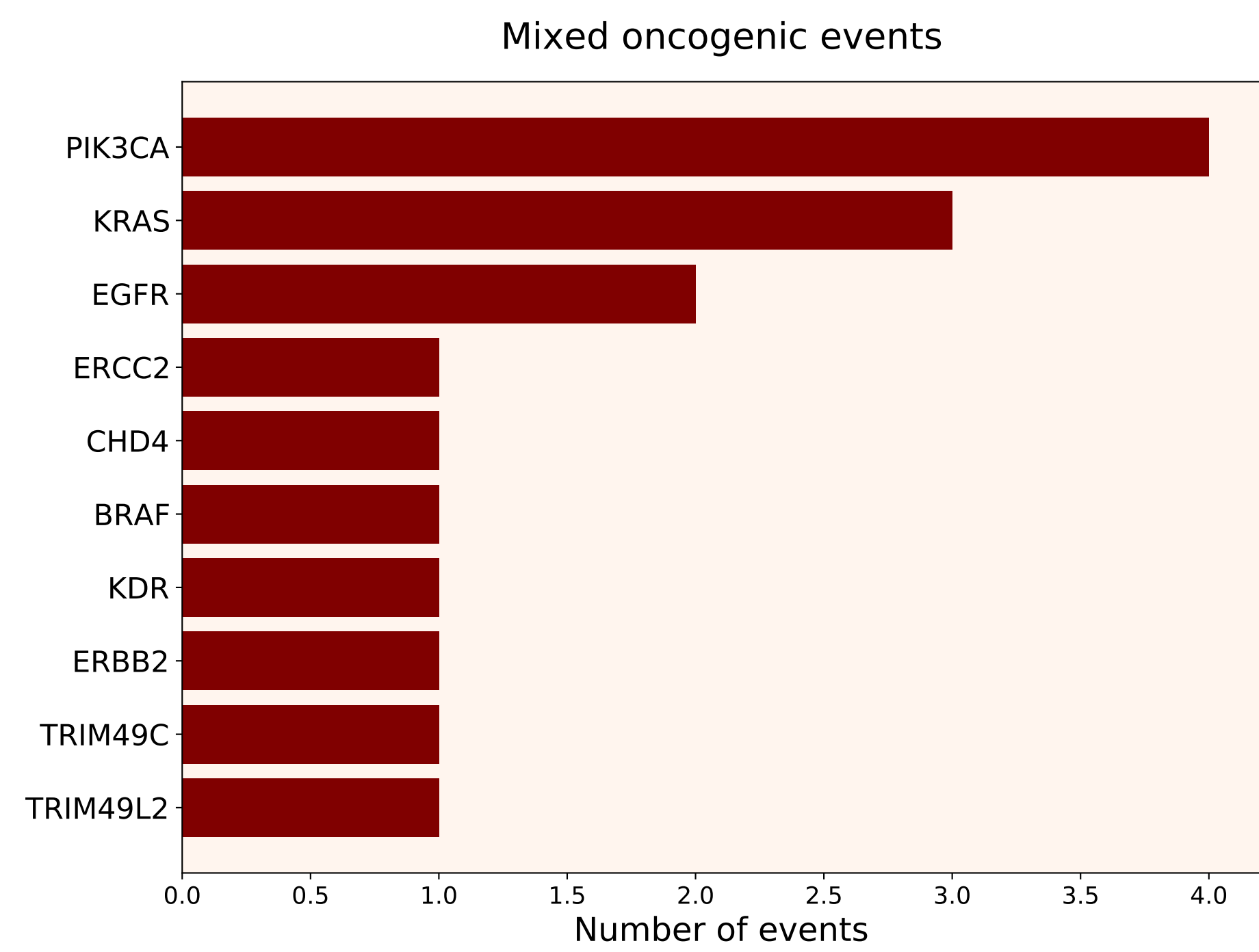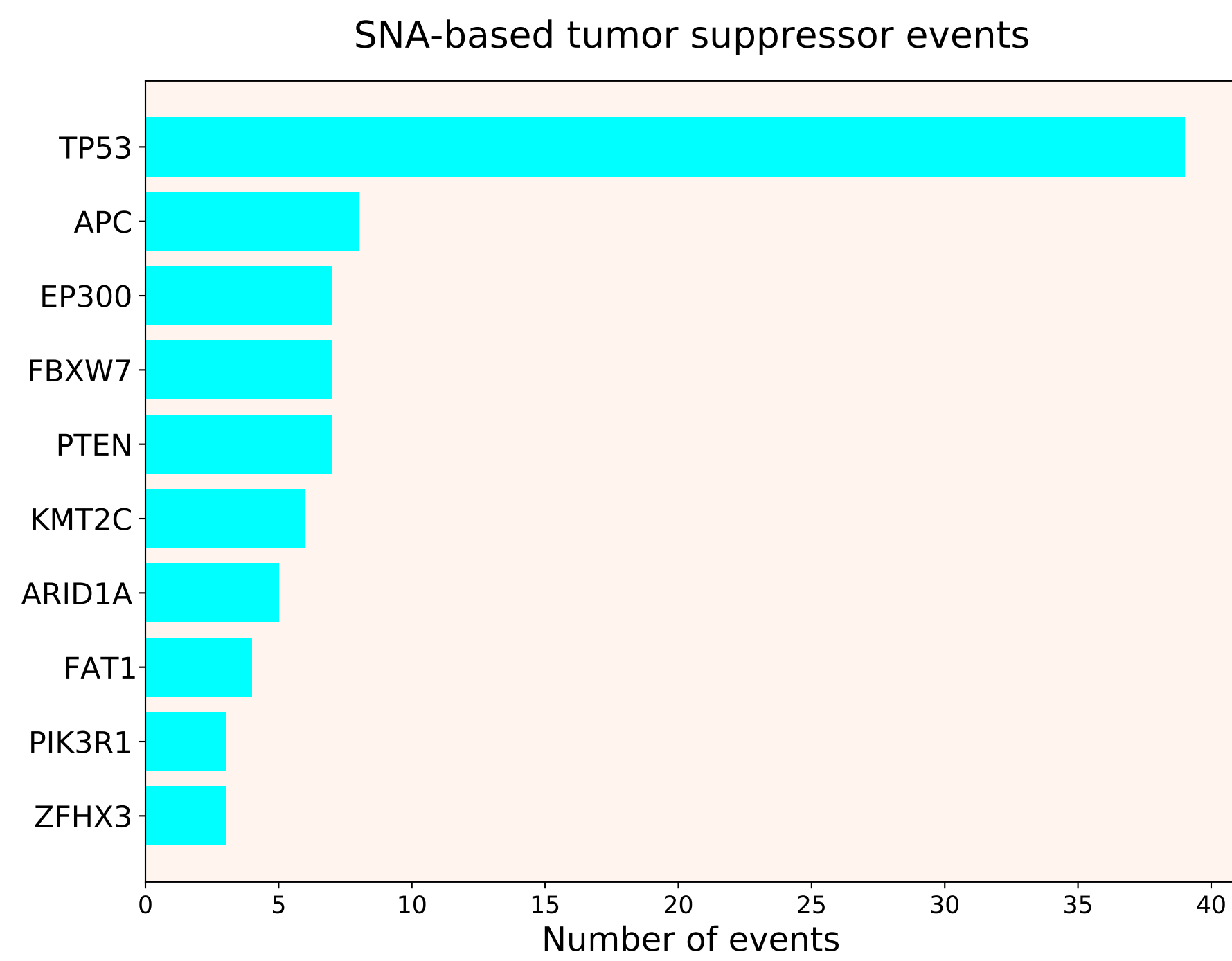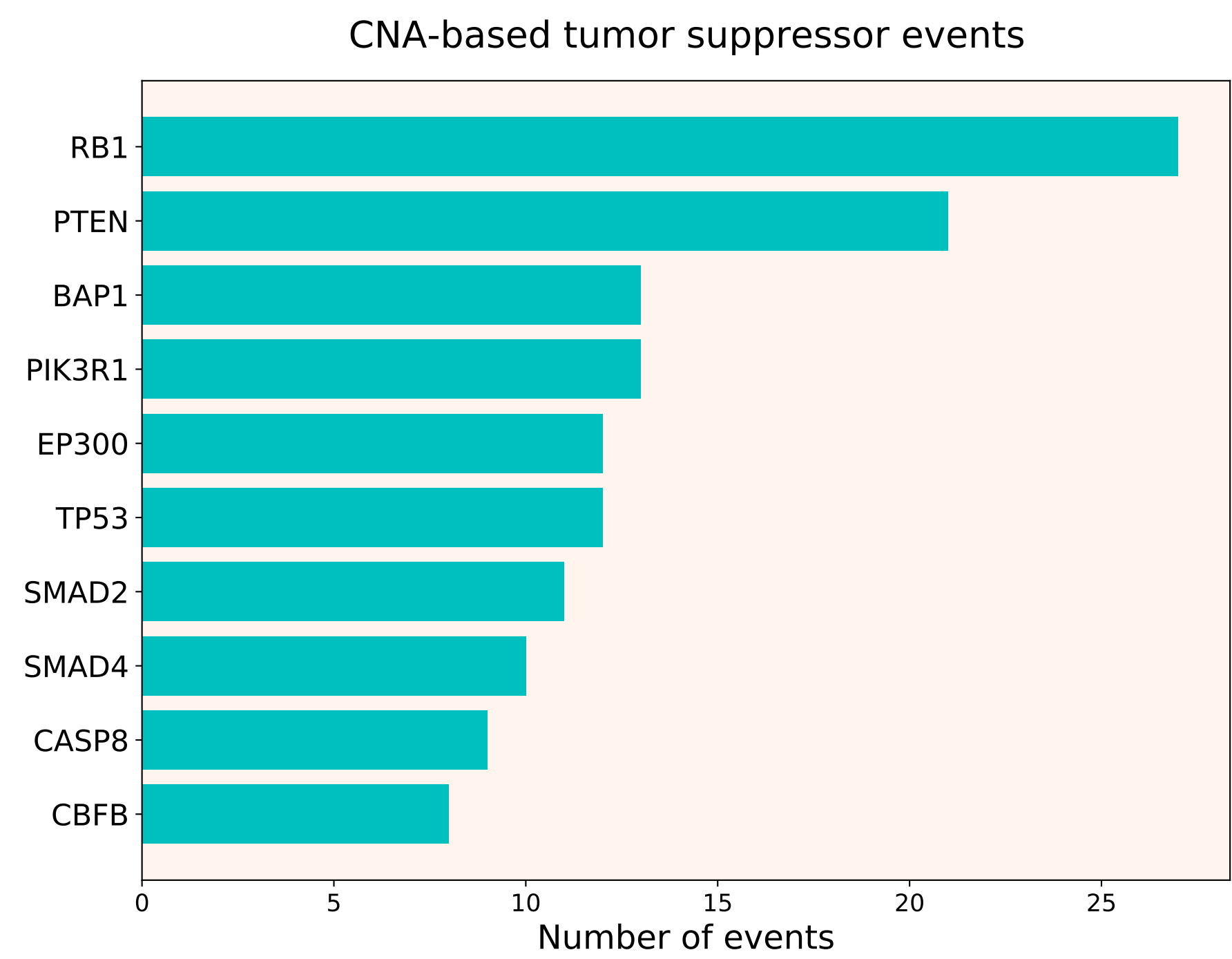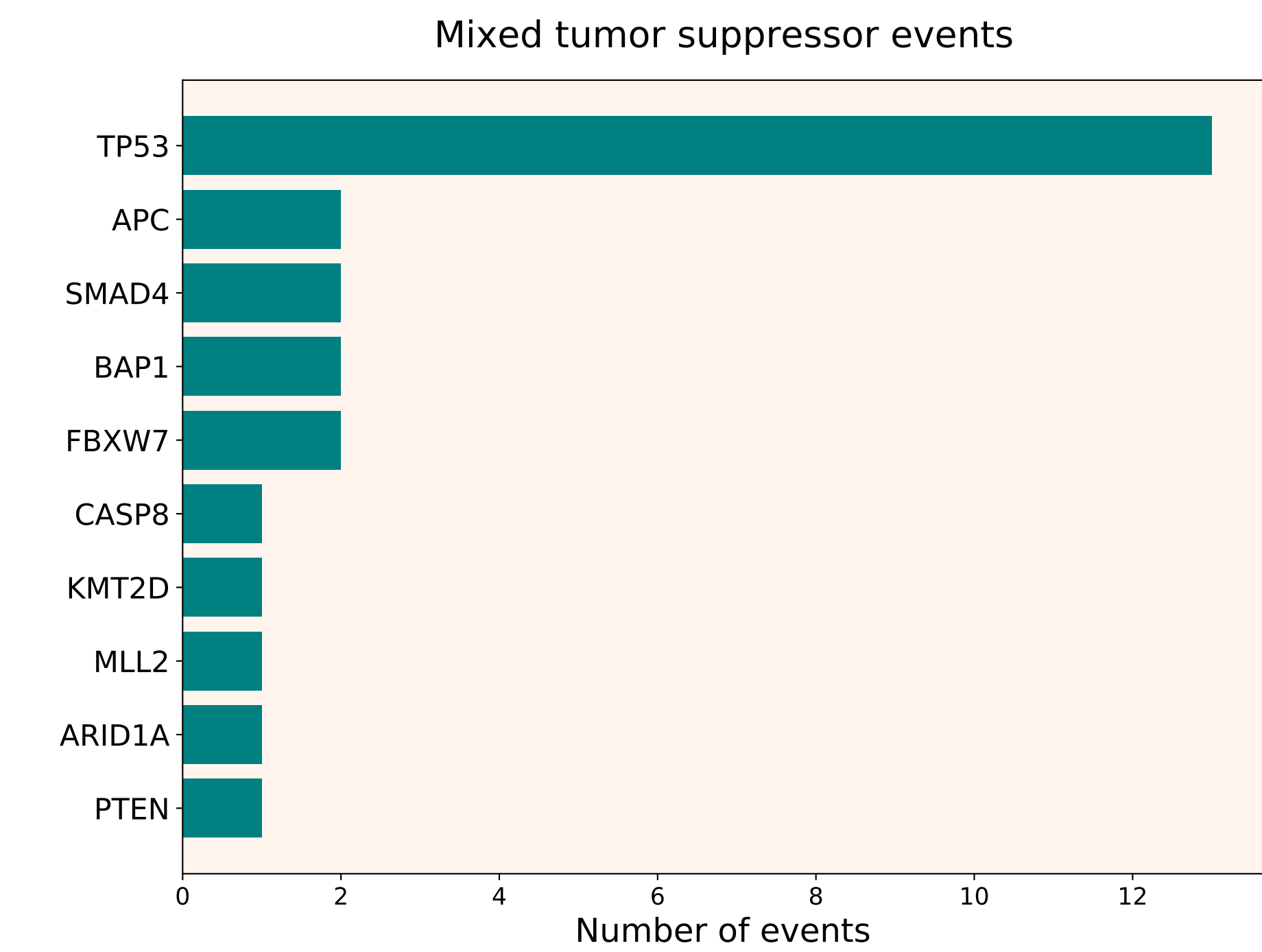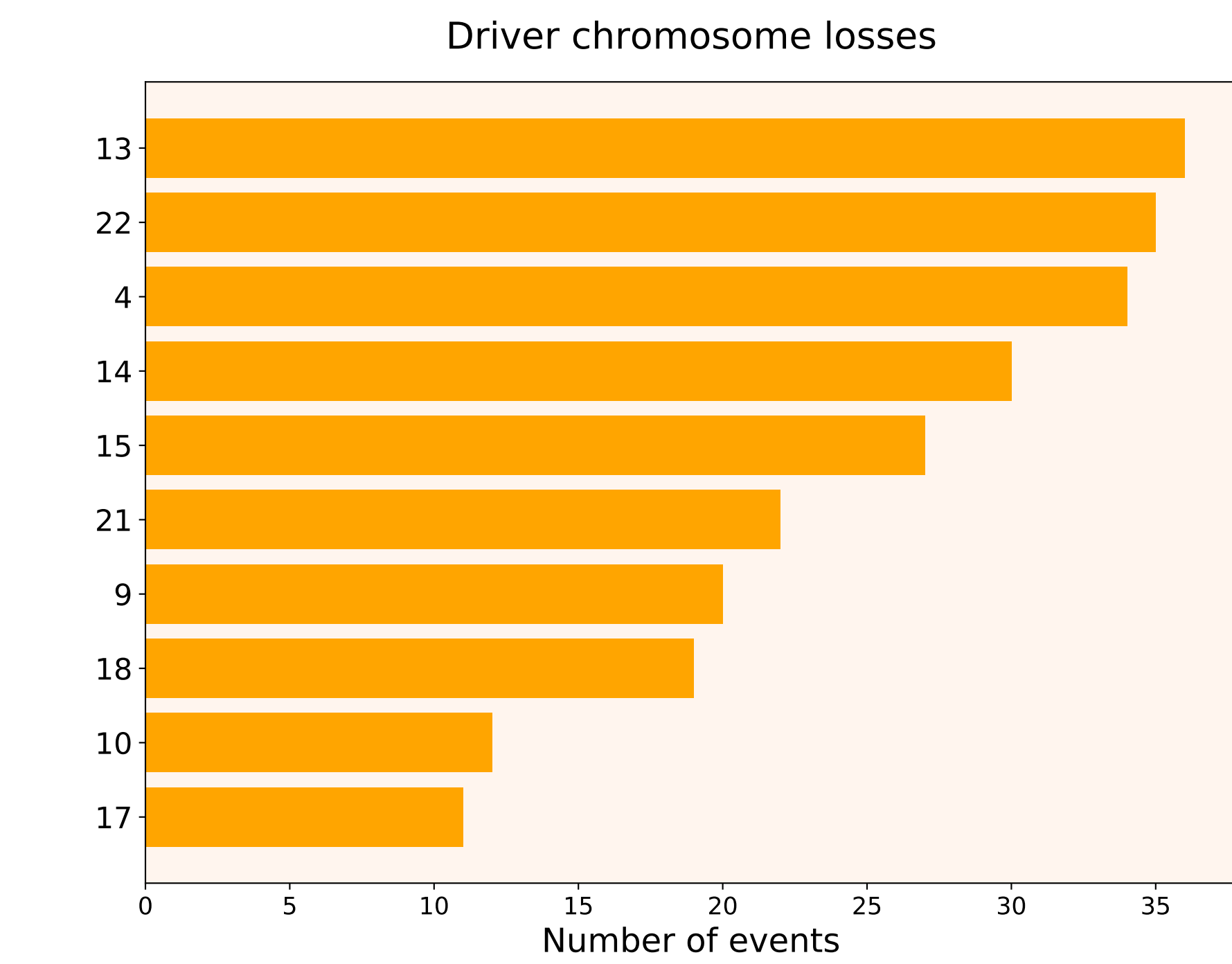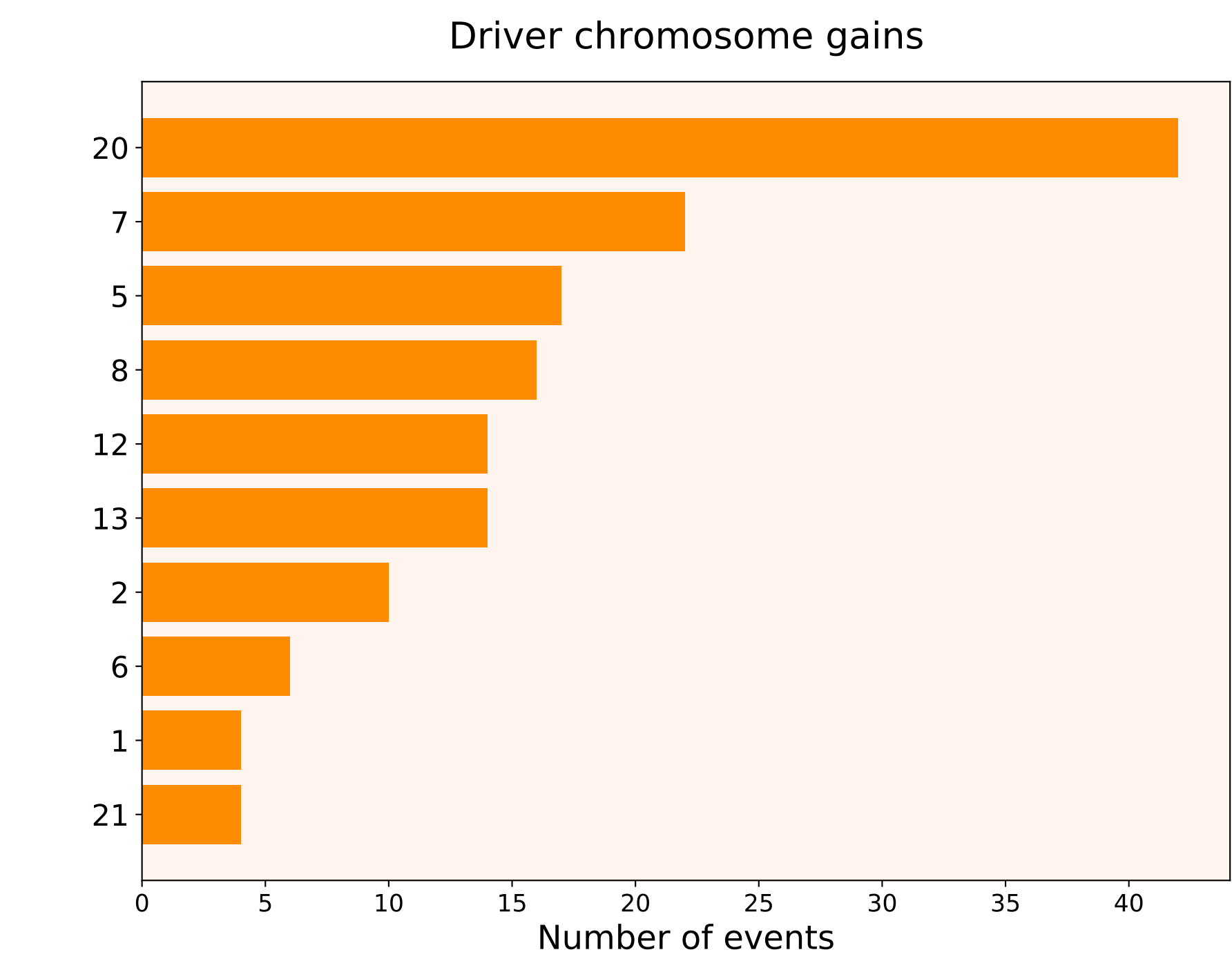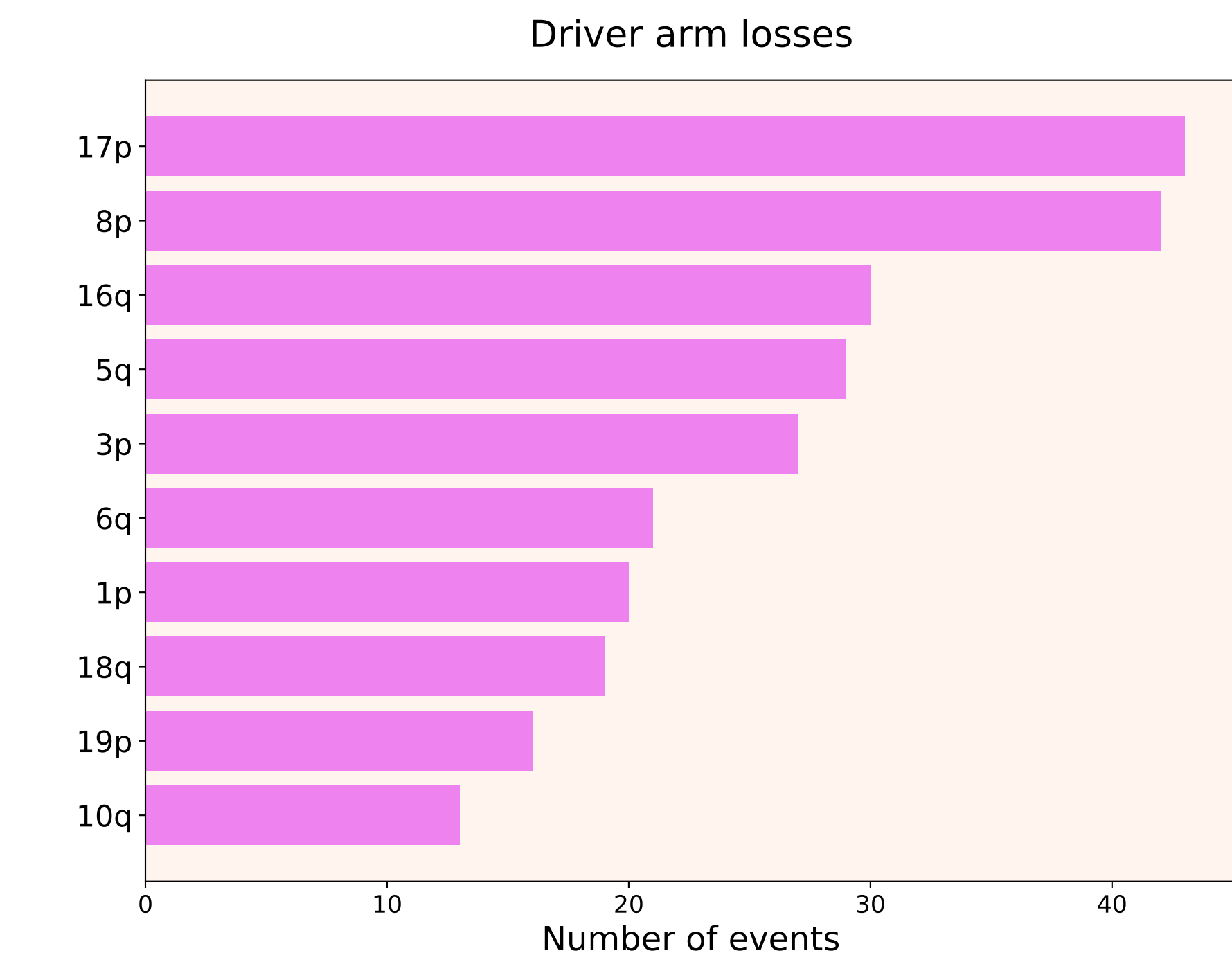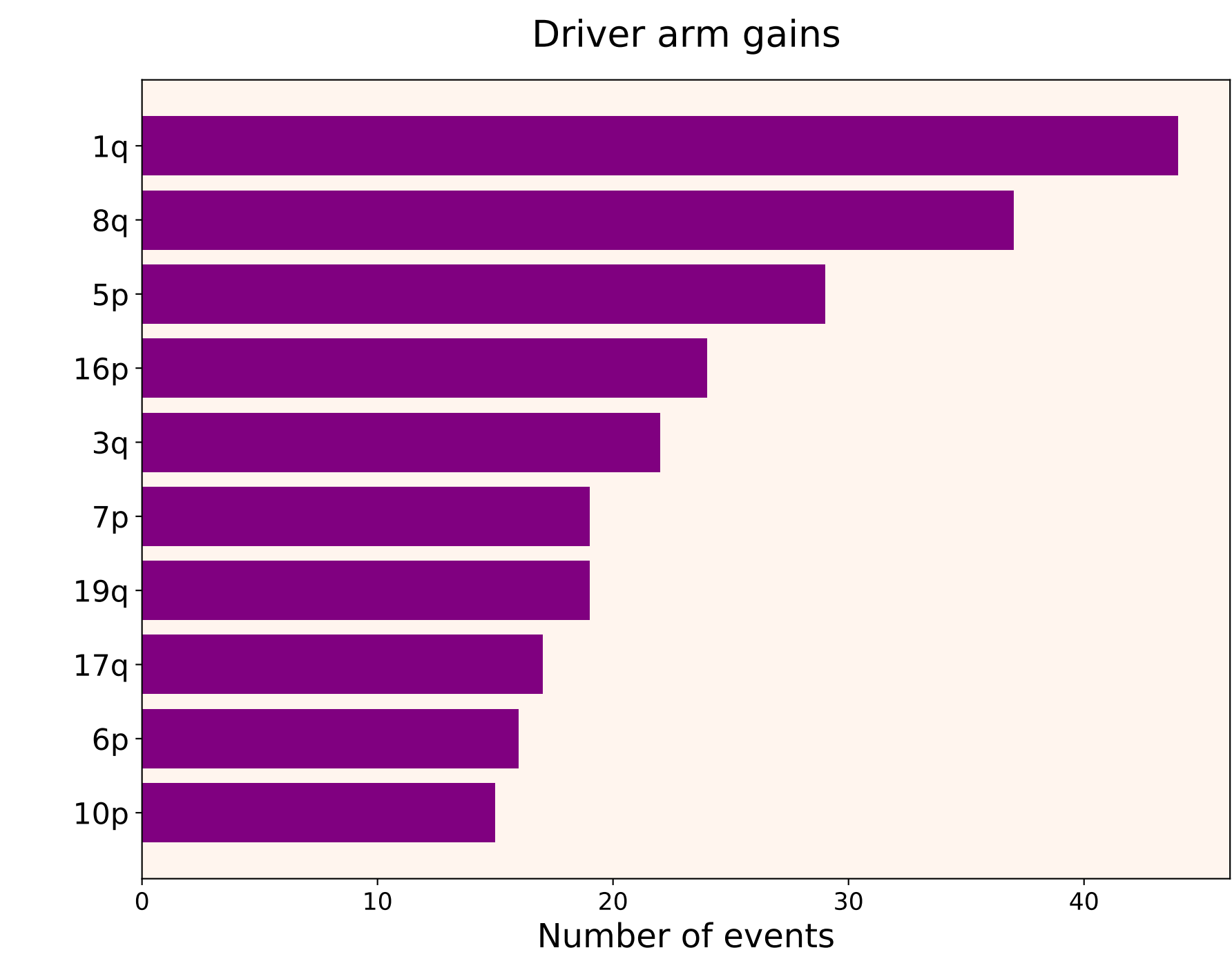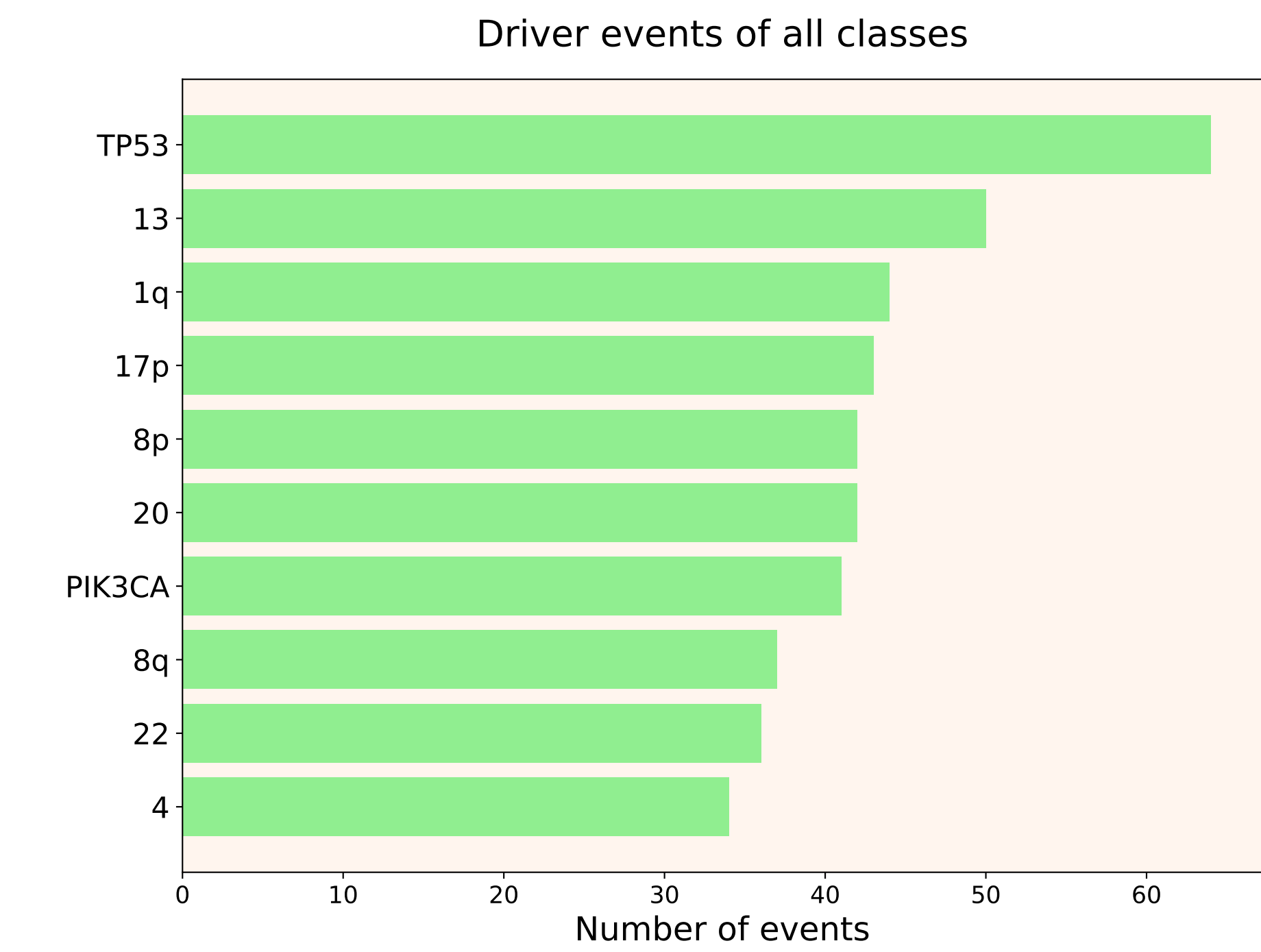

Supplement: Supplemental Information 2 [file peerj-10-13860-s002.zip › COHORTS/genes plots/detailed/2021_8_16_14_9_distribution_events_detailed_females_18.pdf]

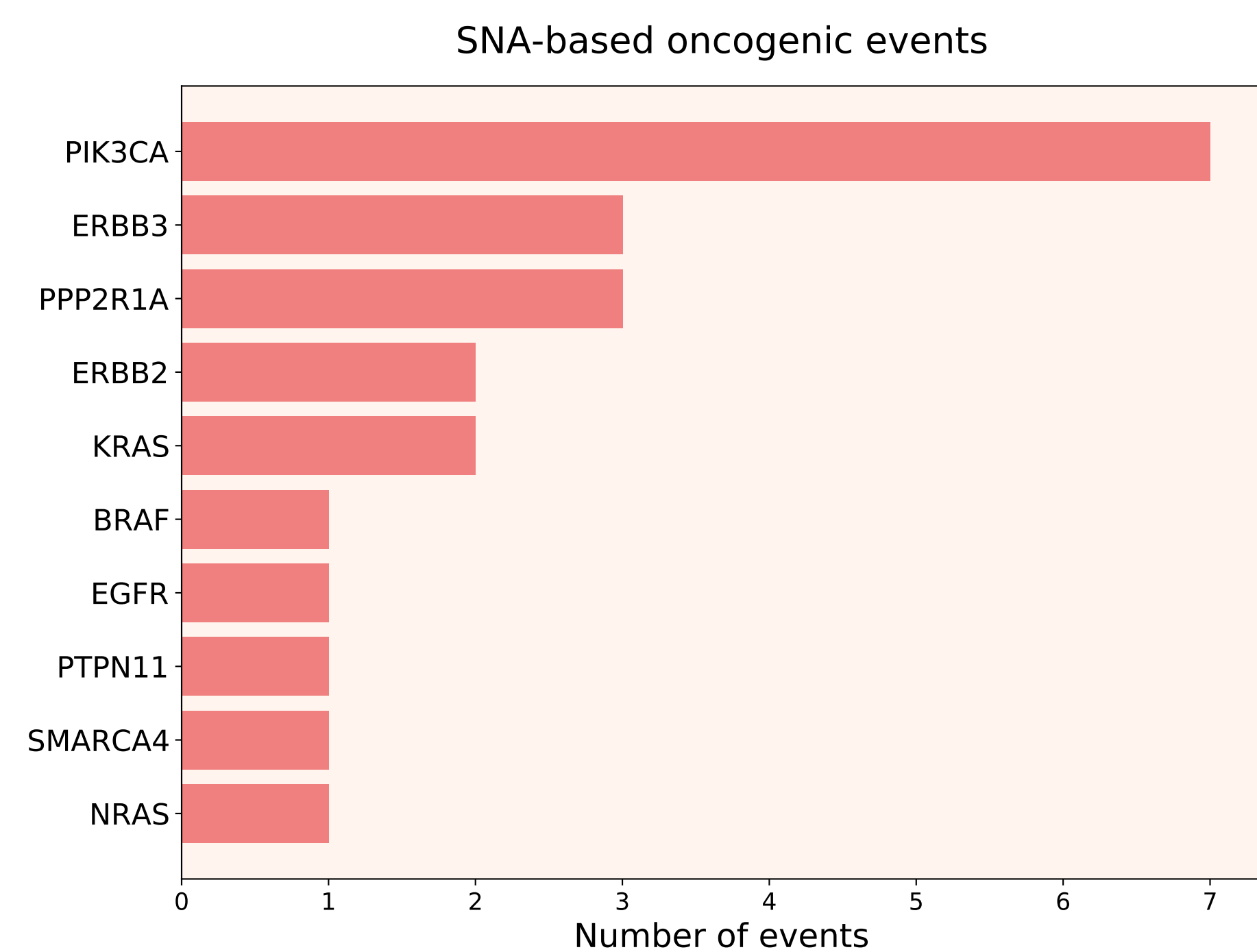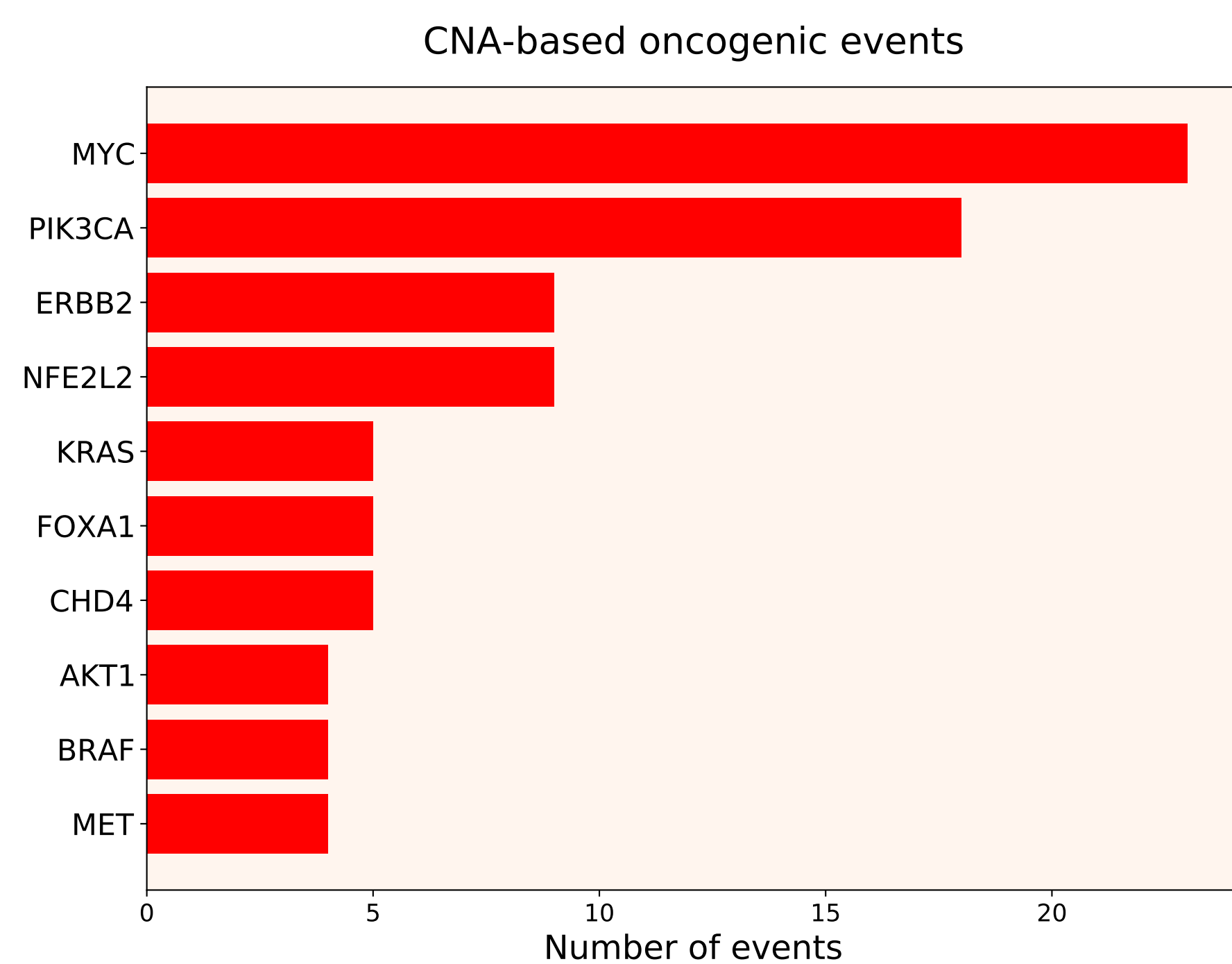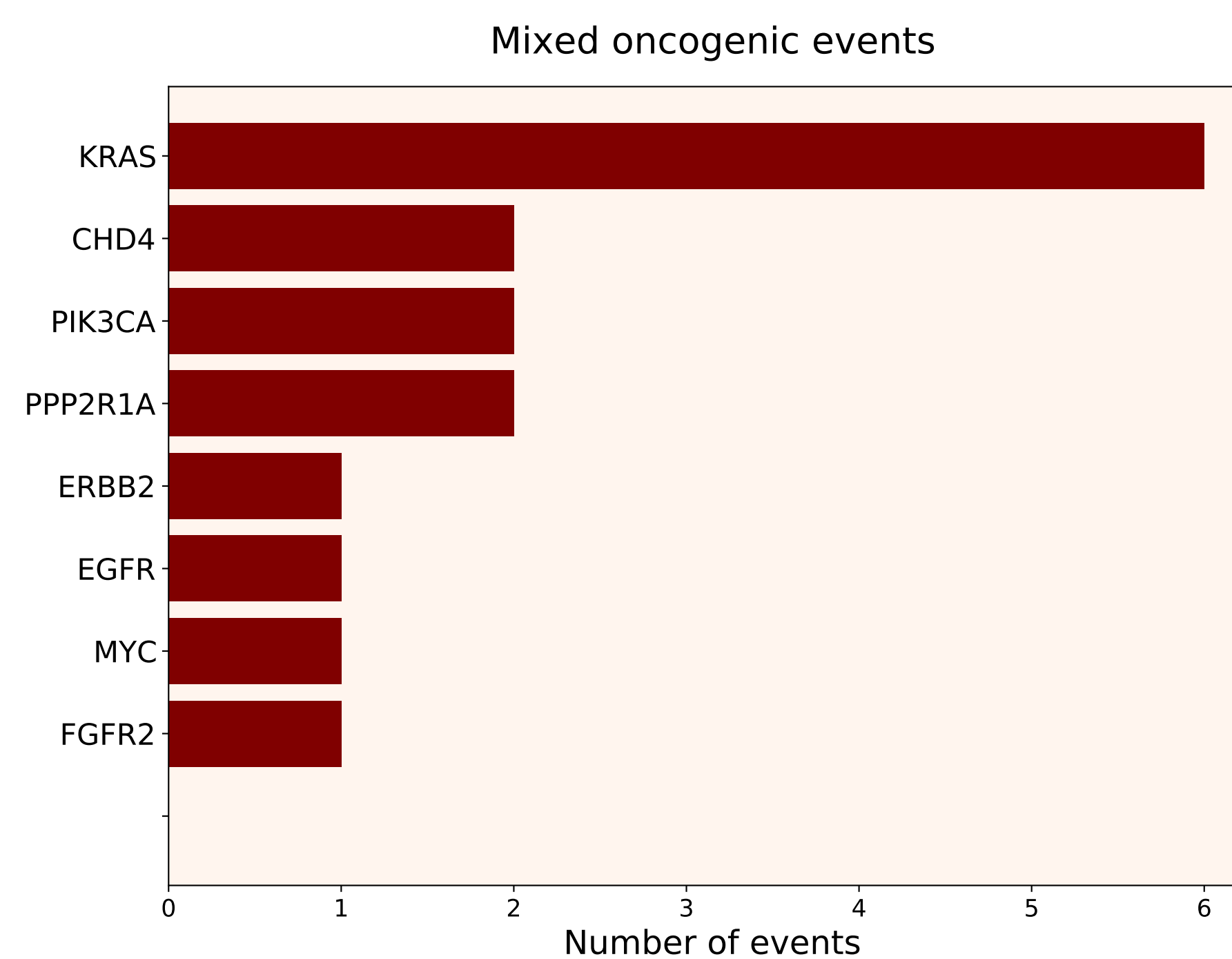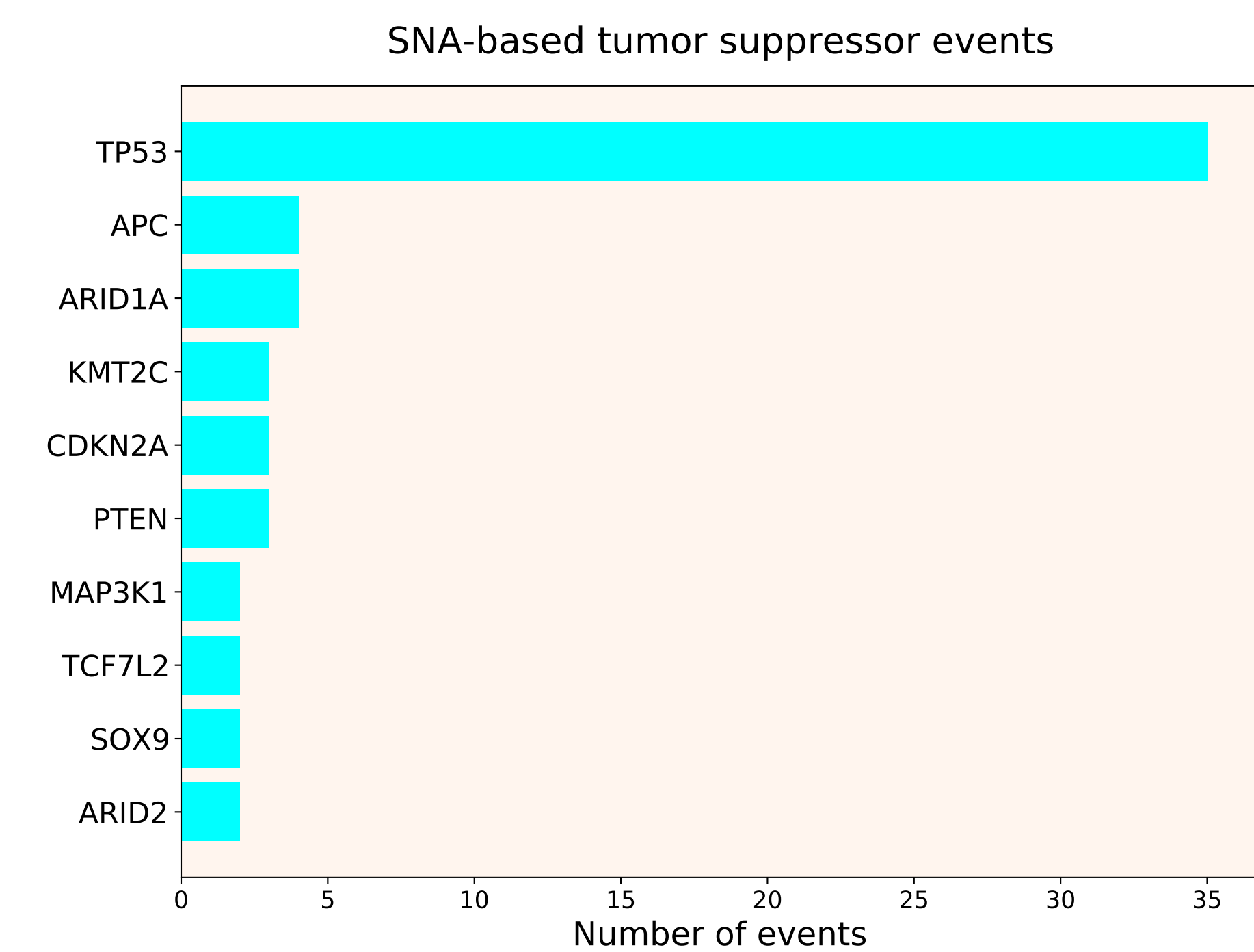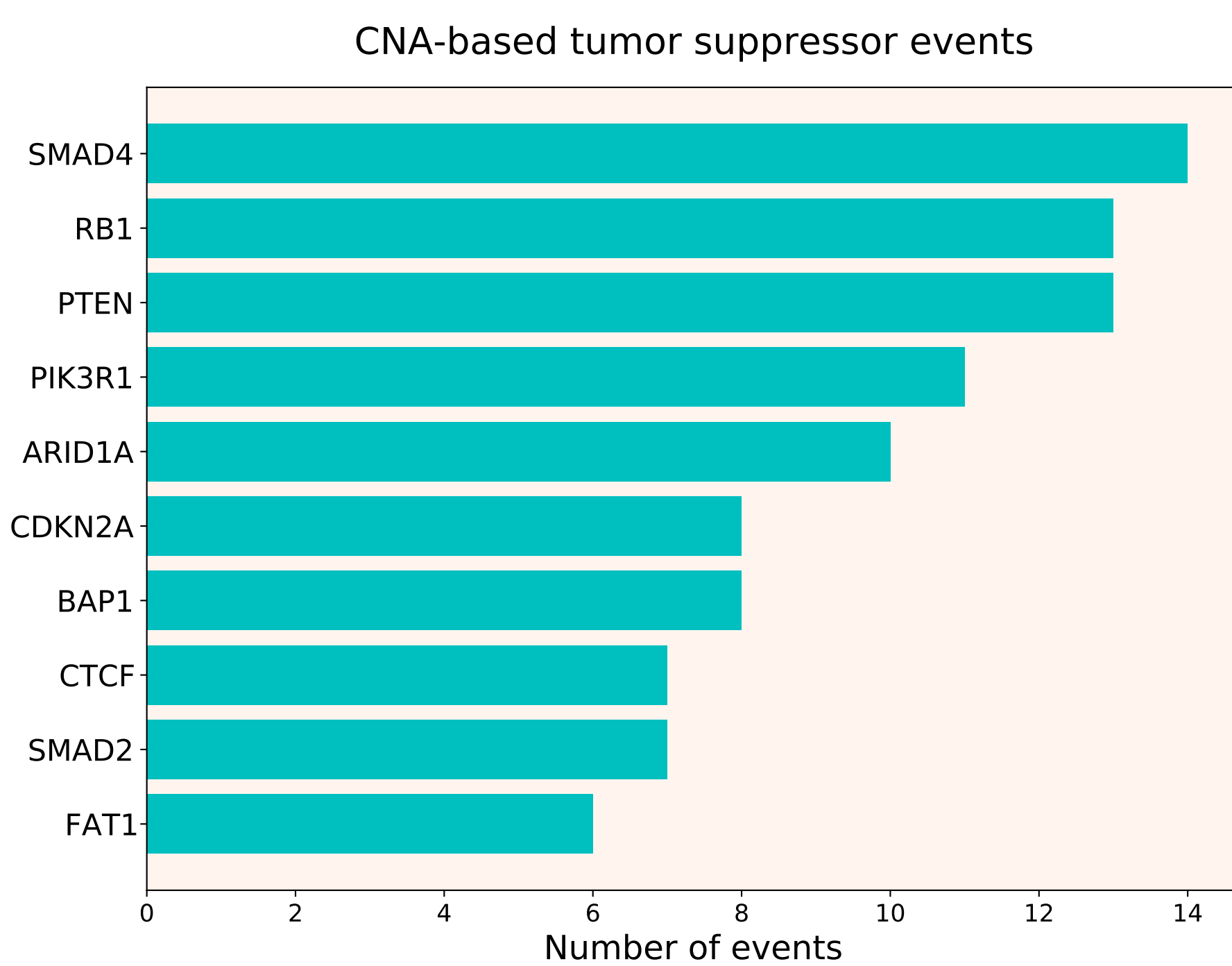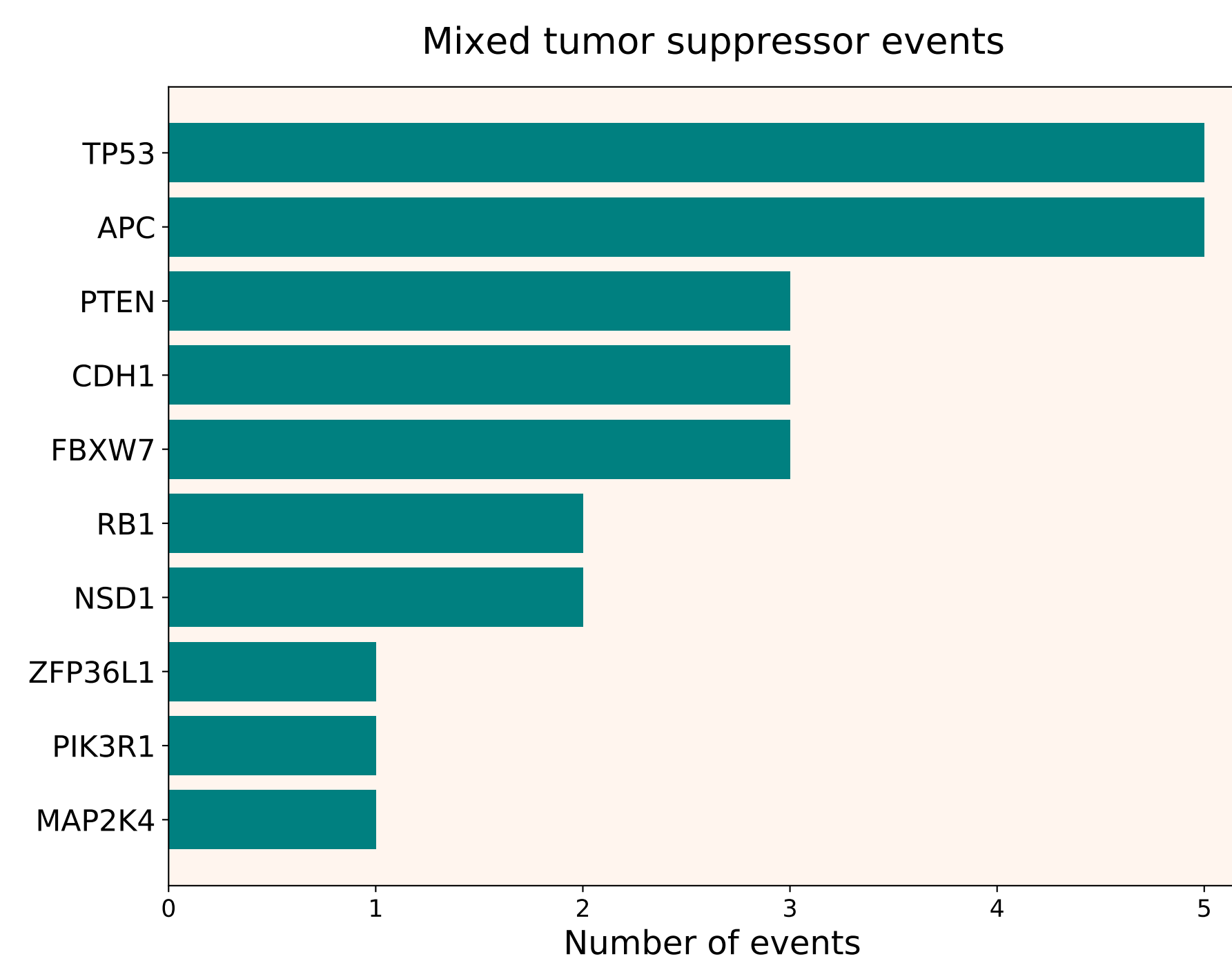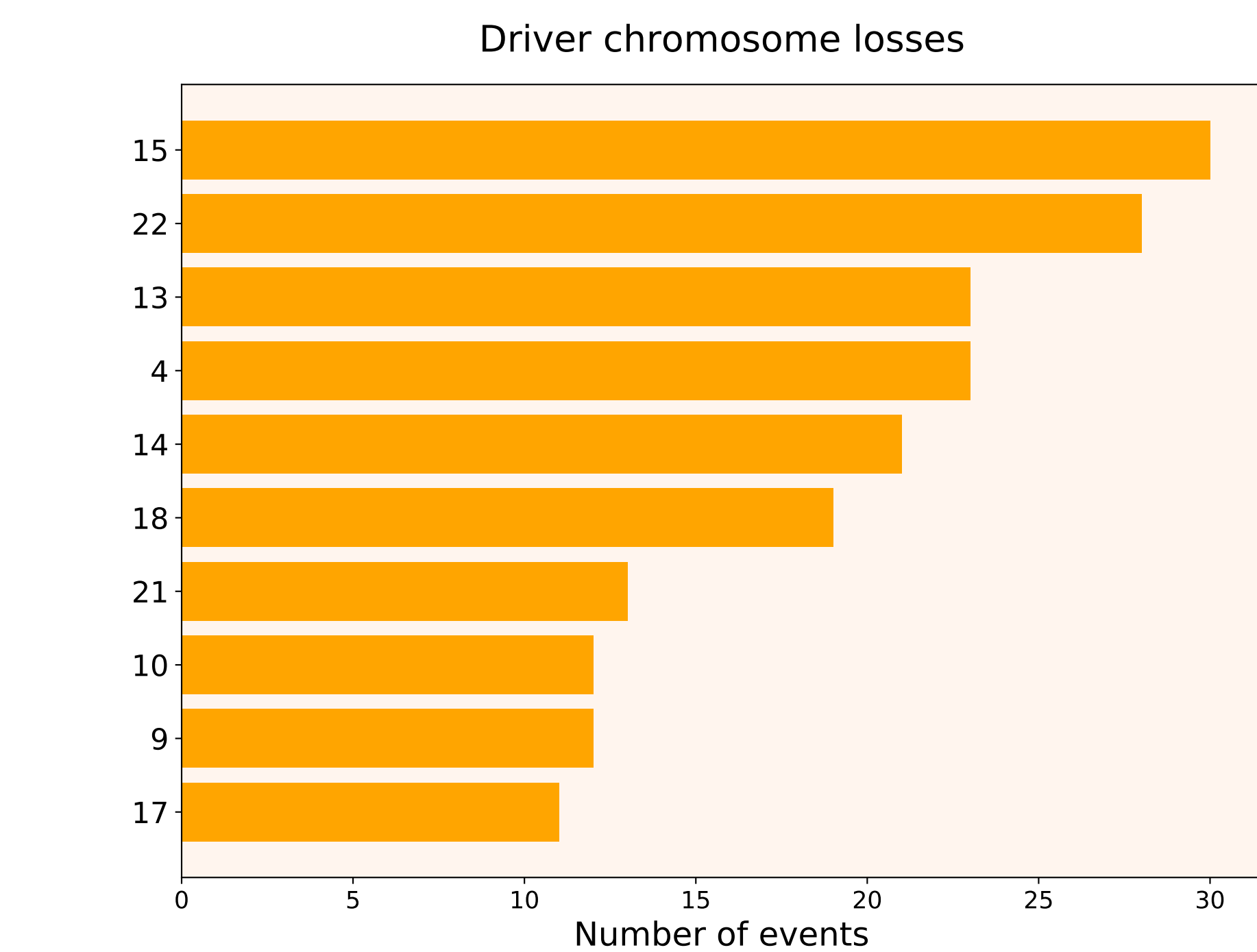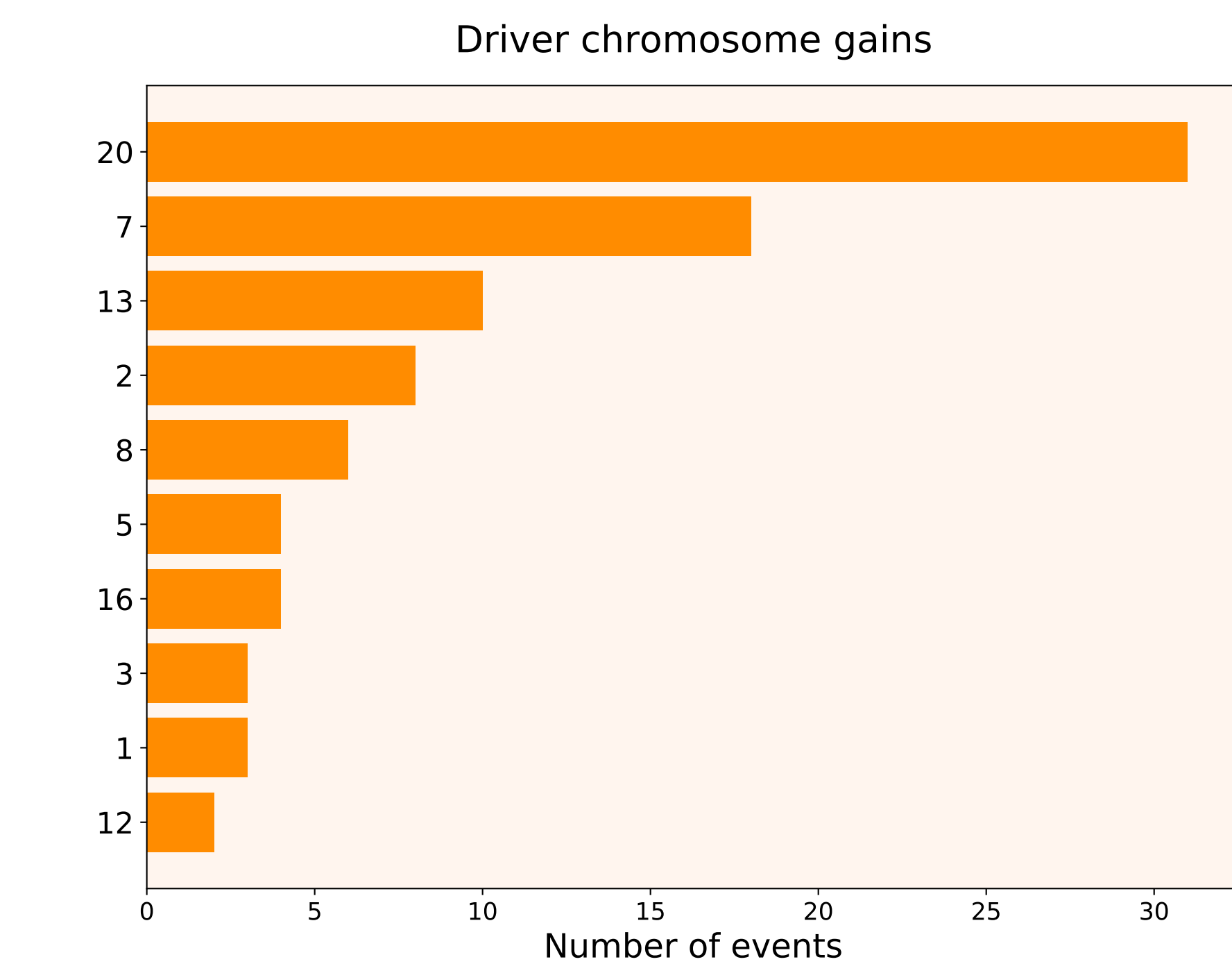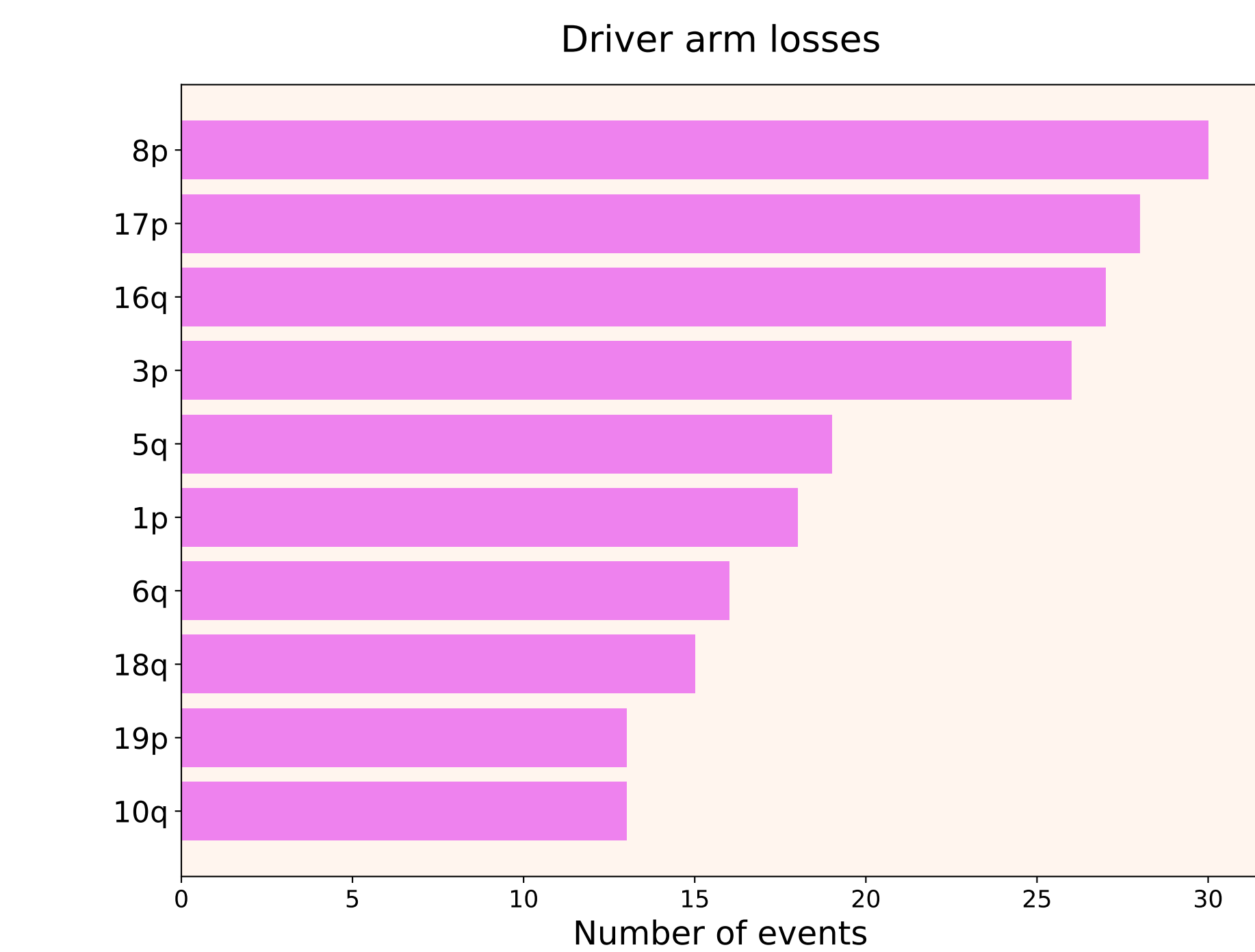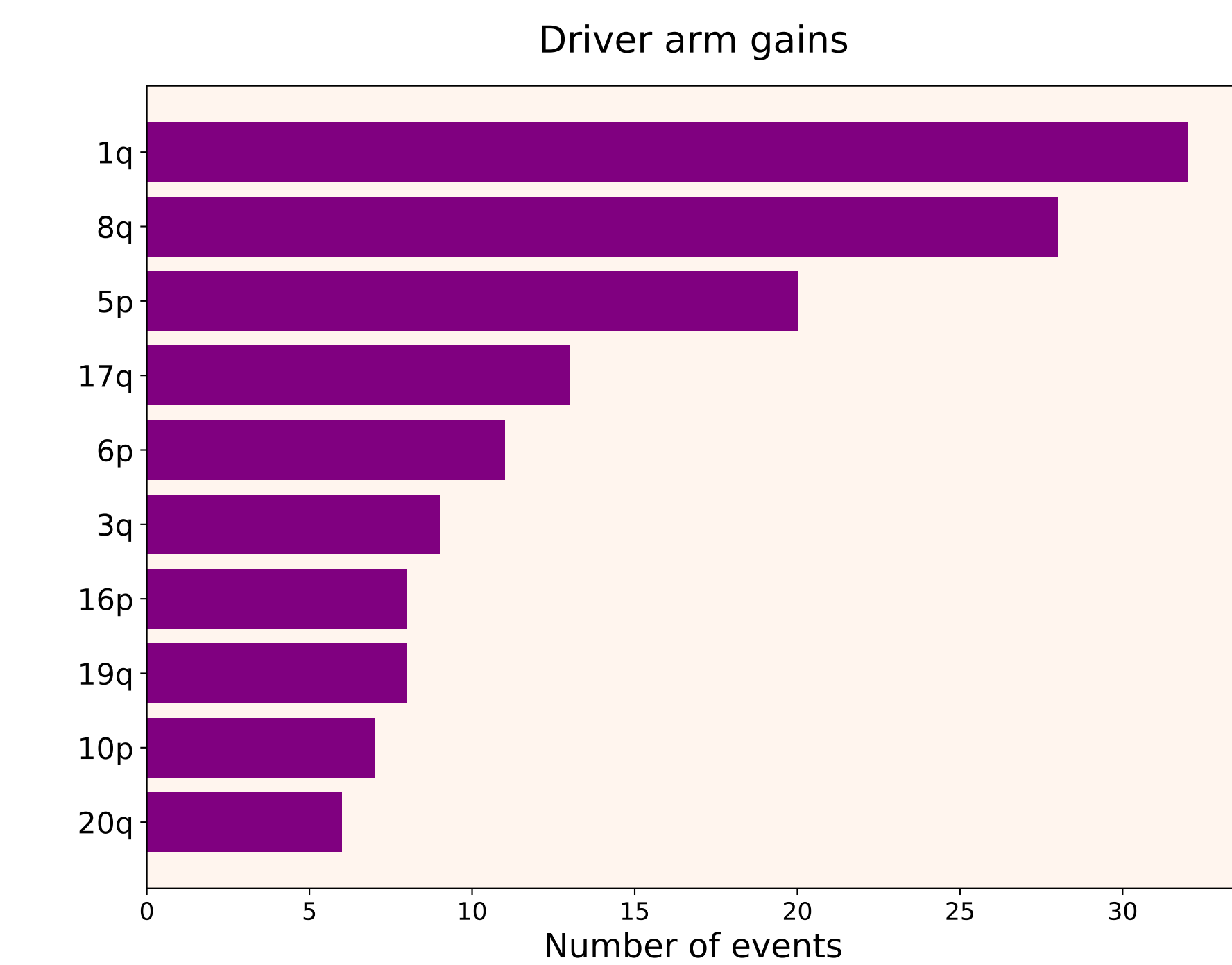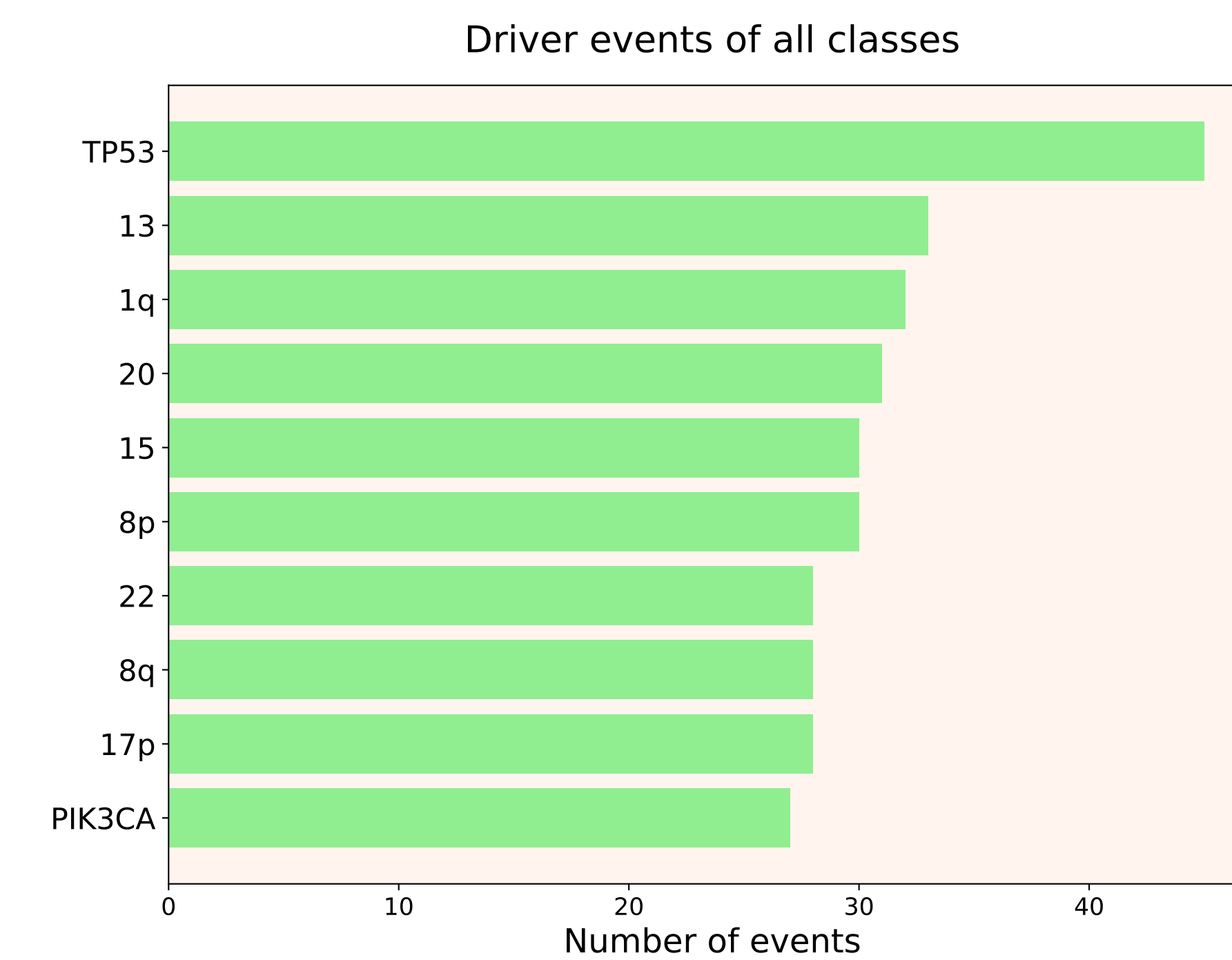

Supplement: Supplemental Information 2 [file peerj-10-13860-s002.zip › COHORTS/genes plots/detailed/2021_8_16_14_9_distribution_events_detailed_females_19.pdf]

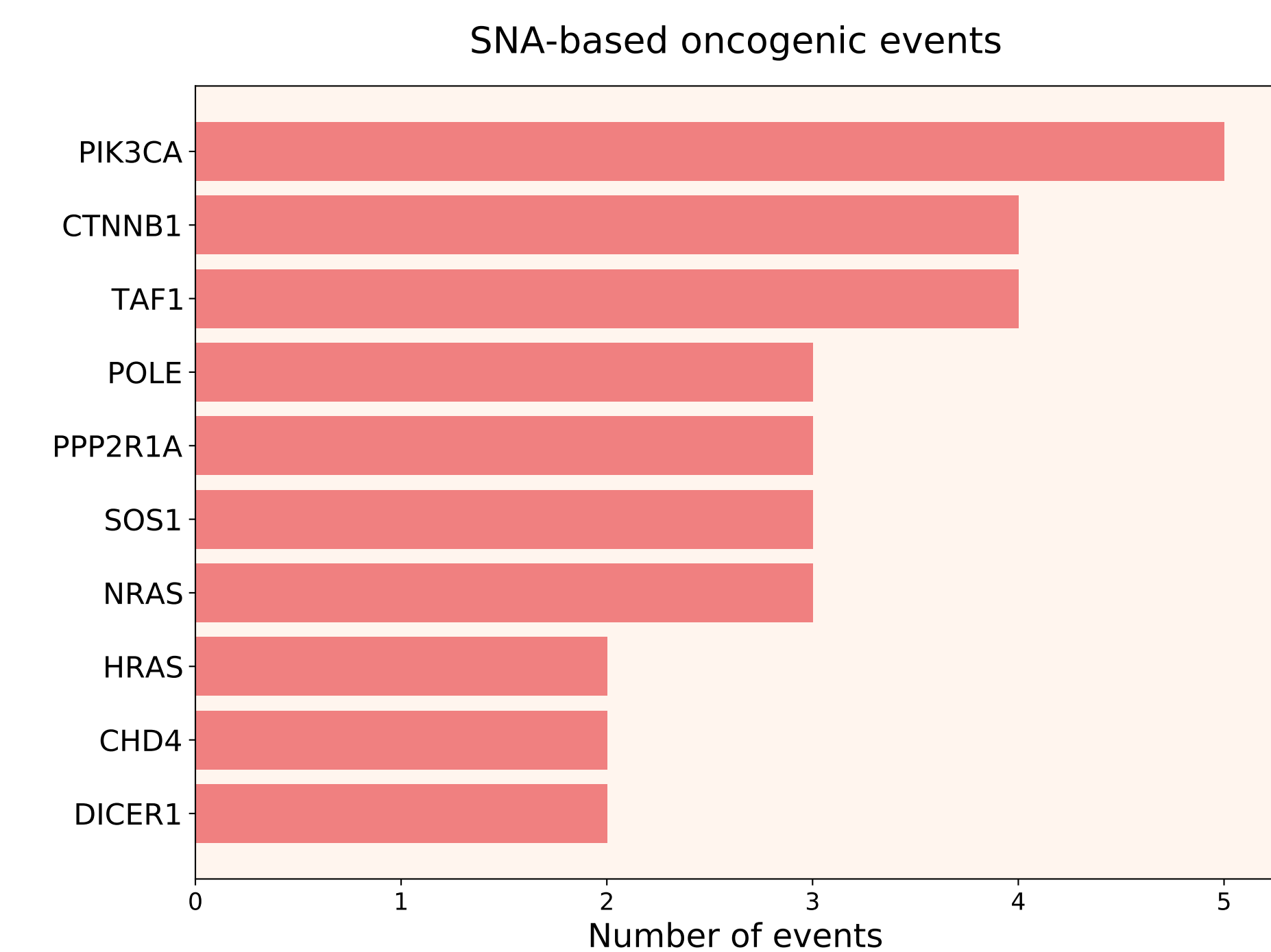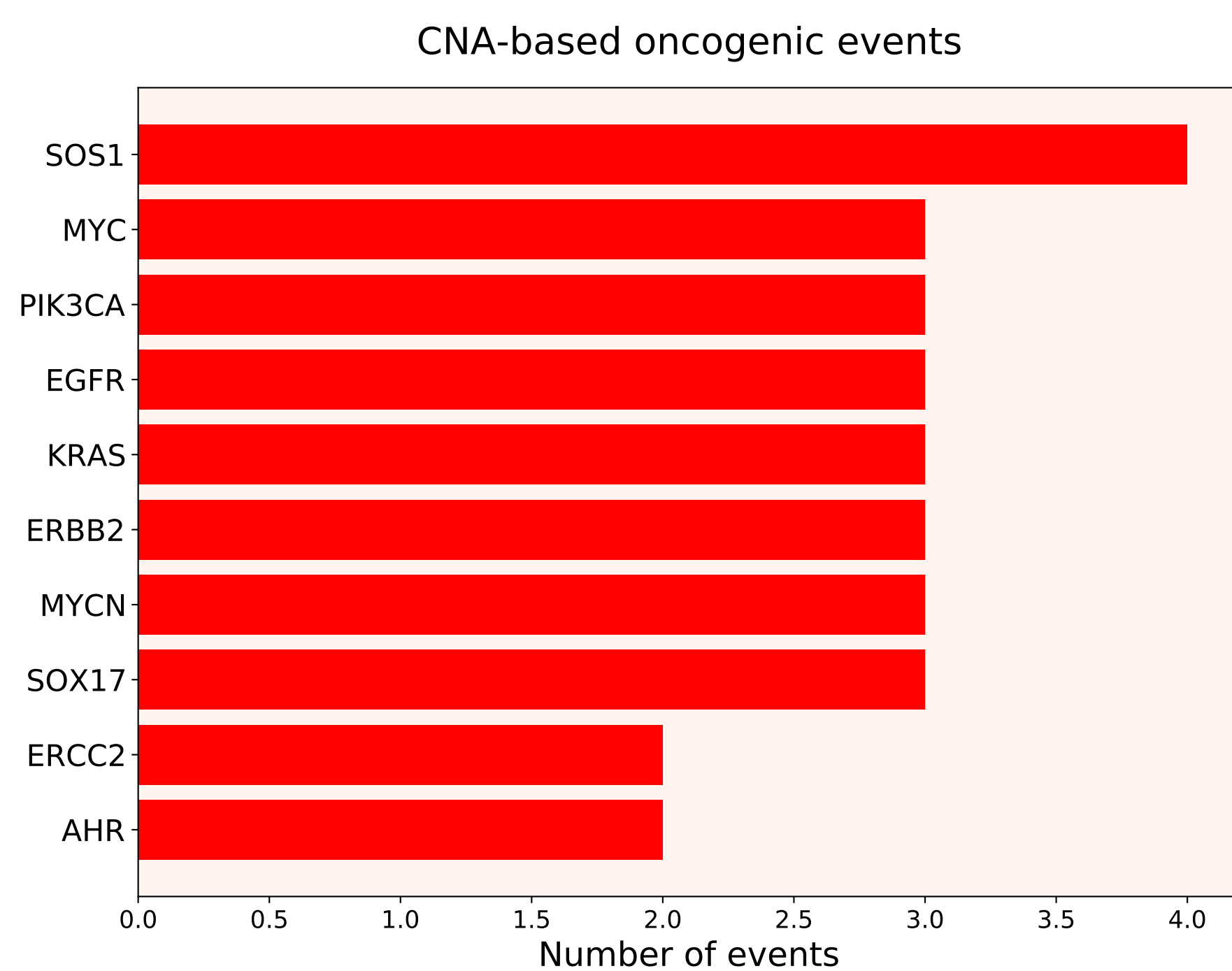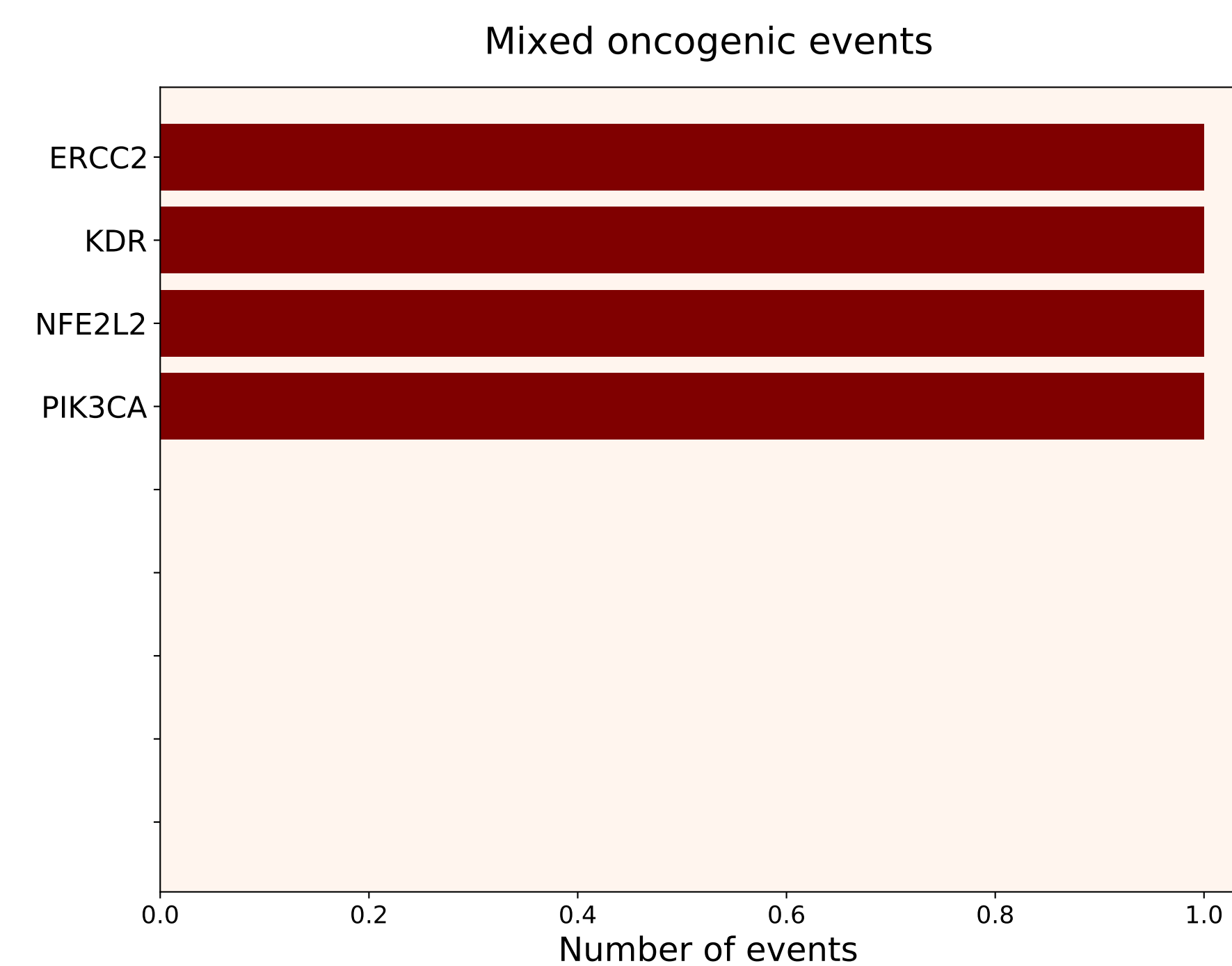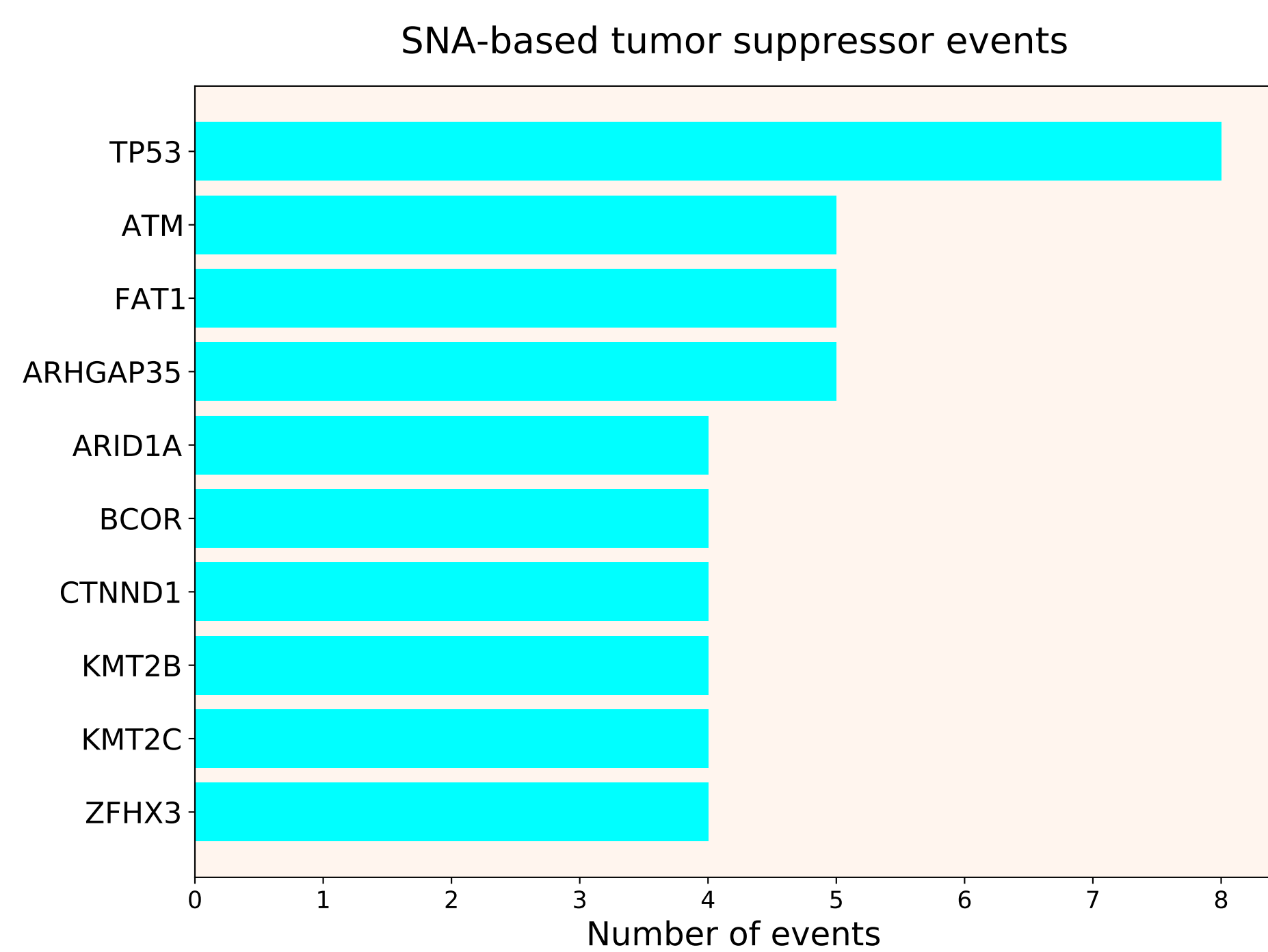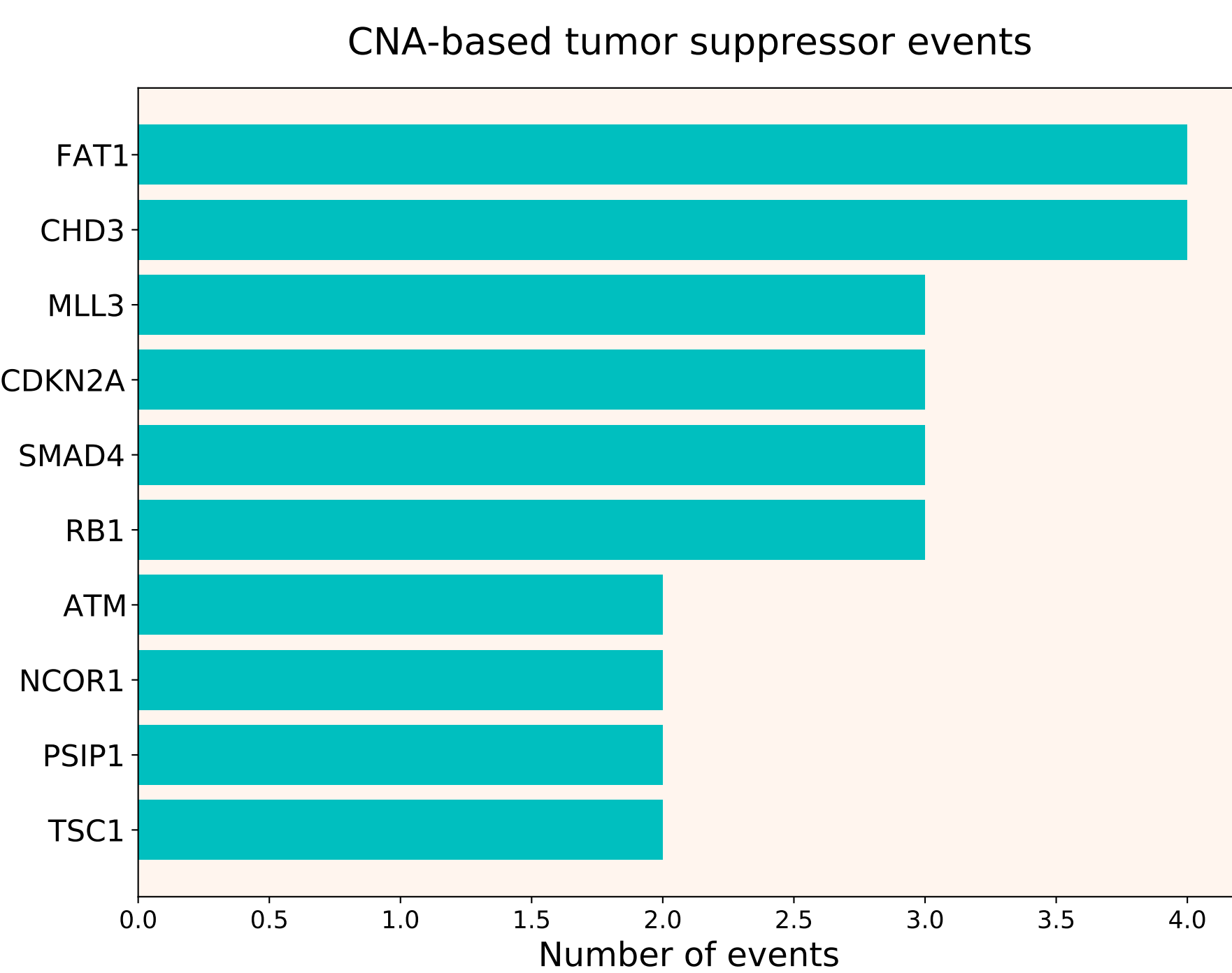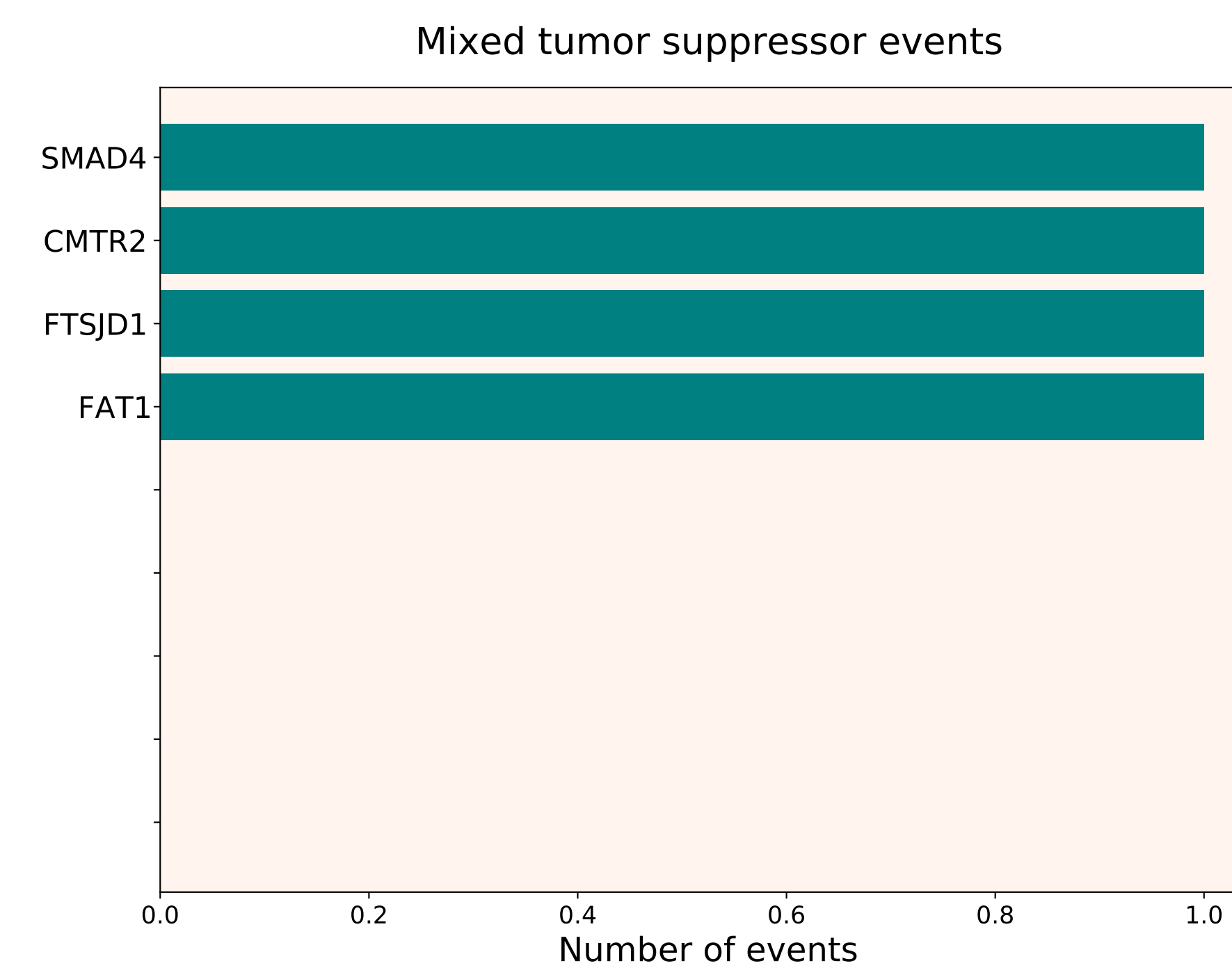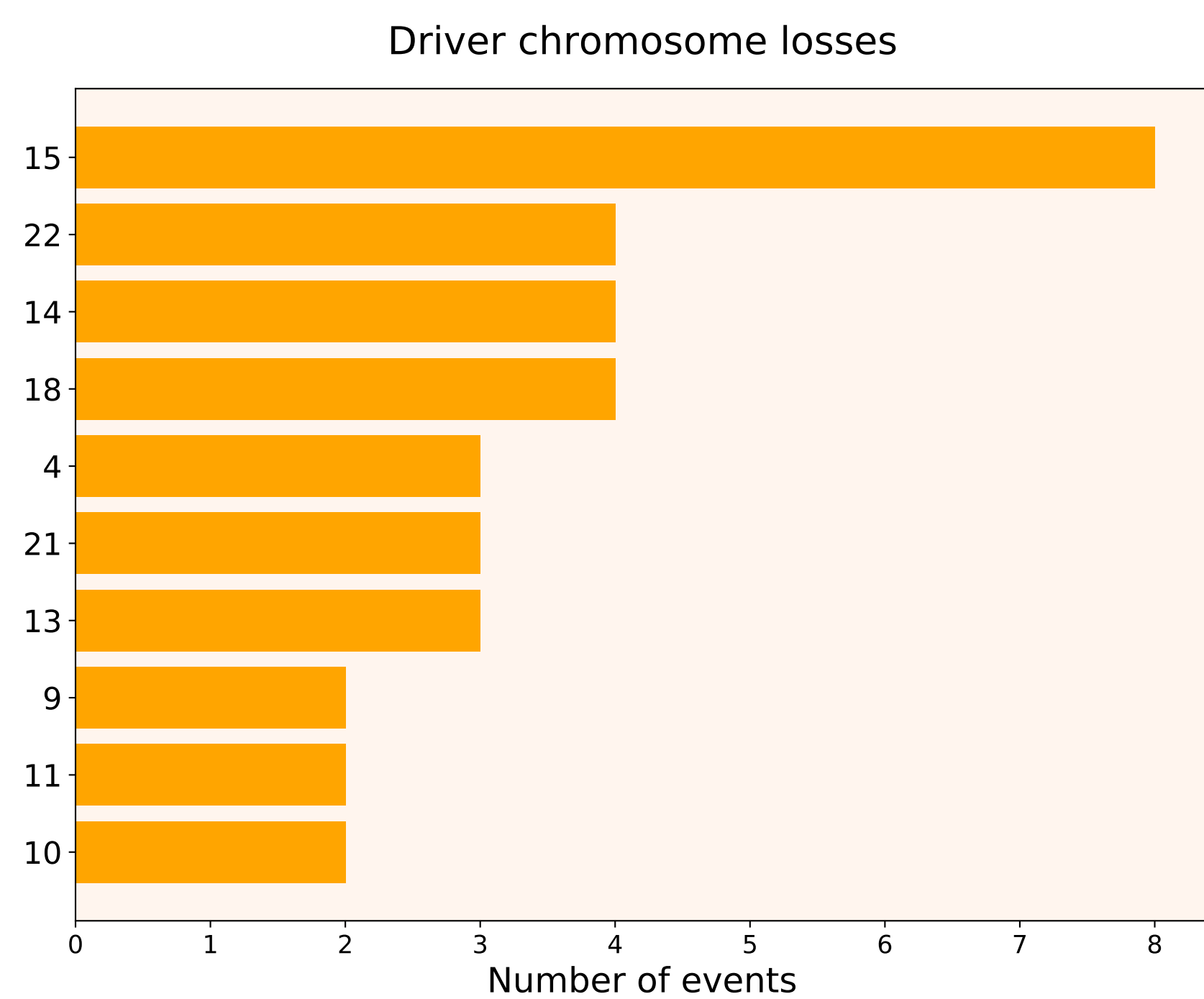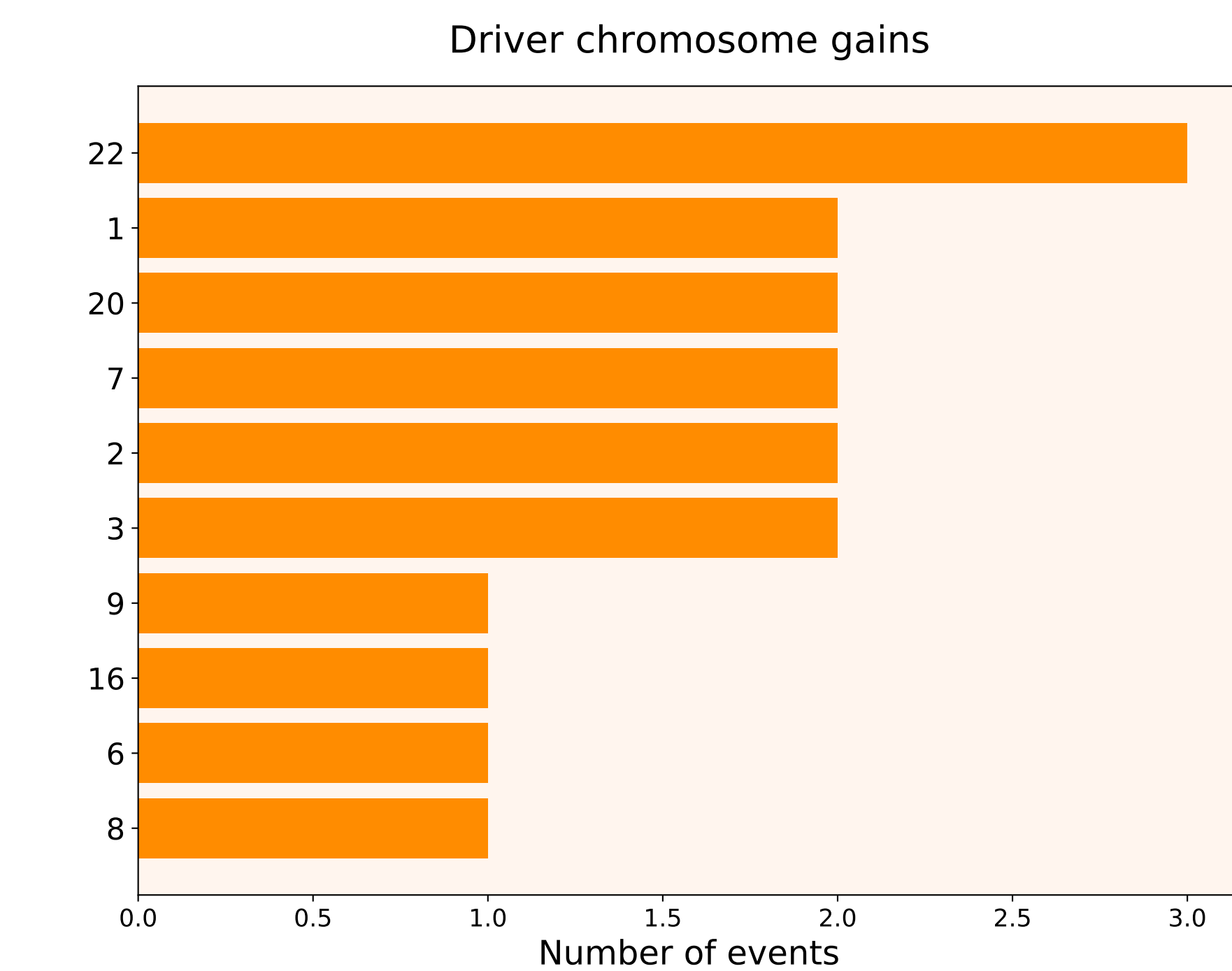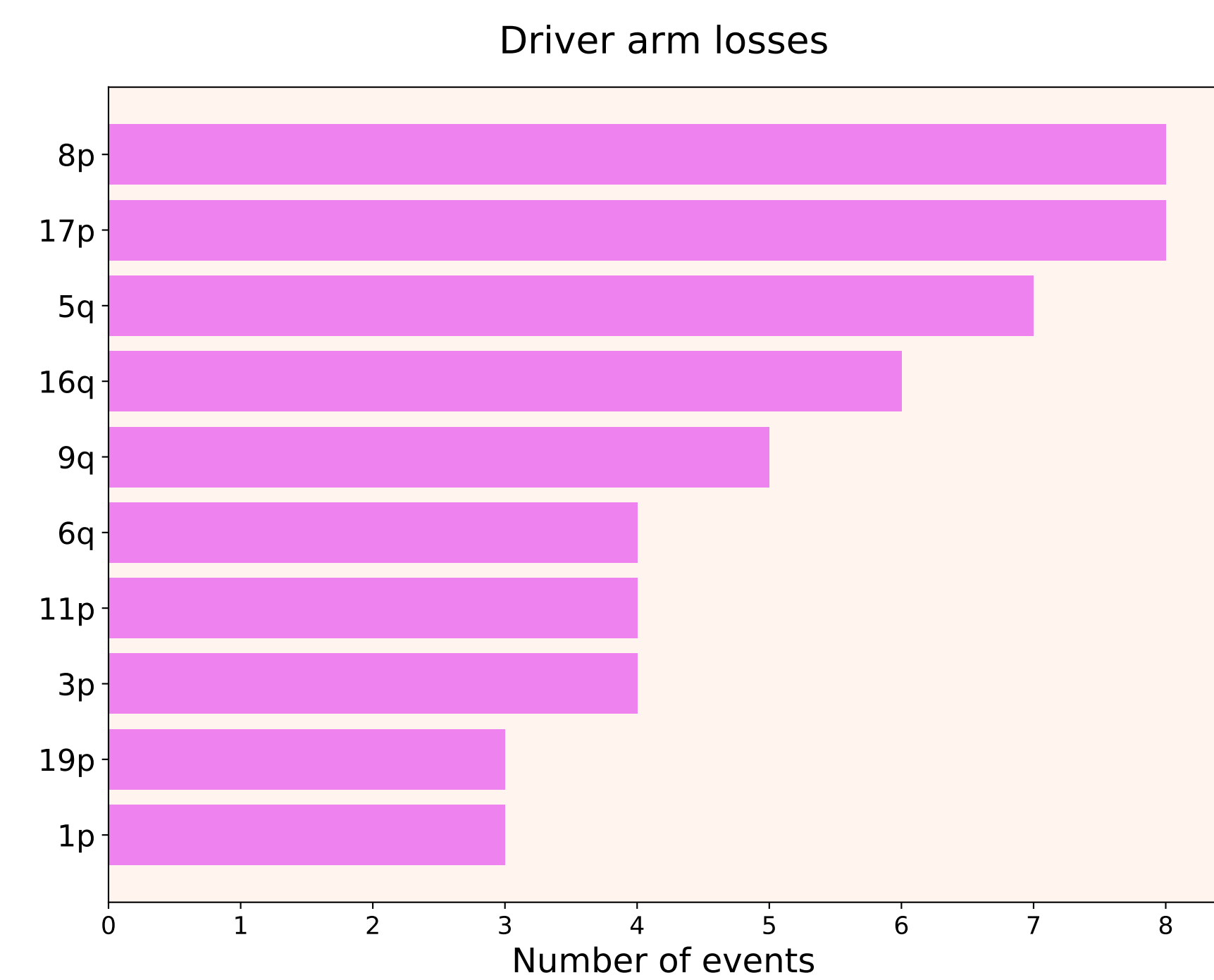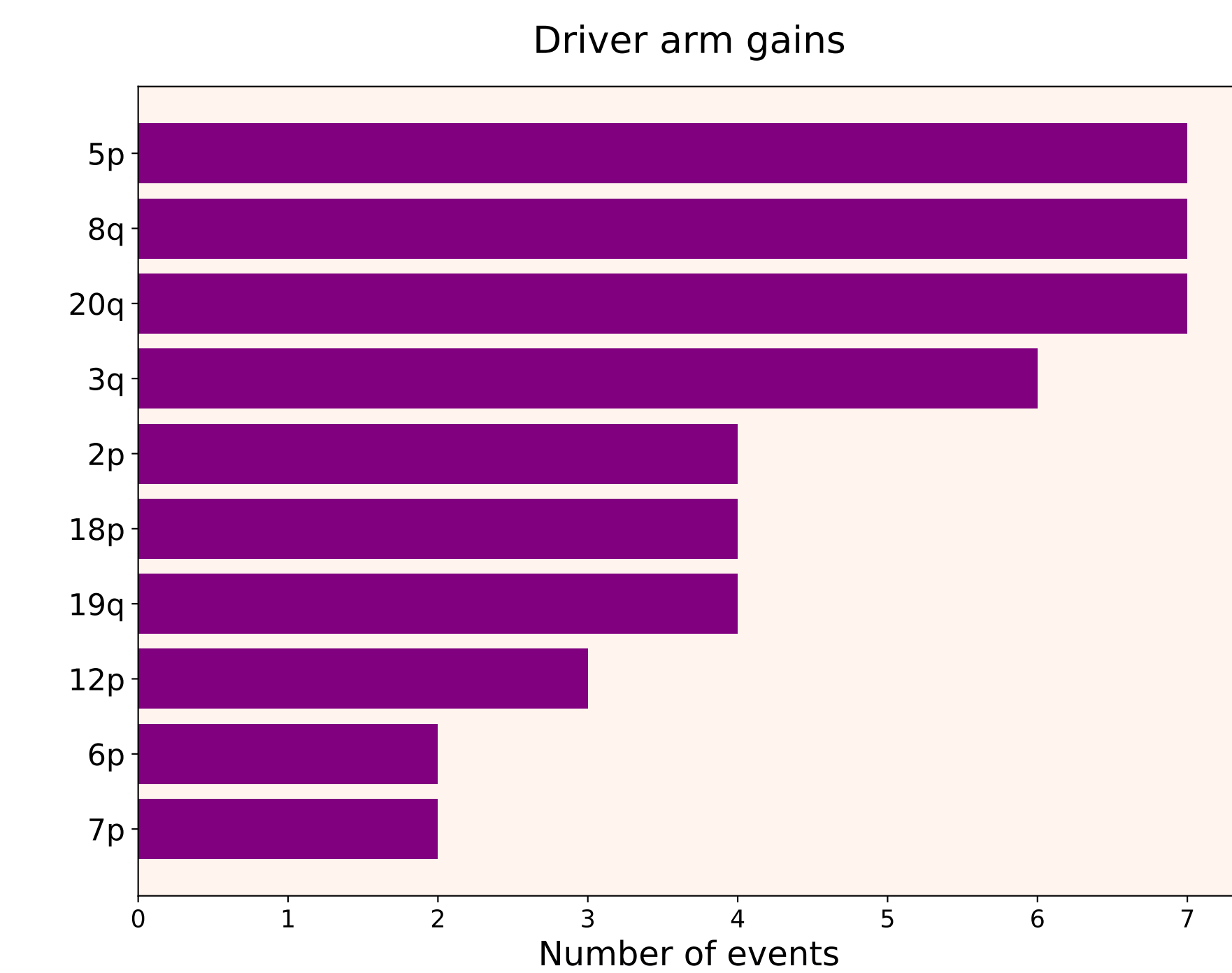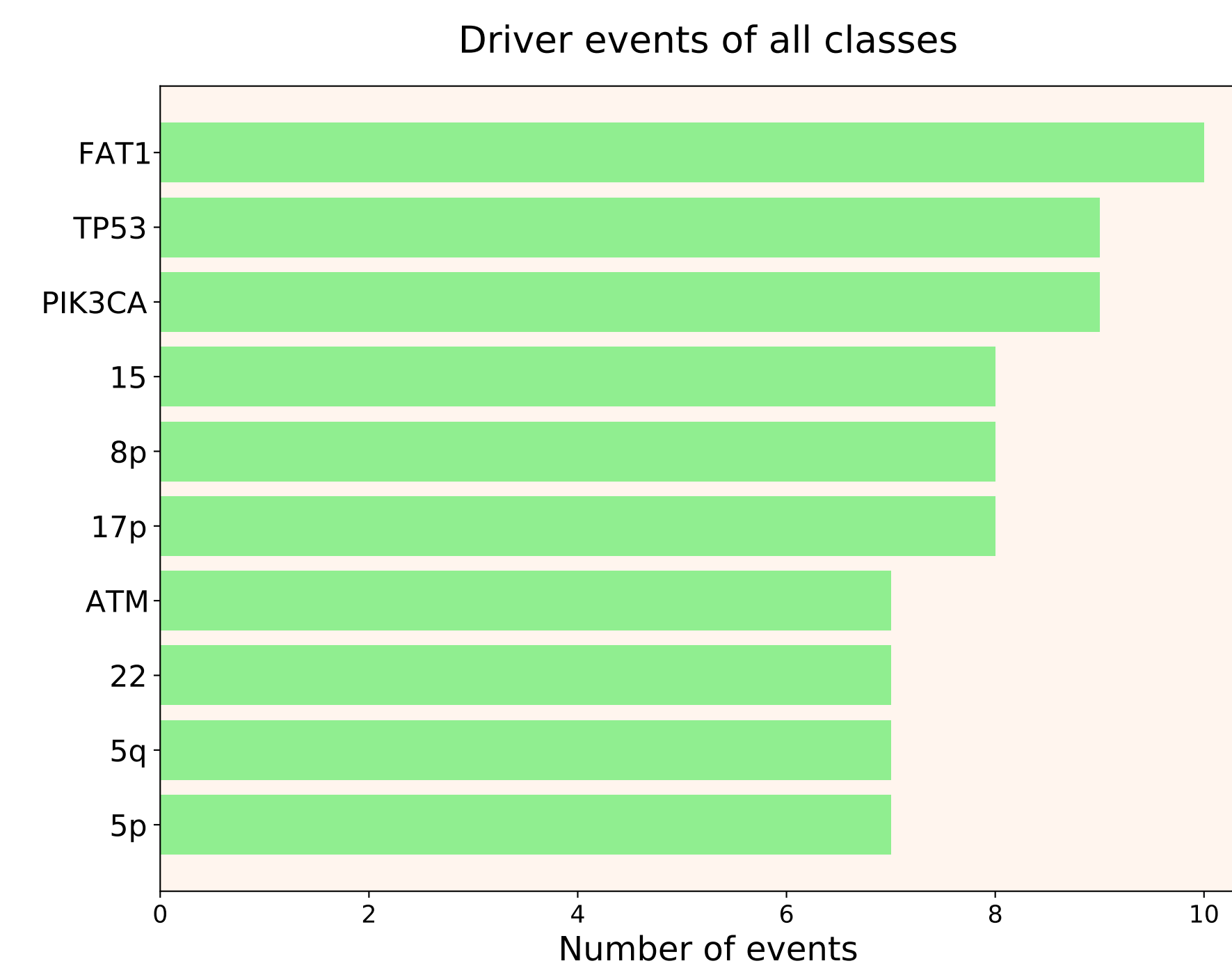

Supplement: Supplemental Information 2 [file peerj-10-13860-s002.zip › COHORTS/genes plots/detailed/2021_8_16_14_9_distribution_events_detailed_females_31.pdf]

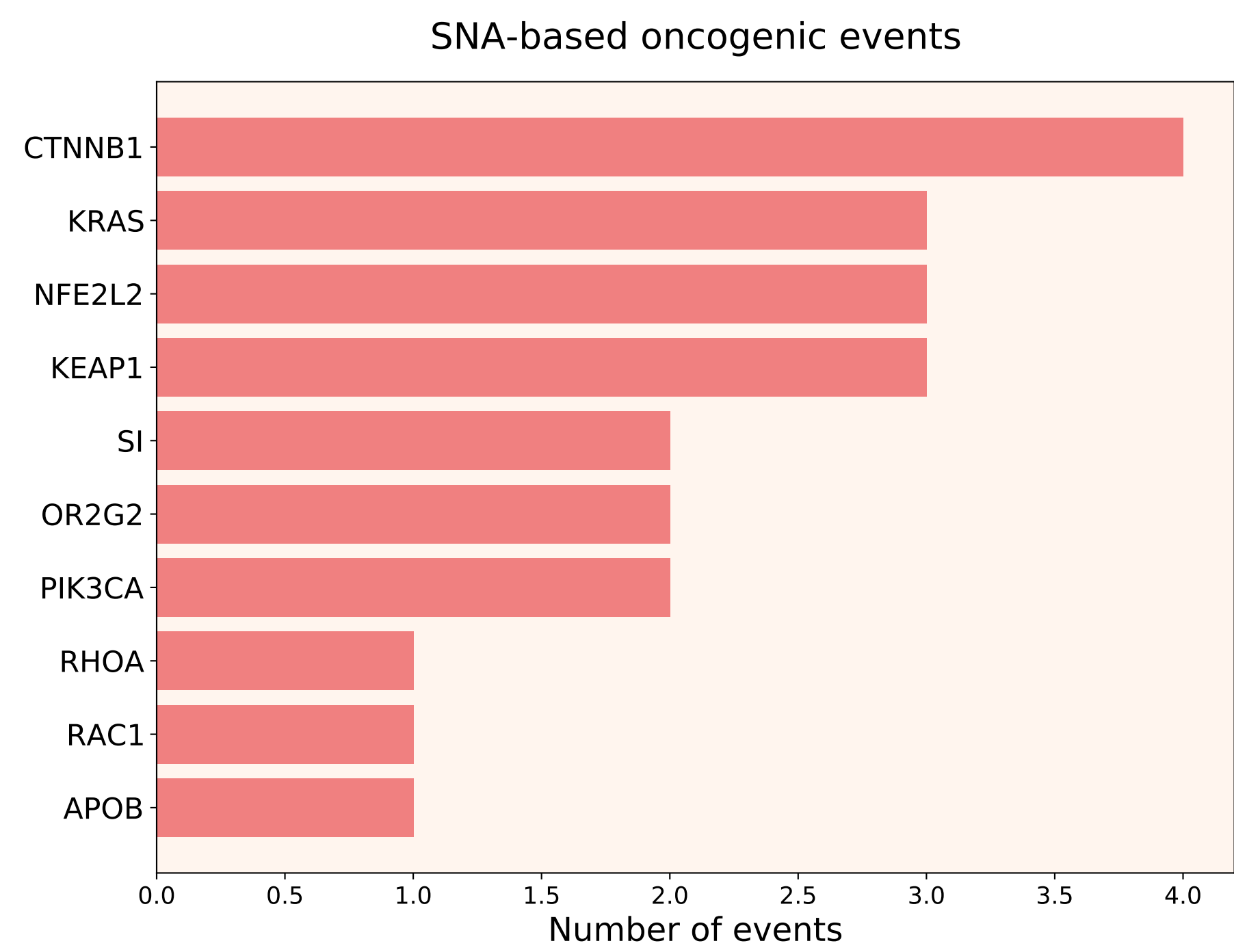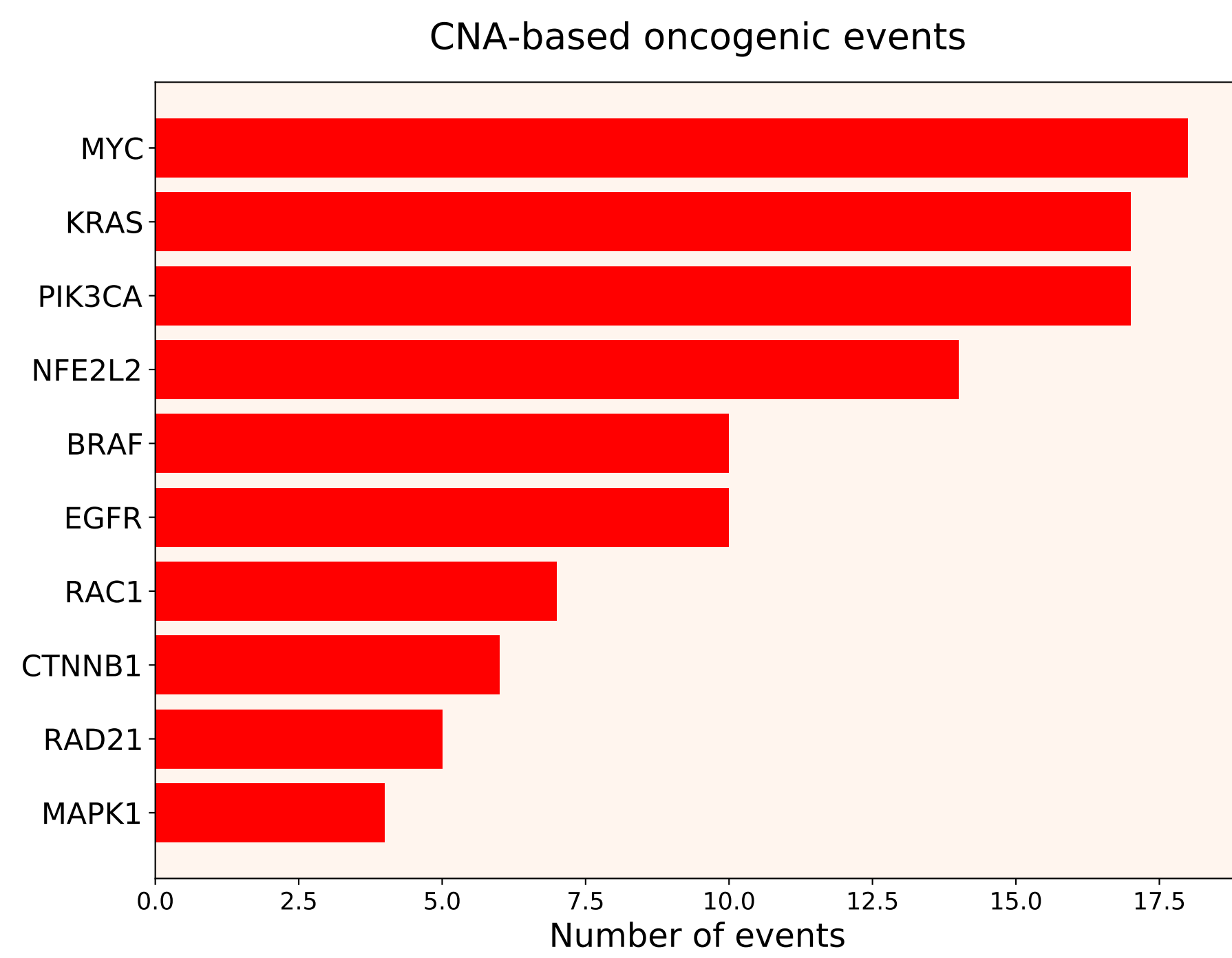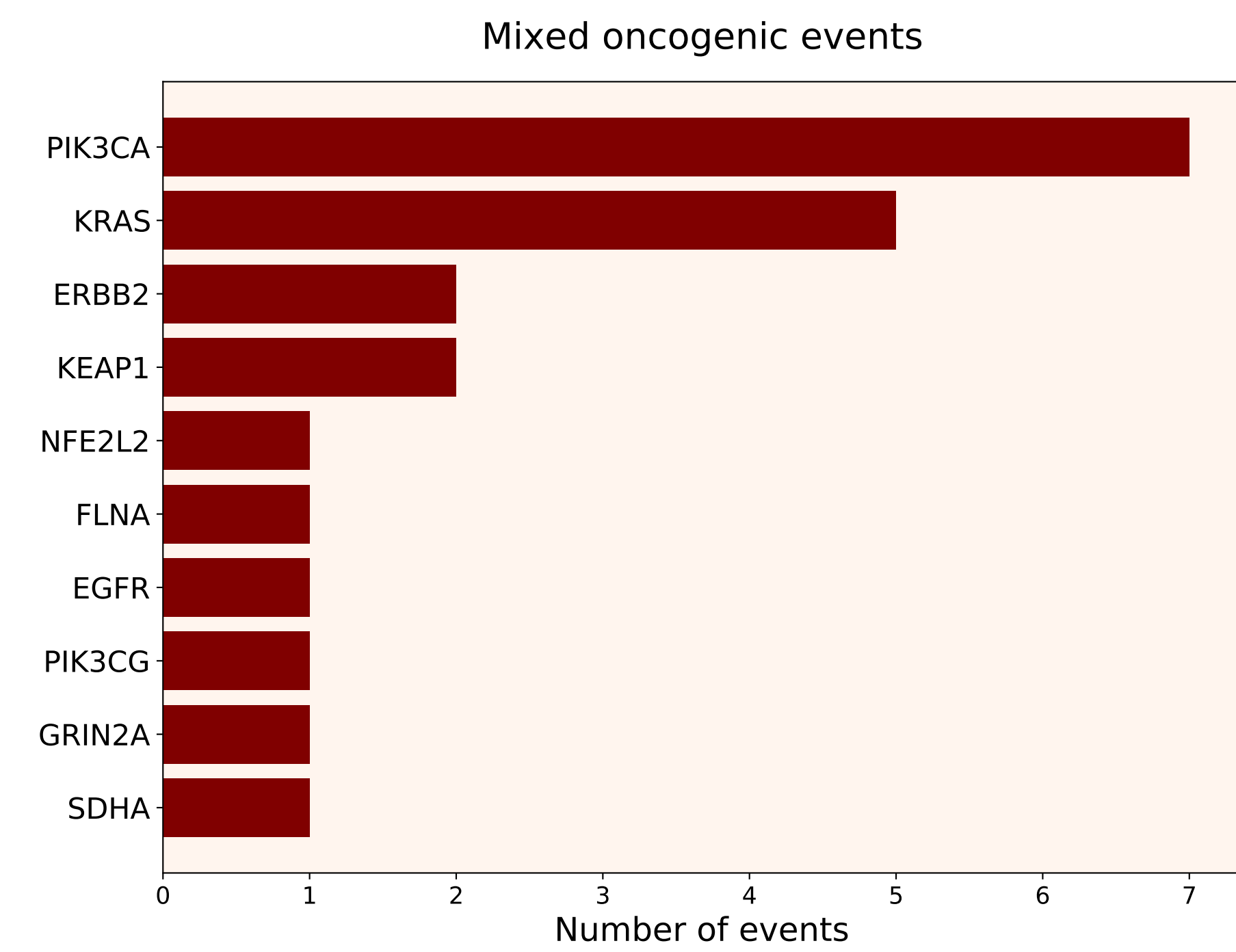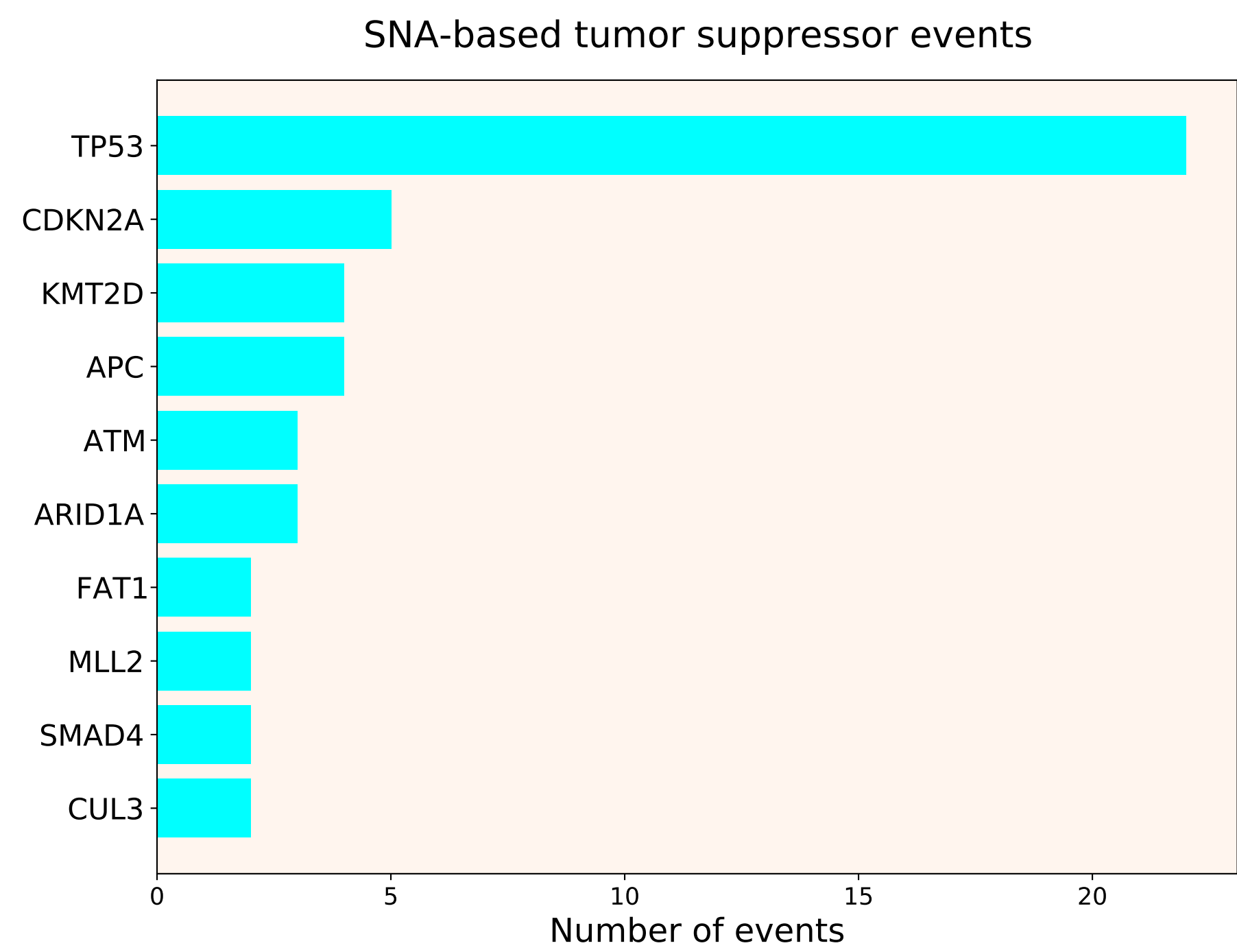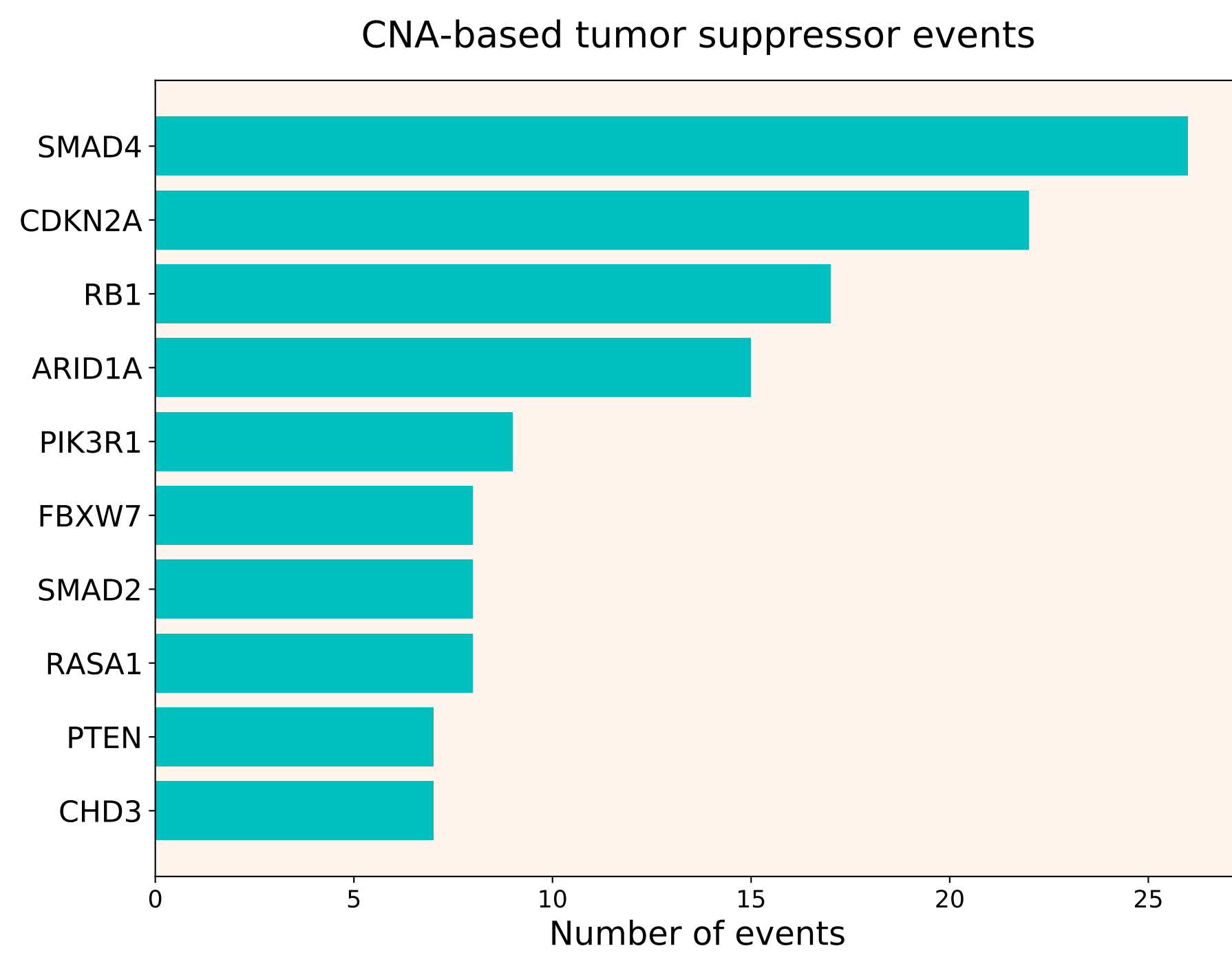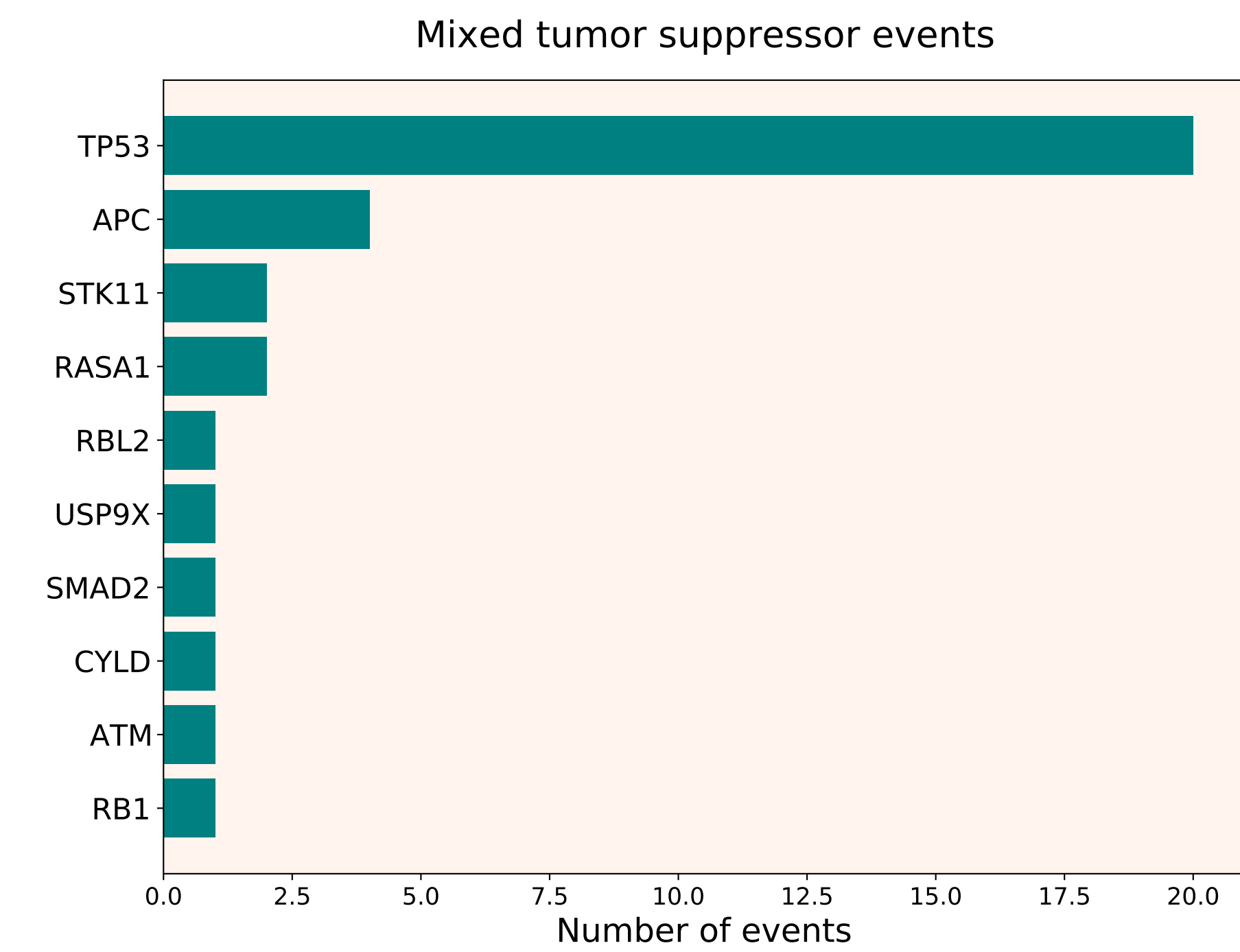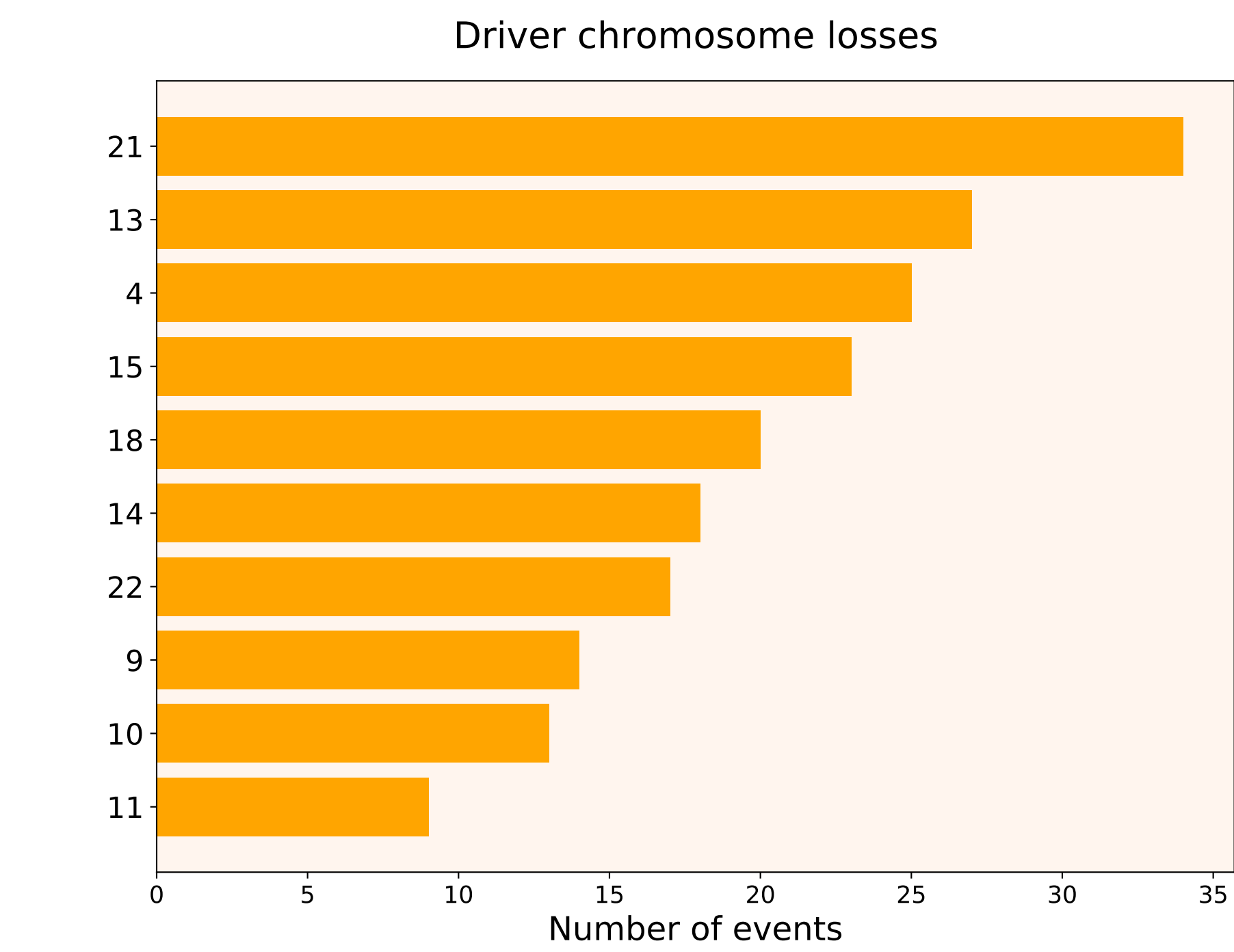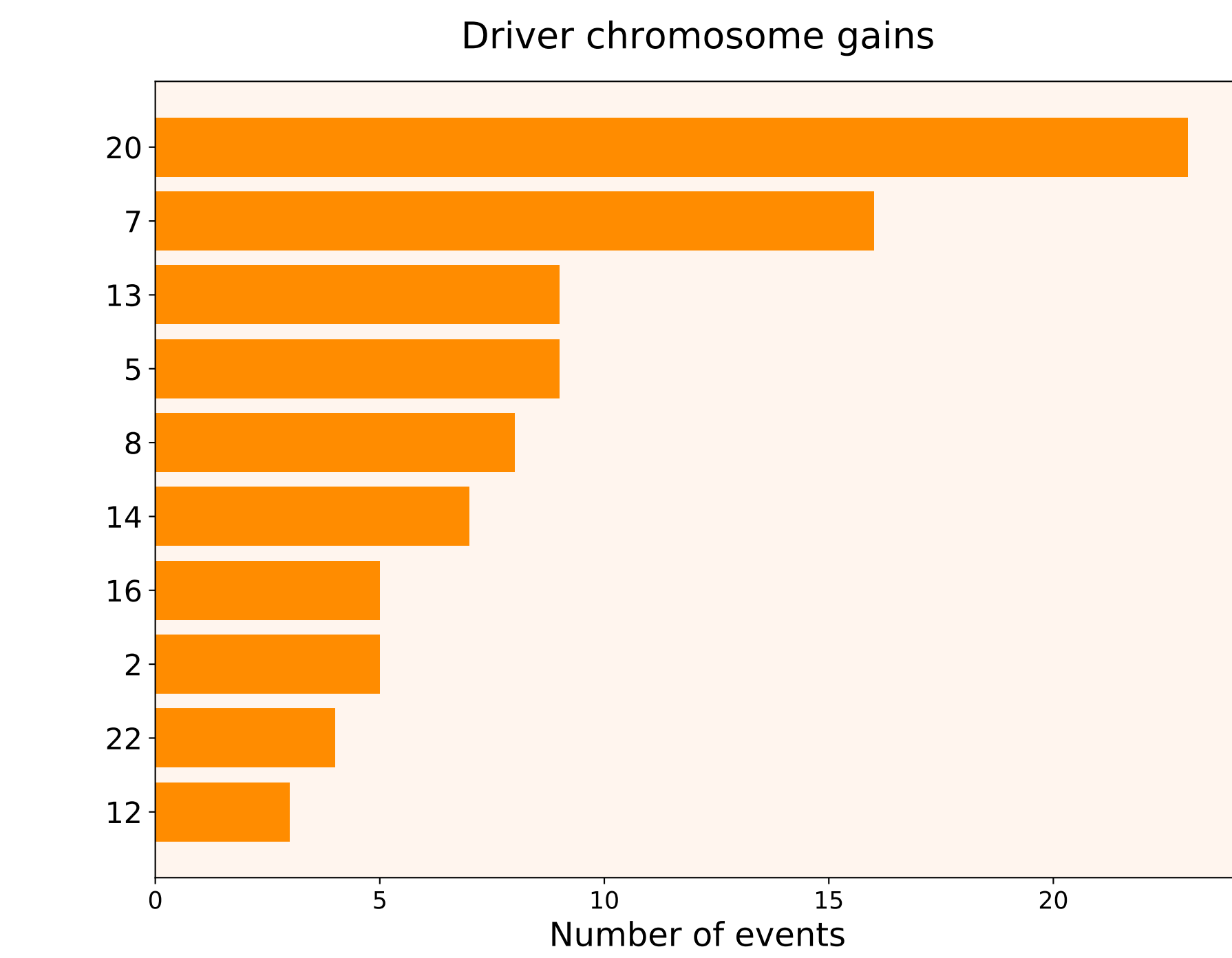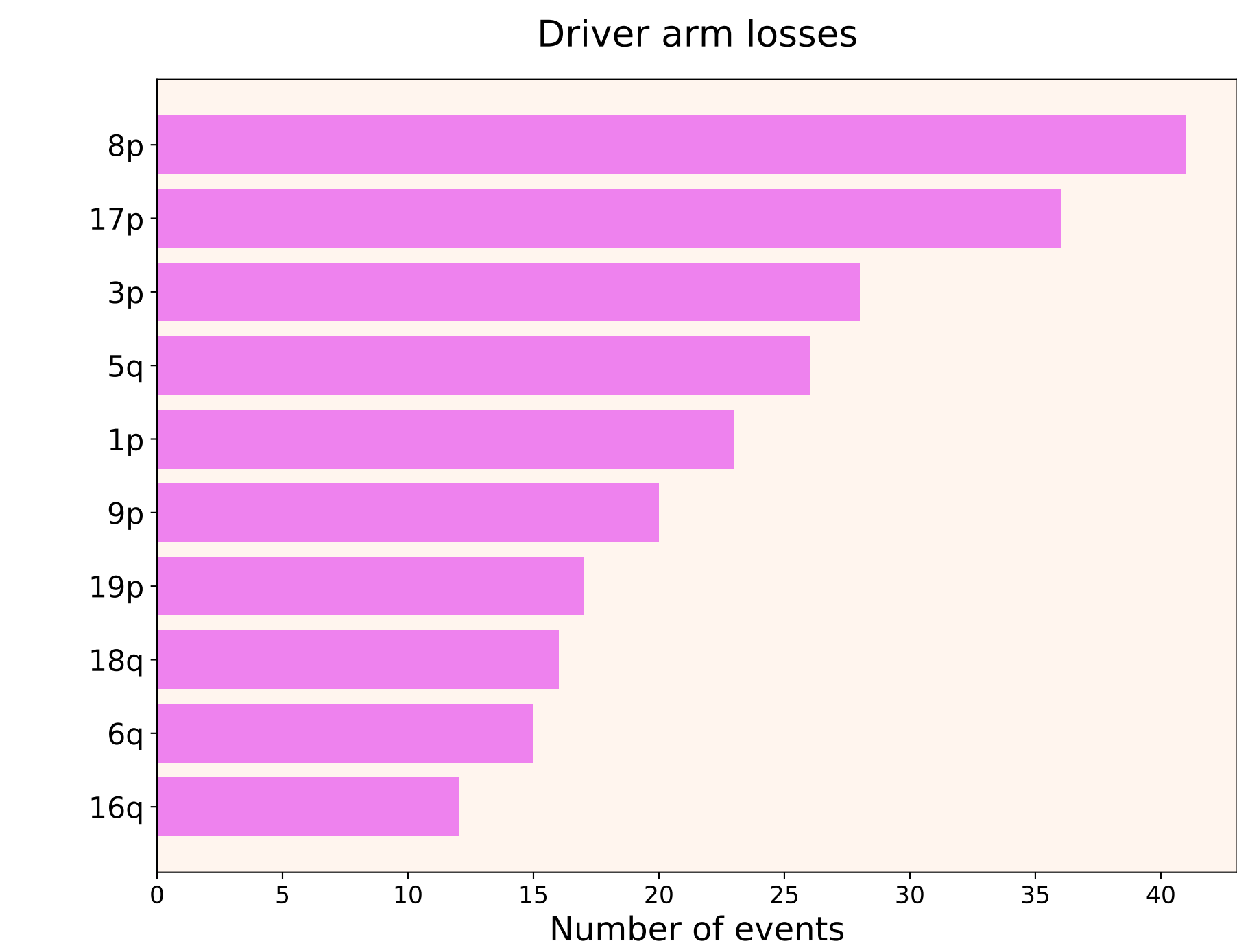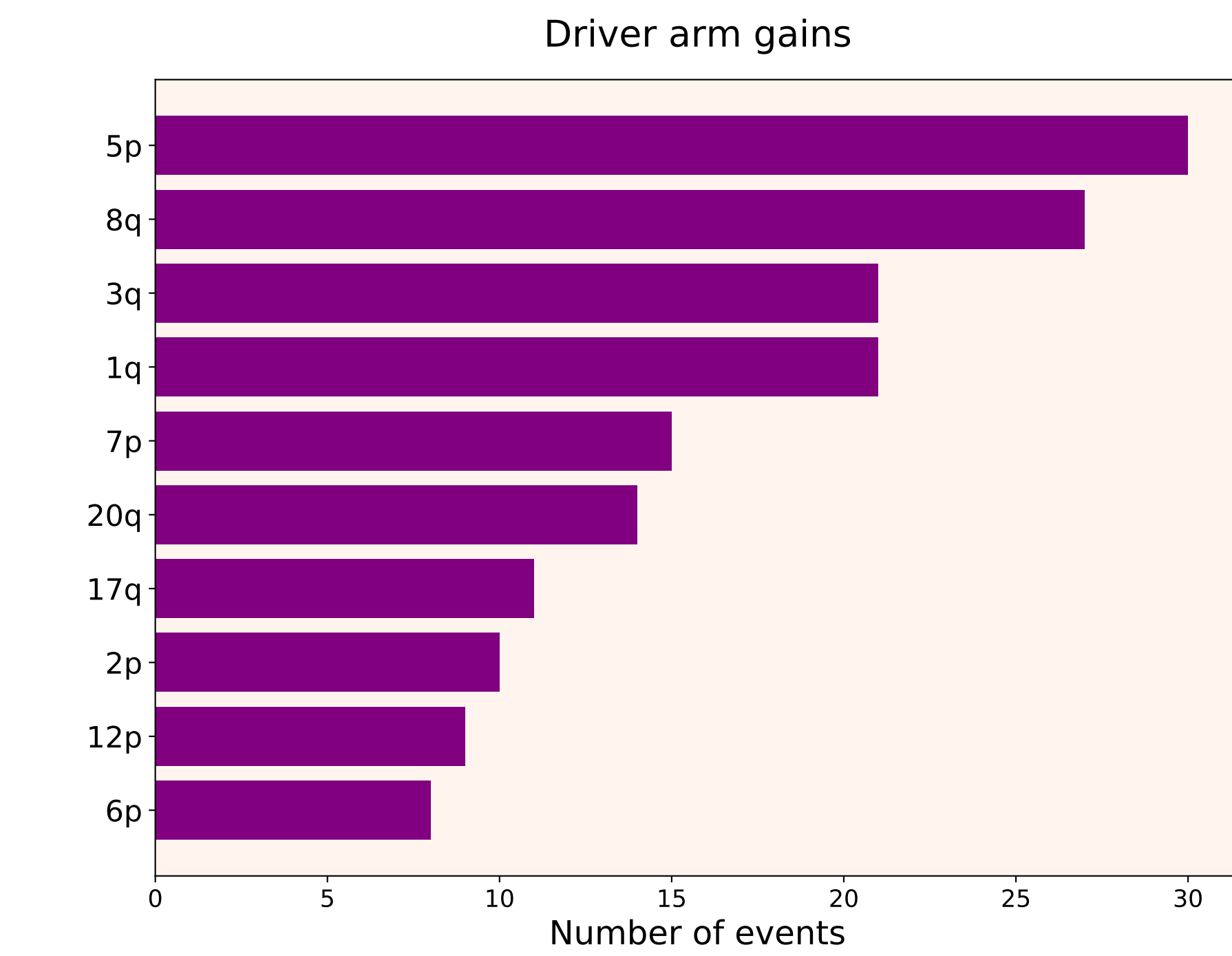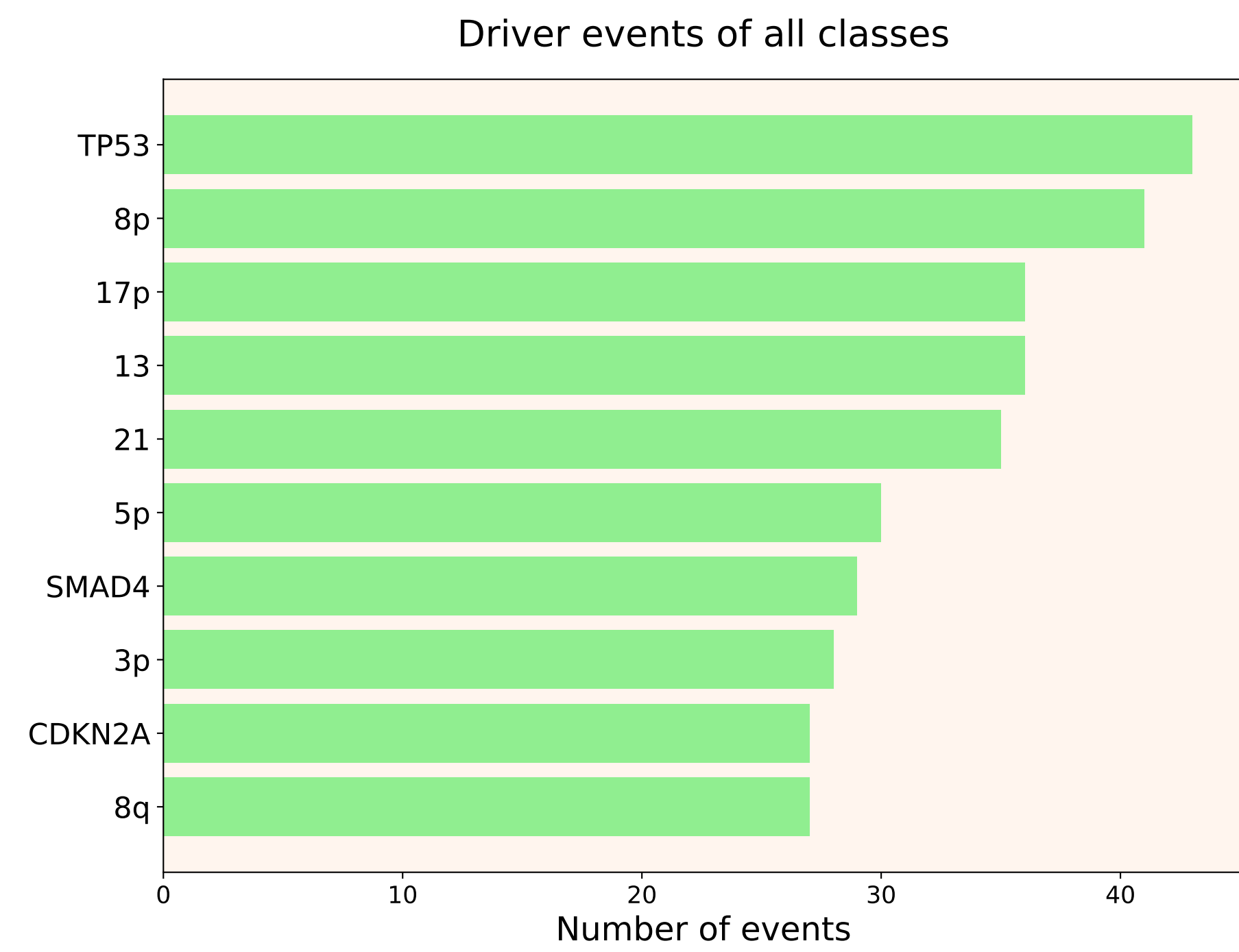

Supplement: Supplemental Information 2 [file peerj-10-13860-s002.zip › COHORTS/genes plots/detailed/2021_8_16_14_9_distribution_events_detailed_males_21.pdf]

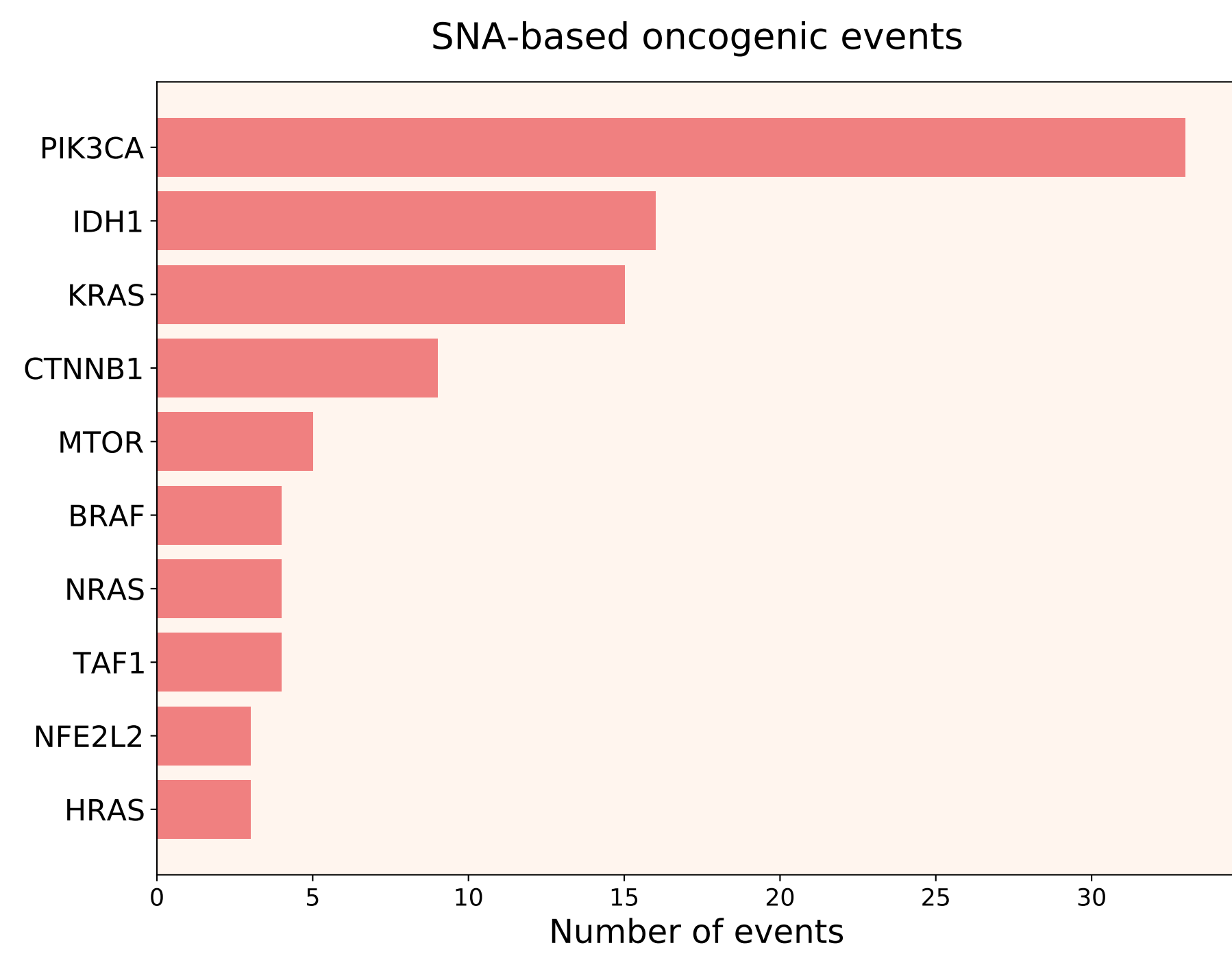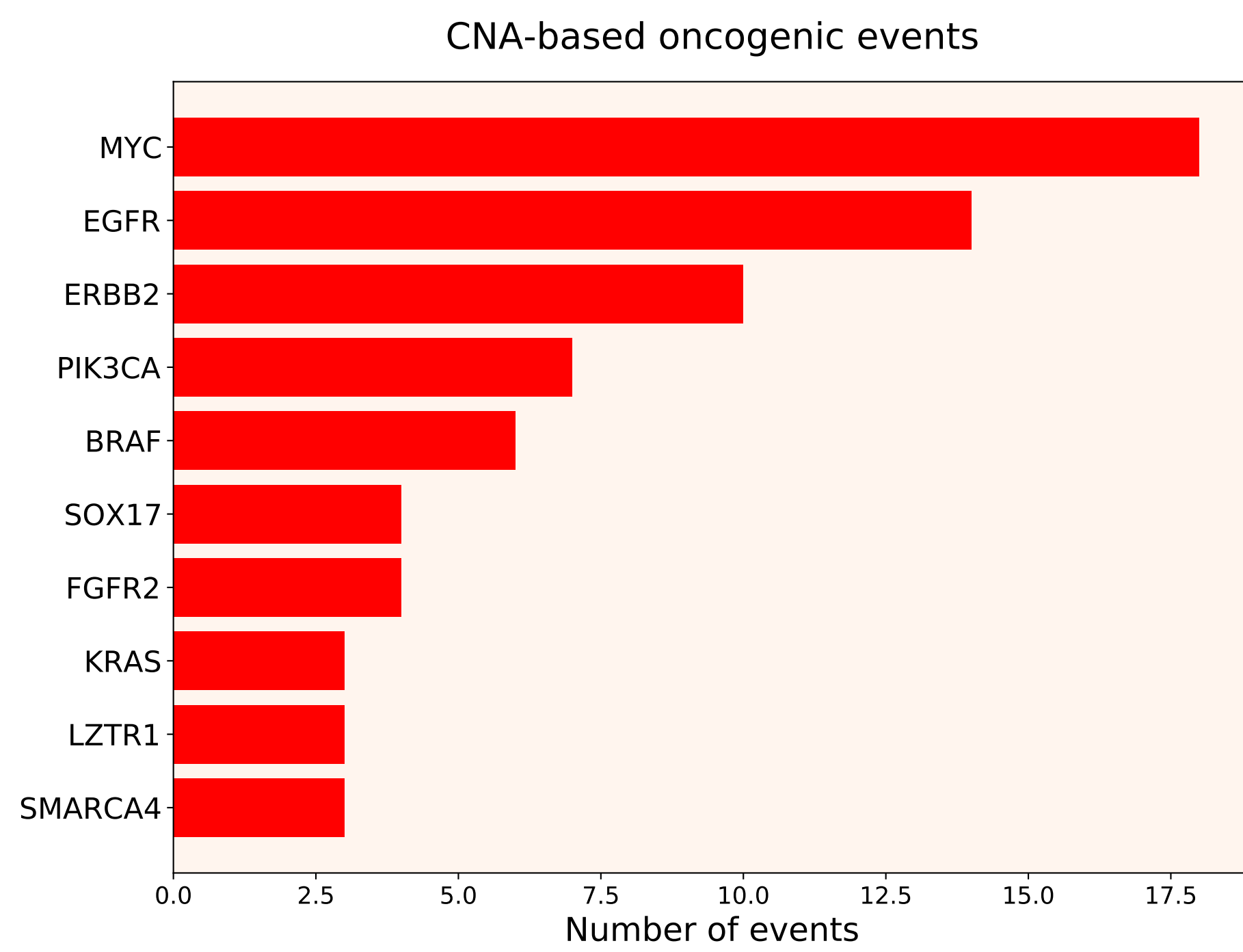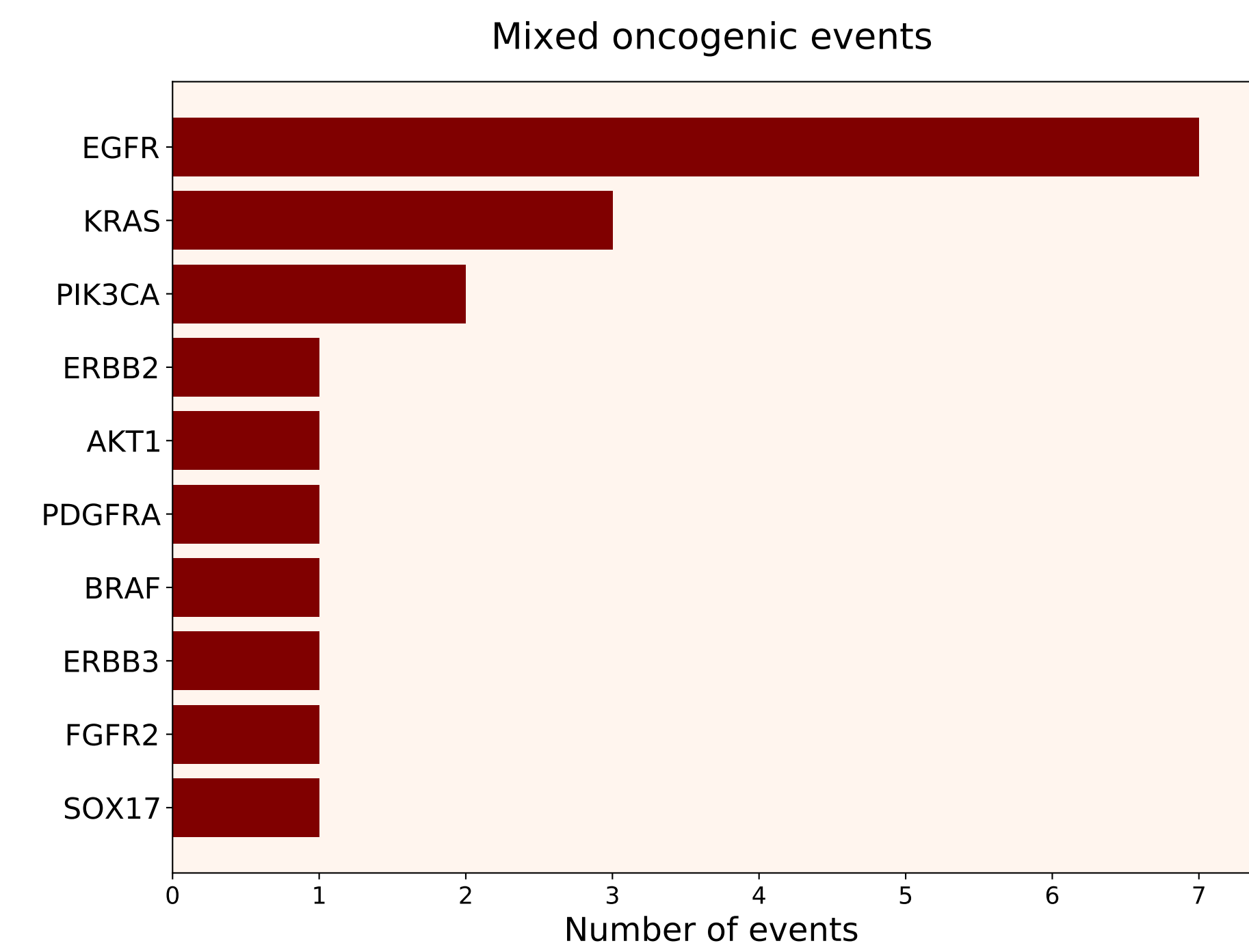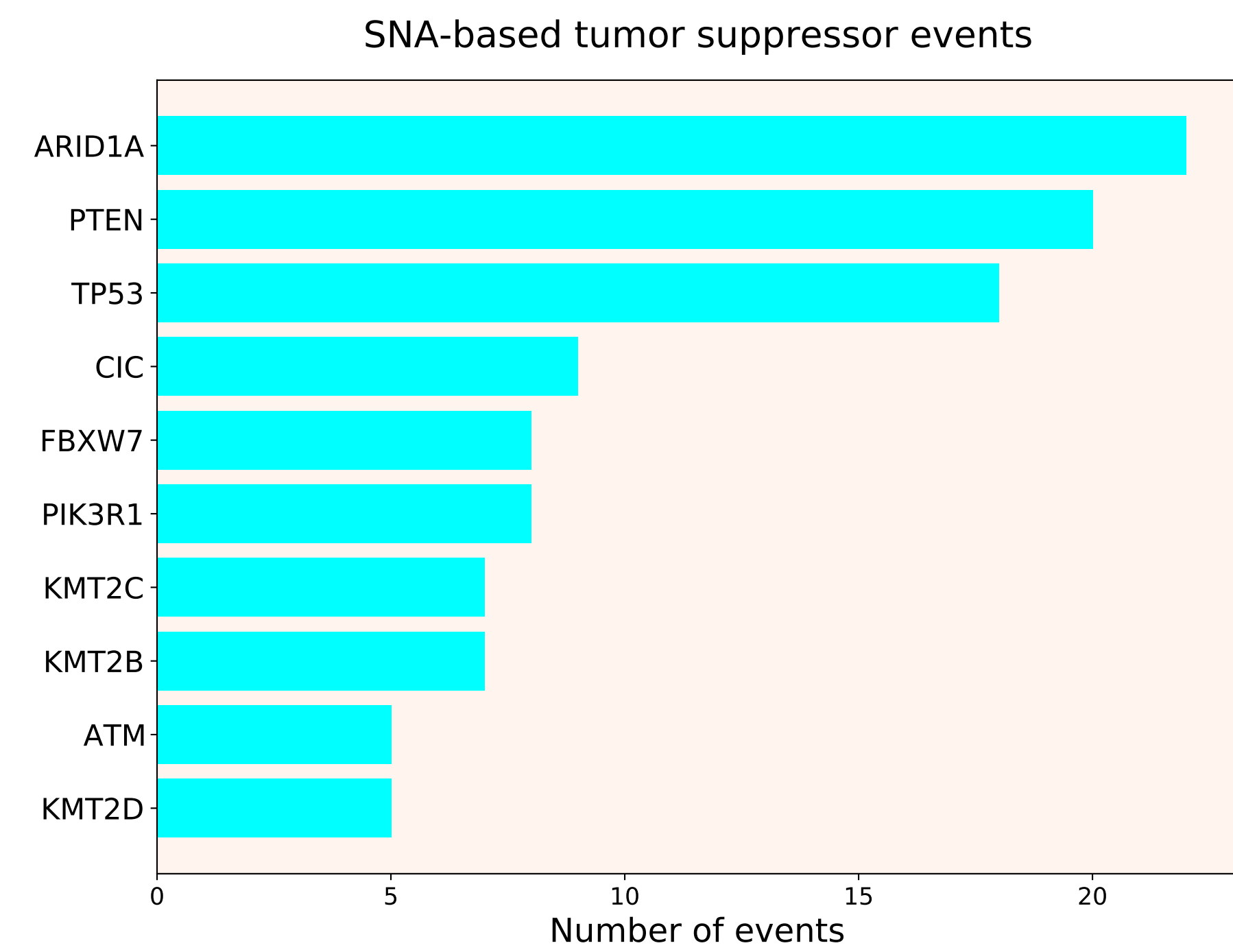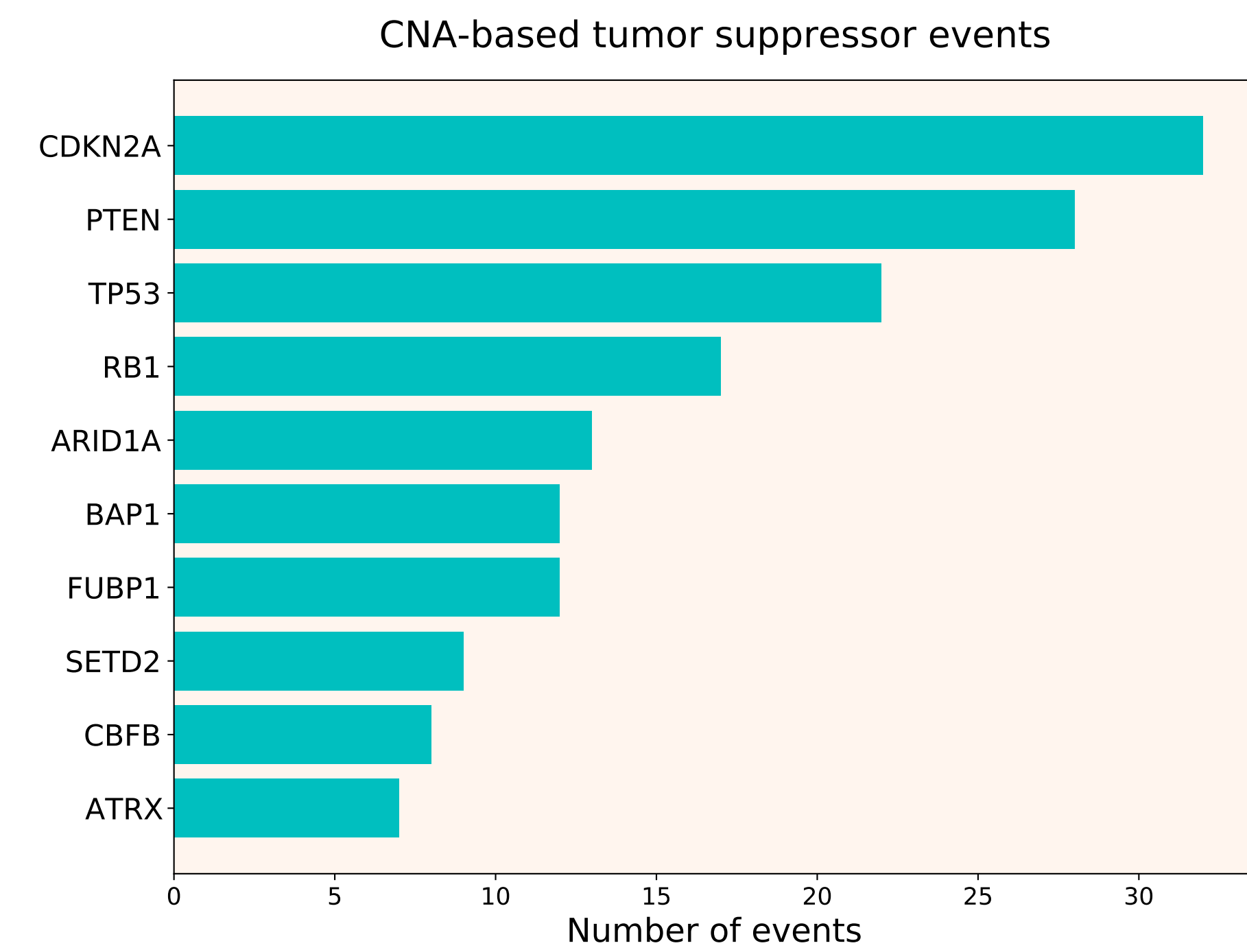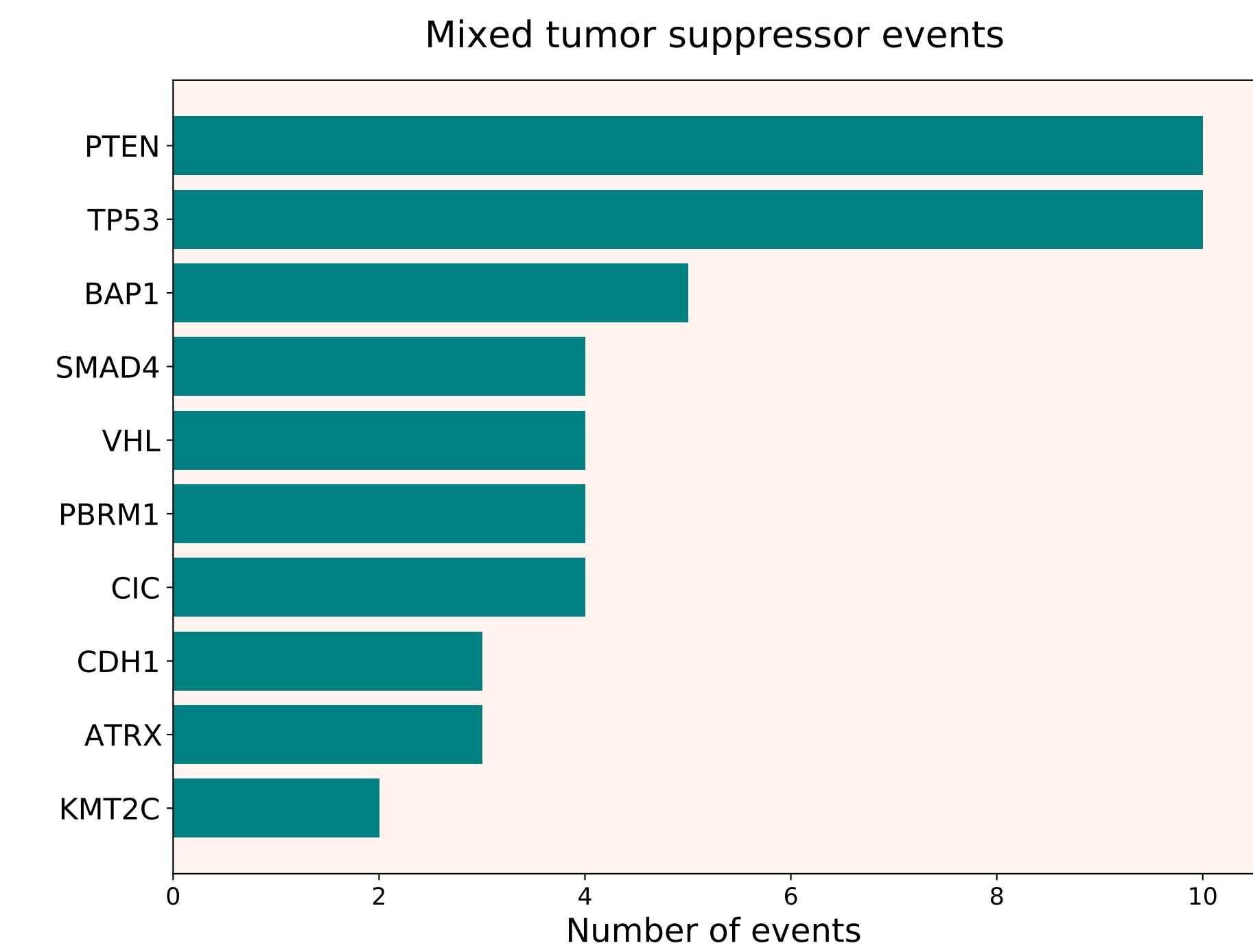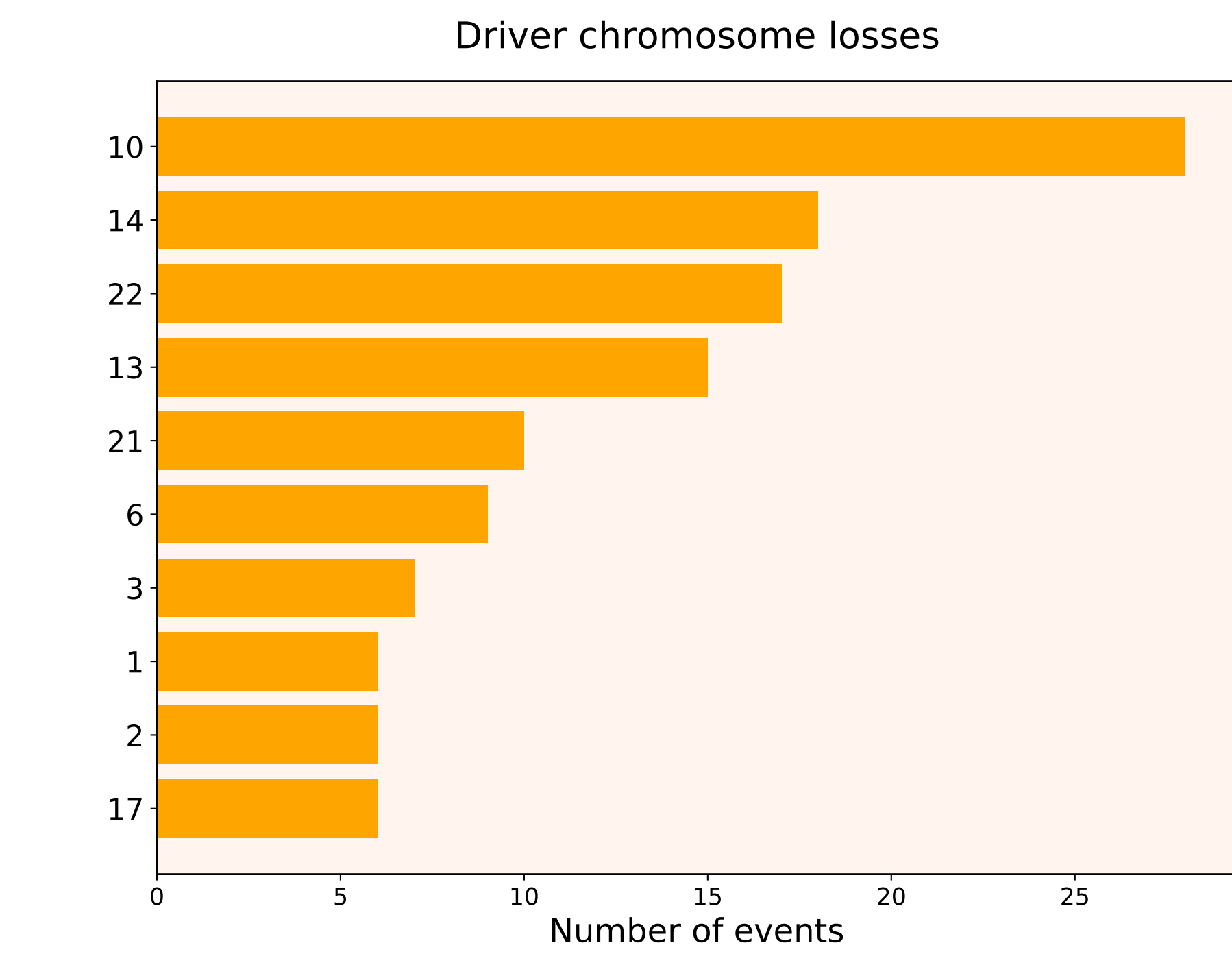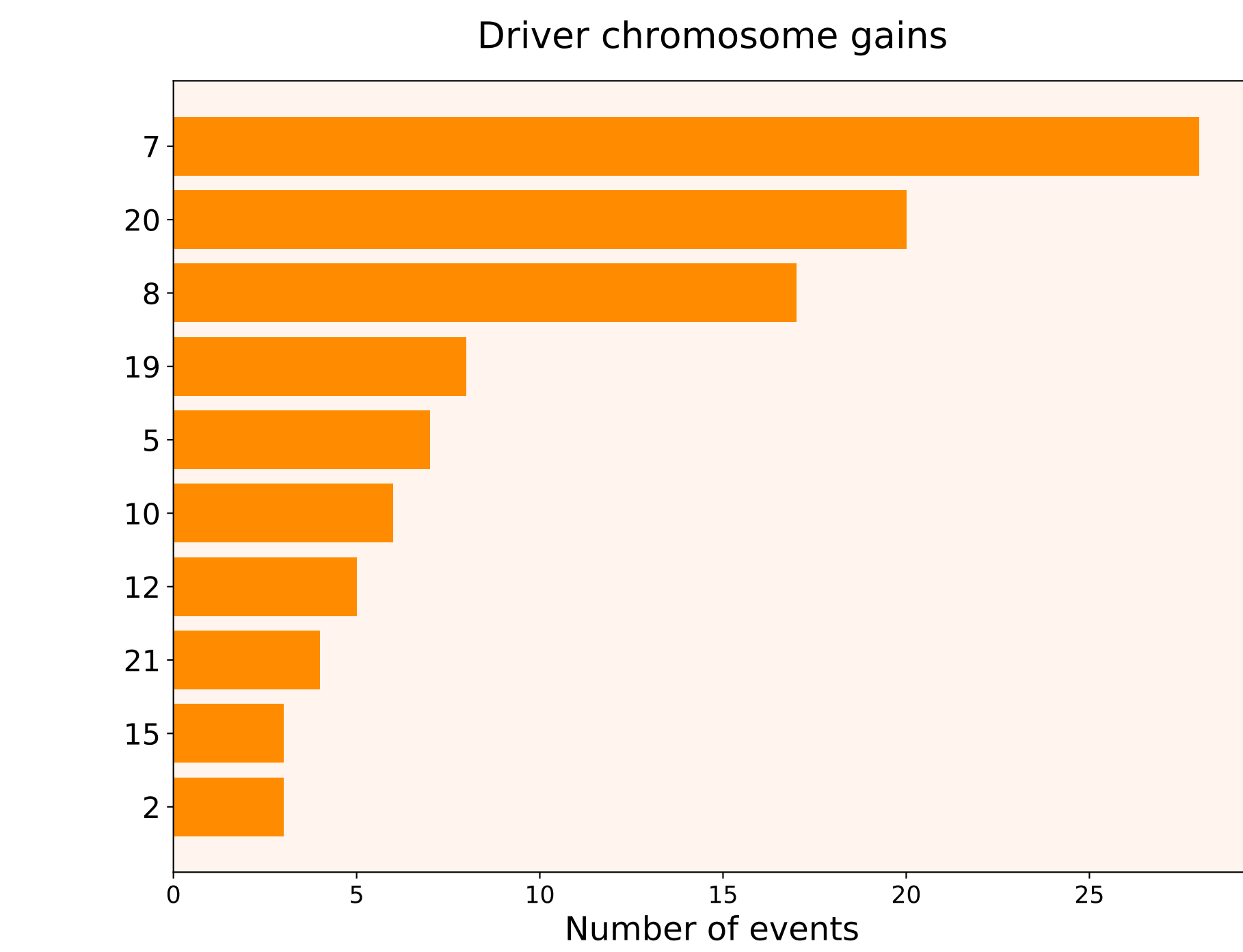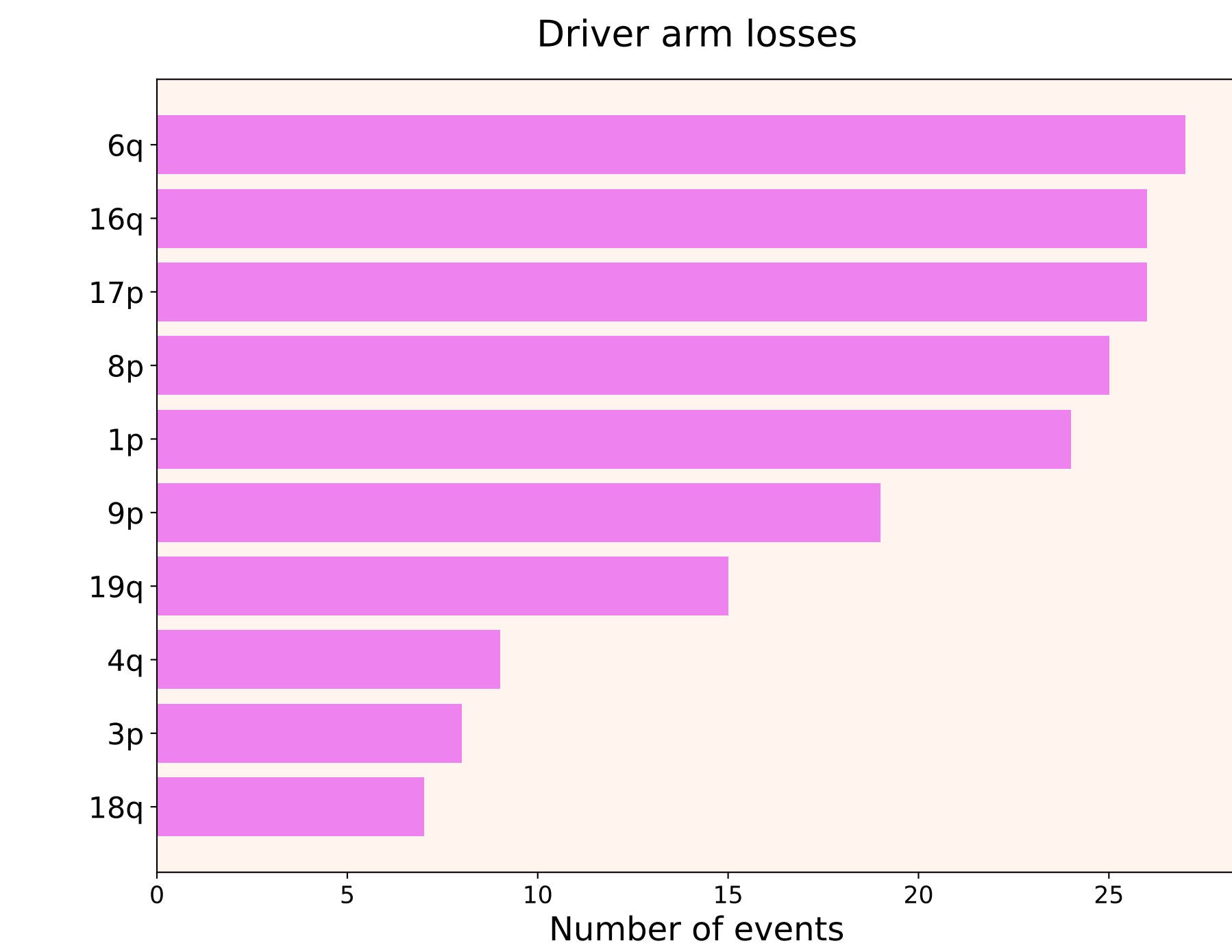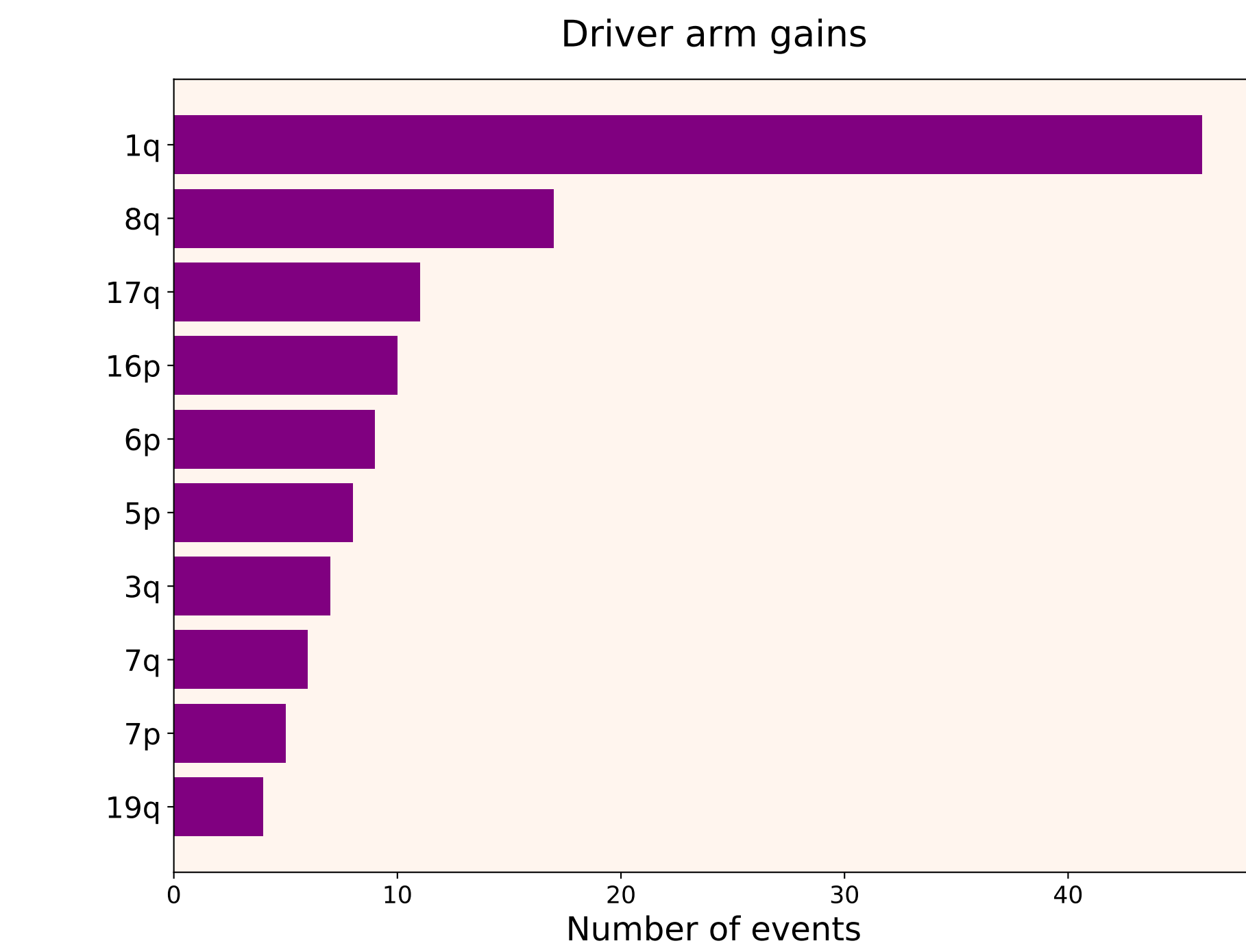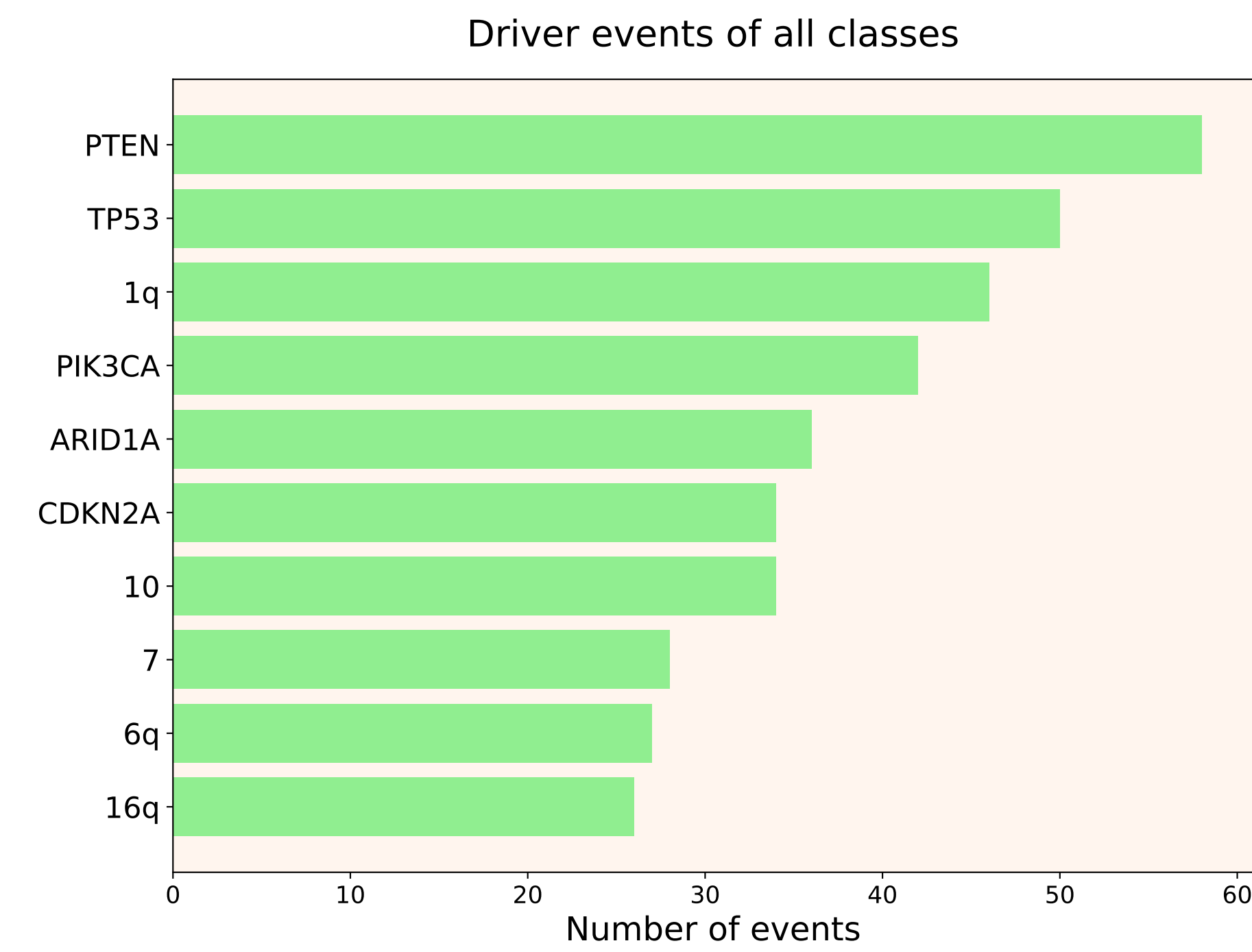

Supplement: Supplemental Information 2 [file peerj-10-13860-s002.zip › COHORTS/genes plots/detailed/2021_8_16_14_9_distribution_events_detailed_females_8.pdf]
