# Supplementary figures and images for: Novel Driver Strength Index highlights important cancer genes in TCGA PanCanAtlas patients (part 1 of 2)
Source: PeerJ. 2022 Aug 11;10:e13860. doi: 10.7717/peerj.13860 (PMC9375969; doi:10.7717/peerj.13860)

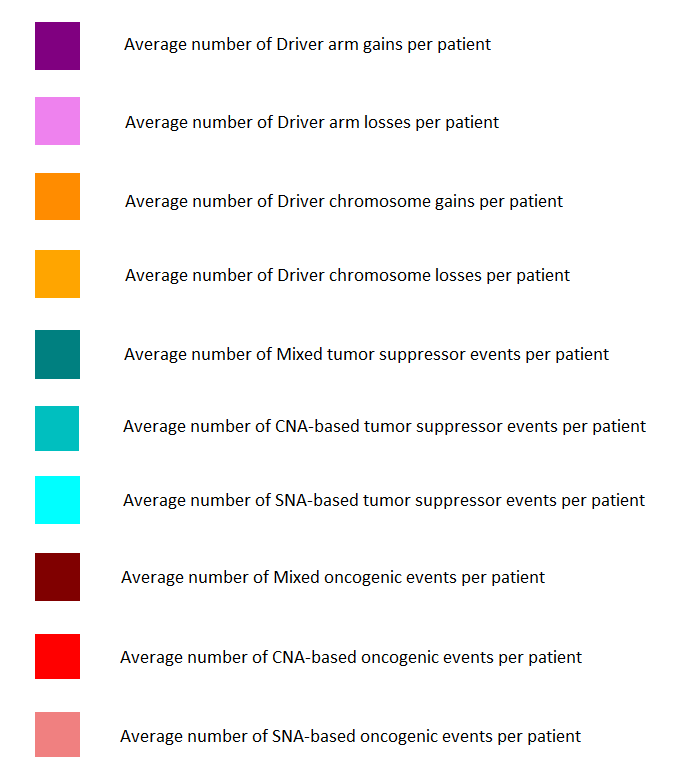

Supplement: Supplemental Information 2 [file peerj-10-13860-s002.zip › COHORTS/cumulative histograms/legeng.png]

Driver event distribution by total number of driver events per patient in males

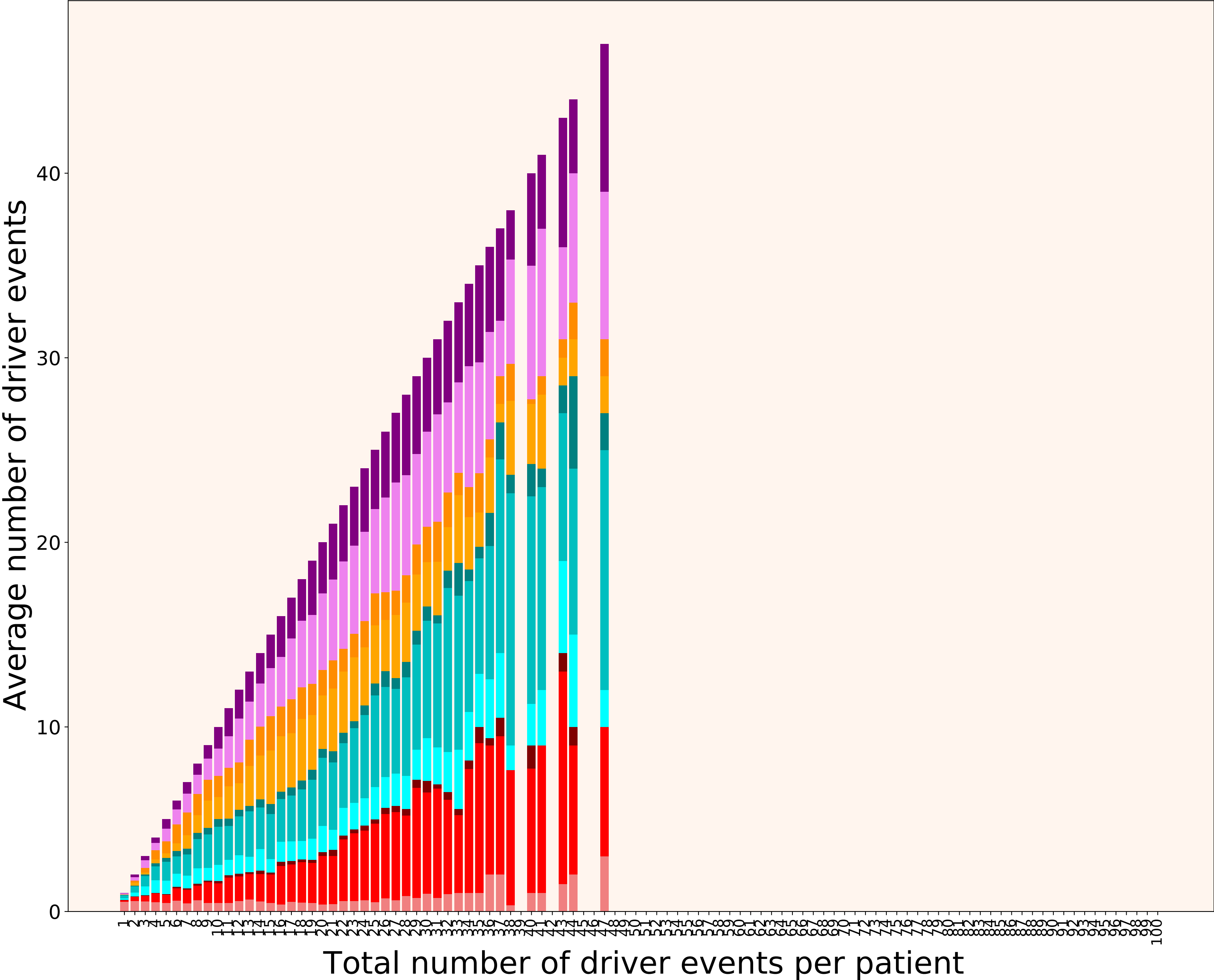

Supplement: Supplemental Information 2 [file peerj-10-13860-s002.zip › COHORTS/cumulative histograms/2021_8_16_14_9_distribution_events_detailed_males.pdf]

Driver event distribution by age in males

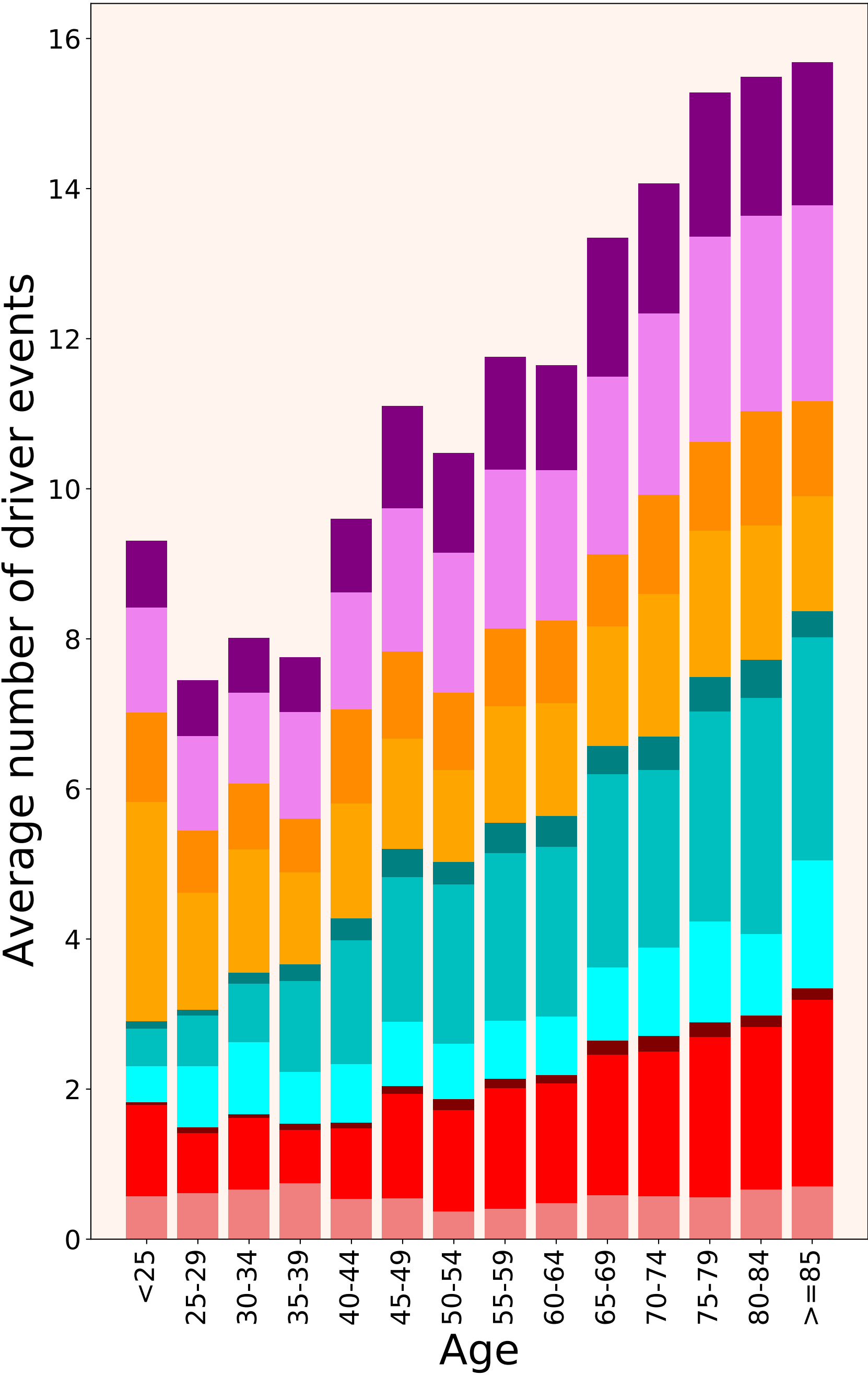

Supplement: Supplemental Information 2 [file peerj-10-13860-s002.zip › COHORTS/cumulative histograms/2021_8_16_14_9_distribution_age_males.pdf]

Driver event distribution by age

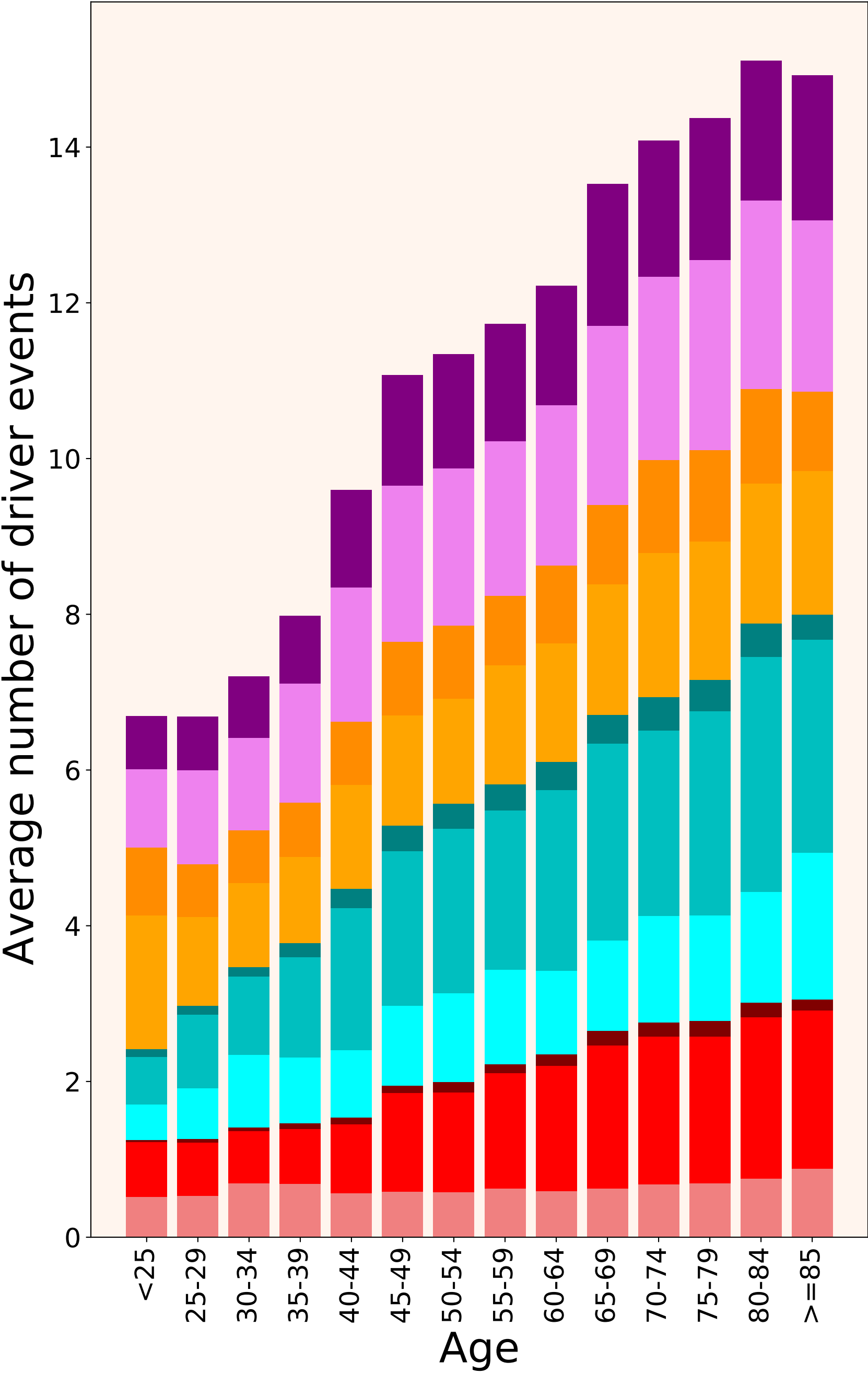

Supplement: Supplemental Information 2 [file peerj-10-13860-s002.zip › COHORTS/cumulative histograms/2021_8_16_14_9_distribution_age.pdf]

Driver event distribution by total number of driver events per patient in females

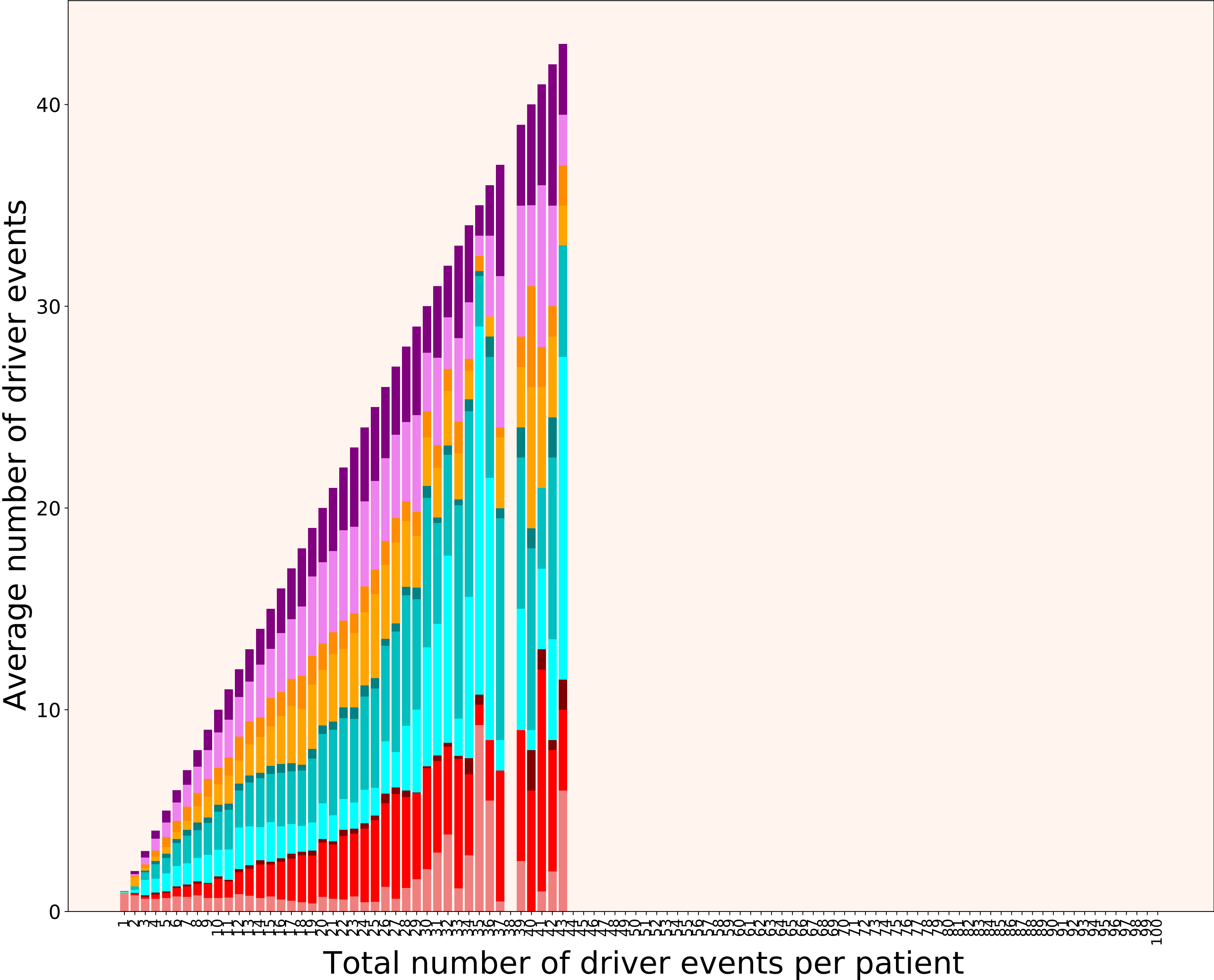

Supplement: Supplemental Information 2 [file peerj-10-13860-s002.zip › COHORTS/cumulative histograms/2021_8_16_14_9_distribution_events_detailed_females.pdf]

Driver event distribution by age in females

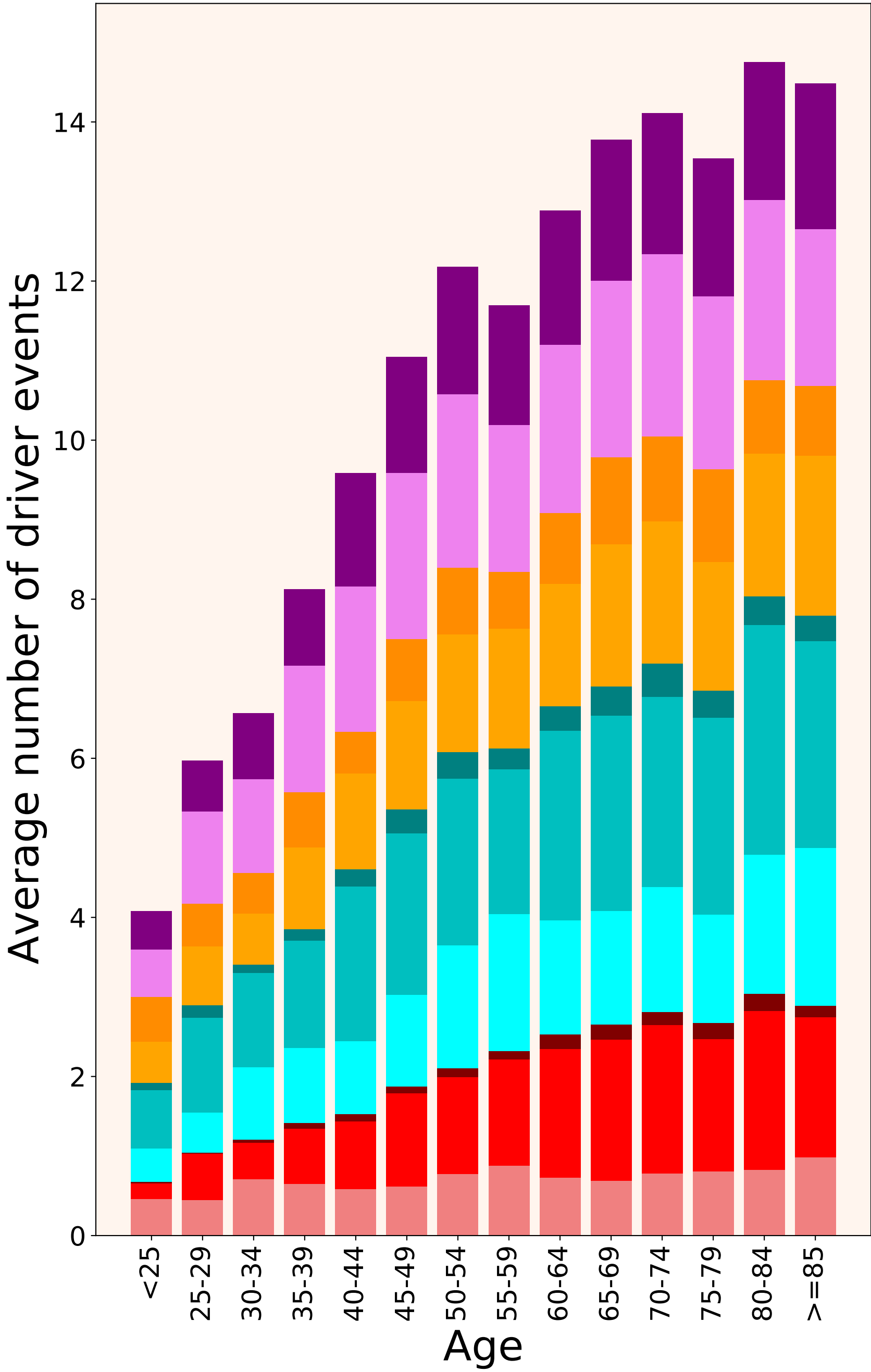

Supplement: Supplemental Information 2 [file peerj-10-13860-s002.zip › COHORTS/cumulative histograms/2021_8_16_14_9_distribution_age_females.pdf]

Driver event distribution by cancer type in males

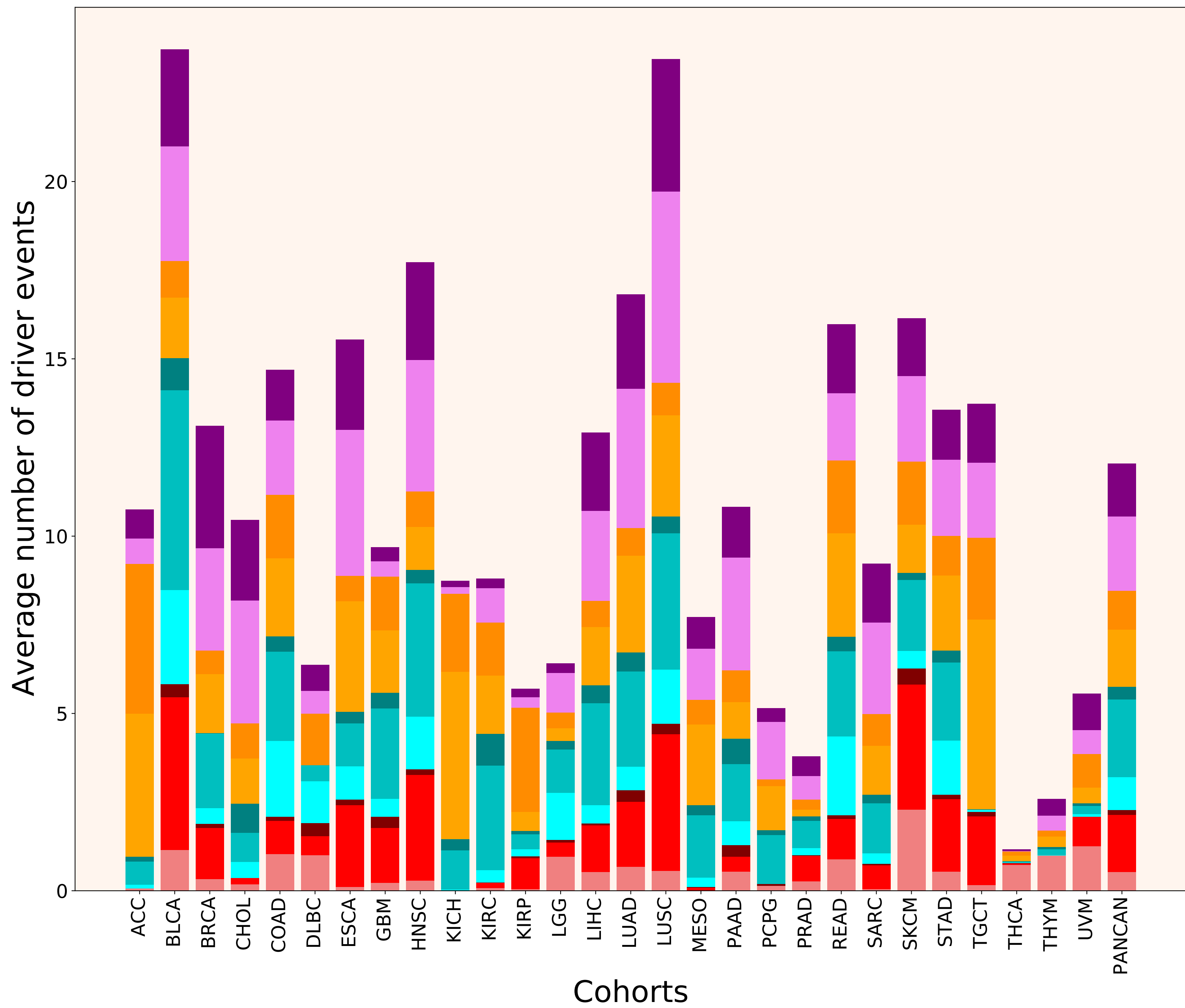

Supplement: Supplemental Information 2 [file peerj-10-13860-s002.zip › COHORTS/cumulative histograms/2021_8_16_14_9_distribution_cohorts_males.pdf]

Driver event distribution by total number of driver events per patient

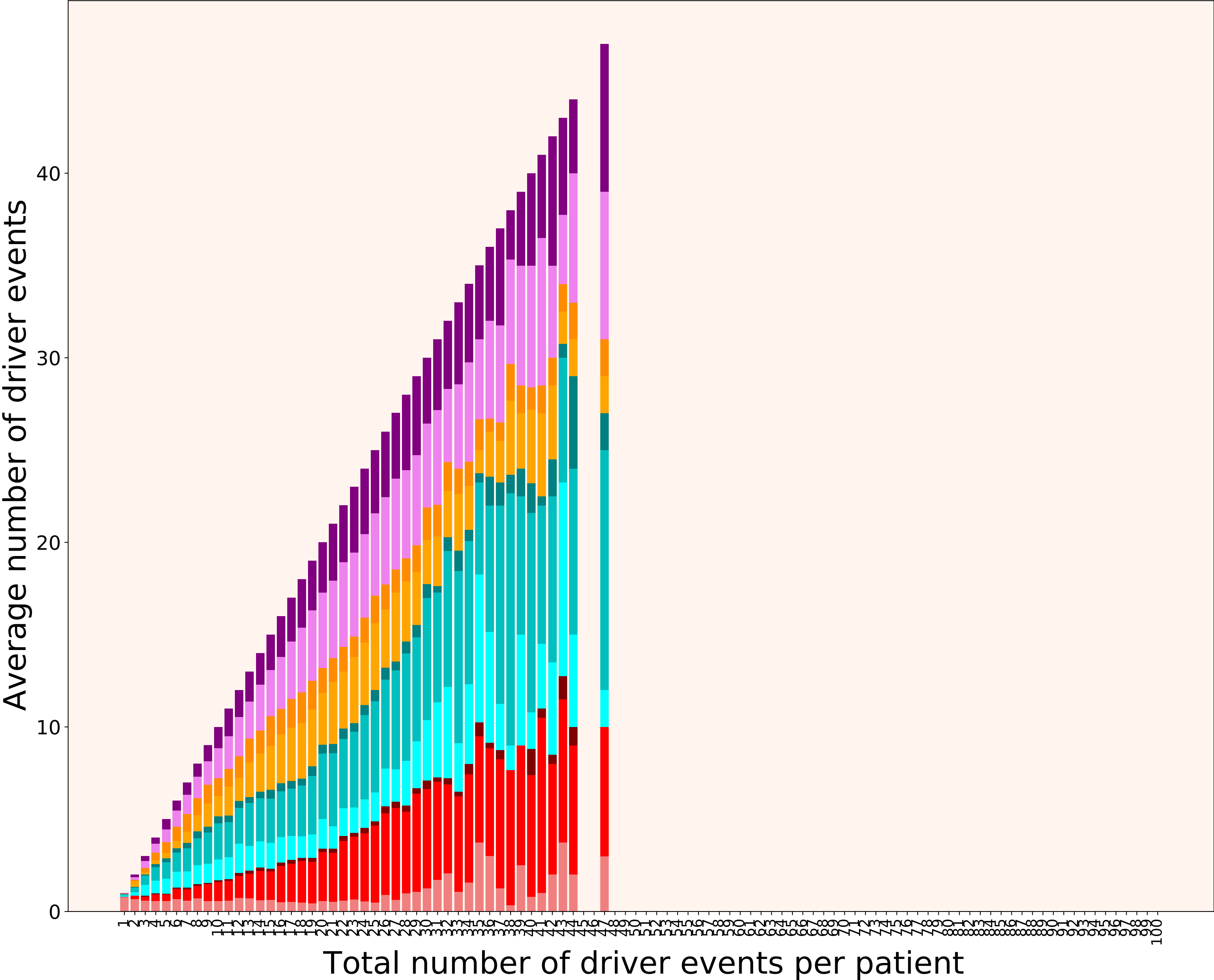

Supplement: Supplemental Information 2 [file peerj-10-13860-s002.zip › COHORTS/cumulative histograms/2021_8_16_14_9_distribution_events_detailed.pdf]

Driver event distribution by cancer stage in males

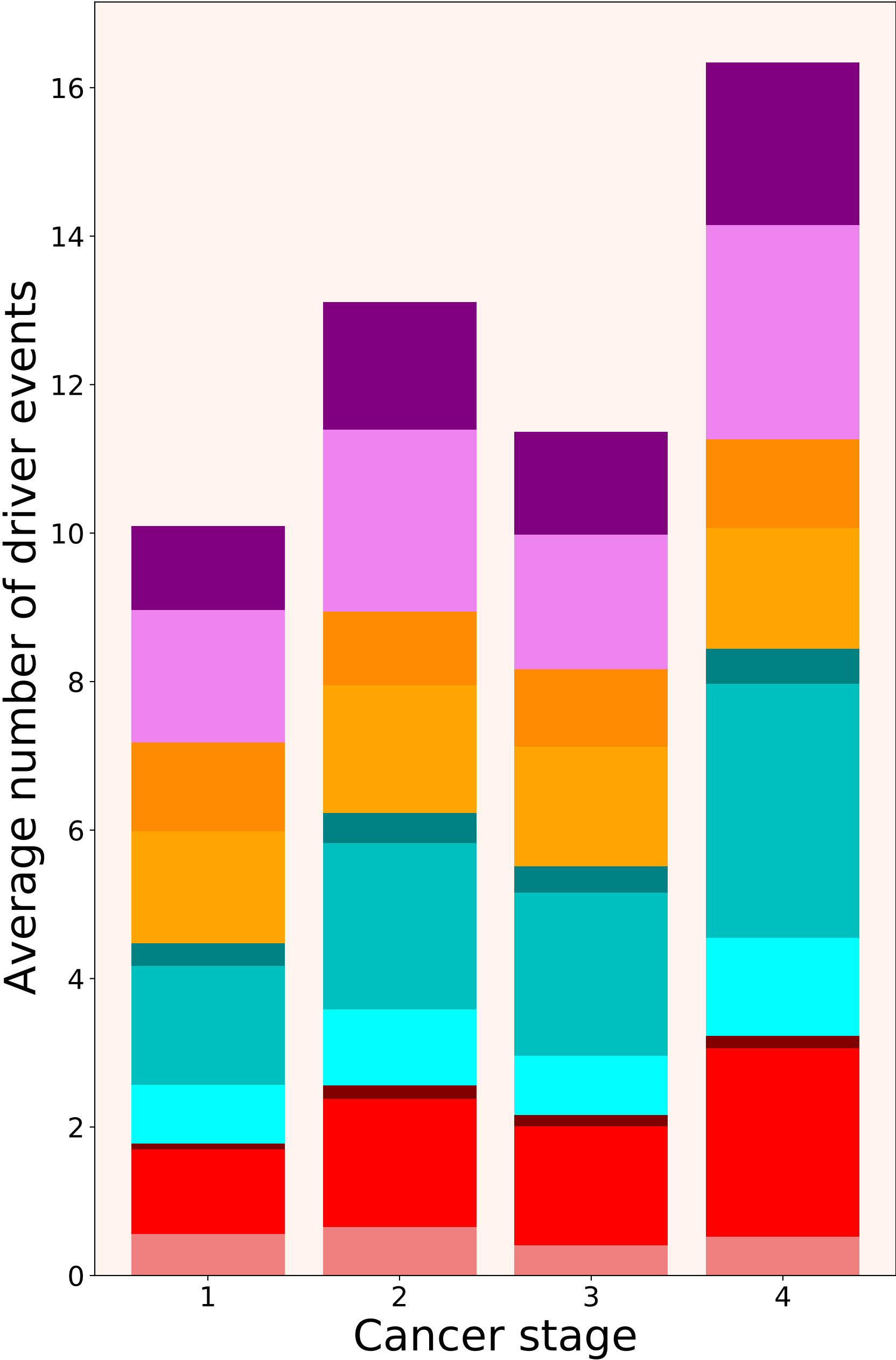

Supplement: Supplemental Information 2 [file peerj-10-13860-s002.zip › COHORTS/cumulative histograms/2021_8_16_14_9_distribution_stages_males.pdf]

Driver event distribution by cancer type in females

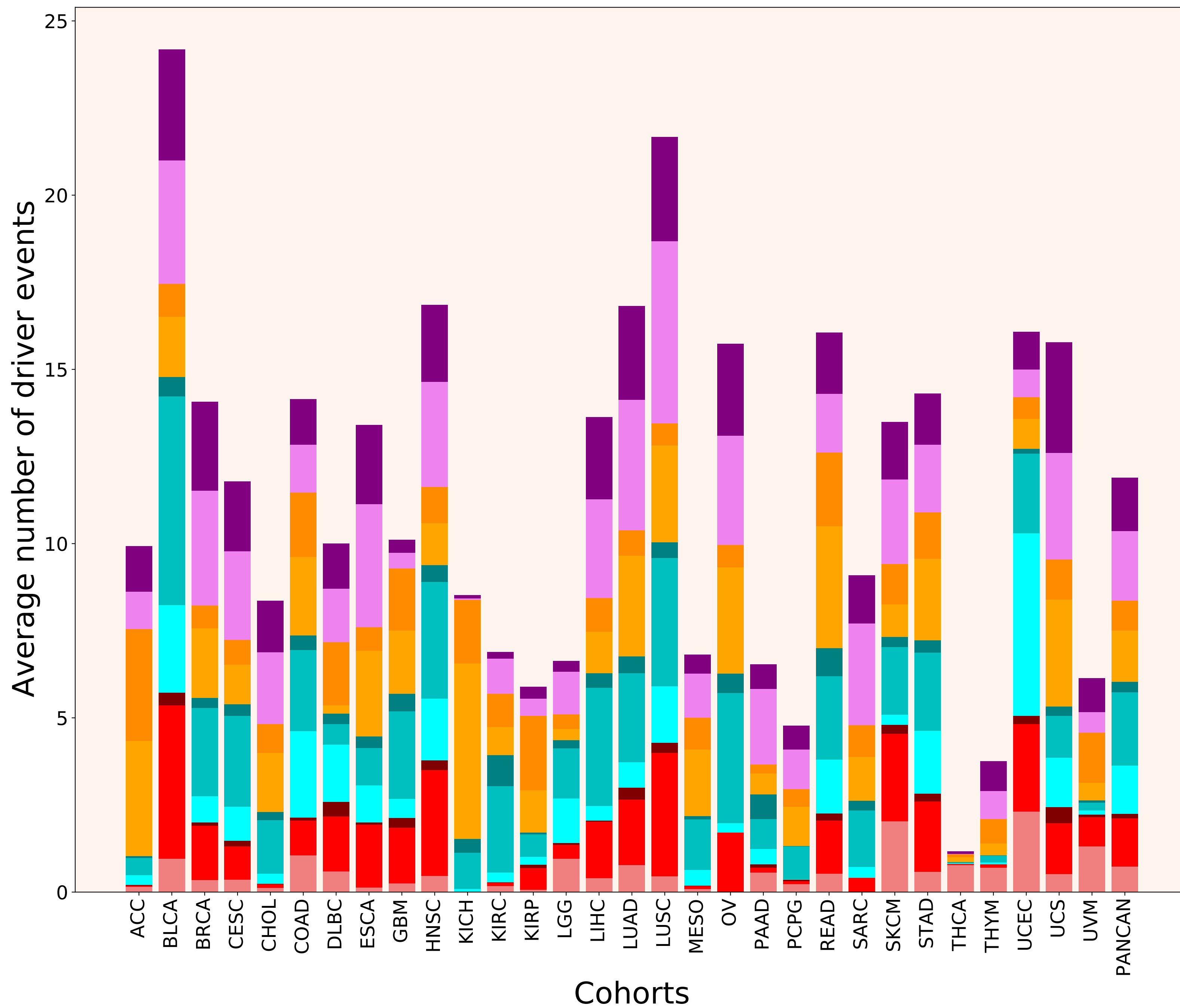

Supplement: Supplemental Information 2 [file peerj-10-13860-s002.zip › COHORTS/cumulative histograms/2021_8_16_14_9_distribution_cohorts_females.pdf]

Driver event distribution by cancer type

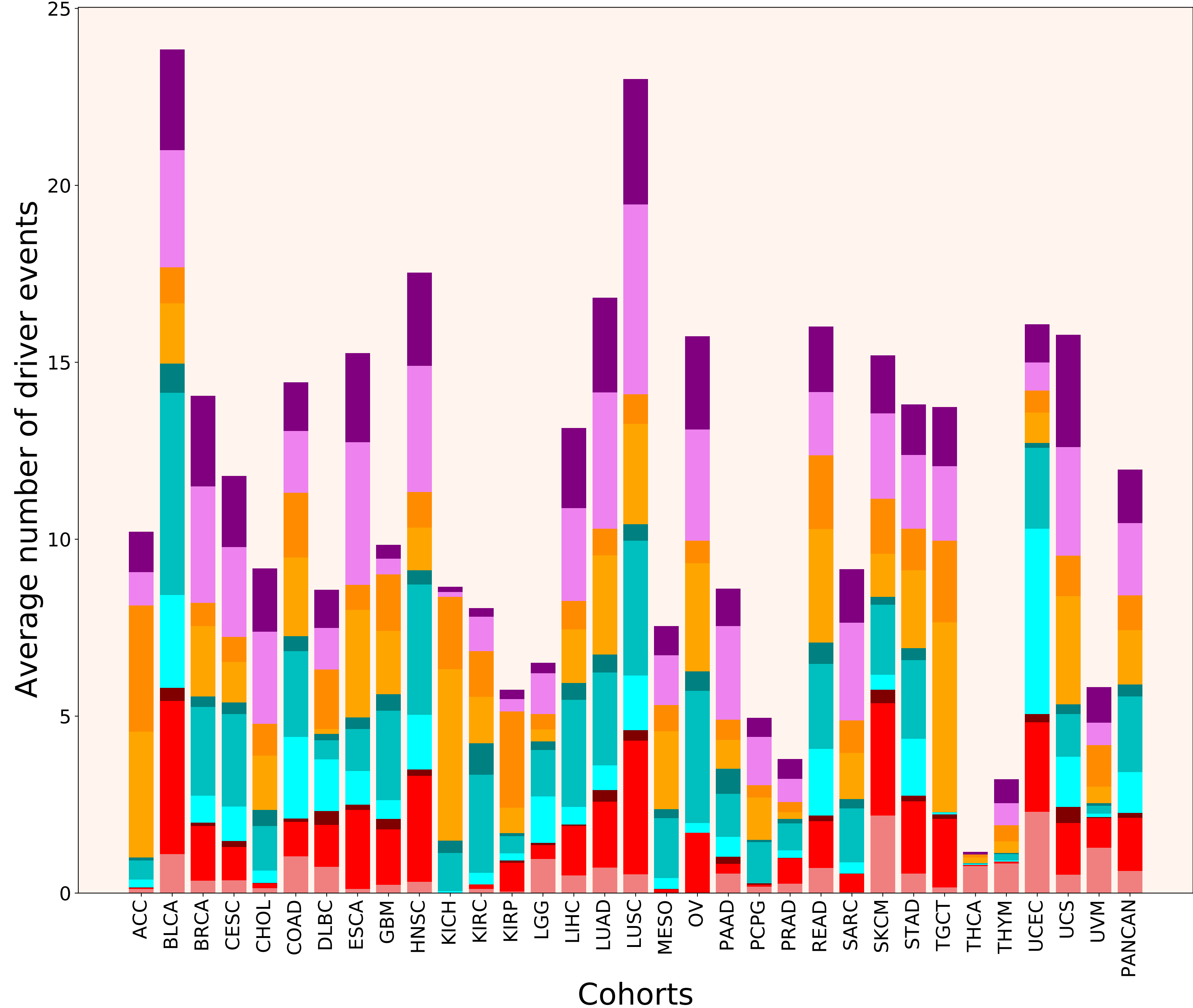

Supplement: Supplemental Information 2 [file peerj-10-13860-s002.zip › COHORTS/cumulative histograms/2021_8_16_14_9_distribution_cohorts.pdf]

Driver event distribution by cancer stage

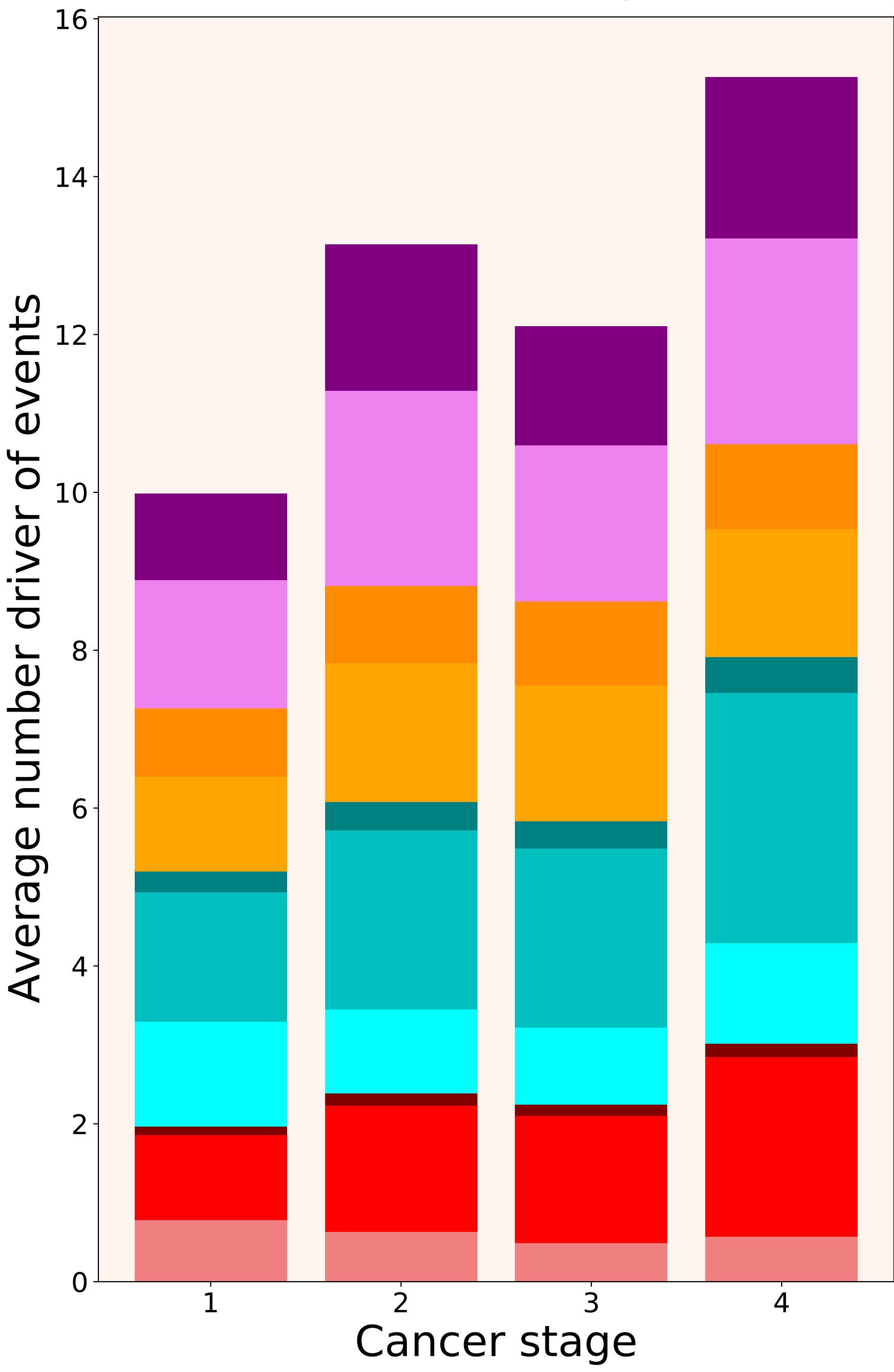

Supplement: Supplemental Information 2 [file peerj-10-13860-s002.zip › COHORTS/cumulative histograms/2021_8_16_14_9_distribution_stages.pdf]

Driver event distribution by gender

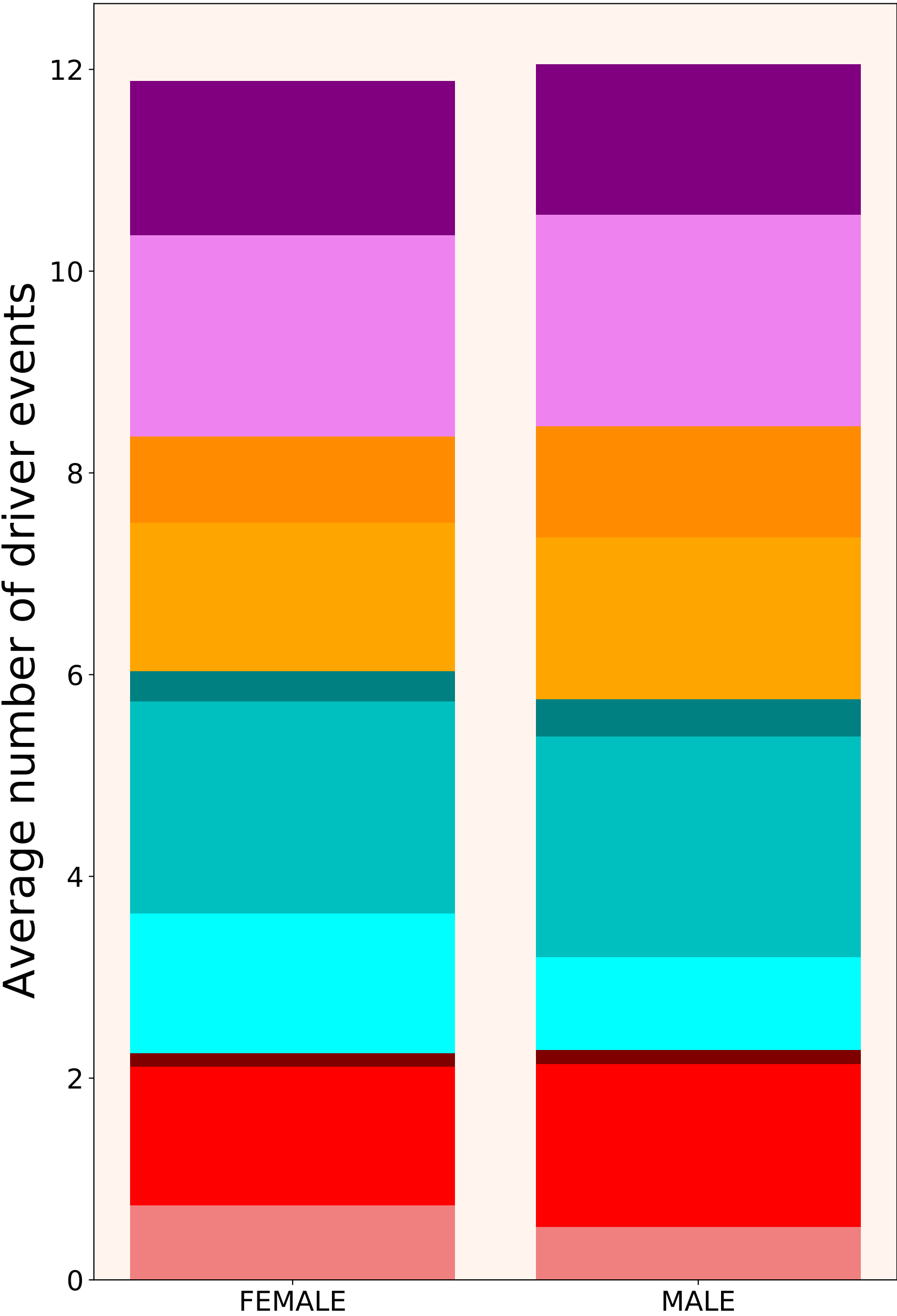

Supplement: Supplemental Information 2 [file peerj-10-13860-s002.zip › COHORTS/cumulative histograms/2021_8_16_14_9_distribution_gender.pdf]

Driver event distribution by cancer stage in females

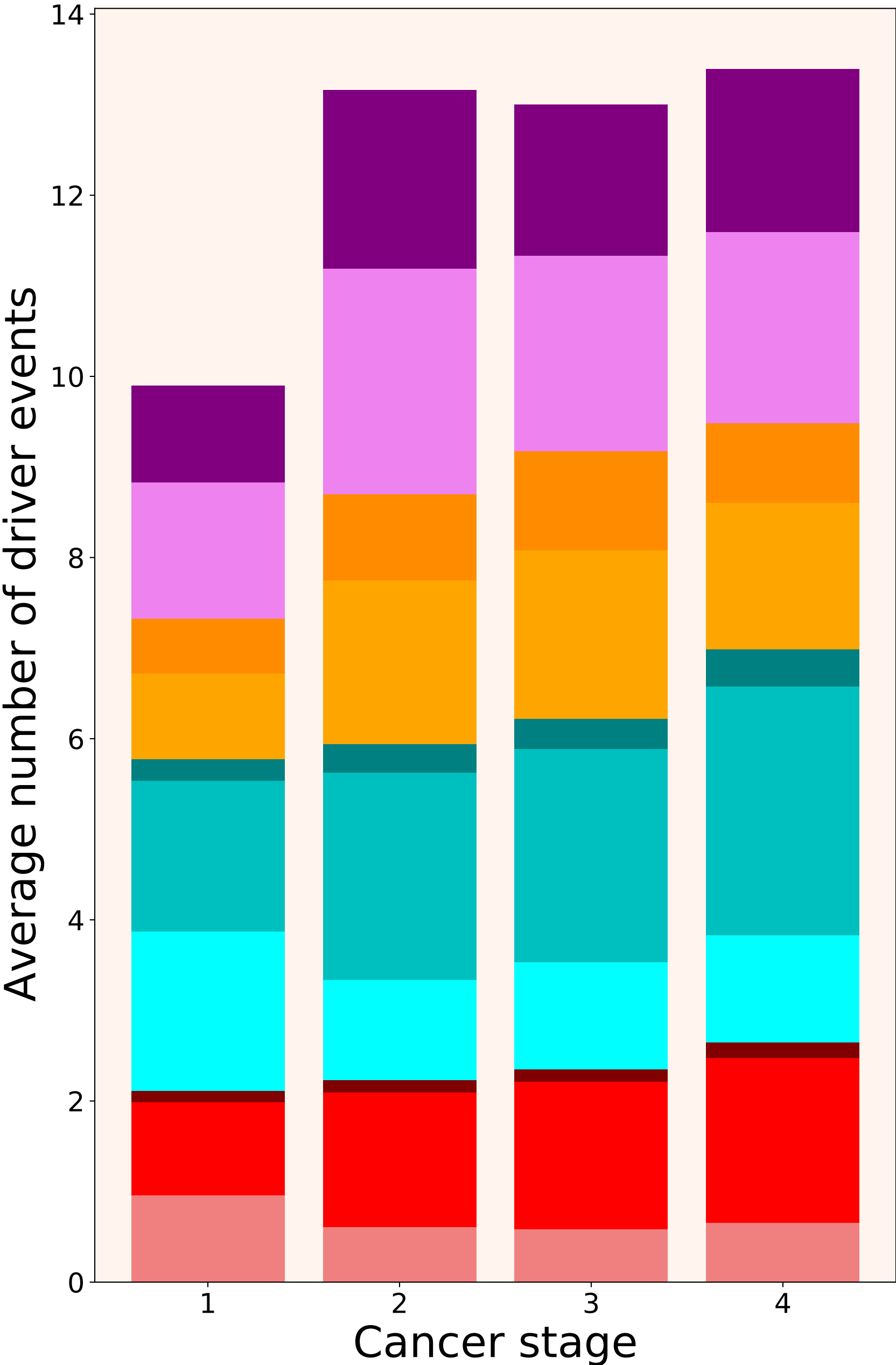

Supplement: Supplemental Information 2 [file peerj-10-13860-s002.zip › COHORTS/cumulative histograms/2021_8_16_14_9_distribution_stages_females.pdf]

# TGCT\_MALE

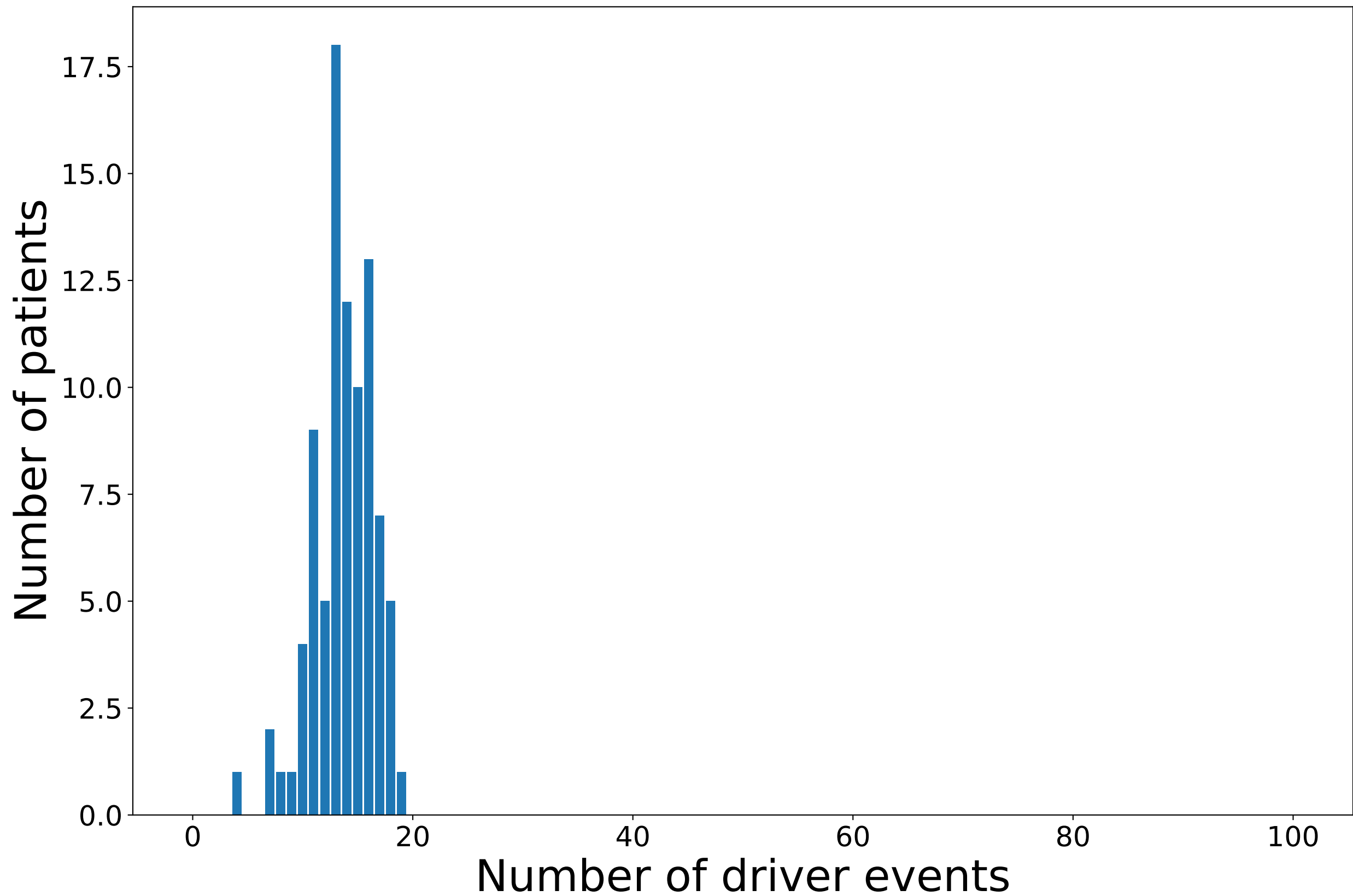

Supplement: Supplemental Information 2 [file peerj-10-13860-s002.zip › COHORTS/patient distributions/2021_8_16_14_9_TGCT_MALE.pdf]

# BRCA

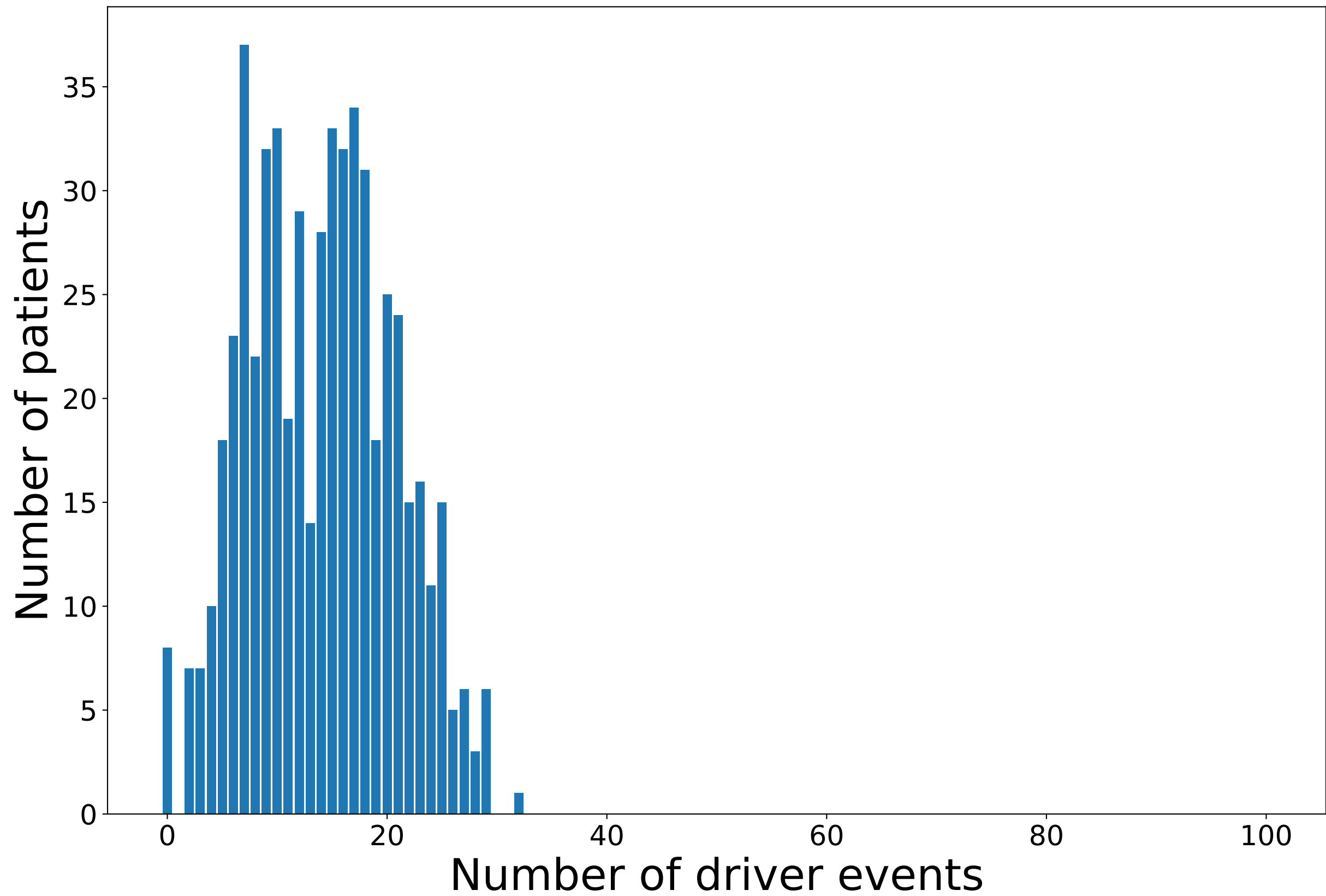

Supplement: Supplemental Information 2 [file peerj-10-13860-s002.zip › COHORTS/patient distributions/2021_8_16_14_9_BRCA.pdf]

# UVM

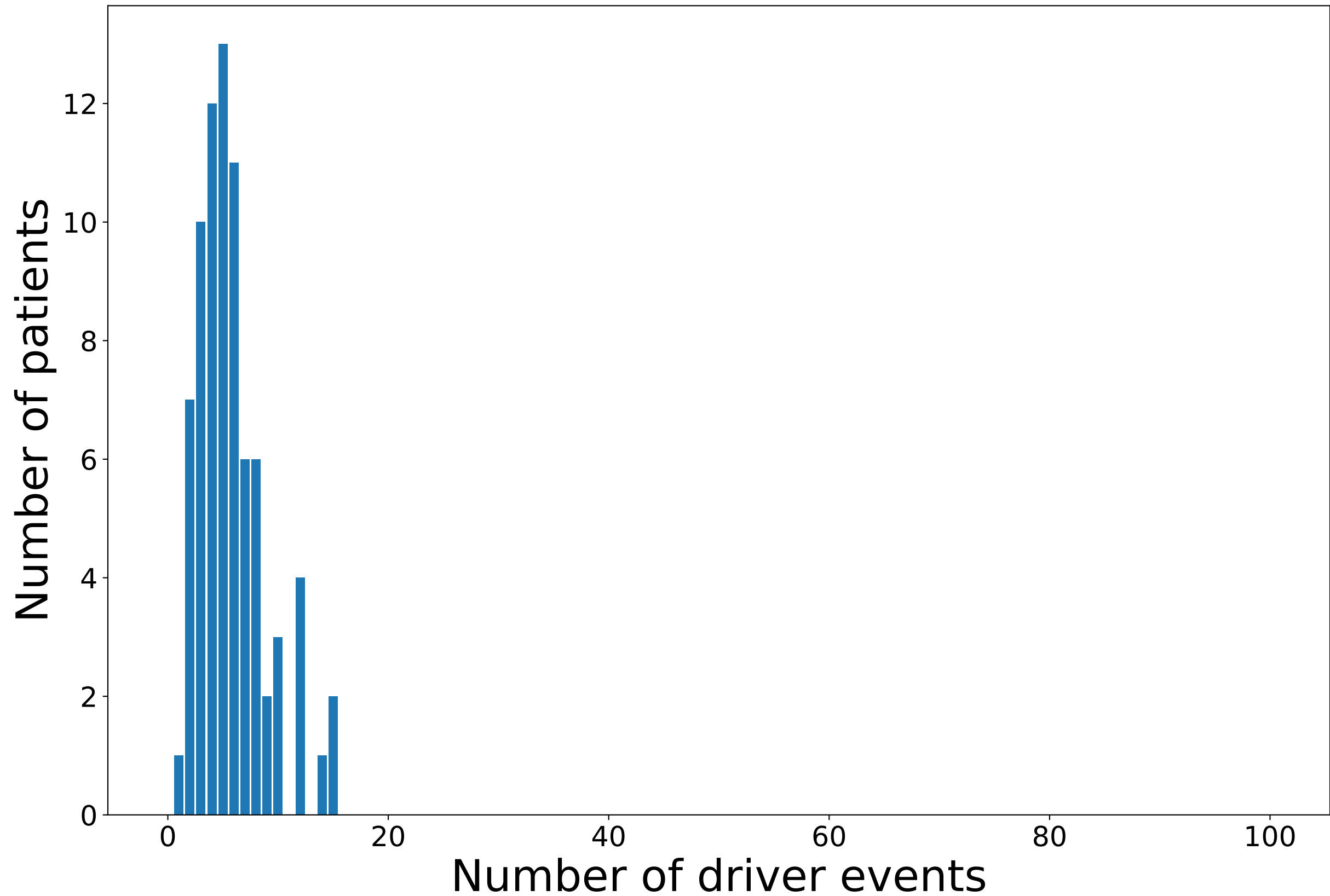

Supplement: Supplemental Information 2 [file peerj-10-13860-s002.zip › COHORTS/patient distributions/2021_8_16_14_9_UVM.pdf]

# CHOL\_FEMALE

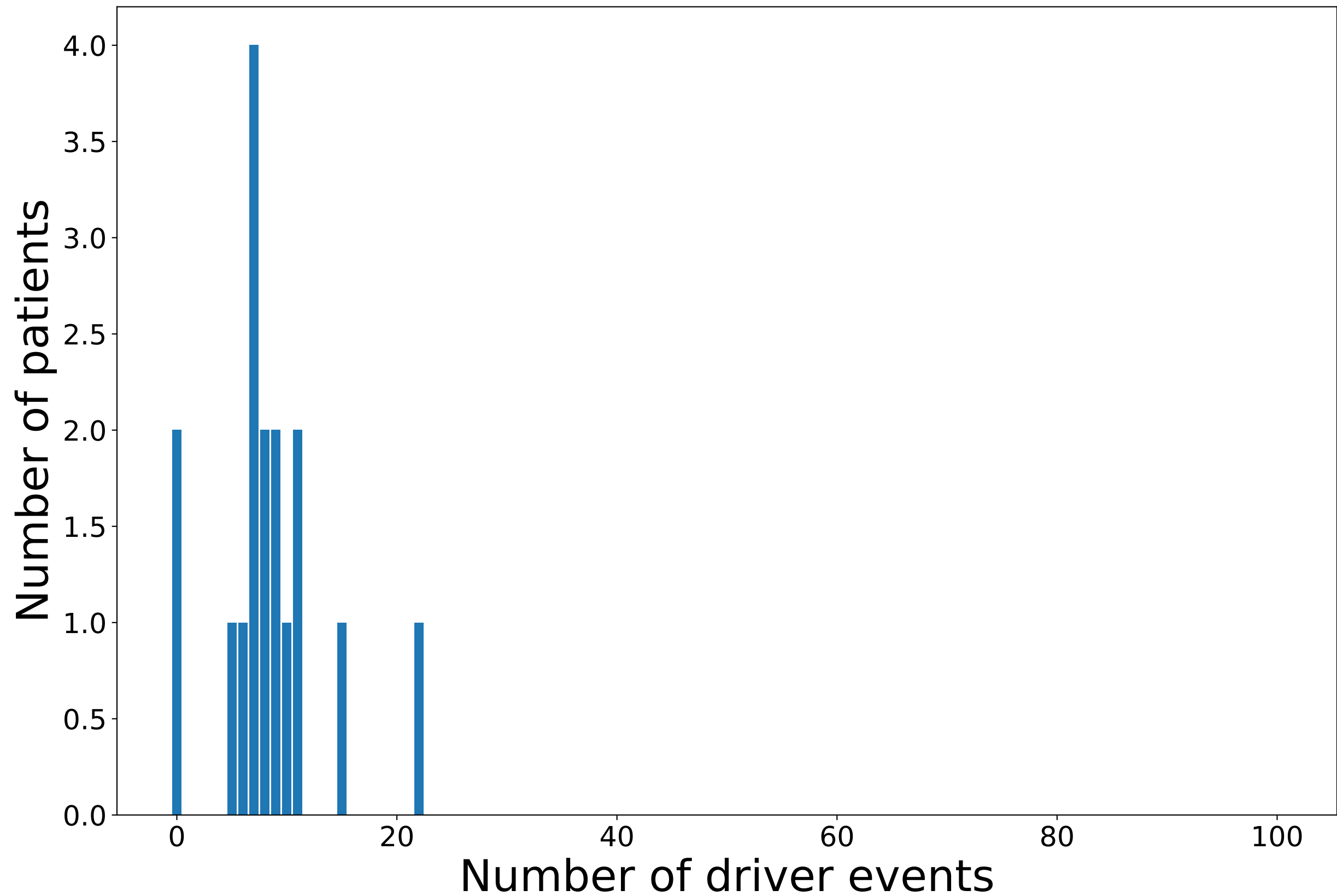

Supplement: Supplemental Information 2 [file peerj-10-13860-s002.zip › COHORTS/patient distributions/2021_8_16_14_9_CHOL_FEMALE.pdf]

OV

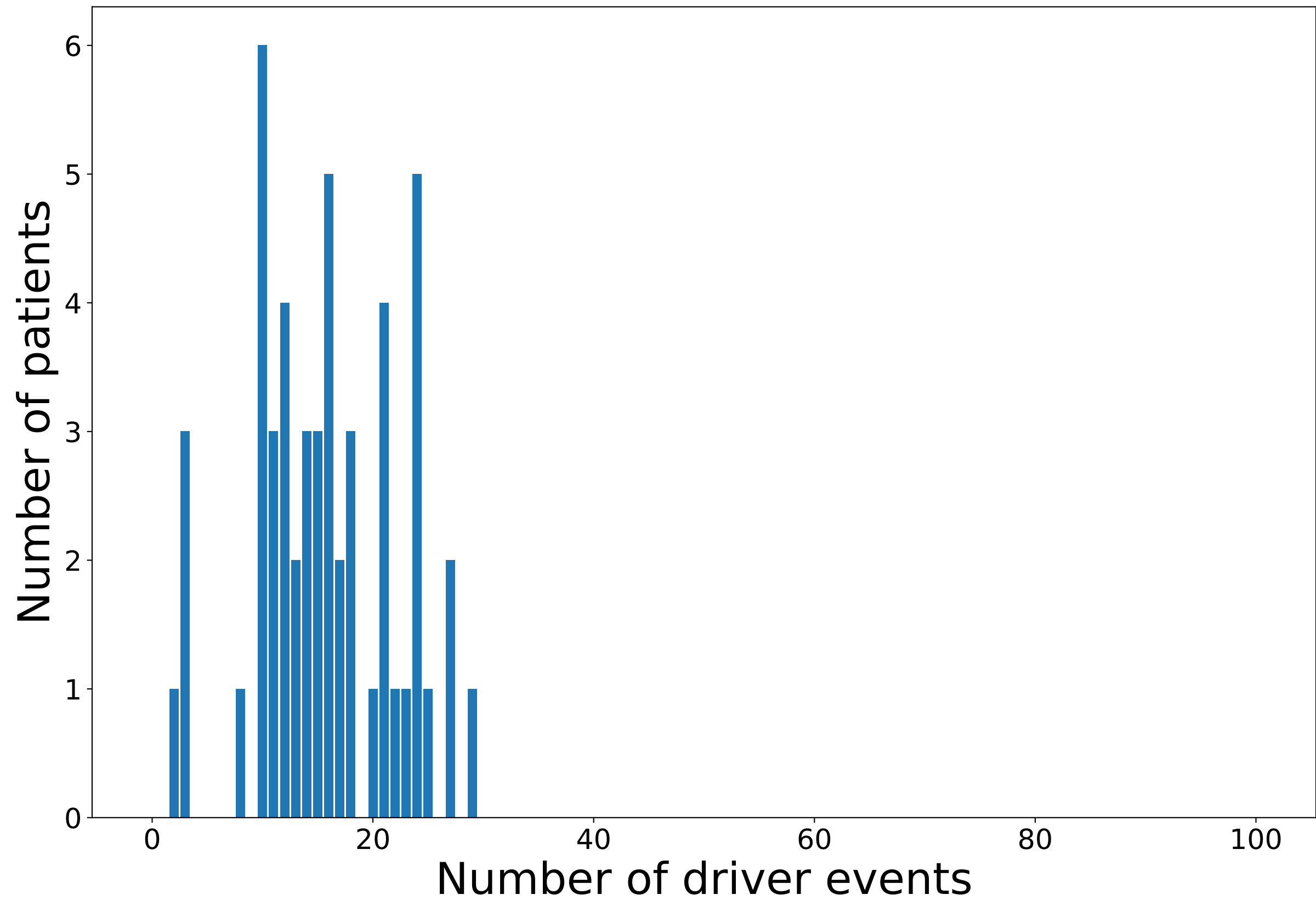

Supplement: Supplemental Information 2 [file peerj-10-13860-s002.zip › COHORTS/patient distributions/2021_8_16_14_9_OV.pdf]

# KIRP\_FEMALE

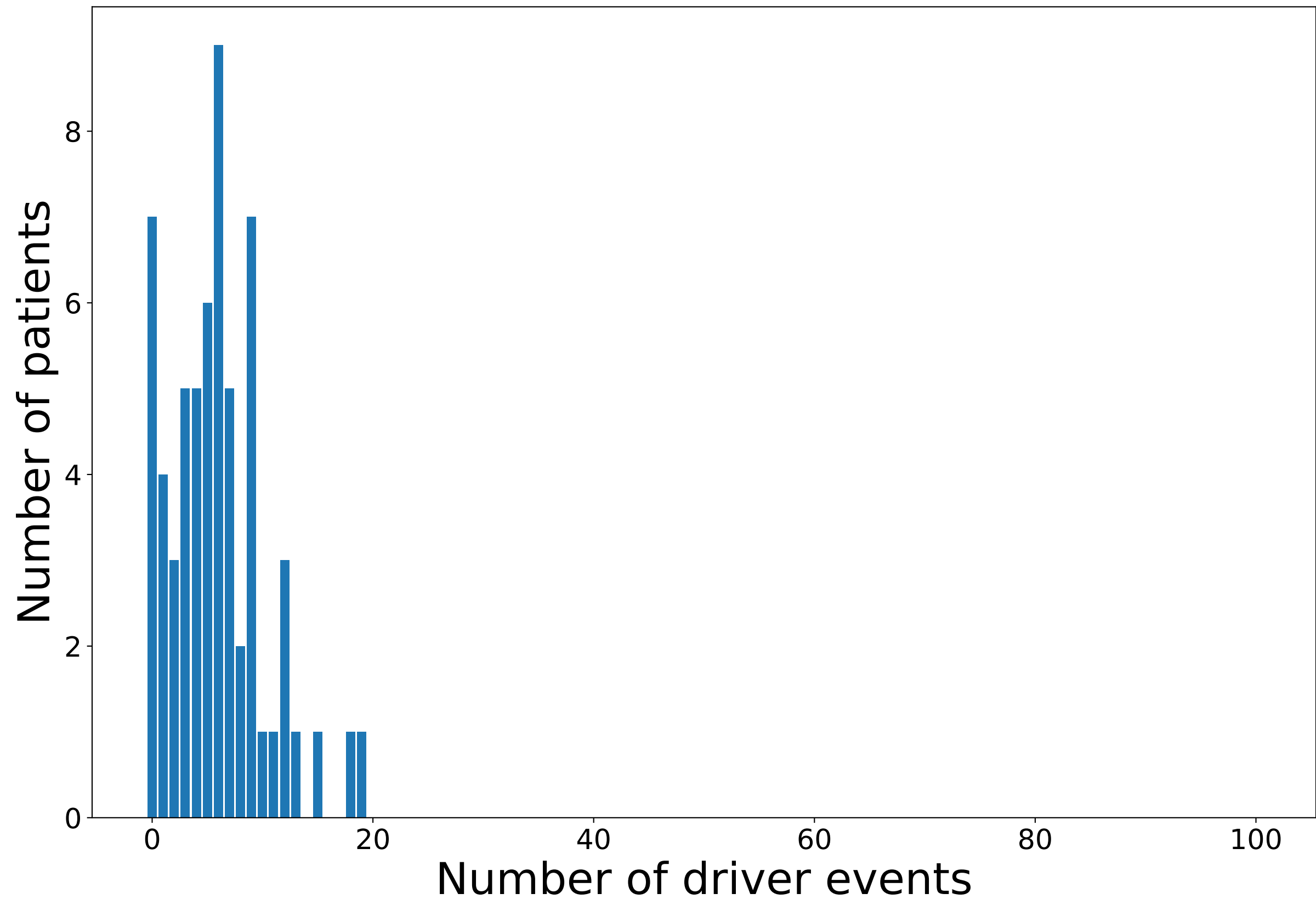

Supplement: Supplemental Information 2 [file peerj-10-13860-s002.zip › COHORTS/patient distributions/2021_8_16_14_9_KIRP_FEMALE.pdf]

# MESO\_MALE

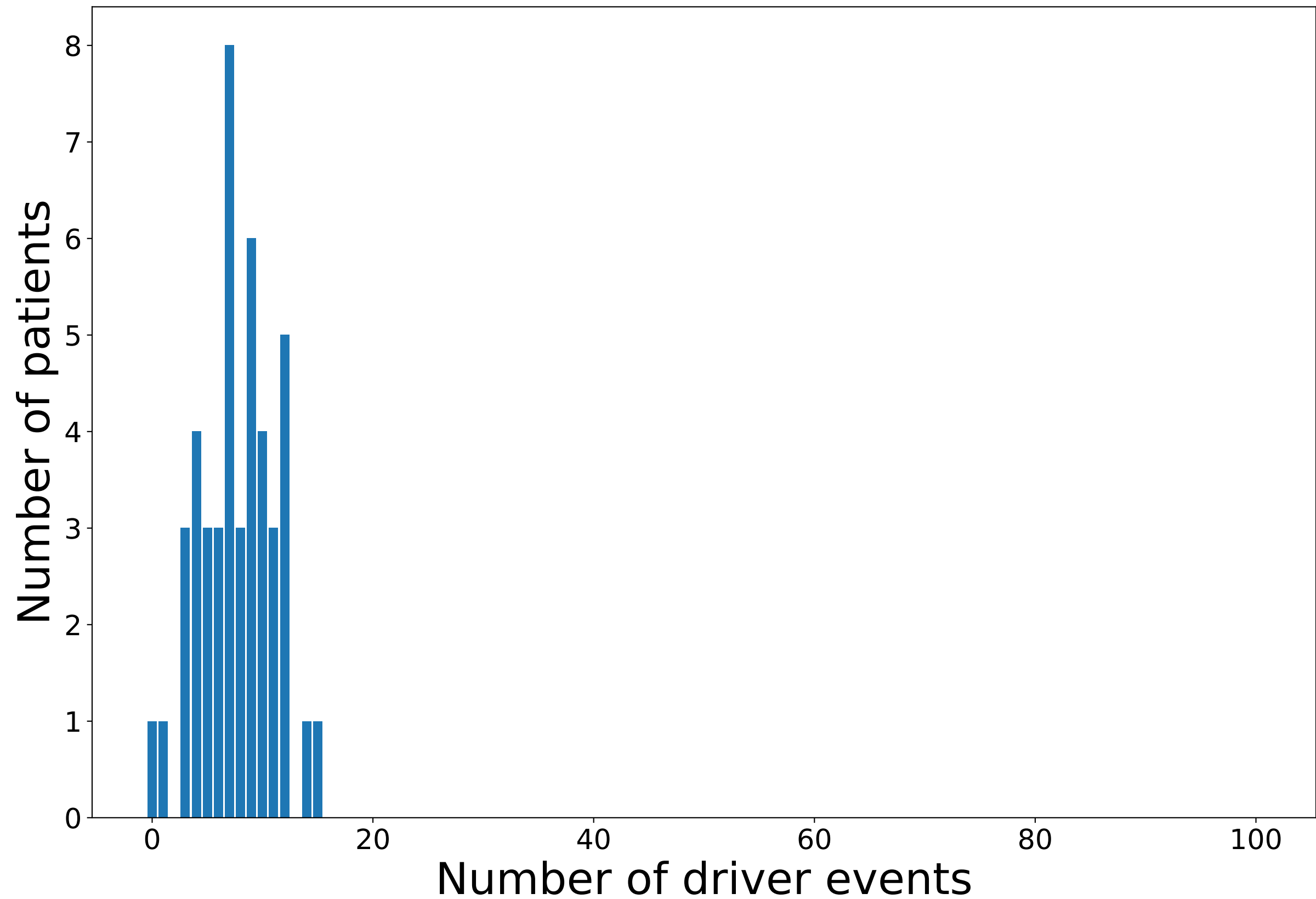

Supplement: Supplemental Information 2 [file peerj-10-13860-s002.zip › COHORTS/patient distributions/2021_8_16_14_9_MESO_MALE.pdf]

# BLCA\_FEMALE

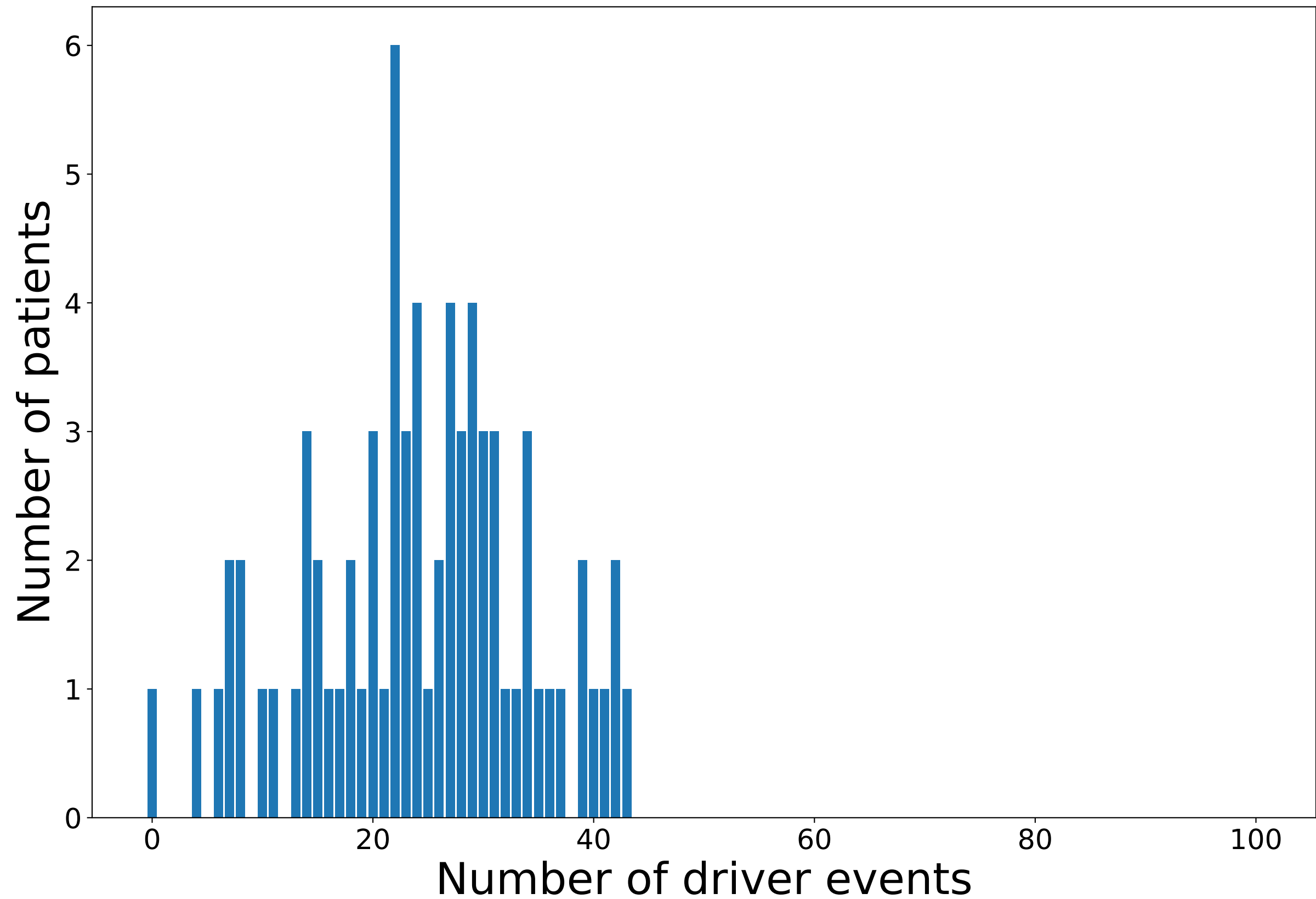

Supplement: Supplemental Information 2 [file peerj-10-13860-s002.zip › COHORTS/patient distributions/2021_8_16_14_9_BLCA_FEMALE.pdf]

# SKCM

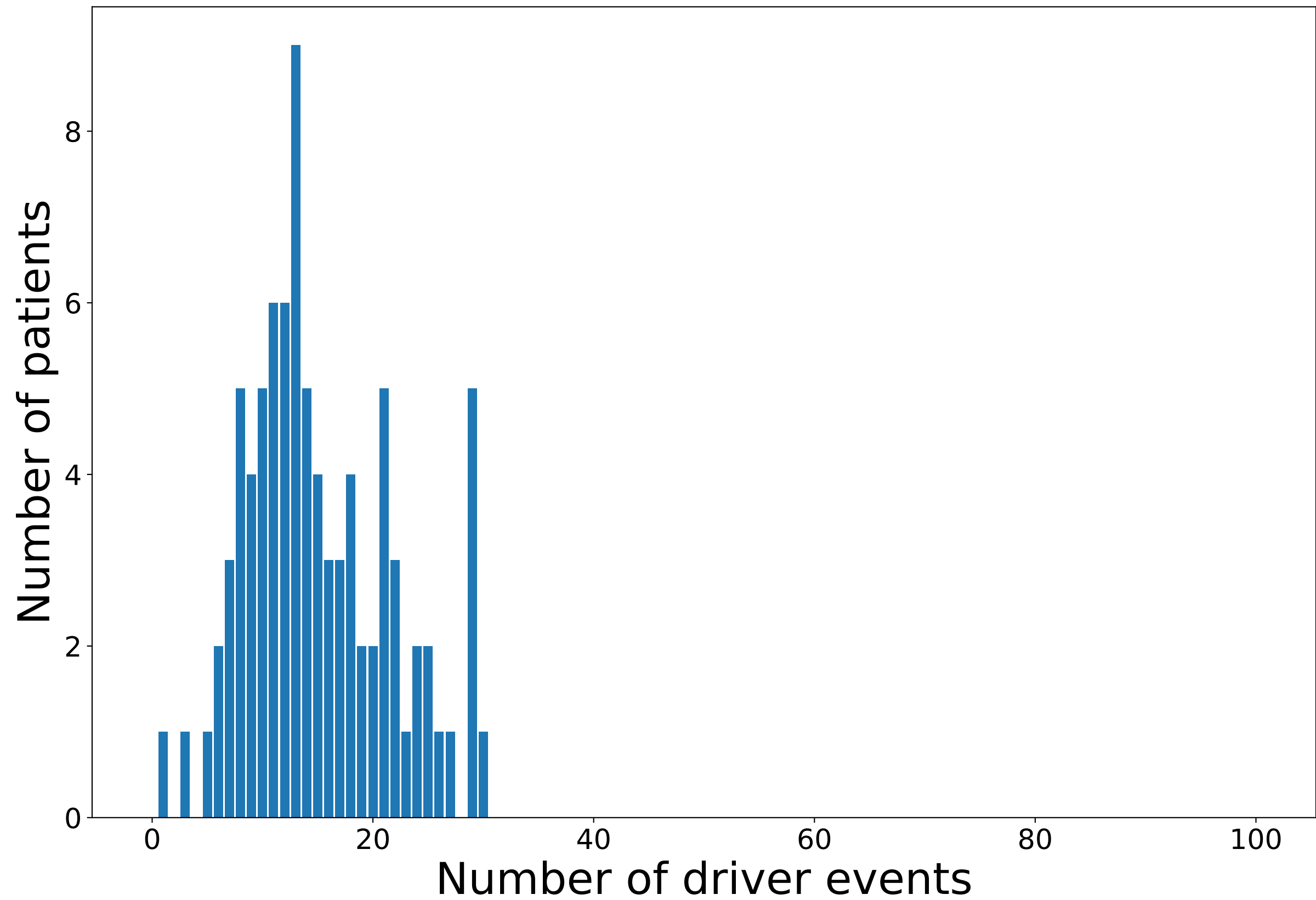

Supplement: Supplemental Information 2 [file peerj-10-13860-s002.zip › COHORTS/patient distributions/2021_8_16_14_9_SKCM.pdf]

# GBM\_MALE

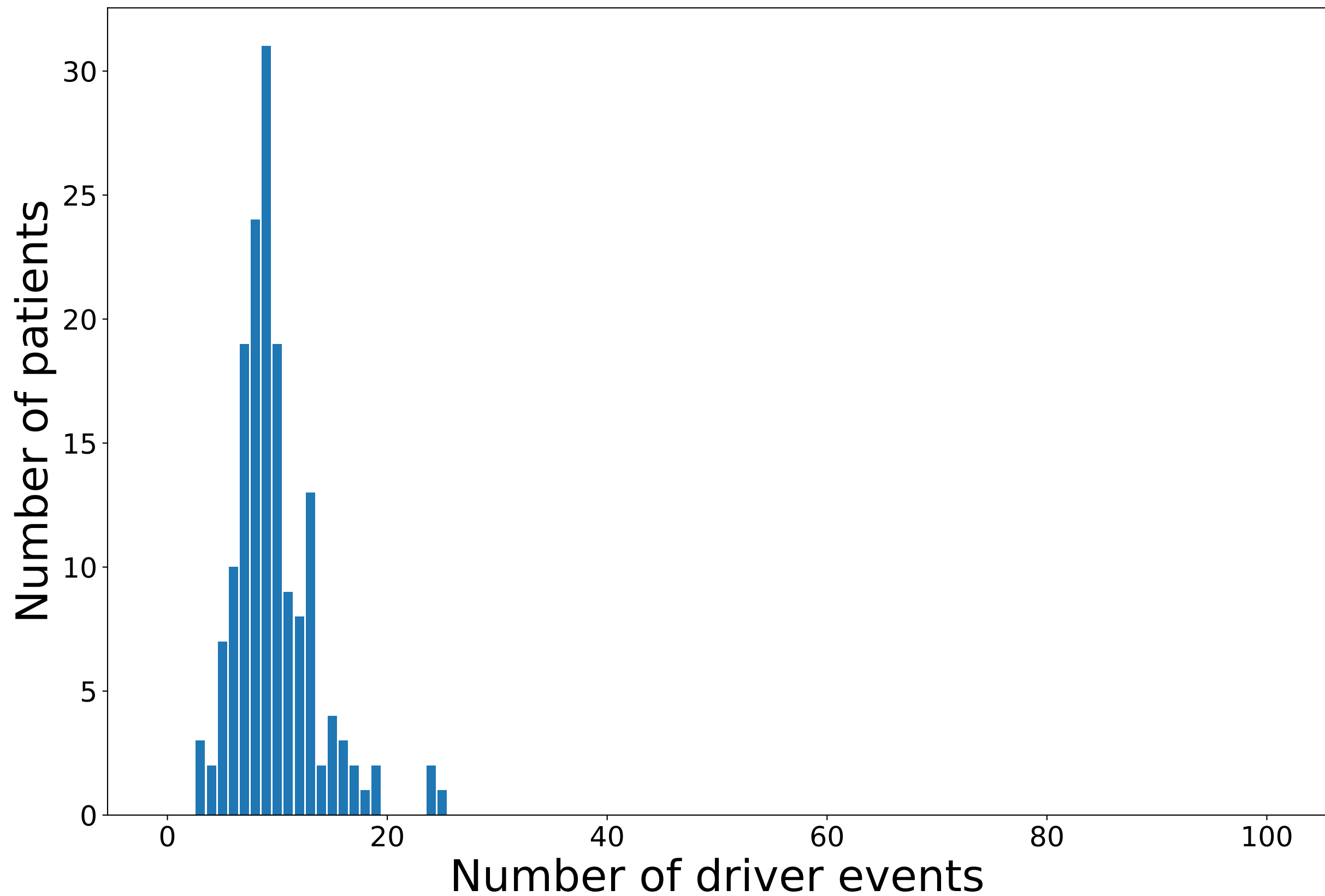

Supplement: Supplemental Information 2 [file peerj-10-13860-s002.zip › COHORTS/patient distributions/2021_8_16_14_9_GBM_MALE.pdf]

# MESO

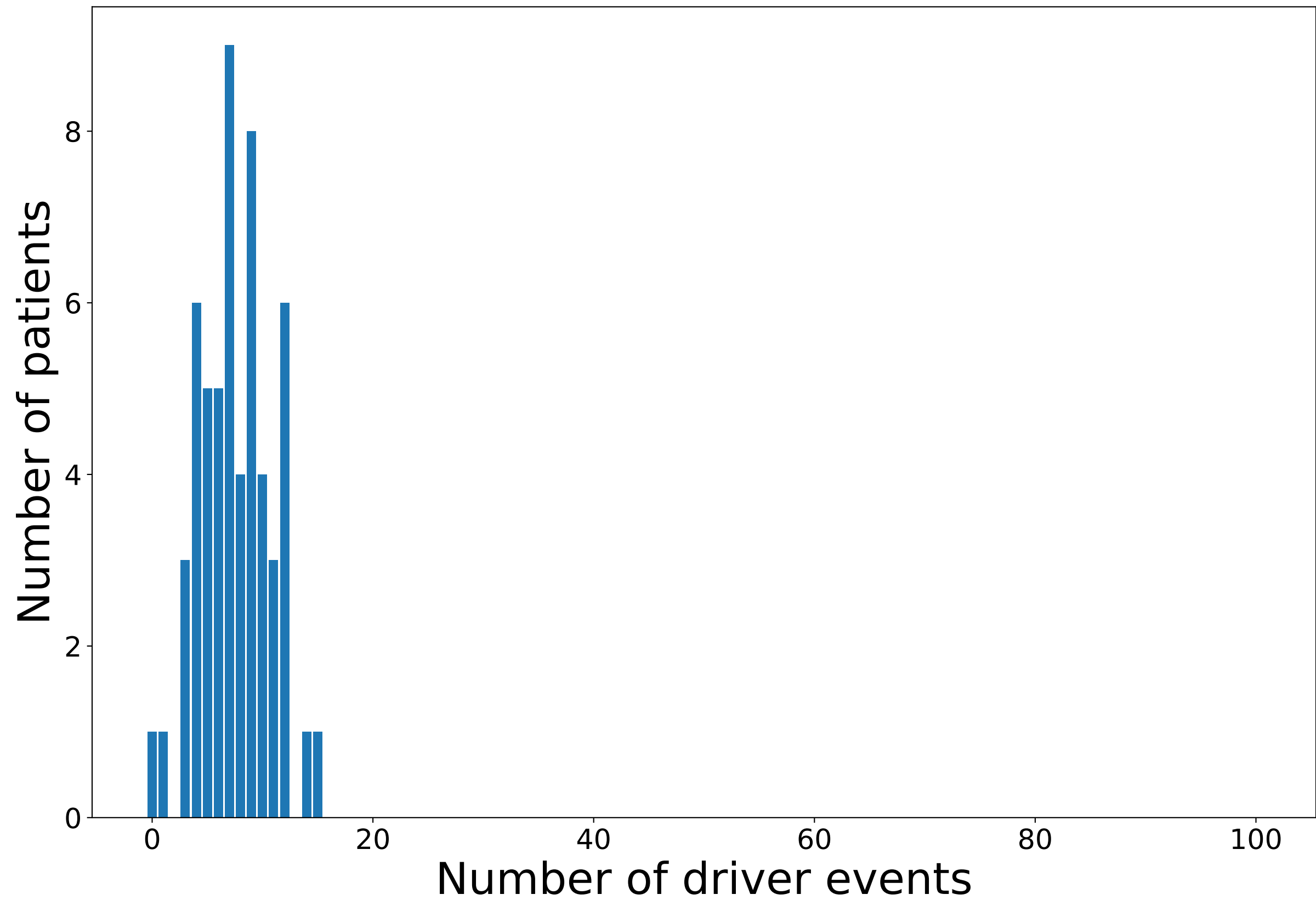

Supplement: Supplemental Information 2 [file peerj-10-13860-s002.zip › COHORTS/patient distributions/2021_8_16_14_9_MESO.pdf]

# DLBC

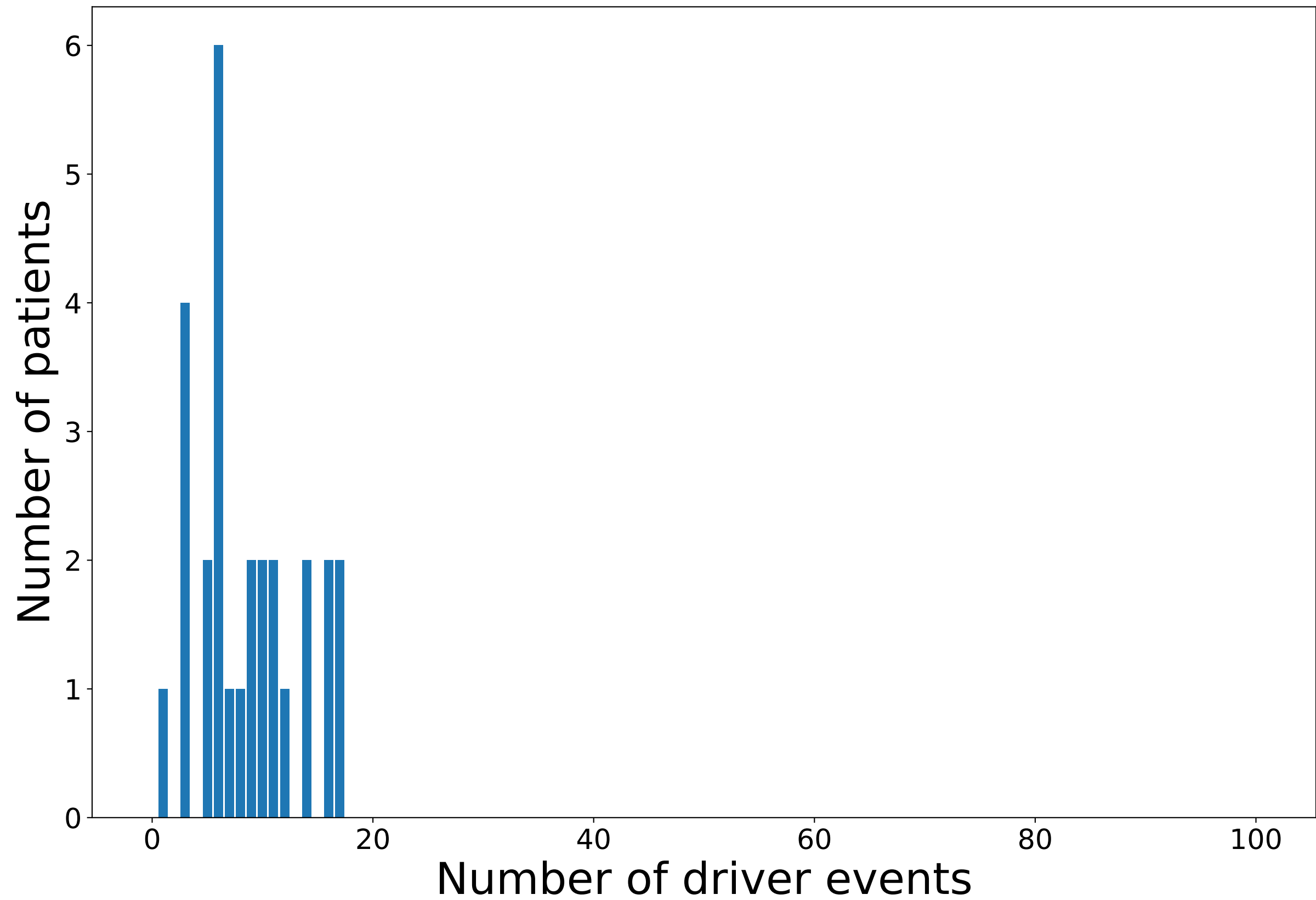

Supplement: Supplemental Information 2 [file peerj-10-13860-s002.zip › COHORTS/patient distributions/2021_8_16_14_9_DLBC.pdf]

# SKCM\_MALE

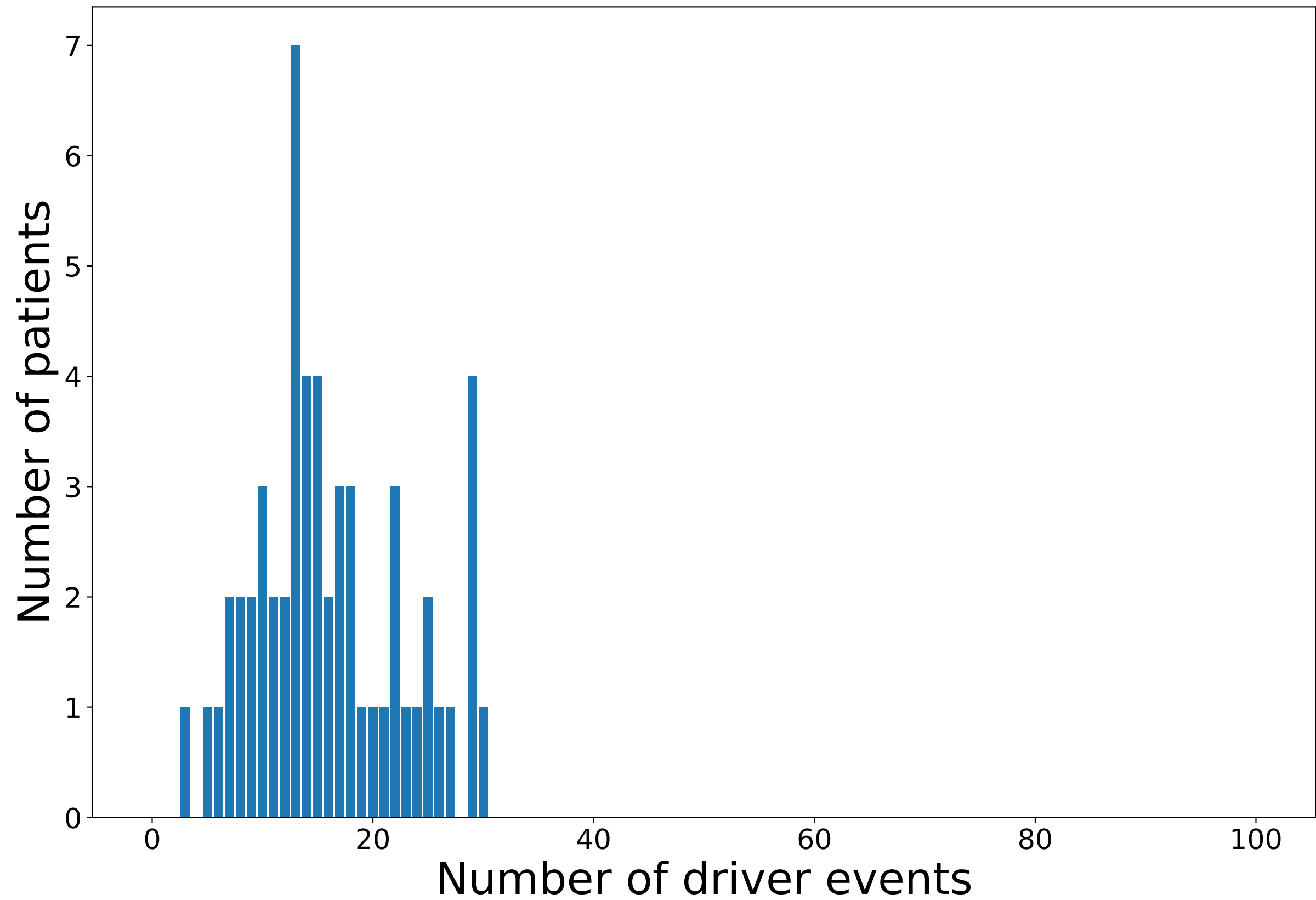

Supplement: Supplemental Information 2 [file peerj-10-13860-s002.zip › COHORTS/patient distributions/2021_8_16_14_9_SKCM_MALE.pdf]

# LIHC\_FEMALE

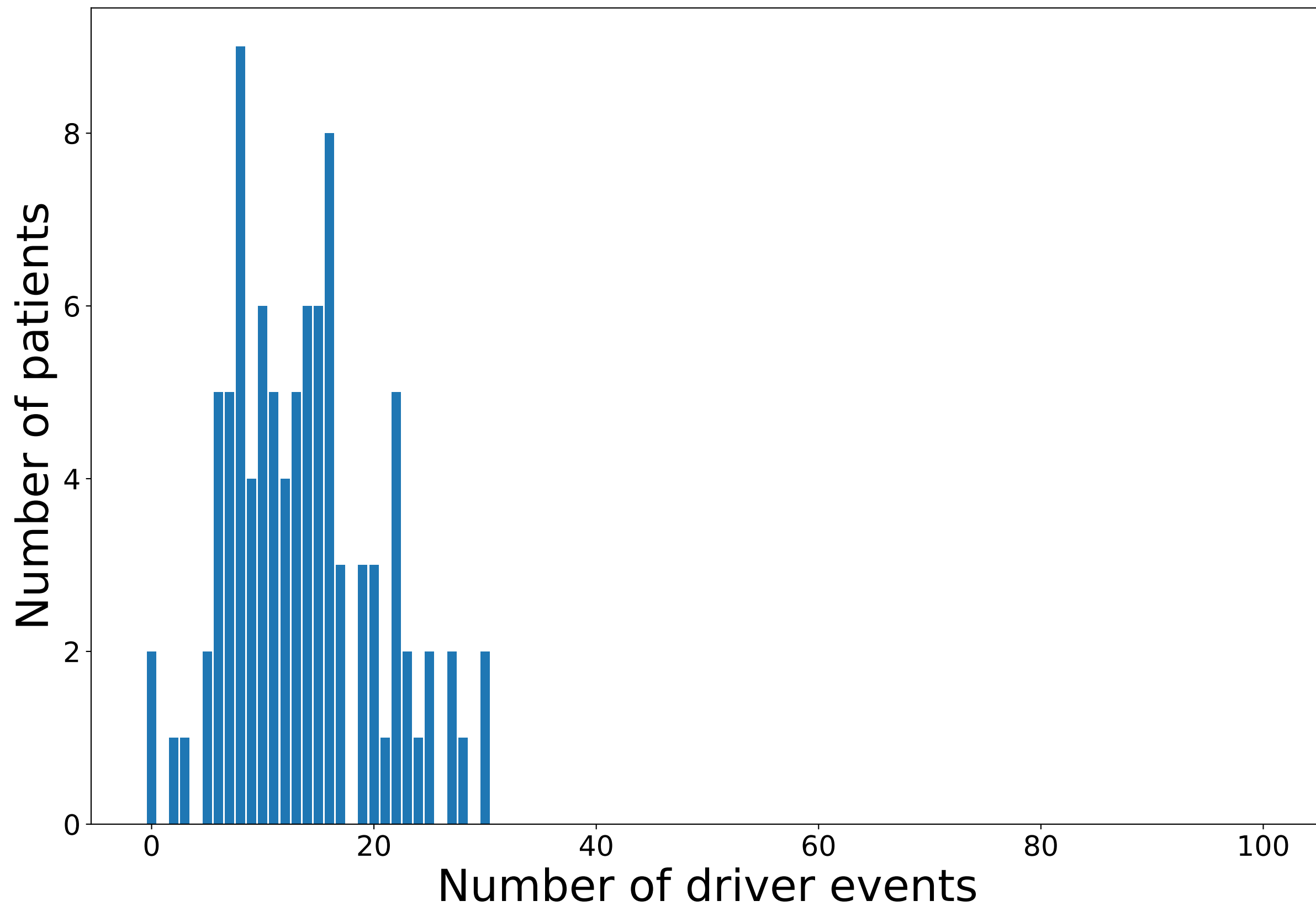

Supplement: Supplemental Information 2 [file peerj-10-13860-s002.zip › COHORTS/patient distributions/2021_8_16_14_9_LIHC_FEMALE.pdf]

# KIRC\_FEMALE

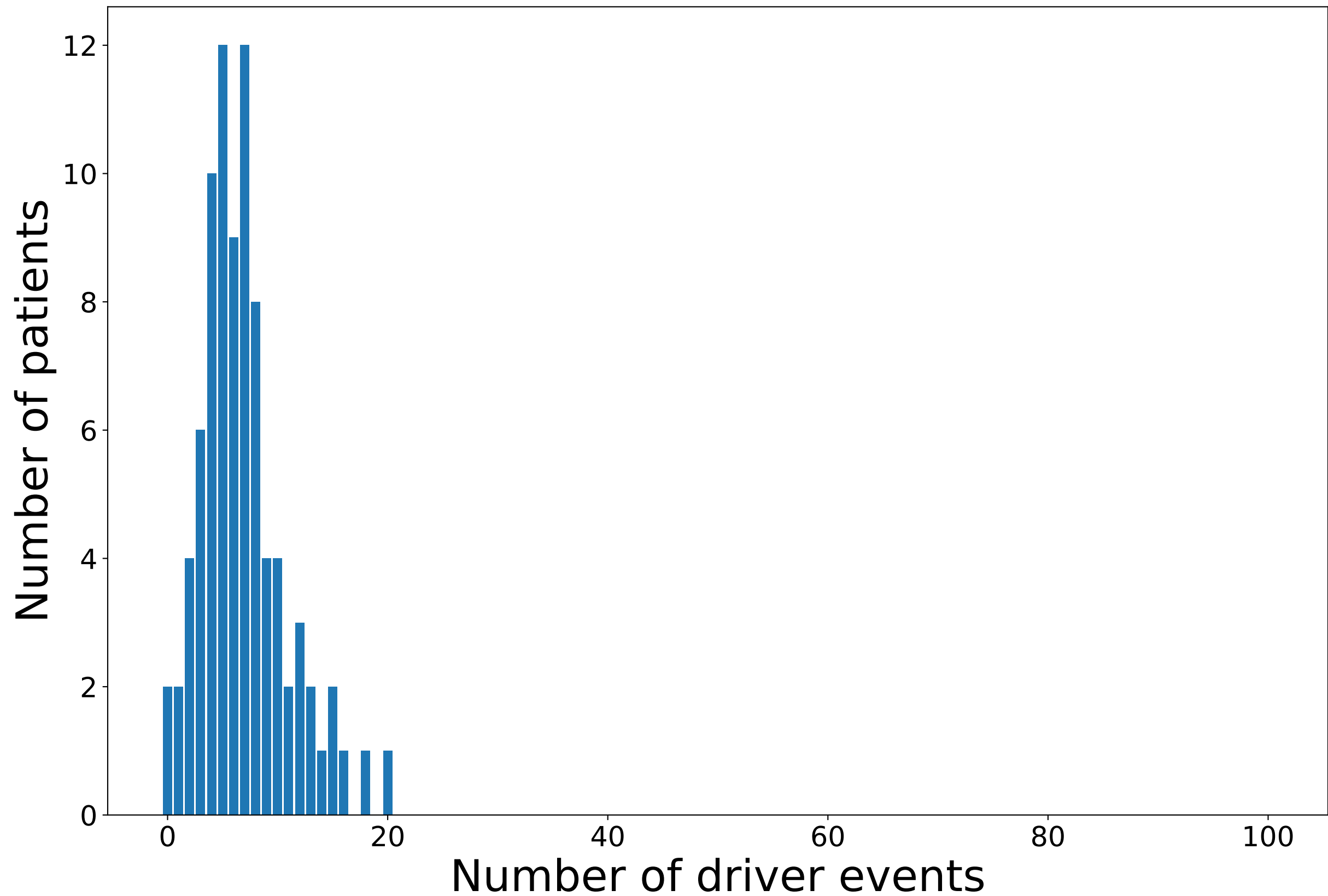

Supplement: Supplemental Information 2 [file peerj-10-13860-s002.zip › COHORTS/patient distributions/2021_8_16_14_9_KIRC_FEMALE.pdf]

# SARC\_MALE

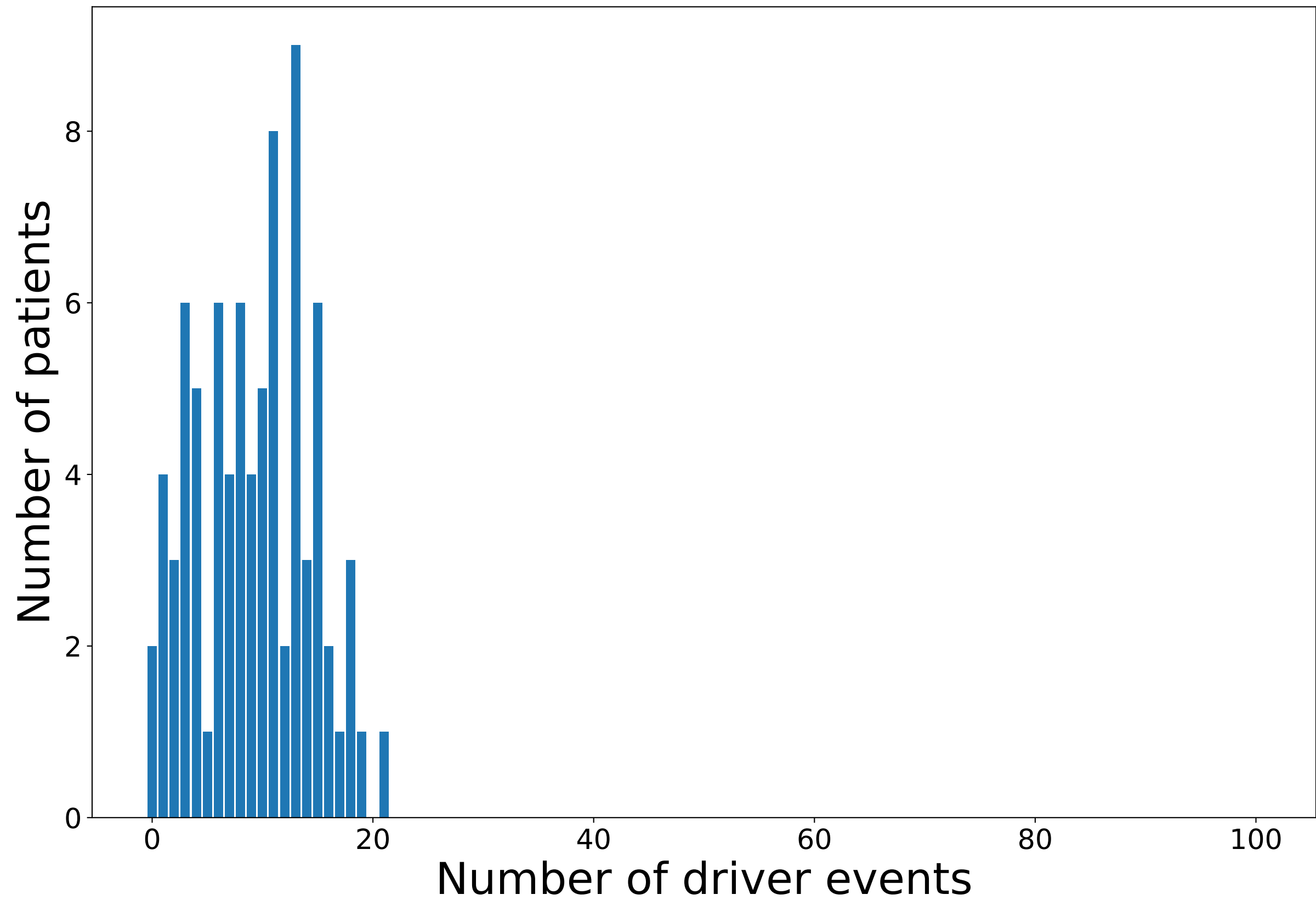

Supplement: Supplemental Information 2 [file peerj-10-13860-s002.zip › COHORTS/patient distributions/2021_8_16_14_9_SARC_MALE.pdf]

# PAAD\_MALE

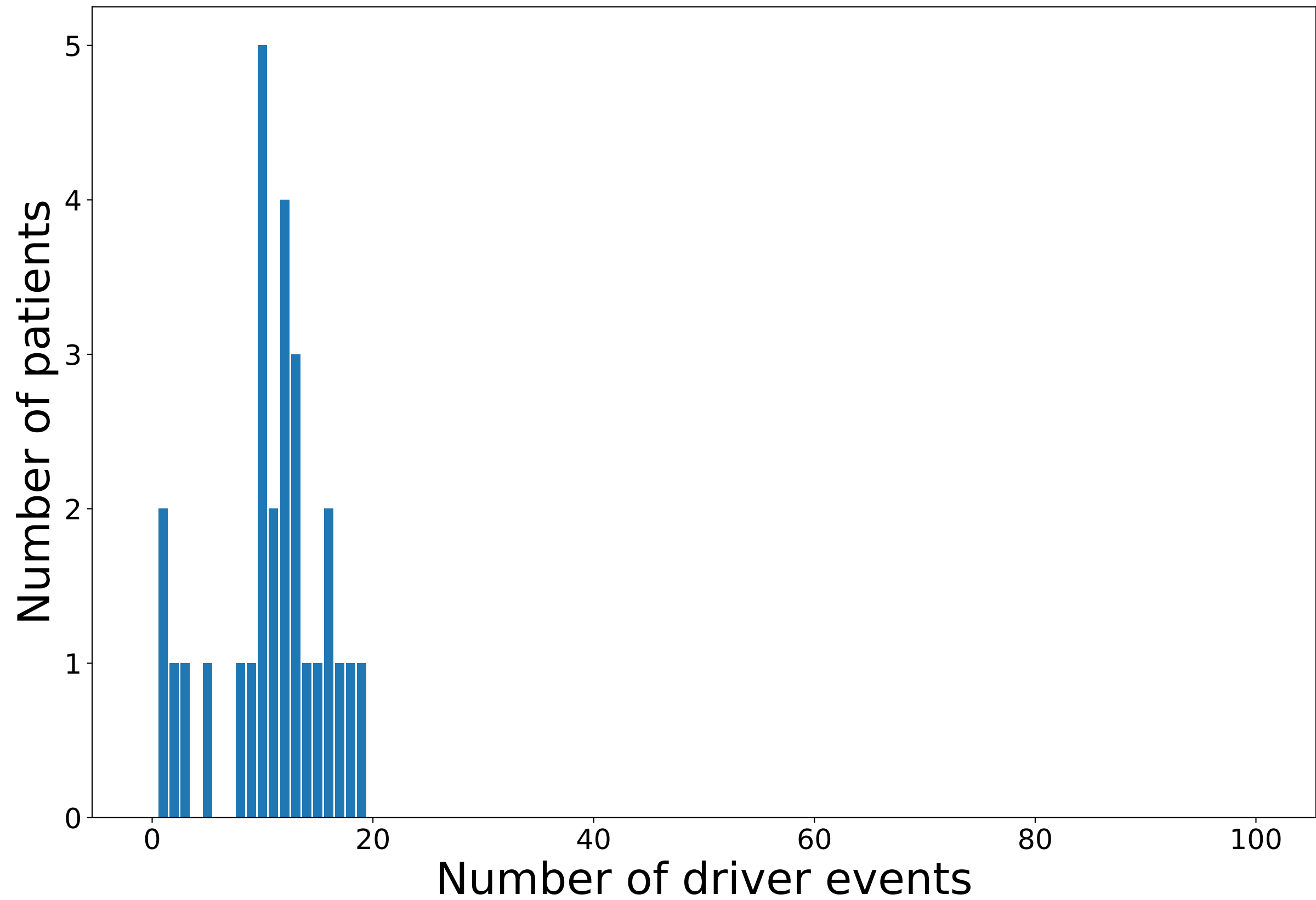

Supplement: Supplemental Information 2 [file peerj-10-13860-s002.zip › COHORTS/patient distributions/2021_8_16_14_9_PAAD_MALE.pdf]

# CHOL

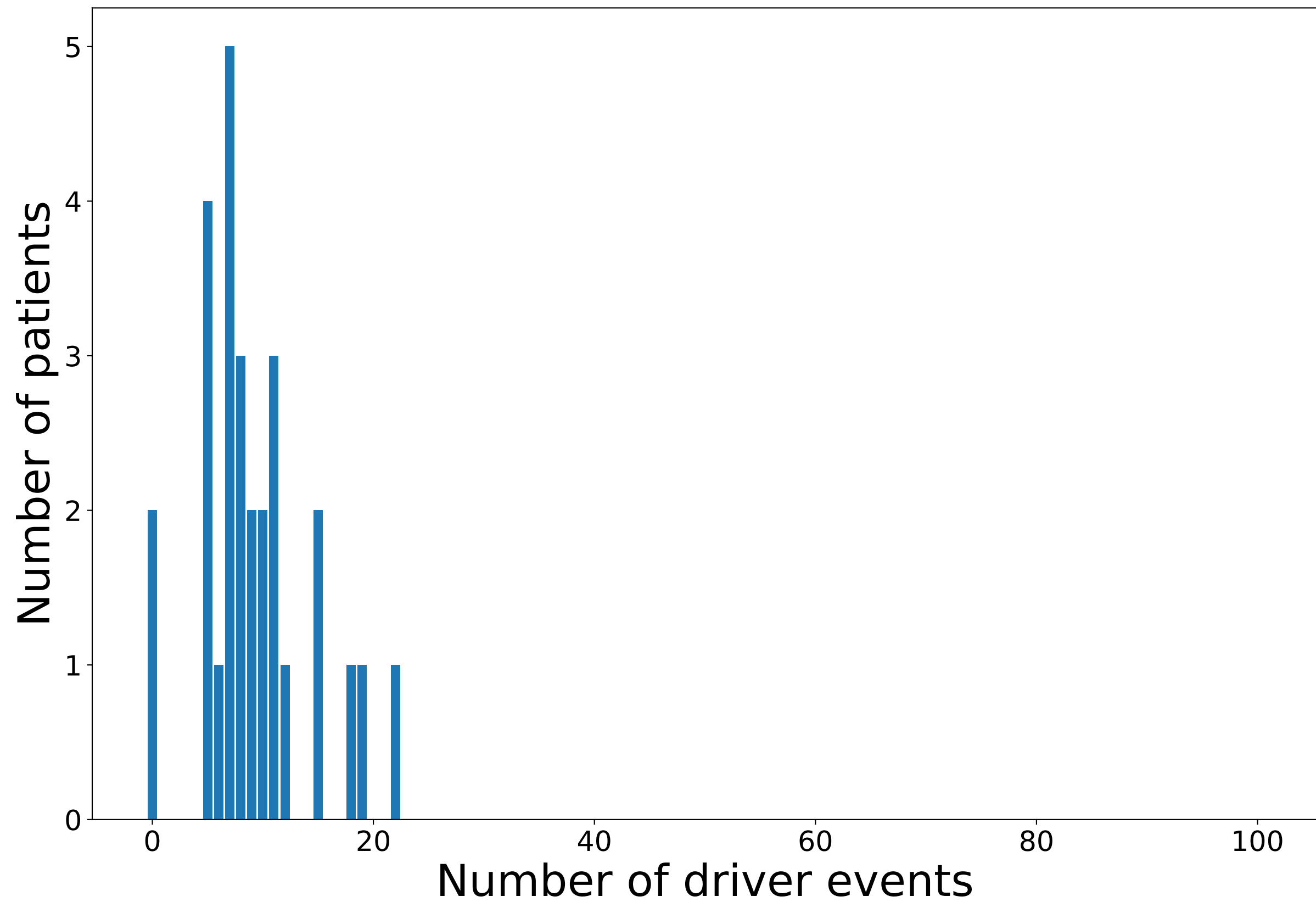

Supplement: Supplemental Information 2 [file peerj-10-13860-s002.zip › COHORTS/patient distributions/2021_8_16_14_9_CHOL.pdf]

# STAD

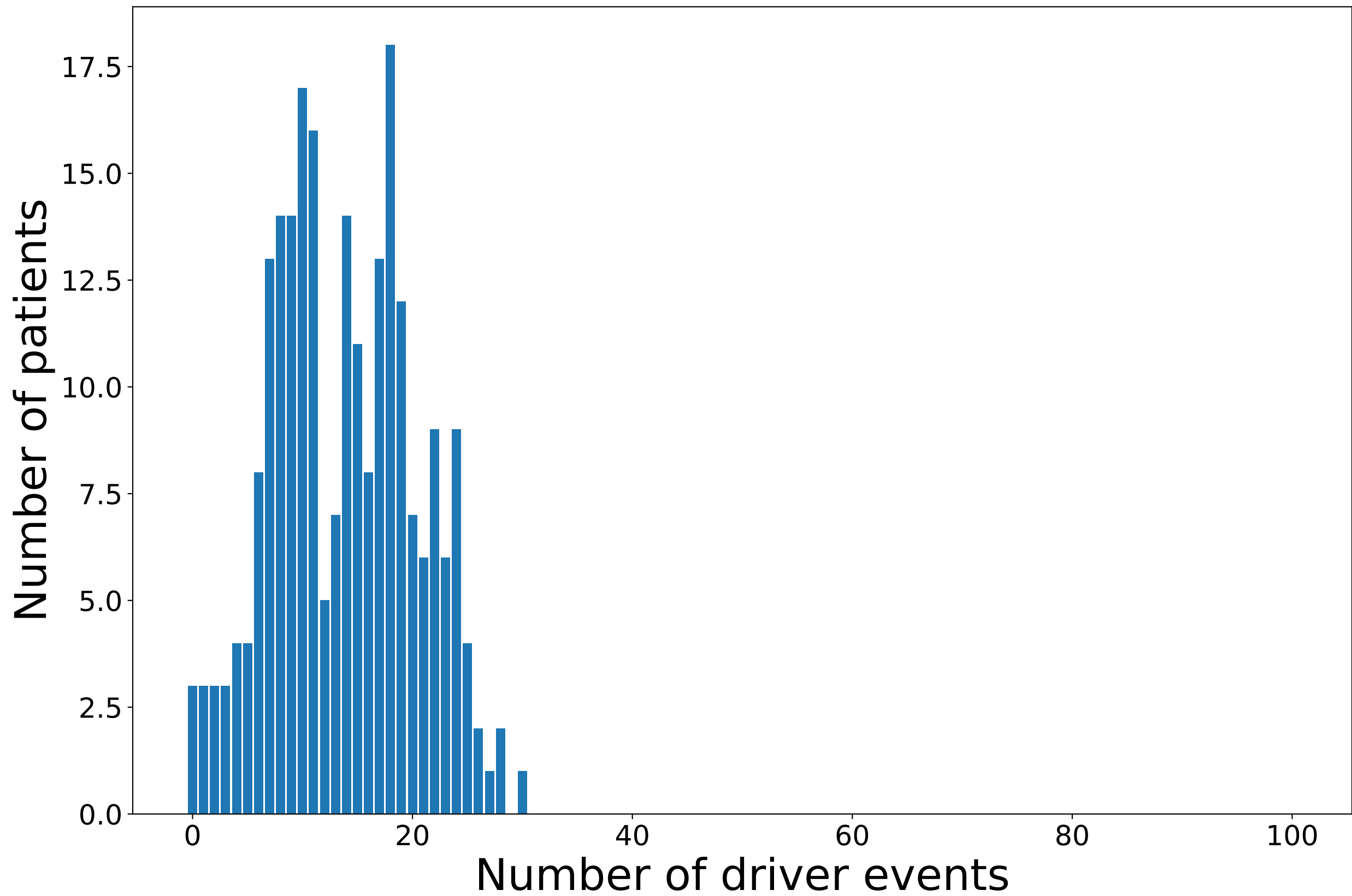

Supplement: Supplemental Information 2 [file peerj-10-13860-s002.zip › COHORTS/patient distributions/2021_8_16_14_9_STAD.pdf]

# LGG\_MALE

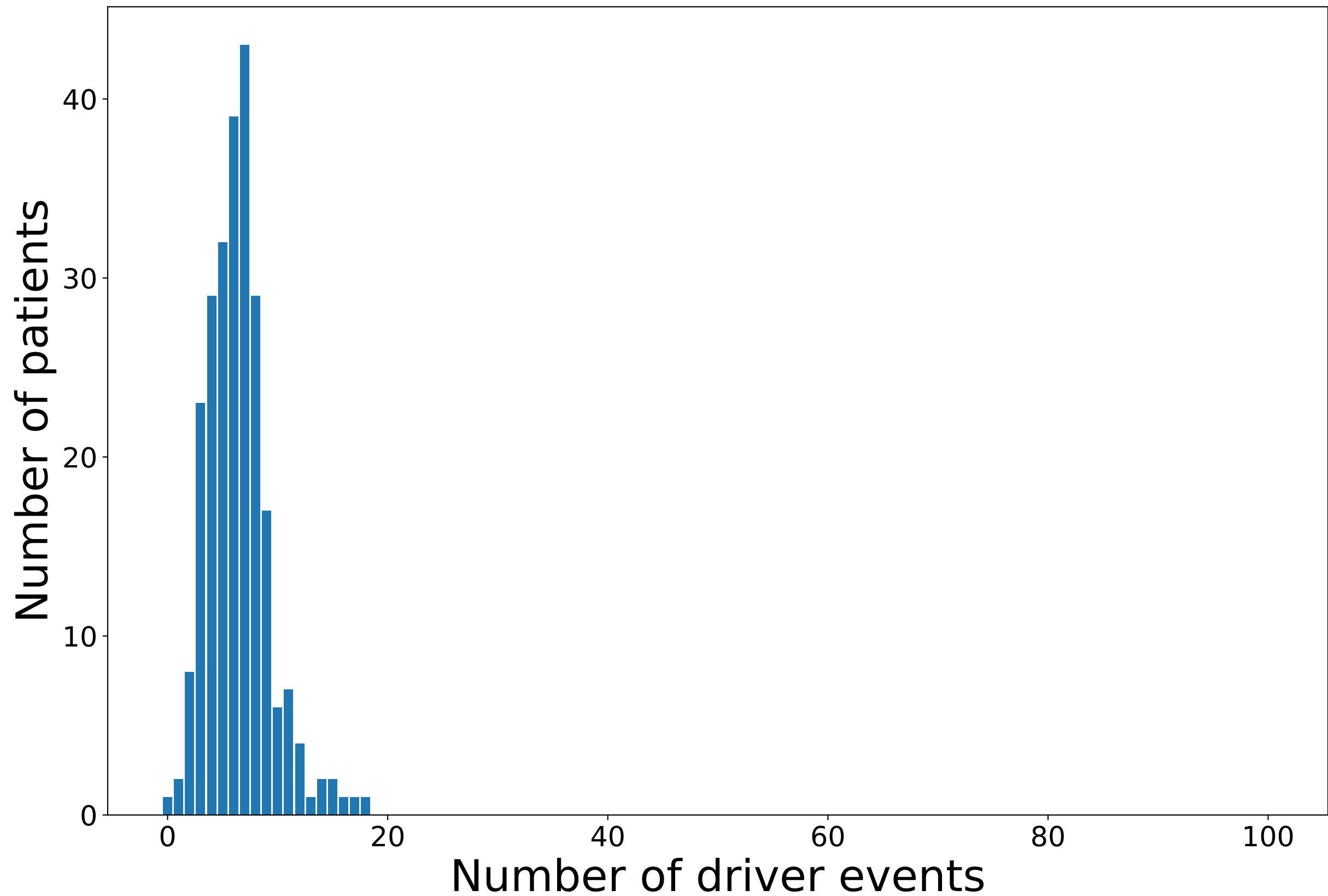

Supplement: Supplemental Information 2 [file peerj-10-13860-s002.zip › COHORTS/patient distributions/2021_8_16_14_9_LGG_MALE.pdf]

# BRCA\_FEMALE

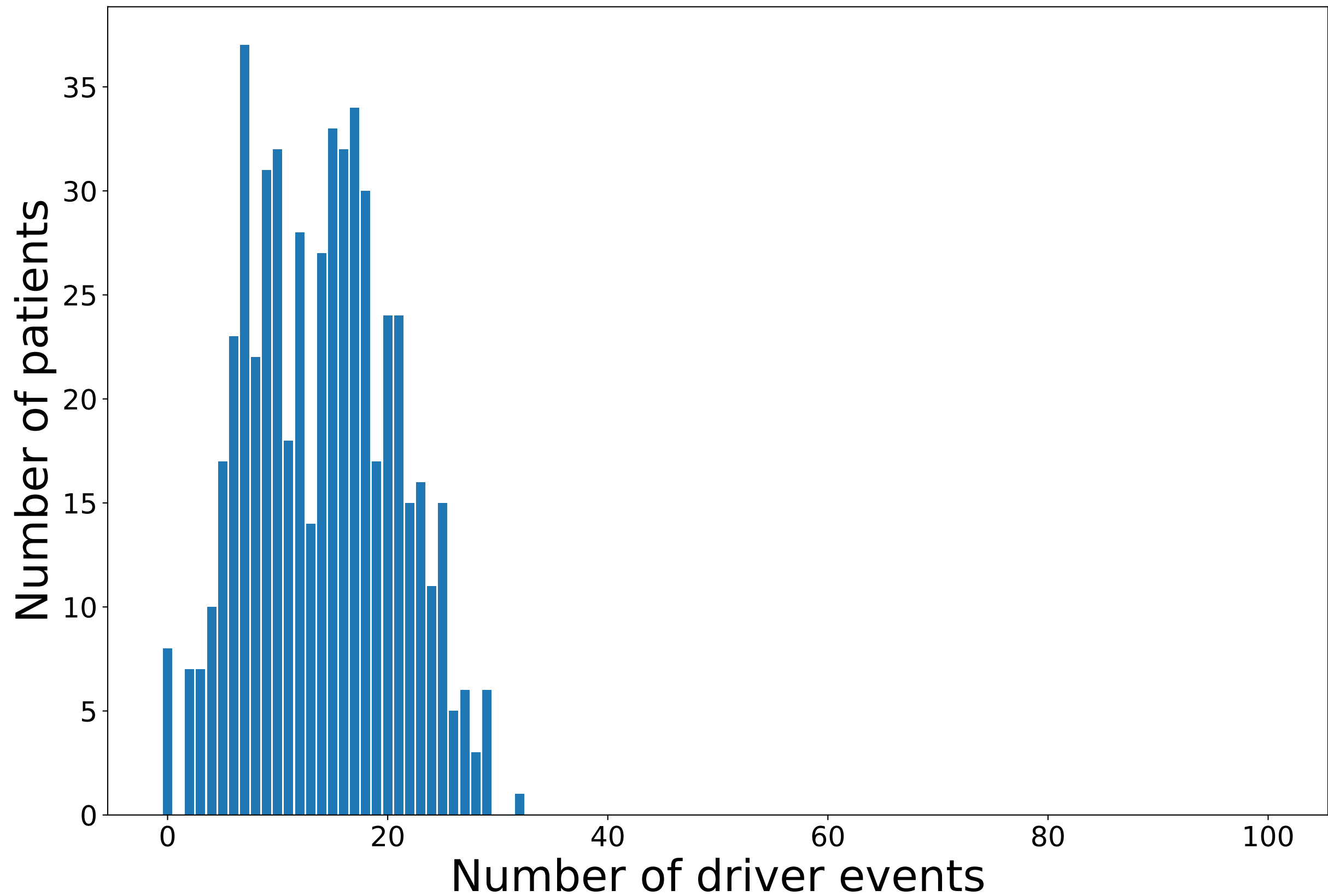

Supplement: Supplemental Information 2 [file peerj-10-13860-s002.zip › COHORTS/patient distributions/2021_8_16_14_9_BRCA_FEMALE.pdf]

# KICH

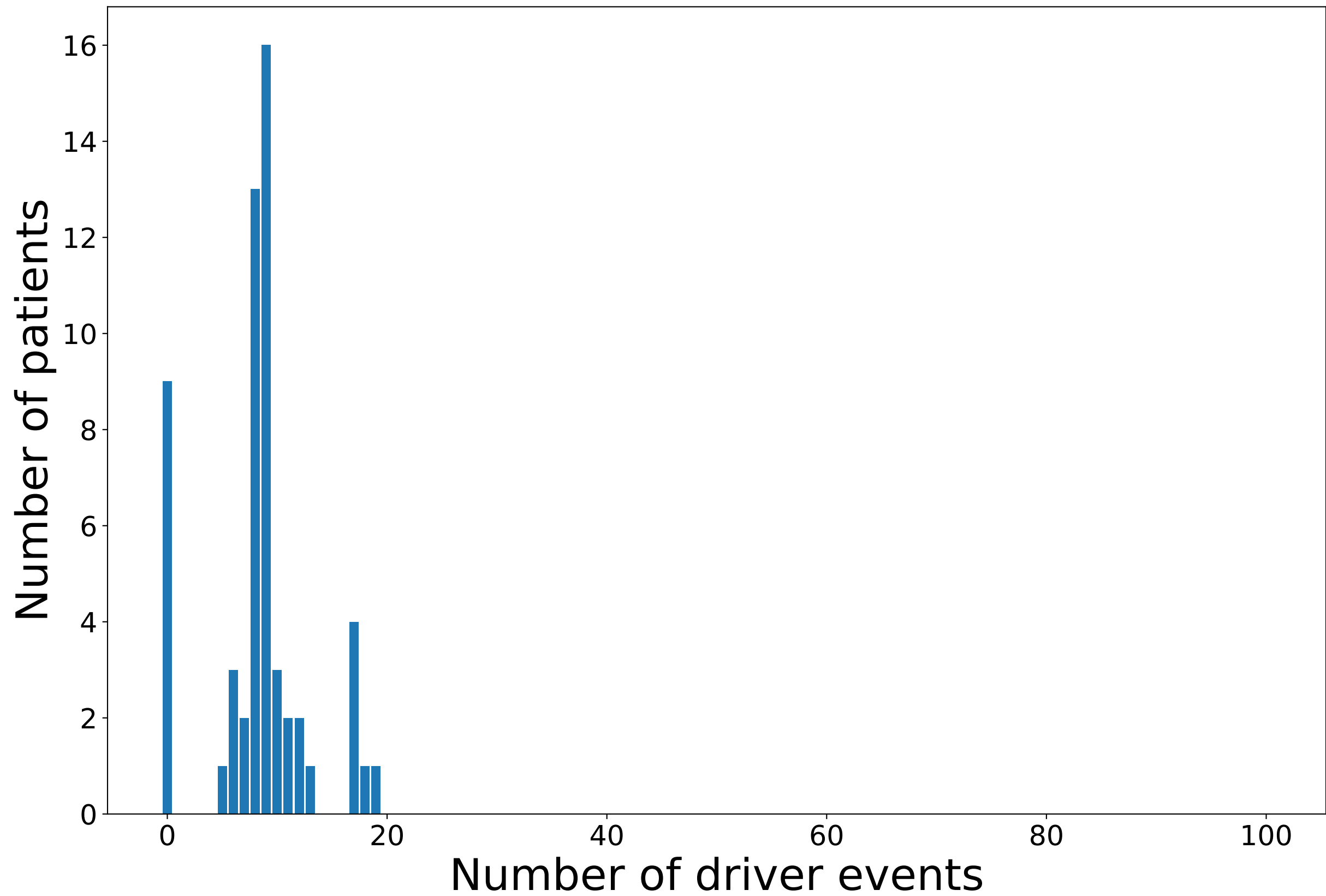

Supplement: Supplemental Information 2 [file peerj-10-13860-s002.zip › COHORTS/patient distributions/2021_8_16_14_9_KICH.pdf]

# UCEC

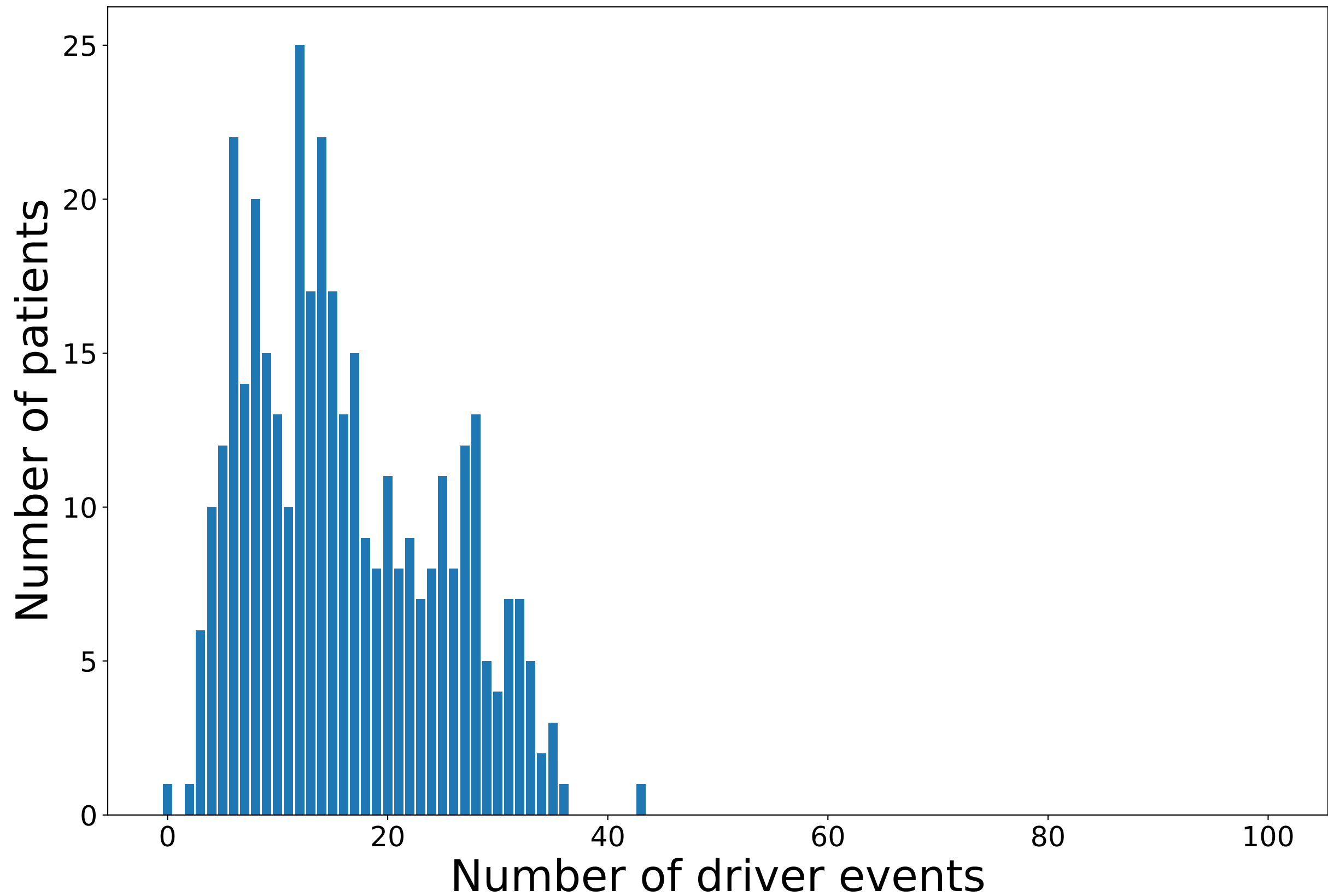

Supplement: Supplemental Information 2 [file peerj-10-13860-s002.zip › COHORTS/patient distributions/2021_8_16_14_9_UCEC.pdf]

# LUAD\_MALE

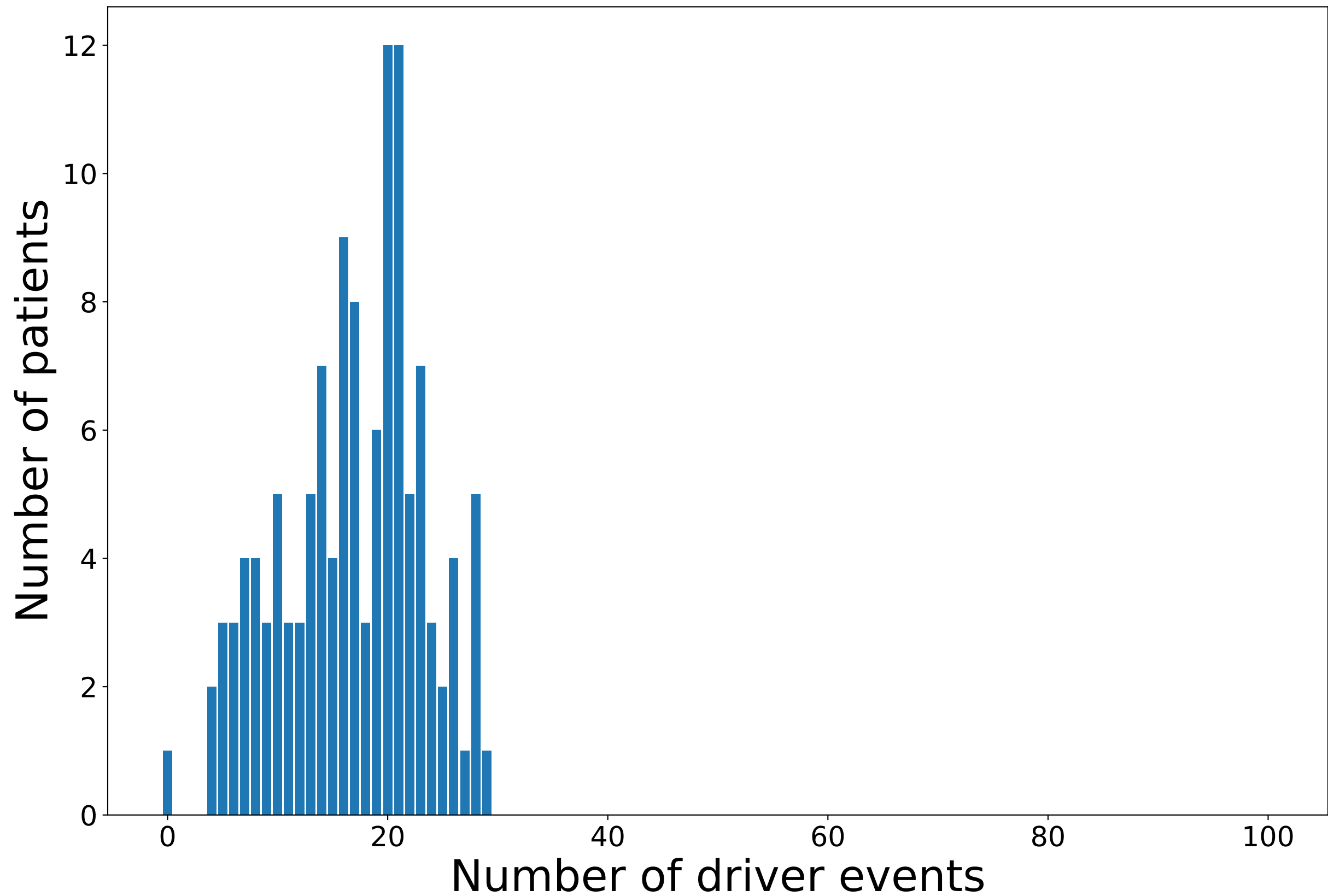

Supplement: Supplemental Information 2 [file peerj-10-13860-s002.zip › COHORTS/patient distributions/2021_8_16_14_9_LUAD_MALE.pdf]

# DLBC\_FEMALE

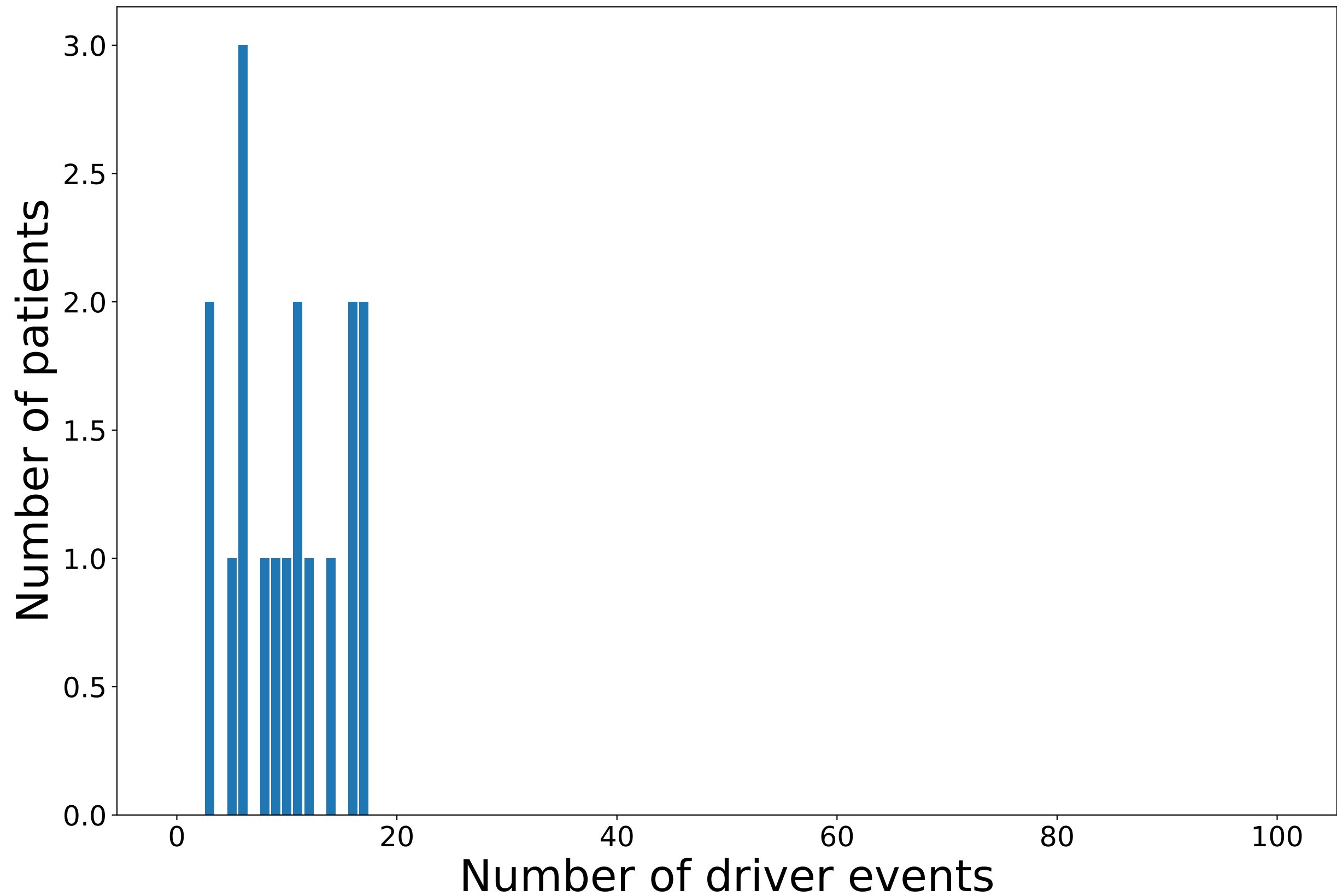

Supplement: Supplemental Information 2 [file peerj-10-13860-s002.zip › COHORTS/patient distributions/2021_8_16_14_9_DLBC_FEMALE.pdf]

# LUSC\_MALE

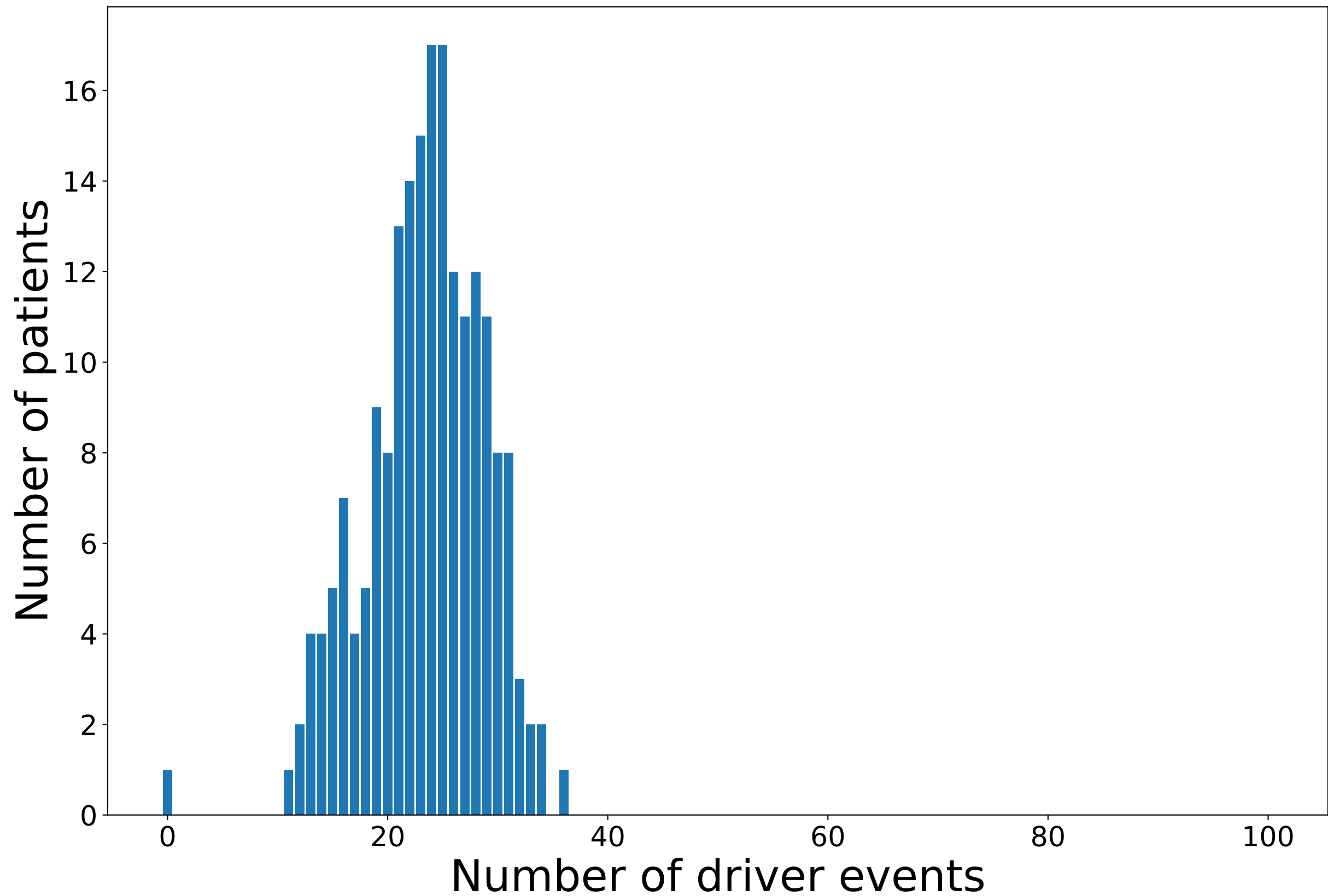

Supplement: Supplemental Information 2 [file peerj-10-13860-s002.zip › COHORTS/patient distributions/2021_8_16_14_9_LUSC_MALE.pdf]

# UVM\_FEMALE

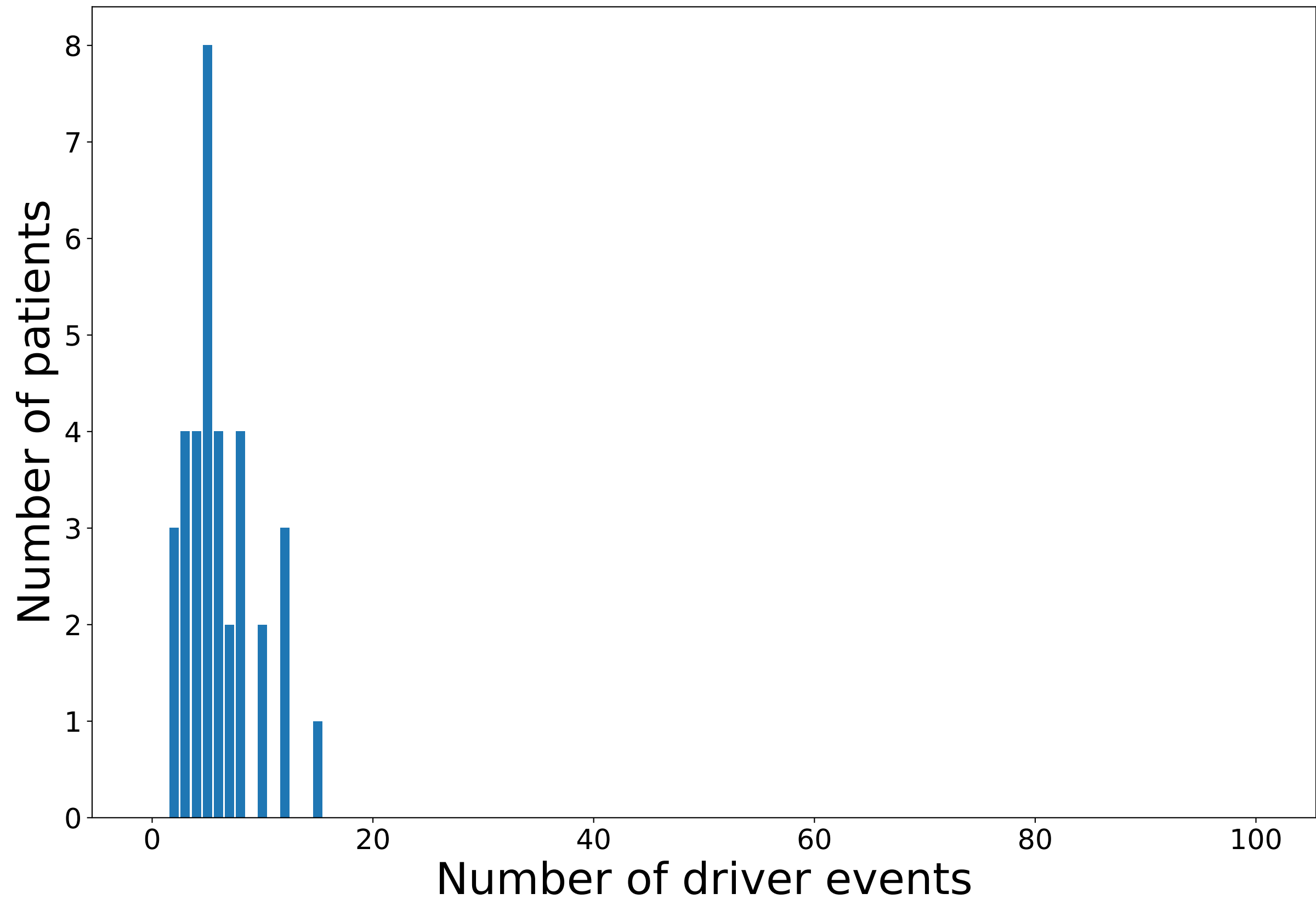

Supplement: Supplemental Information 2 [file peerj-10-13860-s002.zip › COHORTS/patient distributions/2021_8_16_14_9_UVM_FEMALE.pdf]

# PRAD\_MALE

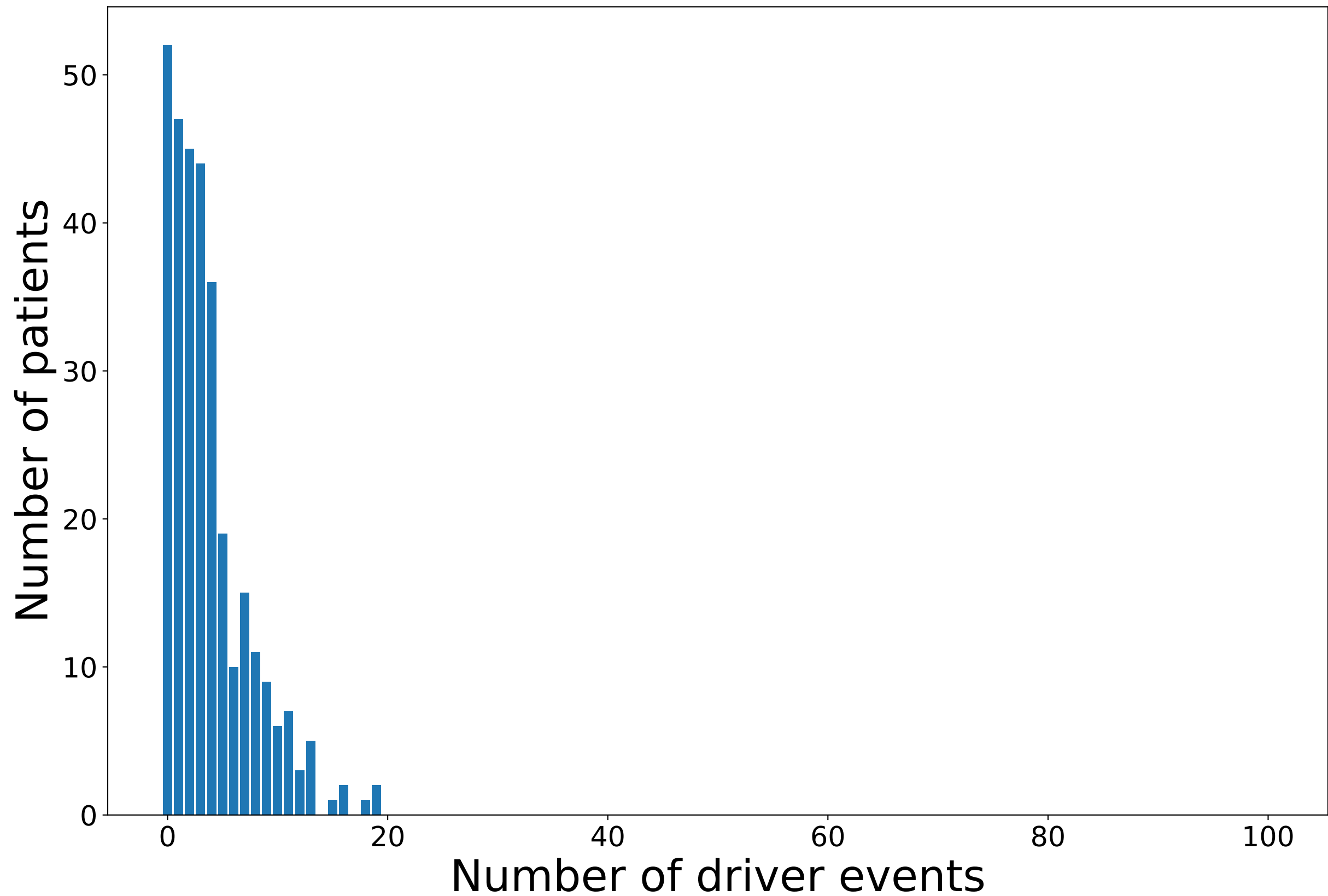

Supplement: Supplemental Information 2 [file peerj-10-13860-s002.zip › COHORTS/patient distributions/2021_8_16_14_9_PRAD_MALE.pdf]

# KICH\_FEMALE

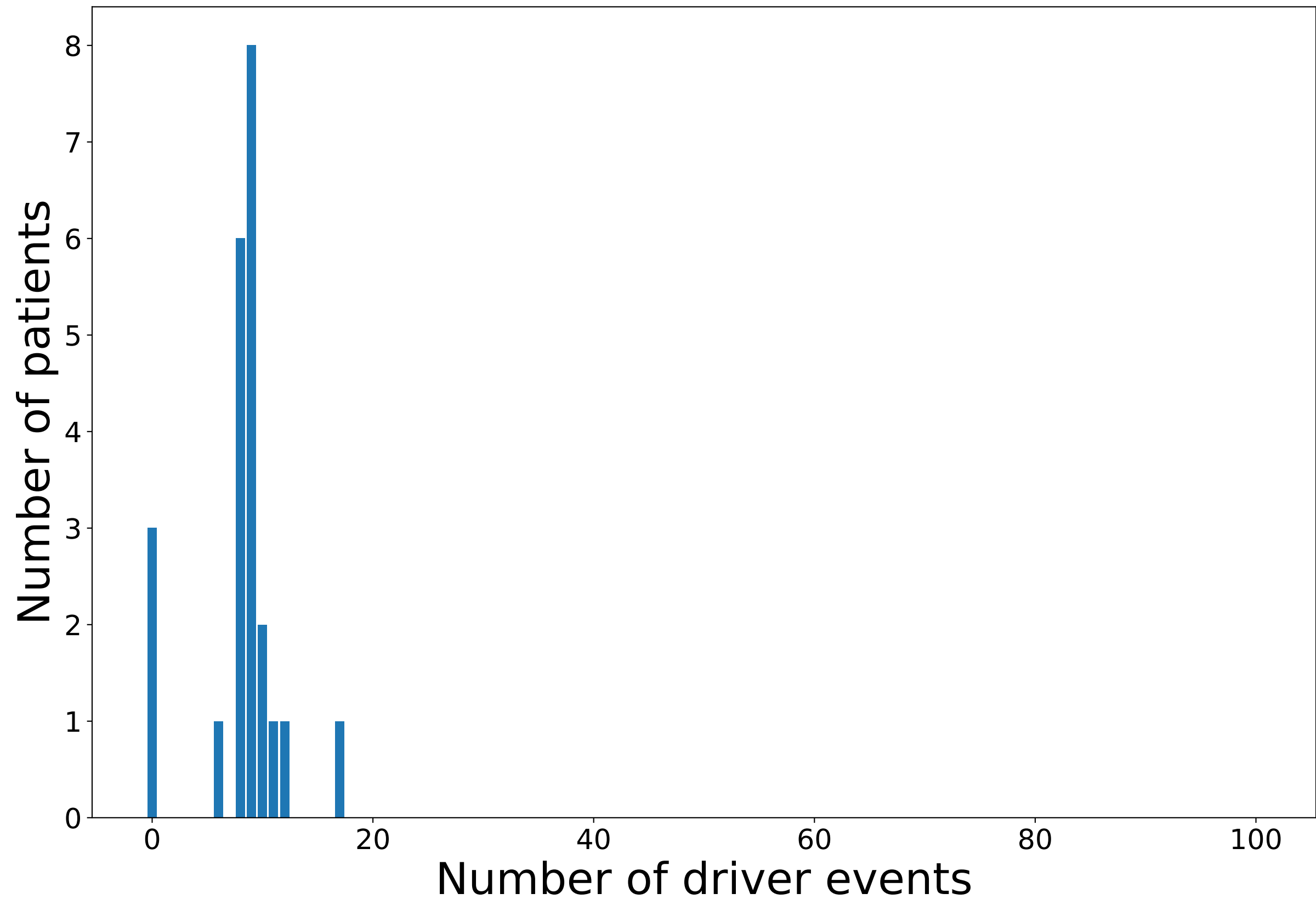

Supplement: Supplemental Information 2 [file peerj-10-13860-s002.zip › COHORTS/patient distributions/2021_8_16_14_9_KICH_FEMALE.pdf]

# ESCA\_FEMALE

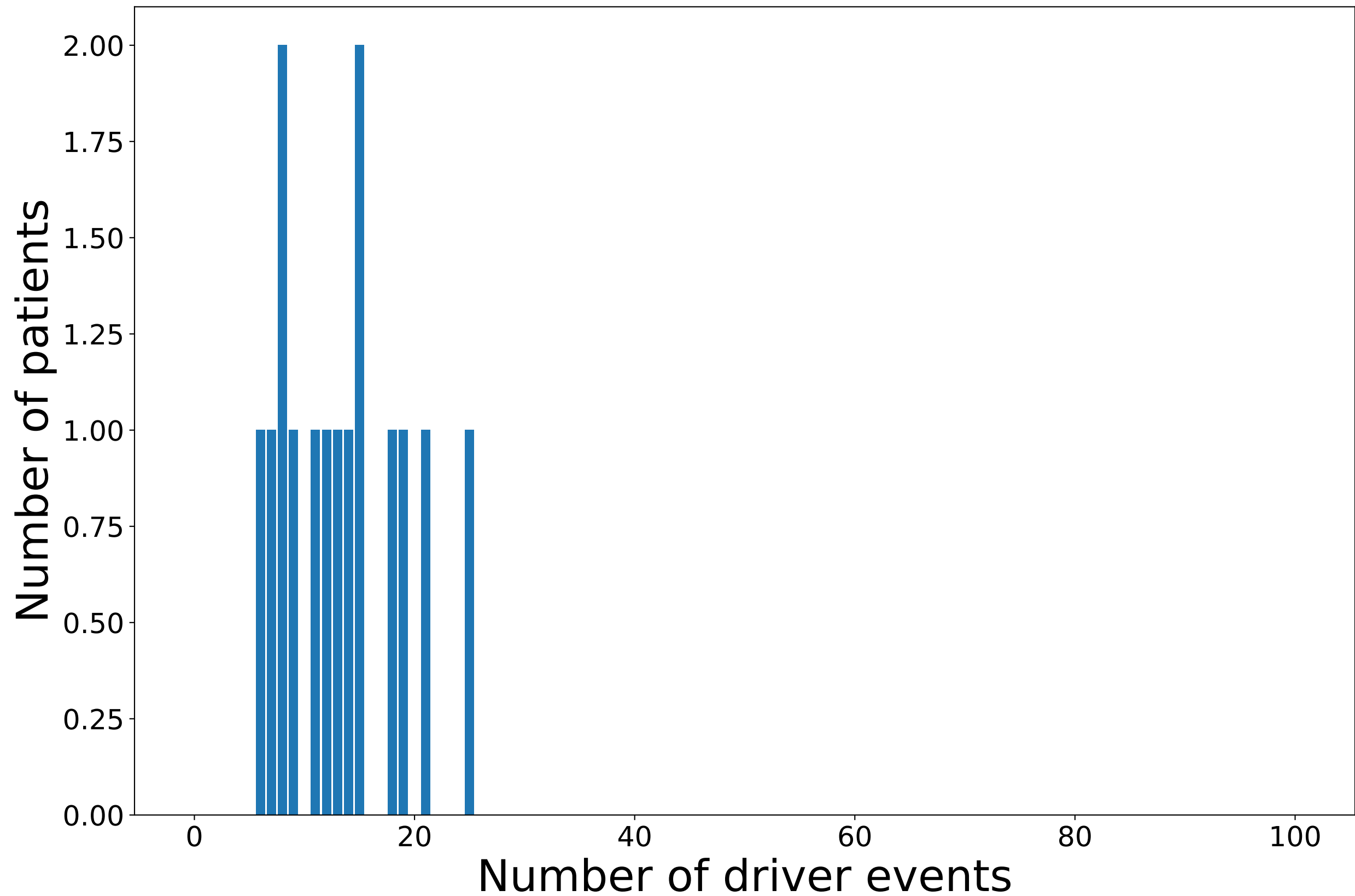

Supplement: Supplemental Information 2 [file peerj-10-13860-s002.zip › COHORTS/patient distributions/2021_8_16_14_9_ESCA_FEMALE.pdf]

# KIRP

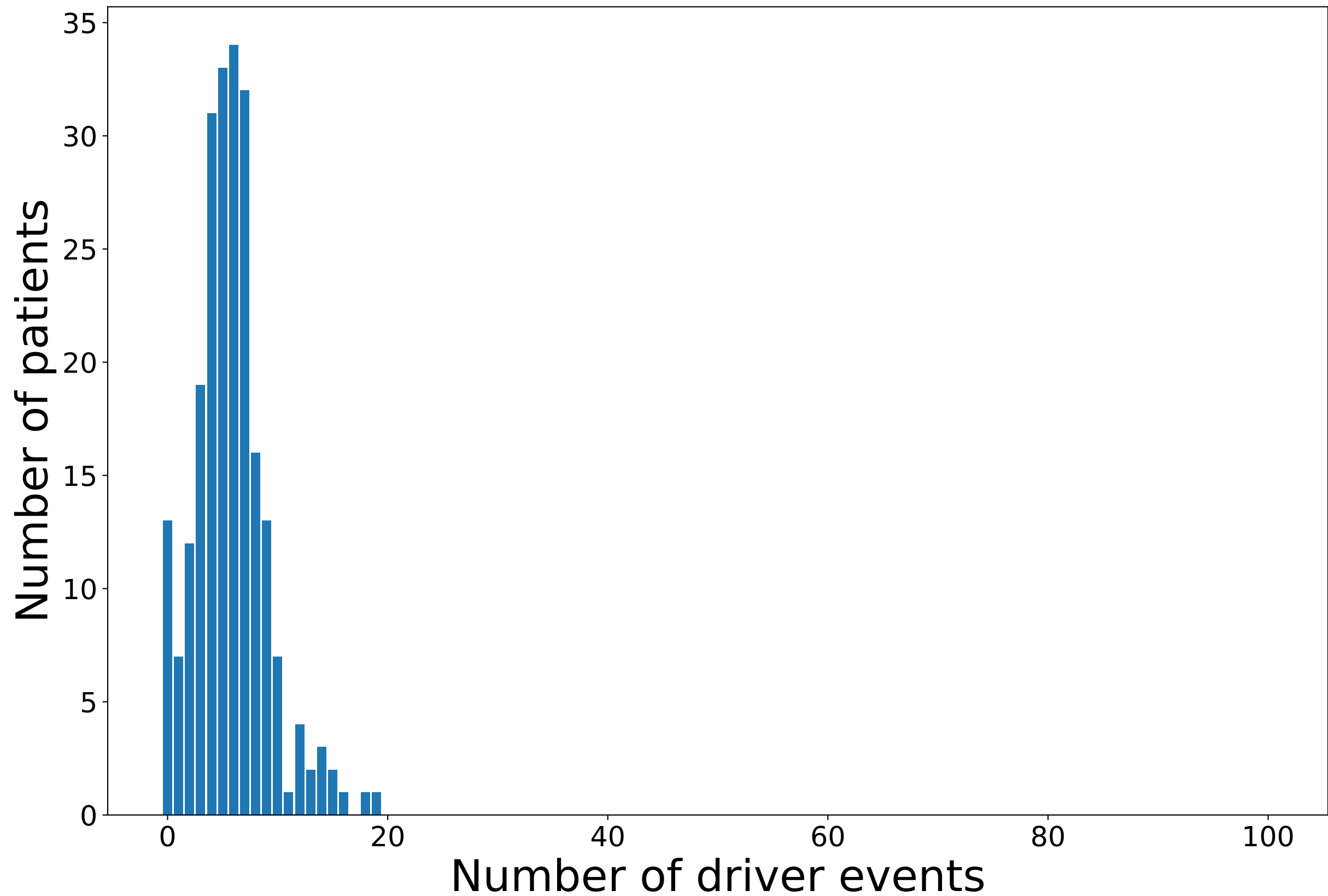

Supplement: Supplemental Information 2 [file peerj-10-13860-s002.zip › COHORTS/patient distributions/2021_8_16_14_9_KIRP.pdf]

# COAD

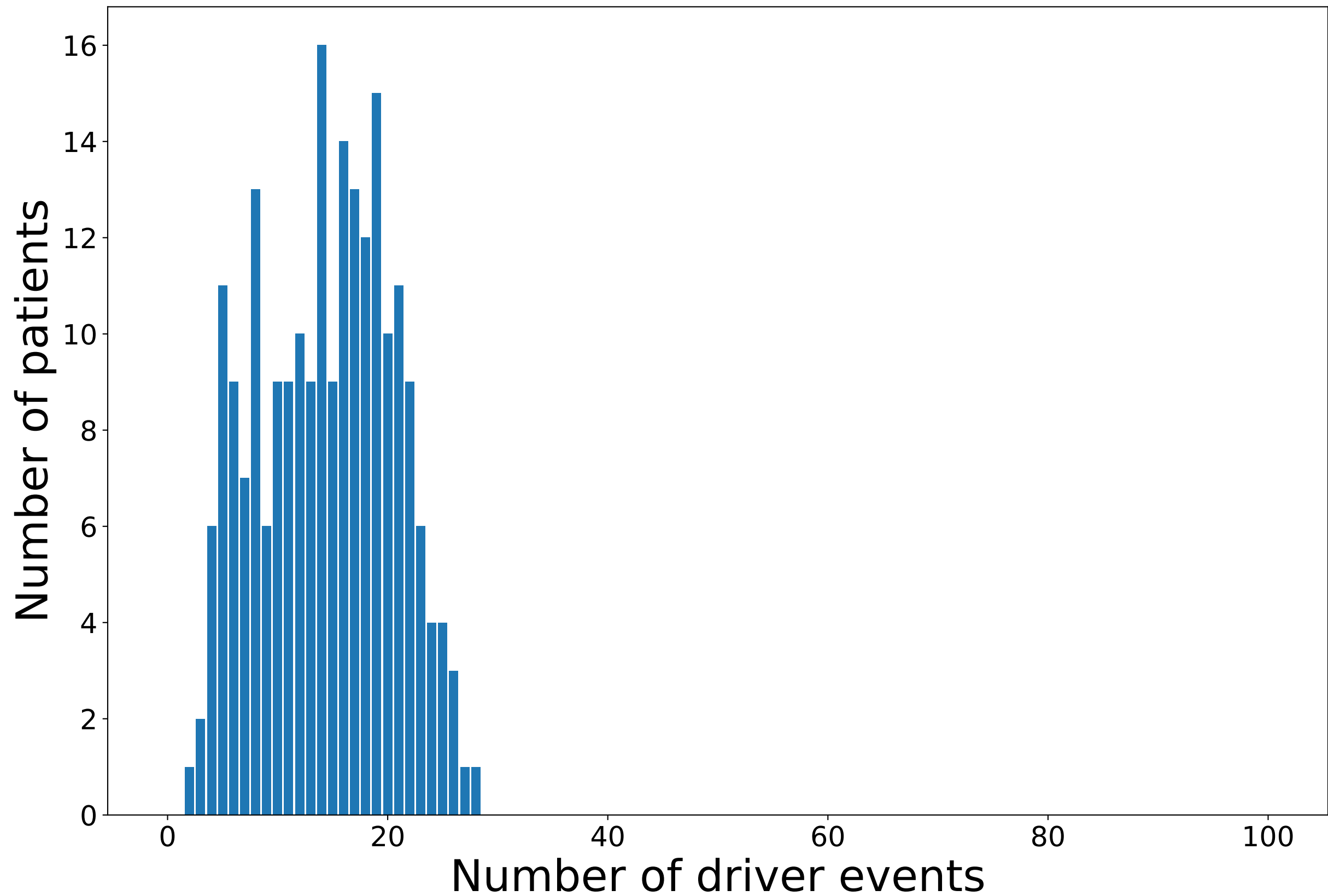

Supplement: Supplemental Information 2 [file peerj-10-13860-s002.zip › COHORTS/patient distributions/2021_8_16_14_9_COAD.pdf]

# DLBC\_MALE

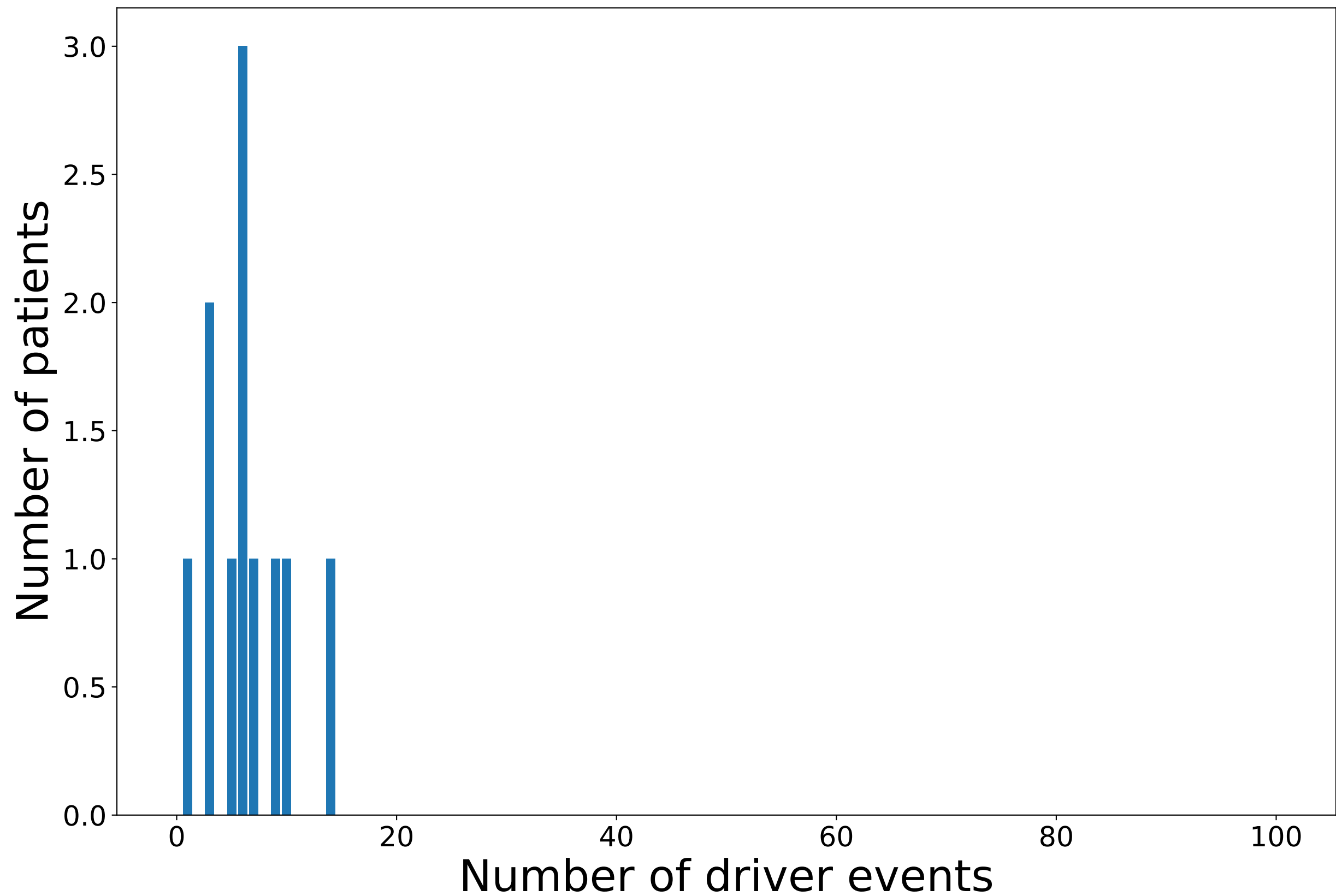

Supplement: Supplemental Information 2 [file peerj-10-13860-s002.zip › COHORTS/patient distributions/2021_8_16_14_9_DLBC_MALE.pdf]

# LUSC\_FEMALE

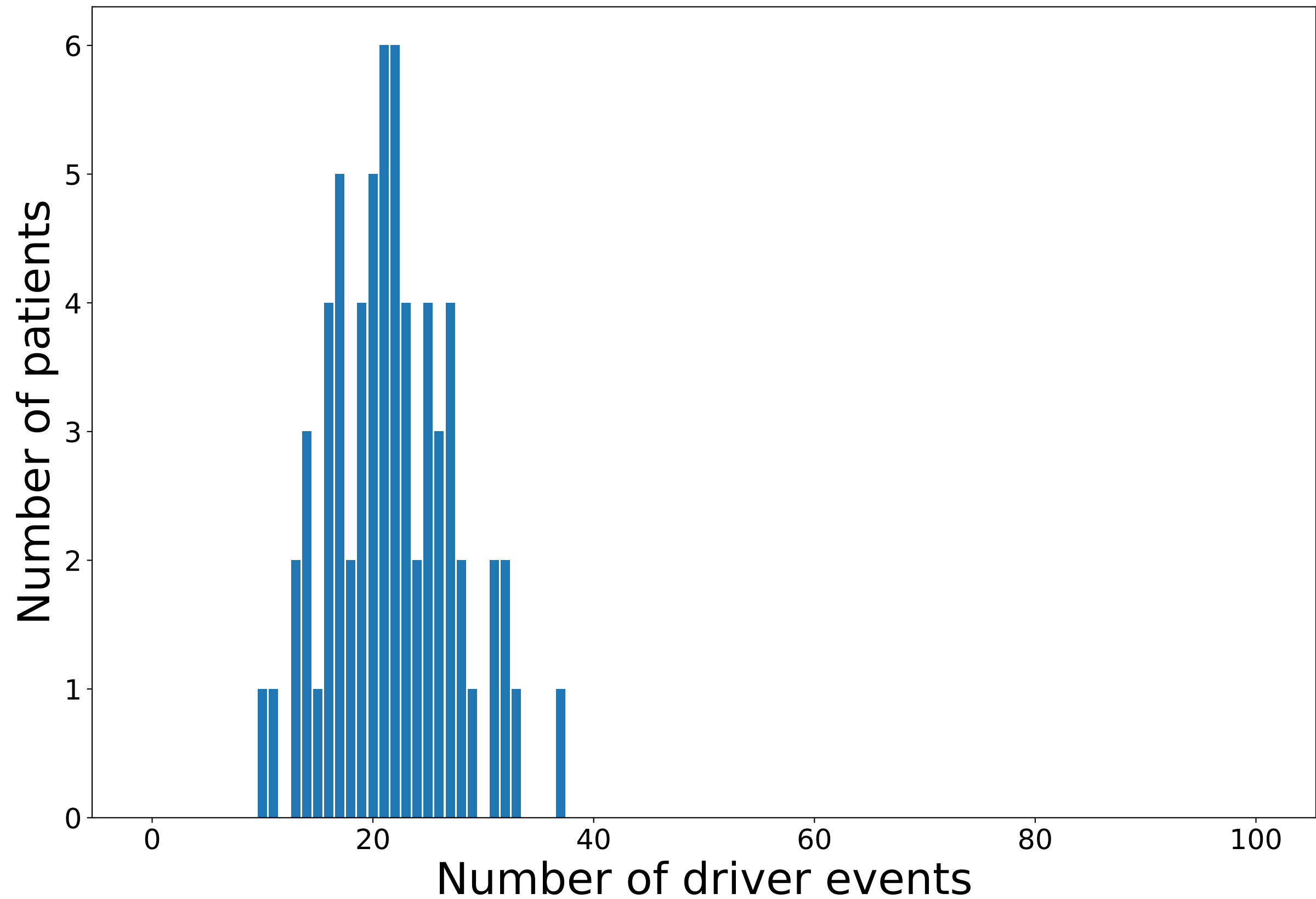

Supplement: Supplemental Information 2 [file peerj-10-13860-s002.zip › COHORTS/patient distributions/2021_8_16_14_9_LUSC_FEMALE.pdf]

# UCEC\_FEMALE

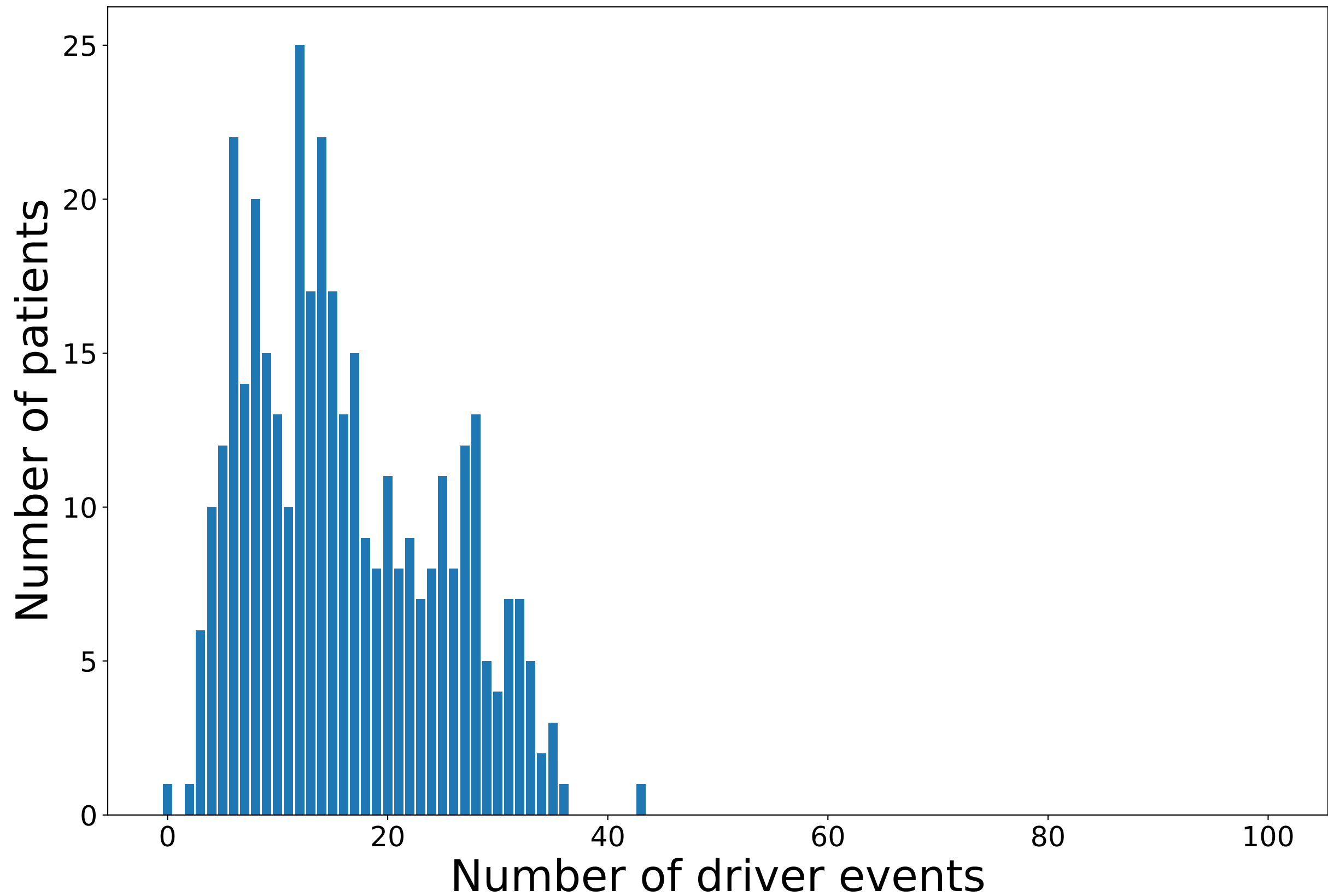

Supplement: Supplemental Information 2 [file peerj-10-13860-s002.zip › COHORTS/patient distributions/2021_8_16_14_9_UCEC_FEMALE.pdf]

# ACC\_MALE

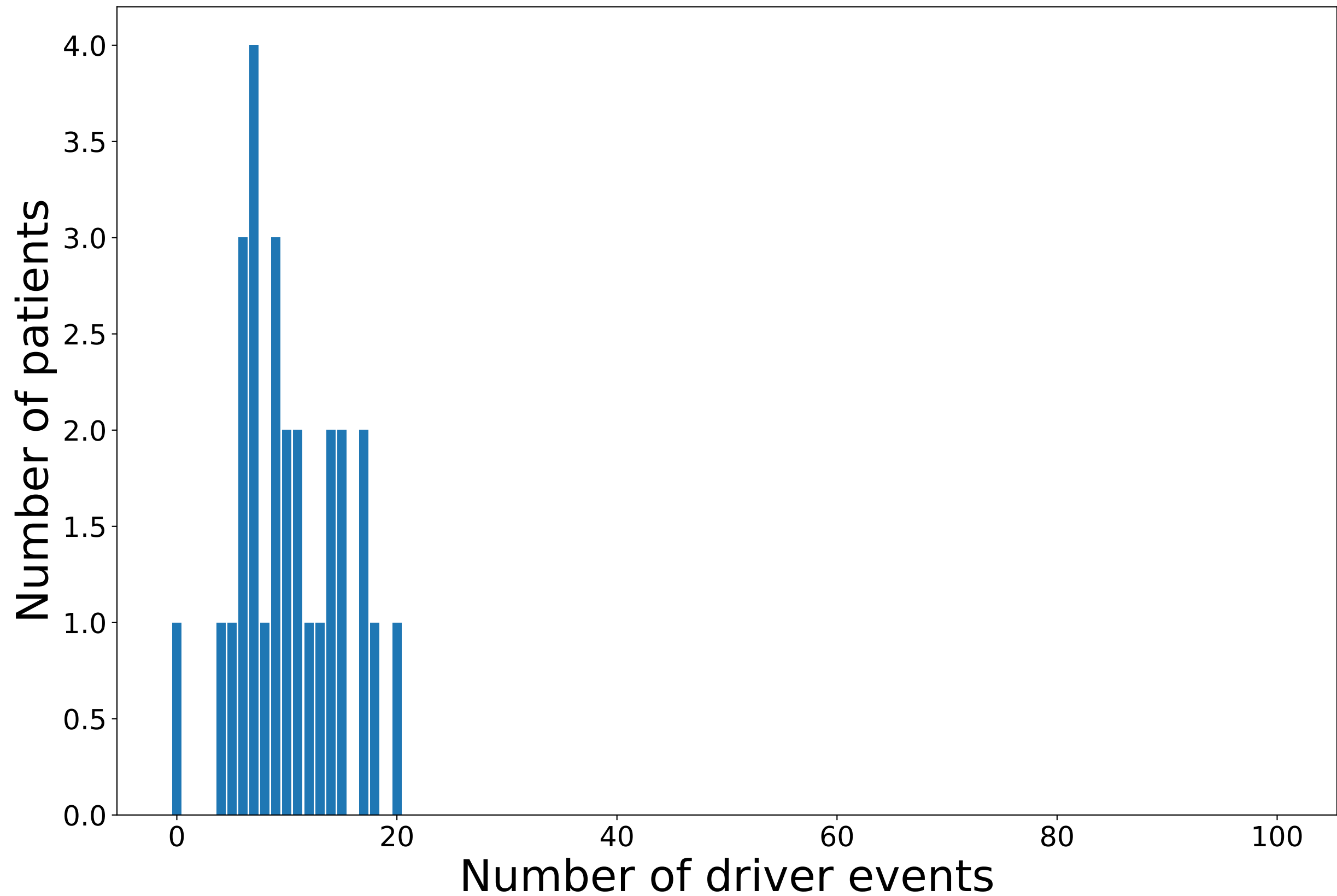

Supplement: Supplemental Information 2 [file peerj-10-13860-s002.zip › COHORTS/patient distributions/2021_8_16_14_9_ACC_MALE.pdf]

# LIHC

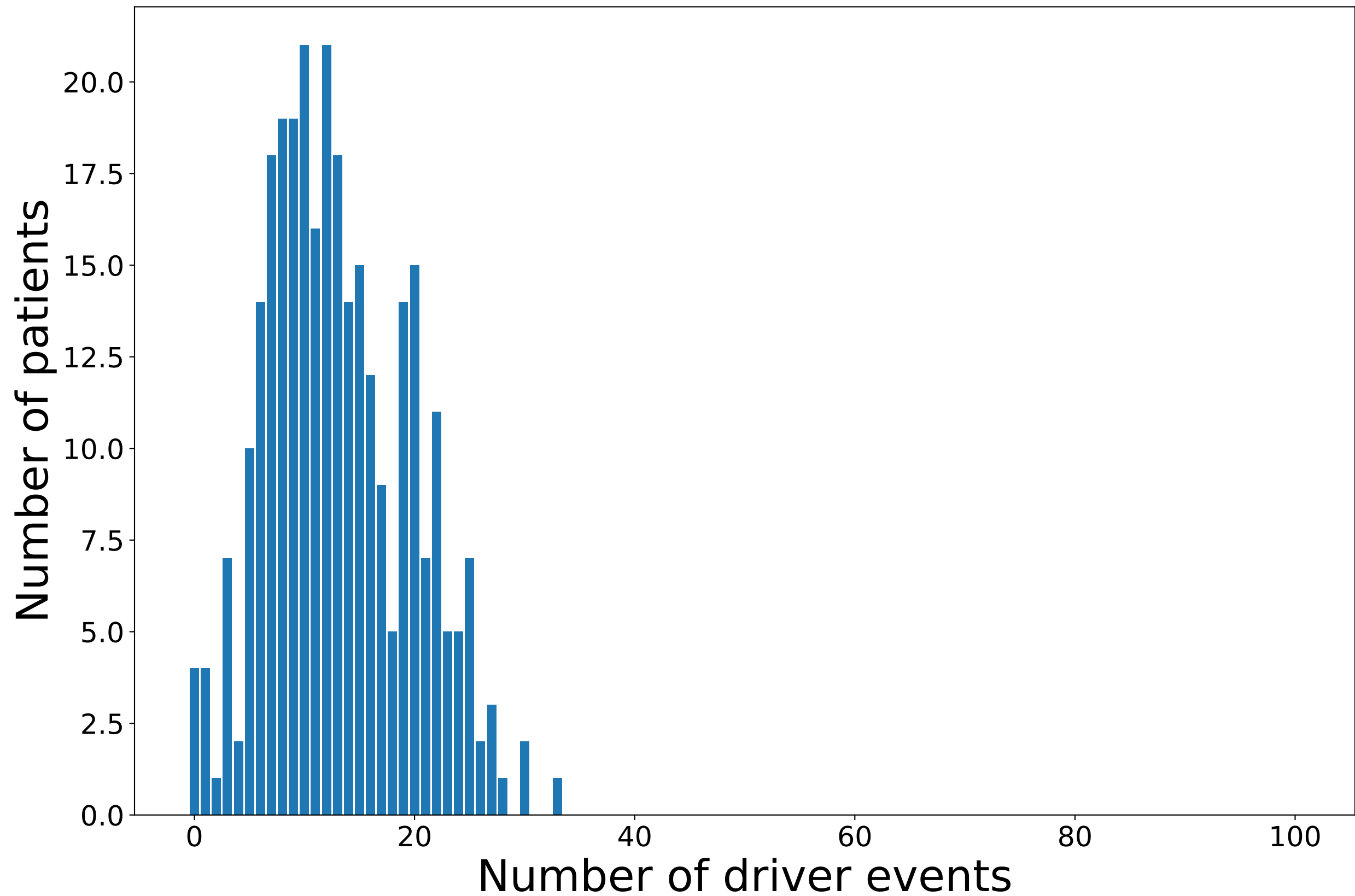

Supplement: Supplemental Information 2 [file peerj-10-13860-s002.zip › COHORTS/patient distributions/2021_8_16_14_9_LIHC.pdf]

# CESC\_FEMALE

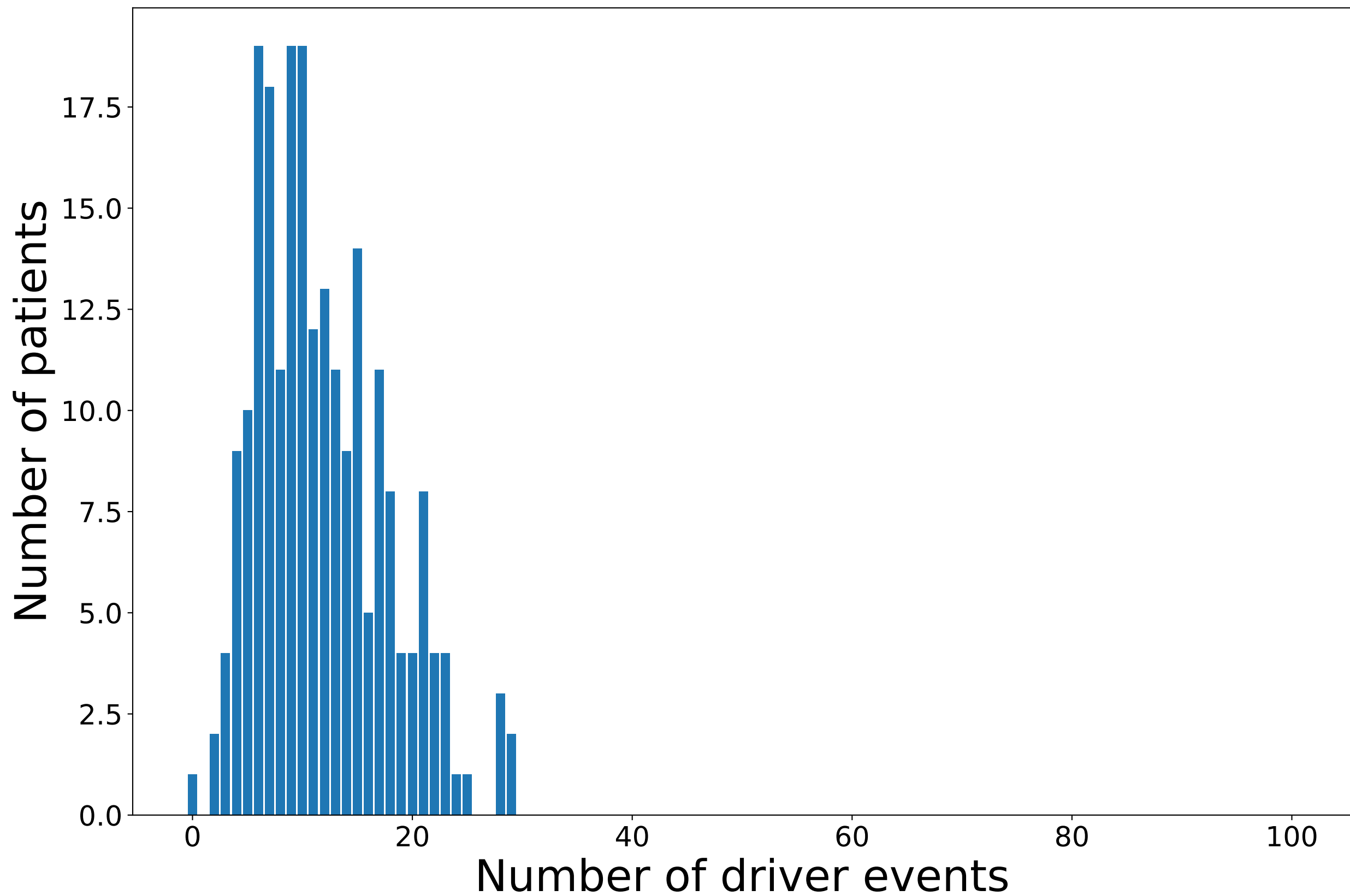

Supplement: Supplemental Information 2 [file peerj-10-13860-s002.zip › COHORTS/patient distributions/2021_8_16_14_9_CESC_FEMALE.pdf]

# UCS\_FEMALE

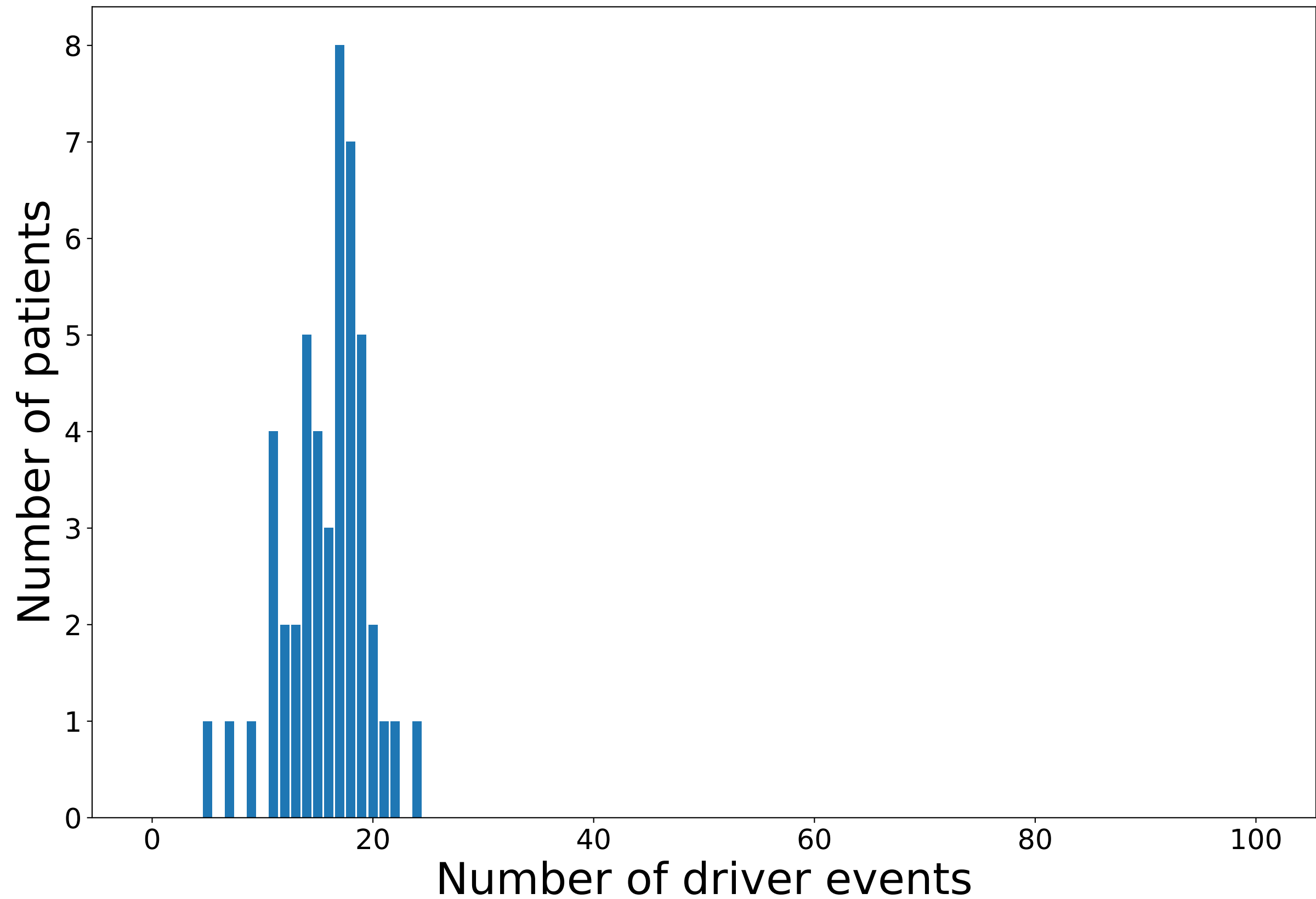

Supplement: Supplemental Information 2 [file peerj-10-13860-s002.zip › COHORTS/patient distributions/2021_8_16_14_9_UCS_FEMALE.pdf]

ACC

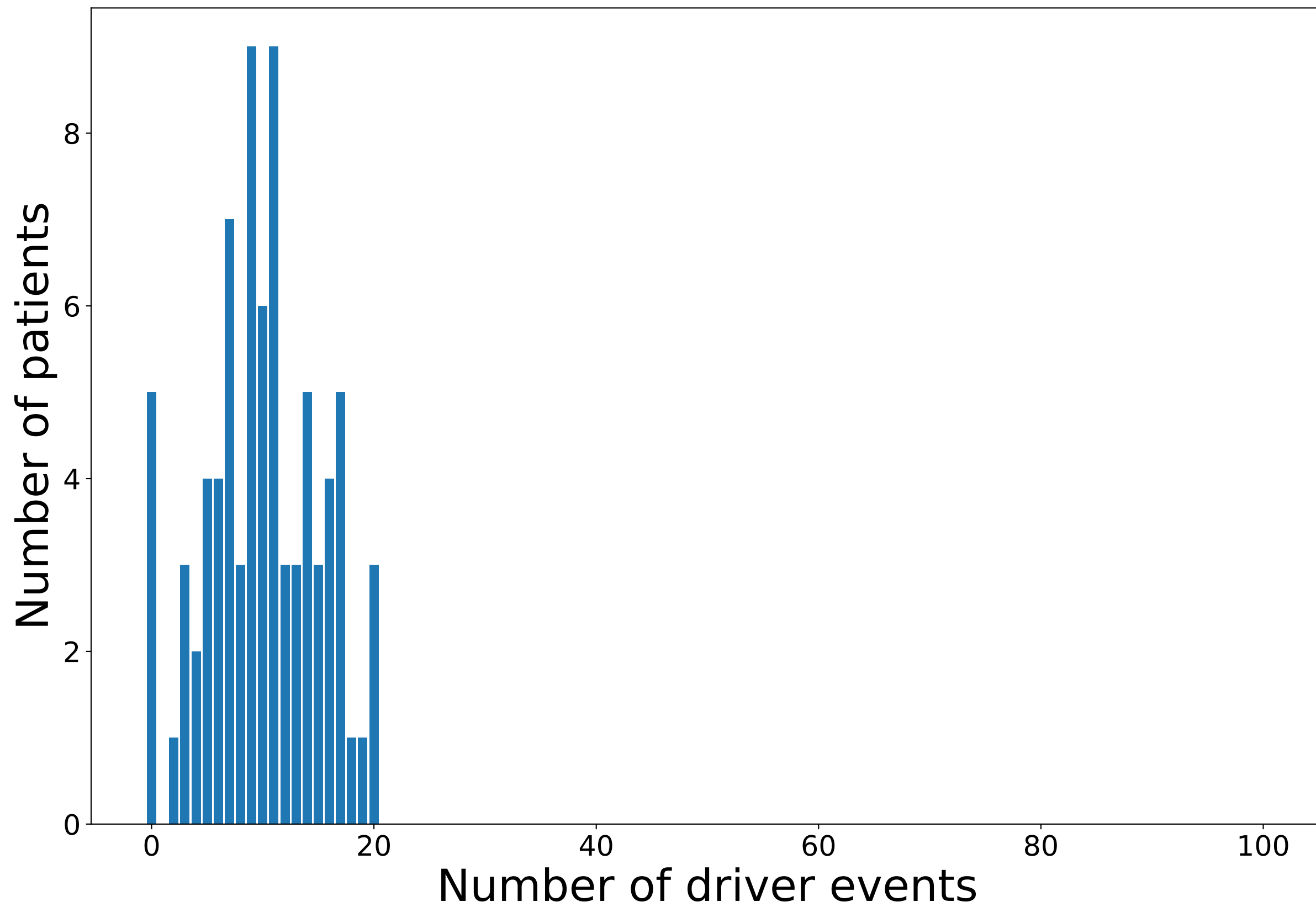

Supplement: Supplemental Information 2 [file peerj-10-13860-s002.zip › COHORTS/patient distributions/2021_8_16_14_9_ACC.pdf]

# PCPG\_FEMALE

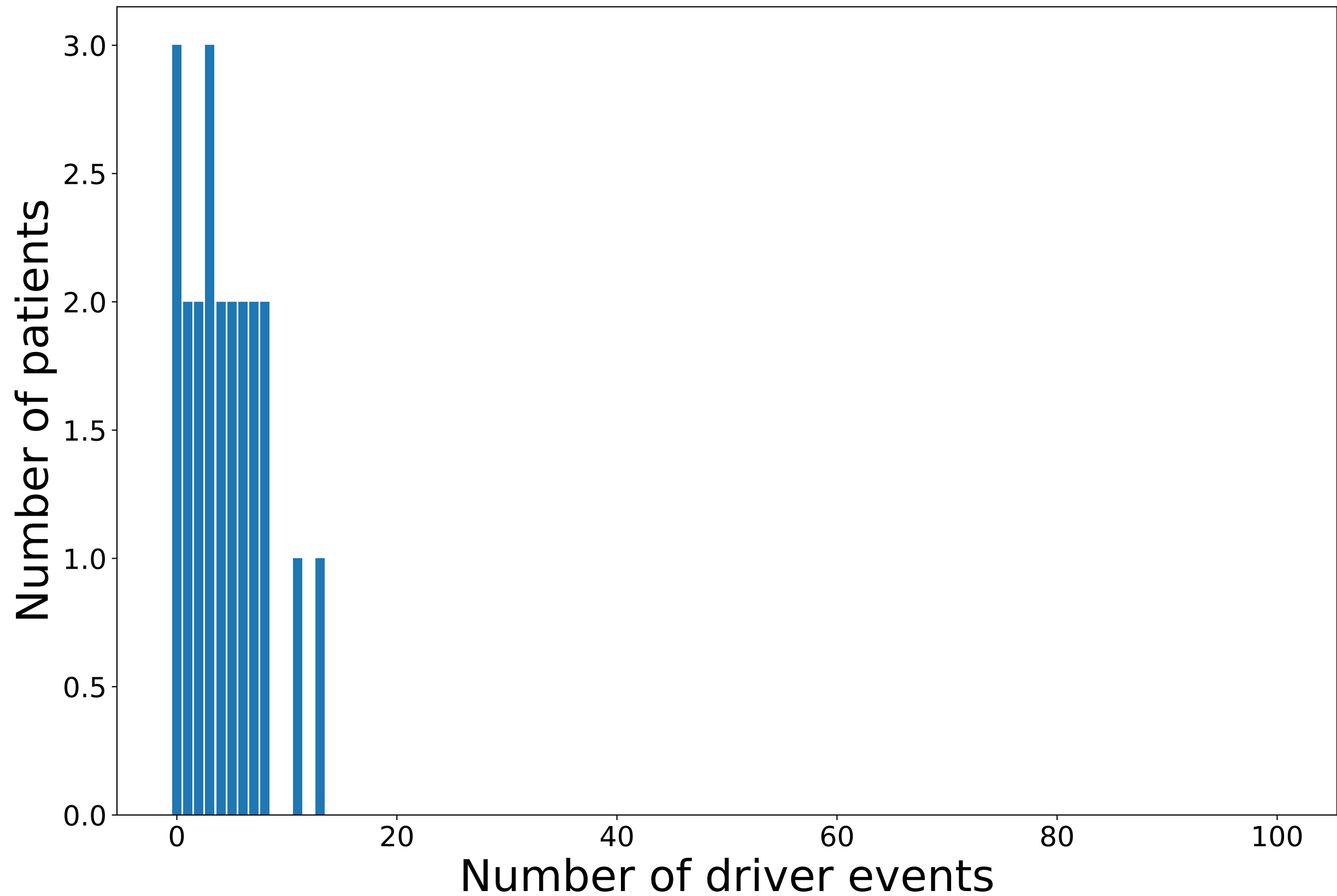

Supplement: Supplemental Information 2 [file peerj-10-13860-s002.zip › COHORTS/patient distributions/2021_8_16_14_9_PCPG_FEMALE.pdf]

# LGG

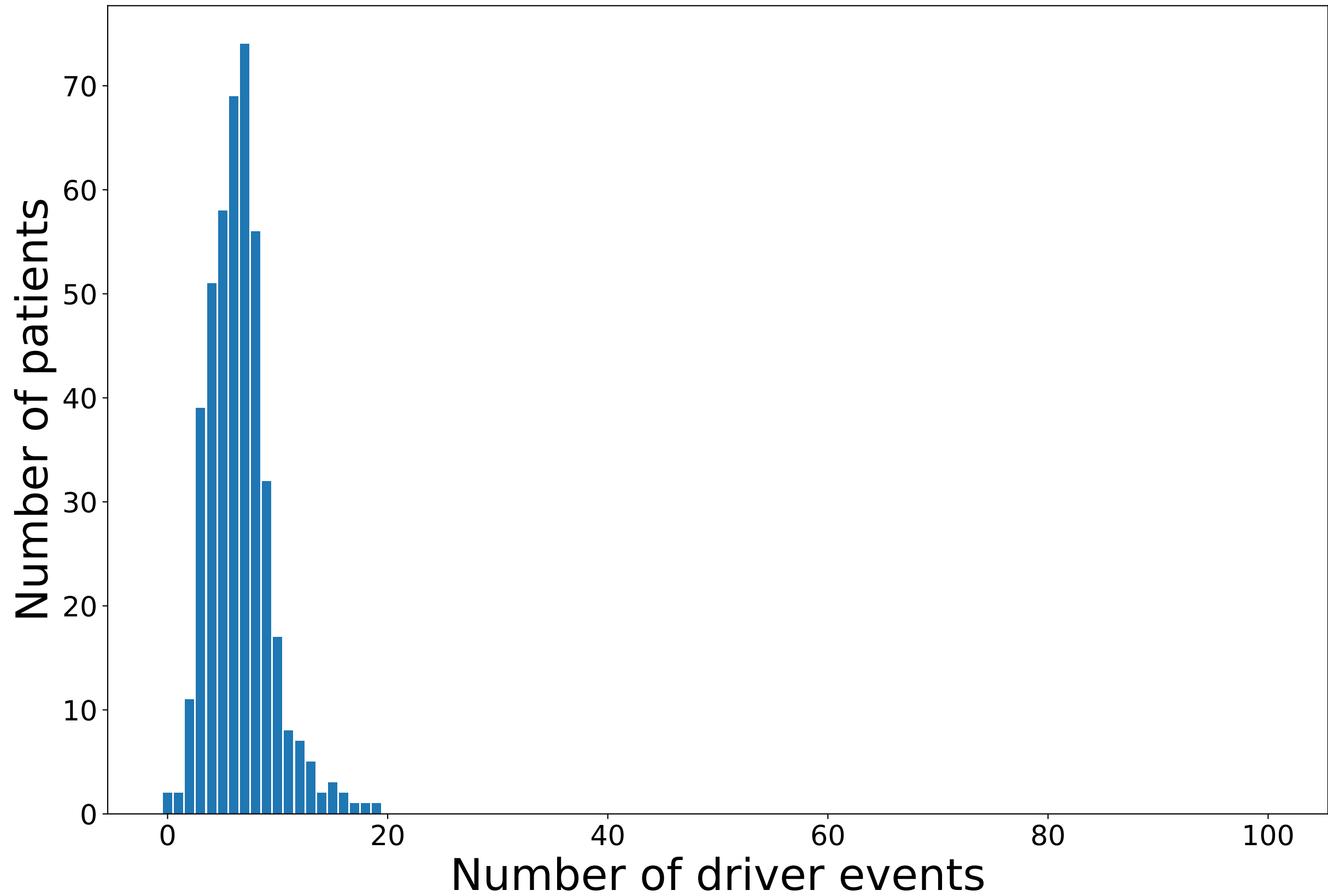

Supplement: Supplemental Information 2 [file peerj-10-13860-s002.zip › COHORTS/patient distributions/2021_8_16_14_9_LGG.pdf]

# READ

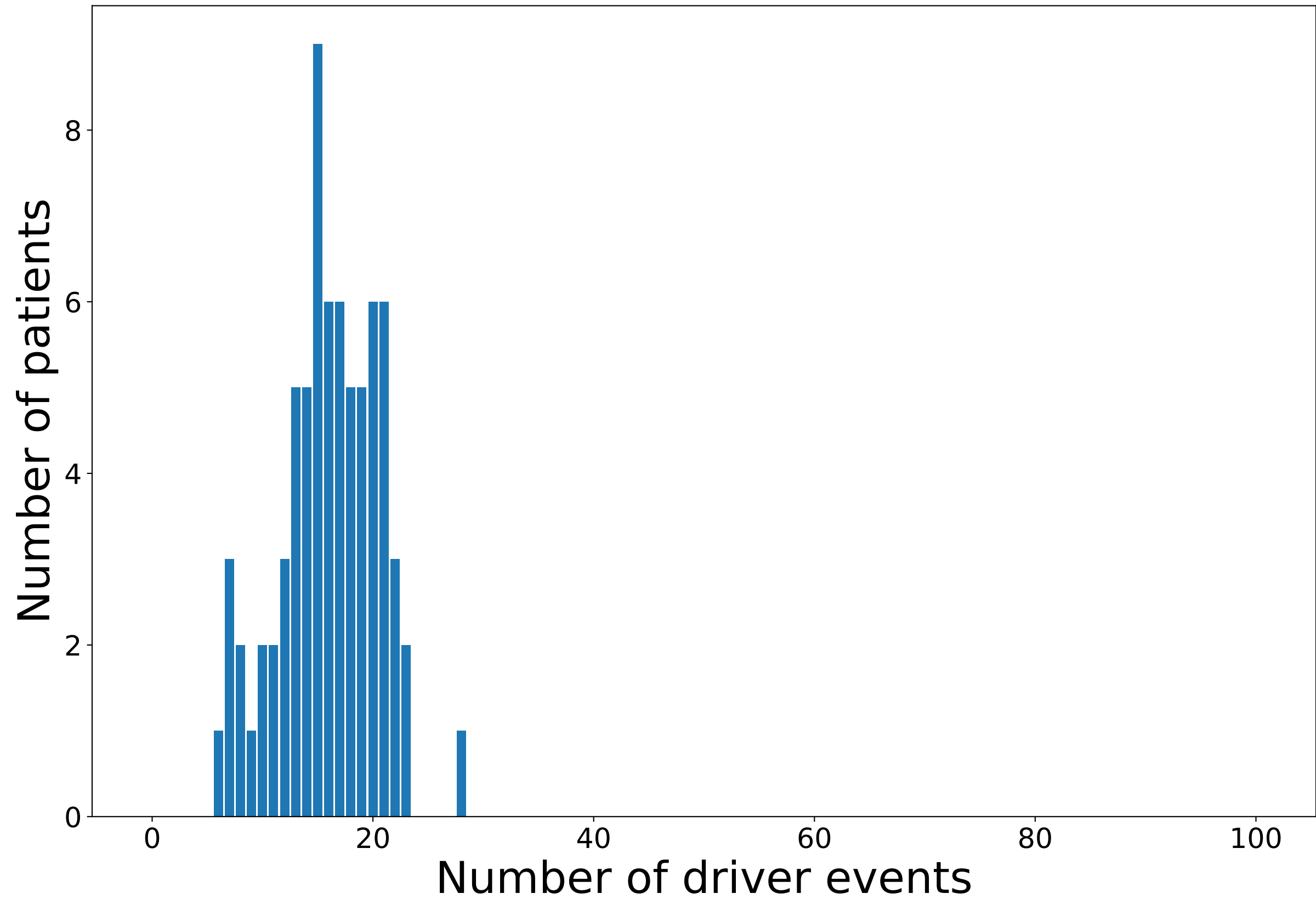

Supplement: Supplemental Information 2 [file peerj-10-13860-s002.zip › COHORTS/patient distributions/2021_8_16_14_9_READ.pdf]

# PAAD

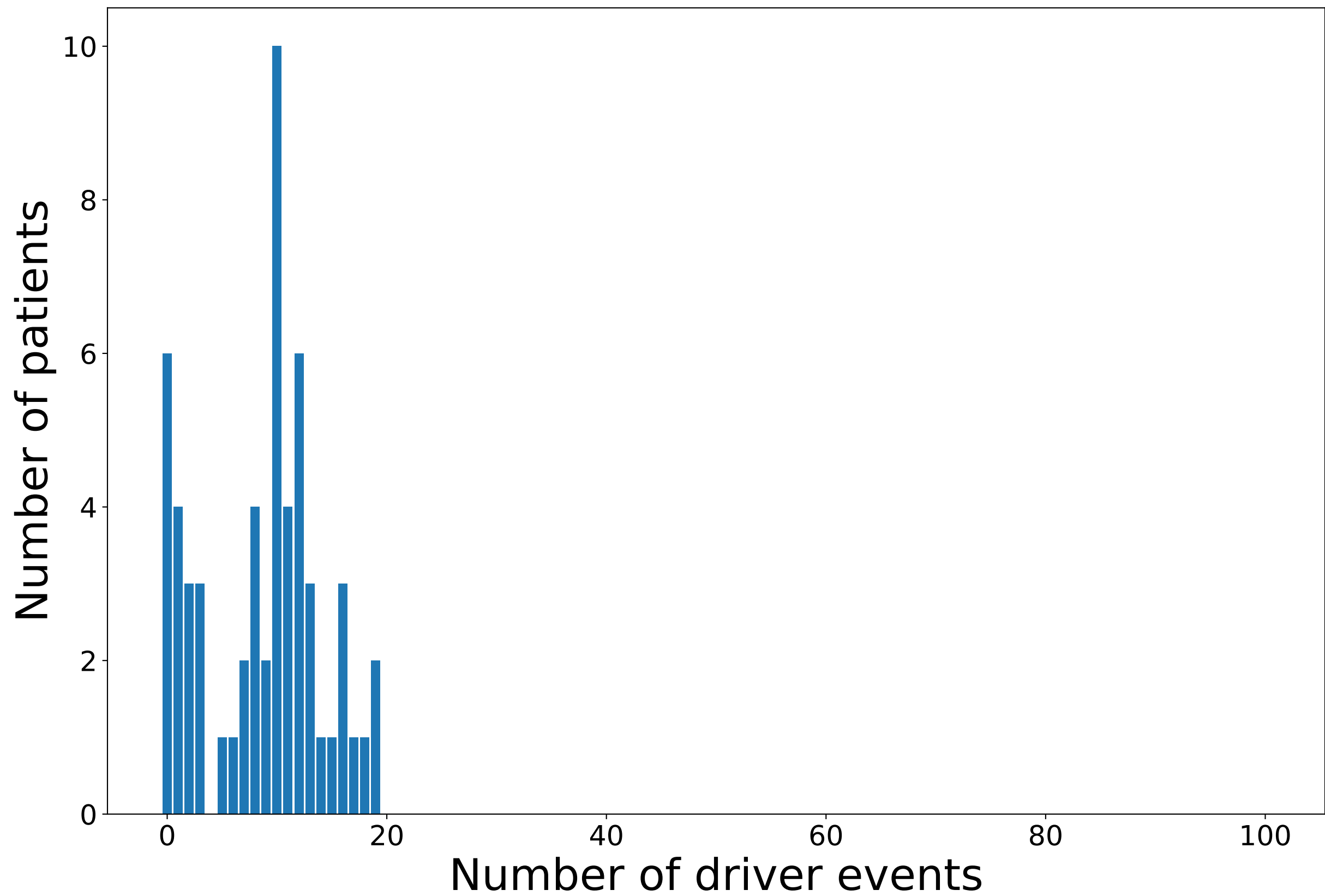

Supplement: Supplemental Information 2 [file peerj-10-13860-s002.zip › COHORTS/patient distributions/2021_8_16_14_9_PAAD.pdf]

# THCA

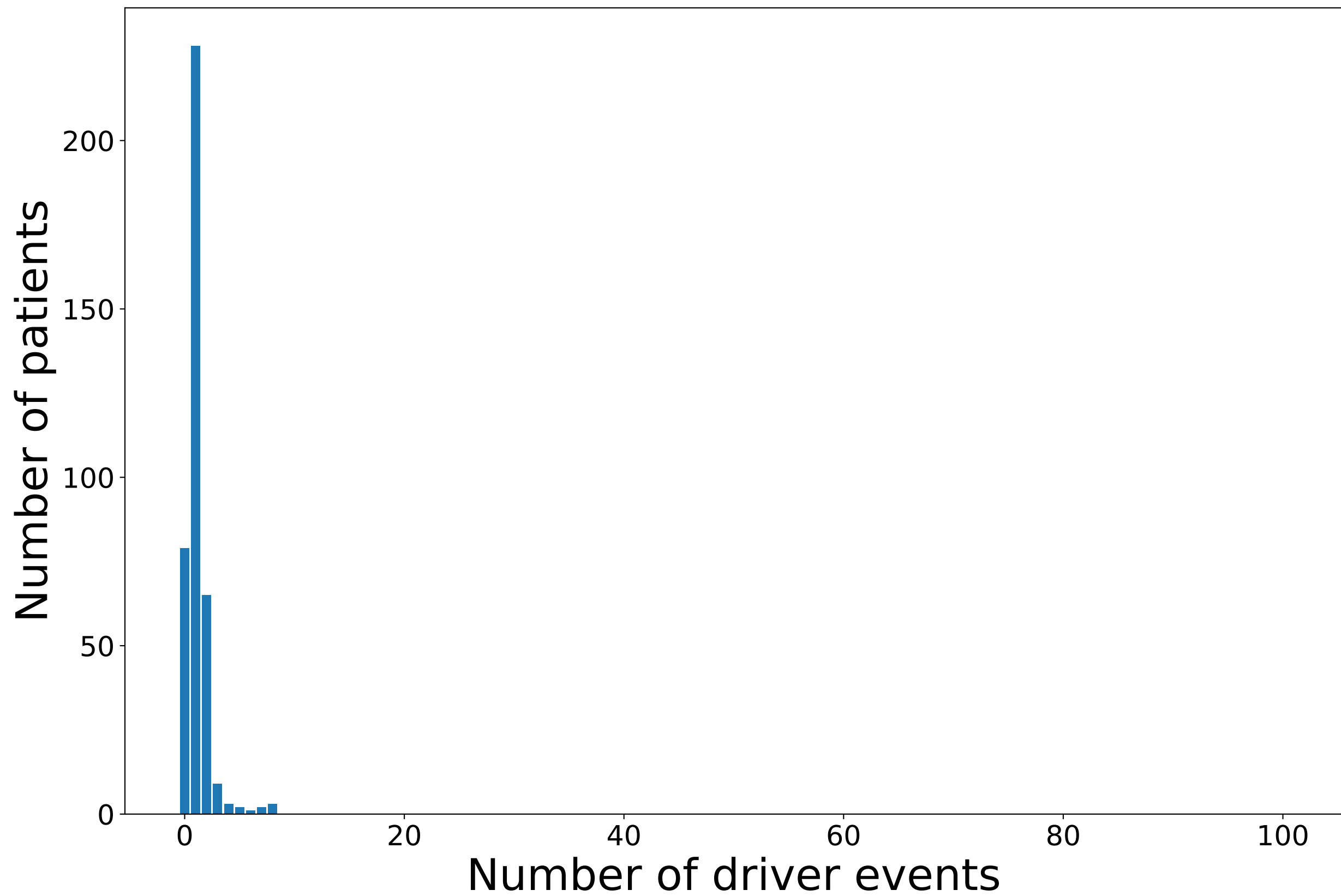

Supplement: Supplemental Information 2 [file peerj-10-13860-s002.zip › COHORTS/patient distributions/2021_8_16_14_9_THCA.pdf]

# BLCA

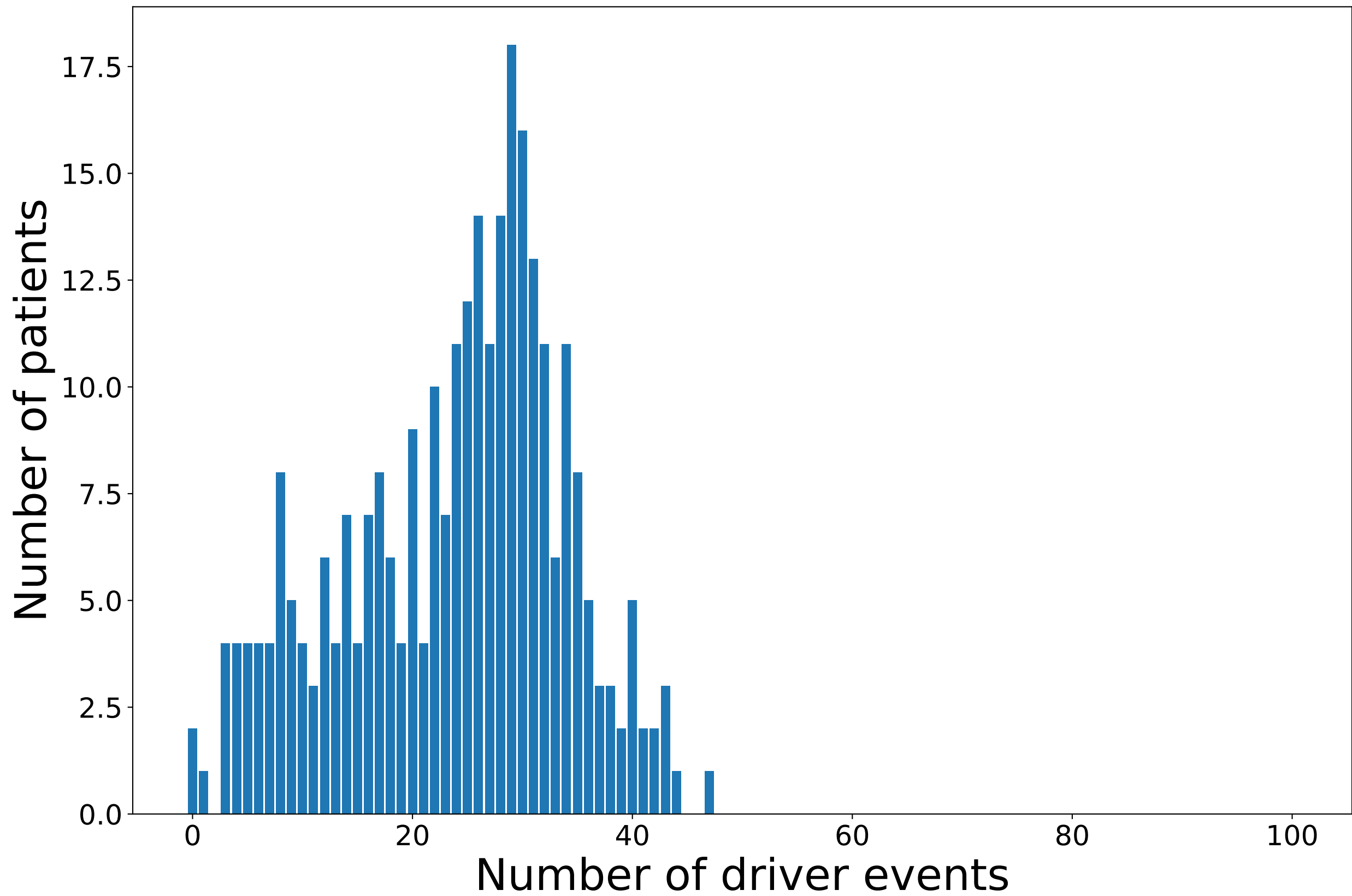

Supplement: Supplemental Information 2 [file peerj-10-13860-s002.zip › COHORTS/patient distributions/2021_8_16_14_9_BLCA.pdf]

# PCPG\_MALE

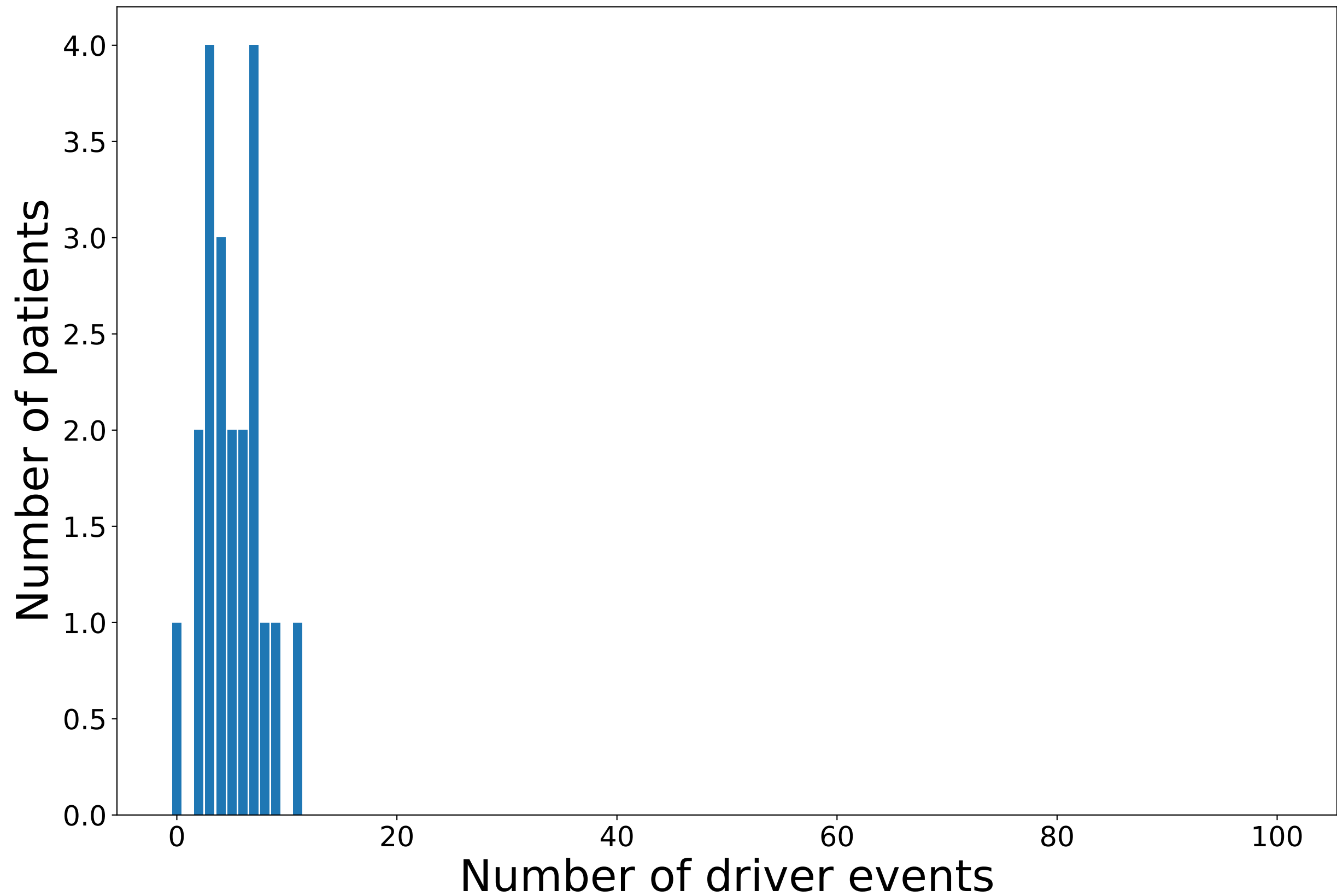

Supplement: Supplemental Information 2 [file peerj-10-13860-s002.zip › COHORTS/patient distributions/2021_8_16_14_9_PCPG_MALE.pdf]

# THCA\_MALE

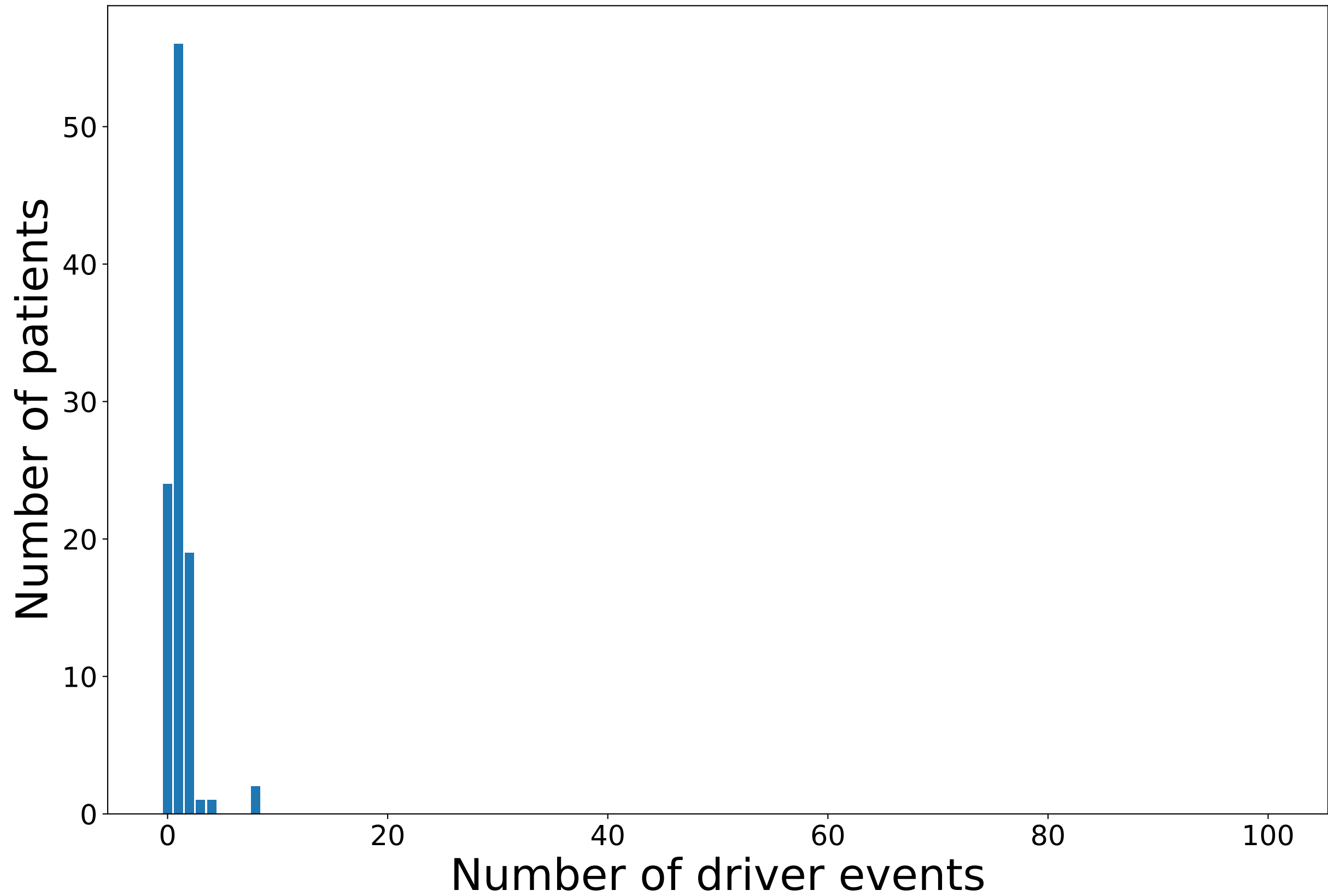

Supplement: Supplemental Information 2 [file peerj-10-13860-s002.zip › COHORTS/patient distributions/2021_8_16_14_9_THCA_MALE.pdf]

# READ\_MALE

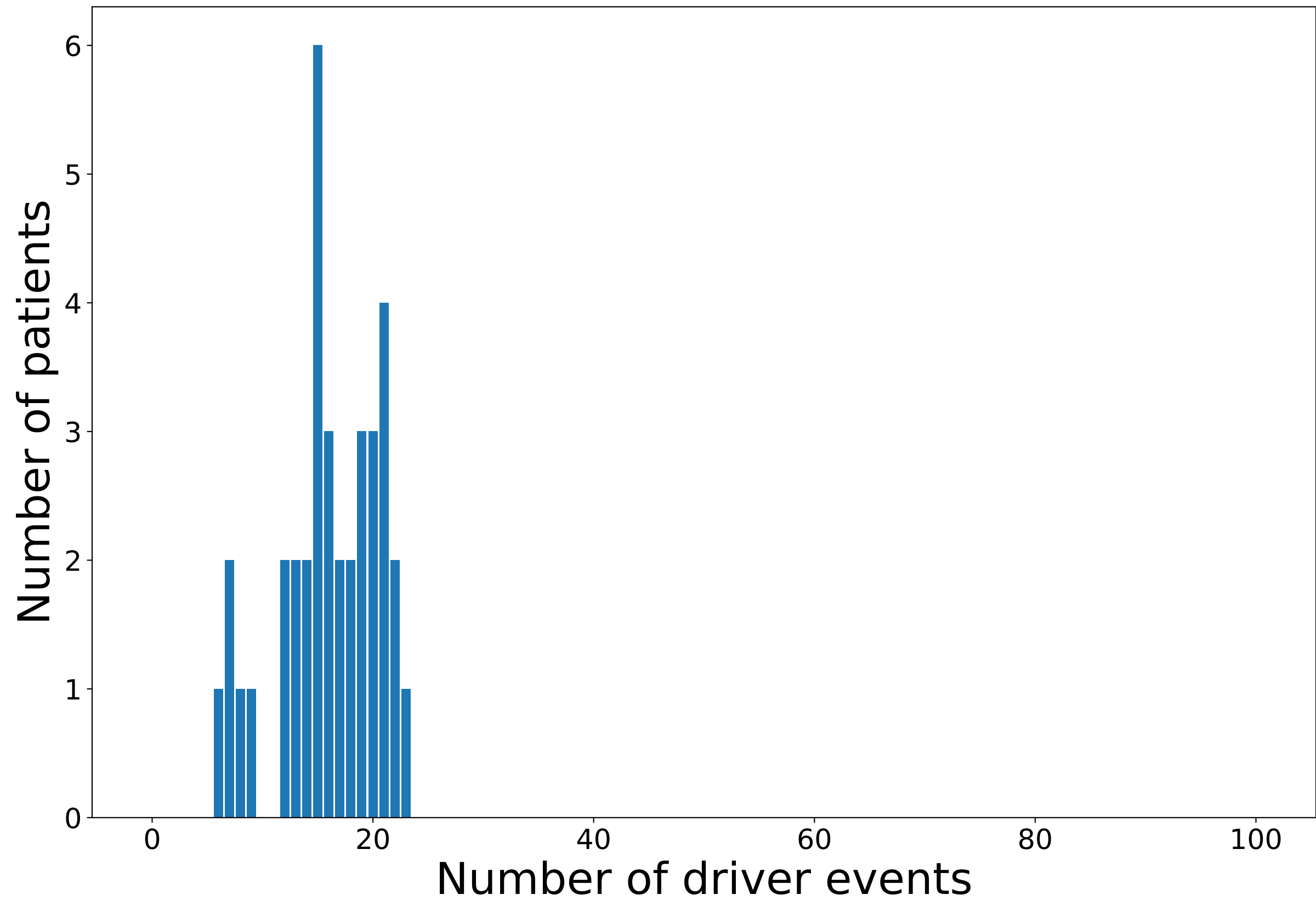

Supplement: Supplemental Information 2 [file peerj-10-13860-s002.zip › COHORTS/patient distributions/2021_8_16_14_9_READ_MALE.pdf]

# THYM

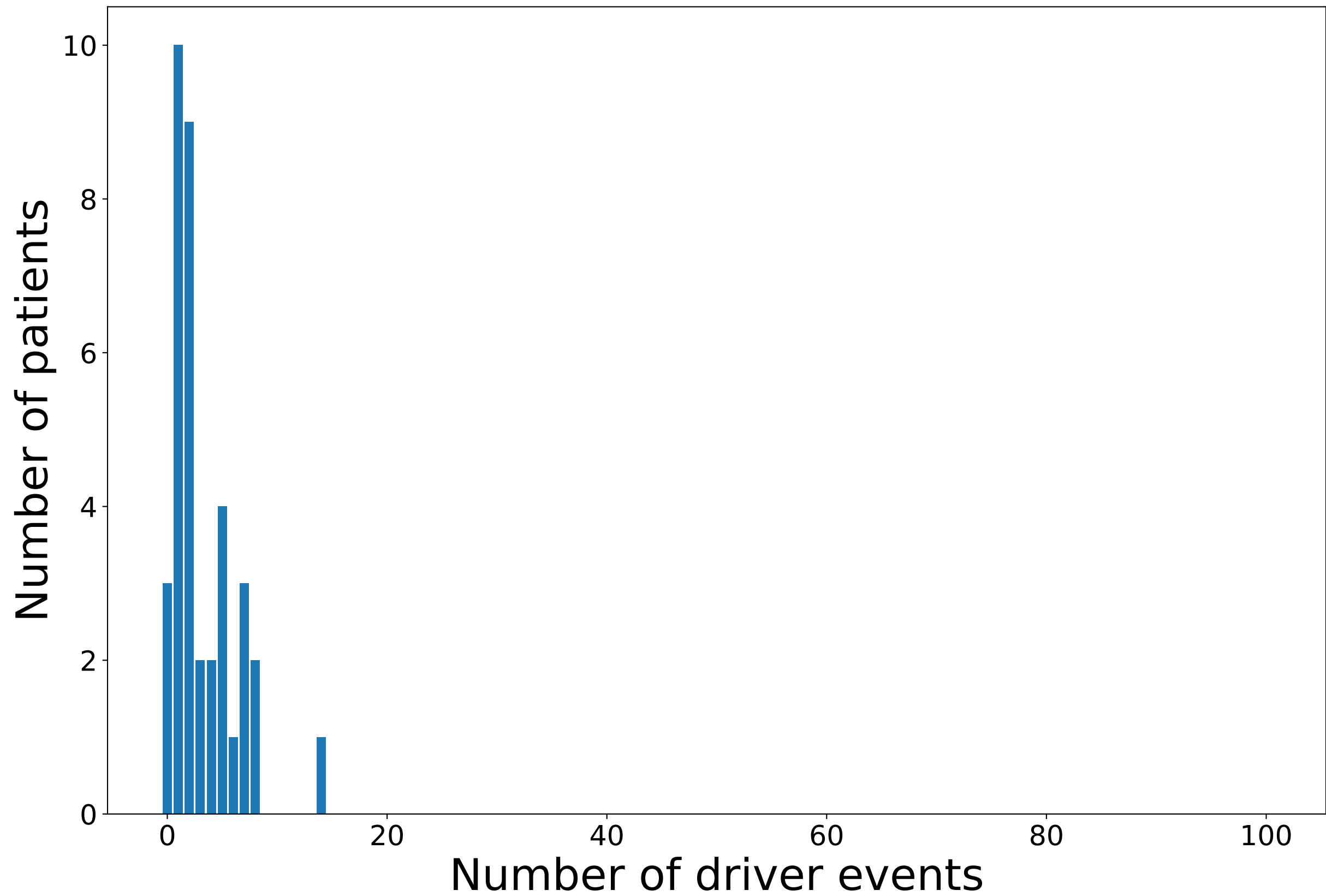

Supplement: Supplemental Information 2 [file peerj-10-13860-s002.zip › COHORTS/patient distributions/2021_8_16_14_9_THYM.pdf]

# THYM\_FEMALE

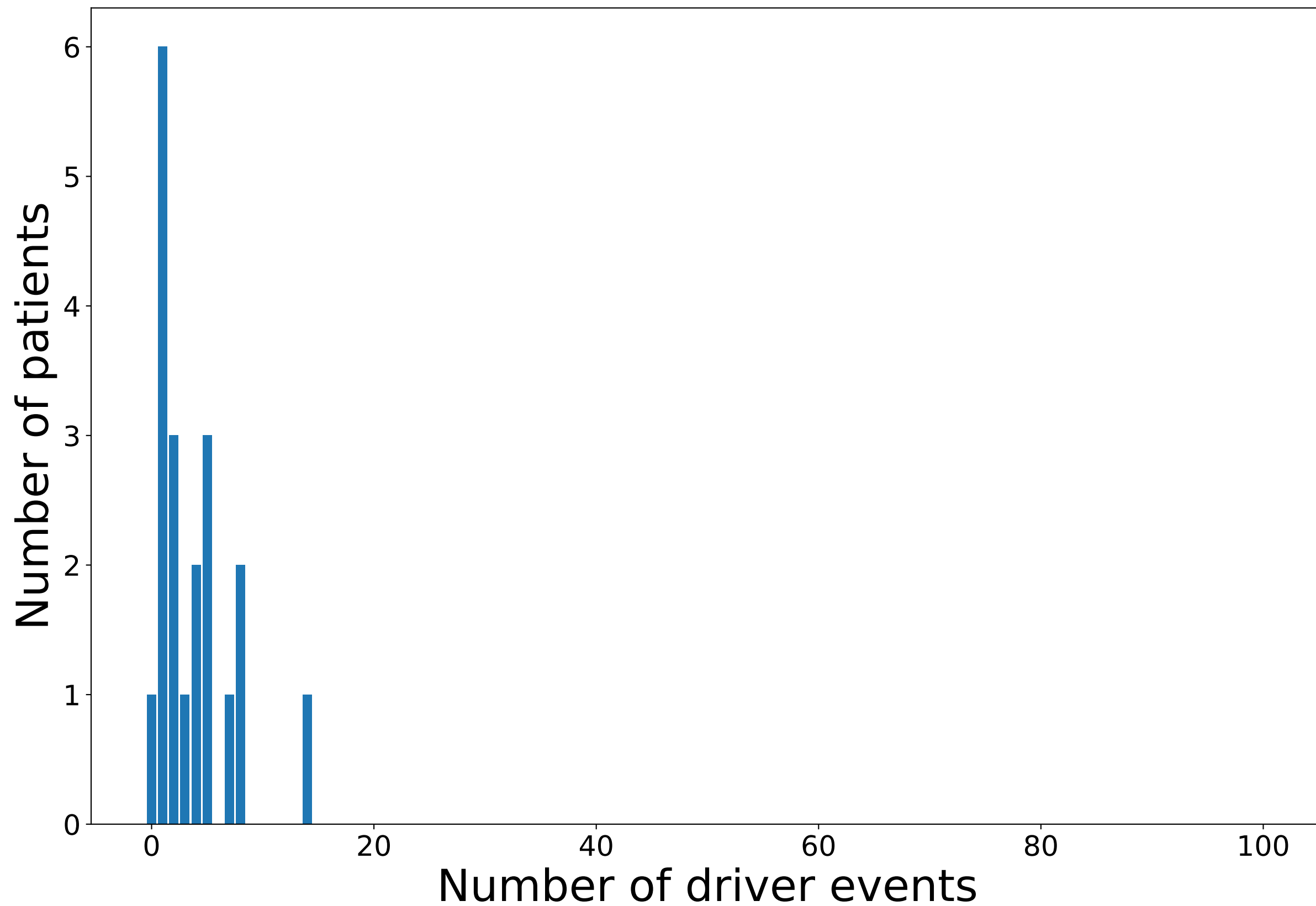

Supplement: Supplemental Information 2 [file peerj-10-13860-s002.zip › COHORTS/patient distributions/2021_8_16_14_9_THYM_FEMALE.pdf]

# KICH\_MALE

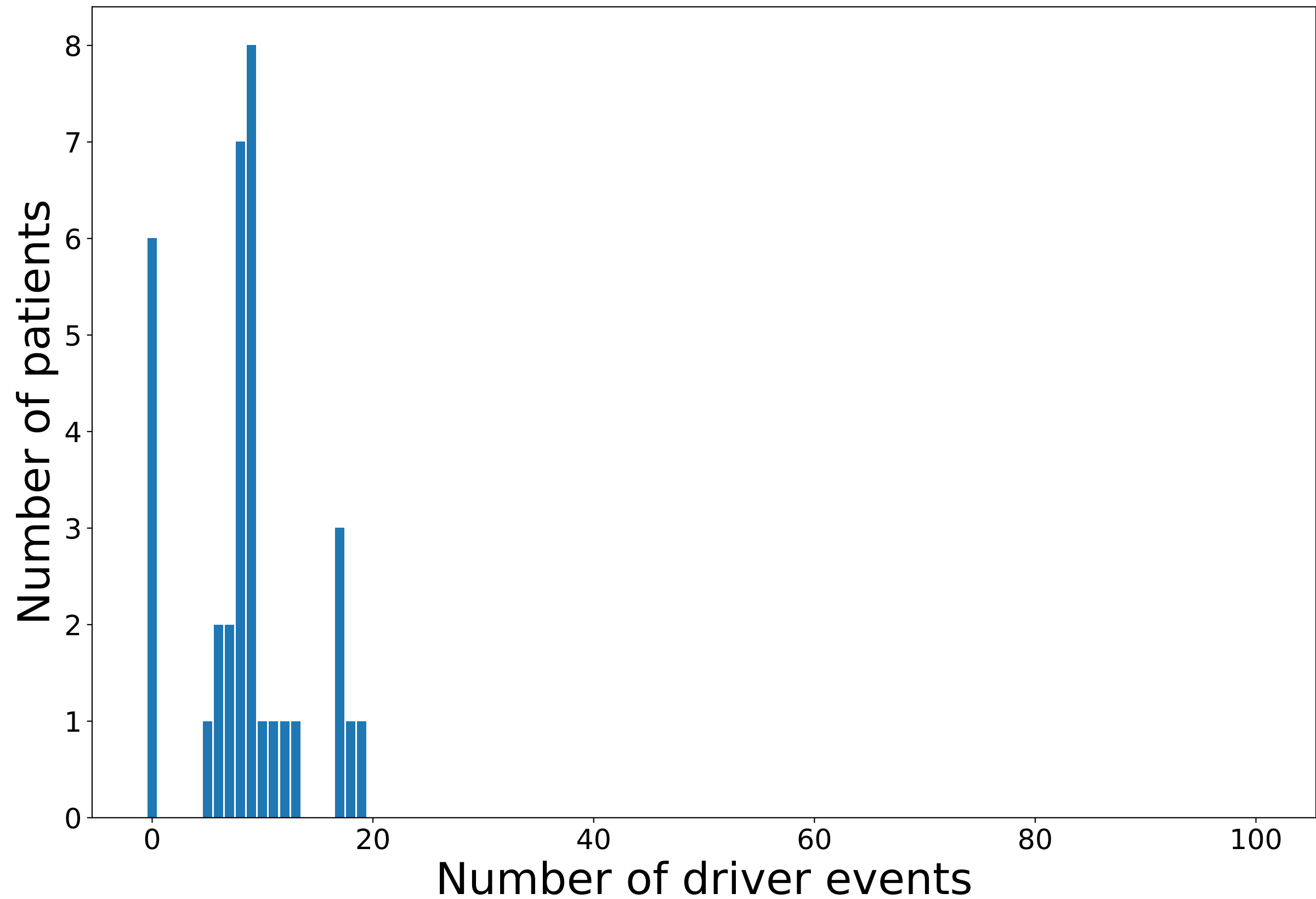

Supplement: Supplemental Information 2 [file peerj-10-13860-s002.zip › COHORTS/patient distributions/2021_8_16_14_9_KICH_MALE.pdf]

# KIRC

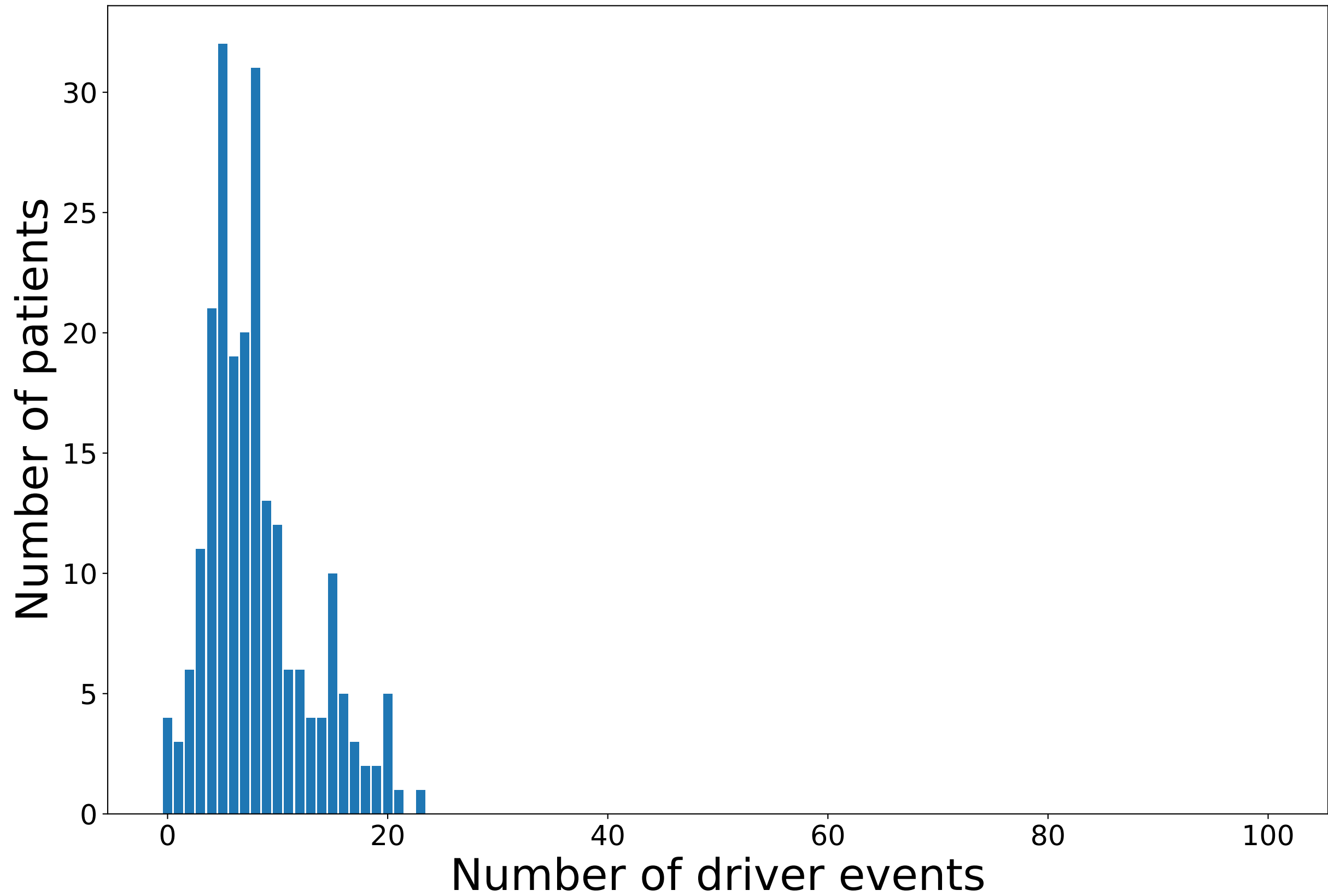

Supplement: Supplemental Information 2 [file peerj-10-13860-s002.zip › COHORTS/patient distributions/2021_8_16_14_9_KIRC.pdf]

# HNSC\_MALE

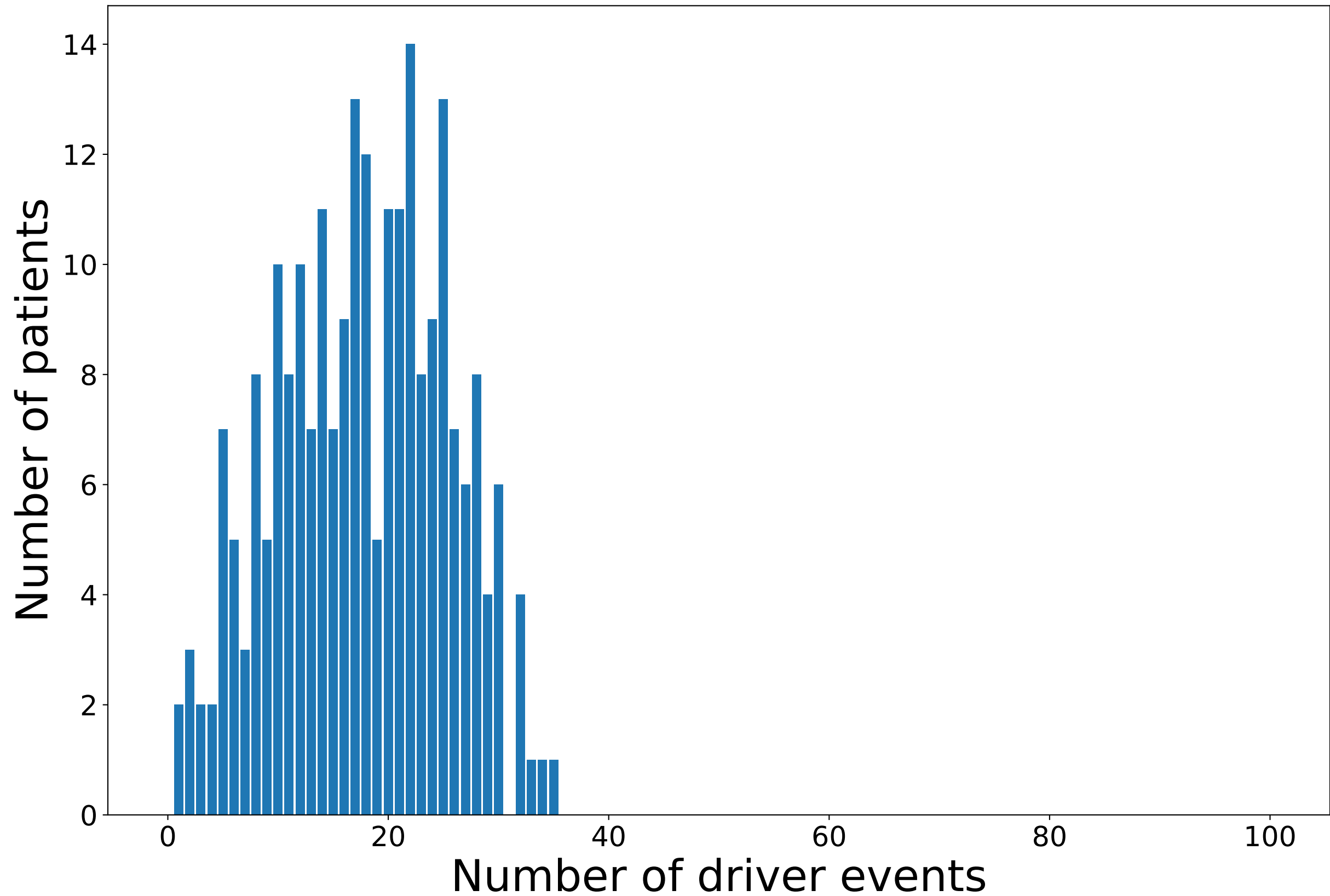

Supplement: Supplemental Information 2 [file peerj-10-13860-s002.zip › COHORTS/patient distributions/2021_8_16_14_9_HNSC_MALE.pdf]

# LGG\_FEMALE

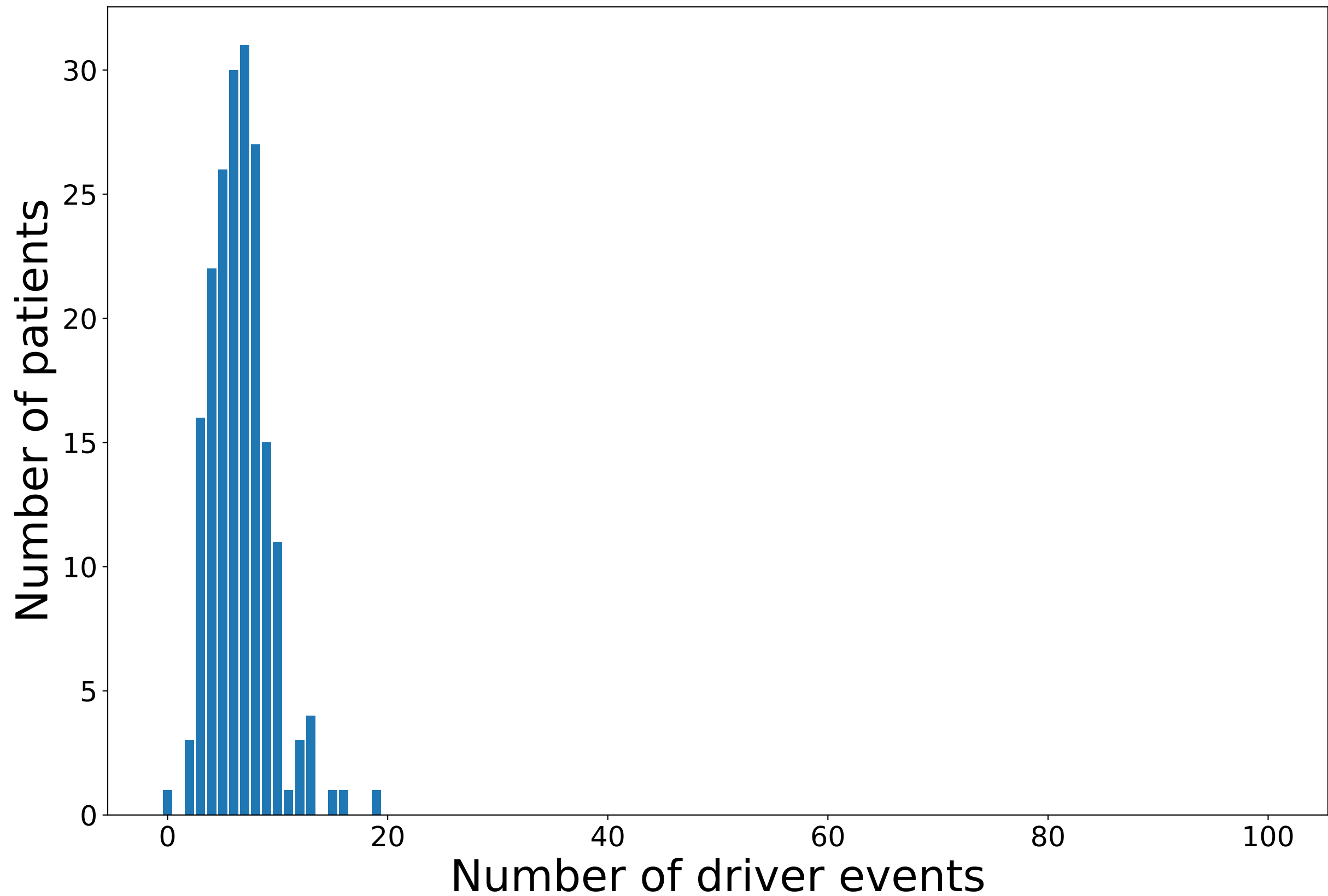

Supplement: Supplemental Information 2 [file peerj-10-13860-s002.zip › COHORTS/patient distributions/2021_8_16_14_9_LGG_FEMALE.pdf]

# KIRP\_MALE

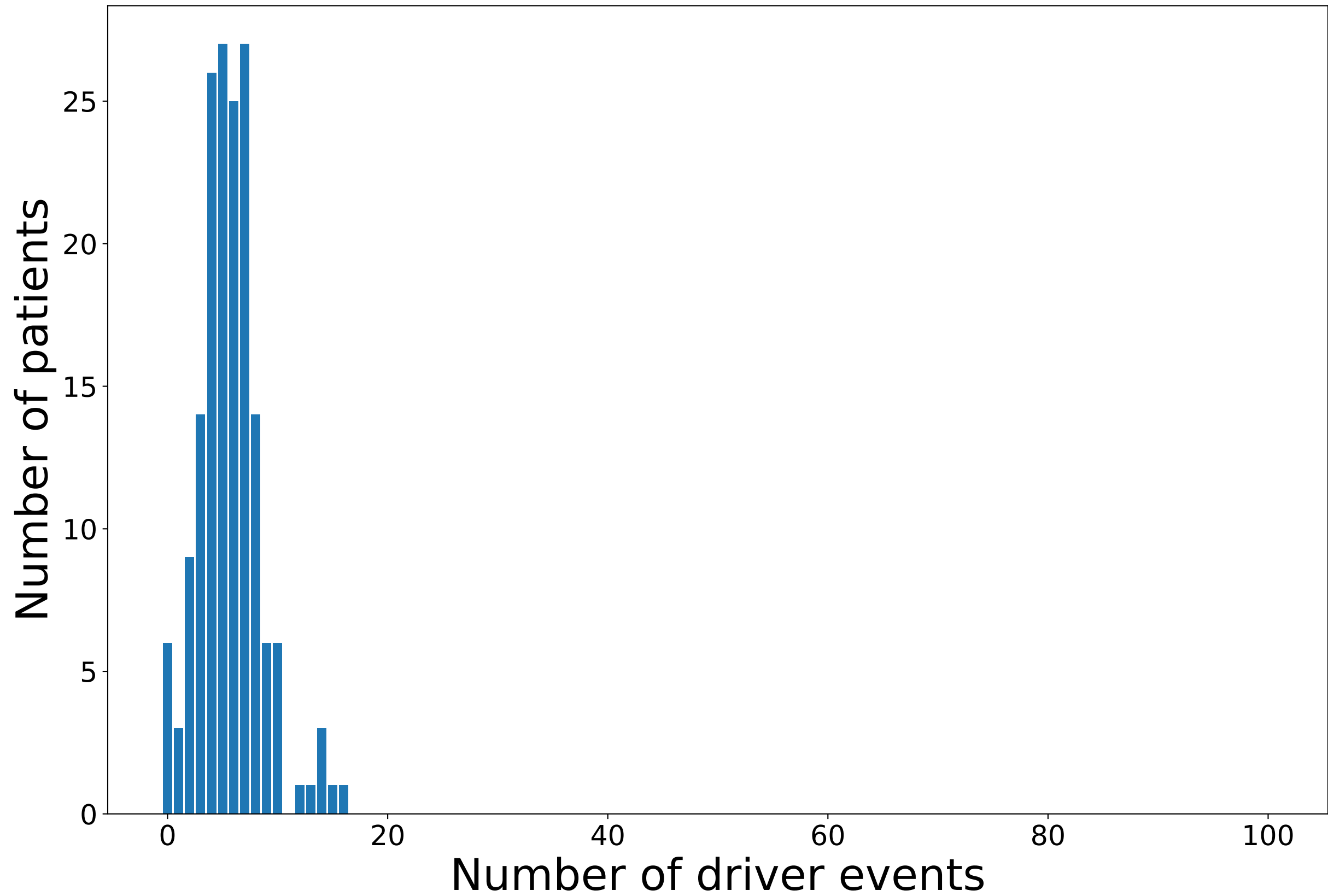

Supplement: Supplemental Information 2 [file peerj-10-13860-s002.zip › COHORTS/patient distributions/2021_8_16_14_9_KIRP_MALE.pdf]

# GBM\_FEMALE

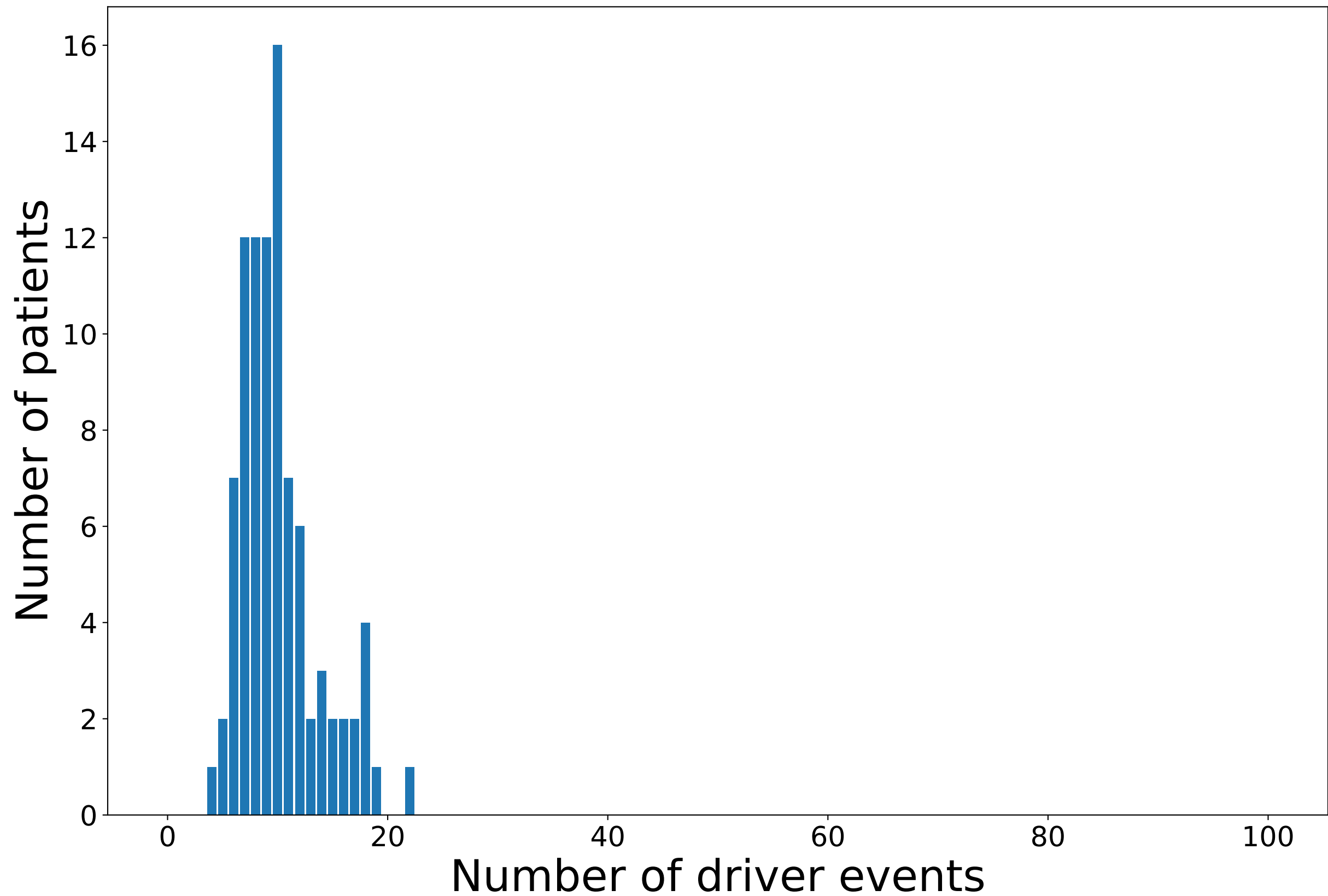

Supplement: Supplemental Information 2 [file peerj-10-13860-s002.zip › COHORTS/patient distributions/2021_8_16_14_9_GBM_FEMALE.pdf]

# MESO\_FEMALE

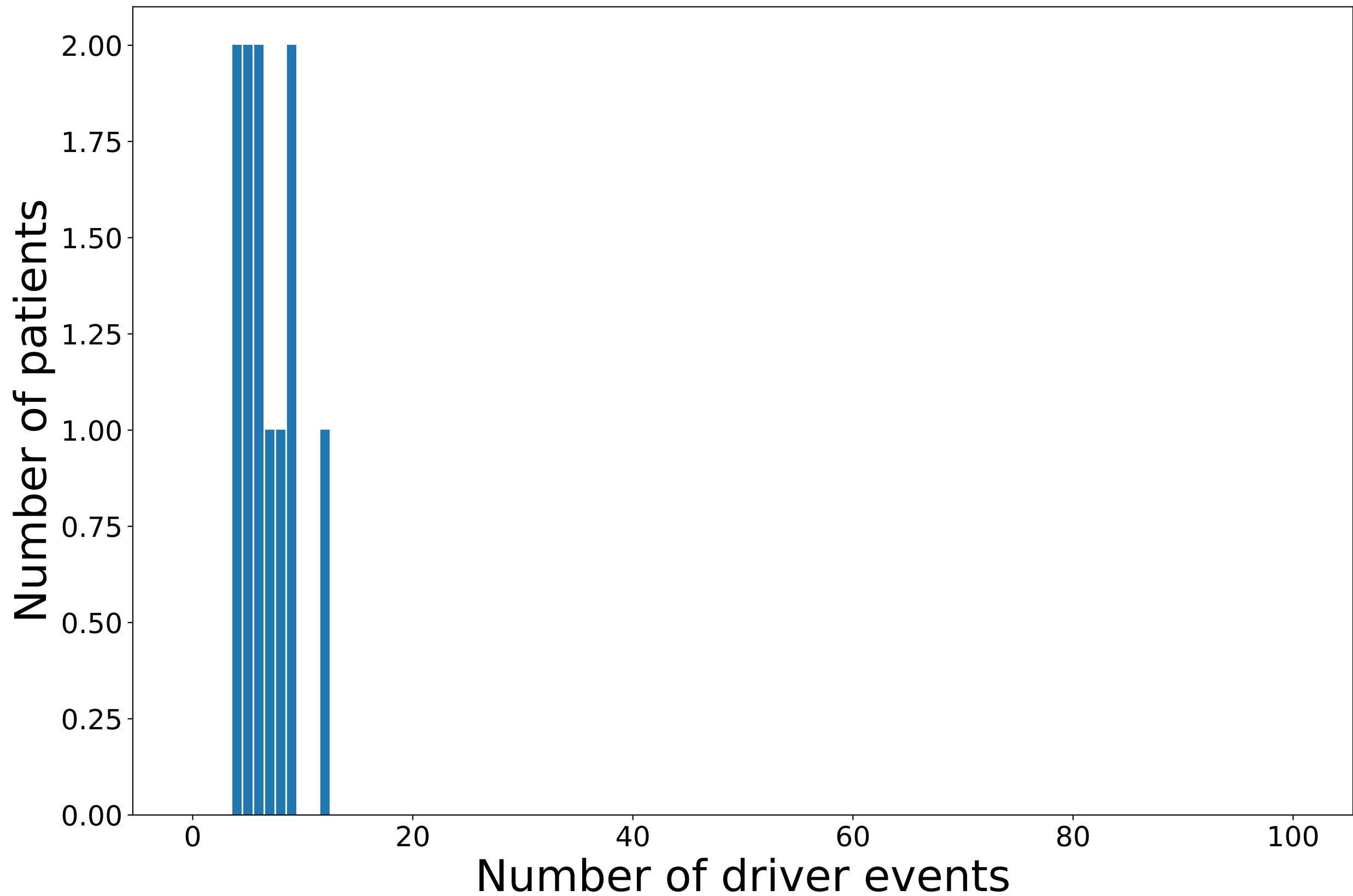

Supplement: Supplemental Information 2 [file peerj-10-13860-s002.zip › COHORTS/patient distributions/2021_8_16_14_9_MESO_FEMALE.pdf]

# ESCA

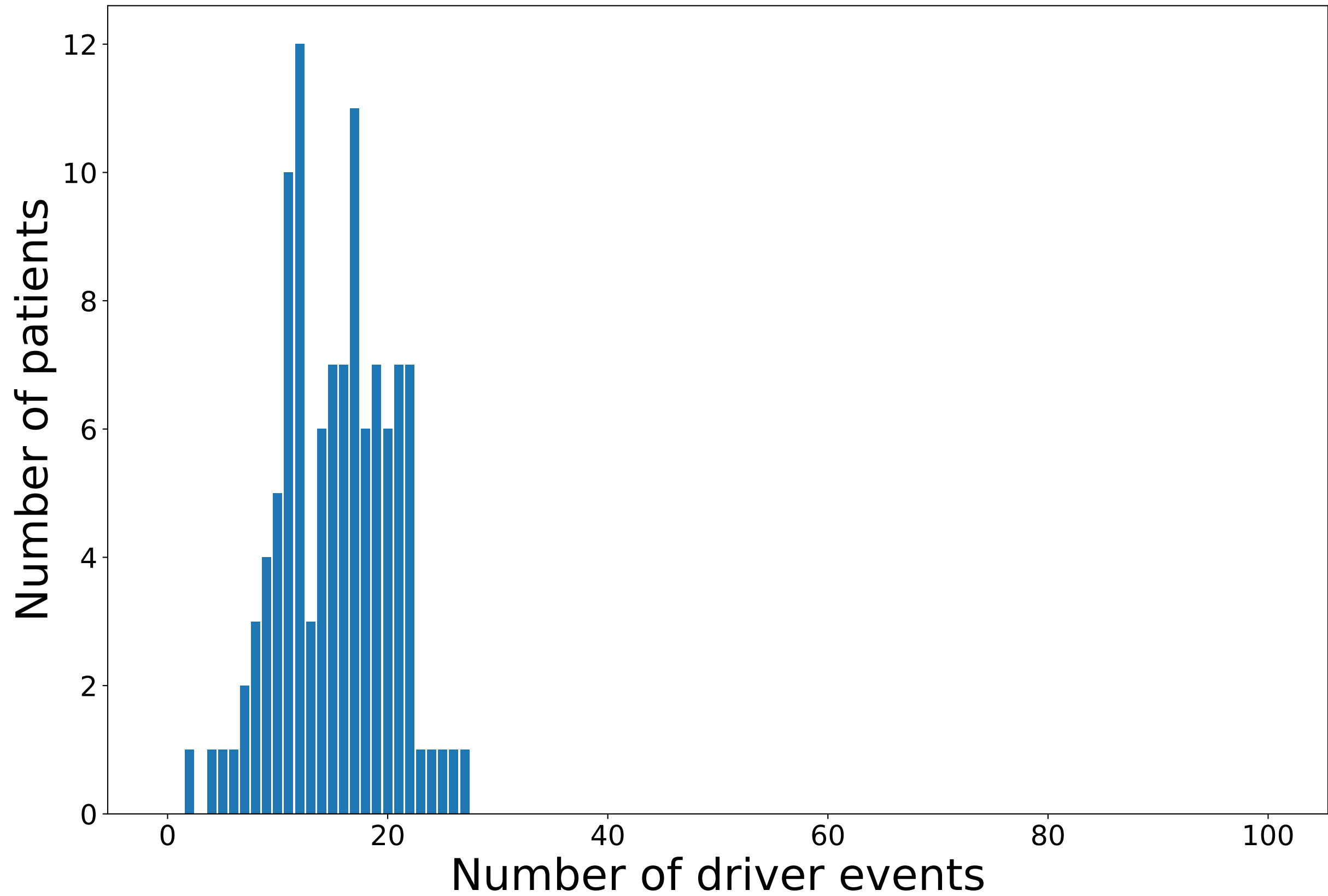

Supplement: Supplemental Information 2 [file peerj-10-13860-s002.zip › COHORTS/patient distributions/2021_8_16_14_9_ESCA.pdf]

# PANCAN\_MALE

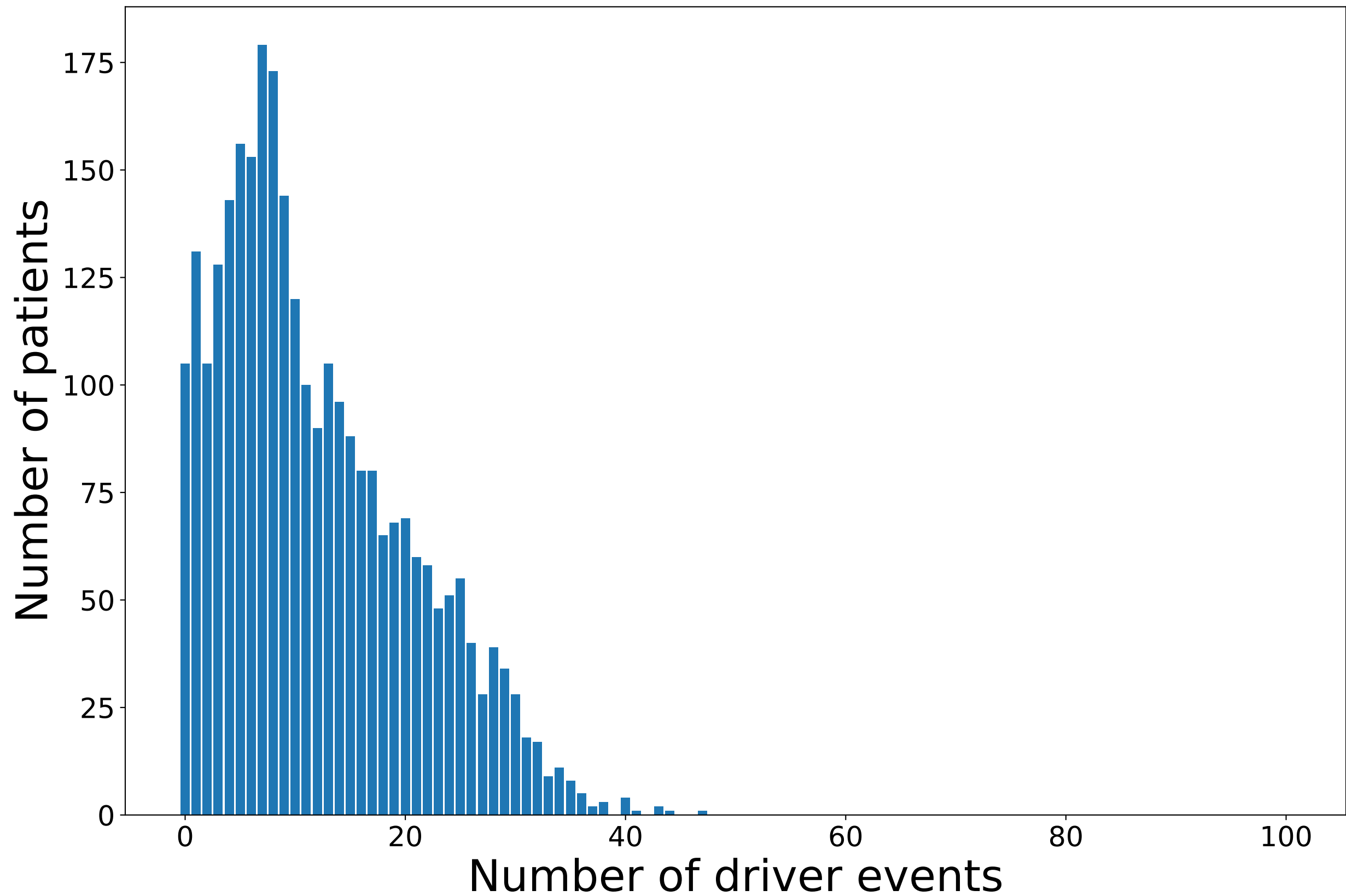

Supplement: Supplemental Information 2 [file peerj-10-13860-s002.zip › COHORTS/patient distributions/2021_8_16_14_9_PANCAN_MALE.pdf]

# HNSC\_FEMALE

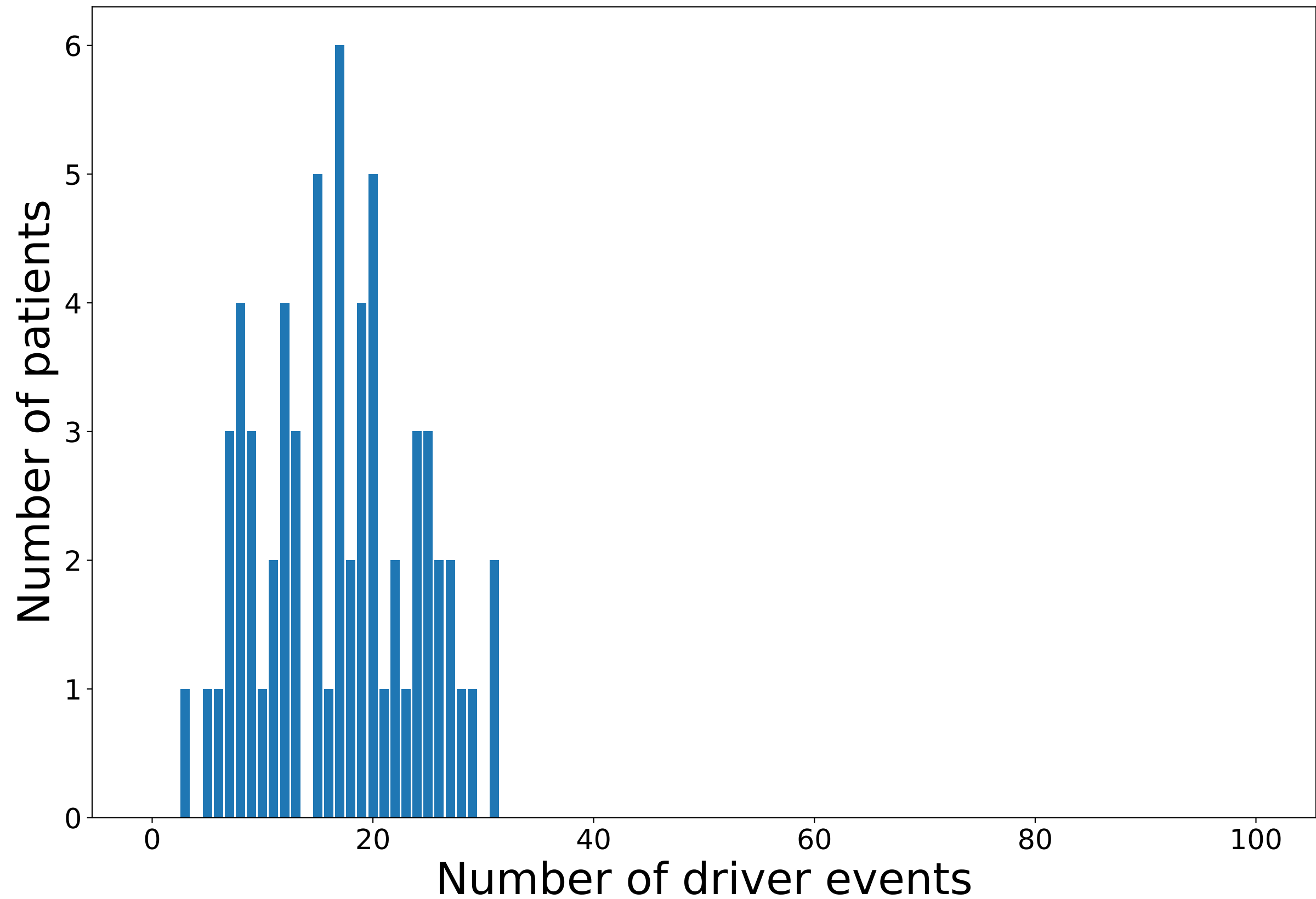

Supplement: Supplemental Information 2 [file peerj-10-13860-s002.zip › COHORTS/patient distributions/2021_8_16_14_9_HNSC_FEMALE.pdf]

# THYM\_MALE

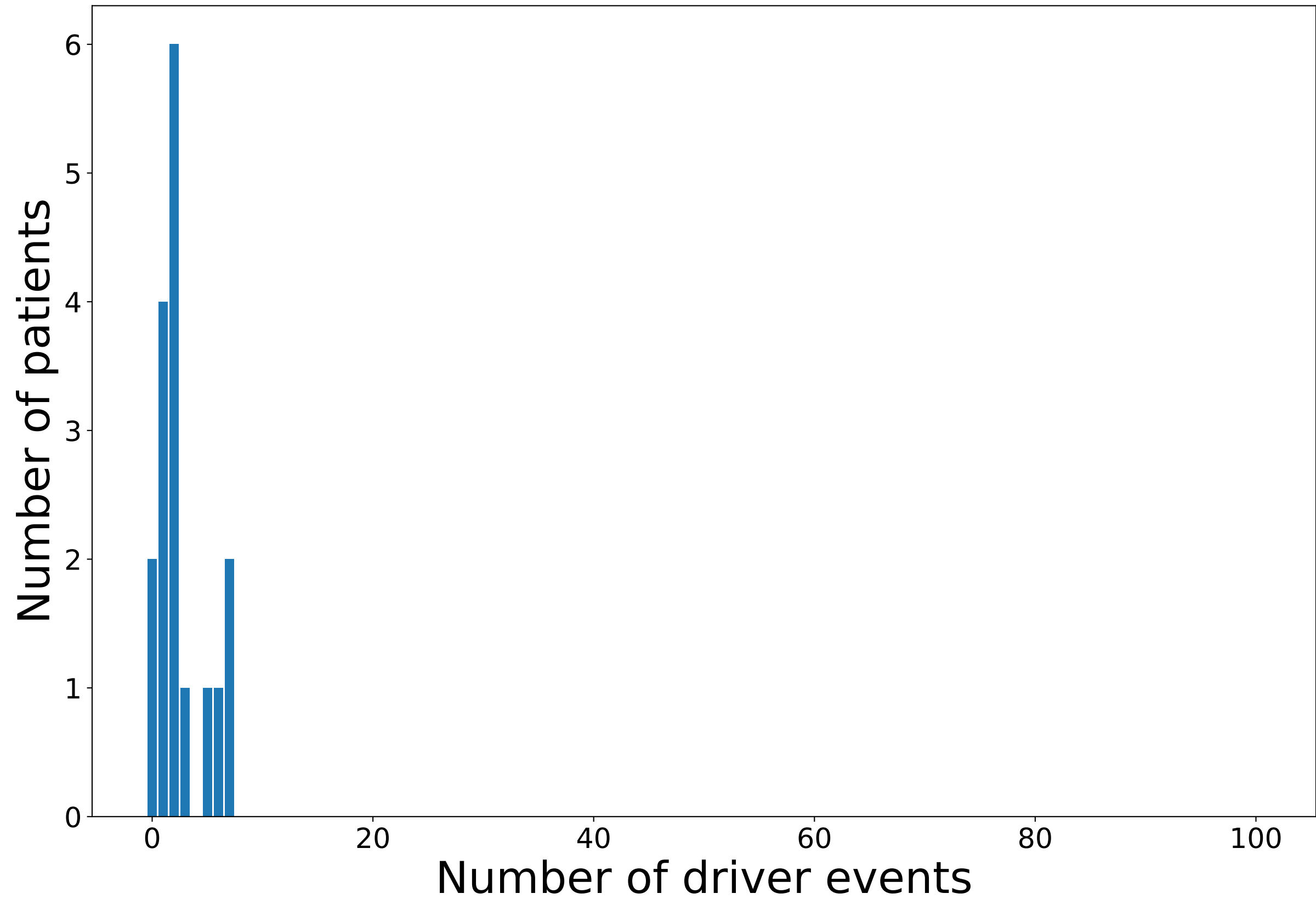

Supplement: Supplemental Information 2 [file peerj-10-13860-s002.zip › COHORTS/patient distributions/2021_8_16_14_9_THYM_MALE.pdf]

# STAD\_MALE

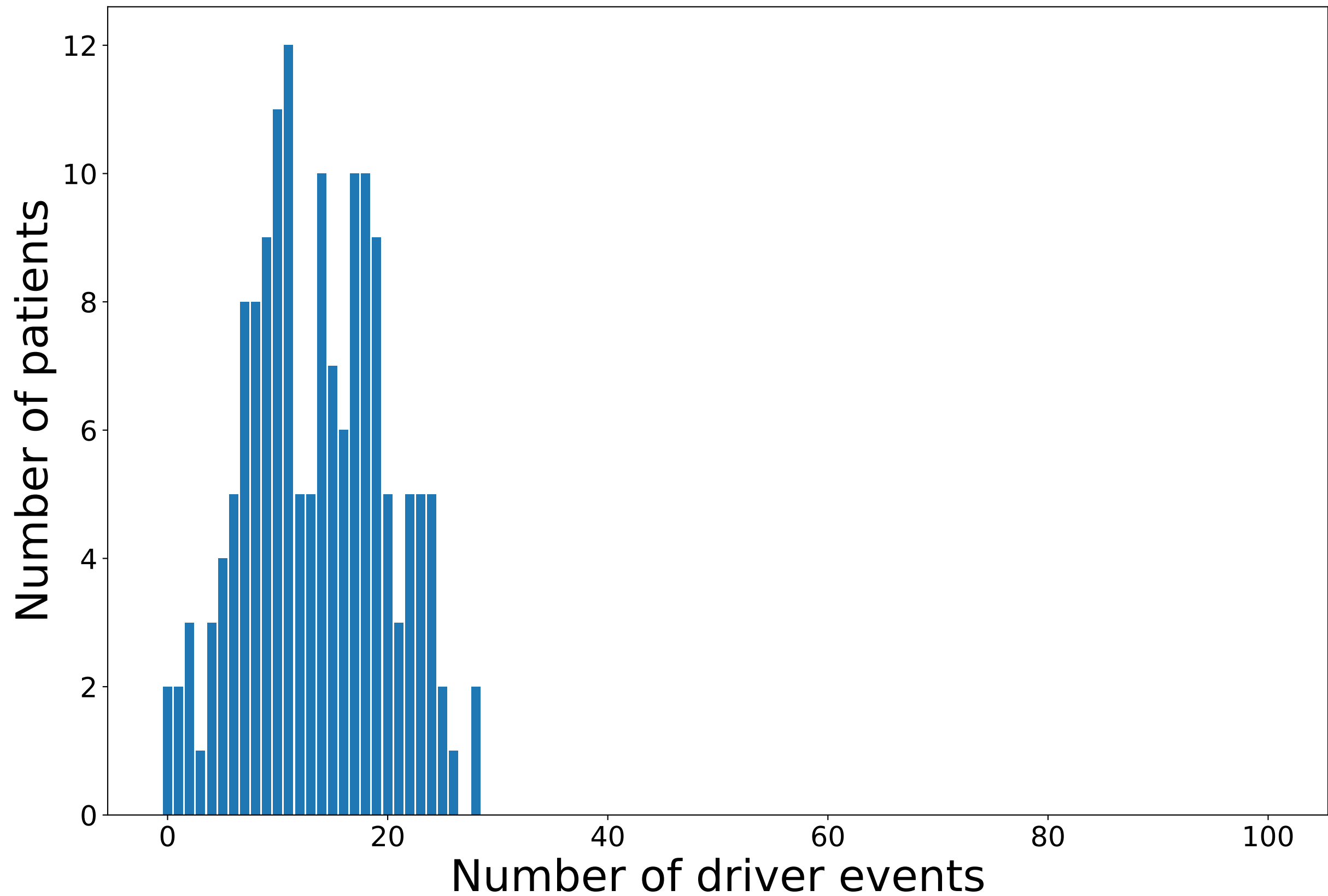

Supplement: Supplemental Information 2 [file peerj-10-13860-s002.zip › COHORTS/patient distributions/2021_8_16_14_9_STAD_MALE.pdf]

# HNSC

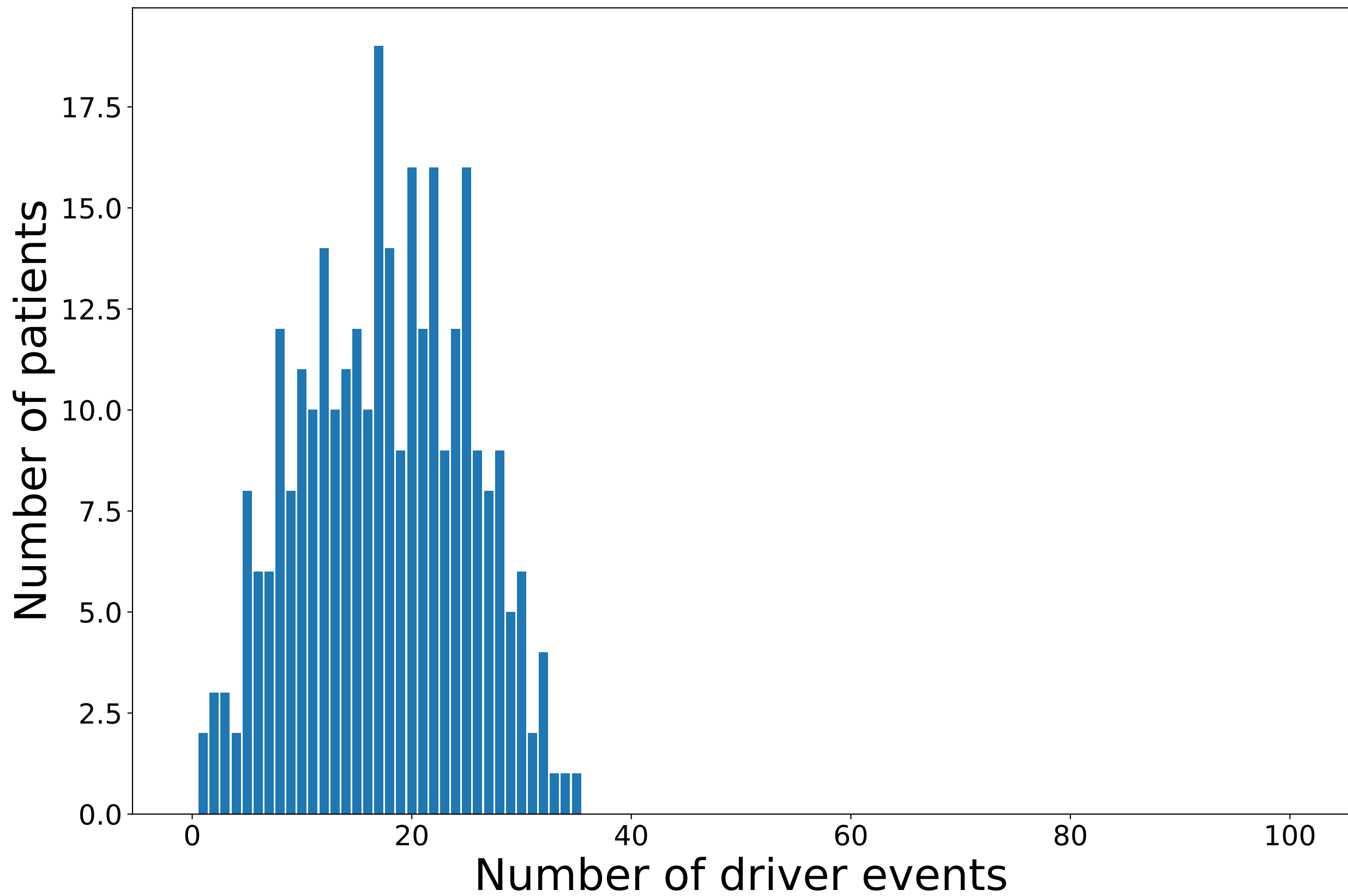

Supplement: Supplemental Information 2 [file peerj-10-13860-s002.zip › COHORTS/patient distributions/2021_8_16_14_9_HNSC.pdf]

# GBM

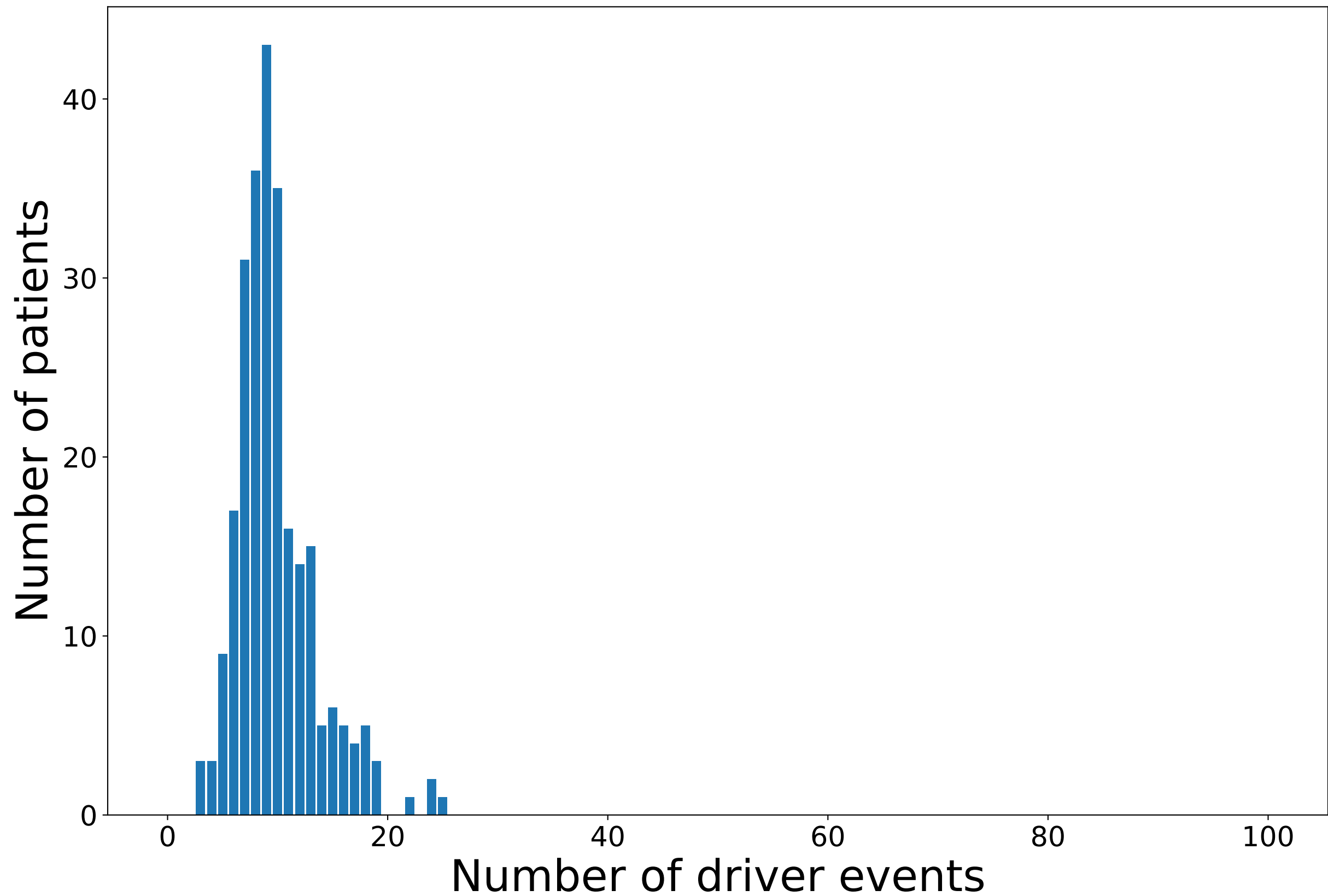

Supplement: Supplemental Information 2 [file peerj-10-13860-s002.zip › COHORTS/patient distributions/2021_8_16_14_9_GBM.pdf]

# ESCA\_MALE

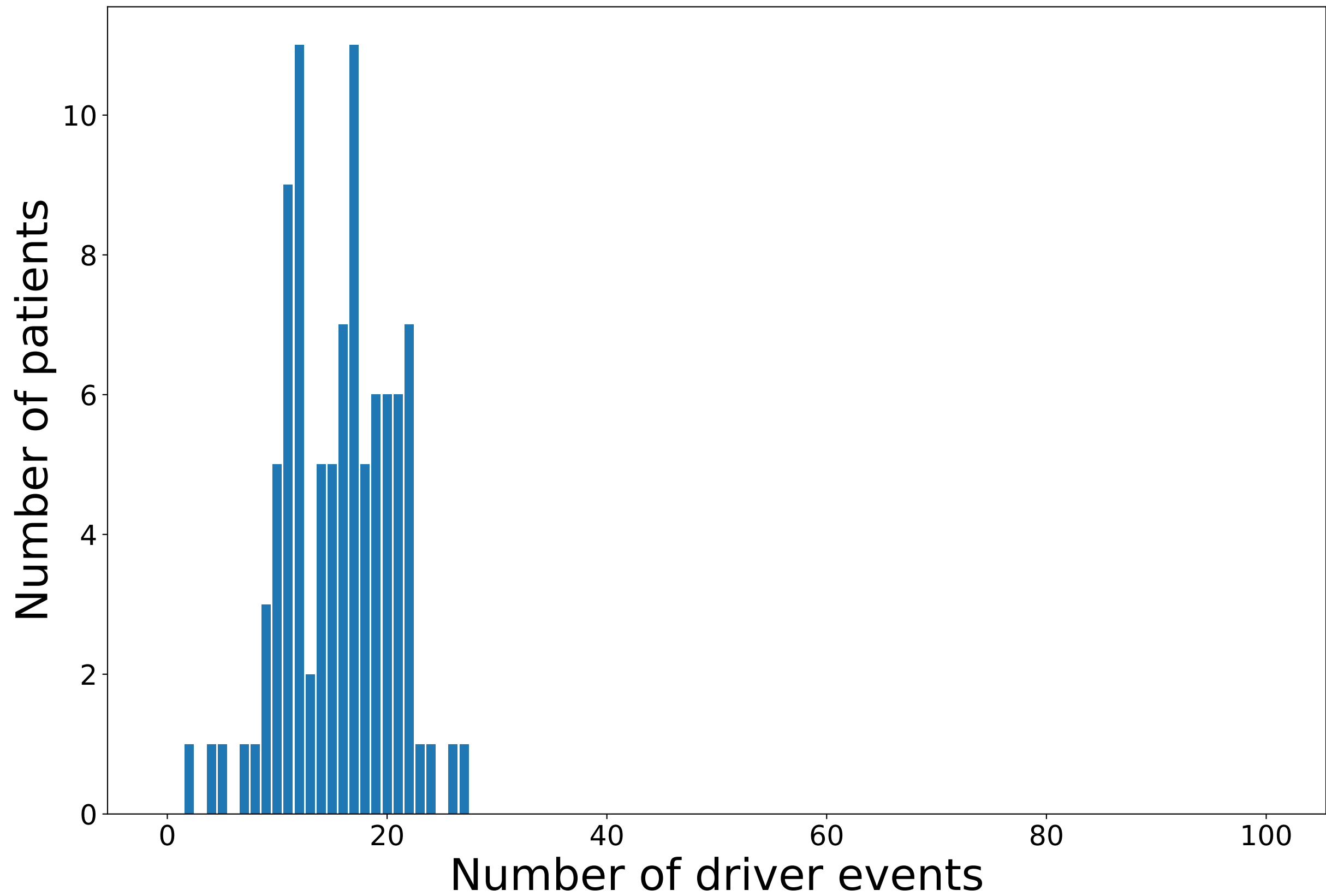

Supplement: Supplemental Information 2 [file peerj-10-13860-s002.zip › COHORTS/patient distributions/2021_8_16_14_9_ESCA_MALE.pdf]

# LIHC\_MALE

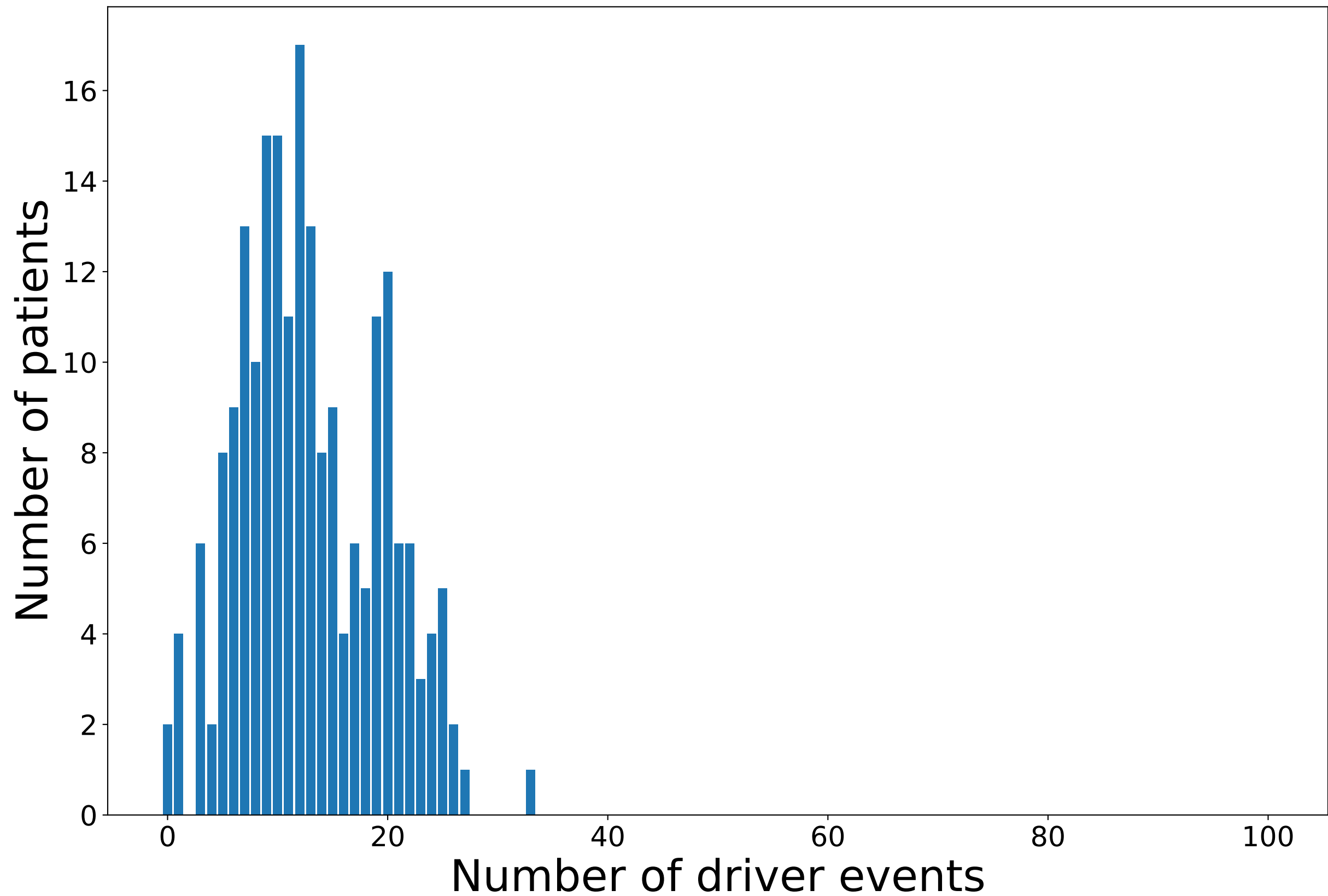

Supplement: Supplemental Information 2 [file peerj-10-13860-s002.zip › COHORTS/patient distributions/2021_8_16_14_9_LIHC_MALE.pdf]

# READ\_FEMALE

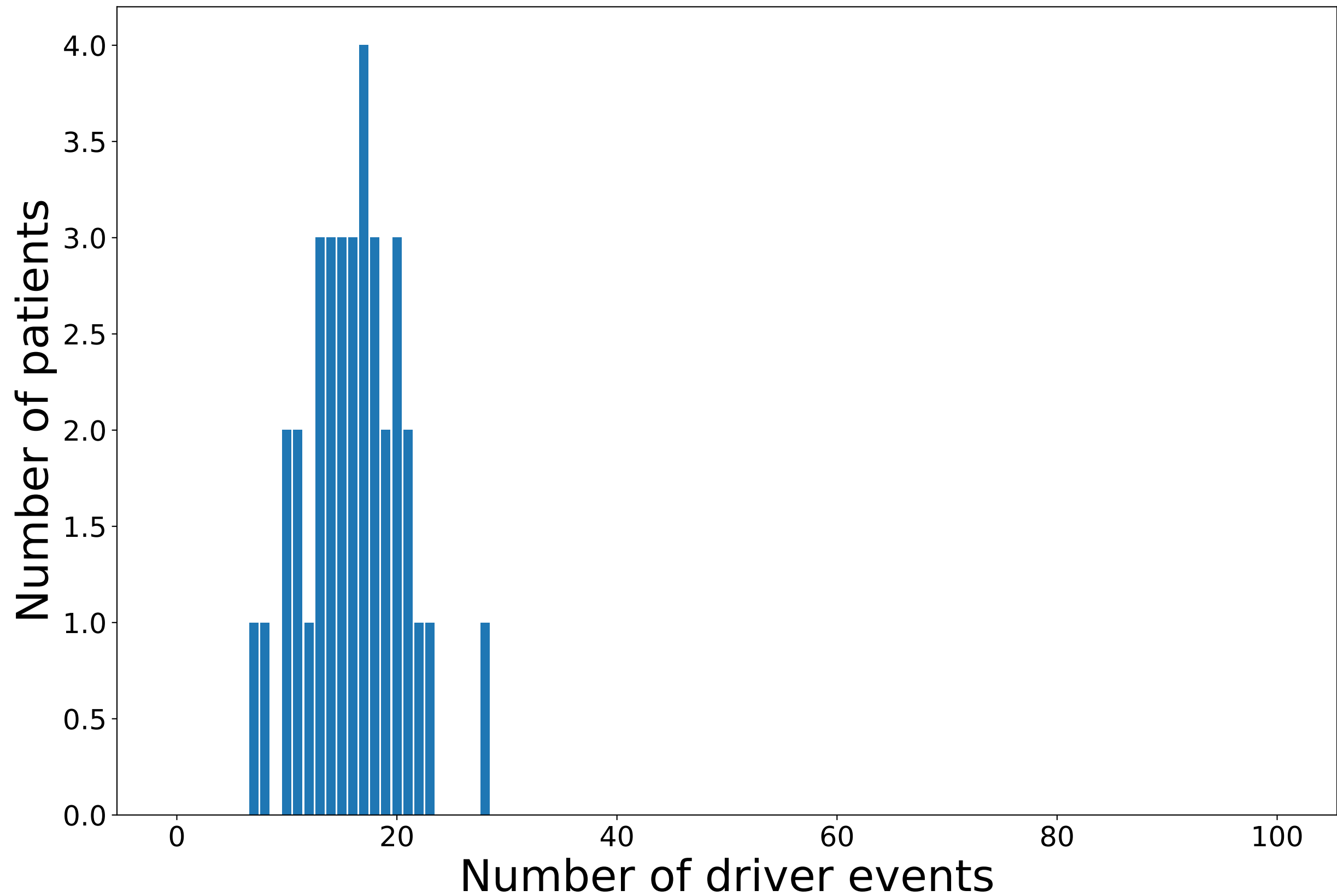

Supplement: Supplemental Information 2 [file peerj-10-13860-s002.zip › COHORTS/patient distributions/2021_8_16_14_9_READ_FEMALE.pdf]

# LUAD\_FEMALE

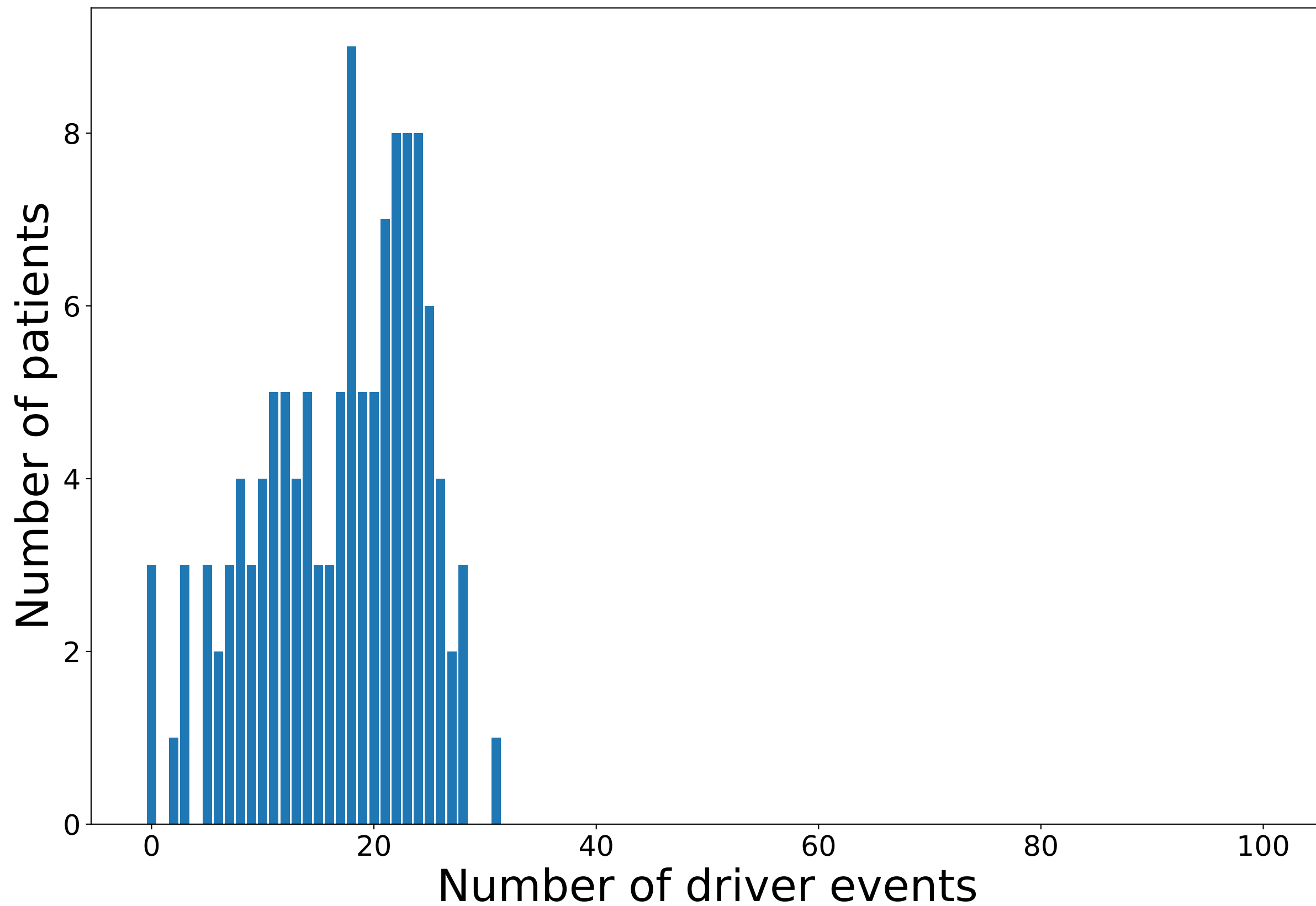

Supplement: Supplemental Information 2 [file peerj-10-13860-s002.zip › COHORTS/patient distributions/2021_8_16_14_9_LUAD_FEMALE.pdf]

PRAD

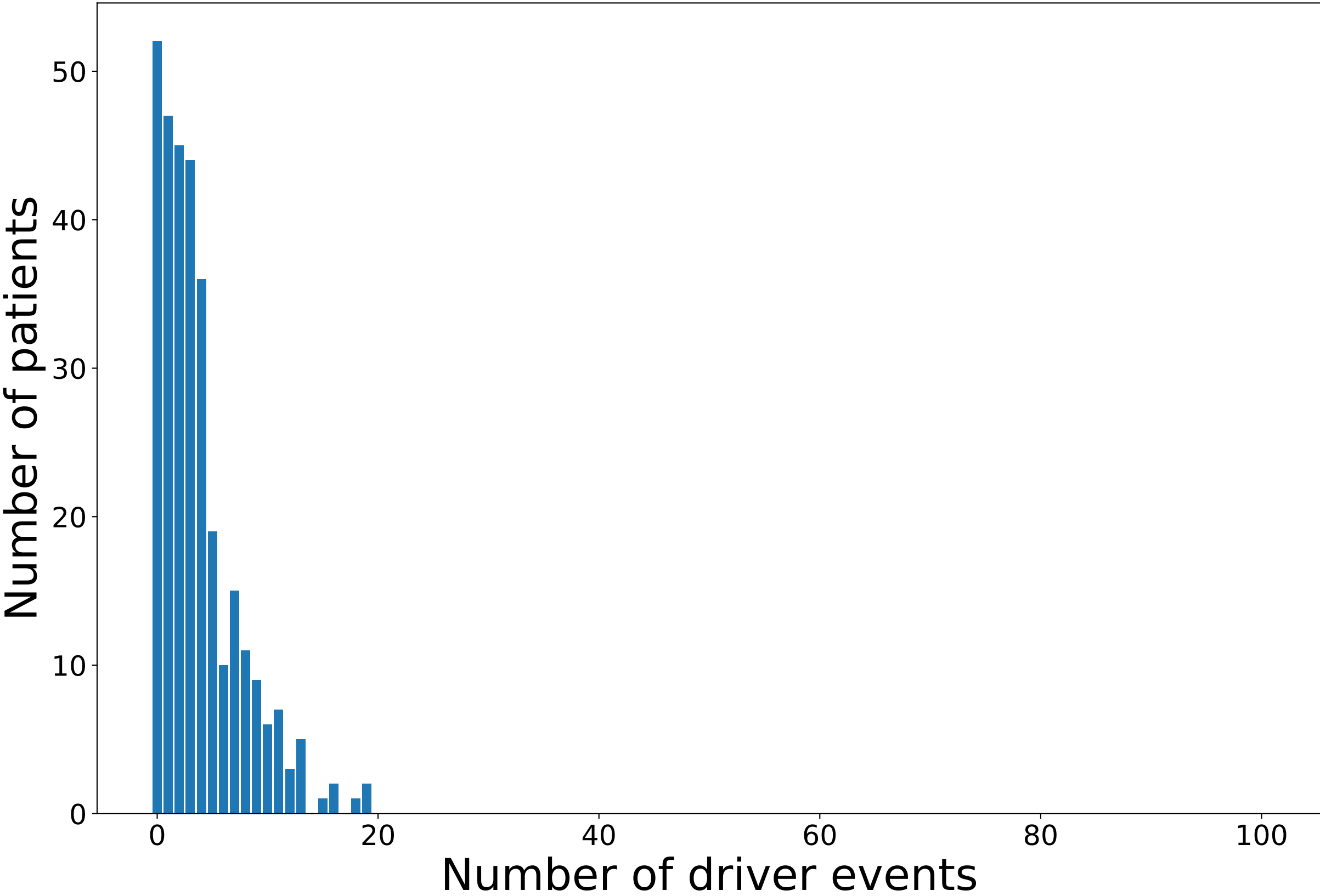

Supplement: Supplemental Information 2 [file peerj-10-13860-s002.zip › COHORTS/patient distributions/2021_8_16_14_9_PRAD.pdf]

# SKCM\_FEMALE

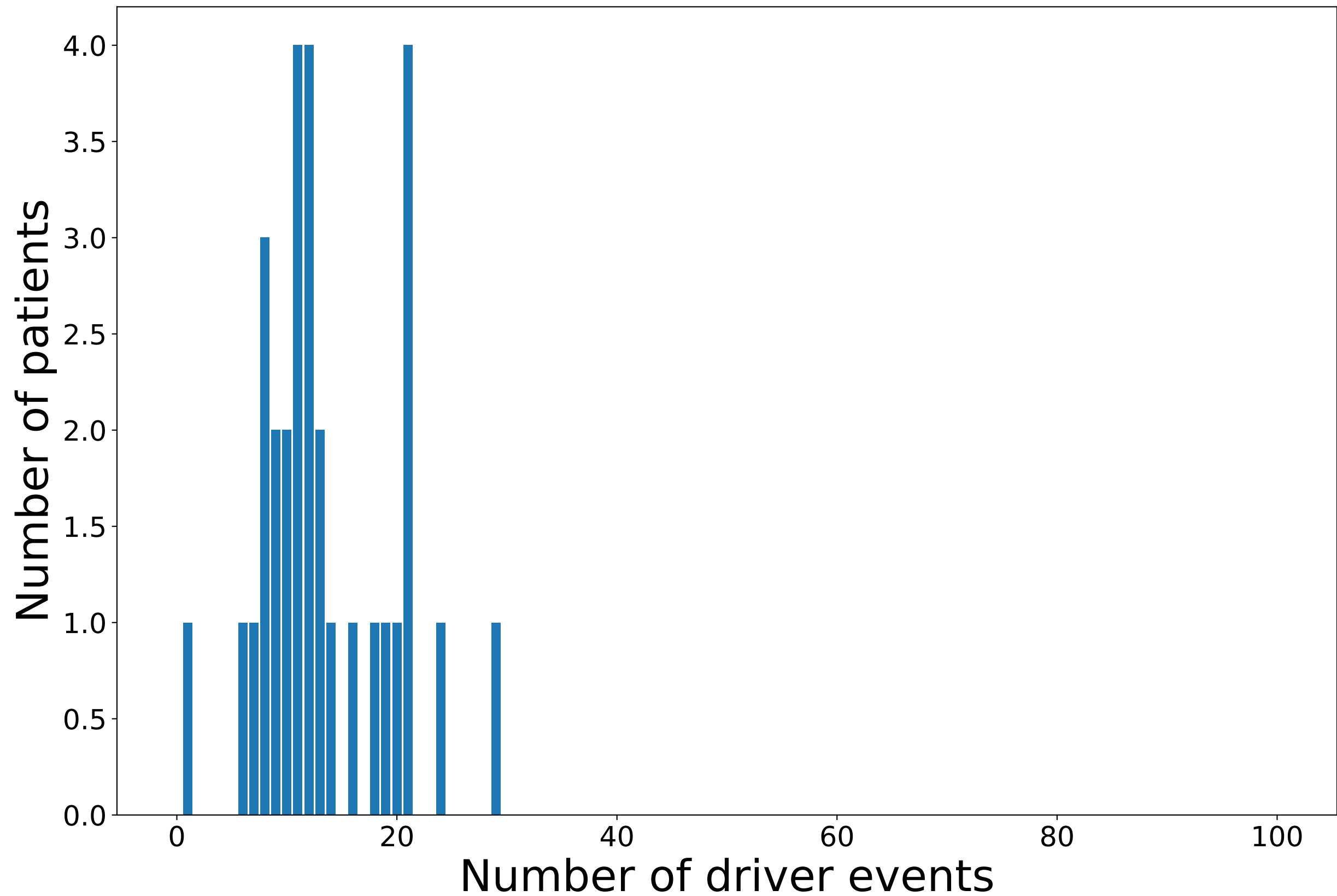

Supplement: Supplemental Information 2 [file peerj-10-13860-s002.zip › COHORTS/patient distributions/2021_8_16_14_9_SKCM_FEMALE.pdf]

# PANCAN\_FEMALE

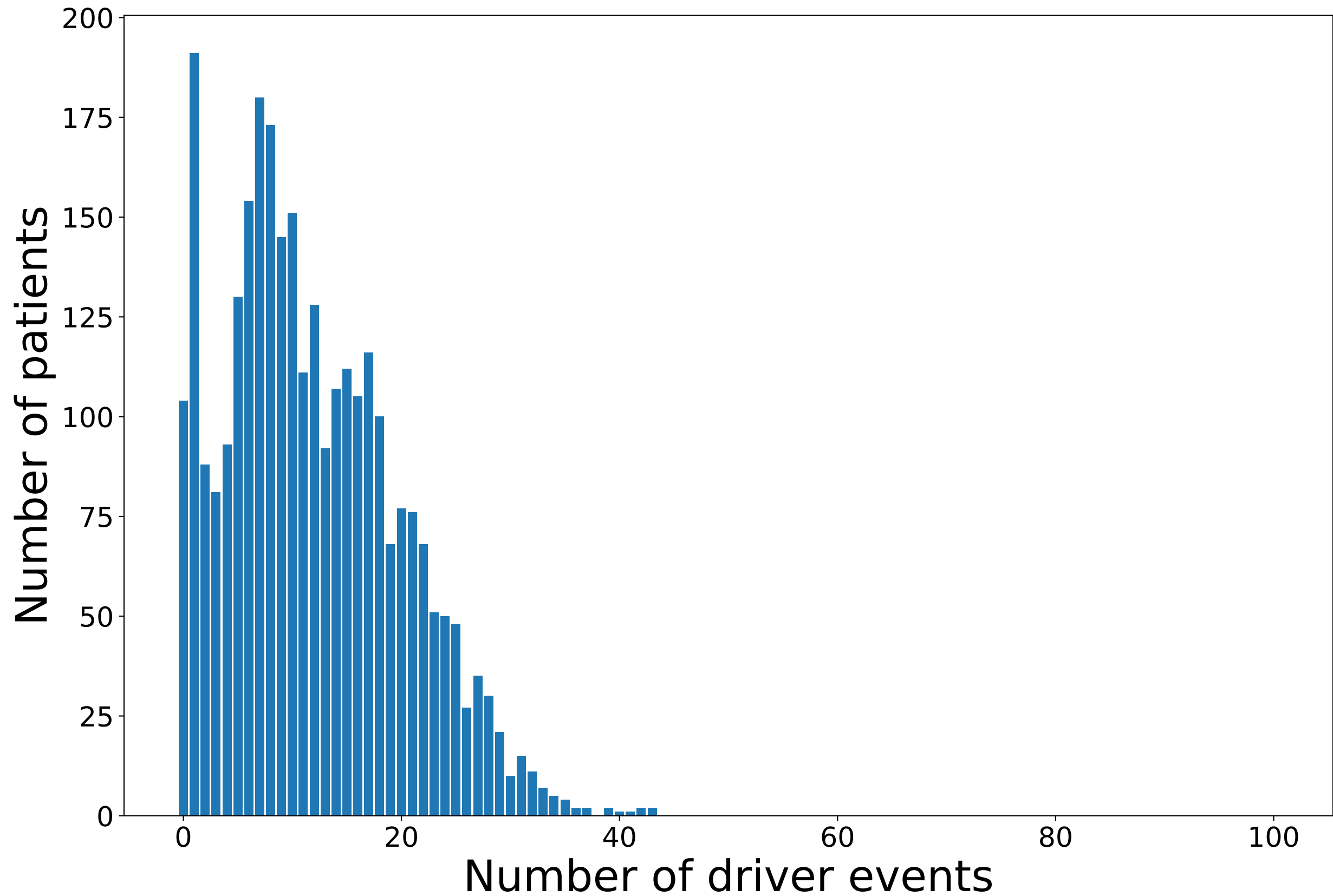

Supplement: Supplemental Information 2 [file peerj-10-13860-s002.zip › COHORTS/patient distributions/2021_8_16_14_9_PANCAN_FEMALE.pdf]

# SARC

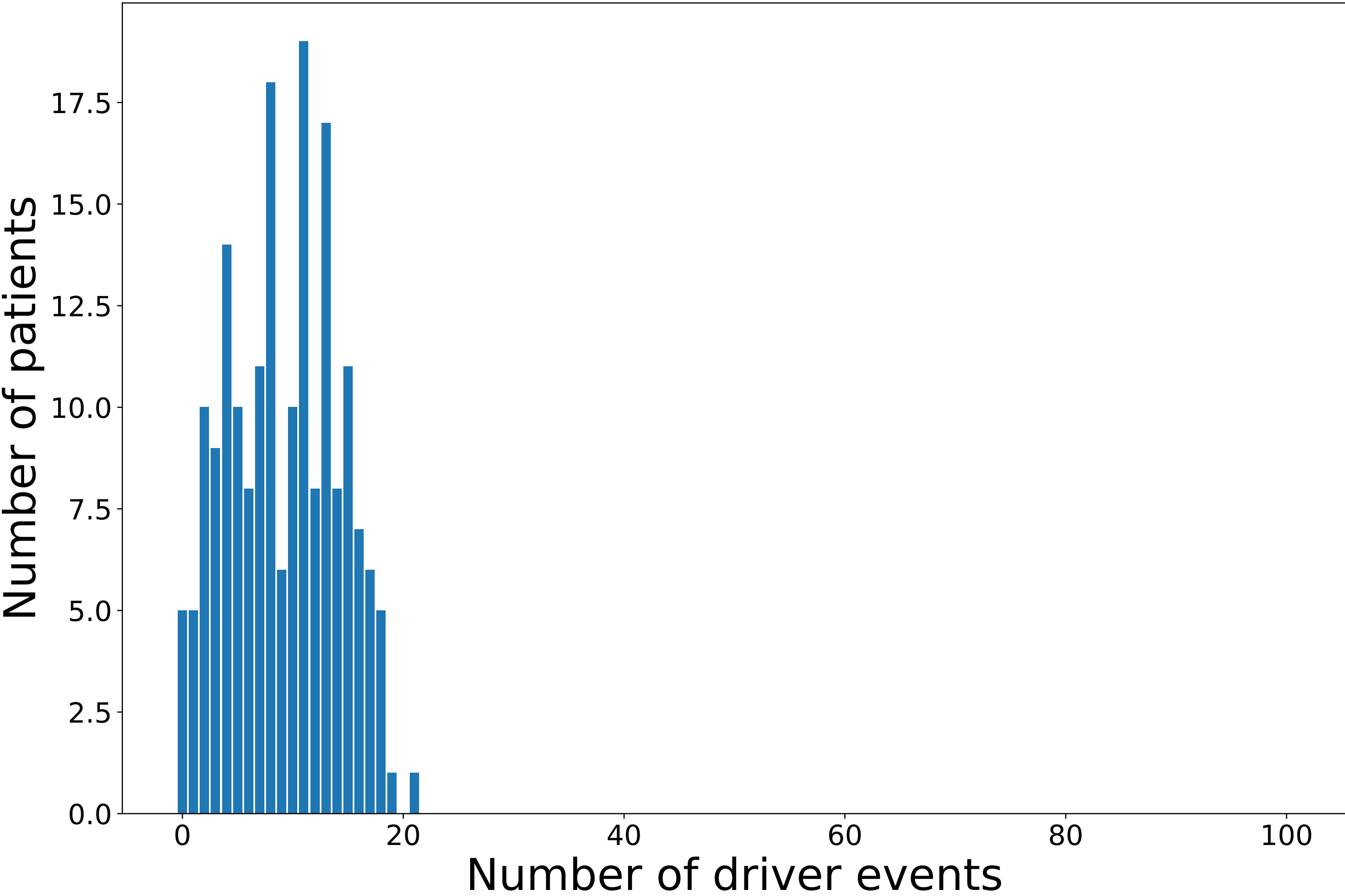

Supplement: Supplemental Information 2 [file peerj-10-13860-s002.zip › COHORTS/patient distributions/2021_8_16_14_9_SARC.pdf]

# PAAD\_FEMALE

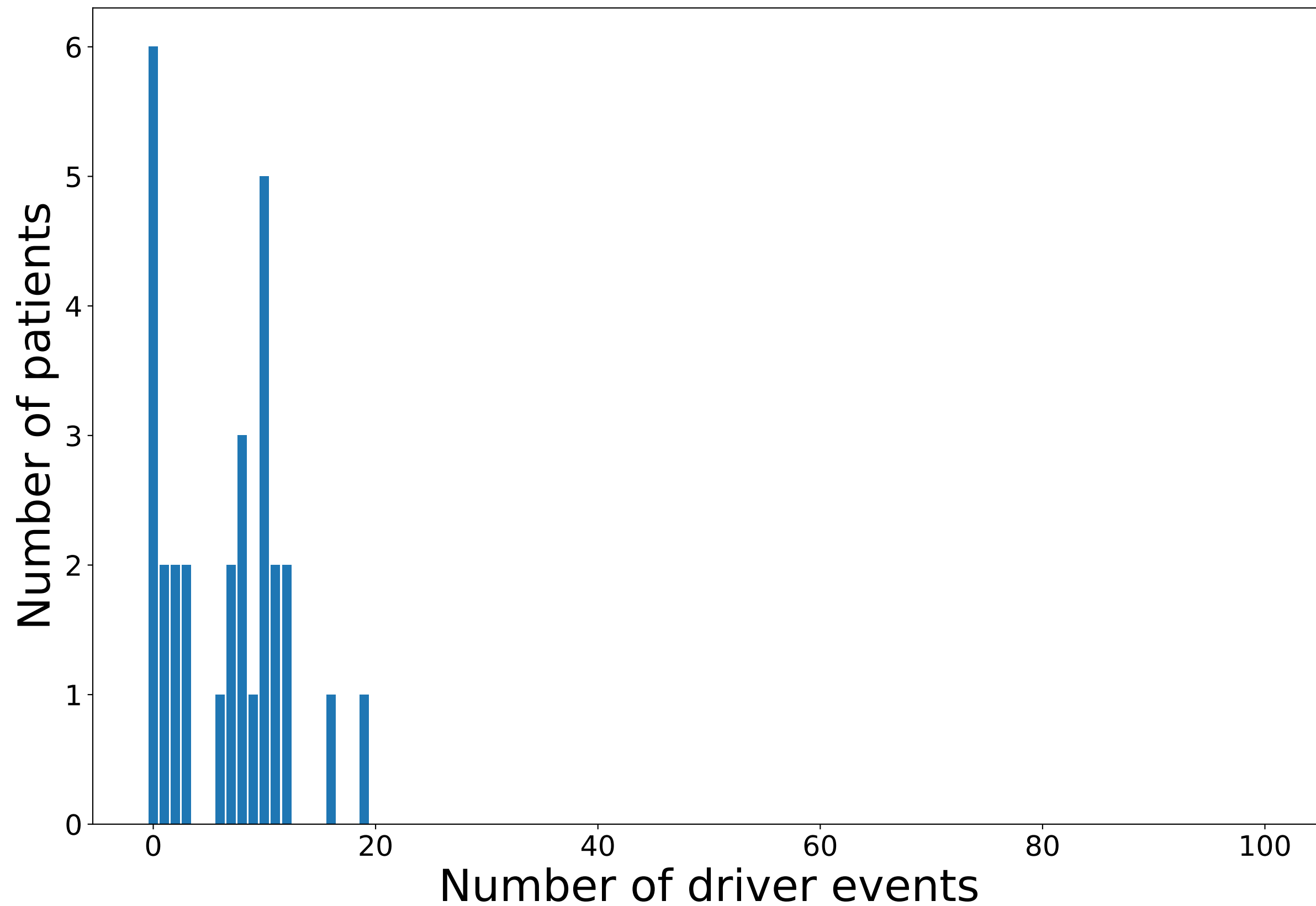

Supplement: Supplemental Information 2 [file peerj-10-13860-s002.zip › COHORTS/patient distributions/2021_8_16_14_9_PAAD_FEMALE.pdf]

# LUAD

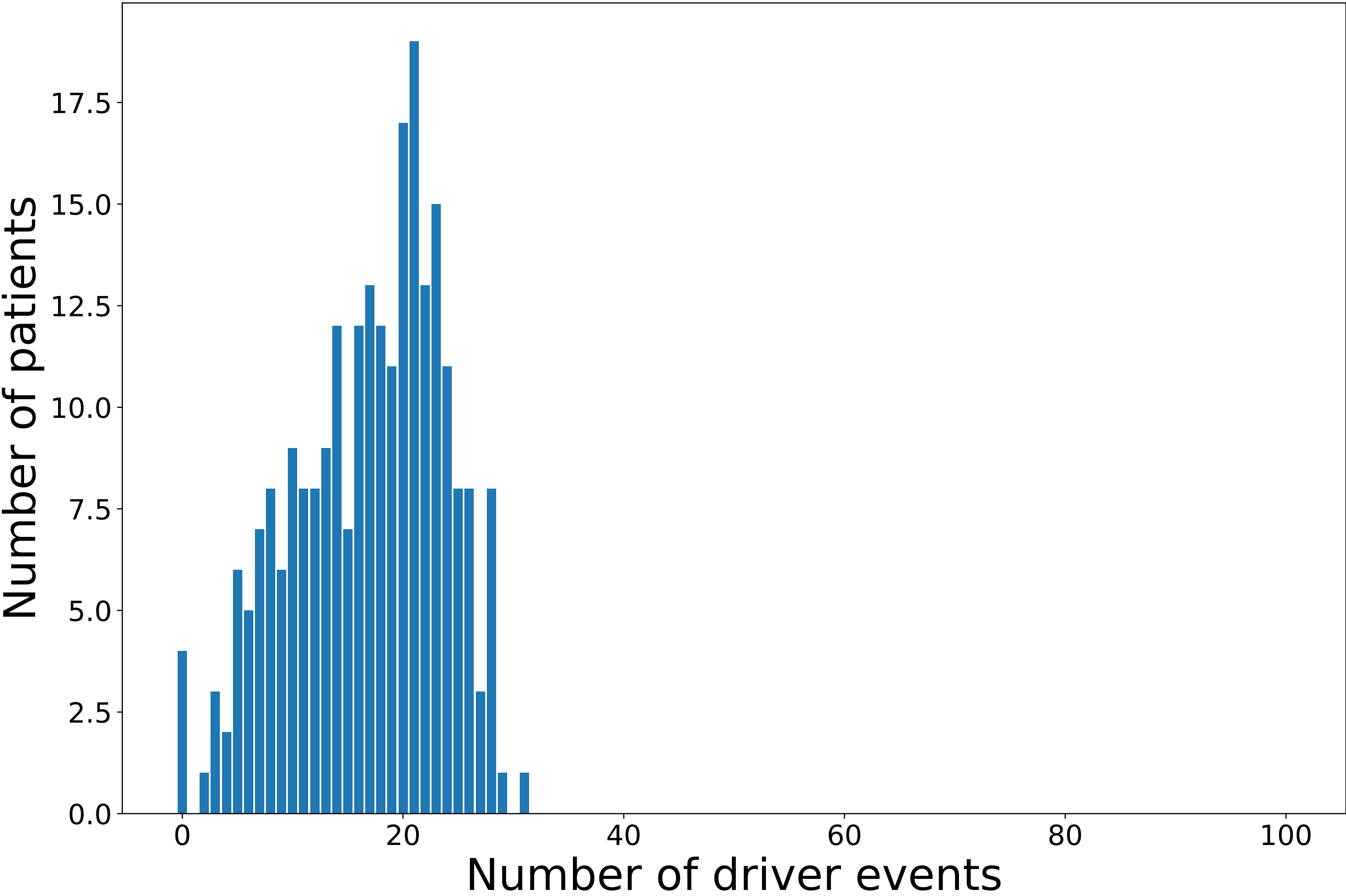

Supplement: Supplemental Information 2 [file peerj-10-13860-s002.zip › COHORTS/patient distributions/2021_8_16_14_9_LUAD.pdf]

# OV\_FEMALE

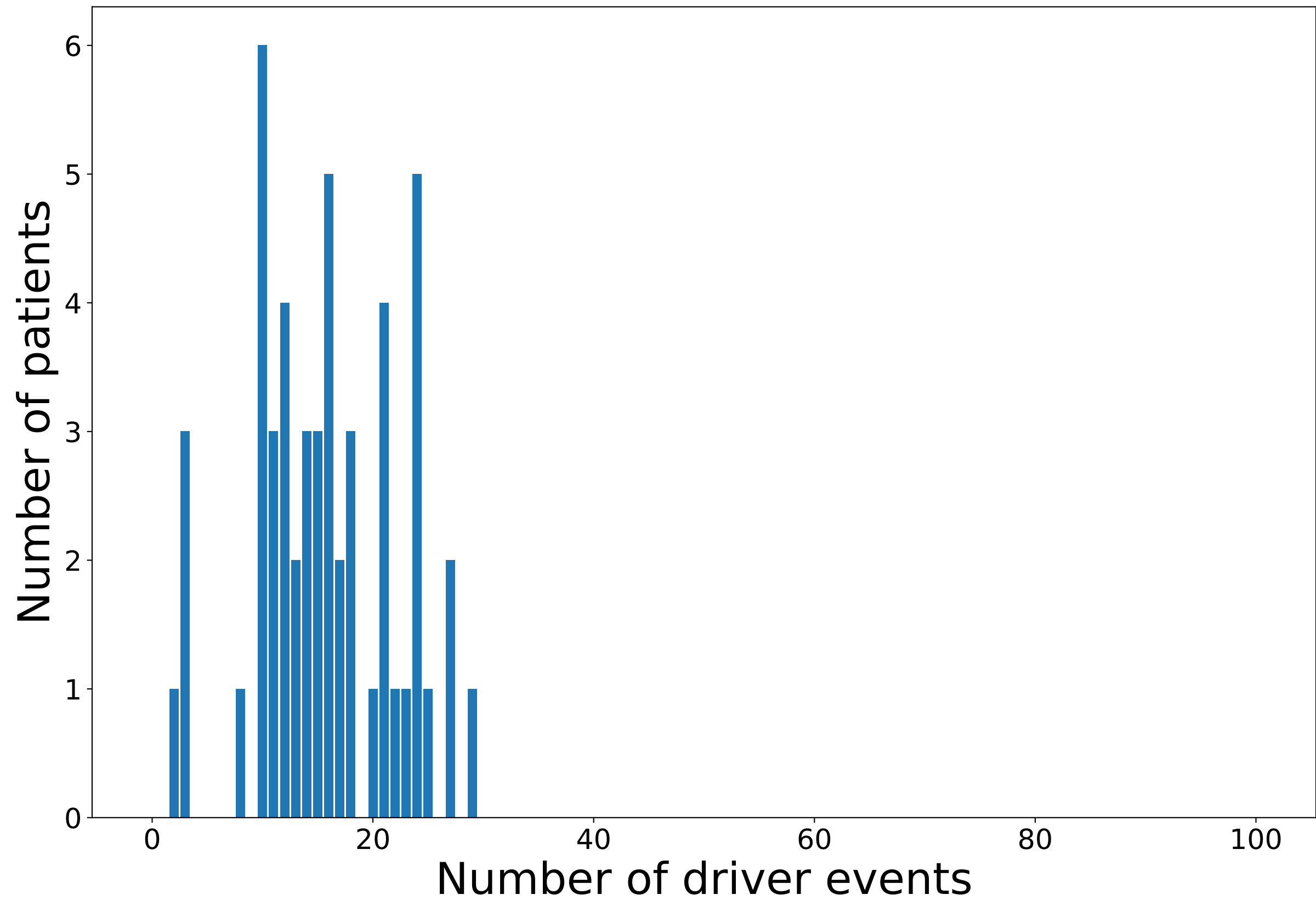

Supplement: Supplemental Information 2 [file peerj-10-13860-s002.zip › COHORTS/patient distributions/2021_8_16_14_9_OV_FEMALE.pdf]

# COAD\_FEMALE

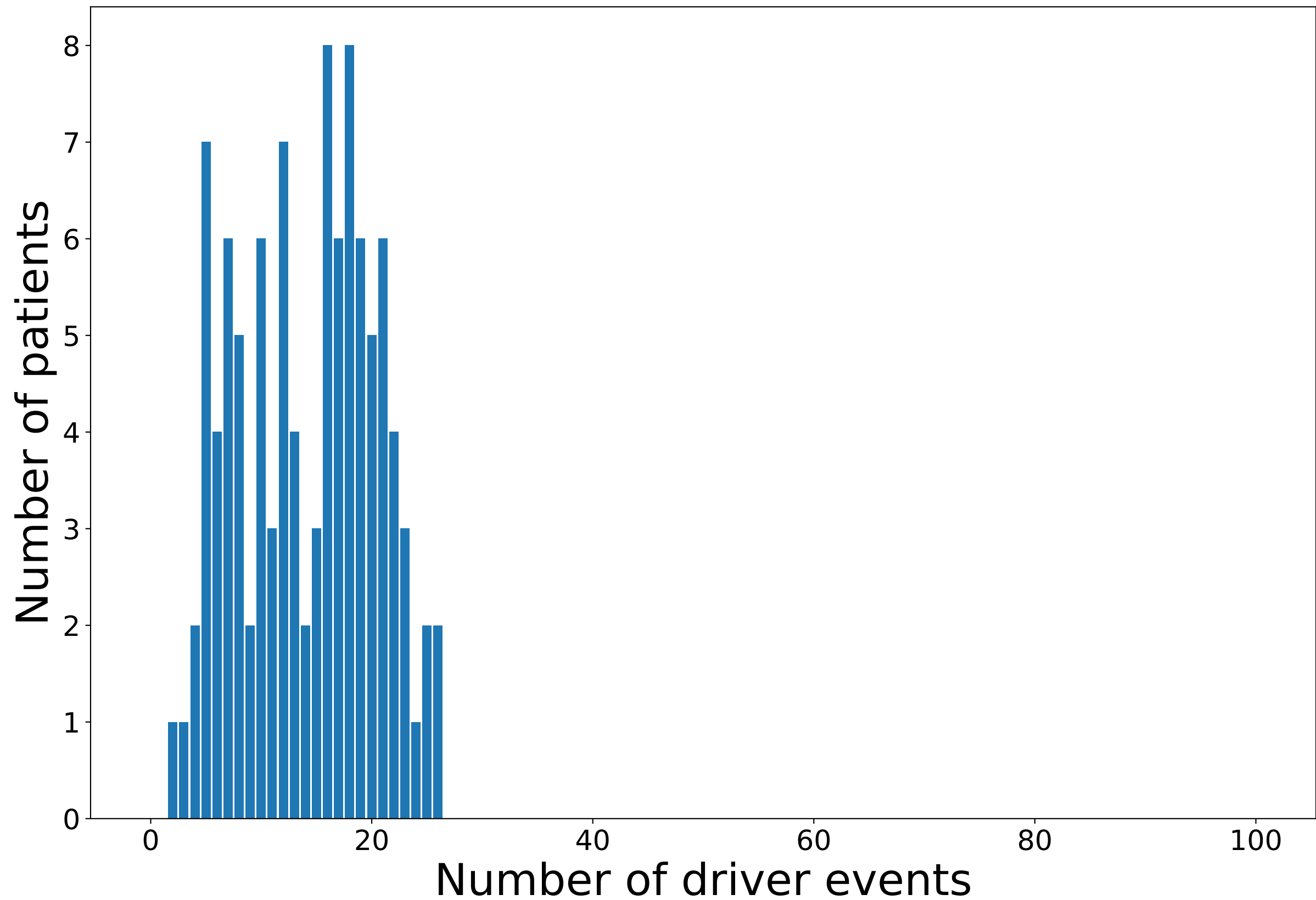

Supplement: Supplemental Information 2 [file peerj-10-13860-s002.zip › COHORTS/patient distributions/2021_8_16_14_9_COAD_FEMALE.pdf]

# TGCT

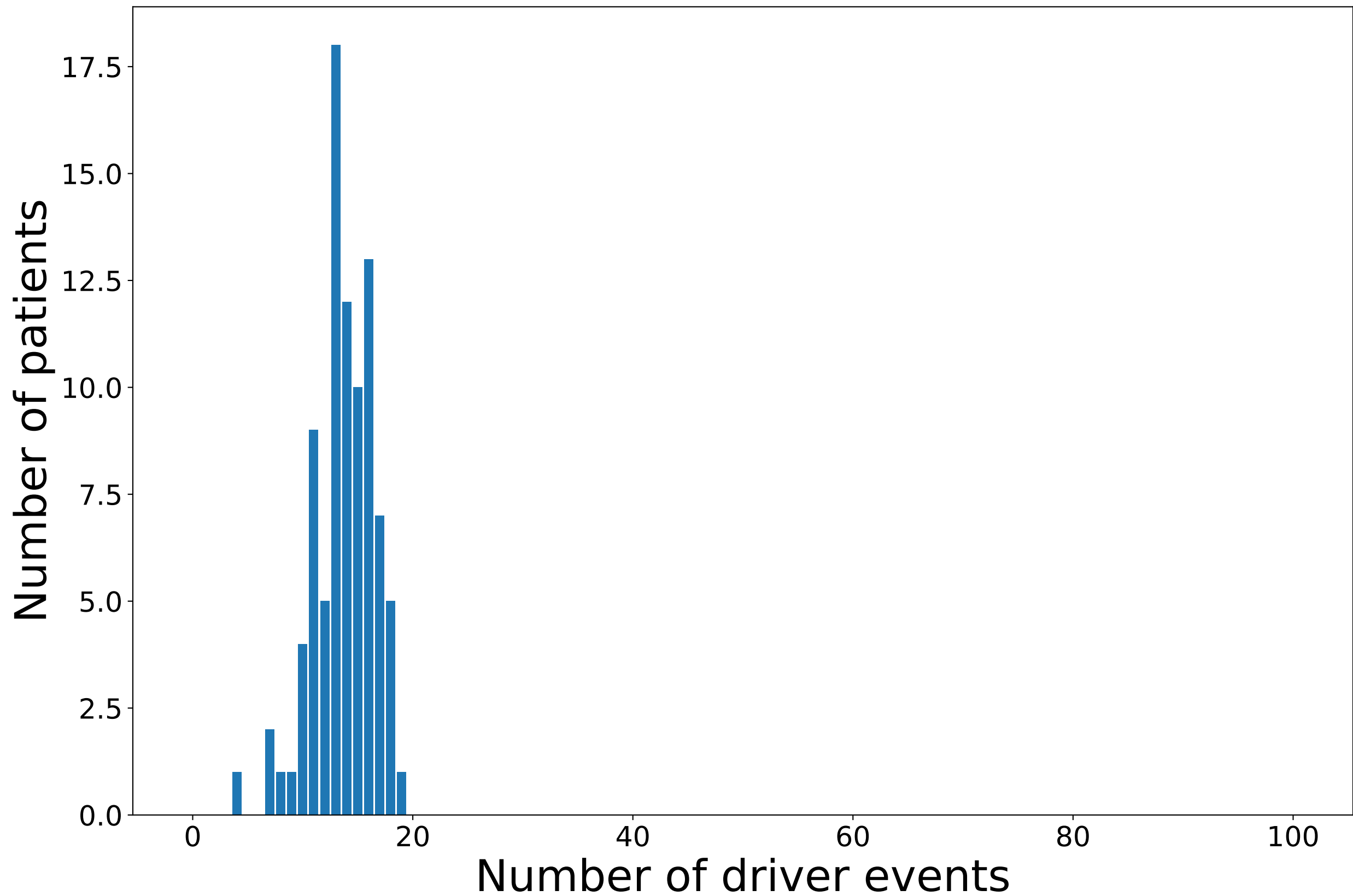

Supplement: Supplemental Information 2 [file peerj-10-13860-s002.zip › COHORTS/patient distributions/2021_8_16_14_9_TGCT.pdf]

# KIRC\_MALE

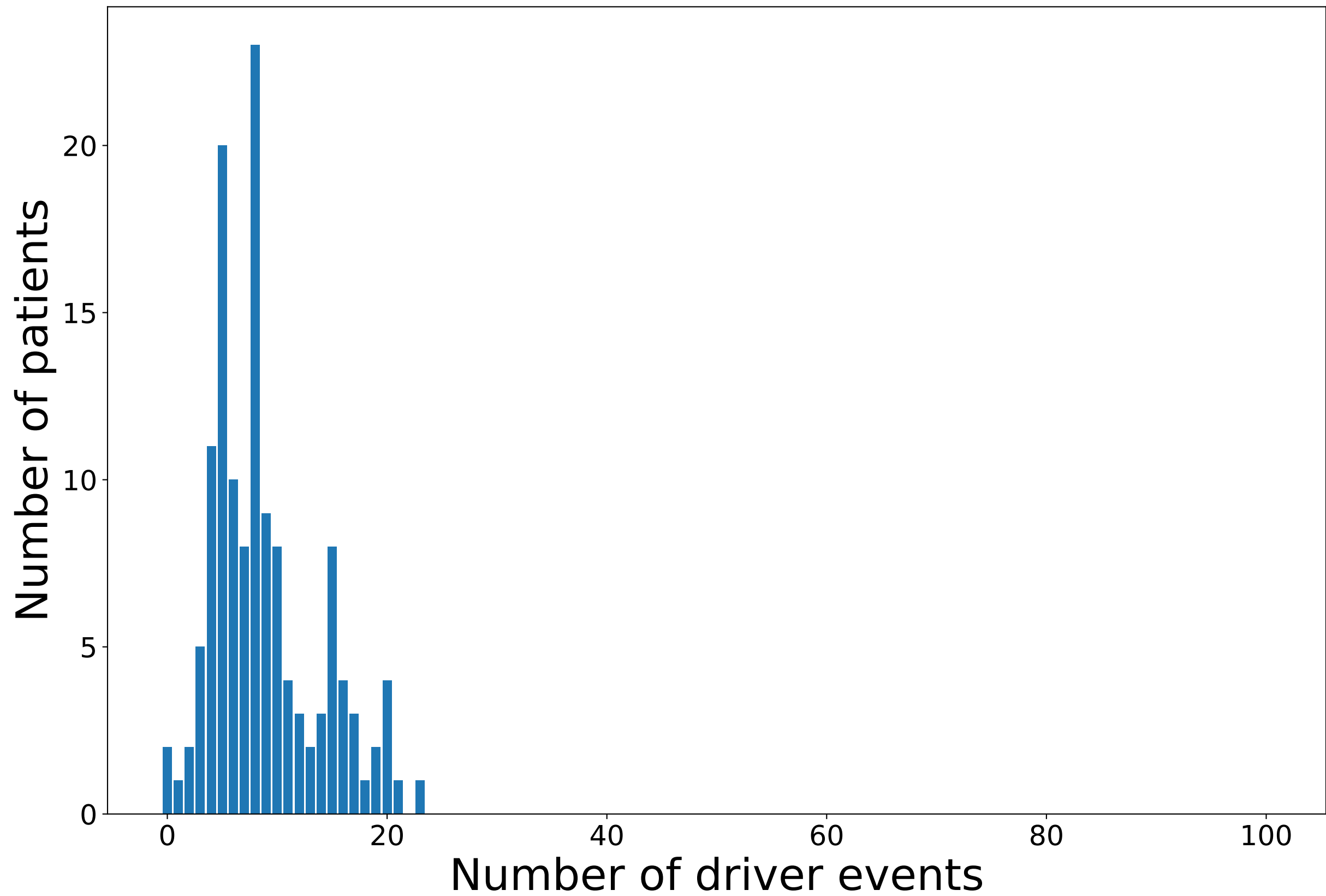

Supplement: Supplemental Information 2 [file peerj-10-13860-s002.zip › COHORTS/patient distributions/2021_8_16_14_9_KIRC_MALE.pdf]

# UCS

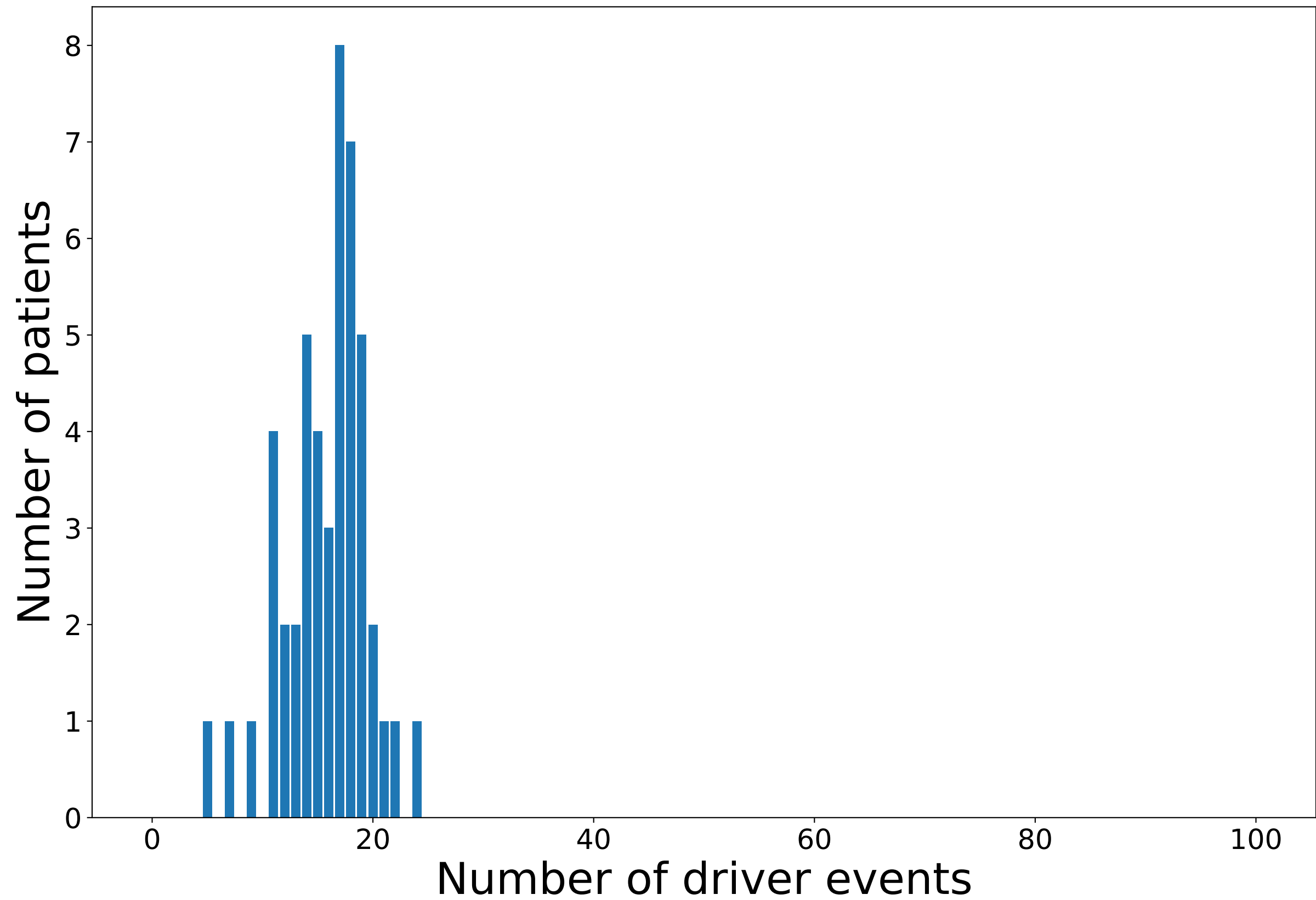

Supplement: Supplemental Information 2 [file peerj-10-13860-s002.zip › COHORTS/patient distributions/2021_8_16_14_9_UCS.pdf]

# CESC

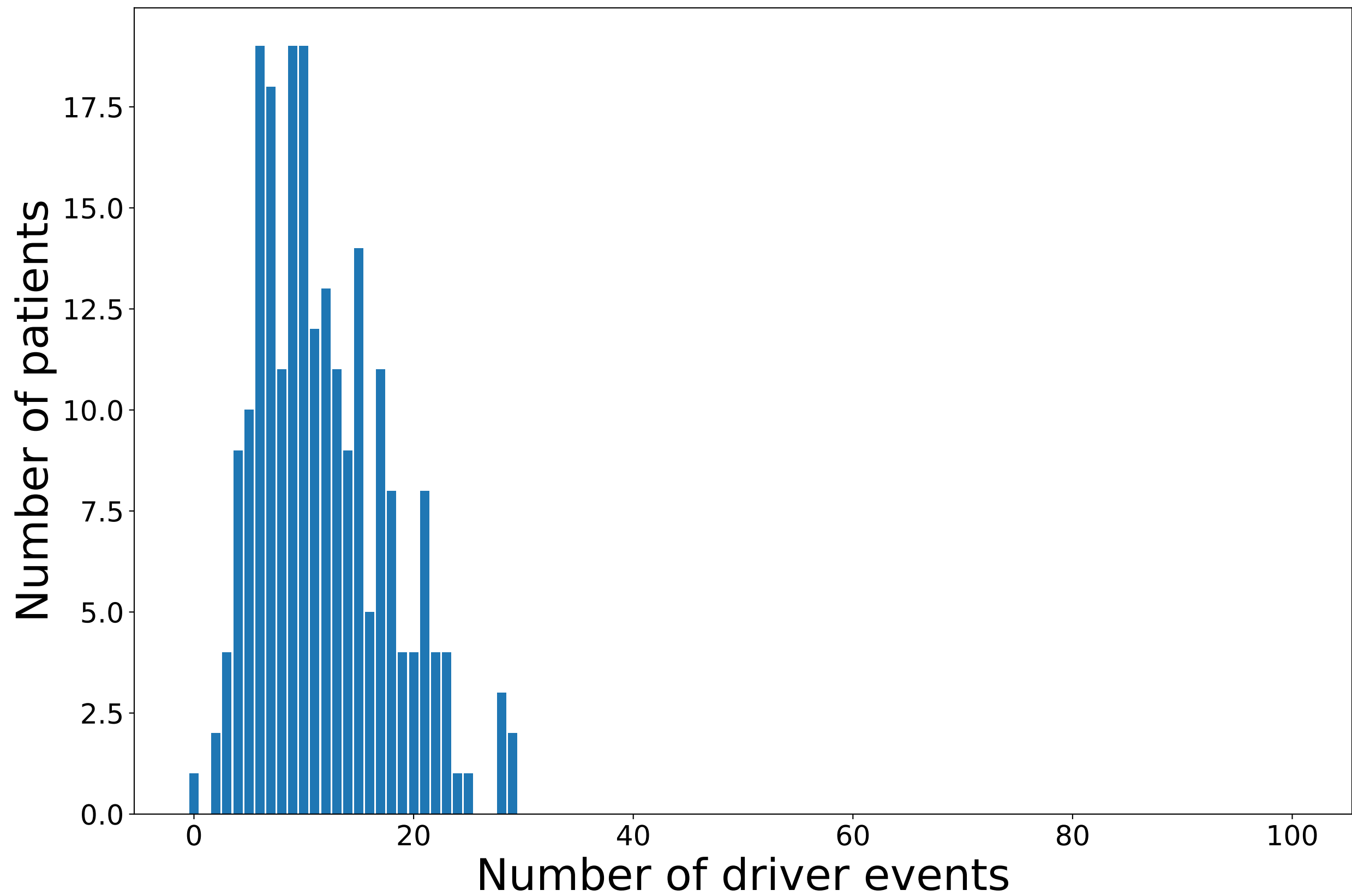

Supplement: Supplemental Information 2 [file peerj-10-13860-s002.zip › COHORTS/patient distributions/2021_8_16_14_9_CESC.pdf]

# SARC\_FEMALE

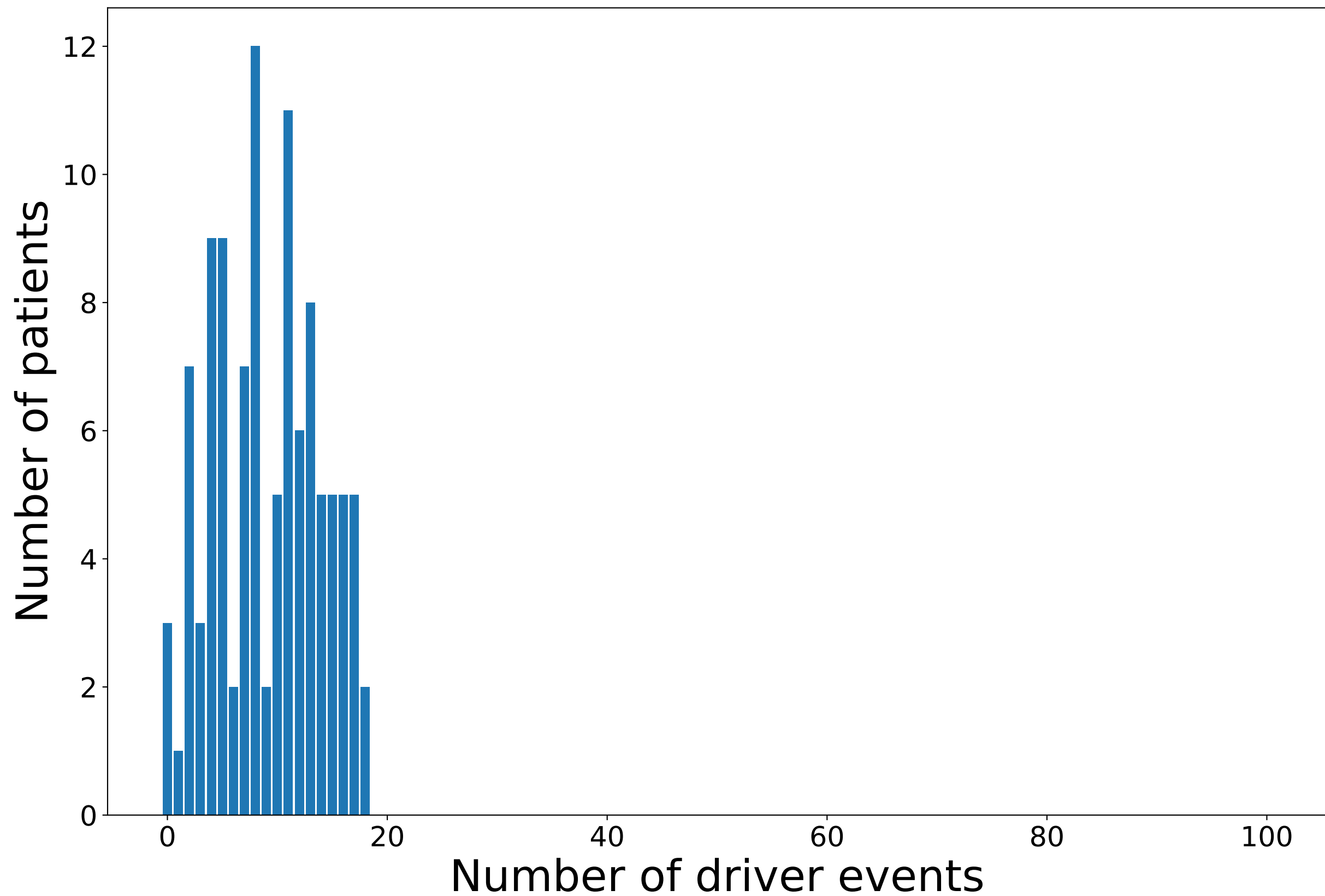

Supplement: Supplemental Information 2 [file peerj-10-13860-s002.zip › COHORTS/patient distributions/2021_8_16_14_9_SARC_FEMALE.pdf]

# ACC\_FEMALE

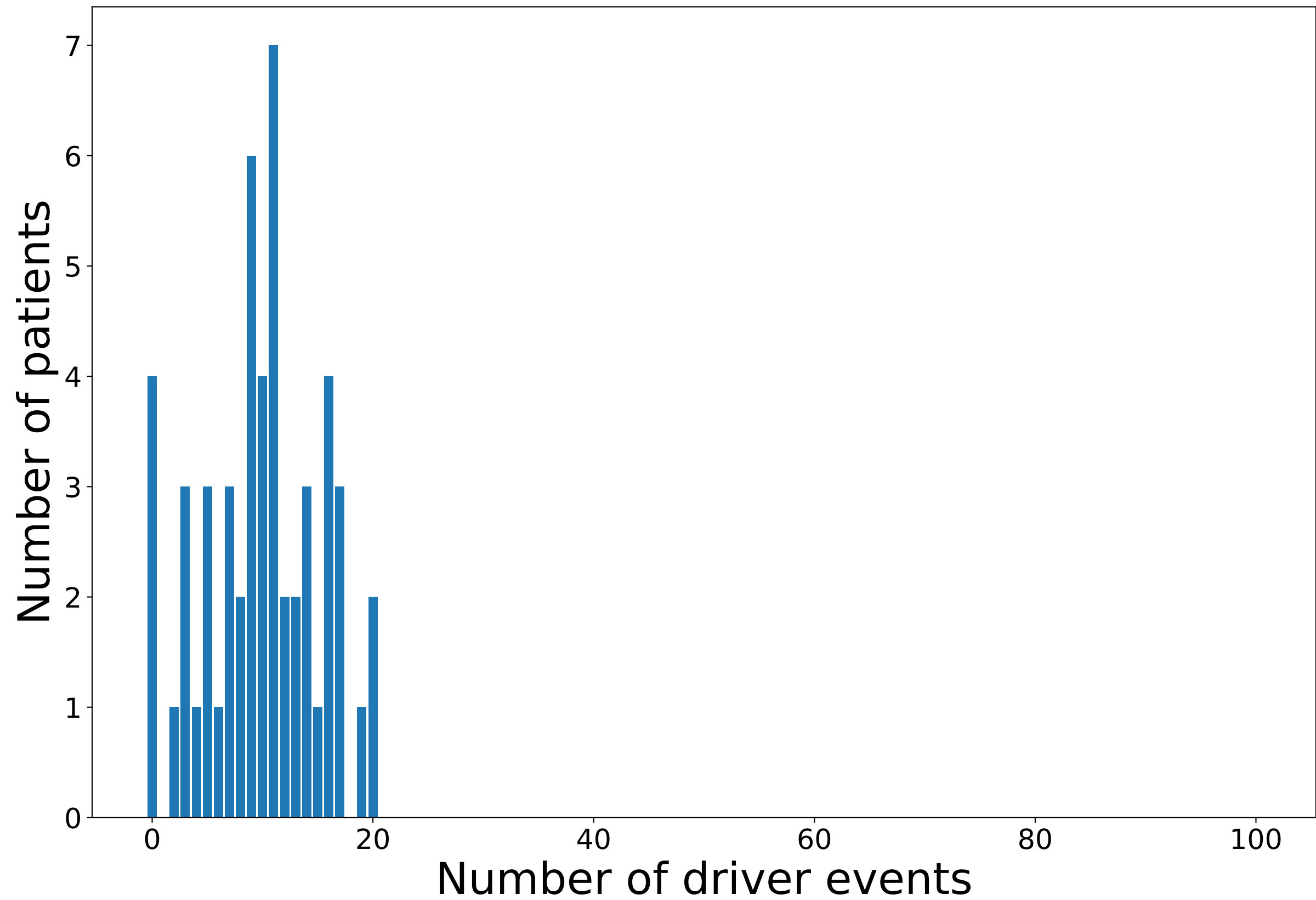

Supplement: Supplemental Information 2 [file peerj-10-13860-s002.zip › COHORTS/patient distributions/2021_8_16_14_9_ACC_FEMALE.pdf]

# STAD\_FEMALE

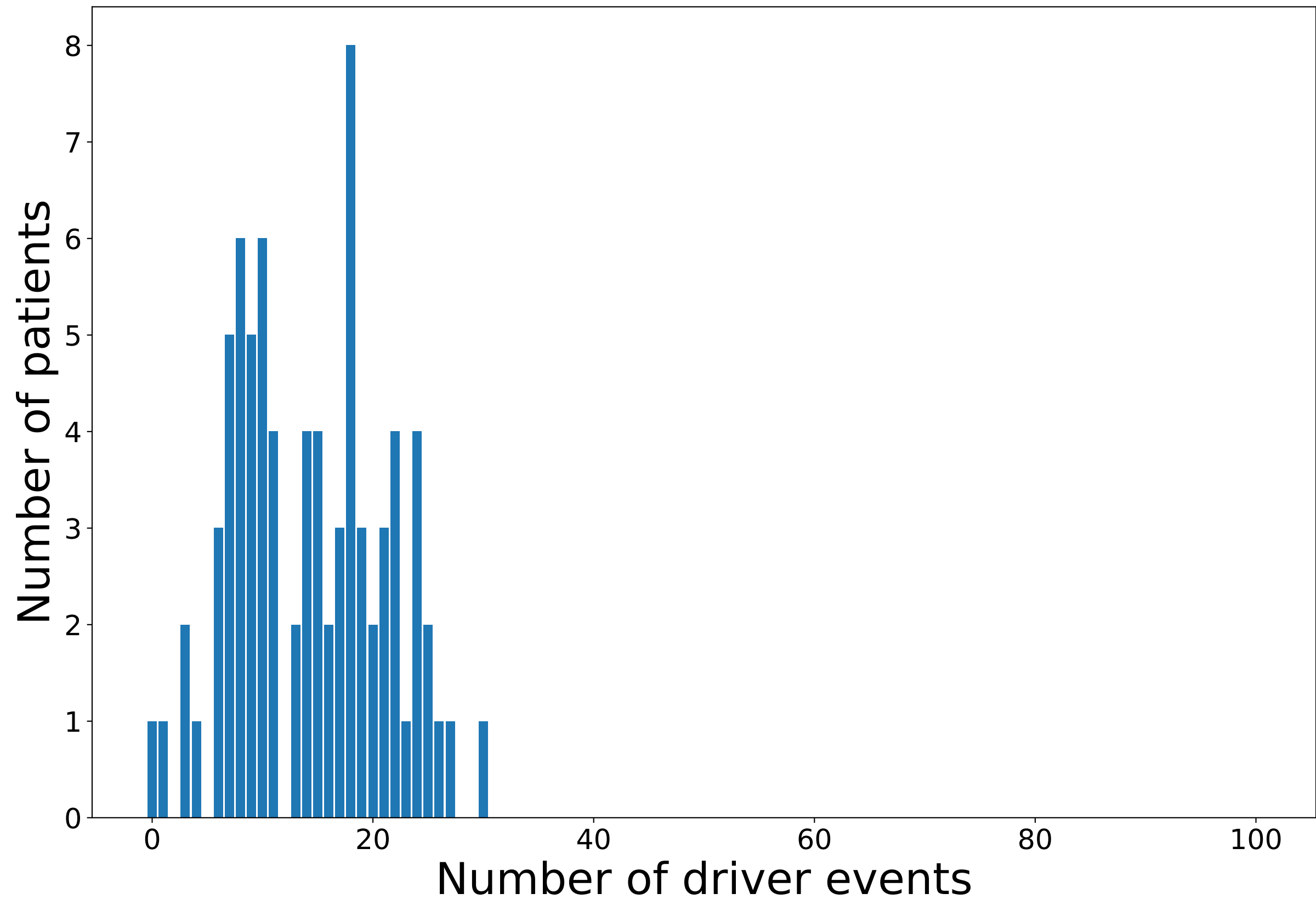

Supplement: Supplemental Information 2 [file peerj-10-13860-s002.zip › COHORTS/patient distributions/2021_8_16_14_9_STAD_FEMALE.pdf]

# UVM\_MALE

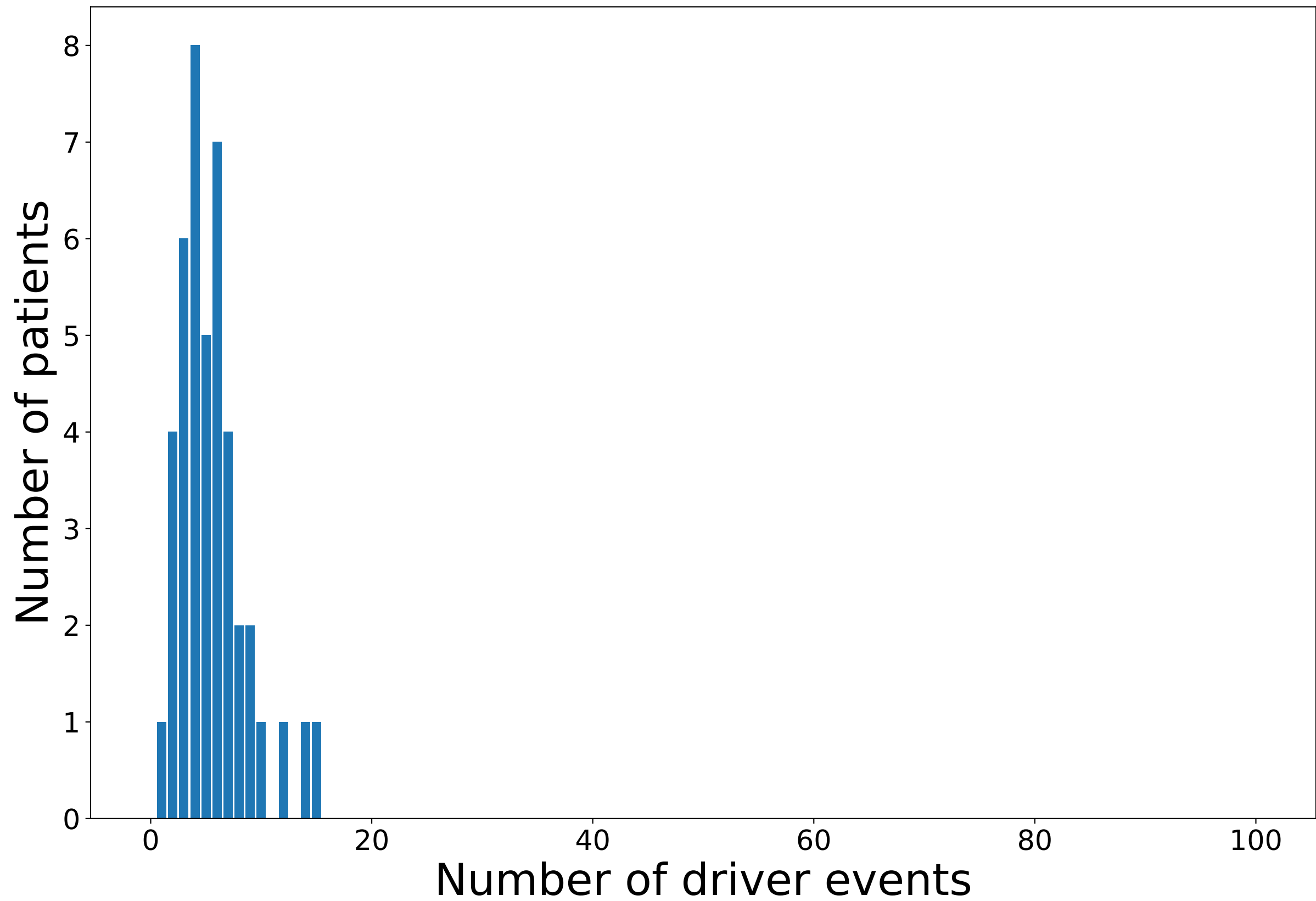

Supplement: Supplemental Information 2 [file peerj-10-13860-s002.zip › COHORTS/patient distributions/2021_8_16_14_9_UVM_MALE.pdf]

# PANCAN

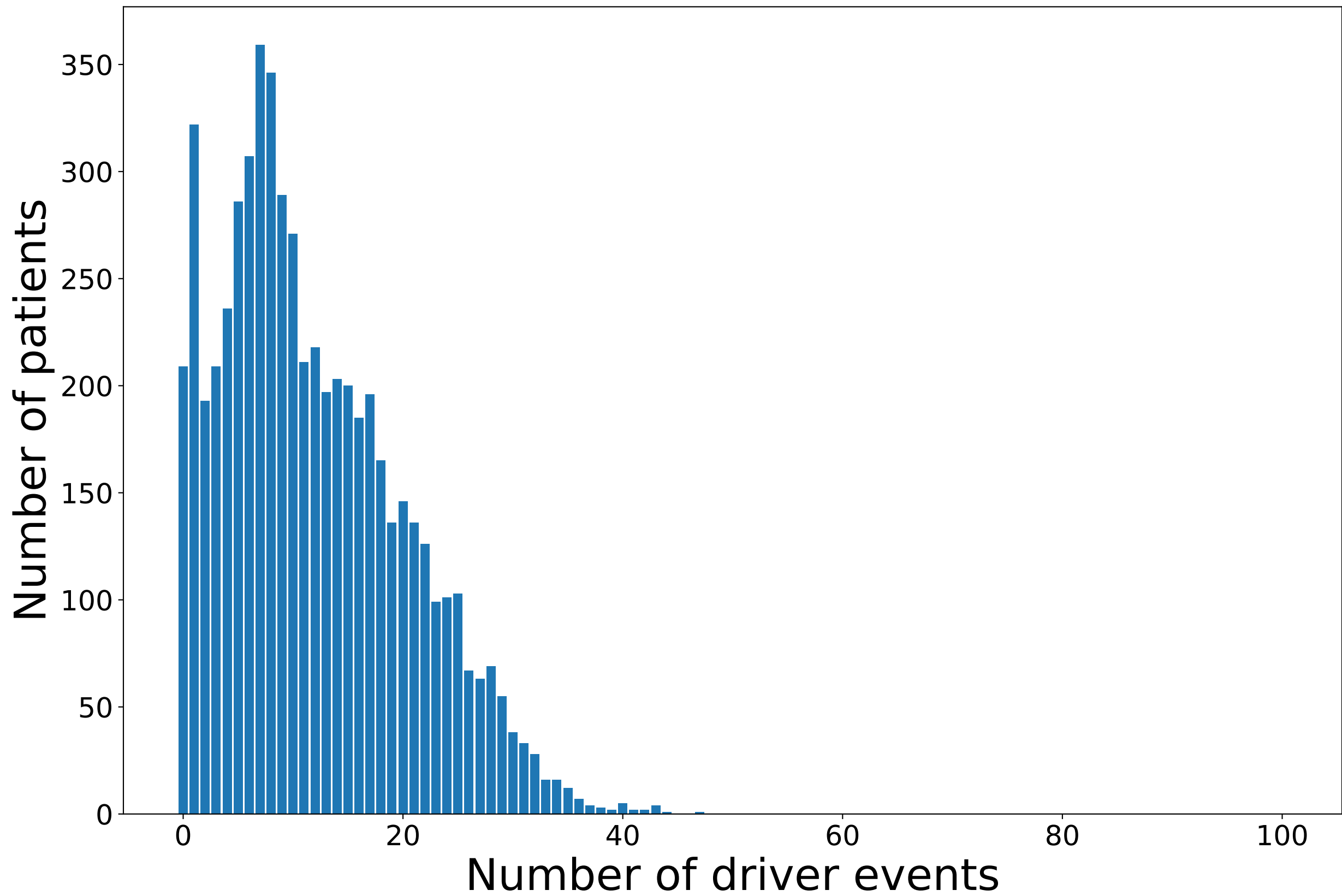

Supplement: Supplemental Information 2 [file peerj-10-13860-s002.zip › COHORTS/patient distributions/2021_8_16_14_9_PANCAN.pdf]
